# Foxf2 represses bone formation via Wnt2b/β-catenin signaling

**DOI:** 10.1038/s12276-022-00779-z

**Published:** 2022-06-06

**Authors:** Tomoyuki Tanaka, Akira Takahashi, Yutaka Kobayashi, Masanori Saito, Sun Xiaolong, Chen Jingquan, Yoshiaki Ito, Tsuyoshi Kato, Hiroki Ochi, Shingo Sato, Toshitaka Yoshii, Atsushi Okawa, Peter Carlsson, Hiroyuki Inose

**Affiliations:** 1 Department of Orthopaedics, Graduate School, Tokyo Medical and Dental University, 1-5-45 Yushima, Bunkyo-Ku, Tokyo, 113-8519 Japan; 2 Research Core, Tokyo Medical and Dental University, 1-5-45 Yushima, Bunkyo-Ku, Tokyo, 113-8519 Japan; 3 Department of Rehabilitation for Movement Functions, Research Institute, National Rehabilitation Center for Persons with Disabilities, 4-1 Namiki, Tokorozawa, Saitama, 359-8555 Japan; 4 Center for Innovative Cancer Treatment, Tokyo Medical and Dental University Hospital, 1-5-45 Yushima, Bunkyo-Ku, Tokyo, 113-8519 Japan; 5 Department of Chemistry and Molecular Biology, University of Gothenburg, Box 462, SE-405 30, Gothenburg, Sweden; 6 Department of Orthopedic and Trauma Research, Graduate School, Tokyo Medical and Dental University, 1-5-45 Yushima, Bunkyo-Ku, Tokyo, 113-8519 Japan

**Keywords:** Osteoporosis, Mechanisms of disease, Mesenchymal stem cells

## Abstract

Differentiation of mesenchymal stem cells (MSCs) into osteoblasts is a critical process for proper skeletal development and acquisition/maintenance of bone mass. However, since this regulatory mechanism has not yet been fully elucidated, the treatment of severe osteoporosis and fractures is a challenge. Here, through a comprehensive analysis of gene expression during the differentiation of MSCs into osteoblasts, we show that the forkhead transcription factor *Foxf2* is a crucial regulator of this process. *Foxf2* expression transiently increased during MSC osteoblastic differentiation. Overexpression of *Foxf2* in MSCs inhibited osteoblastic differentiation, and conversely, knockdown of *Foxf2* expression promoted this process. Osteoprogenitor-specific *Foxf2* knockout mice developed a high bone mass phenotype due to increased bone formation. RNA-seq analysis and molecular experiments revealed that *Foxf2* regulation of bone formation is mediated by *Wnt2b*. Knockdown of *Foxf2* in mouse femurs enhanced bone regeneration in vivo. *FOXF2* expression was correlated with hip bone mineral density in postmenopausal women with low bone mass. Finally, inhibition of *FOXF2* promoted osteoblastic differentiation of human MSCs. This study uncovers a critical role of *Foxf2* in the differentiation of MSCs into osteoblasts and provides insight into the pathogenesis associated with bone-related diseases such as osteoporosis and nonunion after fracture.

## Bone formation: crucial regulatory protein implicated

A regulatory protein called FOXF2 plays a critical role in mediating the formation of bone tissue. Hiroyuki Inose from Tokyo Medical and Dental University, Japan, and colleagues profiled the genes expressed by mouse stem cells as they differentiated into osteoblasts, bone-producing cells. They identified the gene encoding FOXF2 as one of the most highly activated during this developmental transition. To understand how this regulatory protein inhibits bone formation, the researchers blocked the expression of FOXF2, which led to enhanced bone formation, both in mice and human bone marrow mesenchymal stem cells. The researchers also documented a correlation between FOXF2 levels and hip bone mineral density of older women. The findings point to FOXF2 as a promising drug target for the treatment of osteoporosis, non-healing fractures and other bone-related disorders.

## Introduction

Osteoblasts are cells of mesenchymal origin and play an important role in skeletal development and bone formation^1,2^. Thus, understanding the mechanisms that regulate osteoblast differentiation is essential for developing strategies to treat bone-related diseases such as osteoporosis and nonunion after fracture. Over the past three decades, advances in molecular and genetic research have identified various regulatory processes of bone formation. Among them, Wnt signaling plays a central role in this regulation^3^. Binding of Wnt to the receptor complex activates β-catenin-dependent canonical and β-catenin-independent noncanonical signaling pathways^4^. Canonical Wnt binds to the receptor complex of Frizzled and low-density lipoprotein receptor-related protein 5 (*Lrp5*) or *Lrp6*^5^. This complex induces the accumulation of β-catenin in target cells^4^. The accumulation of β-catenin leads to its migration into the nucleus, where it interacts with the T-cell factor/lymphoid enhancer factor (Tcf/Lef) family to initiate the transcription of target genes^5^.

The Wnt canonical pathway is vital for bone formation because loss-of-function or gain-of-function mutations affecting *LRP5* in humans or mice alter bone formation without affecting bone resorption parameters^4^. Regarding the clinical use of the canonical Wnt pathway, sclerostin (SOST)-neutralizing antibodies have been developed^6^. These antibodies are currently used to treat osteoporosis in the clinic^7^, since *SOST*, a Wnt antagonist, interacts with *LRP5* and *LRP6* to inhibit this pathway^8^. However, due to the high cost of antibody drugs, the limited continuous use period of only one year, and the occurrence of vascular adverse events^9^, novel therapeutic targets for this pathway are needed. In addition, with the advent of an aging society in recent years, the number of osteoporotic fractures is increasing^10^. Surprisingly, the incidence of nonunion after fresh osteoporotic vertebral fractures reaches 17.5% in 65- to 85-year-old women^11^, but there are no drugs that can be used to treat nonunion, possibly due to insufficient knowledge of how to effectively stimulate bone formation. Therefore, it is essential to clarify the mechanism of bone formation from a new perspective.

The forkhead transcription factor *Foxf2* is expressed in the mesenchyme of tissues required for morphogenesis and cell differentiation^12^. This molecule is involved in the cascade of interactions between the epithelium and the mesenchyme^12^. *FOXF2* promotes bone metastasis in breast cancer cells by activating the BMP4/SMAD1 signaling pathway^13^. Since *Foxf2* is strongly expressed in limb buds during embryonic development, the importance of *Foxf2* in skeletal development has been suggested^12^. However, the role of *Foxf2* in skeletal development and bone remodeling remains unclear. Interestingly, *Foxf2* is thought to be both an activator and repressor of gene expression^14^, but the role of *Foxf2* in osteoblast differentiation is unknown.

Here, we demonstrate that *Foxf2* regulates the differentiation of mesenchymal stem cells (MSCs) into osteoblasts via canonical Wnt signaling and provide experimental evidence for the role of *Foxf2* in bone remodeling. These findings suggest that targeting the *Foxf2*-canonical Wnt axis is a promising therapeutic strategy to promote bone formation.

## Materials and methods

### Animals

*Foxf2* flox/flox (hereafter, *Foxf2*^f/f^) mice and the paired related homeobox 1 (*Prx1*)-Cre mouse line (hereafter, *Prx1*-Cre mice) have been described previously^15,16^. We crossed *Prx1*-Cre mice with *Foxf2*^f/f^ mice to obtain *Prx1*-Cre tg/*Foxf2*^f/f^ (hereafter, *Foxf2*^*osp*−/−^) mice. We then crossed *Foxf2*^*osp*−/−^ with *Foxf2*^f/f^ mice, and their offspring *Foxf2*^*osp*−/−^ mice and *Foxf2*^f/f^ mice were used for the experiments. All mice were maintained under standard conditions with food and water available ad libitum under a 12 h light/dark cycle.

### RNA-seq analysis

To characterize the transcriptome profile during osteoblast differentiation in ST2 cells, we performed RNA-seq for cells treated with BMP-2 for two days and control cells (Day 0). Total RNA extracted from these cells was sent to Hokkaido System Science Co. for library preparation and sequencing. Total RNA was sequenced at the desired depth (100×) on RNA-seq (Illumina HiSeq). To characterize the transcriptome profile in *Foxf2*-deleted cells, we performed RNA-seq of *Foxf2*^*osp−/−*^ and *Foxf2*^f/f^ samples. Total RNA extracted from calvarial bones was sent to Macrogen Japan Co. for library preparation and sequencing. Captured mRNA by oligo dT primers was sequenced at the desired read count (40 M reads) on RNA-seq (Illumina NovaSeq). The reads were aligned to the mouse genome with the Spliced Transcripts Alignment to a Reference alignment software. The raw read counts were generated using StringTie-count for each annotated gene. The quality and quantity of the RNA obtained was assessed using a NanoDrop 2000 spectrophotometer (Thermo Fisher Scientific, Cleveland, OH, USA) and analyzed with the Agilent 2100 Bioanalyzer system (Agilent Technologies, Santa Clara, CA, USA). Library preparation was performed using the TruSeq Stranded mRNA LT Sample Preparation Kit.

### Pathway analysis

For identification of the pathways affected by *Foxf2* deletion, significantly differentially expressed genes whose expression was increased or decreased by 2.0 times or more in the *Foxf2*^*osp−/−*^ bones compared to the *Foxf2*^f/f^ bones were functionally classified by the PANTHER database (http://pantherdb.org/) into underlying pathways.

### Cell culture

Cells were purchased from the Riken Cell Bank (Tsukuba, Japan). ST2 cells were maintained in RPMI 1640 (Sigma-Aldrich, St Louis, MO, USA) containing 2 mM L-glutamine, 100 units/ml penicillin, 10 µg/ml streptomycin and 10% fetal bovine serum (FBS; Sigma-Aldrich) in 5% CO_2_. Primary murine bone marrow mesenchymal stem cells (BMSCs) were cultured as previously described^17^. Regarding osteogenic differentiation, when the culture plates became 100% confluent, the culture media of each group was changed to osteogenic media containing 10 mM β-glycerophosphate (Sigma-Aldrich) and 50 μg/ml ascorbic acid phosphate (FUJIFILM Wako Pure Chemical Corporation, Osaka, Japan) with 100 nM dexamethasone (Life Technologies, Grand Island, NY, USA) or 100 ng/ml BMP-2 (Funakoshi, Tokyo, Japan). The cells were cultured for two days to examine knockdown efficiency and for seven days to examine osteoblast differentiation, except as specified.

The results are representative of more than three individual experiments.

### Transfection and infection

cDNA fragments of *Foxf2* and *Wnt2b* were chemically synthesized and then cloned into the pBapo CMV vector (TaKaRa, Tokyo, Japan) and PSF-CMV-PURO-NH2-V5 plasmid (Sigma-Aldrich). For the *Foxf2* overexpression study, ST2 cells were seeded and transfected using Lipofectamine LTX reagent (Invitrogen, Carlsbad, CA, USA) according to the manufacturer’s instructions. For the knockdown experiments, *Foxf2* siRNA (ON-TARGET plus L-043398) and *FOXF2* siRNA (ON-TARGET plus L-008261 SMARTpool) were purchased from Dharmacon. As a negative control, ON-TARGETplus Nontargeting Pool (Dharmacon, Chicago, IL, USA) was used. Cells were seeded one day before siRNA treatment and transfected with 20 nM siRNA using Lipofectamine RNAiMAX (Invitrogen). The siRNA sequences are shown in Supplementary Table 1.

### Quantitative real-time PCR analysis

RNA from tissues and cultured cells was also extracted using TRIzol reagent (Invitrogen). Reverse transcription was performed using the High-Capacity cDNA Reverse Transcription Kit (Applied Biosystems, Carlsbad, CA, USA) according to the manufacturer’s instructions. We performed a quantitative analysis of gene expression using the Mx3000p qPCR system (Agilent Technologies). *Gapdh* expression was used as an internal control. Primer sequences are shown in Supplementary Table 2.

### ALP staining

ST2 cells were cultured in osteogenic medium (β-glycerophosphate, ascorbic acid phosphate, and dexamethasone) for 14 days. The cells were then fixed for 20 min in 4% paraformaldehyde and washed three times with PBS. Next, the cells were stained with an ALP Stain Kit (Wako) according to the manufacturer’s instructions.

### Western blot analysis

For immunological detection, 20 μg of cell lysate was separated using SDS-PAGE (7.5–10% Tris gel). Then, the proteins were blotted onto a PVDF membrane, which was incubated with the PVDF blocking reagent Can Get Signal (TOYOBO, Osaka, Japan). Proteins were probed with primary antibodies against β-catenin (1/1000, Abcam, Cambridge, MA, USA), FOXF2 (1/1000, Abnova, Walnut, CA, USA), WNT2B (1/2000, Abcam), LMNB1 (1/2000, Proteintech, Rosemont, IL, USA), and GAPDH (1/2000, MBL, Aichi, Japan). A horseradish peroxidase-conjugated goat anti-rabbit antibody was then added, and secondary antibodies were detected through autoradiography using enhanced chemiluminescence (ECL Plus, General Electric Healthcare, Chicago, IL, USA).

### Microcomputed tomography analysis

We obtained three-dimensional images of distal femurs via microcomputed tomography (μCT, Comscan, Kanagawa, Japan). We examined more than five mice for each group for bone morphometric analysis.

### Biochemistry

Blood samples from mice were collected through cardiac puncture, kept at room temperature for 30 min, and centrifuged at 12,000 g for 15 minutes at 4 °C. Serum procollagen type 1 N-terminal propeptide (P1NP) and C-terminal telopeptide of type 1 collagen (CTX-I) levels were measured via ELISAs for mouse P1NP (Cloud-Clone, Katy, TX, USA) and CTX-I (Immunodiagnostic Systems) according to the manufacturer’s instructions, respectively.

### Histomorphometric analysis

We injected mice with calcein (20 mg/kg, intraperitoneally, Sigma-Aldrich) seven and two days before euthanasia. Undecalcified sections of the distal femur were subjected to toluidine blue staining and TRAP staining, and we performed histomorphometric analyses using an OsteoMeasure Analysis System (Osteometrics Inc., Atlanta, GA, USA) as previously described^18,19^.

### Luciferase reporter assay

ST2 cells were plated into 24-well plates and incubated overnight at 37 °C. The next day, the cells were transfected with siRNA-*Foxf2* or siRNA-negative control (15 pmol each), pTOP-FLASH or pFOP-FLASH reporter plasmids (0.5 μg each), and pGL4.74 hRluc/TK Renilla plasmid using Lipofectamine LTX. Twenty-four hours later, luciferase activity was examined.

### Chromatin immunoprecipitation (ChIP)

ST2 cells were crosslinked with 1% formaldehyde for 10 min and quenched with 0.125 M glycine for 5 min. The cells were washed and lysed with lysis buffer (10 mM Tris-HCl, pH 7.4; 10 mM NaCl; 5 mM MgCl2; 0.2% NP-40) and resuspended with 100 μl of glycerol buffer (10 mM Tris-HCl, pH 7.4; 0.1 mM EDTA; 5 mM MgAc2; 25% glycerol) and 100 μl of reaction buffer (100 mM Tris-HCl, pH 7.4; 50 mM KCl; 8 mM MgCl_2_; 2 mM CaCl_2_). Chromatin was sheared by micrococcal nuclease, the reaction was stopped by EGTA, and the samples were diluted with 1,000 μl of IP buffer (25 mM Tris-HCl, pH 8.0; 150 mM NaCl; 2 mM EDTA; 1% Triton X-100; 0.1% SDS). The chromatin solution was incubated with anti-V5 antibody (Invitrogen)-conjugated Dynabeads Protein G (Invitrogen) at 4 °C for 3 h. Beads were washed with low salt wash buffer (20 mM Tris-HCl, pH 8.0; 150 mM NaCl; 2 mM EDTA; 1% Triton X-100; 0.1% SDS) four times and high salt wash buffer (20 mM Tris-HCl, pH 8.0; 500 mM NaCl; 2 mM EDTA; 1% Triton X-100; 0.1% SDS), and immune complexes were eluted from beads with ChIP elution buffer (50 mM Tris-HCl, pH 8.0; 10 mM EDTA; 1% SDS) at 65 °C. Eluates were additionally incubated at 65 °C to reverse crosslinking and then incubated with proteinase K at 55 °C. DNA was purified by a QIAquick PCR purification kit (Qiagen, Valencia, CA, USA). Aliquots and whole-cell extracts (serving as input samples) were analyzed by PCR amplification. The proximal promoter regions of candidate *Foxf2* target genes in the resulting DNA fragments were PCR amplified using the primers listed in Supplementary Table 3.

### Preparation of patient-derived bone samples and human BMSCs

We enrolled 11 patients who underwent spinal surgeries at the Orthopedic Department of the Tokyo Medical and Dental University Hospital. We obtained written informed consent from all the patients. The inclusion criteria were as follows: (a) postmenopausal women with low bone mineral density (T score ≤ −1.0) who had undergone spinal surgery for a lumbar degenerative disease (e.g., lumbar spondylolisthesis and lumbar spinal stenosis), (b) subjects who had not been treated with antiosteoporosis drugs affecting bone metabolism (e.g., bisphosphonates, selective estrogen receptor modulators, teriparatide, or denosumab), (c) subjects with no systemic disease affecting bone metabolism (e.g., secondary osteoporosis, osteogenesis imperfecta), (d) subjects with no fresh osteoporotic fracture, and (e) subjects with no severe liver or kidney dysfunction. We collected vertebral bone samples.

Human BMSCs were cultured as previously described with modifications^20^. After informed consent was obtained, human BMSCs were cultured from bone marrow aspirates of female patients who had received spine surgery at Tokyo Medical and Dental University under a protocol that was approved by the institutional review board. First, 2 ml of bone marrow aspirate was obtained from the iliac crest of each patient using a bone marrow biopsy needle (Cardinal Health, Dublin, OH, USA). Next, the aspirate was added to 20 ml of standard medium (Dulbecco’s modified Eagle’s medium (DMEM), Sigma-Aldrich) containing 10% fetal bovine serum (Life Technologies) and 1% antibiotic-antimycotic (10,000 U/ml penicillin G sodium, 10,000 μg/ml streptomycin sulfate and 25 μg/ml amphotericin B; Life Technologies) that contained 200 IU sodium heparin (Mochida Pharmaceutical Co., Ltd., Tokyo, Japan) and then centrifuged to remove the fat layer. Bone marrow cells were then resuspended in a standard medium. Subsequently, the bone marrow cells were plated into a 100-cm^2^ dish. The cells were then cultured in each medium at 37 °C in a humidified atmosphere containing 95% air and 5% CO_2_, and the medium was replaced every three days. When primary cultures became nearly confluent, the cells were detached with 0.25% trypsin containing 1 mM EDTA (Life Technologies) and subsequently replated for each assay. The collected hBMSCs were either cultured or preserved separately; the cells from individual donors were assayed independently to prevent cross-contamination of hBMSCs from different donors. Regarding osteogenic differentiation, when the culture plates became 100% confluent, the culture medium of each group was changed to osteogenic medium containing 10 mM β-glycerophosphate (Sigma-Aldrich) and 50 μg/ml ascorbic acid phosphate (Wako) with 100 nM dexamethasone (Life Technologies).

### Bone marrow ablation

Mice were anesthetized intraperitoneally with ketamine and xylazine. After removal of the hair from both hind limbs, the bone marrow of both femora on each animal was ablated as described previously^21^. Briefly, bilateral longitudinal incisions were made on the knees of each mouse to expose the femoral condyle by dislocation of the patella. Next, a hole was made at the intercondylar notch of the femur using a dental drill. A 0.6 mm diameter Kirschner wire was inserted in the proximal end of the femur to confirm the completion of marrow ablation by radiography. Then, 20 µmol of siRNA-*Foxf2* or the negative control was injected into the bone marrow. After injection, the dislocated patella was repositioned, and the skin was sutured. Mice were euthanized at day seven after ablation, and microcomputed tomography analyses were performed.

### Statistics

All data are presented as the mean ± SEM (*n* ≥ 3). We performed statistical analysis using Student’s t test or one-way ANOVA and Tukey’s HSD test. *P* < 0.05 was considered statistically significant.

### Study approval

All animal experiments were performed with the approval of the Animal Study Committee of Tokyo Medical and Dental University and conformed to relevant guidelines and laws. Regarding patient-derived bone samples and human BMSCs, all protocols were approved by the Ethics Committee of Tokyo Medical and Dental University (G2020-031). Written informed consent was received prior to participation.

## Results

### *Foxf2* regulates osteoblastic differentiation of MSCs

**Fig. 1:**
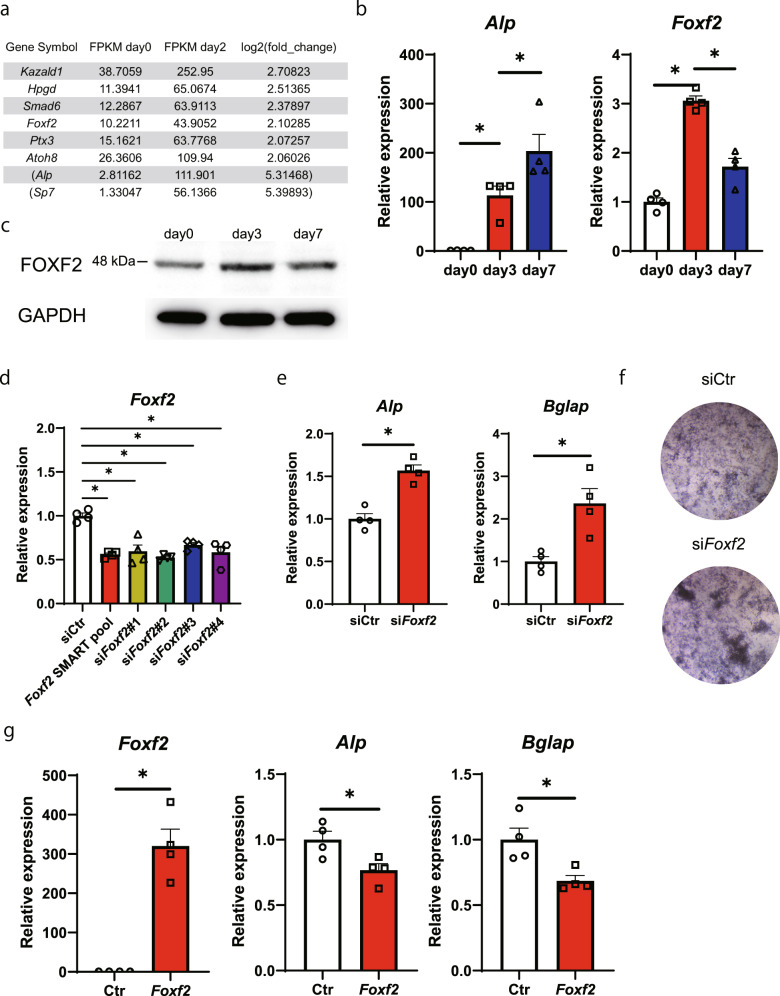
*Foxf2* regulates the osteoblastic differentiation of MSCs. **a** Changes in gene expression were examined between Days 0 and 2 after BMP-2 stimulation by RNA-seq analysis. Only representative genes with FPKM > 10 on Day 0 and whose expression showed a 2-fold increase by BMP-2 treatment are shown. Although *ALP* and *Sp7* did not meet the requirements, they are included in the table as markers of osteoblast differentiation. **b** Changes in *Foxf2* mRNA expression induced by BMP-2 treatment during ST2 cell differentiation, as determined by qPCR. ANOVA and Tukey HSD test. *, *P* < 0.05. **c** Changes in the expression of FOXF2 during osteoblast differentiation as determined at the protein level with Western blot analysis. **d** *Foxf2* knockdown efficiency. ST2 cells were cultured and transiently transfected with siRNA-*Foxf2*. Among the siRNAs tested, #2 resulted in the greatest reduction in *Foxf2* mRNA expression. *, *P* < 0.05. **e** Effect of *Foxf2* knockdown on osteoblastic differentiation. ST2 cells were cultured and transiently transfected with siRNA-*Foxf2*. Note the significant increase in osteoblast differentiation markers in the *Foxf2* knockdown cells. *, *P* < 0.05. **f** *Foxf2* knockdown enhanced the osteogenesis of ST2 cells. Representative images of ALP staining of the ST2 cells transfected with siRNA-*Foxf2*. *N* = 3. **g** Effect of *Foxf2* overexpression on osteoblast differentiation. ST2 cells were cultured and transiently transfected with *Foxf2*. Note the significant decrease in osteoblast differentiation markers in the *Foxf2*-expressing cells. *, *P* < 0.05.

To investigate the genes involved in MSC differentiation into osteoblasts, we first attempted to identify the genes expressed in this process, particularly those genes whose expression changes during osteoblast differentiation. To this end, we administered BMP-2 to pluripotent ST2 mesenchymal progenitors for 2 days, an established model for studying osteoblast differentiation^22^, and analyzed gene expression by RNA-seq. *Alp* and *Sp7*, markers of osteoblast differentiation, showed upregulated expression with BMP-2 treatment, confirming that the experiment was conducted correctly (Fig. 1a). To identify genes that are expressed in undifferentiated MSCs and whose expression is increased by osteogenic differentiation stimulation, we investigated genes with FPKM >10 on Day 0 and whose expression was increased by BMP-2 treatment. Among the genes whose expression increased more than 2-fold by the stimulation of osteoblast differentiation, *Kazald1*, *Hpgd*, and *Smad6* have been previously reported to be associated with osteoblast differentiation and/or activity (Fig. 1a)^23–25^. Accordingly, among the genes whose function in osteoblast differentiation is not well understood, we selected *Foxf2* as the gene with the highest fold change (Fig. 1a). Then, to investigate the changes in *Foxf2* expression during MSC differentiation into osteoblasts, we administered BMP-2 to ST2 cells for seven days and analyzed gene expression by qPCR analysis. While the expression of *Alp*, an osteoblastic differentiation marker, was increased by BMP-2 administration over time, *Foxf2* expression was transiently increased on Day 3 and then decreased on Day 7 (Fig. 1b). The protein levels of FOXF2 were also transiently increased on Day 3 during BMP2-induced osteoblast differentiation (Fig. 1c). The change in the expression of *Foxf2* during osteoblastic differentiation of ST2 cells prompted us to investigate whether *Foxf2* regulates osteoblastic differentiation of MSCs. First, we tested which siRNA would most effectively reduce *Foxf2* mRNA expression. SiRNA#2 resulted in the greatest decrease in the expression of *Foxf2*, so we selected siRNA#2 for use in the following experiments (Fig. 1d). Regarding osteoblast differentiation, knockdown of *Foxf2* significantly induced osteoblastic differentiation of ST2 cells, as demonstrated by the increase in *Alp* and *Bglap* expression (Fig. 1e). We then investigated the effect of *Foxf2* knockdown on the osteogenic potential of ST2 cells by performing ALP staining. *Foxf2* knockdown increased ALP staining compared to the control treatment (Fig. 1f). Since inhibition of *Foxf2* accelerated osteoblastic differentiation, we next investigated whether increasing *Foxf2* expression would inhibit their differentiation. In the transient DNA transfection assay, *Foxf2* repressed the osteoblastic differentiation of ST2 cells (Fig. 1g). Collectively, these results demonstrate that *Foxf2* physiologically regulates the osteoblastic differentiation of MSCs.

### *Foxf2* deficiency in osteoblast progenitors results in high bone volume in vivo

**Fig. 2:**
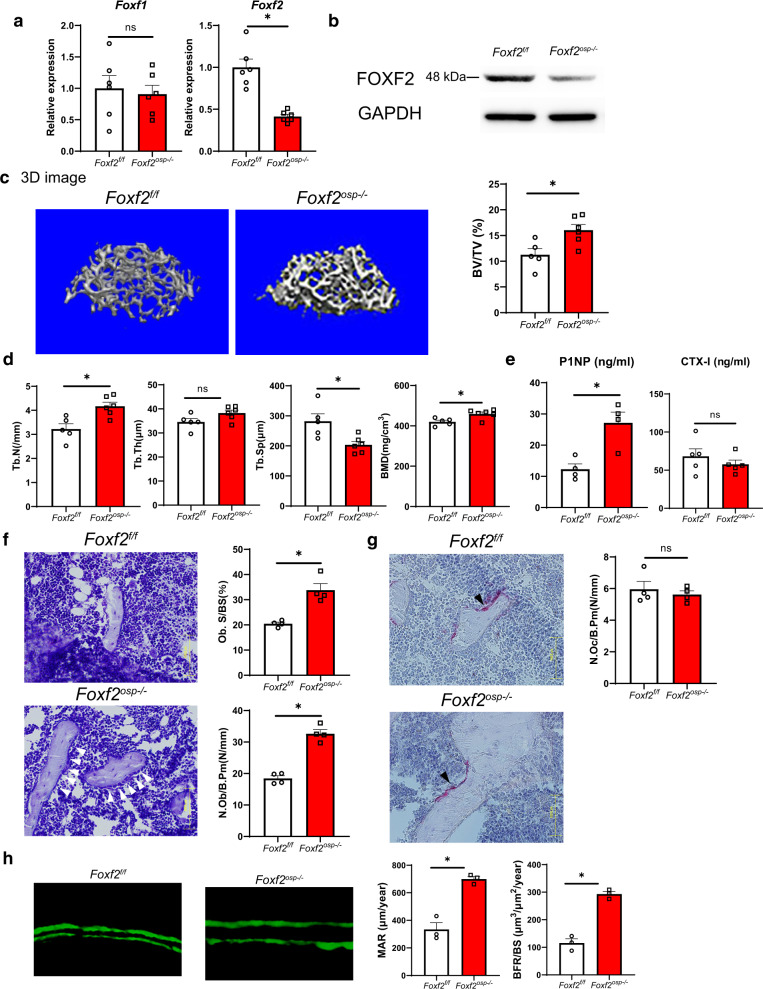
*Foxf2* deficiency in osteoblast progenitors results in high bone volume in vivo. **a** Gene expression of *Foxf2*^f/f^ and *Foxf2*^*osp*−/−^ mouse calvaria (P2). The expression of *Foxf2* was significantly decreased in the *Foxf2*^*osp*−/−^ mice, while no significant difference was observed in *Foxf1*, as demonstrated by qPCR analysis. *, *P* < 0.05 versus the *Foxf2*^f/f^ mice. ns, not significant. **b** FOXF2 protein levels in the *Foxf2*^*osp*−/−^ mice based on Western blot analysis. Calvarial bones were isolated from the *Foxf2*^*f/f*^ (control) or *Foxf2*^*osp*−/−^ mice. **c** Micro-CT analysis of 3-month-old *Foxf2*^*osp*−/−^ and *Foxf2*^f/f^ mice. Representative 3D images of femoral trabecular bone from both groups are shown. *, *P* < 0.05 versus the *Foxf2*^f/f^ mice. **d** Micro-CT analysis of 3-month-old *Foxf2*^*osp*−/−^ and *Foxf2*^f/f^ mice. *, *P* < 0.05 versus the *Foxf2*^f/f^ mice. **e** Serum P1NP and CTX-I levels measured in 3-month-old female mice. P1NP was increased in the *Foxf2*^*osp*−/−^ mice. *, *P* < 0.05 versus the *Foxf2*^f/f^ mice. ns, not significant. (**f** and **g**) Bone histomorphometric analysis of 3-month-old *Foxf2*^*f/f*^ and *Foxf2*^*osp−/−*^ mice. The number of osteoblasts was increased in the *Foxf2*^*osp−/−*^ mice, whereas the number of osteoclasts was not affected. Representative images of toluidine blue staining (**f**) and TRAP staining (**g**). White arrowheads show osteoblasts, and black arrows show osteoclasts. Scale bars, 100 μm. *, *P* < 0.05 versus the *Foxf2*^*f/f*^ mice. ns not significant. **h** Representative images of calcein labeling. *, *P* < 0.05 versus the *Foxf2*^f/f^ mice. All data represent the mean ± SEM.

To investigate the role of *Foxf2* in bone metabolism, we generated conditional mesenchymal osteoblast progenitor-specific *Foxf2* knockout mice because germline *Foxf2*-null mice die shortly after birth^26^. To achieve this, we crossed *Foxf2*^f/f^ mice with *Prx1*-Cre mice to generate *Foxf2*^*osp*−/−^ mice. *Prx1* is expressed in mesenchymal osteoblast progenitors in limb buds and the craniofacial mesenchyme^27^. *Prx1*^+^ cells have also been reported as mesenchymal osteoblast progenitors residing in both the periosteum of cortical bone and the bone marrow in adult mice^28–30^. *Foxf2*^*osp*−/−^ mice were born at the expected Mendelian ratio, indicating that embryonic development can proceed without *Foxf2* expression in mesenchymal osteoblast progenitors in the limb buds and craniofacial mesenchyme. Before analyzing the *Foxf2*^*osp*−/−^ mice, we confirmed that the *Prx1*-cre transgene did not result in a notable phenotype at 3 months of age (not shown). Genetic compensation, a change in the *Foxf1* expression level, was not observed upon deletion of *Foxf2* in the mouse calvaria (Fig. 2a). The deletion of FOXF2 was confirmed by Western blotting (Fig. 2b). Micro-CT analysis of femoral trabecular bone of the 3-month-old *Foxf2*^*osp*−/−^ mice demonstrated a 43% increase in bone mass compared to that of the *Foxf2*^f/f^ mice as measured by BV/TV (Fig. 2c). Furthermore, micro-CT analysis indicated that bone mineral density and trabecular number were increased, and trabecular separation was decreased in the long bones of the *Foxf2*^*osp*−/−^ mice compared to the long bones of the *Foxf2*^f/f^ mice (Fig. 2d). Accordingly, the serum level of P1NP, a biomarker correlating with bone formation^31^, was increased in the *Foxf2*^*osp*−/−^ mice, although CTX-I, a serum bone resorption marker^31^, showed no significant difference between the two groups (Fig. 2e). In addition, toluidine blue staining of femoral sections showed an increase in the number of osteoblasts in the *Foxf2*^*osp*−/−^ mice compared to the *Foxf2*^f/f^ mice (Fig. 2f), whereas the number of osteoclasts was not affected (Fig. 2g). Last, calcein labeling revealed that the bone formation rate was significantly increased in the *Foxf2*^*osp*−/−^ mice compared to the *Foxf2*^f/f^ mice (Fig. 2h). These results indicate that *Foxf2* expression in osteoprogenitors regulates bone formation in vivo.

### Inhibition of *Foxf2* enhances the differentiation of MSCs into osteoblasts via activation of the canonical Wnt signaling pathway

**Fig. 3:**
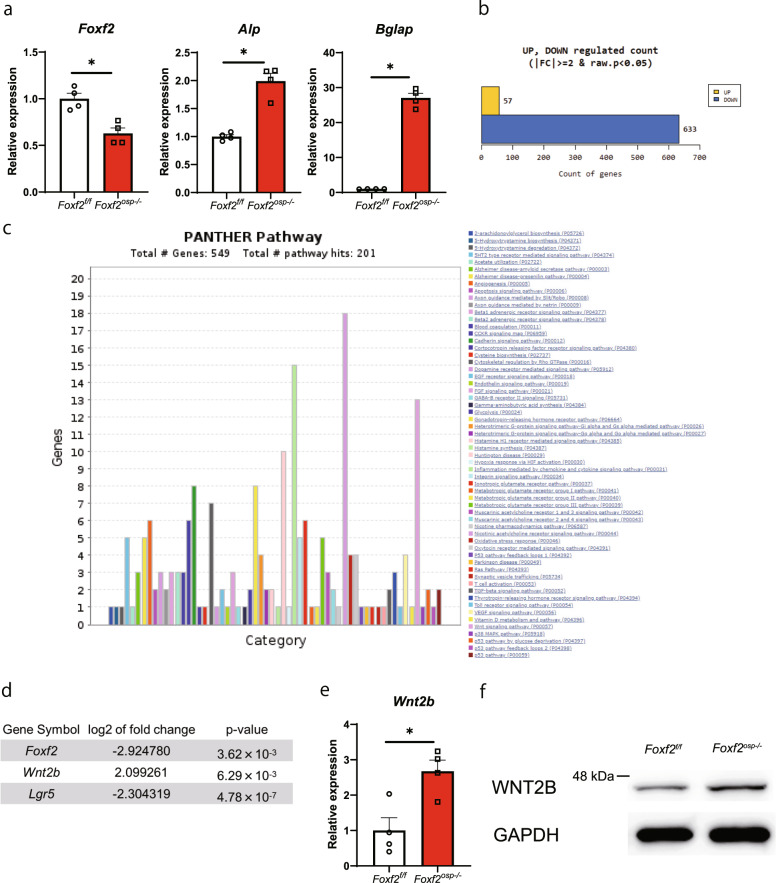
Inhibition of *Foxf2* enhances the differentiation of MSCs into osteoblasts via activation of the canonical Wnt signaling pathway. **a** Gene expression in BMSCs isolated from the *Foxf2*^f/f^ and *Foxf2*^*osp*−/−^ mice by qPCR. *Foxf2*^*osp*−/−^ BMSCs showed a significant increase in osteogenic markers. *, *P* < 0.05 versus the *Foxf2*^f/f^ mice. **b**, **c**, and d RNA-seq data from calvaria of the *Foxf2*^f/f^ and *Foxf2*^*osp*−/−^ mice. **b** Loss of *Foxf2* in osteoprogenitors significantly upregulated the expression of 57 genes while downregulating the expression of 633 genes. **c** Bar chart of pathways with potentially differentially expressed genes extracted from calvarial bone expression data. The PANTHER server with default parameters for pathway analysis was used for pathway analysis. The length of each bar shows how many genes have been assigned to a given pathway. **d** There were significant changes in the expression of *Wnt2b* and *Lgr5*, which are associated with canonical Wnt signaling. **e** Gene expression in calvaria bones isolated from the *Foxf2*^*f/f*^ and *Foxf2*^*osp*−/−^ mice by qPCR. *Foxf2*^*osp−/−*^ calvaria showed a significant increase in *Wnt2b* expression. **f** WNT2B protein levels in the *Foxf2*^*osp*−/−^ mice based on Western blot analysis. Calvaria bones were isolated from the *Foxf2*^*f/f*^ (control) or *Foxf2*^*osp*−/−^ mice. *, *P* < 0.05 versus the *Foxf2*^f/f^ mice. All data represent the mean ± SEM.

To determine whether the increase in bone formation in the *Foxf2*^*osp*−/−^ mice was cell-autonomous, we isolated BMSCs from the *Foxf2*^f/f^ and *Foxf2*^*osp*−/−^ mice. In accordance with the in vivo observations, the *Foxf2*^*osp*−/−^ BMSCs showed an increase in osteoblastic differentiation, as demonstrated by an increase in the expression of osteoblast markers (Fig. 3a). To understand the mechanistic role of *Foxf2* in osteoprogenitors, we performed an in-depth analysis of the effect of *Foxf2* deficiency on the genomic transcriptional network of osteoprogenitors. Whole-transcriptome RNA-seq was performed on calvarial bones derived from either the *Foxf2*^f/f^ or *Foxf2*^*osp*−/−^ mice. RNA-seq analysis revealed that loss of *Foxf2* in osteoprogenitors significantly upregulated the expression of 57 genes while downregulating the expression of 633 genes based on fold change and p value of the comparison pairs (Fig. 3b and Supplementary Tables 4, 5 and 6). To identify target genes of *Foxf2*, we relied on a computational approach using an established database (PANTHER database). The PANTHER pathway analysis revealed altered expression of many genes associated with inflammation mediated by the chemokine and cytokine signaling pathway, nicotinic acetylcholine receptor signaling pathway, and Wnt signaling pathway in the comparison between the *Foxf2*^*osp*−/−^ and *Foxf2*^f/f^ mice (Fig. 3c). Among the many pathways predicted to be potential pathways by the PANTHER database, we focused on the Wnt signaling pathway because that pathway has an important role in osteoblast differentiation and proliferation^4^. Indeed, several of the genes with upregulated or downregulated expression are associated with canonical Wnt signaling, e.g., *Wnt2b* and *Lgr5* (Fig. 3d). We then validated the results of RNA-seq analysis by qPCR using RNAs from the *Foxf2*^*osp*−/−^ and *Foxf2*^f/f^ mouse calvaria. The expression of *Foxf2* was downregulated and that of *Wnt2b* was upregulated in the *Foxf2*^*osp*−/−^ mouse calvaria, reproducing the results of RNA-seq analysis (Figs. 2a and 3e). Furthermore, the increase in WNT2B protein levels in the *Foxf2*^f/f^ mouse calvaria was confirmed by Western blotting (Fig. 3f).

### *Wnt2b* is a molecular target of *Foxf2* in MSCs

**Fig. 4:**
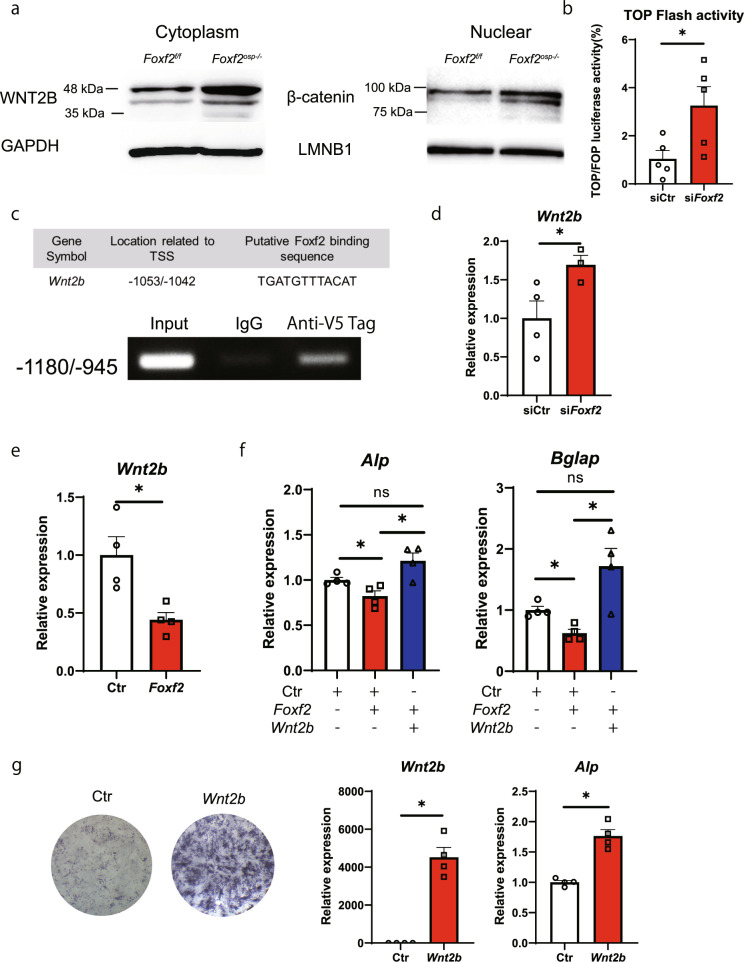
*Wnt2b* is a molecular target of *Foxf2* in MSCs. **a** Protein levels in the *Foxf2*^f/f^ and *Foxf2*^*osp*−/−^ mouse BMSCs. Note the increase in β-catenin and WNT2B protein levels in the *Foxf2*^*osp*−/−^ mice. **b** ST2 cells were transfected with TOPFlash or FOPFlash and siRNA as indicated. TOPFlash activity was increased in the *Foxf2* knockdown cells. *, *P* < 0.05 versus the control. **c** The binding of FOXF2 protein to the *Wnt2b* promoter regions containing the putative *Foxf2* binding sequence was assessed by ChIP PCR in the ST2 cells transfected with PSF-CMV-PURO-NH2-V5-Foxf2 using an anti-V5 antibody or IgG control. **d** Knockdown of *Foxf2* increased the expression of *Wnt2b* in ST2 cells. *, *P* < 0.05 versus the control. **e** Transient overexpression of *Foxf2* decreased *Wnt2b* expression in ST2 cells. *, *P* < 0.05 versus the control. **f** *Wnt2b* rescued the inhibitory effect of *Foxf2* on osteoblast differentiation. ST2 cells overexpressing *Foxf2* were cotransfected with either the *Wnt2b*-expressing plasmid or control plasmid. Subsequently, the expression of osteoblast differentiation markers was analyzed by qPCR. Note that the inhibitory effect of *Foxf2* overexpression on osteoblast differentiation was reversed by coexpression of Wnt2b. *, *P* < 0.05. ns, not significant. **g** Transient overexpression of *Wnt2b* promoted osteoblast differentiation in ST2 cells. Representative images of ALP staining of the ST2 cells transfected with *Wnt2b* or negative control (left). Note the significant increase in osteoblast differentiation markers in the *Wnt2b*-expressing cells by qPCR (right). All data represent the mean ± SEM.

Pathway analysis and the significant increase in *Wnt2b* expression suggest that the Wnt pathway is enhanced in the osteoprogenitors of the *Foxf2*^*osp*−/−^ mice. As expected, the deletion of *Foxf2* increased the cytoplasmic expression of WNT2B and nuclear expression of β-catenin (Fig. 4a). Furthermore, TOPFlash activity was increased by 210% in the ST2 cells with *Foxf2* knockdown (Fig. 4b). Collectively, these results indicate that *Foxf2* acts upstream of the canonical Wnt signaling pathway to repress the osteoblastic differentiation of MSCs. Since *Wnt2b* is a candidate target of *Foxf2*, we investigated whether *Wnt2b* is a direct target gene of *Foxf2*. ChIP-PCR analysis showed that the FOXF2 protein bound to the *Wnt2b* promoter regions containing the putative *Foxf2* binding sequence in ST2 cells (Fig. 4c). In addition, knockdown of *Foxf2* promoted *Wnt2b* expression, whereas overexpression of *Foxf2* suppressed *Wnt2b* expression in ST2 cells (Fig. 4d, e). To further test the concept of *Foxf2* as a critical regulator of Wnt signaling in MSCs, we overexpressed *Wnt2b* in the ST2 cells overexpressing *Foxf2* to rescue the effects of *Foxf2* overexpression. Indeed, the decreased *Alp and Bglap* expression upon *Foxf2* overexpression was reversed by overexpression of the *Wnt2b* plasmid in 7-day-differentiated ST2 cells, which further suggests that *Wnt2b* is a downstream mediator of *Foxf2* signaling during osteoblastic differentiation of MSCs (Fig. 4f). Finally, to investigate the importance of *Wnt2b* in osteoblast differentiation, we tested whether *Wnt2b* overexpression induced osteoblast differentiation. ALP staining and qPCR analysis showed that overexpression of *Wnt2b* promoted osteoblastic differentiation of ST2 cells (Fig. 4g). Collectively, our results suggest that *Foxf2* represses the osteoblastic differentiation of MSCs via the Wnt2b/β-catenin signaling pathway, at least in part.

### Knockdown of *Foxf2* promotes bone formation in vivo, and inhibition of *FOXF2* promotes osteoblastic differentiation of human MSCs

**Fig. 5:**
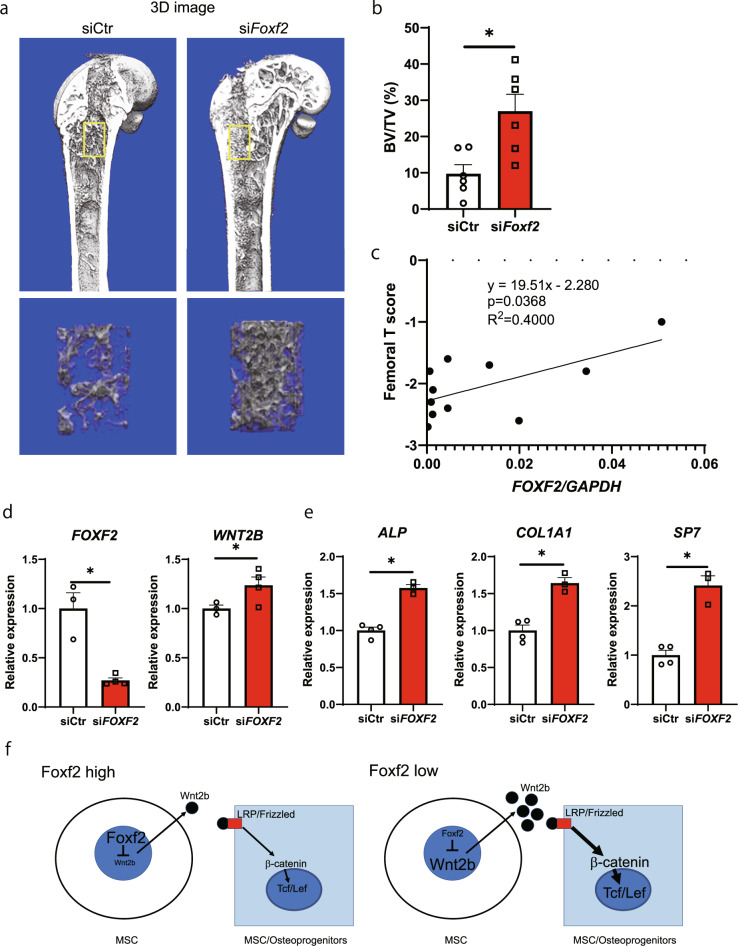
Knockdown of *Foxf2* promotes bone formation in vivo, and inhibition of *FOXF2* promotes osteoblastic differentiation of human MSCs. **a** Representative micro-CT images of bone regeneration after femoral bone marrow ablation in the negative control and si*Foxf2*-treated mice at three months. **b** Bone volume/tissue volume (BV/TV; %). Measurement areas are indicated in (**a**). *, *P* < 0.05. **c** Correlation analysis demonstrated that *FOXF2* mRNA expression was decreased along with the decreasing hip bone mineral density levels in postmenopausal female patients with low bone mass. *n* = 11. **d** Knockdown of *FOXF2* increased the expression of *WNT2B* in human BMSCs. *, *P* < 0.05. **e** Effect of *FOXF2* knockdown on the osteoblastic differentiation of human BMSCs. Note the significant increase in osteoblast differentiation markers in the *FOXF2* knockdown cells. *, *P* < 0.05. **f** A proposed mechanism through which Foxf2 in MSCs inhibits Wnt signaling in osteoprogenitors. All data are expressed as the mean ± SEM.

To address the functional role of *Foxf2* in vivo, we ablated the bone marrow of mouse femurs and then treated them with siRNA-*Foxf2* or control scrambled siRNA. Treatment with siRNA-*Foxf2* enhanced bone regeneration compared with that of the mice treated with control scrambled siRNA at Day 7 after bone marrow ablation (Fig. 5a, b and Supplementary Fig. 1). These results indicate that *Foxf2* also represses the bone regeneration process in adult mice.

To investigate the importance of *FOXF2* expression in humans, we then investigated the correlation between *FOXF2* expression in bone samples of patients with low bone mass (T score ≤1.0) undergoing spinal surgery and bone mass. As a result, *COL1A1* mRNA expression in bone tissue was shown to have a significant correlation with *ALP* mRNA expression and serum P1NP concentration (*p* = 0.01 and 0.01, respectively). Interestingly, *FOXF2* expression was correlated with femoral bone mineral density (*r* = 0.63, *p* = 0.04) (Fig. 5c and Tables 1 and 2). Finally, to investigate the clinical importance of modulating *FOXF2* expression in bone formation, we knocked down *FOXF2* in human MSCs. The expression of *WNT2B* was increased by 30% by suppressing *FOXF2* (Fig. 5d). Furthermore, *FOXF2* knockdown significantly promoted the differentiation of MSCs into osteoblasts, as the expression of *ALP*, *COL1A1*, and *SP7* was increased after seven days of culture (Fig. 5e). These results suggest that *FOXF2* represses the differentiation of MSCs into osteoblasts via the Wnt pathway not only in mice but also in humans.

**Table 1:** Correlations between *FOXF2* expression in bone tissue and other parameters.

	*ALP* mRNA	*COL1A1* mRNA	*FOXF2* mRNA	Serum P1NP	Serum TRACP-5b	Age	Femoral T score
*ALP* mRNA	1	0.74	−0.06	0.33	0.14	−0.56	−0.54
*COL1A1* mRNA	0.74	1	−0.12	0.71	0.42	−0.19	−0.43
*FOXF2* mRNA	−0.06	−0.12	1	0.28	0.44	−0.19	0.63
Serum P1NP	0.33	0.71	0.28	1	0.54	0.13	0.08
Serum TRACP-5b	0.14	0.42	0.44	0.54	1	0.04	0.04
Age	−0.56	−0.19	−0.19	0.13	0.04	1	−0.10
Femoral T score	−0.54	−0.43	0.63	0.08	0.04	−0.10	1

The Pearson correlation coefficient is shown.

*ALP* alkaline phosphatase, *COL1A1* alpha-1 type I collagen, *P1NP* procollagen I N-terminal peptide, *TRACP-5b* tartrate-resistant acid phosphatase-5b.

**Table 2:** Statistical significance of correlations between *FOXF2* expression in bone tissue and other parameters.

	*ALP* mRNA	*COL1A1* mRNA	*FOXF2* mRNA	Serum P1NP	Serum TRACP-5b	Age	Femoral T score
*ALP* mRNA		0.01*	0.85	0.32	0.68	0.07	0.08
*COL1A1* mRNA	0.01*		0.72	0.01*	0.20	0.57	0.19
*FOXF2* mRNA	0.85	0.72		0.41	0.18	0.58	0.04*
Serum P1NP	0.32	0.01*	0.41		0.08	0.70	0.81
Serum TRACP-5b	0.68	0.20	0.18	0.08		0.91	0.90
Age	0.07	0.57	0.58	0.70	0.91		0.78
Femoral T score	0.08	0.19	0.04*	0.81	0.90	0.78	

The significance of Pearson correlation coefficient analysis is shown.

*ALP* alkaline phosphatase, *COL1A1* alpha-1 type I collagen, *P1NP* procollagen I N-terminal peptide, *TRACP-5b* tartrate-resistant acid phosphatase-5b.

*P < 0.05.

## Discussion

The differentiation of MSCs into osteoblasts is essential in bone remodeling, especially bone formation^32^. From a clinical point of view, reduced bone formation has a profound impact on the development of osteoporosis and nonunion after fracture^33,34^. In this study, we found that *Foxf2* was one of the top genes with upregulated expression during MSC osteoblast differentiation, and therefore, we investigated the function of *Foxf2* during this process. Overexpression of *Foxf2* in MSCs inhibited their osteoblastic differentiation, and conversely, knockdown of *Foxf2* expression promoted osteoblast differentiation. Osteoprogenitor-specific *Foxf2* knockout mice developed a high bone mass phenotype due to increased bone formation. RNA-seq analysis and molecular experiments identified *Wnt2b* as a target of *Foxf2*. Knockdown of *Foxf2* in the mouse femur enhanced bone regeneration in vivo. *FOXF2* expression was correlated with femoral bone mineral density in postmenopausal women with low bone mass. Finally, inhibition of *FOXF2* promoted the osteoblastic differentiation of human MSCs. Collectively, *Foxf2* plays a crucial role in the differentiation of MSCs into osteoblasts (Fig. 5f).

Deletion of *Foxf2* in osteoprogenitors in mice resulted in a high bone volume phenotype, which is characterized by increased bone formation. The depletion of *Foxf2* in the *Foxf2*^*osp*−/−^ mouse model is restricted to multipotent mesenchymal progenitors in the appendicular skeleton^29^, suggesting that *Foxf2* affects bone mass by regulating the osteoblastic differentiation of multipotent mesenchymal progenitors. Furthermore, our in vitro assessment showed that the ST2 cells transfected with siRNA-*Foxf2* and *Foxf2*^*osp*−/−^ MSCs were prone to differentiate into osteoblasts, suggesting that regulation of *Foxf2* expression in MSCs is vital in regulating their differentiation into osteoblasts. Of note, a previous study showed that forced expression of *Foxf2* mildly stimulates adipocyte differentiation^35^, suggesting that the expression of *Foxf2* may be important in the determination of MSC differentiation into osteoblasts or adipocytes.

*Foxf2* suppression was shown to activate Wnt signaling^36^, but the detailed mechanism of this effect has not been elucidated. In addition, *WNT2B* was shown to promote osteoblast differentiation of human MSCs^37^. Our study indicated that deletion of *Foxf2* increases *Wnt2b* expression and that *Foxf2* binds directly to the *Wnt2b* promoter in ST2 mesenchymal progenitors. Biochemical assays suggested that the FOXF2 protein contains an activation domain and may function as a transcriptional activator^38^. However, more recent studies, such as the recent findings that *Foxf2* mediated transcriptional repression of target genes during Tgfβ-induced epithelial-mesenchymal transition^39^ and *FOXF2* recruited nuclear receptor corepressor1 (*NCoR1*) and histone deacetylase 3 to the *FOXQ1* promoter to inhibit its translation in BLBC cells^40^, suggest that *Foxf2* may act as both activator and repressor^14,41^. RNA-seq analysis showed that loss of *Foxf2* in osteoprogenitors significantly upregulated the expression of 57 genes while downregulating the expression of 633 genes. Since *Foxf2* deficiency reduced the expression of many genes, *Foxf2* may basically act to activate gene expression. However, it may be important for the osteoblastic differentiation of MSCs that *Foxf2* binds directly to the *Wnt2b* promoter and acts to directly or indirectly repress *Wnt2b* expression. Gene regulation by members of the FOX family is not only achieved through cell-specific expression but is also fine-tuned through myriad post-translational modifications and interactions with specific cofactors, such as *NCoR1*, *NCoR2, and Groucho*^41^. Our study showed that FOXF2 binds directly to the promoter of *Wnt2b*, but it is still unclear what corepressor cooperates with FOXF2 to reduce *Wnt2b* expression. Accordingly, the detailed molecular mechanisms that mediate the activator and repressor functions of *Foxf2* in vivo need to be clarified in the future. Interestingly, although genetic defects in *Foxf2* are involved in the development of cleft palate^42^, overexpression of *Wnt2b* has also been reported to cause cleft palate in vivo^43^. Therefore, modulating *Wnt2b* expression may lead to cleft palate treatment caused by *Foxf2* gene defects.

We found that knockdown of *Foxf2* enhanced bone regeneration compared with that of the mice treated with negative control siRNA at Day 7 after bone marrow ablation. Bone marrow ablation induces a sequence of events, including forming a blood clot with inflammation and capillary invasion, mesenchymal cell migration, osteoblast proliferation, and new trabecular medullary bone formation, followed by osteoclastic bone resorption and bone marrow reconstitution^44^. Of these, the bone formation phase occurs between 6 and 10 days after surgery^44^. Therefore, the increase in bone mass seven days after surgery indicates that the local administration of si*Foxf2* promoted bone formation. Thus, expanding on our research, local administration of a *FOXF2* inhibitor to a fractured site may promote bone formation, leading to accelerated fracture healing.

The correlation analysis revealed that *FOXF2* expression in bone tissue showed a significant correlation with femoral bone mineral density in postmenopausal women with a low bone mass. Given that *Foxf2* is a transcription factor that represses the differentiation of MSCs into osteoblasts, it is interesting that *FOXF2* expression in bone tissue was lower in patients with lower bone mass. Furthermore, based on the results that mice lacking *Foxf2* in osteoprogenitors exhibit high bone mass, there is a discrepancy between human and mouse results. We speculate that this discrepancy may be due to differences in genetic background and the fact that the mice analyzed were young (3 months old), while the humans analyzed were elderly females. In a study investigating changes in gene expression in postmenopausal and premenopausal females, 29 of the 118 genes investigated were significantly altered^45^. Accordingly, the regulation of *FOXF2* expression by estrogen will need to be investigated in the future. Of note, serum P1NP concentration, a marker of bone formation, was shown to be negatively correlated with the bone mineral density of postmenopausal females with low bone mass^46–48^. These results suggest that bone formation was enhanced in inverse proportion to bone mass in postmenopausal women with low bone mass. Consistent with the results of previous studies, *ALP* and *COL1A1* expression in bone tissue, markers of osteoblastic differentiation, tended to show negative correlations with femoral bone mineral density in our study, although these relationships were not significant, possibly due to the small sample size (*p* = 0.08 and 0.19). Collectively, these results suggest that in postmenopausal women with low bone mass, a mechanism may be already in operation that suppresses the expression of *FOXF2*, a repressor of bone formation, to promote bone formation in response to low bone mineral density. How *FOXF2* expression is regulated in postmenopausal women needs to be clarified in the future. Furthermore, it will be necessary to compare the expression level of *FOXF2* in premenopausal female and male bone tissues with bone mineral density in the future.

The fact that *FOXF2* expression in bone tissue showed a stronger association with femoral bone mineral density than the serum concentration of TRACP-5b and P1NP, the representative bone turnover markers currently in use, suggests that examining the expression of *FOXF2* in bone tissue may be a sensitive indicator of osteoporosis. Furthermore, if the expression of *FOXF2* can be systematically reduced by medication to promote bone formation, this strategy may be helpful in the treatment of bone-related diseases such as osteoporosis. Note that since the sample size of patient specimens in this study was small, the possibility of a type II error cannot be excluded. Considering future clinical applications, validation with larger sample sizes is essential, and further research is needed.

In conclusion, we demonstrated that *Foxf2* represses osteoblastic differentiation of MSCs. Thus, from a clinical perspective, systemic or local suppression of *FOXF2* expression may be a promising strategy for treating bone-related diseases such as osteoporosis and fractures.

## Supplementary information

Supplemental figure and Tables

## Data Availability

The results of the RNA-seq analysis are shown in Supplementary Tables 4, 5, and 6.
