# Supplementary material for: Foxf2 represses bone formation via Wnt2b/β-catenin signaling
Source: Exp Mol Med. 2022 Jun 6;54(6):753–64. doi: 10.1038/s12276-022-00779-z (PMC9256714; doi:10.1038/s12276-022-00779-z)
Supplement: Supplementary file 1 — Supplemental figure and Tables [file 12276_2022_779_MOESM1_ESM.pdf]

Supplementary figure 1

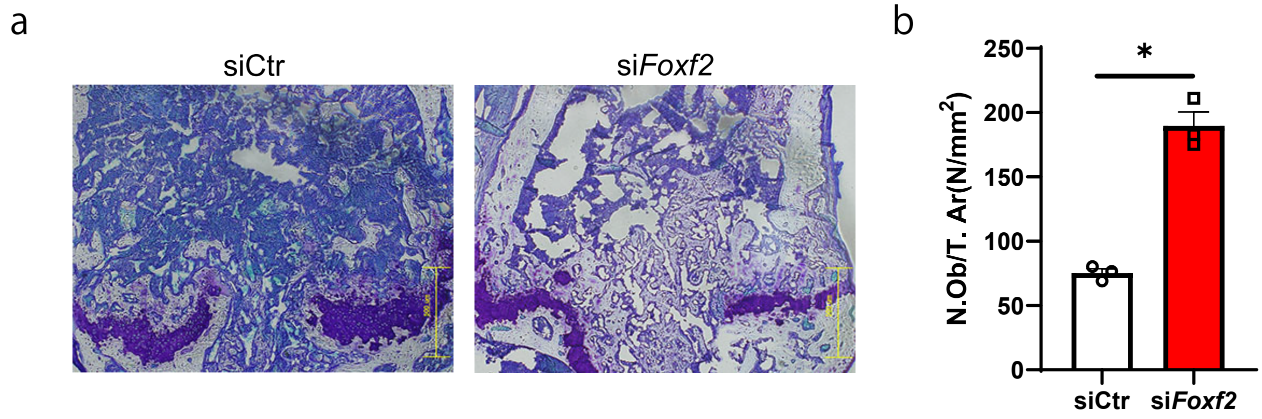

Supplementary Figure 1. (a)Representative images of toluidine blue staining after femoral bone marrow ablation in negative control and si*Foxf2* treated mice at 3 months. (b)The number of osteoblasts was increased in siFoxf2 treated mice. *, $p < 0.05$ versus control. All data represent the mean ± SEM.

Supplementary Tables

Supplementary Table 1.

siRNA used in this study

siRNA	Target sequences
siFoxf2#1	CAUCAUCACCAGAGCGUGU
siFoxf2#2	GGAGUUCUGCUCACCGAUA
siFoxf2#3	ACACUAGCCCUGCGGCACA
siFoxf2#4	UAAUAUCCCAGAAGAGCGA

Supplementary Table 2.

qPCR Primers used in this study

Genes	Primer sequences	
Gapdh	Forward:	AGGTCGGTGTGAACGGATTTG
	Reverse:	GGGGTCGTTGATGGCAACA
Alpl	Forward:	ATCTTTGGTCTGGCTCCCATG
	Reverse:	TTTCCCGTTCACCGTCCAC
Bglap	Forward:	GCAATAAGGTAGTGAACAGACTC
	Reverse:	GTTTGTAGGCGGTCTTCAAGC
Foxf1	Forward:	CTACATCAAGCAACAGCCTC
	Reverse:	TAAGATCCTCCGCCTGTTGT
Foxf2	Forward:	GCGCTTCACCTTACCTCAAG
	Reverse:	GGACGAAATCTTTCCTGTCG
Wnt2b	Forward:	CTGCTGCTGCTACTCCTGACT
	Reverse:	ATGTCTGGGTAGCGTTGACAC
GAPDH	Forward:	GGAGCGAGATCCCTCCAAAAT
	Reverse:	GGCTGTTGTCATACTTCTCATGG
ALPL	Forward:	AACATCAGGGACATTGACGTG

	Reverse:	GTATCTCGGTTTGAAGCTCTTCC
SP7	Forward:	CCTCTGCGGGACTCAACAAC
	Reverse:	AGCCCATTAGTGCTTGTAAAGG
BGLAP	Forward:	CACTCCTCGCCCTATTGGC
	Reverse:	CCCTCCTGCTTGGACACAAAG
FOXF2	Forward:	AATGCCACTCGCCCTACAC
	Reverse:	CGTTCTGGTGCAAGTAGCTCT
WNT2B	Forward:	CGGGACCACACCGTCTTTG
	Reverse:	GCGAGTAATAGCGTGGACTAC

Supplementary Table 3.

Primers for ChIP PCR

Genes	Primer sequences	
Wnt2b	Forward:	CTTCTCTGTCTCTGAGATGTCTGC
	Reverse:	TAACTCCTTCAGCCTCAGTTTCTC

Supplementary Table 4.

Gene_ID	Transcript_ID	Gene_Symbol	Description	gene_biotype	Protein_ID	MGI	IMGT/GENE-DB	Ctr-14_FPKM	Ctr-9_FPKM	KO-11_FPKM	Ctr-1_FPKM	Ctr-2_FPKM	KO-1_FPKM	KO-2_FPKM	KO-3_FPKM	Ctr-14_TPM	Ctr-9_TPM	KO-11_TPM	Ctr-1_TPM	Ctr-2_TPM	KO-1_TPM	KO-2_TPM	KO-3_TPM	test_1/control_1.fc	test_1/control_1.baseMean	test_1/control_1.lfcSE	test_1/control_1.stat	test_1/control_1.raw.pval	test_1/control_1.bh.pval	control_1.mean	test_1.mean	N_KO-1	N_KO-2	N_KO-3	N_KO-11	N_Ctr-1	N_Ctr-2	N_Ctr-9	N_Ctr-14	KO-1_Read_Count	KO-2_Read_Count	KO-3_Read_Count	KO-11_Read_Count	Ctr-1_Read_Count	Ctr-2_Read_Count	Ctr-9_Read_Count	Ctr-14_Read_Count
11287	NM_007376	Pzp	PZP, alpha-2-macroglobulin like	protein_coding	NP_031402.3	87854	.	0.000000	0.000000	0.000000	0.000000	0.006489	0.000000	0.000000	0.000000	0.000000	0.000000	0.000000	0.000000	0.017086	0.000000	0.000000	0.000000	-2.584344	0.222360	3.53133956	-0.387897547	0.698091854	NA	-1.169033	-Inf	-1.725323	-1.725448	-1.725389	-1.725324	-1.725457	-1.718206	-1.725125	-1.725376	0	0	0	0	0	2	0	0
11298	NM_009591,NR_033223,XM_030245473,XM_030245474	Aanat	arylalkylamine N-acetyltransferase	protein_coding	NP_033721.1;XP_030101333.1;XP_030101334.1	1328365	.	0.000000	0.000000	0.026809	0.035355	0.034202	0.000000	0.069876	0.030934	0.000000	0.000000	0.072164	0.094774	0.090049	0.000000	0.188399	0.084095	1.973327	2.766197	1.669834247	0.587262041	0.557027731	NA	0.884542	1.882140	1.436557	1.494907	1.467112	1.460359	1.466056	1.464048	1.436814	1.436491	0	8	4	3	4	4	0	0
11302	NM_001198785,NM_001198787,NM_007377,XM_006531991,XM_006531992,XM_006531993,XM_006531994,XM_006531995,XM_030245475,XR_003949229	Aatk	apoptosis-associated tyrosine kinase	protein_coding	NP_001185714.1;NP_001185716.1;NP_031403.2;XP_006532054.1;XP_006532055.1;XP_006532056.1;XP_006532057.1;XP_006532058.1;XP_030101335.1	1197518	.	0.688293	0.980700	0.676366	2.612958	1.055361	0.972960	0.440915	0.646613	1.810734	2.604030	1.820624	7.004415	2.778609	2.665318	1.188781	1.757812	-1.839122	256.915029	0.385643092	-2.279353189	0.022646079	0.29644958	8.378459	7.500182	7.903630	7.484872	7.667103	7.675024	8.703317	7.933507	7.873874	7.690031	239	134	175	171	689	288	206	180
11303	NM_013454,XM_006537554,XM_006537555,XM_006537556	Abca1	ATP-binding cassette, sub-family A (ABC1), member 1	protein_coding	NP_038482.3;XP_006537617.1;XP_006537618.1;XP_006537619.1	99607	.	13.661382	12.627269	13.480966	9.438054	17.137870	10.686463	9.666058	13.088888	35.939847	33.528872	36.287646	25.300079	45.121485	29.274386	26.061314	35.582003	-1.085443	5501.331738	0.17077318	-0.692634137	0.488539181	0.845903147	12.483463	12.365246	12.255689	12.211155	12.494397	12.510169	12.126593	12.725050	12.424665	12.499619	4580	4699	5938	5819	4357	8199	4824	5924
11304	NM_007378,XM_006500911,XM_006500912,XM_017319439,XM_017319440,XR_003954273,XR_375477,XR_375478	Abca4	ATP-binding cassette, sub-family A (ABC1), member 4	protein_coding	NP_031404.1;XP_006500974.1;XP_006500975.1;XP_017174928.1;XP_017174929.1	109424	.	0.495031	0.577341	0.618394	0.505896	1.149897	0.561885	0.463110	0.970182	1.302307	1.533000	1.664573	1.356128	3.027509	1.539223	1.248624	2.637428	-1.005503	217.192578	0.321395283	-0.024635979	0.980345321	0.996748559	7.766109	7.759545	7.629581	7.544000	8.013156	7.690849	7.566065	8.100005	7.642320	7.538307	179	167	322	197	174	402	164	159
11305	NM_001368624,NM_007379,XM_006497609,XM_006497610,XM_006497611,XM_006497614,XM_006497615,XM_006497616,XM_030246714,XM_030246727	Abca2	ATP-binding cassette, sub-family A (ABC1), member 2	protein_coding	NP_001355553.1;NP_031405.2;XP_006497672.1;XP_006497673.1;XP_006497674.1;XP_006497677.1;XP_006497678.1;XP_006497679.1;XP_030102574.1;XP_030102587.1	99606	.	2.400670	4.328043	3.769379	4.647630	4.756585	4.817001	3.531423	4.637800	6.315593	11.492144	10.146298	12.458650	12.523387	13.195640	9.521309	12.607813	1.057017	1490.933021	0.179033097	0.446833414	0.654995344	0.914954831	10.501384	10.581504	10.674761	10.414175	10.699499	10.436136	10.641202	10.619874	10.561977	10.139520	1662	1372	1765	1312	1729	1821	1336	995
11306	NM_009592	Abcb7	ATP-binding cassette, sub-family B (MDR/TAP), member 7	protein_coding	NP_033722.1	109533	.	3.678730	4.406568	3.950116	4.364166	4.263155	4.489401	4.087504	4.441183	9.677864	11.700648	10.632799	11.698784	11.224258	12.298219	11.020598	12.073310	1.052464	1045.925804	0.101692563	0.725428229	0.46818936	0.83409673	9.993350	10.066844	10.088223	10.054241	10.098247	9.970401	10.062490	10.003768	10.062267	9.880114	1080	1116	1131	957	1131	1145	945	895
11307	NM_009593	Abcg1	ATP binding cassette subfamily G member 1	protein_coding	NP_033723.1	107704	.	1.064530	2.316129	1.664469	1.627134	2.066724	1.492602	1.633061	1.988900	2.800524	6.149960	4.480367	4.361770	5.441380	4.088819	4.403007	5.406805	-1.004539	434.724727	0.232846916	-0.028059395	0.977614779	0.996513546	8.767530	8.760378	8.628615	8.750695	8.884851	8.723020	8.699514	8.869490	8.990883	8.355278	363	451	512	408	426	561	502	262
11308	NM_001077190,NM_001077192,NM_001077193,NM_001331043,NM_001331044,NM_001331045,NM_007380,NM_145994,XM_006497619,XM_030246741	Abi1	abl-interactor 1	protein_coding	NP_001070658.1;NP_001070660.1;NP_001070661.1;NP_001317972.1;NP_001317973.1;NP_001317974.1;NP_031406.2;NP_666106.1;XP_006497682.1;XP_030102601.1	104913	.	21.224535	18.418661	20.012612	17.607727	22.620774	19.215919	19.244456	20.748210	55.836694	48.906605	53.869332	47.200081	59.557160	52.639884	51.886292	56.403792	1.031348	2902.947423	0.095322265	0.467159939	0.640385453	0.908825683	11.480758	11.525500	11.465647	11.529980	11.566158	11.510033	11.368198	11.593068	11.413462	11.554291	2724	3100	3108	2845	2667	3558	2328	3051

[illegible]

11733	NM_001110783,NM_001277280,NM_001277281,NM_001277284,NM_001277286,NM_001277289,NM_001310436,NM_001310437,NM_031158,NR_102385,NR_102386,XM_006508989,XM_006508990,XM_006508991,XM_006508992,XM_006508993,XM_006508994,XM_006508995,XM_006508996,XM_006508997,XM_006508998,XM_006508999,XM_006509000,XM_006509001,XM_006509002,XM_006509003,XM_030243250,XM_030243251,XR_378776	Ank1	ankyrin 1, erythroid	protein_coding	[illegible]
11735	NM_009670,NM_146005,NM_170687,NM_170688,NM_170689,NM_170690,NM_170728,NM_170729,NM_170730,XM_006513122,XM_006513124,XM_006513125,XM_006513126,XM_006513127,XM_006513128,XM_006513129,XM_006513130,XM_006513133,XM_006513134,XM_006513135,XM_006513136,XM_006513137,XM_006513138,XM_006513139,XM_011243290,XM_011243291,XM_011243292,XM_011243293,XM_011243295,XM_011243297,XM_011243299,XM_011243300,XM_011243307,XM_011243308,XM_011243309,XM_017313773,XM_017313774,XM_017313775,XM_017313778,XM_017313780,XM_030244836,XM_030244837,XM_030244838,XM_030244839,XM_030244840,XM_030244841,XM_030244842,XM_030244843,XM_030244844,XM_030244845,XM_030244846,XM_030244847,XM_030244848,XM_030244849	Ank3	ankyrin 3, epithelial	protein_coding	[illegible]
11736	NM_009671,XM_030245483,XM_030245484	Ankfy1	ankyrin repeat and FYVE domain containing 1	protein_coding	[illegible]
11737	NM_009672,XM_006510778,XM_006510779	Anp32a	acidic (leucine-rich) nuclear phosphoprotein 32 family, member A	protein_coding	[illegible]
11739	NM_007450	Slc25a4	solute carrier family 25 (mitochondrial carrier, adenine nucleotide translocator), member 4	protein_coding	[illegible]
11740	NM_007451	Slc25a5	solute carrier family 25 (mitochondrial carrier, adenine nucleotide translocator), member 5	protein_coding	[illegible]
11744	NM_013469,XM_006518451	Anxa11	annexin A11	protein_coding	[illegible]
11745	NM_013470	Anxa3	annexin A3	protein_coding	[illegible]
11746	NM_001331120,NM_013471,XM_006505397,XM_006505398,XM_011241158,XM_030255115	Anxa4	annexin A4	protein_coding	[illegible]
11747	NM_009673	Anxa5	annexin A5	protein_coding	[illegible]
11749	NM_001110211,NM_013472,NR_138556	Anxa6	annexin A6	protein_coding	[illegible]
11750	NM_001110794,NM_009674,XM_006518452,XM_006518453	Anxa7	annexin A7	protein_coding	[illegible]
11752	NM_001281845,NM_013473	Anxa8	annexin A8	protein_coding	[illegible]
11754	NM_009675,XM_006532041,XM_017314230	Aoc3	amine oxidase, copper containing 3	protein_coding	[illegible]
11757	NM_007452	Prdx3	peroxiredoxin 3	protein_coding	[illegible]
11758	NM_001303408,NM_007453,NR_130150,XM_030243163	Prdx6	peroxiredoxin 6	protein_coding	[illegible]
11761	NM_009676,XM_006495630	Aox1	aldehyde oxidase 1	protein_coding	[illegible]
11764	NM_001243043,NM_001243044,NM_001355659,NM_001355660,NM_007454,XM_006514462,XM_030245485,XM_030245486,XM_030245487,XM_030245488,XM_030245489	Ap1b1	adaptor protein complex AP-1, beta 1 subunit	protein_coding	[illegible]
11765	NM_001301211,NM_009677	Ap1g1	adaptor protein complex AP-1, gamma 1 subunit	protein_coding	[illegible]
11766	NM_001303502,NM_007455	Ap1g2	adaptor protein complex AP-1, gamma 2 subunit	protein_coding	[illegible]
11767	NM_007456,XM_006509538,XM_030243252	Ap1m1	adaptor-related protein complex AP-1, mu subunit 1	protein_coding	[illegible]
11768	NM_001110300,NM_009678,XM_006509949,XM_006509950	Ap1m2	adaptor protein complex AP-1, mu 2 subunit	protein_coding	[illegible]
11769	NM_007457,XM_006504348	Ap1s1	adaptor protein complex AP-1, sigma 1	protein_coding	[illegible]
11770	NM_024406,XM_006530048	Fabp4	fatty acid binding protein 4, adipocyte	protein_coding	[illegible]
11771	NM_001077264,NM_007458,XM_006540569,XM_006540570	Ap2a1	adaptor-related protein complex 2, alpha 1 subunit	protein_coding	[illegible]
11772	NM_001357068,NM_007459,XM_006536155	Ap2a2	adaptor-related protein complex 2, alpha 2 subunit	protein_coding	[illegible]
11773	NM_001302970,NM_009679	Ap2m1	adaptor-related protein complex 2, mu 1 subunit	protein_coding	[illegible]
11774	NM_009680,XM_006517523,XR_001780749,XR_382728	Ap3b1	adaptor-related protein complex 3, beta 1 subunit	protein_coding	[illegible]
11775	NM_021492,XM_006540571	Ap3b2	adaptor-related protein complex 3, beta 2 subunit	protein_coding	[illegible]
11776	NM_007460,XM_030244850,XR_003948582	Ap3d1	adaptor-related protein complex 3, delta 1 subunit	protein_coding	[illegible]
11777	NM_001303015,NM_009681,XM_006525526,XM_006525528	Ap3s1	adaptor-related protein complex 3, sigma 1 subunit	protein_coding	[illegible]
11778	NM_009682	Ap3s2	adaptor-related protein complex 3, sigma 2 subunit	protein_coding	[illegible]
11781	NM_021392,XM_006504551,XM_030254076,XM_030254077	Ap4m1	adaptor-related protein complex AP-4, mu 1	protein_coding	[illegible]
11782	NM_001329698,NM_001329699,NM_021710,XM_006515433,XM_006515434	Ap4s1	adaptor-related protein complex AP-4, sigma 1	protein_coding	[illegible]
11783	NM_001042558,NM_001282947,NM_009684	Apaf1	apoptotic peptidase activating factor 1	protein_coding	[illegible]
11784	NM_001291166,NM_001291167,NM_007461,XM_006540573,XM_006540576,XM_017321945,XM_030242006,XM_030242007,XM_030242008,XM_030242009,XM_030242010,XM_030242011,XM_030242012,XM_030242013,XM_030242014	Apba2	amyloid beta (A4) precursor protein-binding, family A, member 2	protein_coding	[illegible]
11785	NM_001253885,NM_001253886,NM_001253887,NM_001310600,NM_009685,XM_006507230,XM_006507231,XM_006507232,XM_006507234,XM_006507235,XM_006507236,XM_017321946,XM_017321947	Apbb1	amyloid beta (A4) precursor protein-binding, family B, member 1	protein_coding	[illegible]
11787	NM_001201413,NM_001201414,NM_001201415,NM_001201416,NM_001310626,NM_009686,XM_006503688,XM_006503690,XM_006503691,XM_006503692,XM_006503693,XM_006503694,XM_006503696,XM_006503697,XM_011240693,XM_017320620,XM_017320622,XM_030254078,XM_030254079,XM_030254080,XM_030254081,XM_030254082,XM_030254083	Apbb2	amyloid beta (A4) precursor protein-binding, family B, member 2	protein_coding	[illegible]
11789	NM_001360979,NM_001360980,NM_007462,XM_006525529,XM_006525530,XM_006525532,XM_006525533,XM_006525534,XM_006525536,XM_006525538,XM_006525539,XM_030250277,XM_030250278,XM_030250279,XM_030250280,XM_030250281,XM_030250282,XM_030250283,XM_030250284,XM_030250285,XM_030250286,XM_030250287,XM_030250288,XM_030250289,XM_030250290,XM_030250291,XM_030250292,XM_030250293,XM_030250294,XM_030250295,XM_030250296	Apc	APC, WNT signaling pathway regulator	protein_coding	[illegible]
11790	NM_001085370,NM_001085371,NM_001173477,NM_007463,XM_006496401,XM_006496402,XM_006496403,XM_006496404,XM_011238643,XM_011238644,XM_011238645,XM_011238646,XM_011238647,XM_011238648,XM_011238649,XM_011238651,XM_017312276,XM_017322354,XM_017322375,XM_030243202,XM_030243273,XM_030243284,XM_030243348,XM_030243368,XM_030243390,XM_030243402,XM_030243447,XM_030243476,XM_030243494,XM_030243512,XM_030243521,XR_003947244	Speg	SPEG complex locus	protein_coding	[illegible]
11792	NM_009687	Apex1	apurinic/apyrimidinic endonuclease 1	protein_coding	[illegible]
11793	NM_001314013,NM_001358596,NM_053069,XM_011243108,XM_017313783	Atg5	autophagy related 5	protein_coding	[illegible]
11796	NM_007464,XM_006509828,XM_006509829,XM_006509830,XM_006509831,XM_011242352,XM_011242353	Birc3	baculoviral IAP repeat-containing 3	protein_coding	[illegible]
11797	NM_001291503,NM_007465,XM_017313110,XM_017313111,XM_030244029,XR_003947764	Birc2	baculoviral IAP repeat-containing 2	protein_coding	[illegible]
11798	NM_001301639,NM_001301641,NM_009688,NR_125870,XM_006541425,XM_006541427,XM_011250976,XM_011250977,XM_017318359,XM_017318360,XM_017318361,XM_017318362,XM_030251200,XM_030251201,XM_030251202,XM_030251203	Xiap	X-linked inhibitor of apoptosis	protein_coding	[illegible]
11799	NM_001012273,NM_009689	Birc5	baculoviral IAP repeat-containing 5	protein_coding	[illegible]
11800	NM_001305258,NM_001349003,NM_007466	Api5	apoptosis inhibitor 5	protein_coding	[illegible]
11801	NM_009690	Cd5l	CD5 antigen-like	protein_coding	[illegible]
11803	NM_007467	Aplp1	amyloid beta (A4) precursor-like protein 1	protein_coding	[illegible]
11804	NM_001102455,NM_001102456,NM_001357698,NM_009691,XM_006509952	Aplp2	amyloid beta (A4) precursor-like protein 2	protein_coding	[illegible]
11806	NM_009692	Apoa1	apolipoprotein A-I	protein_coding	[illegible]
11807	NM_001305549,NM_001305550,NM_001305585,NM_013474,NR_131173	Apoa2	apolipoprotein A-II	protein_coding	[illegible]
11808	NM_007468	Apoa4	apolipoprotein A-IV	protein_coding	[illegible]
11810	NM_001134391,NM_031159,XM_006505401,XM_011241159	Apobec1	apolipoprotein B mRNA editing enzyme, catalytic polypeptide 1	protein_coding	[illegible]
11811	NM_009694	Apobec2	apolipoprotein B mRNA editing enzyme, catalytic polypeptide 2	protein_coding	[illegible]
11812	NM_001110009,NM_007469	Apoc1	apolipoprotein C-I	protein_coding	[illegible]
11813	NM_001277944,NM_001309795	Apoc2	apolipoprotein C-II	protein_coding	[illegible]
11815	NM_001301353,NM_001301354,NM_007470	Apod	apolipoprotein D	protein_coding	[illegible]
11816	NM_001305819,NM_001305843,NM_001305844,NM_009696,XM_030242015	Apoe	apolipoprotein E	protein_coding	[illegible]
11818	NM_013475,XM_006532042	Apoh	apolipoprotein H	protein_coding	[illegible]
11819	NM_009697,NM_183261,XM_006540577,XM_006540578	Nr2f2	nuclear receptor subfamily 2, group F, member 2	protein_coding	[illegible]
11820	NM_001198823,NM_001198824,NM_001198825,NM_001198826,NM_007471,XM_006522873,XM_006522874	App	amyloid beta (A4) precursor protein	protein_coding	[illegible]
11821	NM_009698	Aprt	adenine phosphoribosyl transferase	protein_coding	[illegible]
11826	NM_007472	Aqp1	aquaporin 1	protein_coding	[illegible]
11827	NM_009699	Aqp2	aquaporin 2	protein_coding	[illegible]
11828	NM_016689	Aqp3	aquaporin 3	protein_coding	[illegible]
11829	NM_001308641,NM_001308642,NM_001308643,NM_001308644,NM_001308645,NM_001308646,NM_001308647,NM_001317729,NM_009700	Aqp4	aquaporin 4	protein_coding	[illegible]
11830	NM_009701	Aqp5	aquaporin 5	protein_coding	[illegible]
11831	NM_175087,XM_006520350	Aqp6	aquaporin 6	protein_coding	[illegible]
11832	NM_007473,XM_006537559,XM_006537560,XM_006537561,XM_006537562,XM_006537563,XM_017319925,XM_030253099,XM_030253100,XM_030253101,XM_030253102,XM_030253103,XM_030253104	Aqp7	aquaporin 7	protein_coding	[illegible]
11834	NM_001290788,NM_009702	Aqr	aquarius	protein_coding	[illegible]
11835	NM_013476	Ar	androgen receptor	protein_coding	[illegible]
11836	NM_001159645,NM_009703,XM_006527556,XM_006527557,XM_011247429,XM_030251204,XR_003952995	Araf	Araf proto-oncogene, serine/threonine kinase	protein_coding	[illegible]
11837	NM_007475	Rplp0	ribosomal protein, large, P0	protein_coding	[illegible]
11838	NM_001276684,NM_018790	Arc	activity regulated cytoskeletal-associated protein	protein_coding	[illegible]
11839	NM_009704	Areg	amphiregulin	protein_coding	[illegible]
11840	NM_001130408,NM_007476	Arf1	ADP-ribosylation factor 1	protein_coding	[illegible]
11841	NM_001304574,NM_007477	Arf2	ADP-ribosylation factor 2	protein_coding	[illegible]
11842	NM_001355510,NM_007478	Arf3	ADP-ribosylation factor 3	protein_coding	[illegible]
11843	NM_007479	Arf4	ADP-ribosylation factor 4	protein_coding	[illegible]
11844	NM_007480	Arf5	ADP-ribosylation factor 5	protein_coding	[illegible]
11845	NM_007481	Arf6	ADP-ribosylation factor 6	protein_coding	[illegible]
11846	NM_007482	Arg1	arginase, liver	protein_coding	[illegible]
11847	NM_009705	Arg2	arginase type II	protein_coding	[illegible]
11848	NM_001313961,NM_001313962,NM_016802	Rhoa	ras homolog family member A	protein_coding	[illegible]
11852	NM_007483	Rhob	ras homolog family member B	protein_coding	[illegible]
11853	NM_001291859,NM_007484	Rhoc	ras homolog family member C	protein_coding	[illegible]
11854	NM_001329989,NM_007485,XM_006531644	Rhod	ras homolog family member D	protein_coding	[illegible]
11855	NM_009706,XM_006515438,XM_006515439,XM_011243989,XM_011243990	Arhgap5	Rho GTPase activating protein 5	protein_coding	[illegible]
11856	NM_001287530,NM_009707,NM_178754,NR_109847,XM_006528694,XM_011247783,XR_003952996	Arhgap6	Rho GTPase activating protein 6	protein_coding	[illegible]
11857	NM_001301301,NM_001301303,NM_001301305,NM_001301308,NM_001301309,NM_007486,XM_006505404,XM_030255116,XM_030255117,XM_030255118	Arhgdib	Rho, GDP dissociation inhibitor (GDI) beta	protein_coding	[illegible]
11858	NM_009708	Rnd2	Rho family GTPase 2	protein_coding	[illegible]
11859	NM_008887	Phox2a	paired-like homeobox 2a	protein_coding	[illegible]
11861	NM_001039515,NM_007487	Arl4a	ADP-ribosylation factor-like 4A	protein_coding	[illegible]
11863	NM_001037737,NM_009709,XM_006500930,XM_006500931,XM_006500933,XM_030252391,XM_030252392,XM_030252393,XR_001783643,XR_001783644	Arnt	aryl hydrocarbon receptor nuclear translocator	protein_coding	[illegible]
11864	NM_007488,XM_006507238,XM_006507239,XM_006507240,XM_006507242,XM_006507243,XM_006507244,XM_011241655,XM_030242017	Arnt2	aryl hydrocarbon receptor nuclear translocator 2	protein_coding	[illegible]
11865	NM_001243048,NM_001357070,NM_001368412,NM_007489,XM_006507245,XM_006507246,XM_006507247,XM_006507248,XM_006507249,XM_006507250,XM_006507252,XM_006507253,XM_011241656,XM_017321950,XM_030242018,XM_030242019,XM_030242020,XM_030242021,XM_030242022,XR_001785467,XR_003946377	Arntl	aryl hydrocarbon receptor nuclear translocator-like	protein_coding	[illegible]
11867	NM_023142	Arpc1b	actin related protein 2/3 complex, subunit 1B	protein_coding	[illegible]
11870	NM_009710,XM_011241657,XR_869649	Art1	ADP-ribosyltransferase 1	protein_coding	[illegible]
11871	NR_033804	Art2a	ADP-ribosyltransferase 2a	transcribed_pseudogene	[illegible]
11875	NM_001291354,NM_007491,NR_111935,XM_006507258,XM_006507259,XM_006507260,XM_006507261,XM_006507262,XM_006507263,XM_030242023,XM_030242024,XM_030242025	Art5	ADP-ribosyltransferase 5	protein_coding	[illegible]
11876	NM_001284191,NM_001284192,NM_001284193,NM_009711,XM_006502690,XM_006502691,XM_006502692,XM_006502693,XM_006502694,XM_011240419,XM_030253105	Artn	artemin	protein_coding	[illegible]
11877	NM_001272028,NM_001272029,NM_001272030,NM_001272031,NM_001272032,NM_033474,XM_011245807,XM_017316854,XM_030248935,XR_001781781,XR_001781782	Arvcf	armadillo repeat gene deleted in velocardiofacial syndrome	protein_coding	[illegible]
11878	NM_001305940,NM_007492	Arx	aristaless related homeobox	protein_coding	[illegible]
11881	NM_009712	Arsb	arylsulfatase B	protein_coding	[illegible]
11883	NM_009713	Arsa	arylsulfatase A	protein_coding	[illegible]
11886	NM_019734,XM_017312527,XM_017312528,XM_017312530,XM_017312531,XM_017312532,XM_017312533,XM_017312534,XM_017312535,XM_017312536,XM_030243253,XM_030243254,XM_030243255,XM_030243256	Asah1	N-acylsphingosine amidohydrolase 1	protein_coding	[illegible]
11889	NM_001291131,NM_001291132,NM_009714,XM_017314231	Asgr1	asialoglycoprotein receptor 1	protein_coding	[illegible]
11890	NM_001313925,NM_001313926,NM_001313927,NM_007493	Asgr2	asialoglycoprotein receptor 2	protein_coding	[illegible]
11891	NM_001301230,NM_001301232,NM_023635,XM_006510780	Rab27a	RAB27A, member RAS oncogene family	protein_coding	[illegible]
11898	NM_007494	Ass1	argininosuccinate synthetase 1	protein_coding	[illegible]
11899	NM_001205204,NM_007495,XM_011238739,XM_011238740	Astn1	astrotactin 1	protein_coding	[illegible]
11905	NM_080844,XM_006496626,XM_030243696	Serpinc1	serine (or cysteine) peptidase inhibitor, clade C (antithrombin), member 1	protein_coding	[illegible]
11906	NM_007496,XM_006530585,XM_006530586,XM_011248284,XM_030243257,XM_030243258	Zfhx3	zinc finger homeobox 3	protein_coding	[illegible]
11907	NM_001029895,NM_001136054,NM_001271343,NM_013799,XM_006507264,XM_006507265,XM_006507267,XM_011241659,XM_030242026,XM_030242027,XM_030242028	Ate1	arginyltransferase 1	protein_coding	[illegible]
11908	NM_007497,XM_006520351,XM_006520352	Atf1	activating transcription factor 1	protein_coding	[illegible]
11909	NM_001025093,NM_001284369,NM_001284370,NM_001284371,NM_001284372,NM_001284373,NM_001284374,NM_001284376,NM_009715,XM_030246840,XM_030246841,XM_030246843,XM_030246844,XM_030246846,XM_030246847,XM_030246850,XM_030246853,XM_030246855,XM_030246858,XM_030246867,XM_030246872,XM_030246873	Atf2	activating transcription factor 2	protein_coding	[illegible]
11910	NM_007498	Atf3	activating transcription factor 3	protein_coding	[illegible]
11911	NM_001287180,NM_009716	Atf4	activating transcription factor 4	protein_coding	[illegible]
11920	NM_007499,XM_011242385,XR_003947767,XR_870525	Atm	ataxia telangiectasia mutated	protein_coding	[illegible]
11921	NM_007500	Atoh1	atonal bHLH transcription factor 1	protein_coding	[illegible]
11922	NM_009717	Neurod6	neurogenic differentiation 6	protein_coding	[illegible]
11923	NM_001329489,NM_001329490,NM_007501	Neurod4	neurogenic differentiation 4	protein_coding	[illegible]
11924	NM_009718,XM_030252394	Neurog2	neurogenin 2	protein_coding	[illegible]
11927	NM_009720	Atox1	antioxidant 1 copper chaperone	protein_coding	[illegible]
11928	NM_144900	Atp1a1	ATPase, Na+/K+ transporting, alpha 1 polypeptide	protein_coding	[illegible]
11931	NM_009721	Atp1b1	ATPase, Na+/K+ transporting, beta 1 polypeptide	protein_coding	[illegible]
11932	NM_013415,XM_017314232	Atp1b2	ATPase, Na+/K+ transporting, beta 2 polypeptide	protein_coding	[illegible]
11933	NM_001357212,NM_001357213,NM_007502,XM_011242658,XM_017313112,XM_030244030,XM_030244031	Atp1b3	ATPase, Na+/K+ transporting, beta 3 polypeptide	protein_coding	[illegible]
11936	NM_007503,NM_052823,NR_028499,XM_030244032	Fxyd2	FXYD domain-containing ion transport regulator 2	protein_coding	[illegible]
11937	NM_007504,XM_006507268	Atp2a1	ATPase, Ca++ transporting, cardiac muscle, fast twitch 1	protein_coding	[illegible]
11938	NM_001110140,NM_009722,NR_027838	Atp2a2	ATPase, Ca++ transporting, cardiac muscle, slow twitch 2	protein_coding	[illegible]
11941	NM_001036684,NM_001347365,NM_009723,XM_011241161,XM_011241162,XM_011241163,XM_011241165,XM_011241166,XM_011241167,XM_011241168,XM_011241169,XM_011241170,XM_017321353,XM_030255119,XM_030255120,XM_030255121,XM_030255122,XR_001785094,XR_868849,XR_868850	Atp2b2	ATPase, Ca++ transporting, plasma membrane 2	protein_coding	[illegible]
11944	NM_001290627,NM_018731	Atp4a	ATPase, H+/K+ exchanging, gastric, alpha polypeptide	protein_coding	[illegible]
11946	NM_007505	Atp5a1	ATP synthase, H+ transporting, mitochondrial F1 complex, alpha subunit	protein_coding	[illegible]
11947	NM_016774	Atp5b	ATP synthase, H+ transporting mitochondrial F1 complex, beta subunit	protein_coding	[illegible]
11949	NM_001112738,NM_020615,XM_006497310	Atp5c1	ATP synthase, H+ transporting, mitochondrial F1 complex, gamma polypeptide 1	protein_coding	[illegible]
11950	NM_001304719,NM_009725,XM_030252395	Atp5pb	ATP synthase, H+ transporting, mitochondrial F0 complex, subunit B1	protein_coding	[illegible]
11951	NM_001161419,NM_007506,XM_030245490	Atp5g1	ATP synthase, H+ transporting, mitochondrial F0 complex, subunit C1 (subunit 9)	protein_coding	[illegible]
11957	NM_001302213,NM_001302214,NM_001302215,NM_001302216,NM_001358499,NM_001358500,NM_016755,XM_011246092,XM_030248936,XM_030248937,XM_030248938	Atp5j	ATP synthase, H+ transporting, mitochondrial F0 complex, subunit F	protein_coding	[illegible]
11958	NM_007507,NR_154996	Atp5k	ATP synthase, H+ transporting, mitochondrial F1F0 complex, subunit E	protein_coding	[illegible]
11964	NM_001358203,NM_001358204,NM_007508,XM_006521721	Atp6v1a	ATPase, H+ transporting, lysosomal V1 subunit A	protein_coding	[illegible]
11966	NM_007509	Atp6v1b2	ATPase, H+ transporting, lysosomal V1 subunit B2	protein_coding	[illegible]
11972	NM_013477	Atp6v0d1	ATPase, H+ transporting, lysosomal V0 subunit D1	protein_coding	[illegible]
11973	NM_007510	Atp6v1e1	ATPase, H+ transporting, lysosomal V1 subunit E1	protein_coding	[illegible]
11974	NM_025272	Atp6v0e	ATPase, H+ transporting, lysosomal V0 subunit E	protein_coding	[illegible]

11975	NM_001243049,NM_001243050,NM_001243051,NM_001362636,NM_001362637,NM_001362638,NM_001362639,NM_001362640,NM_001362641,NM_016920,XM_006532049,XM_006532050,XM_006532051,XM_006532054,XM_006532055,XM_030245491,XM_030245492,XR_388335,XR_388336	Atp6v0a1	ATPase, H+ transporting, lysosomal V0 subunit A1	protein_coding
11977	NM_001109757,NM_009726,XM_030251205,XM_030251206	Atp7a	ATPase, Cu++ transporting, alpha polypeptide	protein_coding
11979	NM_007511,XM_006509007,XM_011242119,XM_017312538,XR_378777	Atp7b	ATPase, Cu++ transporting, beta polypeptide	protein_coding
11980	NM_001038999,NM_001284345,NM_009727,XM_006503703,XM_006503704,XM_006503705,XM_011240698,XM_017320623,XM_017320624,XM_017320625,XM_017320626	Atp8a1	ATPase, aminophospholipid transporter (APLT), class I, type 8A, member 1	protein_coding
11981	NM_001289445,NM_001289446,NM_001354977,NM_001354978,NM_015731,XM_006498603,XM_017315066,XM_017315067,XM_030246882	Atp9a	ATPase, class II, type 9A	protein_coding
11982	NM_009728,XM_006540580,XM_006540581,XM_006540583,XM_017321952,XR_882009	Atp10a	ATPase, class V, type 10A	protein_coding
11983	NM_007512,XM_030253106,XM_030253107	Atpif1	ATPase inhibitory factor 1	protein_coding
11984	NM_001361531,NM_001361532,NM_001361533,NM_001361534,NM_001361535,NM_009729	Atp6v0c	ATPase, H+ transporting, lysosomal V0 subunit C	protein_coding
11987	NM_001301424,NM_007513,XM_006504796	Slc7a1	solute carrier family 7 (cationic amino acid transporter, y+ system), member 1	protein_coding
11988	NM_001044740,NM_007514,XM_006509250,XM_006509251,XM_006509252,XM_006509253	Slc7a2	solute carrier family 7 (cationic amino acid transporter, y+ system), member 2	protein_coding
11989	NM_001301840,NM_007515,XM_006527753,XM_011247513,XM_017318364,XM_030251207,XM_030251208	Slc7a3	solute carrier family 7 (cationic amino acid transporter, y+ system), member 3	protein_coding
11990	NM_009730,XM_006498606,XM_011239259	Atrn	attractin	protein_coding
11991	NM_001077265,NM_001077266,NM_001077267,NM_007516,XM_006534735,XR_389257,XR_389258	Hnrnpd	heterogeneous nuclear ribonucleoprotein D	protein_coding
11992	NM_016709,XM_006516846,XM_017315373,XM_030247133,XR_003950422	Auh	AU RNA binding protein/enoyl-coenzyme A hydratase	protein_coding
11993	NM_001301649,NM_007517,NR_125891,XR_377415,XR_377416	Aup1	ancient ubiquitous protein 1	protein_coding
11994	NM_001142735,NM_001142736,NM_001142737,NM_001142738,NM_001142739,NM_001142740,NM_001142741,NM_001142742,NM_001142743,NM_001142746,NM_001142747,NM_001142748,NM_001142760,NM_023115,XM_006513155,XM_006513156,XM_017313784,XM_017313785,XR_001779426,XR_001779427,XR_001779428,XR_001779429,XR_001779430,XR_001779431,XR_001779432,XR_001779433,XR_001779434,XR_001779435,XR_001779436,XR_001779438,XR_001779440,XR_001779441,XR_003948583,XR_003948584,XR_003948585	Pcdh15	protocadherin 15	protein_coding
11997	NM_009731	Akr1b7	aldo-keto reductase family 1, member B7	protein_coding
11998	NM_009732	Avp	arginine vasopressin	protein_coding
12005	NM_001159598,NM_009733,XM_006523515,XM_006523516,XM_030249451,XM_030249452	Axin1	axin 1	protein_coding
12006	NM_015732,XM_006532057,XM_006532058,XM_006532059,XM_006532060,XM_030245493	Axin2	axin 2	protein_coding
12007	NM_013478	Azgp1	alpha-2-glycoprotein 1, zinc	protein_coding
12009	NM_009734,XM_006532061,XR_003949250,XR_003949252,XR_003949253,XR_003949254	Cep131	centrosomal protein 131	protein_coding
12010	NM_009735	B2m	beta-2 microglobulin	protein_coding
12012	NM_007519,XM_006537565	Baat	bile acid-Coenzyme A: amino acid N-acyltransferase	protein_coding
12013	NM_007520,XM_006522879,XM_030248939	Bach1	BTB and CNC homology 1, basic leucine zipper transcription factor 1	protein_coding
12014	NM_001109661,XM_006537566,XM_006537567,XM_011249908,XM_011249909,XM_011249910,XM_030253108,XM_030253109,XM_030253110,XM_030253111,XM_030253112,XM_030253113	Bach2	BTB and CNC homology, basic leucine zipper transcription factor 2	protein_coding
12015	NM_001285453,NM_007522	Bad	BCL2-associated agonist of cell death	protein_coding
12017	NM_001171739,NM_009736	Bag1	BCL2-associated athanogene 1	protein_coding
12018	NM_007523,NR_149749	Bak1	BCL2-antagonist/killer 1	protein_coding
12020	NM_007524	Nkx3-2	NK3 homeobox 2	protein_coding
12021	NM_007525,XM_006495631,XM_006495632,XR_003947336	Bard1	BRCA1 associated RING domain 1	protein_coding
12022	NM_007526	Barx1	BarH-like homeobox 1	protein_coding
12023	NM_013800	Barx2	BarH-like homeobox 2	protein_coding
12028	NM_007527,XM_006540584,XM_011250780	Bax	BCL2-associated X protein	protein_coding
12029	NM_007528,XM_030245494	Bcl6b	B cell CLL/lymphoma 6, member B	protein_coding
12032	NM_001109758,NM_007529,XM_006500936,XM_006500937,XM_030252396	Bcan	brevican	protein_coding
12033	NM_001164090,NM_007530	Bcap29	B cell receptor associated protein 29	protein_coding
12034	NM_007531	Phb2	prohibitin 2	protein_coding
12035	NM_001024468,NM_007532,XM_011241566	Bcat1	branched chain aminotransferase 1, cytosolic	protein_coding
12036	NM_001243052,NM_009737,XM_017321953,XM_017321954,XR_003946378	Bcat2	branched chain aminotransferase 2, mitochondrial	protein_coding
12038	NM_009738,XM_011240000	Bche	butyrylcholinesterase	protein_coding
12039	NM_007533,XM_006539486,XM_030242029,XM_030242030	Bckdha	branched chain ketoacid dehydrogenase E1, alpha polypeptide	protein_coding
12040	NM_001305935,NM_199195,XM_006510786,XM_006510788,XM_017313114	Bckdhb	branched chain ketoacid dehydrogenase E1, beta polypeptide	protein_coding
12041	NM_009739	Bckdk	branched chain ketoacid dehydrogenase kinase	protein_coding
12042	NM_009740	Bcl10	B cell leukemia/lymphoma 10	protein_coding
12043	NM_009741,NM_177410	Bcl2	B cell leukemia/lymphoma 2	protein_coding
12044	NM_009742	Bcl2a1a	B cell leukemia/lymphoma 2 related protein A1a	protein_coding
12045	NM_007534	Bcl2a1b	B cell leukemia/lymphoma 2 related protein A1b	protein_coding
12047	NM_007536	Bcl2a1d	B cell leukemia/lymphoma 2 related protein A1d	protein_coding
12048	NM_001289716,NM_001289717,NM_001289739,NM_001355053,NM_009743,NR_149254,XM_006498611,XM_006498612,XM_011239260,XM_030246899,XR_374396	Bcl2l1	BCL2-like 1	protein_coding
12049	NM_013479	Bcl2l10	Bcl2-like 10	protein_coding
12050	NM_007537,XM_006518457,XM_006518458,XM_030247612	Bcl2l2	BCL2-like 2	protein_coding
12051	NM_033601,XM_006539487	Bcl3	B cell leukemia/lymphoma 3	protein_coding
12053	NM_001348026,NM_009744,XM_030248940,XM_030248941	Bcl6	B cell leukemia/lymphoma 6	protein_coding
12054	NM_009745	Bcl7b	B cell CLL/lymphoma 7B	protein_coding
12055	NM_001347652,NM_009746	Bcl7c	B cell CLL/lymphoma 7C	protein_coding
12057	NM_007538	Opn1sw	opsin 1 (cone pigments), short-wave-sensitive (color blindness, tritan)	protein_coding
12061	NM_007539	Bdkrb1	bradykinin receptor, beta 1	protein_coding
12062	NM_009747,XM_006515442,XM_011243991	Bdkrb2	bradykinin receptor, beta 2	protein_coding
12064	NM_001048139,NM_001048141,NM_001048142,NM_001285416,NM_001285417,NM_001285418,NM_001285419,NM_001285420,NM_001285421,NM_001285422,NM_001316310,NM_007540	Bdnf	brain derived neurotrophic factor	protein_coding
12068	NM_009748,XM_006504971	Bet1	Bet1 golgi vesicular membrane trafficking protein	protein_coding
12069	NM_009749	Bex2	brain expressed X-linked 2	protein_coding
12070	NM_001110233,NM_001110234,NM_009750	Bex3	brain expressed X-linked 3	protein_coding
12075	NM_001291061,NM_009751,XM_011239261,XM_011239264,XM_011239265,XM_011239266,XM_030246932	Bfsp1	beaded filament structural protein 1, in lens-CP94	protein_coding
12091	NM_009752,NR_108101,NR_108102	Glb1	galactosidase, beta 1	protein_coding
12095	NM_001305448,NM_001305449,NM_001305450,NM_031368	Bglap3	bone gamma-carboxyglutamate protein 3	protein_coding
12096	NM_007541	Bglap	bone gamma carboxyglutamate protein	protein_coding
12097	NM_001032298	Bglap2	bone gamma-carboxyglutamate protein 2	protein_coding
12111	NM_007542,XM_006527758	Bgn	biglycan	protein_coding
12116	NM_016668	Bhmt	betaine-homocysteine methyltransferase	protein_coding
12121	NM_001112796,NM_009753,XM_006507014,XM_006507015,XM_006507016,XM_006507017,XM_006507018,XM_006507019,XM_006507020,XM_006507023,XM_006507024,XM_030255123,XR_003956156,XR_869604	Bicd1	BICD cargo adaptor 1	protein_coding
12122	NM_007544,XM_006505409,XM_006505410,XM_006505411	Bid	BH3 interacting domain death agonist	protein_coding
12123	NM_007545	Hrk	harakiri, BCL2 interacting protein (contains only BH3 domain)	protein_coding
12124	NM_007546,XM_006520353,XM_006520354,XM_006520355,XM_006520356,XM_006520357	Bik	BCL2-interacting killer	protein_coding
12125	NM_001284410,NM_001291016,NM_009754,NM_207680,NM_207681,XM_006498614,XM_006498615,XM_006498616,XM_006498617	Bcl2l11	BCL2-like 11 (apoptosis facilitator)	protein_coding
12140	NM_021272	Fabp7	fatty acid binding protein 7, brain	protein_coding
12142	NM_007548,XM_006512502,XM_006512503,XM_006512504,XM_006512505,XM_011243109	Prdm1	PR domain containing 1, with ZNF domain	protein_coding
12143	NM_007549	Blk	B lymphoid kinase	protein_coding
12144	NM_001042527,NM_007550,XM_017321956,XM_017321957,XR_001785468	Blm	Bloom syndrome, RecQ like helicase	protein_coding
12145	NM_007551	Cxcr5	chemokine (C-X-C motif) receptor 5	protein_coding
12151	NM_007552,XM_006497311,XM_006497312	Bmi1	Bmi1 polycomb ring finger oncogene	protein_coding
12153	NM_001360021,NM_009755,NR_033241,XM_011244931,XM_011244932,XM_011244933,XM_017315805,XM_030247613,XM_030247614,XR_001781089,XR_001781090,XR_003950816,XR_003950817,XR_383136	Bmp1	bone morphogenetic protein 1	protein_coding
12155	NM_009757	Bmp15	bone morphogenetic protein 15	protein_coding
12156	NM_007553	Bmp2	bone morphogenetic protein 2	protein_coding
12159	NM_001316360,NM_007554	Bmp4	bone morphogenetic protein 4	protein_coding
12160	NM_007555	Bmp5	bone morphogenetic protein 5	protein_coding
12161	NM_007556	Bmp6	bone morphogenetic protein 6	protein_coding
12162	NM_007557	Bmp7	bone morphogenetic protein 7	protein_coding
12163	NM_001256019,NM_007558	Bmp8a	bone morphogenetic protein 8a	protein_coding
12164	NM_007559,XM_011240420	Bmp8b	bone morphogenetic protein 8b	protein_coding
12165	NM_019506	Gdf2	growth differentiation factor 2	protein_coding
12166	NM_009758,XM_006518469,XM_006518470,XM_017315806,XM_030247615	Bmpr1a	bone morphogenetic protein receptor, type 1A	protein_coding
12167	NM_001277216,NM_001277217,NM_001277218,NM_001277220,NM_001355043,NM_007560,XM_006500939,XM_006500940,XM_006500942,XM_006500943,XM_006500944,XM_030252398	Bmpr1b	bone morphogenetic protein receptor, type 1B	protein_coding
12168	NM_007561,XM_006495633	Bmpr2	bone morphogenetic protein receptor, type II (serine/threonine kinase)	protein_coding
12169	NM_009759	Bmx	BMX non-receptor tyrosine kinase	protein_coding
12173	NM_007562,XM_011250781,XM_011250782	Bnc1	basonuclin 1	protein_coding
12175	NM_001008238,NM_016787,XM_006510789,XM_006510791,XM_006510793	Bnip2	BCL2/adenovirus E1B interacting protein 2	protein_coding
12176	NM_009760	Bnip3	BCL2/adenovirus E1B interacting protein 3	protein_coding
12177	NM_009761,XM_006518471,XM_017315807	Bnip3l	BCL2/adenovirus E1B interacting protein 3-like	protein_coding
12180	NM_001160127,NM_009762	Smyd1	SET and MYND domain containing 1	protein_coding
12181	NM_013481	Bop1	block of proliferation 1	protein_coding
12182	NM_009763,XM_006503706	Bst1	bone marrow stromal cell antigen 1	protein_coding
12183	NM_001363708,NM_001363709,NM_001363710,NM_007563,NR_156731	Bpgm	2,3-bisphosphoglycerate mutase	protein_coding
12189	NM_009764,XM_006532064,XM_006532068,XM_017314233,XM_030245495	Brca1	breast cancer 1, early onset	protein_coding
12190	NM_001081001,NM_009765,XM_006504906,XM_006504907,XM_011241019,XR_001784600,XR_377265	Brca2	breast cancer 2, early onset	protein_coding
12192	NM_007564	Zfp36l1	zinc finger protein 36, C3H type-like 1	protein_coding
12193	NM_001001806	Zfp36l2	zinc finger protein 36, C3H type-like 2	protein_coding
12211	NM_007566,XM_006523518,XM_006523519,XM_006523520,XM_006523521,XM_006523522,XM_006523523,XM_006523524,XM_006523526,XM_006523527,XM_006523528,XM_011246257,XM_030249453,XM_030249454,XM_030249455,XM_030249456,XM_030249457,XM_030249458,XR_003952078,XR_003952080,XR_385268	Birc6	baculoviral IAP repeat-containing 6	protein_coding
12212	NM_009767,XM_006527760	Chic1	cysteine-rich hydrophobic domain 1	protein_coding
12215	NM_001077184,NM_009768	Bsg	basigin	protein_coding
12217	NM_007567,XM_006511634	Bsn	bassoon	protein_coding
12223	NM_007568	Btc	betacellulin, epidermal growth factor family member	protein_coding
12224	NM_009769	Klf5	Kruppel-like factor 5	protein_coding
12226	NM_007569	Btg1	B cell translocation gene 1, anti-proliferative	protein_coding
12227	NM_007570	Btg2	B cell translocation gene 2, anti-proliferative	protein_coding
12228	NM_001297747,NM_009770	Btg3	B cell translocation gene 3	protein_coding
12229	NM_013482,XM_006528483,XM_006528484,XM_006528485	Btk	Bruton agammaglobulinemia tyrosine kinase	protein_coding
12231	NM_013483,XM_006516535	Btn1a1	butyrophilin, subfamily 1, member A1	protein_coding
12234	NM_001037758,NM_001286465,NM_001286466,NM_001360120,NM_001360122,NM_001360124,NM_001360126,NM_001360127,NM_009771,XM_006526612,XM_006526613,XM_006526614,XM_006526615,XM_011247119,XM_011247120,XM_011247121,XM_011247123,XM_011247127,XM_030250711,XM_030250712,XM_030250713,XM_030250714,XM_030250715,XM_030250716,XM_030250718,XM_030250719,XM_030250720,XM_030250721,XM_030250722	Btrc	beta-transducin repeat containing protein	protein_coding
12235	NM_001113179,NM_009772,XM_006498620	Bub1	BUB1, mitotic checkpoint serine/threonine kinase	protein_coding
12236	NM_009773,XM_006498622	Bub1b	BUB1B, mitotic checkpoint serine/threonine kinase	protein_coding
12237	NM_001317350,NM_009774,XM_006507271	Bub3	BUB3 mitotic checkpoint protein	protein_coding
12238	NM_147778	Commd3	COMM domain containing 3	protein_coding
12257	NM_009775	Tspo	translocator protein	protein_coding
12258	NM_009776,XM_006498623	Serping1	serine (or cysteine) peptidase inhibitor, clade G, member 1	protein_coding
12259	NM_007572	C1qa	complement component 1, q subcomponent, alpha polypeptide	protein_coding
12260	NM_009777	C1qb	complement component 1, q subcomponent, beta polypeptide	protein_coding
12261	NM_007573	C1qbp	complement component 1, q subcomponent binding protein	protein_coding
12262	NM_007574,XM_017319927	C1qc	complement component 1, q subcomponent, C chain	protein_coding
12263	NM_013484	C2	complement component 2 (within H-2S)	protein_coding
12265	NM_001243760,NM_001243761,NM_001302618,NM_001302619,NM_007575,XM_006521724,XM_006521726,XM_011245810,XM_030248943,XR_001781783	Ciita	class II transactivator	protein_coding
12266	NM_009778,XM_011246258	C3	complement component 3	protein_coding
12267	NM_009779	C3ar1	complement component 3a receptor 1	protein_coding
12268	NM_009780,XM_006523531,XM_006523532,XM_030249459	C4b	complement component 4B (Chido blood group)	protein_coding
12269	NM_007576,XM_011247906,XM_017312492	C4bp	complement component 4 binding protein	protein_coding
12270	NR_028304	C4bp-ps1	complement component 4 binding protein, pseudogene 1	transcribed_pseudogene
12273	NM_001173550,NM_007577	C5ar1	complement component 5a receptor 1	protein_coding
12274	NM_016704,XM_006519957,XM_006519958,XM_006519959	C6	complement component 6	protein_coding
12279	NM_001368420,NM_001368421,NM_013485	C9	complement component 9	protein_coding
12282	NM_021395,XM_006509959,XM_006509960,XM_006509961,XM_006509962	Hyou1	hypoxia up-regulated 1	protein_coding
12283	NM_001355046,NM_001355047,NM_133781,XM_006529091,XM_011247907,XM_030243907	Cab39	calcium binding protein 39	protein_coding
12286	NM_001252059,NM_001252060,NM_001252061,NM_007578,XM_006530587,XM_006530588,XM_006530589,XM_006530590,XM_006530591,XM_006530592,XM_006530593,XM_006530594,XM_006530595,XM_006530596,XM_006530597,XM_006530598,XM_006530599,XM_006530600,XM_006530601,XM_006530602,XM_006530603,XM_006530605,XM_006530609,XM_011248285,XM_030243260,XM_030243261,XM_030243262,XR_003947221,XR_003947222,XR_003947223,XR_003947224,XR_003947225,XR_003947226,XR_00	Cacna1a	calcium channel, voltage-dependent, P/Q type, alpha 1A subunit	protein_coding
12287	NM_001042528,NM_007579,XM_006497626,XM_006497627,XM_006497628,XM_006497629,XM_006497630,XM_006497631,XM_006497632,XM_006497633,XM_006497634,XM_006497635,XM_006497636,XM_006497637,XM_006497638,XM_006497640,XM_011239014,XM_017315140,XM_017315141,XM_017315146,XM_017315155,XM_017315156,XR_003950091,XR_374052	Cacna1b	calcium channel, voltage-dependent, N type, alpha 1B subunit	protein_coding
12288	NM_001159533,NM_001159534,NM_001159535,NM_001255997,NM_001255998,NM_001255999,NM_001256000,NM_001256001,NM_001256002,NM_001290335,NM_009781,XM_006505415,XM_006505418,XM_006505426,XM_006505427,XM_006505430,XM_006505432,XM_006505434,XM_006505435,XM_006505436,XM_006505437,XM_006505438,XM_006505439,XM_006505440,XM_006505441,XM_006505442,XM_006505443,XM_011241175,XM_011241176,XM_011241184,XM_011241185,XM_011241186,XM_011241187,XM_011241192,XM_011241193,XM_017321356,XM_017321357,XM_017321358,XM_017321359,XM_017321360,XM_017321361,XM_017321362,XM_017321363,XM_017321364,XM_017321365,XM_017321366,XM_017321367,XM_017321368,XM_017321369,XM_030255124,XM_030255125,XM_030255126	Cacna1c	calcium channel, voltage-dependent, L type, alpha 1C subunit	protein_coding
12289	NM_001083616,NM_001302637,NM_028981,XM_006518475,XM_006518479,XM_006518480,XM_017315808,XM_017315809,XM_017315810,XM_017315811,XM_017315812,XM_017315813,XM_017315814,XM_017315815,XM_017315816,XM_017315817,XM_017315818,XM_017315819,XM_017315820,XM_017315821,XM_017315822,XM_017315823,XM_017315824,XM_017315825,XR_001781092	Cacna1d	calcium channel, voltage-dependent, L type, alpha 1D subunit	protein_coding
12290	NM_009782,XM_006529105,XM_011247909,XM_011247916,XM_017312578,XM_017312613,XM_017312619,XM_017312628,XM_017312642,XM_017312660,XM_017312682,XM_017312712,XM_017312730,XM_017312745,XM_017312766,XM_017312795,XM_017312824,XM_017312848,XM_017312855,XM_017312867,XM_030243926,XM_030243929,XM_030244154	Cacna1e	calcium channel, voltage-dependent, R type, alpha 1E subunit	protein_coding

12291	NM_001112813,NM_001177888,NM_001177890,NM_009783,XM_006532069,XM_006532070,XM_006532072,XM_006532073,XM_006532074,XM_006532075,XM_006532076,XM_006532079,XM_006532080,XM_006532081,XM_006532082,XM_006532083,XM_006532084,XM_006532085,XM_006532086,XM_006532087,XM_006532088,XM_011248686,XM_011248687,XM_011248688,XM_011248689,XM_011248690,XM_011248691,XM_011248692,XM_011248693,XM_011248694,XM_011248695,XM_011248696,XM_011248697,XM_011248698,XM_011248699,XM_011248700,XM_017314234,XM_017314235,XM_030245496,XM_030245497,XR_003949260,XR_003949261,XR_388338,XR_388339,XR_879510	Cacna1g	calcium channel, voltage-dependent, T type, alpha 1G subunit	protein_coding	NP_001106284.1;NP_001171359.1;NP_001171361.1;NP_033913.2;XP_006532132.1;XP_006532133.1;XP_006532135.1;XP_006532136.1;XP_006532137.1;XP_006532138.1;XP_006532139.1;XP_006532142.1;XP_006532143.1;XP_006532144.1;XP_006532145.1;XP_006532146.1;XP_006532147.1;XP_006532148.1;XP_006532149.1;XP_006532150.1;XP_006532151.1;XP_011246988.1;XP_011246989.1;XP_011246990.1;XP_011246991.1;XP_011246992.1;XP_011246993.1;XP_011246994.1;XP_011246995.1;XP_011246996.1;XP_011246997.1;XP_011246998.1;XP_011246999.1;XP_011247000.1;XP_011247001.1;XP_011247002.1;XP_017169723.1;XP_017169724.1;XP_030101356.1;XP_030101357.1	1201678	.	28.004422	[illegible]
12292	NM_001081023,NM_014193,XM_006529106	Cacna1s	calcium channel, voltage-dependent, L type, alpha 1S subunit	protein_coding	NP_001074492.1;NP_055008.2;XP_006529169.1	88294	.	2.563396	[illegible]
12293	NM_001110843,NM_001110844,NM_001110845,NM_001110846,NM_009784,XM_006535618,XM_006535619,XM_011249741,XM_011249742,XM_011249743,XM_011249744,XM_017320631,XM_030254085	Cacna2d1	calcium channel, voltage-dependent, alpha2/delta subunit 1	protein_coding	NP_001104313.1;NP_001104314.1;NP_001104315.1;NP_001104316.1;NP_033914.1;XP_006535681.1;XP_006535682.1;XP_011248043.1;XP_011248044.1;XP_011248045.1;XP_011248046.1;XP_017176120.1;XP_030109945.1	88295	.	11.279498	[illegible]
12294	NM_009785,XM_006518486,XM_011244938,XM_030247616,XR_001781093	Cacna2d3	calcium channel, voltage-dependent, alpha2/delta subunit 3	protein_coding	NP_033915.1;XP_006518549.1;XP_011243240.1;XP_030103476.1	1338890	.	0.394661	[illegible]
12295	NM_001159319,NM_001159320,NM_001282977,NM_001282978,NM_031173,NM_145121,XM_006532091,XM_006532092,XM_006532094,XM_017314236,XM_030245498,XM_030245499,XM_030245500	Cacnb1	calcium channel, voltage-dependent, beta 1 subunit	protein_coding	NP_001152791.1;NP_001152792.1;NP_001269906.1;NP_001269907.1;NP_112450.1;NP_660099.2;XP_006532154.1;XP_006532155.1;XP_006532157.1;XP_017169725.1;XP_030101358.1;XP_030101359.1;XP_030101360.1	102522	.	6.062929	[illegible]
12296	NM_001252533,NM_001309519,NM_023116,XM_006497313,XM_006497314,XM_006497315,XM_006497317,XM_006497318,XM_006497319,XM_006497320,XM_006497321,XM_006497323,XM_011238946	Cacnb2	calcium channel, voltage-dependent, beta 2 subunit	protein_coding	NP_001239462.1;NP_001296448.1;NP_075605.1;XP_006497376.1;XP_006497377.1;XP_006497378.1;XP_006497380.1;XP_006497381.1;XP_006497382.1;XP_006497383.1;XP_006497384.1;XP_006497386.1;XP_011237248.1	894644	.	0.243963	[illegible]
12297	NM_001044741,NM_001286226,NM_001356543,NM_007581,XM_006520360,XM_006520361,XM_006520362,XM_006520363,XM_006520364,XM_006520365,XM_006520367,XM_006520370,XM_017316415,XM_017316418,XM_017316419,XM_030248302,XM_030248303,XM_030248304,XM_030248305,XM_030248306,XM_030248307	Cacnb3	calcium channel, voltage-dependent, beta 3 subunit	protein_coding	NP_001038206.1;NP_001273155.1;NP_001343472.1;NP_031607.2;XP_006520423.1;XP_006520424.1;XP_006520425.1;XP_006520426.1;XP_006520427.1;XP_006520428.1;XP_006520430.1;XP_006520433.1;XP_017171904.1;XP_017171907.1;XP_017171908.1;XP_030104162.1;XP_030104163.1;XP_030104164.1;XP_030104165.1;XP_030104166.1;XP_030104167.1	103307	.	13.034701	[illegible]
12298	NM_001037099,NM_001285426,NM_001285427,NM_001285428,NM_001355059,NM_146123,XM_006497641,XM_006497642,XM_011239015,XM_017315163,XM_030247010	Cacnb4	calcium channel, voltage-dependent, beta 4 subunit	protein_coding	NP_001032176.1;NP_001272355.1;NP_001272356.1;NP_001272357.1;NP_001341988.1;NP_666235.1;XP_006497704.1;XP_006497705.1;XP_011237317.1;XP_017170652.1;XP_030102870.1	103301	.	0.318413	[illegible]
12299	NM_007582	Cacng1	calcium channel, voltage-dependent, gamma subunit 1	protein_coding	NP_031608.1	1206582	.	8.881249	[illegible]
12300	NM_007583,XM_006520371	Cacng2	calcium channel, voltage-dependent, gamma subunit 2	protein_coding	NP_031609.1;XP_006520434.1	1316660	.	0.000000	[illegible]
12301	NM_009786	Cacybp	calcyclin binding protein	protein_coding	NP_033916.1	1270839	.	20.682486	[illegible]
12304	NM_001368756,NM_009787	Pdia4	protein disulfide isomerase associated 4	protein_coding	NP_001355685.1;NP_033917.2	104864	.	75.053785	[illegible]
12305	NM_001198831,NM_001198833,NM_007584,NM_172962,XM_006523534,XM_006523535,XM_006523536,XM_006523537,XM_006523538,XM_011246259,XM_030249460,XM_030249461,XM_030249462,XM_030249463,XM_030249464,XR_001782030	Ddr1	discoidin domain receptor family, member 1	protein_coding	NP_001185760.1;NP_001185762.1;NP_031610.1;NP_766550.1;XP_006523597.1;XP_006523598.1;XP_006523599.1;XP_006523600.1;XP_006523601.1;XP_011244561.1;XP_030105320.1;XP_030105321.1;XP_030105322.1;XP_030105323.1;XP_030105324.1	99216	.	5.381075	[illegible]
12306	NM_007585,XM_006510796	Anxa2	annexin A2	protein_coding	NP_031611.1;XP_006510859.1	88246	.	569.417867	[illegible]
12307	NM_009788	Calb1	calbindin 1	protein_coding	NP_033918.1	88248	.	0.000000	[illegible]
12308	NM_001368293,NM_001368294,NM_007586	Calb2	calbindin 2	protein_coding	NP_001355222.1;NP_001355223.1;NP_031612.1	101914	.	0.065394	[illegible]
12310	NM_001033954,NM_001289444,NM_001305616,NM_007587,XM_011241660,XM_030242031,XM_030242032	Calca	calcitonin/calcitonin-related polypeptide, alpha	protein_coding	NP_001029126.1;NP_001276373.1;NP_001292545.1;NP_031613.2;XP_011239962.1;XP_030097891.1;XP_030097892.1	2151253	.	3.239942	[illegible]
12311	NM_001042725,NM_001355192,NM_007588,XM_006504972	Calcr	calcitonin receptor	protein_coding	NP_001036190.1;NP_001342121.1;NP_031614.2;XP_006505035.1	101950	.	0.106037	[illegible]
12313	NM_001313934,NM_009790	Calm1	calmodulin 1	protein_coding	NP_001300863.1;NP_033920.1	88251	.	90.416456	[illegible]
12314	NM_001355703,NM_007589	Calm2	calmodulin 2	protein_coding	NP_001342632.1;NP_031615.1	103250	.	253.717979	[illegible]
12315	NM_007590	Calm3	calmodulin 3	protein_coding	NP_031616.1	103249	.	81.581703	[illegible]
12316	NM_009791,XM_006529108,XM_017312957	Aspm	abnormal spindle microtubule assembly	protein_coding	NP_033921.3;XP_006529171.1;XP_017168446.1	1334448	.	3.312341	[illegible]
12317	NM_007591	Calr	calreticulin	protein_coding	NP_031617.1	88252	.	317.655304	[illegible]
12319	NM_007592	Car8	carbonic anhydrase 8	protein_coding	NP_031618.2	88253	.	0.397518	[illegible]
12321	NM_001285412,NM_007594,NM_184053	Calu	calumenin	protein_coding	NP_001272341.1;NP_031620.1;NP_908942.1	1097158	.	318.986758	[illegible]
12322	NM_001286809,NM_009792,NM_177407,XM_006525541,XM_017317805	Camk2a	calcium/calmodulin-dependent protein kinase II alpha	protein_coding	NP_001273738.1;NP_033922.1;NP_803126.1;XP_006525604.1;XP_017173294.1	88256	.	3.571899	[illegible]
12323	NM_001174053,NM_001174054,NM_007595,XM_006514464,XM_006514465,XM_006514467,XM_006514469,XM_006514470,XM_006514475,XM_006514476,XM_006514477,XM_006514479,XM_017314237,XM_017314238,XM_017314239,XM_017314240,XM_017314241,XM_030245501,XM_030245502,XM_030245503,XM_030245504,XM_030245505,XM_030245506,XM_030245507	Camk2b	calcium/calmodulin-dependent protein kinase II, beta	protein_coding	NP_001167524.1;NP_001167525.1;NP_031621.3;XP_006514527.1;XP_006514528.1;XP_006514530.1;XP_006514532.1;XP_006514533.1;XP_006514538.1;XP_006514539.1;XP_006514540.1;XP_006514542.1;XP_017169726.1;XP_017169727.1;XP_017169728.1;XP_017169729.1;XP_017169730.1;XP_030101361.1;XP_030101362.1;XP_030101363.1;XP_030101364.1;XP_030101365.1;XP_030101366.1;XP_0301	88257	.	1.932976	[illegible]
12325	NM_001039138,NM_001039139,NM_001360186,NM_178597,XM_006518488,XM_006518489,XM_006518490,XM_006518491,XM_006518492,XM_006518493,XM_006518494,XM_006518495,XM_006518496,XM_006518497,XM_006518500,XM_017315826,XM_017315827,XM_030247617,XM_030247618,XM_030247619,XM_030247620,XM_030247621,XM_030247622,XM_030247623,XM_030247624,XM_030247625,XM_030247626,XM_030247627,XM_030247628,XM_030247629,XM_030247630,XR_003950818,XR_003950819,XR_003950820,XR_003950821,XR_003950822,XR_874287	Camk2g	calcium/calmodulin-dependent protein kinase II gamma	protein_coding	NP_001034227.1;NP_001034228.1;NP_001347115.1;NP_848712.2;XP_006518551.1;XP_006518552.1;XP_006518553.1;XP_006518554.1;XP_006518555.1;XP_006518556.1;XP_006518557.1;XP_006518558.1;XP_006518559.1;XP_006518560.1;XP_006518563.1;XP_017171315.1;XP_017171316.1;XP_030103477.1;XP_030103478.1;XP_030103479.1;XP_030103480.1;XP_030103481.1;XP_030103482.1;XP_030103483.1;XP_030103484.1;XP_030103485.1;XP_030103486.1;XP_030103487.1;XP_030103488.1;XP_030103489.1;XP_030103490.1	88259	.	15.059034	[illegible]
12326	NM_009793,XM_006525543,XM_006525544,XM_006525546,XM_030250297,XM_030250298	Camk4	calcium/calmodulin-dependent protein kinase IV	protein_coding	NP_033923.2;XP_006525606.1;XP_006525607.1;XP_006525609.1;XP_030106157.1;XP_030106158.1	88258	.	3.030800	[illegible]
12328	NM_007596	Caml	calcium modulating ligand	protein_coding	NP_031622.2	104728	.	16.246801	[illegible]
12330	NM_001110499,NM_001110500,NM_007597	Canx	calnexin	protein_coding	NP_001103969.1;NP_001103970.1;NP_031623.1	88261	.	81.318119	[illegible]
12331	NM_001301067,NM_001358035,NM_001358036,NM_007598	Cap1	CAP, adenylate cyclase-associated protein 1 (yeast)	protein_coding	NP_001287996.1;NP_001344964.1;NP_001344965.1;NP_031624.2	88262	.	58.285147	[illegible]
12332	NM_001042534,NM_001271395,NM_001271415,NM_007599,XM_017321370,XM_030255128	Capg	capping protein (actin filament), gelsolin-like	protein_coding	NP_001035999.1;NP_001258324.1;NP_001258344.1;NP_031625.2;XP_017176859.1;XP_030110988.1	1098259	.	25.669471	[illegible]
12333	NM_001110504,NM_007600	Capn1	calpain 1	protein_coding	NP_001103974.1;NP_031626.1	88263	.	11.164963	[illegible]
12334	NM_009794	Capn2	calpain 2	protein_coding	NP_033924.2	88264	.	49.812016	[illegible]
12335	NM_001109761,NM_001177799,NM_007601	Capn3	calpain 3	protein_coding	NP_001103231.1;NP_001171270.1;NP_031627.2	107437	.	1.214096	[illegible]
12336	NM_009795,XM_006539488,XM_011250419,XM_011250420	Capns1	calpain, small subunit 1	protein_coding	NP_033925.2;XP_006539551.1;XP_011248721.1;XP_011248722.1	88266	.	102.297613	[illegible]
12337	NM_001301250,NM_007602,XM_006507275,XM_030242033,XM_030242034,XM_030242035,XM_030242036,XM_030242037,XM_030242038	Capn5	calpain 5	protein_coding	NP_001288179.1;NP_031628.1;XP_006507338.1;XP_030097893.1;XP_030097894.1;XP_030097895.1;XP_030097896.1;XP_030097897.1;XP_030097898.1	1100859	.	2.844839	[illegible]
12338	NM_007603	Capn6	calpain 6	protein_coding	NP_031629.3	1100850	.	115.157951	[illegible]
12339	NM_001360187,NM_009796,XM_006518502,XM_030247631,XR_001781094,XR_003950823	Capn7	calpain 7	protein_coding	NP_001347116.1;NP_033926.1;XP_006518565.1;XP_030103491.1	1338030	.	18.697947	[illegible]
12340	NM_001355044,NM_009797	Capza1	capping protein (actin filament) muscle Z-line, alpha 1	protein_coding	NP_001341973.1;NP_033927.2	106227	.	36.044215	[illegible]
12343	NM_007604	Capza2	capping protein (actin filament) muscle Z-line, alpha 2	protein_coding	NP_031630.1	106222	.	49.198452	[illegible]
12345	NM_001037761,NM_001271405,NM_009798,XM_006538504	Capzb	capping protein (actin filament) muscle Z-line, beta	protein_coding	NP_001032850.1;NP_001258334.1;NP_033928.1;XP_006538567.1	104652	.	84.244402	[illegible]
12346	NM_001083957,NM_009799,XM_011248137	Car1	carbonic anhydrase 1	protein_coding	NP_001077426.1;NP_033929.2;XP_011246439.1	88268	.	0.359324	[illegible]
12348	NM_001368349,NM_009800,XM_006540588,XM_006540589	Car11	carbonic anhydrase 11	protein_coding	NP_001355278.1;NP_033930.1;XP_006540651.1;XP_006540652.1	1336193	.	1.128816	[illegible]
12349	NM_001357334,NM_009801	Car2	carbonic anhydrase 2	protein_coding	NP_001344263.1;NP_033931.4	88269	.	4.823887	[illegible]
12350	NM_007606	Car3	carbonic anhydrase 3	protein_coding	NP_031632.2	88270	.	80.440193	[illegible]
12351	NM_007607,XM_006532095	Car4	carbonic anhydrase 4	protein_coding	NP_031633.1;XP_006532158.1	1096574	.	0.136719	[illegible]
12353	NM_009802	Car6	carbonic anhydrase 6	protein_coding	NP_033932.2	1333786	.	0.000000	[illegible]
12354	NM_001301164,NM_001301165,NM_053070,XM_006530617,XM_006530618,XM_017312542,XM_030243263,XM_030243264	Car7	carbonic anhydrase 7	protein_coding	NP_001288093.1;NP_001288094.1;NP_444300.1;XP_006530680.1;XP_006530681.1;XP_017168031.1;XP_030099123.1;XP_030099124.1	103100	.	0.394983	[illegible]
12355	NM_001243062,NM_001243063,NM_009803,XM_006496628,XM_006496629,XM_011238743,XM_030244435,XR_003947935,XR_003947948,XR_003947950,XR_373594,XR_373595	Nr1i3	nuclear receptor subfamily 1, group I, member 3	protein_coding	NP_001229991.1;NP_001229992.1;NP_033933.2;XP_006496691.1;XP_006496692.1;XP_011237045.1;XP_030100295.1	1346307	.	0.000000	[illegible]
12359	NM_009804	Cat	catalase	protein_coding	NP_033934.2	88271	.	40.633926	[illegible]
12361	NM_001284503,NM_001284504,NM_001284505,NM_009806,XM_006527560,XM_006527561,XM_011247431,XM_011247432,XM_011247433,XM_011247434,XM_011247435,XM_011247436,XM_011247437,XM_011247438,XM_011247439,XM_011247440,XM_011247441,XM_017318366,XM_017318367,XM_017318368,XM_017318369,XM_017318370,XM_017318371	Cask	calcium/calmodulin-dependent serine protein kinase (MAGUK family)	protein_coding	NP_001271432.1;NP_001271433.1;NP_001271434.1;NP_033936.2;XP_006527623.1;XP_006527624.1;XP_011245733.1;XP_011245734.1;XP_011245735.1;XP_011245736.1;XP_011245737.1;XP_011245738.1;XP_011245739.1;XP_011245740.1;XP_011245741.1;XP_011245742.1;XP_011245743.1;XP_017173855.1;XP_017173856.1;XP_017173857.1;XP_017173858.1;XP_017173859.1;XP_017173860.1	1309489	.	7.996265	[illegible]
12362	NM_009807	Casp1	caspase 1	protein_coding	NP_033937.2	96544	.	1.410669	[illegible]
12363	NM_007609,XM_006509837,XM_006509838,XM_006509839	Casp4	caspase 4, apoptosis-related cysteine peptidase	protein_coding	NP_031635.2;XP_006509900.1;XP_006509901.1;XP_006509902.1	107700	.	1.387469	[illegible]
12364	NM_009808,XM_006509840	Casp12	caspase 12	protein_coding	NP_033938.3;XP_006509903.1	1312922	.	3.978139	[illegible]
12366	NM_007610,XR_003956158,XR_377417,XR_377418	Casp2	caspase 2	protein_coding	NP_031636.1	97295	.	10.887208	[illegible]
12367	NM_001284409,NM_009810,XM_017312543,XM_030243266	Casp3	caspase 3	protein_coding	NP_001271338.1;NP_033940.1;XP_017168032.1;XP_030099126.1	107739	.	13.057810	[illegible]
12368	NM_009811	Casp6	caspase 6	protein_coding	NP_033941.3	1312921	.	7.959359	[illegible]
12369	NM_007611,XM_006526616	Casp7	caspase 7	protein_coding	NP_031637.1;XP_006526679.2	109383	.	5.531364	[illegible]
12370	NM_001080126,NM_001277926,NM_009812,XM_006495634	Casp8	caspase 8	protein_coding	NP_001073595.1;NP_001264855.1;NP_033942.1;XP_006495697.1	1261423	.	10.642931	[illegible]
12371	NM_001277932,NM_001355176,NM_015733	Casp9	caspase 9	protein_coding	NP_001264861.1;NP_001342105.1;NP_056548.2	1277950	.	7.279606	[illegible]
12372	NM_009813	Casq1	calsequestrin 1	protein_coding	NP_033943.2	1309468	.	21.293787	[illegible]
12373	NM_001355663,NM_009814	Casq2	calsequestrin 2	protein_coding	NP_001342592.1;NP_033944.2	1309469	.	12.460239	[illegible]
12374	NM_013803,XM_006521729,XM_006521730,XM_006521731,XM_006521732,XM_006521735	Casr	calcium-sensing receptor	protein_coding	NP_038831.2;XP_006521792.1;XP_006521793.1;XP_006521794.1;XP_006521795.1;XP_006521798.1	1351351	.	0.122861	[illegible]
12380	NM_001301153,NM_001301155,NM_001301156,NM_001301157,NM_001301158,NM_001301160,NM_001301181,NM_009817,XM_006517056,XM_006517059,XM_011244466,XM_011244467,XM_011244469,XM_017315374	Cast	calpastatin	protein_coding	NP_001288082.1;NP_001288084.1;NP_001288085.1;NP_001288086.1;NP_001288087.1;NP_001288089.1;NP_001288110.1;NP_033947.1;XP_006517119.1;XP_006517122.1;XP_011242768.1;XP_011242769.1;XP_011242771.1;XP_017170863.1	1098236	.	23.598422	[illegible]
12385	NM_009818,XM_006525548,XM_030250299	Ctnna1	catenin (cadherin associated protein), alpha 1	protein_coding	NP_033948.1;XP_006525611.1;XP_030106159.1	88274	.	52.347420	[illegible]
12386	NM_001109764,NM_001355193,NM_009819,NM_145732,XM_011241195,XM_030255129,XM_030255130	Ctnna2	catenin (cadherin associated protein), alpha 2	protein_coding	NP_001103234.1;NP_001342122.1;NP_033949.2;NP_663785.2;XP_011239497.1;XP_030110989.1;XP_030110990.1	88275	.	0.131804	[illegible]
12387	NM_001165902,NM_007614	Ctnnb1	catenin (cadherin associated protein), beta 1	protein_coding	NP_001159374.1;NP_031640.1	88276	.	277.220229	[illegible]
12388	NM_001085448,NM_001085449,NM_001085450,NM_001085453,NM_001355064,NM_001355065,NM_001355066,NM_001355067,NM_007615,XM_006498625,XM_006498626,XM_006498628,XM_006498630,XM_006498631,XM_006498632,XM_006498633,XM_006498634,XM_006498636,XM_006498638,XM_006498639,XM_017315179,XM_017315208,XM_030247016,XM_030247019	Ctnnd1	catenin (cadherin associated protein), delta 1	protein_coding	NP_001078917.1;NP_001078918.1;NP_001078919.1;NP_001078922.1;NP_001341993.1;NP_001341994.1;NP_001341995.1;NP_001341996.1;NP_031641.2;XP_006498688.1;XP_006498689.1;XP_006498691.1;XP_006498693.1;XP_006498694.1;XP_006498695.1;XP_006498696.1;XP_006498697.1;XP_006498699.1;XP_006498701.1;XP_006498702.1;XP_017170668.1;XP_017170697.1;XP_030102876.1;XP_0301	105100	.	35.623453	[illegible]
12389	NM_001243064,NM_007616,XM_006504974,XM_006504975,XM_006504976	Cav1	caveolin 1, caveolae protein	protein_coding	NP_001229993.1;NP_031642.1;XP_006505037.1;XP_006505038.1;XP_006505039.1	102709	.	66.910521	[illegible]
12390	NM_001277756,NM_016900	Cav2	caveolin 2	protein_coding	NP_001264685.1;NP_058596.1	107571	.	11.602677	[illegible]
12391	NM_007617	Cav3	caveolin 3	protein_coding	NP_031643.1	107570	.	5.408276	[illegible]
12393	NM_001145920,NM_001146038,NM_001271627,NM_001271630,NM_001271631,NM_009820,NR_073392,NR_073425	Runx2	runt related transcription factor 2	protein_coding	NP_001139392.1;NP_001139510.1;NP_001258556.1;NP_001258559.1;NP_001258560.1;NP_033950.2	99829	.	38.764042	[illegible]
12394	NM_001111021,NM_001111022,NM_001111023,NM_009821	Runx1	runt related transcription factor 1	protein_coding	NP_001104491.1;NP_001104492.1;NP_001104493.1;NP_033951.2	99852	.	7.431989	[illegible]
12395	NM_001111026,NM_001111027,NM_009822,XM_006537568,XM_006537569,XM_006537571,XM_006537572,XM_030253116,XM_030253117,XM_030253118,XM_030253119,XM_030253120,XM_030253121	Runx1t1	RUNX1 translocation partner 1	protein_coding	NP_001104496.1;NP_001104497.1;NP_033952.1;XP_006537631.1;XP_006537632.1;XP_006537634.1;XP_006537635.1;XP_030108976.1;XP_030108977.1;XP_030108978.1;XP_030108979.1;XP_030108980.1;XP_030108981.1	104793	.	1.143608	[illegible]
12396	NM_001285446,NM_009823,NM_172860,XM_006498640,XM_006498641,XM_006498642,XM_011239268,XM_030247023	Cbfa2t2	CBFA2/RUNX1 translocation partner 2	protein_coding	NP_001272375.1;NP_033953.1;NP_766448.1;XP_006498703.1;XP_006498704.1;XP_006498705.1;XP_011237570.1;XP_030102883.1	1333833	.	7.112307	[illegible]
12397	gene-Cbfa2t2-ps1	Cbfa2t2-ps1	CBFA2/RUNX1 translocation partner 2, pseudogene 1	pseudogene	.	1333788	.	0.084901	[illegible]
12398	NM_001109873,NM_009824,NM_177289,XM_011248289,XM_011248291,XM_011248292,XM_011248293,XM_017312544,XM_017312547,XM_030243267,XM_030243268,XM_030243269,XM_030243270,XM_030243271	Cbfa2t3	CBFA2/RUNX1 translocation partner 3	protein_coding	NP_001103343.1;NP_033954.2;NP_796263.1;XP_011246591.2;XP_011246593.1;XP_011246594.1;XP_011246595.1;XP_017168033.1;XP_017168036.1;XP_030099127.1;XP_030099128.1;XP_030099129.1;XP_030099130.1;XP_030099131.1	1338013	.	1.796091	[illegible]
12399	NM_001369050,NM_019732,XM_006538506	Runx3	runt related transcription factor 3	protein_coding	NP_001355979.1;NP_062706.2;XP_006538569.1	102672	.	4.508928	[illegible]
12400	NM_001161456,NM_001161457,NM_001161458,NM_022309	Cbfb	core binding factor beta	protein_coding	NP_001154928.1;NP_001154929.1;NP_001154930.1;NP_071704.3	99851	.	62.494057	[illegible]
12401	NM_007618	Serpina6	serine (or cysteine) peptidase inhibitor, clade A, member 6	protein_coding	NP_031644.1	88278	.	0.029409	[illegible]
12402	NM_007619,XM_006509963,XM_017313115,XM_030244033	Cbl	Casitas B-lineage lymphoma	protein_coding	NP_031645.2;XP_006510026.1;XP_017168604.1;XP_030099893.1	88279	.	6.827795	[illegible]
12404	NM_019626	Cbln1	cerebellin 1 precursor protein	protein_coding	NP_062600.2	88281	.	0.230224	[illegible]
12405	NM_001302356,NM_001361142,NM_001361143,NM_001361144,NM_001361145,NM_172633	Cbln2	cerebellin 2 precursor protein	protein_coding	NP_001289285.1;NP_001348071.1;NP_001348072.1;NP_001348073.1;NP_001348074.1;NP_766221.1	88282	.	0.960672	[illegible]
12406	NM_001111043,NM_001111044,NM_001285776,NM_009825,XM_006507278,XM_006507279	Serpinh1	serine (or cysteine) peptidase inhibitor, clade H, member 1	protein_coding	NP_001104513.1;NP_001104514.1;NP_001272705.1;NP_033955.2;XP_006507341.1;XP_006507342.1	88283	.	861.755817	[illegible]
12408	NM_007620	Cbr1	carbonyl reductase 1	protein_coding	NP_031646.2	88284	.	17.575979	[illegible]
12409	NM_007621,XM_006532096	Cbr2	carbonyl reductase 2	protein_coding	NP_031647.1;XP_006532159.1	107200	.	3.336439	[illegible]
12411	NM_001271353,NM_144855,NM_178224,XM_006523549,XM_006523550,XM_006523551	Cbs	cystathionine beta-synthase	protein_coding	NP_001258282.1;NP_659104.1;NP_835742.1;XP_006523612.1;XP_006523613.1;XP_006523614.1	88285	.	0.234543	[illegible]
12412	NM_001362560,NM_001362561,NM_001362563,NM_001362564,NM_007622,NR_155761,XM_006532102,XM_017314242,XR_003949267,XR_003949268,XR_003949269	Cbx1	chromobox 1	protein_coding	NP_001349489.1;NP_001349490.1;NP_001349492.1;NP_001349493.1;NP_031648.1;XP_006532165.1;XP_017169731.1	105369	.	37.069087	[illegible]
12416	NM_007623,XM_006532103,XM_006532104,XM_006532105	Cbx2	chromobox 2	protein_coding	NP_031649.2;XP_006532166.1;XP_006532167.1;XP_006532168.1	88289	.	3.226642	[illegible]
12417	NM_001355002,NM_007624,XM_017321374	Cbx3	chromobox 3	protein_coding	NP_001341931.1;NP_031650.3;XP_017176863.1	108515	.	62.484010	[illegible]
12418	NM_007625	Cbx4	chromobox 4	protein_coding	NP_031651.2	1195985	.	4.691426	[illegible]
12419	NM_001076789,NM_001110216,NM_001358950,NM_007626,XM_006520372,XM_006520374,XM_006520375,XM_006520376,XM_006520377,XM_030248308	Cbx5	chromobox 5	protein_coding	NP_001070257.1;NP_001103686.1;NP_001345879.1;NP_031652.1;XP_006520435.1;XP_006520437.1;XP_006520438.1;XP_006520439.1;XP_006520440.1;XP_030104168.1	109372	.	19.942108	[illegible]
12421	NM_009826,XM_006495443,XM_011238338,XM_011238339,XM_011238340,XM_011238341	Rb1cc1	RB1-inducible coiled-coil 1	protein_coding	NP_033956.2;XP_006495506.1;XP_011236640.1;XP_011236641.1;XP_011236642.1;XP_011236643.1	1341850	.	9.372810	[illegible]
12424	NM_001284508,NM_031161,XM_017313116	Cck	cholecystokinin	protein_coding	NP_001271437.1;NP_112438.1;XP_017168605.1	88297	.	0.132377	[illegible]
12426	NM_007627,XM_006507280,XM_006507281	Cckbr	cholecystokinin B receptor	protein_coding	NP_031653.1;XP_006507343.1;XP_006507344.1	99479	.	0.000000	[illegible]
12427	NM_001305221,NM_007628,XM_006500949,XM_006500950,XM_006500951,XM_006500952,XM_006500954,XM_030252399,XM_030252400,XM_030252401,XM_030252402,XR_003954275	Ccna1	cyclin A1	protein_coding	NP_001292150.1;NP_031654.2;XP_006501012.1;XP_006501013.1;XP_006501014.1;XP_006501015.1;XP_006501017.1;XP_030108259.1;XP_030108260.1;XP_030108261.1;XP_030108262.1	108042	.	0.229247	[illegible]
12428	NM_009828	Ccna2	cyclin A2	protein_coding	NP_033958.2	108069	.	18.978966	[illegible]
12442	NM_007630	Ccnb2	cyclin B2	protein_coding	NP_031656.2	88311	.	15.985459	[illegible]
12443	NM_007631,XM_011241977	Ccnd1	cyclin D1	protein_coding	NP_031657.1;XP_011240279.1	88313	.	27.298679	[illegible]
12444	NM_009829,XM_006505459,XM_006505460	Ccnd2	cyclin D2	protein_coding	NP_033959.1;XP_006505522.1;XP_006505523.1	88314	.	58.522519	[illegible]
12445	NM_001081635,NM_001081636,NM_007632,XM_006523552,XM_006523553,XM_011246262	Ccnd3	cyclin D3	protein_coding	NP_001075104.1;NP_001075105.1;NP_031658.1;XP_006523615.1;XP_006523616.1;XP_011244564.1	88315	.	83.171619	[illegible]
12447	NM_007633	Ccne1	cyclin E1	protein_coding	NP_031659.2	88316	.	5.161927	[illegible]
12448	NM_001037134,NM_001282943,NM_009830,XM_006537573,XM_006537574,XM_006537575,XM_011249913	Ccne2	cyclin E2	protein_coding	NP_001032211.1;NP_001269872.1;NP_033960.1;XP_006537636.1;XP_006537637.1;XP_006537638.1;XP_011248215.1	1329034	.	3.933486	[illegible]
12449	NM_007634,XM_006523554	Ccnf	cyclin F	protein_coding	NP_031660.3;XP_006523617.1	102551	.	4.923579	[illegible]
12450	NM_009831,XR_003949271	Ccng1	cyclin G1	protein_coding	NP_033961.1	102890	.	19.763884	[illegible]
12452	NM_007635	Ccng2	cyclin G2	protein_coding	NP_031661.3	1095734	.	10.286982	[illegible]
12453	NM_017367,XM_006534736,XM_030254086	Ccni	cyclin I	protein_coding	NP_059063.2;XP_006534799.1;XP_030109946.1	1341077	.	48.734205	[illegible]
12454	NM_009832,XM_006515443,XM_006515444,XM_006515445	Ccnk	cyclin K	protein_coding	NP_033962.2;XP_006515506.1;XP_006515507.1;XP_006515508.1	1276106	.	8.913343	[illegible]
12455	NM_001368702,NM_009833,NR_160872	Ccnt1	cyclin T1	protein_coding	NP_001355631.1;NP_033963.1	1328363	.	8.379779	[illegible]
12457	NM_009834,XM_006500956	Noct	nocturnin	protein_coding	NP_033964.1;XP_006501019.1	109382	.	3.660829	[illegible]
12458	NM_001190333,NM_001190334,NM_001190335,NM_001190336,NM_001190337,NM_001190338,NM_009835,XM_006523286,XM_006523288,XM_006523291,XM_006523292,XM_011246186,XM_011246188,XM_017317218,XM_017317219,XM_017317220	Ccr6	chemokine (C-C motif) receptor 6	protein_coding	NP_001177262.1;NP_001177263.1;NP_001177264.1;NP_001177265.1;NP_001177266.1;NP_001177267.1;NP_033965.1;XP_006523349.1;XP_006523351.1;XP_006523354.1;XP_006523355.1;XP_011244488.1;XP_011244490.1;XP_017172707.1;XP_017172708.1;XP_01717270	1333797	.	0.000000	[illegible]
12460	NM_016892,XR_003952739	Ccs	copper chaperone for superoxide dismutase	protein_coding	NP_058588.1	1333783	.	21.638165	[illegible]
12461	NM_001358767,NM_007636	Cct2	chaperonin containing Tcp1, subunit 2 (beta)	protein_coding	NP_001345696.1;NP_031662.2	107186	.	112.936486	[illegible]
12462	NM_009836	Cct3	chaperonin containing Tcp1, subunit 3 (gamma)	protein_coding	NP_033966.1	104708	.	63.465179	[illegible]
12464	NM_009837	Cct4	chaperonin containing Tcp1, subunit 4 (delta)	protein_coding	NP_033967.1	104689	.	62.926617	[illegible]
12465	NM_001348035,NM_007637	Cct5	chaperonin containing Tcp1, subunit 5 (epsilon)	protein_coding	NP_001334964.1;NP_031663.1	107185	.	85.514146	[illegible]
12466	NM_009838	Cct6a	chaperonin containing Tcp1, subunit 6a (zeta)	protein_coding	NP_033968.1	107943	.	64.468048	[illegible]
12467	NM_001291242,NM_009839,XM_006532107,XM_006532108,XM_030245508,XR_001779867,XR_003949272,XR_879512	Cct6b	chaperonin containing Tcp1, subunit 6b (zeta)	protein_coding	NP_001278171.1;NP_033969.2;XP_006532170.1;XP_006532171.1;XP_030101368.1	1329013	.	0.151513	[illegible]
12468	NM_007638	Cct7	chaperonin containing Tcp1, subunit 7 (eta)	protein_coding	NP_031664.3	107184	.	50.426941	[illegible]
12469	NM_009840	Cct8	chaperonin containing Tcp1, subunit 8 (theta)	protein_coding	NP_033970.3	107183	.	64.626747	[illegible]
12475	NM_009841	Cd14	CD14 antigen	protein_coding	NP_033971.1	88318	.	1.868014	[illegible]
12476	NM_001111049,NM_001111050,NM_009842	Cd151	CD151 antigen	protein_coding	NP_001104519.1;NP_001104520.1;NP_033972.2	1096360	.	22.765788	[illegible]
12477	NM_001281976,NM_009843	Ctla4	cytotoxic T-lymphocyte-associated protein 4	protein_coding	NP_001268905.1;NP_033973.2	88556	.	0.105684	[illegible]
12478	NM_001357091,NM_009844,XM_006507283,XM_030242039,XM_030242040,XM_030242041,XM_030242042	Cd19	CD19 antigen	protein_coding	NP_001344020.1;NP_033974.2;XP_006507346.1;XP_030097899.1;XP_030097900.1;XP_030097901.1;XP_030097902.1	88319	.	0.119723	[illegible]
12479	NM_007639,XM_006500958,XM_030252403,XM_030252404	Cd1d1	CD1d1 antigen	protein_coding	NP_031665.2;XP_006501021.1;XP_030108263.1;XP_030108264.1	107674	.	18.774539	[illegible]
12480	NR_038188	Cd1d2	CD1d2 antigen	transcribed_pseudogene	.	107675	.	0.508553	[illegible]
12481	NM_013486	Cd2	CD2 antigen	protein_coding	NP_038514.1	88320	.	0.070937	[illegible]
12482	NM_007641	Ms4a1	membrane-spanning 4-domains, subfamily A, member 1	protein_coding	NP_031667.1	88321	.	0.067631	[illegible]
12483	NM_001043317,NM_009845,NR_102722,NR_102723,XM_006539489,XM_006539490,XM_006539491,XM_006539492,XM_006539493,XM_006539494,XM_030242043,XR_391039	Cd22	CD22 antigen	protein_coding	NP_001036782.1;NP_033975.3;XP_006539552.1;XP_006539553.1;XP_006539554.1;XP_006539555.1;XP_006539556.1;XP_006539557.1;XP_030097903.1	88322	.	0.157705	[illegible]
12484	NM_009846	Cd24a	CD24a antigen	protein_coding	NP_033976.1	88323	.	48.701012	[illegible]
12487	NM_007642	Cd28	CD28 antigen	protein_coding	NP_031668.3	88327	.	0.237912	[illegible]
12488	NM_009847,XM_011246263,XM_011246264,XM_011246265,XR_001782031	Cd2ap	CD2-associated protein	protein_coding	NP_033977.3;XP_011244565.1;XP_011244566.1;XP_011244567.1	1330281	.	26.478239	[illegible]
12489	NM_001111058,NM_021293,XM_006540590	Cd33	CD33 antigen	protein_coding	NP_001104528.1;NP_067268.1;XP_006540653.1	99440	.	2.005664	[illegible]
12490	NM_001111059,NM_133654	Cd34	CD34 antigen	protein_coding	NP_001104529.1;NP_598415.1	88329	.	48.281367	[illegible]
12491	NM_001159555,NM_001159556,NM_001159557,NM_001159558,NM_007643,XM_006535620,XM_006535621,XM_006535622,XM_006535623,XM_006535624,XM_006535625,XM_030254088	Cd36	CD36 molecule	protein_coding	NP_001153027.1;NP_001153028.1;NP_001153029.1;NP_001153030.1;NP_031669.3;XP_006535683.1;XP_006535684.1;XP_006535685.1;XP_006535686.1;XP_006535687.1;XP_006535688.1;XP_030109948.	107899	.	29.909905	[illegible]
12492	NM_007644	Scarb2	scavenger receptor class B, member 2	protein_coding	NP_031670.1	1196458	.	22.626503	[illegible]
12493	NM_001290802,NM_001290804,NM_007645,XM_006540592,XM_006540593	Cd37	CD37 antigen	protein_coding	NP_001277731.1;NP_001277733.1;NP_031671.1;XP_006540655.1;XP_006540656.1	88330	.	2.631023	[illegible]
12494	NM_007646	Cd38	CD38 antigen	protein_coding	NP_031672.2	107474	.	2.140313	[illegible]
12495	NM_001304721,NM_009848,XM_006526618,XM_006526619,XM_006526620,XM_017318047	Entpd1	ectonucleoside triphosphate diphosphohydrolase 1	protein_coding	NP_001291650.1;NP_033978.1;XP_006526681.1;XP_006526682.1;XP_006526683.1;XP_017173536.1	102805	.	9.347903	[illegible]
12496	NM_009849	Entpd2	ectonucleoside triphosphate diphosphohydrolase 2	protein_coding	NP_033979.2	1096863	.	2.476082	[illegible]
12497	NM_001355068,NM_001370629,NM_001370630,NM_001370631,NM_172117,XM_017315214,XM_030247027,XM_030247029,XM_030247031,XR_003950191	Entpd6	ectonucleoside triphosphate diphosphohydrolase 6	protein_coding	NP_001341997.1;NP_001357558.1;NP_001357559.1;NP_001357560.1;NP_742115.2;XP_017170703.1;XP_030102887.1;XP_030102889.1;XP_030102891.1	1202295	.	9.729432	[illegible]
12499	NM_001026214,NM_001286049,NM_001286058,NM_007647,XM_030246533	Entpd5	ectonucleoside triphosphate diphosphohydrolase 5	protein_coding	NP_001021385.1;NP_001272978.1;NP_001272987.1;NP_031673.2;XP_030102393.1	1321385	.	7.269340	[illegible]
12500	NM_013487,XM_006509965	Cd3d	CD3 antigen, delta polypeptide	protein_coding	NP_038515.3;XP_006510028.1	88331	.	0.000000	[illegible]
12501	NM_007648,XM_006509966	Cd3e	CD3 antigen, epsilon polypeptide	protein_coding	NP_031674.1;XP_006510029.1	88332	.	0.000000	[illegible]
12502	NM_009850	Cd3g	CD3 antigen, gamma polypeptide	protein_coding	NP_033980.1	88333	.	0.000000	[illegible]

12503	NM_001113391,NM_001113392,NM_001113393,NM_031162,NR_103716,XM_011238745,XM_011238746,XM_017313285	Cd247	CD247 antigen	protein_coding	NP_001106862.1;NP_001106863.1;NP_001106864.2;NP_112439.1;XP_011237047.1;XP_011237048.1;XP_017168774.1	88334	.	0.318029
12504	NM_013488,XM_006505461,XM_006505462	Cd4	CD4 antigen	protein_coding	NP_038516.1;XP_006505524.1;XP_006505525.1	88335	.	0.901657
12505	NM_001039150,NM_001039151,NM_001177785,NM_001177786,NM_001177787,NM_009851,XM_006498645,XM_006498646,XM_006498647,XM_006498648,XM_006498649	Cd44	CD44 antigen	protein_coding	NP_001034239.1;NP_001034240.1;NP_001171256.1;NP_001171257.1;NP_001171258.1;NP_033981.2;XP_006498708.1;XP_006498709.1;XP_006498710.1;XP_006498711.1;XP_006498712.1	88338	.	84.296360
12506	NM_001360767,NM_007649,XM_006496636,XM_006496637,XM_030244776	Cd48	CD48 antigen	protein_coding	NP_001347696.1;NP_031675.1;XP_006496699.1;XP_006496700.1;XP_030100636.1	88339	.	1.545179
12507	NM_007650,XM_006526621	Cd5	CD5 antigen	protein_coding	NP_031676.3;XP_006526684.1	88340	.	0.505135
12508	NM_007651	Cd53	CD53 antigen	protein_coding	NP_031677.1	88341	.	3.283561
12509	NM_001111060,NM_007652,XM_006498650,XM_006498651,XM_030247036	Cd59a	CD59a antigen	protein_coding	NP_001104530.1;NP_031678.1;XP_006498713.1;XP_006498714.1;XP_030102896.1	109177	.	5.836199
12511	NM_001037801,NM_009852,XM_006526622	Cd6	CD6 antigen	protein_coding	NP_001032890.1;NP_033982.3;XP_006526685.1	103566	.	0.000000
12512	NM_001042580,NM_001282966,NM_007653,XM_030244851,XM_030244852	Cd63	CD63 antigen	protein_coding	NP_001036045.1;NP_001269895.1;NP_031679.1;XP_030100711.1;XP_030100712.1	99529	.	372.390196
12514	NM_001291058,NR_110993	Cd68	CD68 antigen	protein_coding	NP_001277987.1	88342	.	27.234577
12515	NM_001033122,XM_006505463	Cd69	CD69 antigen	protein_coding	NP_001028294.1;XP_006505526.1	88343	.	0.028830
12516	NM_009854	Cd7	CD7 antigen	protein_coding	NP_033984.1	88344	.	0.000000
12517	NM_001110320,NM_001110321,NM_001110322,NM_007654,XM_006537576,XM_006537577,XM_006537578,XM_006537579,XM_006537580,XM_006537581,XM_006537582,XM_030253123,XM_030253124,XM_030253125,XM_030253126,XM_030253127,XM_030253128,XM_030253129,XM_030253130,XM_030253131	Cd72	CD72 antigen	protein_coding	NP_001103790.1;NP_001103791.1;NP_001103792.1;NP_031680.2;XP_006537639.1;XP_006537640.1;XP_006537641.1;XP_006537642.1;XP_006537643.1;XP_006537644.1;XP_006537645.1;XP_030108983.1;XP_030108984.1;XP_030108985.1;XP_030108986.1;XP_030108987.1;XP_030108988.1;XP_030108989.1;XP_030108990.1;XP_030108	88345	.	0.930863
12518	NM_007655	Cd79a	CD79A antigen (immunoglobulin-associated alpha)	protein_coding	NP_031681.2	101774	.	0.462532
12519	NM_001359898,NM_009855,XM_006521737,XM_030248944,XM_030248945	Cd80	CD80 antigen	protein_coding	NP_001346827.1;NP_033985.3;XP_006521800.1;XP_030104804.1;XP_030104805.1	101775	.	0.203654
12520	NM_133655	Cd81	CD81 antigen	protein_coding	NP_598416.1	1096398	.	183.206894
12521	NM_001136055,NM_001271430,NM_001271431,NM_001271432,NM_001271461,NM_001271462,NM_007656,XM_030247042	Cd82	CD82 antigen	protein_coding	NP_001129527.1;NP_001258359.1;NP_001258360.1;NP_001258361.1;NP_001258390.1;NP_001258391.1;NP_031682.1;XP_030102902.1	104651	.	19.177496
12522	NM_001289915,NM_009856	Cd83	CD83 antigen	protein_coding	NP_001276844.1;NP_033986.1	1328316	.	0.607008
12523	NM_001252472,NM_001289470,NM_013489,XM_011238747,XM_030244793	Cd84	CD84 antigen	protein_coding	NP_001239401.1;NP_001276399.1;NP_038517.1;XP_011237049.1;XP_030100653.1	1336885	.	3.434106
12524	NM_019388,XM_006521741,XM_011245812,XR_003951744,XR_384517	Cd86	CD86 antigen	protein_coding	NP_062261.3;XP_006521804.1;XP_011244114.1	101773	.	1.126945
12525	NM_001081110,NM_009857	Cd8a	CD8 antigen, alpha chain	protein_coding	NP_001074579.1;NP_033987.1	88346	.	0.000000
12527	NM_007657	Cd9	CD9 antigen	protein_coding	NP_031683.1	88348	.	182.347122
12530	NM_007658,XM_006511929,XM_006511930,XM_006511931	Cdc25a	cell division cycle 25A	protein_coding	NP_031684.3;XP_006511992.1;XP_006511993.1;XP_006511994.1	103198	.	7.486803
12531	NM_001111075,NM_023117,XR_374398	Cdc25b	cell division cycle 25B	protein_coding	NP_001104545.1;NP_075606.1	99701	.	11.739758
12532	NM_009860,XM_006525551,XM_011246819	Cdc25c	cell division cycle 25C	protein_coding	NP_033990.2;XP_006525614.1;XP_011245121.1	88350	.	4.039078
12534	NM_007659	Cdk1	cyclin-dependent kinase 1	protein_coding	NP_031685.2	88351	.	10.701294
12537	NM_001347308,NM_001355567,NM_001355568,NM_001355569,NM_007661,XM_006538508,XM_006538511,XM_017319932,XM_030253132,XM_030253133,XM_030253134,XM_030253135,XM_030253136,XM_030253137,XM_030253138,XR_001784083,XR_001784	Cdk11b	cyclin-dependent kinase 11B	protein_coding	NP_001334237.1;NP_001342496.1;NP_001342497.1;NP_001342498.1;NP_031687.2;XP_006538571.1;XP_006538574.1;XP_017175421.1;XP_030108992.1;XP_030108993.1;XP_030108994.1;XP_030108995.1;XP_030108996.1;XP_030108997.1;XP_030108998.1	88353	.	38.221266
12539	NM_016742,XM_006509967	Cdc37	cell division cycle 37	protein_coding	NP_058022.1;XP_006510030.1	109531	.	52.264095
12540	NM_001243769,NM_009861,XM_030253139,XM_030253140,XM_030253141,XM_030253142	Cdc42	cell division cycle 42	protein_coding	NP_001230698.1;NP_033991.1;XP_030108999.1;XP_030109000.1;XP_030109001.1;XP_030109002.1	106211	.	159.700403
12544	NM_001161623,NM_001358206,NM_009862,XM_006521744	Cdc45	cell division cycle 45	protein_coding	NP_001155095.1;NP_001345135.1;NP_033992.2;XP_006521807.1	1338073	.	4.924610
12545	NM_001271566,NM_001271567,NM_001271568,NM_009863,XM_006534737,XM_017320634,XM_030254089	Cdc7	cell division cycle 7 (S. cerevisiae)	protein_coding	NP_001258495.1;NP_001258496.1;NP_001258497.1;NP_033993.2;XP_006534800.1;XP_017176123.1;XP_030109949.1	1309511	.	6.059488
12549	NM_020260	Arhgap31	Rho GTPase activating protein 31	protein_coding	NP_064656.2	1333857	.	13.575069
12550	NM_009864	Cdh1	cadherin 1	protein_coding	NP_033994.1	88354	.	0.156380
12552	NM_009866,XM_006530624,XM_006530626,XM_030243272	Cdh11	cadherin 11	protein_coding	NP_033996.4;XP_006530687.1;XP_006530689.1;XP_030099132.1	99217	.	145.217752
12554	NM_019707,XM_006530628,XM_011248295,XM_017312550	Cdh13	cadherin 13	protein_coding	NP_062681.2;XP_006530691.2;XP_011246597.1;XP_017168039.1	99551	.	7.563293
12555	NM_007662	Cdh15	cadherin 15	protein_coding	NP_031688.2	106672	.	2.855189
12556	NM_001252627,NM_001252628,NM_007663,XM_006530629	Cdh16	cadherin 16	protein_coding	NP_001239556.1;NP_001239557.1;NP_031689.1;XP_006530692.1	106671	.	0.000000
12557	NM_019753,XM_006537583,XM_006537584	Cdh17	cadherin 17	protein_coding	NP_062727.1;XP_006537646.1;XP_006537647.1	1095414	.	0.039158
12558	NM_007664,XM_006525553	Cdh2	cadherin 2	protein_coding	NP_031690.3;XP_006525616.1	88355	.	29.839874
12560	NM_001037809,NM_007665	Cdh3	cadherin 3	protein_coding	NP_001032898.1;NP_031691.1	88356	.	2.350755
12561	NM_001316723,NM_009867,XM_011239921,XM_030247044,XM_030247045	Cdh4	cadherin 4	protein_coding	NP_001303652.1;NP_033997.1;XP_011238223.1;XP_030102904.1;XP_030102905.1	99218	.	0.475805
12562	NM_009868,XM_006530630	Cdh5	cadherin 5	protein_coding	NP_033998.2;XP_006530693.1	105057	.	32.158742
12563	NM_007666,XM_006520017,XM_006520018,XM_017316420,XM_017316421	Cdh6	cadherin 6	protein_coding	NP_031692.2;XP_006520080.1;XP_006520081.1;XP_017171909.1;XP_017171910.1	107435	.	0.656252
12564	NM_001039154,NM_001285913,NM_001285914,NM_007667,NR_133570,XM_006530631,XM_006530634,XM_017312551,XM_030243274	Cdh8	cadherin 8	protein_coding	NP_001034243.1;NP_001272842.1;NP_001272843.1;NP_031693.2;XP_006530694.1;XP_006530697.1;XP_017168040.1;XP_030099134.1	107434	.	0.732361
12565	NM_009869,XM_006520020,XM_011245337	Cdh9	cadherin 9	protein_coding	NP_033999.1;XP_006520083.1;XP_011243639.1	107433	.	0.000000
12566	NM_016756,NM_183417,XM_006513162,XM_011243321,XM_030244853,XM_030244854	Cdk2	cyclin-dependent kinase 2	protein_coding	NP_058036.1;NP_904326.1;XP_006513225.1;XP_011241623.1;XP_030100713.1;XP_030100714.1	104772	.	23.567931
12567	NM_001355005,NM_009870	Cdk4	cyclin-dependent kinase 4	protein_coding	NP_001341934.1;NP_034000.1	88357	.	116.293943
12568	NM_007668,NR_156449,XM_006535626	Cdk5	cyclin-dependent kinase 5	protein_coding	NP_031694.1;XP_006535689.1	101765	.	13.840637
12569	NM_009871	Cdk5r1	cyclin-dependent kinase 5, regulatory subunit 1 (p35)	protein_coding	NP_034001.1	101764	.	1.447100
12570	NM_009872	Cdk5r2	cyclin-dependent kinase 5, regulatory subunit 2 (p39)	protein_coding	NP_034002.2	1330828	.	0.049181
12571	NM_009873,XM_030254091	Cdk6	cyclin-dependent kinase 6	protein_coding	NP_034003.1;XP_030109951.1	1277162	.	2.940097
12572	NM_009874	Cdk7	cyclin-dependent kinase 7	protein_coding	NP_034004.2	102956	.	8.953503
12575	NM_001111099,NM_007669	Cdkn1a	cyclin-dependent kinase inhibitor 1A (P21)	protein_coding	NP_001104569.1;NP_031695.1	104556	.	57.769356
12576	NM_009875	Cdkn1b	cyclin-dependent kinase inhibitor 1B	protein_coding	NP_034005.2	104565	.	14.745299
12577	NM_001161624,NM_001354981,NM_009876,XM_006508467,XM_006508468,XM_006508469,XM_006508470,XM_030242044,XM_030242045,XM_030242046,XM_030242047,XM_030242048	Cdkn1c	cyclin-dependent kinase inhibitor 1C (P57)	protein_coding	NP_001155096.1;NP_001341910.1;NP_034006.3;XP_006508530.1;XP_006508531.1;XP_006508532.1;XP_006508533.1;XP_030097904.1;XP_030097905.1;XP_030097906.1;XP_030097907.1;XP_030097908.	104564	.	156.944191
12578	NM_001040654,NM_009877	Cdkn2a	cyclin dependent kinase inhibitor 2A	protein_coding	NP_001035744.1;NP_034007.1	104738	.	0.096295
12579	NM_007670	Cdkn2b	cyclin dependent kinase inhibitor 2B	protein_coding	NP_031696.1	104737	.	1.525288
12580	NM_001301368,NM_007671	Cdkn2c	cyclin dependent kinase inhibitor 2C	protein_coding	NP_001288297.1;NP_031697.1	105388	.	14.043467
12581	NM_009878,XM_006509968	Cdkn2d	cyclin dependent kinase inhibitor 2D	protein_coding	NP_034008.2;XP_006510031.1	105387	.	10.179660
12583	NM_033037,NR_151681	Cdo1	cysteine dioxygenase 1, cytosolic	protein_coding	NP_149026.1	105925	.	77.583140
12585	NM_001354986,NM_007672	Cdr2	cerebellar degeneration-related 2	protein_coding	NP_001341915.1;NP_031698.2	1100885	.	14.324436
12587	NM_019394	Mia	melanoma inhibitory activity	protein_coding	NP_062267.2	109615	.	60.900127
12589	NM_001358917,NM_001358918,NM_001358919,NM_009879,XM_006530128,XM_011248162,XM_030254092,XM_030254093,XR_003955576	Ift81	intraflagellar transport 81	protein_coding	NP_001345846.1;NP_001345847.1;NP_001345848.1;NP_034009.2;XP_006530191.1;XP_011246464.1;XP_030109952.1;XP_030109953.1	1098597	.	7.705353
12590	NM_009880	Cdx1	caudal type homeobox 1	protein_coding	NP_034010.3	88360	.	0.027073
12593	NM_001123386,NM_009881,XM_006516536,XM_030247135,XR_003950423,XR_382045	Cdyl	chromodomain protein, Y chromosome-like	protein_coding	NP_001116858.1;NP_034011.1;XP_006516599.1;XP_030102995.1	1339956	.	6.752286
12606	NM_001287514,NM_001287515,NM_001287521,NM_001287523,NM_007678	Cebpa	CCAAT/enhancer binding protein (C/EBP), alpha	protein_coding	NP_001274443.1;NP_001274444.1;NP_001274450.1;NP_001274452.1;NP_031704.2	99480	.	2.317670
12607	NM_001024806,XM_006523558	Cebpz	CCAAT/enhancer binding protein zeta	protein_coding	NP_001019977.1;XP_006523621.1	109386	.	11.856220
12608	NM_001287738,NM_001287739,NM_009883	Cebpb	CCAAT/enhancer binding protein (C/EBP), beta	protein_coding	NP_001274667.1;NP_001274668.1;NP_034013.1	88373	.	1.903538
12609	NM_007679	Cebpd	CCAAT/enhancer binding protein (C/EBP), delta	protein_coding	NP_031705.3	103573	.	4.466208
12611	NM_009884	Cebpg	CCAAT/enhancer binding protein (C/EBP), gamma	protein_coding	NP_034014.1	104982	.	8.287862
12614	NM_009886,XM_006520379,XM_006520380,XM_006520381,XM_006520382,XM_030248310	Celsr1	cadherin, EGF LAG seven-pass G-type receptor 1	protein_coding	NP_034016.2;XP_006520442.1;XP_006520443.1;XP_006520444.1;XP_006520445.1;XP_030104170.1	1100883	.	0.315851
12615	NM_001302129,NM_001302130,NM_001302131,NM_001302132,NM_007681,NR_126074,XM_011240699,XM_030254094	Cenpa	centromere protein A	protein_coding	NP_001289058.1;NP_001289059.1;NP_001289060.1;NP_001289061.1;NP_031707.1;XP_011239001.1;XP_030109954.1	88375	.	16.312179
12616	NM_007682	Cenpb	centromere protein B	protein_coding	NP_031708.2	88376	.	50.250790
12617	NM_001345902,NM_001345903,NM_001345904,NM_007683,XM_017320636,XM_017320638,XM_030254095,XM_030254096,XM_030254097	Cenpc1	centromere protein C1	protein_coding	NP_001332831.1;NP_001332832.1;NP_001332833.1;NP_031709.2;XP_017176125.1;XP_017176127.1;XP_030109955.1;XP_030109956.1;XP_030109957.1	99700	.	9.474998
12622	NM_009887	Cer1	cerberus 1, DAN family BMP antagonist	protein_coding	NP_034017.1	1201414	.	0.154028
12626	NM_007684,XM_006517074	Cetn3	centrin 3	protein_coding	NP_031710.1;XP_006517137.1	1097706	.	35.725427
12627	NM_007685,NR_153806	Cfc1	cripto, FRL-1, cryptic family 1	protein_coding	NP_031711.1	109448	.	0.045643
12628	NM_009888	Cfh	complement component factor h	protein_coding	NP_034018.2	88385	.	112.809677
12630	NM_001329552,NM_007686,XM_006500962,XM_006500963	Cfi	complement component factor i	protein_coding	NP_001316481.1;NP_031712.2;XP_006501025.1;XP_006501026.1	105937	.	0.000000
12631	NM_007687	Cfl1	cofilin 1, non-muscle	protein_coding	NP_031713.1	101757	.	348.973236
12632	NM_007688,XM_006515452	Cfl2	cofilin 2, muscle	protein_coding	NP_031714.1;XP_006515515.1	101763	.	28.052261
12633	NM_001289704,NM_001293804,NM_001293805,NM_001355056,NM_009805,NM_207653,NR_149255,NR_149256,NR_149257,NR_149258,XM_006495635,XM_011238420,XM_011238421,XM_011238422,XM_011238423,XM_017313559,XM_030244988	Cflar	CASP8 and FADD-like apoptosis regulator	protein_coding	NP_001276633.1;NP_001280733.1;NP_001280734.1;NP_001341985.1;NP_033935.2;NP_997536.1;XP_006495698.1;XP_011236722.1;XP_011236723.1;XP_011236724.1;XP_011236725.1;XP_017169048.1;XP_030100848.1	1336166	.	13.383477
12638	NM_021050	Cftr	cystic fibrosis transmembrane conductance regulator	protein_coding	NP_066388.1	88388	.	0.059519
12642	NM_009890	Ch25h	cholesterol 25-hydroxylase	protein_coding	NP_034020.1	1333869	.	1.579131
12643	NM_007689	Chad	chondroadherin	protein_coding	NP_031715.1	1096866	.	30.399437
12648	NM_007690	Chd1	chromodomain helicase DNA binding protein 1	protein_coding	NP_031716.2	88393	.	6.980178
12649	NM_007691,XM_017313117	Chek1	checkpoint kinase 1	protein_coding	NP_031717.2;XP_017168606.1	1202065	.	3.932714
12651	NM_007692,NR_037153,NR_037154	Chkb	choline kinase beta	protein_coding	NP_031718.1	1328313	.	27.355075
12652	NM_007693	Chga	chromogranin A	protein_coding	NP_031719.1	88394	.	0.000000
12653	NM_007694	Chgb	chromogranin B	protein_coding	NP_031720.1	88395	.	0.292488
12654	NM_007695,XM_006529111,XM_006529112	Chil1	chitinase-like 1	protein_coding	NP_031721.2;XP_006529174.1;XP_006529175.1	1340899	.	2.322950
12655	NM_009892	Chil3	chitinase-like 3	protein_coding	NP_034022.2	1330860	.	0.588872
12659	NM_007696	Ovgp1	oviductal glycoprotein 1	protein_coding	NP_031722.1	106661	.	1.667764
12660	NM_001271496,NM_013490,NR_073190,XM_006531646,XM_006531648,XM_011248599,XM_017318048,XM_017318049,XM_030250724,XM_030250725,XM_030250726,XM_030250727,XM_030250728,XM_030250729,XM_030250730,XM_030250731,XR_003952740,XR_388248	Chka	choline kinase alpha	protein_coding	NP_001258425.1;NP_038518.2;XP_006531709.1;XP_006531711.1;XP_011246901.1;XP_017173537.1;XP_017173538.1;XP_030106584.1;XP_030106585.1;XP_030106586.1;XP_030106587.1;XP_030106588.1;XP_030106589.1;XP_030106590.1;XP_030106591.1	107760	.	8.704729
12661	NM_007697,XM_006505467,XM_006505468,XM_006505469,XM_006505470,XM_006505471,XM_006505472,XM_006505473,XM_011241198,XM_017321375,XM_017321376,XM_017321377,XM_030255131,XM_030255132,XM_030255133	Chl1	cell adhesion molecule L1-like	protein_coding	NP_031723.2;XP_006505530.1;XP_006505531.1;XP_006505532.1;XP_006505533.1;XP_006505534.1;XP_006505535.1;XP_006505536.1;XP_011239500.1;XP_017176864.1;XP_017176865.1;XP_017176866.1;XP_030110991.1;XP_030110992.1;XP_030110993.1	1098266	.	0.171022
12662	NM_001370788,NM_001370789,NM_001370790,NM_018818,XM_006528381,XM_006528382,XM_006528383,XM_006528384,XM_030251211,XR_001782732,XR_387043	Chm	choroidermia (RAB escort protein 1)	protein_coding	NP_001357717.1;NP_001357718.1;NP_001357719.1;NP_061288.2;XP_006528444.1;XP_006528445.1;XP_006528446.1;XP_006528447.1;XP_030107071.1	892979	.	6.857272
12663	NM_021350	Chml	choroideremia-like	protein_coding	NP_067325.2	101913	.	2.457545
12667	NM_001278041,NM_001354995,NM_001354996,NM_009893,XM_006521745,XM_011245814,XM_011245815,XM_011245816,XM_011245817,XM_017316860,XM_017316861,XM_030248947,XM_030248948,XM_030248950,XR_001781784,XR_003951745,XR_003951746,XR_875721	Chrd	chordin	protein_coding	NP_001264970.1;NP_001341924.1;NP_001341925.1;NP_034023.1;XP_006521808.1;XP_011244116.1;XP_011244117.1;XP_011244118.1;XP_011244119.1;XP_017172349.1;XP_017172350.1;XP_030104807.1;XP_030104808.1;XP_030104810.1	1313268	.	3.487817
12669	NM_001112697,NM_007698	Chrm1	cholinergic receptor, muscarinic 1, CNS	protein_coding	NP_001106167.1;NP_031724.2	88396	.	0.007532
12671	NM_033269,XM_006516468	Chrm3	cholinergic receptor, muscarinic 3, cardiac	protein_coding	NP_150372.1;XP_006516531.1	88398	.	0.064855
12672	NM_007699,XM_006498654	Chrm4	cholinergic receptor, muscarinic 4	protein_coding	NP_031725.1;XP_006498717.1	88399	.	0.074604
12675	NM_001162410,NM_007700,XM_030250732,XR_003952741,XR_003952742	Chuk	conserved helix-loop-helix ubiquitous kinase	protein_coding	NP_001155882.1;NP_031726.2;XP_030106592.1	99484	.	13.779470
12683	NM_007702	Cidea	cell death-inducing DNA fragmentation factor, alpha subunit-like effector	protein_coding	NP_031728.2	1270845	.	0.755918
12684	NM_009894	Cideb	cell death-inducing DNA fragmentation factor, alpha subunit-like effector	protein_coding	NP_034024.2	1270844	.	0.057442
12695	NM_001005784,NM_001005787,NM_001355177,NM_007704,NM_172696,XM_006502699,XM_006502700,XM_006502701,XM_006502702,XM_006502703,XM_006502704,XM_006502705,XM_006502706,XM_006502707	Patj	PATJ, crumbs cell polarity complex component	protein_coding	NP_001005784.1;NP_001005787.1;NP_001342106.1;NP_031730.1;NP_766284.2;XP_006502762.1;XP_006502763.1;XP_006502764.1;XP_006502765.1;XP_006502766.1;XP_006502767.1;XP_006502768.1;XP_006502769.1;XP_006502770.1	1277960	.	2.076654
12696	NM_007705,NR_152857,XM_006513164,XM_006513165,XM_030244855	Cirbp	cold inducible RNA binding protein	protein_coding	NP_031731.1;XP_006513227.1;XP_006513228.1;XP_030100715.1	893588	.	59.035838
12700	NM_001317354,NM_009895,XM_006511635	Cish	cytokine inducible SH2-containing protein	protein_coding	NP_001304283.1;NP_034025.1;XP_006511698.1	103159	.	3.140751
12702	NM_007707,XM_011248707,XM_030245509	Socs3	suppressor of cytokine signaling 3	protein_coding	NP_031733.1;XP_011247009.1;XP_030101369.1	1201791	.	7.076900
12703	NM_001271603,NM_009896	Socs1	suppressor of cytokine signaling 1	protein_coding	NP_001258532.1;NP_034026.1	1354910	.	5.346711
12704	NM_007708,XM_006530130,XM_006530131,XM_006530132,XM_006530133,XM_006530134,XM_006530135,XM_006530136,XM_006530137,XM_006530138,XM_006530139,XM_006530140,XM_006530141,XM_006530142,XM_006530143,XM_006530145,XM_006530146,XM_006530147,XM_006530148,XM_006530149,XM_006530150,XM_006530151,XM_006530152,XM_006530153,XM_011248163,XM_017320640,XM_017320641,XM_017320643,XM_017320644,XM_017320645,XM_017320646,XM_030254098,XM_030254099,XM_030254100	Cit	citron	protein_coding	NP_031734.3;XP_006530193.1;XP_006530194.1;XP_006530195.1;XP_006530196.1;XP_006530197.1;XP_006530198.1;XP_006530199.1;XP_006530200.1;XP_006530201.1;XP_006530202.1;XP_006530203.1;XP_006530204.1;XP_006530205.1;XP_006530206.1;XP_006530208.1;XP_006530209.1;XP_006530210.1;XP_006530211.1;XP_006530212.1;XP_006530213.1;XP_006530214.1;XP_006530215.1;XP_006530216.1;XP_011246465.1;XP_017176129.1;XP_017176130.1;XP_017176132.1;XP_017176133.1;XP_017176134.1;XP_017176135.1;XP_030109958.1;XP_030109959.1;XP_030109960.1	105313	.	1.640115
12705	NM_001276466,NM_001276473,NM_001276474,NM_007709	Cited1	Cbp/p300-interacting transactivator with Glu/Asp-rich carboxy-terminal domain 1	protein_coding	NP_001263395.1;NP_001263402.1;NP_001263403.1;NP_031735.1	108023	.	1.028202
12708	gene-Ck-ps3	Ck-ps3	creatine kinase pseudogene 3	pseudogene	.	88406	.	0.000000
12709	NM_021273	Ckb	creatine kinase, brain	protein_coding	NP_067248.1	88407	.	84.540726
12715	NM_007710	Ckm	creatine kinase, muscle	protein_coding	NP_031736.1	88413	.	109.508217
12716	NM_001355069,NM_009897,XM_006498656	Ckmt1	creatine kinase, mitochondrial 1, ubiquitous	protein_coding	NP_001341998.1;NP_034027.1;XP_006498719.1	99441	.	0.116692
12721	NM_001301374,NM_009898,XM_006507284,XM_006507285	Coro1a	coronin, actin binding protein 1A	protein_coding	NP_001288303.1;NP_034028.1;XP_006507347.1;XP_006507348.1	1345961	.	6.138381
12722	NM_009899,NR_130895,XM_006500965,XM_006500968,XM_011240004,XR_003954276	Clca3a1	chloride channel accessory 3A1	protein_coding	NP_034029.2;XP_006501028.1;XP_006501031.1;XP_011238306.1	1316732	.	2.160120
12723	NM_001363712,NM_013491,XM_006505476,XR_003956160	Clcn1	chloride channel, voltage-sensitive 1	protein_coding	NP_001350641.1;NP_038519.1;XP_006505539.1	88417	.	0.343595
12724	NM_009900,XM_006521749,XM_017316862,XM_030248952,XR_001781785,XR_003951747,XR_003951748,XR_003951749,XR_384	Clcn2	chloride channel, voltage-sensitive 2	protein_coding	NP_034030.2;XP_006521812.1;XP_017172351.1;XP_030104812.1	105061	.	5.533436
12725	NM_007711,NM_173873,NM_173874,NM_173876,XM_006509259,XM_006509260,XM_017312552,XM_017312553,XR_001778397	Clcn3	chloride channel, voltage-sensitive 3	protein_coding	NP_031737.1;NP_776298.1;NP_776299.1;NP_776301.1;XP_006509322.1;XP_006509323.1;XP_017168041.1;XP_017168042.1	103555	.	5.214593
12727	NM_001302386,NM_001302387,NM_001357102,NM_001357103,NM_001357104,NM_011334,NR_126156,NR_126157,XM_006539502,XM_030242049,XM_030242050,XM_030242051,XM_030242052,XM_030242053,XM_030242054,XM_030242055,XM_030242056,XR_003946379,XR_003946380,XR_391040	Clcn4	chloride channel, voltage-sensitive 4	protein_coding	NP_001289315.1;NP_001289316.1;NP_001344031.1;NP_001344032.1;NP_001344033.1;NP_035464.3;XP_006539565.1;XP_030097909.1;XP_030097910.1;XP_030097911.1;XP_030097912.1;XP_030097913.1;XP_030097914.1;XP_030097915.1;XP_030097916.1	104571	.	21.155488
12728	NM_001243762,NM_016691,XM_006527562,XM_011247442,XM_011247443,XM_011247444,XM_011247445,XM_011247446	Clcn5	chloride channel, voltage-sensitive 5	protein_coding	NP_001230691.1;NP_057900.3;XP_006527625.1;XP_011245744.1;XP_011245745.1;XP_011245746.1;XP_011245747.1;XP_011245748.1	99486	.	5.542962
12729	NM_023671	Clns1a	chloride channel, nucleotide-sensitive, 1A	protein_coding	NP_076160.1	109638	.	12.852720
12733	NM_001146307,NM_024412	Clcnka	chloride channel, voltage-sensitive Ka	protein_coding	NP_001139779.1;NP_077723.3	1329026	.	0.051991
12737	NM_016674	Cldn1	claudin 1	protein_coding	NP_057883.1	1276109	.	2.268709
12738	NM_016675,XM_006528486,XM_006528487,XM_006528488,XM_006528489,XM_006528490	Cldn2	claudin 2	protein_coding	NP_057884.1;XP_006528549.1;XP_006528550.1;XP_006528551.1;XP_006528552.1;XP_006528553.1	1276110	.	0.259561
12739	NM_009902	Cldn3	claudin 3	protein_coding	NP_034032.1	1329044	.	0.101765
12740	NM_009903	Cldn4	claudin 4	protein_coding	NP_034033.1	1313314	.	0.000000
12741	NM_013805	Cldn5	claudin 5	protein_coding	NP_038833.2	1276112	.	10.358324
12745	NM_001329627,NM_001329680,NM_009904,XM_006530636,XM_011248296,XM_011248297,XM_011248298,XM_017312554,XM_030243275,XM_030243276	Clgn	calmegin	protein_coding	NP_001316556.1;NP_001316609.1;NP_034034.2;XP_006530699.1;XP_011246598.1;XP_011246599.1;XP_011246600.1;XP_017168043.1;XP_030099135.1;XP_030099136.1	107472	.	0.182984
12747	NM_001042634,NR_027853,NR_027854	Clk1	CDC-like kinase 1	protein_coding	NP_001036099.1	107403	.	75.530065
12748	NM_001163432,NM_007712	Clk2	CDC-like kinase 2	protein_coding	NP_001156904.1;NP_031738.2	1098669	.	24.281073
12750	NM_001304744,NM_001362535,NM_001362536,NM_001362538,NM_001362539,NM_001362540,NM_001362541,NM_007714,XM_006532112,XM_006532114,XM_006532118,XM_011248708,XM_017314243,XM_030245510,XM_030245511,XM_030245512	Clk4	CDC like kinase 4	protein_coding	NP_001291673.1;NP_001349464.1;NP_001349465.1;NP_001349467.1;NP_001349468.1;NP_001349469.1;NP_001349470.1;NP_031740.1;XP_006532175.1;XP_006532177.1;XP_006532181.1;XP_011247010.1;XP_017169732.1;XP_030101370.1;XP_030101371.1;XP_03010137	1098551	.	29.527037
12751	NM_009906,XM_011241668	Tpp1	tripeptidyl peptidase I	protein_coding	NP_034036.1;XP_011239970.1	1336194	.	23.790369
12752	NM_001146311,NM_001329789,NM_009907,XM_006507288,XM_006507289,XM_006507292,XM_006507293,XM_017321963,XM_030242057,XM_030242058,XM_030242059,XR_378213	Cln3	ceroid lipofuscinosis, neuronal 3, juvenile (Batten, Spielmeyer-Vogt disease)	protein_coding	NP_001139783.1;NP_001316718.1;NP_034037.3;XP_006507351.1;XP_006507352.1;XP_006507355.1;XP_006507356.1;XP_017177452.1;XP_030097917.1;XP_030097918.1;XP_030097919.1	107537	.	5.694728
12753	NM_001289826,NM_001305222,NM_007715,XM_011249402,XM_017320648,XM_017320649,XM_017320650,XM_017320651,XM_030254101,XM_030254102,XM_030254103,XR_001784601,XR_001784602	Clock	circadian locomotor output cycles kaput	protein_coding	NP_001276755.1;NP_001292151.1;NP_031741.1;XP_011247704.1;XP_017176137.1;XP_017176138.1;XP_017176139.1;XP_017176140.1;XP_030109961.1;XP_030109962.1;XP_030109963.1	99698	.	6.183332
12757	NM_001080384,NM_001080385,NM_001080386,NM_001290470,NM_001369156,NM_001369157,NM_001369158,NM_001369159,NM_016760,XM_006537587,XM_030253143,XM_030253145,XM_030253146	Clta	clathrin, light polypeptide (Lca)	protein_coding	NP_001073853.1;NP_001073854.1;NP_001073855.1;NP_001277399.1;NP_001356085.1;NP_001356086.1;NP_001356087.1;NP_001356088.1;NP_058040.2;XP_006537650.1;XP_030109003.1;XP_030109005.1;XP_030109006.1	894297	.	82.886072
12759	NM_013492,XM_006518503,XM_006518504,XM_030247632	Clu	clusterin	protein_coding	NP_038520.2;XP_006518566.1;XP_006518567.1;XP_030103492.1	88423	.	18.095454
12763	NM_001111110,NM_001284519,NM_001284520,NM_007717,XM_011244265,XM_011244266,XM_011244274	Cmah	cytidine monophospho-N-acetylneuraminic acid hydroxylase	protein_coding	NP_001104580.1;NP_001271448.1;NP_001271449.1;NP_031743.3;XP_011242567.1;XP_011242568.1;XP_011242576.1	103227	.	0.146800
12764	NM_009908,XM_030255134	Cmas	cytidine monophospho-N-acetylneuraminic acid synthetase	protein_coding	NP_034038.2;XP_030110994.1	1337124	.	21.138203
12765	NM_009909,XM_006495638	Cxcr2	chemokine (C-X-C motif) receptor 2	protein_coding	NP_034039.1;XP_006495701.1	105303	.	0.177353
12766	NM_009910	Cxcr3	chemokine (C-X-C motif) receptor 3	protein_coding	NP_034040.1	1277207	.	0.201745
12767	NM_001356509,NM_009911,XM_006529114	Cxcr4	chemokine (C-X-C motif) receptor 4	protein_coding	NP_001343438.1;NP_034041.2;XP_006529177.1	109563	.	3.066943
12768	NM_009912	Ccr1	chemokine (C-C motif) receptor 1	protein_coding	NP_034042.3	104618	.	5.045866
12769	NM_001166625,NM_009913	Ccr9	chemokine (C-C motif) receptor 9	protein_coding	NP_001160097.1;NP_034043.1	1341902	.	0.011303
12770	NM_007718	Ccr1l1	chemokine (C-C motif) receptor 1-like 1	protein_coding	NP_031744.3	104617	.	0.021954
12771	NM_009914,XM_017313119,XM_017313120	Ccr3	chemokine (C-C motif) receptor 3	protein_coding	NP_034044.3;XP_017168608.1;XP_017168609.1	104616	.	0.426520
12772	NM_009915,XM_006512428,XM_011243063,XM_011243064	Ccr2	chemokine (C-C motif) receptor 2	protein_coding	NP_034045.1;XP_006512491.1;XP_011241365.1;XP_011241366.1	106185	.	1.330518
12773	NM_009916,XM_006511932,XM_006511933,XM_006511934,XM_011242933	Ccr4	chemokine (C-C motif) receptor 4	protein_coding	NP_034046.2;XP_006511995.1;XP_006511996.1;XP_006511997.1;XP_011241235.1	107824	.	0.000000
12774	NM_009917	Ccr5	chemokine (C-C motif) receptor 5	protein_coding	NP_034047.2	107182	.	0.360311
12775	NM_001301713,NM_007719	Ccr7	chemokine (C-C motif) receptor 7	protein_coding	NP_001288642.1;NP_031745.2	103011	.	0.109493
12776	NM_007720	Ccr8	chemokine (C-C motif) receptor 8	protein_coding	NP_031746.1	1201402	.	0.000000
12777	NM_007721	Ccr10	chemokine (C-C motif) receptor 10	protein_coding	NP_031747.2	1096320	.	0.041528
12778	NM_001271607,NM_007722	Ackr3	atypical chemokine receptor 3	protein_coding	NP_001258536.1;NP_031748.2	109562	.	31.491077
12780	NM_013806,XM_006526625	Abcc2	ATP-binding cassette, sub-family C (CFTR/MRP), member 2	protein_coding	NP_038834.2;XP_006526688.1	1352447	.	0.000000
12785	NM_001109745,NM_001109746,NM_001347325,NM_001355195,NM_013493	Cnbp	cellular nucleic acid binding protein	protein_coding	NP_001103215.1;NP_001103216.1;NP_001334254.1;NP_001342124.1;NP_038521.1	88431	.	115.465948
12789	NM_007724,XM_006527761	Cnga2	cyclic nucleotide gated channel alpha 2	protein_coding	NP_031750.2;XP_006527824.1	108040	.	0.095532
12790	NM_001282010,NM_009918,XM_017313745,XM_017313748	Cnga3	cyclic nucleotide gated channel alpha 3	protein_coding	NP_001268939.1;NP_034048.1;XP_017169234.1;XP_017169237.1	1341818	.	0.000000
12793	NM_001355011,NM_009919	Cnih1	cornichon family AMPA receptor auxiliary protein 1	protein_coding	NP_001341940.1;NP_034049.2	1277202	.	56.994442
12794	NM_001302333,NM_009920	Cnih2	cornichon family AMPA receptor auxiliary protein 2	protein_coding	NP_001289262.1;NP_034050.1	1277225	.	2.161439
12795	NM_001313916,NM_013807	Plk3	polo like kinase 3	protein_coding	NP_001300845.1;NP_038835.2	109604	.	1.869950
12796	NM_009921	Camp	cathelicidin antimicrobial peptide	protein_coding	NP_034051.2	108443	.	0.367281
12797	NM_009922,XM_006509969,XM_011242388	Cnn1	calponin 1	protein_coding	NP_034052.3;XP_006510032.1;XP_011240690.1	104979	.	1.814005
12798	NM_007725	Cnn2	calponin 2	protein_coding	NP_031751.1	105093	.	134.158524

12799	NM_001146318,NM_009923,XM_006532120,XM_017314245	Cnp	2',3'-cyclic nucleotide 3' phosphodiesterase	protein_coding	NP_001139790.1;NP_034053.2;XP_006532183.1;XP_017169734.1	88437	.		8.332540	[illegible]
12801	NM_001355020,NM_001355021,NM_001365881,NM_007726,XM_006537591,XM_006537592,XM_006537593,XM_006537594,XM_006537595,XM_030253148,XM_030253149	Cnr1	cannabinoid receptor 1 (brain)	protein_coding	NP_001341949.1;NP_001341950.1;NP_001352810.1;NP_031752.1;XP_006537654.1;XP_006537655.1;XP_006537656.1;XP_006537657.1;XP_006537658.1;XP_030109008.1;XP_030109009.1	104615	.		0.518614	[illegible]
12802	NM_001305278,NM_009924	Cnr2	cannabinoid receptor 2 (macrophage)	protein_coding	NP_001292207.1;NP_034054.3	104650	.		0.232866	[illegible]
12803	NM_170786	Cntf	ciliary neurotrophic factor	protein_coding	NP_740756.1	88439	.		0.812823	[illegible]
12804	NM_001136056,NM_016673,XM_006537596,XM_006537597,XM_006537598,XM_006537599,XM_011249916,XM_011249917,XM_017319941,XM_017319942,XM_030253151,XM_030253153,XM_030253154	Cntfr	ciliary neurotrophic factor receptor	protein_coding	NP_001129528.1;NP_057882.2;XP_006537659.1;XP_006537660.1;XP_006537661.1;XP_006537662.1;XP_011248218.1;XP_011248219.1;XP_017175430.1;XP_017175431.1;XP_030109011.1;XP_030109013.1;XP_030109014.1	99605	.		7.699261	[illegible]
12805	NM_001159647,NM_001159648,NM_001358051,NM_007727,XM_006520383,XM_017316422,XM_017316423	Cntn1	contactin 1	protein_coding	NP_001153119.1;NP_001153120.1;NP_001344980.1;NP_031753.1;XP_006520446.1;XP_017171911.1;XP_017171912.1	105980	.		0.525662	[illegible]
12807	NM_001146323,NM_001146324,NM_080634,XM_006535377,XM_006535378,XM_006535379,XM_006535380,XM_030252407,XM_030252408,XR_001783645,XR_001783646,XR_003954277,XR_38958	Hps3	HPS3, biogenesis of lysosomal organelles complex 2 subunit 1	protein_coding	NP_001139795.1;NP_001139796.1;NP_542365.3;XP_006535440.1;XP_006535441.1;XP_006535442.1;XP_006535443.1;XP_030108267.1;XP_030108268.1	2153839	.		5.417667	[illegible]
12808	NM_001282993,NM_001282994,NM_172496,XM_006514481,XM_006514485,XM_011243651,XM_011243652,XM_011243653,XM_011243654,XM_011243655,XM_011243656,XM_030245514,XM_030245515	Cobl	cordon-bleu WH2 repeat	protein_coding	NP_001269922.1;NP_001269923.1;NP_766084.3;XP_006514544.1;XP_006514548.1;XP_011241953.1;XP_011241954.1;XP_011241955.1;XP_011241956.1;XP_011241957.1;XP_011241958.1;XP_030101374.1;XP_030101375.1	105056	.		2.516253	[illegible]
12810	NM_001198835,NM_007728,XM_017314943	Coch	cochlin	protein_coding	NP_001185764.1;NP_031754.1;XP_017170432.1	1278313	.		0.239405	[illegible]
12812	NM_016706	Coil	coilin	protein_coding	NP_057915.2	104842	.		4.843760	[illegible]
12813	NM_009925	Col10a1	collagen, type X, alpha 1	protein_coding	NP_034055.1	88445	.		83.414108	[illegible]
12814	NM_007729,XM_006500969,XM_006500970,XM_006500971,XM_030252409,XR_375486	Col11a1	collagen, type XI, alpha 1	protein_coding	NP_031755.2;XP_006501032.1;XP_006501033.1;XP_006501034.1;XP_030108269.1	88446	.		392.662174	[illegible]
12815	NM_001317722,NM_009926,XM_006523559,XM_006523560,XM_006523561,XM_006523562,XM_006523563,XM_006523564,XM_006523566,XM_006523567,XM_006523568,XM_006523569,XM_006523570,XM_006523571,XR_385275,XR_385276	Col11a2	collagen, type XI, alpha 2	protein_coding	NP_001304651.1;NP_034056.1;XP_006523622.1;XP_006523623.1;XP_006523624.1;XP_006523625.1;XP_006523626.1;XP_006523627.1;XP_006523629.1;XP_006523630.1;XP_006523631.1;XP_006523632.1;XP_006523633.1;XP_006523634.1	88447	.		354.509155	[illegible]
12816	NM_001290308,XM_006510797,XM_006510800	Col12a1	collagen, type XII, alpha 1	protein_coding	NP_001277237.1;XP_006510860.1;XP_006510863.1	88448	.		357.961221	[illegible]
12817	NM_001304757,NM_007731,XM_006513170,XM_006513171,XM_011243322,XM_011243323,XM_011243324,XM_011243325,XM_011243326,XM_011243327,XM_011243328,XM_011243329,XM_011243330,XM_011243331,XM_011243332,XM_011243333,XM_011243335,XM_011243336,XM_011243337,XM_011243339,XM_011243341,XM_011243342,XM_011243343,XM_011243344,XM_030244856,XM_030244857,XM_030244858	Col13a1	collagen, type XIII, alpha 1	protein_coding	NP_001291686.1;NP_031757.1;XP_006513233.1;XP_006513234.1;XP_011241624.1;XP_011241625.1;XP_011241626.1;XP_011241627.1;XP_011241628.1;XP_011241629.1;XP_011241630.1;XP_011241631.1;XP_011241632.1;XP_011241633.1;XP_011241634.1;XP_011241635.1;XP_011241637.1;XP_011241638.1;XP_011241639.1;XP_011241641.1;XP_011241643.1;XP_011241644.1;XP_011241645.1;XP_011241646.1;XP_030100716.1;XP_030100717.1;XP_030100718.1	1277201	.		29.820181	[illegible]
12818	NM_001368422,NM_181277,XM_006520385	Col14a1	collagen, type XIV, alpha 1	protein_coding	NP_001355351.1;NP_851794.3;XP_006520448.1	1341272	.		45.063319	[illegible]

[illegible]

13047	NM_001291233,NM_001291234,NM_001291238,NM_001291239,NM_001291240,NM_009986,NM_198602,XM_006504350,XM_006504352,XM_006504354,XM_006504356,XM_017320654,XM_030254106,XM_030254107,XM_030254108,XM_030254109,XM_030254110,XM_030254111,XM_030254112,XM_030254113,XM_030254114,XM_030254115	Cux1	cut-like homeobox 1	protein_coding	NP_001278162.1;NP_001278163.1;NP_001278167.1;NP_001278168.1;NP_001278169.1;NP_034116.3;NP_941004.2;XP_006504413.1;XP_006504415.1;XP_006504417.1;XP_006504419.1;XP_017176143.1;XP_030109966.1;XP_030109967.1;XP_030109968.1;XP_030109969.1;XP_030109970.1;XP_030109971.1;XP_030109972.1;XP_030109973.1;XP_030109974.1;XP_030109975.1	88568	.	12.341609	[illegible]
13048	NM_001312908,NM_007804,XM_006530157,XM_011248166,XM_011248167,XM_011248168,XM_011248169,XM_011248170,XM_011248171,XM_011248172,XM_011248173,XM_011248174,XM_011248176,XM_017320655,XM_030254116,XM_030254117,XM_030254118,XM_030254119,XM_030254120	Cux2	cut-like homeobox 2	protein_coding	NP_001299837.1;NP_031830.2;XP_006530220.1;XP_011246468.1;XP_011246469.1;XP_011246470.1;XP_011246471.1;XP_011246472.1;XP_011246473.2;XP_011246474.1;XP_011246475.1;XP_011246476.1;XP_011246478.1;XP_017176144.1;XP_030109976.1;XP_030109977.1;XP_030109978.1;XP_030109979.1;XP_030109980.1	107321	.	0.244021	[illegible]
13051	NM_009987,XM_011242934	Cx3cr1	chemokine (C-X3-C motif) receptor 1	protein_coding	NP_034117.3;XP_011241236.1	1333815	.	4.317938	[illegible]
13052	NM_001025192,NM_001276263,NM_009988,XM_006522883,XM_006522884,XM_017316864,XM_030248956	Cxadr	coxsackie virus and adenovirus receptor	protein_coding	NP_001020363.1;NP_001263192.1;NP_034118.1;XP_006522946.1;XP_006522947.1;XP_017172353.1;XP_030104816.1	1201679	.	3.759592	[illegible]
13056	NM_001356374,NM_007805,XM_006532131,XM_006532132,XM_006532133,XM_006532134	Cyb561	cytochrome b-561	protein_coding	NP_001343303.1;NP_031831.2;XP_006532194.1;XP_006532195.1;XP_006532196.1;XP_006532197.1	103253	.	1.616922	[illegible]
13057	NM_001301284,NM_007806	Cyba	cytochrome b-245, alpha polypeptide	protein_coding	NP_001288213.1;NP_031832.2	1316658	.	38.071668	[illegible]
13058	NM_007807,XM_006527565	Cybb	cytochrome b-245, beta polypeptide	protein_coding	NP_031833.3;XP_006527628.1	88574	.	1.344401	[illegible]
13063	NM_007808	Cycs	cytochrome c, somatic	protein_coding	NP_031834.1	88578	.	14.839745	[illegible]
13064	gene-Cycs-ps1	Cycs-ps1	cytochrome c, pseudogene 1	pseudogene	.	109500	.	0.000000	[illegible]
13067	NM_009989,XM_011239273	Cyct	cytochrome c, testis	protein_coding	NP_034119.1;XP_011237575.1	88579	.	0.000000	[illegible]
13070	NM_001346787,NM_019779	Cyp11a1	cytochrome P450, family 11, subfamily a, polypeptide 1	protein_coding	NP_001333716.1;NP_062753.3	88582	.	1.035560	[illegible]
13074	NM_007809	Cyp17a1	cytochrome P450, family 17, subfamily a, polypeptide 1	protein_coding	NP_031835.3	88586	.	0.000000	[illegible]
13076	NM_001136059,NM_009992	Cyp1a1	cytochrome P450, family 1, subfamily a, polypeptide 1	protein_coding	NP_001129531.1;NP_034122.1	88588	.	0.000000	[illegible]
13078	NM_001364889,NM_009994	Cyp1b1	cytochrome P450, family 1, subfamily b, polypeptide 1	protein_coding	NP_001351818.1;NP_034124.1	88590	.	2.547890	[illegible]
13079	NM_009995	Cyp21a1	cytochrome P450, family 21, subfamily a, polypeptide 1	protein_coding	NP_034125.2	88591	.	0.000000	[illegible]
13081	NM_009996	Cyp24a1	cytochrome P450, family 24, subfamily a, polypeptide 1	protein_coding	NP_034126.1	88593	.	0.028406	[illegible]
13082	NM_007811,XM_017318053	Cyp26a1	cytochrome P450, family 26, subfamily a, polypeptide 1	protein_coding	NP_031837.2;XP_017173542.1	1096359	.	1.158756	[illegible]
13086	NM_009997	Cyp2a4	cytochrome P450, family 2, subfamily a, polypeptide 4	protein_coding	NP_034127.2	88596	.	0.000000	[illegible]
13090	NM_007814	Cyp2b19	cytochrome P450, family 2, subfamily b, polypeptide 19	protein_coding	NP_031840.1	107303	.	0.000000	[illegible]
13105	NM_010006	Cyp2d9	cytochrome P450, family 2, subfamily d, polypeptide 9	protein_coding	NP_034136.2	88606	.	0.000000	[illegible]
13106	NM_021282	Cyp2e1	cytochrome P450, family 2, subfamily e, polypeptide 1	protein_coding	NP_067257.1	88607	.	0.000000	[illegible]
13107	NM_007817	Cyp2f2	cytochrome P450, family 2, subfamily f, polypeptide 2	protein_coding	NP_031843.2	88608	.	0.194078	[illegible]
13110	NM_010008,XM_006502721,XM_006502722,XM_011240424,XM_030253163	Cyp2j6	cytochrome P450, family 2, subfamily j, polypeptide 6	protein_coding	NP_034138.3;XP_006502784.1;XP_006502785.1;XP_011238726.1;XP_030109023.1	1270148	.	2.681344	[illegible]
13113	NM_007819,XM_030254121,XR_003955579	Cyp3a13	cytochrome P450, family 3, subfamily a, polypeptide 13	protein_coding	NP_031845.1;XP_030109981.1	88610	.	0.335742	[illegible]
13115	NM_010009,XM_006513187	Cyp27b1	cytochrome P450, family 27, subfamily b, polypeptide 1	protein_coding	NP_034139.2;XP_006513250.1	1098274	.	0.371537	[illegible]
13116	NM_010010,XM_030246535,XR_381477	Cyp46a1	cytochrome P450, family 46, subfamily a, polypeptide 1	protein_coding	NP_034140.1;XP_030102395.1	1341877	.	3.352136	[illegible]
13118	NM_172306	Cyp4a12b	cytochrome P450, family 4, subfamily a, polypeptide 12B	protein_coding	NP_758510.2	3611747	.	0.000000	[illegible]
13120	NM_007823	Cyp4b1	cytochrome P450, family 4, subfamily b, polypeptide 1	protein_coding	NP_031849.1	103225	.	0.706688	[illegible]
13121	NM_020010	Cyp51	cytochrome P450, family 51	protein_coding	NP_064394.2	106040	.	10.112535	[illegible]
13123	NM_007825,XM_006535383,XM_006535384	Cyp7b1	cytochrome P450, family 7, subfamily b, polypeptide 1	protein_coding	NP_031851.3;XP_006535446.1;XP_006535447.1	104978	.	1.283382	[illegible]
13124	NM_010012	Cyp8b1	cytochrome P450, family 8, subfamily b, polypeptide 1	protein_coding	NP_034142.3	1338044	.	0.024296	[illegible]
13131	NM_001369046,NM_001369047,NM_001369048,NM_001369049,NM_010014,NM_177259,NR_104385,XM_006502725,XM_006502726,XM_006502727,XM_006502735,XM_011240426,XM_017319948,XM_017319949,XM_017319950,XM_030253165,XM_030253166,XM_030253167,XM_030253169,XM_030253170,XM_030253171,XM_030253172,XM_030253173,XM_030253174,XM_030253175,XM_030253176,XM_030253177,XM_030253178,XM_030253179,XM_030253180,XM_030253182	Dab1	disabled 1	protein_coding	NP_001355975.1;NP_001355976.1;NP_001355977.1;NP_001355978.1;NP_034144.1;NP_796233.2;XP_006502788.1;XP_006502789.1;XP_006502790.1;XP_006502798.1;XP_011238728.1;XP_017175437.1;XP_017175438.1;XP_017175439.1;XP_030109025.1;XP_030109026.1;XP_030109027.1;XP_030109029.1;XP_030109030.1;XP_030109031.1;XP_030109032.1;XP_030109033.1;XP_030109034.1;XP_030109035.1;XP_030109036.1;XP_030109037.1;XP_030109038.1;XP_030109039.1;XP_030109040.1;XP_030109042.1	108554	.	0.184305	[illegible]
13132	NM_001008702,NM_001037905,NM_001102400,NM_001310446,NM_023118,XM_011245324,XM_017316424,XM_030248312,XM_030248313	Dab2	disabled 2, mitogen-responsive phosphoprotein	protein_coding	NP_001008702.1;NP_001032994.1;NP_001095870.1;NP_001297375.1;NP_075607.2;XP_011243626.1;XP_017171913.1;XP_030104172.1;XP_030104173.1	109175	.	60.754548	[illegible]
13134	NM_001038610,NM_007826	Dach1	dachshund family transcription factor 1	protein_coding	NP_001033699.1;NP_031852.1	1277991	.	0.291629	[illegible]
13135	NM_001113358,NM_010015	Dad1	defender against cell death 1	protein_coding	NP_001106829.1;NP_034145.1	101912	.	104.184941	[illegible]
13136	NM_010016,XM_006529115,XM_006529117,XM_030246237,XR_003949488,XR_003949492	Cd55	CD55 molecule, decay accelerating factor for complement	protein_coding	NP_034146.2;XP_006529178.1;XP_006529180.1;XP_030102097.1	104850	.	2.450000	[illegible]
13137	NM_001317361,NM_007827,XM_011247917,XM_017314396,XM_030246278,XM_030246295	Cd55b	CD55 molecule, decay accelerating factor for complement B	protein_coding	NP_001304290.1;NP_031853.2;XP_011246219.1;XP_017169885.1;XP_030102138.1;XP_030102155.1	104849	.	0.000000	[illegible]
13138	NM_001276481,NM_001276482,NM_001276485,NM_001276486,NM_001276492,NM_001276493,NM_010017,XM_006511636,XM_030244040,XM_030244041	Dag1	dystroglycan 1	protein_coding	NP_001263410.1;NP_001263411.1;NP_001263414.1;NP_001263415.1;NP_001263421.1;NP_001263422.1;NP_034147.1;XP_006511699.1;XP_030099900.1;XP_030099901.1	101864	.	38.331170	[illegible]
13139	NM_001358745,NM_001358746,NM_016811,XM_011243351,XM_011243359,XM_011243363,XM_017313786,XM_017313790,XM_030244862,XM_030244863,XM_030244864,XM_030244865,XR_001779449,XR_001779451,XR_003948587,XR_380362	Dgka	diacylglycerol kinase, alpha	protein_coding	NP_001345674.1;NP_001345675.1;NP_058091.2;XP_011241653.1;XP_011241661.1;XP_011241665.1;XP_017169275.1;XP_017169279.1;XP_030100722.1;XP_030100723.1;XP_030100724.1;XP_030100725.1	102952	.	8.144063	[illegible]
13143	NM_010019,XM_006510811	Dapk2	death-associated protein kinase 2	protein_coding	NP_034149.2;XP_006510874.1	1341297	.	29.402530	[illegible]
13144	NM_001190473,NM_001190474,NM_007828,XM_006513194,XM_006513195,XM_030244866	Dapk3	death-associated protein kinase 3	protein_coding	NP_001177402.1;NP_001177403.2;NP_031854.1;XP_006513257.1;XP_006513258.1;XP_030100726.1	1203520	.	12.025018	[illegible]
13162	NM_010020,XM_006517083,XM_006517084,XM_017315377	Slc6a3	solute carrier family 6 (neurotransmitter transporter, dopamine), member	protein_coding	NP_034150.1;XP_006517146.1;XP_006517147.1;XP_017170866.1	94862	.	0.036221	[illegible]
13163	NM_001199733,NM_001355704,NM_007829,NR_149750,XM_006523581,XM_006523582	Daxx	Fas death domain-associated protein	protein_coding	NP_001186662.1;NP_001342633.1;NP_031855.3;XP_006523644.1;XP_006523645.1	1197015	.	8.898803	[illegible]
13164	NM_001277863,NM_010021,XM_006523583	Dazl	deleted in azoospermia-like	protein_coding	NP_001264792.1;NP_034151.3;XP_006523646.1	1342328	.	0.036200	[illegible]
13166	NM_138942	Dbh	dopamine beta hydroxylase	protein_coding	NP_620392.2	94864	.	0.000000	[illegible]
13167	NM_001037999,NM_007830	Dbi	diazepam binding inhibitor	protein_coding	NP_001033088.1;NP_031856.1	94865	.	63.998993	[illegible]
13168	NM_021294	Dbil5	diazepam binding inhibitor-like 5	protein_coding	NP_067269.1	108039	.	0.000000	[illegible]
13169	NM_001146308,NM_001146309,NM_013810	Dbnl	drebrin-like	protein_coding	NP_001139780.1;NP_001139781.1;NP_038838.1	700006	.	19.079063	[illegible]
13170	NM_016974,XM_006540596,XM_006540597	Dbp	D site albumin promoter binding protein	protein_coding	NP_058670.2;XP_006540659.1;XP_006540660.1	94866	.	3.428313	[illegible]
13171	NM_001357344,NM_010022,XM_006500977	Dbt	dihydrolipoamide branched chain transacylase E2	protein_coding	NP_001344273.1;NP_034152.2;XP_006501040.1	105386	.	3.858771	[illegible]
13172	NM_001005232	Dbx1	developing brain homeobox 1	protein_coding	NP_001005232.1	94867	.	0.000000	[illegible]
13175	NM_001111051,NM_001111052,NM_001111053,NM_001195538,NM_001195539,NM_001195540,NM_001357466,NM_001357468,NM_001357469,NM_001357475,NM_001357476,NM_019978,XM_006500981,XM_006500982,XM_006500983,XM_011240007,XM_017319447,XM_030252411,XM_030252412,XR_003954278,XR_00395427	Dclk1	doublecortin-like kinase 1	protein_coding	NP_001104521.1;NP_001104522.1;NP_001104523.1;NP_001182467.1;NP_001182468.1;NP_001182469.1;NP_001344395.1;NP_001344397.1;NP_001344398.1;NP_001344404.1;NP_001344405.1;NP_064362.1;XP_006501044.1;XP_006501045.1;XP_006501046.1;XP_011238309.1;XP_017174936.1;XP_030108271.1;XP_030108272.1	1330861	.	12.617719	[illegible]
13176	NM_007831,XM_006525588	Dcc	deleted in colorectal carcinoma	protein_coding	NP_031857.2;XP_006525651.1	94869	.	0.173409	[illegible]
13177	NM_010023	Eci1	enoyl-Coenzyme A delta isomerase 1	protein_coding	NP_034153.2	94871	.	12.039949	[illegible]
13178	NM_007832	Dck	deoxycytidine kinase	protein_coding	NP_031858.1	102726	.	5.580391	[illegible]
13179	NM_001190451,NM_007833	Dcn	decorin	protein_coding	NP_001177380.1;NP_031859.1	94872	.	295.797275	[illegible]
13180	NM_025273	Pcbd1	pterin 4 alpha carbinolamine dehydratase/dimerization cofactor of hepatocyte nuclear factor 1 alpha (TCF1) 1	protein_coding	NP_079549.1	94873	.	1.803585	[illegible]
13185	NM_007834	Vps26c	VPS26 endosomal protein sorting factor C	protein_coding	NP_031860.1	1206040	.	11.991929	[illegible]
13190	NM_010024,XM_006518510	Dct	dopachrome tautomerase	protein_coding	NP_034154.2;XP_006518573.1	102563	.	0.000000	[illegible]
13191	NM_001198866,NM_001198867,NM_001347310,NM_007835,XM_006505485,XM_006505486,XM_006505487,XM_006505488,XM_006505489,XM_006505490,XM_006505491,XM_006505492,XM_006505493,XM_006505494,XM_006505495,XM_006505496,XM_017321379,XM_030255135,XM_030255136,XM_030255137	Dctn1	dynactin 1	protein_coding	NP_001185795.1;NP_001185796.1;NP_001334239.1;NP_031861.2;XP_006505548.1;XP_006505549.1;XP_006505550.1;XP_006505551.1;XP_006505552.1;XP_006505553.1;XP_006505554.1;XP_006505555.1;XP_006505556.1;XP_006505557.1;XP_006505558.1;XP_006505559.1;XP_017176868.1;XP_030110995.1;XP_030110996.1;XP_030110	107745	.	25.916732	[illegible]
13193	NM_001110222,NM_001110223,NM_001110224,NM_010025,XM_006528698,XM_030251212,XM_030251213,XM_030251214	Dcx	doublecortin	protein_coding	NP_001103692.1;NP_001103693.1;NP_001103694.1;NP_034155.2;XP_006528761.1;XP_030107072.1;XP_030107073.1;XP_030107074.1	1277171	.	0.229982	[illegible]
13194	NM_015735,XM_006526639	Ddb1	damage specific DNA binding protein 1	protein_coding	NP_056550.1;XP_006526702.1	1202384	.	127.501090	[illegible]
13195	NM_001190448,NM_016672,XM_006514486,XM_006514487,XM_006514488,XM_006514489	Ddc	dopa decarboxylase	protein_coding	NP_001177377.1;NP_057881.1;XP_006514549.1;XP_006514550.1;XP_006514551.1;XP_006514552.1	94876	.	1.671844	[illegible]
13196	NM_001276461,NM_001276462,NM_001276463,NM_001276467,NM_010026,XM_006520392,XM_006520393,XM_006520394,XM_006520395,XM_006520396,XM_006520397,XM_006520398,XM_006520399,XM_006520400,XM_006520401,XM_006520402,XM_006520403,XM_011245448,XM_030248315,XM_030248316,XM_030248317	Asap1	ArfGAP with SH3 domain, ankyrin repeat and PH domain1	protein_coding	NP_001263390.1;NP_001263391.1;NP_001263392.1;NP_001263396.1;NP_034156.2;XP_006520455.1;XP_006520456.1;XP_006520457.1;XP_006520458.1;XP_006520459.1;XP_006520460.1;XP_006520461.1;XP_006520462.1;XP_006520463.1;XP_006520464.1;XP_006520465.1;XP_006520466.1;XP_011243750.1;XP_030104175.1;XP_030104176.1;XP_030104177.1	1342335	.	14.770640	[illegible]
13197	NM_007836	Gadd45a	growth arrest and DNA-damage-inducible 45 alpha	protein_coding	NP_031862.1	107799	.	3.572570	[illegible]
13198	NM_001290183,NM_007837,XM_006513197,XM_030244867	Ddit3	DNA-damage inducible transcript 3	protein_coding	NP_001277112.1;NP_031863.3;XP_006513260.1;XP_030100727.1	109247	.	9.370381	[illegible]
13199	NM_001013741	Ddn	dendrin	protein_coding	NP_001013763.1	108101	.	0.111278	[illegible]
13200	NM_007838	Ddost	dolichyl-di-phosphooligosaccharide-protein glycotransferase	protein_coding	NP_031864.2	1194508	.	128.425217	[illegible]
13202	NM_010027	Ddt	D-dopachrome tautomerase	protein_coding	NP_034157.1	1298381	.	13.228768	[illegible]
13204	NM_001042620,NM_007839,NR_104311	Dhx15	DEAH (Asp-Glu-Ala-His) box polypeptide 15	protein_coding	NP_001036085.1;NP_031865.2	1099786	.	47.028433	[illegible]
13205	NM_010028,XM_006527566	Ddx3x	DEAD/H (Asp-Glu-Ala-Asp/His) box polypeptide 3, X-linked	protein_coding	NP_034158.1;XP_006527629.1	103064	.	104.814096	[illegible]
13206	NM_001145885,NM_010029,XM_006517524,XM_011244618,XM_011244619,XM_011244621,XM_011244622	Ddx4	DEAD (Asp-Glu-Ala-Asp) box polypeptide 4	protein_coding	NP_001139357.1;NP_034159.1;XP_006517587.1;XP_011242920.1;XP_011242921.1;XP_011242923.1;XP_011242924.1	102670	.	0.316060	[illegible]
13207	NM_001355676,NM_001355677,NM_007840	Ddx5	DEAD (Asp-Glu-Ala-Asp) box polypeptide 5	protein_coding	NP_001342605.1;NP_001342606.1;NP_031866.2	105037	.	235.952110	[illegible]
13209	NM_001110826,NM_001357702,NM_001357703,NM_007841,NM_181324,XM_011242391,XM_030244042	Ddx6	DEAD (Asp-Glu-Ala-Asp) box polypeptide 6	protein_coding	NP_001104296.1;NP_001344631.1;NP_001344632.1;NP_031867.1;NP_851841.2;XP_011240693.1;XP_030099902.1	104976	.	43.584373	[illegible]
13211	NM_007842,XM_011247918,XM_030246403	Dhx9	DEAH (Asp-Glu-Ala-His) box polypeptide 9	protein_coding	NP_031868.2;XP_011246220.1;XP_030102263.1	108177	.	39.425203	[illegible]
13244	NM_001360783,NM_007853,XM_030246432	Degs1	delta(4)-desaturase, sphingolipid 1	protein_coding	NP_001347712.1;NP_031879.1;XP_030102292.1	1097711	.	64.501739	[illegible]
13340	NM_007854,XM_030250738,XM_030250739,XR_003952750	Slc29a2	solute carrier family 29 (nucleoside transporters), member 2	protein_coding	NP_031880.2;XP_030106598.1;XP_030106599.1	1345278	.	0.622111	[illegible]
13345	NM_007855	Twist2	twist basic helix-loop-helix transcription factor 2	protein_coding	NP_031881.1	104685	.	2.150945	[illegible]
13346	NM_010043	Des	desmin	protein_coding	NP_034173.1	94885	.	40.112560	[illegible]
13347	NM_001025296,NM_010044,NR_104263	Dffa	DNA fragmentation factor, alpha subunit	protein_coding	NP_001020467.1;NP_034174.2	1196227	.	7.385806	[illegible]
13349	NM_010045	Ackr1	atypical chemokine receptor 1 (Duffy blood group)	protein_coding	NP_034175.2	1097689	.	0.211960	[illegible]
13350	NM_010046,XM_006520405,XM_017316426,XR_383978	Dgat1	diacylglycerol O-acyltransferase 1	protein_coding	NP_034176.1;XP_006520468.1;XP_017171915.1	1333825	.	9.234165	[illegible]
13353	NM_001289813,NM_001289814,NM_010047,NR_110380,XM_030248957,XR_003951750	Dgcr6	DiGeorge syndrome critical region gene 6	protein_coding	NP_001276742.1;NP_001276743.1;NP_034177.1;XP_030104817.1	1202877	.	14.084303	[illegible]
13356	NM_001109750,NM_010048	Dgcr2	DiGeorge syndrome critical region gene 2	protein_coding	NP_001103220.1;NP_034178.2	892866	.	22.489090	[illegible]
13358	NM_153150	Slc25a1	solute carrier family 25 (mitochondrial carrier, citrate transporter),	protein_coding	NP_694790.1	1345283	.	13.398589	[illegible]
13360	NM_007856,XM_006508476,XM_006508478,XM_006508479,XM_006508480,XM_006508481,XM_011241979,XM_030242075,XM_030242076	Dhcr7	7-dehydrocholesterol reductase	protein_coding	NP_031882.1;XP_006508539.2;XP_006508541.1;XP_006508542.1;XP_006508543.1;XP_006508544.1;XP_011240281.1;XP_030097935.1;XP_030097936.1	1298378	.	6.548817	[illegible]
13361	NM_010049	Dhfr	dihydrofolate reductase	protein_coding	NP_034179.1	94890	.	2.683989	[illegible]
13363	NM_007857,XM_006520406,XM_017316427,XR_875235	Dhh	desert hedgehog	protein_coding	NP_031883.1;XP_006520469.1;XP_017171916.1	94891	.	0.243174	[illegible]
13367	NM_001305980,NM_001305981,NM_007858,XM_030250316	Diaph1	diaphanous related formin 1	protein_coding	NP_001292909.1;NP_001292910.1;NP_031884.1;XP_030106176.1	1194490	.	7.595282	[illegible]
13368	NM_007859,XM_006538517,XM_011250183,XM_011250184,XM_030253183	Dffb	DNA fragmentation factor, beta subunit	protein_coding	NP_031885.3;XP_006538580.1;XP_011248485.1;XP_011248486.1;XP_030109043.1	1196287	.	5.444613	[illegible]
13371	NM_010050	Dio2	deiodinase, iodothyronine, type II	protein_coding	NP_034180.2	1338833	.	1.966012	[illegible]
13380	NM_010051	Dkk1	dickkopf WNT signaling pathway inhibitor 1	protein_coding	NP_034181.2	1329040	.	24.414734	[illegible]
13382	NM_007861	Dld	dihydrolipoamide dehydrogenase	protein_coding	NP_031887.2	107450	.	36.557613	[illegible]
13383	NM_001252433,NM_001252434,NM_001252435,NM_001252436,NM_001357281,NM_001357282,NM_007862,NR_045516,XM_006521761,XM_006521762,XM_006521763,XM_006521764,XM_006521765,XM_006521766,XM_006521767,XM_006521768,XM_006521769,XM_006521770,XM_006521771,XM_006521772,XM_006521773,XM_017316865,XM_017316867,XM_017316869,XM_017316872,XM_030248959,XM_030248960,XM_030248961,XM_030248962,XR_001781789,XR_003951751,XR_003951752	Dlg1	discs large MAGUK scaffold protein 1	protein_coding	NP_001239362.1;NP_001239363.1;NP_001239364.1;NP_001239365.1;NP_001344210.1;NP_001344211.1;NP_031888.2;XP_006521824.1;XP_006521825.1;XP_006521826.1;XP_006521827.1;XP_006521828.1;XP_006521829.1;XP_006521830.1;XP_006521831.1;XP_006521832.1;XP_006521833.1;XP_006521834.1;XP_006521835.1;XP_006521836.1;XP_017172354.1;XP_017172356.1;XP_017172358.1;XP_017172361.1;XP_030104819.1;XP_030104820.1;XP_030104821.1;XP_030104822.1	107231	.	19.233587	[illegible]
13384	NM_007863,XM_006532138,XM_006532140,XM_030245518	Mpp3	membrane protein, palmitoylated 3 (MAGUK p55 subfamily member 3)	protein_coding	NP_031889.2;XP_006532201.1;XP_006532203.1;XP_030101378.1	1328354	.	1.065955	[illegible]
13385	NM_001109752,NM_001370671,NM_001370672,NM_001370674,NM_001370675,NM_007864,XM_006532144,XM_006532147,XM_006532148,XM_006532150,XM_017314248,XR_001779872	Dlg4	discs large MAGUK scaffold protein 4	protein_coding	NP_001103222.1;NP_001357600.1;NP_001357601.1;NP_001357603.1;NP_001357604.1;NP_031890.1;XP_006532207.1;XP_006532210.3;XP_006532211.1;XP_006532213.1;XP_017169737.1	1277959	.	11.665470	[illegible]
13386	NM_001190703,NM_001190704,NM_001190705,NM_010052,NR_033813,XM_006515457,XM_011243992,XM_030246536	Dlk1	delta like non-canonical Notch ligand 1	protein_coding	NP_001177632.1;NP_001177633.1;NP_001177634.1;NP_034182.2;XP_006515520.1;XP_011242294.1;XP_030102396.1	94900	.	93.813560	[illegible]
13388	NM_007865,XM_011246267,XM_030249466	Dll1	delta like canonical Notch ligand 1	protein_coding	NP_031891.2;XP_011244569.1;XP_030105326.1	104659	.	1.419599	[illegible]
13389	NM_007866	Dll3	delta like canonical Notch ligand 3	protein_coding	NP_031892.2	1096877	.	0.054868	[illegible]
13390	NM_010053,XM_006498672,XM_017315305,XM_030247131,XM_030247132	Dlx1	distal-less homeobox 1	protein_coding	NP_034183.1;XP_006498735.1;XP_017170794.1;XP_030102991.1;XP_030102992.1	94901	.	0.037814	[illegible]
13392	NM_010054	Dlx2	distal-less homeobox 2	protein_coding	NP_034184.1	94902	.	0.037368	[illegible]
13393	NM_010055	Dlx3	distal-less homeobox 3	protein_coding	NP_034185.1	94903	.	12.850066	[illegible]
13394	NM_007867,XM_030245519	Dlx4	distal-less homeobox 4	protein_coding	NP_031893.3;XP_030101379.1	94904	.	0.282790	[illegible]
13395	NM_010056,NM_198854,XM_006504980,XM_006504981	Dlx5	distal-less homeobox 5	protein_coding	NP_034186.2;NP_942151.1;XP_006505043.1;XP_006505044.1	101926	.	46.343860	[illegible]
13396	NM_010057	Dlx6	distal-less homeobox 6	protein_coding	NP_034187.1	101927	.	15.202279	[illegible]
13397	NR_002839	Dlx6os2	distal-less homeobox 6, opposite strand 2	lncRNA	.	1195963	.	0.001983	[illegible]
13400	NM_001190490,NM_001190491,NM_032418,NR_126095,XM_006539516,XM_006539517,XM_006539518,XM_006539519,XM_006539520,XM_006539521,XM_006539522,XM_011250433,XR_391041	Dmpk	dystrophia myotonica-protein kinase	protein_coding	NP_001177419.1;NP_001177420.1;NP_115794.1;XP_006539579.1;XP_006539580.1;XP_006539581.1;XP_006539582.1;XP_006539583.1;XP_006539584.1;XP_006539585.1;XP_011248735.1	94906	.	16.047949	[illegible]
13401	NM_010058,XM_006539524,XM_030242077	Dmwd	dystrophia myotonica-containing WD repeat motif	protein_coding	NP_034188.2;XP_006539587.1;XP_030097937.1	94907	.	20.035872	[illegible]
13404	NM_001278226,NM_010059,NR_103477,XM_006520407,XM_006520408,XM_006520409	Dmc1	DNA meiotic recombinase 1	protein_coding	NP_001265155.1;NP_034189.1;XP_006520470.1;XP_006520471.1;XP_006520472.1	105393	.	4.182384	[illegible]
13405	NM_001314034,NM_001314035,NM_001314036,NM_001314037,NM_001314038,NM_007868,XM_006527767,XM_006527768,XM_006527773,XM_017318373,XM_017318374,XM_017318375,XM_017318376,XM_017318377,XM_017318378,XM_017318379,XM_030251215	Dmd	dystrophin, muscular dystrophy	protein_coding	NP_001300963.1;NP_001300964.1;NP_001300965.1;NP_001300966.1;NP_001300967.1;NP_031894.1;XP_006527830.1;XP_006527831.1;XP_006527836.1;XP_017173862.1;XP_017173863.1;XP_017173864.1;XP_017173865.1;XP_017173866.1;XP_017173867.1;XP_017173868.1;XP_030107075.1	94909	.	3.540506	[illegible]
13406	NM_001359013,NM_016779	Dmp1	dentin matrix protein 1	protein_coding	NP_001345942.1;NP_058059.2	94910	.	235.147148	[illegible]
13409	NM_028953,XM_006526640	Tmc1	transmembrane channel-like gene family 1	protein_coding	NP_083229.1;XP_006526703.1	2151016	.	0.026587	[illegible]
13411	NM_010060,XM_006515463,XM_006515464,XM_006515466,XM_006515467,XM_006515468,XM_017314946,XM_017314947,XM_017314948,XM_017314949	Dnah11	dynein, axonemal, heavy chain 11	protein_coding	NP_034190.3;XP_006515526.1;XP_006515527.1;XP_006515529.1;XP_006515530.1;XP_006515531.1;XP_017170435.1;XP_017170436.1;XP_017170437.1;XP_017170438.1	1100864	.	0.016468	[illegible]
13417	NM_013811,XM_006523588,XM_006523591,XM_006523592,XM_030249467	Dnah8	dynein, axonemal, heavy chain 8	protein_coding	NP_038839.2;XP_006523651.1;XP_006523654.1;XP_006523655.1;XP_030105327.1	107714	.	0.301029	[illegible]
13418	NM_001190817,NM_007869,XM_011238947,XR_865923	Dnajc1	DnaJ heat shock protein family (Hsp40) member C1	protein_coding	NP_001177746.1;NP_031895.1;XP_011237249.1	103268	.	11.362100	[illegible]
13421	NM_007870,XM_006517921	Dnase1l3	deoxyribonuclease 1-like 3	protein_coding	NP_031896.3;XP_006517984.1	1314633	.	0.064525	[illegible]
13423	NM_001368295,NM_001368296,NM_010062	Dnase2a	deoxyribonuclease II alpha	protein_coding	NP_001355224.1;NP_001355225.1;NP_034192.1	1329019	.	6.356506	[illegible]
13424	NM_030238	Dync1h1	dynein cytoplasmic 1 heavy chain 1	protein_coding	NP_084514.2	103147	.	20.124298	[illegible]
13426	NM_001191023,NM_001191025,NM_001191026,NM_001191027,NM_010063,XM_006504982,XM_017321380,XM_030255138,XM_030255139	Dync1i1	dynein cytoplasmic 1 intermediate chain 1	protein_coding	NP_001177952.1;NP_001177954.1;NP_001177955.1;NP_001177956.1;NP_034193.2;XP_006505045.1;XP_017176869.1;XP_030110998.1;XP_030110999.1	107743	.	3.080158	[illegible]
13427	NM_001198872,NM_001198873,NM_001198874,NM_001198875,NM_001198876,NM_001198877,NM_001198878,NM_001347173,NM_010064,XM_006498676,XM_006498677,XM_006498678,XM_006498679,XM_006498680,XM_017315319,XM_017315324,XM_017315326,XM_030247151,XM_030247156,XM_030247159,XM_030247161,XM_030247162	Dync1i2	dynein cytoplasmic 1 intermediate chain 2	protein_coding	NP_001185801.1;NP_001185802.1;NP_001185803.1;NP_001185804.1;NP_001185805.1;NP_001185806.1;NP_001185807.1;NP_001334102.1;NP_034194.1;XP_006498739.1;XP_006498740.1;XP_006498741.1;XP_006498742.1;XP_006498743.1;XP_017170808.1;XP_017170813.1;XP_017170815.1;XP_030103011.1;XP_030103016.1;XP_030103019.1;XP_030103021.1;XP_030103022.1	107750	.	32.720612	[illegible]
13429	NM_001301737,NM_001368679,NM_010065,NR_125959,XM_006497649,XM_006497650,XM_006497652,XM_006497653,XM_006497654,XM_006497655,XM_006497658,XM_006497659,XM_006497660,XM_006497661,XM_006497662,XM_017315328,XR_001780675,XR_001780678,XR_003950433,XR_374055,XR_374057,XR_37405	Dnm1	dynamin 1	protein_coding	NP_001288666.1;NP_001355608.1;NP_034195.2;XP_006497712.2;XP_006497713.2;XP_006497715.2;XP_006497716.2;XP_006497717.2;XP_006497718.2;XP_006497721.2;XP_006497722.2;XP_006497723.2;XP_006497724.2;XP_006497725.1;XP_017170817.1	107384	.	66.945251	[illegible]
13430	NM_001039520,NM_001253893,NM_001253894,NM_001357718,NM_001357719,NM_001357721,NM_007871,XM_006509973,XM_006509974,XM_006509975,XM_006509976,XM_006509978,XM_006509979,XM_006509980,XM_006509981,XM_006509983,XM_006509984,XM_006509985,XM_017313125,XM_017313126,XM_017313127,XM_017313128,XM_030244043,XM_030244044,XM_030244045,XM_030244046,XM_030244047,XR_001778793,XR_003947776	Dnm2	dynamin 2	protein_coding	NP_001034609.1;NP_001240822.1;NP_001240823.1;NP_001344647.1;NP_001344648.1;NP_001344650.1;NP_031897.2;XP_006510036.1;XP_006510037.1;XP_006510038.1;XP_006510039.1;XP_006510041.1;XP_006510042.1;XP_006510043.1;XP_006510044.1;XP_006510046.1;XP_006510047.1;XP_006510048.1;XP_017168614.1;XP_017168615.1;XP_017168616.1;XP_017168617.1;XP_030099903.1;XP_030099904.1;XP_030099905.1;XP_030099906.1;XP_030099907.1	109547	.	23.438726	[illegible]
13433	NM_001199431,NM_001199432,NM_001199433,NM_001314011,NM_010066,XM_006509988,XM_011242393	Dnmt1	DNA methyltransferase (cytosine-5) 1	protein_coding	NP_001186360.2;NP_001186361.1;NP_001186362.1;NP_001300940.1;NP_034196.5;XP_006510051.1;XP_011240695.1	94912	.	17.263209	[illegible]
13434	NM_010067,XM_006497324,XM_006497325,XM_011238948,XM_030247190,XR_003950434,XR_003950435	Trdmt1	tRNA aspartic acid methyltransferase 1	protein_coding	NP_034197.3;XP_006497387.1;XP_006497388.1;XP_011237250.1;XP_030103050.1	1274787	.	2.612515	[illegible]
13435	NM_001271753,NM_007872,NM_153743,XM_006514953,XM_006514956,XM_030246538,XM_030246539,XM_030246540,XM_030246541,XM_030246542	Dnmt3a	DNA methyltransferase 3A	protein_coding	NP_001258682.1;NP_031898.1;NP_714965.1;XP_006515016.1;XP_006515019.1;XP_030102398.1;XP_030102399.1;XP_030102400.1;XP_030102401.1;XP_030102402.1	1261827	.	17.200424	[illegible]
13436	NM_001003960,NM_001003961,NM_001003963,NM_001122997,NM_001271744,NM_001271745,NM_001271746,NM_001271747,NM_010068,XM_006498682,XM_006498683,XM_006498684,XM_006498685,XM_006498686,XM_006498687,XM_006498688,XM_006498689,XM_030247208,XM_030247215,XM_030247217,XM_030247218,XR_374399	Dnmt3b	DNA methyltransferase 3B	protein_coding	NP_001003960.2;NP_001003961.2;NP_001003963.2;NP_001116469.1;NP_001258673.1;NP_001258674.1;NP_001258675.1;NP_001258676.1;NP_034198.3;XP_006498745.1;XP_006498746.1;XP_006498747.1;XP_006498748.1;XP_006498749.1;XP_006498750.1;XP_006498751.1;XP_006498752.1;XP_030103068.1;XP_030103075.1;XP_030103077.1;XP_030103078.1	1261819	.	2.012353	[illegible]
13437	NM_001110831,NM_016878,XM_006496405,XM_006496406	Dnpep	aspartyl aminopeptidase	protein_coding	NP_001104301.1;NP_058574.3;XP_006496468.1;XP_006496469.1	1278328	.	21.806839	[illegible]
13445	NM_013812	Cdk2ap1	CDK2 (cyclin-dependent kinase 2)-associated protein 1	protein_coding	NP_038840.2	1202069	.	52.051548	[illegible]
13446	NM_001368355,NM_001368356,NM_001368357,NM_001368358,NM_010069,XM_030242078,XM_030242079,XM_030242080,XM_030242081,XM_030242082,XM_030242083	Doc2a	double C2, alpha	protein_coding	NP_001355284.1;NP_001355285.1;NP_001355286.1;NP_001355287.1;NP_034199.1;XP_030097938.1;XP_030097939.1;XP_030097940.1;XP_030097941.1;XP_030097942.1;XP_030097943.1	109446	.	0.000003	[illegible]
13447	NM_007873	Doc2b	double C2, beta	protein_coding	NP_031899.2	1100497	.	0.138884	[illegible]
13448	NM_001291799,NM_010070,XM_006505497,XM_030255140	Dok1	docking protein 1	protein_coding	NP_001278728.1;NP_034200.4;XP_006505560.1;XP_030111000.1	893587	.	12.189735	[illegible]
13449	NM_010071,XM_011244943,XM_017315834,XM_030247633,XM_030247634,XM_030247635,XM_030247636,XM_030247637	Dok2	docking protein 2	protein_coding	NP_034201.1;XP_011243245.1;XP_017171323.1;XP_030103493.1;XP_030103494.1;XP_030103495.1;XP_030103496.1;XP_030103497.1	1332623	.	2.131347	[illegible]
13476	NM_007874,XR_003952491	Reep5	receptor accessory protein 5	protein_coding	NP_031900.3	1270152	.	45.210981	[illegible]
13478	NM_001364464,NM_007875,XM_006509991,XM_006509992,XM_006509995,XM_030244048	Dpagt1	dolichyl-phosphate (UDP-N-acetylglucosamine) acetylglucosaminephosphotransferase 1 (GlcNAc-1-P transferase)	protein_coding	NP_001351393.1;NP_031901.2;XP_006510054.1;XP_006510055.1;XP_006510058.1;XP_030099908.1	1196396	.	15.596730	[illegible]
13479	NM_007876,XM_017312558	Dpep1	dipeptidase 1	protein_coding	NP_031902.2;XP_017168047.1	94917	.	5.426389	[illegible]
13480	NM_001310084,NM_010072	Dpm1	dolichol-phosphate (beta-D) mannosyltransferase 1	protein_coding	NP_001297013.1;NP_034202.1	1330239	.	2.505942	[illegible]
13481	NM_010073	Dpm2	dolichol-phosphate (beta-D) mannosyltransferase 2	protein_coding	NP_034203.1	1330238	.	30.347315	[illegible]
13482	NM_001159543,NM_010074,XM_006498691,XM_006498692,XM_011239274,XR_001780739	Dpp4	dipeptidylpeptidase 4	protein_coding	NP_001153015.1;NP_034204.1;XP_006498754.1;XP_006498755.1;XP_011237576.1	94919	.	0.254711	[illegible]
13483	NM_001136060,NM_001198886,NM_010075,NM_207282	Dpp6	dipeptidylpeptidase 6	protein_coding	NP_001129532.1;NP_001185815.1;NP_034205.1;NP_997165.2	94921	.	0.274983	[illegible]
13486	NM_026106	Dr1	down-regulator of transcription 1	protein_coding	NP_080382.2	1100515	.	12.428194	[illegible]
13488	NM_001291801,NM_010076	Drd1	dopamine receptor D1	protein_coding	NP_001278730.1;NP_034206.1	99578	.	0.057157	[illegible]
13489	NM_010077,XM_006509996	Drd2	dopamine receptor D2	protein_coding	NP_034207.2;XP_006510059.1	94924	.	0.000000	[illegible]
13490	NM_007877,XM_006521777	Drd3	dopamine receptor D3	protein_coding	NP_031903.1;XP_006521840.1	94925	.	0.000000	[illegible]
13491	NM_007878,XM_006536156,XM_017321972	Drd4	dopamine receptor D4	protein_coding	NP_031904.1;XP_006536219.1;XP_017177461.1	94926	.	0.445219	[illegible]
13492	NM_013503	Drd5	dopamine receptor D5	protein_coding	NP_038531.1	94927	.	0.000000	[illegible]
13494	NM_007879,XM_017314249	Drg1	developmentally regulated GTP binding protein 1	protein_coding	NP_031905.1;XP_017169738.1	1343297	.	27.721122	[illegible]
13495	NM_021354,XM_006532153	Drg2	developmentally regulated GTP binding protein 2	protein_coding	NP_067329.1;XP_006532216.1	1342307	.	19.247249	[illegible]
13496	NM_001288625,NM_001288626,NM_007880,XM_006513201,XM_006513202	Arid3a	AT rich interactive domain 3A (BRIGHT-like)	protein_coding	NP_001275554.1;NP_001275555.1;NP_031906.1;XP_006513264.1;XP_006513265.1	1328360	.	2.582445	[illegible]

13497	NM_010078,XM_006528496,XM_011247730,XM_017318380,XM_030251216,XM_030251217,XM_030251218,XM_030251219,XM_030251220,XR_001782733,XR_003952997	Drp2	dystrophin related protein 2	protein_coding	[illegible]
13498	NM_007881	Atn1	atrophin 1	protein_coding	[illegible]
13506	NM_001317363,NM_001317365,NM_013505	Dsc2	desmocollin 2	protein_coding	[illegible]
13507	NM_001291809,NM_007882,XM_006525593,XM_006525596	Dsc3	desmocollin 3	protein_coding	[illegible]
13508	NM_031174,XM_006522886,XM_006522887,XM_030248963,XR_003951753,XR_003951754	Dscam	DS cell adhesion molecule	protein_coding	[illegible]
13510	NM_010079,XM_006525597	Dsg1a	desmoglein 1 alpha	protein_coding	[illegible]
13511	NM_007883	Dsg2	desmoglein 2	protein_coding	[illegible]
13516	NM_007884,XM_030244868	Epyc	epiphycan	protein_coding	[illegible]
13518	NM_001276764,NM_010081,NM_133833,NM_134448,XM_006495666,XM_006495669,XM_006495674,XM_006495678,XM_006495679,XM_006495680,XM_006495682,XM_006495684,XM_006495685,XM_006495686,XM_006495688,XM_006495690,XM_006495691,XM_017314621,XM_017314635,XM_017314651,XM_017314665,XM_017314676,XM_017314687,XM_017314689,XM_017314706,XM_017314712,XM_017314716,XM_017314722,XM_017314741,XM_017314750,XM_017314794,XM_017314811,XM_017314848,XM_017314868,XM_030246500,XM_030246701	Dst	dystonin	protein_coding	[illegible]
13521	NM_007885	Slc26a2	solute carrier family 26 (sulfate transporter), member 2	protein_coding	[illegible]
13522	NM_001048175,NM_010082,NM_183366,NR_102399,XM_006518514,XM_017315836,XR_001781095	Adam28	a disintegrin and metallopeptidase domain 28	protein_coding	[illegible]
13524	NM_010084,NR_136908	Adam18	a disintegrin and metallopeptidase domain 18	protein_coding	[illegible]
13527	NM_001285807,NM_001285808,NM_001285810,NM_001285811,NM_001285813,NM_001285817,NM_001361215,NM_001361216,NM_001361217,NM_010087,NM_207650,XM_011246827,XM_011246830,XM_011246833,XM_011246835,XM_011246838,XM_017317811,XM_017317817,XM_030250317,XM_030250318,XM_030250319,XM_030250320,XM_030250321,XM_030250322,XM_030250323,XM_030250324,XM_030250325,XR_001782334,XR_001782335,XR_001782336,XR_001782337,XR_001782338,XR_001782339,XR_001782340,XR_001782341,XR_001782342,XR_003952492,XR_003952493,XR_003952494,XR_003952495,XR_003952496,XR_003952497,XR_003952498,XR_003952499,XR_003952500,XR_003952501	Dtna	dystrobrevin alpha	protein_coding	[illegible]
13528	NM_001162465,NM_001355528,NM_007886,NR_027869,NR_027870,NR_149729,XM_006514958,XM_006514959,XM_006514960,XM_006514961,XM_011243813,XM_011243814,XM_017314951,XM_017314952,XM_017314953,XM_017314954,XM_017314955,XM_030246544,XM_030246545,XM_030246547,XM_030246548,XM_030246549,XM_030246550,XM_030246551,XM_030246553	Dtnb	dystrobrevin, beta	protein_coding	[illegible]
13537	NM_010090	Dusp2	dual specificity phosphatase 2	protein_coding	[illegible]
13542	NM_001356381,NM_010091,XM_006538518,XM_006538519,XM_006538520,XM_006538521,XM_006538522,XM_006538523	Dvl1	dishevelled segment polarity protein 1	protein_coding	[illegible]
13543	NM_007888	Dvl2	dishevelled segment polarity protein 2	protein_coding	[illegible]
13544	NM_001347176,NM_007889,XM_006521780,XR_003951755	Dvl3	dishevelled segment polarity protein 3	protein_coding	[illegible]
13548	NM_001113389,NM_001347731,NM_007890,XM_011246094,XM_011246095,XM_011246096,XM_011246098,XM_011246099	Dyrk1a	dual-specificity tyrosine-(Y)-phosphorylation regulated kinase 1a	protein_coding	[illegible]
13549	NM_001037957,NM_001271370,NM_010092,XM_006539526,XM_006539527,XM_030242084,XR_003946383,XR_003946384,XR_003946385	Dyrk1b	dual-specificity tyrosine-(Y)-phosphorylation regulated kinase 1b	protein_coding	[illegible]
13555	NM_001291105,NM_007891	E2f1	E2F transcription factor 1	protein_coding	[illegible]
13557	NM_001289920,NM_001359994,NM_010093,XM_011244275,XM_011244276,XM_030247136	E2f3	E2F transcription factor 3	protein_coding	[illegible]
13559	NM_007892,XM_006530051,XR_001783649	E2f5	E2F transcription factor 5	protein_coding	[illegible]
13560	NM_001301784,NM_001355708,NM_007893,NR_125990,XM_011246268,XM_017317223,XM_017317224,XR_003952082,XR_003952083	E4f1	E4F transcription factor 1	protein_coding	[illegible]
13586	NM_007894	Ear1	eosinophil-associated, ribonuclease A family, member 1	protein_coding	[illegible]
13587	NM_007895	Ear2	eosinophil-associated, ribonuclease A family, member 2	protein_coding	[illegible]
13589	NM_007896	Mapre1	microtubule-associated protein, RP/EB family, member 1	protein_coding	[illegible]
13590	NM_010094	Lefty1	left right determination factor 1	protein_coding	[illegible]
13591	NM_001290709,NM_001290710,NM_001290711,NM_001362569,NM_001362571,NM_001362574,NM_001362575,NM_001362576,NM_001362577,NM_001362578,NM_001362579,NM_007897,XM_017314250,XM_030245520,XM_030245521,XM_030245522	Ebf1	early B cell factor 1	protein_coding	[illegible]
13592	NM_001276387,NM_001360202,NM_010095	Ebf2	early B cell factor 2	protein_coding	[illegible]
13593	NM_001113414,NM_001113415,NM_010096,XM_006507331,XM_006507332,XM_006507333,XM_006507334,XM_006507335,XM_006507336,XM_006507337,XM_011241672,XM_030242085,XM_030242086,XM_030242087,XM_030242088,XM_030242089,XM_030242090,XM_030242091,XM_030242092,XM_030242093,XM_030242094,XM_030242095	Ebf3	early B cell factor 3	protein_coding	[illegible]
13595	NM_007898	Ebp	phenylalkylamine Ca2+ antagonist (emopamil) binding protein	protein_coding	[illegible]
13599	NM_001277925,NM_021306,XM_017314898,XM_017314902,XM_017314903,XR_001780275,XR_001780293	Ecel1	endothelin converting enzyme-like 1	protein_coding	[illegible]
13601	NM_001252653,NM_001355070,NM_007899,XM_006500984,XM_011240008,XM_011240009,XM_017319449,XM_017319450,XM_030252413	Ecm1	extracellular matrix protein 1	protein_coding	[illegible]
13602	NM_001359014,NM_001359016,NM_010097	Sparcl1	SPARC-like 1	protein_coding	[illegible]
13603	NM_010098,XM_006496642	Opn3	opsin 3	protein_coding	[illegible]
13605	NM_001177625,NM_001177626,NM_007900,XM_006535385,XM_006535386	Ect2	ect2 oncogene	protein_coding	[illegible]
13607	NM_001177937,NM_001177938,NM_001177939,NM_001177940,NM_001177941,NM_001177942,NM_001177943,NM_001177944,NM_010099	Eda	ectodysplasin-A	protein_coding	[illegible]
13608	NM_010100,XM_006513204,XM_006513205,XM_006513206,XM_030244869	Edar	ectodysplasin-A receptor	protein_coding	[illegible]
13609	NM_007901	S1pr1	sphingosine-1-phosphate receptor 1	protein_coding	[illegible]
13610	NM_010101	S1pr3	sphingosine-1-phosphate receptor 3	protein_coding	[illegible]
13611	NM_010102	S1pr4	sphingosine-1-phosphate receptor 4	protein_coding	[illegible]
13612	NM_001037987,NM_010103	Edil3	EGF-like repeats and discoidin I-like domains 3	protein_coding	[illegible]
13614	NM_010104	Edn1	endothelin 1	protein_coding	[illegible]
13615	NM_007902	Edn2	endothelin 2	protein_coding	[illegible]
13616	NM_007903	Edn3	endothelin 3	protein_coding	[illegible]
13617	NM_010332	Ednra	endothelin receptor type A	protein_coding	[illegible]
13618	NM_001136061,NM_001276296,NM_007904,XM_006518515	Ednrb	endothelin receptor type B	protein_coding	[illegible]
13619	NM_001042623,NM_001271579,NM_001355215,NM_007905,NR_073364,XM_006505498,XM_006505499,XM_006505500,XM_017321382,XM_030255141,XM_030255142	Phc1	polyhomeotic 1	protein_coding	[illegible]
13626	NM_021876	Eed	embryonic ectoderm development	protein_coding	[illegible]
13627	NM_010106	Eef1a1	eukaryotic translation elongation factor 1 alpha 1	protein_coding	[illegible]
13628	NM_007906	Eef1a2	eukaryotic translation elongation factor 1 alpha 2	protein_coding	[illegible]
13629	NM_007907	Eef2	eukaryotic translation elongation factor 2	protein_coding	[illegible]
13631	NM_001267710,NM_001267711,NM_007908,XM_006507342,XM_006507343,XM_006507344,XM_006507345,XM_006507346,XR_003946386	Eef2k	eukaryotic elongation factor-2 kinase	protein_coding	[illegible]
13636	NM_001162425,NM_010107	Efna1	ephrin A1	protein_coding	[illegible]
13637	NM_007909	Efna2	ephrin A2	protein_coding	[illegible]
13638	NM_010108,XM_006500988,XM_017319452,XM_030252414,XM_030252415	Efna3	ephrin A3	protein_coding	[illegible]
13639	NM_007910	Efna4	ephrin A4	protein_coding	[illegible]
13640	NM_010109,NM_207654,XM_006523598,XM_030249468,XM_030249469	Efna5	ephrin A5	protein_coding	[illegible]
13641	NM_010110	Efnb1	ephrin B1	protein_coding	[illegible]
13642	NM_001368299,NM_010111,XM_011242036	Efnb2	ephrin B2	protein_coding	[illegible]
13643	NM_007911,XM_006532160	Efnb3	ephrin B3	protein_coding	[illegible]
13644	NM_010112	Efs	embryonal Fyn-associated substrate	protein_coding	[illegible]
13645	NM_001310737,NM_001329594,NM_010113,XM_006500990,XM_011240010,XM_011240011,XR_001783650	Egf	epidermal growth factor	protein_coding	[illegible]
13649	NM_007912,NM_207655	Egfr	epidermal growth factor receptor	protein_coding	[illegible]
13650	NM_001291818,NM_010117,XM_006514494,XM_006514495,XM_006514496,XM_006514499,XM_006514501,XM_006514503,XM_011243658,XM_030245523,XM_030245524,XM_030245525,XM_030245526,XR_001779873,XR_003949287,XR_872202	Rhbdf1	rhomboid 5 homolog 1	protein_coding	[illegible]
13653	NM_007913	Egr1	early growth response 1	protein_coding	[illegible]
13654	NM_001347458,NM_010118,XM_006513208,XM_006513209,XM_006513210,XM_006513211,XM_006513213,XM_011243367,XM_011243368,XM_030244870	Egr2	early growth response 2	protein_coding	[illegible]
13655	NM_001289925,NM_001289927,NM_018781,XM_006518519	Egr3	early growth response 3	protein_coding	[illegible]
13656	NM_020596	Egr4	early growth response 4	protein_coding	[illegible]
13660	NM_010119	Ehd1	EH-domain containing 1	protein_coding	[illegible]
13661	NM_007914,XM_006498695,XM_006498697,XM_006498698,XM_006498699,XM_006498700,XM_006498701,XM_006498702,XM_006498703	Ehf	ets homologous factor	protein_coding	[illegible]
13663	NM_001199494,NM_007915	Ei24	etoposide induced 2.4 mRNA	protein_coding	[illegible]
13664	NM_010120,XM_006525626	Eif1a	eukaryotic translation initiation factor 1A	protein_coding	[illegible]
13665	NM_026114	Eif2s1	eukaryotic translation initiation factor 2, subunit 1 alpha	protein_coding	[illegible]
13666	NM_001313918,NM_010121,XM_006505501,XM_011241202	Eif2ak3	eukaryotic translation initiation factor 2 alpha kinase 3	protein_coding	[illegible]
13667	NM_001127355,NM_001127356,NM_010122	Eif2b4	eukaryotic translation initiation factor 2B, subunit 4 delta	protein_coding	[illegible]
13669	NM_010123	Eif3a	eukaryotic translation initiation factor 3, subunit A	protein_coding	[illegible]
13674	gene-Eif4a-ps4	Eif4a-ps4	eukaryotic translation initiation factor 4A, pseudogene 4	pseudogene	[illegible]
13680	NM_007916,XM_006530650	Ddx19a	DEAD (Asp-Glu-Ala-Asp) box polypeptide 19a	protein_coding	[illegible]
13681	NM_001159375,NM_144958	Eif4a1	eukaryotic translation initiation factor 4A1	protein_coding	[illegible]
13682	NM_001123037,NM_001123038,NM_001347179,NM_013506,NR_110335,NR_152586,XM_006521784,XM_006521785,XM_030248964,XM_030248965,XR_003951756,XR_003951757,XR_003951758,XR_003951759,XR_384524,XR_384525	Eif4a2	eukaryotic translation initiation factor 4A2	protein_coding	[illegible]
13684	NM_001313980,NM_007917,XM_006500993,XM_017319453	Eif4e	eukaryotic translation initiation factor 4E	protein_coding	[illegible]
13685	NM_007918	Eif4ebp1	eukaryotic translation initiation factor 4E binding protein 1	protein_coding	[illegible]
13688	NM_010124,XM_017313792,XM_017313793	Eif4ebp2	eukaryotic translation initiation factor 4E binding protein 2	protein_coding	[illegible]
13690	NM_001040131,NM_013507	Eif4g2	eukaryotic translation initiation factor 4, gamma 2	protein_coding	[illegible]
13706	NM_007919	Cela2a	chymotrypsin-like elastase family, member 2A	protein_coding	[illegible]
13709	NM_001286411,NM_001286412,NM_007920,XM_011244946,XM_011244947,XM_011244948,XM_030247638,XM_030247639,XM_030247640,XM_030247641,XM_030247642	Elf1	E74-like factor 1	protein_coding	[illegible]
13710	NM_001163131,NM_007921,XM_030247017,XM_030247020	Elf3	E74-like factor 3	protein_coding	[illegible]
13712	NM_007922	Elk1	ELK1, member of ETS oncogene family	protein_coding	[illegible]
13713	NM_001282967,NM_001322265,NM_001322268,NM_013508,XM_030244871,XM_030244872	Elk3	ELK3, member of ETS oncogene family	protein_coding	[illegible]
13714	NM_007923,XM_006529126,XM_006529127,XM_006529128,XM_006529129,XM_006529130,XM_006529131,XM_017315041	Elk4	ELK4, member of ETS oncogene family	protein_coding	[illegible]
13716	NM_007924	Ell	elongation factor RNA polymerase II	protein_coding	[illegible]
13717	NM_007925,XM_006504360,XM_011240855	Eln	elastin	protein_coding	[illegible]
13722	NM_001368626,NM_007926	Aimp1	aminoacyl tRNA synthetase complex-interacting multifunctional protein 1	protein_coding	[illegible]
13723	NM_010330	Emb	embigin	protein_coding	[illegible]
13726	NM_007927,XM_011247521	Emd	emerin	protein_coding	[illegible]
13728	NM_001080388,NM_001080389,NM_001080390,NM_007928,XM_006526641,XM_006526642,XM_006526643,XM_006526644,XM_006526645,XM_006526646,XM_006526647,XM_006526648,XM_006526650,XM_006526652,XM_006526654,XM_006526656,XM_017318058,XM_017318059,XM_030250740,XM_030250741,XM_030250742,XM_030250743,XM_030250744	Mark2	MAP/microtubule affinity regulating kinase 2	protein_coding	[illegible]
13730	NM_001288627,NM_001288628,NM_010128,XM_006505502	Emp1	epithelial membrane protein 1	protein_coding	[illegible]
13731	NM_007929	Emp2	epithelial membrane protein 2	protein_coding	[illegible]
13732	NM_001146346,NM_010129	Emp3	epithelial membrane protein 3	protein_coding	[illegible]
13733	NM_001355722,NM_001355723,NM_010130,XM_006523602	Adgre1	adhesion G protein-coupled receptor E1	protein_coding	[illegible]
13796	NM_010131	Emx1	empty spiracles homeobox 1	protein_coding	[illegible]
13797	NM_010132,XM_006526657	Emx2	empty spiracles homeobox 2	protein_coding	[illegible]
13798	NM_010133	En1	engrailed 1	protein_coding	[illegible]
13799	NM_010134	En2	engrailed 2	protein_coding	[illegible]
13800	NM_001083120,NM_001083121,NM_008680,NM_010135,XM_006496643,XM_006496644,XM_006496645,XM_006496646,XM_006496647,XM_006496648,XM_006496649,XM_006496650,XM_006496651,XM_006496652,XM_030247134,XM_030247143,XM_030247158,XM_030247170,XR_001780490	Enah	ENAH actin regulator	protein_coding	[illegible]
13803	NM_007930,XM_030247137,XM_030247138,XM_030247139	Enc1	ectodermal-neural cortex 1	protein_coding	[illegible]
13804	NM_007931,XR_374059	Endog	endonuclease G	protein_coding	[illegible]
13805	NM_001146348,NM_001146350,NM_007932	Eng	endoglin	protein_coding	[illegible]
13806	NM_023119,XM_006538525,XM_017319953	Eno1	enolase 1, alpha non-neuron	protein_coding	[illegible]
13807	NM_001302642,NM_001355220,NM_013509,XM_006505504,XM_030255143	Eno2	enolase 2, gamma neuronal	protein_coding	[illegible]
13808	NM_001136062,NM_001276285,NM_007933,XM_006532161,XM_006532162,XM_030245527	Eno3	enolase 3, beta muscle	protein_coding	[illegible]
13809	NM_007934	Enpep	glutamyl aminopeptidase	protein_coding	[illegible]
13813	NM_001164789,NM_010136,XM_006511940	Eomes	eomesodermin	protein_coding	[illegible]
13819	NM_010137,XM_030249471	Epas1	endothelial PAS domain protein 1	protein_coding	[illegible]
13821	NM_001006664,NM_001291120,NM_001291121,NM_001291122,NM_001291123,NM_001368800,NM_001368801,NM_001368802,NM_001368803,NM_001368804,NM_013510,XM_006498708,XM_006498711,XM_006498713,XM_006498714,XM_011239275,XM_011239276,XM_011239277,XM_011239278,XM_011239279,XM_011239280,XM_011239281,XM_030247261,XM_030247268,XM_030247271,XM_030247275,XM_030247277,XM_030247281,XM_030247282,XM_030247284,XM_030247286,XM_030247288,XM_030247290	Epb41l1	erythrocyte membrane protein band 4.1 like 1	protein_coding	[illegible]
13822	NM_001199265,NM_001358751,NM_001358753,NM_001358754,NM_001358755,NM_013511,XM_006512506,XM_006512507,XM_006512508,XM_006512509,XM_006512510,XM_006512511,XM_006512512,XM_006512513,XM_006512514,XM_006512515,XM_006512517,XM_030244873,XM_030244874,XM_030244875,XR_00177945	Epb41l2	erythrocyte membrane protein band 4.1 like 2	protein_coding	[illegible]
13823	NM_001355732,NM_001355733,NM_001355734,NM_001355735,NM_001355736,NM_013813,XM_006523611,XM_006523612,XM_006523613,XM_006523614,XM_006523615,XM_006523616,XM_006523618,XM_011246278,XM_011246279,XM_011246281,XM_011246283,XM_011246284,XM_011246285,XM_011246288,XM_011246290,XM_017317227,XM_017317231,XM_017317232,XM_017317234,XM_017317238,XM_017317239,XM_030249472,XM_030249473,XM_030249474,XM_030249475,XM_030249476,XM_030249477,XM_030249478,XM_030249479,XM_030249480,XM_030249481,XM_030249482,XM_030249483,XM_030249484,XM_030249485,XM_030249486,XM_030249487,XM_030249488,XM_030249489,XM_030249490,XM_030249491,XM_030249492,XM_030249494,XM_030249495	Epb41l3	erythrocyte membrane protein band 4.1 like 3	protein_coding	[illegible]
13824	NM_013512,XM_006525627,XM_006525628,XM_006525629,XM_006525631,XM_006525635,XM_011246840,XM_030250326,XM_030250327,XM_030250328,XM_030250329,XM_030250330	Epb41l4a	erythrocyte membrane protein band 4.1 like 4a	protein_coding	[illegible]
13828	NM_013513,XM_006498727	Epb42	erythrocyte membrane protein band 4.2	protein_coding	[illegible]
13829	NM_001252662,NM_001252663,NM_001252664,NM_001252665,NM_001252666,NM_001360024,NM_001360025,NM_001360026,NM_001360027,NM_001360028,NM_001360029,NM_001360030,NM_013514	Dmtn	dematin actin binding protein	protein_coding	[illegible]
13830	NM_013515	Stom	stomatin	protein_coding	[illegible]
13831	NM_001276350,NM_007935,NM_027497	Epc1	enhancer of polycomb homolog 1	protein_coding	[illegible]
13835	NM_023580,XM_011241203	Epha1	Eph receptor A1	protein_coding	[illegible]
13836	NM_010139,XM_006538528	Epha2	Eph receptor A2	protein_coding	[illegible]
13837	NM_001362452,NM_010140	Epha3	Eph receptor A3	protein_coding	[illegible]
13838	NM_007936,XM_006496407,XM_006496408	Epha4	Eph receptor A4	protein_coding	[illegible]
13839	NM_007937,XM_006534769,XM_006534770,XM_006534771,XM_006534773,XM_006534775,XM_030254123,XM_030254124,XM_030254125	Epha5	Eph receptor A5	protein_coding	[illegible]
13840	NM_007938,XM_006521787,XM_011245826	Epha6	Eph receptor A6	protein_coding	[illegible]
13841	NM_001122889,NM_001290434,NM_010141,XM_006537606,XM_006537607,XM_017319954,XM_017319955	Epha7	Eph receptor A7	protein_coding	[illegible]
13842	NM_007939,XM_030253187,XM_030253189,XR_003954873	Epha8	Eph receptor A8	protein_coding	[illegible]
13844	NM_001290753,NM_010142,XM_006538531,XM_006538533,XM_006538534,XM_030253190,XM_030253191	Ephb2	Eph receptor B2	protein_coding	[illegible]
13845	NM_010143,XM_006521788	Ephb3	Eph receptor B3	protein_coding	[illegible]
13846	NM_001159571,NM_010144	Ephb4	Eph receptor B4	protein_coding	[illegible]
13848	NM_001146351,NM_007680,XM_030255144,XR_003956161,XR_377419	Ephb6	Eph receptor B6	protein_coding	[illegible]
13849	NM_001312918,NM_010145	Ephx1	epoxide hydrolase 1, microsomal	protein_coding	[illegible]
13850	NM_001271402,NM_001271403,NM_001271421,NM_007940	Ephx2	epoxide hydrolase 2, cytoplasmic	protein_coding	[illegible]

13852	NM_001286033,NM_001286034,NM_001359022,NM_007941,NR_152615,XM_006504276,XR_003955580,XR_003955581,XR_377049	Stx2	syntaxin 2	protein_coding	[illegible]
13853	NM_010146,XR_003948588	Epm2a	epilepsy, progressive myoclonic epilepsy, type 2 gene alpha	protein_coding	[illegible]
13854	NM_001252454,NM_010147,XM_006539529,XM_006539531,XM_006539532,XM_006539533,XM_006539534,XM_006539535,XM_006539536,XM_006539537,XM_017321973,XM_017321974,XM_030242096,XM_030242097,XM_030242098,XM_030242099,XM_030242100,XM_030242102,XM_030242103,XM_030242104,XM_030242105,XM_030242106,XM_030242107,XM_030242108,XM_030242109,XM_030242110,XM_030242111,XM_030242112,XM_030242113,XM_030242114,XM_030242115,XM_030242116,XM_030242117,XM_030242118,XM_030242119,XM_030242121,XM_030242122,XM_030242123,XM_030242124,XM_030242125,XM_030242126,XM_030242127	Epn1	epsin 1	protein_coding	[illegible]
13855	NM_001252188,NM_001252189,NM_001362585,NM_001362586,NM_001362587,NM_010148,XM_006532165,XM_006532166,XM_006532167,XM_006532168,XM_006532169,XM_006532170,XM_006532171,XM_011248711,XM_011248712,XM_011248713,XM_030245528,XM_030245529,XM_030245530,XM_030245531,XM_030245532,XM_030245533,XM_030245534,XM_030245535,XM_030245536,XM_030245537,XM_030245538,XM_030245539,XM_030245540,XM_030245541	Epn2	epsin 2	protein_coding	[illegible]
13857	NM_010149,XM_006509998,XM_017313130,XR_001778795	Epor	erythropoietin receptor	protein_coding	[illegible]
13858	NM_001159964,NM_007943,XM_006502738,XM_006502739,XM_006502740,XM_017319956,XM_030253192	Eps15	epidermal growth factor receptor pathway substrate 15	protein_coding	[illegible]
13859	NM_001122832,NM_001289859,NM_007944,XM_006509544,XM_006509545,XM_006509546,XM_006509547,XM_006509548,XM_006509550,XM_017312559,XM_017312560,XR_001778398,XR_001778399,XR_001778400,XR_001778401,XR_003947228,XR_003947229,XR_003947230,XR_003947231,XR_379029,XR_379030,XR_379031,XR_379032	Eps15l1	epidermal growth factor receptor pathway substrate 15-like 1	protein_coding	[illegible]
13860	NM_001271587,NM_001271588,NM_001271589,NM_001271595,NM_007945,XM_006505508,XM_011241204,XM_017321383,XM_030255145	Eps8	epidermal growth factor receptor pathway substrate 8	protein_coding	[illegible]
13861	NM_007946,XM_006532173,XM_006532174	Epx	eosinophil peroxidase	protein_coding	[illegible]
13864	NM_010150,XM_006509553,XM_011242264,XM_011242265	Nr2f6	nuclear receptor subfamily 2, group F, member 6	protein_coding	[illegible]
13865	NM_001347538,NM_001347539,NM_010151,XM_006517085,XM_017315378,XM_030247140	Nr2f1	nuclear receptor subfamily 2, group F, member 1	protein_coding	[illegible]
13866	NM_001003817	Erbb2	erb-b2 receptor tyrosine kinase 2	protein_coding	[illegible]
13867	NM_010153	Erbb3	erb-b2 receptor tyrosine kinase 3	protein_coding	[illegible]
13869	NM_010154,XM_006495692,XM_006495693,XM_006495694,XM_006495695	Erbb4	erb-b2 receptor tyrosine kinase 4	protein_coding	[illegible]
13870	NM_001127324,NM_007948,XM_006539539	Ercc1	excision repair cross-complementing rodent repair deficiency, complementation group 1	protein_coding	[illegible]
13871	NM_001363981,NM_007949	Ercc2	excision repair cross-complementing rodent repair deficiency, complementation group 2	protein_coding	[illegible]
13872	NM_133658,XM_006525636,XR_385973	Ercc3	excision repair cross-complementing rodent repair deficiency, complementation group 3	protein_coding	[illegible]
13874	NM_007950	Ereg	epiregulin	protein_coding	[illegible]
13875	NM_010155,XM_030242128,XM_030242129,XM_030242130	Erf	Ets2 repressor factor	protein_coding	[illegible]
13876	NM_001302152,NM_001302153,NM_001302154,NM_001302179,NM_001302183,NM_133659,XM_006522894,XM_006522895,XM_006522896,XM_006522899,XM_006522900,XM_006522903,XM_006522904,XM_030248966,XM_030248967,XM_030248968,XM_030248969,XM_030248970,XM_030248971,XM_030248972,XM_030248973	Erg	ETS transcription factor	protein_coding	[illegible]
13877	NM_001347409,NM_007951,XM_011243995	Erh	ERH mRNA splicing and mitosis factor	protein_coding	[illegible]
13885	NM_001285423,NM_016903,XM_006518543,XM_030247645	Esd	esterase D/formylglutathione hydrolase	protein_coding	[illegible]
13924	NR_153383	Ptprv	protein tyrosine phosphatase, receptor type, V	transcribed_pseudogene	[illegible]
13929	NM_001252193,NM_025275	Amz2	archaelysin family metallopeptidase 2	protein_coding	[illegible]
13972	NM_001081682,NM_001285491,NM_001285493,NM_001285494,NM_023120,NR_104347,XM_011245828,XM_011245829,XM_011245830,XM_011245831,XM_011245832,XM_017316874,XM_030248974,XM_030248975,XM_030248976,XR_003951760	Gnb1l	guanine nucleotide binding protein (G protein), beta polypeptide 1-like	protein_coding	[illegible]
13982	NM_001302531,NM_001302532,NM_001302533,NM_007956,NR_126357,XM_006512433,XM_006512434,XM_006512435,XM_011243066,XM_011243067,XM_011243068,XM_017313797	Esr1	estrogen receptor 1 (alpha)	protein_coding	[illegible]
13983	NM_010157,NM_207707,NR_104386	Esr2	estrogen receptor 2 (beta)	protein_coding	[illegible]
13990	NM_001253392,NM_001355248,NM_001355249,NM_001355250,NM_007958,XM_006505510,XM_006505512,XM_006505513,XM_011241205,XR_003956162,XR_003956163	Smarcad1	SWI/SNF-related, matrix-associated actin-dependent regulator of chromatin, subfamily a, containing DEAD/H box 1	protein_coding	[illegible]
13992	NM_010158,XM_006520410,XR_383979	Khdrbs3	KH domain containing, RNA binding, signal transduction associated 3	protein_coding	[illegible]
13996	NR_015349	Etohd2	ethanol decreased 2	lncRNA	[illegible]
13998	NM_053072,XM_006513214,XM_030244876,XR_003948589	Fgd6	FYVE, RhoGEF and PH domain containing 6	protein_coding	[illegible]
13999	NM_001033123,XM_017315404	Gm14288	predicted gene 14288	protein_coding	[illegible]
14000	NM_001130149,NM_026799,XM_006520021,XR_383823	Drosha	drosha, ribonuclease type III	protein_coding	[illegible]
14004	NM_024166	Chchd2	coiled-coil-helix-coiled-coil-helix domain containing 2	protein_coding	[illegible]
14007	NM_001110228,NM_001110229,NM_001110230,NM_001110231,NM_001110232,NM_001160292,NM_001160293,NM_001310447,NM_001347094,NM_010160,XM_006497333,XM_006497334,XM_006497335,XM_006497336,XM_006497337,XM_006497338,XM_006497339,XM_011238949,XM_011238953,XM_017315442,XM_017315443,XM_017315466,XM_030247313,XM_030247314,XM_030247317,XM_030247319,XM_030247320,XM_030247323,XM_030247325,XM_030247326,XM_030247328,XM_030247331,XM_030247333,XM_030247334,XM_030247335,XM_030247336,XM_030247337,XM_030247338,XM_030247339,XM_030247340,XM_030247343,XM_030247344,XM_030247345,XM_030247346,XM_030247347,XM_030247349,XM_030247351,XM_030247354,XM_030247358,XM_030247359,XM_030247360,XM_030247362,XM_030247364	Celf2	CUGBP, Elav-like family member 2	protein_coding	[illegible]
14009	NM_001163154,NM_001347379,NM_007960,XM_006514964,XM_006514965,XM_006514966,XM_006514968,XM_006514969,XM_006514970,XM_006514971,XM_006514972	Etv1	ets variant 1	protein_coding	[illegible]
14011	NM_001303102,NM_007961,XM_011241206,XM_011241207,XM_011241208	Etv6	ets variant 6	protein_coding	[illegible]
14012	NM_001357727,NM_007962	Mpzl2	myelin protein zero-like 2	protein_coding	[illegible]
14013	NM_001361034,NM_001370768,NM_001370769,NM_007963,NM_021442,XM_006535393,XM_006535394,XM_011249656,XM_011249659,XM_011249660,XM_011249661,XM_011249662,XM_011249663,XM_011249664,XM_017319454,XM_030252416	Mecom	MDS1 and EVI1 complex locus	protein_coding	[illegible]
14017	NM_001033711,NM_010161	Evi2a	ecotropic viral integration site 2a	protein_coding	[illegible]
14020	NM_007964,XM_006534776,XM_006534777,XM_006534778,XM_006534779,XM_006534780,XM_006534781,XM_006534783,XM_006534784,XM_006534785,XM_006534786,XM_006534787,XM_011249407,XM_011249408,XM_030254126,XM_030254127,XM_030254128	Evi5	ecotropic viral integration site 5	protein_coding	[illegible]
14025	NM_001159289,NM_001159290,NM_001242934,NM_016707,XM_006514504,XM_006514505,XM_006514507,XM_011243659,XM_011243660,XM_030245542,XM_030245543,XM_030245544,XM_030245545,XM_030245546,XM_030245547,XM_030245548	Bcl11a	B cell CLL/lymphoma 11A (zinc finger protein)	protein_coding	[illegible]
14026	NM_001163394,NM_001163395,NM_001163396,NM_007965,XM_006515470,XM_006515471,XM_030246555,XM_030246556	Evl	Ena-vasodilator stimulated phosphoprotein	protein_coding	[illegible]
14027	NM_025276,XM_017314254,XM_030245549	Evpl	envoplakin	protein_coding	[illegible]
14030	NM_001283061,NM_001283062,NM_001283063,NM_001362612,NM_001362613,NM_001362614,NM_001362615,NM_001362616,NM_001362617,NM_001362618,NM_001362620,NM_007968,XM_006514508,XM_006514509,XM_006514510,XM_017314255,XM_030245550,XR_001779874,XR_003949296	Ewsr1	Ewing sarcoma breakpoint region 1	protein_coding	[illegible]
14038	NM_007969	Wfdc18	WAP four-disulfide core domain 18	protein_coding	[illegible]
14042	NM_010162	Ext1	exostoses (multiple) 1	protein_coding	[illegible]
14043	NM_001355075,NM_001355076,NM_010163,XM_006498730,XM_006498731,XM_006498733	Ext2	exostoses (multiple) 2	protein_coding	[illegible]
14048	NM_001252192,NM_001310459,NM_010164,XM_006495446,XM_006495451,XM_006495452,XM_011238344,XM_011238346,XM_017315348,XM_017315381,XM_017315414,XM_017315422,XM_030247470,XR_001780756,XR_003950638	Eya1	EYA transcriptional coactivator and phosphatase 1	protein_coding	[illegible]
14049	NM_001271962,NM_001271963,NM_010165,XM_006498739	Eya2	EYA transcriptional coactivator and phosphatase 2	protein_coding	[illegible]
14050	NM_010166,NM_210071,NM_211357,XM_006538537,XM_006538538,XM_006538540	Eya3	EYA transcriptional coactivator and phosphatase 3	protein_coding	[illegible]
14051	NM_001347372,NM_010167,XM_006512523,XM_006512524,XM_006512526,XM_006512527,XM_006512528,XM_006512529,XM_006512530,XM_011243112,XM_011243115,XM_017313798,XM_017313799,XM_030244877,XM_030244878,XM_030244879,XM_030244880,XR_001779457	Eya4	EYA transcriptional coactivator and phosphatase 4	protein_coding	[illegible]
14055	NM_007970,XM_006532178	Ezh1	enhancer of zeste 1 polycomb repressive complex 2 subunit	protein_coding	[illegible]
14056	NM_001146689,NM_007971,XM_006505520,XM_006505521,XM_006505523,XM_006505524,XM_006505525,XM_006505526,XM_006505527,XM_006505528,XM_006505529,XM_030255150,XM_030255151,XM_030255152,XM_030255154,XM_030255155	Ezh2	enhancer of zeste 2 polycomb repressive complex 2 subunit	protein_coding	[illegible]
14057	NM_027324,XM_006516849,XM_006516850,XR_003950424,XR_003950425	Sfxn1	sideroflexin 1	protein_coding	[illegible]
14058	NM_001242368,NM_007972	F10	coagulation factor X	protein_coding	[illegible]
14060	NM_031164,XM_006529132,XM_006529133	F13b	coagulation factor XIII, beta subunit	protein_coding	[illegible]
14061	NM_010168,XM_011239288,XM_030247399	F2	coagulation factor II	protein_coding	[illegible]
14062	NM_010169	F2r	coagulation factor II (thrombin) receptor	protein_coding	[illegible]
14063	NM_007974	F2rl1	coagulation factor II (thrombin) receptor-like 1	protein_coding	[illegible]
14064	NM_010170	F2rl2	coagulation factor II (thrombin) receptor-like 2	protein_coding	[illegible]
14065	NM_007975	F2rl3	coagulation factor II (thrombin) receptor-like 3	protein_coding	[illegible]
14066	NM_010171	F3	coagulation factor III	protein_coding	[illegible]
14067	NM_007976	F5	coagulation factor V	protein_coding	[illegible]
14068	NM_010172,XR_003947232	F7	coagulation factor VII	protein_coding	[illegible]
14069	NM_001161373,NM_001161374,NM_007977,XM_006527789,XM_006527790,XM_006527791,XM_006527792	F8	coagulation factor VIII	protein_coding	[illegible]
14070	NM_007978	F8a	factor 8-associated gene A	protein_coding	[illegible]
14071	NM_001305797,NM_007979,XM_030251221	F9	coagulation factor IX	protein_coding	[illegible]
14073	NM_010173,NR_151700,XR_001784089,XR_001784090,XR_001784091	Faah	fatty acid amide hydrolase	protein_coding	[illegible]
14074	gene-Fabp3-ps1	Fabp3-ps1	fatty acid binding protein 3, muscle and heart, pseudogene 1	pseudogene	[illegible]
14077	NM_010174	Fabp3	fatty acid binding protein 3, muscle and heart	protein_coding	[illegible]
14081	NM_001302163,NM_007981,XM_006509262,XM_006509264,XM_006509265,XM_006509266,XM_017312563,XM_030243285	Acsl1	acyl-CoA synthetase long-chain family member 1	protein_coding	[illegible]
14082	NM_010175	Fadd	Fas (TNFRSF6)-associated via death domain	protein_coding	[illegible]
14083	NM_001358045,NM_001358046,NM_007982,XM_006520415,XM_006520416,XM_006520418,XM_006520420,XM_006520421,XM_006520422,XM_006520424,XM_006520425,XM_006520427,XM_006520428,XM_006520429,XM_006520430,XM_006520431,XM_006520432,XM_006520434,XM_006520435,XM_011245452,XM_011245453,XM_030248319,XM_030248320,XM_030248322,XM_030248323,XM_030248324,XM_030248325,XM_030248326,XM_030248328,XM_030248329,XM_030248330,XM_030248331,XM_030248332,XM_030248333,XM_030248334,XM_030248335,XM_030248336,XR_003951347	Ptk2	PTK2 protein tyrosine kinase 2	protein_coding	[illegible]
14084	NM_007983	Faf1	Fas-associated factor 1	protein_coding	[illegible]
14085	NM_010176	Fah	fumarylacetoacetate hydrolase	protein_coding	[illegible]
14086	NM_007984	Fscn1	fascin actin-bundling protein 1	protein_coding	[illegible]
14087	NM_016925,XM_006530661,XM_006530662,XM_006530663,XM_006530664,XM_006530665,XM_006530666,XR_003947233,XR_387	Fanca	Fanconi anemia, complementation group A	protein_coding	[illegible]
14088	NM_001042673,NM_001282942,NM_001347514,NM_001347515,NM_007985,XM_006517087,XM_006517090,XM_011244474,XM_011244475,XM_011244476,XM_011244477,XM_011244478,XM_011244480,XM_011244481,XM_011244482,XM_011244483,XM_011244484,XM_011244485,XM_011244487,XM_017315382,XM_030247144,XM_030247145,XM_030247146,XM_030247147,XM_030247148,XM_030247149,XR_001780750,XR_001780751,XR_003950426	Fancc	Fanconi anemia, complementation group C	protein_coding	[illegible]
14089	NM_007986,XM_006498746,XM_011239289,XM_017315507,XM_017315512	Fap	fibroblast activation protein	protein_coding	[illegible]
14102	NM_001146708,NM_007987,XM_030250746,XM_030250747,XM_030250748,XM_030250749,XM_030250750,XM_030250751	Fas	Fas (TNF receptor superfamily member 6)	protein_coding	[illegible]
14103	NM_001205243,NM_010177	Fasl	Fas ligand (TNF superfamily, member 6)	protein_coding	[illegible]
14104	NM_007988,XM_030245556	Fasn	fatty acid synthase	protein_coding	[illegible]
14105	NM_001080387,NM_001284195,NM_001284196,NM_010178,XM_011250188	Srsf10	serine/arginine-rich splicing factor 10	protein_coding	[illegible]
14106	NM_007989,XM_006520436	Foxh1	forkhead box H1	protein_coding	[illegible]
14107	NM_001081286,XM_006509267,XM_006509268,XM_006509270,XM_006509271,XM_006509272,XM_006509273,XM_006509274,XM_006509276,XM_006509277,XM_011242173,XM_017312564,XM_030243287,XM_030243288,XR_001778405	Fat1	FAT atypical cadherin 1	protein_coding	[illegible]
14109	NM_001160239,NM_001190436,NM_007990	Fau	Finkel-Biskis-Reilly murine sarcoma virus (FBR-MuSV) ubiquitously expressed (fox derived)	protein_coding	[illegible]
14113	NM_007991,XM_030242131	Fbl	fibrillarin	protein_coding	[illegible]
14114	NM_001347088,NM_010180	Fbln1	fibulin 1	protein_coding	[illegible]
14115	NM_001081437,NM_007992,XM_006505530,XM_030255156,XM_030255157	Fbln2	fibulin 2	protein_coding	[illegible]
14118	NM_007993,XM_006498747,XM_011239290,XR_866221	Fbn1	fibrillin 1	protein_coding	[illegible]
14119	NM_010181,XM_017317828,XM_030250333	Fbn2	fibrillin 2	protein_coding	[illegible]
14120	NM_007994	Fbp2	fructose bisphosphatase 2	protein_coding	[illegible]
14121	NM_019395,XM_011244488	Fbp1	fructose bisphosphatase 1	protein_coding	[illegible]
14123	NM_010183,XM_006507348	Fbrs	fibrosin	protein_coding	[illegible]
14125	NM_010184,XM_006496655	Fcer1a	Fc receptor, IgE, high affinity I, alpha polypeptide	protein_coding	[illegible]
14126	NM_001276328,NM_001276329,NM_001276330,NM_013516,XM_017318060	Ms4a2	membrane-spanning 4-domains, subfamily A, member 2	protein_coding	[illegible]
14127	NM_010185	Fcer1g	Fc receptor, IgE, high affinity I, gamma polypeptide	protein_coding	[illegible]
14128	NM_001253737,NM_001253739,NM_001253743,NM_001253745,NM_001253746,NM_001253747,NM_013517,XM_006508697	Fcer2a	Fc receptor, IgE, low affinity II, alpha polypeptide	protein_coding	[illegible]
14129	NM_010186	Fcgr1	Fc receptor, IgG, high affinity I	protein_coding	[illegible]
14130	NM_001077189,NM_010187	Fcgr2b	Fc receptor, IgG, low affinity IIb	protein_coding	[illegible]
14131	NM_001356511,NM_010188,XM_006496658,XM_006496659,XM_006496660,XM_030247672,XM_030247708	Fcgr3	Fc receptor, IgG, low affinity III	protein_coding	[illegible]
14132	NM_001357117,NM_010189	Fcgrt	Fc receptor, IgG, alpha chain transporter	protein_coding	[illegible]
14133	NM_007995,XM_006497673,XM_006497675,XM_011239016	Fcna	ficolin A	protein_coding	[illegible]
14134	NM_010190,XM_006497676	Fcnb	ficolin B	protein_coding	[illegible]
14137	NM_001360211,NM_010191,XM_006518547,XM_017315844	Fdft1	farnesyl diphosphate farnesyl transferase 1	protein_coding	[illegible]
14148	NM_001301728,NM_007996	Fdx1	ferredoxin 1	protein_coding	[illegible]
14149	NM_007997,XM_006532186,XM_017314257,XR_001779876	Fdxr	ferredoxin reductase	protein_coding	[illegible]
14151	NM_001348055,NM_007998,XR_385974	Fech	ferrochelatase	protein_coding	[illegible]
14154	NM_010192	Fem1a	feminization 1 homolog a (C. elegans)	protein_coding	[illegible]
14155	NM_010193	Fem1b	feminization 1 homolog b (C. elegans)	protein_coding	[illegible]
14156	NM_001271614,NM_001271615,NM_007999,XM_006526661,XM_006526663,XM_006526664	Fen1	flap structure specific endonuclease 1	protein_coding	[illegible]
14158	NM_001037997,NM_001286415,NM_008000,XM_006523640,XM_006523641,XM_006523643,XM_006523645,XM_006523647,XM_006523650,XM_011246293,XM_017317242,XM_017317243,XM_017317244,XM_030249496,XR_003952084,XR_003952085,XR_003952086,XR_003952087,XR_385280,XR_385281	Fer	fer (fms/fps related) protein kinase	protein_coding	[illegible]
14159	NM_010194,XM_006540604,XR_003946388	Fes	feline sarcoma oncogene	protein_coding	[illegible]
14160	NM_010195	Lgr5	leucine rich repeat containing G protein coupled receptor 5	protein_coding	[illegible]
14161	NM_001111048,NM_010196	Fga	fibrinogen alpha chain	protein_coding	[illegible]
14163	NM_008001,XM_006528699	Fgd1	FYVE, RhoGEF and PH domain containing 1	protein_coding	[illegible]
14164	NM_010197,XM_006525641,XM_006525642,XM_006525644,XM_006525645,XM_006525647,XM_006525648,XM_006525649,XM_011246841,XM_011246842,XM_011246843,XM_017317830,XM_017317831,XM_017317832	Fgf1	fibroblast growth factor 1	protein_coding	[illegible]
14165	NM_008002	Fgf10	fibroblast growth factor 10	protein_coding	[illegible]
14166	NM_001291104,NM_001362623,NM_001362624,NM_010198,XM_006532188,XM_011248721,XM_011248722	Fgf11	fibroblast growth factor 11	protein_coding	[illegible]
14167	NM_001276419,NM_001276420,NM_001357064,NM_010199,NM_183064	Fgf12	fibroblast growth factor 12	protein_coding	[illegible]
14168	NM_001290414,NM_001290415,NM_001356335,NM_010200,XM_006527793,XM_006527795,XM_006527796,XM_006527797,XM_006527799	Fgf13	fibroblast growth factor 13	protein_coding	[illegible]

14169	NM_010201,NM_207667,XM_006518549,XM_011244952,XM_017315845	Fgf14	fibroblast growth factor 14	protein_coding	NP_034331.2;NP_997550.1;XP_006518612.1;XP_011243254.1;XP_017171334.1	109189	.	0.010413	[illegible]
14170	NM_008003	Fgf15	fibroblast growth factor 15	protein_coding	NP_032029.1	1096383	.	0.000000	[illegible]
14171	NM_001360108,NM_008004	Fgf17	fibroblast growth factor 17	protein_coding	NP_001347037.1;NP_032030.1	1202401	.	0.120952	[illegible]
14172	NM_008005,NR_102395	Fgf18	fibroblast growth factor 18	protein_coding	NP_032031.1	1277980	.	2.216012	[illegible]
14173	NM_008006,XM_006535399	Fgf2	fibroblast growth factor 2	protein_coding	NP_032032.1;XP_006535462.1	95516	.	6.462814	[illegible]
14174	NM_008007	Fgf3	fibroblast growth factor 3	protein_coding	NP_032033.2	95517	.	0.000000	[illegible]
14176	NM_001277268,NM_010203	Fgf5	fibroblast growth factor 5	protein_coding	NP_001264197.1;NP_034333.1	95519	.	0.000000	[illegible]
14177	NM_010204	Fgf6	fibroblast growth factor 6	protein_coding	NP_034334.1	95520	.	0.150968	[illegible]
14178	NM_008008	Fgf7	fibroblast growth factor 7	protein_coding	NP_032034.1	95521	.	1.584922	[illegible]
14180	NM_013518,XM_006518554,XM_030247647,XM_030247648	Fgf9	fibroblast growth factor 9	protein_coding	NP_038546.2;XP_006518617.1;XP_030103507.1;XP_030103508.1	104723	.	1.197382	[illegible]
14181	NM_001271616,NM_008009,XM_006503711,XM_006503712,XM_030254129	Fgfbp1	fibroblast growth factor binding protein 1	protein_coding	NP_001258545.1;NP_032035.2;XP_006503774.1;XP_006503775.1;XP_030109989.1	1096350	.	0.000000	[illegible]
14182	NM_001079908,NM_001079909,NM_010206	Fgfr1	fibroblast growth factor receptor 1	protein_coding	NP_001073377.1;NP_001073378.1;NP_034336.2	95522	.	82.597010	[illegible]
14183	NM_001347638,NM_010207,NM_201601	Fgfr2	fibroblast growth factor receptor 2	protein_coding	NP_001334567.1;NP_034337.2;NP_963895.2	95523	.	66.550331	[illegible]
14184	NM_001163215,NM_001163216,NM_001163217,NM_001205270,NM_001359036,NM_001359037,NM_008010	Fgfr3	fibroblast growth factor receptor 3	protein_coding	NP_001156687.1;NP_001156688.1;NP_001156689.1;NP_001192199.1;NP_001345965.1;NP_001345966.1;NP_032036.2	95524	.	60.033055	[illegible]
14186	NM_008011,XM_006517099,XM_006517100	Fgfr4	fibroblast growth factor receptor 4	protein_coding	NP_032037.2;XP_006517162.3;XP_006517163.1	95525	.	1.435736	[illegible]
14187	NM_008012	Akr1b8	aldo-keto reductase family 1, member B8	protein_coding	NP_032038.1	107673	.	5.125887	[illegible]
14190	NM_008013	Fgl2	fibrinogen-like protein 2	protein_coding	NP_032039.2	103266	.	2.332985	[illegible]
14191	NM_010208,XM_006538544,XM_006538545	Fgr	FGR proto-oncogene, Src family tyrosine kinase	protein_coding	NP_034338.3;XP_006538607.1;XP_006538608.1	95527	.	0.562085	[illegible]
14194	NM_010209	Fh1	fumarate hydratase 1	protein_coding	NP_034339.2	95530	.	24.340168	[illegible]
14198	NM_001308285,NM_001308286,NM_001360141,NM_010210,XM_006517924,XM_011244732,XM_030247649	Fhit	fragile histidine triad gene	protein_coding	NP_001295214.1;NP_001295215.1;NP_001347070.1;NP_034340.1;XP_006517987.1;XP_011243034.1;XP_030103509.1	1277947	.	0.778769	[illegible]
14199	NM_001077361,NM_001077362,NM_001287800,NM_010211,XM_006527800,XM_006527801,XM_006527802,XM_006527803,XM_006527804,XM_006527805,XM_006527807,XM_006527808,XM_006527809,XM_017318381,XM_017318382,XM_030251222,XM_030251223,XM_030251224,XM_030251225	Fhl1	four and a half LIM domains 1	protein_coding	NP_001070829.1;NP_001070830.1;NP_001274729.1;NP_034341.2;XP_006527863.2;XP_006527864.1;XP_006527865.1;XP_006527866.1;XP_006527867.1;XP_006527868.1;XP_006527870.2;XP_006527871.2;XP_006527872.1;XP_017173870.1;XP_017173871.1;XP_030107082.1;XP_030107083.1;XP_030107084.1;XP_030107085.1	1298387	.	94.107212	[illegible]
14200	NM_001289533,NM_010212,XM_011238434	Fhl2	four and a half LIM domains 2	protein_coding	NP_001276462.1;NP_034342.1;XP_011236736.1	1338762	.	12.135733	[illegible]
14201	NM_010213,XM_006502766,XM_006502768,XM_006502769,XR_003954878	Fhl3	four and a half LIM domains 3	protein_coding	NP_034343.2;XP_006502829.1;XP_006502831.1;XP_006502832.1	1341092	.	10.335691	[illegible]
14202	NM_010214	Fhl4	four and a half LIM domains 4	protein_coding	NP_034344.2	1338765	.	0.258612	[illegible]
14204	NM_010215	Il4i1	interleukin 4 induced 1	protein_coding	NP_034345.2	109552	.	0.123143	[illegible]
14205	NM_001308489,NM_010216	Vegfd	vascular endothelial growth factor D	protein_coding	NP_001295418.1;NP_034346.1	108037	.	0.868736	[illegible]
14208	NM_008014	Ppm1g	protein phosphatase 1G (formerly 2C), magnesium-dependent, gamma isoform	protein_coding	NP_032040.1	106065	.	29.553457	[illegible]
14211	NM_001301412,NM_008017	Smc2	structural maintenance of chromosomes 2	protein_coding	NP_001288341.1;NP_032043.3	106067	.	9.074150	[illegible]
14218	NM_001164717,NM_008018,XM_006526669,XM_006526670	Sh3pxd2a	SH3 and PX domains 2A	protein_coding	NP_001158189.1;NP_032044.2;XP_006526732.1;XP_006526733.1	1298393	.	66.783911	[illegible]
14219	NM_010217	Ccn2	cellular communication network factor 2	protein_coding	NP_034347.2	95537	.	75.782120	[illegible]
14221	NM_010218	Fjx1	four jointed box 1	protein_coding	NP_034348.2	1341907	.	3.318007	[illegible]
14225	NM_001302077,NM_001302078,NM_001302079,NM_001302080,NM_001355077,NM_001355078,NM_008019,NR_126058,XM_011239292,XM_017315525	Fkbp1a	FK506 binding protein 1a	protein_coding	NP_001289006.1;NP_001289007.1;NP_001289008.1;NP_001289009.1;NP_001342006.1;NP_001342007.1;NP_032045.1;XP_011237594.2;XP_017171014.2	95541	.	103.755938	[illegible]
14226	NM_016863,XM_006514974,XM_017314959,XR_003949985	Fkbp1b	FK506 binding protein 1b	protein_coding	NP_058559.3;XP_006515037.3;XP_017170448.1	1336205	.	5.875855	[illegible]
14227	NM_001166368,NM_001360286,NM_001360288,NM_008020	Fkbp2	FK506 binding protein 2	protein_coding	NP_001159840.1;NP_001347215.1;NP_001347217.1;NP_032046.1	95542	.	16.724526	[illegible]
14228	NM_010219	Fkbp4	FK506 binding protein 4	protein_coding	NP_034349.1	95543	.	31.405226	[illegible]
14229	NM_010220	Fkbp5	FK506 binding protein 5	protein_coding	NP_034350.1	104670	.	8.161664	[illegible]
14230	NM_001163481,NM_010221	Fkbp10	FK506 binding protein 10	protein_coding	NP_001156953.1;NP_034351.2	104769	.	226.541935	[illegible]
14231	NM_001355079,NM_001355080,NM_010222	Fkbp7	FK506 binding protein 7	protein_coding	NP_001342008.1;NP_001342009.1;NP_034352.1	1336879	.	98.041690	[illegible]
14232	NM_001111066,NM_001199631,NM_010223,XM_006509556,XM_017312565,XM_017312566,XM_017312567,XM_017312568,XR_001778406,XR_003947234,XR_379034	Fkbp8	FK506 binding protein 8	protein_coding	NP_001104536.1;NP_001186560.1;NP_034353.2;XP_006509619.1;XP_017168054.1;XP_017168055.1;XP_017168056.1;XP_017168057.1	1341070	.	65.599473	[illegible]
14233	NM_023907	Foxi1	forkhead box I1	protein_coding	NP_076396.3	1096329	.	0.000000	[illegible]
14234	NM_013519	Foxc2	forkhead box C2	protein_coding	NP_038547.2	1347481	.	4.302252	[illegible]
14235	NM_008021	Foxm1	forkhead box M1	protein_coding	NP_032047.4	1347487	.	7.711923	[illegible]
14236	NM_001355742,NM_001355743,NM_180974,XM_006523652,XM_006523653,XM_006523654,XM_030249497,XM_030249498	Foxn2	forkhead box N2	protein_coding	NP_001342671.1;NP_001342672.1;NP_851305.2;XP_006523715.1;XP_006523716.1;XP_006523717.1;XP_030105357.1;XP_030105358.1	1347478	.	5.936155	[illegible]
14237	NM_008022	Foxd4	forkhead box D4	protein_coding	NP_032048.1	1347467	.	0.020204	[illegible]
14238	NM_010225	Foxf2	forkhead box F2	protein_coding	NP_034355.2	1347479	.	0.174966	[illegible]
14239	NM_010226	Foxs1	forkhead box S1	protein_coding	NP_034356.1	95546	.	3.297200	[illegible]
14241	NM_008024	Foxl1	forkhead box L1	protein_coding	NP_032050.2	1347469	.	0.244369	[illegible]
14245	NM_001130412,NM_001355598,NM_015763,NM_172950,XM_006514975,XM_006514976,XM_006514977,XM_006514978,XM_006514982,XM_006514983,XM_006514984,XM_006514986,XM_006514987,XM_011243815,XM_030246557,XM_030246558,XM_030246559,XM_030246560	Lpin1	lipin 1	protein_coding	NP_001123884.1;NP_001342527.1;NP_056578.2;NP_766538.2;XP_006515038.1;XP_006515039.1;XP_006515040.1;XP_006515041.1;XP_006515045.1;XP_006515046.1;XP_006515047.1;XP_006515049.1;XP_006515050.1;XP_011242117.1;XP_030102417.1;XP_030102418.1;XP_030102419.1;XP_030102420.1	1891340	.	3.276856	[illegible]
14246	XM_017319842	Flg	filaggrin	protein_coding	XP_017175331.1	95553	.	0.000000	[illegible]
14247	NM_008026,XM_006510008,XM_006510009,XM_017313133	Fli1	Friend leukemia integration 1	protein_coding	NP_032052.1;XP_006510071.1;XP_006510072.1;XP_017168622.1	95554	.	9.560413	[illegible]
14248	NM_001302207,NM_022009	Flii	flightless I actin binding protein	protein_coding	NP_001289136.1;NP_071292.1	1342286	.	22.870041	[illegible]
14251	NM_008027,XM_006523656,XM_006523657	Flot1	flotillin 1	protein_coding	NP_032053.1;XP_006523719.1;XP_006523720.1	1100500	.	23.320270	[illegible]
14252	NM_001040403,NM_001284227,NM_001284228,NM_001362625,NM_001362626,NM_001362627,NM_008028,XM_030245557,XM_030245558	Flot2	flotillin 2	protein_coding	NP_001035493.1;NP_001271156.1;NP_001271157.1;NP_001349554.1;NP_001349555.1;NP_001349556.1;NP_032054.1;XP_030101417.1;XP_030101418.1	103309	.	18.525052	[illegible]
14254	NM_001363135,NM_010228,XM_006504801,XM_006504802,XM_006504803,XM_011241000	Flt1	FMS-like tyrosine kinase 1	protein_coding	NP_001350064.1;NP_034358.2;XP_006504864.1;XP_006504865.1;XP_006504866.1;XP_011239302.1	95558	.	6.682995	[illegible]
14255	NM_010229,XM_006504804,XM_006504805,XM_030254130	Flt3	FMS-like tyrosine kinase 3	protein_coding	NP_034359.2;XP_006504867.1;XP_006504868.1;XP_030109990.1	95559	.	0.080875	[illegible]
14256	NM_013520,NR_102725,XM_006540606,XM_006540607,XM_006540608,XM_006540609,XM_006540610,XM_006540612,XM_011250793,XM_011250794,XM_030242133,XR_003946389,XR_882010,XR_882011,XR_882012,XR_882013,XR_882014,XR_882015,XR_882016,XR_882017,XR_882018	Flt3l	FMS-like tyrosine kinase 3 ligand	protein_coding	NP_038548.3;XP_006540669.1;XP_006540670.1;XP_006540671.1;XP_006540672.1;XP_006540673.1;XP_006540675.1;XP_011249095.1;XP_011249096.1;XP_030097993.1	95560	.	3.770939	[illegible]
14257	NM_008029	Flt4	FMS-like tyrosine kinase 4	protein_coding	NP_032055.1	95561	.	2.135873	[illegible]
14260	NM_001285458,NM_001285459,NM_001287817,NM_001287818,NM_010230,XM_006498750,XM_011239294,XM_011239295,XM_011239298,XM_017315533	Fmn1	formin 1	protein_coding	NP_001272387.1;NP_001272388.1;NP_001274746.1;NP_001274747.1;NP_034360.2;XP_006498813.1;XP_011237596.1;XP_011237597.1;XP_011237600.1;XP_017171022.1	101815	.	1.475627	[illegible]
14261	NM_001330291,NM_001330318,NM_010231,XM_006496663,XM_030247879	Fmo1	flavin containing monooxygenase 1	protein_coding	NP_001317220.1;NP_001317247.1;NP_034361.1;XP_006496726.1;XP_030103739.1	1310002	.	31.802557	[illegible]
14262	NM_008030	Fmo3	flavin containing monooxygenase 3	protein_coding	NP_032056.1	1100496	.	0.000000	[illegible]
14263	NM_001161763,NM_001161765,NM_001355119,NM_010232,XM_006500995,XM_006500996,XM_011240013,XM_030252418,XM_030252419,XM_030252420	Fmo5	flavin containing monooxygenase 5	protein_coding	NP_001155235.1;NP_001155237.1;NP_001342048.1;NP_034362.2;XP_006501058.1;XP_006501059.1;XP_011238315.1;XP_030108278.1;XP_030108279.1;XP_030108280.1	1310004	.	0.534583	[illegible]
14264	NM_021355	Fmod	fibromodulin	protein_coding	NP_067330.1	1328364	.	237.580765	[illegible]
14265	NM_001290424,NM_008031,XM_006527810,XM_006527811,XM_006527813,XM_006527814,XR_001782738	Fmr1	fragile X mental retardation 1	protein_coding	NP_001277353.1;NP_032057.2;XP_006527873.1;XP_006527874.1;XP_006527876.1;XP_006527877.1	95564	.	18.566933	[illegible]
14266	NM_008032,XM_006527816,XM_006527817,XM_006527818,XM_006527819,XM_006527820,XM_006527821,XM_030251226	Aff2	AF4/FMR2 family, member 2	protein_coding	NP_032058.2;XP_006527879.1;XP_006527880.1;XP_006527881.1;XP_006527882.1;XP_006527883.1;XP_006527884.1;XP_030107086.1	1202294	.	1.954431	[illegible]
14268	NM_001276408,NM_001276409,NM_001276410,NM_001276411,NM_001276412,NM_001276413,NM_010233,XM_006495697,XM_006495699,XM_006495700,XM_017315775,XM_017315837,XM_017315848,XM_030248098,XM_030248135	Fn1	fibronectin 1	protein_coding	NP_001263337.1;NP_001263338.1;NP_001263339.1;NP_001263340.1;NP_001263341.1;NP_001263342.1;NP_034363.1;XP_006495760.1;XP_006495762.1;XP_006495763.1;XP_017171264.1;XP_017171326.1;XP_017171337.1;XP_030103958.1;XP_030103995.1	95566	.	345.588729	[illegible]
14269	NM_001038700,NM_001177648,NM_001177649,NM_001177650,NM_001355105,NM_001355106,NM_019406,XM_006497684,XM_006497685,XM_006497686,XM_006497687,XM_006497688,XM_006497689,XM_006497690,XM_006497691,XM_006497692,XM_006497693,XM_006497694,XM_006497695,XM_017315551,XM_030247489,XM_030247490,XM_030247491,XM_030247492,XR_374060	Fnbp1	formin binding protein 1	protein_coding	NP_001033789.1;NP_001171119.1;NP_001171120.1;NP_001171121.1;NP_001342034.1;NP_001342035.1;NP_062279.1;XP_006497747.1;XP_006497748.1;XP_006497749.1;XP_006497750.1;XP_006497751.1;XP_006497752.1;XP_006497753.1;XP_006497754.1;XP_006497755.1;XP_006497756.1;XP_006497758.1;XP_017171040.1;XP_030103349.1;XP_030103350.1;XP_030103351.1;XP_0301[illegible]	109606	.	7.753565	[illegible]
14270	NM_001081011,XM_011247923,XR_001781100,XR_001781103	Srgap2	SLIT-ROBO Rho GTPase activating protein 2	protein_coding	NP_001074480.2;XP_011246225.1	109605	.	15.543627	[illegible]
14272	NM_008033	Fnta	farnesyltransferase, CAAX box, alpha	protein_coding	NP_032059.1	104683	.	27.149155	[illegible]
14275	NM_001252552,NM_001252553,NM_001252554,NM_008034,XM_006507360,XM_006507361,XM_006507363,XM_006507364,XM_006507365,XM_006507366,XM_006507367,XM_006507368,XM_006507369,XM_006507370	Folr1	folate receptor 1 (adult)	protein_coding	NP_001239481.1;NP_001239482.1;NP_001239483.1;NP_032060.2;XP_006507423.1;XP_006507424.1;XP_006507426.1;XP_006507427.1;XP_006507428.1;XP_006507429.1;XP_006507430.1;XP_006507431.1;XP_006507432.1;XP_006507433.1	95568	.	0.000000	[illegible]
14276	NM_001303231,NM_001303239,NM_008035,XM_006507371	Folr2	folate receptor 2 (fetal)	protein_coding	NP_001290160.1;NP_001290168.1;NP_032061.1;XP_006507434.1	95569	.	1.689471	[illegible]
14281	NM_010234	Fos	FBJ osteosarcoma oncogene	protein_coding	NP_034364.1	95574	.	16.048798	[illegible]
14282	NM_001347586,NM_008036,XM_006539543	Fosb	FBJ osteosarcoma oncogene B	protein_coding	NP_001334515.1;NP_032062.1;XP_006539606.1	95575	.	3.320256	[illegible]
14283	NM_010235	Fosl1	fos-like antigen 1	protein_coding	NP_034365.1	107179	.	0.085581	[illegible]
14284	NM_008037	Fosl2	fos-like antigen 2	protein_coding	NP_032063.2	102858	.	15.706548	[illegible]
14287	NM_010236,XM_006497696,XM_006497697,XM_006497698,XM_011239021,XM_030247501,XM_030247502,XM_030247503,XR_001780795	Fpgs	folylpolyglutamyl synthetase	protein_coding	NP_034366.2;XP_006497759.2;XP_006497760.1;XP_006497761.4;XP_011237323.1;XP_030103361.1;XP_030103362.1;XP_030103363.1	95576	.	13.307219	[illegible]
14289	NM_008039	Fpr2	formyl peptide receptor 2	protein_coding	NP_032065.1	1278319	.	0.431624	[illegible]
14293	NM_013521	Fpr1	formyl peptide receptor 1	protein_coding	NP_038549.1	107443	.	0.216815	[illegible]
14296	NM_008043	Frat1	frequently rearranged in advanced T cell lymphomas	protein_coding	NP_032069.2	109450	.	0.407983	[illegible]
14297	NM_008044,NR_157400	Fxn	frataxin	protein_coding	NP_032070.1	1096879	.	2.535362	[illegible]
14299	NM_019681,XR_866007,XR_866008	Ncs1	neuronal calcium sensor 1	protein_coding	NP_062655.1	109166	.	7.055274	[illegible]
14300	NM_013522,XM_017312570	Frg1	FSHD region gene 1	protein_coding	NP_038550.2;XP_017168059.1	893597	.	20.250779	[illegible]
14302	NM_001159544,NM_001358769,NM_010237,XM_006512534,XM_006512536	Frk	fyn-related kinase	protein_coding	NP_001153016.1;NP_001345698.1;NP_034367.2;XP_006512597.1;XP_006512599.1	103265	.	3.235627	[illegible]
14308	NM_008045	Fshb	follicle stimulating hormone beta	protein_coding	NP_032071.1	95582	.	0.000000	[illegible]
14311	NM_001301295,NM_178373,XM_006505531	Cidec	cell death-inducing DFFA-like effector c	protein_coding	NP_001288224.1;NP_848460.1;XP_006505594.1	95585	.	0.060640	[illegible]
14312	NM_001204973,NM_010238,NR_037970,XM_017317255,XM_030249499,XM_030249500,XM_030249501,XR_003952088	Brd2	bromodomain containing 2	protein_coding	NP_001191902.1;NP_034368.2;XP_017172744.1;XP_030105359.1;XP_030105360.1;XP_030105361.1	99495	.	40.223252	[illegible]
14313	NM_001301373,NM_001301375,NM_008046,XM_030247150	Fst	follistatin	protein_coding	NP_001288302.1;NP_001288304.1;NP_032072.1;XP_030103010.1	95586	.	1.667080	[illegible]
14314	NM_008047	Fstl1	follistatin-like 1	protein_coding	NP_032073.2	102793	.	414.095337	[illegible]
14319	NM_010239,NR_073181	Fth1	ferritin heavy polypeptide 1	protein_coding	NP_034369.1	95588	.	306.862152	[illegible]
14325	NM_010240	Ftl1	ferritin light polypeptide 1	protein_coding	NP_034370.2	95589	.	864.128052	[illegible]
14337	gene-Ftl2-ps	Ftl2-ps	ferritin light polypeptide 2, pseudogene	pseudogene	.	95590	.	19.135447	[illegible]
14339	NM_001302266,NM_001302267,NM_001302268,NM_001302337,NM_001358934,NM_001358935,NM_001358936,NM_001358937,NM_001358938,NM_010241,XM_006530673,XM_011248303,XM_011248304,XM_011248306,XM_011248307,XM_017312571,XM_017312574,XM_030243289,XM_030243290,XM_030243291,XM_030243292,XM_030243293,XM_030243294,XM_030243295,XM_030243296,XM_030243297	Aktip	thymoma viral proto-oncogene 1 interacting protein	protein_coding	NP_001289195.1;NP_001289196.1;NP_001289197.1;NP_001289266.1;NP_001345863.1;NP_001345864.1;NP_001345865.1;NP_001345866.1;NP_001345867.1;NP_034371.1;XP_006530736.1;XP_011246605.1;XP_011246606.1;XP_011246608.1;XP_011246609.1;XP_017168060.1;XP_017168063.1;XP_030099149.1;XP_030099150.1;XP_030099151.1;XP_030099152.1;XP_030099153.1;XP_030099154.1;XP_030099155.1;XP_030099156.1;XP_030099157.1	3693832	.	11.002375	[illegible]
14343	NM_001271981,NM_008051	Fut1	fucosyltransferase 1	protein_coding	NP_001258910.1;NP_032077.2	109375	.	0.000000	[illegible]
14344	NM_001271993,NM_018876,XM_011250795	Fut2	fucosyltransferase 2	protein_coding	NP_001258922.1;NP_061364.2;XP_011249097.1	109374	.	0.030380	[illegible]
14345	NM_010242	Fut4	fucosyltransferase 4	protein_coding	NP_034372.1	95594	.	0.682057	[illegible]
14347	NM_001177366,NM_001177367,NM_001289453,NM_001289454,NM_001289455,NM_001289456,NM_013524,XM_006497702,XM_006497704,XM_011239022,XM_030247513,XM_030247514	Fut7	fucosyltransferase 7	protein_coding	NP_001170837.1;NP_001170838.1;NP_001276382.1;NP_001276383.1;NP_001276384.1;NP_001276385.1;NP_038552.1;XP_006497765.1;XP_006497767.1;XP_011237324.1;XP_030103373.1;XP_030103374.	107692	.	0.021057	[illegible]
14348	NM_010243	Fut9	fucosyltransferase 9	protein_coding	NP_034373.1	1330859	.	0.007903	[illegible]
14349	NM_010244	Fv1	Friend virus susceptibility 1	protein_coding	NP_034374.2	95595	.	1.772330	[illegible]
14356	NM_001360052,NM_001360053,NM_001360054,NM_001360055,NM_001360056,NM_001360058,NM_001360059,NM_001360060,NM_001360061,NM_019502,NR_153392,NR_153393	Timm10b	translocase of inner mitochondrial membrane 10B	protein_coding	NP_001346981.1;NP_001346982.1;NP_001346983.1;NP_001346984.1;NP_001346985.1;NP_001346987.1;NP_001346988.1;NP_001346989.1;NP_001346990.1;NP_062375.1	1315196	.	22.424816	[illegible]
14357	NM_008052,XM_011248180,XM_011248181,XM_030254131	Dtx1	deltex 1, E3 ubiquitin ligase	protein_coding	NP_032078.2;XP_011246482.1;XP_011246483.1;XP_030109991.1	1352744	.	0.021768	[illegible]
14359	NM_001113188,NM_001113189,NM_008053,XM_006535400,XM_006535401,XM_006535402	Fxr1	fragile X mental retardation gene 1, autosomal homolog	protein_coding	NP_001106659.1;NP_001106660.1;NP_032079.1;XP_006535463.1;XP_006535464.1;XP_006535465.1	104860	.	39.485213	[illegible]
14360	NM_001122892,NM_001122893,NM_008054,XM_006512539,XM_006512540,XM_011243117,XM_030244881	Fyn	Fyn proto-oncogene	protein_coding	NP_001116364.1;NP_001116365.1;NP_032080.2;XP_006512602.1;XP_006512603.1;XP_011241419.1;XP_030100741.1	95602	.	26.164170	[illegible]
14362	NM_021457	Fzd1	frizzled class receptor 1	protein_coding	NP_067432.2	1196625	.	20.712170	[illegible]
14365	NM_021458	Fzd3	frizzled class receptor 3	protein_coding	NP_067433.1	108476	.	1.561271	[illegible]
14366	NM_008055	Fzd4	frizzled class receptor 4	protein_coding	NP_032081.3	108520	.	5.385020	[illegible]
14367	NM_001042659,NM_022721	Fzd5	frizzled class receptor 5	protein_coding	NP_001036124.1;NP_073558.2	108571	.	5.497710	[illegible]
14368	NM_001162494,NM_008056,XM_006520438,XM_011245454,XM_011245455,XM_017316430	Fzd6	frizzled class receptor 6	protein_coding	NP_001155966.1;NP_032082.3;XP_006520501.1;XP_011243756.1;XP_011243757.1;XP_017171919.1	108474	.	8.219869	[illegible]
14369	NM_008057	Fzd7	frizzled class receptor 7	protein_coding	NP_032083.3	108570	.	11.892928	[illegible]
14370	NM_008058	Fzd8	frizzled class receptor 8	protein_coding	NP_032084.2	108460	.	7.377032	[illegible]
14371	NM_010246	Fzd9	frizzled class receptor 9	protein_coding	NP_034376.1	1313278	.	13.991440	[illegible]
14373	NM_008059	G0s2	G0/G1 switch gene 2	protein_coding	NP_032085.1	1316737	.	2.913008	[illegible]
14375	NM_010247,XM_006520442,XM_006520443,XM_006520444,XM_006520445,XM_011245456,XR_383982	Xrcc6	X-ray repair complementing defective repair in Chinese hamster cells 6	protein_coding	NP_034377.2;XP_006520505.1;XP_006520506.1;XP_006520507.1;XP_006520508.1;XP_011243758.1	95606	.	11.522172	[illegible]
14376	NM_001293621,NM_008060,XM_006526677,XM_006526678	Ganab	alpha glucosidase 2 alpha neutral subunit	protein_coding	NP_001280550.1;NP_032086.1;XP_006526740.1;XP_006526741.1	1097667	.	63.385127	[illegible]
14377	NM_008061	G6pc	glucose-6-phosphatase, catalytic	protein_coding	NP_032087.2	95607	.	0.020973	[illegible]
14378	NM_001289856,NM_001289857,NM_021331	G6pc2	glucose-6-phosphatase, catalytic, 2	protein_coding	NP_001276785.1;NP_001276786.1;NP_067306.1	1277193	.	0.000000	[illegible]
14381	NM_008062	G6pdx	glucose-6-phosphate dehydrogenase X-linked	protein_coding	NP_032088.1	105979	.	13.289990	[illegible]
14385	NM_001293630,NM_001293631,NM_001357729,NM_001357730,NM_008063,XM_006510011,XM_006510013,XM_006510016,XM_030244053,XM_030244054,XM_030244055,XM_030244056,XM_030244057	Slc37a4	solute carrier family 37 (glucose-6-phosphate transporter), member 4	protein_coding	NP_001280559.1;NP_001280560.1;NP_001344658.1;NP_001344659.1;NP_032089.2;XP_006510074.1;XP_006510076.1;XP_006510079.1;XP_030099913.1;XP_030099914.1;XP_030099915.1;XP_030099916.1;XP_030099917.1	1316650	.	6.084277	[illegible]
14387	NM_001159324,NM_008064	Gaa	glucosidase, alpha, acid	protein_coding	NP_001152796.1;NP_032090.3	95609	.	37.634784	[illegible]
14388	NM_001301298,NM_021356,XM_006530674,XM_006530676,XM_006530677,XM_011248309,XM_011248310,XM_017312575,XM_017312576,XM_030243298	Gab1	growth factor receptor bound protein 2-associated protein 1	protein_coding	NP_001288227.1;NP_067331.2;XP_006530737.1;XP_006530739.1;XP_006530740.1;XP_011246611.1;XP_011246612.1;XP_017168064.1;XP_017168065.1;XP_030099158.1	108088	.	11.153231	[illegible]
14389	NM_001162477,NM_010248	Gab2	growth factor receptor bound protein 2-associated protein 2	protein_coding	NP_001155949.1;NP_034378.2	1333854	.	2.976371	[illegible]
14390	NM_008065,XM_006522905,XM_006522906,XM_006522907,XM_017316875,XM_017316876,XM_030248977,XR_876129	Gabpa	GA repeat binding protein, alpha	protein_coding	NP_032091.2;XP_006522968.1;XP_006522969.1;XP_006522970.1;XP_017172364.1;XP_017172365.1;XP_030104837.1	95610	.	14.948183	[illegible]
14391	NM_001271467,NM_001271468,NM_001271469,NM_001271470,NM_001271492,NM_001355108,NM_001355109,NM_010249,NM_207669,NR_073183,XM_006498755,XM_006498756,XM_006498757,XM_006498759,XM_006498760,XM_006498761,XM_006498762,XM_006498764,XM_030247518,XM_030247522,XM_030247523,XM_030247525,XM_030247526,XM_030247527,XM_030247528,XM_030247529,XM_030247530	Gabpb1	GA repeat binding protein, beta 1	protein_coding	NP_001258396.1;NP_001258397.1;NP_001258398.1;NP_001258399.1;NP_001258421.1;NP_001342037.1;NP_001342038.1;NP_034379.1;NP_997552.1;XP_006498818.1;XP_006498819.1;XP_006498820.1;XP_006498822.1;XP_006498823.1;XP_006498824.1;XP_006498825.1;XP_006498827.1;XP_030103378.1;XP_030103382.1;XP_030103383.1;XP_030103385.1;XP_030103386.1;XP_030103387.1;XP_030103388.1;XP_030103389.1;XP_030103390.1	95611	.	7.354208	[illegible]
14394	NM_001359035,NM_010250,XM_006532195,XM_017314259	Gabra1	gamma-aminobutyric acid (GABA) A receptor, subunit alpha 1	protein_coding	NP_001345964.1;NP_034380.1;XP_006532258.1;XP_017169748.1	95613	.	0.208277	[illegible]
14395	NM_008066,XM_006503733,XM_030254132	Gabra2	gamma-aminobutyric acid (GABA) A receptor, subunit alpha 2	protein_coding	NP_032092.1;XP_006503796.1;XP_030109992.1	95614	.	0.016001	[illegible]
14396	NM_001357814,NM_001357815,NM_001357816,NM_001358103,NM_008067	Gabra3	gamma-aminobutyric acid (GABA) A receptor, subunit alpha 3	protein_coding	NP_001344743.1;NP_001344744.1;NP_001344745.1;NP_001345032.1;NP_032093.3	95615	.	0.792047	[illegible]
14397	NM_001359041,NM_001359043,NM_001359044,NM_001359045,NM_001359046,NM_001359047,NM_001359048,NM_001359050,NM_010251	Gabra4	gamma-aminobutyric acid (GABA) A receptor, subunit alpha 4	protein_coding	NP_001345970.1;NP_001345972.1;NP_001345973.1;NP_001345974.1;NP_001345975.1;NP_001345976.1;NP_001345977.1;NP_001345979.1;NP_034381.1	95616	.	0.279821	[illegible]
14399	NM_001099641,NM_001359049,NM_008068,XM_006532198,XM_030245559	Gabra6	gamma-aminobutyric acid (GABA) A receptor, subunit alpha 6	protein_coding	NP_001093111.1;NP_001345978.1;NP_032094.2;XP_006532261.1;XP_030101419.1	95618	.	0.000000	[illegible]
14400	NM_001370968,NM_008069	Gabrb1	gamma-aminobutyric acid (GABA) A receptor, subunit beta 1	protein_coding	NP_001357897.1;NP_032095.1	95619	.	0.025782	[illegible]
14401	NM_001347314,NM_001362646,NM_001362647,NM_001362649,NM_008070,XM_030245560	Gabrb2	gamma-aminobutyric acid (GABA) A receptor, subunit beta 2	protein_coding	NP_001334243.1;NP_001349575.1;NP_001349576.1;NP_001349578.1;NP_032096.1;XP_030101420.1	95620	.	0.326239	[illegible]
14402	NM_001038701,NM_008071	Gabrb3	gamma-aminobutyric acid (GABA) A receptor, subunit beta 3	protein_coding	NP_001033790.1;NP_032097.1	95621	.	0.816052	[illegible]
14403	NM_008072,XM_006538546	Gabrd	gamma-aminobutyric acid (GABA) A receptor, subunit delta	protein_coding	NP_032098.2;XP_006538609.2	95622	.	0.023583	[illegible]
14404	NM_017369,XM_011247523,XM_011247524,XM_011247525,XM_011247526,XR_878092	Gabre	gamma-aminobutyric acid (GABA) A receptor, subunit epsilon	protein_coding	NP_059065.2;XP_011245825.1;XP_011245826.1;XP_011245827.1;XP_011245828.1	1330235	.	0.924887	[illegible]
14405	NM_010252,XM_006503737,XM_006503738,XM_011240701	Gabrg1	gamma-aminobutyric acid (GABA) A receptor, subunit gamma 1	protein_coding	NP_034382.2;XP_006503800.1;XP_006503801.1;XP_011239003.1	103156	.	0.000000	[illegible]
14406	NM_001362655,NM_001362656,NM_008073,NM_177408,XM_030245561	Gabrg2	gamma-aminobutyric acid (GABA) A receptor, subunit gamma 2	protein_coding	NP_001349584.1;NP_001349585.1;NP_032099.1;NP_803127.3;XP_030101421.1	95623	.	0.000000	[illegible]
14407	NM_008074,XM_017321978	Gabrg3	gamma-aminobutyric acid (GABA) A receptor, subunit gamma 3	protein_coding	NP_032100.2;XP_017177467.1	95624	.	0.009002	[illegible]
14408	NM_008075,XM_006537612,XM_006537613	Gabrr1	gamma-aminobutyric acid (GABA) C receptor, subunit rho 1	protein_coding	NP_032101.3;XP_006537675.1;XP_006537676.1	95625	.	0.000000	[illegible]
14409	NM_008076,XM_006537616,XM_006537617,XM_030253210	Gabrr2	gamma-aminobutyric acid (GABA) C receptor, subunit rho 2	protein_coding	NP_032102.2;XP_006537679.1;XP_006537680.1;XP_030109070.1	95626	.	0.054184	[illegible]
14411	NM_001347433,NM_133661,XM_006505533,XM_006505534,XM_006505535,XM_006505536,XM_030255158	Slc6a12	solute carrier family 6 (neurotransmitter transporter, betaine/GABA), member 12	protein_coding	NP_001334362.1;NP_598422.2;XP_006505596.1;XP_006505597.1;XP_006505598.1;XP_006505599.1;XP_030111018.1	95628	.	0.224202	[illegible]
14412	NM_144512,XM_006505537,XM_006505538,XM_011241211,XM_030255160	Slc6a13	solute carrier family 6 (neurotransmitter transporter, GABA), member 13	protein_coding	NP_653095.1;XP_006505600.1;XP_006505601.1;XP_011239513.1;XP_030111020.1	95629	.	0.340947	[illegible]
14415	NM_001312900,NM_008077,XM_006498765,XM_006498766,XM_030247531,XR_001780815,XR_003950660,XR_003950661	Gad1	glutamate decarboxylase 1	protein_coding	NP_001299829.1;NP_032103.2;XP_006498828.1;XP_006498829.1;XP_030103391.1	95632	.	0.039221	[illegible]
14417	NM_008078,XM_011239023	Gad2	glutamic acid decarboxylase 2	protein_coding	NP_032104.2;XP_011237325.1	95634	.	0.015136	[illegible]
14419	NM_001329667,NM_010253	Gal	galanin and GMAP prepropeptide	protein_coding	NP_001316596.1;NP_034383.1	95637	.	1.731095	[illegible]
14420	NM_008079,XM_006515472,XM_017314960,XM_030246561	Galc	galactosylceramidase	protein_coding	NP_032105.2;XP_006515535.1;XP_017170449.1;XP_030102421.1	95636	.	4.597053	[illegible]
14421	NM_001244617,NM_001244618,NM_001358577,NM_001358578,NM_008080,NM_027739,NR_045166,XM_006513219,XM_01731380	B4galnt1	beta-1,4-N-acetyl-galactosaminyl transferase 1	protein_coding	NP_001231546.1;NP_001231547.1;NP_001345506.1;NP_001345507.1;NP_032106.1;NP_082015.2;XP_006513282.1;XP_017169292.1	1342057	.	5.908855	[illegible]
14422	NM_008081,XM_006532201	B4galnt2	beta-1,4-N-acetyl-galactosaminyl transferase 2	protein_coding	NP_032107.1;XP_006532264.1	1342058	.	0.000000	[illegible]
14423	NM_001160404,NM_001361200,NM_013814,XM_030250335,XM_030250336	Galnt1	polypeptide N-acetylgalactosaminyltransferase 1	protein_coding	NP_001153876.1;NP_001348129.1;NP_038842.3;XP_030106195.1;XP_030106196.1	894693	.	54.622099	[illegible]
14425	NM_015736,XR_374409	Galnt3	polypeptide N-acetylgalactosaminyltransferase 3	protein_coding	NP_056551.2	894695	.	5.479523	[illegible]
14426	NM_015737	Galnt4	polypeptide N-acetylgalactosaminyltransferase 4	protein_coding	NP_056552.1	894692	.	5.555226	[illegible]
14427	NM_008082	Galr1	galanin receptor 1	protein_coding	NP_032108.1	1096364	.	0.016502	[illegible]
14428	NM_010254,XM_011248727,XM_011248728,XM_011248729	Galr2	galanin receptor 2	protein_coding	NP_034384.3;XP_011247029.1;XP_011247030.1;XP_011247031.1	1337018	.	1.399032	[illegible]
14429	NM_015738	Galr3	galanin receptor 3	protein_coding	NP_056553.2	1329003	.	0.000000	[illegible]
14430	NM_001302511,NM_001369135,NM_001369140,NM_001369141,NM_001369142,NM_001369143,NM_001369144,NM_001369147,NM_016658	Galt	galactose-1-phosphate uridyl transferase	protein_coding	NP_001289440.1;NP_001356064.1;NP_001356069.1;NP_001356070.1;NP_001356071.1;NP_001356072.1;NP_001356073.1;NP_001356076.1;NP_057867.2	95638	.	10.245990	[illegible]
14431	NM_001347119,NM_010255,XM_006513222	Gamt	guanidinoacetate methyltransferase	protein_coding	NP_001334048.1;NP_034385.1;XP_006513285.1	1098221	.	10.181336	[illegible]
14432	NM_008083	Gap43	growth associated protein 43	protein_coding	NP_032109.1	95639	.	5.396281	[illegible]
14433	NM_001289726,NM_008084,XM_017321385	Gapdh	glyceraldehyde-3-phosphate dehydrogenase	protein_coding	NP_001276655.1;NP_032110.1;XP_017176874.1	95640	.	195.448289	[illegible]
14447	NM_001290631,NM_008085,XM_006539545,XM_011250435	Gapdhs	glyceraldehyde-3-phosphate dehydrogenase, spermatogenic	protein_coding	NP_001277560.1;NP_032111.1;XP_006539608.1;XP_011248737.1	95653	.	0.000000	[illegible]
14450	NM_001357351,NM_010256,XM_006522911,XM_006522912	Gart	phosphoribosylglycinamide formyltransferase	protein_coding	NP_001344280.1;NP_034386.2;XP_006522974.1;XP_006522975.1	95654	.	14.923783	[illegible]
14451	NM_008086	Gas1	growth arrest specific 1	protein_coding	NP_032112.1	95655	.	26.800011	[illegible]
14453	NM_001330607,NM_001330608,NM_008087,XM_006540618,XM_006540619,XM_006540620,XM_006540621,XM_006540622,XM_006540623,XM_006540624,XM_006540625,XM_006540626,XM_011250796,XM_011250797,XM_017321979,XM_017321980,XM_017321982,XM_030242134,XM_030242135,XM_030242136,XM_030242137,XM_030242138,XM_030242139,XM_030242140,XM_030242141	Gas2	growth arrest specific 2	protein_coding	NP_001317536.1;NP_001317537.1;NP_032113.1;XP_006540681.1;XP_006540682.1;XP_006540683.1;XP_006540684.1;XP_006540685.1;XP_006540686.1;XP_006540687.1;XP_006540688.1;XP_006540689.1;XP_011249098.1;XP_011249099.1;XP_017177468.1;XP_017177469.1;XP_017177471.1;XP_030097994.1;XP_030097995.1;XP_030097996.1;XP_030097997.1;XP_030097998.1;XP_030097999.1;XP_030098000.1;XP_030098001.1	95657	.	4.123117	[illegible]
14455	NR_153812,NR_153813,NR_153814,NR_153815	Gas5	growth arrest specific 5	lncRNA	.	95659	.	64.485384	[illegible]

14456	NM_019521	Gas6	growth arrest specific 6	protein_coding	NP_062394.2	95660	.	54.574066	[illegible]
14457	NM_001109657,NM_001277079,NM_001277080,NM_008088,XM_006532202,XM_006532205,XM_006532208,XM_006532209,XM_006532210,XM_006532211,XM_017314262,XM_030245562,XM_030245563,XM_030245564	Gas7	growth arrest specific 7	protein_coding	NP_001103127.1;NP_001264008.1;NP_001264009.1;NP_032114.2;XP_006532265.1;XP_006532268.1;XP_006532271.1;XP_006532272.1;XP_006532273.1;XP_006532274.1;XP_017169751.1;XP_030101422.1;XP_030101423.1;XP_030101424.1	1202388	.	3.765221	[illegible]
14459	NM_010257	Gast	gastrin	protein_coding	NP_034387.3	104768	.	0.102772	[illegible]
14460	NM_008089,XM_011247448	Gata1	GATA binding protein 1	protein_coding	NP_032115.1;XP_011245750.1	95661	.	0.241383	[illegible]
14461	NM_001355253,NM_008090	Gata2	GATA binding protein 2	protein_coding	NP_001342182.1;NP_032116.4	95662	.	1.335225	[illegible]
14462	NM_001355110,NM_001355111,NM_001355112,NM_008091,XM_006497354,XM_030247532,XM_030247533	Gata3	GATA binding protein 3	protein_coding	NP_001342039.1;NP_001342040.1;NP_001342041.1;NP_032117.1;XP_006497417.1;XP_030103392.1;XP_030103393.1	95663	.	0.027985	[illegible]
14465	NM_010258,XM_017317834	Gata6	GATA binding protein 6	protein_coding	NP_034388.2;XP_017173323.1	107516	.	0.294905	[illegible]
14466	NM_001077411,NM_008094,NR_122037,XM_006500997,XR_003954280,XR_003954281	Gba	glucosidase, beta, acid	protein_coding	NP_001070879.1;NP_032120.1;XP_006501060.1	95665	.	29.891528	[illegible]
14467	NM_008095,XR_377051,XR_377052,XR_377054	Nipsnap2	nipsnap homolog 2	protein_coding	NP_032121.3	1278343	.	38.343929	[illegible]
14469	NM_010260	Gbp2	guanylate binding protein 2	protein_coding	NP_034390.1	102772	.	1.765140	[illegible]
14470	NM_010261	Rabac1	Rab acceptor 1 (prenylated)	protein_coding	NP_034391.1	1201692	.	88.015701	[illegible]
14472	NM_010262	Gbx2	gastrulation brain homeobox 2	protein_coding	NP_034392.1	95668	.	0.000000	[illegible]
14473	NM_008096	Gc	vitamin D binding protein	protein_coding	NP_032122.1	95669	.	0.000000	[illegible]
14479	NM_001301628,NM_027604,XM_006513225,XM_006513226,XM_006513227,XM_006513228,XR_380365	Usp15	ubiquitin specific peptidase 15	protein_coding	NP_001288557.1;NP_081880.2;XP_006513288.1;XP_006513289.1;XP_006513290.1;XP_006513291.1	101857	.	6.474625	[illegible]
14489	NM_008098	Mtpn	myotrophin	protein_coding	NP_032124.1	99445	.	44.478439	[illegible]
14525	NM_001159297,NM_008099	Gcsam	germinal center associated, signaling and motility	protein_coding	NP_001152769.1;NP_032125.3	102969	.	0.215858	[illegible]
14526	NM_008100	Gcg	glucagon	protein_coding	NP_032126.1	95674	.	0.048155	[illegible]
14527	NM_008101,XM_006532216,XM_006532217,XM_017314263,XM_017314265,XM_017314266,XM_017314267,XM_017314268,XM_017314269,XM_030245565,XM_030245566,XM_030245567	Gcgr	glucagon receptor	protein_coding	NP_032127.2;XP_006532279.1;XP_006532280.1;XP_017169752.1;XP_017169754.1;XP_017169755.1;XP_017169756.1;XP_017169757.1;XP_017169758.1;XP_030101425.1;XP_030101426.1;XP_030101427.	99572	.	0.162087	[illegible]
14528	NM_008102	Gch1	GTP cyclohydrolase 1	protein_coding	NP_032128.1	95675	.	0.506613	[illegible]
14531	NM_008103	Gcm1	glial cells missing homolog 1	protein_coding	NP_032129.2	108045	.	0.000000	[illegible]
14533	NM_015740	Bloc1s1	biogenesis of lysosomal organelles complex-1, subunit 1	protein_coding	NP_056555.2	1195276	.	39.507298	[illegible]
14534	NM_001038010,NM_020004,XM_017314270,XM_017314271,XM_017314272,XM_017314273,XM_030245568,XM_030245569	Kat2a	K(lysine) acetyltransferase 2A	protein_coding	NP_001033099.1;NP_064388.2;XP_017169759.1;XP_017169760.1;XP_017169761.1;XP_017169762.1;XP_030101428.1;XP_030101429.1	1343101	.	14.403208	[illegible]
14536	NM_001159548,NM_001159549,NM_010264,XM_011239025	Nr6a1	nuclear receptor subfamily 6, group A, member 1	protein_coding	NP_001153020.1;NP_001153021.1;NP_034394.1;XP_011237327.1	1352459	.	0.638728	[illegible]
14537	NM_001136484,NM_010265,NM_173442,XM_006526679,XM_011247144,XM_017318062	Gcnt1	glucosaminyl (N-acetyl) transferase 1, core 2	protein_coding	NP_001129956.3;NP_034395.4;NP_775618.3;XP_006526742.1;XP_011245446.1;XP_017173551.1	95676	.	4.096509	[illegible]
14538	NM_008105,NM_023887,NM_133219,XR_003950428,XR_003950429,XR_003950430	Gcnt2	glucosaminyl (N-acetyl) transferase 2, I-branching enzyme	protein_coding	NP_032131.2;NP_076376.3;NP_573482.1	1100870	.	1.788589	[illegible]
14544	NM_010266,XM_006526680,XM_017318064	Gda	guanine deaminase	protein_coding	NP_034396.1;XP_006526743.1;XP_017173553.1	95678	.	0.885956	[illegible]
14545	NM_010267	Gdap1	ganglioside-induced differentiation-associated-protein 1	protein_coding	NP_034397.1	1338002	.	0.106968	[illegible]
14547	NM_010269,NR_104341,XM_006501001,XR_001783651	Gdap2	ganglioside-induced differentiation-associated-protein 2	protein_coding	NP_034399.1;XP_006501064.1	1338001	.	6.359571	[illegible]
14548	NM_001010930,NM_010270	Mrps33	mitochondrial ribosomal protein S33	protein_coding	NP_001010930.1;NP_034400.1	1338046	.	14.555958	[illegible]
14555	NM_010271	Gpd1	glycerol-3-phosphate dehydrogenase 1 (soluble)	protein_coding	NP_034401.1	95679	.	1.592994	[illegible]
14559	NM_001163282,NM_008107	Gdf1	growth differentiation factor 1	protein_coding	NP_001156754.1;NP_032133.2	95683	.	1.345987	[illegible]
14560	NM_145741	Gdf10	growth differentiation factor 10	protein_coding	NP_665684.2	95684	.	29.749041	[illegible]
14561	NM_010272	Gdf11	growth differentiation factor 11	protein_coding	NP_034402.1	1338027	.	2.358821	[illegible]
14563	NM_008109	Gdf5	growth differentiation factor 5	protein_coding	NP_032135.2	95688	.	3.959027	[illegible]
14566	NM_008110,XM_006532220,XM_006532221,XM_030245570	Gdf9	growth differentiation factor 9	protein_coding	NP_032136.2;XP_006532283.1;XP_006532284.1;XP_030101430.1	95692	.	0.861386	[illegible]
14567	NM_010273	Gdi1	guanosine diphosphate (GDP) dissociation inhibitor 1	protein_coding	NP_034403.1	99846	.	47.575481	[illegible]
14569	NM_008112	Gdi2	guanosine diphosphate (GDP) dissociation inhibitor 2	protein_coding	NP_032138.3	99845	.	116.667336	[illegible]
14570	NM_008113	Arhgdig	Rho GDP dissociation inhibitor (GDI) gamma	protein_coding	NP_032139.1	108430	.	0.922945	[illegible]
14571	NM_001145820,NM_010274	Gpd2	glycerol phosphate dehydrogenase 2, mitochondrial	protein_coding	NP_001139292.1;NP_034404.3	99778	.	4.192746	[illegible]
14573	NM_001301332,NM_001301333,NM_001301357,NM_010275	Gdnf	glial cell line derived neurotrophic factor	protein_coding	NP_001288261.1;NP_001288262.1;NP_001288286.1;NP_034405.1	107430	.	0.494842	[illegible]
14579	NM_010276	Gem	GTP binding protein (gene overexpressed in skeletal muscle)	protein_coding	NP_034406.2	99844	.	2.379204	[illegible]
14580	NM_001131020,NM_010277,XM_030245571,XR_388347	Gfap	glial fibrillary acidic protein	protein_coding	NP_001124492.1;NP_034407.2;XP_030101431.1	95697	.	0.150659	[illegible]
14581	NM_001267621,NM_010278	Gfi1	growth factor independent 1 transcription repressor	protein_coding	NP_001254550.1;NP_034408.1	103170	.	0.900705	[illegible]
14582	NM_001160406,NM_008114	Gfi1b	growth factor independent 1B	protein_coding	NP_001153878.1;NP_032140.1	1276578	.	0.046342	[illegible]
14583	NM_013528	Gfpt1	glutamine fructose-6-phosphate transaminase 1	protein_coding	NP_038556.1	95698	.	25.518909	[illegible]
14584	NM_013529	Gfpt2	glutamine fructose-6-phosphate transaminase 2	protein_coding	NP_038557.1	1338883	.	12.406651	[illegible]
14585	NM_001285457,NM_010279,NR_104342,XM_006526681,XM_006526682,XM_006526683,XM_006526684,XM_030250753	Gfra1	glial cell line derived neurotrophic factor family receptor alpha 1	protein_coding	NP_001272386.1;NP_034409.1;XP_006526744.1;XP_006526745.1;XP_006526746.1;XP_006526747.1;XP_030106613.1	1100842	.	2.770139	[illegible]
14586	NM_001302094,NM_001302095,NM_008115	Gfra2	glial cell line derived neurotrophic factor family receptor alpha 2	protein_coding	NP_001289023.1;NP_001289024.1;NP_032141.2	1195462	.	2.701449	[illegible]
14587	NM_010280	Gfra3	glial cell line derived neurotrophic factor family receptor alpha 3	protein_coding	NP_034410.3	1201403	.	0.621768	[illegible]
14588	NM_001136063,NM_001271001,NM_001271002,NM_020014,XM_017315629,XM_030247541,XR_001780825,XR_003950726,XR_003950732,XR_003950738,XR_866224,XR_866225,XR_866226	Gfra4	glial cell line derived neurotrophic factor family receptor alpha 4	protein_coding	NP_001129535.1;NP_001257930.1;NP_001257931.1;NP_064398.1;XP_017171118.1;XP_030103401.1	1341873	.	9.493762	[illegible]
14590	NM_010281,XM_006537619	Ggh	gamma-glutamyl hydrolase	protein_coding	NP_034411.2;XP_006537682.1	1329035	.	18.598059	[illegible]
14593	NM_001331119,NM_001331176,NM_001331177,NM_001331178,NM_001331180,NM_010282,NR_138552,NR_138553,NR_138555,XM_006516549,XM_011244281,XM_017315385,XM_017315387,XM_017315388,XM_030247152,XM_030247153,XM_030247154,XM_030247155,XR_001780754,XR_003950431,XR_003950432	Ggps1	geranylgeranyl diphosphate synthase 1	protein_coding	NP_001318048.1;NP_001318105.1;NP_001318106.1;NP_001318107.1;NP_001318109.1;NP_034412.1;XP_006516612.1;XP_011242583.1;XP_017170874.1;XP_017170876.1;XP_017170877.1;XP_030103012.1;XP_030103013.1;XP_030103014.1;XP_030103015.1	1341724	.	8.904107	[illegible]
14594	NM_001145821,NM_001308299,NM_001308300,NM_010283,XM_006497720,XM_011239026,XM_011239027,XM_011239028,XM_011239029,XM_030247542,XM_030247543,XM_030247544,XM_030247545	Ggta1	glycoprotein galactosyltransferase alpha 1, 3	protein_coding	NP_001139293.1;NP_001295228.1;NP_001295229.1;NP_034413.2;XP_006497783.1;XP_011237328.1;XP_011237329.1;XP_011237330.1;XP_011237331.1;XP_030103402.1;XP_030103403.1;XP_030103404.1;XP_030103405.1	95704	.	4.844109	[illegible]
14595	NM_022305,XR_003954879	B4galt1	UDP-Gal:betaGlcNAc beta 1,4- galactosyltransferase, polypeptide 1	protein_coding	NP_071641.1	95705	.	34.464441	[illegible]
14598	NM_001305992,NM_008116,XM_006513230,XM_006513231,XM_030244882,XM_030244883,XM_030244884,XR_003948591,XR_003948592	Ggt1	gamma-glutamyltransferase 1	protein_coding	NP_001292921.1;NP_032142.1;XP_006513293.1;XP_006513294.1;XP_030100742.1;XP_030100743.1;XP_030100744.1	95706	.	0.105549	[illegible]
14599	NM_008117,XM_006532223	Gh	growth hormone	protein_coding	NP_032143.1;XP_006532286.1	95707	.	0.000000	[illegible]
14600	NM_001048178,NM_001286370,NM_010284	Ghr	growth hormone receptor	protein_coding	NP_001041643.1;NP_001273299.1;NP_034414.2	95708	.	19.080914	[illegible]
14602	NM_001003685	Ghrhr	growth hormone releasing hormone receptor	protein_coding	NP_001003685.2	95710	.	0.000000	[illegible]
14605	NM_001077364,NM_010286,NR_122039,XM_006528499,XM_006528500,XM_006528503,XM_006528504,XM_006528505	Tsc22d3	TSC22 domain family, member 3	protein_coding	NP_001070832.1;NP_034416.3;XP_006528562.1;XP_006528563.1;XP_006528566.1;XP_006528567.1;XP_006528568.1	1196284	.	43.221098	[illegible]
14608	NM_001310734,NM_010287,XM_006510017,XM_030244058	Gpr83	G protein-coupled receptor 83	protein_coding	NP_001297663.1;NP_034417.1;XP_006510080.1;XP_030099918.1	95712	.	0.000000	[illegible]
14609	NM_010288	Gja1	gap junction protein, alpha 1	protein_coding	NP_034418.1	95713	.	156.161072	[illegible]
14610	NM_010289	Gja10	gap junction protein, alpha 10	protein_coding	NP_034419.2	1339969	.	0.000000	[illegible]
14611	NM_001271623,NM_016975,XM_011244959	Gja3	gap junction protein, alpha 3	protein_coding	NP_001258552.1;NP_058671.2;XP_011243261.1	95714	.	0.351206	[illegible]
14612	NM_008120	Gja4	gap junction protein, alpha 4	protein_coding	NP_032146.1	95715	.	3.051008	[illegible]
14613	NM_001271628,NM_008121	Gja5	gap junction protein, alpha 5	protein_coding	NP_001258557.1;NP_032147.1	95716	.	1.605227	[illegible]
14615	NM_001159382,NM_001159383,NM_001359040,NM_008122,XM_006532227,XM_011248736,XM_030245572,XM_030245573	Gjc1	gap junction protein, gamma 1	protein_coding	NP_001152854.1;NP_001152855.1;NP_001345969.1;NP_032148.1;XP_006532290.1;XP_011247038.1;XP_030101432.1;XP_030101433.1	95718	.	13.425799	[illegible]
14616	NM_008123	Gja8	gap junction protein, alpha 8	protein_coding	NP_032149.1	99953	.	0.000000	[illegible]
14617	NM_010290,XM_017315645,XM_030247546	Gjd2	gap junction protein, delta 2	protein_coding	NP_034420.2;XP_017171134.1;XP_030103406.1	1334209	.	0.000000	[illegible]
14618	NM_001302496,NM_001302497,NM_001302498,NM_008124,XM_030251227	Gjb1	gap junction protein, beta 1	protein_coding	NP_001289425.1;NP_001289426.1;NP_001289427.1;NP_032150.2;XP_030107087.1	95719	.	0.000000	[illegible]
14619	NM_008125	Gjb2	gap junction protein, beta 2	protein_coding	NP_032151.1	95720	.	0.696312	[illegible]
14620	NM_001160012,NM_008126	Gjb3	gap junction protein, beta 3	protein_coding	NP_001153484.1;NP_032152.1	95721	.	0.103239	[illegible]
14621	NM_008127,XM_017319975,XM_017319976,XM_030253211	Gjb4	gap junction protein, beta 4	protein_coding	NP_032153.1;XP_017175464.1;XP_017175465.1;XP_030109071.1	95722	.	0.000000	[illegible]
14622	NM_010291	Gjb5	gap junction protein, beta 5	protein_coding	NP_034421.1	95723	.	0.232478	[illegible]
14623	NM_001010937,NM_001271663,XM_006518560,XM_006518561,XM_030247651	Gjb6	gap junction protein, beta 6	protein_coding	NP_001010937.1;NP_001258592.1;XP_006518623.1;XP_006518624.1;XP_030103511.1	107588	.	1.149721	[illegible]
14625	NM_010293	Gykl1	glycerol kinase-like 1	protein_coding	NP_034423.2	891990	.	0.000000	[illegible]
14626	NM_010294	Gk2	glycerol kinase 2	protein_coding	NP_034424.1	1329027	.	0.000000	[illegible]
14628	NM_172416	Ostm1	osteopetrosis associated transmembrane protein 1	protein_coding	NP_766004.1	2655574	.	5.757725	[illegible]
14629	NM_010295,NR_151778,XM_006510812	Gclc	glutamate-cysteine ligase, catalytic subunit	protein_coding	NP_034425.1;XP_006510875.1	104990	.	6.966959	[illegible]
14630	NM_008129,XM_030252423	Gclm	glutamate-cysteine ligase, modifier subunit	protein_coding	NP_032155.1;XP_030108283.1	104995	.	7.412657	[illegible]
14632	NM_010296	Gli1	GLI-Kruppel family member GLI1	protein_coding	NP_034426.2	95727	.	28.283546	[illegible]
14633	NM_001081125,XM_006529141,XM_011247924,XM_030248278	Gli2	GLI-Kruppel family member GLI2	protein_coding	NP_001074594.1;XP_006529204.1;XP_011246226.1;XP_030104138.	95728	.	5.128092	[illegible]
14634	NM_008130,XM_006516552,XM_017315389	Gli3	GLI-Kruppel family member GLI3	protein_coding	NP_032156.2;XP_006516615.1;XP_017170878.1	95729	.	5.706041	[illegible]
14635	NM_016905	Galk1	galactokinase 1	protein_coding	NP_058601.2	95730	.	30.430878	[illegible]
14645	NM_008131	Glul	glutamate-ammonia ligase (glutamine synthetase)	protein_coding	NP_032157.2	95739	.	44.196701	[illegible]
14651	NM_001159626,NM_024284,XM_006523660,XM_006523661	Hagh	hydroxyacyl glutathione hydrolase	protein_coding	NP_001153098.1;NP_077246.2;XP_006523723.1;XP_006523724.1	95745	.	10.926934	[illegible]
14652	NM_021332	Glp1r	glucagon-like peptide 1 receptor	protein_coding	NP_067307.2	99571	.	0.000000	[illegible]
14658	NM_001281969,NM_001345955,NM_001345956,NM_001345957,NM_001345958,NM_001345959,NM_001345996,NM_010298,XM_030252424,XM_030252425,XM_030252426,XM_030252427,XM_030252428,XM_030252429	Glrb	glycine receptor, beta subunit	protein_coding	NP_001268898.1;NP_001332884.1;NP_001332885.1;NP_001332886.1;NP_001332887.1;NP_001332888.1;NP_001332925.1;NP_034428.2;XP_030108284.1;XP_030108285.1;XP_030108286.1;XP_030108287.1;XP_030108288.1;XP_030108289.1	95751	.	1.620866	[illegible]
14659	NM_008132,XM_030248367,XM_030248384	Glrp1	glutamine repeat protein 1	protein_coding	NP_032158.2;XP_030104227.1;XP_030104244.1	108038	.	0.000000	[illegible]
14660	NM_001081081,NM_001113383,XM_006495701,XM_006495702,XM_006495703,XM_006495704,XM_017316236,XM_030248503,XR_001781161,XR_001781162,XR_373241	Gls	glutaminase	protein_coding	NP_001074550.1;NP_001106854.1;XP_006495764.1;XP_006495765.1;XP_006495766.1;XP_006495767.1;XP_017171725.1;XP_030104363.1	95752	.	21.421573	[illegible]
14661	NM_008133	Glud1	glutamate dehydrogenase 1	protein_coding	NP_032159.1	95753	.	41.929455	[illegible]
14664	NM_001355175,NM_001369016,NM_001369017,NM_008135,XM_030253212,XR_867807	Slc6a9	solute carrier family 6 (neurotransmitter transporter, glycine), member 9	protein_coding	NP_001342104.1;NP_001355945.1;NP_001355946.1;NP_032161.2;XP_030109072.1	95760	.	5.618391	[illegible]
14667	NM_010299	Gm2a	GM2 ganglioside activator protein	protein_coding	NP_034429.1	95762	.	12.886922	[illegible]
14670	NM_008136	Gnl1	guanine nucleotide binding protein-like 1	protein_coding	NP_032162.2	95764	.	14.811677	[illegible]
14672	NM_010301	Gna11	guanine nucleotide binding protein, alpha 11	protein_coding	NP_034431.1	95766	.	32.037159	[illegible]
14673	NM_010302	Gna12	guanine nucleotide binding protein, alpha 12	protein_coding	NP_034432.1	95767	.	14.888495	[illegible]
14674	NM_001359034,NM_010303	Gna13	guanine nucleotide binding protein, alpha 13	protein_coding	NP_001345963.1;NP_034433.3	95768	.	13.383860	[illegible]
14675	NM_008137	Gna14	guanine nucleotide binding protein, alpha 14	protein_coding	NP_032163.3	95769	.	0.371054	[illegible]
14676	NM_010304,XM_006513233,XM_006513234,XM_030244885	Gna15	guanine nucleotide binding protein, alpha 15	protein_coding	NP_034434.1;XP_006513296.1;XP_006513297.1;XP_030100745.1	95770	.	0.245209	[illegible]
14677	NM_010305	Gnai1	guanine nucleotide binding protein (G protein), alpha inhibiting 1	protein_coding	NP_034435.1	95771	.	9.074974	[illegible]
14678	NM_008138,XM_006511637	Gnai2	guanine nucleotide binding protein (G protein), alpha inhibiting 2	protein_coding	NP_032164.2;XP_006511700.1	95772	.	123.020298	[illegible]
14679	NM_010306	Gnai3	guanine nucleotide binding protein (G protein), alpha inhibiting 3	protein_coding	NP_034436.1	95773	.	47.586304	[illegible]
14680	NM_010307,NM_177137,XM_030250337	Gnal	guanine nucleotide binding protein, alpha stimulating, olfactory type	protein_coding	NP_034437.1;NP_796111.2;XP_030106197.1	95774	.	0.267458	[illegible]
14681	NM_001113384,NM_010308,XM_011248311	Gnao1	guanine nucleotide binding protein, alpha O	protein_coding	NP_001106855.1;NP_034438.1;XP_011246613.1	95775	.	3.089552	[illegible]
14682	NM_008139	Gnaq	guanine nucleotide binding protein, alpha q polypeptide	protein_coding	NP_032165.3	95776	.	10.535569	[illegible]
14683	NM_001077507,NM_001077510,NM_001310083,NM_001310085,NM_001364030,NM_010309,NM_019690,NM_022000,NM_201616,NM_201617,NM_201618,NR_003258,XM_006498774,XM_006498777,XM_006498778,XM_006498779,XM_006498780,XM_017315658,XM_030247568,XM_030247569,XR_003950807,XR_374413,XR_37	Gnas	GNAS (guanine nucleotide binding protein, alpha stimulating) complex locus	protein_coding	NP_001070975.1;NP_001070978.1;NP_001297012.1;NP_001297014.1;NP_001350959.1;NP_034439.2;NP_062664.2;NP_068840.2;NP_963910.1;NP_963911.1;NP_963912.1;XP_006498837.1;XP_006498840.1;XP_006498841.1;XP_006498842.1;XP_006498843.1;XP_017171147.1;XP_030103428.1;XP_030103429.1	95777	.	496.738085	[illegible]
14685	NM_008140	Gnat1	guanine nucleotide binding protein, alpha transducing 1	protein_coding	NP_032166.1	95778	.	0.000000	[illegible]
14686	NM_008141,XM_006501008,XM_030252431	Gnat2	guanine nucleotide binding protein, alpha transducing 2	protein_coding	NP_032167.1;XP_006501071.1;XP_030108291.1	95779	.	0.581138	[illegible]
14687	NM_001358848,NM_010311,XM_006513235,XM_006513236	Gnaz	guanine nucleotide binding protein, alpha z subunit	protein_coding	NP_001345777.1;NP_034441.1;XP_006513298.1;XP_006513299.1	95780	.	0.236372	[illegible]
14688	NM_001160016,NM_001160017,NM_008142,XM_017319977	Gnb1	guanine nucleotide binding protein (G protein), beta 1	protein_coding	NP_001153488.1;NP_001153489.1;NP_032168.1;XP_017175466.1	95781	.	77.538167	[illegible]
14693	NM_010312,XM_006504553	Gnb2	guanine nucleotide binding protein (G protein), beta 2	protein_coding	NP_034442.1;XP_006504616.1	95784	.	99.086504	[illegible]
14694	NM_008143	Rack1	receptor for activated C kinase 1	protein_coding	NP_032169.1	101849	.	661.138855	[illegible]
14695	NM_013530	Gnb3	guanine nucleotide binding protein (G protein), beta 3	protein_coding	NP_038558.1	95785	.	0.434146	[illegible]
14696	NM_001348062,NM_001348104,NM_001348105,NM_013531,XM_006535404	Gnb4	guanine nucleotide binding protein (G protein), beta 4	protein_coding	NP_001334991.1;NP_001335033.1;NP_001335034.1;NP_038559.2;XP_006535467.1	104581	.	13.760058	[illegible]
14697	NM_010313,NM_138719,NR_073186	Gnb5	guanine nucleotide binding protein (G protein), beta 5	protein_coding	NP_034443.1;NP_619733.1	101848	.	9.757585	[illegible]
14700	NM_025277	Gng10	guanine nucleotide binding protein (G protein), gamma 10	protein_coding	NP_079553.1	1336169	.	27.913349	[illegible]
14701	NM_001177556,NM_001177557,NM_001177558,NM_001177559,NM_001177560,NM_025278,XM_006505542	Gng12	guanine nucleotide binding protein (G protein), gamma 12	protein_coding	NP_001171027.1;NP_001171028.1;NP_001171029.1;NP_001171030.1;NP_001171031.1;NP_079554.1;XP_006505605.1	1336171	.	18.043998	[illegible]
14702	NM_001038637,NM_001285908,NM_001285909,NM_001285910,NM_001285911,NM_010315,XM_030247652	Gng2	guanine nucleotide binding protein (G protein), gamma 2	protein_coding	NP_001033726.1;NP_001272837.1;NP_001272838.1;NP_001272839.1;NP_001272840.1;NP_034445.1;XP_030103512.1	102705	.	10.543044	[illegible]
14704	NM_010316	Gng3	guanine nucleotide binding protein (G protein), gamma 3	protein_coding	NP_034446.1	102704	.	0.108518	[illegible]
14705	NM_001136064,NM_001290823,NM_008144,XM_006526691,XM_030250754	Bscl2	Berardinelli-Seip congenital lipodystrophy 2 (seipin)	protein_coding	NP_001129536.1;NP_001277752.1;NP_032170.3;XP_006526754.1;XP_030106614.1	1298392	.	24.557973	[illegible]
14706	NM_001302997,NM_010317	Gng4	guanine nucleotide binding protein (G protein), gamma 4	protein_coding	NP_001289926.1;NP_034447.1	102703	.	0.287388	[illegible]
14707	NM_010318	Gng5	guanine nucleotide binding protein (G protein), gamma 5	protein_coding	NP_034448.2	109164	.	96.200806	[illegible]
14708	NM_001038655,NM_010319	Gng7	guanine nucleotide binding protein (G protein), gamma 7	protein_coding	NP_001033744.1;NP_034449.2	95787	.	1.775607	[illegible]
14709	NM_010320,XM_006539547,XM_006539548,XM_006539549,XM_011250436	Gng8	guanine nucleotide binding protein (G protein), gamma 8	protein_coding	NP_034450.1;XP_006539610.1;XP_006539611.1;XP_006539612.1;XP_011248738.1	109163	.	5.752107	[illegible]
14710	NM_001038664,NM_001284393,NM_001284397,NM_023121,XM_006532231,XM_011248737,XM_017314274,XM_030245574	Gngt2	guanine nucleotide binding protein (G protein), gamma transducing activity polypeptide 2	protein_coding	NP_001033753.2;NP_001271322.1;NP_001271326.1;NP_075610.1;XP_006532294.1;XP_011247039.1;XP_017169763.1;XP_030101434.1	893584	.	12.262368	[illegible]
14711	NM_001364890,NM_010321,XR_001782039	Gnmt	glycine N-methyltransferase	protein_coding	NP_001351819.1;NP_034451.1	1202304	.	0.667266	[illegible]
14712	NM_010322	Gnpat	glyceronephosphate O-acyltransferase	protein_coding	NP_034452.3	1343460	.	21.104015	[illegible]
14714	NM_008145,NR_133010,XM_006518564	Gnrh1	gonadotropin releasing hormone 1	protein_coding	NP_032171.1;XP_006518627.1	95789	.	2.252692	[illegible]
14718	NM_010324	Got1	glutamic-oxaloacetic transaminase 1, soluble	protein_coding	NP_034454.2	95791	.	7.281321	[illegible]
14719	NM_010325	Got2	glutamatic-oxaloacetic transaminase 2, mitochondrial	protein_coding	NP_034455.1	95792	.	31.491514	[illegible]
14720	gene-Got2-ps1	Got2-ps1	glutamatic-oxaloacetic transaminase 2, mitochondrial, pseudogene 1	pseudogene	.	104721	.	0.869572	[illegible]
14723	NM_010326,XM_006532232	Gp1ba	glycoprotein 1b, alpha polypeptide	protein_coding	NP_034456.2;XP_006532295.1	1333744	.	0.124507	[illegible]
14724	NM_001001999,NM_010327	Gp1bb	glycoprotein Ib, beta polypeptide	protein_coding	NP_001001999.1;NP_034457.1	107852	.	3.824022	[illegible]
14725	NM_001081088	Lrp2	low density lipoprotein receptor-related protein 2	protein_coding	NP_001074557.1	95794	.	0.007601	[illegible]
14726	NM_001290822,NM_010329	Pdpn	podoplanin	protein_coding	NP_001277751.1;NP_034459.2	103098	.	20.180918	[illegible]
14727	NM_001291892,NM_001291893,NM_008147	Lilr4b	leukocyte immunoglobulin-like receptor, subfamily B, member 4B	protein_coding	NP_001278821.1;NP_001278822.1;NP_032173.1	102702	.	2.416589	[illegible]
14728	NM_001291894,NM_013532,XM_030244886,XR_001779463,XR_003948594,XR_003948595,XR_871431,XR_871432,XR_871433	Lilrb4a	leukocyte immunoglobulin-like receptor, subfamily B, member 4A	protein_coding	NP_001278823.1;NP_038560.1;XP_030100746.1	102701	.	5.572662	[illegible]
14729	NM_008148	Gp5	glycoprotein 5 (platelet)	protein_coding	NP_032174.2	1096363	.	0.086181	[illegible]
14731	NM_010331,XM_017316432,XM_030248337	Gpaa1	GPI anchor attachment protein 1	protein_coding	NP_034461.1;XP_017171921.1;XP_030104197.1	1202392	.	15.506998	[illegible]
14732	NM_001356285,NM_008149,XM_006526692,XM_006526694,XM_011247147	Gpam	glycerol-3-phosphate acyltransferase, mitochondrial	protein_coding	NP_001343214.1;NP_032175.2;XP_006526755.1;XP_006526757.1;XP_011245449.1	109162	.	6.678926	[illegible]
14733	NM_016696,XM_006529142,XM_011247925	Gpc1	glypican 1	protein_coding	NP_057905.1;XP_006529205.1;XP_011246227.1	1194891	.	91.377069	[illegible]
14734	NM_016697	Gpc3	glypican 3	protein_coding	NP_057906.2	104903	.	145.411591	[illegible]
14735	NM_008150,XM_011250978	Gpc4	glypican 4	protein_coding	NP_032176.2;XP_011249280.1	104902	.	23.311052	[illegible]
14738	NM_001010941,NM_001359055,NM_001359056,NM_001359057,NM_001359058,NM_008151,XM_006504806,XM_030254133,XM_030254134	Gpr12	G-protein coupled receptor 12	protein_coding	NP_001010941.1;NP_001345984.1;NP_001345985.1;NP_001345986.1;NP_001345987.1;NP_032177.1;XP_006504869.1;XP_030109993.1;XP_030109994.1	101909	.	0.000000	[illegible]
14739	NM_010333,XM_006510019	S1pr2	sphingosine-1-phosphate receptor 2	protein_coding	NP_034463.2;XP_006510082.1	99569	.	14.696448	[illegible]
14744	NM_008152	Gpr65	G-protein coupled receptor 65	protein_coding	NP_032178.1	108031	.	0.718595	[illegible]
14745	NM_001290486,NM_010336,NM_172989,XM_011249928,XM_011249929,XM_011249930,XM_011249931,XM_011249932,XM_011249933,XM_011249934,XM_011249935,XM_030253214,XM_030253215	Lpar1	lysophosphatidic acid receptor 1	protein_coding	NP_001277415.1;NP_034466.2;NP_766577.1;XP_011248230.1;XP_011248231.1;XP_011248232.1;XP_011248233.1;XP_011248234.1;XP_011248235.1;XP_011248236.1;XP_011248237.1;XP_030109074.1;XP_030109075.1	108429	.	23.960263	[illegible]
14747	NM_001359060,NM_008153,XM_006530164,XM_006530165,XM_006530166	Cmklr1	chemokine-like receptor 1	protein_coding	NP_001345989.1;NP_032179.1;XP_006530227.1;XP_006530228.1;XP_006530229.1	109603	.	4.127472	[illegible]
14748	NM_008154	Gpr3	G-protein coupled receptor 3	protein_coding	NP_032180.1	101908	.	0.000000	[illegible]
14751	NM_008155	Gpi1	glucose phosphate isomerase 1	protein_coding	NP_032181.2	95797	.	63.498661	[illegible]
14755	NM_001291025,NM_001357592,NM_011822,XM_006523664,XM_017317256,XM_030249502,XM_030249503,XR_001782040,XR_003952089,XR_003952090,XR_385283	Pigq	phosphatidylinositol glycan anchor biosynthesis, class Q	protein_coding	NP_001277954.1;NP_001344521.1;NP_035952.2;XP_006523727.1;XP_017172745.1;XP_030105362.1;XP_030105363.1	1333114	.	15.388039	[illegible]
14756	NM_008156,XM_011244282,XM_017315390,XM_017315391,XM_017315392,XM_030247157	Gpld1	glycosylphosphatidylinositol specific phospholipase D1	protein_coding	NP_032182.2;XP_011242584.1;XP_017170879.1;XP_017170880.1;XP_017170881.1;XP_030103017.1	106604	.	0.432637	[illegible]
14758	NM_001177955,NM_001177956,NM_001177957,NM_001177958,NM_001177959,NM_001177960,NM_001177961,NM_001177962,NM_023122,XM_011247784,XM_017318385	Gpm6b	glycoprotein m6b	protein_coding	NP_001171426.1;NP_001171427.1;NP_001171428.1;NP_001171429.1;NP_001171430.1;NP_001171431.1;NP_001171432.1;NP_001171433.1;NP_075611.1;XP_011246086.1;XP_017173874.1	107672	.	4.027391	[illegible]
14760	NM_001167694,NM_001167695,NM_001167696,NM_001167697,NM_001167699,NM_001167700,NM_001362048,NM_001362049,NM_001362050,NM_001362051,NM_001362052,NM_008157,NR_155270,XM_006505543,XM_006505544,XM_006505545,XM_006505547,XM_017321390,XM_017321391,XM_017321392,XM_017321393,XM_030255161,XM_030255162,XM_030255163,XM_030255164,XM_030255165,XM_030255166,XM_030255167	Gpr19	G protein-coupled receptor 19	protein_coding	NP_001161166.1;NP_001161167.1;NP_001161168.1;NP_001161169.1;NP_001161171.1;NP_001161172.1;NP_001348977.1;NP_001348978.1;NP_001348979.1;NP_001348980.1;NP_001348981.1;NP_032183.2;XP_006505606.1;XP_006505607.1;XP_006505608.1;XP_006505610.1;XP_017176879.1;XP_017176880.1;XP_017176881.1;XP_017176882.1;XP_030111021.1;XP_030111022.1;XP_030111023.1;XP_030111024.1;XP_030111025.1;XP_030111026.1;XP_030111027.1	892973	.	2.356573	[illegible]
14761	NM_008158	Gpr27	G protein-coupled receptor 27	protein_coding	NP_032184.1	1202299	.	3.808060	[illegible]
14762	NM_008159	Gpr33	G protein-coupled receptor 33	protein_coding	NP_032185.1	1277106	.	0.000000	[illegible]
14763	NM_010338	Gpr37	G protein-coupled receptor 37	protein_coding	NP_034468.2	1313297	.	0.496675	[illegible]
14764	NM_009962,XM_006526695,XM_006526696,XM_011247148	Ptgdr2	prostaglandin D2 receptor 2	protein_coding	NP_034092.1;XP_006526758.1;XP_006526759.1;XP_011245450.1	1330275	.	0.018347	[illegible]
14765	NM_001308501,NM_010340,XM_006527827	Gpr50	G-protein-coupled receptor 50	protein_coding	NP_001295430.1;NP_034470.1;XP_006527890.1	1333877	.	0.000000	[illegible]
14766	NM_001198894,NM_018882,XM_006530680,XM_006530681,XM_006530683,XM_006530685,XM_006530686,XM_006530687,XM_006530688,XM_006530689,XM_006530690,XM_006530691,XM_006530692,XM_006530693,XM_006530694,XM_006530695,XM_006530696,XM_006530697,XM_017312577,XM_030243299	Adgrg1	adhesion G protein-coupled receptor G1	protein_coding	NP_001185823.1;NP_061370.2;XP_006530743.1;XP_006530744.1;XP_006530746.1;XP_006530748.1;XP_006530749.1;XP_006530750.1;XP_006530751.1;XP_006530752.1;XP_006530753.1;XP_006530754.1;XP_006530755.1;XP_006530756.1;XP_006530757.1;XP_006530758.1;XP_006530759.1;XP_006530760.1;XP_017168066.1;XP_030099	1340051	.	2.485818	[illegible]
14767	NM_001319227,NM_010341	Nmur1	neuromedin U receptor 1	protein_coding	NP_001306156.1;NP_034471.1	1341898	.	0.000000	[illegible]
14768	NM_001190984,NM_001190985,NM_021295,XM_011238435	Lancl1	LanC (bacterial lantibiotic synthetase component C)-like 1	protein_coding	NP_001177913.1;NP_001177914.1;NP_067270.1;XP_011236737.1	1336997	.	5.208664	[illegible]
14772	NM_001080743,NM_019497,XM_006503742,XM_017320670,XM_017320671,XM_030254135,XM_030254136,XM_030254137,XM_030254138,XR_003955582,XR_003955584,XR_00395	Grk4	G protein-coupled receptor kinase 4	protein_coding	NP_001074212.1;NP_062370.2;XP_006503805.1;XP_017176159.1;XP_017176160.1;XP_030109995.1;XP_030109996.1;XP_030109997.1;XP_030109998.1	95801	.	2.755610	[illegible]
14773	NM_018869,XM_006526697,XM_011247149,XM_017318066	Grk5	G protein-coupled receptor kinase 5	protein_coding	NP_061357.3;XP_006526760.1;XP_011245451.1;XP_017173555.1	109161	.	3.170303	[illegible]
14775	NM_001329527,NM_001329528,NM_008160	Gpx1	glutathione peroxidase 1	protein_coding	NP_001316456.1;NP_001316457.1;NP_032186.2	104887	.	103.713719	[illegible]
14776	NM_030677	Gpx2	glutathione peroxidase 2	protein_coding	NP_109602.2	106609	.	0.044403	[illegible]
14777	NR_033563	Gpx2-ps1	glutathione peroxidase 2, pseudogene 1	transcribed_pseudogene	.	106627	.	0.043084	[illegible]
14778	NM_001329860,NM_008161	Gpx3	glutathione peroxidase 3	protein_coding	NP_001316789.1;NP_032187.2	105102	.	154.237523	[illegible]
14782	NM_010344	Gsr	glutathione reductase	protein_coding	NP_034474.4	95804	.	15.958761	[illegible]
14783	NM_001177629,NM_001370603,NM_010345,XM_011243664	Grb10	growth factor receptor bound protein 10	protein_coding	NP_001171100.1;NP_001357532.1;NP_034475.2;XP_011241966.1	103232	.	206.265062	[illegible]
14784	NM_001313936,NM_001313937,NM_008163	Grb2	growth factor receptor bound protein 2	protein_coding	NP_001300865.1;NP_001300866.1;NP_032189.1	95805	.	21.232768	[illegible]
14786	NM_010346,XM_006532233,XM_030245575	Grb7	growth factor receptor bound protein 7	protein_coding	NP_034476.1;XP_006532296.1;XP_030101435.1	102683	.	0.012458	[illegible]
14787	NM_001163465,NM_008164,XM_006520447,XM_017316433,XM_017316434	Rhpn1	rhophilin, Rho GTPase binding protein 1	protein_coding	NP_001156937.1;NP_032190.2;XP_006520510.1;XP_017171922.1;XP_017171923.1	1098783	.	0.316791	[illegible]
14788	NM_001355257,NM_013533,XM_006505555,XM_006505557,XM_011241215,XM_030255168,XR_003956164	Gpr162	G protein-coupled receptor 162	protein_coding	NP_001342186.1;NP_038561.1;XP_006505618.1;XP_006505620.1;XP_011239517.1;XP_030111028.1	1315214	.	0.666203	[illegible]
14789	NM_013534,NR_163804,NR_163805,NR_163806,XM_030255169	P3h3	prolyl 3-hydroxylase 3	protein_coding	NP_038562.2;XP_030111029.1	1315208	.	76.503616	[illegible]
14790	NM_013535	Grcc10	gene rich cluster, C10 gene	protein_coding	NP_038563.1	1315201	.	67.319542	[illegible]
14791	NM_013536,XM_030255170	Emg1	EMG1 N1-specific pseudouridine methyltransferase	protein_coding	NP_038564.1;XP_030111030.1	1315195	.	32.040290	[illegible]
14792	NM_145130,NR_149320,XR_377423	Lpcat3	lysophosphatidylcholine acyltransferase 3	protein_coding	NP_660112.1	1315211	.	17.103151	[illegible]
14793	NM_013538,XM_006505564	Cdca3	cell division cycle associated 3	protein_coding	NP_038566.1;XP_006505627.1	1315198	.	18.754269	[illegible]
14794	NM_001306050,NM_001306051,NM_001306052,NM_001306053,NM_013539,XM_006505566,XM_006505567,XM_030255171,XM_030255172,XM_030255173	Spsb2	splA/ryanodine receptor domain and SOCS box containing 2	protein_coding	NP_001292979.1;NP_001292980.1;NP_001292981.1;NP_001292982.1;NP_038567.1;XP_006505629.2;XP_006505630.1;XP_030111031.1;XP_030111032.1;XP_030111033.1	1315199	.	6.436109	[illegible]
14797	NM_001276288,NM_010347,NR_074087	Tle5	TLE family member 5, transcriptional modulator	protein_coding	NP_001263217.1;NP_034477.1	95806	.	81.171501	[illegible]

14799	NM_001113325,NM_001252403,NM_008165,XM_006532234,XM_017314275,XR_388348	Gria1	glutamate receptor, ionotropic, AMPA1 (alpha 1)	protein_coding	NP_001106796.1;NP_001239332.1;NP_032191.2;XP_006532297.1;XP_017169764.1	95808	.	3.077244
14800	NM_001039195,NM_001083806,NM_001357924,NM_001357927,NM_013540,NR_152147,NR_152148,NR_152149,XM_006501014,XM_006501015,XR_001783652,XR_001783653,XR_001783654,XR_001783655,XR_001783656,XR_001783657,XR_001783660,XR_001783662,XR_003954282,XR_003954283,XR_003954284,XR_003954285,XR_003954286,XR_003954287,XR_003954288,XR_003954289,XR_003954290,XR_003954291,XR_375488	Gria2	glutamate receptor, ionotropic, AMPA2 (alpha 2)	protein_coding	NP_001034284.1;NP_001077275.1;NP_001344853.1;NP_001344856.1;NP_038568.2;XP_006501077.1;XP_006501078.1	95809	.	0.033403
14802	NM_001113180,NM_001113181,NM_019691,XM_006509841,XM_006509842,XM_006509843,XM_006509844,XM_006509845,XM_011242355,XR_001778797,XR_001778798,XR_001778799,XR_001778800,XR_379084,XR_379085	Gria4	glutamate receptor, ionotropic, AMPA4 (alpha 4)	protein_coding	NP_001106651.1;NP_001106652.1;NP_062665.3;XP_006509904.1;XP_006509905.1;XP_006509906.1;XP_006509907.1;XP_006509908.1;XP_011240657.1	95811	.	0.552391
14803	NM_008166,XM_006518565,XM_006518566,XM_006518567,XM_011244962	Grid1	glutamate receptor, ionotropic, delta 1	protein_coding	NP_032192.2;XP_006518628.1;XP_006518629.1;XP_006518630.1;XP_011243264.1	95812	.	0.650729
14804	NM_001370966,NM_008167	Grid2	glutamate receptor, ionotropic, delta 2	protein_coding	NP_001357895.1;NP_032193.1	95813	.	0.098487
14805	NM_001346964,NM_010348,NM_146072,XM_006522914,XM_006522915,XM_006522916,XM_006522918,XM_006522919,XM_006522920,XM_006522921,XM_017316880,XR_384929,XR_384930	Grik1	glutamate receptor, ionotropic, kainate 1	protein_coding	NP_001333893.1;NP_034478.1;NP_666184.2;XP_006522977.1;XP_006522978.1;XP_006522979.1;XP_006522981.1;XP_006522982.1;XP_006522983.1;XP_006522984.1;XP_017172369.1	95814	.	0.165678
14806	NM_001111268,NM_001358866,NM_010349,XM_006512545,XM_011243120,XM_011243121,XM_011243122,XM_011243123,XM_011243124,XM_011243125,XM_011243127,XM_030244887,XR_001779464,XR_001779465,XR_003948597	Grik2	glutamate receptor, ionotropic, kainate 2 (beta 2)	protein_coding	NP_001104738.1;NP_001345795.1;NP_034479.2;XP_006512608.1;XP_011241422.1;XP_011241423.1;XP_011241424.1;XP_011241425.1;XP_011241426.1;XP_011241427.1;XP_011241429.1;XP_030100747.1	95815	.	0.352799
14807	NM_001081097,XM_006502772,XM_017319980	Grik3	glutamate receptor, ionotropic, kainate 3	protein_coding	NP_001074566.1;XP_006502835.1;XP_017175469.1	95816	.	0.078967
14809	NM_001360067,NM_008168,XM_006539552,XM_017321983,XM_030242142,XM_030242143,XM_030242144,XM_030242145,XM_030242146,XM_030242147,XM_030242148,XM_030242149	Grik5	glutamate receptor, ionotropic, kainate 5 (gamma 2)	protein_coding	NP_001346996.1;NP_032194.2;XP_006539615.1;XP_017177472.1;XP_030098002.1;XP_030098003.1;XP_030098004.1;XP_030098005.1;XP_030098006.1;XP_030098007.1;XP_030098008.1;XP_030098009.	95818	.	10.428575
14810	NM_001177656,NM_001177657,NM_008169,XM_006497722,XM_006497723,XM_006497724,XM_006497725,XM_006497726	Grin1	glutamate receptor, ionotropic, NMDA1 (zeta 1)	protein_coding	NP_001171127.1;NP_001171128.1;NP_032195.1;XP_006497785.1;XP_006497786.1;XP_006497787.1;XP_006497788.1;XP_006497789.1	95819	.	0.036010
14811	NM_008170,XM_006521795	Grin2a	glutamate receptor, ionotropic, NMDA2A (epsilon 1)	protein_coding	NP_032196.2;XP_006521858.1	95820	.	0.048749
14812	NM_001363750,NM_008171,XM_006505573,XM_006505574,XM_006505575,XM_011241221,XM_017321396	Grin2b	glutamate receptor, ionotropic, NMDA2B (epsilon 2)	protein_coding	NP_001350679.1;NP_032197.3;XP_006505636.1;XP_006505637.1;XP_006505638.1;XP_011239523.1;XP_017176885.1	95821	.	0.040505
14813	NM_010350,XM_006532242,XM_011248738,XM_011248739,XM_011248741,XM_011248742,XM_011248743,XM_017314276,XM_017314277,XM_017314278,XM_017314279,XM_017314280,XM_030245576,XR_388349	Grin2c	glutamate receptor, ionotropic, NMDA2C (epsilon 3)	protein_coding	NP_034480.2;XP_006532305.1;XP_011247040.1;XP_011247041.1;XP_011247043.1;XP_011247044.1;XP_011247045.1;XP_017169765.1;XP_017169766.1;XP_017169767.1;XP_017169768.1;XP_017169769.1;XP_030101436.1	95822	.	0.015701
14814	NM_008172,XM_006540631,XM_011250800,XM_011250802,XM_011250804,XM_030242150,XM_030242151,XM_030242152,XM_030242153	Grin2d	glutamate receptor, ionotropic, NMDA2D (epsilon 4)	protein_coding	NP_032198.2;XP_006540694.2;XP_011249102.1;XP_011249104.1;XP_011249106.2;XP_030098010.1;XP_030098011.1;XP_030098012.1;XP_030098013.1	95823	.	3.682383
14815	NM_001361209,NM_001361210,NM_001361211,NM_001361212,NM_008173,XM_006525658,XM_006525659,XM_006525660,XM_006525661,XM_006525662,XM_006525663,XM_006525664,XM_006525665,XM_030250338,XM_030250339	Nr3c1	nuclear receptor subfamily 3, group C, member 1	protein_coding	NP_001348138.1;NP_001348139.1;NP_001348140.1;NP_001348141.1;NP_032199.3;XP_006525721.1;XP_006525722.1;XP_006525723.1;XP_006525724.1;XP_006525725.1;XP_006525726.1;XP_006525727.1;XP_006525728.1;XP_030106198.1;XP_030106199.1	95824	.	17.652703
14816	NM_001114333,NM_016976,XM_006512549,XM_006512550,XM_006512551,XM_006512554	Grm1	glutamate receptor, metabotropic 1	protein_coding	NP_001107805.1;NP_058672.1;XP_006512612.1;XP_006512613.1;XP_006512614.1;XP_006512617.1	1351338	.	0.000000
14823	NM_001311072,NM_001361125,NM_008174,XM_006504989,XM_006504990,XM_006504991,XM_030255174,XM_030255175	Grm8	glutamate receptor, metabotropic 8	protein_coding	NP_001298001.1;NP_001348054.1;NP_032200.2;XP_006505052.1;XP_006505053.1;XP_006505054.1;XP_030111034.1;XP_030111035.1	1351345	.	0.042351
14824	NM_008175,XM_030245577	Grn	granulin	protein_coding	NP_032201.3;XP_030101437.1	95832	.	78.871164
14825	NM_008176	Cxcl1	chemokine (C-X-C motif) ligand 1	protein_coding	NP_032202.1	108068	.	0.268190
14827	NM_007952	Pdia3	protein disulfide isomerase associated 3	protein_coding	NP_031978.2	95834	.	220.482239
14828	NM_001163434,NM_022310	Hspa5	heat shock protein 5	protein_coding	NP_001156906.1;NP_071705.3	95835	.	226.294908
14829	NM_008177	Grpr	gastrin releasing peptide receptor	protein_coding	NP_032203.1	95836	.	0.130988
14836	NM_010351	Gsc	goosecoid homeobox	protein_coding	NP_034481.1	95841	.	2.564790
14840	NM_001080552,NM_001080553,NM_010352	Gsg1	germ cell associated 1	protein_coding	NP_001074021.1;NP_001074022.1;NP_034482.2	1194499	.	1.116939
14841	NM_010353	Haspin	histone H3 associated protein kinase	protein_coding	NP_034483.1	1194498	.	1.201228
14842	NM_008178	Gsx1	GS homeobox 1	protein_coding	NP_032204.1	95842	.	0.000000
14843	NM_133256	Gsx2	GS homeobox 2	protein_coding	NP_573555.1	95843	.	0.000000
14852	NM_001130008,NM_146066	Gspt1	G1 to S phase transition 1	protein_coding	NP_001123480.1;NP_666178.2	1316728	.	20.462640
14853	NM_008179	Gspt2	G1 to S phase transition 2	protein_coding	NP_032205.2	1316727	.	1.079049
14854	NM_001291111,NM_008180,XM_006498781	Gss	glutathione synthetase	protein_coding	NP_001278040.1;NP_032206.1;XP_006498844.1	95852	.	5.282163
14859	NM_001077353,NM_001288617,NM_010356,XM_030248789	Gsta3	glutathione S-transferase, alpha 3	protein_coding	NP_001070821.1;NP_001275546.1;NP_034486.2;XP_030104649.1	95856	.	0.163484
14860	NM_010357	Gsta4	glutathione S-transferase, alpha 4	protein_coding	NP_034487.2	1309515	.	5.618262
14862	NM_010358,XM_006501017	Gstm1	glutathione S-transferase, mu 1	protein_coding	NP_034488.1;XP_006501080.1	95860	.	13.181910
14863	NM_008183	Gstm2	glutathione S-transferase, mu 2	protein_coding	NP_032209.1	95861	.	24.426037
14864	NM_010359	Gstm3	glutathione S-transferase, mu 3	protein_coding	NP_034489.1	106026	.	0.068977
14865	NM_001160411,NM_026764,XM_006501018,XM_006501020,XM_006501021,XM_017319462	Gstm4	glutathione S-transferase, mu 4	protein_coding	NP_001153883.1;NP_081040.1;XP_006501081.1;XP_006501083.1;XP_006501084.1;XP_017174951.1	95862	.	2.718564
14866	NM_010360	Gstm5	glutathione S-transferase, mu 5	protein_coding	NP_034490.1	1309466	.	36.343750
14867	NM_008184,XM_006501022,XM_006501023,XM_006501024,XM_006501025,XM_030252432,XM_030252433	Gstm6	glutathione S-transferase, mu 6	protein_coding	NP_032210.3;XP_006501085.1;XP_006501086.1;XP_006501087.1;XP_006501088.1;XP_030108292.1;XP_030108293.1	1309467	.	0.962604
14869	NM_181796	Gstp2	glutathione S-transferase, pi 2	protein_coding	NP_861461.1	95864	.	7.433780
14870	NM_013541	Gstp1	glutathione S-transferase, pi 1	protein_coding	NP_038569.1	95865	.	53.409126
14871	NM_001358778,NM_008185,XM_030244888	Gstt1	glutathione S-transferase, theta 1	protein_coding	NP_001345707.1;NP_032211.3;XP_030100748.1	107379	.	8.904017
14872	NM_010361,XM_006513240,XM_006513241,XM_011243373,XM_017313805,XM_030244889	Gstt2	glutathione S-transferase, theta 2	protein_coding	NP_034491.2;XP_006513303.1;XP_006513304.1;XP_011241675.1;XP_017169294.1;XP_030100749.1	106188	.	2.872650
14873	NM_010362	Gsto1	glutathione S-transferase omega 1	protein_coding	NP_034492.1	1342273	.	18.544750
14874	NM_001252555,NM_001252556,NM_001364306,NM_001364307,NM_010363,XM_006515474,XM_006515475	Gstz1	glutathione transferase zeta 1 (maleylacetoacetate isomerase)	protein_coding	NP_001239484.1;NP_001239485.1;NP_001351235.1;NP_001351236.1;NP_034493.1;XP_006515537.1;XP_006515538.1	1341859	.	18.527452
14884	NM_001291075,NM_001360075,NM_001360076,NM_008186	Gtf2h1	general transcription factor II H, polypeptide 1	protein_coding	NP_001278004.1;NP_001347004.1;NP_001347005.1;NP_032212.3	1277216	.	15.785948
14885	NM_010364,XM_006523668,XM_006523669,XM_030249504	Gtf2h4	general transcription factor II H, polypeptide 4	protein_coding	NP_034494.1;XP_006523731.1;XP_006523732.1;XP_030105364.1	1338799	.	7.039974
14886	NM_001080746,NM_001080747,NM_001080748,NM_001080749,NM_001359062,NM_001359063,NM_001359064,NM_001359065,NM_001359066,NM_001359067,NM_010365,XM_006504384,XM_006504385,XM_006504386,XM_006504389,XM_006504391,XM_017320677,XM_017320681,XM_030254140,XM_030254141,XM_030254143,XM_030254144,XM_030254145,XM_030254146,XM_030254147,XM_030254148,XM_030254149,XM_030254150,XM_030254151,XM_030254152,XM_030254153,XM_030254154,XM_030254155,XM_030254156,XM_030254157,XM_030254158,XM_030254159,XM_030254160,XM_030254161,XM_030254162,XM_030254163,XM_030254164,XM_030254165	Gtf2i	general transcription factor II I	protein_coding	NP_001074215.1;NP_001074216.1;NP_001074217.1;NP_001074218.1;NP_001345991.1;NP_001345992.1;NP_001345993.1;NP_001345994.1;NP_001345995.1;NP_001345996.1;NP_034495.2;XP_006504447.1;XP_006504448.1;XP_006504449.1;XP_006504452.1;XP_006504454.1;XP_017176166.1;XP_017176170.1;XP_030110000.1;XP_030110001.1;XP_030110003.1;XP_030110004.1;XP_030110005.1;XP_030110006.1;XP_030110007.1;XP_030110008.1;XP_030110009.1;XP_030110010.1;XP_030110011.1;XP_030110012.1;XP_030110013.1;XP_030110014.1;XP_030110015.1;XP_030110016.1;XP_030110017.1;XP_030110018.1;XP_030110019.1;XP_030110020.1;XP_030110021.1;XP_030110022.1;XP_030110023.1;XP_030110024.1;XP_030110025.	1202722	.	56.975737
14894	NM_008187	Cfap20	cilia and flagella associated protein 20	protein_coding	NP_032213.2	107428	.	34.176456
14897	NM_133975,XM_006496409,XM_006496414,XM_006496416,XM_006496422,XM_006496427,XM_006496429,XM_011238654,XM_011238655,XM_011238656,XM_011238657,XM_011238658,XM_011238659,XM_011238660,XM_011238661,XM_011238662,XM_011238663,XM_011238664,XM_011238665,XM_011238666,XM_011238667,XM_011238668,XM_011238669,XM_011238670,XM_011238671,XM_011238672,XM_011238673,XM_011238674,XM_017316627,XM_017316673,XM_030248874,XM_030248895,XM_030248922,XM_030248942,XM_030248998,XM_030249012,XM_030249060,XM_030249149,XM_030249155,XM_030249168,XM_030249180,XM_030249195,XM_030249247,XM_030249259,XM_030249267,XM_030249285,XM_030249293,XM_030249310,XM_030249325	Trip12	thyroid hormone receptor interactor 12	protein_coding	NP_598736.4;XP_006496472.1;XP_006496477.1;XP_006496479.1;XP_006496485.1;XP_006496490.1;XP_006496492.1;XP_011236956.1;XP_011236957.1;XP_011236958.1;XP_011236959.1;XP_011236960.1;XP_011236961.1;XP_011236962.1;XP_011236963.1;XP_011236964.1;XP_011236965.1;XP_011236966.1;XP_011236967.1;XP_011236968.1;XP_011236969.1;XP_011236970.1;XP_011236971.1;XP_011236972.1;XP_011236973.1;XP_011236974.1;XP_011236975.1;XP_011236976.1;XP_017172116.1;XP_017172162.1;XP_030104734.1;XP_030104755.1;XP_030104782.1;XP_030104802.1;XP_030104858.1;XP_030104872.1;XP_030104920.1;XP_030105009.1;XP_030105015.1;XP_030105028.1;XP_030105040.1;XP_030105055.1;XP_030105107.1;XP_030105119.1;XP_030105127.1;XP_030105145.1;XP_030105153.1;XP_030105170.1;XP_030105185.1	1309481	.	26.556781
14904	NM_013818,XM_006520450	Gtpbp1	GTP binding protein 1	protein_coding	NP_038846.2;XP_006520513.1	109443	.	12.633083
14910	NR_027008,NR_027009,NR_027010	Gt(ROSA)26Sor	gene trap ROSA 26, Philippe Soriano	lncRNA	.	104735	.	7.451278
14911	NM_001355266,NM_008188,XM_006505578,XM_006505579	Thumpd3	THUMP domain containing 3	protein_coding	NP_001342195.1;NP_032214.1;XP_006505641.1;XP_006505642.1	1277973	.	6.699801
14912	NM_183248,NR_027857	Nkx6-2	NK6 homeobox 2	protein_coding	NP_899071.2	1352738	.	0.000339
14913	NM_008189,XM_011246296,XR_001782041	Guca1a	guanylate cyclase activator 1a (retina)	protein_coding	NP_032215.2;XP_011244598.1	102770	.	3.980868
14915	NM_008190	Guca2a	guanylate cyclase activator 2a (guanylin)	protein_coding	NP_032216.1	102738	.	0.045652
14917	NM_001127318,NM_145067	Gucy2c	guanylate cyclase 2c	protein_coding	NP_001120790.1;NP_659504.2	106903	.	0.000000
14919	NM_008192,XM_006532244,XM_030245578	Gucy2e	guanylate cyclase 2e	protein_coding	NP_032218.2;XP_006532307.1;XP_030101438.1	105123	.	0.042316
14923	NM_001159410,NM_008193,XM_006532245,XM_006532246,XM_030245579	Guk1	guanylate kinase 1	protein_coding	NP_001152882.1;NP_032219.2;XP_006532308.1;XP_006532309.1;XP_030101439.1	95871	.	18.685705
14924	NM_001029850,NM_001083320,NM_001083321,NM_001286784,NM_001286785,NM_001286786,NM_001286788,NM_010367,NR_104594,XM_006505580,XM_006505581,XM_006505582,XM_006505583,XM_006505584,XM_006505585,XM_006505586,XM_006505587,XM_006505588,XM_006505589,XM_006505590,XM_006505591,XM_006505592,XM_006505593,XM_006505594,XM_006505595,XM_006505596,XM_006505597,XM_006505598,XM_006505599,XM_006505600,XM_006505601,XM_011241223,XM_017321399,XM_017321400,XM_030255176,XM_030255177,XM_030255178,XM_030255179,XM_030255180,XM_030255181,XM_030255182,XM_030255183,XM_030255184,XM_030255185,XM_030255186,XM_030255187,XM_030255188,XM_030255189,XM_030255190,XR_377424	Magi1	membrane associated guanylate kinase, WW and PDZ domain containing 1	protein_coding	NP_001025021.1;NP_001076789.1;NP_001076790.1;NP_001273713.1;NP_001273714.1;NP_001273715.1;NP_001273717.1;NP_034497.1;XP_006505643.1;XP_006505644.1;XP_006505645.1;XP_006505646.1;XP_006505647.1;XP_006505648.1;XP_006505649.1;XP_006505650.1;XP_006505651.1;XP_006505652.1;XP_006505653.1;XP_006505654.1;XP_006505655.1;XP_006505656.1;XP_006505657.1;XP_006505658.1;XP_006505659.1;XP_006505660.1;XP_006505661.1;XP_006505662.1;XP_006505663.1;XP_006505664.1;XP_011239525.1;XP_017176888.1;XP_017176889.1;XP_030111036.1;XP_030111037.1;XP_030111038.1;XP_030111039.1;XP_030111040.1;XP_030111041.1;XP_030111042.1;XP_030111043.1;XP_030111044.1;XP_030111045.1;XP_030111046.1;XP_030111047.1;XP_030111048.1;XP_03011104	1203522	.	3.236375
14933	NM_001294138,NM_001294140,NM_001331046,NM_008194,NM_212444,XM_006527829,XM_006527831,XM_006527833	Gk	glycerol kinase	protein_coding	NP_001281067.1;NP_001281069.1;NP_001317975.1;NP_032220.1;NP_997609.1;XP_006527892.1;XP_006527894.1;XP_006527896.1	106594	.	1.726854
14934	NM_010369	Gypa	glycophorin A	protein_coding	NP_034499.3	95880	.	0.810181
14936	NM_030678	Gys1	glycogen synthase 1, muscle	protein_coding	NP_109603.2	101805	.	8.554863
14938	NM_010370	Gzma	granzyme A	protein_coding	NP_034500.1	109266	.	0.000000
14939	NM_013542	Gzmb	granzyme B	protein_coding	NP_038570.1	109267	.	0.094003
14940	NM_010371,XM_006518568,XM_011244963	Gzmc	granzyme C	protein_coding	NP_034501.2;XP_006518631.1;XP_011243265.1	109256	.	0.000000
14942	NM_010373	Gzme	granzyme E	protein_coding	NP_034503.2	109265	.	0.000000
14945	NM_008196	Gzmk	granzyme K	protein_coding	NP_032222.1	1298232	.	0.000000
14950	NM_001159551,NM_001159552,NM_001159553,NM_010376,XM_006498783,XM_017315666,XM_030247570,XR_001780858,XR_003950808,XR_374416	H13	histocompatibility 13	protein_coding	NP_001153023.1;NP_001153024.1;NP_001153025.1;NP_034506.1;XP_006498846.1;XP_017171155.1;XP_030103430.1	95886	.	33.348531
14955	NR_130973,NR_130974	H19	H19, imprinted maternally expressed transcript	lncRNA	.	95891	.	3668.192932
14957	NM_145713	H1f3	histone cluster 1, H1d	protein_coding	NP_663759.3	107502	.	0.719094
14958	NM_008197	H1f0	H1 histone family, member 0	protein_coding	NP_032223.2	95893	.	97.096024
14960	NM_010378	H2-Aa	histocompatibility 2, class II antigen A, alpha	protein_coding	NP_034508.2	95895	.	1.695447
14961	NM_207105	H2-Ab1	histocompatibility 2, class II antigen A, beta 1	protein_coding	NP_996988.2	103070	.	1.280255
14962	NM_001142706,NM_008198,XM_006523671,XM_006523672	Cfb	complement factor B	protein_coding	NP_001136178.1;NP_032224.2;XP_006523734.1;XP_006523735.1	105975	.	1.998588
14964	NM_010380	H2-D1	histocompatibility 2, D region locus 1	protein_coding	NP_034510.3	95896	.	36.236889
14969	NM_010382	H2-Eb1	histocompatibility 2, class II antigen E beta	protein_coding	NP_034512.2	95901	.	1.498603
14972	NM_001001892,NM_001347346,XM_006523673,XM_006523674,XM_006523676,XM_006523677,XM_006523678,XM_017317258,XM_017317259,XM_030249505,XM_030249506	H2-K1	histocompatibility 2, K1, K region	protein_coding	NP_001001892.2;NP_001334275.1;XP_006523736.1;XP_006523737.1;XP_006523739.1;XP_006523740.1;XP_006523741.1;XP_017172747.1;XP_017172748.1;XP_030105365.1;XP_030105366.1	95904	.	20.213789
14976	NM_001185182,NM_010385	Pfdn6	prefoldin subunit 6	protein_coding	NP_001172111.1;NP_034515.1	95908	.	24.371002
14977	NM_001077709,NM_008202	Slc39a7	solute carrier family 39 (zinc transporter), member 7	protein_coding	NP_001071177.1;NP_032228.2	95909	.	103.945755
14979	NM_013543	H2-Ke6	H2-K region expressed gene 6	protein_coding	NP_038571.2	95911	.	15.831944
14990	NM_001347387	H2-M2	histocompatibility 2, M region locus 2	protein_coding	NP_001334316.1	95914	.	0.000000
14991	NM_013819	H2-M3	histocompatibility 2, M region locus 3	protein_coding	NP_038847.1	95915	.	1.016189
14997	NM_001368317,NM_008205	H2-M9	histocompatibility 2, M region locus 9	protein_coding	NP_001355246.1;NP_032231.1	1276570	.	0.000000
14998	NM_001360530,NM_010386,XM_006523684,XM_017317260,XM_030249508	H2-DMa	histocompatibility 2, class II, locus DMa	protein_coding	NP_001347459.1;NP_034516.3;XP_006523747.1;XP_017172749.1;XP_030105368.1	95921	.	2.705651
14999	NM_010387	H2-DMb1	histocompatibility 2, class II, locus Mb1	protein_coding	NP_034517.2	95922	.	5.843086
15000	NM_010388,NR_160895,NR_160896	H2-DMb2	histocompatibility 2, class II, locus Mb2	protein_coding	NP_034518.1	95923	.	0.507891
15001	NM_008206	H2-Oa	histocompatibility 2, O region alpha locus	protein_coding	NP_032232.2	95924	.	0.000000
15002	NM_010389,XM_006523686	H2-Ob	histocompatibility 2, O region beta locus	protein_coding	NP_034519.2;XP_006523749.1	95925	.	0.088195
15006	NM_001358790,NM_010390,XM_006523687,XM_006523688,XM_006523689,XM_006523690,XM_006523691,XM_006523692,XM_011246300,XM_011246301,XM_011246302,XM_017317262,XM_030249510	H2-Q1	histocompatibility 2, Q region locus 1	protein_coding	NP_001345719.1;NP_034520.2;XP_006523750.1;XP_006523751.1;XP_006523752.1;XP_006523753.1;XP_006523754.1;XP_006523755.1;XP_011244602.1;XP_011244603.1;XP_011244604.1;XP_017172751.1;XP_030105370.1	95928	.	0.000000
15007	NM_010391	H2-Q10	histocompatibility 2, Q region locus 10	protein_coding	NP_034521.1	95929	.	0.422592
15013	NM_001368740,NM_010392	H2-Q2	histocompatibility 2, Q region locus 2	protein_coding	NP_001355669.1;NP_034522.2	95931	.	0.000000
15015	NM_001143689	H2-Q4	histocompatibility 2, Q region locus 4	protein_coding	NP_001137161.1	95933	.	4.433280
15016	NR_051981	H2-Q5	histocompatibility 2, Q region locus 5	transcribed_pseudogene	.	95934	.	0.159480
15018	NM_001198560,NM_001198561,NM_010394,XR_385292	H2-Q7	histocompatibility 2, Q region locus 7	protein_coding	NP_001185489.1;NP_001185490.1;NP_034524.3	95936	.	0.830506
15024	NR_046286	H2-T10	histocompatibility 2, T region locus 10	transcribed_pseudogene	.	95942	.	0.323863
15039	NM_001347382,NM_001359754,NM_001359755,NM_001359756,NM_010397	H2-T22	histocompatibility 2, T region locus 22	protein_coding	NP_001334311.1;NP_001346683.1;NP_001346684.1;NP_001346685.1;NP_034527.1	95956	.	12.464426
15040	NM_010398	H2-T23	histocompatibility 2, T region locus 23	protein_coding	NP_034528.1	95957	.	7.595638
15042	NM_008207,XM_011246306,XM_011246307,XM_011246308,XM_030249511	H2-T24	histocompatibility 2, T region locus 24	protein_coding	NP_032233.2;XP_011244608.1;XP_011244609.1;XP_011244610.1;XP_030105371.1	95958	.	0.294920
15043	NM_008208	H2-T3	histocompatibility 2, T region locus 3	protein_coding	NP_032234.3	95959	.	0.000000
15061	NM_031367	Ifi44l	interferon-induced protein 44 like	protein_coding	NP_112735.1	95975	.	0.676302
15064	NM_001355062,NM_001355063,NM_008209	Mr1	major histocompatibility complex, class I-related	protein_coding	NP_001341991.1;NP_001341992.1;NP_032235.1	1195463	.	7.158291
15077	NM_178216	H3c14	histone cluster 2, H3c1	protein_coding	NP_835734.2	2448355	.	0.995155
15078	NM_008210	H3f3a	H3 histone, family 3A	protein_coding	NP_032236.1	1097686	.	279.337952
15079	gene-H3f3a-ps1	H3f3a-ps1	H3 histone, family 3A, pseudogene 1	pseudogene	.	1101782	.	11.916471
15080	gene-H3f3a-ps2	H3f3a-ps2	H3 histone, family 3A, pseudogene 2	pseudogene	.	1101758	.	11.057781
15081	NM_008211,XM_006532248	H3f3b	H3 histone, family 3B	protein_coding	NP_032237.1;XP_006532311.1	1101768	.	228.621238
15107	NM_008212	Hadh	hydroxyacyl-Coenzyme A dehydrogenase	protein_coding	NP_032238.2	96009	.	32.893520
15108	NM_016763	Hsd17b10	hydroxysteroid (17-beta) dehydrogenase 10	protein_coding	NP_058043.3	1333871	.	30.010187
15109	NM_010401	Hal	histidine ammonia lyase	protein_coding	NP_034531.1	96010	.	0.006506
15112	NM_010403	Hao1	hydroxyacid oxidase 1, liver	protein_coding	NP_034533.1	96011	.	0.104267
15114	NM_001359052,NM_010404,NM_177981,XM_011248744,XM_011248745,XM_030245580,XM_030245581	Hap1	huntingtin-associated protein 1	protein_coding	NP_001345981.1;NP_034534.1;NP_817090.1;XP_011247046.1;XP_011247047.1;XP_030101440.1;XP_030101441.1	1261831	.	0.187627
15115	NM_008214	Hars	histidyl-tRNA synthetase	protein_coding	NP_032240.3	108087	.	23.609436
15116	NM_008215,XM_011246309	Has1	hyaluronan synthase 1	protein_coding	NP_032241.1;XP_011244611.1	106590	.	0.769029
15117	NM_008216	Has2	hyaluronan synthase 2	protein_coding	NP_032242.3	107821	.	2.872700
15118	NM_001331048,NM_008217,XM_006530700,XM_006530701,XM_030243300	Has3	hyaluronan synthase 3	protein_coding	NP_001317977.1;NP_032243.2;XP_006530763.1;XP_006530764.1;XP_030099160.1	109599	.	0.106441
15122	NM_008218	Hba-a1	hemoglobin alpha, adult chain 1	protein_coding	NP_032244.2	96015	.	412.916412
15135	NM_008221	Hbb-y	hemoglobin Y, beta-like embryonic chain	protein_coding	NP_032247.1	96027	.	0.000000
15139	NM_010406,XM_017315669	Hc	hemolytic complement	protein_coding	NP_034536.3;XP_017171158.1	96031	.	0.021540
15159	NM_001331049,NM_001331050,NM_008222,XM_006528705	Hccs	holocytochrome c synthetase	protein_coding	NP_001317978.1;NP_001317979.1;NP_032248.3;XP_006528768.1	106911	.	7.946469
15160	NM_001331047,NM_008223	Serpind1	serine (or cysteine) peptidase inhibitor, clade D, member 1	protein_coding	NP_001317976.1;NP_032249.3	96051	.	0.872950
15161	NM_008224,XM_006527835,XM_006527836,XM_017318387	Hcfc1	host cell factor C1	protein_coding	NP_032250.2;XP_006527898.1;XP_006527899.1;XP_017173876.1	105942	.	16.979009
15162	NM_001172117,NM_010407	Hck	hemopoietic cell kinase	protein_coding	NP_001165588.1;NP_034537.2	96052	.	6.853827
15163	NM_008225	Hcls1	hematopoietic cell specific Lyn substrate 1	protein_coding	NP_032251.2	104568	.	4.455273
15165	NM_010408	Hcn1	hyperpolarization activated cyclic nucleotide gated potassium channel 1	protein_coding	NP_034538.2	1096392	.	0.000000
15166	NM_008226	Hcn2	hyperpolarization-activated, cyclic nucleotide-gated K+ 2	protein_coding	NP_032252.1	1298210	.	0.089193
15168	NM_008227,XM_030252434,XR_003954292	Hcn3	hyperpolarization-activated, cyclic nucleotide-gated K+ 3	protein_coding	NP_032253.1;XP_030108294.1	1298211	.	0.274979
15170	NM_001077705,NM_013545,XM_030255191	Ptpn6	protein tyrosine phosphatase, non-receptor type 6	protein_coding	NP_001071173.1;NP_038573.2;XP_030111051.1	96055	.	4.725348
15181	gene-Gm10093	Gm10093	predicted pseudogene 10093	pseudogene	.	3704479	.	0.454869
15182	NM_008229,XM_006512555,XM_017313806,XM_030244890	Hdac2	histone deacetylase 2	protein_coding	NP_032255.2;XP_006512618.1;XP_017169295.1;XP_030100750.1	1097691	.	43.035539
15183	NM_010411,XR_385976	Hdac3	histone deacetylase 3	protein_coding	NP_034541.2	1343091	.	39.051459
15184	NM_001077696,NM_001284248,NM_001284249,NM_001284250,NM_001361596,NM_010412,XM_011248755,XM_017314281,XM_030245582,XM_030245583,XM_030245584,XM_030245585,XM_030245586,XM_030245587,XM_030245588,XM_030245589,XR_001779	Hdac5	histone deacetylase 5	protein_coding	NP_001071164.1;NP_001271177.1;NP_001271178.1;NP_001271179.1;NP_001348525.1;NP_034542.3;XP_011247057.1;XP_017169770.1;XP_030101442.1;XP_030101443.1;XP_030101444.1;XP_030101445.1;XP_030101446.1;XP_030101447.1;XP_030101448.1;XP_03010144	1333784	.	20.740290
15185	NM_001130416,NM_010413,XM_006527567,XM_017318389,XM_017318390	Hdac6	histone deacetylase 6	protein_coding	NP_001123888.1;NP_034543.3;XP_006527630.1;XP_017173878.1;XP_017173879.1	1333752	.	7.739382
15186	NM_008230,XM_006498784	Hdc	histidine decarboxylase	protein_coding	NP_032256.3;XP_006498847.1	96062	.	1.598172
15191	NM_001331051,NM_001331052,NM_008231,XM_006501029,XM_030252435,XM_030252436	Hdgf	heparin binding growth factor	protein_coding	NP_001317980.1;NP_001317981.1;NP_032257.3;XP_006501092.1;XP_030108295.1;XP_030108296.1	1194494	.	63.060609
15193	NM_001304784,NM_001304785,NM_001331053,NM_008233,XM_006523708,XM_006523709,XM_006523710,XM_030249512,XM_030249513	Hdgfl2	HDGF like 2	protein_coding	NP_001291713.1;NP_001291714.1;NP_001317982.1;NP_032259.1;XP_006523771.1;XP_006523772.1;XP_006523773.1;XP_030105372.1;XP_030105373.1	1194492	.	22.873332
15194	NM_010414,XM_006503744,XM_017320683,XR_376798	Htt	huntingtin	protein_coding	NP_034544.1;XP_006503807.1;XP_017176172.1	96067	.	5.376660
15199	NM_013546,XM_011241224	Hebp1	heme binding protein 1	protein_coding	NP_038574.3;XP_011239526.1	1333880	.	9.216578
15200	NM_010415	Hbegf	heparin-binding EGF-like growth factor	protein_coding	NP_034545.1	96070	.	1.361443
15201	NM_008234,XM_006526699,XM_006526700	Hells	helicase, lymphoid specific	protein_coding	NP_032260.2;XP_006526762.1;XP_006526763.1	106209	.	5.893084
15203	NM_001159627,NM_001159628,NM_010417,NM_181273	Heph	hephaestin	protein_coding	NP_001153099.1;NP_001153100.1;NP_034547.2;NP_851790.1	1332240	.	3.697805
15204	NM_001360080,NM_010418,XM_006540637,XM_006540639	Herc2	HECT and RLD domain containing E3 ubiquitin protein ligase 2	protein_coding	NP_001347009.1;NP_034548.2;XP_006540700.1;XP_006540702.1	103234	.	6.064058
15205	NM_008235,XM_006521797	Hes1	hes family bHLH transcription factor 1	protein_coding	NP_032261.1;XP_006521860.1	104853	.	15.383621
15207	NM_008237,XM_006538550,XM_011250189,XM_011250190,XM_011250191,XM_011250192,XM_011250193,XM_011250194,XM_030253216	Hes3	hes family bHLH transcription factor 3	protein_coding	NP_032263.2;XP_006538613.1;XP_011248491.1;XP_011248492.1;XP_011248493.1;XP_011248494.1;XP_011248495.1;XP_011248496.1;XP_030109076.1	104877	.	0.017155
15208	NM_001370755,NM_010419,XM_006538562	Hes5	hes family bHLH transcription factor 5	protein_coding	NP_001357684.1;NP_034549.1;XP_006538625.2	104876	.	0.000000
15209	NM_010420,XM_006518570	Hesx1	homeobox gene expressed in ES cells	protein_coding	NP_034550.2;XP_006518633.1	96071	.	0.083265
15211	NM_010421,XM_017313136,XR_379397	Hexa	hexosaminidase A	protein_coding	NP_034551.2;XP_017168625.1	96073	.	67.285356
15212	NM_010422	Hexb	hexosaminidase B	protein_coding	NP_034552.1	96074	.	27.594687
15213	NM_010423,XM_006530053	Hey1	hairy/enhancer-of-split related with YRPW motif 1	protein_coding	NP_034553.2;XP_006530116.1	1341800	.	5.168574
15214	NM_013904	Hey2	hairy/enhancer-of-split related with YRPW motif 2	protein_coding	NP_038932.1	1341884	.	0.582930
15216	NM_001347493,NM_010424,XM_006516556,XM_011244284	Hfe	homeostatic iron regulator	protein_coding	NP_001334422.1;NP_034554.2;XP_006516619.1;XP_011242586.1	109191	.	2.991993
15220	NM_008239	Foxq1	forkhead box Q1	protein_coding	NP_032265.3	1298228	.	0.031752
15221	NM_010425	Foxd3	forkhead box D3	protein_coding	NP_034555.3	1347473	.	0.008500
15223	NM_008240,XM_006532269	Foxj1	forkhead box J1	protein_coding	NP_032266.3;XP_006532332.1	1347474	.	0.208086
15227	NM_010426	Foxf1	forkhead box F1	protein_coding	NP_034556.2	1347470	.	0.370336
15228	NM_001160112,NM_008241	Foxg1	forkhead box G1	protein_coding	NP_001153584.1;NP_032267.1	1347464	.	0.046261
15229	NM_008242	Foxd1	forkhead box D1	protein_coding	NP_032268.2	1347463	.	9.097432
15233	NM_013547,NR_027778,XM_030248978	Hgd	homogentisate 1, 2-dioxygenase	protein_coding	NP_038575.2;XP_030104838.1	96078	.	0.114701
15234	NM_001289458,NM_001289459,NM_001289460,NM_001289461,NM_010427	Hgf	hepatocyte growth factor	protein_coding	NP_001276387.1;NP_001276388.1;NP_001276389.1;NP_001276390.1;NP_034557.3	96079	.	0.678738
15235	NM_008243,XM_006511639,XM_011242856,XM_030244059,XM_030244060	Mst1	macrophage stimulating 1 (hepatocyte growth factor-like)	protein_coding	NP_032269.3;XP_006511702.2;XP_011241158.1;XP_030099919.1;XP_030099920.1	96080	.	0.042271

15239	NM_001159328,NM_008244,XM_006532270,XM_006532271,XM_006532272,XM_006532273,XM_006532274,XM_017314293,XM_030245590,XM_030245591	Hgs	HGF-regulated tyrosine kinase substrate	protein_coding
15242	NM_008245	Hhex	hematopoietically expressed homeobox	protein_coding
15245	NM_020259,XM_006530702	Hhip	Hedgehog-interacting protein	protein_coding
15247	NM_008246,XM_006501030,XM_006501031,XM_011240023	Mfsd14a	major facilitator superfamily domain containing 14A	protein_coding
15248	NM_001098203,NM_010430,XM_006532275,XM_017314294	Hic1	hypermethylated in cancer 1	protein_coding
15251	NM_001313919,NM_001313920,NM_010431,XM_006515478,XM_017314961	Hif1a	hypoxia inducible factor 1, alpha subunit	protein_coding
15254	NM_008248	Hint1	histidine triad nucleotide binding protein 1	protein_coding
15257	NM_001301304,NM_001301306,NM_010432,NR_125731,XM_011240024,XR_003954293	Hipk1	homeodomain interacting protein kinase 1	protein_coding
15258	NM_001136065,NM_001294143,NM_001294144,NM_010433,XM_006505602,XM_006505603,XR_001785097	Hipk2	homeodomain interacting protein kinase 2	protein_coding
15259	NM_001145824,NM_010434	Hipk3	homeodomain interacting protein kinase 3	protein_coding
15260	NM_010435,XM_006521798,XM_006521799,XM_006521800,XM_006521801,XM_030248979,XR_003951761	Hira	histone cell cycle regulator	protein_coding
15267	NM_013549	H2ac18	histone cluster 2, H2aa1	protein_coding
15270	NM_010436	H2afx	H2A histone family, member X	protein_coding
15273	NM_001358782,NM_001358783,NM_010437,XM_006512556,XM_006512558,XM_006512559,XM_011243129,XM_011243131,XM_011243132,XM_017313807,XM_030244891,XM_030244892,XM_030244893,XM_030244894,XM_030244895,XM_030244896,XM_030244897,XM_030244898,XM_030244899,XM_030244900,XM_030244901,XM_030244902,XM_030244903	Hivep2	human immunodeficiency virus type I enhancer binding protein 2	protein_coding
15275	NM_001146100,NM_010438,XM_006513242,XM_006513243,XM_006513244,XM_006513245,XM_006513246,XM_006513247,XM_030244904	Hk1	hexokinase 1	protein_coding
15277	NM_013820,XM_017321401	Hk2	hexokinase 2	protein_coding
15278	NM_001331054,NM_001331055,NM_008249,XM_006496666,XR_001781680	Tfb2m	transcription factor B2, mitochondrial	protein_coding
15284	NM_008250	Hlx	H2.0-like homeobox	protein_coding
15288	NM_001110251,NM_013551	Hmbs	hydroxymethylbilane synthase	protein_coding
15289	NM_001313894,NM_010439	Hmgb1	high mobility group box 1	protein_coding
15295	gene-Hmgb1-ps3	Hmgb1-ps3	high mobility group box 1, pseudogene 3	pseudogene
15296	gene-Hmgb1-rs16	Hmgb1-rs16	high mobility group box 1, related sequence 16	pseudogene
15312	NM_008251	Hmgn1	high mobility group nucleosomal binding domain 1	protein_coding
15331	NM_016957	Hmgn2	high mobility group nucleosomal binding domain 2	protein_coding
15353	NM_001163165,NM_001163166,NM_010440,XM_006513248,XM_006513249,XM_030244905	Hmg20b	high mobility group 20B	protein_coding
15354	NM_001293623,NM_001293624,NM_001293625,NM_008253,XM_006527842	Hmgb3	high mobility group box 3	protein_coding
15356	NM_001357529,NM_001357530,NM_008254,XM_006538564	Hmgcl	3-hydroxy-3-methylglutaryl-Coenzyme A lyase	protein_coding
15357	NM_001360165,NM_001360166,NM_008255	Hmgcr	3-hydroxy-3-methylglutaryl-Coenzyme A reductase	protein_coding
15360	NM_008256	Hmgcs2	3-hydroxy-3-methylglutaryl-Coenzyme A synthase 2	protein_coding
15361	NM_001025427,NM_001039356,NM_001166535,NM_001166536,NM_001166537,NM_001166539,NM_001166540,NM_001166541,NM_001166542,NM_001166543,NM_001166544,NM_001166545,NM_001166546,NM_016660,XM_030249514,XM_030249515	Hmga1	high mobility group AT-hook 1	protein_coding
15364	NM_001347170,NM_010441	Hmga2	high mobility group AT-hook 2	protein_coding
15365	NR_037996	Hmga2-ps1	high mobility group AT-hook 2, pseudogene 1	transcribed_pseudogene
15366	NM_013552,XM_006532276,XM_006532278,XM_006532279	Hmmr	hyaluronan mediated motility receptor (RHAMM)	protein_coding
15368	NM_010442	Hmox1	heme oxygenase 1	protein_coding
15369	NM_001136066,NM_001357050,NM_010443,XM_006521804	Hmox2	heme oxygenase 2	protein_coding
15370	NM_010444,XM_006520455,XM_006520456,XM_006520458,XM_006520459,XM_017316436,XM_030248339	Nr4a1	nuclear receptor subfamily 4, group A, member 1	protein_coding
15371	NM_001363144,NM_010445,XM_030254166	Hmx1	H6 homeobox 1	protein_coding
15372	NM_001368360,NM_145998	Hmx2	H6 homeobox 2	protein_coding
15374	NM_008258	Jpt1	Jupiter microtubule associated homolog 1	protein_coding
15375	NM_008259,XM_006515479,XM_006515481,XM_006515483,XM_017314962,XM_030246562	Foxa1	forkhead box A1	protein_coding
15376	NM_001291065,NM_001291067,NM_010446	Foxa2	forkhead box A2	protein_coding
15377	NM_008260	Foxa3	forkhead box A3	protein_coding
15378	NM_001312906,NM_001312907,NM_008261,XM_006498787,XM_006498788	Hnf4a	hepatic nuclear factor 4, alpha	protein_coding
15381	NM_001170981,NM_001170982,NM_001170983,NM_001170984,NM_001360172,NM_001360173,NM_001360174,NM_001360175,NM_001360176,NM_001360177,NM_001360178,NM_001360179,NM_001360180,NM_001360181,NM_001360182,NM_001360183,NM_001360184,NM_016884,XM_006518571,XM_017315850,XM_017315853,XM_017315855,XM_030247653	Hnrnpc	heterogeneous nuclear ribonucleoprotein C	protein_coding
15382	NM_001039129,NM_010447,NR_104427,XR_383983	Hnrnpa1	heterogeneous nuclear ribonucleoprotein A1	protein_coding
15384	NM_001048061,NM_010448	Hnrnpab	heterogeneous nuclear ribonucleoprotein A/B	protein_coding
15387	NM_001301340,NM_001301341,NM_001301343,NM_001301344,NM_001301345,NM_001360490,NM_001360491,NM_001360493,NM_001360494,NM_001360495,NM_025279,NR_153501,NR_153502,NR_153503,NR_153504,XM_006517103,XM_006517104	Hnrnpk	heterogeneous nuclear ribonucleoprotein K	protein_coding
15388	NM_177301,XM_006539556,XM_006539557,XM_006539558,XM_006539559,XM_006539560,XR_003946390,XR_003946391	Hnrnpl	heterogeneous nuclear ribonucleoprotein L	protein_coding
15394	NM_001311118,NM_010449,XM_006505607	Hoxa1	homeobox A1	protein_coding
15397	NR_015348	Hoxa11os	homeobox A11, opposite strand	lncRNA
15398	NM_008264	Hoxa13	homeobox A13	protein_coding
15399	NM_010451	Hoxa2	homeobox A2	protein_coding
15400	NM_010452,XM_011241225,XM_017321402,XM_017321403,XM_017321404	Hoxa3	homeobox A3	protein_coding
15401	NM_008265	Hoxa4	homeobox A4	protein_coding
15402	NM_010453	Hoxa5	homeobox A5	protein_coding
15403	NM_010454	Hoxa6	homeobox A6	protein_coding
15404	NM_010455	Hoxa7	homeobox A7	protein_coding
15407	NM_008266,XM_006532280	Hoxb1	homeobox B1	protein_coding
15410	NM_001079869,NM_010458,XM_006532283,XM_006532284,XM_006532285,XM_006532286,XM_006532288,XM_006532289,XM_006532290,XM_006532291,XM_011248757	Hoxb3	homeobox B3	protein_coding
15412	NM_010459	Hoxb4	homeobox B4	protein_coding
15416	NM_010461,XM_011248758,XM_017314295,XM_017314296,XM_017314297	Hoxb8	homeobox B8	protein_coding
15417	NM_008270	Hoxb9	homeobox B9	protein_coding
15423	NM_013553,XM_006520461,XM_006520462,XM_006520463,XM_011245460,XM_030248340	Hoxc4	homeobox C4	protein_coding
15424	NM_175730	Hoxc5	homeobox C5	protein_coding
15425	NM_010465,XM_006520468,XM_006520469,XM_017316437	Hoxc6	homeobox C6	protein_coding
15430	NM_013554,XM_006498789	Hoxd10	homeobox D10	protein_coding
15434	NM_010468	Hoxd3	homeobox D3	protein_coding
15436	NM_010469	Hoxd4	homeobox D4	protein_coding
15437	NM_001290730,NM_001290731,NM_008276	Hoxd8	homeobox D8	protein_coding
15438	NM_013555	Hoxd9	homeobox D9	protein_coding
15439	NM_001329965,NM_017370	Hp	haptoglobin	protein_coding
15441	NM_001122896,NM_001122897,NM_001285478,NM_001285479,NM_001285480,NM_001285481,NM_001356431,NM_001356432,NM_010470,XM_006538565,XM_006538566,XM_006538567,XM_006538568,XM_006538570,XM_030253217,XM_030253218,XM_030253219,XM_030253220,XM_030253221,XM_030253222,XM_030253223,XR_001784094,XR_003954880	Hp1bp3	heterochromatin protein 1, binding protein 3	protein_coding
15442	NM_152803	Hpse	heparanase	protein_coding
15444	NM_001130419,NM_001286081,NM_001286083,NM_010471,XM_006502775,XM_006502776,XM_006502777,XM_030253224,XM_030253225,XM_030253226,XM_030253227	Hpca	hippocalcin	protein_coding
15446	NM_008278	Hpgd	hydroxyprostaglandin dehydrogenase 15 (NAD)	protein_coding
15450	NM_001324472,NM_001324473,NM_008280,XM_006510816,XM_006510817,XM_006510818,XM_030244061,XM_030244062	Lipc	lipase, hepatic	protein_coding
15451	NM_001110252,NM_001276269,NM_008281	Hpn	hepsin	protein_coding
15452	NM_013556	Hprt	hypoxanthine guanine phosphoribosyl transferase	protein_coding
15458	NM_017371	Hpx	hemopexin	protein_coding
15460	NM_021877,XM_011244966	Hr	lysine demethylase and nuclear receptor corepressor	protein_coding
15461	NM_001130443,NM_001130444,NM_008284,XM_006536159,XM_030242155,XM_030242156,XR_003946392,XR_003946393,XR_003946394,XR_389950,XR_389951,XR_389952	Hras	Harvey rat sarcoma virus oncogene	protein_coding
15463	NM_001310713,NM_001347077,NM_001368850,NM_010472,XM_006496430,XM_006496431,XM_006496432,XM_006496434,XM_006496435,XM_006496438,XM_006496439,XM_006496440,XM_017316914,XM_030249597,XM_030249652,XM_030249680,XR_001781822,XR_001781825,XR_003952111,XR_003952114,XR_373537	Agfg1	ArfGAP with FG repeats 1	protein_coding
15464	NM_010473	Hrc	histidine rich calcium binding protein	protein_coding
15465	NM_001252642,NM_001252643,NM_001317124,NM_001317125,NM_001317126,NM_008285,XM_006505616,XM_030255192	Hrh1	histamine receptor H1	protein_coding
15466	NM_001010973,NR_153432,XM_006516852,XM_006516854,XM_017315397,XM_030247160	Hrh2	histamine receptor H2	protein_coding
15467	NM_013557,XM_017320685	Eif2ak1	eukaryotic translation initiation factor 2 alpha kinase 1	protein_coding
15468	NM_001077638,NM_001302965,NM_133182,NR_126571	Prmt2	protein arginine N-methyltransferase 2	protein_coding
15469	NM_001252476,NM_001252477,NM_019830,NR_045521,XM_030242157,XM_030242158	Prmt1	protein arginine N-methyltransferase 1	protein_coding
15473	NM_008287	Rida	reactive intermediate imine deaminase A homolog	protein_coding
15476	NM_010474,XM_017320686,XM_030254167	Hs3st1	heparan sulfate (glucosamine) 3-O-sulfotransferase 1	protein_coding
15478	NM_178870,XM_030245593	Hs3st3a1	heparan sulfate (glucosamine) 3-O-sulfotransferase 3A1	protein_coding
15481	NM_001364480,NM_031165	Hspa8	heat shock protein 8	protein_coding
15482	NM_013558	Hspa1l	heat shock protein 1-like	protein_coding
15483	NM_001044751,NM_008288,XM_006497227,XM_006497228,XM_030249832	Hsd11b1	hydroxysteroid 11-beta dehydrogenase 1	protein_coding
15484	NM_008289	Hsd11b2	hydroxysteroid 11-beta dehydrogenase 2	protein_coding
15485	NM_010475,XM_006532297	Hsd17b1	hydroxysteroid (17-beta) dehydrogenase 1	protein_coding
15486	NM_008290,XM_006530703	Hsd17b2	hydroxysteroid (17-beta) dehydrogenase 2	protein_coding
15488	NM_008292	Hsd17b4	hydroxysteroid (17-beta) dehydrogenase 4	protein_coding
15490	NM_010476,XM_006496667,XM_011238758,XM_017317108	Hsd17b7	hydroxysteroid (17-beta) dehydrogenase 7	protein_coding
15497	NM_013821	Hsd3b6	hydroxy-delta-5-steroid dehydrogenase, 3 beta- and steroid delta-isomerase 6	protein_coding
15499	NM_001331152,NM_001331153,NM_001331154,NM_001331214,NM_008296,NR_138588,NR_138589,XM_006520474,XM_006520475,XM_006520476,XM_030248342	Hsf1	heat shock factor 1	protein_coding
15500	NM_008297,XM_006512564,XM_006512565	Hsf2	heat shock factor 2	protein_coding
15502	NM_001164671,NM_001164672,NM_008298	Dnaja1	DnaJ heat shock protein family (Hsp40) member A1	protein_coding
15504	NM_008299	Dnajb3	DnaJ heat shock protein family (Hsp40) member B3	protein_coding
15505	NM_001347534,NM_013559,XM_006504916,XM_030254168	Hsph1	heat shock 105kDa/110kDa protein 1	protein_coding
15507	NM_013560	Hspb1	heat shock protein 1	protein_coding
15508	gene-Hsp25-ps1	Hsp25-ps1	heat shock protein 25, pseudogene 1	pseudogene
15510	NM_001356512,NM_010477	Hspd1	heat shock protein 1 (chaperonin)	protein_coding
15511	NM_010478	Hspa1b	heat shock protein 1B	protein_coding
15512	NM_001002012,NM_008301,XM_006515484	Hspa2	heat shock protein 2	protein_coding
15516	NM_008302	Hsp90ab1	heat shock protein 90 alpha (cytosolic), class B member 1	protein_coding
15519	NM_010480	Hsp90aa1	heat shock protein 90, alpha (cytosolic), class A member 1	protein_coding
15525	NM_008300	Hspa4	heat shock protein 4	protein_coding
15526	NM_010481	Hspa9	heat shock protein 9	protein_coding
15528	NM_008303	Hspe1	heat shock protein 1 (chaperonin 10)	protein_coding
15529	NM_008304	Sdc2	syndecan 2	protein_coding
15530	NM_008305,XM_006538573,XM_006538575,XM_030253228,XM_030253229	Hspg2	perlecan (heparan sulfate proteoglycan 2)	protein_coding
15531	NM_001348100,NM_001348101,NM_001348102,NM_001348103,NM_001348146,NM_008306,XM_006525668,XM_006525673,XM_030250340,XM_030250341,XM_030250342	Ndst1	N-deacetylase/N-sulfotransferase (heparan glucosaminyl) 1	protein_coding
15547	NM_001080999,NM_001081000,NM_001195205,NM_001357146,XM_006521805,XM_030248980,XM_030248981	Trmt2a	TRM2 tRNA methyltransferase 2A	protein_coding
15550	NM_008308	Htr1a	5-hydroxytryptamine (serotonin) receptor 1A	protein_coding
15551	NM_010482,XM_030244063	Htr1b	5-hydroxytryptamine (serotonin) receptor 1B	protein_coding
15552	NM_001285482,NM_001285483,NM_001285484,NM_008309,XM_006538577,XM_006538578,XM_017319986	Htr1d	5-hydroxytryptamine (serotonin) receptor 1D	protein_coding
15557	NM_008310	Htr1f	5-hydroxytryptamine (serotonin) receptor 1F	protein_coding
15558	NM_172812	Htr2a	5-hydroxytryptamine (serotonin) receptor 2A	protein_coding
15559	NM_008311,XM_006529145,XM_006529146,XM_030250037	Htr2b	5-hydroxytryptamine (serotonin) receptor 2B	protein_coding
15560	NM_008312,XM_030251228	Htr2c	5-hydroxytryptamine (serotonin) receptor 2C	protein_coding
15561	NM_001099644,NM_013561,XM_011242398	Htr3a	5-hydroxytryptamine (serotonin) receptor 3A	protein_coding
15562	NM_001364956,NM_001364957,NM_001364958,NM_001364959,NM_008313,XM_006525676,XM_006525678,XM_006525680,XM_006525681,XM_006525682,XM_006525683,XM_011246848,XM_030250343,XM_030250344,XM_030250345,XM_030250346,XM_030250347	Htr4	5 hydroxytryptamine (serotonin) receptor 4	protein_coding
15563	NM_008314,XM_006535631	Htr5a	5-hydroxytryptamine (serotonin) receptor 5A	protein_coding
15564	NM_010483,XM_017317180	Htr5b	5-hydroxytryptamine (serotonin) receptor 5B	protein_coding
15565	NM_021358,XM_011250196	Htr6	5-hydroxytryptamine (serotonin) receptor 6	protein_coding
15566	NM_001347442,NM_001360297,NM_001360298,NM_001360300,NM_001360318,NM_008315,XM_030250756	Htr7	5-hydroxytryptamine (serotonin) receptor 7	protein_coding
15567	NM_010484	Slc6a4	solute carrier family 6 (neurotransmitter transporter, serotonin), member	protein_coding
15568	NM_010485,XM_006508698,XM_030243301,XM_030243302	Elavl1	ELAV (embryonic lethal, abnormal vision)-like 1 (Hu antigen R)	protein_coding
15569	NM_001177883,NM_001347149,NM_001357188,NM_001357189,NM_010486,NM_207685,NM_207686,XM_006502778,XM_006502779,XM_006502781,XM_006502784,XM_006502785,XM_006502787,XM_006502788,XM_006502789,XM_006502790,XM_006502791,XM_011240448,XM_017319987,XM_017319989,XM_017319990,XM_017319991,XM_030253231,XM_030253232,XM_030253233,XM_030253234,XM_030253235,XM_030253236,XM_030253237,XM_030253238,XM_030253239,XM_030253240,XM_030253241,XM_030253242,XM_030253243,XM_030253244,XM_030253245,XM_030253246,XM_030253247,XR_003954881	Elavl2	ELAV like RNA binding protein 1	protein_coding
15571	NM_010487,XM_006510021,XM_006510022,XM_030244064	Elavl3	ELAV like RNA binding protein 3	protein_coding
15572	NM_001038698,NM_001163397,NM_001163399,NM_001347178,NM_001357190,NM_010488,XM_006502793,XM_006502794,XM_006502795,XM_006502796,XM_006502797,XM_006502798,XM_006502799,XM_006502800,XM_006502801,XM_006502802,XM_006502803,XM_030253248,XM_030253249,XM_030253250,XM_030253251,XM_030253252,XM_030253253,XM_030253254,XM_030253255,XM_030253256,XM_030253257,XM_030253258,XM_030253259,XM_030253260	Elavl4	ELAV like RNA binding protein 4	protein_coding
15574	NM_001303532,NM_001303610,NM_008316,NR_130178,XM_006514531	Hus1	HUS1 checkpoint clamp component	protein_coding
15586	NM_001331161,NM_008317	Hyal1	hyaluronoglucosaminidase 1	protein_coding
15587	NM_010489,XM_006511643,XM_006511644,XM_006511645	Hyal2	hyaluronoglucosaminidase 2	protein_coding
15891	NM_008318	Ibsp	integrin binding sialoprotein	protein_coding
15893	NM_001252266,NM_010492,XM_006504993,XM_006504994,XM_006504995,XM_017321405,XM_030255193,XR_001785098,XR_001785099,XR_003956165	Ica1	islet cell autoantigen 1	protein_coding
15894	NM_010493	Icam1	intercellular adhesion molecule 1	protein_coding
15896	NM_010494,XM_006532303,XM_011248761,XM_011248762,XM_011248763,XM_030245594,XM_030245595	Icam2	intercellular adhesion molecule 2	protein_coding
15898	NM_008319	Icam5	intercellular adhesion molecule 5, telencephalin	protein_coding
15900	NM_001301811,NM_008320	Irf8	interferon regulatory factor 8	protein_coding
15901	NM_001355113,NM_001369018,NM_010495	Id1	inhibitor of DNA binding 1, HLH protein	protein_coding
15902	NM_010496	Id2	inhibitor of DNA binding 2	protein_coding
15903	NM_008321	Id3	inhibitor of DNA binding 3	protein_coding
15904	NM_031166	Id4	inhibitor of DNA binding 4	protein_coding
15925	NM_031156	Ide	insulin degrading enzyme	protein_coding
15926	NM_001111320,NM_010497	Idh1	isocitrate dehydrogenase 1 (NADP+), soluble	protein_coding
15929	NM_001356356,NM_008323,XM_006527844,XM_017318392	Idh3g	isocitrate dehydrogenase 3 (NAD+), gamma	protein_coding
15931	NM_010498,XM_006527845,XM_006527846,XM_017318393,XM_017318394,XM_030251229,XM_030251230,XM_030251231,XM_030251232,XM_030251233	Ids	iduronate 2-sulfatase	protein_coding
15932	NM_008325,NM_172997,XM_006534797,XM_006534798,XM_006534799,XR_389260	Idua	iduronidase, alpha-L	protein_coding
15936	NM_010499	Ier2	immediate early response 2	protein_coding
15937	NM_133662	Ier3	immediate early response 3	protein_coding
15939	NM_010500	Ier5	immediate early response 5	protein_coding
15944	NM_001355757,NM_008326,XM_011248764,XM_030245596,XM_030245597	Irgm1	immunity-related GTPase family M member 1	protein_coding
15945	NM_021274	Cxcl10	chemokine (C-X-C motif) ligand 10	protein_coding

15950	NM_001302649,NM_001302650,NM_001302651,XM_006496669,XM_006496671,XM_011238760,XM_011238761,XM_011238762,XM_030250187,XM_030250201,XM_030250215,XM_030250226,XM_030250243,XM_030250260	Ifi203	interferon activated gene 203	protein_coding
15951	NM_008329,XM_006496675	Ifi204	interferon activated gene 204	protein_coding
15953	NM_001271676,NM_001271677,NM_008330	Ifi47	interferon gamma inducible protein 47	protein_coding
15957	NM_008331	Ifit1	interferon-induced protein with tetratricopeptide repeats 1	protein_coding
15958	NM_001355262,NM_008332,XM_006526705	Ifit2	interferon-induced protein with tetratricopeptide repeats 2	protein_coding
15959	NM_010501,XM_011247151,XM_011247152,XM_011247153,XM_030250757	Ifit3	interferon-induced protein with tetratricopeptide repeats 3	protein_coding
15965	NM_010503	Ifna2	interferon alpha 2	protein_coding
15968	NM_010505	Ifna5	interferon alpha 5	protein_coding
15975	NM_010508,XR_384931	Ifnar1	interferon (alpha and beta) receptor 1	protein_coding
15976	NM_001110498,NM_001347258,NM_010509,XM_011246100	Ifnar2	interferon (alpha and beta) receptor 2	protein_coding
15978	NM_008337	Ifng	interferon gamma	protein_coding
15979	NM_010511	Ifngr1	interferon gamma receptor 1	protein_coding
15980	NM_008338	Ifngr2	interferon gamma receptor 2	protein_coding
15982	NM_013562,XM_030246563	Ifrd1	interferon-related developmental regulator 1	protein_coding
15983	NM_025903	Ifrd2	interferon-related developmental regulator 2	protein_coding
15985	NM_001313939,NM_008339	Cd79b	CD79B antigen	protein_coding
16000	NM_001111274,NM_001111275,NM_001111276,NM_001314010,NM_010512,XM_006513255,XM_006513257,XM_006513258,XM_006513261,XM_006513262,XM_006513264,XM_017313810,XM_017313811,XM_017313812,XM_030244906,XM_030244907,XM_030244908,XM_030244909,XM_030244910,XM_030244911,XM_030244912	Igf1	insulin-like growth factor 1	protein_coding
16001	NM_010513,XM_006540641,XM_006540642,XM_006540643,XM_006540644,XM_006540645,XM_017321986	Igf1r	insulin-like growth factor I receptor	protein_coding
16002	NM_001122736,NM_001122737,NM_001315488,NM_001315489,NM_010514,XM_006508487,XM_030242159	Igf2	insulin-like growth factor 2	protein_coding
16004	NM_010515	Igf2r	insulin-like growth factor 2 receptor	protein_coding
16005	NM_001364895,NM_001364896,NM_008340,XM_030249516	Igfals	insulin-like growth factor binding protein, acid labile subunit	protein_coding
16006	NM_008341	Igfbp1	insulin-like growth factor binding protein 1	protein_coding
16007	NM_010516	Ccn1	cellular communication network factor 1	protein_coding
16008	NM_001310659,NM_008342,XM_030250357	Igfbp2	insulin-like growth factor binding protein 2	protein_coding
16009	NM_008343,XM_011243665	Igfbp3	insulin-like growth factor binding protein 3	protein_coding
16010	NM_010517,XM_030245598,XM_030245599	Igfbp4	insulin-like growth factor binding protein 4	protein_coding
16011	NM_010518	Igfbp5	insulin-like growth factor binding protein 5	protein_coding
16012	NM_008344	Igfbp6	insulin-like growth factor binding protein 6	protein_coding
16019	id-Ighm	Ighm	immunoglobulin heavy constant mu	C_region
16068	NM_001363982,NM_010531,XM_017321987,XM_030242160,XM_030242161	Il18bp	interleukin 18 binding protein	protein_coding
16069	NM_152839	Jchain	immunoglobulin joining chain	protein_coding
16071	id-Igkc	Igkc	immunoglobulin kappa constant	C_region
16098	id-Igkv1-117	Igkv1-117	immunoglobulin kappa variable 1-117	V_segment
16136	NM_001190325	Igll1	immunoglobulin lambda-like polypeptide 1	protein_coding
16142	id-Iglv1	Iglv1	immunoglobulin lambda variable 1	V_segment
16145	NM_018738	Igtp	interferon gamma induced GTPase	protein_coding
16147	NM_001313683,NM_010544	Ihh	Indian hedgehog	protein_coding
16149	NM_001042605,NM_010545	Cd74	CD74 antigen (invariant polypeptide of major histocompatibility complex, class II antigen-associated)	protein_coding
16150	NM_001159774,NM_010546,XM_006509019,XM_006509020,XM_006509022,XM_006509023,XM_030243303,XR_003947235	Ikbkb	inhibitor of kappaB kinase beta	protein_coding
16151	NM_001136067,NM_001161421,NM_001161422,NM_001161423,NM_001161424,NM_010547,NM_178590,XM_006527847,XM_006527848,XM_006527849,XM_006527850,XM_006527851,XM_006527852,XM_006527853,XM_011247527	Ikbkg	inhibitor of kappaB kinase gamma	protein_coding
16153	NM_010548	Il10	interleukin 10	protein_coding
16154	NM_001324486,NM_008348,NR_136903	Il10ra	interleukin 10 receptor, alpha	protein_coding
16155	NM_008349,XM_006522924	Il10rb	interleukin 10 receptor, beta	protein_coding
16156	NM_001290423,NM_008350,XM_017321988,XM_030242162,XM_030242163,XM_030242164	Il11	interleukin 11	protein_coding
16157	NM_001163401,NM_001172054,NM_010549	Il11ra1	interleukin 11 receptor, alpha chain 1	protein_coding
16158	NM_010550,XM_006537625	Il11ra2	interleukin 11 receptor, alpha chain 2	protein_coding
16159	NM_001159424,NM_008351,XM_006501044	Il12a	interleukin 12a	protein_coding
16160	NM_001303244	Il12b	interleukin 12b	protein_coding
16161	NM_008353,XM_006509557,XM_006509558,XM_006509559,XM_006509560,XM_006509561,XM_006509562,XM_011242266	Il12rb1	interleukin 12 receptor, beta 1	protein_coding
16162	NM_001311141,NM_008354,XM_006505617	Il12rb2	interleukin 12 receptor, beta 2	protein_coding
16163	NM_008355	Il13	interleukin 13	protein_coding
16164	NM_133990	Il13ra1	interleukin 13 receptor, alpha 1	protein_coding
16165	NM_001306059,NM_008356,XM_006528707,XM_006528708,XM_017318395,XM_030251234	Il13ra2	interleukin 13 receptor, alpha 2	protein_coding
16168	NM_001254747,NM_008357,XM_006530705,XM_006530706,XM_006530707,XM_006530708,XM_006530709,XM_006530710,XM_006530711,XM_006530712,XM_006530713,XM_006530714,XM_006530715,XM_011248312,XM_011248313,XM_011248314,XM_030243304	Il15	interleukin 15	protein_coding
16169	NM_001271497,NM_001271498,NM_001271499,NM_001271500,NM_001271501,NM_008358,NM_133836,NR_073191,NR_073192,XM_006497358,XM_006497359,XM_006497360,XM_006497362,XM_006497364,XM_011238959,XM_017315695,XM_030247643,XM_030247644,XM_030247646,XR_003950824,XR_865924,XR_865925,XR_865926	Il15ra	interleukin 15 receptor, alpha chain	protein_coding
16170	NM_001360087,NM_001360088,NM_001360089,NM_010551,XM_006507385,XM_006507386,XM_011241680,XM_011241681,XR_001785481	Il16	interleukin 16	protein_coding
16172	NM_008359,XM_006505618	Il17ra	interleukin 17 receptor A	protein_coding
16173	NM_001357221,NM_001357222,NM_008360,XM_006510024,XM_017313137,XM_030244065,XM_030244066,XM_030244067	Il18	interleukin 18	protein_coding
16174	NM_010553,XM_006495710,XM_006495712,XM_030250547,XR_003952534	Il18rap	interleukin 18 receptor accessory protein	protein_coding
16175	NM_010554,XM_006498793,XM_006498794	Il1a	interleukin 1 alpha	protein_coding
16176	NM_008361,XM_006498795	Il1b	interleukin 1 beta	protein_coding
16177	NM_001123382,NM_008362,XM_006495713,XM_006495714,XM_006495715,XM_006495719,XM_006495720,XM_006495721,XM_006495722,XM_006495723,XM_011238436,XM_017317595,XM_017317613,XM_030250619	Il1r1	interleukin 1 receptor, type I	protein_coding
16178	NM_001360800,NM_001360801,NM_010555,XM_006495726,XM_011238437,XM_011238438,XM_011238439,XM_011238440,XM_017317722,XM_030250734,XM_030250758,XR_003952762,XR_003952769,XR_003952776	Il1r2	interleukin 1 receptor, type II	protein_coding
16179	NM_001177973,NM_001177974,NM_001177975,NM_001177976,NM_008363	Irak1	interleukin-1 receptor-associated kinase 1	protein_coding
16180	NM_001159317,NM_001159318,NM_008364,NM_134103,NR_152416,XR_875736	Il1rap	interleukin 1 receptor accessory protein	protein_coding
16181	NM_001039701,NM_001159562,NM_031167,XM_006497727	Il1rn	interleukin 1 receptor antagonist	protein_coding
16182	NM_001161842,NM_001161843,NM_008365,XM_006495727,XM_006495728,XM_006495729,XM_006495730,XM_006495732,XM_006495734	Il18r1	interleukin 18 receptor 1	protein_coding
16184	NM_008367	Il2ra	interleukin 2 receptor, alpha chain	protein_coding
16185	NM_008368,XM_006520477,XM_006520478,XM_006520479,XM_006520480,XM_006520481	Il2rb	interleukin 2 receptor, beta chain	protein_coding
16186	NM_001308535,NM_013563,XM_017318396	Il2rg	interleukin 2 receptor, gamma chain	protein_coding
16188	NM_008369,XM_006517935,XM_006517940,XM_006517941,XM_006517942,XM_006517946,XM_006517947,XM_017315860,XM_017315861,XM_017315862,XM_017315863,XM_017315864,XM_017315865,XM_017315866,XM_017315867,XM_017315868,XM_030247654,XM_030247655,XM_030247656,XM_030247657	Il3ra	interleukin 3 receptor, alpha chain	protein_coding
16189	NM_021283,NR_027491	Il4	interleukin 4	protein_coding
16190	NM_001008700,NM_001363983	Il4ra	interleukin 4 receptor, alpha	protein_coding
16191	NM_010558	Il5	interleukin 5	protein_coding
16192	NM_008370,XM_017321406,XM_030255194	Il5ra	interleukin 5 receptor, alpha	protein_coding
16193	NM_001314054,NM_031168	Il6	interleukin 6	protein_coding
16194	NM_001310676,NM_010559	Il6ra	interleukin 6 receptor, alpha	protein_coding
16195	NM_010560	Il6st	interleukin 6 signal transducer	protein_coding
16196	NM_001313888,NM_001313889,NM_001313890,NM_008371	Il7	interleukin 7	protein_coding
16197	NM_001355680,NM_008372,XR_003951356	Il7r	interleukin 7 receptor	protein_coding
16199	NM_001134458,NM_008374,XM_006514533,XM_006514534,XM_006514537,XM_006514538,XM_006514539,XM_006514540,XM_017314298	Il9r	interleukin 9 receptor	protein_coding
16201	NM_001042707,NM_001042708,NM_001042709,NM_001277321,NM_001277322,NM_010561,XM_006510029,XM_006510030,XM_006510031,XM_006510034,XM_006510036,XM_006510037,XM_011242403,XM_011242404,XM_011242405,XM_017313138,XM_017313139,XM_017313140,XM_017313141,XM_017313142,XM_017313143,XM_017313145,XM_017313146,XM_017313147,XM_017313148,XM_017313149,XM_017313150,XM_017313152,XM_030244068,XM_030244069,XM_030244070,XM_030244071,XM_030244072,XM_030244073,XR_001778801,XR_001778802,XR_001778803,XR_001778804,XR_003947786,XR_003947787,XR_003947788,XR_0039	Ilf3	interleukin enhancer binding factor 3	protein_coding
16202	NM_001161724,NM_010562	Ilk	integrin linked kinase	protein_coding
16205	NM_008376,NM_175860	Gimap1	GTPase, IMAP family member 1	protein_coding
16206	NM_001355269,NM_008377,XM_006505622,XM_030255195,XM_030255196,XM_030255197,XM_030255198,XR_377425	Lrig1	leucine-rich repeats and immunoglobulin-like domains 1	protein_coding
16210	NM_001357396,NM_008378,XM_011246850,XR_001782344	Impact	impact, RWD domain protein	protein_coding
16211	NM_008379	Kpnb1	karyopherin (importin) beta 1	protein_coding
16319	NM_001369356,NM_016692,XM_006526707,XM_006526708,XM_006526710,XM_011247154	Incenp	inner centromere protein	protein_coding
16322	NM_001329843,NM_010564,XM_011238675	Inha	inhibin alpha	protein_coding
16323	NM_008380,XM_006516559,XM_011244285	Inhba	inhibin beta-A	protein_coding
16324	NM_008381	Inhbb	inhibin beta-B	protein_coding
16325	NM_010565	Inhbc	inhibin beta-C	protein_coding
16328	NM_001129999,NM_001130000,NM_008383,NM_177217,XM_006498796,XM_006498797,XM_006498798,XM_006498799,XM_006498802,XM_017315701,XM_030247663,XM_030247666,XM_030247667	Cep250	centrosomal protein 250	protein_coding
16329	NM_008384,XM_011238441,XM_011238442	Inpp1	inositol polyphosphate-1-phosphatase	protein_coding
16330	NM_008385,NR_126474,XM_006502804,XM_006502805,XR_003954882	Inpp5b	inositol polyphosphate-5-phosphatase B	protein_coding
16331	NM_001110192,NM_001110193,NM_010566,NR_122078,XM_006529149,XM_006529150,XM_006529151,XM_006529154,XM_030251158,XR_001782395,XR_003952973	Inpp5d	inositol polyphosphate-5-phosphatase D	protein_coding
16332	NM_001122739,NM_010567,XM_011241682,XM_030242165,XR_003946395	Inppl1	inositol polyphosphate phosphatase-like 1	protein_coding
16336	NM_013564	Insl3	insulin-like 3	protein_coding
16337	NM_001330056,NM_010568,XM_006508700,XM_006508701	Insr	insulin receptor	protein_coding
16341	NM_008388	Eif3e	eukaryotic translation initiation factor 3, subunit E	protein_coding
16342	gene-Eif3s6-ps1	Eif3s6-ps1	eukaryotic translation initiation factor 3, subunit 6, pseudogene 1	pseudogene
16343	gene-Eif3s6-ps2	Eif3s6-ps2	eukaryotic translation initiation factor 3, subunit 6, pseudogene 2	pseudogene
16344	gene-Eif3s6-ps3	Eif3s6-ps3	eukaryotic translation initiation factor 3, subunit 6, pseudogene 3	pseudogene
16348	NM_001281977,NM_001281978,NM_010569,XM_006537626,XM_006537629,XM_006537630,XM_006537631,XM_006537632,XM_006537633,XM_006537634,XM_006537635,XM_006537637,XM_011249937,XM_017319994,XM_030253261	Invs	inversin	protein_coding
16351	NM_008389,XM_006502810,XM_006502811	Ipp	IAP promoted placental gene	protein_coding
16362	NM_001159393,NM_001159396,NM_008390,XM_030245600	Irf1	interferon regulatory factor 1	protein_coding
16363	NM_008391,NR_152560,XM_006509288,XM_006509289,XM_011242175,XM_017312580,XM_030243305,XM_030243306,XR_001778	Irf2	interferon regulatory factor 2	protein_coding
16364	NM_001347508,NM_013674	Irf4	interferon regulatory factor 4	protein_coding
16367	NM_010570	Irs1	insulin receptor substrate 1	protein_coding
16369	NM_010571	Irs3	insulin receptor substrate 3	protein_coding
16370	NM_010572	Irs4	insulin receptor substrate 4	protein_coding
16371	NM_010573	Irx1	Iroquois homeobox 1	protein_coding
16372	NM_010574,XM_030247163,XM_030247164	Irx2	Iroquois homeobox 2	protein_coding
16373	NM_001253822,NM_008393	Irx3	Iroquois related homeobox 3	protein_coding
16391	NM_001159417,NM_001159418,NM_008394	Irf9	interferon regulatory factor 9	protein_coding
16392	NM_021459	Isl1	ISL1 transcription factor, LIM/homeodomain	protein_coding
16396	NM_001243712,NM_008395,XR_001781068,XR_001781069,XR_003950826	Itch	itchy, E3 ubiquitin protein ligase	protein_coding
16398	NM_008396	Itga2	integrin alpha 2	protein_coding
16399	NM_010575	Itga2b	integrin alpha 2b	protein_coding
16400	NM_001306071,NM_001306162,NM_013565,XM_006532310,XM_006532311	Itga3	integrin alpha 3	protein_coding
16401	NM_010576	Itga4	integrin alpha 4	protein_coding
16402	NM_001314041,NM_010577	Itga5	integrin alpha 5 (fibronectin receptor alpha)	protein_coding
16403	NM_001277970,NM_008397,XM_011239308,XM_011239309,XM_017315707	Itga6	integrin alpha 6	protein_coding
16404	NM_001330160,NM_008398,XM_006513269,XM_006513270,XM_017313813,XM_030244913,XM_030244914,XM_030244915,XM_030244916	Itga7	integrin alpha 7	protein_coding
16407	NM_001361245,NM_008399,XM_017314299	Itgae	integrin alpha E, epithelial-associated	protein_coding
16408	NM_001253872,NM_001253873,NM_001253874,NM_008400,XM_006507393	Itgal	integrin alpha L	protein_coding
16409	NM_001082960,NM_008401	Itgam	integrin alpha M	protein_coding
16410	NM_008402,XM_006498807	Itgav	integrin alpha V	protein_coding
16411	NM_001363984,NM_001363985,NM_021334,XR_378219	Itgax	integrin alpha X	protein_coding
16412	NM_010578	Itgb1	integrin beta 1 (fibronectin receptor beta)	protein_coding
16413	NM_001355609,NM_008403,XM_006514989,XM_006514990,XM_011243816	Itgb1bp1	integrin beta 1 binding protein 1	protein_coding
16414	NM_008404,NR_157228,NR_157229,XM_006513271,XM_006513272,XM_017313814	Itgb2	integrin beta 2	protein_coding
16415	NM_008405,XM_006522925,XM_006522928,XM_006522929,XM_006522930	Itgb2l	integrin beta 2-like	protein_coding
16416	NM_016780,XM_006532312	Itgb3	integrin beta 3	protein_coding
16418	NM_010579	Eif6	eukaryotic translation initiation factor 6	protein_coding
16419	NM_001145884,NM_010580,XM_030248983	Itgb5	integrin beta 5	protein_coding
16420	NM_001159564,NM_021359,XM_006498808,XM_006498809,XM_006498810,XM_006498811	Itgb6	integrin beta 6	protein_coding
16421	NM_013566,XM_006520482,XM_017316440	Itgb7	integrin beta 7	protein_coding
16423	NM_001368415,NM_001368416,NM_001368417,NM_001368418,NM_010581,XM_006521809,XM_006521810,XM_030248984,XM_030248985,XM_030248986,XR_003951762,XR_003951763,XR_3845	Cd47	CD47 antigen (Rh-related antigen, integrin-associated signal transducer)	protein_coding
16424	NM_001306078,NM_008406,XM_006518584	Itih1	inter-alpha trypsin inhibitor, heavy chain 1	protein_coding
16425	NM_010582	Itih2	inter-alpha trypsin inhibitor, heavy chain 2	protein_coding
16426	NM_008407,XM_006518585,XM_006518586,XM_006518587,XM_006518588,XR_003950825	Itih3	inter-alpha trypsin inhibitor, heavy chain 3	protein_coding
16427	NM_001159299,NM_001289632,NM_001289633,NM_018746	Itih4	inter alpha-trypsin inhibitor, heavy chain 4	protein_coding
16428	NM_001281965,NM_001281966,NM_001281967,NM_001281968,NM_010583	Itk	IL2 inducible T cell kinase	protein_coding
16429	NM_010584,XM_006496676	Itln1	intelectin 1 (galactofuranose binding)	protein_coding
16430	NM_008408,XM_030244074	Stt3a	STT3, subunit of the oligosaccharyltransferase complex, homolog A (S. cerevisiae)	protein_coding
16431	NM_008409	Itm2a	integral membrane protein 2A	protein_coding
16432	NM_008410	Itm2b	integral membrane protein 2B	protein_coding
16433	NM_008411	Cuzd1	CUB and zona pellucida-like domains 1	protein_coding
16434	NM_001362648,NM_025922,XM_017315731	Itpa	inosine triphosphatase (nucleoside triphosphate pyrophosphatase)	protein_coding
16438	NM_010585,XM_006505623,XM_006505624,XM_006505625,XM_006505626,XM_006505627,XM_006505628,XM_006505629,XM_006505630,XM_006505631,XM_006505632,XM_006505633,XM_006505636,XM_006505637,XM_017321407,XM_017321408,XM_017321409,XM_017321410,XM_030255199	Itpr1	inositol 1,4,5-trisphosphate receptor 1	protein_coding
16439	NM_010586,NM_019923,XM_006507026,XM_017321411	Itpr2	inositol 1,4,5-triphosphate receptor 2	protein_coding
16440	NM_080553,XM_006523712	Itpr3	inositol 1,4,5-triphosphate receptor 3	protein_coding
16443	NM_001110275,NM_001110276,NM_010587,XM_006522931,XM_006522932,XM_006522933,XM_006522934,XM_006522935,XM_006522936,XM_006522937,XM_006522938,XM_006522939,XM_006522940,XM_011246102,XR_001781793	Itsn1	intersectin 1 (SH3 domain protein 1A)	protein_coding
16449	NM_013822,XM_011239312	Jag1	jagged 1	protein_coding
16450	NM_010588,XM_006515486,XM_011243998,XM_011243999,XM_011244000,XR_872705,XR_872706,XR_872707	Jag2	jagged 2	protein_coding
16451	NM_013567,NM_146145,XM_006502812,XM_030253262,XM_030253263,XM_030253264,XM_030253265	Jak1	Janus kinase 1	protein_coding

16452	NM_001048177,NM_008413,XM_006526713,XM_006526714,XM_006526715,XM_011247155,XM_017318068	Jak2	Janus kinase 2
16453	NM_001190830,NM_010589	Jak3	Janus kinase 3
16456	NM_172647	F11r	F11 receptor
16467	NM_178662,XM_030244918	Atcay	ataxia, cerebellar, Cayman type
16468	NM_001205043,NM_001205044,NM_001360281,NM_021878,XM_006516858,XM_006516859,XM_006516860	Jarid2	jumonji, AT rich interactive domain 2
16469	NM_008415	Jrk	jerky
16470	NM_176847,XM_017314300,XM_030245601,XM_030245602,XM_030245603,XM_030245604	Ush1g	USH1 protein network component sans
16475	NM_010590	Ajuba	ajuba LIM protein
16476	NM_010591	Jun	jun proto-oncogene
16477	NM_008416	Junb	jun B proto-oncogene
16478	NM_001286944,NM_010592	Jund	jun D proto-oncogene
16480	NM_010593,XM_006532315,XM_006532316,XM_030245605	Jup	junction plakoglobin
16485	NM_010595	Kcna1	potassium voltage-gated channel, shaker-related subfamily, member 1
16490	NM_008417,XM_006501048,XM_006501049,XM_006501050,XM_006501051,XM_006501052,XM_006501053,XM_006501055,XM_030252437	Kcna2	potassium voltage-gated channel, shaker-related subfamily, member 2
16491	NM_008418	Kcna3	potassium voltage-gated channel, shaker-related subfamily, member 3
16492	NM_021275,XM_006498812	Kcna4	potassium voltage-gated channel, shaker-related subfamily, member 4
16493	NM_145983	Kcna5	potassium voltage-gated channel, shaker-related subfamily, member 5
16494	NM_013568,XM_006505638,XM_006505639,XM_011241228	Kcna6	potassium voltage-gated channel, shaker-related, subfamily, member 6
16495	NM_010596	Kcna7	potassium voltage-gated channel, shaker-related subfamily, member 7
16497	NM_001289450,NM_001357358,NM_010597,XM_006501057	Kcnab1	potassium voltage-gated channel, shaker-related subfamily, beta member
16498	NM_001252654,NM_001252655,NM_001252656,NM_001355172,NM_001355173,NM_010598,XM_006538584,XM_006538585,XM_006538586,XM_006538587,XM_011250197,XM_011250198,XM_011250199,XM_017319997,XM_030253266,XM_030253267	Kcnab2	potassium voltage-gated channel, shaker-related subfamily, beta member 2
16499	NM_010599	Kcnab3	potassium voltage-gated channel, shaker-related subfamily, beta member
16500	NM_008420,XM_017315732,XM_017315733,XR_001781070,XR_374417,XR_374418	Kcnb1	potassium voltage gated channel, Shab-related subfamily, member 1
16502	NM_001112739,NM_008421,XM_006540646,XM_006540647,XM_006540648	Kcnc1	potassium voltage gated channel, Shaw-related subfamily, member 1
16504	NM_001290682,NM_008422,XM_006540654,XM_006540655,XM_006540656,XM_006540658,XM_006540659,XM_006540660,XM_006540661,XM_017321992,XM_017321993,XM_017321994,XM_017321995,XM_017321996,XM_017321997,XM_030242166,XM_030242167,XM_030242168,XM_030242169,XM_030242170,XM_030242171,XR_001785482,XR_003946396,XR_003946397,XR_003946398,XR_003946399,XR_003946400,XR_003946401,XR_391319	Kcnc3	potassium voltage gated channel, Shaw-related subfamily, member 3
16506	NM_008423	Kcnd1	potassium voltage-gated channel, Shal-related family, member 1
16508	NM_019697	Kcnd2	potassium voltage-gated channel, Shal-related family, member 2
16509	NM_001362456,NM_008424	Kcne1	potassium voltage-gated channel, Isk-related subfamily, member 1
16510	NM_001038607,NM_010600,XM_006497229,XR_373887	Kcnh1	potassium voltage-gated channel, subfamily H (eag-related), member 1
16511	NM_001294162,NM_013569,XM_006535632,XM_006535633,XM_006535634,XM_006535635,XR_389740	Kcnh2	potassium voltage-gated channel, subfamily H (eag-related), member 2
16512	NM_010601,XM_006520483,XM_006520484,XM_030248343,XM_030248344	Kcnh3	potassium voltage-gated channel, subfamily H (eag-related), member 3
16513	NM_001039484,XM_006496677	Kcnj10	potassium inwardly-rectifying channel, subfamily J, member 10
16514	NM_001204411,NM_010602,XM_006540663	Kcnj11	potassium inwardly rectifying channel, subfamily J, member 11
16515	NM_001267593,NM_010603,XM_006532318,XM_030245606	Kcnj12	potassium inwardly-rectifying channel, subfamily J, member 12
16516	NM_001039056,NM_001039057,NM_001271687,NM_001271689,NM_001271690,NM_001271691,NM_001271692,NM_001271693,NM_001271694,NM_001271695,NM_019664,NR_073406,XM_006522942,XM_006522944,XM_006522945,XM_006522946,XM_006522947,XM_006522950,XM_006522951,XM_006522952,XM_017316883,XM_017316884,XM_017316885	Kcnj15	potassium inwardly-rectifying channel, subfamily J, member 15
16517	NM_001252207,NM_001252208,NM_001252209,NM_001252210,NM_010604	Kcnj16	potassium inwardly-rectifying channel, subfamily J, member 16
16518	NM_008425	Kcnj2	potassium inwardly-rectifying channel, subfamily J, member 2
16519	NM_001304810,NM_001355118,NM_008426,XM_006497731,XM_030247722,XM_030247723,XM_030247725,XM_030247729,XM_030247730,XM_030247736,XM_030247737,XM_030247738,XM_030247739,XM_030247740,XM_030247742,XM_030247749,XM_030247750,XM_030247751,XM_030247752	Kcnj3	potassium inwardly-rectifying channel, subfamily J, member 3
16520	NM_008427,XM_006520486,XM_006520487,XM_006520488	Kcnj4	potassium inwardly-rectifying channel, subfamily J, member 4
16521	NM_010605,NR_157190,XM_006510039,XM_017313153,XM_017313154,XM_017313155	Kcnj5	potassium inwardly-rectifying channel, subfamily J, member 5
16522	NM_001025584,NM_001025585,NM_001025590,NM_010606,XM_011246104	Kcnj6	potassium inwardly-rectifying channel, subfamily J, member 6
16523	NM_001330363,NM_001330366,NM_008428	Kcnj8	potassium inwardly-rectifying channel, subfamily J, member 8
16524	NM_001360808,NM_008429	Kcnj9	potassium inwardly-rectifying channel, subfamily J, member 9
16525	NM_008430,XM_006530716	Kcnk1	potassium channel, subfamily K, member 1
16526	NM_001159850,NM_001281847,NM_001281848,NM_001357119,NM_010607,XM_006497121,XM_006497122,XM_030251361	Kcnk2	potassium channel, subfamily K, member 2
16527	NM_010608	Kcnk3	potassium channel, subfamily K, member 3
16528	NM_008431,XM_006526716,XM_006526717,XM_006526718,XM_006526719,XM_006526720,XM_006526721	Kcnk4	potassium channel, subfamily K, member 4
16529	NM_021542,XM_006518590	Kcnk5	potassium channel, subfamily K, member 5
16530	NM_010609,XM_006531666	Kcnk7	potassium channel, subfamily K, member 7
16531	NM_001253358,NM_001253359,NM_001253360,NM_001253361,NM_001253362,NM_001253363,NM_001253364,NM_001253365,NM_001253366,NM_001253367,NM_001253368,NM_001253369,NM_001253370,NM_001253371,NM_001253372,NM_001253373,NM_001253374,NM_001253375,NM_001253376,NM_001253377,NM_001253378,NM_010610,XM_006518591,XM_006518592,XM_006518593,XM_006518594,XM_006518595,XM_006518599,XM_006518602,XM_006518603,XM_006518605,XM_006518606,XM_006518609,XM_006518610,XM_006518613,XM_011244967,XM_017315870,XM_017315871,XM_017315872,XM_017315873,XM_017315874,XM_017315875,XM_017315876,XM_017315878,XM_017315880,XM_017315881,XM_017315883,XM_017315884,XM_017315885,XM_017315886,XM_017315887,XM_030247658,XM_030247659,XM_030247660,XM_030247661	Kcnma1	potassium large conductance calcium-activated channel, subfamily M, alpha member 1
16532	NM_008432,XM_006509024,XM_017312581,XM_017312582,XM_017312583,XR_003947236,XR_003947237	Kcnu1	potassium channel, subfamily U, member 1
16533	NM_031169,XM_006514541	Kcnmb1	potassium large conductance calcium-activated channel, subfamily M, beta member 1
16534	NM_001163510,NM_008433,XM_006539563,XR_003946402,XR_391046	Kcnn4	potassium intermediate/small conductance calcium-activated channel, subfamily N, member 4
16535	NM_008434,XM_006508488,XM_006508489,XM_006508490,XM_006508491,XM_006508492,XM_011241982,XM_017321998	Kcnq1	potassium voltage-gated channel, subfamily Q, member 1
16536	NM_001003824,NM_001003825,NM_001006668,NM_001006669,NM_001006674,NM_001302888,NM_010611,XM_006500565,XM_006500567,XM_006500568,XM_011239925,XM_017315735,XM_017315738,XM_017315740,XM_017315741,XM_017315742,XM_017315743,XM_017315744,XM_017315746,XM_017315747,XM_017315751,XM_017315753,XM_017315754,XM_017315763,XM_017315767,XM_017315771,XM_017315772,XM_017315773,XM_017315776,XM_017315781,XM_030247769,XM_030247772,XM_030247775,XM_030247788,XM_030247797,XM_030247798,XR_001781085,XR_001781086,XR_003950848	Kcnq2	potassium voltage-gated channel, subfamily Q, member 2
16538	NM_008435,XM_030247816	Kcns1	K+ voltage-gated channel, subfamily S, 1
16539	NM_001271704,NM_181317	Kcns2	K+ voltage-gated channel, subfamily S, 2
16541	NM_008437	Napsa	napsin A aspartic peptidase
16542	NM_001363216,NM_010612,XM_011240817	Kdr	kinase insert domain protein receptor
16543	NM_175088,XM_030255200	Mdfic	MyoD family inhibitor domain containing
16545	NM_008438,XM_006513273	Kera	keratocan
16548	NM_001310524,NM_001310525,NM_001349066,NM_008439,XM_006503748	Khk	ketohexokinase
16549	NM_010613	Khsrp	KH-type splicing regulatory protein
16551	NM_010615	Kif11	kinesin family member 11
16552	NM_001317352,NM_010616,XM_017319998,XM_017319999,XM_017320000	Kif12	kinesin family member 12
16553	NM_010617,XM_006516861,XM_006516862,XM_006516864,XM_011244374,XM_011244375,XM_011244376,XM_017315400,XM_030247165	Kif13a	kinesin family member 13A
16554	NM_001081177,XM_006518619,XM_006518620,XM_006518621,XM_006518622	Kif13b	kinesin family member 13B
16558	NM_001081133,NM_001355123,XM_006498813,XM_006498814,XM_006498815,XM_006498818,XM_006498819,XM_030247819	Kif16b	kinesin family member 16B
16559	NM_001190978,NM_010623	Kif17	kinesin family member 17
16560	NM_001110315,NM_001294149,NM_001294150,NM_001368847,NM_008440,XM_006529155,XM_006529156,XM_006529157,XM_006529158,XM_006529159,XM_006529160,XM_006529161	Kif1a	kinesin family member 1A
16561	NM_001290995,NM_008441,NM_207682,XM_006538590,XM_006538591,XM_006538592,XM_006538593,XM_006538594,XM_006538595,XM_006538596,XM_011250200,XM_030253268,XM_030253269,XM_030253270,XM_030253271,XM_030253272,XM_030253273	Kif1b	kinesin family member 1B
16562	NM_153103,XM_006532320,XM_006532321,XM_006532322,XM_006532323,XR_003949317	Kif1c	kinesin family member 1C
16563	NM_001145779,NM_008442,XM_006517535,XM_006517537,XM_006517538,XM_030247166,XM_030247167,XM_030247168	Kif2a	kinesin family member 2A
16564	NM_001109040,NM_001109041,NM_001109042,NM_001358050,NM_016705,XM_006520495,XM_017316441,XM_017316442,XM_017316443,XM_017316444,XM_017316445,XM_017316446,XM_017316447,XM_017316448,XM_017316449,XM_017316450,XM_017316452,XM_017316453,XM_017316454,XM_017316455,XM_017316456,XM_017316458,XM_017316460,XM_017316461,XM_017316462,XM_017316463,XM_017316464,XM_017316465,XM_017316466,XM_017316468,XM_017316471,XM_017316474,XM_017316475,XM_017316476,XM_017316477,XM_017316478,XM_017316479,XM_030248345,XR_001781495	Kif21a	kinesin family member 21A
16565	NM_001039472,NR_153829	Kif21b	kinesin family member 21B
16568	NM_001290805,NM_001290806,NM_008443,XM_006532325,XM_006532326,XM_006532328	Kif3a	kinesin family member 3A
16569	NM_008444,XR_374419	Kif3b	kinesin family member 3B
16570	NM_008445	Kif3c	kinesin family member 3C
16571	NM_008446,XM_006527855,XM_011247528,XM_017318397	Kif4	kinesin family member 4
16572	NM_001039000,NM_008447,XM_030244919	Kif5a	kinesin family member 5A
16573	NM_008448	Kif5b	kinesin family member 5B
16574	NM_008449	Kif5c	kinesin family member 5C
16576	NM_001291222,NM_010626,XM_006540664,XM_006540665,XM_006540666,XM_006540667,XM_006540668,XM_006540670,XR_003946403	Kif7	kinesin family member 7
16578	NM_001163569,NM_010628,XM_006511941,XM_006511942,XM_006511943,XM_006511944,XM_006511945	Kif9	kinesin family member 9
16579	NM_001305643,NM_010629,XM_006496679,XM_006496681	Kifap3	kinesin-associated protein 3
16580	NM_053173	Kifc5b	kinesin family member C5B
16581	NM_001358909,NM_001358910,NM_001358911,NM_001358912,NM_010630,XM_030248346,XM_030248347,XM_030248348,XM_030248349,XM_030248350,XM_030248351,XM_030248352,XM_030248353,XM_030248354,XM_030248355,XM_030248356,XR_001781497,XR_001781498,XR_003951357,XR_383987,XR_383988	Kifc2	kinesin family member C2
16582	NM_001145831,NM_001145832,NM_010631,XM_006530717,XM_006530718,XM_006530720,XM_006530721,XM_006530722,XM_006530723,XM_006530724,XM_006530726,XM_017312585,XM_030243307,XM_030243308,XM_030243309,XM_030243310,XM_030243311	Kifc3	kinesin family member C3
16588	NM_025280,XM_006497366	Kin	Kin17 DNA and RNA binding protein
16589	NM_001360809,NM_010633,XM_006496682	Uhmk1	U2AF homology motif (UHM) kinase 1
16590	NM_001122733,NM_021099,XM_017320687,XM_030254169	Kit	KIT proto-oncogene receptor tyrosine kinase
16591	NM_013823,XM_006504917	Kl	klotho
16592	NM_001272097,NM_001272098,NM_010634	Fabp5	fatty acid binding protein 5, epidermal
16593	NM_001025358,NM_001025359,NM_001025360,NM_001025361,NM_001025362,NM_001025363,NM_001081959,NM_001361605,NM_001361609,NM_001361610,NM_001361611,NM_001361612,NM_001361613,NM_001361614,NM_008450,XM_006515494,XM_030246565,XM_030246566,XR_001780413,XR_001780414,XR_001780415,XR_003949986,XR_003949987,XR_003949988,XR_003949989	Klc1	kinesin light chain 1
16594	NM_001369360,NM_001369361,NM_001369362,NM_008451,NR_045528	Klc2	kinesin light chain 2
16596	NM_010635	Klf1	Kruppel-like factor 1 (erythroid)
16597	NM_010636,XM_006518623,XM_006518624,XM_006518625,XM_011244968,XM_011244969,XM_011244970,XM_011244971,XM_017315889,XM_030247662,XM_030247664,XM_030247665	Klf12	Kruppel-like factor 12
16598	NM_008452	Klf2	Kruppel-like factor 2 (lung)
16599	NM_008453,XM_006503751,XR_001784628	Klf3	Kruppel-like factor 3 (basic)
16600	NM_010637	Klf4	Kruppel-like factor 4 (gut)
16601	NM_010638	Klf9	Kruppel-like factor 9
16612	NM_001320331,NM_001320332,NM_010639	Klk1	kallikrein 1
16616	NM_010642,XM_006540672,XM_011250808,XM_017322000,XM_017322001,XM_017322002	Klk1b21	kallikrein 1-related peptidase b21
16618	NM_010644,XM_006540675	Klk1b26	kallikrein 1-related petidase b26
16621	NM_008455	Klkb1	kallikrein B, plasma 1
16625	NM_008458,XM_011244002	Serpina3c	serine (or cysteine) peptidase inhibitor, clade A, member 3C
16633	NM_001170851,NM_008462,XM_006505644,XM_017321414,XM_017321415,XM_017321416,XR_001785100	Klra2	killer cell lectin-like receptor, subfamily A, member 2
16636	NM_008463,XM_006505648,XM_030255201	Klra5	killer cell lectin-like receptor, subfamily A, member 5
16641	NM_001136068,NM_001288664,NM_010652,XM_011241236,XM_011241237,XM_017321418	Klrc1	killer cell lectin-like receptor subfamily C, member 1
16643	NM_010654,XM_006505655,XM_006505656	Klrd1	killer cell lectin-like receptor, subfamily D, member 1
16644	NM_001102411,NM_001102412,NM_023125,XM_006521812,XM_006521813,XM_030248988	Kng1	kininogen 1
16646	NM_008465,XM_006521814,XM_030248989	Kpna1	karyopherin (importin) alpha 1
16647	NM_010655	Kpna2	karyopherin (importin) alpha 2
16648	NM_008466	Kpna3	karyopherin (importin) alpha 3
16649	NM_008467	Kpna4	karyopherin (importin) alpha 4
16650	NM_008468,XM_006502814	Kpna6	karyopherin (importin) alpha 6
16651	NM_001310837,NM_010656,XM_006506916	Sspn	sarcospan
16653	NM_021284,XM_006506918,XM_006506919	Kras	Kirsten rat sarcoma viral oncogene homolog
16656	NM_010657,XM_006502815,XM_006502817,XM_011240452,XM_011240453,XM_030253274,XR_001784097,XR_003954883,XR_003954884,XR_867814	Hivep3	human immunodeficiency virus type I enhancer binding protein 3
16658	NM_010658	Mafb	v-maf musculoaponeurotic fibrosarcoma oncogene family, protein B
16661	NM_010660,XM_011248768	Krt10	keratin 10
16663	NM_001313949,NM_010662	Krt13	keratin 13
16664	NM_001313956,NM_001313957,NM_016958	Krt14	keratin 14
16665	NM_008469	Krt15	keratin 15
16666	NM_001313958,NM_008470	Krt16	keratin 16
16667	NM_010663,XM_030245607	Krt17	keratin 17
16668	NM_010664	Krt18	keratin 18
16669	NM_001313963,NM_008471	Krt19	keratin 19
16671	NM_013570	Krt33b	keratin 33B
16673	NM_001174099,XM_017314302	Krt36	keratin 36
16675	NM_010666	Krt27	keratin 27
16678	NM_008473	Krt1	keratin 1
16679	NM_010667	Krt86	keratin 86
16680	NM_008474	Krt84	keratin 84
16681	NM_010668,XM_030248357	Krt2	keratin 2
16682	NM_008475	Krt4	keratin 4
16687	NM_008476	Krt6a	keratin 6A
16691	NM_031170	Krt8	keratin 8
16706	NM_001348207,NM_013571,XM_006532334,XM_006532335,XM_011248770,XM_011248771,XM_011248772,XM_011248773,XM_011248774,XM_011248775,XR_388355	Ksr1	kinase suppressor of ras 1

16709	NM_001293635,NM_001293636,NM_001347519,NM_001347520,NM_001347521,NM_001347522,NM_001347523,NM_001347524,NM_001347525,NM_001347526,NM_001347527,NM_001347528,NM_008477,XM_006518628,XM_006518629,XM_006518630,XM_006518632,XM_006518634,XM_006518637,XM_006518639,XM_006518640,XM_006518641,XM_006518642,XM_006518644,XM_006518649,XM_006518651,XM_006518653,XM_006518655,XM_011244972,XM_017315890,XM_017315891,XM_017315893,XM_017315894,XM_017315895,XM_017315896,XM_017315897,XM_017315899,XM_017315900,XM_017315901,XM_017315904,XM_017315905,XM_030247668,XM_030247669,XM_030247670,XM_030247671,XM_030247673,XM_030247674,XM_030247675,XM_030247676,XM_030247677,XM_030247678,XM_030247679,XM_030247680	Ktn1	kinectin 1
16716	NM_024291,XM_006511646,XM_006511647	Ky	kyphoscoliosis peptidase
16728	NM_008478,XM_006527859,XM_006527860,XM_006527861	L1cam	L1 cell adhesion molecule
16763	NM_133664	Lad1	ladinin
16764	NM_001290814,XM_006495737,XM_011238443,XM_011238444,XM_011238446,XM_011238447,XM_030251556	Aff3	AF4/FMR2 family, member 3
16765	NM_019641	Stmn1	stathmin 1
16768	NM_008479,XR_377427	Lag3	lymphocyte-activation gene 3
16770	NM_010679,XM_006520517	Lalba	lactalbumin, alpha
16772	NM_008480,XM_006523717,XM_006523718,XM_030249517,XM_030249518	Lama1	laminin, alpha 1
16773	NM_008481,XM_006512566,XM_011243134,XM_011243135,XM_017313815	Lama2	laminin, alpha 2
16774	NM_001347461,NM_010680,XM_006525685,XM_006525687,XM_006525689,XM_006525691,XM_030250349,XR_003952502,XR_385977	Lama3	laminin, alpha 3
16775	NM_010681	Lama4	laminin, alpha 4
16776	NM_001081171,XM_006500575,XM_017315832,XR_003950861	Lama5	laminin, alpha 5
16777	NM_008482,XM_006514992,XM_006514993	Lamb1	laminin B1
16779	NM_008483,XM_006511648	Lamb2	laminin, beta 2
16780	NM_001277928,NM_008484,XM_006497232,XM_006497233	Lamb3	laminin, beta 3
16782	NM_008485,XM_011247926,XM_011247927	Lamc2	laminin, gamma 2
16783	NM_001317353,NM_010684	Lamp1	lysosomal-associated membrane protein 1
16784	NM_001017959,NM_001290485,NM_010685,NR_152733	Lamp2	lysosomal-associated membrane protein 2
16785	NM_011029	Rpsa	ribosomal protein SA
16790	NM_008486	Anpep	alanyl (membrane) aminopeptidase
16792	NM_010686,XM_017320001	Laptm5	lysosomal-associated protein transmembrane 5
16795	NM_001317391,NM_010687,XM_030243312,XR_003947238	Large1	LARGE xylosyl- and glucuronyltransferase 1
16796	NM_010688,XM_006532341,XM_006532342	Lasp1	LIM and SH3 protein 1
16797	NM_010689,XM_006507396,XM_006507397,XM_006507398,XM_030242172	Lat	linker for activation of T cells
16798	NM_010690,XM_006512567	Lats1	large tumor suppressor
16800	NM_001198911,NM_001198912,NM_001198913,NM_008487,XM_006501058,XM_006501059,XM_006501060,XM_006501061,XM_006501064,XM_006501065,XM_006501066,XM_006501067,XM_011240027,XM_030252438,XM_030252439	Arhgef2	rho/rac guanine nucleotide exchange factor (GEF) 2
16801	NM_001130150,NM_001130151,NM_001130152,NM_001130153,NM_008488,XM_006539565,XM_017322003,XM_030242173,XM_030242174,XM_030242175,XR_003946404	Arhgef1	Rho guanine nucleotide exchange factor (GEF) 1
16803	NM_008489	Lbp	lipopolysaccharide binding protein
16814	NM_010691	Lbx1	ladybird homeobox 1
16815	NM_010692	Lbx2	ladybird homeobox 2
16816	NM_008490	Lcat	lecithin cholesterol acyltransferase
16818	NM_001162432,NM_001162433,NM_010693,XM_006502818,XM_030253275,XM_030253276	Lck	lymphocyte protein tyrosine kinase
16819	NM_008491	Lcn2	lipocalin 2
16822	NM_010696	Lcp2	lymphocyte cytosolic protein 2
16825	NM_001113408,NM_001360336,NM_010697,XM_006526723,XM_030250759,XM_030250760	Ldb1	LIM domain binding 1
16826	NM_001077398,NM_001286348,NM_001347349,NM_001359070,NM_001359071,NM_010698,XM_006503752,XM_006503754,XM_006503755,XM_006503756,XM_006503758,XM_017320689,XM_030254170,XM_030254171	Ldb2	LIM domain binding 2
16828	NM_001136069,NM_010699,NR_102727,XM_030242176	Ldha	lactate dehydrogenase A
16829	gene-Ldha-ps	Ldha-ps	lactate dehydrogenase A, pseudogene
16830	gene-Ldha-ps2	Ldha-ps2	lactate dehydrogenase A, pseudogene 2
16832	NM_001302765,NM_001316322,NM_008492	Ldhb	lactate dehydrogenase B
16834	NM_013581,XM_006532343,XM_006532344,XM_006532345,XM_006532346,XR_001779880,XR_388356,XR_388357	Cog1	component of oligomeric golgi complex 1
16835	NM_001252658,NM_001252659,NM_010700	Ldlr	low density lipoprotein receptor
16840	NM_001310655,NM_010701,XM_006518663,XM_006518664,XM_006518665,XM_030247681	Cnmd	chondromodulin
16842	NM_001276402,NM_001276403,NM_010703,XM_006501068,XM_006501069,XM_006501070,XM_030252440,XM_030252441,XM_030252442	Lef1	lymphoid enhancer binding factor 1
16846	NM_008493	Lep	leptin
16847	NM_001122899,NM_010704,NM_146146,XM_017320002	Lepr	leptin receptor
16848	NM_008494	Lfng	LFNG O-fucosylpeptide 3-beta-N-acetylglucosaminyltransferase
16852	NM_008495	Lgals1	lectin, galactose binding, soluble 1
16854	NM_001145953,NM_010705	Lgals3	lectin, galactose binding, soluble 3
16855	NM_010706,XM_006539566	Lgals4	lectin, galactose binding, soluble 4
16858	NM_008496,XM_006539569	Lgals7	lectin, galactose binding, soluble 7
16859	NM_001159301,NM_010708	Lgals9	lectin, galactose binding, soluble 9
16865	NM_001136070,NM_010709,XM_006529165,XM_006529166,XM_006529167,XM_011247928,XM_011247929,XM_017318526,XM_017318536,XM_017318549,XM_030251679,XR_003953306,XR_38714	Eif2d	eukaryotic translation initiation factor 2D
16866	NM_008497,XM_006541350,XM_011250810,XM_017322004,XM_030242177	Lhb	luteinizing hormone beta
16867	NM_001364898,NM_013582,NR_157357,XM_006523719,XM_006523720,XM_006523721,XM_006523722,XM_006523723,XM_011246	Lhcgr	luteinizing hormone/choriogonadotropin receptor
16869	NM_008498	Lhx1	LIM homeobox protein 1
16870	NM_001290646,NM_010710,XM_006497733,XM_006497734,XM_006497735,XM_006497736,XM_006497738,XM_006497739	Lhx2	LIM homeobox protein 2
16872	NM_010712,XM_006529169	Lhx4	LIM homeobox protein 4
16873	NM_008499	Lhx5	LIM homeobox protein 5
16874	NM_001083125,NM_001083126,NM_001083127,NM_001355125,NM_008500,XM_006497744,XR_374064	Lhx6	LIM homeobox protein 6
16875	NM_010713,XM_006501072	Lhx8	LIM homeobox protein 8
16876	NM_001025565,NM_001042577,NM_010714,XM_006529170,XM_006529171,XM_006529172,XM_006529173,XM_017318637,XM_030251696,XM_030251701,XM_030251703,XM_030251723,XM_030251724,XM_030251726,XM_030251728	Lhx9	LIM homeobox protein 9
16878	NM_001039537,NM_008501,XM_006514542,XM_006514543,XM_006514544	Lif	leukemia inhibitory factor
16880	NM_001113386,NM_001358593,NM_013584,XM_006519969,XM_006519970,XM_006519971,XM_011245326	Lifr	LIF receptor alpha
16881	NM_001083188,NM_001199310,NM_010715	Lig1	ligase I, DNA, ATP-dependent
16882	NM_001291245,NM_001291246,NM_001291247,NM_010716,XM_006532347,XM_006532348,XM_006532350,XM_006532353	Lig3	ligase III, DNA, ATP-dependent
16885	NM_001305875,NM_010717,NR_131247,XM_006504396	Limk1	LIM-domain containing, protein kinase
16886	NM_001034030,NM_001361623,NM_010718,NM_173053,XM_006514547,XM_006514548,XM_006514552,XM_030245608,XM_030245609,XM_030245610,XM_030245611,XR_003949323	Limk2	LIM motif-containing protein kinase 2
16889	NM_001111100,NM_021460	Lipa	lysosomal acid lipase A
16890	NM_001039507,NM_010719,XM_006539570,XM_006539572,XM_030242178,XM_030242179,XM_030242180,XM_030242181	Lipe	lipase, hormone sensitive
16891	NM_010720,XM_006525692	Lipg	lipase, endothelial
16897	NM_001159404,NM_001159405,NM_008502,XM_006532354,XM_030245612,XM_030245613	Llgl1	LLGL1 scribble cell polarity complex component
16898	NM_008503,XM_030249519	Rps2	ribosomal protein S2
16904	NM_001302485,NM_001302499,NM_008504,NR_126325,XM_006513277,XM_017313816	Gzmm	granzyme M (lymphocyte met-ase 1)
16905	NM_001002011,NM_001111102,NM_019390,XM_006501073	Lmna	lamin A
16906	NM_010721	Lmnb1	lamin B1
16907	NM_001347140,NM_010722	Lmnb2	lamin B2
16909	NM_001142335,NM_001142336,NM_001142337,NM_008505,XM_006498820,XM_030247876	Lmo2	LIM domain only 2
16911	NM_001161769,NM_001161770,NM_010723	Lmo4	LIM domain only 4
16912	NM_013585,XM_006523730	Psmb9	proteasome (prosome, macropain) subunit, beta type 9 (large multifunctional peptidase 2)
16913	NM_010724	Psmb8	proteasome (prosome, macropain) subunit, beta type 8 (large multifunctional peptidase 7)
16917	NM_010725,XM_006497746,XM_006497747	Lmx1b	LIM homeobox transcription factor 1 beta
16918	NM_001303121,NM_008506,XM_006502821	Mycl	v-myc avian myelocytomatosis viral oncogene lung carcinoma derived
16922	NM_010726	Phyh	phytanoyl-CoA hydroxylase
16923	NM_001306126,NM_001306127,NM_001306128,NM_008507,XM_006530170,XM_006530171,XM_006530172,XM_006530173	Sh2b3	SH2B adaptor protein 3
16924	NM_001159577,NM_001159578,NM_001159579,NM_001159580,NM_001359074,NM_010727,XM_006504254,XM_017320690,XM_017320691,XM_030254173,XM_030254175,XR_001784629,XR_001784630,XR_003955587,XR_003955588,XR_003955589	Lnx1	ligand of numb-protein X 1
16939	NM_008508	Lor	loricrin
16948	NM_001286181,NM_001286182,NM_010728	Lox	lysyl oxidase
16949	NM_010729	Loxl1	lysyl oxidase-like 1
16950	NM_013586	Loxl3	lysyl oxidase-like 3
16952	NM_010730	Anxa1	annexin A1
16956	NM_008509	Lpl	lipoprotein lipase
16969	NM_010731,XM_006513279,XM_006513280,XM_006513281,XM_006513282,XM_030244921	Zbtb7a	zinc finger and BTB domain containing 7a
16970	NM_001281980,NM_001281981,NM_001361633,NM_001368864,NM_008511,XM_006506924,XM_011241567,XM_011241568,XM_011241569,XM_011241570,XM_011241571,XM_030255203,XM_030255204	Lrmp	lymphoid-restricted membrane protein
16971	NM_008512	Lrp1	low density lipoprotein receptor-related protein 1
16973	NM_008513,XR_388250	Lrp5	low density lipoprotein receptor-related protein 5
16974	NM_008514,XM_006505657	Lrp6	low density lipoprotein receptor-related protein 6
16975	NM_001080926,NM_001369051,NM_001369052,NM_001369053,NM_001369054,NM_053073,NR_033496,XM_011240454,XM_011240455,XM_011240456,XM_011240458,XM_011240459,XM_011240460,XM_011240461,XM_011240462,XM_011240463,XM_011240464,XM_011240465,XM_011240466,XM_011240467,XM_017320003,XM_030253277,XM_030253278,XM_030253279,XM_030253280,XM_030253281,XM_030253282,XM_030253283,XM_030253284	Lrp8	low density lipoprotein receptor-related protein 8, apolipoprotein e receptor
16976	NM_013587	Lrpap1	low density lipoprotein receptor-related protein associated protein 1
16977	NM_001302555,XM_006505658,XM_006505659,XM_006505660,XM_006505663,XM_011241239,XM_011241240,XM_017321422	Lrrc23	leucine rich repeat containing 23
16978	NM_001111311,NM_001111312,NM_001310732,NM_008515,XM_006529175,XM_006529176,XM_006529178,XM_006529189,XM_006529194,XM_006529196,XM_006529199,XM_006529200,XM_006529201,XM_006529203,XM_006529204,XM_006529205,XM_006529206,XM_006529208,XM_006529209,XM_006529212,XM_006529213,XM_006529214,XM_006529215,XM_006529216,XM_017318722,XM_017318761,XM_017318958,XM_017318960,XM_017318961,XM_017318965,XM_017318966,XM_017318969,XM_017318974,XM_017318996,XM_017318999,XM_017319000,XM_017319004,XM_017319017,XM_030251771,XM_030251773,XM_030251790,XM_030251800,XM_030251811,XM_030251812,XM_030251813,XM_030251814,XM_030251816,XM_030251821,XM_030251823,XM_030251824	Lrrfip1	leucine rich repeat (in FLII) interacting protein 1
16979	NM_008516	Lrrn1	leucine rich repeat protein 1, neuronal
16980	NM_010732	Lrrn2	leucine rich repeat protein 2, neuronal
16981	NM_001271708,NM_001271709,NM_010733	Lrrn3	leucine rich repeat protein 3, neuronal
16985	NM_001136071,NM_001271508,NM_001271509,NM_001271510,NM_001271523,NM_019391,XM_006508494,XM_006508495,XM_006508496,XM_030242182,XM_030242183,XM_030242184	Lsp1	lymphocyte specific 1
16987	NM_146006,XM_006513283,XM_006513284,XM_030244922	Lss	lanosterol synthase
16988	NM_010734	Lst1	leukocyte specific transcript 1
16992	NM_010735,XM_006523731	Lta	lymphotoxin A
16993	NM_001313897,NM_008517	Lta4h	leukotriene A4 hydrolase
16994	NM_008518	Ltb	lymphotoxin B
16995	NM_008519,XM_006518666	Ltb4r1	leukotriene B4 receptor 1
16997	NM_001370743,NM_013589,XM_006515502,XM_017314966	Ltbp2	latent transforming growth factor beta binding protein 2
16998	NM_008520,XM_006531668,XM_006531669,XM_006531670,XM_006531671,XM_006531672,XM_006531673,XM_006531674,XM_017318071,XM_017318072,XM_017318073	Ltbp3	latent transforming growth factor beta binding protein 3
17000	NM_010736	Ltbr	lymphotoxin B receptor
17001	NM_001313968,NM_008521,NR_132732,NR_132733	Ltc4s	leukotriene C4 synthase
17002	NM_008522	Ltf	lactotransferrin
17005	NM_008523,NM_203345,NM_206941,NM_206942	Ltk	leukocyte tyrosine kinase
17022	NM_008524	Lum	lumican
17025	NM_001276446,NM_008525,XM_006537639	Alad	aminolevulinate, delta-, dehydratase
17035	NM_016753	Lxn	latexin
17057	NM_001159902,NM_010737	Klrb1a	killer cell lectin-like receptor subfamily B member 1A
17059	NM_001159904,NM_008527	Klrb1c	killer cell lectin-like receptor subfamily B member 1C
17060	NM_001365054,NM_008528	Blnk	B cell linker
17063	NM_010739,XM_006521815,XM_011245835	Muc13	mucin 13, epithelial transmembrane
17064	NM_010740	Cd93	CD93 antigen
17067	NM_001252055,NM_001252056,NM_001252057,NM_001252058,NM_010741,XM_006520521,XM_030248358	Ly6c1	lymphocyte antigen 6 complex, locus C1
17068	NM_010742	Ly6d	lymphocyte antigen 6 complex, locus D
17069	NM_001164036,NM_001164037,NM_001164038,NM_001164039,NM_001164040,NM_008529,XM_030248359,XM_030248360	Ly6e	lymphocyte antigen 6 complex, locus E
17075	NM_008532	Epcam	epithelial cell adhesion molecule
17076	NM_013825	Ly75	lymphocyte antigen 75
17079	NM_001360519,NM_008533	Cd180	CD180 antigen
17082	NM_001025602,NM_001294171,NM_010743,XM_006495741,XM_006495744,XM_017319082	Il1rl1	interleukin 1 receptor-like 1
17083	NM_001324510,NM_010744,NR_136911,NR_136912	Tmed1	transmembrane p24 trafficking protein 1
17084	NM_010745	Ly86	lymphocyte antigen 86
17085	NM_001277968,NM_008534,NR_102726	Ly9	lymphocyte antigen 9
17087	NM_001159711,NM_016923	Ly96	lymphocyte antigen 96
17089	NM_025281,XM_006503759,XM_006503760,XM_006503761,XM_006503762,XM_006503763,XM_006503764,XM_006503765,XM_006503766,XM_030254181,XM_030254182	Lyar	Ly1 antibody reactive clone
17095	NM_008535,XM_006530744,XM_006530745	Lyl1	lymphoblastomic leukemia 1
17096	NM_001111096,NM_010747,XM_030253285	Lyn	LYN proto-oncogene, Src family tyrosine kinase
17101	NM_010748,XM_006516561,XM_006516562,XM_006516563	Lyst	lysosomal trafficking regulator
17105	NM_017372	Lyz2	lysozyme 2
17110	NM_013590	Lyz1	lysozyme 1
17112	NM_001355130,NM_008536,XM_030252445,XM_030252446	Tm4sf1	transmembrane 4 superfamily member 1
17113	NM_010749	M6pr	mannose-6-phosphate receptor, cation dependent
17114	gene-M6pr-ps	M6pr-ps	mannose-6-phosphate receptor, pseudogene
17116	NM_010750	Mab21l1	mab-21-like 1
17117	NM_008537,XM_011245338	Amacr	alpha-methylacyl-CoA racemase
17118	NM_008538	Marcks	myristoylated alanine rich protein kinase C substrate
17119	NM_010751,XM_006505670	Mxd1	MAX dimerization protein 1
17120	NM_001359025,NM_001359027,NM_010752,NR_152617,XM_006504652,XM_017320695,XM_017320696	Mad1l1	MAD1 mitotic arrest deficient 1-like 1
17121	NM_016662,XR_873434	Mxd3	Max dimerization protein 3
17122	NM_010753	Mxd4	Max dimerization protein 4
17123	NM_001358785,NM_013591	Madcam1	mucosal vascular addressin cell adhesion molecule 1
17125	NM_008539,XM_006530746	Smad1	SMAD family member 1
17126	NM_001252481,NM_001311070,NM_010754,XM_006525699,XM_017317842,XM_030250351	Smad2	SMAD family member 2
17127	NM_016769,XM_006510819,XM_006510821	Smad3	SMAD family member 3
17128	NM_001364967,NM_001364968,NM_008540,NR_157374,XM_006525701,XM_011246855	Smad4	SMAD family member 4
17129	NM_001164041,NM_001164042,NM_008541,XM_006517117,XM_006517118,XM_006517119,XM_030247171	Smad5	SMAD family member 5
17130	NM_008542,XM_006510822	Smad6	SMAD family member 6

17131	NM_001042660,XM_006525703	Smad7	SMAD family member 7	protein_coding	NP_001036125.1;XP_006525766.1	1100518	.	4.716588	[illegible]
17132	NM_001025577	Maf	avian musculoaponeurotic fibrosarcoma oncogene homolog	protein_coding	NP_001020748.2	96909	.	24.065073	[illegible]
17133	NM_001304830,NM_001304831,NM_001304832,NM_010755,XM_006520546,XM_006520550,XM_011245484	Maff	v-maf musculoaponeurotic fibrosarcoma oncogene family, protein F (avian)	protein_coding	NP_001291759.1;NP_001291760.1;NP_001291761.1;NP_034885.1;XP_006520609.3;XP_006520613.1;XP_011243786.1	96910	.	1.782229	[illegible]
17134	NM_010756,XM_017314307,XM_017314308,XM_017314309,XM_017314311,XM_030245626,XM_030245627	Mafg	v-maf musculoaponeurotic fibrosarcoma oncogene family, protein G (avian)	protein_coding	NP_034886.1;XP_017169796.1;XP_017169797.1;XP_017169798.1;XP_017169800.1;XP_030101486.1;XP_030101487.1	96911	.	15.979265	[illegible]
17135	NM_010757,XM_006504653	Mafk	v-maf musculoaponeurotic fibrosarcoma oncogene family, protein K	protein_coding	NP_034887.1;XP_006504716.1	99951	.	3.329826	[illegible]
17136	NM_001346084,NM_001346085,NM_001346086,NM_001346087,NM_001346088,NM_001346089,NM_001369262,NM_010758,XM_011250439,XM_011250440,XM_017322007,XM_030242188,XM_030242189,XM_030242190	Mag	myelin-associated glycoprotein	protein_coding	NP_001333013.1;NP_001333014.1;NP_001333015.1;NP_001333016.1;NP_001333017.1;NP_001333018.1;NP_001356191.1;NP_034888.1;XP_011248741.1;XP_011248742.1;XP_017177496.1;XP_030098048.1;XP_030098049.1;XP_030098050.1	96912	.	0.070724	[illegible]
17147	NM_008545	Mageb3	melanoma antigen, family B, 3	protein_coding	NP_032571.2	105109	.	0.035404	[illegible]
17149	NM_001282737	Magoh	mago homolog, exon junction complex core component	protein_coding	NP_001269666.1	1330312	.	41.446339	[illegible]
17150	NM_001161799,NM_001369292,NM_008546,NR_161285,XM_006538612,XM_006538614	Mfap2	microfibrillar-associated protein 2	protein_coding	NP_001155271.2;NP_001356221.1;NP_032572.3;XP_006538675.1;XP_006538677.1	99559	.	182.585598	[illegible]
17151	NM_001355127,NM_010761,XM_006498852,XM_011239329	Ccndbp1	cyclin D-type binding-protein 1	protein_coding	NP_001342056.1;NP_034891.2;XP_006498915.1;XP_011237631.1	109595	.	13.893533	[illegible]
17152	NM_001145802,NM_001145803,NM_008547,XM_006516868,XM_006516869,XM_006516870,XM_006516871,XM_006516872,XM_006516873	Mak	male germ cell-associated kinase	protein_coding	NP_001139274.1;NP_001139275.1;NP_032573.2;XP_006516931.1;XP_006516932.1;XP_006516933.1;XP_006516934.1;XP_006516935.1;XP_006516936.1	96913	.	0.000000	[illegible]
17153	NM_001171187,NM_010762	Mal	myelin and lymphocyte protein, T cell differentiation protein	protein_coding	NP_001164658.1;NP_034892.1	892970	.	0.402810	[illegible]
17155	NM_008548,XM_006512568,XM_006512569,XM_011243136,XM_030244925,XR_001779471,XR_003948601	Man1a	mannosidase 1, alpha	protein_coding	NP_032574.1;XP_006512631.1;XP_006512632.1;XP_011241438.1;XP_030100785.1	104677	.	16.502940	[illegible]
17156	NM_010763	Man1a2	mannosidase, alpha, class 1A, member 2	protein_coding	NP_034893.1	104676	.	15.546629	[illegible]
17158	NM_008549,XM_030249522	Man2a1	mannosidase 2, alpha 1	protein_coding	NP_032575.2;XP_030105382.1	104669	.	15.514612	[illegible]
17159	NM_010764,XM_006530747,XM_006530748,XM_006530749	Man2b1	mannosidase 2, alpha B1	protein_coding	NP_034894.2;XP_006530810.1;XP_006530811.1;XP_006530812.1	107286	.	21.098124	[illegible]
17160	NM_008550,XM_030254184	Man2b2	mannosidase 2, alpha B2	protein_coding	NP_032576.2;XP_030110044.1	1195262	.	19.490967	[illegible]
17161	NM_173740	Maoa	monoamine oxidase A	protein_coding	NP_776101.3	96915	.	5.633713	[illegible]
17164	NM_008551,XM_030251925	Mapkapk2	MAP kinase-activated protein kinase 2	protein_coding	NP_032577.1;XP_030107785.1	109298	.	28.557716	[illegible]
17165	NM_010765	Mapkapk5	MAP kinase-activated protein kinase 5	protein_coding	NP_034895.1	1333110	.	15.503373	[illegible]
17167	NM_010766	Marco	macrophage receptor with collagenous structure	protein_coding	NP_034896.1	1309998	.	0.183796	[illegible]
17168	NM_001284359,NM_001284360,NM_181569,NR_104306	Nprl3	nitrogen permease regulator-like 3	protein_coding	NP_001271288.1;NP_001271289.1;NP_853547.1	109258	.	8.247739	[illegible]
17169	NM_001370744,NM_001370745,NM_021516,NM_022801,XM_006515511,XM_006515512,XM_006515513,XM_006515514,XM_006515517,XM_006515518,XM_006515519,XM_011244006,XM_011244007,XM_030246567,XM_030246568,XR_003949991	Mark3	MAP/microtubule affinity regulating kinase 3	protein_coding	NP_001357673.1;NP_001357674.1;NP_067491.2;NP_073712.2;XP_006515574.1;XP_006515575.1;XP_006515576.1;XP_006515577.1;XP_006515580.1;XP_006515581.1;XP_006515582.1;XP_011242308.1;XP_011242309.1;XP_030102427.1;XP_030102428.1	1341865	.	25.436068	[illegible]
17171	NM_008552,XM_006523293,XM_006523294,XM_006523296,XM_011246190,XM_011246191,XM_011246192,XM_011246193,XM_017317270,XM_030249523,XM_030249524	Mas1	MAS1 oncogene	protein_coding	NP_032578.2;XP_006523356.1;XP_006523357.1;XP_006523359.1;XP_011244492.1;XP_011244493.1;XP_011244494.1;XP_011244495.1;XP_017172759.1;XP_030105383.1;XP_030105384.1	96918	.	0.297302	[illegible]
17172	NM_008553	Ascl1	achaete-scute family bHLH transcription factor 1	protein_coding	NP_032579.2	96919	.	0.054547	[illegible]
17173	NM_008554	Ascl2	achaete-scute family bHLH transcription factor 2	protein_coding	NP_032580.2	96920	.	0.209227	[illegible]
17174	NM_001359083,NM_008555,XM_006521829,XR_003951766	Masp1	mannan-binding lectin serine peptidase 1	protein_coding	NP_001346012.1;NP_032581.2;XP_006521892.1	88492	.	1.723444	[illegible]
17175	NM_001003893,NM_010767,XM_011250202	Masp2	mannan-binding lectin serine peptidase 2	protein_coding	NP_001003893.1;NP_034897.1;XP_011248504.1	1330832	.	0.878390	[illegible]
17178	NM_008557	Fxyd3	FXYD domain-containing ion transport regulator 3	protein_coding	NP_032583.1	107497	.	0.501860	[illegible]
17179	NM_001285853,NM_001285854,NM_001285855,NM_010768,XM_006513294,XM_006513295,XM_006513298,XM_006513299,XM_011243376,XM_011243377	Matk	megakaryocyte-associated tyrosine kinase	protein_coding	NP_001272782.1;NP_001272783.1;NP_001272784.1;NP_034898.1;XP_006513357.1;XP_006513358.1;XP_006513361.1;XP_006513362.1;XP_011241678.1;XP_011241679.1	99259	.	1.658809	[illegible]
17180	NM_010769	Matn1	matrilin 1, cartilage matrix protein	protein_coding	NP_034899.2	106591	.	562.556335	[illegible]
17181	NM_001358780,NM_016762	Matn2	matrilin 2	protein_coding	NP_001345709.1;NP_058042.2	109613	.	42.435570	[illegible]
17182	NM_010770	Matn3	matrilin 3	protein_coding	NP_034900.4	1328350	.	67.299782	[illegible]
17183	NM_001252563,NM_013592,XM_011239331,XR_866232	Matn4	matrilin 4	protein_coding	NP_001239492.1;NP_038620.2;XP_011237633.1	1328314	.	55.097596	[illegible]
17184	NM_010771	Matr3	matrin 3	protein_coding	NP_034901.2	1298379	.	46.893749	[illegible]
17187	NM_001146176,NM_001361663,NM_001361664,NM_008558,XM_006515523,XM_017314968,XM_030246570,XM_030246571,XR_003949992,XR_872708	Max	Max protein	protein_coding	NP_001139648.1;NP_001348592.1;NP_001348593.1;NP_032584.2;XP_006515586.1;XP_017170457.1;XP_030102430.1;XP_030102431.1	96921	.	33.490564	[illegible]
17188	NM_010772,XM_006507402,XM_017322009,XM_017322010,XR_001785483	Maz	MYC-associated zinc finger protein (purine-binding transcription factor)	protein_coding	NP_034902.1;XP_006507465.1;XP_017177498.1;XP_017177499.1	1338823	.	44.572548	[illegible]
17189	NM_001164047,NM_001164048,NM_013593	Mb	myoglobin	protein_coding	NP_001157519.1;NP_001157520.1;NP_038621.2	96922	.	7.144348	[illegible]
17190	NM_001357421,NM_001357422,NM_001357423,NM_013594,XM_006525705,XM_006525707,XM_006525708,XM_006525709,XM_017317847,XM_030250352,XM_030250353,XM_030250354,XM_030250355	Mbd1	methyl-CpG binding domain protein 1	protein_coding	NP_001344350.1;NP_001344351.1;NP_001344352.1;NP_038622.2;XP_006525768.1;XP_006525770.1;XP_006525771.1;XP_006525772.1;XP_017173336.1;XP_030106212.1;XP_030106213.1;XP_030106214.1;XP_030106215.1	1333811	.	13.032252	[illegible]
17191	NM_001311071,NM_010773,XM_006525711,XR_001782347	Mbd2	methyl-CpG binding domain protein 2	protein_coding	NP_001298000.1;NP_034903.2;XP_006525774.1	1333813	.	30.039771	[illegible]
17192	NM_001306143,NM_013595	Mbd3	methyl-CpG binding domain protein 3	protein_coding	NP_001293072.1;NP_038623.1	1333812	.	43.135843	[illegible]
17193	NM_010774,XM_006505679,XM_006505680,XM_006505681,XM_006505683,XR_001785104,XR_001785105,XR_003956166	Mbd4	methyl-CpG binding domain protein 4	protein_coding	NP_034904.2;XP_006505742.1;XP_006505743.1;XP_006505744.1;XP_006505746.1	1333850	.	3.447283	[illegible]
17196	NM_001025245,NM_001025251,NM_001025254,NM_001025255,NM_001025256,NM_001025258,NM_001025259,NM_010777,XM_006526452,XM_006526453,XM_006526454,XM_006526455,XM_006526456,XM_006526457,XM_006526458,XM_030250356,XM_030250358	Mbp	myelin basic protein	protein_coding	NP_001020416.1;NP_001020422.1;NP_001020425.1;NP_001020426.1;NP_001020427.1;NP_001020429.1;NP_001020430.1;NP_034907.1;XP_006526515.1;XP_006526516.1;XP_006526517.1;XP_006526518.1;XP_006526519.1;XP_006526520.1;XP_006526521.1;XP_030106216.1;XP_030106218.1	96925	.	1.141279	[illegible]
17199	NM_008559	Mc1r	melanocortin 1 receptor	protein_coding	NP_032585.2	99456	.	0.000000	[illegible]
17200	NM_001271716,NM_001271717,NM_001301372,NM_008560	Mc2r	melanocortin 2 receptor	protein_coding	NP_001258645.1;NP_001258646.1;NP_001288301.1;NP_032586.1	96928	.	0.031720	[illegible]
17201	NM_008561	Mc3r	melanocortin 3 receptor	protein_coding	NP_032587.1	96929	.	0.000000	[illegible]
17202	NM_016977	Mc4r	melanocortin 4 receptor	protein_coding	NP_058673.2	99457	.	0.100374	[illegible]
17203	NM_013596,XM_030250359,XR_385979	Mc5r	melanocortin 5 receptor	protein_coding	NP_038624.3;XP_030106219.1	99420	.	0.417755	[illegible]
17207	NM_001159485,NM_001159486,NM_001371043,NM_001371044,NM_001371045,NM_001371046,NM_001371047,NM_001371048,NM_001371049,NM_001371050,NM_178076	Mcf2l	mcf.2 transforming sequence-like	protein_coding	NP_001152957.1;NP_001152958.1;NP_001357972.1;NP_001357973.1;NP_001357974.1;NP_001357975.1;NP_001357976.1;NP_001357977.1;NP_001357978.1;NP_001357979.1;NP_835177.2	103263	.	2.109983	[illegible]
17210	NM_008562,XM_006501088	Mcl1	myeloid cell leukemia sequence 1	protein_coding	NP_032588.1;XP_006501151.1	101769	.	42.305406	[illegible]
17215	NM_008563,XM_030251926	Mcm3	minichromosome maintenance complex component 3	protein_coding	NP_032589.1;XP_030107786.1	101845	.	12.978501	[illegible]
17216	NM_008564	Mcm2	minichromosome maintenance complex component 2	protein_coding	NP_032590.2	105380	.	12.497507	[illegible]
17217	NM_008565	Mcm4	minichromosome maintenance complex component 4	protein_coding	NP_032591.3	103199	.	14.252070	[illegible]
17218	NM_001302540,NM_008566,XR_001778410	Mcm5	minichromosome maintenance complex component 5	protein_coding	NP_001289469.1;NP_032592.2	103197	.	11.623124	[illegible]
17219	NM_001313695,NM_008567	Mcm6	minichromosome maintenance complex component 6	protein_coding	NP_001300624.1;NP_032593.1	1298227	.	21.467625	[illegible]
17220	NM_001356576,NM_001356577,NM_008568,XR_001784632	Mcm7	minichromosome maintenance complex component 7	protein_coding	NP_001343505.1;NP_001343506.1;NP_032594.1	1298398	.	24.285430	[illegible]
17221	NM_010778,NR_132621,XM_006497234,XM_006497235,XM_006497236,XM_006497237,XM_006497239,XM_011238929,XM_011238930,XR_001783154,XR_373888,XR_373889	Cd46	CD46 antigen, complement regulatory protein	protein_coding	NP_034908.1;XP_006497297.1;XP_006497298.1;XP_006497299.1;XP_006497300.1;XP_006497302.1;XP_011237231.1;XP_011237232.1	1203290	.	1.818097	[illegible]
17222	NM_008569,XM_011239332,XM_011239333,XM_030248086	Anapc1	anaphase promoting complex subunit 1	protein_coding	NP_032595.2;XP_011237634.1;XP_011237635.1;XP_030103946.1	103097	.	16.553873	[illegible]
17223	NR_028284	Mcpt-ps1	mast cell protease, pseudogene 1	transcribed_pseudogene	.	102769	.	0.105884	[illegible]
17227	NM_010779	Mcpt4	mast cell protease 4	protein_coding	NP_034909.2	96940	.	11.052429	[illegible]
17228	NM_010780	Cma1	chymase 1, mast cell	protein_coding	NP_034910.2	96941	.	22.310211	[illegible]
17229	NM_010781,XM_006523740,XM_030249525	Tpsb2	tryptase beta 2	protein_coding	NP_034911.3;XP_006523803.1;XP_030105385.1	96942	.	22.610265	[illegible]
17231	NM_008572,XM_006518671	Mcpt8	mast cell protease 8	protein_coding	NP_032598.1;XP_006518734.1	1261780	.	0.000000	[illegible]
17237	NM_001252437,NM_001357061,NM_001357062,NM_029657,XM_006521832,XM_006521837	Mgrn1	mahogunin, ring finger 1	protein_coding	NP_001239366.1;NP_001343990.1;NP_001343991.1;NP_083933.1;XP_006521895.1;XP_006521900.1	2447670	.	26.867765	[illegible]
17240	NM_001109973,NM_001276390,NM_001276391,NM_010783,XM_006523742,XM_006523743,XM_006523744,XM_006523745	Mdfi	MyoD family inhibitor	protein_coding	NP_001103443.1;NP_001263319.1;NP_001263320.1;NP_034913.2;XP_006523805.2;XP_006523806.1;XP_006523807.1;XP_006523808.1	107687	.	29.767379	[illegible]
17242	NM_001012335,NM_001012336,NM_001291481,NM_001291482,NM_001291483,NM_010784,XM_011239335	Mdk	midkine	protein_coding	NP_001012335.1;NP_001012336.1;NP_001278410.1;NP_001278411.1;NP_001278412.1;NP_034914.1;XP_011237637.1	96949	.	82.491396	[illegible]
17243	gene-Mdk-ps1	Mdk-ps1	midkine pseudogene 1	pseudogene	.	103029	.	0.000000	[illegible]
17245	NM_001162904,NM_001162905,NM_010785,NM_148922,XM_006513305,XM_006513306,XM_006513307,XM_006513308,XM_006513309,XM_030244929,XM_030244930,XM_030244931,XM_030244932,XM_030244933,XM_030244934,XM_030244935,XM_030244936,XM_030244937	Mdm1	transformed mouse 3T3 cell double minute 1	protein_coding	NP_001156376.1;NP_001156377.1;NP_034915.2;NP_683724.2;XP_006513368.1;XP_006513369.1;XP_006513370.1;XP_006513371.1;XP_006513372.1;XP_030100789.1;XP_030100790.1;XP_030100791.1;XP_030100792.1;XP_030100793.1;XP_030100794.1;XP_030100795.1;XP_030100796.1;XP_030100797.1	96951	.	3.271943	[illegible]
17246	NM_001288586,NM_010786,XM_017313822,XM_030244938	Mdm2	transformed mouse 3T3 cell double minute 2	protein_coding	NP_001275515.1;NP_034916.1;XP_017169311.1;XP_030100798.1	96952	.	17.602830	[illegible]
17248	NM_001302801,NM_001302802,NM_001302803,NM_001302804,NM_008575,NR_126506,XM_006529233,XR_003953685,XR_003953686,XR_387151	Mdm4	transformed mouse 3T3 cell double minute 4	protein_coding	NP_001289730.1;NP_001289731.1;NP_001289732.1;NP_001289733.1;NP_032601.2;XP_006529296.1	107934	.	29.539383	[illegible]
17250	NM_008576,XM_006521839,XM_017316888,XR_003951767	Abcc1	ATP-binding cassette, sub-family C (CFTR/MRP), member 1	protein_coding	NP_032602.1;XP_006521902.2;XP_017172377.1	102676	.	5.332953	[illegible]
17252	NM_001362269,NM_001362270,NM_021557	Rdh11	retinol dehydrogenase 11	protein_coding	NP_001349198.1;NP_001349199.1;NP_067532.2	102581	.	7.810475	[illegible]
17254	NM_001161413,NM_008577,XM_006526733,XM_017318078	Slc3a2	solute carrier family 3 (activators of dibasic and neutral amino acid transport), member 2	protein_coding	NP_001154885.1;NP_032603.3;XP_006526796.1;XP_017173567.1	96955	.	27.122577	[illegible]
17256	NM_001277309,NM_001277310,NM_001277311,NM_001277312,NM_010787,NR_102392,XM_006523746,XM_006523748,XM_006523749,XM_006523750	Mea1	male enhanced antigen 1	protein_coding	NP_001264238.1;NP_001264239.1;NP_001264240.1;NP_001264241.1;NP_034917.1;XP_006523809.1;XP_006523811.1;XP_006523812.1;XP_006523813.1	96957	.	23.264891	[illegible]
17257	NM_001081979,NM_010788,XM_030251240	Mecp2	methyl CpG binding protein 2	protein_coding	NP_001075448.1;NP_034918.1;XP_030107100.1	99918	.	6.268117	[illegible]
17258	NM_001033713,NM_001291191,NM_001291192,NM_001291195,NM_001291196,NM_001357324,NM_001357325,XM_006540684,XM_017322011,XM_030242191	Mef2a	myocyte enhancer factor 2A	protein_coding	NP_001028885.1;NP_001278120.1;NP_001278121.1;NP_001278124.1;NP_001278125.1;NP_001344253.1;NP_001344254.1;XP_006540747.1;XP_017177500.1;XP_030098051.1	99532	.	27.498520	[illegible]
17259	NM_001045484	Mef2b	myocyte enhancer factor 2B	protein_coding	NP_001038949.1	104526	.	0.199109	[illegible]
17260	NM_001170537,NM_001347564,NM_001347566,NM_001347567,NM_001347568,NM_001347569,NM_001347571,NM_001347572,NM_001347573,NM_001347574,NM_001347575,NM_001347576,NM_001347577,NM_001347578,NM_001347579,NM_001347580,NM_001347581,NM_025282,XM_006517123,XM_006517124,XM_006517126,XM_006517127,XM_006517128,XM_006517130,XM_006517132,XM_011244492,XM_011244495,XM_017315405,XM_030247172,XM_030247173,XM_030247174,XM_030247175,XM_030247176,XM_030247177	Mef2c	myocyte enhancer factor 2C	protein_coding	NP_001164008.1;NP_001334493.1;NP_001334495.1;NP_001334496.1;NP_001334497.1;NP_001334498.1;NP_001334500.1;NP_001334501.1;NP_001334502.1;NP_001334503.1;NP_001334504.1;NP_001334505.1;NP_001334506.1;NP_001334507.1;NP_001334508.1;NP_001334509.1;NP_001334510.1;NP_079558.1;XP_006517186.1;XP_006517187.1;XP_006517189.1;XP_006517190.1;XP_006517191.1;XP_006517193.1;XP_006517195.1;XP_011242794.1;XP_011242797.1;XP_017170894.1;XP_030103032.1;XP_030103033.1;XP_030103034.1;XP_030103035.1;XP_030103036.1;XP_030103037.1	99458	.	39.654321	[illegible]
17261	NM_001310587,NM_001310593,NM_133665,XM_006501089,XM_006501090,XM_011240032,XM_011240033,XM_011240034,XM_017319472,XM_017319473,XM_030252450,XM_030252451,XM_030252452,XM_030252453	Mef2d	myocyte enhancer factor 2D	protein_coding	NP_001297516.1;NP_001297522.1;NP_598426.1;XP_006501152.1;XP_006501153.1;XP_011238334.1;XP_011238335.1;XP_011238336.1;XP_017174961.1;XP_017174962.1;XP_030108310.1;XP_030108311.1;XP_030108312.1;XP_030108313.1	99533	.	22.181495	[illegible]
17263	NR_003633,NR_027651,NR_027652	Meg3	maternally expressed 3	lncRNA	.	1202886	.	362.808127	[illegible]
17268	NM_001193271,NM_010789,XM_006514559,XM_017314312,XM_017314313,XM_030245628,XR_001779899,XR_001779900,XR_003949337	Meis1	Meis homeobox 1	protein_coding	NP_001180200.1;NP_034919.1;XP_006514622.1;XP_017169801.1;XP_017169802.1;XP_030101488.1	104717	.	3.232992	[illegible]
17274	NM_023126	Rab8a	RAB8A, member RAS oncogene family	protein_coding	NP_075615.2	96960	.	25.658337	[illegible]
17279	NM_010790,XM_006537640	Melk	maternal embryonic leucine zipper kinase	protein_coding	NP_034920.2;XP_006537703.1	106924	.	4.578579	[illegible]
17281	NM_001110253,NM_148925,XM_017313159,XM_030244077,XM_030244078	Fyco1	FYVE and coiled-coil domain containing 1	protein_coding	NP_001103723.2;NP_683727.3;XP_017168648.1;XP_030099937.1;XP_030099938.1	107277	.	7.650562	[illegible]
17283	NM_001168488,NM_001168489,NM_001168490,NM_008583	Men1	multiple endocrine neoplasia 1	protein_coding	NP_001161960.1;NP_001161961.1;NP_001161962.1;NP_032609.1	1316736	.	13.310445	[illegible]
17285	NM_010791	Meox1	mesenchyme homeobox 1	protein_coding	NP_034921.1	103220	.	3.910751	[illegible]
17286	NM_008584	Meox2	mesenchyme homeobox 2	protein_coding	NP_032610.1	103219	.	7.441774	[illegible]
17287	NM_008585,XM_006523751	Mep1a	meprin 1 alpha	protein_coding	NP_032611.2;XP_006523814.1	96963	.	0.036237	[illegible]
17289	NM_008587,XM_006498860	Mertk	MER proto-oncogene tyrosine kinase	protein_coding	NP_032613.1;XP_006498923.1	96965	.	15.184175	[illegible]
17292	NM_008588	Mesp1	mesoderm posterior 1	protein_coding	NP_032614.2	107785	.	0.000000	[illegible]
17293	NM_008589	Mesp2	mesoderm posterior 2	protein_coding	NP_032615.2	1096325	.	0.047005	[illegible]
17294	NM_001252292,NM_001252293,NM_008590,XM_017321427,XM_030255208	Mest	mesoderm specific transcript	protein_coding	NP_001239221.1;NP_001239222.1;NP_032616.1;XP_017176916.1;XP_030111068.1	96968	.	308.276702	[illegible]
17295	NM_008591,XM_006505002,XM_011241042,XM_011241043,XM_030255209	Met	met proto-oncogene	protein_coding	NP_032617.2;XP_006505065.1;XP_011239344.1;XP_011239345.1;XP_030111069.1	96969	.	1.185072	[illegible]
17299	NM_010792,XM_006513312,XM_006513313	Mettl1	methyltransferase like 1	protein_coding	NP_034922.1;XP_006513375.1;XP_006513376.1	1339986	.	13.649674	[illegible]
17300	NM_008592	Foxc1	forkhead box C1	protein_coding	NP_032618.2	1347466	.	10.299969	[illegible]
17301	NM_008593	Foxd2	forkhead box D2	protein_coding	NP_032619.1	1347471	.	0.874768	[illegible]
17304	NM_001045489,NM_008594	Mfge8	milk fat globule-EGF factor 8 protein	protein_coding	NP_001038954.1;NP_032620.2	102768	.	73.017544	[illegible]
17305	NM_008595,XM_006520551,XM_006520552,XM_017316492,XR_003951359,XR_875250	Mfng	MFNG O-fucosylpeptide 3-beta-N-acetylglucosaminyltransferase	protein_coding	NP_032621.1;XP_006520614.1;XP_006520615.1;XP_017171981.1	1095404	.	4.390627	[illegible]
17306	NM_008596,XM_006501094	Sypl2	synaptophysin-like 2	protein_coding	NP_032622.1;XP_006501157.1	1328311	.	4.541700	[illegible]
17308	NM_001110148,NM_001110149,NM_001110150,NM_010794,XM_006532397,XM_006532398,XM_006532399,XM_006532400,XM_017314314,XM_030245629,XM_030245630	Mgat1	mannoside acetylglucosaminyltransferase 1	protein_coding	NP_001103618.1;NP_001103619.1;NP_001103620.1;NP_034924.3;XP_006532460.1;XP_006532461.1;XP_006532462.1;XP_006532463.1;XP_017169803.1;XP_030101489.1;XP_030101490.1	96973	.	29.553102	[illegible]
17309	NM_010795,XM_006520553,XM_006520554,XM_006520555,XM_006520556,XM_006520557,XM_011245485	Mgat3	mannoside acetylglucosaminyltransferase 3	protein_coding	NP_034925.2;XP_006520616.1;XP_006520617.1;XP_006520618.1;XP_006520619.1;XP_006520620.1;XP_011243787.1	104532	.	3.551927	[illegible]
17311	NM_001347156,NM_013598,XM_006513314,XM_006513315	Kitl	kit ligand	protein_coding	NP_001334085.1;NP_038626.1;XP_006513377.1;XP_006513378.1	96974	.	4.336920	[illegible]
17312	NM_001204252,NM_010796	Clec10a	C-type lectin domain family 10, member A	protein_coding	NP_001191181.1;NP_034926.1	96975	.	2.336545	[illegible]
17313	NM_008597	Mgp	matrix Gla protein	protein_coding	NP_032623.1	96976	.	714.555237	[illegible]
17314	NM_008598,XM_006507405,XM_006507406,XM_006507407,XM_006507408,XM_006507409,XM_006507410,XM_030242192	Mgmt	O-6-methylguanine-DNA methyltransferase	protein_coding	NP_032624.1;XP_006507468.1;XP_006507469.1;XP_006507470.1;XP_006507471.1;XP_006507472.1;XP_006507473.1;XP_030098052.1	96977	.	3.629823	[illegible]
17318	NM_001290504,NM_001290505,NM_001290506,NM_001290512,NM_010797,XM_006528736,XM_006528737,XM_006528739,XM_011247789,XM_017318408,XM_017318409,XM_017318410,XM_017318411,XM_017318412,XM_017318413,XM_030251241,XM_030251242,XM_030251243,XM_030251244,XR_878259	Mid1	midline 1	protein_coding	NP_001277433.1;NP_001277434.1;NP_001277435.1;NP_001277441.1;NP_034927.2;XP_006528799.1;XP_006528800.1;XP_006528802.1;XP_011246091.1;XP_017173897.1;XP_017173898.1;XP_017173899.1;XP_017173900.1;XP_017173901.1;XP_017173902.1;XP_030107101.1;XP_030107102.1;XP_030107103.1;XP_030107104.1	1100537	.	4.467338	[illegible]
17319	NM_010798	Mif	macrophage migration inhibitory factor (glycosylation-inhibiting factor)	protein_coding	NP_034928.1	96982	.	74.448860	[illegible]
17329	NM_008599	Cxcl9	chemokine (C-X-C motif) ligand 9	protein_coding	NP_032625.2	1352449	.	0.191591	[illegible]
17330	NM_010799	Minpp1	multiple inositol polyphosphate histidine phosphatase 1	protein_coding	NP_034929.1	1336159	.	13.994405	[illegible]
17341	NM_010800,XM_006504808	Bhlha15	basic helix-loop-helix family, member a15	protein_coding	NP_034930.1;XP_006504871.1	891976	.	0.475665	[illegible]
17342	NM_001113198,NM_001178049,NM_008601,XM_006505684,XM_006505685,XM_006505689,XM_006505691,XM_006505692,XM_006505694,XM_006505695,XM_006505696,XM_017321428,XM_030255210,XM_030255211,XM_030255212,XM_030255213,XM_030255214	Mitf	melanogenesis associated transcription factor	protein_coding	NP_001106669.1;NP_001171520.1;NP_032627.1;XP_006505747.1;XP_006505748.1;XP_006505752.1;XP_006505754.1;XP_006505755.1;XP_006505757.1;XP_006505758.1;XP_006505759.1;XP_017176917.1;XP_030111070.1;XP_030111071.1;XP_030111072.1;XP_030111073.1;XP_030111074.1	104554	.	1.913090	[illegible]
17344	NM_001164167,NM_001164168,NM_001164169,NM_001164170,NM_008602,XM_006526423,XM_006526425,XM_030250360,XR_001782348,XR_001782349,XR_003952503	Pias2	protein inhibitor of activated STAT 2	protein_coding	NP_001157639.1;NP_001157640.1;NP_001157641.1;NP_001157642.1;NP_032628.3;XP_006526486.1;XP_006526488.1;XP_030106220.1	1096566	.	14.340504	[illegible]
17345	NM_001081117,XM_006507411,XM_006507412,XM_006507413	Mki67	antigen identified by monoclonal antibody Ki 67	protein_coding	NP_001074586.2;XP_006507474.1;XP_006507475.1;XP_006507476.	106035	.	16.628837	[illegible]
17346	NM_001285487,NM_001285488,NM_001355174,NM_001369238,NM_021461,XM_006502822,XM_006502823,XM_006502825,XM_030253286,XM_030253287,XM_030253288,XM_030253289,XM_030253291	Mknk1	MAP kinase-interacting serine/threonine kinase 1	protein_coding	NP_001272416.1;NP_001272417.1;NP_001342103.1;NP_001356167.1;NP_067436.1;XP_006502885.1;XP_006502886.1;XP_006502888.1;XP_030109146.1;XP_030109147.1;XP_030109148.1;XP_030109149.1;XP_030109151.1	894316	.	12.368866	[illegible]
17347	NM_021462,XM_006513316,XM_017313824	Mknk2	MAP kinase-interacting serine/threonine kinase 2	protein_coding	NP_067437.2;XP_006513379.1;XP_017169313.1	894279	.	21.958372	[illegible]
17349	NM_001039543,NM_010801	Mlf1	myeloid leukemia factor 1	protein_coding	NP_001034632.1;NP_034931.2	1341819	.	7.210348	[illegible]
17350	NM_001324522,NM_026810,XM_006511948,XM_006511951,XM_011242935,XM_030244079,XR_001778805,XR_003947795,XR_379848,XR_379849	Mlh1	mutL homolog 1	protein_coding	NP_001311451.1;NP_081086.2;XP_006512011.1;XP_006512014.1;XP_011241237.1;XP_030099939.1	101938	.	5.452220	[illegible]
17354	NM_001252560,NM_001252561,NM_010804,NR_045538,NR_045539,NR_149308,XM_006497371,XM_006497373,XM_006497374,XM_006497375,XM_006497377,XM_006497378,XM_006497379,XM_006497382,XM_006497383,XM_006497384,XM_006497389,XM_006497390,XM_017316073,XM_017316080,XM_030248112,XM_030248113,XM_030248114,XM_030248115,XM_030248116,XM_030248120,XM_030248127,XM_030248130,XM_030248136,XM_030248137,XM_030248139,XM_030248150,XM_030248152,XM_030248154,XM_030248155,XM_030248156,XM_030248162,XM_030248168,XM_030248178,XM_030248181,XM_030248187,XM_030248191,XM_030248192,XM_030248193,XM_030248194,XM_030248195,XM_030248196,XM_030248197,XM_030248200,XM_030248206,XM_03024	Mllt10	myeloid/lymphoid or mixed-lineage leukemia; translocated to, 10	protein_coding	NP_001239489.1;NP_001239490.1;NP_034934.2;XP_006497434.2;XP_006497436.2;XP_006497437.2;XP_006497438.2;XP_006497440.2;XP_006497441.1;XP_006497442.1;XP_006497445.2;XP_006497446.2;XP_006497447.1;XP_006497452.1;XP_006497453.1;XP_017171562.1;XP_017171569.1;XP_030103972.1;XP_030103973.1;XP_030103974.1;XP_030103975.1;XP_030103976.1;XP_030103980.1;XP_030103987.1;XP_030103990.1;XP_030103996.1;XP_030103997.1;XP_030103999.1;XP_030104010.1;XP_030104012.1;XP_030104014.1;XP_030104015.1;XP_030104016.1;XP_030104022.1;XP_030104028.1;XP_030104038.1;XP_030104041.1;XP_030104047.1;XP_030104051.1;XP_030104052.1;XP_030104053.1;XP_030104054.1;XP_030104055.1;XP_030104056.1;XP_030104057.1;XP_030104060.1;XP_03010406	1329038	.	11.057659	[illegible]
17355	NM_001080798,NM_133919,XM_006534801	Aff1	AF4/FMR2 family, member 1	protein_coding	NP_001074267.1;NP_598680.3;XP_006534864.1	1100819	.	7.439166	[illegible]
17356	NM_010806,NR_151731,XM_006523752,XM_006523753,XM_006523754,XM_006523755,XM_006523756,XM_006523757,XM_006523758,XM_006523759,XM_006523760,XM_006523762,XM_006523763,XM_006523764,XM_006523765,XM_006523766,XM_006523767,XM_017317275,XM_017317276,XM_030249526,XM_030249527,XM_030249528,XR_001782046,XR_001782047,XR_003952091,XR_	Afdn	afadin, adherens junction formation factor	protein_coding	NP_034936.1;XP_006523815.1;XP_006523816.1;XP_006523817.1;XP_006523818.1;XP_006523819.1;XP_006523820.1;XP_006523821.1;XP_006523822.1;XP_006523823.1;XP_006523825.1;XP_006523826.1;XP_006523827.1;XP_006523828.1;XP_006523829.1;XP_006523830.1;XP_017172764.1;XP_017172765.1;XP_030105386.1;XP_030105387.1;XP_030105388.1	1314653	.	10.455254	[illegible]
17357	NM_010807	Marcksl1	MARCKS-like 1	protein_coding	NP_034937.1	97143	.	44.169910	[illegible]
17364	NM_001039104,NM_018752,XM_017322012,XM_017322013	Trpm1	transient receptor potential cation channel, subfamily M, member 1	protein_coding	NP_001034193.2;NP_061222.3;XP_017177501.1;XP_017177502.1	1330305	.	0.033119	[illegible]
17380	NM_001289462,NM_001289463,NM_001357335,NM_008604,XM_006501098	Mme	membrane metallo endopeptidase	protein_coding	NP_001276391.1;NP_001276392.1;NP_001344264.1;NP_032630.2;XP_006501161.1	97004	.	18.418400	[illegible]
17381	NM_001320076,NM_001320077,NM_008605,XM_017313160	Mmp12	matrix metallopeptidase 12	protein_coding	NP_001307005.1;NP_001307006.1;NP_032631.3;XP_017168649.1	97005	.	0.064251	[illegible]
17384	NM_019471	Mmp10	matrix metallopeptidase 10	protein_coding	NP_062344.1	97007	.	0.000000	[illegible]
17385	NM_001306184,NM_008606,XM_017313825	Mmp11	matrix metallopeptidase 11	protein_coding	NP_001293113.1;NP_032632.1;XP_017169314.1	97008	.	6.989844	[illegible]
17386	NM_008607	Mmp13	matrix metallopeptidase 13	protein_coding	NP_032633.1	1340026	.	211.932693	[illegible]
17387	NM_008608	Mmp14	matrix metallopeptidase 14 (membrane-inserted)	protein_coding	NP_032634.3	101900	.	231.016678	[illegible]
17388	NM_008609	Mmp15	matrix metallopeptidase 15	protein_coding	NP_032635.1	109320	.	2.986352	[illegible]
17389	NM_019724,NR_135158,XM_017320017	Mmp16	matrix metallopeptidase 16	protein_coding	NP_062698.2;XP_017175506.1	1276107	.	11.763211	[illegible]
17390	NM_008610,XM_006530751	Mmp2	matrix metallopeptidase 2	protein_coding	NP_032636.1;XP_006530814.1	97009	.	374.339600	[illegible]
17391	NM_010808,XM_030248212	Mmp24	matrix metallopeptidase 24	protein_coding	NP_034938.3;XP_030104072.1	1341867	.	0.027406	[illegible]
17392	NM_010809	Mmp3	matrix metallopeptidase 3	protein_coding	NP_034939.1	97010	.	0.683574	[illegible]
17394	NM_008611	Mmp8	matrix metallopeptidase 8	protein_coding	NP_032637.3	1202395	.	0.206569	[illegible]
17395	NM_013599,XM_006498861	Mmp9	matrix metallopeptidase 9	protein_coding	NP_038627.1;XP_006498924.1	97011	.	211.799183	[illegible]
17420	NM_008612,XM_006515526,XM_011244008	Mnat1	menage a trois 1	protein_coding	NP_032638.2;XP_006515589.1;XP_011242310.1	106207	.	7.986836	[illegible]
17423	NM_010811,XM_030247682,XR_003950827	Ndst2	N-deacetylase/N-sulfotransferase (heparan glucosaminyl) 2	protein_coding	NP_034941.2;XP_030103542.1	97040	.	12.456061	[illegible]
17425	NM_199068,XM_006504654	Foxk1	forkhead box K1	protein_coding	NP_951031.2;XP_006504717.1	1347488	.	9.277335	[illegible]
17427	NM_008613,XM_006510824,XM_011242665	Mns1	meiosis-specific nuclear structural protein 1	protein_coding	NP_032639.1;XP_006510887.3;XP_011240967.1	107933	.	2.669624	[illegible]
17428	NM_010813,XM_006532402,XM_006532403	Mnt	max binding protein	protein_coding	NP_034943.3;XP_006532465.1;XP_006532466.1	109150	.	6.147372	[illegible]
17433	NM_001039365,NM_008614,NR_153958,NR_153959,NR_153960	Mobp	myelin-associated oligodendrocytic basic protein	protein_coding	NP_001034454.1;NP_032640.1	108511	.	0.000000	[illegible]
17434	NM_001113374,NM_001113375,NM_013826,XM_030247178	Mocs2	molybdenum cofactor synthesis 2	protein_coding	NP_001106845.1;NP_001106846.1;NP_038854.2;XP_030103038.1	1336894	.	15.005928	[illegible]
17436	NM_001198933,NM_008615,XM_011242666	Me1	malic enzyme 1, NADP(+)-dependent, cytosolic	protein_coding	NP_001185862.1;NP_032641.2;XP_011240968.1	97043	.	16.002577	[illegible]
17441	NM_010814,XM_006523768	Mog	myelin oligodendrocyte glycoprotein	protein_coding	NP_034944.2;XP_006523831.1	97435	.	0.055510	[illegible]
17444	NM_001289442,NM_010815	Grap2	GRB2-related adaptor protein 2	protein_coding	NP_001276371.1;NP_034945.1	1333842	.	0.081908	[illegible]
17448	NM_008617	Mdh2	malate dehydrogenase 2, NAD (mitochondrial)	protein_coding	NP_032643.2	97050	.	105.990334	[illegible]
17449	NM_001316675,NM_008618	Mdh1	malate dehydrogenase 1, NAD (soluble)	protein_coding	NP_001303604.1;NP_032644.3	97051	.	46.688030	[illegible]
17450	NM_010816	Morc1	microrchidia 1	protein_coding	NP_034946.1	1316740	.	0.000000	[illegible]
17451	NM_020021	Mos	Moloney sarcoma oncogene	protein_coding	NP_064405.2	97052	.	0.085848	[illegible]
17454	NM_001163440,NM_001163441,NM_008619,XM_006501100,XM_006501101,XM_030252454,XM_030252455,XR_375493	Mov10	Mov10 RISC complex RNA helicase	protein_coding	NP_001156912.1;NP_001156913.1;NP_032645.2;XP_006501163.1;XP_006501164.1;XP_030108314.1;XP_030108315.1	97054	.	8.130781	[illegible]
17463	NM_010817	Psmd7	proteasome (prosome, macropain) 26S subunit, non-ATPase, 7	protein_coding	NP_034947.1	1351511	.	56.570198	[illegible]
17470	NM_001358443,NM_010818,XM_017316890	Cd200	CD200 antigen	protein_coding	NP_001345372.1;NP_034948.3;XP_017172379.1	1196990	.	58.745075	[illegible]

17472	NM_001256005,NM_001359079,NM_001359080,NM_001359081,NM_001359082,NM_008620,XM_006534804,XM_006534805,XM_011249411,XM_030254185,XM_030254186	Gbp4	guanylate binding protein 4	protein_coding	NP_001242934.1;NP_001346008.1;NP_001346009.1;NP_001346010.1;NP_001346011.1;NP_032646.2;XP_006534867.1;XP_006534868.1;XP_011247713.1;XP_030110045.1;XP_030110046.1	97072	.	1.474333	[illegible]
17474	NM_001163161,NM_010819	Clec4d	C-type lectin domain family 4, member d	protein_coding	NP_001156633.1;NP_034949.3	1298389	.	0.871984	[illegible]
17475	NM_001305284,NM_001305286,NM_010820,XM_006537643,XM_006537644,XM_006537646,XM_006537647,XM_006537649,XM_006537650,XM_011249940,XM_030253293	Mpdz	multiple PDZ domain protein	protein_coding	NP_001292213.1;NP_001292215.1;NP_034950.2;XP_006537706.1;XP_006537707.1;XP_006537709.1;XP_006537710.1;XP_006537712.1;XP_006537713.1;XP_011248242.1;XP_030109153.1	1343489	.	9.333702	[illegible]
17476	NM_010821,XM_006526734	Mpeg1	macrophage expressed gene 1	protein_coding	NP_034951.2;XP_006526797.1	1333743	.	3.301868	[illegible]
17480	NM_001122949,NM_001285496,NM_001285497,NM_010823,XM_011240468,XM_030253295	Mpl	myeloproliferative leukemia virus oncogene	protein_coding	NP_001116421.1;NP_001272425.1;NP_001272426.1;NP_034953.2;XP_011238770.1;XP_030109155.1	97076	.	0.061133	[illegible]
17523	NM_010824	Mpo	myeloperoxidase	protein_coding	NP_034954.2	97137	.	0.295804	[illegible]
17524	NM_008621	Mpp1	membrane protein, palmitoylated	protein_coding	NP_032647.1	105941	.	16.339516	[illegible]
17527	NM_001294322,NM_001294324,NM_001310527,NM_001310528,NM_008622	Mpv17	MpV17 mitochondrial inner membrane protein	protein_coding	NP_001281251.1;NP_001281253.1;NP_001297456.1;NP_001297457.1;NP_032648.1	97138	.	17.426000	[illegible]
17528	NM_001315499,NM_001315500,NM_008623	Mpz	myelin protein zero	protein_coding	NP_001302428.1;NP_001302429.1;NP_032649.2	103177	.	0.499764	[illegible]
17532	NM_008624,XM_006510825,XM_030244080,XM_030244081,XM_030244082	Mras	muscle and microspikes RAS	protein_coding	NP_032650.1;XP_006510888.1;XP_030099940.1;XP_030099941.1;XP_030099942.1	1100856	.	11.765431	[illegible]
17533	NM_008625	Mrc1	mannose receptor, C type 1	protein_coding	NP_032651.2	97142	.	19.945425	[illegible]
17534	NM_008626,XM_006532406	Mrc2	mannose receptor, C type 2	protein_coding	NP_032652.3;XP_006532469.1	107818	.	69.144180	[illegible]
17535	NM_001310728,NM_018736,XM_006510048,XM_006510049,XM_006510051,XM_006510053,XM_006510054,XM_030244083,XR_379107	Mre11a	MRE11A homolog A, double strand break repair nuclease	protein_coding	NP_001297657.1;NP_061206.1;XP_006510111.1;XP_006510112.1;XP_006510114.1;XP_006510116.1;XP_006510117.1;XP_030099943.1	1100512	.	4.269268	[illegible]
17536	NM_001136072,NM_001159567,NM_001159568,NM_001159569,NM_001159570,NM_001346036,NM_001346038,NM_001346041,NM_001346044,NM_001346045,NM_001346047,NM_001346053,NM_001346055,NM_001346056,NM_010825,XM_006498864,XM_006498871,XM_011239336,XM_011239337,XM_030248218,XM_030248220,XM_030248221,XM_030248222,XM_030248223,XM_030248224,XM_030248225,XM_030248226,XM_030248227	Meis2	Meis homeobox 2	protein_coding	NP_001129544.1;NP_001153039.1;NP_001153040.1;NP_001153041.1;NP_001153042.1;NP_001332965.1;NP_001332967.1;NP_001332970.1;NP_001332973.1;NP_001332974.1;NP_001332976.1;NP_001332982.1;NP_001332984.1;NP_001332985.1;NP_034955.1;XP_006498927.1;XP_006498934.1;XP_011237638.1;XP_011237639.1;XP_030104078.1;XP_030104080.1;XP_030104081.1;XP_030104082.1;XP_030104083.1;XP_030104084.1;XP_030104085.1;XP_030104086.1;XP_0	108564	.	3.515262	[illegible]
17537	NM_001277988,NM_001277989,NM_001302472,NM_008627,NR_102730,XM_006539603,XM_006539605,XM_017322015,XM_017322016,XM_017322017,XM_017322018,XM_017322019,XM_030242193,XM_030242194,XM_030242195,XM_030242196,XM_030242197,XM_030242198,XR_003946410,XR_003946411	Meis3	Meis homeobox 3	protein_coding	NP_001264917.1;NP_001264918.1;NP_001289401.1;NP_032653.2;XP_006539666.1;XP_006539668.1;XP_017177504.1;XP_017177505.1;XP_017177506.1;XP_017177507.1;XP_017177508.1;XP_030098053.1;XP_030098054.1;XP_030098055.1;XP_030098056.1;XP_030098057.1;XP_030098058.1	108519	.	13.730532	[illegible]
17540	NM_010826,NM_194464,XM_006507415,XM_006507416,XM_006507418,XM_011241686,XM_011241687,XR_869664	Mrvi1	MRV integration site 1	protein_coding	NP_034956.2;NP_919446.2;XP_006507478.1;XP_006507479.1;XP_006507481.1;XP_011239988.1;XP_011239989.1	1338023	.	1.042509	[illegible]
17681	NM_001360810,NM_010827,XM_006495458,XM_011238348	Msc	musculin	protein_coding	NP_001347739.1;NP_034957.1;XP_006495521.1;XP_011236650.1	1333884	.	0.251236	[illegible]
17684	NM_010828	Cited2	Cbp/p300-interacting transactivator, with Glu/Asp-rich carboxy-terminal domain, 2	protein_coding	NP_034958.2	1306784	.	9.436042	[illegible]
17685	NM_008628	Msh2	mutS homolog 2	protein_coding	NP_032654.1	101816	.	7.138187	[illegible]
17686	NM_001311120,NM_010829,XM_006517542,XM_006517543,XM_006517547,XM_017315412,XM_017315413	Msh3	mutS homolog 3	protein_coding	NP_001298049.1;NP_034959.2;XP_006517605.1;XP_006517606.1;XP_006517610.1;XP_017170901.1;XP_017170902.1	109519	.	6.575235	[illegible]
17687	NM_001146215,NM_013600,NR_130640,NR_130641,XM_006523770,XM_006523778,XM_006523780,XM_011246314,XM_030249530,XM_030249531,XM_030249532,XM_030249533,XM_030249534,XM_030249535,XM_030249536,XM_030249537,XM_030249538,XM_030249539,XM_030249540,XM_030249541,XM_030249542,XM_030249543,XM_030249545,XM_030249546,XR_003952092,XR_003952093,XR_003952094,XR_003952095,XR_003952096	Msh5	mutS homolog 5	protein_coding	NP_001139687.1;NP_038628.2;XP_006523833.1;XP_006523841.1;XP_006523843.1;XP_011244616.1;XP_030105390.1;XP_030105391.1;XP_030105392.1;XP_030105393.1;XP_030105394.1;XP_030105395.1;XP_030105396.1;XP_030105397.1;XP_030105398.1;XP_030105399.1;XP_030105400.1;XP_030105401.1;XP_030105402.1;XP_030105403.1;XP_030105405.1;XP_030105406.1	1329021	.	0.701483	[illegible]
17688	NM_010830	Msh6	mutS homolog 6	protein_coding	NP_034960.1	1343961	.	7.894097	[illegible]
17690	NM_008629,XM_006530177,XM_006530178,XM_006530179,XM_006530180,XM_006530181,XM_006530182,XM_030254187,XM_030254188	Msi1	musashi RNA-binding protein 1	protein_coding	NP_032655.1;XP_006530240.1;XP_006530241.1;XP_006530242.1;XP_006530243.1;XP_006530244.1;XP_006530245.1;XP_030110047.1;XP_030110048.1	107376	.	12.835477	[illegible]
17691	NM_010831	Sik1	salt inducible kinase 1	protein_coding	NP_034961.2	104754	.	3.616954	[illegible]
17692	NM_001370791,NM_001370792,NM_001370793,NM_001370794,NM_001370795,NM_001370796,NM_001370797,NM_001370798,NM_001370799,NM_010832,XM_017318415	Msl3	MSL complex subunit 3	protein_coding	NP_001357720.1;NP_001357721.1;NP_001357722.1;NP_001357723.1;NP_001357724.1;NP_001357725.1;NP_001357726.1;NP_001357727.1;NP_001357728.1;NP_034962.2;XP_017173904.1	1341851	.	10.748426	[illegible]
17698	NM_010833	Msn	moesin	protein_coding	NP_034963.2	97167	.	106.119675	[illegible]
17700	NM_010834	Mstn	myostatin	protein_coding	NP_034964.1	95691	.	0.305556	[illegible]
17701	NM_010835	Msx1	msh homeobox 1	protein_coding	NP_034965.2	97168	.	16.714094	[illegible]
17702	NM_013601	Msx2	msh homeobox 2	protein_coding	NP_038629.2	97169	.	9.463955	[illegible]
17703	NM_001347609,NM_010836	Msx3	msh homeobox 3	protein_coding	NP_001334538.1;NP_034966.1	106587	.	0.041432	[illegible]
17705	gene-ATP6	ATP6	ATP synthase F0 subunit 6	protein_coding	NP_904333.1	99927	.	4016.086914	[illegible]
17706	gene-ATP8	ATP8	ATP synthase F0 subunit 8	protein_coding	NP_904332.1	99926	.	3581.559814	[illegible]
17708	gene-COX1	COX1	cytochrome c oxidase subunit I	protein_coding	NP_904330.1	102504	.	7096.599121	[illegible]
17709	gene-COX2	COX2	cytochrome c oxidase subunit II	protein_coding	NP_904331.1	102503	.	3252.917969	[illegible]
17710	gene-COX3	COX3	cytochrome c oxidase subunit III	protein_coding	NP_904334.1	102502	.	2678.571045	[illegible]
17711	gene-CYTB	CYTB	cytochrome b	protein_coding	NP_904340.1	102501	.	3957.165283	[illegible]
17713	NM_024478	Grpel1	GrpE-like 1, mitochondrial	protein_coding	NP_077798.1	1334417	.	8.701919	[illegible]
17714	NM_021296,XR_385980	Grpel2	GrpE-like 2, mitochondrial	protein_coding	NP_067271.1	1334416	.	5.800275	[illegible]
17716	gene-ND1	ND1	NADH dehydrogenase subunit 1	protein_coding	NP_904328.1	101787	.	3462.867188	[illegible]
17717	gene-ND2	ND2	NADH dehydrogenase subunit 2	protein_coding	NP_904329.1	102500	.	1427.298096	[illegible]
17718	gene-ND3	ND3	NADH dehydrogenase subunit 3	protein_coding	NP_904335.1	102499	.	517.439819	[illegible]
17719	gene-ND4	ND4	NADH dehydrogenase subunit 4	protein_coding	NP_904337.1	102498	.	2029.170532	[illegible]
17720	gene-ND4L	ND4L	NADH dehydrogenase subunit 4L	protein_coding	NP_904336.1	102497	.	1232.230347	[illegible]
17721	gene-ND5	ND5	NADH dehydrogenase subunit 5	protein_coding	NP_904338.1	102496	.	1492.330566	[illegible]
17722	gene-ND6	ND6	NADH dehydrogenase subunit 6	protein_coding	NP_904339.1	102495	.	207.952332	[illegible]
17724	rna-mt-Rnr1	mt-Rnr1	s-rRNA	rRNA	.	102493	.	183.036438	[illegible]
17725	rna-mt-Rnr2	mt-Rnr2	16S ribosomal RNA	rRNA	.	102492	.	203.505051	[illegible]
17726	rna-TrnA	TrnA	tRNA-Ala	tRNA	.	102491	.	16.723942	[illegible]
17727	rna-TrnC	TrnC	tRNA-Cys	tRNA	.	102490	.	3.610542	[illegible]
17728	rna-TrnD	TrnD	tRNA-Asp	tRNA	.	102489	.	9.033259	[illegible]
17729	rna-TrnE	TrnE	tRNA-Glu	tRNA	.	102488	.	133.954697	[illegible]
17730	rna-TrnF	TrnF	tRNA-Phe	tRNA	.	102487	.	1.162368	[illegible]
17731	rna-TrnG	TrnG	tRNA-Gly	tRNA	.	102486	.	13.824245	[illegible]
17732	rna-TrnH	TrnH	tRNA-His	tRNA	.	102485	.	0.087516	[illegible]
17733	rna-TrnI	TrnI	tRNA-Ile	tRNA	.	102484	.	2.974012	[illegible]
17734	rna-TrnK	TrnK	tRNA-Lys	tRNA	.	102483	.	8.169316	[illegible]
17735	rna-TrnL1	TrnL1	tRNA-Leu	tRNA	.	102482	.	352.918549	[illegible]
17736	rna-TrnL2	TrnL2	tRNA-Leu	tRNA	.	102481	.	6.762082	[illegible]
17737	rna-TrnM	TrnM	tRNA-Met	tRNA	.	102480	.	39.987896	[illegible]
17738	rna-TrnN	TrnN	tRNA-Asn	tRNA	.	102479	.	3.574304	[illegible]
17739	rna-TrnP	TrnP	tRNA-Pro	tRNA	.	102478	.	12.308261	[illegible]
17740	rna-TrnQ	TrnQ	tRNA-Gln	tRNA	.	102477	.	2.844527	[illegible]
17741	rna-TrnR	TrnR	tRNA-Arg	tRNA	.	102476	.	13.355160	[illegible]
17742	rna-TrnS1	TrnS1	tRNA-Ser	tRNA	.	102475	.	573.157654	[illegible]
17743	rna-TrnS2	TrnS2	tRNA-Ser	tRNA	.	102474	.	0.616204	[illegible]
17744	rna-TrnT	TrnT	tRNA-Thr	tRNA	.	102473	.	10.095855	[illegible]
17745	rna-TrnV	TrnV	tRNA-Val	tRNA	.	102472	.	13.351960	[illegible]
17746	rna-TrnW	TrnW	tRNA-Trp	tRNA	.	102471	.	26.157928	[illegible]
17747	rna-TrnY	TrnY	tRNA-Tyr	tRNA	.	102470	.	18.119328	[illegible]
17748	NM_013602,XM_006530752	Mt1	metallothionein 1	protein_coding	NP_038630.1;XP_006530815.1	97171	.	14.535068	[illegible]
17749	NM_001039368,NM_023127	Polr2k	polymerase (RNA) II (DNA directed) polypeptide K	protein_coding	NP_001034457.1;NP_075616.2	102725	.	12.722452	[illegible]
17750	NM_008630	Mt2	metallothionein 2	protein_coding	NP_032656.1	97172	.	7.971090	[illegible]
17751	NM_013603	Mt3	metallothionein 3	protein_coding	NP_038631.1	97173	.	4.103196	[illegible]
17754	NM_001173506,NM_032393,XM_006498872	Map1a	microtubule-associated protein 1 A	protein_coding	NP_001166977.1;NP_115769.1;XP_006498935.1	1306776	.	2.466139	[illegible]
17755	NM_008634,XM_006517548	Map1b	microtubule-associated protein 1B	protein_coding	NP_032660.2;XP_006517611.1	1306778	.	5.884449	[illegible]
17756	NM_001039934,NM_001310634,NM_008632,XM_006495746,XM_006495747,XM_006495748,XM_006495749,XM_006495750,XM_006495751,XM_006495752,XM_006495753,XM_006495754,XM_006495756,XM_006495757,XM_006495758,XM_006495759,XM_006495760,XM_006495761,XM_006495762,XM_011238448,XM_017319165,XM_017319166,XM_017319169,XM_017319175,XM_017319194,XM_030251971,XM_030251976,XM_030251978,XM_030251982,XM_030251986,XM_030251989,XM_030251990,XM_030251993,XM_030251996	Map2	microtubule-associated protein 2	protein_coding	NP_001035023.1;NP_001297563.1;NP_032658.2;XP_006495809.1;XP_006495810.1;XP_006495811.1;XP_006495812.1;XP_006495813.1;XP_006495814.1;XP_006495815.1;XP_006495816.1;XP_006495817.1;XP_006495819.1;XP_006495820.1;XP_006495821.1;XP_006495822.1;XP_006495823.1;XP_006495824.1;XP_006495825.1;XP_011236750.1;XP_017174654.1;XP_017174655.1;XP_017174658.1;XP_017174664.1;XP_017174683.1;XP_030107831.1;XP_030107836.1;XP_030107838.1;XP_030107842.1;XP_030107846.1;XP_030107849.1;XP_030107850.1;XP_030107853.1;XP_030107856.1	97175	.	1.939494	[illegible]
17758	NM_001205330,NM_001205331,NM_001205332,NM_001311163,NM_001311164,NM_008633,XM_006511955,XM_006511956,XM_006511960,XM_006511962,XM_006511964,XM_011242938,XM_017313161,XM_017313162,XM_017313163,XM_017313174,XM_017313175,XM_017313176,XM_017313177,XM_017313178,XM_017313179,XM_017313180,XM_017313181,XM_017313182,XM_017313183,XM_017313184,XM_017313185,XM_017313186,XM_017313187,XM_017313188,XM_017313189,XM_030244084,XM_030244085,XM_030244086,XM_030244087,XM_030244088,XM_030244089,XM_030244090,XM_030244091,XM_030244092,XM_030244093,XM_030244094,XM_030244095,XM_030244096,XM_030244097,XM_030244098,XM_030244099,XM_030244100,XM_030244101,XM_030244102	Map4	microtubule-associated protein 4	protein_coding	NP_001192259.1;NP_001192260.1;NP_001192261.1;NP_001298092.1;NP_001298093.1;NP_032659.2;XP_006512018.1;XP_006512019.1;XP_006512023.1;XP_006512025.1;XP_006512027.1;XP_011241240.1;XP_017168650.1;XP_017168651.1;XP_017168652.1;XP_017168663.1;XP_017168664.1;XP_017168665.1;XP_017168666.1;XP_017168667.1;XP_017168668.1;XP_017168669.1;XP_017168670.1;XP_017168671.1;XP_017168672.1;XP_017168673.1;XP_017168674.1;XP_017168675.1;XP_017168676.1;XP_017168677.1;XP_017168678.1;XP_030099944.1;XP_030099945.1;XP_030099946.1;XP_030099947.1;XP_030099948.1;XP_030099949.1;XP_030099950.1;XP_030099951.1;XP_030099952.1;XP_030099953.1;XP_030099954.1;XP_030099955.1;XP_030099956.1;XP_030099957.1;XP_030099958.1;XP_030099959.1;XP_030099960.1;XP_030099961.1;XP_030099962.1	97178	.	62.795491	[illegible]
17760	NM_001043355,NM_001048167,NM_010837,XR_001785484,XR_001785485,XR_003946412,XR_003946413,XR_003946414,XR_003946415,XR_378220,XR_378221,XR_378223,XR_869665,XR_869666,XR_869667	Map6	microtubule-associated protein 6	protein_coding	NP_001036820.2;NP_001041632.1;NP_034967.2	1201690	.	2.375060	[illegible]
17761	NM_001198635,NM_001358787,NM_008635,XM_006512577,XM_006512579,XM_006512580,XM_006512581,XM_006512582,XM_006512583,XM_006512584,XM_006512585	Map7	microtubule-associated protein 7	protein_coding	NP_001185564.1;NP_001345716.1;NP_032661.2;XP_006512640.1;XP_006512642.1;XP_006512643.1;XP_006512644.1;XP_006512645.1;XP_006512646.1;XP_006512647.1;XP_006512648.1	1328328	.	0.596979	[illegible]
17762	NM_001038609,NM_001285454,NM_001285455,NM_001285456,NM_010838,XM_006532407,XM_006532408,XM_006532409,XM_017314315,XM_030245631	Mapt	microtubule-associated protein tau	protein_coding	NP_001033698.1;NP_001272383.1;NP_001272384.1;NP_001272385.1;NP_034968.3;XP_006532470.1;XP_006532471.1;XP_006532472.1;XP_017169804.1;XP_030101491.1	97180	.	0.377889	[illegible]
17763	NM_001039373	Mtcp1	mature T cell proliferation 1	protein_coding	NP_001034462.1	102699	.	4.647518	[illegible]
17764	NM_008636	Mtf1	metal response element binding transcription factor 1	protein_coding	NP_032662.3	101786	.	2.170774	[illegible]
17765	NM_001253877,NM_001253878,NM_001253879,NM_001253880,NM_001359088,NM_001359089,NM_013827,NR_152817,XM_011249412,XM_011249416,XM_011249417,XM_030254189,XM_030254190,XM_030254191,XM_030254192,XM_030254193,XM_030254194,XM_030254195,XM_030254196,XR_389261	Mtf2	metal response element binding transcription factor 2	protein_coding	NP_001240806.1;NP_001240807.1;NP_001240808.1;NP_001240809.1;NP_001346017.1;NP_001346018.1;NP_038855.2;XP_011247714.1;XP_011247718.1;XP_011247719.1;XP_030110049.1;XP_030110050.1;XP_030110051.1;XP_030110052.1;XP_030110053.1;XP_030110054.1;XP_030110055.1;XP_030110056.1	105050	.	9.366330	[illegible]
17766	NM_001356583,NM_001356584,NM_001356585,NM_008637,XM_006504655	Nudt1	nudix (nucleoside diphosphate linked moiety X)-type motif 1	protein_coding	NP_001343512.1;NP_001343513.1;NP_001343514.1;NP_032663.1;XP_006504718.1	109280	.	3.887260	[illegible]
17768	NM_008638,XM_006505697,XM_011241245	Mthfd2	methylenetetrahydrofolate dehydrogenase (NAD+ dependent), methenyltetrahydrofolate cyclohydrolase	protein_coding	NP_032664.1;XP_006505760.1;XP_011239547.1	1338850	.	14.386270	[illegible]
17769	NM_001161798,NM_010840,NR_027809	Mthfr	methylenetetrahydrofolate reductase	protein_coding	NP_001155270.1;NP_034970.2	106639	.	13.582528	[illegible]
17771	NM_001039657,NM_001039658,NM_001286557,NM_001286558	Tesmin	testis expressed metallothionein like	protein_coding	NP_001034746.1;NP_001034747.1;NP_001273486.1;NP_001273487.	1340029	.	0.303034	[illegible]
17772	NM_001164190,NM_001164191,NM_001164192,NM_001164193,NM_001358113,NM_019926,XM_006527890,XM_030251245,XM_030251246	Mtm1	X-linked myotubular myopathy gene 1	protein_coding	NP_001157662.1;NP_001157663.1;NP_001157664.1;NP_001157665.1;NP_001345042.1;NP_064310.2;XP_006527953.1;XP_030107105.1;XP_030107106.1	1099452	.	1.428359	[illegible]
17773	NM_008639,XM_006509291,XM_006509292,XM_011242176,XM_017312597	Mtnr1a	melatonin receptor 1A	protein_coding	NP_032665.1;XP_006509354.1;XP_006509355.2;XP_011240478.1;XP_017168086.1	102967	.	0.000000	[illegible]
17775	NM_008640	Laptm4a	lysosomal-associated protein transmembrane 4A	protein_coding	NP_032666.2	108017	.	226.919174	[illegible]
17776	NM_001042743,NM_001369061,NM_001369062,NM_008641,XM_006502834,XM_006502835,XM_006502836,XM_006502839,XM_017320018,XM_030253297	Mast2	microtubule associated serine/threonine kinase 2	protein_coding	NP_001036208.1;NP_001355990.1;NP_001355991.1;NP_032667.2;XP_006502897.1;XP_006502898.1;XP_006502899.1;XP_006502902.1;XP_017175507.1;XP_030109157.1	894676	.	15.772987	[illegible]
17777	NM_001163457,NM_001355051,NM_001355052,NM_008642,XM_006501102,XM_006501103	Mttp	microsomal triglyceride transfer protein	protein_coding	NP_001156929.1;NP_001341980.1;NP_001341981.1;NP_032668.2;XP_006501165.1;XP_006501166.1	106926	.	1.303787	[illegible]
17826	NM_023166,NM_181452	Fam89b	family with sequence similarity 89, member B	protein_coding	NP_075655.2;NP_852117.2	106595	.	24.423179	[illegible]
17827	NM_001161824,NM_001357367,NM_013604	Mtx1	metaxin 1	protein_coding	NP_001155296.2;NP_001344296.2;NP_038632.3	103025	.	20.918268	[illegible]
17828	NM_139063,XM_006516573	Bloc1s5	biogenesis of lysosomal organelles complex-1, subunit 5, muted	protein_coding	NP_620702.1;XP_006516636.1	2178598	.	7.732111	[illegible]
17829	NM_013605	Muc1	mucin 1, transmembrane	protein_coding	NP_038633.1	97231	.	5.799687	[illegible]
17831	NM_023566	Muc2	mucin 2	protein_coding	NP_076055.3	1339364	.	0.320234	[illegible]
17833	NM_010844,XM_006508499,XM_006508500	Muc5ac	mucin 5, subtypes A and C, tracheobronchial/gastric	protein_coding	NP_034974.2;XP_006508562.1;XP_006508563.1	104697	.	0.001331	[illegible]
17836	NM_008645,XM_006505698	Mug1	murinoglobulin 1	protein_coding	NP_032671.2;XP_006505761.1	99837	.	0.000000	[illegible]
17840	NM_001163010,NM_001163011,NM_031188	Mup1	major urinary protein 1	protein_coding	NP_001156482.1;NP_001156483.1;NP_112465.2	97233	.	0.000000	[illegible]
17841	NM_001045550,NM_001286096,NM_008647,XM_006537651	Mup2	major urinary protein 2	protein_coding	NP_001039015.1;NP_001273025.1;NP_032673.3;XP_006537714.1	97234	.	0.079855	[illegible]
17844	NM_008649,XM_017320019	Mup5	major urinary protein 5	protein_coding	NP_032675.2;XP_017175508.1	104974	.	0.000000	[illegible]
17846	NM_001361661,NM_144514,XM_006514563,XM_017314316	Commd1	COMM domain containing 1	protein_coding	NP_001348590.1;NP_653097.2;XP_006514626.1;XP_017169805.1	109474	.	33.526237	[illegible]
17847	NM_001190401,XM_006514565,XM_006514566,XM_006514567,XM_017314317,XM_030245632,XM_030245633	Usp34	ubiquitin specific peptidase 34	protein_coding	NP_001177330.2;XP_006514628.1;XP_006514629.1;XP_006514630.1;XP_017169806.1;XP_030101492.1;XP_030101493.1	109473	.	14.350299	[illegible]
17850	NM_008650,XM_017317291	Mmut	methylmalonyl-Coenzyme A mutase	protein_coding	NP_032676.2;XP_017172780.1	97239	.	6.412021	[illegible]
17855	NM_001306205,NM_023556,XM_006530183,XM_006530185	Mvk	mevalonate kinase	protein_coding	NP_001293134.1;NP_076045.1;XP_006530246.1;XP_006530248.1	107624	.	6.865878	[illegible]
17857	NR_003520	Mx1	MX dynamin-like GTPase 1	transcribed_pseudogene	.	97243	.	0.635710	[illegible]
17858	NR_003508	Mx2	MX dynamin-like GTPase 2	transcribed_pseudogene	.	97244	.	0.703823	[illegible]
17859	NM_001008542,NM_001008543,NM_001360345,NM_001360346,NM_001360347,NM_001360348,NM_010847,XM_006526735,XM_006526739,XM_006526741,XM_030250763,XM_030250764,XM_030250765	Mxi1	MAX interactor 1, dimerization protein	protein_coding	NP_001008542.2;NP_001008543.1;NP_001347274.1;NP_001347275.1;NP_001347276.1;NP_001347277.1;NP_034977.1;XP_006526798.1;XP_006526802.1;XP_006526804.2;XP_030106623.1;XP_030106624.1;XP_030106625.1	97245	.	14.704740	[illegible]
17863	NM_001198914,NM_010848	Myb	myeloblastosis oncogene	protein_coding	NP_001185843.1;NP_034978.3	97249	.	0.230631	[illegible]
17864	NM_001290397,NM_008651,XM_006495461,XM_011238349,XM_011238350	Mybl1	myeloblastosis oncogene-like 1	protein_coding	NP_001277326.1;NP_032677.2;XP_006495524.1;XP_011236651.1;XP_011236652.1	99925	.	1.536644	[illegible]
17865	NM_008652,XM_006498873,XM_006498875,XM_006498876,XM_006498878	Mybl2	myeloblastosis oncogene-like 2	protein_coding	NP_032678.1;XP_006498936.1;XP_006498938.1;XP_006498939.1;XP_006498941.1	101785	.	3.165829	[illegible]
17868	NM_008653,XM_006498882,XM_011239341	Mybpc3	myosin binding protein C, cardiac	protein_coding	NP_032679.2;XP_006498945.1;XP_011237643.1	102844	.	0.089112	[illegible]
17869	NM_001177352,NM_001177353,NM_001177354,NM_010849	Myc	myelocytomatosis oncogene	protein_coding	NP_001170823.1;NP_001170824.1;NP_001170825.1;NP_034979.3	97250	.	15.492356	[illegible]
17872	NM_008654	Ppp1r15a	protein phosphatase 1, regulatory subunit 15A	protein_coding	NP_032680.1	1927072	.	8.280099	[illegible]
17873	NM_008655	Gadd45b	growth arrest and DNA-damage-inducible 45 beta	protein_coding	NP_032681.1	107776	.	24.825996	[illegible]
17874	NM_010851	Myd88	myeloid differentiation primary response gene 88	protein_coding	NP_034981.1	108005	.	14.722150	[illegible]
17876	NM_001162417,NM_001162418,NM_010852,XM_006498884,XM_017316131,XM_017316132,XM_017316134,XM_030248228,XR_001781147,XR_001781148,XR_001781149,XR_001781150,XR_001781151,XR_001781152,XR_003951183	Myef2	myelin basic protein expression factor 2, repressor	protein_coding	NP_001155889.1;NP_001155890.1;NP_034982.2;XP_006498947.1;XP_017171620.1;XP_017171621.1;XP_017171623.1;XP_030104088.1	104592	.	13.012350	[illegible]
17877	NM_008656,XM_006513319	Myf5	myogenic factor 5	protein_coding	NP_032682.1;XP_006513382.1	97252	.	0.043865	[illegible]
17878	NM_008657	Myf6	myogenic factor 6	protein_coding	NP_032683.1	97253	.	1.714558	[illegible]
17879	NM_030679,XM_017314318	Myh1	myosin, heavy polypeptide 1, skeletal muscle, adult	protein_coding	NP_109604.1;XP_017169807.1	1339711	.	15.910914	[illegible]
17880	NM_001161775,NM_013607,XM_006521843,XM_006521844,XM_006521845	Myh11	myosin, heavy polypeptide 11, smooth muscle	protein_coding	NP_001155247.1;NP_038635.2;XP_006521906.1;XP_006521907.1;XP_006521908.1	102643	.	1.641206	[illegible]
17882	NM_001039545	Myh2	myosin, heavy polypeptide 2, skeletal muscle, adult	protein_coding	NP_001034634.2	1339710	.	16.225330	[illegible]
17883	NM_001099635,XM_006532412	Myh3	myosin, heavy polypeptide 3, skeletal muscle, embryonic	protein_coding	NP_001093105.1;XP_006532475.1	1339709	.	19.045311	[illegible]
17884	NM_010855	Myh4	myosin, heavy polypeptide 4, skeletal muscle	protein_coding	NP_034985.2	1339713	.	4.877117	[illegible]
17885	NM_177369	Myh8	myosin, heavy polypeptide 8, skeletal muscle, perinatal	protein_coding	NP_796343.2	1339712	.	112.705315	[illegible]
17886	NM_022410	Myh9	myosin, heavy polypeptide 9, non-muscle	protein_coding	NP_071855.2	107717	.	41.191288	[illegible]
17888	NM_001164171,NM_010856,XM_006518677,XM_006518678	Myh6	myosin, heavy polypeptide 6, cardiac muscle, alpha	protein_coding	NP_001157643.1;NP_034986.1;XP_006518740.1;XP_006518741.1	97255	.	0.948229	[illegible]
17896	NM_001355754,NM_001355755,NM_010858,XM_030245634,XM_030245635,XM_030245636,XM_030245637,XM_030245638	Myl4	myosin, light polypeptide 4	protein_coding	NP_001342683.1;NP_001342684.1;NP_034988.2;XP_030101494.1;XP_030101495.1;XP_030101496.1;XP_030101497.1;XP_030101498.1	97267	.	8.451058	[illegible]
17897	NM_001364484,NM_010859	Myl3	myosin, light polypeptide 3	protein_coding	NP_001351413.1;NP_034989.1	97268	.	1.917546	[illegible]
17901	NM_001113387,NM_021285,XM_006495763,XM_011238451	Myl1	myosin, light polypeptide 1	protein_coding	NP_001106858.1;NP_067260.1;XP_006495826.1;XP_011236753.1	97269	.	153.320434	[illegible]
17902	gene-Mylf-ps	Mylf-ps	myosin light chain, alkali, fast skeletal muscle, pseudogene	pseudogene	.	97270	.	12.478088	[illegible]
17904	NM_001317217,NM_001317218,NM_001317219,NM_001317220,NM_010860,XM_030244939	Myl6	myosin, light polypeptide 6, alkali, smooth muscle and non-muscle	protein_coding	NP_001304146.1;NP_001304147.1;NP_001304148.1;NP_001304149.1;NP_034990.1;XP_030100799.1	109318	.	431.314046	[illegible]
17906	NM_010861,XM_006530186	Myl2	myosin, light polypeptide 2, regulatory, cardiac, slow	protein_coding	NP_034991.3;XP_006530249.2	97272	.	15.538651	[illegible]
17907	NM_001347655,NM_016754,XM_006507421	Mylpf	myosin light chain, phosphorylatable, fast skeletal muscle	protein_coding	NP_001334584.1;NP_058034.1;XP_006507484.1	97273	.	228.542776	[illegible]
17909	NM_001353141,NM_001353142,NM_019472,XM_006520025,XM_006520026,XM_030248368,XM_030248369	Myo10	myosin X	protein_coding	NP_001340070.1;NP_001340071.1;NP_062345.2;XP_006520088.1;XP_006520089.1;XP_030104228.1;XP_030104229.1	107716	.	20.645971	[illegible]
17910	NM_001103171,NM_010862,NM_182698,XM_017314319,XM_017314320,XM_017314321,XM_017314322,XM_017314323,XM_017314324,XM_017314325,XM_017314326,XM_017314327,XM_017314328,XM_017314329,XM_017314330,XM_017314331,XR_001779911	Myo15	myosin XV	protein_coding	NP_001096641.1;NP_034992.2;NP_874357.2;XP_017169808.1;XP_017169809.1;XP_017169810.1;XP_017169811.1;XP_017169812.1;XP_017169813.1;XP_017169814.1;XP_017169815.1;XP_017169816.1;XP_017169817.1;XP_017169818.1;XP_017169819.1;XP_017169820.1	1261811	.	0.050308	[illegible]
17912	NM_001161817,NM_001290982,NM_010863,XM_006495764,XM_006495765,XM_006495766,XM_006495767,XM_006495769,XM_017319213,XM_030252028	Myo1b	myosin IB	protein_coding	NP_001155289.1;NP_001277911.1;NP_034993.2;XP_006495827.1;XP_006495828.1;XP_006495829.1;XP_006495830.1;XP_006495832.1;XP_017174702.1;XP_030107888.1	107752	.	46.111345	[illegible]
17913	NM_001080774,NM_001080775,NM_001370611,NM_008659	Myo1c	myosin IC	protein_coding	NP_001074243.1;NP_001074244.1;NP_001357540.1;NP_032685.1	106612	.	32.139089	[illegible]
17916	NM_053214	Myo1f	myosin IF	protein_coding	NP_444444.2	107711	.	1.952802	[illegible]
17918	NM_010864,XM_006510827,XM_006510828,XM_006510829,XM_006510830,XM_006510831,XM_006510832,XM_030244103	Myo5a	myosin VA	protein_coding	NP_034994.2;XP_006510890.1;XP_006510891.1;XP_006510892.1;XP_006510893.1;XP_006510894.1;XP_006510895.1;XP_030099963.1	105976	.	9.555529	[illegible]
17919	NM_201600,XM_006525714,XM_006525715,XM_006525716	Myo5b	myosin VB	protein_coding	NP_963894.1;XP_006525777.1;XP_006525778.1;XP_006525779.1	106598	.	0.059121	[illegible]
17920	NM_001039546,XM_006510835,XM_006510836,XM_006510838,XM_011242667,XM_011242668,XM_011242669,XM_011242670,XM_017313191,XM_030244104,XR_003947803,XR_003947804	Myo6	myosin VI	protein_coding	NP_001034635.2;XP_006510898.1;XP_006510899.1;XP_006510901.1;XP_011240969.1;XP_011240970.1;XP_011240971.1;XP_011240972.1;XP_017168680.1;XP_030099964.1	104785	.	7.813996	[illegible]
17921	NM_001256081,NM_001256082,NM_001256083,NM_008663,XM_006507427,XM_006507428,XM_006507429,XM_011241688,XM_011241689,XM_011241690,XM_011241691	Myo7a	myosin VIIA	protein_coding	NP_001243010.1;NP_001243011.1;NP_001243012.1;NP_032689.2;XP_006507490.1;XP_006507491.1;XP_006507492.1;XP_011239990.1;XP_011239991.1;XP_011239992.1;XP_011239993.1	104510	.	3.132703	[illegible]
17922	NM_032394	Myo7b	myosin VIIB	protein_coding	NP_115770.2	107709	.	0.000000	[illegible]
17925	NM_001142322,NM_001142323,NM_015742,XM_006509566,XM_006509567,XM_006509568,XM_006509569,XM_006509571,XM_006509572,XM_006509575,XM_006509576,XM_006509577,XM_006509578,XM_006509579,XM_006509580,XM_006509581,XM_006509582,XM_006509584,XM_006509585,XM_006509586,XM_017312598,XM_017312599,XM_017312600,XM_017312602,XM_017312603,XM_017312604,XM_017312606,XM_017312607,XM_030243331,XM_030243332,XM_030243333,XM_030243334,XM_030243335,XR_379036	Myo9b	myosin IXb	protein_coding	NP_001135794.1;NP_001135795.1;NP_056557.2;XP_006509629.1;XP_006509630.1;XP_006509631.1;XP_006509632.1;XP_006509634.1;XP_006509635.1;XP_006509638.1;XP_006509639.1;XP_006509640.1;XP_006509641.1;XP_006509642.1;XP_006509643.1;XP_006509644.1;XP_006509645.1;XP_006509647.1;XP_006509648.1;XP_006509649.1;XP_017168087.1;XP_017168088.1;XP_017168089.1;XP_017168091.1;XP_017168092.1;XP_017168093.1;XP_017168095.1;XP_017168096.1;XP_030099191.1;XP_030099192.1;XP_030099193.1;XP_030099194.1;XP_030099195.1	106624	.	22.116695	[illegible]
17926	NM_010865	Myoc	myocilin	protein_coding	NP_034995.3	1202864	.	0.109378	[illegible]
17927	NM_010866	Myod1	myogenic differentiation 1	protein_coding	NP_034996.2	97275	.	0.462887	[illegible]
17928	NM_031189	Myog	myogenin	protein_coding	NP_112466.1	97276	.	2.312626	[illegible]

17929	NM_001083934,NM_010867,XM_006523787,XM_006523788,XM_030249548,XM_030249549,XR_385297	Myom1	myomesin 1	protein_coding	NP_001077403.1;NP_034997.2;XP_006523850.1;XP_006523851.1;XP_030105408.1;XP_030105409.1	1341430	.	12.395264	[illegible]	[illegible]	[illegible]	[illegible]	[illegible]	[illegible]	[illegible]
17930	NM_008664,XM_006508727,XM_006508728	Myom2	myomesin 2	protein_coding	NP_032690.2;XP_006508790.1;XP_006508791.1	1328358	.	9.283532	[illegible]	[illegible]	[illegible]	[illegible]	[illegible]	[illegible]	[illegible]
17931	NM_001368736,NM_001368737,NM_027892,XM_006513327,XM_006513329	Ppp1r12a	protein phosphatase 1, regulatory subunit 12A	protein_coding	NP_001355665.1;NP_001355666.1;NP_082168.1;XP_006513390.1;XP_006513392.1	1309528	.	15.925664	[illegible]	[illegible]	[illegible]	[illegible]	[illegible]	[illegible]	[illegible]
17932	NM_001171615,NM_001171616,NM_001171680,NM_008665,XM_006500577,XM_006500578,XM_006500579,XM_006500580,XM_006500581,XM_011239932,XM_017316169,XR_001781155,XR_37539	Myt1	myelin transcription factor 1	protein_coding	NP_001165086.1;NP_001165087.1;NP_001165151.1;NP_032691.2;XP_006500640.2;XP_006500641.1;XP_006500642.2;XP_006500643.1;XP_006500644.2;XP_011238234.1;XP_017171658.1	1100535	.	0.000000	0.000000	0.000000	0.638253	0.373323	0.015053	0.050956	0.021168
17933	NM_001093775,NM_001093776,NM_001093778,NM_001361655,NM_001361656,NM_001361657,NM_001361658,NM_001361659,NM_001361660,NM_008666,XM_011243818,XM_011243819,XM_011243820,XM_011243821,XM_011243823,XM_011243824,XM_011243825,XM_011243827,XM_011243831,XM_011243832,XM_011243833,XM_011243834,XM_017314969,XM_017314971,XM_017314972,XM_017314974,XM_017314976,XM_017314978,XM_017314980,XM_030246574,XM_030246576,XM_030246577,XM_030246578,XM_030246579,XM_030246580,XM_030246581,XM_030246582,XM_030246583,XM_030246584,XM_030246585,XM_030246586,XM_030246587,XM_030246588,XM_030246589,XM_030246590,XM_030246591,XM_030246592,XM_030246593,XM_030246594,XM_030246595,XM_030246596	Myt1l	myelin transcription factor 1-like	protein_coding	NP_001087244.1;NP_001087245.1;NP_001087247.1;NP_001348584.1;NP_001348585.1;NP_001348586.1;NP_001348587.1;NP_001348588.1;NP_001348589.1;NP_032692.2;XP_011242120.1;XP_011242121.1;XP_011242122.1;XP_011242123.1;XP_011242125.1;XP_011242126.1;XP_011242127.1;XP_011242129.1;XP_011242133.1;XP_011242134.1;XP_011242135.1;XP_011242136.1;XP_017170458.1;XP_017170460.1;XP_017170461.1;XP_017170463.1;XP_017170465.1;XP_017170467.1;XP_017170469.1;XP_030102434.1;XP_030102436.1;XP_030102437.1;XP_030102438.1;XP_030102439.1;XP_030102440.1;XP_030102441.1;XP_030102442.1;XP_030102443.1;XP_030102444.1;XP_030102445.1;XP_030102446.1;XP_030102447.1;XP_030102448.1;XP_030102449.1;XP_030102450.1;XP_030102451.1;XP_030102452.1;XP_030102453.1;XP_030102454.1;XP_030102455.1;XP_030102	1100511	.	0.010579	0.000000	0.025379	4.063244	1.173901	0.946212	0.366160	0.132939
17936	NM_008667,XM_006495770,XM_006495771,XM_030252048	Nab1	Ngfi-A binding protein 1	protein_coding	NP_032693.2;XP_006495833.1;XP_006495834.1;XP_030107908.1	107564	.	12.909654	12.002973	12.106539	12.777835	13.797846	12.436278	13.890779	11.569251
17937	NM_001122895,NM_008668	Nab2	Ngfi-A binding protein 2	protein_coding	NP_001116367.1;NP_032694.2	107563	.	21.088445	23.576473	21.395275	15.310120	27.540652	21.354486	20.467583	22.502368
17938	NM_001113199,NM_001282976,NM_013608	Naca	nascent polypeptide-associated complex alpha polypeptide	protein_coding	NP_001106670.1;NP_001269905.1;NP_038636.2	106095	.	217.761636	176.344519	215.133632	106.299943	211.827365	209.436848	191.037304	192.360117
17939	NM_001355546,NM_008669,XM_006520561,XM_006520563,XM_030248370,XM_030248371,XM_030248372	Naga	N-acetyl galactosaminidase, alpha	protein_coding	NP_001342475.1;NP_032695.3;XP_006520624.1;XP_006520626.1;XP_030104230.1;XP_030104231.1;XP_030104232.1	1261422	.	32.892275	29.133527	32.717271	21.982462	28.051772	24.338454	25.994214	25.566624
17940	NM_008670	Naip1	NLR family, apoptosis inhibitory protein 1	protein_coding	NP_032696.2	1298223	.	0.024165	0.004862	0.026760	0.000000	0.010038	0.008937	0.000000	0.032361
17948	NM_001126182,NM_010872	Naip2	NLR family, apoptosis inhibitory protein 2	protein_coding	NP_001119654.1;NP_035002.2	1298226	.	0.417471	0.416520	0.233626	0.312222	0.419687	0.457351	0.305229	0.504618
17951	NM_010870,XM_011244628,XM_011244631,XM_011244633,XM_017315415,XM_017315416,XM_017315417	Naip5	NLR family, apoptosis inhibitory protein 5	protein_coding	NP_035000.2;XP_011242930.1;XP_011242933.1;XP_011242935.1;XP_017170904.1;XP_017170905.1;XP_017170906.1	1298220	.	0.389701	0.426722	0.330379	0.268014	0.338404	0.264913	0.201642	0.383159
17952	NM_010871	Naip6	NLR family, apoptosis inhibitory protein 6	protein_coding	NP_035001.2	1298222	.	0.181180	0.289045	0.170173	0.158109	0.216187	0.245666	0.169740	0.261012
17954	NM_008671	Nap1l2	nucleosome assembly protein 1-like 2	protein_coding	NP_032697.2	106654	.	0.072091	0.041237	0.168223	0.617230	0.343258	0.290417	0.111389	0.169887
17955	NM_001285489,NM_001285490,NM_008672,XM_006508504,XM_006508505,XM_006508506,XM_006508507,XM_006508508,XM_030242200,XM_030242201,XM_030242202,XM_030242203,XM_030242204,XM_030242205,XM_030242206	Nap1l4	nucleosome assembly protein 1-like 4	protein_coding	NP_001272418.1;NP_001272419.1;NP_032698.1;XP_006508567.1;XP_006508568.1;XP_006508569.1;XP_006508570.1;XP_006508571.1;XP_030098060.1;XP_030098061.1;XP_030098062.1;XP_030098063.1;XP_030098064.1;XP_030098065.1;XP_030098066.1	1316687	.	43.048341	40.531860	42.201823	45.864452	40.089994	42.305369	38.832296	39.919259
17957	NM_019632,XM_030248234	Napb	N-ethylmaleimide sensitive fusion protein attachment protein beta	protein_coding	NP_062606.1;XP_030104094.1	104562	.	1.812975	1.952079	1.467023	4.400699	2.599750	1.529510	1.666311	1.617023
17960	NM_008673	Nat1	N-acetyl transferase 1	protein_coding	NP_032699.1	97279	.	0.111123	0.103066	0.060850	0.135156	0.052292	0.073397	0.142574	0.132654
17961	NM_001168577,NM_010874,XM_006509592	Nat2	N-acetyltransferase 2 (arylamine N-acetyltransferase)	protein_coding	NP_001162049.1;NP_035004.1;XP_006509655.1	109201	.	1.573192	1.964870	1.226172	1.505636	2.145595	1.622948	1.040871	1.648863
17962	NM_008674,XM_011242275	Nat3	N-acetyltransferase 3	protein_coding	NP_032700.1;XP_011240577.1	102537	.	0.000000	0.101633	0.000000	0.000000	0.055581	0.000000	0.058426	0.058944
17965	NM_008675,XM_006538625,XM_030253299,XM_030253300,XM_030253301,XM_030253302	Nbl1	NBL1, DAN family BMP antagonist	protein_coding	NP_032701.2;XP_006538688.1;XP_030109159.1;XP_030109160.1;XP_030109161.1;XP_030109162.1	104591	.	43.309369	29.168766	38.600624	36.709601	90.470458	51.898215	45.971397	45.092283
17966	NM_001252220,NM_001252222,NM_001252223,NM_008676,NR_152788,XM_006532430,XM_006532431,XM_006532432,XM_006532433,XM_006532434,XM_006532435,XM_030245639,XM_030245640,XM_030245641,XM_030245642,XR_388361	Nbr1	NBR1, autophagy cargo receptor	protein_coding	NP_001239149.1;NP_001239151.1;NP_001239152.1;NP_032702.1;XP_006532493.1;XP_006532494.1;XP_006532495.1;XP_006532496.1;XP_006532497.1;XP_006532498.1;XP_030101499.1;XP_030101500.1;XP_030101501.1;XP_030101502.1	108498	.	35.656522	34.798999	31.185503	29.350736	34.742400	24.645575	27.880009	29.521772
17967	NM_001081445,NM_001113204,NM_001311065,NM_010875,XM_006510055,XM_006510056,XM_006510057,XM_006510058,XM_006510059,XM_006510060,XM_006510061,XM_006510062,XM_006510063,XM_006510064,XM_006510065,XM_006510066,XM_006510067,XM_006510068,XM_006510069,XM_006510070,XM_006510071,XM_006510073,XM_006510074,XM_006510075,XM_011242413,XM_011242414,XM_011242415,XM_011242416,XM_017313194,XM_017313195,XM_017313198,XM_030244105,XM_030244106	Ncam1	neural cell adhesion molecule 1	protein_coding	NP_001074914.1;NP_001106675.1;NP_001297994.1;NP_035005.2;XP_006510118.1;XP_006510119.1;XP_006510120.1;XP_006510121.1;XP_006510122.1;XP_006510123.1;XP_006510124.1;XP_006510125.1;XP_006510126.1;XP_006510127.1;XP_006510128.1;XP_006510129.1;XP_006510130.1;XP_006510131.1;XP_006510132.1;XP_006510133.1;XP_006510134.1;XP_006510136.1;XP_006510137.1;XP_006510138.1;XP_011240715.1;XP_011240716.1;XP_011240717.1;XP_011240718.1;XP_017168683.1;XP_017168684.1;XP_017168687.1;XP_030099965.1;XP_030099966.1	97281	.	20.920905	21.998329	19.916885	29.732049	19.114414	22.415428	22.392032	19.396618
17968	NM_001113208,NM_010954	Ncam2	neural cell adhesion molecule 2	protein_coding	NP_001106679.1;NP_035084.1	97282	.	0.052297	0.070809	0.103710	0.718329	0.241761	0.125579	0.103122	0.203011
17969	NM_001286037,NM_010876,NR_104383,XR_377103	Ncf1	neutrophil cytosolic factor 1	protein_coding	NP_001272966.1;NP_035006.3	97283	.	8.481344	7.231907	6.760273	4.907362	9.665229	6.303845	5.590906	6.574369
17970	NM_010877,XM_006529236	Ncf2	neutrophil cytosolic factor 2	protein_coding	NP_035007.1;XP_006529299.1	97284	.	2.198172	3.520407	3.689060	1.772053	3.321146	3.415803	2.716222	3.349500
17972	NM_008677,XM_006520564,XM_006520565,XM_030248373	Ncf4	neutrophil cytosolic factor 4	protein_coding	NP_032703.2;XP_006520627.1;XP_006520628.1;XP_030104233.1	109186	.	2.446081	5.786710	3.359182	2.449567	3.707524	4.227879	4.053295	4.918279
17973	NM_001324530,NM_010878,XM_006510840	Nck1	non-catalytic region of tyrosine kinase adaptor protein 1	protein_coding	NP_001311459.1;NP_035008.2;XP_006510903.1	109601	.	9.410133	9.181567	9.528989	9.009617	9.056160	8.453263	7.856755	8.575853
17974	NM_010879,XM_030252054	Nck2	non-catalytic region of tyrosine kinase adaptor protein 2	protein_coding	NP_035009.3;XP_030107914.1	1306821	.	7.658571	7.401690	7.555847	9.267691	8.842721	6.984360	6.863029	6.874572
17975	NM_010880,XM_006529238	Ncl	nucleolin	protein_coding	NP_035010.3;XP_006529301.1	97286	.	54.952833	43.617604	56.807979	57.193052	59.355628	66.968492	57.213235	61.270810
17977	NM_010881,XM_006515005,XM_006515006,XM_006515007,XM_030246597,XM_030246598	Ncoa1	nuclear receptor coactivator 1	protein_coding	NP_035011.1;XP_006515068.1;XP_006515069.1;XP_006515070.1;XP_030102457.1;XP_030102458.1	1276523	.	4.889726	5.446715	4.495071	6.898066	6.049661	4.716323	4.249697	4.954451
17978	NM_001302702,NM_008678,XM_011238361,XM_017319229,XM_017319231,XM_017319232,XM_017319233,XM_017319234,XM_017319235,XM_017319241,XM_017319243,XM_030252066,XM_030252068,XM_030252070,XM_030252072,XM_030252075,XM_030252077,XM_030252082,XM_030252083,XM_030252087,XM_030252088,XM_030252090,XM_030252093,XM_030252095	Ncoa2	nuclear receptor coactivator 2	protein_coding	NP_001289631.1;NP_032704.2;XP_011236663.1;XP_017174718.1;XP_017174720.1;XP_017174721.1;XP_017174722.1;XP_017174723.1;XP_017174724.1;XP_017174730.1;XP_017174732.1;XP_030107926.1;XP_030107928.1;XP_030107930.1;XP_030107932.1;XP_030107935.1;XP_030107937.1;XP_030107943.1;XP_030107947.1;XP_030107948.1;XP_030107950.1;XP_030107953.1;XP_0301	1276533	.	8.822926	9.463139	8.419546	7.993354	8.932239	7.210134	6.932008	7.773072
17979	NM_008679,XM_006498888,XM_030248240,XM_030248242	Ncoa3	nuclear receptor coactivator 3	protein_coding	NP_032705.2;XP_006498951.1;XP_030104100.1;XP_030104102.1	1276535	.	9.211586	8.813565	9.118375	9.379898	11.984141	9.560980	8.608067	9.582158
17984	NM_010882	Ndn	necdin	protein_coding	NP_035012.2	97290	.	31.295176	34.187386	35.875076	39.033913	26.477171	27.871096	25.493496	26.049595
17986	NM_010883	Ndp	Norrie disease (pseudoglioma) (human)	protein_coding	NP_035013.1	102570	.	0.142734	0.237877	0.192074	0.342836	0.260741	0.156200	0.132461	0.066672
17988	NM_008681	Ndrg1	N-myc downstream regulated gene 1	protein_coding	NP_032707.2	1341799	.	20.962513	20.061522	20.855597	18.371607	22.785999	17.732206	16.649538	20.920938
17991	NM_010885	Ndufa2	NADH:ubiquinone oxidoreductase subunit A2	protein_coding	NP_035015.2	1343103	.	51.549995	59.258732	56.151936	44.817028	37.570724	43.175259	46.083160	44.915070
17992	NM_010886	Ndufa4	Ndufa4, mitochondrial complex associated	protein_coding	NP_035016.1	107686	.	98.340797	133.189789	104.682091	118.462921	86.468422	90.796806	90.734299	103.004646
17993	NM_010887	Ndufs4	NADH:ubiquinone oxidoreductase core subunit S4	protein_coding	NP_035017.2	1343135	.	13.746916	20.567425	14.595000	19.423519	14.173990	15.457048	14.165465	17.022671
17995	NM_133666	Ndufv1	NADH:ubiquinone oxidoreductase core subunit V1	protein_coding	NP_598427.1	107851	.	37.208992	53.765244	36.357395	52.696548	45.343937	40.098457	38.314159	41.993687
17996	NM_010889,XM_006497749,XM_006497751,XM_006497754,XM_006497755,XM_006497757,XM_006497758,XM_006497759,XM_006497760,XM_006497762,XM_006497763,XM_006497764,XM_006497765,XM_006497766,XM_006497767,XM_006497768,XM_006497769,XM_006497770,XM_006497771,XM_006497774,XM_006497775,XM_006497776,XM_011239032,XM_017316181,XM_017316188,XM_017316215,XM_017316216,XM_030248251,XM_030248252	Neb	nebulin	protein_coding	NP_035019.1;XP_006497812.1;XP_006497814.1;XP_006497817.1;XP_006497818.1;XP_006497820.1;XP_006497821.1;XP_006497822.1;XP_006497823.1;XP_006497825.1;XP_006497826.1;XP_006497827.1;XP_006497828.1;XP_006497829.1;XP_006497830.1;XP_006497831.1;XP_006497832.1;XP_006497833.1;XP_006497834.1;XP_006497837.1;XP_006497838.1;XP_006497839.1;XP_011237334.1;XP_017171670.1;XP_017171677.1;XP_017171704.1;XP_017171705.1;XP_030104111.1;XP_030104112.1	97292	.	5.054599	22.671440	2.673894	24.813612	5.140569	0.042416	3.634110	6.611667
17997	NM_008682,XM_006513334	Nedd1	neural precursor cell expressed, developmentally down-regulated gene 1	protein_coding	NP_032708.2;XP_006513397.1	97293	.	6.493631	5.858949	6.093146	5.516641	5.375764	6.109280	5.647736	5.369794
17999	NM_001357998,NM_010890,XM_011242672	Nedd4	neural precursor cell expressed, developmentally down-regulated 4	protein_coding	NP_001344927.1;NP_035020.2;XP_011240974.1	97297	.	287.339637	260.052674	278.770759	283.746865	278.492891	261.127159	243.679457	266.833321
18000	NM_001159717,NM_001159718,NM_001159719,NM_010891,XM_006529240,XM_006529241,XM_006529242,XM_011247934,XM_030252110	Sept2	septin 2	protein_coding	NP_001153189.1;NP_001153190.1;NP_001153191.1;NP_035021.1;XP_006529303.1;XP_006529304.1;XP_006529305.1;XP_011246236.1;XP_030107970.1	97298	.	106.198675	93.920979	99.889545	105.687515	92.269409	108.557870	101.920426	101.406645
18002	NM_008683	Nedd8	neural precursor cell expressed, developmentally down-regulated gene 8	protein_coding	NP_032709.1	97301	.	70.310562	68.527222	72.306114	61.572998	65.198982	73.558853	70.366783	71.557457
18003	NM_001111324,NM_017464,XM_030247179	Nedd9	neural precursor cell expressed, developmentally down-regulated gene 9	protein_coding	NP_001104794.1;NP_059492.3;XP_030103039.1	97302	.	18.152739	17.676235	16.228683	16.242449	20.870577	17.847998	18.473053	15.861569
18004	NM_001293637,NM_001293638,NM_001293639,NM_175089,XM_006509294,XM_030243338,XM_030243339,XM_030243340	Nek1	NIMA (never in mitosis gene a)-related expressed kinase 1	protein_coding	NP_001280566.1;NP_001280567.1;NP_001280568.1;NP_780298.2;XP_006509357.1;XP_030099198.1;XP_030099199.1;XP_030099200.1	97303	.	6.293571	5.199108	5.910483	5.394449	5.250540	4.771426	4.726513	5.478403
18005	NM_010892,XM_006497123	Nek2	NIMA (never in mitosis gene a)-related expressed kinase 2	protein_coding	NP_035022.2;XP_006497186.1	109359	.	3.815572	3.221601	4.102176	3.223497	1.343017	3.752777	3.559800	3.205419
18007	NM_001042752,NM_008684,XM_006510842,XM_006510843,XM_006510844,XM_006510845,XM_006510846,XM_006510847,XM_030244107,XM_030244108	Neo1	neogenin	protein_coding	NP_001036217.1;NP_032710.2;XP_006510905.1;XP_006510906.1;XP_006510907.1;XP_006510908.1;XP_006510909.1;XP_006510910.1;XP_030099967.1;XP_030099968.1	1097159	.	54.415559	44.471115	49.511347	44.133876	56.367559	53.765451	48.014400	50.571760
18008	NM_016701	Nes	nestin	protein_coding	NP_057910.3	101784	.	6.710513	12.340061	6.023055	11.535839	5.922203	6.611828	10.472099	9.002382
18010	NM_010893	Neu1	neuraminidase 1	protein_coding	NP_035023.3	97305	.	5.693305	7.976947	6.286583	5.433187	8.210788	6.638933	7.018980	7.308312
18011	NM_001163480,NM_021360	Neurl1a	neuralized E3 ubiquitin protein ligase 1A	protein_coding	NP_001156952.1;NP_067335.4	1334263	.	1.899050	3.677246	1.715727	3.359150	1.855815	1.093926	1.619852	1.967427
18012	NM_010894	Neurod1	neurogenic differentiation 1	protein_coding	NP_035024.1	1339708	.	0.000000	0.000000	0.000000	1.519746	1.589464	0.159604	0.016816	0.000000
18013	NM_010895,XM_030245643	Neurod2	neurogenic differentiation 2	protein_coding	NP_035025.3;XP_030101503.1	107755	.	0.000000	0.000000	0.027714	5.367463	1.616527	1.437070	0.439419	0.153587
18015	NM_010897,XM_006532436,XM_006532437,XM_006532438,XM_006532439,XM_006532440,XM_006532441,XM_006532442,XM_030245644	Nf1	neurofibromin 1	protein_coding	NP_035027.1;XP_006532499.1;XP_006532500.1;XP_006532501.1;XP_006532502.1;XP_006532503.1;XP_006532504.1;XP_006532505.1;XP_030101504.1	97306	.	6.574489	5.889880	6.200253	6.384458	8.114326	7.348442	6.145979	6.630913
18016	NM_001252250,NM_001252251,NM_001252252,NM_001252253,NM_001361675,NM_001361676,NM_001361677,NM_010898,XM_006514569,XM_006514570,XM_006514571,XM_006514572,XM_006514574,XM_011243669,XM_011243670,XM_030245645	Nf2	neurofibromin 2	protein_coding	NP_001239179.1;NP_001239180.1;NP_001239181.1;NP_001239182.1;NP_001348604.1;NP_001348605.1;NP_001348606.1;NP_035028.2;XP_006514632.1;XP_006514633.1;XP_006514634.1;XP_006514635.1;XP_006514637.1;XP_011241971.1;XP_011241972.1;XP_030101505.1	97307	.	19.734833	21.441555	20.946629	25.538251	25.183702	25.673714	21.872675	22.464828
18018	NM_001164109,NM_001164110,NM_001164111,NM_001164112,NM_016791,NR_198429	Nfatc1	nuclear factor of activated T cells, cytoplasmic, calcineurin dependent 1	protein_coding	NP_001157581.1;NP_001157582.1;NP_001157583.1;NP_001157584.1;NP_058071.2;NP_940821.1	102469	.	21.322836	26.254405	26.807884	20.930676	29.598982	27.756905	31.298783	29.325320
18019	NM_001037177,NM_001037178,NM_001136073,NM_001291168,NM_001291169,NM_001291170,NM_001291171,NM_001291172,NM_001291173,NM_001291174,NM_001291175,NM_001291176,NM_001291177,NM_001291178,NM_001291179,NM_010899,NR_111897,NR_111898	Nfatc2	nuclear factor of activated T cells, cytoplasmic, calcineurin dependent 2	protein_coding	NP_001032254.1;NP_001032255.1;NP_001129545.1;NP_001278097.1;NP_001278098.1;NP_001278099.1;NP_001278100.1;NP_001278101.1;NP_001278102.1;NP_001278103.1;NP_001278104.1;NP_001278105.1;NP_001278106.1;NP_001278107.1;NP_001278108.1;NP_035029.2	102463	.	7.435508	5.800958	6.383219	6.441388	4.753595	4.068420	6.060616	4.745519
18020	NM_010900,XM_006507430	Nfatc2ip	nuclear factor of activated T cells, cytoplasmic, calcineurin dependent 2 interacting protein	protein_coding	NP_035030.2;XP_006507493.1	1329015	.	2.611056	2.628538	2.435283	2.290408	2.391281	2.543410	2.965686	2.312556
18021	NM_001368796,NM_001368797,NM_010901,XM_006530754	Nfatc3	nuclear factor of activated T cells, cytoplasmic, calcineurin dependent 3	protein_coding	NP_001355725.1;NP_001355726.1;NP_035031.2;XP_006530817.1	103296	.	15.211065	14.105269	16.744959	15.893501	16.207480	17.419907	15.029836	16.515100
18022	NM_001302338,NM_001302339,NM_001302340,NM_001302341,NM_001302343,NM_008685,XM_006520569,XM_006520573,XM_030248374,XM_030248375	Nfe2	nuclear factor, erythroid derived 2	protein_coding	NP_001289267.1;NP_001289268.1;NP_001289269.1;NP_001289270.1;NP_001289272.1;NP_032711.2;XP_006520632.1;XP_006520636.1;XP_030104234.1;XP_030104235.1	97308	.	0.494980	1.690264	0.260548	0.520538	0.804104	0.580832	0.204462	1.030212
18023	NM_001130450,NM_001130451,NM_001130452,NM_001130453,NM_001130454,NM_001361682,NM_008686	Nfe2l1	nuclear factor, erythroid derived 2,-like 1	protein_coding	NP_001123922.1;NP_001123923.1;NP_001123924.1;NP_001123925.1;NP_001123926.1;NP_001348611.1;NP_032712.2	99421	.	42.837795	48.154527	44.125152	51.931501	47.685816	43.430613	43.084791	45.266054
18024	NM_010902,NR_132727	Nfe2l2	nuclear factor, erythroid derived 2, like 2	protein_coding	NP_035032.1	108420	.	19.280715	19.123143	16.251119	14.846859	24.690478	16.838514	15.904711	20.867407
18025	NM_010903	Nfe2l3	nuclear factor, erythroid derived 2, like 3	protein_coding	NP_035033.1	1339958	.	0.035517	0.021080	0.090105	0.732446	0.419105	0.196421	0.092645	0.054409
18027	NM_001122952,NM_001122953,NM_010905,XM_006502841,XM_006502842,XM_006502843,XM_006502844,XM_006502845,XM_030253303	Nfia	nuclear factor I/A	protein_coding	NP_001116424.1;NP_001116425.1;NP_035035.1;XP_006502904.1;XP_006502905.1;XP_006502906.1;XP_006502907.1;XP_006502908.1;XP_030109163.1	108056	.	5.253501	4.403013	4.690956	5.400929	5.207660	4.609727	4.765091	4.658567
18028	NM_001113209,NM_001113210,NM_001286127,NM_001286131,NM_008687,XM_006537653,XM_006537654,XM_017320021,XM_017320022,XM_030253304	Nfib	nuclear factor I/B	protein_coding	NP_001106680.1;NP_001106681.1;NP_001273056.1;NP_001273060.1;NP_032713.3;XP_006537716.1;XP_006537717.1;XP_017175510.1;XP_017175511.1;XP_030109164.1	103188	.	11.124811	11.399534	9.746188	15.261759	12.357668	13.259692	11.447031	13.033608
18029	NM_008688,NM_026756,XM_006513335,XM_030244940	Nfic	nuclear factor I/C	[illegible]	NP_032714.1;NP_081032.2;XP_006513398.1;XP_030100800.1	109591	.	27.872355	30.399241	30.789548	38.810504	30.913979	36.353146	34.797368	32.391927

18175	NM_001286552,NM_008733,NM_198059,XM_006526745,XM_006526746,XM_006526747,XM_006526748	Nrap	nebulin-related anchoring protein	protein_coding	[illegible]
18176	NM_001368638,NM_010937,XM_006501118,XM_006501119,XM_006501120,XM_006501121,XM_006501122	Nras	neuroblastoma ras oncogene	protein_coding	[illegible]
18181	NM_001164226,NM_001164227,NM_001164228,NM_001164229,NM_001164230,NM_001361692,NM_001361693,NM_001361694,NM_001361695,NM_010938,XM_006505005,XM_006505006,XM_006505008,XM_011241044,XM_011241046,XM_011241048,XM_017321440,XM_017321441,XM_030255217,XM_030255218,XM_030255219	Nrf1	nuclear respiratory factor 1	protein_coding	[illegible]
18183	NM_001190187,NM_001190188,NM_008734,XM_006518685,XM_006518686,XM_006518687,XM_011244987,XM_017315910,XM_017315911,XM_030247684	Nrg3	neuregulin 3	protein_coding	[illegible]
18185	NM_001136074,NM_001271916,NM_001271917,NM_008736,XM_006518688	Nrl	neural retina leucine zipper gene	protein_coding	[illegible]
18186	NM_001358959,NM_001358960,NM_008737,XM_006530766,XM_006530768,XM_030243349	Nrp1	neuropilin 1	protein_coding	[illegible]
18187	NM_001077403,NM_001077404,NM_001077405,NM_001077406,NM_001077407,NM_010939	Nrp2	neuropilin 2	protein_coding	[illegible]
18188	NM_008738,XM_006523801	Nrtn	neurturin	protein_coding	[illegible]
18189	NM_001346957,NM_001346958,NM_001346959,NM_001346960,NM_001346961,NM_001346962,NM_001347419,NM_020252,NM_177284,XM_006523811,XM_006523812,XM_006523815,XM_006523816,XM_011246318,XM_017317294,XM_017317295,XM_017317296,XM_017317297,XM_017317298,XM_017317299,XM_017317300,XM_017317301,XM_017317302,XM_017317303,XM_017317304,XM_017317306,XM_017317307,XM_017317308,XM_017317309,XM_017317310,XM_017317311,XM_030249557,XM_030249558,XM_030249559,XM_030249560,XM_030249561,XM_030249562,XM_030249563,XM_030249564,XM_030249565,XM_030249566,XM_030249567,XM_030249568,XM_030249569,XM_030249570	Nrxn1	neurexin I	protein_coding	[illegible]
18190	NM_001205234,NM_001205235,NM_001369363,NM_020253	Nrxn2	neurexin II	protein_coding	[illegible]
18191	NM_001198587,NM_001252074,NM_172544,XM_006515534,XM_006515535,XM_006515536,XM_006515537,XM_006515538,XM_006515539,XM_006515540,XM_006515542,XM_006515543,XM_006515544,XM_006515545,XM_006515546,XM_006515547,XM_006515548,XM_006515549,XM_006515551,XM_006515552,XM_006515553,XM_006515554,XM_006515555,XM_006515556,XM_006515557,XM_006515558,XM_006515559,XM_006515560,XM_006515568,XM_006515569,XM_006515570,XM_011244011,XM_017314985,XM_017314988,XM_017314989,XM_030246600,XM_030246601,XM_030246602,XM_030246603,XM_030246604,XM_030246605,XM_030246606,XM_030246607,XM_030246608,XM_030246609,XM_030246610,XR_001780421,XR_001780422,XR_001780424,XR_001780425,	Nrxn3	neurexin III	protein_coding	[illegible]
18193	NM_008739,XM_006517134,XM_006517135,XM_006517136,XM_006517137,XM_006517138,XM_006517140,XM_006517141,XM_006517142,XM_006517143,XM_006517144,XM_006517146,XM_006517147,XM_006517148,XM_011244496,XM_030247180,XM_030247181,XM_030247182,XM_030247183,XM_030247184,XM_030247185,XM_030247186,XM_030247187,XR_382424	Nsd1	nuclear receptor-binding SET-domain protein 1	protein_coding	[illegible]
18194	NM_010941	Nsdhl	NAD(P) dependent steroid dehydrogenase-like	protein_coding	[illegible]
18195	NM_008740	Nsf	N-ethylmaleimide sensitive fusion protein	protein_coding	[illegible]
18196	NM_001359090,NM_001359091,NM_001359092,NM_010942	Nsg1	neuron specific gene family member 1	protein_coding	[illegible]
18197	NM_001290680,NM_001290681,NM_001347080,NM_008741,XM_030245647	Nsg2	neuron specific gene family member 2	protein_coding	[illegible]
18198	NM_001037127,NM_001037128,NM_001037129,NM_001037130,NM_001165996,NM_010944,XM_006537659,XM_006537661	Musk	muscle, skeletal, receptor tyrosine kinase	protein_coding	[illegible]
18201	NM_010945,XM_006537662,XM_006537663,XM_006537664,XM_011249943,XM_011249944,XM_030253309,XR_390314	Nsmaf	neutral sphingomyelinase (N-SMase) activation associated factor	protein_coding	[illegible]
18203	NM_001346106,NM_001346107,NM_001346108,NM_001346109,NM_001346110,NM_001346111,NM_010946,NR_144374,XM_006521850,XM_006521851,XM_006521855,XM_030248995,XM_030248996,XM_030248997,XM_030249000	Ntan1	N-terminal Asn amidase	protein_coding	[illegible]
18205	NM_001164034,NM_001164035,NM_008742,XM_006505715,XM_006505716	Ntf3	neurotrophin 3	protein_coding	[illegible]
18207	NM_001357615,NM_008743,XM_006523819	Nthl1	nth (endonuclease III)-like 1 (E.coli)	protein_coding	[illegible]
18208	NM_008744	Ntn1	netrin 1	protein_coding	[illegible]
18209	NM_010947	Ntn3	netrin 3	protein_coding	[illegible]
18211	NM_001033124,XM_006501124	Ntrk1	neurotrophic tyrosine kinase, receptor, type 1	protein_coding	[illegible]
18212	NM_001025074,NM_001282961,NM_008745,XM_006517149,XM_006517150,XM_006517151,XM_006517152,XM_030247188,XM_030247189	Ntrk2	neurotrophic tyrosine kinase, receptor, type 2	protein_coding	[illegible]
18213	NM_008746,NM_182809,XM_006540691,XM_006540692,XM_011250816,XM_011250817,XM_017322023,XR_001785486,XR_001785487,XR_001785488,XR_882022,XR_882023,XR_882025	Ntrk3	neurotrophic tyrosine kinase, receptor, type 3	protein_coding	[illegible]
18214	NM_022563,XM_006496696,XM_006496697,XM_006496698	Ddr2	discoidin domain receptor family, member 2	protein_coding	[illegible]
18216	NM_018766	Ntsr1	neurotensin receptor 1	protein_coding	[illegible]
18217	NM_001361702,NM_001361703,NM_008747,XM_006515011	Ntsr2	neurotensin receptor 2	protein_coding	[illegible]
18218	NM_008748,XM_006508509,XM_011241989	Dusp8	dual specificity phosphatase 8	protein_coding	[illegible]
18220	NM_001163662,NM_008749,XM_006540696,XM_017322024	Nucb1	nucleobindin 1	protein_coding	[illegible]
18221	NM_010948	Nudc	nudC nuclear distribution protein	protein_coding	[illegible]
18222	NM_001136075,NM_001272055,NM_001272056,NM_010949,NR_073563,NR_073564,XM_006515572,XM_006515573,XM_006515574,XM_006515575,XM_006515576,XM_006515577,XM_006515578,XM_011244012	Numb	NUMB endocytic adaptor protein	protein_coding	[illegible]
18223	NM_010950,XM_006539615,XM_006539616,XM_006539617,XM_011250452,XM_030242207	Numbl	numb-like	protein_coding	[illegible]
18226	NM_053074	Nup62	nucleoporin 62	protein_coding	[illegible]
18227	NM_001139509,NM_013613,XM_011239033,XM_011239034,XR_003951343	Nr4a2	nuclear receptor subfamily 4, group A, member 2	protein_coding	[illegible]
18230	NM_008750	Nxn	nucleoredoxin	protein_coding	[illegible]
18231	NM_008751,XM_006505010	Nxph1	neurexophilin 1	protein_coding	[illegible]
18232	NM_008752,XM_011239035	Nxph2	neurexophilin 2	protein_coding	[illegible]
18241	NM_010951,XM_006528746	Gpr143	G protein-coupled receptor 143	protein_coding	[illegible]
18242	NM_016978	Oat	ornithine aminotransferase	protein_coding	[illegible]
18245	NM_001301034,NM_008753	Oaz1	ornithine decarboxylase antizyme 1	protein_coding	[illegible]
18247	NM_001301307,NM_010952	Oaz2	ornithine decarboxylase antizyme 2	protein_coding	[illegible]
18256	NM_001347038,NM_010953,XM_011245488,XM_011245489,XM_011245490,XM_011245492,XM_011245493,XM_011245494	Oc90	otoconin 90	protein_coding	[illegible]
18260	NM_001360536,NM_001360537,NM_001360538,NM_001360539,NM_008756,XM_006517566	Ocln	occludin	protein_coding	[illegible]
18263	NM_013614,XM_017314990	Odc1	ornithine decarboxylase, structural 1	protein_coding	[illegible]
18285	NM_008757	Odf1	outer dense fiber of sperm tails 1	protein_coding	[illegible]
18286	NM_001113213,NM_001113214,NM_001177659,NM_001177661,NM_001355136,NM_001355137,NM_001355138,NM_001369060,NM_013615,XM_017316272,XM_017316273,XM_017316275,XM_017316276,XM_017316279,XM_017316283,XM_017316288,XM_017316290,XM_017316291,XM_017316292,XM_017316293,XM_017316294,XM_017316295,XM_017316299,XM_017316300,XM_017316301,XM_017316304,XM_030248309,XM_030248311,XM_030248314,XM_030248321,XM_030248327,XR_003951348,XR_003951349,XR_003951350,XR_003951351,XR_003951352,XR_003951353,XR_00	Odf2	outer dense fiber of sperm tails 2	protein_coding	[illegible]
18291	NM_130869,XM_030255220	Nobox	NOBOX oogenesis homeobox	protein_coding	[illegible]
18292	NM_008759	Sebox	SEBOX homeobox	protein_coding	[illegible]
18293	NM_001252282,NM_001252283,NM_001252287,NM_001252288,NM_001361902,NM_001361903,NM_001361904,NM_001361905,NM_010956,XM_006514582,XM_006514584,XM_030245648,XM_030245649,XM_030245650,XM_030245651,XM_030245652	Ogdh	oxoglutarate (alpha-ketoglutarate) dehydrogenase (lipoamide)	protein_coding	[illegible]
18294	NM_010957,NR_149720,XM_006505720,XM_006505722	Ogg1	8-oxoguanine DNA-glycosylase 1	protein_coding	[illegible]
18295	NM_008760	Ogn	osteoglycin	protein_coding	[illegible]
18301	NM_001111073,NM_001287213,NM_001287217,NM_008761	Fxyd5	FXYD domain-containing ion transport regulator 5	protein_coding	[illegible]
18302	NM_010959	Oit3	oncoprotein induced transcript 3	protein_coding	[illegible]
18307	NM_206822	Olfr10	olfactory receptor 10	protein_coding	[illegible]
18315	NM_146563	Olfr18	olfactory receptor 18	protein_coding	[illegible]
18356	NM_010999	Olfr56	olfactory receptor 56	protein_coding	[illegible]
18377	NM_019409	Omg	oligodendrocyte myelin glycoprotein	protein_coding	[illegible]
18378	NM_011010	Omp	olfactory marker protein	protein_coding	[illegible]
18383	NM_008764	Tnfrsf11b	tumor necrosis factor receptor superfamily, member 11b	protein_coding	[illegible]
18386	NM_013622,XM_006538628	Oprd1	opioid receptor, delta 1	protein_coding	[illegible]
18389	NM_001252565,NM_001318919,NM_001318920,NM_001318922,NM_001318923,NM_001318924,NM_001318925,NM_011012,NR_134926,XM_006500582,XM_006500584,XM_017316323,XM_017316325,XM_017316329,XM_017316330,XM_030248361,XM_030248363,XM_030248365,XM_030248366	Oprl1	opioid receptor-like 1	protein_coding	[illegible]
18390	NM_001039652,NM_001302793,NM_001302794,NM_001302795,NM_001302796,NM_001304937,NM_001304938,NM_001304948,NM_001304950,NM_001304955,XM_006512437,XM_017313826,XM_017313827,XM_017313828,XM_017313829	Oprm1	opioid receptor, mu 1	protein_coding	[illegible]
18391	NM_001286538,NM_001286539,NM_001286540,NM_001286541,NM_001286542,NM_001286551,NM_001286605,NM_011014	Sigmar1	sigma non-opioid intracellular receptor 1	protein_coding	[illegible]
18392	NM_011015,XM_011240469,XM_030253310	Orc1	origin recognition complex, subunit 1	protein_coding	[illegible]
18393	NM_001025378,NM_001271526,NM_008765,XM_011238453,XM_030252150	Orc2	origin recognition complex, subunit 2	protein_coding	[illegible]
18399	NM_008766	Slc22a6	solute carrier family 22 (organic anion transporter), member 6	protein_coding	[illegible]
18400	NM_001042760,NM_008767,XM_006508510,XM_006508511,XM_006508512,XM_006508513,XM_006508514,XM_006508515,XM_030242208	Slc22a18	solute carrier family 22 (organic cation transporter), member 18	protein_coding	[illegible]
18405	NM_008768	Orm1	orosomucoid 1	protein_coding	[illegible]
18406	NM_011016	Orm2	orosomucoid 2	protein_coding	[illegible]
18407	NM_013623,XM_006537666,XM_006537667,XM_006537669,XM_006537670	Orm3	orosomucoid 3	protein_coding	[illegible]
18408	NM_001358971,NM_181325	Slc25a15	solute carrier family 25 (mitochondrial carrier ornithine transporter), member 15	protein_coding	[illegible]
18412	NM_001290769,NM_011018	Sqstm1	sequestosome 1	protein_coding	[illegible]
18413	NM_001013365,XM_030245653	Osm	oncostatin M	protein_coding	[illegible]
18414	NM_001310469,NM_011019,XM_006519973,XM_006519976,XM_030248385	Osmr	oncostatin M receptor	protein_coding	[illegible]
18415	NM_011020,XM_006500766,XM_006500767	Hspa4l	heat shock protein 4 like	protein_coding	[illegible]
18416	NM_008769,XM_011247449	Otc	ornithine transcarbamylase	protein_coding	[illegible]
18417	NM_008770	Cldn11	claudin 11	protein_coding	[illegible]
18419	NM_013624,XM_017322025	Otog	otogelin	protein_coding	[illegible]
18423	NM_011023,XM_030245654	Otx1	orthodenticle homeobox 1	protein_coding	[illegible]
18424	NM_001286481,NM_001286482,NM_001286483,NM_001360225,NM_001360226,NM_144841,XM_011244988,XM_011244989,XM_017315914,XM_017315916,XM_017315917,XM_030247685	Otx2	orthodenticle homeobox 2	protein_coding	[illegible]
18429	NM_011025,XM_006498910	Oxt	oxytocin	protein_coding	[illegible]
18430	NM_001081147,XM_006505723	Oxtr	oxytocin receptor	protein_coding	[illegible]
18431	NM_021879,NR_157030,NR_157031,XM_006540699,XM_0065407	Oca2	oculocutaneous albinism II	protein_coding	[illegible]
18432	NM_016776	Mybbp1a	MYB binding protein (P160) 1a	protein_coding	[illegible]
18436	NM_008771,XM_006532451	P2rx1	purinergic receptor P2X, ligand-gated ion channel, 1	protein_coding	[illegible]
18438	NM_001310718,NM_001310720,NM_011026,XM_006530196,XM_030254197	P2rx4	purinergic receptor P2X, ligand-gated ion channel 4	protein_coding	[illegible]
18439	NM_001038839,NM_001038845,NM_001038887,NM_001284402,NM_011027	P2rx7	purinergic receptor P2X, ligand-gated ion channel, 7	protein_coding	[illegible]
18440	NM_001159561,NM_011028,XR_003951768	P2rx6	purinergic receptor P2X, ligand-gated ion channel, 6	protein_coding	[illegible]
18441	NM_001282016,NM_008772	P2ry1	purinergic receptor P2Y, G-protein coupled 1	protein_coding	[illegible]
18442	NM_001302346,NM_001302347,NM_008773	P2ry2	purinergic receptor P2Y, G-protein coupled 2	protein_coding	[illegible]
18451	NM_001316370,NM_001316371,NM_011030,XR_001779474,XR_001779475,XR_003948602,XR_003948603,XR_380368,XR_380369	P4ha1	procollagen-proline, 2-oxoglutarate 4-dioxygenase (proline 4-hydroxylase), alpha 1 polypeptide	protein_coding	[illegible]
18452	NM_001136076,NM_011031,XM_006532452	P4ha2	procollagen-proline, 2-oxoglutarate 4-dioxygenase (proline 4-hydroxylase), alpha II polypeptide	protein_coding	[illegible]
18453	NM_011032	P4hb	prolyl 4-hydroxylase, beta polypeptide	protein_coding	[illegible]
18457	NM_019788	Bloc1s6	biogenesis of lysosomal organelles complex-1, subunit 6, pallidin	protein_coding	[illegible]
18458	NM_008774,XM_011245339	Pabpc1	poly(A) binding protein, cytoplasmic 1	protein_coding	[illegible]
18472	NM_013625,NR_037610	Pafah1b1	platelet-activating factor acetylhydrolase, isoform 1b, subunit 1	protein_coding	[illegible]
18475	NM_001357238,NM_001357239,NM_001357240,NM_001357241,NM_001357243,NM_001357244,NM_001357245,NM_001357246,NM_008775,XM_030244123,XM_030244124	Pafah1b2	platelet-activating factor acetylhydrolase, isoform 1b, subunit 2	protein_coding	[illegible]
18476	NM_001357350,NM_008776,XM_017322027	Pafah1b3	platelet-activating factor acetylhydrolase, isoform 1b, subunit 3	protein_coding	[illegible]
18477	NM_011034	Prdx1	peroxiredoxin 1	protein_coding	[illegible]
18479	NM_001357362,NM_001357363,NM_001357364,NM_001357365,NM_011035,XM_006507436,XM_006507437,XM_011241694	Pak1	p21 (RAC1) activated kinase 1	protein_coding	[illegible]
18481	NM_001195046,NM_001195047,NM_001195048,NM_001195049,NM_008778,XM_006528747,XM_006528752,XM_006528753,XM_006528754,XM_011247790,XM_017318417,XM_017318418,XM_017318419,XM_017318420,XM_017318421,XM_030251247,XM_030251248,XM_030251249,XM_030251250,XM_030251251	Pak3	p21 (RAC1) activated kinase 3	protein_coding	[illegible]
18483	NM_001161747,NM_023128,XM_017313830,XM_017313831	Palm	paralemmin	protein_coding	[illegible]
18484	NM_001357127,NM_013626,XM_006529243,XM_011247937,XM_011247938,XM_011247939,XM_017319282,XM_017319284,XM_017319286,XM_017319289,XM_017319290,XM_030252162,XM_030252165,XM_030252167,XM_030252177,XM_030252184,XM_030252185,XM_030252186,XM_030252187,XM_030252190	Pam	peptidylglycine alpha-amidating monooxygenase	protein_coding	[illegible]
18488	NM_008779,XM_006505725,XM_006505727,XM_006505728,XM_006505729,XM_011241248,XM_017321448,XR_001785107,XR_377	Cntn3	contactin 3	protein_coding	[illegible]
18491	NM_021362	Pappa	pregnancy-associated plasma protein A	protein_coding	[illegible]
18503	NM_008780,XM_006498911	Pax1	paired box 1	protein_coding	[illegible]
18504	NM_001368743,NM_001368744,NM_001368745,NM_001368746,NM_001368747,NM_001368748,NM_001368749,NM_001368750,NM_001368751,NM_011037	Pax2	paired box 2	protein_coding	[illegible]
18505	NM_001159520,NM_008781	Pax3	paired box 3	protein_coding	[illegible]
18507	NM_008782	Pax5	paired box 5	protein_coding	[illegible]
18508	NM_001244198,NM_001244200,NM_001244201,NM_001244202,NM_001310144,NM_001310145,NM_001310146,NM_013627	Pax6	paired box 6	protein_coding	[illegible]
18509	NM_011039,XM_006538629,XM_006538630,XM_006538631	Pax7	paired box 7	protein_coding	[illegible]
18510	NM_011040	Pax8	paired box 8	protein_coding	[illegible]
18511	NM_011041,XM_006515579,XM_006515580,XM_006515581,XM_011244013,XM_017314991	Pax9	paired box 9	protein_coding	[illegible]
18514	NM_001291508,NM_001291509,NM_008783,NM_183355,XM_006496699,XM_006496700	Pbx1	pre B cell leukemia homeobox 1	protein_coding	[illegible]
18515	NM_017463	Pbx2	pre B cell leukemia homeobox 2	protein_coding	[illegible]
18516	NM_001290576,NM_016768,XM_006497782,XM_006497784,XM_030248420	Pbx3	pre B cell leukemia homeobox 3	protein_coding	[illegible]
18518	NM_008784	Igbp1	immunoglobulin (CD79A) binding protein 1	protein_coding	[illegible]
18519	NM_001190846,NM_020005,NR_151733,XM_006523820,XR_001782056	Kat2b	K(lysine) acetyltransferase 2B	protein_coding	[illegible]
18521	NM_001103165,NM_001103166,NM_001174073,NM_011042,XM_006520582,XM_006520583,XM_006520584,XM_006520585,XM_006520586,XM_011245495,XM_011245496,XM_011245497,XM_011245498,XM_011245499,XM_011245500,XM_011245501,XM_011245502,XM_011245503,XM_011245504,XM_011245505,XM_011245506,XM_011245507,XM_011245508,XM_011245509,XM_017316497,XM_017316498,XM_017316499,XM_017316500,XM_017316501,XM_030248386,XM_030248387,XM_030248388,XM_030248389,XM_030248390,XM_030248391,XM_030248392,XM_030248393,XM_030248394,XM_030248395,XM_030248396,XM_030248397,XM_030248398,XM_030248399,XM_030248400,XM_030248401,XM_030248402,XM_030248403,XM_030248404,XM_030248405,XM_030248406	Pcbp2	poly(rC) binding protein 2	protein_coding	[illegible]
18526	NM_001098170,NM_001098171,NM_001098172,NM_011043	Pcdh10	protocadherin 10	protein_coding	[illegible]
18530	NM_001042726,NM_021543	Pcdh8	protocadherin 8	protein_coding	[illegible]
18534	NM_011044	Pck1	phosphoenolpyruvate carboxykinase 1, cytosolic	protein_coding	[illegible]
18536	NM_023662,XM_006509298,XM_006509299,XM_006509300,XM_006509301,XM_006509302,XM_006509304,XM_011242179,XM_017312615,XR_001778411	Pcm1	pericentriolar material 1	protein_coding	[illegible]
18537	NM_001347228,NM_001358647,NM_001358649,NM_001358651,NM_001358652,NM_001358653,NM_001358654,NM_001358657,NM_008786,XM_006512589,XM_006512595,XM_011243140,XM_011243141,XM_017313832,XM_030244942,XM_030244943,XM_030244944,XM_030244945,XM_030244946,XM_030244947,XM_030244948,XM_030244949	Pcmt1	protein-L-isoaspartate (D-aspartate) O-methyltransferase 1	protein_coding	[illegible]
18538	NM_011045	Pcna	proliferating cell nuclear antigen	protein_coding	[illegible]

18540	gene-Pcna-ps2	Pcna-ps2	proliferating cell nuclear antigen pseudogene 2	pseudogene	[illegible]
18541	NM_001282992,NM_008787,XM_006513348,XM_006513353,XM_006513356,XM_011243382,XM_011243383,XM_011243384,XM_017313838,XM_017313839,XM_017313840,XM_017313841,XM_017313842,XM_017313843,XM_017313844,XM_017313845,XM_030244950	Pcnt	pericentrin (kendrin)	protein_coding	[illegible]
18542	NM_008788,XM_006504558	Pcolce	procollagen C-endopeptidase enhancer protein	protein_coding	[illegible]
18545	NM_001129803,NM_001129804,NM_008790,XM_006508729,XM_006508730,XM_006508731,XM_006508732,XM_006508733,XM_030243350,XM_030243352	Pcp2	Purkinje cell protein 2 (L7)	protein_coding	[illegible]
18546	NM_008791	Pcp4	Purkinje cell protein 4	protein_coding	[illegible]
18548	NM_013628,XM_006517153,XM_006517154,XM_006517155	Pcsk1	proprotein convertase subtilisin/kexin type 1	protein_coding	[illegible]
18549	NM_008792	Pcsk2	proprotein convertase subtilisin/kexin type 2	protein_coding	[illegible]
18550	NM_001081454,NM_011046,XM_011250818,XM_011250819,XM_011250820,XM_011250821,XM_011250822,XM_011250823,XM_030242209,XM_030242210	Furin	furin (paired basic amino acid cleaving enzyme)	protein_coding	[illegible]
18551	NM_008793,XM_006513358,XM_006513359,XM_006513360,XM_006513361,XM_006513362,XM_006513363,XM_006513364,XM_006513365,XM_006513368,XM_006513369,XM_030244951,XR_001779482,XR_001779483,XR_003948604,XR_003948605,XR_003948606,XR_003948607,XR_003948608,XR_003948609,XR_003948610,XR_003948611,XR_003948612,XR_003948613,XR_003948614,XR_003948615,XR_380370,XR_380371,XR_380372,XR_380374,XR_3	Pcsk4	proprotein convertase subtilisin/kexin type 4	protein_coding	[illegible]
18552	NM_001163144,NM_001190483,XM_006526759,XM_006526760,XM_006526761,XM_011247162,XM_017318080,XM_030250766,XR_386446	Pcsk5	proprotein convertase subtilisin/kexin type 5	protein_coding	[illegible]
18553	NM_001291184,NM_011048	Pcsk6	proprotein convertase subtilisin/kexin type 6	protein_coding	[illegible]
18554	NM_001281934,NM_008794,NR_104055,XM_006510088,XM_006510089,XM_006510090,XR_001778807,XR_001778808,XR_001778809,XR_001778810,XR_001778811,XR_870526	Pcsk7	proprotein convertase subtilisin/kexin type 7	protein_coding	[illegible]
18555	NM_001310456,NM_011049,XM_006527570,XM_017318424,XM_017318425	Cdk16	cyclin-dependent kinase 16	protein_coding	[illegible]
18557	NM_008795,XM_011247940,XR_001783189,XR_003953745,XR_387152	Cdk18	cyclin-dependent kinase 18	protein_coding	[illegible]
18559	NM_001316372,NM_008796	Pctp	phosphatidylcholine transfer protein	protein_coding	[illegible]
18563	NM_001162946,NM_008797,XM_006531678	Pcx	pyruvate carboxylase	protein_coding	[illegible]
18566	NM_008798	Pdcd1	programmed cell death 1	protein_coding	[illegible]
18567	NM_008799,XM_030249571	Pdcd2	programmed cell death 2	protein_coding	[illegible]
18569	NM_001168491,NM_001168492,NM_011050,XM_006526762,XM_030250768,XM_030250769,XM_030250770	Pdcd4	programmed cell death 4	protein_coding	[illegible]
18570	NM_001359996,NM_011051	Pdcd6	programmed cell death 6	protein_coding	[illegible]
18571	NM_001164677,NM_001164678,NM_011052,XM_011242941,XM_030244125	Pdcd6ip	programmed cell death 6 interacting protein	protein_coding	[illegible]
18572	NM_011053,XR_003952751,XR_003952752,XR_877666	Pdcd11	programmed cell death 11	protein_coding	[illegible]
18573	NM_001009978,NM_001009979,NM_001159582,NM_001355140,NM_001355141,NM_001355142,NM_001355144,NM_016744,XM_006498919,XM_011239356,XM_030248436	Pde1a	phosphodiesterase 1A, calmodulin-dependent	protein_coding	[illegible]
18574	NM_001285890,NM_001357980,NM_008800,XM_030248407	Pde1b	phosphodiesterase 1B, Ca2+-calmodulin dependent	protein_coding	[illegible]
18575	NM_001025568,NM_001159952,NM_001159953,NM_001159955,NM_001159956,NM_001159957,NM_001159960,NM_001355475,NM_011054,XM_006505730,XM_006505733,XM_006505734,XM_006505735,XM_017321454,XM_030255221,XR_003956167,XR_003956168,XR_003956169,XR_003956170,XR_003956171,XR_003956172,XR_377431	Pde1c	phosphodiesterase 1C	protein_coding	[illegible]
18576	NM_011055,XM_006507438,XR_001785490,XR_003946416	Pde3b	phosphodiesterase 3B, cGMP-inhibited	protein_coding	[illegible]
18577	NM_001310750,NM_019798,NM_183408,XM_006510091,XM_006510093,XM_017313211,XM_030244126,XM_030244127,XR_003947813	Pde4a	phosphodiesterase 4A, cAMP specific	protein_coding	[illegible]
18578	NM_001177980,NM_001177981,NM_001177982,NM_001177983,NM_019840,XM_006502855,XM_006502856,XM_006502857	Pde4b	phosphodiesterase 4B, cAMP specific	protein_coding	[illegible]
18582	NM_001360816,NM_001360817,NM_008801,XM_030252232	Pde6d	phosphodiesterase 6D, cGMP-specific, rod, delta	protein_coding	[illegible]
18583	NM_001122759,NM_008802,XM_006535405,XM_006535406,XM_006535408,XM_011249666,XM_030252456,XR_389588	Pde7a	phosphodiesterase 7A	protein_coding	[illegible]
18584	NM_008803	Pde8a	phosphodiesterase 8A	protein_coding	[illegible]
18585	NM_001163748,NM_008804,XM_006523821,XM_017317319,XR_001782057	Pde9a	phosphodiesterase 9A	protein_coding	[illegible]
18588	NM_012065,NR_126089,XM_006532455	Pde6g	phosphodiesterase 6G, cGMP-specific, rod, gamma	protein_coding	[illegible]
18590	NM_001363271,NM_008808,NR_156469,XM_006504659,XM_011240971,XM_030254198,XM_030254199	Pdgfa	platelet derived growth factor, alpha	protein_coding	[illegible]
18591	NM_011057,XM_006520592,XM_030248408,XM_030248409,XM_030248410,XM_030248411	Pdgfb	platelet derived growth factor, B polypeptide	protein_coding	[illegible]
18595	NM_001083316,NM_001347718,NM_001347719,NM_011058,NR_144636,XM_006504261,XM_006504262,XM_006504263	Pdgfra	platelet derived growth factor receptor, alpha polypeptide	protein_coding	[illegible]
18596	NM_001146268,NM_008809,XM_011246856,XM_030250361	Pdgfrb	platelet derived growth factor receptor, beta polypeptide	protein_coding	[illegible]
18597	NM_008810	Pdha1	pyruvate dehydrogenase E1 alpha 1	protein_coding	[illegible]
18598	NM_008811	Pdha2	pyruvate dehydrogenase E1 alpha 2	protein_coding	[illegible]
18599	NM_011059	Padi1	peptidyl arginine deiminase, type I	protein_coding	[illegible]
18600	NM_008812,XM_006538632	Padi2	peptidyl arginine deiminase, type II	protein_coding	[illegible]
18601	NM_011060	Padi3	peptidyl arginine deiminase, type III	protein_coding	[illegible]
18602	NM_011061,XM_006538633	Padi4	peptidyl arginine deiminase, type IV	protein_coding	[illegible]
18604	NM_001361915,NM_133667	Pdk2	pyruvate dehydrogenase kinase, isoenzyme 2	protein_coding	[illegible]
18605	NM_001308327,NM_001308329,NM_008813,XM_006512598,XM_006512599	Enpp1	ectonucleotide pyrophosphatase/phosphodiesterase 1	protein_coding	[illegible]
18606	NM_001136077,NM_001285994,NM_001285995,NM_015744,XM_006520593,XM_006520594,XM_006520595,XM_006520596,XM_006520598,XM_011245510,XM_017316504,XM_017316505,XM_017316507	Enpp2	ectonucleotide pyrophosphatase/phosphodiesterase 2	protein_coding	[illegible]
18607	NM_001080773,NM_001286662,NM_011062,XM_017317320,XM_017317321,XM_017317322,XM_017317323,XM_017317324,XM_017317325,XM_017317326,XM_030249572,XM_030249573,XM_030249574,XM_030249575	Pdpk1	3-phosphoinositide dependent protein kinase 1	protein_coding	[illegible]
18610	NM_001286502,NM_018863	Pdyn	prodynorphin	protein_coding	[illegible]
18611	NM_001329869,NM_001329871,NM_011063,XM_006496702	Pea15a	phosphoprotein enriched in astrocytes 15A	protein_coding	[illegible]
18612	NM_001316365,NM_001316366,NM_008815,XM_006532458,XM_006532459,XM_006532460,XM_006532461,XM_006532462,XM_030245655,XM_030245656	Etv4	ets variant 4	protein_coding	[illegible]
18613	NM_001032378,NM_001305157,NM_001305158,NM_008816	Pecam1	platelet/endothelial cell adhesion molecule 1	protein_coding	[illegible]
18616	NM_008817,XM_017322029,XM_017322030,XM_017322031,XM_017322033,XM_030242211,XM_030242212,XM_030242213	Peg3	paternally expressed 3	protein_coding	[illegible]
18617	NM_008818	Rhox5	reproductive homeobox 5	protein_coding	[illegible]
18618	NM_001290011,NM_001290012,NM_001290013,NM_001290014,NM_008819,XM_006532478	Pemt	phosphatidylethanolamine N-methyltransferase	protein_coding	[illegible]
18619	NM_001002927,NM_001348209	Penk	preproenkephalin	protein_coding	[illegible]
18624	NM_008820,XM_006539622	Pepd	peptidase D	protein_coding	[illegible]
18626	NM_001159367,NM_011065,XM_006532480,XM_006532481,XM_030245657	Per1	period circadian clock 1	protein_coding	[illegible]
18627	NM_011066,XM_006529249,XM_006529250	Per2	period circadian clock 2	protein_coding	[illegible]
18628	NM_001289877,NM_001289878,NM_011067,XM_011250203,XM_017320031,XM_017320032,XM_017320033,XM_017320034,XM_017320036,XM_030253311,XR_001784108,XR_003954892	Per3	period circadian clock 3	protein_coding	[illegible]
18631	NM_011068,NR_153403,XM_011250824	Pex11a	peroxisomal biogenesis factor 11 alpha	protein_coding	[illegible]
18632	NM_001162387,NM_001162388,NM_011069,NR_027844,NR_0278	Pex11b	peroxisomal biogenesis factor 11 beta	protein_coding	[illegible]
18633	NM_145122,NR_028114	Pex16	peroxisomal biogenesis factor 16	protein_coding	[illegible]
18634	NM_001161825,NM_008822	Pex7	peroxisomal biogenesis factor 7	protein_coding	[illegible]
18636	NM_008823	Cfp	complement factor properdin	protein_coding	[illegible]
18637	NM_001360824,NM_001360825,NM_011070	Pfdn2	prefoldin 2	protein_coding	[illegible]
18639	NM_008824,XM_006528755,XM_006528756,XM_006528757,XM_011247791,XM_030251252,XM_030251253,XM_030251254	Pfkfb1	6-phosphofructo-2-kinase/fructose-2,6-biphosphatase 1	protein_coding	[illegible]
18640	NM_001162415,NM_001162416,NM_001368843,NM_008825,NR_027859	Pfkfb2	6-phosphofructo-2-kinase/fructose-2,6-biphosphatase 2	protein_coding	[illegible]
18641	NM_001358793,NM_008826	Pfkl	phosphofructokinase, liver, B-type	protein_coding	[illegible]
18642	NM_001163487,NM_001163488,NM_001357688,NM_021514,XM_006520601,XM_030248412	Pfkm	phosphofructokinase, muscle	protein_coding	[illegible]
18643	NM_011072,XM_030245658	Pfn1	profilin 1	protein_coding	[illegible]
18645	NM_019410	Pfn2	profilin 2	protein_coding	[illegible]
18646	NM_011073,XM_006513370	Prf1	perforin 1 (pore forming protein)	protein_coding	[illegible]
18647	NM_001310448,NM_011074,XM_006503544,XM_006503547,XM_017320708	Cdk14	cyclin-dependent kinase 14	protein_coding	[illegible]
18648	NM_023418	Pgam1	phosphoglycerate mutase 1	protein_coding	[illegible]
18654	NM_001271705,NM_008827,XM_006515585,XM_011244016	Pgf	placental growth factor	protein_coding	[illegible]
18655	NM_008828	Pgk1	phosphoglycerate kinase 1	protein_coding	[illegible]
18667	NM_008829,XM_006509848	Pgr	progesterone receptor	protein_coding	[illegible]
18669	NM_011075,XM_030254200	Abcb1b	ATP-binding cassette, sub-family B (MDR/TAP), member 1B	protein_coding	[illegible]
18670	NM_008830,XM_006503551,XM_006503552,XM_006503553,XM_006503554	Abcb4	ATP-binding cassette, sub-family B (MDR/TAP), member 4	protein_coding	[illegible]
18671	NM_011076,XM_006503556	Abcb1a	ATP-binding cassette, sub-family B (MDR/TAP), member 1A	protein_coding	[illegible]
18673	NM_008831	Phb	prohibitin	protein_coding	[illegible]
18674	NM_133668,XM_006513371	Slc25a3	solute carrier family 25 (mitochondrial carrier, phosphate carrier),	protein_coding	[illegible]
18675	NM_011077	Phex	phosphate regulating endopeptidase homolog, X-linked	protein_coding	[illegible]
18676	NM_011078	Phf2	PHD finger protein 2	protein_coding	[illegible]
18679	NM_008832,NM_173021,XM_006527894,XM_006527895,XM_017318427	Phka1	phosphorylase kinase alpha 1	protein_coding	[illegible]
18682	NM_011079,NR_145486,NR_145487,XM_011240871	Phkg1	phosphorylase kinase gamma 1	protein_coding	[illegible]
18685	NM_001163467,NM_001163468,NM_001163469,NM_013629,XM_006501125,XM_006501126,XM_017319483,XM_017319484,XM_030252457,XM_030252458,XM_030252459	Phtf1	putative homeodomain transcription factor 1	protein_coding	[illegible]
18689	NR_028271	Phxr4	per-hexamer repeat gene 4	lncRNA	[illegible]
18693	NM_001045558,NM_008837,XM_006520603,XM_006520604,XM_006520605,XM_006520607,XM_030248413,XM_030248414,XM_030248415,XR_003951360	Pick1	protein interacting with C kinase 1	protein_coding	[illegible]
18700	NM_011081,NR_152315,XM_011247793,XM_017318428	Piga	phosphatidylinositol glycan anchor biosynthesis, class A	protein_coding	[illegible]
18701	NM_008838	Pigf	phosphatidylinositol glycan anchor biosynthesis, class F	protein_coding	[illegible]
18703	NM_011082	Pigr	polymeric immunoglobulin receptor	protein_coding	[illegible]
18704	NM_011083,XM_006507440,XM_006507441,XM_030242214	Pik3c2a	phosphatidylinositol-4-phosphate 3-kinase catalytic subunit type 2 alpha	protein_coding	[illegible]
18705	NM_011084,NM_207683	Pik3c2g	phosphatidylinositol-4-phosphate 3-kinase catalytic subunit type 2	protein_coding	[illegible]
18706	NM_008839,XM_006535409,XM_006535410	Pik3ca	phosphatidylinositol-4,5-bisphosphate 3-kinase catalytic subunit alpha	protein_coding	[illegible]
18707	NM_001029837,NM_001164049,NM_001164050,NM_001164051,NM_001164052,NM_008840,XM_006538641,XM_006538642,XM_006538643,XM_006538644,XM_006538645,XM_006538646,XM_006538647,XM_006538648,XM_006538649,XM_006538650,XM_006538651,XM_006538652,XM_006538653,XM_017320037,XM_017320038,XM_030253312,XM_030253313,XM_030253314,XM_030253315	Pik3cd	phosphatidylinositol-4,5-bisphosphate 3-kinase catalytic subunit delta	protein_coding	[illegible]
18708	NM_001024955,NM_001077495,XM_006517569,XM_006517570	Pik3r1	phosphoinositide-3-kinase regulatory subunit 1	protein_coding	[illegible]
18709	NM_008841	Pik3r2	phosphoinositide-3-kinase regulatory subunit 2	protein_coding	[illegible]
18710	NM_001355584,NM_001355585,NM_001355586,NM_181585,XM_006502859,XM_006502860,XM_006502861,XM_030253316	Pik3r3	phosphoinositide-3-kinase regulatory subunit 3	protein_coding	[illegible]
18711	NM_001310624,NM_011086,XM_006495776,XM_006495777,XM_006495779,XM_006495781,XM_006495782,XM_011238454,XM_030252267,XM_030252271	Pikfyve	phosphoinositide kinase, FYVE type zinc finger containing	protein_coding	[illegible]
18712	NM_001364913,NM_008842	Pim1	proviral integration site 1	protein_coding	[illegible]
18715	NM_138606	Pim2	proviral integration site 2	protein_coding	[illegible]
18717	NM_001146687,NM_001293646,NM_001293647,NM_008844,XM_030244952,XR_003948616,XR_003948617,XR_003948618,XR_003948619,XR_003948620,XR_003948621,XR_380377	Pip5k1c	phosphatidylinositol-4-phosphate 5-kinase, type 1 gamma	protein_coding	[illegible]
18718	NM_001355146,NM_001355147,NM_001355148,NM_001355149,NM_008845,XM_006497394,XM_006497396,XM_006497397	Pip4k2a	phosphatidylinositol-5-phosphate 4-kinase, type II, alpha	protein_coding	[illegible]
18719	NM_008846,XM_006526763	Pip5k1b	phosphatidylinositol-4-phosphate 5-kinase, type 1 beta	protein_coding	[illegible]
18720	NM_001293707,NM_008847,XM_017319485,XR_003954295,XR_003954296,XR_375498,XR_375499	Pip5k1a	phosphatidylinositol-4-phosphate 5-kinase, type 1 alpha	protein_coding	[illegible]
18722	NM_001371068,NR_163846,XM_017322036,XM_017322037,XM_017322038,XM_030242215	Pira1	paired-Ig-like receptor A1	protein_coding	[illegible]
18725	NM_011089,XM_006539623,XM_006539625,XM_017322040,XM_017322041	Pira2	paired-Ig-like receptor A2	protein_coding	[illegible]
18726	NM_011090,NR_028115,XM_006539629,XM_006539630,XM_006539631,XM_006539632	Lilra6	leukocyte immunoglobulin-like receptor, subfamily A (with TM domain), member 6	protein_coding	[illegible]
18733	NM_001357394,NM_011095,XM_006539634,XM_006539635,XM_030242216,XR_003946417	Pirb	paired Ig-like receptor B	protein_coding	[illegible]
18738	NM_008850	Pitpna	phosphatidylinositol transfer protein, alpha	protein_coding	[illegible]
18739	NM_008851,NR_075078,XM_006531680,XM_030250773,XR_003952753,XR_003952754,XR_003952755,XR_388251	Pitpnm1	phosphatidylinositol transfer protein, membrane-associated 1	protein_coding	[illegible]
18740	NM_011097,XM_006517157,XM_006517158	Pitx1	paired-like homeodomain transcription factor 1	protein_coding	[illegible]
18741	NM_001042502,NM_001042504,NM_001286942,NM_001287048,NM_011098,XM_006501131,XM_006501132,XM_006501133,XM_030252460	Pitx2	paired-like homeodomain transcription factor 2	protein_coding	[illegible]
18742	NM_008852,XM_006526764	Pitx3	paired-like homeodomain transcription factor 3	protein_coding	[illegible]
18744	NM_001083110,NM_001290555,NM_001290556,NM_008853,XM_011247544,XM_030251255,XM_030251256	Pja1	praja ring finger ubiquitin ligase 1	protein_coding	[illegible]
18746	NM_001253883,NM_011099,XM_006510855,XM_006510856,XM_011242673,XM_011242674	Pkm	pyruvate kinase, muscle	protein_coding	[illegible]
18747	NM_001277898,NM_008854	Prkaca	protein kinase, cAMP dependent, catalytic, alpha	protein_coding	[illegible]
18749	NM_001164198,NM_001164199,NM_001164200,NM_011100	Prkacb	protein kinase, cAMP dependent, catalytic, beta	protein_coding	[illegible]
18750	NM_011101	Prkca	protein kinase C, alpha	protein_coding	[illegible]
18751	NM_001316672,NM_008855,XM_030242217	Prkcb	protein kinase C, beta	protein_coding	[illegible]
18752	NM_001291434,NM_011102,XM_030242218	Prkcg	protein kinase C, gamma	protein_coding	[illegible]
18753	NM_001310682,NM_011103,XM_006518695,XM_006518697,XM_006518698,XM_017315918,XM_030247686,XM_030247687	Prkcd	protein kinase C, delta	protein_coding	[illegible]
18754	NM_011104,XM_006523833,XM_017317328,XM_030249576	Prkce	protein kinase C, epsilon	protein_coding	[illegible]
18755	NM_001313977,NM_008856	Prkch	protein kinase C, eta	protein_coding	[illegible]
18759	NM_008857,XM_006535411	Prkci	protein kinase C, iota	protein_coding	[illegible]
18760	NM_008858,XM_006515586,XM_006515587,XM_006515590,XM_006515591,XM_011244017,XR_001780427,XR_001780428,XR_001780429,XR_001780430,XR_001780431,XR_001780432,XR_00394	Prkd1	protein kinase D1	protein_coding	[illegible]
18761	NM_008859,XM_006497398,XM_006497399,XM_030248462,XM_030248463	Prkcq	protein kinase C, theta	protein_coding	[illegible]
18762	NM_001039079,NM_001355178,NM_008860,XM_006538656,XM_011250207,XM_017320042,XM_030253317	Prkcz	protein kinase C, zeta	protein_coding	[illegible]
18763	NM_013630,XM_006523834,XM_006523841,XM_006523842,XM_006523843,XM_006523844,XM_006523845,XM_006523846,XM_006523847,XM_006523852,XM_030249577,XM_030249578,XM_030249579,XM_030249580,XM_030249581,XM_030249582,XM_030249583,XM_030249584,XM_030249585,XM_030249586,XM_030249587,XM_030249588,XM_030249589	Pkd1	polycystin 1, transient receptor poteintial channel interacting	protein_coding	[illegible]
18764	NM_008861,XM_006534815,XR_001784633,XR_003955591	Pkd2	polycystin 2, transient receptor potential cation channel	protein_coding	[illegible]
18766	NM_011105	Pkdrej	polycystin (PKD) family receptor for egg jelly	protein_coding	[illegible]
18767	NM_008862,XM_006530056,XM_030252461	Pkia	protein kinase inhibitor, alpha	protein_coding	[illegible]
18768	NM_001039050,NM_001039051,NM_001039052,NM_001039053,NM_001190402,NM_008863,XM_006512602,XM_006512604,XM_006512605,XM_017313847	Pkib	protein kinase inhibitor beta, cAMP dependent, testis specific	protein_coding	[illegible]
18769	NM_001039390,NM_001039391,NM_001164053,NM_001164055,NM_001277096,NM_011106,XM_006498926,XM_006498927,XM_030248482,XM_030248483,XM_030248487,XR_003951364	Pkig	protein kinase inhibitor, gamma	protein_coding	[illegible]
18770	NM_001099779,NM_013631,XM_006501134,XM_006501135	Pklr	pyruvate kinase liver and red blood cell	protein_coding	[illegible]
18771	NM_016670,NR_027493,XM_006523854,XM_006523855,XM_006523856,XM_006523857,XM_006523858,XM_030249590,XM_030249	Pknox1	Pbx/knotted 1 homeobox	protein_coding	[illegible]
18772	NM_001313701,NM_019645	Pkp1	plakophilin 1	protein_coding	[illegible]
18777	NM_001355712,NM_008866	Lypla1	lysophospholipase 1	protein_coding	[illegible]
18779	NM_008867,XM_006498928	Pla2r1	phospholipase A2 receptor 1	protein_coding	[illegible]
18781	NM_008868	Pla2g2c	phospholipase A2, group IIC	protein_coding	[illegible]
18782	NM_001347230,NM_011109	Pla2g2d	phospholipase A2, group IID	protein_coding	[illegible]
18783	NM_001305632,NM_008869	Pla2g4a	phospholipase A2, group IVA (cytosolic, calcium-dependent)	protein_coding	[illegible]
18784	NM_001122954,NM_011110,XM_006538658,XM_017320043,XM_017320044,XM_030253319	Pla2g5	phospholipase A2, group V	protein_coding	[illegible]
18786	NM_172695	Plaa	phospholipase A2, activating protein	protein_coding	[illegible]
18787	NM_008871	Serpine1	serine (or cysteine) peptidase inhibitor, clade E, member 1	protein_coding	[illegible]
18788	NM_001174170,NM_011111,XM_011247942	Serpinb2	serine (or cysteine) peptidase inhibitor, clade B, member 2	protein_coding	[illegible]
18789	NM_001347440,NM_011112,XM_006515592,XM_006515593,XM_006515594,XM_006515595,XM_006515596,XM_017314992,XM_017314994,XM_030246611,XM_030246612,XM_030246613,XR_003949999	Papola	poly (A) polymerase alpha	protein_coding	[illegible]
18791	NM_008872	Plat	plasminogen activator, tissue	protein_coding	[illegible]
18792	NM_008873,XM_017315919	Plau	plasminogen activator, urokinase	protein_coding	[illegible]
18793	NM_011113,XM_006539639,XM_006539640	Plaur	plasminogen activator, urokinase receptor	protein_coding	[illegible]

18795	NM_001145830,NM_019677,XM_006498930	Plcb1	phospholipase C, beta 1	protein_coding	NP_001139302.1;NP_062651.2;XP_006498993.1	97613	0.870318 [illegible]
18796	NM_001290790,NM_177568,XM_006498935,XM_017316357,XM_017316359,XM_030248513,XR_003951369	Plcb2	phospholipase C, beta 2	protein_coding	NP_001277719.1;NP_808236.2;XP_006498998.1;XP_017171846.1;XP_017171848.1;XP_030104373.1	107465	0.534774 [illegible]
18797	NM_001290349,NM_008874,XM_006526766,XM_006526767	Plcb3	phospholipase C, beta 3	protein_coding	NP_001277278.1;NP_032900.2;XP_006526829.1;XP_006526830.1	104778	12.519737 [illegible]
18798	NM_001355153,NM_001355154,NM_001355155,NM_001355156,NM_013829,XM_006498936,XM_006498937,XM_006498938,XM_006498939,XM_006498941,XM_006498943,XM_006498945,XM_006498946,XM_006498947,XM_006498948,XM_006498950,XM_011239357,XM_011239358,XM_011239359,XM_030248529,XM_030248532,XM_030248537,XM_030248539,XM_030248556	Plcb4	phospholipase C, beta 4	protein_coding	NP_001342082.1;NP_001342083.1;NP_001342084.1;NP_001342085.1;NP_038857.1;XP_006498999.1;XP_006499000.1;XP_006499001.1;XP_006499002.1;XP_006499004.1;XP_006499006.1;XP_006499008.1;XP_006499009.1;XP_006499010.1;XP_006499011.1;XP_006499013.1;XP_011237659.1;XP_011237660.1;XP_011237661.1;XP_030104389.1;XP_030104392.1;XP_030104397.1;XP_030104399.1;XP_0301[illegible]	107464	6.711444 [illegible]
18799	NM_001293648,NM_019676,NR_121571,XM_006511984	Plcd1	phospholipase C, delta 1	protein_coding	NP_001280577.1;NP_062650.1;XP_006512047.1	97614	44.879794 [illegible]
18802	NM_001081456,NM_148937,XM_006495783,XM_006495784,XM_006495785,XM_006495786,XM_006495787,XM_006495788,XM_006495789,XM_006495790,XM_017319346,XM_030252282,XM_030252283,XM_030252284,XR_001783207,XR_001783208,XR_865222	Plcd4	phospholipase C, delta 4	protein_coding	NP_001074925.1;NP_683739.2;XP_006495846.1;XP_006495847.1;XP_006495848.1;XP_006495849.1;XP_006495850.1;XP_006495851.1;XP_006495852.1;XP_006495853.1;XP_017174835.1;XP_030108142.1;XP_030108143.1;XP_030108144.1	107469	0.377450 [illegible]
18803	NM_021280	Plcg1	phospholipase C, gamma 1	protein_coding	NP_067255.2	97615	20.240673 [illegible]
18805	NM_001164056,NM_001368667,NM_008875,XM_017319489,XM_017319490,XM_017319491	Pld1	phospholipase D1	protein_coding	NP_001157528.1;NP_001355596.1;NP_032901.2;XP_017174978.1;XP_017174979.1;XP_017174980.1	109585	4.525806 [illegible]
18806	NM_001302475,NM_001302476,NM_001361935,NM_008876,XM_011248794,XM_011248795,XM_011248796,XM_011248797,XM_030245659,XM_030245660,XR_001779917,XR_001779918,XR_00394	Pld2	phospholipase D2	protein_coding	NP_001289404.1;NP_001289405.1;NP_001348864.1;NP_032902.1;XP_011247096.1;XP_011247097.1;XP_011247098.1;XP_011247099.1;XP_030101519.1;XP_030101520.1	892877	8.266013 [illegible]
18807	NM_001317355,NM_011116,XM_006539642,XM_006539643,XM_006539645,XM_006539646,XM_011250456,XM_017322042,XM_030242219	Pld3	phospholipase D family, member 3	protein_coding	NP_001304284.1;NP_035246.1;XP_006539705.1;XP_006539706.1;XP_006539708.1;XP_006539709.1;XP_011248758.1;XP_017177531.1;XP_030098079.1	1333782	28.173364 [illegible]
18810	NM_001163540,NM_001163542,NM_001163549,NM_001164203,NM_011117,NM_201385,NM_201386,NM_201387,NM_201388,NM_201389,NM_201390,NM_201391,NM_201392,NM_201393,NM_201394	Plec	plectin	protein_coding	NP_001157012.1;NP_001157014.1;NP_001157021.1;NP_001157675.1;NP_035247.2;NP_958787.2;NP_958788.2;NP_958789.3;NP_958790.2;NP_958791.2;NP_958792.2;NP_958793.2;NP_958794.2;NP_958795.2;NP_958796.2	1277961	23.770922 [illegible]
18813	NM_011119	Pa2g4	proliferation-associated 2G4	protein_coding	NP_035249.1	894684	41.275440 [illegible]
18815	NM_008877	Plg	plasminogen	protein_coding	NP_032903.3	97620	0.000000 [illegible]
18816	NM_008878	Serpinf2	serine (or cysteine) peptidase inhibitor, clade F, member 2	protein_coding	NP_032904.1	107173	0.021400 [illegible]
18817	NM_011121	Plk1	polo like kinase 1	protein_coding	NP_035251.3	97621	9.042304 [illegible]
18818	gene-Plk-ps1	Plk-ps1	polo like kinase, pseudogene 1	pseudogene	.	103247	0.017612 [illegible]
18821	NM_001141927,NM_023129	Pln	phospholamban	protein_coding	NP_001135399.1;NP_075618.1	97622	0.038113 [illegible]
18822	NM_011122	Plod1	procollagen-lysine, 2-oxoglutarate 5-dioxygenase 1	protein_coding	NP_035252.1	99907	42.373360 [illegible]
18823	NM_001290561,NM_001359117,NM_001359118,NM_001359119,NM_001359120,NM_011123,XM_030251257	Plp1	proteolipid protein (myelin) 1	protein_coding	NP_001277490.1;NP_001346046.1;NP_001346047.1;NP_001346048.1;NP_001346049.1;NP_035253.1;XP_030107117.1	97623	1.619729 [illegible]
18824	NM_019755	Plp2	proteolipid protein 2	protein_coding	NP_062729.1	[illegible]	[illegible]
18826	NM_001247984,NM_008879,XM_006518699,XM_006518701,XM_006518703,XM_006518704,XM_017315920	Lcp1	lymphocyte cytosolic protein 1	protein_coding	NP_001234913.1;NP_032905.2;XP_006518762.1;XP_006518766.1;XP_006518767.1;XP_017171409.1	[illegible]	[illegible]
18828	NM_001195084,NM_008880,XM_006510857,XM_011242676	Plscr2	phospholipid scramblase 2	protein_coding	NP_001182013.1;NP_032906.2;XP_006510920.1;XP_[illegible]	[illegible]	[illegible]
18829	NM_011124	Ccl21a	chemokine (C-C motif) ligand 21A (serine)	protein_coding	NP_035254.1	[illegible]	[illegible]
18830	NM_011125	Pltp	phospholipid transfer protein	protein_coding	NP_035255.1	[illegible]	[illegible]
18844	NM_008881	Plxna1	plexin A1	protein_coding	NP_032907.1	[illegible]	[illegible]
18845	NM_008882,XM_006497240,XR_373891	Plxna2	plexin A2	protein_coding	NP_032908.2;XP_006497303.1	[illegible]	[illegible]
18846	NM_001358129,NM_008883,XM_006527902,XM_006527903,XM_030251258,XM_030251259,XM_030251260,XM_030251261	Plxna3	plexin A3	protein_coding	NP_001345058.1;NP_032909.2;XP_006527965.1;XP_030107118.1;XP_030107119.1;XP_030107120.1;[illegible]	[illegible]	[illegible]
18854	NM_001311088,NM_008884,NM_178087,XM_006510860,XM_006510861,XM_006510862,XM_006510864,XM_017313212,XM_030244128,XR_003947814,XR_003947816	Pml	promyelocytic leukemia	protein_coding	NP_001298017.1;NP_032910.3;NP_835188.2;[illegible]	[illegible]	[illegible]
18857	NM_001030305	Pmp2	peripheral myelin protein 2	protein_coding	NP_001025476.1	[illegible]	[illegible]
18858	NM_001302255,NM_001302257,NM_001302258,NM_001302259,NM_001302260,NM_001302261,NM_008885,XM_030245661,XM_030245662	Pmp22	peripheral myelin protein 22	protein_coding	NP_001289184.1;NP_001289186.1;NP_001289187.1;NP_001289189.1;NP_001289190.1;NP_032911.1;[illegible];XP_030101522.1	[illegible]	[illegible]
18861	NM_008886,XM_006504812,XM_017320710,XM_017320712,XM_030254201,XM_030254202,XM_030254203,XM_030254204,XR_001784634,XR_001784636,XR_001784637,XR_003955592,XR_003955593,XR_003955594,XR_003955595,XR_003955596,XR_377191	Pms2	PMS1 homolog2, mismatch repair system component	protein_coding	NP_032912.2;XP_006504875.1;XP_017176199.1;XP_030110061.1;XP_030110062.1;XP_030110063.1;[illegible]	[illegible]	[illegible]
18933	NM_001025570,NM_011127,NM_175686,XM_006496703	Prrx1	paired related homeobox 1	protein_coding	NP_001020741.1;NP_035257.1;NP_783617.2;[illegible]	[illegible]	[illegible]
18938	NM_008889	Ppp1r14b	protein phosphatase 1, regulatory inhibitor subunit 14B	protein_coding	NP_032915.2	[illegible]	[illegible]
18946	NM_018874	Pnliprp1	pancreatic lipase related protein 1	protein_coding	NP_061362.1	[illegible]	[illegible]
18947	NM_011128	Pnliprp2	pancreatic lipase-related protein 2	protein_coding	NP_035258.2	[illegible]	[illegible]
18948	NM_008890	Pnmt	phenylethanolamine-N-methyltransferase	protein_coding	NP_032916.1	[illegible]	[illegible]
18949	NM_008891	Pnn	pinin	protein_coding	NP_032917.2	[illegible]	[illegible]
18950	NM_013632	Pnp	purine-nucleoside phosphorylase	protein_coding	NP_038660.1	[illegible]	[illegible]
18951	NM_213614	Sept5	septin 5	protein_coding	NP_998779.2	[illegible]	[illegible]
18952	NM_001284392,NM_001284394,NM_001284398,NM_001361936,NM_001368775,NM_001368776,NM_001368777,NM_001368778,NM_001368779,NM_011129,XM_006532493,XM_006532497,XM_030245663,XM_030245664,XM_030245665,XM_030245666,XM_030245667,XR_001779919,XR_388364	Sept4	septin 4	protein_coding	NP_001271321.1;NP_001271323.1;NP_001271327.1;NP_001355704.1;NP_001355705.1;[illegible];NP_001355708.1;NP_035259.1;XP_006532556.1;[illegible];XP_030101523.1;XP_030101524.1;XP_030101525.[illegible];XP_030101527.1	[illegible]	[illegible]
18968	NM_008892,XM_006527905,XR_003953001,XR_878096	Pola1	polymerase (DNA directed), alpha 1	protein_coding	NP_032918.1;XP_006527968.1	[illegible]	[illegible]
18969	NM_001164057,NM_008893,XM_006531681,XM_006531683,XM_006531684,XM_011248607,XM_011248608,XM_011248609,XM_011248610,XM_030250774,XM_030250775,XM_030250776	Pola2	polymerase (DNA directed), alpha 2	protein_coding	NP_001157529.1;NP_032919.2;XP_006531744.1;XP_006531747.1;XP_011246909.1;XP_011246910.1;XP_011246912.1;XP_030106634.1;XP_030106635.1;[illegible]	[illegible]	[illegible]
18970	NM_011130,XM_006509030,XM_011242126,XM_030243354,XM_030243355,XR_378779	Polb	polymerase (DNA directed), beta	protein_coding	NP_035260.1;XP_006509093.1;XP_011240428.1;XP_030099215.1	[illegible]	[illegible]
18971	NM_011131,XM_030242220	Pold1	polymerase (DNA directed), delta 1, catalytic subunit	protein_coding	NP_035261.3;XP_030098080.1	[illegible]	[illegible]
18972	NM_001361942,NM_008894	Pold2	polymerase (DNA directed), delta 2, regulatory subunit	protein_coding	NP_001348871.1;NP_032920.2	[illegible]	[illegible]
18973	NM_011132,XM_017320715	Pole	polymerase (DNA directed), epsilon	protein_coding	NP_035262.2;XP_017176204.1	[illegible]	[illegible]
18974	NM_011133,XM_006515598,XM_006515599,XM_011244018	Pole2	polymerase (DNA directed), epsilon 2 (p59 subunit)	protein_coding	NP_035263.1;XP_006515661.1;XP_006515662.1;XP_[illegible]	[illegible]	[illegible]
18975	NM_001360095,NM_001360096,NM_017462,NR_153404,XM_006540708,XM_006540711,XM_030242221,XR_003946418,XR_391322,XR_391323	Polg	polymerase (DNA directed), gamma	protein_coding	NP_001347024.1;NP_001347025.1;NP_059490.2;XP_006540774.1;XP_030098081.1	[illegible]	[illegible]
18976	NM_001278581,NM_001278582,NM_001278583,NM_001278584,NM_008895	Pomc	pro-opiomelanocortin-alpha	protein_coding	NP_001265510.1;NP_001265511.1;NP_001265512.1;NP_032921.1	[illegible]	[illegible]
18979	NM_011134,XM_006505011,XM_011241049,XM_030255222	Pon1	paraoxonase 1	protein_coding	NP_035264.2;XP_006505074.1;XP_011239351.1;XP_[illegible]	[illegible]	[illegible]
18983	NM_001271542,NM_001271543,NM_001361938,NM_001361939,NM_011135,NR_155265,NR_155266,XM_006509306,XM_006509307,XR_378909	Cnot7	CCR4-NOT transcription complex, subunit 7	protein_coding	NP_001258471.1;NP_001258472.1;NP_001348867.1;NP_035265.1;XP_006509369.1;XP_006509370.1	[illegible]	[illegible]
18984	NM_008898,XM_006504398,XM_006504399	Por	P450 (cytochrome) oxidoreductase	protein_coding	NP_032924.1;XP_006504461.1;XP_006504462.1	[illegible]	[illegible]
18985	NM_011136	Pou2af1	POU domain, class 2, associating factor 1	protein_coding	NP_035266.1	[illegible]	[illegible]
18986	NM_001368808,NM_011137,NM_198932,NM_198933,NM_198934,XM_006496704,XM_011238767,XM_017319360,XM_030252286,XM_030252287,XR_373598	Pou2f1	POU domain, class 2, transcription factor 1	protein_coding	NP_001355737.1;NP_035267.2;NP_945150.2;[illegible]	[illegible]	[illegible]
18987	NM_001163554,NM_001163555,NM_001163556,NM_011138,XM_006539647,XM_006539648,XM_006539649,XM_006539650,XM_006539651,XM_006539652,XM_006539653,XM_006539654,XM_006539655,XM_006539656,XM_006539658,XM_006539659,XM_006539660,XM_006539661,XM_006539662,XM_011250457,XM_030242222,XM_030242223,XM_030242224,XM_030242225,XM_030242226,XM_030242227,XM_030242228,XM_030242229,XM_030242230,XM_030242232,XM_030242233,XM_030242234	Pou2f2	POU domain, class 2, transcription factor 2	protein_coding	[illegible]	[illegible]	[illegible]
18988	NM_011139,XM_011242420	Pou2f3	POU domain, class 2, transcription factor 3	protein_coding	NP_035269.2;XP_011240722.1	[illegible]	[illegible]
18991	NM_011141	Pou3f1	POU domain, class 3, transcription factor 1	protein_coding	NP_035271.1	[illegible]	[illegible]
18992	NM_008899	Pou3f2	POU domain, class 3, transcription factor 2	protein_coding	NP_032925.1	[illegible]	[illegible]
18993	NM_008900	Pou3f3	POU domain, class 3, transcription factor 3	protein_coding	NP_032926.2	[illegible]	[illegible]
18994	NM_008901	Pou3f4	POU domain, class 3, transcription factor 4	protein_coding	NP_032927.1	[illegible]	[illegible]
18996	NM_011143,XM_006518705,XM_006518706,XM_030247688,XR_383144	Pou4f1	POU domain, class 4, transcription factor 1	protein_coding	NP_035273.3;XP_006518768.1;XP_006518769.1;XP_[illegible]	[illegible]	[illegible]
18997	NM_138944	Pou4f2	POU domain, class 4, transcription factor 2	protein_coding	NP_620394.2	[illegible]	[illegible]
19009	NM_001304965,XM_006520608,XM_006520609,XM_006520614,XM_017316508,XM_017316509,XM_017316510,XM_030248416,XM_030248417,XM_030248418,XM_030248419	Pou6f1	POU domain, class 6, transcription factor 1	protein_coding	[illegible]	[illegible]	[illegible]
19011	NM_001168693,NM_008902,XM_006520617,XM_006520618,XM_017316511	Endou	endonuclease, polyU-specific	protein_coding	NP_001162164.1;NP_032928.1;XP_006520680.2;XP_017172000.1	[illegible]	[illegible]
19012	NM_008247,NM_008903	Plpp1	phospholipid phosphatase 1	protein_coding	NP_032273.1;NP_032929.1	[illegible]	[illegible]
19013	NM_001113418,NM_011144,XM_006520619,XM_006520620,XM_006520621,XM_006520622,XM_006520623,XM_011245516,XM_011245517,XM_011245519,XM_030248421,XM_030248422,XM_030248423,XM_030248424	Ppara	peroxisome proliferator activated receptor alpha	protein_coding	[illegible]	[illegible]	[illegible]
19014	NM_001080118,NM_001361950,NM_001361951,NM_013634,NM_134027,XM_006532504,XM_006532505,XR_001779920,XR_388366	Med1	mediator complex subunit 1	protein_coding	NP_001073587.1;NP_001348879.1;NP_001348880.1;NP_598788.2;XP_006532567.1;XP_006532568.1	[illegible]	[illegible]
19015	NM_011145	Ppard	peroxisome proliferator activator receptor delta	protein_coding	NP_035275.1	[illegible]	[illegible]
19016	NM_001127330,NM_001308352,NM_001308354,NM_011146,XM_006505737,XM_006505738,XM_006505739,XM_006505743,XM_011241252,XM_017321455,XM_017321456,XM_030255223,XR_001785108	Pparg	peroxisome proliferator activated receptor gamma	protein_coding	[illegible]	[illegible]	[illegible]
19017	NM_008904,NR_027710,NR_132764,XM_006503774,XM_006503775,XM_006503776,XM_006503777,XM_006503778,XM_006503779,XM_017320716,XM_017320717,XM_017320718,XM_030254205	Ppargc1a	peroxisome proliferative activated receptor, gamma, coactivator 1 alpha	protein_coding	[illegible]	[illegible]	[illegible]
19018	NM_020255	Scand1	SCAN domain-containing 1	protein_coding	NP_064651.2	[illegible]	[illegible]
19023	NM_011148,XM_030254206	Ppef2	protein phosphatase, EF hand calcium-binding domain 2	protein_coding	NP_035278.1;XP_030110066.1	[illegible]	[illegible]
19024	NM_001163557,NM_001357436,NM_001357437,NM_001357438,NM_008905,XM_006507443,XM_006507444,XM_006507446,XM_006507448,XM_006507449,XM_006507450,XM_006507451,XM_006507452,XM_006507453,XM_006507454,XM_006507455,XM_030242235	Ppfibp2	PTPRF interacting protein, binding protein 2 (liprin beta 2)	protein_coding	[illegible]	[illegible]	[illegible]
19025	NM_001038492,NM_008906,XM_030248567	Ctsa	cathepsin A	protein_coding	NP_001033581.1;NP_032932.2;XP_030104427.1	[illegible]	[illegible]
19027	NM_013635,NM_198710,XM_011243842,XM_011243843,XR_3811	Sypl	synaptophysin-like protein	protein_coding	NP_038663.2;NP_942003.1;XP_011242144.1;[illegible]	[illegible]	[illegible]
19034	gene-Gm10123	Gm10123	predicted pseudogene 10123	pseudogene	.	[illegible]	[illegible]
19035	NM_011149	Ppib	peptidylprolyl isomerase B	protein_coding	NP_035279.2	[illegible]	[illegible]
19038	NM_008908	Ppic	peptidylprolyl isomerase C	protein_coding	NP_032934.1	[illegible]	[illegible]
19039	NM_011150	Lgals3bp	lectin, galactoside-binding, soluble, 3 binding protein	protein_coding	NP_035280.1	[illegible]	[illegible]
19041	NM_008909	Ppl	periplakin	protein_coding	NP_032935.2	[illegible]	[illegible]
19042	NM_008910,XM_006515603	Ppm1a	protein phosphatase 1A, magnesium dependent, alpha isoform	protein_coding	NP_032936.1;XP_006515666.1	[illegible]	[illegible]
19043	NM_001159496,NM_001159497,NM_001159498,NM_011151,XM_006523860,XM_006523861,XM_006523862,XM_011246321,XM_011246322,XM_030249592,XM_030249593,XM_030249594	Ppm1b	protein phosphatase 1B, magnesium dependent, beta isoform	protein_coding	[illegible]	[illegible]	[illegible]
19044	NM_008911,XM_006496707,XM_006496709,XM_011238772,XM_011238773,XM_030252290,XM_030252291,XM_030252292	Ppox	protoporphyrinogen oxidase	protein_coding	[illegible]	[illegible]	[illegible]
19045	NM_031868	Ppp1ca	protein phosphatase 1 catalytic subunit alpha	protein_coding	NP_114074.1	[illegible]	[illegible]
19046	NM_172707,XM_006503780	Ppp1cb	protein phosphatase 1 catalytic subunit beta	protein_coding	NP_766295.2;XP_006503843.1	[illegible]	[illegible]
19047	NM_013636,NR_156471,NR_156472,XM_006530200	Ppp1cc	protein phosphatase 1 catalytic subunit gamma	protein_coding	NP_038664.2;XP_006530263.1	[illegible]	[illegible]
19049	NM_001313970,NM_144828	Ppp1r1b	protein phosphatase 1, regulatory inhibitor subunit 1B	protein_coding	NP_001300899.1;NP_659077.1	[illegible]	[illegible]
19052	NM_019411	Ppp2ca	protein phosphatase 2 (formerly 2A), catalytic subunit, alpha isoform	protein_coding	NP_062284.1	[illegible]	[illegible]
19053	NM_017374,XM_006509033	Ppp2cb	protein phosphatase 2 (formerly 2A), catalytic subunit, beta isoform	protein_coding	NP_059070.1;XP_006509096.1	[illegible]	[illegible]
19054	NM_001163415,NM_011154,NR_028110,NR_102716	Ppp2r3d	protein phosphatase 2 (formerly 2A), regulatory subunit B'', delta	protein_coding	NP_001156887.1;NP_035284.2	[illegible]	[illegible]
19055	NM_001293622,NM_008913,XM_017319492,XM_017319493,XM_030252462,XM_030252463,XM_030252464	Ppp3ca	protein phosphatase 3, catalytic subunit, alpha isoform	protein_coding	[illegible]	[illegible]	[illegible]
19056	NM_001310426,NM_001310427,NM_008914,XM_006518707,XM_006518708,XM_006518711,XM_017315921,XM_030247689	Ppp3cb	protein phosphatase 3, catalytic subunit, beta isoform	protein_coding	[illegible]	[illegible]	[illegible]
19057	NM_001304991,NM_001304992,NM_001360229,NM_008915,XM_006518714,XM_017315922,XM_017315924,XM_030247690,XM_030247691,XR_003950828,XR_003950829	Ppp3cc	protein phosphatase 3, catalytic subunit, gamma isoform	protein_coding	[illegible]	[illegible]	[illegible]
19058	NM_024459	Ppp3r1	protein phosphatase 3, regulatory subunit B, alpha isoform (calcineurin B, type I)	protein_coding	NP_077779.2	[illegible]	[illegible]
19059	NM_001004025	Ppp3r2	protein phosphatase 3, regulatory subunit B, alpha isoform (calcineurin B, type II)	protein_coding	NP_001004025.1	[illegible]	[illegible]
19060	NM_011155,XM_006539663,XM_017322043,XM_030242236	Ppp5c	protein phosphatase 5, catalytic subunit	protein_coding	NP_035285.2;XP_006539726.1;XP_017177532.1;[illegible]	[illegible]	[illegible]
19062	NM_008916,XM_006532507,XR_388367	Inpp5k	inositol polyphosphate 5-phosphatase K	protein_coding	NP_032942.1;XP_006532570.1	[illegible]	[illegible]
19063	NM_008917,XR_376299	Ppt1	palmitoyl-protein thioesterase 1	protein_coding	NP_032943.2	[illegible]	[illegible]
19065	NM_008919	Npy4r	neuropeptide Y receptor Y4	protein_coding	NP_032945.3	[illegible]	[illegible]
19069	NM_001083331,NM_001276406,NM_172394,NR_075087	Nup88	nucleoporin 88	protein_coding	NP_001076800.1;NP_001263335.1;NP_765982.2	[illegible]	[illegible]
19070	NM_001310585,NM_025283	Mob4	MOB family member 4, phocein	protein_coding	NP_001297514.1;NP_079559.2	[illegible]	[illegible]
19072	NM_011156,XM_011243142	Prep	prolyl endopeptidase	protein_coding	NP_035286.1;XP_011241444.1	[illegible]	[illegible]
19073	NM_001358965,NM_011157,XM_006513374,XM_006513376,XM_006513377,XM_030244953,XM_030244954	Srgn	serglycin	protein_coding	NP_001345894.1;NP_035287.1;XP_006513437.1;XP_006513440.1;XP_030100813.1;XP_030100814.1	[illegible]	[illegible]
19074	NM_008920	Prg2	proteoglycan 2, bone marrow	protein_coding	NP_032946.1	[illegible]	[illegible]
19075	NM_008921	Prim1	DNA primase, p49 subunit	protein_coding	NP_032947.1	[illegible]	[illegible]
19076	NM_008922,XM_006495793,XM_006495797,XM_011238455,XM_030252295,XR_373246,XR_373247	Prim2	DNA primase, p58 subunit	protein_coding	NP_032948.1;XP_006495856.1;XP_006495860.1;XP_030108155.1	[illegible]	[illegible]
19079	NM_031869,XM_006530201,XR_003955597	Prkab1	protein kinase, AMP-activated, beta 1 non-catalytic subunit	protein_coding	NP_114075.1;XP_006530264.1	[illegible]	[illegible]
19082	NM_016781,XM_011245520	Prkag1	protein kinase, AMP-activated, gamma 1 non-catalytic subunit	protein_coding	NP_058061.2;XP_011243822.1	[illegible]	[illegible]
19084	NM_001313973,NM_001313974,NM_001313975,NM_001313976,NM_001362677,NM_021880,XM_006532509	Prkar1a	protein kinase, cAMP dependent regulatory, type I, alpha	protein_coding	NP_001300902.1;NP_001300903.1;NP_001300904.1;NP_001349606.1;NP_068680.1;XP_006532572.1	[illegible]	[illegible]
19085	NM_001253890,NM_001359097,NM_001359098,NM_001359099,NM_001359100,NM_001359101,NM_008923,XM_006504660	Prkar1b	protein kinase, cAMP dependent regulatory, type I beta	protein_coding	[illegible]	[illegible]	[illegible]
19087	NM_008924	Prkar2a	protein kinase, cAMP dependent regulatory, type II alpha	protein_coding	NP_032950.1	[illegible]	[illegible]
19088	NM_001364407,NM_001364408,NM_011158,XM_030246614	Prkar2b	protein kinase, cAMP dependent regulatory, type II beta	protein_coding	NP_001351336.1;NP_001351337.1;NP_035288.2;[illegible]	[illegible]	[illegible]
19089	NM_001293650,NM_001293651,NM_008925,XM_006510095,XM_006510096	Prkcsh	protein kinase C substrate 80K-H	protein_coding	NP_001280579.1;NP_001280580.1;NP_032951.1;XP_006510159.1	[illegible]	[illegible]
19090	NM_011159,XM_006521861,XM_011245841,XM_017316892,XR_003951769	Prkdc	protein kinase, DNA activated, catalytic polypeptide	protein_coding	NP_035289.2;XP_006521924.1;XP_011244143.1;[illegible]	[illegible]	[illegible]
19091	NM_001013833,NM_011160,XM_006526768	Prkg1	protein kinase, cGMP-dependent, type I	protein_coding	NP_001013855.1;NP_035290.1;XP_006526831.1	[illegible]	[illegible]
19092	NM_008926,XM_006534820,XM_006534821	Prkg2	protein kinase, cGMP-dependent, type II	protein_coding	NP_032952.3;XP_006534883.1;XP_006534884.1	[illegible]	[illegible]
19094	NM_011161	Mapk11	mitogen-activated protein kinase 11	protein_coding	NP_035291.4	[illegible]	[illegible]
19099	NM_001202445,NM_001202446,NM_011162,XM_006498953,XM_006498954	Mapk8ip1	mitogen-activated protein kinase 8 interacting protein 1	protein_coding	NP_001189374.1;NP_001189375.1;NP_035292.2;XP_006499017.1	[illegible]	[illegible]
19106	NM_011163,XM_006523863	Eif2ak2	eukaryotic translation initiation factor 2-alpha kinase 2	protein_coding	NP_035293.1;XP_006523926.1	[illegible]	[illegible]
19108	NM_016979,XM_006527909,XM_006527910	Prkx	protein kinase, X-linked	protein_coding	NP_058675.1;XP_006527972.1;XP_006527973.1	[illegible]	[illegible]
19116	NM_001253781,NM_001253782,NM_011169,XM_006520034,XM_006520035,XM_006520036,XM_006520037,XM_006520038,XM_030248425,XR_383824	Prlr	prolactin receptor	protein_coding	[illegible]	[illegible]	[illegible]
19122	NM_001278256,NM_011170	Prnp	prion protein	protein_coding	NP_001265185.1;NP_035300.1	[illegible]	[illegible]
19123	NM_001042767,NM_001042768,NM_001313938,NM_008934,XM_006525721,XM_006525722,XM_011246857,XM_030250362	Proc	protein C	protein_coding	[illegible]	[illegible]	[illegible]
19124	NM_011171,XM_006498955	Procr	protein C receptor, endothelial	protein_coding	NP_035301.2;XP_006499018.1	[illegible]	[illegible]
19125	NM_011172,XM_006521863,XR_001781795,XR_001781796	Prodh	proline dehydrogenase	protein_coding	NP_035302.2;XP_006521926.1	[illegible]	[illegible]
19126	NM_001163577,NM_001163578,NM_001163581,NM_001163582,NM_001163583,NM_001163584,NM_001163585,NM_008935,XM_006503786,XM_006503788,XM_006503789,XM_006503790,XM_006503791,XM_017320720,XM_017320722,XM_017320723,XM_017320724,XM_017320725,XM_030254207,XR_001784640,XR_001784642,XR_001784643	Prom1	prominin 1	protein_coding	[illegible]	[illegible]	[illegible]
19128	NM_011173,XM_011245843	Pros1	protein S (alpha)	protein_coding	NP_035303.1;XP_011244145.1	[illegible]	[illegible]
19130	NM_001360827,NM_008937,XM_006497124,XM_006497125,XM_006497126,XM_006497127	Prox1	prospero homeobox 1	protein_coding	NP_001347756.1;NP_032963.1;XP_006497187.1;XP_006497189.1;XP_006497190.1	[illegible]	[illegible]
19132	NM_001163588,NM_001163589,NM_013639,XM_006520631,XM_006520632,XM_030248426	Prph	peripherin	protein_coding	NP_001157060.1;NP_001157061.1;NP_038667.2;XP_006520695.1;XP_030104286.1	[illegible]	[illegible]
19133	NM_008938	Prph2	peripherin 2	protein_coding	NP_032964.1	[illegible]	[illegible]
19134	NM_013830,XM_006516582,XM_006516583,XM_006516584,XM_017315425,XM_017315426,XM_017315428,XM_017315429,XM_017315430,XM_017315431,XM_017315432,XM_017315433,XM_017315434,XM_017315435,XM_017315436,XM_017315437,XM_017315439,XM_017315440,XM_017315441,XM_030247191,XM_030247192,XM_030247193,XM_030247194,XM_030247195,XM_030247196,XM_030247197,XM_030247198,XM_030247199,XM_030247200,XM_030247201	Prpf4b	pre-mRNA processing factor 4B	protein_coding	[illegible]	[illegible]	[illegible]
19139	NM_021463	Prps1	phosphoribosyl pyrophosphate synthetase 1	protein_coding	NP_067438.1	[illegible]	[illegible]
19141	NM_011175,XM_017314995	Lgmn	legumain	protein_coding	NP_035305.1;XP_017170484.1	[illegible]	[illegible]
19142	NM_008939	Prss12	protease, serine 12 neurotrypsin (motopsin)	protein_coding	NP_032965.1	[illegible]	[illegible]
19143	NM_011176,XM_006510098,XM_006510099	St14	suppression of tumorigenicity 14 (colon carcinoma)	protein_coding	NP_035306.2;XP_006510161.1;XP_006510162.1	[illegible]	[illegible]
19152	NM_011178	Prtn3	proteinase 3	protein_coding	NP_035308.2	[illegible]	[illegible]
19153	NM_019412,NM_198048,XM_006539666	Prx	periaxin	protein_coding	NP_062285.1;NP_932165.2;XP_006539729.1	[illegible]	[illegible]
19155	NM_008942,XM_011248802,XM_030245668,XM_030245669,XM_030245670,XR_879535	Npepps	aminopeptidase puromycin sensitive	protein_coding	NP_032968.2;XP_011247104.1;XP_030101528.1;XP_030101530.1	[illegible]	[illegible]
19156	NM_001146120,NM_001146121,NM_001146122,NM_001146123,NM_001146124,NM_011179,XM_017313848,XM_017313849	Psap	prosaposin	protein_coding	[illegible]	[illegible]	[illegible]
19157	NM_001112699,NM_001112700,NM_011180,XM_006532519,XM_011248803,XM_011248805,XM_030245671,XM_030245672,XM_030245673,XR_001779921,XR_003949350,XR_003949351	Cyth1	cytohesin 1	protein_coding	[illegible]	[illegible]	[illegible]
19158	NM_001112701,NM_011181,XR_003946419	Cyth2	cytohesin 2	protein_coding	NP_001106171.1;NP_035311.1	[illegible]	[illegible]
19159	NM_001163548,NM_011182,XM_017320729,XM_030254209	Cyth3	cytohesin 3	protein_coding	NP_001157020.1;NP_035312.3;XP_017176218.1;[illegible]	[illegible]	[illegible]
19164	NM_001362271	Psen1	presenilin 1	protein_coding	NP_001349200.1	[illegible]	[illegible]
19165	NM_001128605,NM_011183,XM_006496712,XM_011238776,XR_373599	Psen2	presenilin 2	protein_coding	NP_001122077.1;NP_035313.2;XP_006496775.1;[illegible]	[illegible]	[illegible]
19166	NM_008944	Psma2	proteasome (prosome, macropain) subunit, alpha type 2	protein_coding	NP_032970.2	[illegible]	[illegible]
19167	NM_001310595,NM_001310596,NM_011184,XM_017314996,XM_030246616,XM_030246617	Psma3	proteasome (prosome, macropain) subunit, alpha type 3	protein_coding	NP_001297524.1;NP_001297525.1;NP_035314.3;XP_030102476.1;XP_030102477.1	[illegible]	[illegible]
19170	NM_011185	Psmb1	proteasome (prosome, macropain) subunit, beta type 1	protein_coding	NP_035315.1	[illegible]	[illegible]
19171	NM_013640	Psmb10	proteasome (prosome, macropain) subunit, beta type 10	protein_coding	NP_038668.2	[illegible]	[illegible]
19172	NM_008945	Psmb4	proteasome (prosome, macropain) subunit, beta type 4	protein_coding	NP_032971.2	[illegible]	[illegible]

19173	NM_011186	Psmb5	proteasome (prosome, macropain) subunit, beta type 5	protein_coding	NP_035316.1	1194513	.	42.247265
19175	NM_008946,XM_030245674	Psmb6	proteasome (prosome, macropain) subunit, beta type 6	protein_coding	NP_032972.3;XP_030101534.1	104880	.	73.551263
19177	NM_011187,XM_006497785	Psmb7	proteasome (prosome, macropain) subunit, beta type 7	protein_coding	NP_035317.1;XP_006497848.1	107637	.	62.745809
19179	NM_008947	Psmc1	protease (prosome, macropain) 26S subunit, ATPase 1	protein_coding	NP_032973.1	106054	.	53.345329
19181	NM_001368661,NM_011188	Psmc2	proteasome (prosome, macropain) 26S subunit, ATPase 2	protein_coding	NP_001355590.1;NP_035318.2	109555	.	46.071767
19182	NM_008948	Psmc3	proteasome (prosome, macropain) 26S subunit, ATPase 3	protein_coding	NP_032974.2	1098754	.	65.416245
19183	NM_008949,XM_006532524,XM_030245675	Psmc3ip	proteasome (prosome, macropain) 26S subunit, ATPase 3, interacting	protein_coding	NP_032975.1;XP_006532587.1;XP_030101535.1	1098610	.	4.551860
19184	NM_008950	Psmc5	protease (prosome, macropain) 26S subunit, ATPase 5	protein_coding	NP_032976.1	105047	.	52.129520
19185	NM_001282017,NM_008951,XM_006501137,XM_030252465	Psmd4	proteasome (prosome, macropain) 26S subunit, non-ATPase, 4	protein_coding	NP_001268946.1;NP_032977.1;XP_006501200.1;XP_030108325.1	1201670	.	48.287893
19186	NM_011189	Psme1	proteasome (prosome, macropain) activator subunit 1 (PA28 alpha)	protein_coding	NP_035319.1	1096367	.	29.923136
19188	NM_001029855,NM_011190	Psme2	proteasome (prosome, macropain) activator subunit 2 (PA28 beta)	protein_coding	NP_001025026.1;NP_035320.1	1096365	.	31.299241
19192	NM_011192,XM_030245676	Psme3	proteaseome (prosome, macropain) activator subunit 3 (PA28 gamma, Ki)	protein_coding	NP_035322.1;XP_030101536.1	1096366	.	24.722067
19193	NM_008952,XM_011248807	Pipox	pipecolic acid oxidase	protein_coding	NP_032978.2;XP_011247109.1	1197006	.	0.173726
19197	NM_008954	Pspn	persephin	protein_coding	NP_032980.2	1201684	.	0.000000
19200	NM_011193,XM_006510865,XM_006510866,XM_006510867,XM_006510868,XM_011242677	Pstpip1	proline-serine-threonine phosphatase-interacting protein 1	protein_coding	NP_035323.2;XP_006510928.1;XP_006510929.1;XP_006510930.1;XP_006510931.1;XP_011240979.1	1321396	.	9.298413
19201	NM_013831	Pstpip2	proline-serine-threonine phosphatase-interacting protein 2	protein_coding	NP_038859.3	1335088	.	2.941586
19204	NM_001081211	Ptafr	platelet-activating factor receptor	protein_coding	NP_001074680.1	106066	.	0.892773
19205	NM_001077363,NM_001283013,NM_008956,XR_003948622,XR_003948623	Ptbp1	polypyrimidine tract binding protein 1	protein_coding	NP_001070831.1;NP_001269942.1;NP_032982.2	97791	.	106.129485
19206	NM_001328514,NM_008957	Ptch1	patched 1	protein_coding	NP_001315443.1;NP_032983.1	105373	.	15.757271
19207	NM_001312903,NM_008958	Ptch2	patched 2	protein_coding	NP_001299832.1;NP_032984.1	1095405	.	0.658119
19208	NM_011195	Ptcra	pre T cell antigen receptor alpha	protein_coding	NP_035325.1	104857	.	0.000000
19210	NM_008959	Ptdss1	phosphatidylserine synthase 1	protein_coding	NP_032985.2	1276575	.	15.812209
19211	NM_008960,XM_006526769	Pten	phosphatase and tensin homolog	protein_coding	NP_032986.1;XP_006526832.1	109583	.	11.933364
19212	NM_001305436,NM_001305437,NM_008961,XM_006497401,XM_006497402,XM_017316438	Pter	phosphotriesterase related	protein_coding	NP_001292365.1;NP_001292366.1;NP_032987.1;XP_006497464.1;XP_006497465.1;XP_017171927.1	107372	.	3.470546
19214	NM_008962	Ptgdr	prostaglandin D receptor	protein_coding	NP_032988.3	102966	.	1.128563
19215	NM_008963,XM_006497786,XM_006497787	Ptgds	prostaglandin D2 synthase (brain)	protein_coding	NP_032989.2;XP_006497849.1;XP_006497850.1	99261	.	6.297849
19216	NM_013641,XM_006530771	Ptger1	prostaglandin E receptor 1 (subtype EP1)	protein_coding	NP_038669.1;XP_006530834.1	97793	.	1.002292
19217	NM_008964,XM_006518720	Ptger2	prostaglandin E receptor 2 (subtype EP2)	protein_coding	NP_032990.1;XP_006518783.1	97794	.	0.137066
19218	NM_001359745,NM_011196,XM_006501140,XM_011240038	Ptger3	prostaglandin E receptor 3 (subtype EP3)	protein_coding	NP_001346674.1;NP_035326.2;XP_006501203.1;XP_011238340.1	97795	.	0.613793
19219	NM_001136079,NM_008965	Ptger4	prostaglandin E receptor 4 (subtype EP4)	protein_coding	NP_001129551.1;NP_032991.1	104311	.	0.415790
19220	NM_008966	Ptgfr	prostaglandin F receptor	protein_coding	NP_032992.1	97796	.	1.948132
19221	NM_011197,XM_006501147,XM_017319503	Ptgfrn	prostaglandin F2 receptor negative regulator	protein_coding	NP_035327.2;XP_006501210.1;XP_017174992.1	1277114	.	35.423740
19222	NM_008967,XM_006539670	Ptgir	prostaglandin I receptor (IP)	protein_coding	NP_032993.2;XP_006539733.1	99535	.	0.535355
19223	NM_008968,XM_030248627	Ptgis	prostaglandin I2 (prostacyclin) synthase	protein_coding	NP_032994.1;XP_030104487.1	1097156	.	86.232193
19224	NM_008969,XM_006497790,XM_006497791,XM_006497794,XM_011239036,XM_017316496,XM_030248641	Ptgs1	prostaglandin-endoperoxide synthase 1	protein_coding	NP_032995.1;XP_006497853.1;XP_006497854.1;XP_006497857.1;XP_011237338.1;XP_017171985.1;XP_030104501.1	97797	.	4.815572
19225	NM_011198	Ptgs2	prostaglandin-endoperoxide synthase 2	protein_coding	NP_035328.2	97798	.	1.828700
19227	NM_008970	Pthlh	parathyroid hormone-like peptide	protein_coding	NP_032996.2	97800	.	2.526262
19228	NM_001083935,NM_001083936,NM_011199,XM_006511985,XM_006511987,XM_006511988,XM_011242942,XM_017313214,XM_017313215	Pth1r	parathyroid hormone 1 receptor	protein_coding	NP_001077404.1;NP_001077405.1;NP_035329.2;XP_006512048.1;XP_006512050.1;XP_006512051.1;XP_011241244.1;XP_017168703.1;XP_017168704.1	97801	.	87.463643
19229	NM_001162365,NM_001162366,NM_001360233,NM_172498,XM_017315926,XM_017315927,XM_017315928,XM_017315929,XM_017315930	Ptk2b	PTK2 protein tyrosine kinase 2 beta	protein_coding	NP_001155837.1;NP_001155838.1;NP_001347162.1;NP_766086.2;XP_017171415.1;XP_017171416.1;XP_017171417.1;XP_017171418.1;XP_017171419.1	104908	.	1.710480
19230	NM_008971,XM_006520638	Twf1	twinfilin actin binding protein 1	protein_coding	NP_032997.3;XP_006520701.1	1100520	.	25.850069
19231	NM_001360830,NM_001360831,NM_008972,NR_153833,NR_1538	Ptma	prothymosin alpha	protein_coding	NP_001347759.1;NP_001347760.1;NP_032998.1	97803	.	490.182136
19240	NM_001039392,NM_001190327,NM_025284	Tmsb10	thymosin, beta 10	protein_coding	NP_001034481.1;NP_001177256.1;NP_079560.1	109146	.	214.402874
19241	NM_021278,XM_006528759	Tmsb4x	thymosin, beta 4, X chromosome	protein_coding	NP_067253.1;XP_006528822.1	99510	.	445.160563
19242	NM_008973,XM_006505757	Ptn	pleiotrophin	protein_coding	NP_032999.1;XP_006505820.3	97804	.	388.097459
19243	NM_011200,XM_006495798,XM_006495799	Ptp4a1	protein tyrosine phosphatase 4a1	protein_coding	NP_035330.1;XP_006495861.1;XP_006495862.1	1277096	.	13.799785
19244	NM_001164745,NM_008974,XM_006502863,XM_017320045,XM_030253321,XM_030253322	Ptp4a2	protein tyrosine phosphatase 4a2	protein_coding	NP_001158217.1;NP_033000.1;XP_006502926.1;XP_017175534.1;XP_030109181.1;XP_030109182.1	1277117	.	70.543245
19245	NM_001166388,NM_001166389,NM_001166390,NM_001357591,NM_008975,XM_006520642,XM_011245523,XM_030248427,XM_030248428	Ptp4a3	protein tyrosine phosphatase 4a3	protein_coding	NP_001159860.1;NP_001159861.1;NP_001159862.1;NP_001344520.1;NP_033001.2;XP_006520705.1;XP_011243825.1;XP_030104287.1;XP_030104288.1	1277098	.	11.537531
19246	NM_011201,XM_006498981	Ptpn1	protein tyrosine phosphatase, non-receptor type 1	protein_coding	NP_035331.3;XP_006499044.1	97805	.	10.997112
19247	NM_001109992,NM_011202	Ptpn11	protein tyrosine phosphatase, non-receptor type 11	protein_coding	NP_001103462.1;NP_035332.1	99511	.	24.801126
19248	NM_001356590,NM_001356591,NM_001356592,NM_011203,XM_030254216,XM_030254217	Ptpn12	protein tyrosine phosphatase, non-receptor type 12	protein_coding	NP_001343519.1;NP_001343520.1;NP_001343521.1;NP_035333.2;XP_030110076.1;XP_030110077.1	104673	.	18.512172
19249	NM_011204	Ptpn13	protein tyrosine phosphatase, non-receptor type 13	protein_coding	NP_035334.2	103293	.	12.675673
19250	NM_008976,XM_006497129,XM_006497131,XM_006497133,XM_006497134,XM_030252297	Ptpn14	protein tyrosine phosphatase, non-receptor type 14	protein_coding	NP_033002.2;XP_006497192.1;XP_006497194.1;XP_006497196.1;XP_006497197.1;XP_030108157.1	102467	.	10.892604
19252	NM_013642	Dusp1	dual specificity phosphatase 1	protein_coding	NP_038670.1	105120	.	10.166030
19253	NM_011206,XM_006495800,XM_017319374	Ptpn18	protein tyrosine phosphatase, non-receptor type 18	protein_coding	NP_035336.2;XP_006495863.1;XP_017174863.1	108410	.	2.092919
19255	NM_001127177,NM_008977,XM_011246858,XM_011246859,XM_030250364	Ptpn2	protein tyrosine phosphatase, non-receptor type 2	protein_coding	NP_001120649.1;NP_033003.1;XP_011245160.1;XP_011245161.1;XP_030106224.1	97806	.	11.178901
19256	NM_008978,XM_006518728,XM_006518730,XM_011244996,XM_030247694,XM_030247695,XR_383149	Ptpn20	protein tyrosine phosphatase, non-receptor type 20	protein_coding	NP_033004.1;XP_006518791.1;XP_006518793.1;XP_011243298.1;XP_030103554.1;XP_030103555.1	1196295	.	0.036216
19258	NM_019933,XM_006529253,XM_006529254,XM_006529255,XM_006529256,XM_030252299,XM_030252300,XM_030252301,XM_030252302,XM_030252303	Ptpn4	protein tyrosine phosphatase, non-receptor type 4	protein_coding	NP_064317.2;XP_006529316.1;XP_006529317.1;XP_006529318.1;XP_006529319.1;XP_030108159.1;XP_030108160.1;XP_030108161.1;XP_030108162.1;XP_030108163.1	1099792	.	4.278213
19259	NM_001163565,NM_013643,XM_006540713,XM_006540714,XM_006540715,XM_017322046,XM_030242238,XM_030242239	Ptpn5	protein tyrosine phosphatase, non-receptor type 5	protein_coding	NP_001157037.1;NP_038671.2;XP_006540776.1;XP_006540777.1;XP_006540778.1;XP_017177535.1;XP_030098098.1;XP_030098099.1	97807	.	0.018474
19260	NM_008979,NR_104070,XM_006501148,XM_006501149,XM_006501150,XM_011240041,XM_011240042,XM_017319504	Ptpn22	protein tyrosine phosphatase, non-receptor type 22 (lymphoid)	protein_coding	NP_033005.1;XP_006501211.1;XP_006501212.1;XP_006501213.1;XP_011238343.1;XP_011238344.1;XP_017174993.1	107170	.	2.127310
19261	NM_001177647,NM_001291019,NM_001291020,NM_001291021,NM_001291022,NM_001355158,NM_001355160,NM_007547,XM_006498986,XM_017316564,XM_017316568,XM_017316569,XM_030248735,XM_030248738,XM_030248740	Sirpa	signal-regulatory protein alpha	protein_coding	NP_001171118.1;NP_001277948.1;NP_001277949.1;NP_001277950.1;NP_001277951.1;NP_001342087.1;NP_001342089.1;NP_031573.2;XP_006499049.1;XP_017172053.1;XP_017172057.1;XP_017172058.1;XP_030104595.1;XP_030104598.1;XP_030104600.1	108563	.	22.956364
19262	NM_001163688,NM_001355161,NM_008980,XM_006498990	Ptpra	protein tyrosine phosphatase, receptor type, A	protein_coding	NP_001157160.1;NP_001342090.1;NP_033006.2;XP_006499053.1	97808	.	28.398562
19263	NM_029928,XM_006513378	Ptprb	protein tyrosine phosphatase, receptor type, B	protein_coding	NP_084204.2;XP_006513441.1	97809	.	6.201108
19264	NM_001111316,NM_001268286,NM_011210,XM_006529257,XM_006529258,XM_006529259,XM_006529260,XM_006529261,XM_006529262	Ptprc	protein tyrosine phosphatase, receptor type, C	protein_coding	NP_001104786.2;NP_001255215.1;NP_035340.3;XP_006529320.1;XP_006529321.1;XP_006529322.1;XP_006529323.1;XP_006529324.1;XP_006529325.1	97810	.	2.262693
19265	NM_016933	Ptprcap	protein tyrosine phosphatase, receptor type, C polypeptide-associated protein	protein_coding	NP_058629.2	97811	.	0.102023
19266	NM_001352628,NM_001352629,NM_001352630,NM_001368991,NM_001368992,NM_001368993,NM_011211,NR_160919,XM_011249962,XM_011249963,XM_011249964,XM_011249965,XM_011249966,XM_017320046,XM_017320064,XM_030253324,XM_030253325,XM_030253326,XM_030253327,XM_030253328,XM_030253329,XM_030253330,XM_030253331,XM_030253332,XM_030253333,XM_030253334,XM_030253335,XM_030253336,XM_030253337,XM_030253338,XM_030253339,XM_030253340,XM_030253341,XM_030253342,XM_030253343,XM_030253344,XM_030253345,XM_030253346,XM_030253347,XM_030253348,XM_030253349,XM_030253350,XM_030253351,XM_030253352,XM_030253353,XM_030253354,XM_030253355,XM_030253356,XM_030253357,XM_030253358,XM_030253359,XR_003954893	Ptprd	protein tyrosine phosphatase, receptor type, D	protein_coding	NP_001339557.1;NP_001339558.1;NP_001339559.1;NP_001355920.1;NP_001355921.1;NP_001355922.1;NP_035341.2;XP_011248264.1;XP_011248265.1;XP_011248266.1;XP_011248267.1;XP_011248268.1;XP_017175535.1;XP_017175553.1;XP_030109184.1;XP_030109185.1;XP_030109186.1;XP_030109187.1;XP_030109188.1;XP_030109189.1;XP_030109190.1;XP_030109191.1;XP_030109192.1;XP_030109193.1;XP_030109194.1;XP_030109195.1;XP_030109196.1;XP_030109197.1;XP_030109198.1;XP_030109199.1;XP_030109200.1;XP_030109201.1;XP_030109202.1;XP_030109203.1;XP_030109204.1;XP_030109205.1;XP_030109206.1;XP_030109207.1;XP_030109208.1;XP_030109209.1;XP_030109210.1;XP_030109211.1;XP_030109212.1;XP_030109213.1;XP_030109214.1;XP_030109215.1;XP_030109216.1;XP_030109217.1;XP_030109218.1;XP_030109219.1	97812	.	46.760433
19267	NM_001316678,NM_001316679,NM_001316680,NM_001316681,NM_011212,XR_001785494	Ptpre	protein tyrosine phosphatase, receptor type, E	protein_coding	NP_001303607.1;NP_001303608.1;NP_001303609.1;NP_001303610.1;NP_035342.3	97813	.	2.629708
19268	NM_011213,XM_006502864,XM_006502865,XM_006502866,XM_006502867,XM_006502868,XM_006502869,XM_006502870,XM_006502871,XM_006502872,XM_006502874,XM_006502875,XM_006502876,XM_030253360,XM_030253361,XM_030253362,XM_030253363	Ptprf	protein tyrosine phosphatase, receptor type, F	protein_coding	NP_035343.2;XP_006502927.1;XP_006502928.1;XP_006502929.1;XP_006502930.1;XP_006502931.1;XP_006502932.1;XP_006502933.1;XP_006502934.1;XP_006502935.1;XP_006502937.1;XP_006502938.1;XP_006502939.1;XP_030109220.1;XP_030109221.1;XP_030109222.1;XP_030109223.1	102695	.	15.902584
19270	NM_001347593,NM_008981,XM_006517956	Ptprg	protein tyrosine phosphatase, receptor type, G	protein_coding	NP_001334522.1;NP_033007.2;XP_006518019.1	97814	.	7.759395
19271	NM_001135657,NM_008982,XM_006498991,XM_006498992,XM_006498993,XM_006498994	Ptprj	protein tyrosine phosphatase, receptor type, J	protein_coding	NP_001129129.1;NP_033008.3;XP_006499054.1;XP_006499055.1;XP_006499056.1;XP_006499057.1	104574	.	4.679626
19272	NM_001359355,NM_001359356,NM_008983,XM_006512606,XM_006512607,XM_006512608,XM_006512610,XM_006512611,XM_006512612,XM_006512614,XM_006512615,XM_006512617,XM_006512618,XM_006512619,XM_006512620,XM_011243143,XM_011243144,XM_011243145,XM_011243146,XM_017313850,XM_030244960	Ptprk	protein tyrosine phosphatase, receptor type, K	protein_coding	NP_001346284.1;NP_001346285.1;NP_033009.1;XP_006512669.1;XP_006512670.1;XP_006512671.1;XP_006512673.1;XP_006512674.1;XP_006512675.1;XP_006512677.1;XP_006512678.1;XP_006512680.1;XP_006512681.1;XP_006512682.1;XP_006512683.1;XP_011241445.1;XP_011241446.1;XP_011241447.1;XP_011241448.1;XP_017169339.1;XP_030100820.1	103310	.	10.220140
19273	NM_001083119,NM_011214,XM_006538666,XM_006538667,XM_006538668,XM_006538670,XM_006538672,XM_006538673,XM_006538674,XM_011250210,XM_011250211,XM_011250212,XM_011250213,XM_011250214,XM_011250215,XM_011250216	Ptpru	protein tyrosine phosphatase, receptor type, U	protein_coding	NP_001076588.1;NP_035344.2;XP_006538729.1;XP_006538730.1;XP_006538733.1;XP_006538735.1;XP_006538736.1;XP_006538737.1;XP_011248512.1;XP_011248513.1;XP_011248514.1;XP_011248515.1;XP_011248516.1;XP_011248517.1;XP_011248518[illegible]	1321151	.	8.478766
19274	NM_001357625,NM_008984	Ptprm	protein tyrosine phosphatase, receptor type, M	protein_coding	NP_001344554.1;NP_033010.2	102694	.	9.481125
19275	NM_001359281,NM_008985,XM_006496444,XM_006496445,XM_011238677	Ptprn	protein tyrosine phosphatase, receptor type, N	protein_coding	NP_001346210.1;NP_033011.2;XP_006496507.1;XP_006496508.1;XP_011236979.1	102765	.	0.114253
19276	NM_011215,XM_011244019	Ptprn2	protein tyrosine phosphatase, receptor type, N polypeptide 2	protein_coding	NP_035345.2;XP_011242321.1	107418	.	0.170062
19277	NM_001164401,NM_001164402,NM_001164403,NM_011216,XM_030255238	Ptpro	protein tyrosine phosphatase, receptor type, O	protein_coding	NP_001157873.1;NP_001157874.1;NP_001157875.1;NP_035346.3;XP_030111098.1	1097152	.	0.626105
19279	NM_001161837,NM_001161838,NM_001161839,NM_001161840,NM_001358484,NM_011217,XM_006513382	Ptprr	protein tyrosine phosphatase, receptor type, R	protein_coding	NP_001155309.1;NP_001155310.1;NP_001155311.1;NP_001155312.1;NP_001345413.1;NP_035347.1;XP_006513445.1	109559	.	0.699334
19280	NM_001252453,NM_001252455,NM_001252456,NM_011218,XM_006523874,XM_006523875,XM_006523876,XM_006523877,XM_006523878,XM_006523879,XM_006523880,XM_006523881,XM_006523883,XM_006523884,XM_006523886,XM_017317349,XM_017317350,XM_030249595,XM_030249596,XR_003952097,XR_385302	Ptprs	protein tyrosine phosphatase, receptor type, S	protein_coding	NP_001239382.1;NP_001239384.1;NP_001239385.1;NP_035348.2;XP_006523937.1;XP_006523938.1;XP_006523939.1;XP_006523940.1;XP_006523941.1;XP_006523942.1;XP_006523943.1;XP_006523944.1;XP_006523946.1;XP_006523947.1;XP_006523949.1;XP_017172838.1;XP_017172839.1;XP_030105455.1;XP_030105456.1	97815	.	76.950002
19281	NM_001291149,NM_001291150,NM_001291151,NM_021464,XM_011239384,XM_011239385,XM_017316586,XM_017316587,XM_017316590,XM_017316592,XM_017316593,XM_017316594,XM_017316595,XM_017316597,XM_017316598,XM_017316600,XM_030248790	Ptprt	protein tyrosine phosphatase, receptor type, T	protein_coding	NP_001278078.1;NP_001278079.1;NP_001278080.1;NP_067439.1;XP_011237686.1;XP_011237687.1;XP_017172075.1;XP_017172076.1;XP_017172079.1;XP_017172081.1;XP_017172082.1;XP_017172083.1;XP_017172084.1;XP_017172086.1;XP_017172087.1;XP_017172089.1;XP_030104650.1	1321152	.	0.094930
19283	NM_001081306,NM_001311064,NM_001361349,NM_011219,XM_006505012,XM_006505013,XM_006505014,XM_006505015,XM_006505017	Ptprz1	protein tyrosine phosphatase, receptor type Z, polypeptide 1	protein_coding	NP_001074775.1;NP_001297993.1;NP_001348278.1;NP_035349.2;XP_006505075.1;XP_006505076.1;XP_006505077.1;XP_006505078.1;XP_006505080.1	97816	.	20.005065
19285	NM_001359105,NM_008986	Cavin1	caveolae associated 1	protein_coding	NP_001346034.1;NP_033012.1	1277968	.	112.955646
19286	NM_001364488,NM_001364489,NM_001364490,NM_011220,XM_030244130,XM_030244131,XM_030244132,XM_030244133,XM_030244134,XM_030244135,XM_030244136,XM_030244137,XM_030244138,XM_030244139,XM_030244140	Pts	6-pyruvoyl-tetrahydropterin synthase	protein_coding	NP_001351417.1;NP_001351418.1;NP_001351419.1;NP_035350.1;XP_030099990.1;XP_030099991.1;XP_030099992.1;XP_030099993.1;XP_030099994.1;XP_030099995.1;XP_030099996.1;XP_030099997.1;XP_030099998.1;XP_030099999.1;XP_030100000.1	1338783	.	14.730681
19288	NM_008987	Ptx3	pentraxin related gene	protein_coding	NP_033013.3	104641	.	15.305584
19289	NM_001357257,NM_008988,XM_011242679	Igdcc3	immunoglobulin superfamily, DCC subclass, member 3	protein_coding	NP_001344186.1;NP_033014.1;XP_011240981.1	1202390	.	0.184906
19290	NM_008989,XM_006525724,XM_011246860	Pura	purine rich element binding protein A	protein_coding	NP_033015.1;XP_006525787.1;XP_011245162.1	103079	.	4.994973
19291	NM_011221	Purb	purine rich element binding protein B	protein_coding	NP_035351.1	1338779	.	15.600435
19293	NM_001330686,NM_013645	Pvalb	parvalbumin	protein_coding	NP_001317615.1;NP_038673.2	97821	.	1.144097
19294	NM_001159724,NM_008990	Nectin2	nectin cell adhesion molecule 2	protein_coding	NP_001153196.1;NP_033016.3	97822	.	8.871309
19296	NR_003368,NR_132746,NR_132747	Pvt1	Pvt1 oncogene	lncRNA	.	97824	.	2.192599
19298	NM_001159525,NM_001356513,NM_001356514,NM_023041	Pex19	peroxisomal biogenesis factor 19	protein_coding	NP_001152997.1;NP_001343442.1;NP_001343443.1;NP_075528.3	1334458	.	18.537469
19299	NM_001355756,NM_008991	Abcd3	ATP-binding cassette, sub-family D (ALD), member 3	protein_coding	NP_001342685.1;NP_033017.2	1349216	.	21.014849
19300	NM_008992,XM_006515613,XM_006515614,XM_006515616,XM_006515617,XM_030246618,XM_030246619,XR_001780435,XR_001780438,XR_003950000,XR_003950001,XR_003950002	Abcd4	ATP-binding cassette, sub-family D (ALD), member 4	protein_coding	NP_033018.2;XP_006515676.1;XP_006515677.1;XP_006515679.1;XP_006515680.1;XP_030102478.1;XP_030102479.1	1349217	.	5.126274
19301	NM_008993,XM_011249418	Pxmp2	peroxisomal membrane protein 2	protein_coding	NP_033019.2;XP_011247720.1	107487	.	0.986968
19302	NM_001163301,NM_001163302,NM_001163305,NM_001163306,NM_001267714,NM_001267715,NM_008994,XM_006530058,XM_006530059,XM_011248140,XM_017319505,XM_030252469,XM_030252470,XM_030252471,XM_030252472,XM_030252473,XM_030252474,XM_030252475	Pex2	peroxisomal biogenesis factor 2	protein_coding	NP_001156773.1;NP_001156774.1;NP_001156777.1;NP_001156778.1;NP_001254643.1;NP_001254644.1;NP_033020.2;XP_006530121.1;XP_006530122.1;XP_011246442.1;XP_017174994.1;XP_030108329.1;XP_030108330.1;XP_030108331.1;XP_030108332.1;XP_030108333.1;XP_030108334.1;XP_030108335.1	107486	.	13.973455
19303	NM_011223,NM_133915,XM_006530202,XM_006530204,XM_006530205,XM_006530207,XM_030254218	Pxn	paxillin	protein_coding	NP_035353.1;NP_598676.2;XP_006530265.1;XP_006530267.1;XP_006530268.1;XP_006530270.1;XP_030110078.1	108295	.	20.548878
19305	NM_001277330,NM_001277805,NM_001360570,NM_008995,NM_175933,XM_006505758,XM_006505759	Pex5	peroxisomal biogenesis factor 5	protein_coding	NP_001264259.1;NP_001264734.1;NP_001347499.1;NP_033021.2;NP_787947.1;XP_006505821.1;XP_006505822.1	1098808	.	15.996264
19309	NM_011224	Pygm	muscle glycogen phosphorylase	protein_coding	NP_035354.1	97830	.	17.260859
19317	NM_001159516,NM_001159517,NM_021881,XM_006523300,XM_030249598,XR_385167,XR_385168,XR_385169,XR_385170	Qk	quaking	protein_coding	NP_001152988.1;NP_001152989.1;NP_068681.1;XP_006523363.1;XP_030105458.1	97837	.	33.367536
19324	NM_008996	Rab1a	RAB1A, member RAS oncogene family	protein_coding	NP_033022.1	97842	.	75.697365
19325	NM_016676	Rab10	RAB10, member RAS oncogene family	protein_coding	NP_057885.1	105066	.	27.511587
19326	NM_008997,XM_006523887,XM_030249599	Rab11b	RAB11B, member RAS oncogene family	protein_coding	NP_033023.1;XP_006523950.1;XP_030105459.1	99425	.	21.967936
19327	gene-Rab11b-ps1	Rab11b-ps1	RAB11B, member RAS oncogene family, pseudogene 1	pseudogene	.	99426	.	0.888024
19328	NM_024448,XM_006523889,XR_876464	Rab12	RAB12, member RAS oncogene family	protein_coding	NP_077768.2;XP_006523952.1	894284	.	28.937775
19329	NM_001159725,NM_008998,XM_006529263	Rab17	RAB17, member RAS oncogene family	protein_coding	NP_001153197.2;NP_033024.1;XP_006529326.1	104640	.	0.482451
19330	NM_001278447,NM_181070	Rab18	RAB18, member RAS oncogene family	protein_coding	NP_001265376.1;NP_851415.1	102790	.	30.413535
19331	NM_011226,XM_006505760	Rab19	RAB19, member RAS oncogene family	protein_coding	NP_035356.1;XP_006505823.1	103292	.	0.000000
19332	NM_011227,XM_011242042	Rab20	RAB20, member RAS oncogene family	protein_coding	NP_035357.1;XP_011240344.1	102789	.	1.081261
19334	NM_024436,XM_017316603	Rab22a	RAB22A, member RAS oncogene family	protein_coding	NP_077756.2;XP_017172092.1	105072	.	13.760661
19335	NM_001159729,NM_008999,XM_006495801,XM_006495802	Rab23	RAB23, member RAS oncogene family	protein_coding	NP_001153201.1;NP_033025.2;XP_006495864.1;XP_006495865.1	99833	.	9.178139
19336	NM_009000,XM_006517165	Rab24	RAB24, member RAS oncogene family	protein_coding	NP_033026.1;XP_006517228.1	105065	.	14.862099
19337	NM_011228,XM_030251262,XM_030251263	Rab33a	RAB33A, member RAS oncogene family	protein_coding	NP_035358.1;XP_030107122.1;XP_030107123.1	109493	.	0.304980
19338	NM_016858	Rab33b	RAB33B, member RAS oncogene family	protein_coding	NP_058554.1	1330805	.	9.265797
19339	NM_001166399,NM_001328047,NM_009001,XM_006509603,XM_030243356	Rab3a	RAB3A, member RAS oncogene family	protein_coding	NP_001159871.1;NP_001314976.1;NP_033027.1;XP_006509666.1;XP_030099216.1	97843	.	4.625025
19340	NM_001324531,NM_031874,XM_011242422,XM_011242423	Rab3d	RAB3D, member RAS oncogene family	protein_coding	NP_001311460.1;NP_114080.2;XP_011240724.1;XP_011240725.1	97844	.	4.817502
19341	NM_009003,XM_006530773	Rab4a	RAB4A, member RAS oncogene family	protein_coding	NP_033029.2;XP_006530836.1	105069	.	5.958458
19342	NM_029391	Rab4b	RAB4B, member RAS oncogene family	protein_coding	NP_083667.1	105071	.	29.331318
19344	NM_177411,XM_006513384	Rab5b	RAB5B, member RAS oncogene family	protein_coding	NP_803130.1;XP_006513447.1	105938	.	28.257757
19345	NM_001305003,NM_024456	Rab5c	RAB5C, member RAS oncogene family	protein_coding	NP_001291932.1;NP_077776.2	105306	.	50.933929
19346	NM_001163663,NM_024287,XR_003946421	Rab6a	RAB6A, member RAS oncogene family	protein_coding	NP_001157135.1;NP_077249.1	894313	.	39.508795
19347	NM_001311139,NM_021494	Dennd5a	DENN/MADD domain containing 5A	protein_coding	NP_001298068.1;NP_067469.1	1201681	.	32.124203
19348	NM_001166406,NM_001166407,NM_009004	Kif20a	kinesin family member 20A	protein_coding	NP_001159878.1;NP_001159879.1;NP_033030.1	1201682	.	8.951956
19349	NM_001293652,NM_001293653,NM_001293654,NM_001293655,NM_009005	Rab7	RAB7, member RAS oncogene family	protein_coding	NP_001280581.1;NP_001280582.1;NP_001280583.1;NP_001280584.1;NP_033031.2	105068	.	87.123659
19350	gene-Rab7-ps1	Rab7-ps1	RAB7, member RAS oncogene family, pseudogene 1	pseudogene	.	1276565	.	2.324003
19352	NM_001163478,NM_001163479,NM_011231,NR_028094,NR_028095,NR_028096,NR_028097,NR_028098	Rabggtb	Rab geranylgeranyl transferase, b subunit	protein_coding	NP_001156950.1;NP_001156951.1;NP_035361.2	99537	.	34.622115
19353	NM_001347530,NM_009007	Rac1	Rac family small GTPase 1	protein_coding	NP_001334459.1;NP_033033.1	97845	.	97.450323
19354	NM_009008	Rac2	Rac family small GTPase 2	protein_coding	NP_033034.1	97846	.	5.897106
19355	NM_001289447,NM_001289448,NM_011232	Rad1	RAD1 checkpoint DNA exonuclease	protein_coding	NP_001276376.1;NP_001276377.1;NP_035362.2	1316678	.	8.533272
19356	NM_001044371,NM_001283011,NM_011233	Rad17	RAD17 checkpoint clamp loader component	protein_coding	NP_001037836.1;NP_001269940.1;NP_035363.2	1333807	.	7.654302
19357	NM_009009,XM_006520644,XM_006520645	Rad21	RAD21 cohesin complex component	protein_coding	NP_033035.3;XP_006520707.1;XP_006520708.1	108016	.	47.355711
19358	NM_001297606,NM_001297607,NM_009010,XM_011248327,XM_011248328,XM_030243357,XM_030243358	Rad23a	RAD23 homolog A, nucleotide excision repair protein	protein_coding	NP_001284535.1;NP_001284536.1;NP_033036.2;XP_011246629.1;XP_011246630.1;XP_030099217.1;XP_030099218.1	105126	.	16.208976
19359	NM_009011	Rad23b	RAD23 homolog B, nucleotide excision repair protein	protein_coding	NP_033037.2	105128	.	35.041412
19360	NM_009012	Rad50	RAD50 double strand break repair protein	protein_coding	NP_033038.2	109292	.	5.961026
19361	NM_011234,NR_156017	Rad51	RAD51 recombinase	protein_coding	NP_035364.1	97890	.	5.299825
19362	NM_001347455,NM_009013,XM_030255239,XM_030255240,XR_377433	Rad51ap1	RAD51 associated protein 1	protein_coding	NP_001334384.1;NP_033039.2;XP_030111099.1;XP_030111100.1	1098224	.	4.806958
19363	NM_001252562,NM_009014,XM_006515622,XM_006515623,XM_006515624,XM_006515625,XM_011244039,XM_017315003,XM_030246621,XR_003950003,XR_003950004,XR_003950005,XR_003950006,XR_003950007,XR_003950008,XR_003950009,XR_003950010,XR_003950011,XR_003950012,XR_003950013,XR_003950014,XR_003950015,XR_003950016,XR_003950017	Rad51b	RAD51 paralog B	protein_coding	NP_001239491.1;NP_033040.2;XP_006515685.1;XP_006515686.1;XP_006515687.1;XP_006515688.1;XP_011242341.1;XP_017170492.1;XP_030102481.1	1099436	.	0.939943
19364	NM_001277938,NM_001277939,NM_001277941,NM_001277942,NM_011235,NR_102717,NR_102718,NR_102719,NR_102720	Rad51d	RAD51 paralog D	protein_coding	NP_001264867.1;NP_001264868.1;NP_001264870.1;NP_001264871.1;NP_035365.1	1261809	.	2.166506
19365	NM_001166381,NM_001166382,NM_001166383,NM_011236,NR_149727,XM_006505764,XM_006505765,XM_006505766,XM_006505767,XM_011241256,XR_001785110,XR_001785111,XR_00395617	Rad52	RAD52 homolog, DNA repair protein	protein_coding	NP_001159853.1;NP_001159854.1;NP_001159855.1;NP_035366.2;XP_006505827.1;XP_006505828.1;XP_006505829.1;XP_006505830.1;XP_011239558.1	101949	.	6.367094
19366	NM_001122958,NM_001122959,NM_009015,XM_006502877,XM_006502878,XM_006502879,XM_030253364,XM_030253365,XM_030253366,XR_867829	Rad54l	RAD54 like (S. cerevisiae)	protein_coding	NP_001116430.1;NP_001116431.1;NP_033041.3;XP_006502940.1;XP_006502941.1;XP_006502942.1;XP_030109224.1;XP_030109225.1;XP_030109226.1	894697	.	2.370999
19367	NM_011237,XR_879420	Rad9a	RAD9 checkpoint clamp component A	protein_coding	NP_035367.1	1328356	.	7.492370
19373	NM_009019	Rag1	recombination activating 1	protein_coding	NP_033045.2	97848	.	0.013927
19374	NM_009020,XM_017316606	Rag2	recombination activating gene 2	protein_coding	NP_033046.1;XP_017172095.1	97849	.	0.041450
19376	NM_001159482,NM_033475	Rab34	RAB34, member RAS oncogene family	protein_coding	NP_001152954.1;NP_258436.2	104606	.	50.946685
19377	NM_001037764,NM_009021,XR_003949361,XR_879537,XR_879538,XR_879539	Rai1	retinoic acid induced 1	protein_coding	NP_001032853.1;NP_033047.2	103291	.	14.772503
19378	NM_009022	Aldh1a2	aldehyde dehydrogenase family 1, subfamily A2	protein_coding	NP_033048.2	107928	.	4.470149
19383	NM_001139511,NM_001139512,NM_001139513,NM_023130,XM_006499013,XM_006499014,XM_006499015,XM_006499016,XM_017316631,XM_030248812	Raly	hnRNP-associated with lethal yellow	protein_coding	NP_001132983.1;NP_001132984.1;NP_001132985.1;NP_075619.2;XP_006499076.1;XP_006499077.1;XP_006499078.1;XP_006499079.1;XP_017172120.1;XP_030104672.1	97850	.	43.294303
19384	NM_009391,XM_006504280	Ran	RAN, member RAS oncogene family	protein_coding	NP_033417.1;XP_006504343.1	1333112	.	63.747533
19385	NM_011239	Ranbp1	RAN binding protein 1	protein_coding	NP_035369.2	96269	.	66.409882
19386	NM_011240	Ranbp2	RAN binding protein 2	protein_coding	NP_035370.2	894323	.	16.601377
19387	NM_001146174,NM_001358622,NM_011241,NR_152441,XM_011245524,XM_030248429,XR_383994	Rangap1	RAN GTPase activating protein 1	protein_coding	NP_001139646.1;NP_001345551.1;NP_035371.4;XP_011243826.1;XP_030104289.1	103071	.	15.088696
19395	NM_001365062,NM_001365063,NM_011242,NR_157554,XM_006531685,XM_006531686,XM_006531687,XM_006531688,XM_006531689,XM_006531690,XM_006531691,XM_006531692,XM_011248613,XM_011248615,XM_017318082,XM_030250780,XM_030250781,XM_030250782,XM_030250783,XM_030250784,XM_030250785,XM_030250786,XM_030250787,XM_030250788,XM_030250789,XM_030250790,XM_030250791,XM_030250792,XR_003952758,XR_0	Rasgrp2	RAS, guanyl releasing protein 2	protein_coding	NP_001351991.1;NP_001351992.1;NP_035372.2;XP_006531748.1;XP_006531749.1;XP_006531750.1;XP_006531751.1;XP_006531752.1;XP_006531753.1;XP_006531754.1;XP_006531755.1;XP_011246915.1;XP_011246917.1;XP_017173571.1;XP_030106640.1;XP_030106641.1;XP_030106642.1;XP_030106643.1;XP_030106644.1;XP_030106645.1;XP_030106646.1;XP_030106647.1;XP_030106648.1;XP_030106649.1;XP_030106650.1;XP_030106651.1;XP_030106652.1	1333849	.	3.575009
19400	NM_009023,XM_006499017	Rapsn	receptor-associated protein of the synapse	protein_coding	NP_033049.2;XP_006499080.1	99422	.	0.731797

19401	NM_001176528,NM_001177302,NM_001177303,NM_001361954,NM_009024,XM_006532592,XM_006532593,XM_006532597,XM_030245700,XM_030245701,XM_030245702	Rara	retinoic acid receptor, alpha	protein_coding
19411	NM_001042727,NM_011244,XM_006520650,XM_011245525	Rarg	retinoic acid receptor, gamma	protein_coding
19414	NM_009025,XM_006508734	Rasa3	RAS p21 protein activator 3	protein_coding
19415	NM_001281999,NM_001359144,NM_001359145,NM_001359146,NM_001359147,NM_013832,XM_006530208,XM_006530210,XM_030254219,XM_030254220,XM_030254221,XR_003955599,XR_003955600,XR_003955601	Rasal1	RAS protein activator like 1 (GAP1 like)	protein_coding
19416	NM_009026	Rasd1	RAS, dexamethasone-induced 1	protein_coding
19417	NM_001039655,NM_001357743,NM_001357744,NM_011245,XR_001778822	Rasgrf1	RAS protein-specific guanine nucleotide-releasing factor 1	protein_coding
19418	NM_009027	Rasgrf2	RAS protein-specific guanine nucleotide-releasing factor 2	protein_coding
19419	NM_011246	Rasgrp1	RAS guanyl releasing protein 1	protein_coding
19428	NM_009028	Rasl2-9	RAS-like, family 2, locus 9	protein_coding
19434	NM_013833	Rax	retina and anterior neural fold homeobox	protein_coding
19645	NM_009029,XR_383150	Rb1	RB transcriptional corepressor 1	protein_coding
19646	NM_009030,XR_001784111	Rbbp4	retinoblastoma binding protein 4, chromatin remodeling factor	protein_coding
19647	NM_011247,NM_175023,XM_006507470,XM_030242242	Rbbp6	retinoblastoma binding protein 6, ubiquitin ligase	protein_coding
19649	NM_001164767,XM_006510107,XM_011242424,XM_011242425,XM_011242426,XM_011242427,XM_030244141	Robo3	roundabout guidance receptor 3	protein_coding
19650	NM_001139516,NM_011249,XM_006499018	Rbl1	RB transcriptional corepressor like 1	protein_coding
19651	NM_001282000,NM_001282001,NM_011250	Rbl2	RB transcriptional corepressor like 2	protein_coding
19652	NM_001166409,NM_001166410,NM_001166411,NM_001293658,NM_016809	Rbm3	RNA binding motif (RNP1, RRM) protein 3	protein_coding
19653	NM_001290122,NM_001290123,NM_001290124,NM_001290125,NM_009032	Rbm4	RNA binding motif protein 4	protein_coding
19654	NM_011251,NM_029169,XM_006511650,XM_006511651,XM_006511653,XM_011242860,XM_017313216,XM_030244142,XM_030244143,XM_030244144,XM_030244145	Rbm6	RNA binding motif protein 6	protein_coding
19655	NM_001166623,NM_011252,NR_029425,XR_003953002,XR_878097,XR_878098	Rbmx	RNA binding motif protein, X chromosome	protein_coding
19656	NM_001252089,NM_001358974,NM_001358975,NM_009033	Rbmxl1	RNA binding motif protein, X-linked like-1	protein_coding
19659	NM_011254	Rbp1	retinol binding protein 1, cellular	protein_coding
19660	NM_009034	Rbp2	retinol binding protein 2, cellular	protein_coding
19661	NM_015745	Rbp3	retinol binding protein 3, interstitial	protein_coding
19662	NM_001159487,NM_011255	Rbp4	retinol binding protein 4, plasma	protein_coding
19663	NM_001042674,NM_001042675,NM_001286168,NM_019733,XM_006509034,XM_006509035,XM_006509037,XM_017312620,XM_017312621,XM_030243359,XM_030243360,XR_001778413,XR_003947246,XR_378780,XR_378781,XR_378782,XR_378783,XR_870205	Rbpms	RNA binding protein gene with multiple splicing	protein_coding
19664	NM_001080927,NM_001080928,NM_001277116,NM_001359152,NM_009035,XM_006503796,XM_006503799,XM_017320742,XM_030254222	Rbpj	recombination signal binding protein for immunoglobulin kappa J region	protein_coding
19667	gene-Rbpj-ps3	Rbpj-ps3	recombination signal binding protein for immunoglobulin kappa J region, pseudogene 3	pseudogene
19668	NM_009036,XM_030248825	Rbpjl	recombination signal binding protein for immunoglobulin kappa J region-	protein_coding
19671	NM_001368712,NM_001368713,NM_001368714,NM_001368715,NM_023131,NR_160877,NR_160878,NR_160879,NR_160880,NR_160881,NR_160882,NR_160883,NR_160884,NR_160885,XM_030250	Rce1	Ras converting CAAX endopeptidase 1	protein_coding
19672	NM_009037	Rcn1	reticulocalbin 1	protein_coding
19674	NM_009038	Rcvrn	recoverin	protein_coding
19679	NM_001289472,NM_011256,XM_006530214,XM_006530215,XM_006530216,XM_006530217,XM_006530218,XM_030254223,XM_030254224,XM_030254225,XM_030254226,XM_030254227	Pitpnm2	phosphatidylinositol transfer protein, membrane-associated 2	protein_coding
19682	NM_001358527,NM_134006,XM_006513385,XM_006513386,XR_003948629	Rdh5	retinol dehydrogenase 5	protein_coding
19683	NM_009040,XM_006513389	Rdh16	retinol dehydrogenase 16	protein_coding
19684	NM_001104616,NM_001104617,NM_009041	Rdx	radixin	protein_coding
19687	NM_001347357,NM_001347358,NM_011258,XM_006503801	Rfc1	replication factor C (activator 1) 1	protein_coding
19691	NM_001204906,NM_001204907,NM_001355511,NM_023042,XM_011241573,XM_017321468,XM_030255271	Recql	RecQ protein-like	protein_coding
19695	NM_011260	Reg3g	regenerating islet-derived 3 gamma	protein_coding
19696	NM_009044	Rel	reticuloendotheliosis oncogene	protein_coding
19697	NM_001365067,NM_009045,XM_006531694,XM_006531695	Rela	v-rel reticuloendotheliosis viral oncogene homolog A (avian)	protein_coding
19698	NM_001290457,NM_009046,XM_006539675,XM_011250459,XM_011250460,XM_011250461,XM_030242243	Relb	avian reticuloendotheliosis viral (v-rel) oncogene related B	protein_coding
19699	NM_001310464,NM_011261	Reln	reelin	protein_coding
19700	NM_009047	Rem1	rad and gem related GTP binding protein 1	protein_coding
19701	NM_031192	Ren1	renin 1 structural	protein_coding
19703	NM_001164704,NM_023132	Renbp	renin binding protein	protein_coding
19704	NM_001122829,NM_030680,XM_006509604,XM_006509605	Upf1	UPF1 regulator of nonsense transcripts homolog (yeast)	protein_coding
19707	NM_001111065,NM_009048,XM_006512621,XM_017313852,XM_017313853,XM_030244961	Reps1	RalBP1 associated Eps domain containing protein	protein_coding
19708	NM_001291078,NM_011262	Dpf2	D4, zinc and double PHD fingers family 2	protein_coding
19711	NM_009049,NR_138251	Resp18	regulated endocrine-specific protein 18	protein_coding
19712	NM_011263,XM_006534842	Rest	RE1-silencing transcription factor	protein_coding
19713	NM_001080780,NM_009050	Ret	ret proto-oncogene	protein_coding
19714	NM_011264,XM_006512622,XM_006512623,XM_006512624,XM_017313854,XR_380057	Rev3l	REV3 like, DNA directed polymerase zeta catalytic subunit	protein_coding
19716	NM_009052	Bex1	brain expressed X-linked 1	protein_coding
19718	NM_020022	Rfc2	replication factor C (activator 1) 2	protein_coding
19719	NM_009053,XM_006532606,XR_001779932,XR_003949362	Rfng	RFNG O-fucosylpeptide 3-beta-N-acetylglucosaminyltransferase	protein_coding
19720	NM_009054,XM_006516606,XR_003950436	Trim27	tripartite motif-containing 27	protein_coding
19724	NM_009055,XM_006530778,XM_006530779,XM_006530780,XM_011248329,XM_030243361,XM_030243362,XM_030243363,XR_003947247	Rfx1	regulatory factor X, 1 (influences HLA class II expression)	protein_coding
19725	NM_009056,NM_027787	Rfx2	regulatory factor X, 2 (influences HLA class II expression)	protein_coding
19726	NM_001166414,NM_001360357,NM_001360358,NM_011265,XM_006526778,XM_006526784,XM_006526785,XM_011247167,XM_011247169,XM_017318087,XM_030250795,XM_030250796,XR_003952760,XR_003952761,XR_877668	Rfx3	regulatory factor X, 3 (influences HLA class II expression)	protein_coding
19727	NM_001025589,NM_011266	Rfxank	regulatory factor X-associated ankyrin-containing protein	protein_coding
19729	NM_009057,XM_006501155,XM_006501156,XM_030252477	Slc50a1	solute carrier family 50 (sugar transporter), member 1	protein_coding
19730	NM_001145834,NM_001145835,NM_001145836,NM_009058,XM_006497796,XM_006497797,XM_006497798,XM_006497799,XM_030248854,XM_030248855,XM_030248857,XM_030248861	Ralgds	ral guanine nucleotide dissociation stimulator	protein_coding
19731	NM_001346117,NM_001346118,NM_001346119,NM_016846,XM_006529266,XM_006529267,XM_006529268	Rgl1	ral guanine nucleotide dissociation stimulator,-like 1	protein_coding
19732	NM_009059,XM_006523910,XR_003952098,XR_385304	Rgl2	ral guanine nucleotide dissociation stimulator-like 2	protein_coding
19733	NM_009060,XM_006527572	Rgn	regucalcin	protein_coding
19734	NM_011267	Rgs16	regulator of G-protein signaling 16	protein_coding
19735	NM_009061	Rgs2	regulator of G-protein signaling 2	protein_coding
19736	NM_009062	Rgs4	regulator of G-protein signaling 4	protein_coding
19737	NM_001313705,NM_009063	Rgs5	regulator of G-protein signaling 5	protein_coding
19739	NM_001165934,NM_011268,XM_011248828	Rgs9	regulator of G-protein signaling 9	protein_coding
19743	NM_011269,XM_006523912,XM_006523913	Rhag	Rhesus blood group-associated A glycoprotein	protein_coding
19744	NM_053075	Rheb	Ras homolog enriched in brain	protein_coding
19746	NM_011270,XR_003954896	Rhd	Rh blood group, D antigen	protein_coding
19752	NM_011271	Rnase1	ribonuclease, RNase A family, 1 (pancreatic)	protein_coding
19762	NM_009065	Rit2	Ras-like without CAAX 2	protein_coding
19763	NM_009066	Ring1	ring finger protein 1	protein_coding
19765	NM_001198949,NM_009067,XM_006523915	Ralbp1	ralA binding protein 1	protein_coding
19766	NM_001359997,NM_009068,XM_006516609,XM_030247206	Ripk1	receptor (TNFRSF)-interacting serine-threonine kinase 1	protein_coding
19769	NM_001163310,NM_009069,NR_028056,XM_006501158,XM_006501160,XM_017319506,XM_030252478	Rit1	Ras-like without CAAX 1	protein_coding
19771	NM_001173483,NM_001357450,NM_020599,XM_006540717,XM_011250827,XM_030242244,XM_030242245,XM_030242246	Rlbp1	retinaldehyde binding protein 1	protein_coding
19773	NM_011272	Rln1	relaxin 1	protein_coding
19775	NM_011273	Xpr1	xenotropic and polytropic retrovirus receptor 1	protein_coding
19777	NM_011274,XM_006539676,XM_006539677,XM_006539678	Uri1	URI1, prefoldin-like chaperone	protein_coding
19782	NR_001460	Rmrp	RNA component of mitochondrial RNAase P	[illegible]
19799	NR_002841	Rn4.5s	4.5S RNA	rRNA
19817	NR_030687	Rn7sk	RNA, 7SK, nuclear	snRNA
19819	NM_001286865,NM_011275,NR_104608,XR_001780439,XR_001780440	Rnaseh1	ribonuclease H1	protein_coding
19820	NM_001358205,NM_011276,XM_006527918,XM_006527920,XM_006527921,XM_011247551,XM_011247552,XM_030251269	Rlim	ring finger protein, LIM domain interacting	protein_coding
19821	NM_001360844,NM_001360845,NM_001360847,NM_011277,XM_006529269	Rnf2	ring finger protein 2	protein_coding
19822	NM_001304269,NM_001304270,NM_011278	Rnf4	ring finger protein 4	protein_coding
19823	NM_001311135,NM_011279	Rnf7	ring finger protein 7	protein_coding
19824	NM_011280,XM_017317359,XM_017317360,XM_017317361	Trim10	tripartite motif-containing 10	protein_coding
19826	NM_001080127,NM_001080128,NM_001357626,NM_009070	Rnps1	ribonucleic acid binding protein S1	protein_coding
19842	NR_004411	Rnu1a1	U1a1 small nuclear RNA	snRNA
19844	NR_004412	Rnu1b1	U1b1 small nuclear RNA	snRNA
19847	NR_004413	Rnu1b6	U1b6 small nuclear RNA	snRNA
19848	NR_004414	Rnu2-10	U2 small nuclear RNA 10	snRNA
19850	NR_002842	Rnu3a	U3A small nuclear RNA	snRNA
19853	gene-Rnu3b-ps2	Rnu3b-ps2	U3B small nuclear RNA pseudogene 2	pseudogene
19858	NR_004415	Rnu3b1	U3B small nuclear RNA 1	snRNA
19859	NR_027863	Rnu3b2	U3B small nuclear RNA 2	snRNA
19860	NR_004416	Rnu3b3	U3B small nuclear RNA 3	snRNA
19861	NR_002843	Rnu3b4	U3B small nuclear RNA 4	snRNA
19862	NR_003027	Rnu6	U6 small nuclear RNA	snRNA
19866	NR_024201	Rnu7	U7 small nuclear RNA	snRNA
19870	NR_004417	Snord73a	small nucleolar RNA, C/D box U73A	snoRNA
19871	NR_004418	Rnu73b	U73B small nuclear RNA	snRNA
19872	NR_004419	Rny1	RNA, Y1 small cytoplasmic, Ro-associated	Y_RNA
19874	NR_024202	Rny3	RNA, Y3 small cytoplasmic (associated with Ro protein)	Y_RNA
19876	NM_019413,XM_006522957,XM_006522958,XM_006522960,XM_006522961,XM_006522962,XM_006522963,XM_006522964,XM_006522965,XM_011246105,XM_011246106,XM_017316893,XM_017316894,XM_030249003,XM_030249005	Robo1	roundabout guidance receptor 1	protein_coding
19877	NM_009071,XM_006525725,XR_001782350	Rock1	Rho-associated coiled-coil containing protein kinase 1	protein_coding
19878	NM_009072,XM_006515019,XM_006515020	Rock2	Rho-associated coiled-coil containing protein kinase 2	protein_coding
19879	NM_001164634,NM_001164635,NM_031194,XM_011247171,XM_030250797	Slc22a8	solute carrier family 22 (organic anion transporter), member 8	protein_coding
19881	NM_009073	Rom1	rod outer segment membrane protein 1	protein_coding
19882	NM_001287261,NM_009074,NR_109782,XM_006511654,XM_006511655,XM_006511656,XM_006511658,XM_017313218,XR_379756,XR_379757	Mst1r	macrophage stimulating 1 receptor (c-met-related tyrosine kinase)	protein_coding
19883	NM_001289916,NM_001289917,NM_013646,XM_006510877,XM_006510878,XM_006510880,XM_011242682,XM_030244146,XM_030244147	Rora	RAR-related orphan receptor alpha	protein_coding
19885	NM_001293734,NM_011281,NR_121656,XM_006501162,XM_006501163,XM_006501164	Rorc	RAR-related orphan receptor gamma	protein_coding
19886	NM_011282,XM_011243147,XM_017313855,XM_017313856,XM_017313857,XM_017313858,XM_017313859	Ros1	Ros1 proto-oncogene	protein_coding
19888	NM_001195662,NM_001370921,NM_011283,XM_011238412,XM_017313202,XM_017313203,XM_030252325	Rp1	retinitis pigmentosa 1 (human)	protein_coding
19889	NM_001290643,NM_001290644,NM_133669,NR_110966,NR_110967,XM_017318439	Rp2	retinitis pigmentosa 2 homolog	protein_coding
19891	NM_011284	Rpa2	replication protein A2	protein_coding
19892	NM_029987,XM_006501165,XR_001783668	Rpe65	retinal pigment epithelium 65	protein_coding
19893	NM_001177950,NM_001177951,NM_001177952,NM_001177953,NM_001177954,NM_011285	Rpgr	retinitis pigmentosa GTPase regulator	protein_coding
19894	NM_001302344,NM_001302345,NM_011286,XM_011248185,XM_011248186,XM_011248187,XM_011248188	Rph3a	rabphilin 3A	protein_coding
19895	NM_009075	Rpia	ribose 5-phosphate isomerase A	protein_coding
19896	NM_001357628,NM_011287	Rpl10a	ribosomal protein L10A	protein_coding
19897	gene-Gm10275	Gm10275	predicted pseudogene 10275	pseudogene
19899	NM_009077,XM_017322049,XM_030242247	Rpl18	ribosomal protein L18	protein_coding
19921	NM_001159483,NM_009078,XM_006532610,XM_030245705	Rpl19	ribosomal protein L19	protein_coding
19933	NM_019647,XM_017320746,XM_017320747	Rpl21	ribosomal protein L21	protein_coding
19934	NM_001277113,NM_001277114,NM_009079,NR_102291,NR_1022	Rpl22	ribosomal protein L22	protein_coding
19935	NM_011288,XM_006508516,XM_006508517,XM_006508518,XM_017322050	Mrpl23	mitochondrial ribosomal protein L23	protein_coding
19941	NM_009080	Rpl26	ribosomal protein L26	protein_coding
19942	NM_011289	Rpl27	ribosomal protein L27	protein_coding
19943	NM_009081,XM_006539679	Rpl28	ribosomal protein L28	protein_coding
19944	NM_001324533,NM_001324534,NM_009082,XM_006511660	Rpl29	ribosomal protein L29	protein_coding
19946	NM_001163485,NM_009083	Rpl30	ribosomal protein L30	protein_coding
19947	gene-Rpl30-ps1	Rpl30-ps1	ribosomal protein L30, pseudogene 1	pseudogene
19948	gene-Rpl30-ps2	Rpl30-ps2	ribosomal protein L30, pseudogene 2	pseudogene
19949	gene-Rpl30-ps3	Rpl30-ps3	ribosomal protein L30, pseudogene 3	pseudogene
19951	NM_172086	Rpl32	ribosomal protein L32	protein_coding
19952	gene-Rpl32-ps	Rpl32-ps	ribosomal protein L32, pseudogene	pseudogene
19981	NM_009084	Rpl37a	ribosomal protein L37a	protein_coding
19982	NM_019865	Rpl36a	ribosomal protein L36A	protein_coding
19988	NM_011290,XM_006530227	Rpl6	ribosomal protein L6	protein_coding
19989	NM_011291	Rpl7	ribosomal protein L7	protein_coding
20005	NM_011292	Rpl9	ribosomal protein L9	protein_coding
20014	NM_001355163,NM_001355164,NM_001355165,NM_019642,XM_006499021,XM_006499024,XM_030248863,XM_030248864	Rpn2	ribophorin II	protein_coding
20016	NM_009085	Polr1c	polymerase (RNA) I polypeptide C	protein_coding
20017	NM_009086,XM_006499025,XR_374430	Polr1b	polymerase (RNA) I polypeptide B	protein_coding
20018	NM_009087,NM_181730,XM_030254228,XM_030254229,XM_030254230,XM_030254231,XM_030254232,XM_030254233	Polr1d	polymerase (RNA) I polypeptide D	protein_coding
20019	NM_009088,XM_006505804	Polr1a	polymerase (RNA) I polypeptide A	protein_coding
20020	NM_001291068	Polr2a	polymerase (RNA) II (DNA directed) polypeptide A	protein_coding
20021	NM_009090	Polr2c	polymerase (RNA) II (DNA directed) polypeptide C	protein_coding
20022	NM_011293	Polr2j	polymerase (RNA) II (DNA directed) polypeptide J	protein_coding
20024	NM_011294,XM_006520042	Sub1	SUB1 homolog, transcriptional regulator	protein_coding
20028	NM_001159730,NM_024458	Pdc	phosducin	protein_coding
20042	NM_011295,XM_030244962,XR_003948630	Rps12	ribosomal protein S12	protein_coding
20044	NM_020600	Rps14	ribosomal protein S14	protein_coding
20054	NM_001310726,NM_009091	Rps15	ribosomal protein S15	protein_coding
20055	NM_013647	Rps16	ribosomal protein S16	protein_coding
20068	NM_009092	Rps17	ribosomal protein S17	protein_coding
20084	NM_011296	Rps18	ribosomal protein S18	protein_coding
20085	NM_001360115,NM_001360116,NM_001360118,NM_001360119,NM_001360125,NM_001360128,NM_023133,NR_153408,NR_153409	Rps19	ribosomal protein S19	protein_coding
20088	NM_001360584,NM_001360585,NM_001360586,NM_011297,NM_207634,NM_207635,XM_030247696	Rps24	ribosomal protein S24	protein_coding
20090	NM_009093	Rps29	ribosomal protein S29	protein_coding
20091	NM_016959	Rps3a1	ribosomal protein S3A1	protein_coding
20102	NM_009094,XM_011247553	Rps4x	ribosomal protein S4, X-linked	protein_coding
20103	NM_009095	Rps5	ribosomal protein S5	protein_coding
20104	NM_009096	Rps6	ribosomal protein S6	protein_coding
20111	NM_001285505,NM_001285506,NM_009097,XM_006538677	Rps6ka1	ribosomal protein S6 kinase polypeptide 1	protein_coding
20112	NM_011299,XM_006523198,XM_006523199,XM_006523200,XM_006523201	Rps6ka2	ribosomal protein S6 kinase, polypeptide 2	protein_coding
20115	NM_011300	Rps7	ribosomal protein S7	protein_coding
20116	NM_009098	Rps8	ribosomal protein S8	protein_coding
20128	NM_001357467,NM_009099	Trim30a	tripartite motif-containing 30A	protein_coding
20129	NM_009100	Rptn	repetin	protein_coding
20130	NM_001360132,NM_001360133,NM_009101	Rras	related RAS viral (r-ras) oncogene	protein_coding
20132	NM_009102,XM_017319507	Rrh	retinal pigment epithelium derived rhodopsin homolog	protein_coding
20133	NM_009103	Rrm1	ribonucleotide reductase M1	protein_coding
20135	NM_009104	Rrm2	ribonucleotide reductase M2	protein_coding
20147	NM_011302,XM_030251270	Rs1	retinoschisis (X-linked, juvenile) 1 (human)	protein_coding
20148	NM_001172424,NM_001357274,NM_001357275,NM_001357276,NM_001357277,NM_011303,XM_006538681,XM_030253368,XR_003954897	Dhrs3	dehydrogenase/reductase (SDR family) member 3	protein_coding
20163	NM_009105,XM_006497405	Rsu1	Ras suppressor protein 1	protein_coding
20166	NM_001136227,NM_001355524,NM_001355525,NM_009106,NM_133641,XM_006505805,XM_006505807,XM_006505808,XM_006505809,XM_006505810,XM_006505811,XM_006505812,XM_030255273,XM_030255274,XM_030255275	Rtkn	rhotekin	protein_coding
20167	NM_001025364,NM_013648	Rtn2	reticulon 2 (Z-band associated protein)	protein_coding
20168	NM_001003933,NM_001003934,NM_001271486,NM_001271487,NM_053076,XM_017318088	Rtn3	reticulon 3	protein_coding

20170	NM_176785	Hps6	HPS6, biogenesis of lysosomal organelles complex 2 subunit 3	protein_coding
20174	NM_011304,XM_006540722,XM_006540723	Ruvbl2	RuvB-like protein 2	protein_coding
20181	NM_001290481,NM_001290482,NM_011305,XM_006497805,XM_030248867	Rxra	retinoid X receptor alpha	protein_coding
20182	NM_001205214,NM_001205215,NM_001205216,NM_011306	Rxrb	retinoid X receptor beta	protein_coding
20183	NM_001159731,NM_009107,XM_030252326	Rxrg	retinoid X receptor gamma	protein_coding
20184	NM_001293660,NM_001359757,NM_001359758,NM_011307,NR_121592,XM_006517167,XM_011244499,XM_030247207	Uimc1	ubiquitin interaction motif containing 1	protein_coding
20185	NM_001252313,NM_011308,NM_177229,XM_006532613,XM_006532614,XM_006532615,XM_006532616,XM_006532618,XM_006532619,XM_006532621,XM_006532623,XM_006532624,XM_006532626,XM_006532628,XM_017314356,XM_017314359,XM_017314361,XM_017314362,XM_017314367,XM_017314368,XM_017314369,XM_017314370,XM_017314372,XM_017314373,XM_017314374,XM_017314375,XM_017314376,XM_017314377,XM_017314378,XM_017314379,XM_017314380,XM_017314381,XM_017314382,XM_017314383,XM_017314384,XM_017314385,XM_017314388,XM_017314389,XM_017314390,XM_030245706,XM_030245707,XM_030245708,XM_030245709,XM_030245710,XM_030245711,XM_030245712,XM_030245713,XM_030245714,XM_030245715,XM_030245716,XM_030245717,XM_030245718,XM_030245719	Ncor1	nuclear receptor co-repressor 1	protein_coding
20186	NM_001163504,NM_001163700,NM_009108,XM_006513391,XM_006513393,XM_030244963,XR_001779493	Nr1h4	nuclear receptor subfamily 1, group H, member 4	protein_coding
20187	NM_001042607,NM_001284258,NM_013649	Ryk	receptor-like tyrosine kinase	protein_coding
20190	NM_009109,XM_006539688,XM_006539689,XM_006539692,XM_030242248,XM_030242249,XM_030242250,XM_030242251,XM_030242252,XM_030242253,XM_030242254,XM_030242255,XM_030242256,XR_003946422	Ryr1	ryanodine receptor 1, skeletal muscle	protein_coding
20191	NM_023868,XM_006516610,XM_011244296,XM_017315445,XM_017315446,XM_017315447,XM_017315448,XM_017315449,XM_017315450,XM_017315451,XM_017315452,XM_017315453,XM_017315454,XM_017315455,XM_017315457,XM_017315460,XM_017315461,XM_030247209,XR_003950437,XR_003950438,XR_003950439	Ryr2	ryanodine receptor 2, cardiac	protein_coding
20192	NM_001319156,XM_017316700,XM_017316702,XM_017316705,XM_017316708,XM_017316709,XM_017316711,XM_017316712,XR_003951556	Ryr3	ryanodine receptor 3	protein_coding
20193	NM_011309	S100a1	S100 calcium binding protein A1	protein_coding
20194	NM_009112,XM_030252479	S100a10	S100 calcium binding protein A10 (calpactin)	protein_coding
20195	NM_016740,XM_030252480	S100a11	S100 calcium binding protein A11	protein_coding
20196	NM_009113,XM_030252481	S100a13	S100 calcium binding protein A13	protein_coding
20197	NM_001355597,NM_001355600,NM_001355602,NM_011310,XM_006501175,XM_006501176,XM_011240047,XM_017319509,XM_030252482	S100a3	S100 calcium binding protein A3	protein_coding
20198	NM_011311,XM_006501177,XM_006501178	S100a4	S100 calcium binding protein A4	protein_coding
20200	NM_011313	S100a6	S100 calcium binding protein A6 (calcyclin)	protein_coding
20201	NM_013650	S100a8	S100 calcium binding protein A8 (calgranulin A)	protein_coding
20202	NM_001281852,NM_009114	S100a9	S100 calcium binding protein A9 (calgranulin B)	protein_coding
20203	NM_009115	S100b	S100 protein, beta polypeptide, neural	protein_coding
20204	NM_009116,XM_006497806	Prrx2	paired related homeobox 2	protein_coding
20210	NM_011315,XM_030242257	Saa3	serum amyloid A 3	protein_coding
20215	NM_009118,XM_006529274,XM_011247944,XM_017319395,XM_017319396,XM_017319397,XM_030252327,XM_030252328,XM_030252329	Sag	S-antigen, retina and pineal gland (arrestin)	protein_coding
20216	NM_016870,NM_212441,NM_212442,XM_006507475,XM_017322060,XM_017322061,XM_017322062,XM_017322063	Acsm3	acyl-CoA synthetase medium-chain family member 3	protein_coding
20218	NM_011317,NR_045036,XR_001784112,XR_376300	Khdrbs1	KH domain containing, RNA binding, signal transduction associated 1	protein_coding
20220	NM_009119	Sap18	Sin3-associated polypeptide 18	protein_coding
20222	NM_013651	Sf3a2	splicing factor 3a, subunit 2	protein_coding
20224	NM_001358487,NM_001358488,NM_009120,XM_006513401,XM_030244964,XM_030244965	Sar1a	secretion associated Ras related GTPase 1A	protein_coding
20226	NM_001204979,NM_011319	Sars	seryl-aminoacyl-tRNA synthetase	protein_coding
20227	NM_016882,XM_030250798	Sart1	squamous cell carcinoma antigen recognized by T cells 1	protein_coding
20229	NM_001291865,NM_009121,XM_030251271	Sat1	spermidine/spermine N1-acetyl transferase 1	protein_coding
20230	NM_001163630,NM_001163631,NM_001163632,NM_001357636,NM_001357637,NM_001357638,NM_001357640,NM_009122,XM_006523924,XM_006523925,XM_006523926,XM_006523927,XM_017317363,XM_017317365,XM_017317366	Satb1	special AT-rich sequence binding protein 1	protein_coding
20231	NM_009123	Nkx1-2	NK1 homeobox 2	protein_coding
20238	NM_001199304,NM_001199305,NM_009124,XM_011244381,XM_011244383,XM_011244385,XM_011244386,XM_011244387,XM_011244388,XM_011244389,XM_011244390,XM_011244391,XM_011244392,XM_011244393,XM_017315463,XM_030247210,XM_030247211,XM_030247212,XM_030247213	Atxn1	ataxin 1	protein_coding
20239	NM_001359153,NM_009125,XM_006530228,XM_017320748,XM_017320749,XM_017320750,XM_030254234,XM_030254235,XM_030254236,XM_030254237,XM_030254238,XM_030254239,XM_030254240,XM_030254241,XM_030254242,XM_030254243,XM_030254244,XM_030254245,XM_030254246,XM_030254247,XM_030254248,XM_030254249,XM_030254250,XM_030254251,XM_030254252,XM_030254253,XM_030254254,XM_030254255,XM_030254256,XM_030254257,XM_030254258,XM_030254259,XM_030254260,XM_030254261,XM_030254262,XM_030254263,XM_030254264,XM_030254265,XM_030254266,XR_003955602	Atxn2	ataxin 2	protein_coding
20249	NM_009127	Scd1	stearoyl-Coenzyme A desaturase 1	protein_coding
20250	NM_009128	Scd2	stearoyl-Coenzyme A desaturase 2	protein_coding
20254	NM_001310680,NM_009129	Scg2	secretogranin II	protein_coding
20255	NM_001164790,NM_009130,XM_006510881,XM_017313220	Scg3	secretogranin III	protein_coding
20256	NM_009131	Clec11a	C-type lectin domain family 11, member a	protein_coding
20257	NM_025285	Stmn2	stathmin-like 2	protein_coding
20259	NM_001146196,NM_009132	Scin	scinderin	protein_coding
20262	NM_009133	Stmn3	stathmin-like 3	protein_coding
20264	NM_001205321,NM_009134,XM_017313221,XM_017313222,XM_017313223	Scn10a	sodium channel, voltage-gated, type X, alpha	protein_coding
20265	NM_001313997,NM_018733,XM_006499027,XM_006499028,XR_001781558,XR_001781559	Scn1a	sodium channel, voltage-gated, type I, alpha	protein_coding
20266	NM_011322,XM_030242258	Scn1b	sodium channel, voltage-gated, type I, beta	protein_coding
20269	NM_001355166,NM_001355167,NM_001355168,NM_001355169,NM_001355170,NM_001355171,NM_018732,XM_006499031,XM_011239391	Scn3a	sodium channel, voltage-gated, type III, alpha	protein_coding
20271	NM_001253860,NM_021544,XM_006511993,XM_006511995,XM_006511996,XM_006511997	Scn5a	sodium channel, voltage-gated, type V, alpha	protein_coding
20272	NM_009135	Scn7a	sodium channel, voltage-gated, type VII, alpha	protein_coding
20273	NM_001077499,NM_011323,XM_006520651,XM_006520655,XM_006520656,XM_017316512,XM_017316513,XM_017316514,XM_017316515,XM_017316516,XM_017316517,XM_017316519	Scn8a	sodium channel, voltage-gated, type VIII, alpha	protein_coding
20274	NM_001290674,NM_001290675,XM_006499033,XM_006499035,XM_006499036,XR_001781564,XR_374432	Scn9a	sodium channel, voltage-gated, type IX, alpha	protein_coding
20276	NM_011324,XM_017321471,XM_030255276	Scnn1a	sodium channel, nonvoltage-gated 1 alpha	protein_coding
20277	NM_001272023,NM_011325,NR_073548	Scnn1b	sodium channel, nonvoltage-gated 1 beta	protein_coding
20280	NM_011327,XM_011240477	Scp2	sterol carrier protein 2, liver	protein_coding
20284	NM_009136,XM_017312622	Scrg1	scrapie responsive gene 1	protein_coding
20286	NM_001081016,XM_011245526,XM_017316520	Zc3h7b	zinc finger CCCH type containing 7B	protein_coding
20287	NM_001287171,NM_001309439,NM_011328,XM_017322065	Sct	secretin	protein_coding
20288	NM_001113326,NM_031195,XM_006509311,XM_006509312	Msr1	macrophage scavenger receptor 1	protein_coding
20289	NM_198885,XM_006520660	Scx	scleraxis	protein_coding
20292	NM_011330	Ccl11	chemokine (C-C motif) ligand 11	protein_coding
20293	NM_011331	Ccl12	chemokine (C-C motif) ligand 12	protein_coding
20295	NM_011332,XM_006530782	Ccl17	chemokine (C-C motif) ligand 17	protein_coding
20296	NM_011333	Ccl2	chemokine (C-C motif) ligand 2	protein_coding
20299	NM_009137	Ccl22	chemokine (C-C motif) ligand 22	protein_coding
20300	NM_009138,NR_033527,XM_006508736,XM_011242043,XM_011242044,XM_011242045,XM_030243364	Ccl25	chemokine (C-C motif) ligand 25	protein_coding
20301	NM_001048179,NM_001164044,NM_001164045,NM_001347065,NM_001361246,NM_011336,NR_154591,XM_006537704,XM_006537705,XM_006537706,XM_011249967,XM_030253369,XM_030253370,XM_030253371	Ccl27a	chemokine (C-C motif) ligand 27A	protein_coding
20302	NM_011337	Ccl3	chemokine (C-C motif) ligand 3	protein_coding
20303	NM_013652,XM_011248832	Ccl4	chemokine (C-C motif) ligand 4	protein_coding
20304	NM_013653	Ccl5	chemokine (C-C motif) ligand 5	protein_coding
20305	NM_009139	Ccl6	chemokine (C-C motif) ligand 6	protein_coding
20306	NM_013654	Ccl7	chemokine (C-C motif) ligand 7	protein_coding
20307	NM_021443	Ccl8	chemokine (C-C motif) ligand 8	protein_coding
20308	NM_011338	Ccl9	chemokine (C-C motif) ligand 9	protein_coding
20309	NM_011339	Cxcl15	chemokine (C-X-C motif) ligand 15	protein_coding
20310	NM_009140	Cxcl2	chemokine (C-X-C motif) ligand 2	protein_coding
20311	NM_009141	Cxcl5	chemokine (C-X-C motif) ligand 5	protein_coding
20312	NM_009142	Cx3cl1	chemokine (C-X3-C motif) ligand 1	protein_coding
20315	NM_001012477,NM_013655,NM_021704	Cxcl12	chemokine (C-X-C motif) ligand 12	protein_coding
20316	NM_001359106,NM_009143,XM_030245720	Sdf2	stromal cell derived factor 2	protein_coding
20317	NM_011340	Serpinf1	serine (or cysteine) peptidase inhibitor, clade F, member 1	protein_coding
20318	NM_001302467,NM_001302468,NM_001302469,NM_011341	Sdf4	stromal cell derived factor 4	protein_coding
20319	NM_009144,XM_006501183	Sfrp2	secreted frizzled-related protein 2	protein_coding
20320	NM_001293673,NM_001357751,NM_009145,NR_151824,XM_006510882,XM_006510883,XM_006510886,XM_030244148,XR_003947819,XR_003947820	Nptn	neuroplastin	protein_coding
20321	NM_001113478,NM_009146,XM_006501185,XM_017319511,XR_001783669	Frrs1	ferric-chelate reductase 1	protein_coding
20322	NM_146126	Sord	sorbitol dehydrogenase	protein_coding
20324	NM_138741	Cavin2	caveolae associated 2	protein_coding
20333	NM_011342	Sec22b	SEC22 homolog B, vesicle trafficking protein	protein_coding
20334	NM_001361956,NM_009147	Sec23a	SEC23 homolog A, COPII coat complex component	protein_coding
20335	NM_001109971,NM_001109972,NM_011343	Sec61g	SEC61, gamma subunit	protein_coding
20336	NM_001347089,NM_009148,XM_011241262	Exoc4	exocyst complex component 4	protein_coding
20338	NM_001039089,NM_011344	Sel1l	sel-1 suppressor of lin-12-like (C. elegans)	protein_coding
20339	NM_011345,XM_006496715,XM_017319404	Sele	selectin, endothelial cell	protein_coding
20340	NM_001368315,NM_009149	Glg1	golgi apparatus protein 1	protein_coding
20341	NM_009150	Selenbp1	selenium binding protein 1	protein_coding
20342	NM_019414	Selenbp2	selenium binding protein 2	protein_coding
20343	NM_001164059,NM_011346,XM_006496717,XM_006496718,XM_030252331,XM_030252333	Sell	selectin, lymphocyte	protein_coding
20344	NM_011347	Selp	selectin, platelet	protein_coding
20345	NM_009151,XM_006530229	Selplg	selectin, platelet (p-selectin) ligand	protein_coding
20346	NM_001243072,NM_001243073,NM_009152,XM_006503557,XM_006503558,XM_006503559,XM_011240665	Sema3a	sema domain, immunoglobulin domain (Ig), short basic domain, secreted, (semaphorin) 3A	protein_coding
20347	NM_001042779,NM_001291537,NM_001291538,NM_001291539,NM_009153,NR_111986,XM_006511664,XM_006511665,XM_006511666,XM_006511671,XM_006511673,XM_006511674,XM_011242862,XM_011242863,XM_011242864,XM_011242865,XM_011242867,XM_017313225	Sema3b	sema domain, immunoglobulin domain (Ig), short basic domain, secreted, (semaphorin) 3B	protein_coding
20348	NM_013657	Sema3c	sema domain, immunoglobulin domain (Ig), short basic domain, secreted, (semaphorin) 3C	protein_coding
20349	NM_011348	Sema3e	sema domain, immunoglobulin domain (Ig), short basic domain, secreted, (semaphorin) 3E	protein_coding
20350	NM_001311151,NM_011349,XM_006511676,XM_017313226,XM_017313227,XM_017313228,XM_017313229,XM_017313230,XR_379758,XR_379759,XR_871078	Sema3f	sema domain, immunoglobulin domain (Ig), short basic domain, secreted, (semaphorin) 3F	protein_coding
20351	NM_001163489,NM_001163490,NM_001163491,NM_013658,XM_006501187,XM_006501188,XM_006501189,XM_006501190,XM_006501191,XM_011240051,XM_030252484	Sema4a	sema domain, immunoglobulin domain (Ig), transmembrane domain (TM) and short cytoplasmic domain, (semaphorin) 4A	protein_coding
20352	NM_001360135,NM_013659,XM_030242259	Sema4b	sema domain, immunoglobulin domain (Ig), transmembrane domain (TM) and short cytoplasmic domain, (semaphorin) 4B	protein_coding
20353	NM_001126047,NM_001304329,NM_001304330,XM_006495803,XM_006495804,XM_006495805,XM_006495807,XM_006495808,XM_006495809,XM_006495810,XM_017319410,XM_030252338	Sema4c	sema domain, immunoglobulin domain (Ig), transmembrane domain (TM) and short cytoplasmic domain, (semaphorin) 4C	protein_coding
20354	NM_001281880,NM_013660,XM_030247214,XM_030247216	Sema4d	sema domain, immunoglobulin domain (Ig), transmembrane domain (TM) and short cytoplasmic domain, (semaphorin) 4D	protein_coding
20355	NM_001308374,NM_011350,XM_011241264,XM_011241265	Sema4f	sema domain, immunoglobulin domain (Ig), TM domain, and short cytoplasmic domain	protein_coding
20356	NM_009154,XM_006520043,XM_011245340,XM_030248430,XM_030248431	Sema5a	sema domain, seven thrombospondin repeats (type 1 and type 1-like), transmembrane domain (TM) and short cytoplasmic domain, (semaphorin)	protein_coding
20357	NM_013661,XM_006521869,XM_011245844,XM_011245847,XM_030249006,XR_003951770,XR_875743	Sema5b	sema domain, seven thrombospondin repeats (type 1 and type 1-like), transmembrane domain (TM) and short cytoplasmic domain, (semaphorin)	protein_coding
20358	NM_001311097,NM_018744,XM_006525726,XM_006525727	Sema6a	sema domain, transmembrane domain (TM), and cytoplasmic domain, (semaphorin) 6A	protein_coding
20359	NM_001130456,NM_013662,XM_006523930	Sema6b	sema domain, transmembrane domain (TM), and cytoplasmic domain, (semaphorin) 6B	protein_coding
20360	NM_001272024,NM_011351,NR_073549,NR_073550,NR_073551,XM_006501192,XM_006501193,XM_006501194,XM_006501195,XM_006501196,XM_006501197,XM_006501198,XM_006501201,XM_006501202,XM_006501204,XM_006501205,XM_006501206,XM_006501207,XM_006501208,XM_006501209,XM_030252486,XM_030252487,XM_030252489,XM_030252490,XM_030252491,XM_030252492,XM_030252493,XM_030252494,XM_030252495,XM_030252496,XM_030252498,XM_030252499,XM_030252500,XM_030252501,XM_030252502,XM_030252503,XM_030252504,XM_030252505,XM	Sema6c	sema domain, transmembrane domain (TM), and cytoplasmic domain, (semaphorin) 6C	protein_coding
20361	NM_011352,XM_006510888,XM_006510889,XM_030244149	Sema7a	sema domain, immunoglobulin domain (Ig), and GPI membrane anchor, (semaphorin) 7A	protein_coding
20362	NM_001252332,NM_001252333,NM_001347496,NM_001361968,NM_001361969,NM_033144,XM_006532633,XM_006532635,XM_011248834,XM_017314392,XM_030245721,XM_030245722	Sept8	septin 8	protein_coding
20363	NM_001042613,NM_001042614,NM_009155	Selenop	selenoprotein P	protein_coding
20364	NM_009156	Selenow	selenoprotein W	protein_coding
20365	NM_011353	Serf1	small EDRK-rich factor 1	protein_coding
20370	NM_001291225,NM_021286,XM_006532636,XM_006532637,XM_006532639,XM_006532640,XM_030245723	Sez6	seizure related gene 6	protein_coding
20371	NM_001199347,NM_001199348,NM_054039	Foxp3	forkhead box P3	protein_coding
20375	NM_011355,XM_006499038,XM_011239397,XM_017316733	Spi1	spleen focus forming virus (SFFV) proviral integration oncogene	protein_coding
20377	NM_013834,XM_011242128	Sfrp1	secreted frizzled-related protein 1	protein_coding
20378	NM_011356	Frzb	frizzled-related protein	protein_coding
20379	NM_016687	Sfrp4	secreted frizzled-related protein 4	protein_coding
20382	NM_011358,XM_011248835,XM_030245724,XR_003949363,XR_003949364,XR_003949365,XR_003949366	Srsf2	serine/arginine-rich splicing factor 2	protein_coding
20383	NM_013663,NR_036613	Srsf3	serine/arginine-rich splicing factor 3	protein_coding
20384	NM_001079694,NM_001079695,NM_001347415,NM_001347416,NM_009159,XM_006515630	Srsf5	serine/arginine-rich splicing factor 5	protein_coding
20391	NM_009161,XM_011248836,XM_017314395	Sgca	sarcoglycan, alpha (dystrophin-associated glycoprotein)	protein_coding
20392	NM_001130188,NM_001130189,NM_001130190,NM_001130191,NM_011360,XM_006505019,XM_006505020,XM_006505022,XM_006505023,XM_006505024,XM_011241051,XM_030255277,XM_030255278,XM_030255280,XM_030255281	Sgce	sarcoglycan, epsilon	protein_coding
20393	NM_001161845,NM_001161847,NM_001161848,NM_001161849,NM_001161850,NM_011361	Sgk1	serum/glucocorticoid regulated kinase 1	protein_coding
20394	NM_009162	Scg5	secretogranin V	protein_coding
20397	NM_001316673,NM_001316674,NM_009163,XM_017313860	Sgpl1	sphingosine phosphate lyase 1	protein_coding
20399	NM_001081459,NM_001289538,NM_001289539,NM_001289540,NM_001289541,NM_001289542,NM_011363,NR_110346,XM_006507478,XM_006507480,XM_006507481,XM_006507482,XM_006507485,XM_006507487,XM_030242260,XM_030242261,XR_003946424	Sh2b1	SH2B adaptor protein 1	protein_coding
20401	NM_001316684,NM_001316685,NM_001316686,NM_009164,NR_133564,XM_017316521,XM_030248432	Sh3bp1	SH3-domain binding protein 1	protein_coding
20402	NM_011743,XM_006499042,XM_006499044,XM_030248878,XM_030248880	Zfp106	zinc finger protein 106	protein_coding
20403	NM_001198968,NM_001198969,NM_011365,XM_006515023,XM_006515024,XM_006515025,XM_006515026,XM_006515027,XM_030246627	Itsn2	intersectin 2	protein_coding
20404	NM_019535,XM_006537710	Sh3gl2	SH3-domain GRB2-like 2	protein_coding
20405	NM_001252471,NM_001357649,NM_013664	Sh3gl1	SH3-domain GRB2-like 1	protein_coding
20408	NM_001277954,NM_001277955,NM_017400,XM_030242262,XM_030242263,XM_030242264,XM_030242265,XM_030242266,XM_030242267,XM_030242268,XM_030242269	Sh3gl3	SH3-domain GRB2-like 3	protein_coding
20409	NM_017375	Ostf1	osteoclast stimulating factor 1	protein_coding

20410	NM_001271407,NM_001271408,NM_001271409,NM_011366,XM_006518736,XM_006518737,XM_006518741,XM_006518742,XM_006518743,XM_030247697,XM_030247698,XM_030247699,XM_030247700,XM_030247701,XR_003950830	Sorbs3	sorbin and SH3 domain containing 3	protein_coding	NP_001258336.1;NP_001258337.1;NP_001258338.1;NP_035496.1;XP_006518799.1;XP_006518800.1;XP_006518804.1;XP_006518805.1;XP_006518806.1;XP_030103557.1;XP_030103558.1;XP_030103559.1;XP_030103560.1;XP_030103561.1 · 700013 · . · 26.895706 · [illegible]
20411	NM_001034962,NM_001034963,NM_001034964,NM_009166,NM_178362,XM_006526810,XM_006526813,XM_011247172,XM_011247173,XM_011247175,XM_011247176,XM_011247180,XM_011247181,XM_011247183,XM_011247190,XM_011247191,XM_011247193,XM_011247198,XM_011247199,XM_017318090,XM_017318091,XM_017318093,XM_017318095,XM_017318096,XM_017318097,XM_030250799,XM_030250800,XM_030250801,XM_030250802,XM_030250803,XM_030250804,XM_030250805,XM_030250806,XM_030250807,XM_030250808,XM_030250809,XM_030250810,XM_030250811,XM_030250812,XM_030250813,XM_030250814,XM_030250815,XM_030250816,XM_030250817,XM_030250818,XM_030250819,XM_030250820,XM_030250821,XM_030250822,XM_030250823,XM_030250824,XM_030250825,XM_030250826,XM_030250827	Sorbs1	sorbin and SH3 domain containing 1	protein_coding	700014 · . · 14.135437 · [illegible]
20415	NM_011367,XM_011248837,XM_011248838	Shbg	sex hormone binding globulin	protein_coding	NP_035497.1;XP_011247139.1;XP_011247140.1 · 98295 · . · 0.000000 · [illegible]
20416	NM_001113331,NM_011368,XM_006501212,XM_006501213	Shc1	src homology 2 domain-containing transforming protein C1	protein_coding	NP_001106802.1;NP_035498.2;XP_006501275.1;XP_006501276.1 · 98296 · . · 44.877162 · [illegible]
20418	NM_009167	Shc3	src homology 2 domain-containing transforming protein C3	protein_coding	NP_033193.3 · 106179 · . · 0.072782 · [illegible]
20419	NM_011369	Shcbp1	Shc SH2-domain binding protein 1	protein_coding	NP_035499.1 · 1338802 · . · 6.200848 · [illegible]
20420	NM_001159523,NM_009168,XM_011246334,XM_030249612	Shd	src homology 2 domain-containing transforming protein D	protein_coding	NP_001152995.1;NP_033194.1;XP_011244636.1;XP_030105472.1 · 1099461 · . · 1.072203 · [illegible]
20422	NM_009169	Sem1	SEM1, 26S proteasome complex subunit	protein_coding	NP_033195.1 · 109238 · . · 163.901199 · [illegible]
20423	NM_009170,XM_006535648,XM_006535649	Shh	sonic hedgehog	protein_coding	NP_033196.1;XP_006535711.1;XP_006535712.1 · 98297 · . · 0.000000 · [illegible]
20425	NM_009171,XM_006532644	Shmt1	serine hydroxymethyltransferase 1 (soluble)	protein_coding	NP_033197.2;XP_006532707.1 · 98299 · . · 3.030319 · [illegible]
20429	NM_001302357,NM_001302358,NM_001302359,NM_013665,XM_006501215,XM_006501216,XM_006501217,XM_030252507,XM_030252508	Shox2	short stature homeobox 2	protein_coding	NP_001289286.1;NP_001289287.1;NP_001289288.1;NP_038693.1;XP_006501278.1;XP_006501279.1;XP_006501280.1;XP_030108367.1;XP_030108368.1 · 1201673 · . · 0.050122 · [illegible]
20430	NM_001164661,NM_001164662,NM_011370,XM_006540729,XM_017322067,XR_001785497	Cyfip1	cytoplasmic FMR1 interacting protein 1	protein_coding	NP_001158133.1;NP_001158134.1;NP_035500.2;XP_006540792.1;XP_017177556.1 · 1338801 · . · 16.992630 · [illegible]
20431	NM_021882,XM_006513405,XM_030244967	Pmel	premelanosome protein	protein_coding	NP_068682.2;XP_006513468.1;XP_030100827.1 · 98301 · . · 0.581139 · [illegible]
20437	NM_009172,XM_006530784	Siah1a	siah E3 ubiquitin protein ligase 1A	protein_coding	NP_033198.1;XP_006530847.3 · 108064 · . · 7.976822 · [illegible]
20438	NM_001308385,NM_001308386,NM_009173	Siah1b	siah E3 ubiquitin protein ligase 1B	protein_coding	NP_001295314.1;NP_001295315.1;NP_033199.1 · 108063 · . · 2.139302 · [illegible]
20439	NM_009174,XR_001783671	Siah2	siah E3 ubiquitin protein ligase 2	protein_coding	NP_033200.2 · 108062 · . · 3.970026 · [illegible]
20440	NM_001252505,NM_001252506,NM_145933,XM_006521873,XM_006521874,XM_006521875,XM_006521876,XM_011245848	St6gal1	beta galactoside alpha 2,6 sialyltransferase 1	protein_coding	NP_001239434.1;NP_001239435.1;NP_666045.1;XP_006521936.1;XP_006521937.1;XP_006521938.1;XP_006521939.1;XP_011244150.1 · 108470 · . · 5.350189 · [illegible]
20441	NM_001161774,NM_001285520,NM_001285521,NM_009176,XM_006502890,XM_006502891,XM_006502892,XM_006502893,XM_006502894,XM_006502895,XM_006502896,XM_006502897,XM_006502898,XM_006502899,XM_030253372,XM_030253373,XM_030253374,XM_030253375,XR_376301,XR_376302	St3gal3	ST3 beta-galactoside alpha-2,3-sialyltransferase 3	protein_coding	NP_001155246.1;NP_001272449.1;NP_001272450.1;NP_033202.3;XP_006502953.1;XP_006502954.1;XP_006502955.1;XP_006502956.1;XP_006502957.1;XP_006502958.1;XP_006502959.1;XP_006502960.1;XP_006502961.1;XP_006502962.1;XP_030109232.1;XP_030109233.1;XP_030109234.1;XP_030109235.1 · 1316659 · . · 5.031015 · [illegible]
20442	NM_009177,XM_006520664,XM_006520665,XM_011245529	St3gal1	ST3 beta-galactoside alpha-2,3-sialyltransferase 1	protein_coding	NP_033203.1;XP_006520727.1;XP_006520728.1;XP_011243831.1 · 98304 · . · 18.319915 · [illegible]
20443	NM_009178,XM_006510108,XM_006510109,XM_006510110,XM_006510111,XM_006510112,XM_011242429,XM_011242433	St3gal4	ST3 beta-galactoside alpha-2,3-sialyltransferase 4	protein_coding	22.398429 · [illegible]
20444	NM_009179,XM_006530786,XM_006530787,XM_006530788,XM_017312624	St3gal2	ST3 beta-galactoside alpha-2,3-sialyltransferase 2	protein_coding	[illegible]
20445	NM_011371	St6galnac1	ST6 (alpha-N-acetyl-neuraminyl-2,3-beta-galactosyl-1,3)-N-acetylgalactosaminide alpha-2,6-sialyltransferase 1	protein_coding	[illegible]
20446	NM_009180,XR_388384	St6galnac2	ST6 (alpha-N-acetyl-neuraminyl-2,3-beta-galactosyl-1,3)-N-acetylgalactosaminide alpha-2,6-sialyltransferase 2	protein_coding	[illegible]
20447	NM_011372,XM_006501219,XM_006501220,XM_006501221,XM_006501222,XM_011240054,XM_030252509,XM_030252510,XM_030252511,XM_030252512,XR_003954301,XR_003954302	St6galnac3	ST6 (alpha-N-acetyl-neuraminyl-2,3-beta-galactosyl-1,3)-N-acetylgalactosaminide alpha-2,6-sialyltransferase 3	protein_coding	[illegible]
20448	NM_001276425,NM_011373	St6galnac4	ST6 (alpha-N-acetyl-neuraminyl-2,3-beta-galactosyl-1,3)-N-acetylgalactosaminide alpha-2,6-sialyltransferase 4	protein_coding	[illegible]
20449	NM_011374,XM_006506928,XM_006506929,XM_006506930,XM_011241574,XM_011241575,XM_011241576,XM_017321472,XM_030255283	St8sia1	ST8 alpha-N-acetyl-neuraminide alpha-2,8-sialyltransferase 1	protein_coding	[illegible]
20450	NM_009181,XM_006540731	St8sia2	ST8 alpha-N-acetyl-neuraminide alpha-2,8-sialyltransferase 2	protein_coding	[illegible]
20451	NM_009182,XM_030250367	St8sia3	ST8 alpha-N-acetyl-neuraminide alpha-2,8-sialyltransferase 3	protein_coding	[illegible]
20452	NM_001159745,NM_009183,XM_006529275	St8sia4	ST8 alpha-N-acetyl-neuraminide alpha-2,8-sialyltransferase 4	protein_coding	[illegible]
20454	NM_001035228,NM_011375,XM_006505816,XM_006505817,XM_006505818,XM_006505819,XM_006505820,XM_017321473	St3gal5	ST3 beta-galactoside alpha-2,3-sialyltransferase 5	protein_coding	[illegible]
20459	NM_001356304,NM_009184,XM_006500585	Ptk6	PTK6 protein tyrosine kinase 6	protein_coding	[illegible]
20460	NM_001304551,NM_001304553,NM_001304555,NM_001304559,NM_009185,XM_006502906,XM_006502907,XM_017320088,XM_030253376	Stil	Scl/Tal1 interrupting locus	protein_coding	[illegible]
20461	gene-Gm14270	Gm14270	predicted gene 14270	pseudogene	. · [illegible]
20462	NM_001330554,NM_001330555,NM_009186,XM_017316896	Tra2b	transformer 2 beta	protein_coding	[illegible]
20463	NM_001159529,NM_009187	Cox7a2l	cytochrome c oxidase subunit 7A2 like	protein_coding	[illegible]
20464	NM_011376,XM_006512626,XM_006512627,XM_006512628,XM_006512629,XM_006512630,XM_006512631,XM_006512632	Sim1	single-minded family bHLH transcription factor 1	protein_coding	[illegible]
20465	NM_011377,XM_006522966,XM_006522967,XM_006522968,XM_017316897	Sim2	single-minded family bHLH transcription factor 2	protein_coding	[illegible]
20466	NM_001110350,NM_001110351,NM_001357754,NM_011378,XM_006510890,XM_006510891,XM_006510892,XM_006510893,XM_011242683,XM_011242684,XM_011242685,XM_011242686,XM_030244150	Sin3a	transcriptional regulator, SIN3A (yeast)	protein_coding	[illegible]
20467	NM_001113248,NM_009188,XM_006530789	Sin3b	transcriptional regulator, SIN3B (yeast)	protein_coding	[illegible]
20469	NM_001164480,NM_001164481,NM_001164482,NM_001164568,NM_011379,XM_030250828	Sipa1	signal-induced proliferation associated gene 1	protein_coding	[illegible]
20471	NM_009189	Six1	sine oculis-related homeobox 1	protein_coding	[illegible]
20472	NM_011380,XM_006523931,XM_011246335	Six2	sine oculis-related homeobox 2	protein_coding	[illegible]
20474	NM_001362272,NM_011382,XM_006515632	Six4	sine oculis-related homeobox 4	protein_coding	[illegible]
20475	NM_011383,XM_006539694	Six5	sine oculis-related homeobox 5	protein_coding	[illegible]
20479	NM_009190	Vps4b	vacuolar protein sorting 4B	protein_coding	[illegible]
20480	NM_001363991,NM_009191,XM_006507488,XM_006507489,XM_011241702,XM_030242270,XM_030242271,XM_030242272	Clpb	ClpB caseinolytic peptidase B	protein_coding	[illegible]
20481	NM_001357191,NM_011385	Ski	ski sarcoma viral oncogene homolog (avian)	protein_coding	[illegible]
20482	NM_001039090,NM_001271772,NM_011386,XM_006535427	Skil	SKI-like	protein_coding	[illegible]
20491	NM_001029841,NM_009192,XM_006520668,XM_011245530,XM_017316522,XM_030248433	Sla	src-like adaptor	protein_coding	[illegible]
20492	NM_001289724,NM_001289725,NM_009193	Slbp	stem-loop binding protein	protein_coding	[illegible]
20495	NM_001079690,NM_183354,XM_030248885,XM_030248886,XM_030248887,XM_030248888,XM_030248889,XM_030248890,XM_030248891,XM_030248892	Slc12a1	solute carrier family 12, member 1	protein_coding	[illegible]
20496	NM_009194,XM_006525732,XR_001782351,XR_001782352	Slc12a2	solute carrier family 12, member 2	protein_coding	[illegible]
20497	NM_001205311,NM_019415,XM_017312625,XM_017312626,XM_017312627,XR_001778414	Slc12a3	solute carrier family 12, member 3	protein_coding	[illegible]
20498	NM_001253804,NM_009195,NR_045594,XM_006530792	Slc12a4	solute carrier family 12, member 4	protein_coding	[illegible]
20499	NM_001360694,NM_011390,XM_006517170,XM_006517171,XM_006517173,XM_006517174,XM_006517175	Slc12a7	solute carrier family 12, member 7	protein_coding	[illegible]
20500	NM_022411,XM_006532645,XM_006532647,XM_006532648,XM_006532649,XR_003949367	Slc13a2	solute carrier family 13 (sodium-dependent dicarboxylate transporter), member 2	protein_coding	[illegible]
20501	NM_009196	Slc16a1	solute carrier family 16 (monocarboxylic acid transporters), member 1	protein_coding	[illegible]
20502	NM_009197,XM_030251272	Slc16a2	solute carrier family 16 (monocarboxylic acid transporters), member 2	protein_coding	[illegible]
20503	NM_001358496,NM_001358915,NM_011391,XM_006513406,XM_006513408,XM_006513409,XM_006513410,XM_006513413,XM_017313861,XM_030244968,XM_030244969,XM_030244970	Slc16a7	solute carrier family 16 (monocarboxylic acid transporters), member 7	protein_coding	[illegible]
20504	NM_001170638,NM_009198,NR_121611,XM_006516616,XM_006516617	Slc17a1	solute carrier family 17 (sodium phosphate), member 1	protein_coding	[illegible]
20505	NM_011392,XM_006517177,XM_006517178	Slc34a1	solute carrier family 34 (sodium phosphate), member 1	protein_coding	[illegible]
20508	NM_021712	Slc18a3	solute carrier family 18 (vesicular monoamine), member 3	protein_coding	[illegible]
20509	NM_001199271,NM_031196,XM_006513414,XM_006513415,XM_006513416,XM_006513417,XM_006513419,XM_030244971,XR_003948631,XR_003948632,XR_003948633,XR_003948634,XR_003948635,XR_003948636	Slc19a1	solute carrier family 19 (folate transporter), member 1	protein_coding	[illegible]
20510	NM_009199	Slc1a1	solute carrier family 1 (neuronal/epithelial high affinity glutamate transporter, system Xag), member 1	protein_coding	[illegible]
20511	NM_001077514,NM_001077515,NM_001361018,NM_011393,XM_006499047,XM_011239398,XM_030248893,XM_030248894	Slc1a2	solute carrier family 1 (glial high affinity glutamate transporter), member 2	protein_coding	[illegible]
20512	NM_148938	Slc1a3	solute carrier family 1 (glial high affinity glutamate transporter), member	protein_coding	[illegible]
20513	NM_009200,XM_006513422,XM_006513424,XM_030244972	Slc1a6	solute carrier family 1 (high affinity aspartate/glutamate transporter), member 6	protein_coding	[illegible]
20514	NM_009201,XM_006539695,XM_030242273,XR_391052	Slc1a5	solute carrier family 1 (neutral amino acid transporter), member 5	protein_coding	[illegible]
20515	NM_001159593,NM_001355252,NM_015747,XM_011239399	Slc20a1	solute carrier family 20, member 1	protein_coding	[illegible]
20516	NM_011394,XM_006509043,XM_006509044,XM_030243366,XM_030243367	Slc20a2	solute carrier family 20, member 2	protein_coding	[illegible]
20517	NM_009202	Slc22a1	solute carrier family 22 (organic cation transporter), member 1	protein_coding	[illegible]
20518	NM_001355767,NM_013667	Slc22a2	solute carrier family 22 (organic cation transporter), member 2	protein_coding	[illegible]
20519	NM_011395	Slc22a3	solute carrier family 22 (organic cation transporter), member 3	protein_coding	[illegible]
20520	NM_001362711,NM_001362712,NM_011396,XM_006532650,XM_006532651,XR_001779934	Slc22a5	solute carrier family 22 (organic cation transporter), member 5	protein_coding	[illegible]
20522	NM_011397,XM_017317851,XR_003952504	Slc23a1	solute carrier family 23 (nucleobase transporters), member 1	protein_coding	[illegible]
20523	NM_001166450,NM_001290703,NM_001290704,NM_001290705,NM_001359136,NM_011398,XM_006541446,XM_030251273	Slc25a14	solute carrier family 25 (mitochondrial carrier, brain), member 14	protein_coding	[illegible]
20524	NM_011399	Slc25a17	solute carrier family 25 (mitochondrial carrier, peroxisomal membrane protein), member 17	protein_coding	[illegible]
20525	NM_011400,XM_006502908	Slc2a1	solute carrier family 2 (facilitated glucose transporter), member 1	protein_coding	[illegible]
20526	NM_031197	Slc2a2	solute carrier family 2 (facilitated glucose transporter), member 2	protein_coding	[illegible]
20527	NM_011401,XM_006505822,XM_006505823,XM_011241268,XM_030255284,XM_030255285	Slc2a3	solute carrier family 2 (facilitated glucose transporter), member 3	protein_coding	[illegible]
20528	NM_001359114,NM_009204,XM_006532654,XM_006532655,XM_030245725	Slc2a4	solute carrier family 2 (facilitated glucose transporter), member 4	protein_coding	[illegible]
20529	NM_175090,XM_006537711	Slc31a1	solute carrier family 31, member 1	protein_coding	[illegible]
20530	NM_001290518,NM_025286,XM_006537712	Slc31a2	solute carrier family 31, member 2	protein_coding	[illegible]
20531	NM_011402,XM_006503807	Slc34a2	solute carrier family 34 (sodium phosphate), member 2	protein_coding	[illegible]
20532	NM_009205	Slc3a1	solute carrier family 3, member 1	protein_coding	[illegible]
20533	NM_011403	Slc4a1	solute carrier family 4 (anion exchanger), member 1	protein_coding	[illegible]
20534	NM_001347328,NM_001359154,NM_001359156,NM_001359157,NM_009206,XM_006503810,XM_017320752	Slc4a1ap	solute carrier family 4 (anion exchanger), member 1, adaptor protein	protein_coding	[illegible]
20535	NM_001253892,NM_009207,XM_006535650,XM_006535651,XM_006535653,XM_006535655,XM_006535656	Slc4a2	solute carrier family 4 (anion exchanger), member 2	protein_coding	[illegible]
20536	NM_001357149,NM_001357150,NM_009208,XM_006496450,XM_006496451,XM_006496453,XM_006496454,XM_006496455,XM_006496456,XM_006496457,XM_006496458,XM_011238679,XM_030252387,XM_030252388	Slc4a3	solute carrier family 4 (anion exchanger), member 3	protein_coding	[illegible]
20537	NM_019810	Slc5a1	solute carrier family 5 (sodium/glucose cotransporter), member 1	protein_coding	[illegible]
20538	NM_009209,XM_006530793,XM_006530794	Slc6a2	solute carrier family 6 (neurotransmitter transporter, noradrenalin),	protein_coding	[illegible]
20539	NM_011404	Slc7a5	solute carrier family 7 (cationic amino acid transporter, y+ system), member 5	protein_coding	[illegible]
20540	NM_001253679,NM_001253680,NM_011405,XM_006518746,XM_006518748,XM_006518751,XM_006518752,XM_006518753,XM_006518754,XM_006518755,XM_030247702,XM_030247703,XM_030247704,XM_030247705	Slc7a7	solute carrier family 7 (cationic amino acid transporter, y+ system), member 7	protein_coding	[illegible]
20541	NM_001112798,NM_001286684,NM_011406,NR_104580,XM_006523932,XM_006523933,XM_006523934,XM_006523935,XM_006523936,XM_006523937,XM_006523938,XM_006523939,XM_006523940,XM_006523941,XM_006523942,XM_006523943,XM_006523944,XM_017317368,XM_017317369,XM_017317370,XM_017317371	Slc8a1	solute carrier family 8 (sodium/calcium exchanger), member 1	protein_coding	[illegible]
20544	NM_001358455,NM_016981,XM_006538686,XM_017320089	Slc9a1	solute carrier family 9 (sodium/hydrogen exchanger), member 1	protein_coding	[illegible]
20555	NM_011407	Slfn1	schlafen 1	protein_coding	[illegible]
20556	NM_011408	Slfn2	schlafen 2	protein_coding	[illegible]
20557	NM_011409	Slfn3	schlafen 3	protein_coding	[illegible]
20558	NM_001302559,NM_011410,XM_006532659,XM_006532660,XM_006532661,XM_006532663,XM_030245726,XM_030245727,XM_030245728,XM_030245729	Slfn4	schlafen 4	protein_coding	[illegible]
20562	NM_015748,XM_030250829,XM_030250830	Slit1	slit guidance ligand 1	protein_coding	[illegible]
20563	NM_001291227,NM_001291228,NM_178804,NR_111900,XM_006503813,XM_006503814,XM_006503815,XM_006503816	Slit2	slit guidance ligand 2	protein_coding	[illegible]
20564	NM_011412	Slit3	slit guidance ligand 3	protein_coding	[illegible]
20568	NM_011414	Slpi	secretory leukocyte peptidase inhibitor	protein_coding	[illegible]
20583	NM_011415	Snai2	snail family zinc finger 2	protein_coding	[illegible]
20585	NM_001355097,NM_009210,NR_105046,NR_105047,XM_006535429,XR_001783672,XR_001783673,XR_001783674,XR_001783675	Hltf	helicase-like transcription factor	protein_coding	[illegible]
20586	NM_001174078,NM_001174079,NM_001357764,NM_011417,XM_006510117,XM_006510119,XM_006510120,XM_006510122,XM_011242434,XM_011242435,XM_011242436,XM_011242437,XM_011242438,XM_030244151,XM_030244152,XM_030244153	Smarca4	SWI/SNF related, matrix associated, actin dependent regulator of chromatin, subfamily a, member 4	protein_coding	[illegible]
20587	NM_001161853,NM_011418	Smarcb1	SWI/SNF related, matrix associated, actin dependent regulator of chromatin, subfamily b, member 1	protein_coding	[illegible]
20588	NM_009211	Smarcc1	SWI/SNF related, matrix associated, actin dependent regulator of chromatin, subfamily c, member 1	protein_coding	[illegible]
20589	NM_009212,XM_006531702,XR_001782563,XR_001782564,XR_003952763	Ighmbp2	immunoglobulin mu binding protein 2	protein_coding	[illegible]
20591	NM_013668,XM_006528769,XM_006528770,XM_006528771,XM_006528772,XM_006528773,XM_017318442,XM_017318443,XM_030251274,XR_003953003	Kdm5c	lysine (K)-specific demethylase 5C	protein_coding	[illegible]
20592	NM_011419,XM_006531578,XM_011248580,XM_017318684,XM_030251562,XR_003953250	Kdm5d	lysine (K)-specific demethylase 5D	protein_coding	[illegible]
20595	NM_001252629,NM_011420,XM_011244637	Smn1	survival motor neuron 1	protein_coding	[illegible]
20597	NM_011421	Smpd1	sphingomyelin phosphodiesterase 1, acid lysosomal	protein_coding	[illegible]
20598	NM_009213,XM_017313862,XM_030244973	Smpd2	sphingomyelin phosphodiesterase 2, neutral	protein_coding	[illegible]
20599	NM_011422,XM_017320753	Smr3a	submaxillary gland androgen regulated protein 3A	protein_coding	[illegible]
20602	NM_001253904,NM_001253905,NM_011424,XM_006504282,XM_006504283,XM_011240820,XM_011240821,XM_011240825,XM_011240828,XM_011240831,XM_011240833,XM_011240834,XM_017320755,XM_017320757,XM_030254269,XM_030254270,XM_030254271,XM_030254272,XM_030254273,XM_030254274,XM_030254275,XM_030254276,XM_030254277,XM_030254278,XM_030254279,XM_030254280,XM_030254281,XM_030254283,XM_030254284,XM_030254285,XM_030254286,XM_030254287,XM_030254288,XM_030254289,XM_030254290,XM_030254291,XM_030254292,XM_030254294,XM_030254295,XM_030254296,XM_030254297,XM_030254298,XM_030254300,XM_030254301,XM_030254302,XM_030254303,XM_030254304,XR_003955604,XR_003955605,XR_003955606,	Ncor2	nuclear receptor co-repressor 2	protein_coding	[illegible]
20603	NM_001359185,NM_009214	Sms	spermine synthase	protein_coding	[illegible]
20604	NM_009215,XM_030249009	Sst	somatostatin	protein_coding	[illegible]
20605	NM_009216,XM_006515634	Sstr1	somatostatin receptor 1	protein_coding	[illegible]
20606	NM_001042606,NM_009217	Sstr2	somatostatin receptor 2	protein_coding	[illegible]
20607	NM_001356961,NM_009218,XM_006520670,XM_011245531	Sstr3	somatostatin receptor 3	protein_coding	[illegible]
20608	NM_009219	Sstr4	somatostatin receptor 4	protein_coding	[illegible]
20609	NM_001191008,NM_011425	Sstr5	somatostatin receptor 5	protein_coding	[illegible]
20610	NM_001301671,NM_001301672,NM_001301673,NM_019929,NR_125903,NR_125904	Sumo3	small ubiquitin-like modifier 3	protein_coding	[illegible]
20612	NM_011426,XM_006499049,XM_006499050,XM_006499051,XM_030248900	Siglec1	sialic acid binding Ig-like lectin 1, sialoadhesin	protein_coding	[illegible]
20613	NM_011427	Snai1	snail family zinc finger 1	protein_coding	[illegible]
20614	NM_001291056,NM_001355254,NM_001355255,NM_011428,NR_149318,XM_017316760,XM_017316761	Snap25	synaptosomal-associated protein 25	protein_coding	[illegible]
20615	NM_133854	Snapin	SNAP-associated protein	protein_coding	[illegible]
20616	NM_001277982,NM_001277983,NM_001277985,NM_001277986,NM_001357768,NM_001357770,NM_001357772,NM_013669,XM_006510895,XM_006510896,XM_006510897,XM_006510898,XM_006510899,XM_006510900,XM_006510901,XM_006510902,XM_006510903,XM_006510904,XM_017313237,XM_030244155,XM_030244156,XM_030244157,XM_030244158,XM_030244159	Snap91	synaptosomal-associated protein 91	protein_coding	[illegible]
20617	NM_001042451,NM_009221	Snca	synuclein, alpha	protein_coding	[illegible]
20618	NM_011430	Sncg	synuclein, gamma	protein_coding	[illegible]
20619	NM_001177792,NM_001177793,NM_009222,XM_006499054,XM_006499055,XM_006499056,XM_006499057	Snap23	synaptosomal-associated protein 23	protein_coding	[illegible]
20620	NM_152804	Plk2	polo like kinase 2	protein_coding	[illegible]
20621	NM_009223	Snn	stannin	protein_coding	[illegible]
20623	NM_001164572,NM_001357774,NM_001357775,NM_001357776,NM_133741,XM_006511999,XM_006512000,XM_006512002	Snrk	SNF related kinase	protein_coding	[illegible]
20624	NM_001109995,NM_011431,XM_006532665	Eftud2	elongation factor Tu GTP binding domain containing 2	protein_coding	[illegible]
20630	NM_011432,XM_030249614	Snrpc	U1 small nuclear ribonucleoprotein C	protein_coding	[illegible]
20637	NM_009224,NR_126538,NR_126539,XM_006540732,XM_011250831,XR_001785499,XR_003946425,XR_003946426	Snrnp70	small nuclear ribonucleoprotein 70 (U1)	protein_coding	[illegible]
20638	NM_009225,XM_006499059	Snrpb	small nuclear ribonucleoprotein B	protein_coding	[illegible]
20639	NM_021335	Snrpb2	U2 small nuclear ribonucleoprotein B	protein_coding	[illegible]
20641	NM_009226	Snrpd1	small nuclear ribonucleoprotein D1	protein_coding	[illegible]

20643	NM_009227	Snrpe	small nuclear ribonucleoprotein E	protein_coding	[illegible]
20646	NM_001082961,NM_001082962,NM_001349690,NM_001349691,NM_001349692,NM_001349693,NM_001349694,NM_001349695,NM_013670	Snrpn	small nuclear ribonucleoprotein N	protein_coding	[illegible]
20648	NM_009228,XM_030248934	Snta1	syntrophin, acidic 1	protein_coding	[illegible]
20649	NM_016667,XM_006520671	Sntb1	syntrophin, basic 1	protein_coding	[illegible]
20650	NM_001368316,NM_009229	Sntb2	syntrophin, basic 2	protein_coding	[illegible]
20652	NM_009230,XM_006496719,XM_011238778	Soat1	sterol O-acyltransferase 1	protein_coding	[illegible]
20655	NM_011434	Sod1	superoxide dismutase 1, soluble	protein_coding	[illegible]
20656	NM_013671	Sod2	superoxide dismutase 2, mitochondrial	protein_coding	[illegible]
20657	NM_011435	Sod3	superoxide dismutase 3, extracellular	protein_coding	[illegible]
20658	NM_019973,NM_178880	Son	Son DNA binding protein	protein_coding	[illegible]
20660	NM_001357261,NM_011436	Sorl1	sortilin-related receptor, LDLR class A repeats-containing	protein_coding	[illegible]
20661	NM_001271599,NM_019972,XM_006501223,XM_006501224,XM_030252513,XM_030252515	Sort1	sortilin 1	protein_coding	[illegible]
20662	NM_009231	Sos1	SOS Ras/Rac guanine nucleotide exchange factor 1	protein_coding	[illegible]
20663	NM_001135559,XM_006515635	Sos2	SOS Ras/Rho guanine nucleotide exchange factor 2	protein_coding	[illegible]
20664	NM_009233	Sox1	SRY (sex determining region Y)-box 1	protein_coding	[illegible]
20665	NM_011437	Sox10	SRY (sex determining region Y)-box 10	protein_coding	[illegible]
20666	NM_009234	Sox11	SRY (sex determining region Y)-box 11	protein_coding	[illegible]
20667	NM_011438	Sox12	SRY (sex determining region Y)-box 12	protein_coding	[illegible]
20668	NM_011439,XM_006529276,XM_006529277,XM_011247945,XM_030252397	Sox13	SRY (sex determining region Y)-box 13	protein_coding	[illegible]
20670	NM_009235	Sox15	SRY (sex determining region Y)-box 15	protein_coding	[illegible]
20671	NM_001289464,NM_001289465,NM_001289466,NM_001289467,NM_011441,XM_006495474,XM_030252405,XM_030252406,XM_030252410	Sox17	SRY (sex determining region Y)-box 17	protein_coding	[illegible]
20672	NM_009236	Sox18	SRY (sex determining region Y)-box 18	protein_coding	[illegible]
20674	NM_011443	Sox2	SRY (sex determining region Y)-box 2	protein_coding	[illegible]
20675	NM_009237	Sox3	SRY (sex determining region Y)-box 3	protein_coding	[illegible]
20677	NM_009238	Sox4	SRY (sex determining region Y)-box 4	protein_coding	[illegible]
20678	NM_001113559,NM_001243163,NM_001347506,NM_011444,XM_006506931,XM_006506932,XM_006506933,XM_006506934,XM_006506935,XM_006506936,XM_006506937,XM_006506938,XM_006506939,XM_006506940,XM_006506941,XM_006506942,XM_006506944,XM_006506945,XM_011241577,XM_011241578,XM_011241579,XM_011241580,XM_011241581,XM_011241582,XM_017321475,XM_030255287,XM_030255289,XM_030255290	Sox5	SRY (sex determining region Y)-box 5	protein_coding	[illegible]
20679	NM_001025559,NM_001025560,NM_001277326,NM_001277327,NM_001277328,NM_011445,XM_006507495,XM_006507496,XM_006507497,XM_006507498,XM_006507499,XM_006507501,XM_006507504,XM_006507514,XM_011241707,XM_011241709,XM_011241713,XM_011241715,XM_017322068,XM_017322070,XM_017322071,XM_030242274,XM_030242275,XM_030242276,XM_030242277,XM_030242278,XM_030242279,XM_030242280,XM_030242281,XM_030242282,XM_030242283,XM_030242284,XM_030242285,XM_030242286,XM_030242287,XM_030242288,XM_030242289,XM_030242290,XM_030242291,XM_030242292,XM_030242293,XM_030242294,XM_030242295,XM_030242296,XM_030242297,XM_030242298,XM_030242299,XM_030242300,XM_030242301,XM_030242302,XM_030242303	Sox6	SRY (sex determining region Y)-box 6	protein_coding	[illegible]
20680	NM_011446	Sox7	SRY (sex determining region Y)-box 7	protein_coding	[illegible]
20681	NM_011447	Sox8	SRY (sex determining region Y)-box 8	protein_coding	[illegible]
20682	NM_011448	Sox9	SRY (sex determining region Y)-box 9	protein_coding	[illegible]
20683	NM_013672,XM_017316523	Sp1	trans-acting transcription factor 1	protein_coding	[illegible]
20684	NM_001313712,NM_001313713,NM_001313714,NM_013673,XM_006529280,XM_006529281,XM_006529282,XM_006529283,XM_006529284,XM_006529285,XM_006529286,XM_006529288,XM_011247950,XM_017319442,XM_017319443,XM_030252417,XM_030252422,XM_030252430	Sp100	nuclear antigen Sp100	protein_coding	[illegible]
20686	NM_001324545,NM_011449,XM_011242439	Spa17	sperm autoantigenic protein 17	protein_coding	[illegible]
20687	NM_001018042,NM_001098425	Sp3	trans-acting transcription factor 3	protein_coding	[illegible]
20688	NM_001166385,NM_009239,XM_017315007	Sp4	trans-acting transcription factor 4	protein_coding	[illegible]
20689	NM_001369133,NM_178280,XM_030250368	Sall3	spalt like transcription factor 3	protein_coding	[illegible]
20692	NM_001290817,NM_009242,XM_030245730	Sparc	secreted acidic cysteine rich glycoprotein	protein_coding	[illegible]
20698	NM_001172472,NM_001172473,NM_001172475,NM_011451,NM_025367,XM_006532669,XM_006532670,XM_006532671,XM_006532672,XM_006532673,XM_006532674,XM_017314398,XM_030245731,XM_030245732	Sphk1	sphingosine kinase 1	protein_coding	[illegible]
20700	NM_001252569,NM_009243	Serpina1a	serine (or cysteine) peptidase inhibitor, clade A, member 1A	protein_coding	[illegible]
20701	NM_009244	Serpina1b	serine (or cysteine) preptidase inhibitor, clade A, member 1B	protein_coding	[illegible]
20702	NM_009245	Serpina1c	serine (or cysteine) peptidase inhibitor, clade A, member 1C	protein_coding	[illegible]
20703	NM_009246	Serpina1d	serine (or cysteine) peptidase inhibitor, clade A, member 1D	protein_coding	[illegible]
20704	NM_009247	Serpina1e	serine (or cysteine) peptidase inhibitor, clade A, member 1E	protein_coding	[illegible]
20706	NM_011452	Serpinb9b	serine (or cysteine) peptidase inhibitor, clade B, member 9b	protein_coding	[illegible]
20708	NM_011454,XM_006516621,XM_006516622	Serpinb6b	serine (or cysteine) peptidase inhibitor, clade B, member 6b	protein_coding	[illegible]
20713	NM_001357514,NM_009250	Serpini1	serine (or cysteine) peptidase inhibitor, clade I, member 1	protein_coding	[illegible]
20714	NM_011458	Serpina3k	serine (or cysteine) peptidase inhibitor, clade A, member 3K	protein_coding	[illegible]
20715	NM_001353612,NM_001353613	Serpina3g	serine (or cysteine) peptidase inhibitor, clade A, member 3G	protein_coding	[illegible]
20716	NM_009252,XM_011244046	Serpina3n	serine (or cysteine) peptidase inhibitor, clade A, member 3N	protein_coding	[illegible]
20717	NM_009253,XM_006515637	Serpina3m	serine (or cysteine) peptidase inhibitor, clade A, member 3M	protein_coding	[illegible]
20719	NM_001164117,NM_001164118,NM_001243192,NM_009254,XM_011244299,XM_011244300,XM_011244301,XM_030247219	Serpinb6a	serine (or cysteine) peptidase inhibitor, clade B, member 6a	protein_coding	[illegible]
20720	NM_009255,XM_006496459	Serpine2	serine (or cysteine) peptidase inhibitor, clade E, member 2	protein_coding	[illegible]
20723	NM_009256,XM_006516625,XM_006516626,XM_006516628,XM_006516629,XM_017315468	Serpinb9	serine (or cysteine) peptidase inhibitor, clade B, member 9	protein_coding	[illegible]
20724	NM_001360853,NM_001360854,NM_009257	Serpinb5	serine (or cysteine) peptidase inhibitor, clade B, member 5	protein_coding	[illegible]
20725	NM_001159748,NM_011459,XM_006529292,XM_006529293	Serpinb8	serine (or cysteine) peptidase inhibitor, clade B, member 8	protein_coding	[illegible]
20728	NM_011461	Spic	Spi-C transcription factor (Spi-1/PU.1 related)	protein_coding	[illegible]
20729	NM_001283028,NM_001283029,NM_001283030,NM_011462,NM_146043,XM_006516893,XM_006516894,XM_017315469	Spin1	spindlin 1	protein_coding	[illegible]
20732	NM_016907,XM_006499062,XM_030248958	Spint1	serine protease inhibitor, Kunitz type 1	protein_coding	[illegible]
20733	NM_001082548,NM_011464,XM_017322072	Spint2	serine protease inhibitor, Kunitz type 2	protein_coding	[illegible]
20737	NM_001037810,NM_009259,NR_110337,XM_006507522,XM_011241716,XM_011241717,XR_001785500	Spn	sialophorin	protein_coding	[illegible]
20739	NM_011465,XR_001783648,XR_373600	Spta1	spectrin alpha, erythrocytic 1	protein_coding	[illegible]
20740	NM_001177667,NM_001177668,NM_001309460,NM_001369325,XM_006497809,XM_006497810,XM_006497812,XM_006497813,XM_006497814,XM_006497815,XM_006497816	Sptan1	spectrin alpha, non-erythrocytic 1	protein_coding	[illegible]
20741	NM_013675	Sptb	spectrin beta, erythrocytic	protein_coding	[illegible]
20742	NM_009260,NM_175836,XM_006514599,XM_006514600,XM_006514601,XM_030245736,XM_030245737,XM_030245738	Sptbn1	spectrin beta, non-erythrocytic 1	protein_coding	[illegible]
20743	NM_021287,XM_006531703,XM_006531704,XM_006531705	Sptbn2	spectrin beta, non-erythrocytic 2	protein_coding	[illegible]
20744	NM_001355259,NM_001355260,NM_009261,XM_011239043,XM_011239044,XM_011239045,XM_011239046,XM_011239047,XM_017316819,XM_017316820,XM_017316823,XM_030248982,XM_030248987,XM_030248990,XR_001781603,XR_001781604,XR_003951764,XR_003951765,XR_866009,XR_866010,XR_866011	Strbp	spermatid perinuclear RNA binding protein	protein_coding	[illegible]
20745	NM_001166463,NM_001166464,NM_001166465,NM_001166466,NM_009262	Spock1	sparc/osteonectin, cwcv and kazal-like domains proteoglycan 1	protein_coding	[illegible]
20747	NM_001359107,NM_025287	Spop	speckle-type BTB/POZ protein	protein_coding	[illegible]
20750	NM_001204201,NM_001204202,NM_001204203,NM_001204233,NM_009263	Spp1	secreted phosphoprotein 1	protein_coding	[illegible]
20751	NM_011467	Spr	sepiapterin reductase	protein_coding	[illegible]
20753	NM_009264	Sprr1a	small proline-rich protein 1A	protein_coding	[illegible]
20768	NM_009266	Sephs2	selenophosphate synthetase 2	protein_coding	[illegible]
20773	NM_011479	Sptlc2	serine palmitoyltransferase, long chain base subunit 2	protein_coding	[illegible]
20775	NM_009270	Sqle	squalene epoxidase	protein_coding	[illegible]
20776	NM_146260,XM_006512007,XM_006512008,XM_006512014,XM_030244163	Tmie	transmembrane inner ear	protein_coding	[illegible]
20778	NM_001205082,NM_001205083,NM_016741,XM_017320764,XM_030254308,XM_030254309,XM_030254310	Scarb1	scavenger receptor class B, member 1	protein_coding	[illegible]
20779	NM_001025395,NM_009271,XM_006499063,XM_006499065,XM_006499066,XM_011239402,XM_011239403,XM_011239404,XM_030248999,XM_030249001,XM_030249002,XM_030249004,XM_030249007,XM_030249008,XM_030249010	Src	Rous sarcoma oncogene	protein_coding	[illegible]
20787	NM_001313979,NM_001358314,NM_001358315,NM_011480,XM_006532716,XM_030245747,XM_030245748	Srebf1	sterol regulatory element binding transcription factor 1	protein_coding	[illegible]
20788	NM_033218,XM_006520685	Srebf2	sterol regulatory element binding factor 2	protein_coding	[illegible]
20807	NM_020493,XM_006523957,XM_011246336	Srf	serum response factor	protein_coding	[illegible]
20810	NM_009272	Srm	spermidine synthase	protein_coding	[illegible]
20811	NM_011481,XM_017316827	Srms	src-related kinase lacking C-terminal regulatory tyrosine and N-terminal myristylation sites	protein_coding	[illegible]
20813	NM_009273	Srp14	signal recognition particle 14	protein_coding	[illegible]
20815	NM_016795,XM_006523958,XM_011246337,XR_385308	Srpk1	serine/arginine-rich protein specific kinase 1	protein_coding	[illegible]
20817	NM_001359172,NM_001359173,NM_001359174,NM_001359175,NM_001359176,NM_009274,NR_152854,XM_006535660,XM_011249751,XM_011249752,XM_011249754,XM_011249755,XM_011249756,XM_011249757,XM_011249758,XM_011249759,XM_017320767,XM_017320768,XM_017320770,XM_017320772,XM_017320773,XM_017320774,XM_017320775,XM_017320776,XM_017320777,XM_030254312,XM_030254313,XM_030254314,XM_030254315,XM_030254316,XM_030254317,XM_030254318,XM_030254319,XM_030254320,XM_030254321,XM_030254322,XM_030254323,XM_030254324,XM_030254325,XM_030254326,XM_030254327,XM_030254328	Srpk2	serine/arginine-rich protein specific kinase 2	protein_coding	[illegible]
20818	NM_009275	Srprb	signal recognition particle receptor, B subunit	protein_coding	[illegible]
20821	NM_001082552,NM_009277	Trim21	tripartite motif-containing 21	protein_coding	[illegible]
20822	NM_013835,XM_017319451	Ro60	Ro60, Y RNA binding protein	protein_coding	[illegible]
20823	NM_001110145,NM_001355265,NM_009278,NR_149321	Ssb	Sjogren syndrome antigen B	protein_coding	[illegible]
20826	NM_011482	Snu13	SNU13 homolog, small nuclear ribonucleoprotein (U4/U6.U5)	protein_coding	[illegible]
20832	NM_001166480,NM_001358212,NM_001358213,NM_009279	Ssr4	signal sequence receptor, delta	protein_coding	[illegible]
20833	NM_001136081,NM_182990,XM_006499070,XM_011239405	Ssrp1	structure specific recognition protein 1	protein_coding	[illegible]
20840	NM_001324546,NM_016853,XM_006512016	Stac	src homology three (SH3) and cysteine rich domain	protein_coding	[illegible]
20841	NM_009281,XM_006507530,XM_006507531,XM_006507532	Zfp143	zinc finger protein 143	protein_coding	[illegible]
20842	NM_001357264,NM_001357265,NM_009282,XM_006510923,XM_006510924,XM_006510925,XM_011242692,XM_030244172,XM_030244173	Stag1	stromal antigen 1	protein_coding	[illegible]
20843	NM_001077712,NM_001290713,NM_001358225,NM_021465,XM_011250984,XM_011250985,XM_011250987,XM_011250988,XM_011250989,XM_030251276,XM_030251277,XM_030251278,XM_030251279	Stag2	stromal antigen 2	protein_coding	[illegible]
20844	NM_001356337,NM_011484,XR_373935	Stam	signal transducing adaptor molecule (SH3 domain and ITAM motif) 1	protein_coding	[illegible]
20845	NM_011485	Star	steroidogenic acute regulatory protein	protein_coding	[illegible]
20846	NM_001205313,NM_001205314,NM_001357627,NM_009283,XM_017319463,XM_017319464,XM_030252467,XR_003954297,XR_003954298	Stat1	signal transducer and activator of transcription 1	protein_coding	[illegible]
20847	NM_019963	Stat2	signal transducer and activator of transcription 2	protein_coding	[illegible]
20848	NM_011486,NM_213659,NM_213660,XM_011248846	Stat3	signal transducer and activator of transcription 3	protein_coding	[illegible]
20849	NM_001308266,NM_011487,XM_006495814,XM_006495815,XM_006495816	Stat4	signal transducer and activator of transcription 4	protein_coding	[illegible]
20850	NM_001164062,NM_001362680,NM_011488,XM_006532721,XM_017314402	Stat5a	signal transducer and activator of transcription 5A	protein_coding	[illegible]
20851	NM_001113563,NM_001362682,NM_011489,XM_006532723,XM_030245749	Stat5b	signal transducer and activator of transcription 5B	protein_coding	[illegible]
20852	NM_009284,XM_030244975	Stat6	signal transducer and activator of transcription 6	protein_coding	[illegible]
20853	NM_001109905,NM_001109906,NM_001291160,NM_011490,NR_111895,XM_006499071,XM_006499072,XM_011239406,XM_030249042	Stau1	staufen double-stranded RNA binding protein 1	protein_coding	[illegible]
20855	NM_009285	Stc1	stanniocalcin 1	protein_coding	[illegible]
20856	NM_011491	Stc2	stanniocalcin 2	protein_coding	[illegible]
20861	NM_001082543	Stfa1	stefin A1	protein_coding	[illegible]
20862	NM_001082545	Stfa2	stefin A2	protein_coding	[illegible]
20863	NM_025288	Stfa3	stefin A3	protein_coding	[illegible]
20866	NM_009287,XM_006507533,XM_006507534,XM_006507535,XM_006507536,XM_006507537,XM_017322078	Stim1	stromal interaction molecule 1	protein_coding	[illegible]
20867	NM_016737	Stip1	stress-induced phosphoprotein 1	protein_coding	[illegible]
20868	NM_001359177,NM_001359178,NM_009288	Stk10	serine/threonine kinase 10	protein_coding	[illegible]
20869	NM_001301853,NM_001301854,NM_011492,NR_126043,XM_006513439,XM_006513443,XR_003948637,XR_003948638,XR_003948	Stk11	serine/threonine kinase 11	protein_coding	[illegible]
20871	NM_001080965,NM_001080966,NM_020572,XM_006539697,XM_006539701,XM_017322079	Aurkc	aurora kinase C	protein_coding	[illegible]
20872	NM_001277992,NM_011494,NR_102731,XM_006496461,XM_006496462,XM_030252514,XM_030252516	Stk16	serine/threonine kinase 16	protein_coding	[illegible]
20873	NM_011495,NM_173169,NR_045576,XM_006500768,XM_006500770,XM_011239968,XM_030252518	Plk4	polo like kinase 4	protein_coding	[illegible]
20874	NM_001164639,NM_009289,XM_006526827	Slk	STE20-like kinase	protein_coding	[illegible]
20877	NM_011496,XM_006532725	Aurkb	aurora kinase B	protein_coding	[illegible]
20878	NM_001291185,NM_011497,XM_006499075	Aurka	aurora kinase A	protein_coding	[illegible]
20887	NM_133670,XM_006507538,XM_017322084	Sult1a1	sulfotransferase family 1A, phenol-preferring, member 1	protein_coding	[illegible]
20890	NM_009290	Wnt8a	wingless-type MMTV integration site family, member 8A	protein_coding	[illegible]
20892	NM_001347611,NM_001347612,NM_016665,XM_006532729,XM_011248848,XM_011248849,XM_011248850,XM_011248851,XM_011248852,XM_011248853	Cenpx	centromere protein X	protein_coding	[illegible]
20893	NM_011498	Bhlhe40	basic helix-loop-helix family, member e40	protein_coding	[illegible]
20897	NM_001162475,NM_001162476,NM_001162479,NM_009291,XM_006510941,XM_006510943,XM_006510944,XM_006510945,XM_006510946,XM_017313262,XM_030244180	Stra6	stimulated by retinoic acid gene 6	protein_coding	[illegible]
20899	NM_009292,XM_006505829,XM_011241270	Stra8	stimulated by retinoic acid gene 8	protein_coding	[illegible]
20901	NM_011499	Strap	serine/threonine kinase receptor associated protein	protein_coding	[illegible]
20907	NM_001359179,NM_016801,NR_133683,NR_152855,XM_0065044	Stx1a	syntaxin 1A (brain)	protein_coding	[illegible]
20908	NM_001025307,NM_001286543,NM_001360386,NM_011502,NM_152220,NR_153433,NR_153434,XM_006526844,XR_001782567,XR_001782568,XR_001782569,XR_003952771,XR_003952772,XR_003952773,XR_003952774,XR_003952775	Stx3	syntaxin 3	protein_coding	[illegible]
20909	NM_009294,XM_006507539,XM_006507540,XM_006507541,XM_006507542,XM_006507543,XM_030242310,XM_030242311	Stx4a	syntaxin 4A (placental)	protein_coding	[illegible]
20910	NM_001113569,NM_009295	Stxbp1	syntaxin binding protein 1	protein_coding	[illegible]
20911	NM_001357168,NM_011503,XM_006508743,XM_006508746,XM_011242046,XM_017312632,XM_030243371,XM_030243373,XM_030243374,XM_030243375,XM_030243376,XM_030243377,XR_001778417,XR_001778418,XR_001778419	Stxbp2	syntaxin binding protein 2	protein_coding	[illegible]
20912	NM_011504	Stxbp3	syntaxin binding protein 3	protein_coding	[illegible]
20913	NM_011505,XM_006532736,XM_006532737,XM_006532738,XM_006532739,XM_006532740,XM_030245750,XR_388390	Stxbp4	syntaxin binding protein 4	protein_coding	[illegible]
20916	NM_001361638,NM_011506,XM_006518757	Sucla2	succinate-Coenzyme A ligase, ADP-forming, beta subunit	protein_coding	[illegible]
20917	NM_001326558,NM_011507,XM_030255295,XR_003956174	Suclg2	succinate-Coenzyme A ligase, GDP-forming, beta subunit	protein_coding	[illegible]
20918	NM_011508	Eif1	eukaryotic translation initiation factor 1	protein_coding	[illegible]
20922	NM_001355753,NM_009296,NR_149756,NR_149757	Supt4a	SPT4A, DSIF elongation factor subunit	protein_coding	[illegible]
20924	NM_013676,XM_006539702,XM_006539703	Supt5	suppressor of Ty 5, DSIF elongation factor subunit	protein_coding	[illegible]
20926	NM_009297,XM_006532743,XR_001779939	Supt6	SPT6, histone chaperone and transcription elongation factor	protein_coding	[illegible]
20927	NM_001357538,NM_011510,XM_006540749,XM_030242316,XR_001785502,XR_001785504,XR_001785505,XR_001785506,XR_001785511,XR_001785512,XR_003946427,XR_882027,XR_882028	Abcc8	ATP-binding cassette, sub-family C (CFTR/MRP), member 8	protein_coding	[illegible]
20928	NM_001044720,NM_001310143,NM_011511,NM_021041,NM_021042,XM_006506948,XM_006506949,XM_006506950,XM_006506951,XM_006506952	Abcc9	ATP-binding cassette, sub-family C (CFTR/MRP), member 9	protein_coding	[illegible]
20930	NM_001271724,NM_013677,NR_073420,XM_006497821,XM_006497823,XM_006497824,XM_006497825,XM_006497826,XM_030249117,XR_003951791,XR_003951793,XR_374066	Surf1	surfeit gene 1	protein_coding	[illegible]
20931	NM_013678,XM_006497827	Surf2	surfeit gene 2	protein_coding	[illegible]
20932	NM_011512,XM_006497828	Surf4	surfeit gene 4	protein_coding	[illegible]
20933	NM_001033908,NM_011513,XM_006497829	Med22	mediator complex subunit 22	protein_coding	[illegible]
20935	NM_009298	Surf6	surfeit gene 6	protein_coding	[illegible]
20937	NM_001290716,NM_001358237,NM_011514,XM_006527574,XM_011247452,XM_030251290,XR_003953005	Suv39h1	suppressor of variegation 3-9 1	protein_coding	[illegible]
20947	NM_009302,XM_017322086	Swap70	SWA-70 protein	protein_coding	[illegible]
20957	NM_011516,XM_006501229	Sycp1	synaptonemal complex protein 1	protein_coding	[illegible]
20962	NM_011517	Sycp3	synaptonemal complex protein 3	protein_coding	[illegible]
20963	NM_001198977,NM_011518,XM_006516895,XM_006516897,XM_017315470,XM_017315471	Syk	spleen tyrosine kinase	protein_coding	[illegible]
20964	NM_001110780,NM_013680	Syn1	synapsin I	protein_coding	[illegible]
20965	NM_001111015,NM_001326560,NM_013681	Syn2	synapsin II	protein_coding	[illegible]

20969	NM_011519,XM_006515045,XM_006515046	Sdc1	syndecan 1	protein_coding	NP_035649.1;XP_006515108.1;XP_006515109.1	1349162	.	39.502597	[illegible]
20970	NM_011520,XM_006538688,XM_017320090,XM_017320091	Sdc3	syndecan 3	protein_coding	NP_035650.2;XP_006538751.1;XP_017175579.1;XP_017175580.1	1349163	.	51.039325	[illegible]
20971	NM_011521	Sdc4	syndecan 4	protein_coding	NP_035651.1	1349164	.	49.192741	[illegible]
20972	NM_009303,NM_207708,XM_006520695,XM_030248440	Syngr1	synaptogyrin 1	protein_coding	NP_033329.1;NP_997591.1;XP_006520758.1;XP_030104300.1	1328323	.	0.681954	[illegible]
20973	NM_009304,XM_030245751	Syngr2	synaptogyrin 2	protein_coding	NP_033330.1;XP_030101611.1	1328324	.	28.530736	[illegible]
20974	NM_011522	Syngr3	synaptogyrin 3	protein_coding	NP_035652.2	1341881	.	0.046693	[illegible]
20975	NM_001113351,NM_001113352,NM_001113353,NM_001290698,NM_001357665,NM_001357667,NM_011523,NR_151772,XM_006523202,XM_006523203,XM_006523204,XM_006523205,XM_006523206,XM_006523208,XM_006523212,XM_017317372,XR_385128	Synj2	synaptojanin 2	protein_coding	NP_001106822.1;NP_001106823.1;NP_001106824.1;NP_001277627.1;NP_001344594.1;NP_001344596.1;NP_035653.2;XP_006523265.1;XP_006523266.1;XP_006523267.1;XP_006523268.1;XP_006523269.1;XP_006523271.1;XP_006523275.1;XP_017172861.1	1201671	.	5.650721	[illegible]
20977	NM_009305	Syp	synaptophysin	protein_coding	NP_033331.2	98467	.	0.304377	[illegible]
20979	NM_001252341,NM_001252342,NM_001358501,NM_001358502,NM_001358503,NM_001358504,NM_001358506,NM_009306,XM_006513447,XM_006513448,XM_030244984,XM_030244985,XM_030244986	Syt1	synaptotagmin I	protein_coding	NP_001239270.1;NP_001239271.1;NP_001345430.1;NP_001345431.1;NP_001345432.1;NP_001345433.1;NP_001345435.1;NP_033332.1;XP_006513510.1;XP_006513511.1;XP_030100844.1;XP_030100845.1;XP_030100846.1	99667	.	0.029087	[illegible]
20980	NM_001355726,NM_009307,XM_006529322,XM_006529324	Syt2	synaptotagmin II	protein_coding	NP_001342655.1;NP_033333.2;XP_006529385.1;XP_006529387.1	99666	.	0.117386	[illegible]
20981	NM_001114116,NM_016663,XM_006540755,XM_017322087,XM_017322088,XM_017322089,XM_030242317,XM_030242318,XM_030242319	Syt3	synaptotagmin III	protein_coding	NP_001107588.1;NP_057872.3;XP_006540818.1;XP_017177576.1;XP_017177577.1;XP_017177578.1;XP_030098177.1;XP_030098178.1;XP_030098179.1	99665	.	0.241737	[illegible]
20983	NM_009308,XM_017317852	Syt4	synaptotagmin IV	protein_coding	NP_033334.2;XP_017173341.1	101759	.	0.044011	[illegible]
20997	NM_009309	T	brachyury, T-box transcription factor T	protein_coding	NP_033335.1	98472	.	0.091479	[illegible]
21331	NM_001161832,XM_030249624,XM_030249625,XM_030249626,XM_030249627,XM_030249628,XM_030249629,XM_030249630,XM_030249631,XM_030249632,XM_030249633,XR_003952102	T2	brachyury 2	protein_coding	NP_001155304.1;XP_030105484.1;XP_030105485.1;XP_030105486.1;XP_030105487.1;XP_030105488.1;XP_030105489.1;XP_030105490.1;XP_030105491.1;XP_030105492.1;XP_030105493.1	104658	.	0.115431	[illegible]
21333	NM_001311060,NM_009311,XM_006505027	Tac1	tachykinin 1	protein_coding	NP_001297989.1;NP_033337.1;XP_006505090.1	98474	.	3.314217	[illegible]
21334	NM_001199971,NM_009312	Tac2	tachykinin 2	protein_coding	NP_001186900.1;NP_033338.2	98476	.	0.000000	[illegible]
21335	NM_001040435,NM_001310541,XM_006503832,XM_006503835,XM_011240715,XM_011240716,XM_017320784,XM_030254349,XM_030254350,XM_030254351,XM_030254352,XR_003955613,XR_003955614,XR_003955615,XR_868281	Tacc3	transforming, acidic coiled-coil containing protein 3	protein_coding	NP_001035525.1;NP_001297470.1;XP_006503895.1;XP_006503898.1;XP_011239017.1;XP_011239018.1;XP_017176273.1;XP_030110209.1;XP_030110210.1;XP_030110211.1;XP_030110212.1	1341163	.	16.263254	[illegible]
21336	NM_009313,XM_006505863,XM_006505864,XM_006505865	Tacr1	tachykinin receptor 1	protein_coding	NP_033339.2;XP_006505926.1;XP_006505927.1;XP_006505928.1	98475	.	0.046345	[illegible]
21337	NM_009314	Tacr2	tachykinin receptor 2	protein_coding	NP_033340.3	98477	.	0.000000	[illegible]
21338	NM_021382	Tacr3	tachykinin receptor 3	protein_coding	NP_067357.1	892968	.	0.012587	[illegible]
21339	NM_001277957,NM_001277958,NM_001277959,NM_021466	Taf1a	TATA-box binding protein associated factor, RNA polymerase I, A	protein_coding	NP_001264886.1;NP_001264887.1;NP_001264888.1;NP_067441.1	109578	.	6.576831	[illegible]
21340	NM_020614,NR_028341,XM_006515051	Taf1b	TATA-box binding protein associated factor, RNA polymerase I, B	protein_coding	NP_065639.2;XP_006515114.1	109577	.	6.367258	[illegible]
21341	NM_021441,XM_006530821,XM_006530822,XM_006530823	Taf1c	TATA-box binding protein associated factor, RNA polymerase I, C	protein_coding	NP_067416.2;XP_006530884.1;XP_006530885.1;XP_006530886.1	109576	.	4.442742	[illegible]
21343	NM_001356600,NM_001356601,NM_001356602,NM_009315,XM_011240936,XM_011240937,XM_011240938,XM_011240939,XM_011240940,XM_030254353,XM_030254354	Taf6	TATA-box binding protein associated factor 6	protein_coding	NP_001343529.1;NP_001343530.1;NP_001343531.1;NP_033341.1;XP_011239238.1;XP_011239239.1;XP_011239240.1;XP_011239241.1;XP_011239242.1;XP_030110213.1;XP_030110214.1	109129	.	13.546146	[illegible]
21345	NM_011526	Tagln	transgelin	protein_coding	NP_035656.1	106012	.	29.823950	[illegible]
21346	NM_178598	Tagln2	transgelin 2	protein_coding	NP_848713.1	1312985	.	202.604019	[illegible]
21349	NM_001287388,NM_011527,XM_006502909,XM_006502910,XM_006502912,XM_006502913,XM_006502914,XM_006502915,XM_006502916,XM_030253382	Tal1	T cell acute lymphocytic leukemia 1	protein_coding	NP_001274317.1;NP_035657.1;XP_006502972.1;XP_006502973.1;XP_006502975.1;XP_006502976.1;XP_006502977.1;XP_006502978.1;XP_006502979.1;XP_030109242.1	98480	.	1.482829	[illegible]
21350	NM_009317	Tal2	T cell acute lymphocytic leukemia 2	protein_coding	NP_033343.1	99540	.	0.260166	[illegible]
21351	NM_011528	Taldo1	transaldolase 1	protein_coding	NP_035658.1	1274789	.	36.114670	[illegible]
21353	NM_001164071,NM_001164072,NM_001355267,NM_001355268,NM_011529,NR_149323,NR_149324,XM_006499087,XM_006499089,XM_017316913,XM_030249258,XR_866264	Tank	TRAF family member-associated Nf-kappa B activator	protein_coding	NP_001157543.1;NP_001157544.1;NP_001342196.1;NP_001342197.1;NP_035659.1;XP_006499150.1;XP_006499152.1;XP_017172402.1;XP_030105118.1	107676	.	12.464130	[illegible]
21354	NM_001161730,NM_013683	Tap1	transporter 1, ATP-binding cassette, sub-family B (MDR/TAP)	protein_coding	NP_001155202.1;NP_038711.2	98483	.	2.930914	[illegible]
21355	NM_011530	Tap2	transporter 2, ATP-binding cassette, sub-family B (MDR/TAP)	protein_coding	NP_035660.3	98484	.	3.783491	[illegible]
21356	NM_001025313,NM_009318	Tapbp	TAP binding protein	protein_coding	NP_001020484.1;NP_033344.1	1201689	.	23.339738	[illegible]
21357	NM_001253795,NM_009319,XM_006520719,XM_006520720,XM_006520721,XM_006520723,XM_006520724,XM_030248453,XM_030248454	Tarbp2	TARBP2, RISC loading complex RNA binding subunit	protein_coding	NP_001240724.1;NP_033345.2;XP_006520782.1;XP_006520783.1;XP_006520784.1;XP_006520786.1;XP_006520787.1;XP_030104313.1;XP_030104314.1	103027	.	11.827985	[illegible]
21366	NM_009320,XM_006505870,XM_006505871,XM_030255303,XM_030255304,XR_003956177,XR_003956178,XR_003956179	Slc6a6	solute carrier family 6 (neurotransmitter transporter, taurine), member 6	protein_coding	NP_033346.2;XP_006505933.1;XP_006505934.1;XP_030111163.1;XP_030111164.1	98488	.	43.837584	[illegible]
21367	NM_177129,XM_006529362,XR_878318	Cntn2	contactin 2	protein_coding	NP_796103.2;XP_006529425.1	104518	.	0.206231	[illegible]
21371	NM_009321	Tbca	tubulin cofactor A	protein_coding	NP_033347.1	107549	.	52.543407	[illegible]
21372	NM_020601,XM_006527939,XM_030251299,XM_030251300	Tbl1x	transducin (beta)-like 1 X-linked	protein_coding	NP_065626.1;XP_006528002.1;XP_030107159.1;XP_030107160.1	1336172	.	21.426141	[illegible]
21374	NM_013684	Tbp	TATA box binding protein	protein_coding	NP_038712.3	101838	.	11.034539	[illegible]
21375	NM_009322,XM_006499098	Tbr1	T-box brain gene 1	protein_coding	NP_033348.2;XP_006499161.1	107404	.	0.141663	[illegible]
21376	NM_025289	Tbrg1	transforming growth factor beta regulated gene 1	protein_coding	NP_079565.2	1100877	.	117.879578	[illegible]
21378	NR_027799	Tbrg3	transforming growth factor beta regulated gene 3	lncRNA	.	1100876	.	0.017813	[illegible]
21379	NM_001130457,NM_001362713,NM_001362714,NM_001362715,NM_001362716,NM_134011,XM_006514607,XM_011243672,XR_001779944	Tbrg4	transforming growth factor beta regulated gene 4	protein_coding	NP_001123929.1;NP_001349642.1;NP_001349643.1;NP_001349644.1;NP_001349645.1;NP_598772.1;XP_006514670.1;XP_011241974.1	1100868	.	10.073685	[illegible]
21380	NM_001285472,NM_001285476,NM_011532,XM_006521978,XM_006521979,XM_017316925	Tbx1	T-box 1	protein_coding	NP_001272401.1;NP_001272405.1;NP_035662.1;XP_006522041.1;XP_006522042.1;XP_017172414.1	98493	.	0.130471	[illegible]
21384	NM_009323,XM_006501279,XM_006501280	Tbx15	T-box 15	protein_coding	NP_033349.2;XP_006501342.1;XP_006501343.1	1277234	.	12.993615	[illegible]
21385	NM_009324	Tbx2	T-box 2	protein_coding	NP_033350.2	98494	.	18.984821	[illegible]
21386	NM_011535,NM_198052,XM_006530276	Tbx3	T-box 3	protein_coding	NP_035665.2;NP_932169.1;XP_006530339.1	98495	.	0.186169	[illegible]
21387	NM_011536,NM_172798	Tbx4	T-box 4	protein_coding	NP_035666.2;NP_766386.1	102556	.	0.380053	[illegible]
21388	NM_011537,XM_006530279,XM_006530280,XM_006530281,XM_006530282,XM_006530283,XM_011248191	Tbx5	T-box 5	protein_coding	NP_035667.1;XP_006530342.1;XP_006530343.1;XP_006530344.1;XP_006530345.1;XP_006530346.1;XP_011246493.1	102541	.	0.000000	[illegible]
21389	NM_011538	Tbx6	T-box 6	protein_coding	NP_035668.2	102539	.	2.163132	[illegible]
21390	NM_001277265,NM_001358512,NM_009325	Tbxa2r	thromboxane A2 receptor	protein_coding	NP_001264194.1;NP_001345441.1;NP_033351.1	98496	.	1.196374	[illegible]
21391	NM_011539,XM_006505876,XM_006505877,XM_006505878,XM_006505879,XM_006505880,XM_006505881,XM_011241280,XM_017321499,XM_017321500	Tbxas1	thromboxane A synthase 1, platelet	protein_coding	NP_035669.3;XP_006505939.1;XP_006505940.1;XP_006505941.1;XP_006505942.1;XP_006505943.1;XP_006505944.1;XP_011239582.1;XP_017176988.1;XP_017176989.1	98497	.	1.525284	[illegible]
21393	NM_011540	Tcap	titin-cap	protein_coding	NP_035670.2	1330233	.	9.283750	[illegible]
21399	NM_001159750,NM_001159751,NM_011541	Tcea1	transcription elongation factor A (SII) 1	protein_coding	NP_001153222.1;NP_001153223.1;NP_035671.1	1196624	.	34.033644	[illegible]
21400	NM_001356342,NM_001356343,NM_009326	Tcea2	transcription elongation factor A (SII), 2	protein_coding	NP_001343271.1;NP_001343272.1;NP_033352.1	107368	.	7.259474	[illegible]
21401	NM_011542,XM_006538710,XM_006538711,XM_006538712	Tcea3	transcription elongation factor A (SII), 3	protein_coding	NP_035672.1;XP_006538773.1;XP_006538774.1;XP_006538775.1	1196908	.	11.211551	[illegible]
21402	NM_011543,XM_006532786	Skp1a	S-phase kinase-associated protein 1A	protein_coding	NP_035673.3;XP_006532849.1	103575	.	82.914787	[illegible]
21406	NM_001253862,NM_001253863,NM_001253864,NM_001253865,NM_011544,XM_006510994,XM_006510995,XM_006510996,XM_006510997,XM_006510998,XM_006510999,XM_006511000,XM_006511002,XM_017313270,XM_030244212,XM_030244213,XM_030244214,XM_030244215	Tcf12	transcription factor 12	protein_coding	NP_001240791.1;NP_001240792.1;NP_001240793.1;NP_001240794.1;NP_035674.2;XP_006511057.1;XP_006511058.1;XP_006511059.1;XP_006511060.1;XP_006511061.1;XP_006511062.1;XP_006511063.1;XP_006511065.1;XP_017168759.1;XP_030100072.1;XP_030100073.1;XP_030100074.1;XP_030100075.1	101877	.	40.626501	[illegible]
21407	NM_009328,XM_006499100	Tcf15	transcription factor 15	protein_coding	NP_033354.2;XP_006499163.1	104664	.	0.818908	[illegible]
21408	NM_001326566,NM_001347495,NM_009329,XM_006532789,XM_006532790,XM_011248885,XM_011248886	Zfp354a	zinc finger protein 354A	protein_coding	NP_001313495.1;NP_001334424.1;NP_033355.2;XP_006532852.1;XP_006532853.1;XP_011247187.1;XP_011247188.1	103172	.	2.767894	[illegible]
21410	NM_001291268,NM_001291269,NM_009330,XM_006532791,XM_006532793,XM_006532794,XM_006532796	Hnf1b	HNF1 homeobox B	protein_coding	NP_001278197.1;NP_001278198.1;NP_033356.2;XP_006532854.1;XP_006532856.1;XP_006532857.1;XP_006532859.1	98505	.	0.000000	[illegible]
21411	NM_001114140,NM_013836,XM_006520732,XM_006520733,XM_011245551,XM_011245552,XM_011245553,XM_011245554	Tcf20	transcription factor 20	protein_coding	NP_001107612.1;NP_038864.3;XP_006520795.1;XP_006520796.1;XP_011243853.1;XP_011243854.1;XP_011243855.1;XP_011243856.1	108399	.	7.727330	[illegible]
21412	NM_011545	Tcf21	transcription factor 21	protein_coding	NP_035675.1	1202715	.	0.075188	[illegible]
21413	NM_001083967,NM_001361126,NM_001361127,NM_001361128,NM_001361129,NM_013685,XM_006525746,XM_006525748,XM_006525751,XM_006525752,XM_006525753,XM_006525757,XM_006525759,XM_006525760,XM_006525762,XM_006525764,XM_006525766,XM_017317854,XM_017317856,XM_017317857,XM_017317858,XM_017317859,XM_017317860,XM_017317861,XM_017317862,XM_017317863,XM_030250373,XM_030250374,XM_030250375,XM_030250376,XM_030250377,XM_030250378,XM_030250379,XM_030250380,XM_030250381,XM_030250382,XM_030250383,XM_030250384,XM_030250385,XM_030250386,XM_030250387,XM_030250388,XM_030250389,XM_030250390,XM_030250391,XM_030250392,XM_030250393,XM_030250394,XM_030250395,XM_030250397	Tcf4	transcription factor 4	protein_coding	NP_001077436.1;NP_001348055.1;NP_001348056.1;NP_001348057.1;NP_001348058.1;NP_038713.1;XP_006525809.1;XP_006525811.1;XP_006525814.1;XP_006525815.1;XP_006525816.1;XP_006525820.1;XP_006525822.1;XP_006525823.1;XP_006525825.1;XP_006525827.1;XP_006525829.1;XP_017173343.1;XP_017173345.1;XP_017173346.1;XP_017173347.1;XP_017173348.1;XP_017173349.1;XP_017173350.1;XP_017173351.1;XP_017173352.1;XP_030106233.1;XP_030106234.1;XP_030106235.1;XP_030106236.1;XP_030106237.1;XP_030106238.1;XP_030106239.1;XP_030106240.1;XP_030106241.1;XP_030106242.1;XP_030106243.1;XP_030106244.1;XP_030106245.1;XP_030106246.1;XP_030106247.1;XP_030106248.1;XP_030106249.1;XP_030106250.1;XP_030106251.1;XP_030106252.1;XP_030106253.1;XP_030106254.1;XP_030106255.1;XP_030106257.1	98506	.	12.457201	[illegible]
21414	NM_001313981,NM_009331,XM_030245766,XM_030245767,XM_030245768,XM_030245769,XM_030245770,XM_030245771,XM_030245772,XM_030245773,XM_030245774,XM_030245775	Tcf7	transcription factor 7, T cell specific	protein_coding	NP_001300910.1;NP_033357.1;XP_030101626.1;XP_030101627.1;XP_030101628.1;XP_030101629.1;XP_030101630.1;XP_030101631.1;XP_030101632.1;XP_030101633.1;XP_030101634.1;XP_030101635.	98507	.	24.649462	[illegible]
21415	NM_001079822,NM_009332,XM_006505882	Tcf7l1	transcription factor 7 like 1 (T cell specific, HMG box)	protein_coding	NP_001073290.1;NP_033358.2;XP_006505945.1	1202876	.	13.782291	[illegible]
21416	NM_001142918,NM_001142919,NM_001142920,NM_001142921,NM_001142922,NM_001142923,NM_001142924,NM_001331135,NM_001331136,NM_001331137,NM_001331138,NM_001331139,NM_001331140,NM_001331141,NM_001331142,NM_001331143,NM_001331144,NM_001331145,NM_001331146,NM_001331147,NM_001331148,NM_001331149,NM_001331150,NM_009333,NR_138565,XM_006526860,XM_006526861,XM_006526866,XM_006526888,XM_006526894,XM_006526895,XM_006526897,XM_017318110,XM_017318112,XM_017318114,XM_017318115,XM_017318116,XM_017318117,XM_017318119,XM_017318126,XM_017318127,XM_017318131,XM_017318132,XM_017318135,XM_030250847,XM_030250849,XM_030250851,XM_030250852,XM_030250853,XM_030250854,XM_030250855,XM_030250856,XM_030250857,XM_030250859,XM_030250860	Tcf7l2	transcription factor 7 like 2, T cell specific, HMG box	protein_coding	NP_001136390.1;NP_001136391.1;NP_001136392.1;NP_001136393.1;NP_001136394.1;NP_001136395.1;NP_001136396.1;NP_001318064.1;NP_001318065.1;NP_001318066.1;NP_001318067.1;NP_001318068.1;NP_001318069.1;NP_001318070.1;NP_001318071.1;NP_001318072.1;NP_001318073.1;NP_001318074.1;NP_001318075.1;NP_001318076.1;NP_001318077.1;NP_001318078.1;NP_001318079.1;NP_033359.2;XP_006526923.1;XP_006526924.1;XP_006526929.1;XP_006526951.1;XP_006526957.1;XP_006526958.1;XP_006526960.1;XP_017173599.1;XP_017173601.1;XP_017173603.1;XP_017173604.1;XP_017173605.1;XP_017173606.1;XP_017173608.1;XP_017173615.1;XP_017173616.1;XP_017173620.1;XP_017173621.1;XP_017173624.1;XP_030106707.1;XP_030106709.1;XP_030106711.1;XP_030106712.1;XP_030106713.1;XP_030106714.1;XP_030106715.1;XP_030106716.1;XP_030106717.1;XP_030106719.1;XP_030106720.1	1202879	.	7.737628	[illegible]
21417	NM_001360981,NM_001360982,NM_011546,XM_006525769,XM_006525775,XM_030250398,XM_030250399,XM_030250400,XM_030250401,XM_030250402,XM_030250403,XM_030250404,XM_030250405,XM_030250406,XM_030250407,XM_030250408,XM_030250409,XM_030250410,XM_030250411	Zeb1	zinc finger E-box binding homeobox 1	protein_coding	NP_001347910.1;NP_001347911.1;NP_035676.1;XP_006525832.1;XP_006525838.1;XP_030106258.1;XP_030106259.1;XP_030106260.1;XP_030106261.1;XP_030106262.1;XP_030106263.1;XP_030106264.1;XP_030106265.1;XP_030106266.1;XP_030106267.1;XP_030106268.1;XP_030106269.1;XP_030106270.1;XP_030106271.1	1344313	.	16.409556	[illegible]
21418	NM_001122948,NM_001301674,NM_011547,XM_006516902,XM_006516903,XM_017315479,XM_030247224	Tfap2a	transcription factor AP-2, alpha	protein_coding	NP_001116420.1;NP_001288603.1;NP_035677.2;XP_006516965.1;XP_006516966.1;XP_017170968.1;XP_030103084.1	104671	.	0.055082	[illegible]
21419	NM_001025305,NM_001286340,NM_009334,XM_006495491,XM_006495494,XM_006495495,XM_030252835,XM_030252837	Tfap2b	transcription factor AP-2 beta	protein_coding	NP_001020476.1;NP_001273269.1;NP_033360.2;XP_006495554.1;XP_006495557.1;XP_006495558.1;XP_030108695.1;XP_030108697.1	104672	.	0.061011	[illegible]
21420	NM_001159696,NM_009335,XM_006499102,XM_006499103,XM_017316957	Tfap2c	transcription factor AP-2, gamma	protein_coding	NP_001153168.1;NP_033361.2;XP_006499165.1;XP_006499166.1;XP_017172446.1	106032	.	0.000000	[illegible]
21422	NM_001289603,NM_001355497,NM_033476,XM_006520738,XM_017316537,XM_017316538,XM_017316539,XM_017316541,XM_030248455,XM_030248456,XM_030248457	Tfcp2	transcription factor CP2	protein_coding	NP_001276532.1;NP_001342426.1;NP_258437.1;XP_006520801.2;XP_017172026.1;XP_017172027.1;XP_017172028.1;XP_017172030.1;XP_030104315.1;XP_030104316.1;XP_030104317.1	98509	.	7.767569	[illegible]
21423	NM_001164147,NM_001164148,NM_001164149,NM_001164150,NM_001164151,NM_001164152,NM_001164153,NM_011548,XM_017313871,XM_017313872,XM_017313873,XM_017313874,XM_030244992,XM_030244993,XM_030244994,XM_030244995,XM_030244996,XM_030244997,XR_001779500,XR_003948651	Tcf3	transcription factor 3	protein_coding	NP_001157619.1;NP_001157620.1;NP_001157621.1;NP_001157622.1;NP_001157623.1;NP_001157624.1;NP_001157625.1;NP_035678.3;XP_017169360.1;XP_017169361.1;XP_017169362.1;XP_017169363.1;XP_030100852.1;XP_030100853.1;XP_030100854.1;XP_030100855.1;XP_030100856.1;XP_030100857.1	98510	.	25.556048	[illegible]
21425	NM_001161722,NM_001161723,NM_011549,XM_006524003,XM_006524004,XM_006524006,XM_006524007,XM_030249637	Tfeb	transcription factor EB	protein_coding	NP_001155194.1;NP_001155195.1;NP_035679.3;XP_006524066.2;XP_006524067.1;XP_006524069.1;XP_006524070.1;XP_030105497.1	103270	.	5.101638	[illegible]
21426	NM_031198,XM_006505035,XM_006505036,XM_006505037,XM_011241053,XM_017321504	Tfec	transcription factor EC	protein_coding	NP_112475.1;XP_006505098.1;XP_006505099.1;XP_006505100.1;XP_011239355.1;XP_017176993.1	1333760	.	0.859664	[illegible]
21427	NM_009336,XM_030252547,XM_030252548	Vps72	vacuolar protein sorting 72	protein_coding	NP_033362.2;XP_030108407.1;XP_030108408.1	1202305	.	17.956825	[illegible]
21428	NM_001159384,NM_001159385,NM_011550,XM_006532809	Mlx	MAX-like protein X	protein_coding	NP_001152856.1;NP_001152857.1;NP_035680.3;XP_006532872.1	108398	.	10.757146	[illegible]
21429	NM_001044383,NM_001302954,NM_001302955,NM_001362544,NM_001362545,NM_001362547,NM_001362548,NM_001362549,NM_001362550,NM_001362551,NM_001362552,NM_011551,XM_011248888,XM_017314430,XM_030245777,XM_030245778	Ubtf	upstream binding transcription factor, RNA polymerase I	protein_coding	NP_001037848.1;NP_001289883.1;NP_001289884.1;NP_001349473.1;NP_001349474.1;NP_001349476.1;NP_001349477.1;NP_001349478.1;NP_001349479.1;NP_001349480.1;NP_001349481.1;NP_035681.2;XP_011247190.1;XP_017169919.1;XP_030101637.1;XP_03010163	98512	.	34.643072	[illegible]
21452	NM_001130458,NM_001130459,NM_015749,XM_006514610,XM_006514611,XM_006514612,XM_006514613	Tcn2	transcobalamin 2	protein_coding	NP_001123930.1;NP_001123931.1;NP_056564.1;XP_006514673.1;XP_006514674.1;XP_006514675.1;XP_006514676.1	98534	.	30.248577	[illegible]
21453	NM_001198984,NM_011552,XM_006525777,XM_011246874,XM_011246875,XR_001782353,XR_385982	Tcof1	treacle ribosome biogenesis factor 1	protein_coding	NP_001185913.1;NP_035682.1;XP_006525840.1;XP_011245176.1;XP_011245177.1	892003	.	6.504559	[illegible]
21454	NM_001290712,NM_013686	Tcp1	t-complex protein 1	protein_coding	NP_001277641.1;NP_038714.2	98535	.	90.893281	[illegible]
21461	NM_011553,NR_046291,XM_006523213	Tcp10a	t-complex protein 10a	protein_coding	NP_035683.2;XP_006523276.1	98541	.	0.000000	[illegible]
21462	NM_009341,XM_006523313	Tcp10b	t-complex protein 10b	protein_coding	NP_033367.2;XP_006523376.1	98542	.	0.093003	[illegible]
21463	NM_001085555,NM_001357681,NM_013687,XM_006524010,XM_006524011,XM_006524014,XM_006524015,XM_006524016,XM_030249638,XM_030249639	Tcp11	t-complex protein 11	protein_coding	NP_001079024.1;NP_001344610.1;NP_038715.2;XP_006524073.1;XP_006524074.1;XP_006524077.1;XP_006524078.1;XP_006524079.1;XP_030105498.1;XP_030105499.1	98544	.	1.315152	[illegible]
21636	id-Trgv2	Trgv2	T cell receptor gamma variable 2	V_segment	.	98632	TRGV2	0.000000	[illegible]
21645	NM_013688	Tcte1	t-complex-associated testis expressed 1	protein_coding	NP_038716.2	98640	.	0.000000	[illegible]
21646	NM_022311,XM_030249642	Tcte2	t-complex-associated testis expressed 2	protein_coding	NP_071706.2;XP_030105502.1	98641	.	0.207738	[illegible]
21647	NM_011560,NM_198104	Tcte3	t-complex-associated testis expressed 3	protein_coding	NP_035690.2;NP_932772.1	98642	.	0.048889	[illegible]
21648	NM_009342,XM_030249643	Dynlt1b	dynein light chain Tctex-type 1B	protein_coding	NP_033368.1;XP_030105503.1	98643	.	17.541575	[illegible]
21652	NM_001302397,NM_009343,NR_126158	Phf1	PHD finger protein 1	protein_coding	NP_001289326.1;NP_033369.2	98647	.	13.226367	[illegible]
21664	NM_009344	Phlda1	pleckstrin homology like domain, family A, member 1	protein_coding	NP_033370.2	1096880	.	5.443249	[illegible]
21665	NM_001358517,NM_011561,NM_172552,XM_030245048	Tdg	thymine DNA glycosylase	protein_coding	NP_001345446.1;NP_035691.2;NP_766140.2;XP_030100908.1	108247	.	14.890201	[illegible]
21667	NM_011562,XM_017313275	Tdgf1	teratocarcinoma-derived growth factor 1	protein_coding	NP_035692.2;XP_017168764.1	98658	.	0.000000	[illegible]
21672	NM_001317385,NM_011563	Prdx2	peroxiredoxin 2	protein_coding	NP_001304314.1;NP_035693.3	109486	.	88.739813	[illegible]
21673	NM_001043228,NM_009345	Dntt	deoxynucleotidyltransferase, terminal	protein_coding	NP_001036693.1;NP_033371.2	98659	.	0.000000	[illegible]
21676	NM_001166584,NM_001166585,NM_009346	Tead1	TEA domain family member 1	protein_coding	NP_001160056.2;NP_001160057.2;NP_033372.2	101876	.	10.619313	[illegible]
21677	NM_001285498,NM_001285500,NM_011565,XM_006540777,XM_030242342	Tead2	TEA domain family member 2	protein_coding	NP_001272427.1;NP_001272429.1;NP_035695.1;XP_006540840.1;XP_030098202.1	104904	.	29.429558	[illegible]
21678	NM_001098226,NM_001204156,NM_011566	Tead3	TEA domain family member 3	protein_coding	NP_001091696.2;NP_001191085.1;NP_035696.3	109241	.	11.448327	[illegible]
21679	NM_001080979,NM_011567	Tead4	TEA domain family member 4	protein_coding	NP_001074448.2;NP_035697.3	106907	.	0.787351	[illegible]
21681	NM_011568,XR_001779950	Alyref	Aly/REF export factor	protein_coding	NP_035698.1	1341044	.	24.001021	[illegible]
21682	NM_001113460,NM_001113461,NM_001113464,NM_013689,XM_006503845,XM_006503846,XM_006503847,XM_006503848,XM_011240718,XM_011240719	Tec	tec protein tyrosine kinase	protein_coding	NP_001106931.1;NP_001106932.1;NP_001106935.1;NP_038717.2;XP_006503908.1;XP_006503909.1;XP_006503910.1;XP_006503911.1;XP_011239020.1;XP_011239021.1	98662	.	3.478725	[illegible]
21683	NM_001324548,NM_009347,XM_006510152,XM_017313276,XR_379110	Tecta	tectorin alpha	protein_coding	NP_001311477.1;NP_033373.2;XP_006510215.1;XP_017168765.1	109575	.	0.006919	[illegible]
21684	NM_009348,XM_006526902,XM_006526903	Tectb	tectorin beta	protein_coding	NP_033374.2;XP_006526965.1;XP_006526966.1	109574	.	0.000000	[illegible]
21685	NM_017376,NM_153484,XM_006520744,XM_011245557,XM_011245558,XM_011245559,XM_011245560,XM_030248460,XM_030248461	Tef	thyrotroph embryonic factor	protein_coding	NP_059072.1;NP_705617.2;XP_006520807.1;XP_011243859.1;XP_011243860.1;XP_011243861.1;XP_011243862.1;XP_030104320.1;XP_030104321.1	98663	.	18.231336	[illegible]
21687	NM_001290549,NM_001290551,NM_013690,XM_006502930	Tek	TEK receptor tyrosine kinase	protein_coding	NP_001277478.1;NP_001277480.1;NP_038718.2;XP_006502993.1	98664	.	7.640610	[illegible]
21689	NM_001282006,NM_001282007,NM_011569,XM_006532933	Tekt1	tektin 1	protein_coding	NP_001268935.1;NP_001268936.1;NP_035699.2;XP_006532996.1	1333819	.	0.113352	[illegible]
21743	NM_009349	Inmt	indolethylamine N-methyltransferase	protein_coding	NP_033375.1	102963	.	0.208865	[illegible]
21744	NM_009350	Adad1	adenosine deaminase domain containing 1 (testis specific)	protein_coding	NP_033376.2	103258	.	0.065415	[illegible]
21745	NM_009351,XM_030247719,XR_003950834	Tep1	telomerase associated protein 1	protein_coding	NP_033377.1;XP_030103579.1	109573	.	4.041425	[illegible]
21748	NR_001579	Terc	telomerase RNA component	telomerase_RNA	.	109558	.	0.036038	[illegible]
21749	NM_001286628,NM_009352,NR_152944,XM_030252966	Terf1	telomeric repeat binding factor 1	protein_coding	NP_001273557.1;NP_033378.1;XP_030108826.1	109634	.	6.207051	[illegible]
21750	NM_001083118,NM_001286200,NM_001371065,NM_001371066,NM_001371067,NM_009353,NR_104410,XM_006530838,XM_006530840,XR_003947250,XR_003947251,XR_003947252	Terf2	telomeric repeat binding factor 2	protein_coding	NP_001076587.1;NP_001273129.1;NP_001357994.1;NP_001357995.1;NP_001357996.1;NP_033379.2;XP_006530901.1;XP_006530903.1	1195972	.	12.548379	[illegible]
21752	NM_001362387,NM_001362388,NM_009354	Tert	telomerase reverse transcriptase	protein_coding	NP_001349316.1;NP_001349317.1;NP_033380.1	1202709	.	1.423320	[illegible]
21753	NM_207176	Tes	testis derived transcript	protein_coding	NP_997059.1	105081	.	2.287498	[illegible]
21754	NM_011571	Tesk1	testis specific protein kinase 1	protein_coding	NP_035701.3	1201675	.	10.888286	[illegible]
21761	NM_001039147,NM_001357780,NM_024431	Morf4l1	mortality factor 4 like 1	protein_coding	NP_001034236.1;NP_001344709.1;NP_077751.1	1096551	.	179.893204	[illegible]
21762	NM_134101,NR_027485,XM_030249039	Psmd2	proteasome (prosome, macropain) 26S subunit, non-ATPase, 2	protein_coding	NP_598862.1;XP_030104899.1	1096584	.	49.648152	[illegible]
21763	NM_198292,XM_006533143,XM_006533144,XM_006533145,XM_006533146,XM_006533147,XM_006533148	Tex2	testis expressed gene 2	protein_coding	NP_938034.2;XP_006533206.1;XP_006533207.1;XP_006533208.1;XP_006533209.1;XP_006533210.1;XP_006533211.1	102465	.	3.549503	[illegible]
21766	NM_009357,XM_006505886,XM_006505888,XM_030255311	Tex261	testis expressed gene 261	protein_coding	NP_033383.1;XP_006505949.1;XP_006505951.1;XP_030111171.1	1096575	.	25.496894	[illegible]
21767	NM_001081654,NM_001286498,NM_011573,NR_104459	Tex264	testis expressed gene 264	protein_coding	NP_001075123.1;NP_001273427.1;NP_035703.2	1096570	.	24.899505	[illegible]
21769	NM_001356265,NM_148926,NR_151442,NR_151443,XM_0065240	Zfand3	zinc finger, AN1-type domain 3	protein_coding	NP_001343194.1;NP_683728.1;XP_006524091.1	1096572	.	17.524382	[illegible]
21770	NM_001357684,NM_009358	Ppp2r5d	protein phosphatase 2, regulatory subunit B', delta	protein_coding	NP_001344613.1;NP_033384.2	2388481	.	21.023916	[illegible]
21771	NM_001358982,NM_011574	Utp4	UTP4 small subunit processome component	protein_coding	NP_001345911.1;NP_035704.2	1096573	.	12.628468	[illegible]
21778	NM_009359,XM_006511019,XM_006511021,XM_006511022,XM_030244237,XM_030244238,XR_001778850,XR_003947843,XR_003947844,XR_379410	Tex9	testis expressed gene 9	protein_coding	NP_033385.2;XP_006511082.1;XP_006511084.1;XP_006511085.1;XP_030100097.1;XP_030100098.1	1201610	.	3.325873	[illegible]
21780	NM_009360,XM_017313918	Tfam	transcription factor A, mitochondrial	protein_coding	NP_033386.1;XP_017169407.1	107810	.	6.638165	[illegible]
21781	NM_001291765,NM_001291766,NM_001291768,NM_001358991,NM_009361,XM_006508751,XM_006508755,XM_011242048,XM_017312657,XM_017312658,XM_017312659,XM_030243403,XM_030243404,XM_030243405,XM_030243406,XM_030243407,XM_030243408,XM_030243409,XM_030243410,XM_030243411	Tfdp1	transcription factor Dp 1	protein_coding	NP_001278694.1;NP_001278695.1;NP_001278697.1;NP_001345920.1;NP_033387.1;XP_006508814.1;XP_006508818.1;XP_011240350.1;XP_017168146.1;XP_017168147.1;XP_017168148.1;XP_030099263.1;XP_030099264.1;XP_030099265.1;XP_030099266.1;XP_030099267.1;XP_030099268.1;XP_030099269.1;XP_030099270.1;XP_030099	101934	.	37.014738	[illegible]
21782	gene-Tfdp1-ps	Tfdp1-ps	transcription factor Dp 1, pseudogene	pseudogene	.	109560	.	0.000000	[illegible]
21784	NM_009362,XM_006524030	Tff1	trefoil factor 1	protein_coding	NP_033388.1;XP_006524093.1	88135	.	0.000000	[illegible]
21787	NM_001252443,NM_001357124,NM_001357125,NM_001357126,NM_019678,XM_006521980	Tfg	Trk-fused gene	protein_coding	NP_001239372.1;NP_001344053.1;NP_001344054.1;NP_001344055.1;NP_062652.1;XP_006522043.1	1338041	.	43.818471	[illegible]
21788	NM_001177319,NM_001177320,NM_001355271,NM_001355273,NM_011576,XM_006499145,XM_006499146,XM_006499149,XM_006499150,XM_030249392,XM_030249395	Tfpi	tissue factor pathway inhibitor	protein_coding	NP_001170790.1;NP_001170791.1;NP_001342200.1;NP_001342202.1;NP_035706.1;XP_006499208.1;XP_006499209.1;XP_006499212.1;XP_006499213.1;XP_030105252.1;XP_030105255.1	1095418	.	18.856729	[illegible]
21789	NM_009364,NR_138566,XM_006505043,XM_006505044,XM_011241055,XM_011241056	Tfpi2	tissue factor pathway inhibitor 2	protein_coding	NP_033390.1;XP_006505106.1;XP_006505107.1;XP_011239357.1;XP_011239358.1	108543	.	2.819549	[illegible]
21802	NM_031199,XM_030255312	Tgfa	transforming growth factor alpha	protein_coding	NP_112476.1;XP_030111172.1	98724	.	1.346392	[illegible]
21803	NM_011577	Tgfb1	transforming growth factor, beta 1	protein_coding	NP_035707.1	98725	.	40.000969	[illegible]
21804	NM_001289550,NM_001289551,NM_001289552,NM_001289553,XM_011241728,XM_011241729,XM_030242343,XM_030242344	Tgfb1i1	transforming growth factor beta 1 induced transcript 1	protein_coding	NP_001276479.1;NP_001276480.1;NP_001276481.1;NP_001276482.1;XP_011240030.1;XP_011240031.1;XP_030098203.1;XP_03009820	102784	.	41.007816	[illegible]
21807	NM_001177751,NM_001310646,NM_001310647,NM_009366,NM_207652,XM_006518799,XM_006518800,XM_006518801,XM_006518803,XM_017315942,XM_017315943,XM_017315944,XM_017315945,XR_001781104,XR_001781105,XR_003950835,XR_003950836,XR_003950837,XR_383154,XR_383156	Tsc22d1	TSC22 domain family, member 1	protein_coding	NP_001171222.1;NP_001297575.1;NP_001297576.1;NP_033392.1;NP_997535.2;XP_006518862.1;XP_006518863.1;XP_006518864.1;XP_006518866.1;XP_017171431.1;XP_017171432.1;XP_017171433.1;XP_017171434.1	109127	.	157.733645	[illegible]
21808	NM_001329107,NM_009367,XM_006497136,XM_030252968	Tgfb2	transforming growth factor, beta 2	protein_coding	NP_001316036.1;NP_033393.2;XP_006497199.1;XP_030108828.1	98726	.	9.846263	[illegible]
21809	NM_009368	Tgfb3	transforming growth factor, beta 3	protein_coding	NP_033394.2	98727	.	44.919056	[illegible]
21810	NM_009369,NR_155548	Tgfbi	transforming growth factor, beta induced	protein_coding	NP_033395.1	99959	.	136.495029	[illegible]
21812	NM_001312868,NM_001312869,NM_009370,XM_030253397	Tgfbr1	transforming growth factor, beta receptor I	protein_coding	NP_001299797.1;NP_001299798.1;NP_033396.1;XP_030109257.1	98728	.	18.100003	[illegible]
21813	NM_009371,NM_029575	Tgfbr2	transforming growth factor, beta receptor II	protein_coding	NP_033397.3;NP_083851.3	98729	.	44.259510	[illegible]
21814	NM_011578	Tgfbr3	transforming growth factor, beta receptor III	protein_coding	NP_035708.2	104637	.	13.911929	[illegible]
21815	NM_001164074,NM_001164075,NM_001164076,NM_001164077,NM_009372,XM_006524031,XM_006524032,XM_011246363	Tgif1	TGFB-induced factor homeobox 1	protein_coding	NP_001157546.1;NP_001157547.1;NP_001157548.1;NP_001157549.1;NP_033398.2;XP_006524094.1;XP_006524095.1;XP_011244665.1	1194497	.	13.227244	[illegible]
21816	NM_001161714,NM_001161715,NM_019984,XM_006518808,XM_006518809	Tgm1	transglutaminase 1, K polypeptide	protein_coding	NP_001155186.1;NP_001155187.1;NP_064368.3;XP_006518871.1;XP_006518872.1	98730	.	0.296316	[illegible]

21817	NM_009373	Tgm2	transglutaminase 2, C polypeptide	protein_coding	NP_033399.1	98731	.	19.680868	24.111898	17.405684	19.267488	28.437679	19.434029	18.784401	24.318409	51.775673	64.023712	46.852077	51.649311	74.872215	53.237373	50.645901	66.109337
21818	NM_009374,XM_006499151,XM_011239437	Tgm3	transglutaminase 3, E polypeptide	protein_coding	NP_033400.2;XP_006499214.1;XP_011237739.1	98732	.	0.018281	0.025932	0.019441	0.000000	0.051620	0.000000	0.000000	0.000000	0.048094	0.068857	0.052331	0.000000	0.135909	0.000000	0.000000	0.000000
21819	NM_009375,XM_006520745	Tg	thyroglobulin	protein_coding	NP_033401.2;XP_006520808.2	98733	.	0.458269	0.437666	0.449737	0.320549	0.299311	0.330310	0.401253	0.406858	1.205598	1.162121	1.210588	0.859279	0.788042	0.904846	1.081847	1.106041
21821	NM_009376,XM_006518810,XM_006518811,XM_006518812,XM_011245008,XR_003950838,XR_003950839,XR_383157	Ift88	intraflagellar transport 88	protein_coding	NP_033402.2;XP_006518873.1;XP_006518874.1;XP_006518875.1;XP_011243310.1	98715	.	6.927948	5.065794	6.413937	5.592177	5.485673	5.470497	5.107126	5.132358	18.225783	13.451077	17.264837	14.990651	14.442967	14.985820	13.769670	13.952262
21822	NM_011579	Tgtp1	T cell specific GTPase 1	protein_coding	NP_035709.3	98734	.	0.423093	0.442464	0.352153	0.226371	0.693545	0.861128	0.431845	1.091008	1.113057	1.174865	0.947915	0.606821	1.826001	2.358966	1.164326	2.965893
21823	NM_009377	Th	tyrosine hydroxylase	protein_coding	NP_033403.1	98735	.	0.085898	0.000000	0.000000	0.000000	0.000000	0.090587	0.057081	0.000000	0.225978	0.000000	0.000000	0.000000	0.000000	0.248153	0.153899	0.000000
21824	NM_009378	Thbd	thrombomodulin	protein_coding	NP_033404.1	98736	.	16.042608	13.190945	14.802080	13.086186	24.964506	17.277489	12.473885	23.966290	42.204281	35.025585	39.843781	35.079430	65.727867	47.329773	33.631687	65.152100
21825	NM_001313914,NM_011580	Thbs1	thrombospondin 1	protein_coding	NP_001300843.1;NP_035710.2	98737	.	126.204902	130.552246	112.884220	107.852196	112.414681	91.816632	95.924038	78.971203	332.015030	346.652115	303.858230	289.113540	295.971283	251.521492	258.627319	214.682389
21826	NM_011581	Thbs2	thrombospondin 2	protein_coding	NP_035711.2	98738	.	339.958771	307.349640	271.816162	297.899292	287.878754	275.667419	240.403366	223.621017	894.350525	816.097778	731.666382	798.562500	757.942261	755.160339	648.167847	607.911377
21827	NM_013691,NR_149740,XM_006501295,XM_011240072	Thbs3	thrombospondin 3	protein_coding	NP_038719.2;XP_006501358.1;XP_011238374.1	98739	.	116.147888	104.600454	98.256082	54.267040	49.987788	50.849979	79.290851	61.414678	305.557438	277.742965	264.482654	145.470712	131.610478	139.297889	213.781457	166.955156
21828	NM_011582	Thbs4	thrombospondin 4	protein_coding	NP_035712.1	1101779	.	4.255140	11.285126	1.192181	10.179740	3.558623	0.767745	2.060599	3.034065	11.194263	29.965113	3.209077	27.288280	9.369331	2.103153	5.555720	8.248075
21832	NM_001173505,NM_001289894,NM_001289896,NM_009379,XM_006521987,XM_017316926,XM_017316927,XM_017316928,XM_017316929,XM_017316930,XM_017316931,XM_017316932,XM_017316933	Thpo	thrombopoietin	protein_coding	NP_001166976.1;NP_001276823.1;NP_001276825.1;NP_033405.1;XP_006522050.1;XP_017172415.1;XP_017172416.1;XP_017172417.1;XP_017172418.1;XP_017172419.1;XP_017172420.1;XP_017172421.1;XP_017172422.1	101875	.	1.230076	1.053398	0.564337	1.201145	1.654755	0.818002	0.794683	0.803927	3.236038	2.797061	1.519065	3.219847	4.356728	2.240825	2.142596	2.185469
21833	NM_001313983,NM_178060,XM_030245916	Thra	thyroid hormone receptor alpha	protein_coding	NP_001300912.1;NP_835161.1;XP_030101776.1	98742	.	40.890852	45.517254	38.241048	67.934593	54.461972	42.755705	40.105005	38.098533	107.574090	120.860821	102.936068	182.108592	143.390348	117.124523	108.129829	103.570461
21834	NM_001113417,NM_009380,XM_006517966,XM_006517967,XM_006517968,XM_006517970,XM_006517971,XM_006517972,XM_011244737,XM_011244738,XM_011244739,XM_011244740,XM_011244741,XM_011244742,XM_011244743,XM_011244745,XM_017315947,XM_017315948,XM_030247720,XM_030247721	Thrb	thyroid hormone receptor beta	protein_coding	NP_001106888.1;NP_033406.1;XP_006518029.1;XP_006518030.1;XP_006518031.1;XP_006518033.1;XP_006518034.1;XP_006518035.1;XP_011243039.1;XP_011243040.1;XP_011243041.1;XP_011243042.1;XP_011243043.1;XP_011243044.1;XP_011243045.1;XP_011243047.1;XP_017171436.1;XP_017171437.1;XP_030103580.1;XP_030103	98743	.	0.478211	0.627587	0.568072	0.514772	0.647541	0.599977	0.503967	0.733863	1.258057	1.666415	1.529118	1.379924	1.704881	1.643572	1.358780	1.994998
21835	NM_009381	Thrsp	thyroid hormone responsive	protein_coding	NP_033407.1	109126	.	1.052464	3.128995	1.374284	4.959908	0.878624	0.241910	0.583144	1.032221	2.768783	8.308342	3.699255	13.295757	2.313288	0.662686	1.572256	2.806081
21838	NM_009382	Thy1	thymus cell antigen 1, theta	protein_coding	NP_033408.1	98747	.	22.202242	23.240927	20.582727	23.592239	26.243235	26.025841	19.571365	22.344797	58.408810	61.711052	55.403950	63.242439	69.094566	71.294907	52.767689	60.744095
21841	NM_001164078,NM_001164079,NM_011585,XM_006505889,XM_006505891,XM_006505893,XM_011241285,XM_017321512,XM_017321513,XM_030255313,XR_003956184,XR_003956185,XR_003956186,XR_377438	Tia1	cytotoxic granule-associated RNA binding protein 1	protein_coding	NP_001157550.1;NP_001157551.1;NP_035715.1;XP_006505952.1;XP_006505954.1;XP_006505956.1;XP_011239587.1;XP_017177001.1;XP_017177002.1;XP_030111173.1	107914	.	24.184611	22.318476	24.624344	23.754479	22.112225	20.744466	20.839525	22.680987	63.623953	59.261687	66.283048	63.677349	58.218229	56.827173	56.186867	61.658021
21843	NM_001347640,NM_001347641,NM_009383,XM_006507571,XM_017322108,XM_030242346,XM_030242347,XM_030242348,XM_030242349,XM_030242350,XM_030242351,XM_030242352,XM_030242353,XM_030242354,XM_030242355,XR_378230,XR_378231	Tial1	Tia1 cytotoxic granule-associated RNA binding protein-like 1	protein_coding	NP_001334569.1;NP_001334570.1;NP_033409.1;XP_006507634.1;XP_017177597.1;XP_030098206.1;XP_030098207.1;XP_030098208.1;XP_030098209.1;XP_030098210.1;XP_030098211.1;XP_030098212.1;XP_030098213.1;XP_030098214.1;XP_030098215.1	107913	.	35.143595	28.473181	32.186781	28.232667	31.122216	29.337367	29.648636	30.705188	92.454430	75.604126	86.639376	75.681781	81.940202	80.366463	79.937704	83.471727
21844	NM_001145886,NM_001145887,NM_009384,XM_006522978,XM_006522980,XM_006522982,XM_006522983,XM_011246112,XM_017316936,XM_030249040,XM_030249041	Tiam1	T cell lymphoma invasion and metastasis 1	protein_coding	NP_001139358.1;NP_001139359.1;NP_033410.2;XP_006523041.1;XP_006523043.1;XP_006523045.1;XP_006523046.1;XP_011244414.1;XP_017172425.1;XP_030104900.1;XP_030104901.1	103306	.	2.705210	3.019561	2.929037	3.100956	3.126740	3.767009	3.154579	3.232052	7.116762	8.017764	7.884292	8.312560	8.232244	10.319304	8.505276	8.786299
21846	NM_011587	Tie1	tyrosine kinase with immunoglobulin-like and EGF-like domains 1	protein_coding	NP_035717.2	99906	.	12.447351	12.337230	10.013606	10.743243	9.847727	9.539652	10.155810	9.205471	32.746017	32.758739	26.954317	28.798830	25.927612	26.132818	27.381771	25.024977
21847	NM_001289471,NM_001357677,NM_013692	Klf10	Kruppel-like factor 10	protein_coding	NP_001276400.1;NP_001344606.1;NP_038720.1	1101353	.	23.469547	15.162833	20.765447	17.694373	25.465907	16.073565	19.922974	19.028524	61.742790	40.261491	55.895788	47.432355	67.047979	44.031752	53.715686	51.728841
21848	NM_001272064,NM_001272076,NM_145076,XM_006505895	Trim24	tripartite motif-containing 24	protein_coding	NP_001258993.1;NP_001259005.1;NP_659542.3;XP_006505958.1	102752	.	6.534777	6.248364	6.169636	6.250550	6.665578	5.994295	6.305439	6.457941	17.191441	16.591127	16.607236	16.755513	17.549483	16.420709	17.000526	17.555847
21849	NM_011588	Trim28	tripartite motif-containing 28	protein_coding	NP_035718.2	102749	.	48.883026	48.844505	49.660629	50.479084	53.921444	50.380421	47.924820	44.571095	128.599609	129.695602	133.674942	135.316544	141.967209	138.011597	129.213379	121.166046
21853	NM_001136082,NM_001164080,NM_001164081,NM_011589,XM_006513580,XM_006513581,XM_006513582,XM_006513583,XM_030245049,XM_030245050	Timeless	timeless circadian clock 1	protein_coding	NP_001129554.1;NP_001157552.1;NP_001157553.1;NP_035719.1;XP_006513643.1;XP_006513644.1;XP_006513645.1;XP_006513646.1;XP_030100909.1;XP_030100910.1	1321393	.	5.839368	5.463451	6.015211	5.128065	3.096923	6.054798	5.905983	5.399758	15.361986	14.506965	16.191560	13.746525	8.153739	16.586448	15.923525	14.679182
21854	NM_001360857,NM_001360858,NM_011590	Timm17a	translocase of inner mitochondrial membrane 17a	protein_coding	NP_001347786.1;NP_001347787.1;NP_035720.1	1343131	.	27.375621	31.715300	27.945354	32.494829	26.023705	31.598972	29.274964	28.525093	72.018740	84.212845	75.222439	87.107134	68.516583	86.561893	78.930223	77.545167
21855	NM_001313693,NM_011591,NR_132589	Timm17b	translocase of inner mitochondrial membrane 17b	protein_coding	NP_001300622.1;NP_035721.1	1343176	.	10.598211	10.871036	10.311698	9.925434	9.940148	9.915296	9.711139	9.701985	27.881369	28.865591	27.756707	26.606573	26.170947	27.161857	26.182865	26.374746
21856	NM_011592	Timm44	translocase of inner mitochondrial membrane 44	protein_coding	NP_035722.2	1343262	.	18.832350	22.364561	18.837769	23.460733	23.246199	20.549515	19.856689	20.311321	49.543423	59.384064	50.706924	62.889919	61.203812	56.293121	53.536968	55.216114
21857	NM_001044384,NM_001294280,NM_011593	Timp1	tissue inhibitor of metalloproteinase 1	protein_coding	NP_001037849.1;NP_001281209.1;NP_035723.2	98752	.	158.332815	138.452181	158.704588	117.408014	166.561598	196.233069	177.107744	157.455858	416.535923	367.628597	427.196104	314.729310	438.532120	537.558779	477.512238	428.042083
21858	NM_011594	Timp2	tissue inhibitor of metalloproteinase 2	protein_coding	NP_035724.2	98753	.	198.490234	196.956375	189.606918	180.163147	233.414261	207.514587	206.402496	189.250565	522.180542	522.973328	510.378021	482.953583	614.545349	568.463257	556.495850	514.475708
21859	NM_011595	Timp3	tissue inhibitor of metalloproteinase 3	protein_coding	NP_035725.1	98754	.	74.273399	66.294609	70.882278	73.562378	98.687782	100.033478	71.981201	85.715652	195.395630	176.030411	190.798706	197.194687	259.830383	274.030640	194.073410	233.017105
21869	NM_009385,XM_006515791,XM_011244101	Nkx2-1	NK2 homeobox 1	protein_coding	NP_033411.3;XP_006515854.1;XP_011242403.1	108067	.	0.000000	0.000000	0.000000	0.023580	0.000000	0.000000	0.000000	0.000000	0.000000	0.000000	0.000000	0.063209	0.000000	0.000000	0.000000	0.000000
21871	NM_011596	Atp6v0a2	ATPase, H+ transporting, lysosomal V0 subunit A2	protein_coding	NP_035726.2	104855	.	13.510999	13.207936	15.761622	12.898703	13.118515	15.333962	13.069133	14.208526	35.544220	35.070702	42.426647	34.576855	34.539120	42.005695	35.236580	38.625725
21872	NM_001163574,NM_009386,XM_006540778,XM_006540779,XM_006540780,XM_006540781,XM_006540782,XM_006540783,XM_006540784,XM_006540785,XM_006540786,XM_011250843,XM_030242356	Tjp1	tight junction protein 1	protein_coding	NP_001157046.1;NP_033412.2;XP_006540841.1;XP_006540842.1;XP_006540843.1;XP_006540844.1;XP_006540845.1;XP_006540846.1;XP_006540847.1;XP_006540848.1;XP_006540849.1;XP_011249145.1;XP_030098216.1	98759	.	19.964295	20.008808	22.244090	19.521366	22.384307	20.207577	19.614331	25.755209	52.521309	53.128887	59.875953	52.329865	58.934582	55.356422	52.883532	70.015261
21873	NM_001198985,NM_001360391,NM_001360392,NM_011597,XM_006526904,XM_006526907,XM_006526908,XM_006526909,XM_017318136,XM_030250861	Tjp2	tight junction protein 2	protein_coding	NP_001185914.1;NP_001347320.1;NP_001347321.1;NP_035727.2;XP_006526967.1;XP_006526970.1;XP_006526971.1;XP_006526972.1;XP_017173625.1;XP_030106721.1	1341872	.	4.012776	4.348211	4.056025	4.781748	6.144203	5.170444	4.305013	6.069386	10.556656	11.545695	10.917884	12.818175	16.176783	14.163861	11.607040	16.499562
21877	NM_001271729,NM_009387,XM_006533149,XM_006533151,XM_030245917	Tk1	thymidine kinase 1	protein_coding	NP_001258658.1;NP_033413.2;XP_006533212.1;XP_006533214.1;XP_030101777.1	98763	.	17.540602	18.713032	20.955999	18.951356	6.126732	20.859003	19.735715	18.116766	46.145150	49.688250	56.408709	50.801874	16.130784	57.140927	53.210811	49.250234
21881	NM_009388	Tkt	transketolase	protein_coding	NP_033414.1	105992	.	34.666542	36.334576	38.497852	35.371441	37.607410	40.064415	37.560135	37.124638	91.199417	96.478287	103.627327	94.818306	99.014771	109.752029	101.268425	100.922935
21885	NM_001285529,NM_001285530,NM_001285531,NM_001285532,NM_011599,XM_006537758,XM_006537759,XM_006537760,XM_006537761,XM_006537766,XM_011249978,XM_011249979,XM_011249980,XM_011249981,XM_011249982,XM_011249985,XM_011249986,XM_017320104,XM_017320105,XM_017320106,XM_017320107,XM_017320108,XM_017320109,XM_017320110,XM_017320111,XM_017320112,XM_030253398,XM_030253399,XM_030253400,XM_030253401,XM_030253402,XM_030253403	Tle1	transducin-like enhancer of split 1	protein_coding	NP_001272458.1;NP_001272459.1;NP_001272460.1;NP_001272461.1;NP_035729.3;XP_006537821.1;XP_006537822.1;XP_006537823.1;XP_006537824.1;XP_006537829.1;XP_011248280.1;XP_011248281.1;XP_011248282.1;XP_011248283.1;XP_011248284.2;XP_011248287.2;XP_011248288.1;XP_017175593.1;XP_017175594.1;XP_017175595.1;XP_017175596.1;XP_017175597.1;XP_017175598.1;XP_017175599.1;XP_017175600.1;XP_017175601.1;XP_030109258.1;XP_030109259.1;XP_030109260.1;XP_030109261.1;XP_030109262.1;XP_	104636	.	4.558149	5.100220	4.900740	4.506279	5.121806	4.499021	4.058329	4.444403	11.991414	13.542483	13.191661	12.079737	13.484966	12.324569	10.941931	12.082064
21886	NM_001252401,NM_019725,XM_006513584,XM_006513585,XM_006513586,XM_006513588,XM_006513589,XM_011243425,XM_011243426,XM_011243427,XM_011243428,XM_011243429,XM_011243430,XM_011243431,XM_011243432,XM_011243433,XM_011243434,XM_011243435,XM_011243436,XM_017313919,XM_017313920,XM_017313921,XM_017313922,XM_017313923,XM_017313924,XM_030245051,XM_030245052,XM_030245053,XM_030245054,XM_030245055,XM_030245056,XM_030245057,XM_030245058,XM_030245059,XM_030245060,XR_003948682,XR_380389,XR_380390	Tle2	transducin-like enhancer of split 2	protein_coding	NP_001239330.1;NP_062699.1;XP_006513647.1;XP_006513648.1;XP_006513649.1;XP_006513651.1;XP_006513652.1;XP_011241727.1;XP_011241728.1;XP_011241729.1;XP_011241730.1;XP_011241731.1;XP_011241732.1;XP_011241733.1;XP_011241734.1;XP_011241735.1;XP_011241736.1;XP_011241737.1;XP_011241738.1;XP_017169408.1;XP_017169409.1;XP_017169410.1;XP_017169411.1;XP_017169412.1;XP_017169413.1;XP_030100911.1;XP_030100912.1;XP_030100913.1;XP_030100914.1;XP_030100915.1;XP_030100916.1;XP_030100917.1;XP_030100918.1;XP_030100919.1;XP_030100920.1	104635	.	24.458936	32.331999	28.431874	20.500063	23.651120	28.462297	26.212164	30.423568	64.345636	85.850354	76.532035	54.953411	62.269904	77.969313	70.672393	82.706151
21887	NM_001083927,NM_001083928,NM_009389,XM_006511025,XM_006511026,XM_006511027,XM_006511028,XM_006511029,XM_006511030,XM_006511031,XM_017313277,XM_017313278,XM_017313279,XR_003947845	Tle3	transducin-like enhancer of split 3	protein_coding	NP_001077396.1;NP_001077397.1;NP_033415.2;XP_006511088.1;XP_006511089.1;XP_006511090.1;XP_006511091.1;XP_006511092.1;XP_006511093.1;XP_006511094.1;XP_017168766.1;XP_017168767.1;XP_017168768.1	104634	.	10.255287	11.454134	10.355696	10.981085	12.060480	11.946011	8.897667	11.569139	26.979218	30.413873	27.875145	29.436399	31.753466	32.724773	23.989608	31.450579
21888	NM_001302947,NM_001302950,NM_001302951,NM_011600,XM_006526913,XM_006526914,XM_030250862,XM_030250863,XM_030250864	Tle4	transducin-like enhancer of split 4	protein_coding	NP_001289876.1;NP_001289879.1;NP_001289880.1;NP_035730.2;XP_006526976.1;XP_006526977.1;XP_030106722.1;XP_030106723.1;XP_030106724.1	104633	.	3.710125	4.278827	3.758280	5.083733	4.303260	4.022581	3.706403	3.517033	9.760456	11.361460	10.116424	13.627689	11.329846	11.019414	9.993082	9.561019
21892	NM_009390,XM_006509314,XM_006509315,XM_011242185	Tll1	tolloid-like	protein_coding	NP_033416.2;XP_006509377.1;XP_006509378.1;XP_011240487.1	106923	.	1.830125	1.771066	1.311921	1.890373	2.025967	2.781186	1.307337	2.056902	4.814622	4.702670	3.531389	5.067423	5.334071	7.618752	3.524800	5.591664
21894	NM_011602,XM_006537768,XM_006537769,XM_006537770	Tln1	talin 1	protein_coding	NP_035732.2;XP_006537831.1;XP_006537832.3;XP_006537833.1	1099832	.	34.863504	44.378730	42.201005	40.007969	42.610856	47.074097	41.420054	41.757145	91.717577	117.837711	113.595363	107.247188	112.188107	128.954282	111.675427	113.516353
21897	NM_001276445,NM_030682,XM_006503851,XM_006503852,XM_006503853,XM_006503854,XM_006503856,XM_011240720,XM_030254363	Tlr1	toll-like receptor 1	protein_coding	NP_001263374.1;NP_109607.1;XP_006503914.1;XP_006503915.1;XP_006503916.1;XP_006503917.1;XP_006503919.1;XP_011239022.1;XP_030110223.1	1341295	.	0.767979	0.447363	0.635904	0.612006	0.902646	0.875617	0.362991	0.501027	2.020370	1.187871	1.711707	1.640572	2.376533	2.398656	0.978685	1.362035
21898	NM_021297	Tlr4	toll-like receptor 4	protein_coding	NP_067272.1	96824	.	2.683053	2.653389	2.040547	2.336750	3.130040	2.611121	2.866741	2.792021	7.058473	7.045476	5.492681	6.263999	8.240934	7.152877	7.729216	7.590078
21899	NM_001359180,NM_011604,XM_006503859,XM_006503860,XM_030254364	Tlr6	toll-like receptor 6	protein_coding	NP_001346109.2;NP_035734.4;XP_006503922.1;XP_006503923.1;XP_030110224.1	1341296	.	1.825425	1.466608	1.119590	1.340973	1.408496	1.414406	0.898910	1.279244	4.802258	3.894248	3.013678	3.594673	3.708362	3.874607	2.423613	3.477613
21906	NM_172709	Otop1	otopetrin 1	protein_coding	NP_766297.2	2388363	.	0.000000	0.000000	0.064218	0.028231	0.013281	0.000000	0.024606	0.000000	0.000000	0.000000	0.172861	0.075678	0.034967	0.000000	0.066341	0.000000
21907	NM_152229,XM_006512701	Nr2e1	nuclear receptor subfamily 2, group E, member 1	protein_coding	NP_689415.1;XP_006512764.1	1100526	.	0.000000	0.000000	0.000000	0.305878	0.151247	0.080302	0.000000	0.000000	0.000000	0.000000	0.000000	0.819952	0.398212	0.219977	0.000000	0.000000

[illegible]									
22123	NM_009439	Psmd3	proteasome (prosome, macropain) 26S subunit, non-ATPase, 3	protein_coding	NP_033465.1	98858	.	30.835096	[illegible]
22127	NM_009440,XM_006527940,XM_006527941,XM_030251301	Tsx	testis specific X-linked gene	protein_coding	NP_033466.1;XP_006528003.1;XP_006528004.1;XP_030107161.1	108118	.	0.579863	[illegible]
22129	NM_009441,XM_006522984,XM_006522985,XM_006522986,XM_006522987,XM_006522988,XM_006522989,XM_006522990,XM_006522991,XM_006522992,XM_006522993,XM_006522994,XM_006522995,XM_006522996,XM_006522997,XM_006522999,XM_006523000,XM_011246113,XM_017316939,XM_017316940,XM_017316941,XM_017316942,XM_030249046,XM_030249047,XM_030249048,XM_030249049,XM_030249050,XM_030249051,XM_030249052,XM_030249053,XM_030249054,XM_030249055,XM_030249056,XM_030249057,XM_030249058,XM_030249059,XM_030249061	Ttc3	tetratricopeptide repeat domain 3	protein_coding	NP_033467.2;XP_006523047.1;XP_006523048.1;XP_006523049.1;XP_006523050.1;XP_006523051.1;XP_006523052.1;XP_006523053.1;XP_006523054.1;XP_006523055.1;XP_006523056.1;XP_006523057.1;XP_006523058.1;XP_006523059.1;XP_006523060.1;XP_006523062.1;XP_006523063.1;XP_011244415.1;XP_017172428.1;XP_017172429.1;XP_017172430.1;XP_017172431.1;XP_030104906.1;XP_030104907.1;XP_030104908.1;XP_030104909.1;XP_030104910.1;XP_030104911.1;XP_030104912.1;XP_030104913.1;XP_030104914.1;XP_030104915.1;XP_030104916.1;XP_030104917.1;XP_030104918.1;XP_030104919.1;XP_030104921.1	1276539		27.723226	[illegible]
22130	NM_009442,XM_006497851,XM_006497852,XR_374068,XR_374069,XR_374070	Ttf1	transcription termination factor, RNA polymerase I	protein_coding	NP_033468.2;XP_006497914.1;XP_006497915.1	105044	.	4.408683	[illegible]
22134	NM_009443	Tgoln1	trans-golgi network protein	protein_coding	NP_033469.1	105080	.	18.375927	[illegible]
22137	NM_001110265,NM_001284272,NM_009445,XM_006511058,XM_006511059,XM_006511060	Ttk	Ttk protein kinase	protein_coding	NP_001103735.1;NP_001271201.1;NP_033471.2;XP_006511121.1;XP_006511122.1;XP_006511123.1	1194921	.	4.048611	[illegible]
22138	NM_011652,NM_028004,XM_011239438,XM_017317139	Ttn	titin	protein_coding	NP_035782.3;NP_082280.2;XP_011237740.1;XP_017172628.1	98864	.	2.603827	[illegible]
22139	NM_013697,XM_030250412	Ttr	transthyretin	protein_coding	NP_038725.1;XP_030106272.1	98865	.	0.000000	[illegible]
22141	NM_021885	Tub	tubby bipartite transcription factor	protein_coding	NP_068685.1	2651573	.	2.971503	[illegible]
22142	NM_011653	Tuba1a	tubulin, alpha 1A	protein_coding	NP_035783.1	98869	.	254.648056	[illegible]
22143	NM_011654	Tuba1b	tubulin, alpha 1B	protein_coding	NP_035784.1	107804	.	237.765244	[illegible]
22144	NM_009446	Tuba3a	tubulin, alpha 3A	protein_coding	NP_033472.1	1095406	.	0.000000	[illegible]
22145	NM_001313723,NM_001313724,NM_009447,XM_006496478	Tuba4a	tubulin, alpha 4A	protein_coding	NP_001300652.1;NP_001300653.1;NP_033473.1;XP_006496541.1	1095410	.	2.498478	[illegible]
22146	NM_009448	Tuba1c	tubulin, alpha 1C	protein_coding	NP_033474.1	1095409	.	88.917793	[illegible]
22147	NM_009449	Tuba3b	tubulin, alpha 3B	protein_coding	NP_033475.1	1095408	.	0.000000	[illegible]
22151	NM_009450	Tubb2a	tubulin, beta 2A class IIA	protein_coding	NP_033476.1	107861	.	64.797684	[illegible]
22152	NM_023279	Tubb3	tubulin, beta 3 class III	protein_coding	NP_075768.1	107813	.	4.538591	[illegible]
22153	NM_009451	Tubb4a	tubulin, beta 4A class IVA	protein_coding	NP_033477.2	107848	.	0.310361	[illegible]
22154	NM_011655	Tubb5	tubulin, beta 5 class I	protein_coding	NP_035785.1	107812	.	271.275574	[illegible]
22156	NM_001293728,NM_011656,XM_006501307	Tuft1	tuftelin 1	protein_coding	NP_001280657.1;NP_035786.1;XP_006501370.1	109572	.	1.585746	[illegible]
22157	NM_021478,XM_017317394	Tulp1	tubby like protein 1	protein_coding	NP_067453.1;XP_017172883.1	109571	.	1.134475	[illegible]
22158	NM_011657,XM_030255314,XR_003956187,XR_003956188	Tulp3	tubby-like protein 3	protein_coding	NP_035787.1;XP_030111174.1	1329045	.	11.521944	[illegible]
22160	NM_011658	Twist1	twist basic helix-loop-helix transcription factor 1	protein_coding	NP_035788.1	98872	.	47.500156	[illegible]
22163	NM_011659,XM_006538724	Tnfrsf4	tumor necrosis factor receptor superfamily, member 4	protein_coding	NP_035789.1;XP_006538787.3	104512	.	1.060588	[illegible]
22164	NM_009452	Tnfsf4	tumor necrosis factor (ligand) superfamily, member 4	protein_coding	NP_033478.1	104511	.	0.029445	[illegible]
22165	NM_001122754,NM_001289494,NM_001289495,NM_013698,XM_011240722	Txk	TXK tyrosine kinase	protein_coding	NP_001116226.1;NP_001276423.1;NP_001276424.1;NP_038726.2;XP_011239024.1	102960	.	0.038394	[illegible]
22166	NM_011660	Txn1	thioredoxin 1	protein_coding	NP_035790.1	98874	.	86.817093	[illegible]
[illegible]									

22377	NM_001083922,NM_001083923,NM_016757	Wbp1	WW domain binding protein 1	protein_coding	[illegible]
22378	NM_001347642,NM_001361446,NM_016852	Wbp2	WW domain binding protein 2	protein_coding	[illegible]
22379	NM_001310622,NM_001310623,NM_011711,XM_006520844,XM_030248512	Fmnl3	formin-like 3	protein_coding	[illegible]
22380	NM_018765	Wbp4	WW domain binding protein 4	protein_coding	[illegible]
22381	NM_011712	Tceal9	transcription elongation factor A like 9	protein_coding	[illegible]
22384	NM_001312867,NM_033561	Eif4h	eukaryotic translation initiation factor 4H	protein_coding	[illegible]
22385	NM_011714,XM_011240880	Baz1b	bromodomain adjacent to zinc finger domain, 1B	protein_coding	[illegible]
22388	NM_011715,XM_006503863	Wdr1	WD repeat domain 1	protein_coding	[illegible]
22390	NM_001355058,NM_009516	Wee1	WEE 1 homolog 1 (S. pombe)	protein_coding	[illegible]
22393	NM_011716	Wfs1	wolframin ER transmembrane glycoprotein	protein_coding	[illegible]
22401	NM_009517,XM_006535441	Zmat3	zinc finger matrin type 3	protein_coding	[illegible]
22402	NM_018865,NR_157336,XM_006520889	Ccn4	cellular communication network factor 4	protein_coding	[illegible]
22403	NM_016873,XM_006499169	Ccn5	cellular communication network factor 5	protein_coding	[illegible]
22404	NM_011717,NM_212438,NR_033525,XM_006524053,XM_006524054,XM_006524055,XM_006524056,XM_006524057,XM_006524058,XM_006524059,XM_006524061,XM_006524062,XM_006524063,XM_006524065,XM_006524073,XM_006524074,XM_011246379,XM_011246380,XM_017317399,XM_017317401,XM_017317402,XM_030249653,XM_030249654,XM_030249655,XM_030249656,XM_030249657,XM_030249658,XM_030249659,XM_030249660,XM_030249661,XM_030249662,XM_030249664,XM_030249665,XM_030249666,XM_030249667,XM_030249668,XM_030249669,XM_030249670,XM_030249671,XM_030249672,XM_030249673	Wiz	widely-interspaced zinc finger motifs	protein_coding	[illegible]
22408	NM_021279	Wnt1	wingless-type MMTV integration site family, member 1	protein_coding	[illegible]
22409	NM_009518	Wnt10a	wingless-type MMTV integration site family, member 10A	protein_coding	[illegible]
22410	NM_011718,XM_006520891,XM_006520892,XM_006520893	Wnt10b	wingless-type MMTV integration site family, member 10B	protein_coding	[illegible]
22411	NM_001285792,NM_001285794,NM_001285795,NM_009519,XM_006507586,XM_006507587,XM_011241733	Wnt11	wingless-type MMTV integration site family, member 11	protein_coding	[illegible]
22412	NM_011719,XM_030245931	Wnt9b	wingless-type MMTV integration site family, member 9B	protein_coding	[illegible]
22413	NM_023653,XM_006505045	Wnt2	wingless-type MMTV integration site family, member 2	protein_coding	[illegible]
22414	NM_009520	Wnt2b	wingless-type MMTV integration site family, member 2B	protein_coding	[illegible]
22415	NM_009521,XM_006533168	Wnt3	wingless-type MMTV integration site family, member 3	protein_coding	[illegible]
22416	NM_009522	Wnt3a	wingless-type MMTV integration site family, member 3A	protein_coding	[illegible]
22417	NM_009523	Wnt4	wingless-type MMTV integration site family, member 4	protein_coding	[illegible]
22418	NM_001256224,NM_009524,XM_006518923,XM_006518924,XM_006518926,XM_030247779	Wnt5a	wingless-type MMTV integration site family, member 5A	protein_coding	[illegible]
22419	NM_001271757,NM_001271758,NM_009525,XM_006505924,XM_030255319	Wnt5b	wingless-type MMTV integration site family, member 5B	protein_coding	[illegible]
22420	NM_009526,XM_006495887,XM_006495888,XM_006495889	Wnt6	wingless-type MMTV integration site family, member 6	protein_coding	[illegible]
22421	NM_001363757,NM_009527	Wnt7a	wingless-type MMTV integration site family, member 7A	protein_coding	[illegible]
22422	NM_001163633,NM_001163634,NM_009528	Wnt7b	wingless-type MMTV integration site family, member 7B	protein_coding	[illegible]
22423	NM_011720	Wnt8b	wingless-type MMTV integration site family, member 8B	protein_coding	[illegible]
22427	NM_001122822,NM_011721,XM_006509091,XM_006509093,XM_017312662	Wrn	Werner syndrome RecQ like helicase	protein_coding	[illegible]
22428	NM_001293757,NM_001293758,NM_001293759,NM_011722,XM_006509094,XM_030243416,XM_030243417	Dctn6	dynactin 6	protein_coding	[illegible]
22431	NM_144783	Wt1	Wilms tumor 1 homolog	protein_coding	[illegible]
22433	NM_001271730,NM_013842	Xbp1	X-box binding protein 1	protein_coding	[illegible]
22436	NM_011723,XM_011246381,XR_003952104	Xdh	xanthine dehydrogenase	protein_coding	[illegible]
22437	NM_011724,XM_030244248,XM_030244249	Xirp1	xin actin-binding repeat containing 1	protein_coding	[illegible]
22439	NM_023500	Xk	X-linked Kx blood group	protein_coding	[illegible]
22441	NM_001291747,NM_001291748,NM_011725,XM_011249649,XM_011249650,XM_011249651	Xlr	X-linked lymphocyte-regulated	protein_coding	[illegible]
22445	NM_001110784,XM_011247556,XM_011247557,XM_011247558,XM_011247559	Xlr3a	X-linked lymphocyte-regulated 3A	protein_coding	[illegible]
22446	NM_011727,XM_006527945,XM_006527946,XM_006527947	Xlr3c	X-linked lymphocyte-regulated 3C	protein_coding	[illegible]
22589	NM_009530	Atrx	ATRX, chromatin remodeler	protein_coding	[illegible]
22590	NM_011728,XR_001784116,XR_003954906,XR_390317	Xpa	xeroderma pigmentosum, complementation group A	protein_coding	[illegible]
22591	NM_009531	Xpc	xeroderma pigmentosum, complementation group C	protein_coding	[illegible]
22592	NM_011729,XM_006495890,XM_030253066	Ercc5	excision repair cross-complementing rodent repair deficiency, complementation group 5	protein_coding	[illegible]
22594	NM_001360168,NM_001360169,NM_001360170,NM_009532,XM_011250480,XR_001785520,XR_881685	Xrcc1	X-ray repair complementing defective repair in Chinese hamster cells 1	protein_coding	[illegible]
22596	NM_001357519,NM_001357520,NM_009533,XM_006495892,XM_006495893	Xrcc5	X-ray repair complementing defective repair in Chinese hamster cells 5	protein_coding	[illegible]
22598	NM_001040692,NM_001136087,NM_001168643,NM_001168644,NM_001168645,NM_001168646	Slc6a18	solute carrier family 6 (neurotransmitter transporter), member 18	protein_coding	[illegible]
22599	NM_011731	Slc6a20b	solute carrier family 6 (neurotransmitter transporter), member 20B	protein_coding	[illegible]
22601	NM_001171147,NM_009534,XM_006509852,XM_006509853,XM_006509854,XM_006509855,XM_006509856,XM_006509857,XM_030244250,XM_030244251	Yap1	yes-associated protein 1	protein_coding	[illegible]
22608	NM_011732,XM_006502933,XM_006502934,XM_030253407	Ybx1	Y box protein 1	protein_coding	[illegible]
22612	NM_001205132,NM_001205133,NM_009535	Yes1	YES proto-oncogene 1, Src family tyrosine kinase	protein_coding	[illegible]
22619	NM_011734	Siae	sialic acid acetylesterase	protein_coding	[illegible]
22625	NM_011737,XM_006529396	Map3k19	mitogen-activated protein kinase kinase kinase 19	protein_coding	[illegible]
22626	NM_194333,XM_006496479,XM_006496480,XM_006496481,XM_030253070,XR_373540	Slc23a3	solute carrier family 23 (nucleobase transporters), member 3	protein_coding	[illegible]
22627	NM_009536	Ywhae	tyrosine 3-monooxygenase/tryptophan 5-monooxygenase activation protein, epsilon polypeptide	protein_coding	[illegible]
22628	NM_018871	Ywhag	tyrosine 3-monooxygenase/tryptophan 5-monooxygenase activation protein, gamma polypeptide	protein_coding	[illegible]
22629	NM_011738	Ywhah	tyrosine 3-monooxygenase/tryptophan 5-monooxygenase activation protein, eta polypeptide	protein_coding	[illegible]
22630	NM_011739	Ywhaq	tyrosine 3-monooxygenase/tryptophan 5-monooxygenase activation protein theta	protein_coding	[illegible]
22631	NM_001253805,NM_001253806,NM_001253807,NM_001356569,NM_011740,XM_030248527	Ywhaz	tyrosine 3-monooxygenase/tryptophan 5-monooxygenase activation protein, zeta polypeptide	protein_coding	[illegible]
22632	NM_009537,XM_006515820	Yy1	YY1 transcription factor	protein_coding	[illegible]
22634	NM_001364643,NM_001364644,NM_001364645,NM_009538	Plagl1	pleiomorphic adenoma gene-like 1	protein_coding	[illegible]
22635	NM_011741,XM_017320803,XM_017320804,XM_017320805,XM_017320806,XM_017320807,XM_017320808,XM_017320809,XM_017320810,XM_017320811,XM_017320813,XM_017320814,XR_001784665,XR_001784666,XR_001784667,XR_003955622,XR_0039556	Zan	zonadhesin	protein_coding	[illegible]
22637	NM_001289612,NM_001289765,NM_001289766,NM_009539,XM_006495896	Zap70	zeta-chain (TCR) associated protein kinase	protein_coding	[illegible]
22639	NR_037920	Zfa-ps	zinc finger protein, autosomal, pseudogene	transcribed_pseudogene	[illegible]
22640	NM_001037665,NM_001358993,NM_011742,NR_156445,NR_156446,XM_030243418,XM_030243420	Zfp1	zinc finger protein 1	protein_coding	[illegible]
22642	NM_009541,XM_006538727	Zbtb17	zinc finger and BTB domain containing 17	protein_coding	[illegible]
22643	NM_009542,XM_011246418,XM_011246419,XM_017317452	Zfp101	zinc finger protein 101	protein_coding	[illegible]
22644	NM_001308303,NM_009543,XM_006505926,XM_030255320	Rnf103	ring finger protein 103	protein_coding	[illegible]
22646	NM_009544,XM_011242959	Zfp105	zinc finger protein 105	protein_coding	[illegible]
22648	NM_172462	Zfp11	zinc finger protein 11	protein_coding	[illegible]
22652	NM_011746	Mkrn3	makorin, ring finger protein, 3	protein_coding	[illegible]
22654	NM_001356268,NM_011747,XM_006524205,XM_006524210,XM_006524211,XM_011246420,XM_011246421,XM_011246422,XM_011246423,XM_030249748,XM_030249749,XM_030249750	Zfp13	zinc finger protein 13	protein_coding	[illegible]
22658	NM_001163307,NM_001163308,NM_009545,XM_011248972,XM_011248973,XM_011248975,XM_017314503	Pcgf2	polycomb group ring finger 2	protein_coding	[illegible]
22661	NM_001358569,NM_001358570,NM_011749,XM_006522098,XM_006522099,XM_006522100,XM_006522101,XM_006522103,XM_006522104,XM_011245888,XM_030249100	Zfp148	zinc finger protein 148	protein_coding	[illegible]
22666	NM_001356282,NM_001356283,NM_009547	Zbtb14	zinc finger and BTB domain containing 14	protein_coding	[illegible]
22668	NM_001110791,NM_001360393,NM_011750,NR_122118,XM_006531733,XM_006531734,XM_006531735,XM_006531736,XM_006531737,XM_006531738,XM_006531740,XM_030250923,XM_030250925,XM_030250926,XM_030250927,XM_030250928,XM_030250929,XM_030250930,XM_030250931	Sf1	splicing factor 1	protein_coding	[illegible]
22670	NM_001025599,NM_001286726,NM_001286727,XM_006524213,XM_006524214	Trim26	tripartite motif-containing 26	protein_coding	[illegible]
22671	NM_001291024,NM_001359130,NM_009548,XM_006533178,XM_006533180,XM_006533181,XM_006533182,XM_006533183,XM_006533184,XM_006533185,XM_017314504,XM_017314505,XM_030245933,XR_003949415	Rnf112	ring finger protein 112	protein_coding	[illegible]
22673	NM_001109043,NM_009549,XM_006527961,XM_006527962,XM_006527963	Zfp185	zinc finger protein 185	protein_coding	[illegible]
22678	NM_001044697,NM_001044698,NM_001044700,NM_001294323,NM_178447,XM_017314506,XM_017314507,XM_017314508,XM_030245935,XM_030245936,XM_030245937,XM_030245938,XM_030245939,XM_030245940,XM_030245941	Zfp2	zinc finger protein 2	protein_coding	[illegible]
22680	NM_001130169,NM_001130170,NM_001130171,NM_001362721,NM_011751,NR_045038,NR_156116,NR_156117,NR_156118,NR_156119,NR_156120,NR_156121,NR_156122,XM_006533189,XR_001779976,XR_001779977,XR_001779978,XR_001779982,XR_001779983,XR_001779985,XR_001779986,XR_001779988,XR_001779989,XR_001779990,XR_001779991,XR_001779996,XR_003949416,XR_003949417,XR_003949418,XR_003949419,XR_003949420,XR_003949421,XR_003949422,XR_003949423,XR_003949424,XR_003949425,XR_003949426,XR_003949427,XR_003949428,XR_003949429,XR_003949430,XR_003949431,XR_003949432,XR_388432,	Zfp207	zinc finger protein 207	protein_coding	[illegible]
22682	NM_009551,XM_006527058	Zfand5	zinc finger, AN1-type domain 5	protein_coding	[illegible]
22685	NM_001001792,NM_001360938,NM_008616,XM_030255321,XM_030255322,XM_030255323,XM_030255324,XM_030255325	Zfp239	zinc finger protein 239	protein_coding	[illegible]
22687	NM_011752,XR_003947859,XR_003947860	Zpr1	ZPR1 zinc finger	protein_coding	[illegible]
22688	NM_001357809,NM_011753,XM_011242451,XM_011242452,XM_011242453,XM_011242454,XM_011242455,XM_011242456,XM_017313287,XM_030244252,XM_030244253,XM_030244254,XM_030244255	Zfp26	zinc finger protein 26	protein_coding	[illegible]
22689	NM_001037707,NM_001285797,NM_001285798,NM_011754,XM_011250481,XM_011250482,XM_017322132,XM_030242388,XM_030242389	Zfp27	zinc finger protein 27	protein_coding	[illegible]
22690	NM_175247,XM_017322133,XM_030242390,XM_030242391,XM_030242392	Zfp28	zinc finger protein 28	protein_coding	[illegible]
22691	NM_009553,XM_006540805,XM_006540806,XM_006540807,XM_006540808,XM_006540809,XM_006540810,XM_006540811,XM_006540812	Zscan2	zinc finger and SCAN domain containing 2	protein_coding	[illegible]
22693	NM_001360207,NM_001360208,NM_001360209,NM_001360210,NM_001360212,NM_001360213,NM_013705	Zfp30	zinc finger protein 30	protein_coding	[illegible]
22694	NM_011755	Zfp35	zinc finger protein 35	protein_coding	[illegible]
22695	NM_011756	Zfp36	zinc finger protein 36	protein_coding	[illegible]
22696	NM_001290350,NM_001290351,NM_001290353,NM_001348347,NM_009554,XM_006537800	Zfp37	zinc finger protein 37	protein_coding	[illegible]
22697	NM_001044703,NM_001044704,NM_001044705,NM_011757,XM_011240942	Zscan21	zinc finger and SCAN domain containing 21	protein_coding	[illegible]
22698	NM_011758	Zfp39	zinc finger protein 39	protein_coding	[illegible]
22700	NM_001357697,NM_009555,XM_017317456,XM_030249752,XM_030249753	Zfp40	zinc finger protein 40	protein_coding	[illegible]
22701	NM_001044718,NM_011759,XM_006520894,XM_006520895,XM_006520896,XM_030248528	Zfp41	zinc finger protein 41	protein_coding	[illegible]
22703	gene-Zfp42-ps1	Zfp42-ps1	zinc finger protein 48, pseudogene 1	pseudogene	[illegible]
22704	NM_001356308,NM_001370651,NM_009557,XM_006538728,XM_006538732,XM_011250229	Zfp46	zinc finger protein 46	protein_coding	[illegible]
22709	NM_001308323,NM_009558,XM_030249754	Zfp51	zinc finger protein 51	protein_coding	[illegible]
22710	NM_144515,XM_011246426	Zfp52	zinc finger protein 52	protein_coding	[illegible]
22712	NM_011760,XM_030249756,XM_030249757,XM_030249758,XM_030249759,XM_030249760,XM_030249761	Zfp54	zinc finger protein 54	protein_coding	[illegible]
22715	NM_001013745,NM_001168501,NM_001168502,NR_033137,XM_006524224,XM_017317459,XM_030249763	Zfp57	zinc finger protein 57	protein_coding	[illegible]
22717	NM_011762,XM_030242393,XM_030242394,XM_030242395,XM_030242396,XM_030242397	Zfp59	zinc finger protein 59	protein_coding	[illegible]
22718	NM_009560,NM_029531	Zfp60	zinc finger protein 60	protein_coding	[illegible]
22719	NM_009561,XM_011250486,XM_017322140,XM_030242398	Zfp61	zinc finger protein 61	protein_coding	[illegible]
22720	NM_001024846,NM_001362725,NM_001362726,NM_001362727,NM_001362728,NM_001362729,NM_001362730,NM_001362731,NM_009562,XM_006533199,XM_017314510,XM_030245946,XM_030245947,XM_030245949,XM_030245950,XM_030245951	Zfp62	zinc finger protein 62	protein_coding	[illegible]
22722	NM_001369059,NM_001370946,NM_009564	Zfp64	zinc finger protein 64	protein_coding	[illegible]
22724	NM_001355206,NM_001355211,NM_001355212,NM_009565,XM_006501313,XM_006501314,XM_006501316,XM_030252554,XM_030252555	Zbtb7b	zinc finger and BTB domain containing 7B	protein_coding	[illegible]
22746	NM_001001130,XM_030247245	Zfp85	zinc finger protein 85	protein_coding	[illegible]
22750	NM_011763,XM_011241293	Zfp9	zinc finger protein 9	protein_coding	[illegible]
22751	NM_001368328,NM_001368329,NM_001368330,NM_001368331,NM_011764,XM_006530849	Zfp90	zinc finger protein 90	protein_coding	[illegible]
22754	NM_001310531,NM_009566,XM_006527964,XM_017318463,XR_386920	Zfp92	zinc finger protein 92	protein_coding	[illegible]
22755	NM_001360227,NM_009567,NR_153420,XM_017322143	Zfp93	zinc finger protein 93	protein_coding	[illegible]
22756	NM_001199321,NM_009568,XM_011250487,XM_011250491,XM_030242400,XM_030242402	Zfp94	zinc finger protein 94	protein_coding	[illegible]
22757	NM_001167944,NM_001359187,NM_001359188,NM_001359189,NM_001359190,NM_001359191,NM_001359192,NM_016683,XM_006504822,XM_011241010	Zkscan5	zinc finger with KRAB and SCAN domains 5	protein_coding	[illegible]
22758	NM_016684,XM_006516660,XM_030247246	Zscan12	zinc finger and SCAN domain containing 12	protein_coding	[illegible]
22759	NM_011765,XM_030249764	Zfp97	zinc finger protein 97	protein_coding	[illegible]
22761	NM_009569,XM_006530851,XM_030243422	Zfpm1	zinc finger protein, multitype 1	protein_coding	[illegible]
22762	NM_001356998,NM_011766	Zfpm2	zinc finger protein, multitype 2	protein_coding	[illegible]
22763	NM_001358823,NM_011767,XM_006520057,XM_006520058,XM_006520059,XM_017316588,XM_030248530,XR_001781520,XR_001781521,XR_001781522	Zfr	zinc finger RNA binding protein	protein_coding	[illegible]
22764	NM_001044386,NM_001358354,NM_001358355,NM_001358356,NM_001358357,NM_011768,XM_006527965,XM_006527967,XM_011247577,XM_011247578,XM_017318464,XM_030251310,XM_030251311	Zfx	zinc finger protein X-linked	protein_coding	[illegible]
22770	NM_001042438,NM_009572	Zhx1	zinc fingers and homeoboxes 1	protein_coding	[illegible]
22771	NM_009573,XM_006511061	Zic1	zinc finger protein of the cerebellum 1	protein_coding	[illegible]
22772	NM_009574	Zic2	zinc finger protein of the cerebellum 2	protein_coding	[illegible]
22773	NM_009575	Zic3	zinc finger protein of the cerebellum 3	protein_coding	[illegible]
22774	NM_009576,XM_006511067,XM_006511078,XM_006511079,XM_006511080,XM_011242719,XM_011242720,XM_011242722,XM_011242723,XM_011242725,XM_011242726,XM_011242727,XM_017313289,XM_017313290,XM_017313291,XM_017313292,XM_017313293,XM_030244256,XM_030244257,XM_030244258,XM_030244259,XM_030244260,XM_030244261,XM_030244262,XM_030244263,XM_030244264,XM_030244265,XM_030244266,XM_030244267,XM_030244268,XM_030244269,XM_030244270,XM_030244271,XM_030244272,XM_030244273,XM_030244274	Zic4	zinc finger protein of the cerebellum 4	protein_coding	[illegible]
22775	NM_009577	Zik1	zinc finger protein interacting with K protein 1	protein_coding	[illegible]
22776	NM_011769,XM_011250495,XM_011250497,XM_011250498,XM_011250499,XM_030242403	Zim1	zinc finger, imprinted 1	protein_coding	[illegible]
22778	NM_001025597,NM_001301863,NM_001301865,NM_001301866,NM_001301868,NM_009578,XM_006514676,XM_006514680,XM_006514682,XM_011243700,XM_011243701,XM_011243702,XM_011243703,XM_011243704,XM_011243705,XM_011243706,XM_011243707,XM_011243708,XM_011243709,XM_011243710,XM_011243711,XM_011243712,XM_011243713,XM_011243714,XM_011243715,XM_011243716,XM_011243717,XM_011243718,XM_030245952,XM_030245953,XM_030245954,XM_030245955,XM_030245956	Ikzf1	IKAROS family zinc finger 1	protein_coding	[illegible]
22779	NM_011770,XM_006495959,XM_006495961,XM_006495965,XM_006495966,XM_006495967,XM_006495968,XM_011238504,XM_011238508,XM_011238509,XM_017320399,XM_030253862,XM_030253863,XM_030253865,XM_030253867,XM_030253869,XM_030253878	Ikzf2	IKAROS family zinc finger 2	protein_coding	[illegible]
22780	NM_011771,XM_006533204,XM_006533205,XM_006533206	Ikzf3	IKAROS family zinc finger 3	protein_coding	[illegible]
22781	NM_001358536,NM_001358537,NM_011772,XM_006513612,XM_006513613,XM_006513616,XM_011243440,XM_011243441,XM_011243442,XM_017313932,XM_017313933,XM_030245074,XM_030245075,XM_030245076,XM_030245077,XM_030245078	Ikzf4	IKAROS family zinc finger 4	protein_coding	[illegible]
22782	NM_009579,XM_006497158	Slc30a1	solute carrier family 30 (zinc transporter), member 1	protein_coding	[illegible]
22784	NM_001347323,NM_011773,XM_006503865,XM_030254369,XM_030254370	Slc30a3	solute carrier family 30 (zinc transporter), member 3	protein_coding	[illegible]

22785	NM_001290993,NM_011774	Slc30a4	solute carrier family 30 (zinc transporter), member 4	protein_coding	[illegible]
22787	NM_011775,XM_006507589,XM_006507590	Zp2	zona pellucida glycoprotein 2	protein_coding	[illegible]
22788	NM_011776	Zp3	zona pellucida glycoprotein 3	protein_coding	[illegible]
22791	NM_009584,XM_006535679	Dnajc2	DnaJ heat shock protein family (Hsp40) member C2	protein_coding	[illegible]
22793	NM_001289617,NM_001289618,NM_001289619,NM_011777,XM_006505936,XM_030255326,XM_030255327,XM_030255328,XM_030255329	Zyx	zyxin	protein_coding	[illegible]
23789	NM_011778,XM_006531741,XM_006531742	Coro1b	coronin, actin binding protein 1B	protein_coding	[illegible]
23790	NM_011779	Coro1c	coronin, actin binding protein 1C	protein_coding	[illegible]
23792	NM_011780,XM_006495969,XM_006495970	Adam23	a disintegrin and metallopeptidase domain 23	protein_coding	[illegible]
23794	NM_011782	Adamts5	a disintegrin-like and metallopeptidase (reprolysin type) with thrombospondin type 1 motif, 5 (aggrecanase-2)	protein_coding	[illegible]
23796	NM_011784	Aplnr	apelin receptor	protein_coding	[illegible]
23797	NM_011785,XM_006496818,XM_006496819,XM_006496821,XM_011238805,XM_030253910,XM_030253917	Akt3	thymoma viral proto-oncogene 3	protein_coding	[illegible]
23801	NM_011786,XM_030245981,XR_003949435,XR_003949436,XR_003949437,XR_388449,XR_388452	Aloxe3	arachidonate lipoxygenase 3	protein_coding	[illegible]
23802	NM_011787,XM_017312768	Amfr	autocrine motility factor receptor	protein_coding	[illegible]
23805	NM_011789,XM_006513643,XM_006513644,XM_006513645,XM_006513646,XM_030245088,XM_030245089	Apc2	adenomatosis polyposis coli 2	protein_coding	[illegible]
23806	NM_019927,XM_006511140,XM_006511141,XM_011242739	Arih1	ariadne RBR E3 ubiquitin protein ligase 1	protein_coding	[illegible]
23807	NM_001357283,NM_001357285,NM_001357286,NM_011790,NR_151664,XM_011242881,XM_030244351,XM_030244352,XM_030244353	Arih2	ariadne RBR E3 ubiquitin protein ligase 2	protein_coding	[illegible]
23808	NM_001080793,NM_001286207,NM_001359012,NM_011791,XM_011242136,XR_001778438	Ash2l	ASH2 like histone lysine methyltransferase complex subunit	protein_coding	[illegible]
23821	NM_001145947,NM_011792,XM_011242512,XM_011242513	Bace1	beta-site APP cleaving enzyme 1	protein_coding	[illegible]
23825	NM_001038231,NM_001286608,NM_011793,NR_104482	Banf1	barrier to autointegration factor 1	protein_coding	[illegible]
23827	NM_001347210,NM_001347212,NM_011794,XM_006497160,XM_006497161,XM_030253921	Bpnt1	bisphosphate 3'-nucleotidase 1	protein_coding	[illegible]
23828	NM_024285,XM_006512736,XM_006512737	Bves	blood vessel epicardial substance	protein_coding	[illegible]
23829	NM_011795	C1ql1	complement component 1, q subcomponent-like 1	protein_coding	[illegible]
23830	NM_011796	Capn10	calpain 10	protein_coding	[illegible]
23831	NM_001355750,NM_001355751,NM_001355752,NM_011797,XM_006501442,XM_006501443	Car14	carbonic anhydrase 14	protein_coding	[illegible]
23832	NM_011798	Xcr1	chemokine (C motif) receptor 1	protein_coding	[illegible]
23833	NM_013706	Cd52	CD52 antigen	protein_coding	[illegible]
23834	NM_001025779,NM_001362735,NM_011799,NR_156128,XR_001780004,XR_003949441	Cdc6	cell division cycle 6	protein_coding	[illegible]
23836	NM_011800,XM_030253939,XM_030253941	Cdh20	cadherin 20	protein_coding	[illegible]
23837	NM_001368335,NM_001368336,NM_001368337,NM_001368338,NM_011801	Cfdp1	craniofacial development protein 1	protein_coding	[illegible]
23844	NM_017474,XM_006501444	Clca1	chloride channel accessory 1	protein_coding	[illegible]
23845	NM_001038604,NM_021364	Clec5a	C-type lectin domain family 5, member a	protein_coding	[illegible]
23849	NM_011803	Klf6	Kruppel-like factor 6	protein_coding	[illegible]
23850	NM_001085376,XM_006496824	Pappa2	pappalysin 2	protein_coding	[illegible]
23853	NM_027185,XM_030249766	Def6	differentially expressed in FDCP 6	protein_coding	[illegible]
23854	NM_001253783,NM_001253784,NM_001281803,NM_054046,XM_006530975,XM_006530976,XM_006530977,XM_006530978	Def8	differentially expressed in FDCP 8	protein_coding	[illegible]
23856	NM_001291432,NM_001291433,NM_011805,NM_175551,NM_177852,NR_111965,XM_006500608,XM_006500609,XM_006500610,XM_006500611,XM_006500612,XM_006500615,XM_006500616	Dido1	death inducer-obliterator 1	protein_coding	[illegible]
23857	NM_001110327,NM_011806,NR_152858,NR_152859,NR_152860,NR_152861,XM_006503572,XM_006503574,XM_011240668,XM_011240669,XM_030254463,XM_030254464,XM_030254465,XM_030254466,XR_001784690,XR_001784692,XR_003955639	Dmtf1	cyclin D binding myb-like transcription factor 1	protein_coding	[illegible]
23859	NM_001243046,NM_001243047,NM_011807,XM_006507767,XM_006507769,XM_006507770,XM_006507771,XM_006507772,XM_006507773,XM_006507776,XM_006507777,XM_006507778,XM_006507779,XM_006507780,XM_006507781,XM_006507782,XM_006507783,XM_006507784,XM_006507785,XM_006507786,XM_006507787,XM_006507788,XM_006507789,XM_006507790,XM_011241786,XM_030242541,XM_030242542,XM_030242543	Dlg2	discs large MAGUK scaffold protein 2	protein_coding	[illegible]
23863	NM_201227,NR_033145	Dand5	DAN domain family member 5, BMP antagonist	protein_coding	[illegible]
23871	NM_001038642,NM_011808,XM_006510269	Ets1	E26 avian leukemia oncogene 1, 5' domain	protein_coding	[illegible]
23872	NM_011809	Ets2	E26 avian leukemia oncogene 2, 3' domain	protein_coding	[illegible]
23873	NM_001122851,NM_011810	Faim	Fas apoptotic inhibitory molecule	protein_coding	[illegible]
23874	NM_001278075,NM_011811,NR_102757,NR_102758,XR_865550	Farsb	phenylalanyl-tRNA synthetase, beta subunit	protein_coding	[illegible]
23876	NM_001361987,NM_011812,XM_006515865,XM_006515866	Fbln5	fibulin 5	protein_coding	[illegible]
23877	NM_001110328,NM_001110329,NM_001110330,NM_001360241,NM_001360242,NM_001360243,NM_011813	Fiz1	Flt3 interacting zinc finger protein 1	protein_coding	[illegible]
23879	NM_011814	Fxr2	fragile X mental retardation, autosomal homolog 2	protein_coding	[illegible]
23880	NM_001278269,NM_011815,XM_006519982,XM_006519983,XM_006519985,XM_006519986,XM_011245328,XM_011245329,XM_030248531,XR_003951372,XR_003951373	Fyb	FYN binding protein	protein_coding	[illegible]
23881	NM_001080794,NM_001080795,NM_001080796,NM_001080797,NM_001359195,NM_001359196,NM_001359197,NM_011816,XM_030254467	G3bp2	GTPase activating protein (SH3 domain) binding protein 2	protein_coding	[illegible]
23882	NM_011817	Gadd45g	growth arrest and DNA-damage-inducible 45 gamma	protein_coding	[illegible]
23885	NM_011818,XM_006506056,XR_003956194,XR_377446	Gmcl1	germ cell-less, spermatogenesis associated 1	protein_coding	[illegible]
23886	NM_001330687,NM_011819,XM_011242304	Gdf15	growth differentiation factor 15	protein_coding	[illegible]
23887	NM_001359425,NM_011820,NR_153110,XM_006513647,XM_030245090,XR_001779525	Ggt5	gamma-glutamyltransferase 5	protein_coding	[illegible]
23888	NM_001079844,NM_011821,XM_006518929	Gpc6	glypican 6	protein_coding	[illegible]
23890	NM_011823,XM_006527624,XM_006527625	Gpr34	G protein-coupled receptor 34	protein_coding	[illegible]
23892	NM_011824,XM_030250924	Grem1	gremlin 1, DAN family BMP antagonist	protein_coding	[illegible]
23893	NM_011825,XM_006496826,XM_017320448	Grem2	gremlin 2, DAN family BMP antagonist	protein_coding	[illegible]
23894	NM_001360706,NM_022011,NR_153794,XM_006517662,XR_873756,XR_873759	Gtf2h2	general transcription factor II H, polypeptide 2	protein_coding	[illegible]
23897	NM_001282032,NM_001310681,NM_011826,XM_006501446,XM_017319570	Hax1	HCLS1 associated X-1	protein_coding	[illegible]
23900	NM_011827	Hcst	hematopoietic cell signal transducer	protein_coding	[illegible]
23908	NM_001355234,NM_011828,XM_006501450	Hs2st1	heparan sulfate 2-O-sulfotransferase 1	protein_coding	[illegible]
23912	NM_175092,XM_030254468,XM_030254469,XM_030254470,XM_030254471,XM_030254472,XM_030254473,XM_030254474,XM_030254475,XM_030254476,XM_030254477,XM_030254478,XM_030254479,XM_030254480,XM_030254481,XM_030254482,XM_030254483,XM_030254484,XM_030254485,XM_030254486,XM_030254487,XM_030254488,XM_030254489,XM_030254490,XM_030254491,XM_030254492,XM_030254493	Rhof	ras homolog family member F (in filopodia)	protein_coding	[illegible]
23917	NM_001302933,NM_001302934,NM_011829,XM_006505065,XM_006505066,XM_011241058,XM_030255367	Impdh1	inosine monophosphate dehydrogenase 1	protein_coding	[illegible]
23918	NM_011830,XM_006511730	Impdh2	inosine monophosphate dehydrogenase 2	protein_coding	[illegible]
23919	NM_001290648,NM_011831	Insl5	insulin-like 5	protein_coding	[illegible]
23920	NM_011832,XM_006501451,XM_006501452,XM_006501453,XM_006501454	Insrr	insulin receptor-related receptor	protein_coding	[illegible]
23921	NM_001302938,NM_001302939,NM_018825,XM_006504420,XM_006504422,XM_011240882,XM_030254494,XM_030254495	Sh2b2	SH2B adaptor protein 2	protein_coding	[illegible]
23922	NM_206924	Jtb	jumping translocation breakpoint	protein_coding	[illegible]
23923	NM_011834,XM_006509397	Aadat	aminoadipate aminotransferase	protein_coding	[illegible]
23924	NM_011835,XM_030245091	Katna1	katanin p60 (ATPase-containing) subunit A1	protein_coding	[illegible]
23925	NM_032540,XM_006506057,XM_006506058,XM_006506059,XM_011241331,XM_017321572,XM_017321573,XR_001785127,XR_377447,XR_377448	Kel	Kell blood group	protein_coding	[illegible]
23928	NM_011836	Lamc3	laminin gamma 3	protein_coding	[illegible]
23934	NM_001135688,NM_001135689,NM_011837,XM_006520900,XM_006520901,XM_011245606,XM_030248534,XM_030248535,XM_030248536	Ly6h	lymphocyte antigen 6 complex, locus H	protein_coding	[illegible]
23936	NM_011838	Lynx1	Ly6/neurotoxin 1	protein_coding	[illegible]
23937	NM_011839	Mab21l2	mab-21-like 2	protein_coding	[illegible]
23938	NM_001364492,NM_001364493,NM_001364494,NM_011840,XM_006511146,XM_011242740,XM_030244354	Map2k5	mitogen-activated protein kinase kinase 5	protein_coding	[illegible]
23939	NM_001291033,NM_001291034,NM_001291035,NM_001291036,NM_001291037,NM_001361989,NM_011841,XM_006533277,XM_006533280,XM_006533284,XM_006533285,XM_006533286,XM_030245985,XM_030245986,XM_030245987,XM_030245988,XM_030245989,XM_030245990,XM_030245991,XM_030245992,XM_030245993	Mapk7	mitogen-activated protein kinase 7	protein_coding	[illegible]
23942	NM_011842,XM_030250932,XM_030250933	Mta2	metastasis-associated gene family, member 2	protein_coding	[illegible]
23943	NM_011843	Esyt1	extended synaptotagmin-like protein 1	protein_coding	[illegible]
23945	NM_001166249,NM_001166250,NM_001166251,NM_011844,XM_011241332	Mgll	monoglyceride lipase	protein_coding	[illegible]
23947	NM_001358366,NM_001358367,NM_011845,XM_006528524	Mid2	midline 2	protein_coding	[illegible]
23948	NM_011846,XR_001784693	Mmp17	matrix metallopeptidase 17	protein_coding	[illegible]
23950	NM_001037940,NM_001037941,NM_001127367,NM_001359198,NM_011847,NR_152863,XM_006535707	Dnajb6	DnaJ heat shock protein family (Hsp40) member B6	protein_coding	[illegible]
23954	NM_001162947,NM_011848,XM_006509111,XR_003947272,XR_003947273,XR_378785	Nek3	NIMA (never in mitosis gene a)-related expressed kinase 3	protein_coding	[illegible]
23955	NM_001308328,NM_011849,XM_011245068,XM_011245069	Nek4	NIMA (never in mitosis gene a)-related expressed kinase 4	protein_coding	[illegible]
23956	NM_001160163,NM_001160164,NM_001160165,NM_015750,XM_006529524	Neu2	neuraminidase 2	protein_coding	[illegible]
23957	NM_011850	Nr0b2	nuclear receptor subfamily 0, group B, member 2	protein_coding	[illegible]
23958	NM_013708,XM_006511149,XM_006511150,XM_011242742	Nr2e3	nuclear receptor subfamily 2, group E, member 3	protein_coding	[illegible]
23959	NM_011851	Nt5e	5' nucleotidase, ecto	protein_coding	[illegible]
23960	NM_011852,NR_126529	Oas1g	2'-5' oligoadenylate synthetase 1G	protein_coding	[illegible]
23961	NR_003507	Oas1b	2'-5' oligoadenylate synthetase 1B	transcribed_pseudogene	[illegible]
23962	NM_001359181,NM_011854,XM_006530314,XM_006530315	Oasl2	2'-5' oligoadenylate synthetase-like 2	protein_coding	[illegible]
23963	NM_011855,XM_006541491,XM_006541492,XM_011251005,XM_011251006,XM_011251007,XM_017318483,XM_017318484	Tenm1	teneurin transmembrane protein 1	protein_coding	[illegible]
23964	NM_001290702,NM_011856,XM_006533287,XM_006533288,XM_006533289,XM_006533290,XM_006533291,XM_006533292,XM_006533293,XM_006533294,XM_006533295,XM_006533296,XM_006533297,XM_006533298,XM_006533301,XM_011249009,XM_011249010,XM_017314548,XM_017314549,XM_030245994,XM_030245995,XM_030245996	Tenm2	teneurin transmembrane protein 2	protein_coding	[illegible]
23965	NM_001145937,NM_011857,XM_006509404,XM_006509405,XM_006509406,XM_006509407,XM_006509408,XM_006509409,XM_011242216,XM_011242217,XM_011242218,XM_017312772,XM_017312774,XM_017312775,XM_017312776,XM_017312778,XM_017312779,XM_017312780,XM_017312781,XM_017312782,XM_030243523,XM_030243524,XM_030243525,XM_030243526,XM_030243527,XM_030243528	Tenm3	teneurin transmembrane protein 3	protein_coding	[illegible]
23966	NM_001310760,NM_001310762,NM_011858,XM_006507801,XM_006507803,XM_006507804,XM_006507805,XM_006507806,XM_006507807,XM_006507808,XM_006507812,XM_006507813,XM_011241787,XM_011241788,XM_011241789,XM_011241790,XM_011241792,XM_011241793,XM_017322229,XM_030242544,XM_030242545,XM_030242546,XM_030242547,XM_030242548,XM_030242549,XM_030242550,XM_030242551,XM_030242552	Tenm4	teneurin transmembrane protein 4	protein_coding	[illegible]
23967	NM_011859	Osr1	odd-skipped related transcription factor 1	protein_coding	[illegible]
23969	NM_001286743,NM_001286744,NM_011861,NM_178365,XM_006524226,XM_011246431	Pacsin1	protein kinase C and casein kinase substrate in neurons 1	protein_coding	[illegible]
23970	NM_001159509,NM_001159510,NM_001358641,NM_001358642,NM_001358643,NM_001358644,NM_011862,XM_006520980,XM_006520981,XM_006520982,XM_006520983,XM_011245633,XM_030248565,XM_030248566	Pacsin2	protein kinase C and casein kinase substrate in neurons 2	protein_coding	[illegible]
23971	NM_001289477,NM_001289478,NM_001289479,NM_011863,XM_030252605,XM_030252606	Papss1	3'-phosphoadenosine 5'-phosphosulfate synthase 1	protein_coding	[illegible]
23972	NM_001201470,NM_001360403,NM_011864,XM_006527064	Papss2	3'-phosphoadenosine 5'-phosphosulfate synthase 2	protein_coding	[illegible]
23980	NM_018858	Pebp1	phosphatidylethanolamine binding protein 1	protein_coding	[illegible]
23983	NM_011865	Pcbp1	poly(rC) binding protein 1	protein_coding	[illegible]
23984	NM_001290707,NM_001347321,NM_011866,XM_006523316,XM_006523318,XM_006523319,XM_006523320,XM_006523321,XM_006523323,XM_017317460,XM_017317462	Pde10a	phosphodiesterase 10A	protein_coding	[illegible]
23985	NM_011867,XM_006515091,XM_006515092,XM_011243870,XM_017315068,XR_003950047	Slc26a4	solute carrier family 26, member 4	protein_coding	[illegible]
23986	NM_001110331,NM_011868,NR_073427,XM_006516673,XM_006516674,XM_006516675,XM_006516676,XM_006516677,XM_030247264,XM_030247265,XM_030247266,XM_030247267	Eci2	enoyl-Coenzyme A delta isomerase 2	protein_coding	[illegible]
23988	NM_001364495,NM_023371	Pin1	protein (peptidyl-prolyl cis/trans isomerase) NIMA-interacting 1	protein_coding	[illegible]
23989	NM_001361993,NM_001361994,NM_001361995,NM_011869,XM_006533302,XM_030245997	Med24	mediator complex subunit 24	protein_coding	[illegible]
23991	NM_001291275,NM_001291276,NM_011870,XM_011250861,XM_030242553	Cib1	calcium and integrin binding 1 (calmyrin)	protein_coding	[illegible]
23992	NM_011871,XM_017318023,XM_030250934,XM_030250935	Prkra	protein kinase, interferon inducible double stranded RNA dependent	protein_coding	[illegible]
23993	NM_011872	Klk7	kallikrein related-peptidase 7 (chymotryptic, stratum corneum)	protein_coding	[illegible]
23994	NM_011873,XM_006520986	Dazap2	DAZ associated protein 2	protein_coding	[illegible]
23996	NM_011874	Psmc4	proteasome (prosome, macropain) 26S subunit, ATPase, 4	protein_coding	[illegible]
23997	NM_011875	Psmd13	proteasome (prosome, macropain) 26S subunit, non-ATPase, 13	protein_coding	[illegible]
23999	NM_011876	Twf2	twinfilin actin binding protein 2	protein_coding	[illegible]
24000	NM_001146199,NM_011877,XM_011244112	Ptpn21	protein tyrosine phosphatase, non-receptor type 21	protein_coding	[illegible]
24001	NM_001122998,NM_001286757,NM_001286758,NM_011878,XM_006523225,XM_006523226,XM_006523227,XM_006523229,XM_006523230,XM_006523231,XM_006523233,XM_017317463,XM_030249767	Tiam2	T cell lymphoma invasion and metastasis 2	protein_coding	[illegible]
24004	NM_001103367,NM_198409	Rai2	retinoic acid induced 2	protein_coding	[illegible]
24010	NM_011879	Ik	IK cytokine	protein_coding	[illegible]
24012	NM_001199003,NM_001347195,NM_001357129,NM_011880,XM_006496828,XM_006496829,XM_006496830,XM_006496831,XM_006496834,XM_011238808,XM_011238809,XM_017320465,XM_017320468,XM_030253948,XM_030253949	Rgs7	regulator of G protein signaling 7	protein_coding	[illegible]
24013	NM_011881	Grk1	G protein-coupled receptor kinase 1	protein_coding	[illegible]
24014	NM_011882,XM_006529527,XM_006529528,XM_006529530,XM_011247993,XM_011247994,XM_030253950,XM_030253951,XM_030253953	Rnasel	ribonuclease L (2', 5'-oligoisoadenylate synthetase-dependent)	protein_coding	[illegible]
24015	NM_015751,NR_152614	Abce1	ATP-binding cassette, sub-family E (OABP), member 1	protein_coding	[illegible]
24017	NM_001113413,NM_001304454,NM_001304456,NM_001357080,NM_011883,NR_130743,XM_006501458,XM_030252608	Rnf13	ring finger protein 13	protein_coding	[illegible]
24018	NM_001305273,NM_011884,XM_006537889,XM_006537890,XM_011250015,XM_017320189,XM_030253517	Rngtt	RNA guanylyltransferase and 5'-phosphatase	protein_coding	[illegible]
24030	NM_001360250,NM_001360252,NM_001360254,NM_011885	Mrps12	mitochondrial ribosomal protein S12	protein_coding	[illegible]
24044	NM_022813,XM_006511152	Scamp2	secretory carrier membrane protein 2	protein_coding	[illegible]
24045	NM_001309909,NM_001309910,NM_011886	Scamp3	secretory carrier membrane protein 3	protein_coding	[illegible]
24046	NM_011887,XM_017313362	Scn11a	sodium channel, voltage-gated, type XI, alpha	protein_coding	[illegible]
24047	NM_011888	Ccl19	chemokine (C-C motif) ligand 19	protein_coding	[illegible]
24050	NM_001358836,NM_001358837,NM_001368642,NM_001368643,NM_001368644,NM_001368645,NM_001368646,NM_001368647,NM_001368784,NM_001368785,NM_011889,XM_017316609,XM_030248568,XM_030248569,XM_030248570,XM_030248571	Sept3	septin 3	protein_coding	[illegible]
24051	NM_011890	Sgcb	sarcoglycan, beta (dystrophin-associated glycoprotein)	protein_coding	[illegible]
24052	NM_011891,XM_006533306,XM_006533307,XM_011249012,XM_011249013	Sgcd	sarcoglycan, delta (dystrophin-associated glycoprotein)	protein_coding	[illegible]
24053	NM_011892,XM_006519012	Sgcg	sarcoglycan, gamma (dystrophin-associated glycoprotein)	protein_coding	[illegible]
24055	NM_001145858,NM_001145859,NM_011893,XM_006503954,XM_006503955	Sh3bp2	SH3-domain binding protein 2	protein_coding	[illegible]
24056	NM_001347585,NM_011894,XM_006519015,XM_030247814,XM_030247815	Sh3bp5	SH3-domain binding protein 5 (BTK-associated)	protein_coding	[illegible]
24057	NM_001289480,NM_013709,NR_110343,XR_003950048,XR_87240	Sh3yl1	Sh3 domain YSC-like 1	protein_coding	[illegible]
24058	NM_001355055,NM_023059,XM_006536175,XM_006536177,XM_006536178,XM_006536179,XM_006536180,XM_011249842,XM_030242554	Sigirr	single immunoglobulin and toll-interleukin 1 receptor (TIR) domain	protein_coding	[illegible]
24059	NM_033314,NR_157194,XM_006511731	Slco2a1	solute carrier organic anion transporter family, member 2a1	protein_coding	[illegible]
24060	NM_011895,XM_006537891,XM_006537892,XR_001784138	Slc35a1	solute carrier family 35 (CMP-sialic acid transporter), member 1	protein_coding	[illegible]
24061	NM_019710,XM_006528840	Smc1a	structural maintenance of chromosomes 1A	protein_coding	[illegible]
24063	NM_001305440,NM_001305441,NM_001305442,NM_011896,XM_030252610	Spry1	sprouty RTK signaling antagonist 1	protein_coding	[illegible]
24064	NM_011897,XM_006519016	Spry2	sprouty RTK signaling antagonist 2	protein_coding	[illegible]
24066	NM_011898	Spry4	sprouty RTK signaling antagonist 4	protein_coding	[illegible]
24067	NM_011899,XM_030246710	Srp54a	signal recognition particle 54A	protein_coding	[illegible]
24068	NM_001164406,NM_025291,NR_028355	Sra1	steroid receptor RNA activator 1	protein_coding	[illegible]
24069	NM_001025391,NM_015752	Sufu	SUFU negative regulator of hedgehog signaling	protein_coding	[illegible]
24070	NM_001301710,NM_001301711,NM_011900,NR_125917	Mpdu1	mannose-P-dolichol utilization defect 1	protein_coding	[illegible]

24071	NM_001309814,NM_025292,NR_132136,NR_132142	Synj2bp	synaptojanin 2 binding protein	protein_coding	NP_001296743.1;NP_079568.1	1344347	.	4.464324
24074	NM_175770	Taf7	TATA-box binding protein associated factor 7	protein_coding	NP_786964.1	1346348	.	3.632150
24075	NM_020024,XM_006507814	Taf10	TATA-box binding protein associated factor 10	protein_coding	NP_064408.2;XP_006507877.1	1346320	.	19.280592
24082	NR_027800	Map2k3os	mitogen-activated protein kinase kinase 3, opposite strand	lncRNA		1344334	.	0.984081
24083	NM_025294	Natd1	N-acetyltransferase domain containing 1	protein_coding	NP_079570.1	1344388	.	8.644878
24084	NM_001357262,NM_001357263,NM_011902	Tekt2	tektin 2	protein_coding	NP_001344191.1;NP_001344192.1;NP_036032.2	1346335	.	2.897431
24086	NM_001112705,NM_001294329,NM_001294330,NM_001294331,NM_001294334,NM_001361996,XM_006533312,XM_006533313,XM_006533314,XM_006533315,XM_006533316,XM_006533320,XM_011249014,XM_017314550,XM_017314551,XM_030245998,XM_030245999,XM_030246000,XM_030246001,XM_030246002	Tlk2	tousled-like kinase 2 (Arabidopsis)	protein_coding	NP_001106176.1;NP_001281258.1;NP_001281259.1;NP_001281260.1;NP_001281263.1;NP_001348925.1;XP_006533375.1;XP_006533376.1;XP_006533377.2;XP_006533378.4;XP_006533379.2;XP_006533383.1;XP_011247316.1;XP_017170039.2;XP_017170040.2;XP_030101858.1;XP_030101859.1;XP_030101860.1;XP_030101861.1;XP_030101862.1	1346023	.	13.600770
24087	NM_011904,XM_006527092,XM_006527095,XM_017318201	Tll2	tolloid-like 2	protein_coding	NP_036034.1;XP_006527155.1;XP_006527158.1;XP_017173690.1	1346044	.	0.000000
24088	NM_011905,XM_006501460,XM_030252611	Tlr2	toll-like receptor 2	protein_coding	NP_036035.3;XP_006501523.1;XP_030108471.1	1346060	.	2.306271
24099	NM_001347309,NM_033622,XM_006508775,XR_378661	Tnfsf13b	tumor necrosis factor (ligand) superfamily, member 13b	protein_coding	NP_001334238.1;NP_296371.1;XP_006508838.1	1344376	.	1.309721
24100	NM_011906,XM_006506061,XM_006506062,XM_006506063,XM_006506064,XR_377450	Tpra1	transmembrane protein, adipocyte asscociated 1	protein_coding	NP_036036.2;XP_006506124.1;XP_006506125.1;XP_006506126.1;XP_006506127.1	1345190	.	9.050306
24102	NM_011907	Trex2	three prime repair exonuclease 2	protein_coding	NP_036037.1	1346343	.	0.000000
24105	NM_001083921,NM_001355282,NM_019705,XM_006499445,XM_006499446,XM_006499447,XM_030250944,XM_030250945,XM_030250946	Rbck1	RanBP-type and C3HC4-type zinc finger containing 1	protein_coding	NP_001077390.1;NP_001342211.1;NP_062679.2;XP_006499508.1;XP_006499509.1;XP_006499510.1;XP_030106804.1;XP_030106805.1;XP_030106806.1	1344372	.	24.323025
24109	NM_001359199,NM_001359200,NM_001359201,NM_001359202,NM_011908,XM_006504832	Ubl3	ubiquitin-like 3	protein_coding	NP_001346128.1;NP_001346129.1;NP_001346130.1;NP_001346131.1;NP_036038.1;XP_006504895.1	1344373	.	14.122116
24110	NM_011909	Usp18	ubiquitin specific peptidase 18	protein_coding	NP_036039.2	1344364	.	0.718859
24115	NM_011913	Best1	bestrophin 1	protein_coding	NP_036043.2	1346332	.	0.878128
24116	NM_011914,XM_030254497	Nelfa	negative elongation factor complex member A, Whsc2	protein_coding	NP_036044.1;XP_030110357.1	1346098	.	11.135109
24117	NM_011915	Wif1	Wnt inhibitory factor 1	protein_coding	NP_036045.1	1344332	.	144.143738
24127	NM_001311130,NM_011916,XM_006511154,XM_006511155,XM_006511156,XM_011242743,XR_001778898	Xrn1	5'-3' exoribonuclease 1	protein_coding	NP_001298059.1;NP_036046.2;XP_006511217.1;XP_006511218.1;XP_006511219.1;XP_011241045.1	891964	.	7.894552
24128	NM_001356402,NM_001356403,NM_011917,XM_011239519,XM_030250983	Xrn2	5'-3' exoribonuclease 2	protein_coding	NP_001343331.1;NP_001343332.1;NP_036047.2;XP_011237821.1;XP_030106843.1	894687	.	21.046489
24131	NM_001039071,NM_001039072,NM_001039073,NM_001039074,NM_001039075,NM_001039076,NM_011918,XM_006519017,XM_006519018,XM_006519019,XM_017316019	Ldb3	LIM domain binding 3	protein_coding	NP_001034160.1;NP_001034161.1;NP_001034162.1;NP_001034163.1;NP_001034164.1;NP_001034165.1;NP_036048.3;XP_006519080.1;XP_006519081.1;XP_006519082.1;XP_017171508.1	1344412	.	12.244108
24132	NM_013843,XM_030249806,XM_030249807,XM_030249808,XM_030249809,XR_003952122	Zfp53	zinc finger protein 53	protein_coding	NP_038871.1;XP_030105666.1;XP_030105667.1;XP_030105668.1;XP_030105669.1	99200	.	1.998696
24135	NM_001044747,NM_001163797,NM_013844	Zfp68	zinc finger protein 68	protein_coding	NP_001038212.1;NP_001157269.1;NP_038872.1	1344427	.	10.403396
24136	NM_001289521,NM_001355288,NM_001355289,NM_001355290,NM_001355291,NM_015753,NR_149354	Zeb2	zinc finger E-box binding homeobox 2	protein_coding	NP_001276450.1;NP_001342217.1;NP_001342218.1;NP_001342219.1;NP_001342220.1;NP_056568.2	1344407	.	14.513578
26356	NM_001302451,NM_001302457,NM_001302458,NM_001302459,NM_001302460,NM_011919	Ing1	inhibitor of growth family, member 1	protein_coding	NP_001289380.1;NP_001289386.1;NP_001289387.1;NP_001289388.1;NP_001289389.1;NP_036049.2	1349481	.	15.482977
26357	NM_001355477,NM_011920,XM_006506148,XM_006506149,XM_006506150,XM_006506151,XM_011241362,XM_030255399,XM_030255400,XM_030255402	Abcg2	ATP binding cassette subfamily G member 2 (Junior blood group)	protein_coding	NP_001342406.1;NP_036050.1;XP_006506211.1;XP_006506212.1;XP_006506213.1;XP_006506214.1;XP_011239664.1;XP_030111259.1;XP_030111260.1;XP_030111262.1	1347061	.	11.606359
26358	NM_011921	Aldh1a7	aldehyde dehydrogenase family 1, subfamily A7	protein_coding	NP_036051.1	1347050	.	0.183763
26360	NM_011923,XM_006498051	Angptl2	angiopoietin-like 2	protein_coding	NP_036053.2;XP_006498114.1	1347002	.	145.026251
26361	NM_011924	Avpr1b	arginine vasopressin receptor 1B	protein_coding	NP_036054.1	1347010	.	0.000000
26362	NM_001190974,NM_001190975,NM_009465,XM_006539989,XM_006539991,XM_006539992,XM_006539993	Axl	AXL receptor tyrosine kinase	protein_coding	NP_001177903.1;NP_001177904.1;NP_033491.2;XP_006540052.1;XP_006540054.1;XP_006540055.1;XP_006540056.1	1347244	.	22.879240
26363	NM_025295,XM_006519029	Btd	biotinidase	protein_coding	NP_079571.1;XP_006519092.1	1347001	.	19.088925
26364	NM_001163029,NM_001163030,NM_001163031,NM_011925,XM_006531033,XM_006531035,XM_017312836	Adgre5	adhesion G protein-coupled receptor E5	protein_coding	NP_001156501.1;NP_001156502.1;NP_001156503.1;NP_036055.2;XP_006531096.1;XP_006531098.1;XP_017168325.1	1347095	.	23.791760
26365	NM_001039185,NM_001039186,NM_001039187,NM_011926,XM_030242604	Ceacam1	carcinoembryonic antigen-related cell adhesion molecule 1	protein_coding	NP_001034274.1;NP_001034275.1;NP_001034276.1;NP_036056.2;XP_030098464.1	1347245	.	2.068482
26366	NM_007675,XM_006539994,XM_030242605	Ceacam10	carcinoembryonic antigen-related cell adhesion molecule 10	protein_coding	NP_031701.3;XP_006540057.1;XP_030098465.1	1347248	.	0.000000
26367	NM_001113368,NM_001113369,NM_007543	Ceacam2	carcinoembryonic antigen-related cell adhesion molecule 2	protein_coding	NP_001106839.1;NP_001106840.1;NP_031569.1	1347246	.	0.244911
26370	NM_019405	Cetn2	centrin 2	protein_coding	NP_062278.2	1347085	.	13.445291
26371	NM_025296	Ciao1	cytosolic iron-sulfur protein assembly 1	protein_coding	NP_079572.2	1346998	.	8.191618
26372	NM_011929	Clcn6	chloride channel, voltage-sensitive 6	protein_coding	NP_036059.1	1347049	.	6.966215
26373	NM_001317404,NM_011930,XR_001782075,XR_003952123,XR_385328	Clcn7	chloride channel, voltage-sensitive 7	protein_coding	NP_001304333.1;NP_036060.1	1347048	.	13.110055
26374	NM_001360878,NM_011931,XM_006496860,XM_030254393,XM_030254396,XM_030254397	Cop1	COP1, E3 ubiquitin ligase	protein_coding	NP_001347807.1;NP_036061.1;XP_006496923.1;XP_030110253.1;XP_030110256.1;XP_030110257.1	1347046	.	5.378156
26377	NM_001310753,NM_011932,XM_017319585	Dapp1	dual adaptor for phosphotyrosine and 3-phosphoinositides 1	protein_coding	NP_001297682.1;NP_036062.1;XP_017175074.1	1347063	.	4.111583
26378	NM_011933,XM_030249816	Decr2	2-4-dienoyl-Coenzyme A reductase 2, peroxisomal	protein_coding	NP_036063.1;XP_030105676.1	1347059	.	8.594232
26379	NM_007953,XM_006527111,XM_017318209,XM_030250949,XM_030250950	Esrra	estrogen related receptor, alpha	protein_coding	NP_031979.2;XP_006527174.1;XP_017173698.1;XP_030106809.1;XP_030106810.1	1346831	.	6.035626
26380	NM_001159500,NM_011934,XM_006515881,XM_006515882,XM_006515883	Esrrb	estrogen related receptor, beta	protein_coding	NP_001152972.1;NP_036064.3;XP_006515944.1;XP_006515945.1;XP_006515946.1	1346832	.	0.109908
26381	NM_001243792,NM_001357534,NM_001357535,NM_001357536,NM_011935,XM_006497165,XM_006497170,XM_011238915,XM_011238916,XM_011238917,XM_011238918,XM_011238919,XM_011238920,XM_030254422,XM_030254424	Esrrg	estrogen-related receptor gamma	protein_coding	NP_001230721.1;NP_001344463.1;NP_001344464.1;NP_001344465.1;NP_036065.1;XP_006497228.1;XP_006497233.1;XP_011237217.1;XP_011237218.1;XP_011237219.1;XP_011237220.1;XP_011237221.1;XP_011237222.1;XP_030110282.1;XP_030110284.1	1347056	.	0.310125
26382	NM_001159538,NM_013710,XM_006524299,XM_006524300,XM_011246472,XM_011246473,XM_017317478,XM_030249817,XM_030249818,XR_003952124,XR_003952125,XR_003952126,XR_003952127,XR_003952128,XR_385330,XR_385331	Fgd2	FYVE, RhoGEF and PH domain containing 2	protein_coding	NP_001153010.1;NP_038738.2;XP_006524362.1;XP_006524363.1;XP_011244774.1;XP_011244775.1;XP_017172967.1;XP_030105677.1;XP_030105678.1	1347084	.	2.013809
26383	NM_011936,XM_006531036	Fto	fat mass and obesity associated	protein_coding	NP_036066.2;XP_006531099.1	1347093	.	32.240674
26384	NM_011937	Gnpda1	glucosamine-6-phosphate deaminase 1	protein_coding	NP_036067.2	1347054	.	8.603347
26385	NM_001038018,NM_001112711,NM_001286063,NM_001286064,NM_001286065,NM_001286066,NM_011938,XM_030247269,XM_030247270	Grk6	G protein-coupled receptor kinase 6	protein_coding	NP_001033107.1;NP_001106182.1;NP_001272992.1;NP_001272993.1;NP_001272994.1;NP_001272995.1;NP_036068.2;XP_030103129.1;XP_030103130.1	1347078	.	16.716826
26386	NM_001256042,NM_001256044,NM_011939,NR_045689,XM_006531038,XM_006531039,XM_006531040,XM_006531041,XM_017312837,XR_003947285	Hsf4	heat shock transcription factor 4	protein_coding	NP_001242971.1;NP_001242973.1;NP_036069.1;XP_006531101.1;XP_006531102.1;XP_006531103.1;XP_006531104.1;XP_017168326.1	1347058	.	0.573766
26388	NM_008327,NM_011940	Ifi202b	interferon activated gene 202B	protein_coding	NP_032353.2;NP_036070.2	1347083	.	0.239375
26390	NM_001355293,NM_011941,XM_017318653,XM_030251566	Mapkbp1	mitogen-activated protein kinase binding protein 1	protein_coding	NP_001342222.1;NP_036071.3;XP_017174142.1;XP_030107426.1	1347004	.	9.405012
26394	NM_011942,XM_006538878	Lypla2	lysophospholipase 2	protein_coding	NP_036072.1;XP_006538941.1	1347000	.	22.946819
26395	NM_008927,XM_006511196	Map2k1	mitogen-activated protein kinase kinase 1	protein_coding	NP_032953.1;XP_006511259.1	1346866	.	16.922382
26396	NM_001347144,NM_001358539,NM_023138,XM_006513683	Map2k2	mitogen-activated protein kinase kinase 2	protein_coding	NP_001334073.1;NP_001345468.1;NP_075627.2;XP_006513746.1	1346867	.	26.810808
26397	NM_008928,NR_157293,XM_006533368	Map2k3	mitogen-activated protein kinase kinase 3	protein_coding	NP_032954.1;XP_006533431.1	1346868	.	23.034489
26398	NM_001316367,NM_001316368,NM_001316369,NM_001362739,NM_001362740,NM_009157,XM_011249033,XM_030246018	Map2k4	mitogen-activated protein kinase kinase 4	protein_coding	NP_001303296.1;NP_001303297.1;NP_001303298.1;NP_001349668.1;NP_001349669.1;NP_033183.1;XP_011247335.1;XP_030101878.1	1346869	.	18.571521
26399	NM_001356345,NM_001356346,NM_001356347,NM_001356352,NM_001356353,NM_011943,XM_011249034,XM_011249035	Map2k6	mitogen-activated protein kinase kinase 6	protein_coding	NP_001343274.1;NP_001343275.1;NP_001343276.1;NP_001343281.1;NP_001343282.1;NP_036073.1;XP_011247336.1;XP_011247337.1	1346870	.	5.727309
26400	NM_001042557,NM_001164172,NM_001291777,NM_001291778,NM_001291783,NM_011944	Map2k7	mitogen-activated protein kinase kinase 7	protein_coding	NP_001036022.1;NP_001157644.1;NP_001278706.1;NP_001278707.1;NP_001278712.1;NP_036074.2	1346871	.	20.031336
26401	NM_011945,XM_017315516,XM_017315517	Map3k1	mitogen-activated protein kinase kinase kinase 1	protein_coding	NP_036075.2;XP_017171005.1;XP_017171006.1	1346872	.	4.543729
26403	NM_022012,XM_006531752,XM_006531753,XM_030250951,XM_030250952,XM_030250953	Map3k11	mitogen-activated protein kinase kinase kinase 11	protein_coding	NP_071295.2;XP_006531815.1;XP_006531816.1;XP_030106811.1;XP_030106812.1;XP_030106813.1	1346880	.	8.251423
26404	NM_001163643,NM_001358844,NM_009582,XM_006521013,XM_017316624,XM_017316625,XM_017316626,XM_030248589,XM_030248590,XM_030248591	Map3k12	mitogen-activated protein kinase kinase kinase 12	protein_coding	NP_001157115.1;NP_001345773.1;NP_033608.3;XP_006521076.1;XP_017172113.1;XP_017172114.1;XP_017172115.1;XP_030104449.1;XP_030104450.1;XP_030104451.1	1346881	.	5.175063
26405	NM_011946	Map3k2	mitogen-activated protein kinase kinase kinase 2	protein_coding	NP_036076.2	1346873	.	5.468640
26406	NM_011947,XM_011249037,XM_030246020	Map3k3	mitogen-activated protein kinase kinase kinase 3	protein_coding	NP_036077.1;XP_011247339.1;XP_030101880.1	1346874	.	16.318870
26407	NM_001357722,NM_011948,XM_030249819,XM_030249820	Map3k4	mitogen-activated protein kinase kinase kinase 4	protein_coding	NP_001344651.1;NP_036078.2;XP_030105679.1;XP_030105680.1	1346875	.	6.806229
26408	NM_008580,XM_006512739	Map3k5	mitogen-activated protein kinase kinase kinase 5	protein_coding	NP_032606.4;XP_006512802.1	1346876	.	3.333582
26409	NM_009316,NM_172688,XM_006537948,XR_390323	Map3k7	mitogen-activated protein kinase kinase kinase 7	protein_coding	NP_033342.1;NP_766276.1;XP_006538011.1	1346877	.	17.949694
26410	NM_007746,NR_157375,XM_006525944,XR_003952522	Map3k8	mitogen-activated protein kinase kinase kinase 8	protein_coding	NP_031772.1;XP_006526007.1	1346878	.	2.433214
26411	NM_001355054,NM_008279,XM_006539995,XM_006539996,XM_006539997,XM_006539998,XM_006540000,XM_017322280	Map4k1	mitogen-activated protein kinase kinase kinase kinase 1	protein_coding	NP_001341983.1;NP_032305.2;XP_006540058.1;XP_006540059.1;XP_006540060.1;XP_006540061.1;XP_006540063.1;XP_017177769.1	1346882	.	0.527562
26412	NM_001291787,NM_009006,NR_117093,XM_006531755,XM_006531756,XM_006531757,XM_006531760,XM_017318211,XM_017318212,XR_001782591	Map4k2	mitogen-activated protein kinase kinase kinase kinase 2	protein_coding	NP_001278716.1;NP_033032.1;XP_006531818.1;XP_006531819.1;XP_006531820.1;XP_006531823.1;XP_017173700.1;XP_017173701.1	1346883	.	7.424596
26413	NM_001038663,NM_001357115,NM_011949	Mapk1	mitogen-activated protein kinase 1	protein_coding	NP_001033752.1;NP_001344044.1;NP_036079.1	1346858	.	28.457525
26414	NM_001081567,NM_001310683,NM_001310685,NM_001310686,NM_001318102,NM_001318131,NM_009158,XM_006534950,XM_011249450,XM_017320886,XM_017320888,XM_017320889,XM_017320890,XM_017320891,XM_030254516,XM_030254518,XM_030254519	Mapk10	mitogen-activated protein kinase 10	protein_coding	NP_001075036.1;NP_001297612.1;NP_001297614.1;NP_001297615.1;NP_001305031.1;NP_001305060.1;NP_033184.2;XP_006535013.1;XP_011247752.1;XP_017176375.1;XP_017176377.1;XP_017176378.1;XP_017176379.1;XP_017176380.1;XP_030110376.1;XP_030110378.1;XP_030110379.1	1346863	.	0.032488
26415	NM_011950,XM_017317479	Mapk13	mitogen-activated protein kinase 13	protein_coding	NP_036080.2;XP_017172968.1	1346864	.	2.844933
26416	NM_001168508,NM_001168513,NM_001168514,NM_001357724,NM_011951	Mapk14	mitogen-activated protein kinase 14	protein_coding	NP_001161980.1;NP_001161985.1;NP_001161986.1;NP_001344653.1;NP_036081.1	1346865	.	23.050269
26417	NM_011952	Mapk3	mitogen-activated protein kinase 3	protein_coding	NP_036082.1	1346859	.	40.572380
26419	NM_001310452,NM_001310453,NM_001310454,NM_016700,XM_006519032,XM_030247820,XM_030247821	Mapk8	mitogen-activated protein kinase 8	protein_coding	NP_001297381.1;NP_001297382.1;NP_001297383.1;NP_057909.1;XP_006519095.1;XP_030103680.1;XP_030103681.1	1346861	.	7.690097
26420	NM_001163671,NM_001163672,NM_016961,NM_207692,XM_017314562,XM_017314563,XM_030246022,XM_030246023	Mapk9	mitogen-activated protein kinase 9	protein_coding	NP_001157143.1;NP_001157144.1;NP_058657.1;NP_997575.2;XP_017170051.1;XP_017170052.1;XP_030101882.1;XP_030101883.1	1346862	.	11.570866
26422	NM_030595,XM_006501519,XM_006501520,XM_006501521,XM_006501522,XM_006501523,XM_006501525,XM_006501526,XM_006501527,XM_011240127,XM_017319586,XM_017319587,XM_030252626,XR_001783693	Nbea	neurobeachin	protein_coding	NP_085098.1;XP_006501582.1;XP_006501583.1;XP_006501584.1;XP_006501585.1;XP_006501586.1;XP_006501588.1;XP_006501589.1;XP_006501590.1;XP_011238429.1;XP_017175075.1;XP_017175076.1;XP_030108486.1	1347075	.	2.871860
26423	NM_001316687,NM_139051	Nr5a1	nuclear receptor subfamily 5, group A, member 1	protein_coding	NP_001303616.1;NP_620639.1	1346833	.	0.030082
26424	NM_001159769,NM_030676,XM_006529621,XM_006529622	Nr5a2	nuclear receptor subfamily 5, group A, member 2	protein_coding	NP_001153241.1;NP_109601.1;XP_006529684.1;XP_006529685.1	1346834	.	0.390502
26425	NM_011955,XR_384565	Nubp1	nucleotide binding protein 1	protein_coding	NP_036085.1	1347073	.	7.077499
26426	NM_001355396,NM_011956,XM_006524306,XM_006524307,XM_006524309,XM_030249821	Nubp2	nucleotide binding protein 2	protein_coding	NP_001342325.1;NP_036086.1;XP_006524369.3;XP_006524370.1;XP_006524372.1;XP_030105681.1	1347072	.	9.880823
26427	NM_011957	Creb3l1	cAMP responsive element binding protein 3-like 1	protein_coding	NP_036087.2	1347062	.	62.669994
26428	NM_001177313,NM_001355296,NM_011958,NR_033442,NR_149355,NR_149356,XM_006498063,XM_006498064,XM_006498066,XM_030251581,XM_030251584,XM_030251588,XR_001782769,XR_001782774,XR_374094	Orc4	origin recognition complex, subunit 4	protein_coding	NP_001170784.1;NP_001342225.1;NP_036088.3;XP_006498126.1;XP_006498127.1;XP_006498129.1;XP_030107441.1;XP_030107444.1;XP_030107448.1	1347043	.	7.658572
26429	NM_011959,XM_006535716	Orc5	origin recognition complex, subunit 5	protein_coding	NP_036089.1;XP_006535779.1	1347044	.	8.722291
26430	NM_001359915,NM_011960,XM_030247822,XM_030247823,XM_030247824	Parg	poly (ADP-ribose) glycohydrolase	protein_coding	NP_001346844.1;NP_036090.2;XP_030103682.1;XP_030103683.1;XP_030103684.1	1347094	.	6.504803
26431	NM_001077359,NM_001077360,NM_001347400,NM_019834,XM_006530331,XM_006530333,XM_006530334,XM_006530335,XM_006530336,XM_006530337,XM_006530338,XM_006530339,XM_017320893,XM_030254520,XM_030254522,XM_030254523,XM_030254524,XM_030254525,XM_030254526,XM_030254527,XM_030254528,XM_030254529,XM_030254530	Git2	GIT ArfGAP 2	protein_coding	NP_001070827.1;NP_001070828.1;NP_001334329.1;NP_062808.3;XP_006530394.1;XP_006530396.1;XP_006530397.1;XP_006530398.1;XP_006530399.1;XP_006530400.1;XP_006530401.1;XP_006530402.1;XP_017176382.1;XP_030110380.1;XP_030110382.1;XP_030110383.1;XP_030110384.1;XP_030110385.1;XP_030110386.1;XP_030110387.1;XP_030110388.1;XP_030110389.1;XP_030110390.1	1347053	.	10.135493
26432	NM_001142916,NM_011961,XM_030244393	Plod2	procollagen lysine, 2-oxoglutarate 5-dioxygenase 2	protein_coding	NP_001136388.1;NP_036091.2;XP_030100253.1	1347007	.	61.205282
26433	NM_011962	Plod3	procollagen-lysine, 2-oxoglutarate 5-dioxygenase 3	protein_coding	NP_036092.1	1347008	.	40.916447
26434	NM_001126338,NM_001278257,NM_001278520,NM_023043	Prnd	prion like protein doppel	protein_coding	NP_001119810.1;NP_001265186.1;NP_001265449.1;NP_075530.1	1346999	.	8.339092
26436	NM_001310628,NM_007676,XM_030242606,XM_030242607	Psg16	pregnancy specific glycoprotein 16	protein_coding	NP_001297557.1;NP_031702.3;XP_030098466.1;XP_030098467.1	1347249	.	0.031945
26437	NM_007677	Psg17	pregnancy specific glycoprotein 17	protein_coding	NP_031703.1	1347250	.	0.045243
26440	NM_011965	Psma1	proteasome (prosome, macropain) subunit, alpha type 1	protein_coding	NP_036095.1	1347005	.	47.647850
26441	NM_011966,XM_006511198	Psma4	proteasome (prosome, macropain) subunit, alpha type 4	protein_coding	NP_036096.1;XP_006511261.1	1347060	.	77.989869
26442	NM_011967	Psma5	proteasome (prosome, macropain) subunit, alpha type 5	protein_coding	NP_036097.1	1347009	.	50.453426
26443	NM_001310583,NM_011968	Psma6	proteasome (prosome, macropain) subunit, alpha type 6	protein_coding	NP_001297512.1;NP_036098.1	1347006	.	60.960580
26444	NM_001289476,NM_011969	Psma7	proteasome (prosome, macropain) subunit, alpha type 7	protein_coding	NP_001276405.1;NP_036099.1	1347070	.	75.482335
26445	NM_011970,XM_006503117	Psmb2	proteasome (prosome, macropain) subunit, beta type 2	protein_coding	NP_036100.3;XP_006503180.1	1347045	.	65.138644
26446	NM_011971	Psmb3	proteasome (prosome, macropain) subunit, beta type 3	protein_coding	NP_036101.1	1347014	.	65.922325
26447	NM_001136090,NM_001289515,NM_001289516,NM_011972,XM_006525945,XM_006525946,XM_011246933,XM_011246934,XM_017317910,XM_017317911,XM_030250465,XM_030250466,XM_030250467,XR_001782369	Poli	polymerase (DNA directed), iota	protein_coding	NP_001129562.1;NP_001276444.1;NP_001276445.1;NP_036102.2;XP_006526008.1;XP_006526009.1;XP_011245235.1;XP_011245236.1;XP_017173399.1;XP_017173400.1;XP_030106325.1;XP_030106326.1;XP_030106327.1	1347081	.	2.471787
26448	NM_011973,XM_006515885,XM_006515886,XM_006515887,XM_006515889,XM_006515890,XM_006515891,XM_006515892,XM_006515893,XM_011244115,XM_011244116,XM_017315070,XM_017315071,XM_030246711,XM_030246712,XM_030246713,XM_030246715,XM_030246716,XM_030246717,XM_030246718,XM_030246719,XM_030246720,XM_030246721,XM_030246722,XM_030246723,XM_030246724,XM_030246725,XM_030246726,XR_003950049,XR_003950050,XR_003950051,XR_003950052	Mok	MOK protein kinase	protein_coding	NP_036103.1;XP_006515948.1;XP_006515949.1;XP_006515950.1;XP_006515952.1;XP_006515953.1;XP_006515954.1;XP_006515955.1;XP_006515956.1;XP_011242417.1;XP_011242418.1;XP_017170559.1;XP_017170560.1;XP_030102571.1;XP_030102572.1;XP_030102573.1;XP_030102575.1;XP_030102576.1;XP_030102577.1;XP_030102578.1;XP_030102579.1;XP_030102580.1;XP_030102581.1;XP_030102582.1;XP_030102583.1;XP_030102584.1;XP_030102585.1;XP_030102586.1	1336881	.	1.407963
26450	NM_015754	Rbbp9	retinoblastoma binding protein 9, serine hydrolase	protein_coding	NP_056569.2	1347074	.	16.911369
26451	NM_011975	Rpl27a	ribosomal protein L27A	protein_coding	NP_036105.2	1347076	.	411.027802
26456	NM_011976,XR_003952792,XR_386476,XR_386477,XR_386478,XR_386479	Sema4g	sema domain, immunoglobulin domain (Ig), transmembrane domain (TM) and short cytoplasmic domain, (semaphorin) 4G	protein_coding	NP_036106.1	1347047	.	2.340018
26457	NM_001357180,NM_001357181,NM_001357182,NM_011977,XM_006509671,XM_006509674,XM_006509675,XM_006509677,XM_006509678,XM_030243574,XM_030243575,XM_030243576,XM_030243578,XM_030243579,XM_030243580,XM_030243581,XM_030243582,XM_030243583,XM_030243585,XM_030243586	Slc27a1	solute carrier family 27 (fatty acid transporter), member 1	protein_coding	NP_001344109.1;NP_001344110.1;NP_001344111.1;NP_036107.1;XP_006509734.1;XP_006509737.1;XP_006509738.1;XP_006509740.1;XP_006509741.1;XP_030099434.1;XP_030099435.1;XP_030099436.1;XP_030099438.1;XP_030099439.1;XP_030099440.1;XP_030099441.1;XP_030099442.1;XP_030099443.1;XP_030099445.1;XP_030099	1347098	.	11.522964
26458	NM_011978	Slc27a2	solute carrier family 27 (fatty acid transporter), member 2	protein_coding	NP_036108.2	1347099	.	0.000000
26459	NM_009512	Slc27a5	solute carrier family 27 (fatty acid transporter), member 5	protein_coding	NP_033538.2	1347100	.	0.070564
26462	NM_001353143,NM_013711	Txnrd2	thioredoxin reductase 2	protein_coding	NP_001340072.1;NP_038739.2	1347023	.	4.047285
26464	NM_011979	Vnn3	vanin 3	protein_coding	NP_036109.3	1347055	.	0.138187
26465	NM_001357557,NM_011980,XM_006540008,XM_030242608,XM_030242609	Zfp146	zinc finger protein 146	protein_coding	NP_001344486.1;NP_036110.1;XP_006540071.1;XP_030098468.1;XP_030098469.1	1347092	.	31.202717
26466	NM_011981,XM_011250583,XM_011250584	Zfp260	zinc finger protein 260	protein_coding	NP_036111.2;XP_011248885.1;XP_011248886.1	1347071	.	37.920932
26549	NM_013712,XM_030251354,XM_030251355,XM_030251356,XR_003953009,XR_003953010,XR_003953011	Itgb1bp2	integrin beta 1 binding protein 2	protein_coding	NP_038740.1;XP_030107214.1;XP_030107215.1;XP_030107216.1	1353420	.	16.492066
26554	NM_001313728,NM_016716,XM_006496498,XM_030254438,XM_030254443	Cul3	cullin 3	protein_coding	NP_001300657.1;NP_057925.1;XP_006496561.1;XP_030110298.1;XP_030110303.1	1347360	.	18.972752
26556	NM_001284189,NM_001347598,NM_011982,NM_147176,NM_152134	Homer1	homer scaffolding protein 1	protein_coding	NP_001271118.1;NP_001334527.1;NP_036112.1;NP_671705.2;NP_687036.1	1347345	.	2.203659
26557	NM_001164086,NM_001164087,NM_011983,XM_006540909,XM_030242610,XM_030242611	Homer2	homer scaffolding protein 2	protein_coding	NP_001157558.1;NP_001157559.1;NP_036113.1;XP_006540972.1;XP_030098470.1;XP_030098471.1	1347354	.	1.026049
26558	NM_001146153,NM_011984,XM_006509679,XM_006509680,XM_006509681,XM_030243587,XM_030243588,XM_030243589,XR_003947286	Homer3	homer scaffolding protein 3	protein_coding	NP_001139625.1;NP_036114.2;XP_006509742.1;XP_006509743.1;XP_006509744.1;XP_030099447.1;XP_030099448.1;XP_030099449.1	1347359	.	11.941497
26559	NM_015755,XM_011246130,XR_384941	Hunk	hormonally upregulated Neu-associated kinase	protein_coding	NP_056570.1;XP_011244432.1	1347352	.	3.435381
26561	NM_001320235,NM_011985,XM_006538879,XM_006538880,XM_006538881,XM_006538882,XM_006538884	Mmp23	matrix metallopeptidase 23	protein_coding	NP_001307164.1;NP_036115.1;XP_006538942.1;XP_006538943.1;XP_006538944.1;XP_006538945.1;XP_006538947.1	1347361	.	35.884237
26562	NM_001355412,NM_001355413,NM_011986,XM_030253580	Ncdn	neurochondrin	protein_coding	NP_001342341.1;NP_001342342.1;NP_036116.3;XP_030109440.1	1347351	.	8.414662
26563	NM_001312690,NM_013845,XM_006503121	Ror1	receptor tyrosine kinase-like orphan receptor 1	protein_coding	NP_001299619.1;NP_038873.2;XP_006503184.1	1347520	.	2.423055
26564	NM_013846	Ror2	receptor tyrosine kinase-like orphan receptor 2	protein_coding	NP_038874.3	1347521	.	9.937476
26565	NM_001291009,NM_011987,NR_110990,XM_017316987	Pla2g10	phospholipase A2, group X	protein_coding	NP_001277938.1;NP_036117.1;XP_017172476.1	1347522	.	0.251007
26568	NM_001316688,NM_011988,XM_011240129,XM_011240130	Slc27a3	solute carrier family 27 (fatty acid transporter), member 3	protein_coding	NP_001303617.1;NP_036118.2;XP_011238431.1;XP_011238432.1	1347358	.	10.118122
26569	NM_011989	Slc27a4	solute carrier family 27 (fatty acid transporter), member 4	protein_coding	NP_036119.1	1347347	.	6.867299
26570	NM_011990,XM_006500776,XM_006500777,XM_006500778,XM_017319590	Slc7a11	solute carrier family 7 (cationic amino acid transporter, y+ system), member 11	protein_coding	NP_036120.1;XP_006500839.1;XP_006500840.1;XP_006500841.1;XP_017175079.1	1347355	.	0.061489
26572	NM_011991,XM_006533383,XM_030246025,XM_030246026,XM_030246027,XR_003949446	Cops3	COP9 signalosome subunit 3	protein_coding	NP_036121.1;XP_006533446.1;XP_030101885.1;XP_030101886.1;XP_030101887.1	1349409	.	35.017515
26611	NM_001278274,NM_011992	Rcn2	reticulocalbin 2	protein_coding	NP_001265203.1;NP_036122.2	1349765	.	67.286917
26754	NM_001277101,NM_013715,NR_102282	Cops5	COP9 signalosome subunit 5	protein_coding	NP_001264030.1;NP_038743.1	1349415	.	27.180430
26757	NM_011993,XM_006536192,XM_006536193,XM_030242612,XM_030242613	Dpysl4	dihydropyrimidinase-like 4	protein_coding	NP_036123.3;XP_006536255.1;XP_006536256.1;XP_030098472.1;XP_030098473.1	1349764	.	2.804921
26874	NM_001358967,NM_011994	Abcd2	ATP-binding cassette, sub-family D (ALD), member 2	protein_coding	NP_001345896.1;NP_036124.2	1349467	.	1.890912
26875	NM_001110796,NM_011995,XM_006503582,XM_006503583,XM_006503584,XM_006503586,XR_003955647,XR_003955648,XR_003955649,XR_003955650	Pclo	piccolo (presynaptic cytomatrix protein)	protein_coding	NP_001104266.1;NP_036125.4;XP_006503645.2;XP_006503646.2;XP_006503647.2;XP_006503649.2	1349390	.	0.018631
26877	NM_020283,XM_006499591,XM_011239571,XM_011239572,XM_011239573,XM_017318675,XR_003953258	B3galt1	UDP-Gal:betaGlcNAc beta 1,3-galactosyltransferase, polypeptide 1	protein_coding	NP_064679.2;XP_006499654.1;XP_011237873.1;XP_011237874.1;XP_011237875.1;XP_017174164.1	1349403	.	0.865227
26878	NM_020025	B3galt2	UDP-Gal:betaGlcNAc beta 1,3-galactosyltransferase, polypeptide 2	protein_coding	NP_064409.3	1349461	.	1.157676
26879	NM_020026,XM_006501529	B3galnt1	UDP-GalNAc:betaGlcNAc beta 1,3-galactosaminyltransferase,	protein_coding	NP_064410.1;XP_006501592.1	1349405	.	9.314425
26885	NM_001122978,NM_011997,XM_006537949,XM_006537950	Casp8ap2	caspase 8 associated protein 2	protein_coding	NP_001116450.1;NP_036127.2;XP_006538012.1;XP_006538013.1	1349399	.	4.865556
26886	NM_021886,XM_006517678,XM_030247278,XM_030247279,XM_030247280,XR_003950454	Cenph	centromere protein H	protein_coding	NP_068686.1;XP_006517741.1;XP_030103138.1;XP_030103139.1;XP_030103140.1	1349448	.	5.901682
26887	NM_011998,XM_006531043,XM_006531044,XM_006531045	Chst4	carbohydrate sulfotransferase 4	protein_coding	NP_036128.3;XP_006531106.1;XP_006531107.1;XP_006531108.1	1349479	.	0.023478
26888	NM_001170332,NM_001170333,NM_011999,XM_006506155,XM_011241364,XM_030255403	Clec4a2	C-type lectin domain family 4, member a2	protein_coding	NP_001163803.1;NP_001163804.1;NP_036129.1;XP_006506218.1;XP_011239666.1;XP_030111263.1	1349412	.	3.585886
26889	NM_012000,XM_006508806,XM_006508807	Cln8	ceroid-lipofuscinosis, neuronal 8	protein_coding	NP_036130.1;XP_006508869.1;XP_006508870.1	1349447	.	4.409278
26891	NM_001359203,NM_001359204,NM_012001,XM_006534953,XM_006534955	Cops4	COP9 signalosome subunit 4	protein_coding	NP_001346132.1;NP_001346133.1;NP_036131.1;XP_006535016.1;XP_006535018.1	1349414	.	40.980105
26893	NM_012002	Cops6	COP9 signalosome subunit 6	protein_coding	NP_036132.1	1349439	.	47.741417
26894	NM_001164089,NM_001355541,NM_001355542,NM_012003,XM_006506157,XM_006506159,XM_006506160,XM_006506161,XM_011241365,XM_011241366,XM_030255405,XM_030255406	Cops7a	COP9 signalosome subunit 7A	protein_coding	NP_001157561.1;NP_001342470.1;NP_001342471.1;NP_036133.1;XP_006506220.1;XP_006506222.1;XP_006506223.1;XP_006506224.1;XP_011239667.1;XP_011239668.1;XP_030111265.1;XP_030111266.	1349400	.	38.057101
26895	NM_001360887,NM_001360888,NM_172974,XM_006529625,XM_030254496	Cops7b	COP9 signalosome subunit 7B	protein_coding	NP_001347816.1;NP_001347817.1;NP_766562.1;XP_006529688.1;XP_030110356.1	1349388	.	6.425534
26896	NM_001048208,NM_012005,XM_006527629,XM_006527630,XM_017318510,XM_030251357,XR_878029	Med14	mediator complex subunit 14	protein_coding	NP_001041673.1;NP_036135.3;XP_006527692.1;XP_006527693.1;XP_017173999.1;XP_030107217.1	1349442	.	8.330089
26897	NM_012006	Acot1	acyl-CoA thioesterase 1	protein_coding	NP_036136.1	1349396	.	6.451975
26898	NM_001356291,NM_012007	Ctsj	cathepsin J	protein_coding	NP_001343220.1;NP_036137.1	1349426	.	0.000000
26900	NM_012008,XM_006531603,XM_006531607,XM_011248592,XM_030251568	Ddx3y	DEAD (Asp-Glu-Ala-Asp) box polypeptide 3, Y-linked	protein_coding	NP_036138.1;XP_006531666.1;XP_006531670.1;XP_011246894.1;XP_030107428.1	1349406	.	0.018988
26901	NM_026794	Ss18l2	SS18, nBAF chromatin remodeling complex subunit like 2	protein_coding	NP_081070.1	1349474	.	14.492902

26903	NM_001077694,NM_001310152,NM_021469,XM_006506163,XM_006506164,XM_006506165,XM_006506166,XM_006506167,XM_006506168,XM_006506169,XM_006506170,XM_006506171,XM_006506172,XM_006506173,XM_006506174,XM_006506175,XM_006506176,XM_006506177,XM_006506178,XM_006506179,XM_006506181,XM_006506182,XM_006506183,XM_006506184,XM_006506185,XM_017321594,XM_030255407,XR_377460,XR_377461	Dysf	dysferlin	protein_coding	NP_001071162.1;NP_001297081.1;NP_067444.2;XP_006506226.1;XP_006506227.1;XP_006506228.1;XP_006506229.1;XP_006506230.1;XP_006506231.1;XP_006506232.1;XP_006506233.1;XP_006506234.1;XP_006506235.1;XP_006506236.1;XP_006506237.1;XP_006506238.1;XP_006506239.1;XP_006506240.1;XP_006506241.1;XP_006506242.1;XP_006506244.1;XP_006506245.1;XP_006506246.1;XP_006506247.1;XP_006506248.1;XP_017177083.1;XP_030111267.1	1349385	.	6.147268	[illegible]
26904	NM_012009,NR_153837	Sh2d1b1	SH2 domain containing 1B1	protein_coding	NP_036139.3	1349420	.	0.086917	[illegible]
26905	NM_012010	Eif2s3x	eukaryotic translation initiation factor 2, subunit 3, structural gene X-	protein_coding	NP_036140.1	1349431	.	35.440285	[illegible]
26908	NM_001357094,NM_012011,XM_006531610,XM_011248593,XM_030251569,XR_001782883,XR_001782884,XR_001782885,XR_001782886,XR_001782887,XR_003953251,XR_003953252,XR_388245,XR_879402	Eif2s3y	eukaryotic translation initiation factor 2, subunit 3, structural gene Y-linked	protein_coding	NP_001344023.1;NP_036141.1;XP_006531673.1;XP_011246895.1;XP_030107429.1	1349430	.	0.055086	[illegible]
26909	NM_012012,XM_006496862,XM_030254499	Exo1	exonuclease 1	protein_coding	NP_036142.2;XP_006496925.1;XP_030110359.1	1349427	.	1.295525	[illegible]
26912	NM_001161712,NM_013847	Gcat	glycine C-acetyltransferase (2-amino-3-ketobutyrate-coenzyme A	protein_coding	NP_001155184.1;NP_038875.3	1349389	.	1.716128	[illegible]
26913	NM_001316710,NM_012014	Gprin1	G protein-regulated inducer of neurite outgrowth 1	protein_coding	NP_001303639.1;NP_036144.2	1349455	.	0.220788	[illegible]
26914	NM_001159513,NM_001159514,NM_001159515,NM_012015,XM_006517257,XM_006517258,XR_001780784,XR_001780785,XR_001780786,XR_003950455,XR_003950456,XR_003950457,XR_003950458,XR_382440	Macroh2a1	H2A histone family, member Y	protein_coding	NP_001152985.1;NP_001152986.1;NP_001152987.1;NP_036145.1;XP_006517320.1;XP_006517321.1	1349392	.	36.227777	[illegible]
26918	NM_001316689,NM_012016,NR_133565,XM_011241817,XM_011241818,XM_011241819,XM_011241820,XM_011241822,XM_011241823,XM_017322282,XM_030242614,XM_030242615,XR_869729	Ern2	endoplasmic reticulum (ER) to nucleus signalling 2	protein_coding	NP_001303618.1;NP_036146.2;XP_011240119.1;XP_011240120.1;XP_011240121.1;XP_011240122.1;XP_011240124.1;XP_011240125.1;XP_017177771.1;XP_030098474.1;XP_030098475.1	1349436	.	0.000000	[illegible]
26919	NM_012017,XM_006517259,XM_006517260,XM_030247283	Zfp346	zinc finger protein 346	protein_coding	NP_036147.1;XP_006517322.1;XP_006517323.1;XP_030103143.1	1349417	.	6.958696	[illegible]
26920	NM_001290635,NM_012018,XM_006498068,XM_006498069,XM_006498072,XM_006498073,XM_006498074,XM_006498075,XM_006498076,XM_006498077,XM_030251616,XM_030251617,XM_030251620,XR_003953259,XR_374096,XR_866015	Cntrl	centriolin	protein_coding	NP_001277564.1;NP_036148.2;XP_006498131.1;XP_006498132.1;XP_006498135.1;XP_006498136.1;XP_006498137.1;XP_006498138.1;XP_006498139.1;XP_006498140.1;XP_030107476.1;XP_030107477.1;XP_030107480.1	1889576	.	7.428650	[illegible]
26921	NM_001252200,NM_001252201,NM_001252202,NM_008696,XM_006496041,XM_006496042,XM_006496043,XM_006496044,XM_006496045,XM_006496046,XM_011238530,XM_017320994,XM_017320998,XM_017321001,XM_017321002,XM_017321004,XM_017321010,XM_017321011,XM_017321013,XM_017321018,XM_017321021,XM_030254710,XM_030254720,XR_001784729,XR_003955681	Map4k4	mitogen-activated protein kinase kinase kinase kinase 4	protein_coding	NP_001239129.1;NP_001239130.1;NP_001239131.1;NP_032722.2;XP_006496104.1;XP_006496105.1;XP_006496106.1;XP_006496107.1;XP_006496108.1;XP_006496109.1;XP_011236832.1;XP_017176483.1;XP_017176487.1;XP_017176490.1;XP_017176491.1;XP_017176493.1;XP_017176499.1;XP_017176500.1;XP_017176502.1;XP_017176507.1;XP_017176510.1;XP_030110570.1;XP_030110580.1	1349394	.	46.366069	[illegible]
26922	NM_025297,XM_006538885,XM_006538889,XM_011250261,XM_011250262,XM_011250263,XM_030253581,XM_030253582,XR_001784148,XR_001784149,XR_003954954	Mecr	mitochondrial trans-2-enoyl-CoA reductase	protein_coding	NP_079573.2;XP_006538948.1;XP_006538952.1;XP_011248563.1;XP_011248564.1;XP_011248565.1;XP_030109441.1;XP_030109442.1	1349441	.	8.196548	[illegible]

27402	NM_175094	Pdhx	pyruvate dehydrogenase complex, component X	protein_coding	NP_780303.1	1351627	.	[illegible]
27403	NM_001347081,NM_013850,XM_006513711,XM_006513712,XM_006513714,XM_017313958,XR_001779531,XR_003948698,XR_003948699	Abca7	ATP-binding cassette, sub-family A (ABC1), member 7	protein_coding	NP_001334010.1;NP_038878.1;XP_006513774.1;XP_006513775.1;XP_006513777.1;XP_017169447.1	1351646	.	[illegible]
27404	NM_001362749,NM_013851,XM_006533459,XM_006533460,XM_006533461,XM_006533463,XM_006533464,XM_030246047	Abca8b	ATP-binding cassette, sub-family A (ABC1), member 8b	protein_coding	NP_001349678.1;NP_038879.2;XP_006533522.1;XP_006533523.1;XP_006533524.1;XP_006533526.1;XP_006533527.1;XP_030101907.1	1351668	.	[illegible]
27405	NM_030239,XM_017320904,XM_017320905,XM_017320906	Abcg3	ATP binding cassette subfamily G member 3	protein_coding	NP_084515.2;XP_017176393.1;XP_017176394.1;XP_017176395.1	1351624	.	[illegible]
27406	NM_013852,XR_001781829,XR_384567	Abcf3	ATP-binding cassette, sub-family F (GCN20), member 3	protein_coding	NP_038880.1	1351656	.	[illegible]
27407	NM_001190443,NM_001359205,NM_013853,XM_006535727	Abcf2	ATP-binding cassette, sub-family F (GCN20), member 2	protein_coding	NP_001177372.1;NP_001346134.1;NP_038881.1;XP_006535790.1	1351657	.	[illegible]
27409	NM_031884	Abcg5	ATP binding cassette subfamily G member 5	protein_coding	NP_114090.1	1351659	.	[illegible]
27410	NM_001039581,NM_013855,XM_006524370,XR_001782080	Abca3	ATP-binding cassette, sub-family A (ABC1), member 3	protein_coding	NP_001034670.1;NP_038883.2;XP_006524433.1	1351617	.	[illegible]
27411	NM_001110273,NM_001110274,NM_030683,NM_207651,XM_006526488,XM_017317915	Slc14a2	solute carrier family 14 (urea transporter), member 2	protein_coding	NP_001103743.1;NP_001103744.1;NP_109608.1;NP_997534.2;XP_006526551.1;XP_017173404.1	1351653	.	[illegible]
27412	NM_013788	Peg12	paternally expressed 12	protein_coding	NP_038816.1	1351637	.	[illegible]
27413	NM_001363492,NM_021022,XM_006499670	Abcb11	ATP-binding cassette, sub-family B (MDR/TAP), member 11	protein_coding	NP_001350421.1;NP_066302.2;XP_006499733.1	1351619	.	[illegible]
27414	NM_013789,XM_006540943,XM_006540944,XM_011250869,XM_030242638,XM_030242639,XM_030242640,XM_030242641,XM_030242642,XR_003946486	Sergef	secretion regulating guanine nucleotide exchange factor	protein_coding	NP_038817.1;XP_006541006.1;XP_006541007.1;XP_011249171.1;XP_030098498.1;XP_030098499.1;XP_030098500.1;XP_030098501.1;XP_030098502.1	1351630	.	[illegible]
27416	NM_013790,NM_176839,XM_006522208,XM_006522209,XM_006522212,XM_006522213,XM_006522214,XM_006522215,XM_017317029,XM_030249144,XM_030249145,XM_030249146,XM_030249147,XM_030249148,XR_001781830,XR_001781831,XR_001781832,XR_001781833,XR_001781834,XR_001781835,XR_001781836,XR_384568,XR_384569	Abcc5	ATP-binding cassette, sub-family C (CFTR/MRP), member 5	protein_coding	NP_038818.2;NP_789809.1;XP_006522271.1;XP_006522272.1;XP_006522275.1;XP_006522276.1;XP_006522277.1;XP_006522278.1;XP_017172518.1;XP_030105004.1;XP_030105005.1;XP_030105006.1;XP_030105007.1;XP_030105008.1	1351644	.	[illegible]
27418	NM_013791,XM_017321605,XM_030255436,XM_030255437	Mkln1	muskelin 1, intracellular mediator containing kelch motifs	protein_coding	NP_038819.1;XP_017177094.1;XP_030111296.1;XP_030111297.1	1351638	.	[illegible]
27419	NM_013792,XM_006533465,XM_030246048,XM_030246049	Naglu	alpha-N-acetylglucosaminidase (Sanfilippo disease IIIB)	protein_coding	NP_038820.2;XP_006533528.1;XP_030101908.1;XP_030101909.1	1351641	.	[illegible]
27421	NM_018795,XM_006540945,XM_006540946,XM_006540947,XM_006540948,XM_006540949,XR_391346	Abcc6	ATP-binding cassette, sub-family C (CFTR/MRP), member 6	protein_coding	NP_061265.2;XP_006541008.3;XP_006541009.1;XP_006541010.1;XP_006541011.1;XP_006541012.1	1351634	.	[illegible]
27425	NM_013795	Atp5l	ATP synthase, H+ transporting, mitochondrial F0 complex, subunit G	protein_coding	NP_038823.2	1351597	.	[illegible]
27426	NM_013796,NR_027804,XM_006522216	Nagpa	N-acetylglucosamine-1-phosphodiester alpha-N-acetylglucosaminidase	protein_coding	NP_038824.2;XP_006522279.1	1351598	.	[illegible]
27428	NM_001077595,NM_001077596,NM_015756,XM_006534966,XM_006534968	Shroom3	shroom family member 3	protein_coding	NP_001071063.1;NP_001071064.1;NP_056571.2;XP_006535029.1;XP_006535031.1	1351655	.	[illegible]
27493	XR_003946774,XR_003946775,XR_003946776,XR_003946777,XR_391494	A230006K03Rik	RIKEN cDNA A230006K03 gene, transcript variant X5	lncRNA	.	3644205	.	[illegible]
27494	NM_001290274,NM_153319,XM_006528864,XM_006528867,XM_011247828,XM_030251359,XM_030251360	Amot	angiomotin	protein_coding	NP_001277203.1;NP_695231.3;XP_006528927.1;XP_006528930.1;XP_011246130.1;XP_030107219.1;XP_030107220.1	108440	.	[illegible]
27528	NM_001109988,NM_001109989,NM_001267717,NM_053078,XM_030250488	Nrep	neuronal regeneration related protein	protein_coding	NP_001103458.1;NP_001103459.1;NP_001254646.1;NP_444308.1;XP_030106348.1	99444	.	[illegible]
27632	NM_001045863,NM_001045864,NM_138580	Nelfe	negative elongation factor complex member E, Rdbp	protein_coding	NP_001039328.1;NP_001039329.1;NP_613046.2	102744	.	[illegible]
27643	NM_145405,NR_045779	Ubl4a	ubiquitin-like 4A	protein_coding	NP_663380.1	95049	.	[illegible]
27660	NM_001310576,NM_001310579,NM_001310581,NM_138581	1700088E04Rik	RIKEN cDNA 1700088E04 gene	protein_coding	NP_001297505.1;NP_001297508.1;NP_001297510.1;NP_613047.1	1920774	.	[illegible]
27681	NM_001356362,NM_033568,XM_006533467,XR_003949456	Snf8	SNF8, ESCRT-II complex subunit, homolog (S. cerevisiae)	protein_coding	NP_001343291.1;NP_291046.1;XP_006533530.1	1343161	.	[illegible]
27756	NM_001110101,NM_001204273,NM_001204274,NM_030597	Lsm2	LSM2 homolog, U6 small nuclear RNA and mRNA degradation associated	protein_coding	NP_001103571.1;NP_001191202.1;NP_001191203.1;NP_085100.1	90676	.	[illegible]
27762	NM_001347347,NM_138582,XM_017317497	Vwa7	von Willebrand factor A domain containing 7	protein_coding	NP_001334276.1;NP_613048.1;XP_017172986.1	1306798	.	[illegible]
27784	NM_001356611,NM_001356612,NM_001356613,NM_001356614,NM_178599,XM_030254548	Commd8	COMM domain containing 8	protein_coding	NP_001343540.1;NP_001343541.1;NP_001343542.1;NP_001343543.1;NP_848714.1;XP_030110408.1	1343485	.	[illegible]
27801	NM_172151,XM_006522223,XM_006522224	Zdhhc8	zinc finger, DHHC domain containing 8	protein_coding	NP_742163.4;XP_006522286.1;XP_006522287.1	1338012	.	[illegible]
27878	NM_030245,XM_030254784	Tada1	transcriptional adaptor 1	protein_coding	NP_084521.1;XP_030110644.1	1196415	.	[illegible]
27883	NM_001359108,NM_001359109,NM_001359110,NM_001359111,NM_138583,XM_006522230,XM_011245929,XM_017317031,XR_003951794,XR_384570,XR_384571	Tango2	transport and golgi organization 2	protein_coding	NP_001346037.1;NP_001346038.1;NP_001346039.1;NP_001346040.1;NP_613049.2;XP_006522293.1;XP_011244231.1;XP_017172520.1	101825	.	[illegible]
27886	NM_001081633,NM_022408,XM_011245930,XM_011245931,XM_030249151	Ess2	ess-2 splicing factor	protein_coding	NP_001075102.1;NP_071853.2;XP_011244232.1;XP_011244233.1;XP_030105011.1	107854	.	[illegible]
27965	NM_001357813,NM_138584,XM_030244409	Spg21	SPG21, maspardin	protein_coding	NP_001344742.1;NP_613050.1;XP_030100269.1	106403	.	[illegible]
27966	NM_145620	Rrp9	RRP9, small subunit (SSU) processome component, homolog (yeast)	protein_coding	NP_663595.1	2384313	.	[illegible]
27967	NM_001359024,NM_138585	Cherp	calcium homeostasis endoplasmic reticulum protein	protein_coding	NP_001345953.1;NP_613051.3	106417	.	[illegible]
27973	NM_178600,XM_030242648	Vkorc1	vitamin K epoxide reductase complex, subunit 1	protein_coding	NP_848715.1;XP_030098508.1	106442	.	[illegible]
27979	NM_133916	Eif3b	eukaryotic translation initiation factor 3, subunit B	protein_coding	NP_598677.1	106478	.	[illegible]
27981	NM_023665	Rsrp1	arginine/serine rich protein 1	protein_coding	NP_076154.3	106498	.	[illegible]
27984	NM_025994	Efhd2	EF hand domain containing 2	protein_coding	NP_080270.2	106504	.	[illegible]
27993	NM_178601	Imp4	IMP4, U3 small nucleolar ribonucleoprotein	protein_coding	NP_848716.2	106572	.	[illegible]
27998	NM_138586,NR_104358	Exosc5	exosome component 5	protein_coding	NP_613052.1	107889	.	[illegible]
27999	NM_138587,XM_006505102,XM_006505103	Fam3c	family with sequence similarity 3, member C	protein_coding	NP_613053.3;XP_006505165.1;XP_006505166.1	107892	.	[illegible]
28000	NM_001253843,NM_001253844,NM_134129	Prpf19	pre-mRNA processing factor 19	protein_coding	NP_001240772.1;NP_001240773.1;NP_598890.1	106247	.	[illegible]
28006	NM_026585,XM_006506214,XM_030255438,XR_003956205	Washc2	WASH complex subunit 2`	protein_coding	NP_080861.2;XP_006506277.1;XP_030111298.1	106463	.	[illegible]
28010	NM_001025365,XM_006538950,XM_006538951,XR_001784152,XR_003954958	Miip	migration and invasion inhibitory protein	protein_coding	NP_001020536.2;XP_006539013.1;XP_006539014.1	106506	.	[illegible]
28014	gene-D9Wsu149	D9Wsu149	DNA segment, Chr 9, Wayne State University 149	pseudogene	.	107281	.	[illegible]
28015	NM_001164793,NM_178602	Polr2m	polymerase (RNA) II (DNA directed) polypeptide M	protein_coding	NP_001158265.1;NP_848717.1	107282	.	[illegible]
28018	NM_138589,XM_006507913,XM_006507914,XM_006507915	Ubfd1	ubiquitin family domain containing 1	protein_coding	NP_613055.2;XP_006507976.3;XP_006507977.3;XP_006507978.3	107301	.	[illegible]
28019	NM_001368695,NM_133345	Ing4	inhibitor of growth family, member 4	protein_coding	NP_001355624.1;NP_579923.1	107307	.	[illegible]
28028	NM_178603	Mrpl50	mitochondrial ribosomal protein L50	protein_coding	NP_848718.1	107329	.	[illegible]
28030	NM_138591	Gfm1	G elongation factor, mitochondrial 1	protein_coding	NP_613057.2	107339	.	[illegible]
28035	NM_138592,XM_006506216,XM_017321609	Usp39	ubiquitin specific peptidase 39	protein_coding	NP_613058.1;XP_006506279.1;XP_017177098.1	107622	.	[illegible]
28036	NM_138593	Larp7	La ribonucleoprotein domain family, member 7	protein_coding	NP_613059.2	107634	.	[illegible]
28040	NM_138594,XM_006506217	D6Wsu163e	DNA segment, Chr 6, Wayne State University 163, expressed	protein_coding	NP_613060.1;XP_006506280.1	107893	.	[illegible]
28042	NM_027652,NR_137634	Selenoi	selenoprotein I	protein_coding	NP_081928.2	107898	.	[illegible]
28064	NM_145353	Yipf3	Yip1 domain family, member 3	protein_coding	NP_663328.2	106280	.	[illegible]
28071	NM_172253	Twistnb	twist basic helix-loop-helix transcription factor 1 neighbor	protein_coding	NP_758457.1	106292	.	[illegible]
28075	NM_001356541,NM_134095,XR_384015	Desi1	desumoylating isopeptidase 1	protein_coding	NP_001343470.1;NP_598856.1	106313	.	[illegible]
28077	NM_138596,XM_006517275,XM_011244527	Med10	mediator complex subunit 10	protein_coding	NP_613062.1;XP_006517338.1;XP_011242829.1	106331	.	[illegible]
28080	NM_138597	Atp5o	ATP synthase, H+ transporting, mitochondrial F1 complex, O subunit	protein_coding	NP_613063.1	106341	.	[illegible]
28081	NM_001362751,NM_138598,XM_011249064	Fam104a	family with sequence similarity 104, member A	protein_coding	NP_001349680.1;NP_613064.2;XP_011247366.1	106351	.	[illegible]
28084	NM_001284411,NM_001284412,NM_001284414,NM_026776,XM_006533504,XR_003949457,XR_003949458	Vps25	vacuolar protein sorting 25	protein_coding	NP_001271340.1;NP_001271341.1;NP_001271343.1;NP_081052.2;XP_006533567.2	106354	.	[illegible]
28088	NM_145422	Rtcb	RNA 2',3'-cyclic phosphate and 5'-OH ligase	protein_coding	NP_663397.1	106379	.	[illegible]
28105	NM_001170855,NM_178872	Trim36	tripartite motif-containing 36	protein_coding	NP_001164326.1;NP_849203.2	106264	.	[illegible]
28106	NM_080837	Mydgf	myeloid derived growth factor	protein_coding	NP_543027.1	2156020	.	[illegible]
28109	NM_026579	D10Wsu102e	DNA segment, Chr 10, Wayne State University 102, expressed	protein_coding	NP_080855.1	106381	.	[illegible]
28113	NM_145705,XM_006519080	Tinf2	Terf1 (TRF1)-interacting nuclear factor 2	protein_coding	NP_663751.2;XP_006519143.1	107246	.	[illegible]
28114	NM_145354	Nsun2	NOL1/NOP2/Sun domain family member 2	protein_coding	NP_663329.3	107252	.	[illegible]
28126	NM_178605,XM_030247292	Nop16	NOP16 nucleolar protein	protein_coding	NP_848720.1;XP_030103152.1	107862	.	[illegible]
28135	NM_001081122,NM_001301689,XM_006511739,XM_006511741,XM_006511744,XM_006511745,XM_006511747,XM_011242882,XM_011242883,XM_011242884,XM_011242885,XM_011242886,XM_011242890,XM_011242891,XM_017313406,XM_017313407,XM_017313408,XM_017313409,XM_017313410,XM_017313411,XM_017313412,XM_017313413,XM_017313414,XM_017313415,XM_017313416,XM_030244410,XM_030244411,XR_001778922,XR_001778923,XR_001778924,XR_003947926	Cep63	centrosomal protein 63	protein_coding	NP_001074591.1;NP_001288618.1;XP_006511802.1;XP_006511804.1;XP_006511807.1;XP_006511808.1;XP_006511810.1;XP_011241184.1;XP_011241185.1;XP_011241186.1;XP_011241187.1;XP_011241188.1;XP_011241192.1;XP_011241193.1;XP_017168895.1;XP_017168896.1;XP_017168897.1;XP_017168898.1;XP_017168899.1;XP_017168900.1;XP_017168901.1;XP_017168902.1;XP_017168903.1;XP_017168904.1;XP_017168905.1;XP_030100270.1;XP_030100271.1	2158560	.	[illegible]
28146	NM_030685	Serp1	stress-associated endoplasmic reticulum protein 1	protein_coding	NP_109610.1	92638	.	[illegible]
28169	NM_053014,XM_006513716,XM_006513717,XM_006513718,XM_006513719,XM_006513720,XM_006513721,XM_006513722,XM_006513723,XM_030245110	Agpat3	1-acylglycerol-3-phosphate O-acyltransferase 3	protein_coding	NP_443747.2;XP_006513779.1;XP_006513780.1;XP_006513781.1;XP_006513782.1;XP_006513783.1;XP_006513784.1;XP_006513785.1;XP_006513786.1;XP_030100970.1	1336186	.	[illegible]
28185	NM_138599	Tomm70a	translocase of outer mitochondrial membrane 70A	protein_coding	NP_613065.2	106295	.	[illegible]
28193	NM_001204915,NM_178606,XM_006513726,XM_006513727,XM_006513728	Reep3	receptor accessory protein 3	protein_coding	NP_001191844.1;NP_848721.1;XP_006513789.1;XP_006513790.1;XP_006513791.1	88930	.	[illegible]
28194	NM_133996	Apon	apolipoprotein N	protein_coding	NP_598757.1	88931	.	[illegible]
28199	NM_001199009,NM_133734,NR_037572,XM_006519081,XM_006519083,XM_006519084,XM_006519085,XM_006519086,XM_006519087,XM_006519088,XM_006519090,XM_006519091,XM_006519092,XM_030247846,XM_030247847,XM_030247848,XM_030247849,XM_030247850,XM_030247851	Dcaf11	DDB1 and CUL4 associated factor 11	protein_coding	NP_001185938.1;NP_598495.1;XP_006519144.1;XP_006519146.1;XP_006519147.1;XP_006519148.1;XP_006519149.1;XP_006519150.1;XP_006519151.1;XP_006519153.1;XP_006519154.1;XP_006519155.1;XP_030103706.1;XP_030103707.1;XP_030103708.1;XP_030103709.1;XP_030103710.1;XP_030103711.1	90168	.	[illegible]
28200	NM_001037938,NM_030686,XM_030247852	Dhrs4	dehydrogenase/reductase (SDR family) member 4	protein_coding	NP_001033027.2;NP_109611.1;XP_030103712.1	90169	.	[illegible]
28240	NM_138301,XM_006513730,XM_006513731,XM_006513732,XM_006513733,XM_011243470,XM_017313959,XM_017313960,XM_017313961,XM_017313962,XM_030245111,XM_030245112,XR_001779532,XR_871714	Trpm2	transient receptor potential cation channel, subfamily M, member 2	protein_coding	NP_612174.2;XP_006513793.1;XP_006513794.1;XP_006513795.1;XP_006513796.1;XP_011241772.1;XP_017169448.1;XP_017169449.1;XP_017169450.1;XP_017169451.1;XP_030100971.1;XP_030100972.1	1351901	.	[illegible]
28250	NM_001355577,NM_030687,XM_011241584,XM_011241585,XM_011241586,XR_003956206,XR_003956207	Slco1a4	solute carrier organic anion transporter family, member 1a4	protein_coding	NP_001342506.1;NP_109612.1;XP_011239886.1;XP_011239887.1;XP_011239888.1	1351896	.	[illegible]
28253	NM_020495,XM_006506963,XM_017321611	Slco1b2	solute carrier organic anion transporter family, member 1b2	protein_coding	NP_065241.1;XP_006507026.1;XP_017177100.1	1351899	.	[illegible]
28295	NM_001364646,NM_138601	Gatd3a	glutamine amidotransferase like class 1 domain containing 3A	protein_coding	NP_001351575.1;NP_613067.1	1351861	.	[illegible]
29805	NM_013859	Znhit2	zinc finger, HIT domain containing 2	protein_coding	NP_038887.2	1352481	.	[illegible]
29806	NM_013860,XM_011242977	Limd1	LIM domains containing 1	protein_coding	NP_038888.2;XP_011241279.1	1352502	.	[illegible]
29807	NM_001311111,NM_013861,XM_006506218,XM_006506219	Tpk1	thiamine pyrophosphokinase	protein_coding	NP_001298040.1;NP_038889.1;XP_006506281.1;XP_006506282.1	1352500	.	[illegible]
29808	NM_001164274,NM_013720,XM_006499687,XM_006499688,XM_006499689,XM_006499690,XM_006499691,XM_006499697,XM_006499698,XM_011239589,XM_011239590,XM_011239591,XM_011239592,XM_017318962,XM_017318963,XM_017318964,XM_030251727,XR_866290,XR_866291	Mga	MAX gene associated	protein_coding	NP_001157746.1;NP_038748.2;XP_006499750.1;XP_006499751.1;XP_006499752.1;XP_006499753.1;XP_006499754.1;XP_006499760.1;XP_006499761.1;XP_011237891.1;XP_011237892.1;XP_011237893.1;XP_011237894.1;XP_017174451.1;XP_017174452.1;XP_017174453.1;XP_030107587.1	1352483	.	[illegible]
29809	NM_001038621,NM_013862,XM_006496875,XM_006496876,XM_006496877,XM_006496878,XM_006496881,XM_006496883,XM_030254802,XM_030254804,XM_030254805	Rabgap1l	RAB GTPase activating protein 1-like	protein_coding	NP_001033710.1;NP_038890.3;XP_006496938.1;XP_006496939.1;XP_006496940.1;XP_006496941.1;XP_006496944.1;XP_006496946.1;XP_030110662.1;XP_030110664.1;XP_030110665.1	1352507	.	[illegible]
29810	NM_013863	Bag3	BCL2-associated athanogene 3	protein_coding	NP_038891.4	1352493	.	[illegible]
29811	NM_001145959,NM_001360264,NM_001360265,NM_001360266,NM_001360267,NM_001360268,NM_001360269,NM_001360270,NM_001360271,NM_001360272,NM_001360273,NM_001360274,NM_001360276,NM_013864,XM_017316049,XM_030247853	Ndrg2	N-myc downstream regulated gene 2	protein_coding	NP_001139431.1;NP_001347193.1;NP_001347194.1;NP_001347195.1;NP_001347196.1;NP_001347197.1;NP_001347198.1;NP_001347199.1;NP_001347200.1;NP_001347201.1;NP_001347202.1;NP_001347203.1;NP_001347205.1;NP_038892.1;XP_017171538.1;XP_0301037	1352498	.	[illegible]
29812	NM_001355391,NM_001355392,NM_001355393,NM_001355394,NM_013865,NM_180956,XM_011239593,XM_011239596,XM_017318968,XM_030251732,XM_030251733,XM_030251734,XM_030251735,XM_030251736	Ndrg3	N-myc downstream regulated gene 3	protein_coding	NP_001342320.1;NP_001342321.1;NP_001342322.1;NP_001342323.1;NP_038893.1;NP_851287.1;XP_011237895.1;XP_011237898.1;XP_017174457.1;XP_030107592.1;XP_030107593.1;XP_030107594.1;XP_030107595.1;XP_030107596.1	1352499	.	[illegible]
29813	NM_001356627,NM_013866,XM_006521053,XM_006521054,XM_006521055,XM_006521056,XM_011245656	Zfp385a	zinc finger protein 385A	protein_coding	NP_001343556.1;NP_038894.2;XP_006521116.1;XP_006521117.1;XP_006521118.1;XP_006521119.1;XP_011243958.1	1352495	.	[illegible]
29815	NM_001355264,NM_013867,XM_006501559	Bcar3	breast cancer anti-estrogen resistance 3	protein_coding	NP_001342193.1;NP_038895.1;XP_006501622.1	1352501	.	[illegible]
29816	NM_145070,XM_006530369,XM_006530370,XM_006530373,XR_387567,XR_387568,XR_387569	Hip1r	huntingtin interacting protein 1 related	protein_coding	NP_659507.3;XP_006530432.1;XP_006530433.1;XP_006530436.1	1352504	.	[illegible]
29817	NM_001159518,NM_008048	Igfbp7	insulin-like growth factor binding protein 7	protein_coding	NP_001152990.1;NP_032074.3	1352480	.	[illegible]
29818	NM_013868	Hspb7	heat shock protein family, member 7 (cardiovascular)	protein_coding	NP_038896.2	1352494	.	[illegible]
29819	NM_001111272,NM_001347044,NM_001347045,NM_025303,XM_006495528,XM_006495532,XM_011238387,XM_011238388,XM_011238389,XM_011238390,XM_017321102,XM_017321109,XM_030254814,XM_030254823	Stau2	staufen double-stranded RNA binding protein 2	protein_coding	NP_001104742.1;NP_001333973.1;NP_001333974.1;NP_079579.2;XP_006495591.1;XP_006495595.1;XP_011236689.1;XP_011236690.1;XP_011236691.1;XP_011236692.1;XP_017176591.1;XP_017176598.1;XP_030110674.1;XP_030110683.1	1352508	.	[illegible]
29820	NM_001164155,NM_013869	Tnfrsf19	tumor necrosis factor receptor superfamily, member 19	protein_coding	NP_001157627.1;NP_038897.4	1352474	.	[illegible]
29846	NM_019474	Olfr156	olfactory receptor 156	protein_coding	NP_062347.2	1352683	.	[illegible]
29849	NM_019476	Olfr159	olfactory receptor 159	protein_coding	NP_062349.1	1352686	.	[illegible]
29856	NM_001159284,NM_001284427,NM_001284428,NM_001284429,NM_013870,XM_006514724,XM_017314586,XM_030246062,XM_030246063,XM_030246064,XM_030246065	Smtn	smoothelin	protein_coding	NP_001152756.1;NP_001271356.1;NP_001271357.1;NP_001271358.1;NP_038898.2;XP_006514787.1;XP_017170075.1;XP_030101922.1;XP_030101923.1;XP_030101924.1;XP_030101925.1	1354727	.	[illegible]
29857	NM_013871,XM_006521058,XM_006521059,XM_006521060,XM_006521061	Mapk12	mitogen-activated protein kinase 12	protein_coding	NP_038899.1;XP_006521121.1;XP_006521122.1;XP_006521123.1;XP_006521124.1	1353438	.	[illegible]
29858	NM_001282040,NM_001282041,NM_013872,XR_875301	Pmm1	phosphomannomutase 1	protein_coding	NP_001268969.1;NP_001268970.1;NP_038900.1	1353418	.	[illegible]
29859	NM_001356515,NM_013873	Sult4a1	sulfotransferase family 4A, member 1	protein_coding	NP_001343444.1;NP_038901.3	1888971	.	[illegible]
29861	NM_001368371,NM_013874,XM_006540032,XM_006540039,XM_006540041,XM_011250589,XM_030242653,XM_030242654,XM_030242655,XM_030242656,XM_030242657,XM_030242658,XM_030242659,XM_030242660,XM_030242661,XM_030242662,XM_030242663,XM_030242664	Dpf1	D4, zinc and double PHD fingers family 1	protein_coding	NP_001355300.1;NP_038902.1;XP_006540095.1;XP_006540102.1;XP_006540104.3;XP_011248891.1;XP_030098513.1;XP_030098514.1;XP_030098515.1;XP_030098516.1;XP_030098517.1;XP_030098518.1;XP_030098519.1;XP_030098520.1;XP_030098521.1;XP_030098522.1;XP_030098523.1;XP_030098524.1	1352748	.	[illegible]
29862	NM_001347432,NM_016718,XM_006506223,XM_006506224,XM_017321614	Ninj2	ninjurin 2	protein_coding	NP_001334361.1;NP_057927.1;XP_006506286.1;XP_006506287.1;XP_017177103.1	1352751	.	[illegible]
29863	NM_001347366,NM_013875	Pde7b	phosphodiesterase 7B	protein_coding	NP_001334295.1;NP_038903.3	1352752	.	[illegible]
29864	NM_013876	Rnf11	ring finger protein 11	protein_coding	NP_038904.1	1352759	.	[illegible]
29865	NM_013877,XM_017322297,XM_017322298	Cabp5	calcium binding protein 5	protein_coding	NP_038905.1;XP_017177786.1;XP_017177787.1	1352746	.	[illegible]
29867	NM_001310712,NM_001310715,NM_013879	Cabp1	calcium binding protein 1	protein_coding	NP_001297641.1;NP_001297644.1;NP_038907.1	1352750	.	[illegible]
29869	NM_013881,XM_006533508,XM_006533509,XM_030246066	Ulk2	unc-51 like kinase 2	protein_coding	NP_038909.3;XP_006533571.1;XP_006533572.1;XP_030101926.1	1352758	.	[illegible]
29870	NM_001168672,NM_013882,XR_001781542	Gtse1	G two S phase expressed protein 1	protein_coding	NP_001162143.1;NP_038910.1	1352755	.	[illegible]
29871	NM_001159630,NM_001355423,NM_001355424,NM_013883,XM_006503149,XM_011240550,XM_017320252,XM_017320253,XM_030253598,XM_030253599,XM_030253600,XR_001784153,XR_001784155,XR_003954959,XR_003954960	Scmh1	sex comb on midleg homolog 1	protein_coding	NP_001153102.1;NP_001342352.1;NP_001342353.1;NP_038911.1;XP_006503212.1;XP_011238852.1;XP_017175741.1;XP_017175742.1;XP_030109458.1;XP_030109459.1;XP_030109460.1	1352762	.	[illegible]
29873	NM_001166273,NM_013884	Cspg5	chondroitin sulfate proteoglycan 5	protein_coding	NP_001159745.1;NP_038912.3	1352747	.	[illegible]
29875	NM_016721,XM_006540950,XR_001785567,XR_391347	Iqgap1	IQ motif containing GTPase activating protein 1	protein_coding	NP_057930.2;XP_006541013.2	1352757	.	[illegible]
29876	NM_013885	Clic4	chloride intracellular channel 4 (mitochondrial)	protein_coding	NP_038913.1	1352754	.	[illegible]
29877	NM_013886	Hdgfl3	HDGF like 3	protein_coding	NP_038914.2	1352760	.	[illegible]
30044	NM_001128599,NM_013887	Opn4	opsin 4 (melanopsin)	protein_coding	NP_001122071.1;NP_038915.1	1353425	.	[illegible]
30045	NM_001253685,NM_013888	Dnajc12	DnaJ heat shock protein family (Hsp40) member C12	protein_coding	NP_001240614.1;NP_038916.1	1353428	.	[illegible]
30046	NM_013889,XM_006537978	Zfp292	zinc finger protein 292	protein_coding	NP_038917.2;XP_006538041.1	1353423	.	[illegible]
30049	NM_024450	Scd3	stearoyl-coenzyme A desaturase 3	protein_coding	NP_077770.1	1353437	.	[illegible]
30050	NM_001164768,NM_001164769,NM_001164770,NM_001164772,NM_013890,XM_006498117,XM_006498118	Fbxw2	F-box and WD-40 domain protein 2	protein_coding	NP_001158240.1;NP_001158241.1;NP_001158242.1;NP_001158244.1;NP_038918.3;XP_006498180.1;XP_006498181.1	1353435	.	[illegible]
30051	NM_001357728,NM_013891,XM_006524379,XM_006524380,XM_006524381	Spdef	SAM pointed domain containing ets transcription factor	protein_coding	NP_001344657.1;NP_038919.1;XP_006524442.1;XP_006524443.1;XP_006524444.1	1353422	.	[illegible]
30052	NM_013892	Pcsk1n	proprotein convertase subtilisin/kexin type 1 inhibitor	protein_coding	NP_038920.2	1353431	.	[illegible]
30054	NM_001033043,XM_006519108,XM_006519109,XM_006519110,XM_006519112,XM_030247854,XM_030247855,XM_030247856	Rnf17	ring finger protein 17	protein_coding	NP_001028215.1;XP_006519171.1;XP_006519172.1;XP_006519173.1;XP_006519175.1;XP_030103714.1;XP_030103715.1;XP_03010371	1353419	.	[illegible]
30055	NM_013895	Timm13	translocase of inner mitochondrial membrane 13	protein_coding	NP_038923.1	1353432	.	[illegible]
30056	NM_001024853,NM_001024854,NM_001286203,NM_013896,XM_011244122,XM_017315088	Timm9	translocase of inner mitochondrial membrane 9	protein_coding	NP_001020024.1;NP_001020025.1;NP_001273132.1;NP_038924.1;XP_011242424.1;XP_017170577.1	1353436	.	[illegible]
30057	NM_013897	Timm8b	translocase of inner mitochondrial membrane 8B	protein_coding	NP_038925.1	1353424	.	[illegible]
30058	NM_013898	Timm8a1	translocase of inner mitochondrial membrane 8A1	protein_coding	NP_038926.1	1353433	.	[illegible]
30059	NM_013899	Timm10	translocase of inner mitochondrial membrane 10	protein_coding	NP_038927.2	1353429	.	[illegible]
30060	NM_013900,XM_006522232	Meltf	melanotransferrin	protein_coding	NP_038928.1;XP_006522295.1	1353421	.	[illegible]
30785	NM_080285,NR_149735,XM_006505104,XM_011241065	Cttnbp2	cortactin binding protein 2	protein_coding	NP_525024.1;XP_006505167.1;XP_011239367.1	1353467	.	[illegible]
30791	NM_013901,XM_006501560	Slc39a1	solute carrier family 39 (zinc transporter), member 1	protein_coding	NP_038929.2;XP_006501623.1	1353474	.	[illegible]
30794	NM_019417,XM_006533511,XM_030246067	Pdlim4	PDZ and LIM domain 4	protein_coding	NP_062290.2;XP_006533574.1;XP_030101927.1	1353470	.	[illegible]
30795	NM_013902	Fkbp3	FK506 binding protein 3	protein_coding	NP_038930.1	1353460	.	[illegible]
30800	NM_013903	Mmp20	matrix metallopeptidase 20 (enamelysin)	protein_coding	NP_038931.1	1353466	.	[illegible]
30805	NM_001330304,NM_019687,XM_006533512,XM_011249065,XM_017314588,XR_003949459,XR_003949460	Slc22a4	solute carrier family 22 (organic cation transporter), member 4	protein_coding	NP_001317233.1;NP_062661.1;XP_006533575.1;XP_011247367.1;XP_017170077.1	1353479	.	[illegible]
30806	NM_001326292,NM_013906	Adamts8	a disintegrin-like and metallopeptidase (reprolysin type) with thrombospondin type 1 motif, 8	protein_coding	NP_001313221.1;NP_038934.2	1353468	.	[illegible]
30838	NM_013907,XM_006527131,XM_011247258,XM_011247259,XM_011247261,XR_001782592,XR_386481,XR_386482,XR_877675	Fbxw4	F-box and WD-40 domain protein 4	protein_coding	NP_038935.1;XP_006527194.1;XP_011245560.1;XP_011245561.1;XP_011245563.1	1354698	.	[illegible]
30839	NM_001356407,NM_001356408,NM_013908	Fbxw5	F-box and WD-40 domain protein 5	protein_coding	NP_001343336.1;NP_001343337.1;NP_038936.1	1354731	.	[illegible]
30840	NM_013909	Fbxl6	F-box and leucine-rich repeat protein 6	protein_coding	NP_038937.2	1354705	.	[illegible]
30841	NM_001003953,NM_013910,XM_006530376,XM_011248208,XM_011248209,XM_011248210,XM_011248211,XM_011248212,XM_011248213,XM_011248214,XM_011248215,XM_011248216,XM_011248217,XM_030254558,XM_030254560	Kdm2b	lysine (K)-specific demethylase 2B	protein_coding	NP_001003953.1;NP_038938.1;XP_006530439.1;XP_011246510.1;XP_011246511.1;XP_011246512.1;XP_011246513.1;XP_011246514.1;XP_011246515.1;XP_011246516.1;XP_011246517.1;XP_011246518.1;XP_011246519.1;XP_030110418.1;XP_030110420.1	1354737	.	[illegible]
30843	NM_001002846,NM_001286529,NM_001286530,NM_013911,XM_006510397,XM_006510398,XM_006510399	Fbxl12	F-box and leucine-rich repeat protein 12	protein_coding	NP_001002846.1;NP_001273458.1;NP_001273459.1;NP_038939.2;XP_006510460.1;XP_006510461.1;XP_006510462.1	1354738	.	[illegible]
30853	NM_001170341,NM_145385	Mlf2	myeloid leukemia factor 2	protein_coding	NP_001163812.1;NP_663360.1	1353554	.	[illegible]
30877	NM_153547,NR_104298	Gnl3	guanine nucleotide binding protein-like 3 (nucleolar)	protein_coding	NP_705775.2	1353651	.	[illegible]
30878	NM_013912	Apln	apelin	protein_coding	NP_038940.1	1353624	.	[illegible]
30924	NM_013913	Angptl3	angiopoietin-like 3	protein_coding	NP_038941.1	1353627	.	[illegible]
30925	NM_001347186,NM_001347187,NM_030710,XM_006496884,XM_006496886,XM_011238824,XM_011238825,XR_373611	Slamf6	SLAM family member 6	protein_coding	NP_001334115.1;NP_001334116.1;NP_109635.1;XP_006496947.1;XP_006496949.1;XP_011237126.1;XP_011237127.1	1353620	.	[illegible]
30926	NM_001348093,NM_023140	Glrx3	glutaredoxin 3	protein_coding	NP_001335022.1;NP_075629.2	1353653	.	[illegible]
30927	NM_013914,XM_006531074	Snai3	snail family zinc finger 3	protein_coding	NP_038942.1;XP_006531137.1	1353563	.	[illegible]
30928	NM_001012330,NM_001355098,NM_001355099,NM_001355100,NM_001355101,NM_001355102,NM_001355103,NM_013915,XM_006496889,XM_006496896,XM_030254843,XM_030254845,XM_030254847,XM_030254851	Zbtb18	zinc finger and BTB domain containing 18	protein_coding	NP_001012330.1;NP_001342027.1;NP_001342028.1;NP_001342029.1;NP_001342030.1;NP_001342031.1;NP_001342032.1;NP_038943.3;XP_006496952.1;XP_006496959.1;XP_030110703.1;XP_030110705.1;XP_030110707.1;XP_030110711.1	1353609	.	[illegible]
30930	NM_001113355,NM_001358543,NM_133672	Vps26a	VPS26 retromer complex component A	protein_coding	NP_001106826.1;NP_001345472.1;NP_598433.1	1353654	.	[illegible]
30931	NM_144884	Tor1a	torsin family 1, member A (torsin A)	protein_coding	NP_659133.1	1353568	.	[illegible]
30932	NM_001368343,NM_001368344,NM_001368345,NM_001368346,NM_145600	Zfp330	zinc finger protein 330	protein_coding	NP_001355272.1;NP_001355273.1;NP_001355274.1;NP_001355275.1;NP_663575.1	1353574	.	[illegible]
30933	NM_001369222,NM_001369223,NM_001369224,NM_001369225,NM_152800,NR_045485,XM_030251737	Tor2a	torsin family 2, member A	protein_coding	NP_001356151.1;NP_001356152.1;NP_001356153.1;NP_001356154.1;NP_690013.1;XP_030107597.1	1353596	.	[illegible]
30934	NM_133673,XM_017318972,XR_374100	Tor1b	torsin family 1, member B	protein_coding	NP_598434.2;XP_017174461.1	1353605	.	[illegible]
30935	NM_023141	Tor3a	torsin family 3, member A	protein_coding	NP_075630.2	1353652	.	[illegible]
30937	NM_144799	Lmcd1	LIM and cysteine-rich domains 1	protein_coding	NP_659048.1	1353635	.	[illegible]
30938	NM_015759,XM_006516929,XM_006516930,XM_006516931	Fgd3	FYVE, RhoGEF and PH domain containing 3	protein_coding	NP_056574.2;XP_006516992.1;XP_006516993.1;XP_006516994.1	1353657	.	[illegible]
30939	NM_001131054,NM_001362799,NM_013917,XM_006533516,XM_030246068	Pttg1	pituitary tumor-transforming gene 1	protein_coding	NP_001124526.1;NP_001349728.1;NP_038945.2;XP_006533579.1;XP_030101928.1	1353578	.	[illegible]
30940	NM_013918,XM_006523055,XM_006523056,XM_006523057	Usp25	ubiquitin specific peptidase 25	protein_coding	NP_038946.2;XP_006523118.1;XP_006523119.1;XP_006523120.1	1353655	.	[illegible]
30941	NM_013919	Usp21	ubiquitin specific peptidase 21	protein_coding	NP_038947.2	1353665	.	[illegible]
30942	NM_013920,XM_006530072	Hnf4g	hepatocyte nuclear factor 4, gamma	protein_coding	NP_038948.1;XP_006530135.1	1353604	.	[illegible]
30943	NM_013921,XM_006524382,XM_011246483,XM_011246484,XM_011246485	Prss30	protease, serine 30	protein_coding	NP_038949.2;XP_006524445.1;XP_011244785.1;XP_011244786.1;XP_011244787.1	1353645	.	[illegible]
30944	NM_013922	Zfp354c	zinc finger protein 354C	protein_coding	NP_038950.3	1353621	.	[illegible]
30945	NM_001357631,NM_013923	Rnf19a	ring finger protein 19A	protein_coding	NP_001344560.1;NP_038951.1	1353623	.	[illegible]
30946	NM_013924	Abt1	activator of basal transcription 1	protein_coding	NP_038952.1	1353636	.	[illegible]

30947	NM_001357184,NM_001357185,NM_001357186,NM_013925,XM_006531075	Adat1	adenosine deaminase, tRNA-specific 1	protein_coding	[illegible]
30948	NM_001083334,NM_001360876,NM_009668,XM_006525978,XM_006525979,XM_006525980,XM_006525981,XM_006525982,XM_006525984,XM_006525985,XM_006525986,XM_006525987,XM_006525988,XM_006525989,XM_006525990,XM_006525991,XM_006525992,XM_006525993,XM_006525994,XM_006525995,XM_017317916,XM_017317917,XM_030250489,XM_030250490,XM_030250491,XM_030250492,XM_030250493,XM_030250495,XM_030250496,XM_030250497	Bin1	bridging integrator 1	protein_coding	[illegible]
30949	NM_001355050,NM_025304,XM_030242666	Lcmt1	leucine carboxyl methyltransferase 1	protein_coding	[illegible]
30951	NM_013926,XM_017314589	Cbx8	chromobox 8	protein_coding	[illegible]
30952	NM_013927	Cngb3	cyclic nucleotide gated channel beta 3	protein_coding	[illegible]
30953	NM_001113420,NM_001113421,NM_001282044,NM_001282045,NM_013928	Schip1	schwannomin interacting protein 1	protein_coding	[illegible]
30954	NM_001161737,NM_013929	Siva1	SIVA1, apoptosis-inducing factor	protein_coding	[illegible]
30955	NM_001146200,NM_001146201,NM_020272,NR_157094,XM_006515112,XM_030246758	Pik3cg	phosphatidylinositol-4,5-bisphosphate 3-kinase catalytic subunit gamma	protein_coding	[illegible]
30956	NM_013930	Aass	aminoadipate-semialdehyde synthase	protein_coding	[illegible]
30957	NM_001163447,NM_001163448,NM_001163449,NM_001163450,NM_001163451,NM_001163453,NM_013931	Mapk8ip3	mitogen-activated protein kinase 8 interacting protein 3	protein_coding	[illegible]
30959	NM_013932	Ddx25	DEAD (Asp-Glu-Ala-Asp) box polypeptide 25	protein_coding	[illegible]
30960	NM_001355402,NM_013933,XM_017317500	Vapa	vesicle-associated membrane protein, associated protein A	protein_coding	[illegible]
30962	NM_001199015,NM_001199016,NM_021291,XM_006540044,XM_006540045,XM_006540046,XM_006540047,XM_011250591,XM_011250592	Slc7a9	solute carrier family 7 (cationic amino acid transporter, y+ system), member 9	protein_coding	[illegible]
30963	NM_001012396,NM_013935	Hacd1	3-hydroxyacyl-CoA dehydratase 1	protein_coding	[illegible]
30964	gene-Rps12-ps1	Rps12-ps1	ribosomal protein S12, pseudogene 1	pseudogene	[illegible]
50490	NM_001285833,NM_001285835,NM_015760,XM_006508010,XM_006508011,XM_006508012,XM_011241854,XM_017322347	Nox4	NADPH oxidase 4	protein_coding	[illegible]
50492	NM_022653	Thop1	thimet oligopeptidase 1	protein_coding	[illegible]
50493	NM_001042513,NM_001042514,NM_001042523,NM_015762	Txnrd1	thioredoxin reductase 1	protein_coding	[illegible]
50496	NM_033270,NR_027677,XM_006515147,XM_006515148,XM_006515149,XM_011243882	E2f6	E2F transcription factor 6	protein_coding	[illegible]
50497	NM_001355397,NM_015765,NR_103465,NR_149715	Hspa14	heat shock protein 14	protein_coding	[illegible]
50498	NM_015766,XM_006524599	Ebi3	Epstein-Barr virus induced gene 3	protein_coding	[illegible]
50500	NM_001317723,NM_015767,XM_017320306,XM_030253651	Ttpa	tocopherol (alpha) transfer protein	protein_coding	[illegible]
50501	NM_001037539,NM_001170419,NM_015768	Prok2	prokineticin 2	protein_coding	[illegible]
50505	NM_015769,XR_384576	Ercc4	excision repair cross-complementing rodent repair deficiency, complementation group 4	protein_coding	[illegible]
50518	NM_015770,XM_011239689,XM_011239690,XM_011239691,XM_017319110,XM_017319112,XM_017319115,XM_017319117,XM_017319119,XM_017319120,XM_017319121,XM_030251858,XM_030251859,XM_030251860	a	nonagouti	protein_coding	[illegible]
50523	NM_015771,NM_153382,XM_006519224,XM_006519225	Lats2	large tumor suppressor 2	protein_coding	[illegible]
50524	NM_001244916,NM_015772	Sall2	spalt like transcription factor 2	protein_coding	[illegible]
50525	NM_015773,XM_017317063	Spag6l	sperm associated antigen 6-like	protein_coding	[illegible]
50527	NM_015774	Ero1l	ERO1-like (S. cerevisiae)	protein_coding	[illegible]
50528	NM_015775,XM_006523064,XM_006523065	Tmprss2	transmembrane protease, serine 2	protein_coding	[illegible]
50529	NM_025305	Mrps7	mitchondrial ribosomal protein S7	protein_coding	[illegible]
50530	NM_001347434,NM_015776	Mfap5	microfibrillar associated protein 5	protein_coding	[illegible]
50701	NM_015779	Elane	elastase, neutrophil expressed	protein_coding	[illegible]
50706	NM_001198765,NM_001198766,NM_001313898,NM_001313899,NM_001368678,NM_015784	Postn	periostin, osteoblast specific factor	protein_coding	[illegible]
50708	NM_015786	H1f2	histone cluster 1, H1c	protein_coding	[illegible]
50709	NM_015787	H1f4	histone cluster 1, H1e	protein_coding	[illegible]
50720	NM_015788,NM_172809,XM_006519226,XM_006519227,XM_006519229,XM_030247880,XM_030247881,XM_030247882,XM_030247883	Sacs	sacsin	protein_coding	[illegible]
50721	NM_001163430,NM_181586,XM_006513861,XM_006513863,XM_011243507	Sirt6	sirtuin 6	protein_coding	[illegible]
50722	NM_015789,XM_006540985	Dkkl1	dickkopf-like 1	protein_coding	[illegible]
50723	NM_015790	Icosl	icos ligand	protein_coding	[illegible]
50724	NM_001081168	Sap30l	SAP30-like	protein_coding	[illegible]
50753	NM_001368347,NM_001368348,NM_015791	Fbxo8	F-box protein 8	protein_coding	[illegible]
50754	NM_001177773,NM_001177774,NM_080428,XM_006501656,XM_006501657,XM_006501658,XM_030252669	Fbxw7	F-box and WD-40 domain protein 7	protein_coding	[illegible]
50755	NM_001348234,NM_001348235,NM_001348236,NM_001348237,NM_015792,XM_006497505,XM_030251861	Fbh1	F-box DNA helicase 1	protein_coding	[illegible]
50757	NM_001311159,NM_015793,XM_006512182,XM_017313455,XM_017313456,XM_017313457	Fbxw14	F-box and WD-40 domain protein 14	protein_coding	[illegible]
50758	NM_015794,XM_006524600,XR_385343	Fbxl17	F-box and leucine-rich repeat protein 17	protein_coding	[illegible]
50759	NM_001360381,NM_001360383,NM_015795	Fbxo16	F-box protein 16	protein_coding	[illegible]
50760	NM_001310687,NM_015796,XM_006540169,XR_391073,XR_3910	Fbxo17	F-box protein 17	protein_coding	[illegible]
50762	NM_001163704,NM_001163705,NM_001163706,NM_001163707,NM_015797	Fbxo6	F-box protein 6	protein_coding	[illegible]
50764	NM_001367965,NM_015798,XM_011247086,XM_030250527,XR_877588	Fbxo15	F-box protein 15	protein_coding	[illegible]
50765	NM_001289507,NM_001289509,NM_001289511,NM_001359206,NM_015799,XM_006504593,XM_006504594,XM_030254655	Tfr2	transferrin receptor 2	protein_coding	[illegible]
50766	NM_015800,XM_006524601,XM_006524602,XM_006524603,XM_030249892	Crim1	cysteine rich transmembrane BMP regulator 1 (chordin like)	protein_coding	[illegible]
50767	NM_001122818,NM_001359121,NM_001359122,NM_001359123,NM_001359124,NM_015801,NR_152827,XM_006508824,XM_006508825,XM_006508827,XM_006508828,XM_006508829,XM_006508830,XM_006508831,XM_006508832,XM_011242066,XR_003947314,XR_378664	Pnpla6	patatin-like phospholipase domain containing 6	protein_coding	[illegible]
50768	NM_001194940,NM_001194941,NM_015802,XM_006509166,XM_006509167,XM_006509168,XM_006509169,XM_011242152,XM_011242153,XM_017312892	Dlc1	deleted in liver cancer 1	protein_coding	[illegible]
50769	NM_015803	Atp8a2	ATPase, aminophospholipid transporter-like, class I, type 8A, member 2	protein_coding	[illegible]
50770	NM_001293667,NM_001293668,NM_015804,NR_121603,XM_006508834,XM_006508837,XM_006508839,XM_006508840,XM_011242067,XM_011242068,XM_011242069,XM_017312893	Atp11a	ATPase, class VI, type 11A	protein_coding	[illegible]
50771	NM_001201569,NM_015805,XM_006526506,XM_006526507,XM_006526508,XM_006526511,XM_030250528,XM_030250529,XM_030250530,XM_030250531,XM_030250532,XM_030250533,XM_030250534,XM_030250535,XR_001782381,XR_001782382	Atp9b	ATPase, class II, type 9B	protein_coding	[illegible]
50772	NM_015806,NM_027418,XM_011242767	Mapk6	mitogen-activated protein kinase 6	protein_coding	[illegible]
50773	NM_015807,XM_011249123,XR_003949472	Nt5c	5',3'-nucleotidase, cytosolic	protein_coding	[illegible]
50776	NM_015810,NR_027785,NR_148430,NR_148431	Polg2	polymerase (DNA directed), gamma 2, accessory subunit	protein_coding	[illegible]
50778	NM_015811	Rgs1	regulator of G-protein signaling 1	protein_coding	[illegible]
50779	NM_001282061,NM_001310478,NM_015812,XM_006516070,XM_011244133,XM_011244134,XM_011244135,XM_011244136,XM_011244137,XM_011244138	Rgs6	regulator of G-protein signaling 6	protein_coding	[illegible]
50780	NM_001081650,NM_001310706,NM_019492,NM_134257,XM_006538057,XM_006538058,XM_006538060,XM_006538067,XM_006538068,XM_006538069,XM_006538070,XM_006538072,XM_011250065,XM_017320307,XM_017320308,XM_030253652	Rgs3	regulator of G-protein signaling 3	protein_coding	[illegible]
50781	NM_001360257,NM_001360260,NM_001360261,NM_015814	Dkk3	dickkopf WNT signaling pathway inhibitor 3	protein_coding	[illegible]
50782	NM_001081069,XM_030249893	Rgs11	regulator of G-protein signaling 11	protein_coding	[illegible]
50783	NM_001359042,NM_015816,XM_030243665	Lsm4	LSM4 homolog, U6 small nuclear RNA and mRNA degradation associated	protein_coding	[illegible]
50784	NM_001302389,NM_001302390,NM_001302442,NM_015817	Plpp2	phospholipid phosphatase 2	protein_coding	[illegible]
50785	NM_015818	Hs6st1	heparan sulfate 6-O-sulfotransferase 1	protein_coding	[illegible]
50786	NM_001077202,NM_001290467,NM_001290468,NM_015819,XM_006541516,XM_006541518,XM_006541519	Hs6st2	heparan sulfate 6-O-sulfotransferase 2	protein_coding	[illegible]
50787	NM_015820,XM_006519238,XM_011245113	Hs6st3	heparan sulfate 6-O-sulfotransferase 3	protein_coding	[illegible]
50788	NM_015821,XM_011248436,XM_030243666	Fbxl8	F-box and leucine-rich repeat protein 8	protein_coding	[illegible]
50789	NM_001347600,NM_001347601,NM_001360341,NM_001360342,NM_015822,XM_006519240,XM_006519243	Fbxl3	F-box and leucine-rich repeat protein 3	protein_coding	[illegible]
50790	NM_001033600,NM_019477,NM_207625,XM_011247842,XM_011247843,XM_030251379	Acsl4	acyl-CoA synthetase long-chain family member 4	protein_coding	[illegible]
50791	NM_001170745,NM_001170746,NM_015823,XM_006535743,XM_006535744,XM_006535745,XM_006535746,XM_006535747,XM_006535748,XM_006535749,XM_006535750,XM_006535751,XM_006535752,XM_011249777,XM_017320968,XM_017320969,XM_030254656,XM_030254657	Magi2	membrane associated guanylate kinase, WW and PDZ domain containing 2	protein_coding	[illegible]
50793	NM_001159563,NM_015824,XM_011250066,XM_017320309,XM_030253653,XM_030253654	Orc3	origin recognition complex, subunit 3	protein_coding	[illegible]
50794	NM_021366	Klf13	Kruppel-like factor 13	protein_coding	[illegible]
50795	NM_015825	Sh3bgr	SH3-binding domain glutamic acid-rich protein	protein_coding	[illegible]
50796	NM_015826,XM_030250985	Dmrt1	doublesex and mab-3 related transcription factor 1	protein_coding	[illegible]
50797	NM_015827	Copb2	coatomer protein complex, subunit beta 2 (beta prime)	protein_coding	[illegible]
50798	NM_001190414,NM_001357539,NM_015828,XM_006538073,XM_030253655	Gne	glucosamine (UDP-N-acetyl)-2-epimerase/N-acetylmannosamine kinase	protein_coding	[illegible]
50799	NM_001177572,NM_015829,XM_006505136,XM_006505137,XM_030255494	Slc25a13	solute carrier family 25 (mitochondrial carrier, adenine nucleotide translocator), member 13	protein_coding	[illegible]
50817	NM_001347334,NM_015830,NR_151779,XM_006524605,XM_006524606,XM_006524607,XM_006524608,XM_006524609,XM_017317570,XM_017317571,XR_001782087,XR_003952148,XR_003952149,XR_003952150	Capn15	calpain 15	protein_coding	[illegible]
50849	NM_001302448,NM_001302449,NM_016698,XM_006530404,XM_017320970,XM_030254658	Rnf10	ring finger protein 10	protein_coding	[illegible]
50850	NM_001162870,NM_001357738,NM_016962,XM_006524610,XM_011246530,XR_001782088	Spast	spastin	protein_coding	[illegible]
50868	NM_001110305,NM_001110306,NM_001110307,NM_016679	Keap1	kelch-like ECH-associated protein 1	protein_coding	[illegible]
50873	NM_001317726,NM_016694,XM_006523336,XM_006523337,XM_006523339,XM_017317572,XM_017317573,XM_017317574,XM_017317575,XM_017317576,XM_030249894,XM_030249895	Prkn	parkin RBR E3 ubiquitin protein ligase	protein_coding	[illegible]
50874	NM_016712,XM_017319636,XM_017319637,XM_030252670,XM_030252671,XM_030252672	Tmod4	tropomodulin 4	protein_coding	[illegible]
50875	NM_001357828,NM_016963	Tmod3	tropomodulin 3	protein_coding	[illegible]
50876	NM_001038710,NM_016711,XM_006511267,XM_006511268,XM_017313459,XM_030244444,XM_030244445	Tmod2	tropomodulin 2	protein_coding	[illegible]
50877	NM_016720,XM_030242761	Neu3	neuraminidase 3	protein_coding	[illegible]
50878	NM_016964,XM_006504596,XM_006504600,XM_006504601,XM_011240948,XM_017320971,XM_017320972,XR_377146	Stag3	stromal antigen 3	protein_coding	[illegible]
50880	NM_016717,XM_006529743,XM_006529744,XM_006529745,XM_006529746,XR_003956210,XR_003956213,XR_387181	Scly	selenocysteine lyase	protein_coding	[illegible]
50883	NM_001363308,NM_016681,XM_006535068,XM_006535069,XM_006535070,XM_006535071,XM_030254659,XM_030254660,XR_001784718,XR_003955670,XR_003955671,XR_003955672	Chek2	checkpoint kinase 2	protein_coding	[illegible]
50884	NM_001290745,NM_001355410,NM_001355411,NM_016965	Nckap1	NCK-associated protein 1	protein_coding	[illegible]
50887	NM_016710	Hmgn5	high-mobility group nucleosome binding domain 5	protein_coding	[illegible]
50905	NM_019583,XM_030247884	Il17rb	interleukin 17 receptor B	protein_coding	[illegible]
50907	NM_001294302,NM_016703,XM_006504015,XM_006504017,XR_376814	Preb	prolactin regulatory element binding	protein_coding	[illegible]
50908	NM_001097617,NM_144938,XM_006506365,XM_011241404,XM_011241405	C1s1	complement component 1, s subcomponent 1	protein_coding	[illegible]
50909	NM_023143	C1ra	complement component 1, r subcomponent A	protein_coding	[illegible]
50911	NM_019393	Exosc9	exosome component 9	protein_coding	[illegible]
50912	NM_001355489,NM_001355490,NM_016699,NR_131061,XM_006539023,XM_006539024,XM_011250300,XM_017320312	Exosc10	exosome component 10	protein_coding	[illegible]
50913	NM_016967	Olig2	oligodendrocyte transcription factor 2	protein_coding	[illegible]
50914	NM_016968	Olig1	oligodendrocyte transcription factor 1	protein_coding	[illegible]
50915	NM_016719,XM_006499883	Grb14	growth factor receptor bound protein 14	protein_coding	[illegible]
50917	NM_001193645,NM_016722,XM_006531177,XM_017312894,XM_017312895,XR_387755,XR_387756	Galns	galactosamine (N-acetyl)-6-sulfate sulfatase	protein_coding	[illegible]
50918	NM_001093764,NM_001093765,NM_001093766,NM_016969,XM_006540171	Myadm	myeloid-associated differentiation marker	protein_coding	[illegible]
50926	NM_016690,NR_152866,XM_006535074,XM_006535076,XM_006535077,XM_011249510,XM_030254661,XR_003955673,XR_389282	Hnrnpdl	heterogeneous nuclear ribonucleoprotein D-like	protein_coding	[illegible]
50927	NM_001081475,NM_001284229,NM_016777,XM_030253656	Nasp	nuclear autoantigenic sperm protein (histone-binding)	protein_coding	[illegible]
50928	NM_016970	Klrg1	killer cell lectin-like receptor subfamily G, member 1	protein_coding	[illegible]
50930	NM_019418	Tnfsf14	tumor necrosis factor (ligand) superfamily, member 14	protein_coding	[illegible]
50931	NM_016671,XM_011248437,XM_030243668,XR_001778452,XR_003947315	Il27ra	interleukin 27 receptor, alpha	protein_coding	[illegible]
50932	NM_001045959,NM_001045964,NM_016713,NM_176893,XM_006533677,XM_006533678,XM_006533679,XM_006533681,XM_006533682,XM_017314645,XM_017314646,XM_030246117,XM_030246118,XR_388490	Mink1	misshapen-like kinase 1 (zebrafish)	protein_coding	[illegible]
50933	NM_016723,XM_006519246,XM_006519247,XM_006519248	Uchl3	ubiquitin carboxyl-terminal esterase L3 (ubiquitin thiolesterase)	protein_coding	[illegible]
50934	NM_016972,XM_006519250,XM_011245114	Slc7a8	solute carrier family 7 (cationic amino acid transporter, y+ system), member 8	protein_coding	[illegible]
50935	NM_001025310,NM_001025311,NM_001289547,NM_001289548,NM_001289549,NM_016973,NR_110347	St6galnac6	ST6 (alpha-N-acetyl-neuraminyl-2,3-beta-galactosyl-1,3)-N-acetylgalactosaminide alpha-2,6-sialyltransferase 6	protein_coding	[illegible]
50994	NR_015480,NR_027802	Mtag2	metastasis associated gene 2	lncRNA	[illegible]
50995	NM_016682,XM_017322348	Uba2	ubiquitin-like modifier activating enzyme 2	protein_coding	[illegible]
50996	NM_016688	Pdcd7	programmed cell death 7	protein_coding	[illegible]
50997	NM_001356324,NM_016695,XM_006533684,XM_006533685,XM_030246119,XM_030246120	Mpp2	membrane protein, palmitoylated 2 (MAGUK p55 subfamily member 2)	protein_coding	[illegible]
51786	NM_016856,XM_006516071	Cpsf2	cleavage and polyadenylation specific factor 2	protein_coding	[illegible]
51788	NM_001316995,NM_016750	H2afz	H2A histone family, member Z	protein_coding	[illegible]
51789	NM_001110147,NM_001289443,NM_001347185,NM_016788,XM_006522332,XM_006522333,XM_006522334,XM_006522335,XM_006522336,XM_006522337,XM_006522338,XM_006522339,XM_006522340,XM_006522342,XM_006522343,XM_006522344,XM_011245946,XM_017317065,XM_017317066,XM_030249174,XM_030249175,XM_030249176	Tnk2	tyrosine kinase, non-receptor, 2	protein_coding	[illegible]
51791	NM_001360714,NM_016758	Rgs14	regulator of G-protein signaling 14	protein_coding	[illegible]
51792	NM_016891	Ppp2r1a	protein phosphatase 2, regulatory subunit A, alpha	protein_coding	[illegible]
51793	NM_001190449,NM_016765,XM_006524612,XM_030249896	Ddah2	dimethylarginine dimethylaminohydrolase 2	protein_coding	[illegible]
51795	NM_001365087,NM_016911	Srpx	sushi-repeat-containing protein	protein_coding	[illegible]
51796	NM_001130477,NM_001369067,NM_001369068,NM_001369069,NM_001369070,NM_001369071,NM_001369072,NM_001369073,NM_001369074,NM_016799,XM_006539026,XM_006539029,XM_006539032,XM_006539033,XM_017320313,XM_030253657,XM_030253658,XM_030253659,XM_030253660,XM_030253661,XM_030253662,XM_030253663,XM_030253664,XM_030253665,XM_030253666,XM_030253667,XM_030253668,XM_030253669,XR_003954981,XR_003954982	Srrm1	serine/arginine repetitive matrix 1	protein_coding	[illegible]
51797	NM_001355491,NM_016748	Ctps	cytidine 5'-triphosphate synthase	protein_coding	[illegible]
51798	NM_016772	Ech1	enoyl coenzyme A hydratase 1, peroxisomal	protein_coding	[illegible]
51799	NM_001252347,NM_001362803,NM_001362804,NM_016759,XM_006533688,XM_006533689,XM_006533690,XM_006533691,XM_006533692,XM_006533693,XM_006533694,XM_011249124,XM_017314647,XM_017314648,XM_030246121,XR_001780024,XR_003949473,XR_003949474,XR_003949475,XR_003949476	Rundc3a	RUN domain containing 3A	protein_coding	[illegible]
51800	NM_016778	Bok	BCL2-related ovarian killer	protein_coding	[illegible]
51801	NM_001168392,NM_016894,NM_178401,XM_011248050,XM_017321671	Ramp1	receptor (calcitonin) activity modifying protein 1	protein_coding	[illegible]
51810	NM_016805,NR_149827,XR_003956223,XR_003956224	Hnrnpu	heterogeneous nuclear ribonucleoprotein U	protein_coding	[illegible]
51811	NM_016751	Clec4f	C-type lectin domain family 4, member f	protein_coding	[illegible]
51812	NM_001164156,NM_016766,XM_030248640	Mcrs1	microspherule protein 1	protein_coding	[illegible]
51813	NM_001122982,NM_001290420,NM_001290422,NM_016746	Ccnc	cyclin C	protein_coding	[illegible]
51869	NM_001355414,NM_001355415,NM_175238,XM_006498179,XM_011239135,XM_011239136,XM_011239137,XM_030251862	Rif1	replication timing regulatory factor 1	protein_coding	[illegible]
51875	NM_001040130,NM_001109993,XM_006498180	Tmem141	transmembrane protein 141	protein_coding	[illegible]
51885	NM_001290824,NM_153387,NR_077245,NR_110985,XM_0302518	Tubgcp4	tubulin, gamma complex associated protein 4	protein_coding	[illegible]
51886	NM_001355366,NM_001355367,NM_001355368,NM_001355369,NM_001355370,NM_001355371,NM_001355372,NM_001355373,NM_001355374,NM_057172,NR_149713,XM_006501660,XM_006501662,XM_006501663,XM_006501664,XM_006501672,XM_030252673,XM_030252674,XR_001783705,XR_001783706,XR_001783707,XR_001783708,XR_001783709,XR_001783710,XR_001783711,XR_001783712,XR_001783714,XR_003954350,XR_003954351,XR_003954352,XR_003954353,XR_003954354,XR_003954355,XR_003954356,XR_375543	Fubp1	far upstream element (FUSE) binding protein 1	protein_coding	[illegible]
51897	NM_001355416,NM_001355417,NM_001355418,NM_001355419,NM_001355420,NM_001355421,NM_145528,XM_006499892	Atg13	autophagy related 13	protein_coding	[illegible]
51902	NM_178607	Rnf24	ring finger protein 24	protein_coding	[illegible]

51938	NM_026222,XM_006535498,XM_006535499,XM_006535500,XM_006535502,XM_011249686,XR_001783715,XR_003954357,XR_003954358,XR_003954359	Ccdc39	coiled-coil domain containing 39	protein_coding	NP_080498.1;XP_006535561.1;XP_006535562.1;XP_006535563.1;XP_006535565.1;XP_011247988.1	1289263	.	0.889697
51944	NM_026412,XM_017319123,XR_374487,XR_374488	Knstrn	kinetochore-localized astrin/SPAG5 binding	protein_coding	NP_080688.2;XP_017174612.1	1289298	.	8.920850
51960	NM_001159864,NM_001159865,NR_027630,XM_006496135,XM_006496136,XM_017321676,XM_017321677	Kctd18	potassium channel tetramerisation domain containing 18	protein_coding	NP_001153336.1;NP_001153337.1;XP_006496198.1;XP_006496199.1;XP_017177165.1;XP_017177166.1	3603813	.	4.754327
52004	NM_026373,XM_006531787	Cdk2ap2	CDK2-associated protein 2	protein_coding	NP_080649.1;XP_006531850.1	1098779	.	5.351588
52009	NM_198937	Jpt2	Jupiter microtubule associated homolog 2	protein_coding	NP_945175.1	1196260	.	20.227812
52013	NM_177464,XM_006527182	R3hcc1l	R3H domain and coiled-coil containing 1 like	protein_coding	NP_803415.1;XP_006527245.1	1196316	.	5.661565
52014	NM_030250	Nus1	NUS1 dehydrodolichyl diphosphate synthase subunit	protein_coding	NP_084526.1	1196365	.	16.860821
52020	NM_177465,XM_006524614	Umodl1	uromodulin-like 1	protein_coding	NP_803416.2;XP_006524677.1	1929785	.	0.000000
52023	NM_029320,XM_006519251,XM_006519253,XM_006519256,XM_030247885,XR_001781130,XR_003950865,XR_383193	Pibf1	progesterone immunomodulatory binding factor 1	protein_coding	NP_083596.2;XP_006519314.1;XP_006519316.1;XP_006519319.1;XP_030103745.1	1261910	.	2.683393
52024	NM_024204	Ankrd22	ankyrin repeat domain 22	protein_coding	NP_077166.4	1277101	.	0.000000
52028	NM_001033128,XM_006531788	Bbs1	Bardet-Biedl syndrome 1 (human)	protein_coding	NP_001028300.1;XP_006531851.1	1277215	.	2.028158
52033	NM_023209,XM_006519257	Pbk	PDZ binding kinase	protein_coding	NP_075698.1;XP_006519320.1	1289156	.	9.539195
52036	NM_001164159,NM_001360429,NM_001360430,NM_028999,NM_029456,XM_006531792,XM_006531794,XM_006531797,XM_011248640,XM_011248641,XM_011248642,XM_017318248,XM_017318249,XM_017318250,XM_030250986,XM_030250987,XM_030250988,XR_001782603	Ppp6r3	protein phosphatase 6, regulatory subunit 3	protein_coding	NP_001157631.1;NP_001347358.1;NP_001347359.1;NP_083275.1;NP_083732.1;XP_006531855.1;XP_006531857.1;XP_006531860.1;XP_011246942.1;XP_011246943.1;XP_011246944.1;XP_017173737.1;XP_017173738.1;XP_017173739.1;XP_030106846.1;XP_030106847.1;XP_030106848.1	1921807	.	23.287792
52040	NM_001163818,NM_001357740,XM_006524616,XM_006524617,XM_006524619,XM_030249898	Ppp1r10	protein phosphatase 1, regulatory subunit 10	protein_coding	NP_001157290.1;NP_001344669.1;XP_006524679.1;XP_006524680.1;XP_006524682.1;XP_030105758.1	1289273	.	9.254965
52055	NM_001003955,NM_177466,XM_006506366	Rab11fip5	RAB11 family interacting protein 5 (class I)	protein_coding	NP_001003955.1;NP_803417.3;XP_006506429.1	1098586	.	10.242656
52064	NM_026504,XR_387572	Coq5	coenzyme Q5 methyltransferase	protein_coding	NP_080780.1	1098643	.	7.323417
52065	NM_001081279,NM_001364259,NR_157105	Mfhas1	malignant fibrous histiocytoma amplified sequence 1	protein_coding	NP_001074748.1;NP_001351188.1	1098644	.	1.647381
52076	NM_028053,XM_006538083	Tmem38b	transmembrane protein 38B	protein_coding	NP_082329.1;XP_006538146.1	1098718	.	4.234149
52118	NM_027514	Pvr	poliovirus receptor	protein_coding	NP_081790.1	107741	.	4.483687
52120	NM_029884	Hgsnat	heparan-alpha-glucosaminide N-acetyltransferase	protein_coding	NP_084160.1	1196297	.	23.194159
52123	NM_026792	Agpat5	1-acylglycerol-3-phosphate O-acyltransferase 5 (lysophosphatidic acid acyltransferase, epsilon)	protein_coding	NP_081068.1	1196345	.	6.691185
52132	NM_001368381,NM_001368382,NM_001368383,NM_001368384,NM_028771,NR_160796	Ccdc97	coiled-coil domain containing 97	protein_coding	NP_001355310.1;NP_001355311.1;NP_001355312.1;NP_001355313.1;NP_083047.1	1196455	.	14.835922
52150	NM_001033525	Kcnk6	potassium inwardly-rectifying channel, subfamily K, member 6	protein_coding	NP_001028697.2	1891291	.	11.000983
52163	NM_133926	Camk1	calcium/calmodulin-dependent protein kinase I	protein_coding	NP_598687.1	1098535	.	34.232082
52174	NM_025667,NR_110978,XM_006539036	Tmem222	transmembrane protein 222	protein_coding	NP_079943.2;XP_006539099.1	1098568	.	17.611090
52184	NM_001162538,NM_001162539,NM_025714,XM_006501675,XM_006501676,XM_006501677,XM_006501678,XM_006501679,XM_006501680,XM_006501682,XM_006501683,XM_006501688,XM_011240157,XM_011240158,XM_011240161,XM_017319642,XM_017319643,XM_017319645,XM_030252676,XM_030252677,XM_030252678,XM_030252679,XM_030252680,XM_030252681,XM_030252682,XM_030252683	Odf2l	outer dense fiber of sperm tails 2-like	protein_coding	NP_001156010.1;NP_001156011.1;NP_079990.2;XP_006501738.1;XP_006501739.1;XP_006501740.1;XP_006501741.1;XP_006501742.1;XP_006501743.1;XP_006501744.1;XP_006501745.1;XP_006501746.1;XP_006501751.1;XP_011238459.1;XP_011238460.1;XP_011238463.1;XP_017175131.1;XP_017175132.1;XP_017175134.1;XP_030108536.1;XP_030108537.1;XP_030108538.1;XP_030108539.1;XP_030108540.1;XP_030108541.1;XP_030108542.1;XP_030108543.1	1098600	.	6.664289
52187	NM_001369055,NM_027491	Rragd	Ras-related GTP binding D	protein_coding	NP_001355984.1;NP_081767.2	1098604	.	3.147162
52202	NM_172762,XM_006531182,XR_001778453	Rbm34	RNA binding motif protein 34	protein_coding	NP_766350.2;XP_006531245.1	1098653	.	4.281775
52206	NM_024213	Anapc4	anaphase promoting complex subunit 4	protein_coding	NP_077175.1	1098673	.	30.380888
52222	XR_881941	D7Ertd128e	DNA segment, Chr 7, ERATO Doi 128, expressed	lncRNA	.	1098716	.	0.000000
52231	NM_001267620,NM_026187,NR_051998,NR_051999,XM_006496507,XM_011238696,XM_030255491,XM_030255492,XM_030255493,XR_003956227	Ankzf1	ankyrin repeat and zinc finger domain containing 1	protein_coding	NP_001254549.1;NP_080463.3;XP_006496570.1;XP_011236998.1;XP_030111351.1;XP_030111352.1;XP_030111353.1	1098746	.	8.006993
52245	NM_175095	Commd2	COMM domain containing 2	protein_coding	NP_780304.2	1098806	.	5.246090
52250	NM_178608,XM_030255495	Reep1	receptor accessory protein 1	protein_coding	NP_848723.1;XP_030111355.1	1098827	.	3.941226
52276	NM_026560	Cdca8	cell division cycle associated 8	protein_coding	NP_080836.3	1196274	.	9.920119
52285	NR_027803	D6Ertd474e	DNA segment, Chr 6, ERATO Doi 474, expressed	lncRNA	.	1196315	.	0.034319
52323	NM_001161800,NM_026448,XM_006535753,XR_880834	Klhl7	kelch-like 7	protein_coding	NP_001155272.1;NP_080724.2;XP_006535816.1	1196453	.	12.189050
52331	NM_175096	Stbd1	starch binding domain 1	protein_coding	NP_780305.1	1261768	.	8.463520
52335	NM_001080930,NM_001368350,NM_001368351,NM_001368352,XM_011248439,XM_030243670	Atxn1l	ataxin 1-like	protein_coding	NP_001074399.1;NP_001355279.1;NP_001355280.1;NP_001355281.1;XP_011246741.1;XP_030099530.1	3694797	.	8.076041
52348	NM_033560,XM_030243671,XR_003947316,XR_003947317	Vps37a	vacuolar protein sorting 37A	protein_coding	NP_291038.2;XP_030099531.1	1261835	.	5.881969
52357	NM_133791	Wwc2	WW, C2 and coiled-coil domain containing 2	protein_coding	NP_598552.2	1261872	.	10.023547
52372	NM_001167937,NM_001167938,XM_006506367,XM_006506368,XM_017321675	D6Ertd527e	DNA segment, Chr 6, ERATO Doi 527, expressed	protein_coding	NP_001161409.1;NP_001161410.1;XP_006506430.1;XP_006506431.1;XP_017177164.1	1261919	.	0.277495
52377	NM_001355045,NM_026555,XM_006540987	Rcn3	reticulocalbin 3, EF-hand calcium binding domain	protein_coding	NP_001341974.1;NP_080831.2;XP_006541050.1	1277122	.	426.497762
52389	NM_177469,XM_006536203	Adgra1	adhesion G protein-coupled receptor A1	protein_coding	NP_803420.2;XP_006536266.1	1277167	.	0.058118
52392	NM_001355071,NM_001355072,NM_001355073,NM_001355074,NM_133825,XM_006529751	D1Ertd622e	DNA segment, Chr 1, ERATO Doi 622, expressed	protein_coding	NP_001342000.1;NP_001342001.1;NP_001342002.1;NP_001342003.1;NP_598586.1;XP_006529814.1	1277184	.	5.938376
52397	NM_001363318,NM_001363319,NM_001363320,NM_001363321,NM_001363322,NM_001363323,NM_026856,NR_156476,XM_006535079,XM_006535083,XM_006535084,XM_006535085,XM_006535086,XM_006535087,XM_006535090,XM_006535097,XM_011249511,XM_011249512,XM_011249516,XM_011249517,XM_011249524,XM_011249526,XM_011249527,XM_011249528,XM_017320974,XM_030254662,XM_030254663,XM_030254664,XM_030254665,XM_030254666,XM_030254667,XM_030254668,XM_030254669,XM_030254670,XM_030254671,XM_030254672,XM_030254673,XM_030254674,XM_030254675,XM_030254676,XM_030254677,XM_030254678,XM_030254679,XM_030254680,XM_030254681,XM_030254682,XM_030254683,XM_030254684,XM_030254685,XM_030254686,XM_030254687,XM_030254688	Zfp644	zinc finger protein 644	protein_coding	NP_001350247.1;NP_001350248.1;NP_001350249.1;NP_001350250.1;NP_001350251.1;NP_001350252.1;NP_081132.2;XP_006535142.1;XP_006535146.1;XP_006535147.1;XP_006535148.1;XP_006535149.1;XP_006535150.1;XP_006535153.1;XP_006535160.1;XP_011247813.1;XP_011247814.1;XP_011247818.1;XP_011247819.1;XP_011247826.1;XP_011247828.1;XP_011247829.1;XP_011247830.1;XP_017176463.1;XP_030110522.1;XP_030110523.1;XP_030110524.1;XP_030110525.1;XP_030110526.1;XP_030110527.1;XP_030110528.1;XP_030110529.1;XP_030110530.1;XP_030110531.1;XP_030110532.1;XP_030110533.1;XP_030110534.1;XP_030110535.1;XP_030110536.1;XP_030110537.1;XP_030110538.1;XP_030110539.1;XP_030110540.1;XP_030110541.1;XP_030110542.1;XP_030110543.1;XP_030110544.1;XP_030110545.1;XP_030110546.1;XP_030110547.1;XP_030110	1277212	.	7.314172
52398	NM_001009818,NM_001310669,NM_001310671,NM_001347377,XM_006535098,XM_006535099,XM_006535100,XM_006535101,XM_006535103,XM_006535104,XM_006535106,XM_006535108,XM_011249531,XM_011249532,XM_017320978,XM_017320980,XM_017320981,XM_017320982,XM_030254690,XM_030254691,XR_001784719,XR_389284	Sept11	septin 11	protein_coding	NP_001009818.1;NP_001297598.1;NP_001297600.1;NP_001334306.1;XP_006535161.1;XP_006535162.1;XP_006535163.1;XP_006535164.1;XP_006535166.1;XP_006535167.1;XP_006535169.1;XP_006535171.1;XP_011247833.1;XP_011247834.1;XP_017176467.1;XP_017176469.1;XP_017176470.1;XP_017176471.1;XP_030110550.1;XP_030110551.1	1277214	.	28.327293
52401	NR_131933	D5Ertd615e	DNA segment, Chr 5, ERATO Doi 615, expressed	lncRNA	.	1277236	.	0.020400
52428	NM_027897,XR_391076	Rhpn2	rhophilin, Rho GTPase binding protein 2	protein_coding	NP_082173.3	1289234	.	3.276653
52430	NM_001254754,NM_026728,XM_011240573,XM_017320314,XM_030253670,XR_003954983	Echdc2	enoyl Coenzyme A hydratase domain containing 2	protein_coding	NP_001241683.1;NP_081004.2;XP_011238875.1;XP_017175803.1;XP_030109530.1	1289238	.	13.917409
52432	NM_001347618,NM_026391,XM_006508015,XM_006508020,XM_017322350,XM_030242762,XM_030242763,XM_030242764,XM_030242765,XM_030242766	Ppp2r2d	protein phosphatase 2, regulatory subunit B, delta	protein_coding	NP_001334547.1;NP_080667.1;XP_006508078.1;XP_006508083.1;XP_017177839.1;XP_030098622.1;XP_030098623.1;XP_030098624.1;XP_030098625.1;XP_030098626.1	1289252	.	19.471321
52440	NM_001355595,NM_001355596,NM_025816,XM_006506370	Tax1bp1	Tax1 (human T cell leukemia virus type I) binding protein 1	protein_coding	NP_001342524.1;NP_001342525.1;NP_080092.2;XP_006506433.1	1289308	.	32.960565
52443	NM_198831,NR_003559,NR_153424,NR_153425,NR_153426,NR_153427,NR_153428,XM_006508021,XM_030242767	Mrpl48	mitochondrial ribosomal protein L48	protein_coding	NP_942128.2;XP_006508084.1;XP_030098627.1	1289321	.	19.038267
52463	NM_001253857,XM_006513867,XM_017314027,XM_030245174,XM_030245175,XR_003948738	Tet1	tet methylcytosine dioxygenase 1	protein_coding	NP_001240786.1;XP_006513930.1;XP_017169516.1;XP_030101034.1;XP_030101035.1	1098693	.	1.955851
52466	NM_026740	Slc46a1	solute carrier family 46, member 1	protein_coding	NP_081016.2	1098733	.	3.624602
52468	NM_001113470,NM_146012	Ctdsp2	CTD (carboxy-terminal domain, RNA polymerase II, polypeptide A) small phosphatase 2	protein_coding	NP_001106941.1;NP_666124.1	1098748	.	76.615868
52469	NM_026618	Coa3	cytochrome C oxidase assembly factor 3	protein_coding	NP_080894.1	1098757	.	27.703197
52477	NM_001199020,NM_001357131,NM_001357132,NM_001357133,NM_001357134,NM_021421,NR_151630,XM_006497183,XM_006497184,XM_006497186,XM_017321690,XM_030255501,XM_030255504,XM_030255505,XM_030255506,XM_030255508,XR_003956234,XR_003956236,XR_373773	Angel2	angel homolog 2	protein_coding	NP_001185949.1;NP_001344060.1;NP_001344061.1;NP_001344062.1;NP_001344063.1;NP_067396.3;XP_006497246.1;XP_006497247.1;XP_006497249.1;XP_017177179.1;XP_030111361.1;XP_030111364.1;XP_030111365.1;XP_030111366.1;XP_030111368.1	1196310	.	21.505920
52480	NR_146211	Snhg14	small nucleolar RNA host gene 14	lncRNA	.	1289201	.	0.108903
52502	NM_025821,XM_006522346,XM_006522347,XM_006522348	Carhsp1	calcium regulated heat stable protein 1	protein_coding	NP_080097.1;XP_006522409.2;XP_006522410.1;XP_006522411.1	1196368	.	16.386404
52504	NM_001361641,NM_134046,XM_006515152,XM_011243883,XM_011243884,XM_030246806,XM_030246807,XR_381196,XR_872422	Cenpo	centromere protein O	protein_coding	NP_001348570.1;NP_598807.2;XP_006515215.1;XP_011242185.1;XP_011242186.1;XP_030102666.1;XP_030102667.1	1923800	.	4.426165
52512	NR_105025	D14Ertd670e	DNA segment, Chr 14, ERATO Doi 670, expressed	lncRNA	.	1277153	.	0.000000
52513	NM_026538,XM_011243731,XM_017314650	Ddx56	DEAD (Asp-Glu-Ala-Asp) box polypeptide 56	protein_coding	NP_080814.1;XP_011242033.1;XP_017170139.1	1277172	.	8.422218
52521	NM_144523	Zfp622	zinc finger protein 622	protein_coding	NP_653106.1	1289282	.	10.093385
52530	NM_001364736,NM_026631,NR_157294	Nhp2	NHP2 ribonucleoprotein	protein_coding	NP_001351665.1;NP_080907.1	1098547	.	23.975332
52535	NM_001029990	Mettl17	methyltransferase like 17	protein_coding	NP_001025161.1	1098577	.	9.992287
52538	NM_177470	Acaa2	acetyl-Coenzyme A acyltransferase 2 (mitochondrial 3-oxoacyl-Coenzyme A thiolase)	protein_coding	NP_803421.1	1098623	.	33.911728
52551	NM_001358549,NM_001358550,NM_024499,XM_006513877,XM_030245176	Sgta	small glutamine-rich tetratricopeptide repeat (TPR)-containing, alpha	protein_coding	NP_001345478.1;NP_001345479.1;NP_078775.1;XP_006513940.1;XP_030101036.1	1098703	.	32.230653
52552	NM_001081009,XM_006517718,XM_006517719,XM_006517720,XM_011244679,XM_011244680,XM_030247321,XM_030247322	Parp8	poly (ADP-ribose) polymerase family, member 8	protein_coding	NP_001074478.2;XP_006517781.1;XP_006517782.1;XP_006517783.1;XP_011242981.1;XP_011242982.1;XP_030103181.1;XP_03010318	1098713	.	8.071751
52563	NM_178347,XM_006526084	Cdc23	CDC23 cell division cycle 23	protein_coding	NP_848124.1;XP_006526147.1	1098815	.	19.933821
52570	NM_177471,XM_011249125,XR_003949477	Ccdc69	coiled-coil domain containing 69	protein_coding	NP_803422.2;XP_011247427.1	1196234	.	0.294598
52575	NM_029092	Trmt10c	tRNA methyltransferase 10C, mitochondrial RNase P subunit	protein_coding	NP_083368.1	1196261	.	6.884938
52585	NM_026819,XM_030247886	Dhrs1	dehydrogenase/reductase (SDR family) member 1	protein_coding	NP_081095.2;XP_030103746.1	1196314	.	21.804377
52588	NM_001316748,NM_145928,XM_006519261,XM_017316081	Tspan14	tetraspanin 14	protein_coding	NP_001303677.1;NP_666040.1;XP_006519324.1;XP_017171570.1	1196325	.	18.082937
52589	NM_001170866,NM_001170867,NM_001170868,NM_001359839,NM_001359840,NM_001359841,NM_001359842,NM_001359843,NM_001359844,NM_001359845,NM_134094,XM_006520111,XM_006520112,XM_006520113,XM_011245362,XM_011245364,XM_011245366,XM_017316693,XM_030248642,XM_030248643,XM_030248644,XM_030248645,XM_030248646,XM_030248647,XM_030248648,XM_030248649,XM_030248650,XM_030248651,XM_030248652,XM_030248653,XM_030248654,XM_030248655,XM_030248656	Ncald	neurocalcin delta	protein_coding	NP_001164337.1;NP_001164338.1;NP_001164339.1;NP_001346768.1;NP_001346769.1;NP_001346770.1;NP_001346771.1;NP_001346772.1;NP_001346773.1;NP_001346774.1;NP_598855.2;XP_006520174.1;XP_006520175.1;XP_006520176.1;XP_011243664.1;XP_011243666.1;XP_011243668.1;XP_017172182.1;XP_030104502.1;XP_030104503.1;XP_030104504.1;XP_030104505.1;XP_030104506.1;XP_030104507.1;XP_030104508.1;XP_030104509.1;XP_030104510.1;XP_030104511.1;XP_030104512.1;XP_030104513.1;XP_030104514.1;XP_030104515.1;XP_030104516.1	1196326	.	4.109562
52592	NM_001037756,NR_155205,XR_381530	Brms1l	breast cancer metastasis-suppressor 1-like	protein_coding	NP_001032845.1	1196337	.	15.815221
52609	NM_144811,XM_006521144,XM_006521147,XM_006521148,XM_011245690,XM_011245691,XM_011245692,XR_003951404,XR_384	Cbx7	chromobox 7	protein_coding	NP_659060.1;XP_006521207.1;XP_006521210.1;XP_006521211.1;XP_011243992.1;XP_011243993.1;XP_011243994.1	1196439	.	1.140161
52614	NM_139138	Adgre4	adhesion G protein-coupled receptor E4	protein_coding	NP_631877.2	1196464	.	0.125435
52615	NM_001163018,NM_199196	Suz12	SUZ12 polycomb repressive complex 2 subunit	protein_coding	NP_001156490.1;NP_954666.1	1261758	.	13.053729
52626	NM_029976	Cdkn2aipnl	CDKN2A interacting protein N-terminal like	protein_coding	NP_084252.1	1261797	.	10.801312
52633	NM_023175	Nit2	nitrilase family, member 2	protein_coding	NP_075664.1	1261838	.	11.198849
52635	NM_028731,XM_006516072,XM_006516075,XM_011244139,XM_017315129,XM_017315130,XM_017315131,XM_030246808,XM_030246810,XR_001780466,XR_001780467,XR_003950062,XR_003950063,XR_003950064	Esyt2	extended synaptotagmin-like protein 2	protein_coding	NP_083007.2;XP_006516135.1;XP_006516138.1;XP_011242441.1;XP_017170618.1;XP_017170619.1;XP_017170620.1;XP_030102668.1;XP_030102670.1	1261845	.	20.618165
52637	NM_134007	Cisd1	CDGSH iron sulfur domain 1	protein_coding	NP_598768.1	1261855	.	11.611464
52639	NM_145940	Wipi1	WD repeat domain, phosphoinositide interacting 1	protein_coding	NP_666052.1	1261864	.	38.319462
52653	NM_001290697,NM_026023,XM_030246122	Nudcd2	NudC domain containing 2	protein_coding	NP_001277626.1;NP_080299.4;XP_030101982.1	1277103	.	25.087241
52662	NM_001357444,NM_172631,XM_006526085,XM_006526086,XM_006526087,XM_006526088,XM_006526089,XM_006526092,XM_011246973,XM_030250536,XM_030250537,XM_030250538,XM_030250539,XM_030250540	Ldlrad4	low density lipoprotein receptor class A domain containing 4	protein_coding	NP_001344373.1;NP_766219.2;XP_006526148.1;XP_006526149.1;XP_006526150.1;XP_006526151.1;XP_006526152.1;XP_006526155.1;XP_011245275.1;XP_030106396.1;XP_030106397.1;XP_030106398.1;XP_030106399.1;XP_030106400.1	1277150	.	8.869935
52665	NM_001110195,NM_025855,XM_006512797	Echdc1	enoyl Coenzyme A hydratase domain containing 1	protein_coding	NP_001103665.1;NP_080131.4;XP_006512860.1	1277169	.	2.009736
52666	NM_001166413,NM_001358557,NM_001358558,NM_028027,XM_006513879,XM_006513881,XM_030245177,XR_871718	Arhgef25	Rho guanine nucleotide exchange factor (GEF) 25	protein_coding	NP_001159885.1;NP_001345486.1;NP_001345487.1;NP_082303.2;XP_006513942.1;XP_006513944.1;XP_030101037.1	1277173	.	54.468175
52668	NM_001364173,NM_001364174,NM_026790,NM_194066,NM_194067,NM_194068,NM_194069,XM_006516077	Ifi27	interferon, alpha-inducible protein 27	protein_coding	NP_001351102.1;NP_001351103.1;NP_081066.2;NP_919042.2;NP_919043.1;NP_919044.1;NP_919045.1;XP_006516140.1	1277180	.	30.089672
52670	NM_001164532,NM_001362806,NM_026682,NM_029794,XM_006533701,XM_006533704,XM_006533705,XM_006533706,XM_006533707,XM_006533709,XM_006533710,XM_017314655,XM_017314656,XM_017314657,XM_030246123,XM_030246124,XM_030246125,XM_030246126,XM_030246127	Cpsf4l	cleavage and polyadenylation specific factor 4-like	protein_coding	NP_001158004.1;NP_001349735.1;NP_080958.2;NP_084070.1;XP_006533764.1;XP_006533767.1;XP_006533768.1;XP_006533769.1;XP_006533770.1;XP_006533772.1;XP_006533773.1;XP_017170144.1;XP_017170145.1;XP_017170146.1;XP_030101983.1;XP_030101984.1;XP_030101985.1;XP_030101986.1;XP_030101987.1	1277182	.	0.092130
52673	XR_003950532	D13Ertd608e	DNA segment, Chr 13, ERATO Doi 608, expressed	misc_RNA	.	1277199	.	0.000000
52679	NM_001358560,NM_178609,XM_006513883	E2f7	E2F transcription factor 7	protein_coding	NP_001345489.1;NP_848724.2;XP_006513946.1	1289147	.	2.168128
52683	NM_001115132,NM_001271600,NM_001271601,NR_073376,XM_006521149,XM_006521150,XR_001781550,XR_001781551	Ncaph2	non-SMC condensin II complex, subunit H2	protein_coding	NP_001108604.1;NP_001258529.1;NP_001258530.1;XP_006521212.1;XP_006521213.1	1289164	.	30.975450
52685	NM_001160711,NM_001160712,NM_001160713,NM_027987,XM_006533713	Cd300lg	CD300 molecule like family member G	protein_coding	NP_001154183.1;NP_001154184.1;NP_001154185.1;NP_082263.2;XP_006533776.1	1289168	.	5.878961
52686	NM_172567,XM_006533714,XM_006533715	Mettl2	methyltransferase like 2	protein_coding	NP_766155.3;XP_006533777.1;XP_006533778.1	1289171	.	7.143680
52690	NM_001364266,NM_001364267,NM_001364268,NM_001364269,NM_001364270,NM_028262,XM_006516078,XM_006516081,XM_017315133,XM_030246811,XR_003950065,XR_003950066	Setd3	SET domain containing 3	protein_coding	NP_001351195.1;NP_001351196.1;NP_001351197.1;NP_001351198.1;NP_001351199.1;NP_082538.2;XP_006516141.4;XP_006516144.4;XP_017170622.1;XP_030102671.1	1289184	.	16.959549
52696	NM_001293683,NM_001293684,NM_025635	Zwint	ZW10 interactor	protein_coding	NP_001280612.1;NP_001280613.1;NP_079911.1	1289227	.	23.743583
52700	NM_026559	Txndc17	thioredoxin domain containing 17	protein_coding	NP_080835.1	1289248	.	28.109972
52705	NM_178610	Krr1	KRR1, small subunit (SSU) processome component, homolog (yeast)	protein_coding	NP_848725.2	1289274	.	6.373725
52708	NM_001252582,NM_001252583,NM_001364223,NM_144833,NR_045546,NR_045615,NR_157102,NR_157103,XM_006516085,XM_006516086,XM_006516087,XR_003950067	Zfp410	zinc finger protein 410	protein_coding	NP_001239511.1;NP_001239512.1;NP_001351152.1;NP_659082.1;XP_006516148.1;XP_006516149.1;XP_006516150.1	1289280	.	10.891258
52710	NM_029643,XM_006521151	Slc52a2	solute carrier protein 52, member 2	protein_coding	NP_083919.1;XP_006521214.1	1289288	.	1.230471
52712	NM_001302374,NM_001302375,NM_001362834,NM_026107,XM_006533717,XM_030246128,XM_030246129	Zkscan6	zinc finger with KRAB and SCAN domains 6	protein_coding	NP_001289303.1;NP_001289304.1;NP_001349763.1;NP_080383.2;XP_006533780.1;XP_030101988.1;XP_030101989.1	1289293	.	4.096442
52713	NM_025602	Ccdc59	coiled-coil domain containing 59	protein_coding	NP_079878.1	1289302	.	21.749847
52715	NM_025918,XM_006533720	Ccdc43	coiled-coil domain containing 43	protein_coding	NP_080194.1;XP_006533783.1	1289318	.	8.438898
52717	NM_001347451,NM_025514	Anapc16	anaphase promoting complex subunit 16	protein_coding	NP_001334380.1;NP_079790.1	1289325	.	20.106818
52808	NM_001358369,NM_029836	Tspyl2	TSPY-like 2	protein_coding	NP_001345298.1;NP_084112.1	106244	.	18.770001
52815	NM_027570	Ldhd	lactate dehydrogenase D	protein_coding	NP_081846.3	106428	.	0.648241
52822	NM_001289774,NM_001289775,NM_001289776,NM_001289777,NM_001359208,NM_001359209,NM_027530,XM_006535109,XM_006535111,XM_006535112,XM_006535114,XM_006535115,XM_006535116,XM_017320983,XM_017320984,XR_001784720,XR_001784721,XR_001784722,XR_001784723,XR_001784724,XR_001784725,XR_003955674,XR_003955675,XR_389285	Rufy3	RUN and FYVE domain containing 3	protein_coding	NP_001276703.1;NP_001276704.1;NP_001276705.1;NP_001276706.1;NP_001346137.1;NP_001346138.1;NP_081806.1;XP_006535172.1;XP_006535174.1;XP_006535175.1;XP_006535177.1;XP_006535178.1;XP_006535179.1;XP_017176472.1;XP_017176473.1	106484	.	7.374099
52829	NM_026821	Lurap1l	leucine rich adaptor protein 1-like	protein_coding	NP_081097.2	106510	.	6.432196
52830	NM_026383,XM_006539038,XM_006539039	Pnrc2	proline-rich nuclear receptor coactivator 2	protein_coding	NP_080659.1;XP_006539101.1;XP_006539102.1	106512	.	46.111378
52837	NM_029148	Tmx4	thioredoxin-related transmembrane protein 4	protein_coding	NP_083424.1	106558	.	8.968721
52838	NM_001139503,NM_001139504,NM_026828	Dnlz	DNL-type zinc finger	protein_coding	NP_001132975.1;NP_001132976.1;NP_081104.1	106559	.	4.710533
52840	NM_001048227,NM_001048228,NM_001048229,NM_026797,XM_006499895,XM_030251864	Dbndd2	dysbindin (dystrobrevin binding protein 1) domain containing 2	protein_coding	NP_001041692.1;NP_001041693.1;NP_001041694.1;NP_081073.1;XP_006499958.1;XP_030107724.1	106562	.	3.075181
52846	NM_028043	Cnot11	CCR4-NOT transcription complex, subunit 11	protein_coding	NP_082319.1	106580	.	7.029983
52850	NM_001162965,NM_001254731,NM_001309526,NM_001309528,NM_172718,XM_006535122	Sgsm1	small G protein signaling modulator 1	protein_coding	NP_001156437.1;NP_001241660.1;NP_001296455.1;NP_001296457.1;NP_766306.2;XP_006535185.1	107320	.	0.751980
52855	NM_001113474,NM_001302675,NM_001302676,NM_001302677,NM_001302681,NM_001302682,NM_001302683,NM_178611,XM_006540175,XM_006540176,XM_006540179,XM_006540183,XM_017322357,XM_017322358,XM_017322359	Lair1	leukocyte-associated Ig-like receptor 1	protein_coding	NP_001106945.1;NP_001289604.1;NP_001289605.1;NP_001289606.1;NP_001289610.1;NP_001289611.1;NP_001289612.1;NP_848726.1;XP_006540238.1;XP_006540239.1;XP_006540242.1;XP_006540246.1;XP_017177846.1;XP_017177847.1;XP_017177848.1	105492	.	0.438739
52856	NM_001083328,NM_001083329,NM_028422,NM_181424,XM_006500672,XM_006500674,XM_006500675,XM_006500676,XM_006500677,XM_011239951,XM_017319124,XM_017319125,XM_017319127,XM_030251865,XM_030251866,XM_030251867,XM_030251868,XM_030251869,XM_030251870,XM_030251871,XM_030251872,XM_030251873,XM_030251874,XM_030251875,XR_001783146,XR_003953666,XR_003953667,XR_003953668,XR_003953669,XR_003953670,XR_003953671,XR_375400	Mtg2	mitochondrial ribosome associated GTPase 2	protein_coding	NP_001076797.1;NP_001076798.1;NP_082698.1;NP_852089.2;XP_006500735.1;XP_006500737.1;XP_006500738.1;XP_006500739.1;XP_006500740.1;XP_011238253.1;XP_017174613.1;XP_017174614.1;XP_017174616.1;XP_030107725.1;XP_030107726.1;XP_030107727.1;XP_030107728.1;XP_030107729.1;XP_030107730.1;XP_030107731.1;XP_030107732.1;XP_030107733.1;XP_030107734.1;XP_030107735.1	106565	.	5.017168
52857	NM_001360351,NM_027898,NR_153431,XM_006540184,XM_006540185,XM_006540186,XM_006540188,XM_030242768,XR_003946	Gramd1a	GRAM domain containing 1A	protein_coding	NP_001347280.1;NP_082174.3;XP_006540247.1;XP_006540248.1;XP_006540249.1;XP_006540251.1;XP_030098628.1	105490	.	24.266230
52858	NM_001347656,NM_026638,XM_006508022	Cdipt	CDP-diacylglycerol--inositol 3-phosphatidyltransferase (phosphatidylinositol synthase)	protein_coding	NP_001334585.1;NP_080914.1;XP_006508085.1	105491	.	19.336439
52864	NM_177472,XM_006522349,XM_006522350,XM_006522352,XM_006522353,XM_006522354,XM_006522355,XM_006522359,XM_017317068,XM_030249177,XM_030249178,XM_030249179,XM_030249181,XM_030249182,XM_030249183,XM_030249185,XM_030249186,XM_030249187,XR_003951798	Slx4	SLX4 structure-specific endonuclease subunit homolog (S. cerevisiae)	protein_coding	NP_803423.2;XP_006522412.1;XP_006522413.1;XP_006522415.1;XP_006522416.1;XP_006522417.1;XP_006522418.1;XP_006522422.1;XP_017172557.1;XP_030105037.1;XP_030105038.1;XP_030105039.1;XP_030105041.1;XP_030105042.1;XP_030105043.1;XP_030105045.1;XP_030105046.1;XP_030105047.1	106299	.	7.220020
52874	NM_177474,NR_131940,XM_006527183	Pum3	pumilio RNA-binding family member 3	protein_coding	NP_803425.1;XP_006527246.1	106253	.	10.241382
52882	NM_029879,XM_006517722	Rgs7bp	regulator of G-protein signalling 7 binding protein	protein_coding	NP_084155.2;XP_006517785.1	106334	.	2.598558
52892	NM_001040026,XM_006533721	Sco1	SCO1 cytochrome c oxidase assembly protein	protein_coding	NP_001035115.1;XP_006533784.1	106362	.	2.579937
52897	NM_001024931,NM_001039167,NM_001039168,NM_001285436,NM_001285437,NM_001285438,XM_006533722,XM_006533725,XM_006533726,XM_006533727,XM_017314661,XM_017314662,XM_017314663,XM_030246130,XM_030246131,XM_030246132,XM_030246133,XM_030246134,XM_030246135,XM_030246136,XM_030246137,XM_030246138,XM_030246139,XM_030246140,XM_030246141	Rbfox3	RNA binding protein, fox-1 homolog (C. elegans) 3	protein_coding	NP_001020102.2;NP_001034256.1;NP_001034257.1;NP_001272365.1;NP_001272366.1;NP_001272367.1;XP_006533785.1;XP_006533788.1;XP_006533789.1;XP_006533790.1;XP_017170150.1;XP_017170151.1;XP_017170152.1;XP_030101990.1;XP_030101991.1;XP_030101992.1;XP_030101993.1;XP_030101994.1;XP_030101995.1;XP_030101996.1;XP_030101997.1;XP_030101998.1;XP_030101999.1;XP_030102000.1;XP_030102001.1	106368	.	0.011397
52898	NM_173742	Rnasek	ribonuclease, RNase K	protein_coding	NP_776103.1	106369	.	55.040936
52906	NM_001177776,NM_001358561,NM_026203,XR_001779555,XR_001779556,XR_003948741,XR_380069	Ahi1	Abelson helper integration site 1	protein_coding	NP_001171247.1;NP_001345490.1;NP_080479.2	87971	.	3.245033
52915	NM_001005867,NM_028601,XM_006514748,XM_006514750,XM_011243732,XM_011243733,XM_011243734,XM_011243735,XM_017314664,XM_030246142,XM_030246143,XM_030246144,XM_030246145,XM_030246146,XM_030246147,XM_030246148,XM_030246149	Zmiz2	zinc finger, MIZ-type containing 2	protein_coding	NP_001005867.1;NP_082877.2;XP_006514811.1;XP_006514813.1;XP_011242034.1;XP_011242035.1;XP_011242036.1;XP_011242037.1;XP_017170153.1;XP_030102002.1;XP_030102003.1;XP_030102004.1;XP_030102005.1;XP_030102006.1;XP_030102007.1;XP_030102008.1;XP_030102009.1	106374	.	11.589052
53310	NM_001177778,NM_001177779,NM_001177780,NM_001290402,NM_016747,XM_006528123,XM_006528124,XM_006528127,XM_030251380,XR_001782754	Dlg3	discs large MAGUK scaffold protein 3	protein_coding	NP_001171249.1;NP_001171250.1;NP_001171251.1;NP_001277331.1;NP_058027.1;XP_006528186.1;XP_006528187.1;XP_006528190.1;XP_030107240.1	1888986	.	8.593319
53311	NM_001357515,NM_016749,XM_006529753,XR_003956240,XR_003956241	Mybph	myosin binding protein H	protein_coding	NP_001344444.1;NP_058029.2;XP_006529816.1	1858196	.	0.749196
53312	NM_001305264,NM_016736	Nub1	negative regulator of ubiquitin-like proteins 1	protein_coding	NP_001292193.1;NP_058016.2	1889001	.	14.882049
53313	NM_001163336,NM_001163337,NM_016745	Atp2a3	ATPase, Ca++ transporting, ubiquitous	protein_coding	NP_001156808.1;NP_001156809.1;NP_058025.2	1194503	.	0.746319
53314	NM_016767	Batf	basic leucine zipper transcription factor, ATF-like	protein_coding	NP_058047.1	1859147	.	4.290790
53317	NM_016784	Plrg1	pleiotropic regulator 1	protein_coding	NP_058064.2	1858197	.	18.520493
53318	NM_016798,XM_006509465	Pdlim3	PDZ and LIM domain 3	protein_coding	NP_058078.1;XP_006509528.1	1859274	.	10.063600
53319	NM_001276704,NM_016813	Nxf1	nuclear RNA export factor 1	protein_coding	NP_001263633.1;NP_058093.2	1858330	.	48.003851

ID	Transcripts	Symbol	Description	Type	Proteins	Gene ID		Value 1	Other columns
53320	NM_001159706,NM_016770,XM_006508023,XM_011241861,XR_003946512	Folh1	folate hydrolase 1	protein_coding	NP_001153178.1;NP_058050.3;XP_006508086.1;XP_011240163.1	1858193	.	0.000000	[illegible]
53321	NM_016782,XM_006533729,XM_030246150,XR_003949478	Cntnap1	contactin associated protein-like 1	protein_coding	NP_058062.2;XP_006533792.1;XP_030102010.1	1858201	.	1.037608	[illegible]
53322	NM_001130479,NM_001360375,XM_006508025,XM_006508026,XM_006508027,XM_006508028,XR_001785587,XR_003946513,XR_378260	Nucb2	nucleobindin 2	protein_coding	NP_001123951.1;NP_001347304.1;XP_006508088.1;XP_006508089.1;XP_006508090.1;XP_006508091.1	1858179	.	38.582522	[illegible]
53323	NM_001310618,NM_001310619,NM_016786,NR_144566	Ube2k	ubiquitin-conjugating enzyme E2K	protein_coding	NP_001297547.1;NP_001297548.1;NP_058066.2	1858216	.	11.633319	[illegible]
53324	NM_016789	Nptx2	neuronal pentraxin 2	protein_coding	NP_058069.1	1858209	.	0.018572	[illegible]
53325	NM_001110100,NM_001285981,NM_001285983,NM_016812,XM_011248442,XM_011248445,XM_017312899,XM_030243672,XM_030243673,XM_030243674,XM_030243675	Banp	BTG3 associated nuclear protein	protein_coding	NP_001103570.1;NP_001272910.1;NP_001272912.1;NP_058092.2;XP_011246744.1;XP_011246747.1;XP_017168388.1;XP_030099532.1;XP_030099533.1;XP_030099534.1;XP_030099535.1	1889023	.	5.207324	[illegible]
53328	NM_016783	Pgrmc1	progesterone receptor membrane component 1	protein_coding	NP_058063.2	1858305	.	60.040649	[illegible]
53330	NM_001347125,NM_001356526,NM_001356527,NM_016796	Vamp4	vesicle-associated membrane protein 4	protein_coding	NP_001334054.1;NP_001343455.1;NP_001343456.1;NP_058076.2	1858730	.	9.681329	[illegible]
53331	NM_001358563,NM_016797,XM_006512801	Stx7	syntaxin 7	protein_coding	NP_001345492.1;NP_058077.2;XP_006512864.1	1858210	.	20.916394	[illegible]
53332	NM_001313702,NM_001313703,NM_001313704,NM_001313706,NM_001313707,NM_016985,XM_006528136,XM_006528137,XM_011247629	Mtmr1	myotubularin related protein 1	protein_coding	NP_001300631.1;NP_001300632.1;NP_001300633.1;NP_001300635.1;NP_001300636.1;NP_058681.1;XP_006528199.1;XP_006528200.1;XP_011245931.1	1858271	.	9.823180	[illegible]
53333	NM_001109748,NM_016871,XM_030242769	Tomm40	translocase of outer mitochondrial membrane 40	protein_coding	NP_001103218.1;NP_058567.2;XP_030098629.1	1858259	.	14.080267	[illegible]
53334	NM_001356321,NM_016810	Gosr1	golgi SNAP receptor complex member 1	protein_coding	NP_001343250.1;NP_058090.2	1858260	.	10.781098	[illegible]
53356	NM_016876	Eif3g	eukaryotic translation initiation factor 3, subunit G	protein_coding	NP_058572.2	1858258	.	59.854275	[illegible]
53357	NM_001199023,NM_001199024,NM_001199025,NM_016915,XM_006521152,XM_006521153,XM_006521154,XM_006521155,XM_006521156,XM_006521157,XM_006521158,XM_006521159,XM_006521160,XM_017316698,XM_017316699,XM_030248657,XM_030248658,XR_001781552,XR_003951405,XR_003951406,XR_003951407,XR_003951408,XR_003951409	Pla2g6	phospholipase A2, group VI	protein_coding	NP_001185952.1;NP_001185953.1;NP_001185954.1;NP_058611.1;XP_006521215.1;XP_006521216.1;XP_006521217.1;XP_006521218.1;XP_006521219.1;XP_006521220.1;XP_006521221.1;XP_006521222.1;XP_006521223.1;XP_017172187.1;XP_017172188.1;XP_030104517.1;XP_030104518.1	1859152	.	4.649193	[illegible]
53374	NM_016803,XM_011243510,XM_011243512,XM_011243513,XM_017314030	Chst3	carbohydrate sulfotransferase 3	protein_coding	NP_058083.2;XP_011241812.1;XP_011241814.1;XP_011241815.1;XP_017169519.1	1858224	.	1.669846	[illegible]
53375	NM_016804	Mtx2	metaxin 2	protein_coding	NP_058084.3	1859652	.	17.037792	[illegible]
53376	NM_016808,NM_198091,NM_198092,XM_006510480,XM_006510486,XM_011242577,XM_030244446,XM_030244447,XM_030244448,XM_030244449,XM_030244450,XM_030244451	Usp2	ubiquitin specific peptidase 2	protein_coding	NP_058088.2;NP_932759.1;NP_932760.2;XP_006510543.1;XP_006510549.1;XP_011240879.1;XP_030100306.1;XP_030100307.1;XP_030100308.1;XP_030100309.1;XP_030100310.1;XP_030100311.1	1858178	.	3.292147	[illegible]
53378	NM_001098227,NM_016807,XM_006538084,XM_030253672	Sdcbp	syndecan binding protein	protein_coding	NP_001091697.1;NP_058087.2;XP_006538147.1;XP_030109532.1	1337026	.	56.333216	[illegible]
53379	NM_016806,NM_182650,NR_104468,XM_006506373,XM_006506374,XM_030255496,XM_030255497,XM_030255498,XM_030255499,XM_030255500,XR_003956228,XR_003956229,XR_003956230,XR_003956231,XR_003956232,XR_377482,XR_377483,XR_868864,XR_868865,XR_868868	Hnrnpa2b1	heterogeneous nuclear ribonucleoprotein A2/B1	protein_coding	NP_058086.2;NP_872591.1;XP_006506436.2;XP_006506437.2;XP_030111356.1;XP_030111357.1;XP_030111358.1;XP_030111359.1;XP_030111360.1	104819	.	301.438187	[illegible]
53380	NM_001164177,NM_016883	Psmd10	proteasome (prosome, macropain) 26S subunit, non-ATPase, 10	protein_coding	NP_001157649.1;NP_058579.2	1858898	.	6.655296	[illegible]
53381	NM_001313711,NM_016764,XM_011247844	Prdx4	peroxiredoxin 4	protein_coding	NP_001300640.1;NP_058044.1;XP_011246146.1	1859815	.	92.329257	[illegible]
53382	NM_016792	Txnl1	thioredoxin-like 1	protein_coding	NP_058072.2	1860078	.	24.194706	[illegible]
53404	NM_001364648,NM_016864	Atoh7	atonal bHLH transcription factor 7	protein_coding	NP_001351577.1;NP_058560.1	1355553	.	0.000000	[illegible]
53412	NM_016854	Ppp1r3c	protein phosphatase 1, regulatory subunit 3C	protein_coding	NP_058550.1	1858229	.	6.848728	[illegible]
53413	NM_001162872,NM_001347636,NM_001362838,NM_001362839,NM_016857,XM_006533733,XM_006533734,XM_006533736	Exoc7	exocyst complex component 7	protein_coding	NP_001156344.1;NP_001334565.1;NP_001349767.1;NP_001349768.1;NP_058553.2;XP_006533796.1;XP_006533797.1;XP_006533799.1	1859270	.	13.002134	[illegible]
53414	NM_016859	Bysl	bystin-like	protein_coding	NP_058555.3	1858419	.	4.815369	[illegible]
53415	NM_001146049,NM_001146050,NM_001146052,NM_001146053,NM_016865,XM_006540989,XM_030242770	Htatip2	HIV-1 Tat interactive protein 2	protein_coding	NP_001139521.1;NP_001139522.1;NP_001139524.1;NP_001139525.1;NP_058561.2;XP_006541052.1;XP_030098630.1	1859271	.	5.205485	[illegible]
53416	NM_016866,XM_011239693,XM_017319128,XM_017319129,XM_017319130,XM_030251876,XM_030251878,XR_001783147,XR_003953672	Stk39	serine/threonine kinase 39	protein_coding	NP_058562.1;XP_011237995.1;XP_017174617.1;XP_017174618.1;XP_017174619.1;XP_030107736.1;XP_030107738.1	1858416	.	12.463862	[illegible]
53417	NM_001162950,NM_016868,XM_011250628	Hif3a	hypoxia inducible factor 3, alpha subunit	protein_coding	NP_001156422.1;NP_058564.2;XP_011248930.1	1859778	.	2.412722	[illegible]
53418	NM_001253381,NM_017377,NR_045570,XM_006503230,XM_011240575,XM_017320316,XM_030253673,XR_003954984,XR_867887	B4galt2	UDP-Gal:betaGlcNAc beta 1,4- galactosyltransferase, polypeptide 2	protein_coding	NP_001240310.1;NP_059073.1;XP_006503293.1;XP_011238877.1;XP_017175805.1;XP_030109533.1	1858493	.	14.903453	[illegible]
53419	NM_001122756,NM_016869,XM_011240755,XM_030254695	Corin	corin, serine peptidase	protein_coding	NP_001116228.1;NP_058565.2;XP_011239057.1;XP_030110555.1	1349451	.	0.346479	[illegible]
53420	NM_001360421,NM_001360422	Syt5	synaptotagmin V	protein_coding	NP_001347350.1;NP_001347351.1	1926368	.	0.132604	[illegible]
53421	NM_016906	Sec61a1	Sec61 alpha 1 subunit (S. cerevisiae)	protein_coding	NP_058602.1	1858417	.	130.285019	[illegible]
53422	NM_001347634,NM_016875,XM_006533738,XM_006533739,XM_006533740,XM_006533742,XM_030246151	Ybx2	Y box protein 2	protein_coding	NP_001334563.1;NP_058571.2;XP_006533801.1;XP_006533802.1;XP_006533803.1;XP_006533805.1;XP_030102011.1	1096372	.	0.909322	[illegible]
53424	NM_016909	Tsnax	translin-associated factor X	protein_coding	NP_058605.1	1855672	.	34.165077	[illegible]
53598	NM_001159565,NM_016890	Dctn3	dynactin 3	protein_coding	NP_001153037.1;NP_058586.3	1859251	.	19.366392	[illegible]
53599	NM_016898	Cd164	CD164 antigen	protein_coding	NP_058594.1	1859568	.	91.402725	[illegible]
53600	NM_016897	Timm23	translocase of inner mitochondrial membrane 23	protein_coding	NP_058593.2	1858317	.	33.752445	[illegible]
53601	NM_017378	Pcdh12	protocadherin 12	protein_coding	NP_059074.2	1855700	.	3.254343	[illegible]
53602	NM_001361645,NM_016677,XM_006515157,XM_030246812,XM_030246813,XM_030246814,XM_030246815,XM_030246816,XM_030246817,XM_030246818,XM_030246819,XM_030246820	Hpcal1	hippocalcin-like 1	protein_coding	NP_001348574.1;NP_057886.1;XP_006515220.1;XP_030102672.1;XP_030102673.1;XP_030102674.1;XP_030102675.1;XP_030102676.1;XP_030102677.1;XP_030102678.1;XP_030102679.1;XP_030102680.	1855689	.	14.638241	[illegible]
53603	NM_021367,NR_033206	Tslp	thymic stromal lymphopoietin	protein_coding	NP_067342.1	1855696	.	0.536806	[illegible]
53604	NM_001185153,NM_015785,XM_006514755,XM_011243736,XM_030246152,XR_003949479,XR_380974	Zpbp	zona pellucida binding protein	protein_coding	NP_001172082.1;NP_056600.1;XP_006514818.1;XP_011242038.1;XP_030102012.1	1855701	.	0.235783	[illegible]
53605	NM_001358932,NM_015781,NR_152558,XM_030245178,XM_030245179,XR_001779558	Nap1l1	nucleosome assembly protein 1-like 1	protein_coding	NP_001345861.1;NP_056596.1;XP_030101038.1;XP_030101039.1	1855693	.	76.697974	[illegible]
53607	NM_001046637,NM_001285825,NM_015782	Snrpa	small nuclear ribonucleoprotein polypeptide A	protein_coding	NP_001040102.1;NP_001272754.1;NP_056597.3	1855690	.	34.432265	[illegible]
53608	NM_016693,XM_006539040,XM_006539041	Map3k6	mitogen-activated protein kinase kinase kinase 6	protein_coding	NP_057902.5;XP_006539103.1;XP_006539104.1	1855691	.	2.512870	[illegible]
53609	NM_016680,NR_125832,NR_125833,NR_125834,NR_125835,NR_125836,XM_006540191,XM_011250629,XM_017322361,XM_017322362,XM_017322363,XM_030242771,XM_030242772,XM_030242773,XM_030242774,XM_030242775,XM_030242776,XM_030242777,XM_030242778,XM_030242779,XR_003946514,XR_003946515,XR_003946516,XR_003946517	Clasrp	CLK4-associating serine/arginine rich protein	protein_coding	NP_057889.3;XP_006540254.1;XP_011248931.1;XP_017177850.1;XP_017177851.1;XP_017177852.1;XP_030098631.1;XP_030098632.1;XP_030098633.1;XP_030098634.1;XP_030098635.1;XP_030098636.1;XP_030098637.1;XP_030098638.1;XP_030098639.1	1855695	.	20.648095	[illegible]
53610	NM_001252518,NM_023144,XM_030251381,XM_030251382	Nono	non-POU-domain-containing, octamer binding protein	protein_coding	NP_001239447.1;NP_075633.2;XP_030107241.1;XP_030107242.1	1855692	.	88.518986	[illegible]
53611	NM_001293685,NM_001293686,NM_001360431,NM_001360432,NM_016862,XM_006527184,XM_006527185,XM_006527188,XM_006527191,XM_006527192,XM_006527195,XM_006527196,XM_006527198,XM_017318251,XM_030250990,XM_030250991,XM_030250992,XM_030250993	Vti1a	vesicle transport through interaction with t-SNAREs 1A	protein_coding	NP_001280614.1;NP_001280615.1;NP_001347360.1;NP_001347361.1;NP_058558.1;XP_006527247.1;XP_006527248.1;XP_006527251.1;XP_006527254.1;XP_006527255.1;XP_006527258.1;XP_006527259.1;XP_006527261.1;XP_017173740.1;XP_030106850.1;XP_030106851.1;XP_030106852.1;XP_030106853.1	1855699	.	4.626036	[illegible]
53612	NM_001364374,NM_016800,XM_011244143	Vti1b	vesicle transport through interaction with t-SNAREs 1B	protein_coding	NP_001351303.1;NP_058080.2;XP_011242445.1	1855688	.	20.659245	[illegible]
53614	NM_016678,XM_030253674	Reck	reversion-inducing-cysteine-rich protein with kazal motifs	protein_coding	NP_057887.2;XP_030109534.1	1855698	.	30.347425	[illegible]
53618	NM_001252614,NM_001252615,NM_001252616,NM_016893,NR_045554,XM_011244144,XM_011244145,XM_030246821,XM_030246822	Fut8	fucosyltransferase 8	protein_coding	NP_001239543.1;NP_001239544.1;NP_001239545.1;NP_058589.2;XP_011242446.1;XP_011242447.1;XP_030102681.1;XP_030102682.1	1858901	.	17.663838	[illegible]
53619	NM_001355426,NM_016916	Blcap	bladder cancer associated protein	protein_coding	NP_001342355.1;NP_058612.3	1858907	.	15.695081	[illegible]
53620	NM_001080742,NM_016872	Vamp5	vesicle-associated membrane protein 5	protein_coding	NP_001074211.1;NP_058568.1	1858622	.	2.816075	[illegible]
53621	NM_001164411,NM_001164412,NM_001164413,NM_001164414,NM_016877,XM_011241407,XM_011241408,XR_001785150,XR_001785151,XR_001785152,XR_001785153	Cnot4	CCR4-NOT transcription complex, subunit 4	protein_coding	NP_001157883.1;NP_001157884.1;NP_001157885.1;NP_001157886.1;NP_058573.3;XP_011239709.1;XP_011239710.1	1859026	.	5.139204	[illegible]
53623	NM_001281929,NM_001290451,NM_001358361,NM_016886,NR_104053,XM_017318545,XM_017318546,XM_017318547	Gria3	glutamate receptor, ionotropic, AMPA3 (alpha 3)	protein_coding	NP_001268858.1;NP_001277380.1;NP_001345290.1;NP_058582.3;XP_017174034.1;XP_017174035.1;XP_017174036.1	95810	.	4.862761	[illegible]
53624	NM_001193619,NM_016887	Cldn7	claudin 7	protein_coding	NP_001180548.1;NP_058583.1	1859285	.	0.108595	[illegible]
53625	NM_001169114,NM_016888	B3gnt2	UDP-GlcNAc:betaGal beta-1,3-N-acetylglucosaminyltransferase 2	protein_coding	NP_001162585.1;NP_058584.3	1889505	.	6.971175	[illegible]
53626	NM_016889	Insm1	insulinoma-associated 1	protein_coding	NP_058585.2	1859980	.	0.015667	[illegible]
53627	NM_001308474,NM_016913,NM_023638,NM_145907,NM_145908,NR_131893,XM_006527644,XM_006527645,XM_030251383,XM_030251384,XR_001782756,XR_001782757,XR_003953014,XR_878	Porcn	porcupine O-acyltransferase	protein_coding	NP_001295403.1;NP_058609.1;NP_076127.1;NP_665914.1;NP_665915.1;XP_006527707.1;XP_006527708.1;XP_030107243.1;XP_030107244.1	1890212	.	8.137532	[illegible]
53761	NM_001199044,NM_020027,XM_006524620,XM_006524621,XM_030249899,XM_030249900	Prrc2a	proline-rich coiled-coil 2A	protein_coding	NP_001185973.1;NP_064411.2;XP_006524683.1;XP_006524684.1;XP_030105759.1;XP_030105760.1	1915467	.	38.676763	[illegible]
53791	NM_016928,XM_017321697,XM_017321698,XM_017321699,XM_017321700,XM_017321701,XM_017321702,XM_017321703,XM_017321704	Tlr5	toll-like receptor 5	protein_coding	NP_058624.2;XP_017177186.1;XP_017177187.1;XP_017177188.1;XP_017177189.1;XP_017177190.1;XP_017177191.1;XP_017177192.1;XP_017177193.1	1858171	.	1.673062	[illegible]
53814	NM_016901	Oaz3	ornithine decarboxylase antizyme 3	protein_coding	NP_058597.2	1858170	.	0.250620	[illegible]
53817	NM_001252457,NM_019693	Ddx39b	DEAD (Asp-Glu-Ala-Asp) box polypeptide 39B	protein_coding	NP_001239386.1;NP_062667.1	99240	.	104.529932	[illegible]
53856	NM_016914,XM_006499901,XR_866323	Prg3	proteoglycan 3	protein_coding	NP_058610.1;XP_006499964.1	1858200	.	0.000000	[illegible]
53857	NM_017379	Tuba8	tubulin, alpha 8	protein_coding	NP_059075.1	1858225	.	0.119267	[illegible]
53858	NM_016924	Rwdd2b	RWD domain containing 2B	protein_coding	NP_058620.2	1858215	.	3.065558	[illegible]
53859	NM_016896	Map3k14	mitogen-activated protein kinase kinase kinase 14	protein_coding	NP_058592.1	1858204	.	2.281076	[illegible]
53860	NM_001113486,NM_001113487,NM_001113488,NM_017380,XM_006533744,XM_006533745,XM_006533746,XM_030246153,XM_030246154	Sept9	septin 9	protein_coding	NP_001106958.1;NP_001106959.1;NP_001106960.1;NP_059076.1;XP_006533807.1;XP_006533808.2;XP_006533809.1;XP_030102013.1;XP_030102014.1	1858222	.	31.835152	[illegible]
53861	NM_001355381,NM_001355386,NM_017381,XM_017319646	Zranb2	zinc finger, RAN-binding domain containing 2	protein_coding	NP_001342310.1;NP_001342315.1;NP_059077.1;XP_017175135.1	1858211	.	30.520978	[illegible]
53867	NM_001317388,NM_016919,XM_017313471,XR_003947951	Col5a3	collagen, type V, alpha 3	protein_coding	NP_001304317.1;NP_058615.1;XP_017168960.1	1858212	.	7.189359	[illegible]
53868	NM_016899	Rab25	RAB25, member RAS oncogene family	protein_coding	NP_058595.2	1858203	.	0.000000	[illegible]
53869	NM_017382	Rab11a	RAB11A, member RAS oncogene family	protein_coding	NP_059078.2	1858202	.	22.177692	[illegible]
53870	NM_017383,XM_006506381,XM_006506382,XM_006506383,XM_006506384,XM_011241409,XM_017321678,XM_017321679,XR_003956233	Cntn6	contactin 6	protein_coding	NP_059079.2;XP_006506444.1;XP_006506445.1;XP_006506446.1;XP_006506447.1;XP_011239711.1;XP_017177167.1;XP_017177168.1	1858223	.	0.076466	[illegible]
53871	NM_001163004,NM_016927,XM_006526097,XM_006526101,XM_006526104,XM_017317944,XM_017317946,XM_017317947,XM_017317948,XM_017317949,XM_017317950,XM_017317951	Pkd2l2	polycystic kidney disease 2-like 2	protein_coding	NP_001156476.1;NP_058623.2;XP_006526160.1;XP_006526164.1;XP_006526167.1;XP_017173433.1;XP_017173435.1;XP_017173436.1;XP_017173437.1;XP_017173438.1;XP_017173439.1;XP_017173440.	1858231	.	0.167007	[illegible]
53872	NM_001111289,NM_001111290,NM_001111291,NM_001111292,NM_016739,XM_030251880	Caprin1	cell cycle associated protein 1	protein_coding	NP_001104759.1;NP_001104760.1;NP_001104761.1;NP_001104762.1;NP_058019.2;XP_030107740.1	1858234	.	45.507805	[illegible]
53881	NM_017391	Slc5a3	solute carrier family 5 (inositol transporters), member 3	protein_coding	NP_059087.2	1858226	.	4.302922	[illegible]
53883	NM_001004177,NM_017392,XM_006501691,XM_006501692,XM_017319649	Celsr2	cadherin, EGF LAG seven-pass G-type receptor 2	protein_coding	NP_001004177.2;NP_059088.2;XP_006501754.1;XP_006501755.1;XP_017175138.1	1858235	.	0.258360	[illegible]
53885	NM_001291012,NM_001291013,NM_001355429,NM_001369236,NM_016902,XM_011239695,XM_011239696,XM_011239697,XM_030251881	Nphp1	nephronophthisis 1 (juvenile) homolog (human)	protein_coding	NP_001277941.1;NP_001277942.1;NP_001342358.1;NP_001356165.1;NP_058598.1;XP_011237997.1;XP_011237998.1;XP_011237999.1;XP_030107741.1	1858233	.	10.547730	[illegible]
53886	NM_001276315,NM_016912,NM_177270,XM_011249537,XM_011249538,XM_011249539,XM_017320987,XM_017320988,XM_017320989,XM_017320990,XM_030254698	Cdkl2	cyclin-dependent kinase-like 2 (CDC2-related kinase)	protein_coding	NP_001263244.1;NP_058608.1;NP_796244.2;XP_011247839.1;XP_011247840.1;XP_011247841.1;XP_017176476.1;XP_017176477.1;XP_017176478.1;XP_017176479.1;XP_030110558.1	1858227	.	0.497736	[illegible]
53890	NM_001356621,NM_001356623,NM_016926,NR_151532,XM_006530406,XM_030254699	Sart3	squamous cell carcinoma antigen recognized by T cells 3	protein_coding	NP_001343550.1;NP_001343552.1;NP_058622.1;XP_006530469.1;XP_030110559.1	1858230	.	8.497437	[illegible]
53892	NM_016910	Ppm1d	protein phosphatase 1D magnesium-dependent, delta isoform	protein_coding	NP_058606.3	1858214	.	5.785450	[illegible]
53893	NM_016918,XM_006497511	Nudt5	nudix (nucleoside diphosphate linked moiety X)-type motif 5	protein_coding	NP_058614.1;XP_006497574.1	1858232	.	7.451340	[illegible]
53895	NM_017393	Clpp	caseinolytic mitochondrial matrix peptidase proteolytic subunit	protein_coding	NP_059089.1	1858213	.	16.940735	[illegible]
53896	NM_017394,XM_006540197,XM_030242780,XM_030242781,XM_030242782	Slc7a10	solute carrier family 7 (cationic amino acid transporter, y+ system), member 10	protein_coding	NP_059090.3;XP_006540260.1;XP_030098640.1;XP_030098641.1;XP_030098642.1	1858261	.	0.495074	[illegible]
53897	NM_001177691,NM_001177703,NM_016922,XM_006514756,XM_006514757,XM_030246155,XM_030246156,XM_030246157	Gal3st1	galactose-3-O-sulfotransferase 1	protein_coding	NP_001171162.1;NP_001171174.1;NP_058618.2;XP_006514819.2;XP_006514820.1;XP_030102015.1;XP_030102016.1;XP_030102017.1	1858277	.	0.242532	[illegible]
53898	gene-Naip3-ps1	Naip3-ps1	NLR family, apoptosis inhibitory protein 3, pseudogene 1	pseudogene	.	1858253	.	0.049222	[illegible]
53901	NM_001286653,NM_001286654,NM_001357742,NM_030598,NM_207649	Rcan2	regulator of calcineurin 2	protein_coding	NP_001273582.1;NP_001273583.1;NP_001344671.1;NP_085101.1;NP_997532.1	1858219	.	3.062001	[illegible]
53902	NM_022980,XM_017320317	Rcan3	regulator of calcineurin 3	protein_coding	NP_075356.1;XP_017175806.1	1858220	.	4.705476	[illegible]
53906	NM_001145644	Phgr1	proline/histidine/glycine-rich 1	protein_coding	NP_001139116.1	1858382	.	0.083859	[illegible]
53945	NM_016917,XM_006496137,XM_017321709	Slc40a1	solute carrier family 40 (iron-regulated transporter), member 1	protein_coding	NP_058613.2;XP_006496200.1;XP_017177198.1	1315204	.	16.803971	[illegible]
53951	NM_181649,XM_006524623,XM_017317577,XM_030249901	Gpatch11	G patch domain containing 11	protein_coding	NP_857632.4;XP_006524686.1;XP_017173066.1;XP_030105761.1	1858435	.	3.089399	[illegible]
53970	NM_001355705,NM_001355706,NM_001355707,NM_017395,XM_006501697,XM_006501698,XM_006501699,XM_011240164,XM_017319651,XM_030252684,XM_030252685,XM_030252686,XM_030252687	Rfx5	regulatory factor X, 5 (influences HLA class II expression)	protein_coding	NP_001342634.1;NP_001342635.1;NP_001342636.1;NP_059091.2;XP_006501760.1;XP_006501761.1;XP_006501762.1;XP_011238466.1;XP_017175140.1;XP_030108544.1;XP_030108545.1;XP_030108546.1;XP_030108547.1	1858421	.	7.083679	[illegible]
53972	NM_001111314,NM_019867	Ngef	neuronal guanine nucleotide exchange factor	protein_coding	NP_001104784.1;NP_063920.2	1858414	.	4.163556	[illegible]
53975	NM_017397,XM_006501701	Ddx20	DEAD (Asp-Glu-Ala-Asp) box polypeptide 20	protein_coding	NP_059093.3;XP_006501764.1	1858415	.	7.563111	[illegible]
53978	NM_020028	Lpar2	lysophosphatidic acid receptor 2	protein_coding	NP_064412.2	1858422	.	0.564245	[illegible]
54003	NM_001289653,NM_001356950,NM_001356951,XM_006521163,XM_006521164	Nell2	NEL-like 2	protein_coding	NP_001276582.1;NP_001343879.1;NP_001343880.1;XP_006521226.1;XP_006521227.1	1858510	.	1.023091	[illegible]
54004	NM_172493,XM_006528581,XM_006528582,XM_030251385	Diaph2	diaphanous related formin 2	protein_coding	NP_766081.1;XP_006528644.1;XP_006528645.1;XP_030107245.1	1858500	.	2.666395	[illegible]
54006	NM_001282072,NM_001282073,NM_001282076,NM_016874,XM_006536204,XM_006536205,XM_006536206,XM_006536207,XM_006536209,XM_006536210,XM_006536211,XM_006536212,XM_006536213,XM_017322368,XM_017322370,XM_030242783,XM_030242784,XM_030242785,XM_030242786,XM_030242787,XM_030242788,XM_030242789,XM_030242790,XM_030242791,XR_389957	Deaf1	DEAF1, transcription factor	protein_coding	NP_001269001.1;NP_001269002.1;NP_001269005.1;NP_058570.1;XP_006536267.1;XP_006536268.1;XP_006536269.1;XP_006536270.1;XP_006536272.1;XP_006536273.1;XP_006536274.1;XP_006536275.1;XP_006536276.1;XP_017177857.1;XP_017177859.1;XP_030098643.1;XP_030098644.1;XP_030098645.1;XP_030098646.1;XP_030098647.1;XP_030098648.1;XP_030098649.1;XP_030098650.1;XP_0300	1858496	.	21.936479	[illegible]
54120	NM_016867,XM_011240165	Gipc2	GIPC PDZ domain containing family, member 2	protein_coding	NP_058563.1;XP_011238467.1	1889209	.	0.000000	[illegible]
54122	NM_001040695,XM_006540993,XM_006540994,XR_001785588,XR_003946518,XR_391351,XR_391352	Uevld	UEV and lactate/malate dehyrogenase domains	protein_coding	NP_001035785.1;XP_006541056.1;XP_006541057.1	1860490	.	4.767925	[illegible]
54123	NM_001252600,NM_001252601,NM_016850,XM_006536214	Irf7	interferon regulatory factor 7	protein_coding	NP_001239529.1;NP_001239530.1;NP_058546.1;XP_006536277.1	1859212	.	2.053048	[illegible]
54124	NM_016904	Cks1b	CDC28 protein kinase 1b	protein_coding	NP_058600.1	1889208	.	34.589531	[illegible]
54125	NM_017401,XM_006514758,XM_006514759,XM_030246158	Polm	polymerase (DNA directed), mu	protein_coding	NP_059097.2;XP_006514821.1;XP_006514822.1;XP_030102018.1	1860191	.	6.847918	[illegible]
54126	NM_001113517,NM_001113518,NM_017402,XM_006508841,XM_006508843,XM_006508844,XM_006508845,XM_017312901,XM_030243676,XM_030243677	Arhgef7	Rho guanine nucleotide exchange factor (GEF7)	protein_coding	NP_001106989.1;NP_001106990.1;NP_059098.2;XP_006508904.3;XP_006508906.3;XP_006508907.3;XP_006508908.1;XP_017168390.2;XP_030099536.1;XP_030099537.1	1860493	.	11.457480	[illegible]
54127	NM_001355384,NM_016844	Rps28	ribosomal protein S28	protein_coding	NP_001342313.1;NP_058540.1	1859516	.	485.176659	[illegible]
54128	NM_001362485,NM_016881,XM_006522360	Pmm2	phosphomannomutase 2	protein_coding	NP_001349414.1;NP_058577.1;XP_006522423.1	1859214	.	16.105584	[illegible]
54130	NM_001365070,NM_016860	Actr1a	ARP1 actin-related protein 1A, centractin alpha	protein_coding	NP_001351999.1;NP_058556.1	1858964	.	42.561955	[illegible]
54131	NM_016849,NR_045611,XM_006540995,XM_006540996	Irf3	interferon regulatory factor 3	protein_coding	NP_058545.1;XP_006541058.1;XP_006541059.1	1859179	.	22.796568	[illegible]
54132	NM_016861	Pdlim1	PDZ and LIM domain 1 (elfin)	protein_coding	NP_058557.2	1860611	.	12.237071	[illegible]
54135	NM_001164184,NM_001164185,NM_017405,XM_011250631,XM_011250632,XM_011250633	Lsr	lipolysis stimulated lipoprotein receptor	protein_coding	NP_001157656.1;NP_001157657.1;NP_059101.1;XP_011248933.1;XP_011248934.1;XP_011248935.1	1927471	.	0.703737	[illegible]
54137	NM_001127340,NM_016845	Acrbp	proacrosin binding protein	protein_coding	NP_001120812.1;NP_058541.2	1859515	.	4.278266	[illegible]
54138	NM_016843	Atxn10	ataxin 10	protein_coding	NP_058539.2	1859293	.	42.556080	[illegible]
54139	NM_016851,XM_006497262,XM_006497263	Irf6	interferon regulatory factor 6	protein_coding	NP_058547.2;XP_006497325.1;XP_006497326.1	1859211	.	0.307038	[illegible]
54140	NM_016847	Avpr1a	arginine vasopressin receptor 1A	protein_coding	NP_058543.2	1859216	.	0.372845	[illegible]
54141	NM_017407,NR_156178,XM_006533747,XM_006533748,XR_001780026,XR_001780027,XR_003949480	Spag5	sperm associated antigen 5	protein_coding	NP_059103.1;XP_006533810.1;XP_006533811.1	1927470	.	6.294292	[illegible]
54151	NM_001276321,NM_001276322,NM_001276333,NM_001276334,NM_001276335,NM_001357882,NM_019396,NM_180962,XM_006521167,XM_006521168,XM_006521169,XM_011245694,XM_030248659,XM_030248660	Cyhr1	cysteine and histidine rich 1	protein_coding	NP_001263250.1;NP_001263251.1;NP_001263262.1;NP_001263263.1;NP_001263264.1;NP_001344811.1;NP_062269.1;NP_851293.1;XP_006521230.1;XP_006521231.1;XP_006521232.1;XP_011243996.1;XP_030104519.1;XP_030104520.1	1859320	.	23.372400	[illegible]
54152	NM_017470,XM_006521170	Dnal4	dynein, axonemal, light chain 4	protein_coding	NP_059498.2;XP_006521233.1	1859217	.	14.709511	[illegible]
54153	NM_001039103,NM_133914,XM_006504443,XM_030254701,XM_030254702,XR_001784726,XR_377106	Rasa4	RAS p21 protein activator 4	protein_coding	NP_001034192.1;NP_598675.2;XP_006504506.1;XP_030110561.1;XP_030110562.1	1858600	.	9.499981	[illegible]
54156	NM_019397	Egfl6	EGF-like-domain, multiple 6	protein_coding	NP_062270.1	1858599	.	11.715861	[illegible]
54159	NM_019398	Rnase2b	ribonuclease, RNase A family, 2B (liver, eosinophil-derived neurotoxin)	protein_coding	NP_062271.2	1858598	.	0.000000	[illegible]
54160	NM_017478,NR_149738,XR_001785154	Copg2	coatomer protein complex, subunit gamma 2	protein_coding	NP_059506.1	1858683	.	11.548420	[illegible]
54161	NM_017477,NM_201244,XR_001785155	Copg1	coatomer protein complex, subunit gamma 1	protein_coding	NP_059505.1;NP_957696.1	1858696	.	68.209155	[illegible]
54167	NM_017480,XM_006496138,XM_006496139,XM_006496140	Icos	inducible T cell co-stimulator	protein_coding	NP_059508.2;XP_006496201.1;XP_006496202.1;XP_006496203.1	1858745	.	0.088636	[illegible]
54169	NM_001205241,NM_017479,XM_006519263,XM_006519264,XM_006519265,XM_006519268,XM_006519269,XM_017316082,XM_017316083,XM_017316084,XM_030247887,XM_030247888	Kat6b	K(lysine) acetyltransferase 6B	protein_coding	NP_001192170.1;NP_059507.2;XP_006519326.1;XP_006519327.1;XP_006519328.1;XP_006519331.1;XP_006519332.1;XP_017171571.1;XP_017171572.1;XP_017171573.1;XP_030103747.1;XP_030103748.	1858746	.	7.006712	[illegible]
54170	NM_017475	Rragc	Ras-related GTP binding C	protein_coding	NP_059503.2	1858751	.	24.390051	[illegible]
54188	NM_001291248,NM_001291249,NM_178576,XM_006504845	Cpsf4	cleavage and polyadenylation specific factor 4	protein_coding	NP_001278177.1;NP_001278178.1;NP_848671.1;XP_006504908.1	1861602	.	12.038033	[illegible]
54189	NM_001291141,NM_001291142,NM_001291143,NM_019400,XM_006533750	Rabep1	rabaptin, RAB GTPase binding effector protein 1	protein_coding	NP_001278070.1;NP_001278071.1;NP_001278072.1;NP_062273.2;XP_006533813.1	1860236	.	9.684102	[illegible]
54194	NM_017476,XM_011246534,XM_030249902,XM_030249903,XM_030249904,XM_030249905,XR_003952151,XR_003952152,XR_003952153,XR_003952154,XR_003952155,XR_003952156,XR_003952157,XR_003952158,XR_003952159,XR_003952160,XR_003952161,XR_003952162,XR_003952163,XR_003952164,XR_003952165,XR_003952166,XR_003952167	Akap8l	A kinase (PRKA) anchor protein 8-like	protein_coding	NP_059504.2;XP_011244836.1;XP_030105762.1;XP_030105763.1;XP_030105764.1;XP_030105765.1	1860606	.	32.667052	[illegible]
54195	NM_001161796,NM_017469	Gucy1b1	guanylate cyclase 1, soluble, beta 1	protein_coding	NP_001155268.1;NP_059497.1	1860604	.	2.558065	[illegible]
54196	NM_019402	Pabpn1	poly(A) binding protein, nuclear 1	protein_coding	NP_062275.1	1859158	.	81.657639	[illegible]
54197	NM_019403	Rnf5	ring finger protein 5	protein_coding	NP_062276.1	1860076	.	17.066153	[illegible]
54198	NM_017472	Snx3	sorting nexin 3	protein_coding	NP_059500.2	1860188	.	57.744186	[illegible]
54199	NM_001302376,NM_001302377,NM_017466	Ccrl2	chemokine (C-C motif) receptor-like 2	protein_coding	NP_001289305.1;NP_001289306.1;NP_059494.2	1920904	.	0.288608	[illegible]
54200	NM_001360784,NM_017465,XM_006540998,XM_006540999,XM_030242792,XM_030242793	Sult2b1	sulfotransferase family, cytosolic, 2B, member 1	protein_coding	NP_001347713.1;NP_059493.2;XP_006541061.1;XP_006541062.1;XP_030098652.1;XP_030098653.1	1926342	.	0.000000	[illegible]
54201	NM_017467,XM_006504718	Zfp316	zinc finger protein 316	protein_coding	NP_059495.3;XP_006504781.1	1860402	.	1.817913	[illegible]
54204	NM_017461,XM_006508029,XM_006508030,XM_006508031,XM_011241862,XM_030242795,XM_030242796,XM_030242797,XR_003946519,XR_003946520,XR_003946521,XR_003946522,XR_00394	Sept1	septin 1	protein_coding	NP_059489.2;XP_006508092.1;XP_006508093.1;XP_006508094.1;XP_011240164.1;XP_030098655.1;XP_030098656.1;XP_030098657.1	1858916	.	1.140721	[illegible]
54208	NM_019419	Arl6ip1	ADP-ribosylation factor-like 6 interacting protein 1	protein_coding	NP_062292.1	1858943	.	31.188242	[illegible]
54214	NM_018748,XM_006512187,XM_006512189,XM_017313473,XM_017313474,XM_017313475,XM_017313476,XM_017313477,XM_017313478,XM_017313479,XM_017313480,XM_017313481,XM_017313482,XM_017313483,XM_017313484,XM_017313485,XR_001778939,XR_001778940,XR_003947952,XR_003947953,XR_379873,XR_379875,XR_379876,XR_871210	Golga4	golgi autoantigen, golgin subfamily a, 4	protein_coding	NP_061218.2;XP_006512250.1;XP_006512252.1;XP_017168962.1;XP_017168963.1;XP_017168964.1;XP_017168965.1;XP_017168966.1;XP_017168967.1;XP_017168968.1;XP_017168969.1;XP_017168970.1;XP_017168971.1;XP_017168972.1;XP_017168973.1;XP_017168974.1	1859646	.	15.977617	[illegible]
54215	NM_001163496,NM_001163497,NM_018767	Cd160	CD160 antigen	protein_coding	NP_001156968.1;NP_001156969.1;NP_061237.3	1860383	.	0.000000	[illegible]
54216	NM_001122758,NM_001310606,NM_001310608,NM_018764,XM_006504019,XM_006504020,XM_006504022,XM_006504023,XM_006504024,XM_006504025,XM_006504028,XM_006504029,XM_006504030,XM_017320993	Pcdh7	protocadherin 7	protein_coding	NP_001116230.1;NP_001297535.1;NP_001297537.1;NP_061234.2;XP_006504082.1;XP_006504083.1;XP_006504085.1;XP_006504086.1;XP_006504087.1;XP_006504088.1;XP_006504091.1;XP_006504092.1;XP_006504093.1;XP_017176482.1	1860487	.	8.765556	[illegible]
54217	NM_018730,XM_030249907	Rpl36	ribosomal protein L36	protein_coding	NP_061200.2;XP_030105767.1	1860603	.	484.153988	[illegible]
54218	NM_019420	B3galt4	UDP-Gal:betaGlcNAc beta 1,3-galactosyltransferase, polypeptide 4	protein_coding	NP_062293.1	1859517	.	2.156845	[illegible]
54219	NM_001305169,NM_019421	Cd320	CD320 antigen	protein_coding	NP_001292098.1;NP_062294.3	1860083	.	3.998632	[illegible]
54324	NM_133674,XM_006506387	Arhgef5	Rho guanine nucleotide exchange factor (GEF) 5	protein_coding	NP_598435.1;XP_006506450.1	1858952	.	4.736174	[illegible]
54325	NM_001039175,NM_001039176,NM_019422,XM_006503233	Elovl1	elongation of very long chain fatty acids (FEN1/Elo2, SUR4/Elo3, yeast)-like 1	protein_coding	NP_001034264.1;NP_001034265.1;NP_062295.1;XP_006503296.1	1858959	.	26.392065	[illegible]
54326	NM_001311121,NM_019423	Elovl2	elongation of very long chain fatty acids (FEN1/Elo2, SUR4/Elo3, yeast)-like 2	protein_coding	NP_001298050.1;NP_062296.1	1858960	.	0.218543	[illegible]
54338	NM_001355430,NM_001355431,NM_018824,XM_006499906,XM_006499907,XM_011239698,XM_030251882,XM_030251883	Slc23a2	solute carrier family 23 (nucleobase transporters), member 2	protein_coding	NP_001342359.1;NP_001342360.1;NP_061294.2;XP_006499969.1;XP_006499970.1;XP_011238000.1;XP_030107742.1;XP_030107743.1	1859682	.	16.507915	[illegible]
54339	gene-Tes3-ps	Tes3-ps	testis derived transcript 3, pseudogene	pseudogene	.	3582925	.	0.677013	[illegible]
54342	NM_019425,XM_006519274,XM_006519275,XM_006519276	Gnpnat1	glucosamine-phosphate N-acetyltransferase 1	protein_coding	NP_062298.1;XP_006519337.2;XP_006519338.1;XP_006519339.1	1858963	.	8.520380	[illegible]

54343	NM_019426,XM_006506388,XM_006506389,XM_006506390,XM_006506391,XM_006506392,XM_017321681,XM_030255502,XM_030255503	Atf7ip	activating transcription factor 7 interacting protein	protein_coding	[illegible]
54351	NM_001253700,NM_018740,XM_006533752,XM_030246159	Elp5	elongator acetyltransferase complex subunit 5	protein_coding	[illegible]
54352	NM_018826	Irx5	Iroquois homeobox 5	protein_coding	[illegible]
54353	NM_018773	Skap2	src family associated phosphoprotein 2	protein_coding	[illegible]
54354	NM_001311094,NM_001313731,NM_018750	Rassf5	Ras association (RalGDS/AF-6) domain family member 5	protein_coding	[illegible]
54357	NM_001355492,NM_019427,XM_006538087,XM_006538089,XM_011250068,XM_011250069,XM_030253675	Epb41l4b	erythrocyte membrane protein band 4.1 like 4b	protein_coding	[illegible]
54364	NM_019428,XR_386495	Rpp30	ribonuclease P/MRP 30 subunit	protein_coding	[illegible]
54366	NM_018761,XM_017320319	Ctnnal1	catenin (cadherin associated protein), alpha-like 1	protein_coding	[illegible]
54367	NM_018759,NR_030777,XM_006535128,XM_006535131,XM_030254703	Zfp326	zinc finger protein 326	protein_coding	[illegible]
54368	NM_018762,XM_006506393,XM_006506394,XM_011241410,XM_017321682	Gp9	glycoprotein 9 (platelet)	protein_coding	[illegible]
54369	NM_018757,XM_006512197,XM_006512198,XM_006512199,XM_006512200	Nme6	NME/NM23 nucleoside diphosphate kinase 6	protein_coding	[illegible]
54371	NM_018763	Chst2	carbohydrate sulfotransferase 2	protein_coding	[illegible]
54373	NM_019429	Prss16	protease, serine 16 (thymus)	protein_coding	[illegible]
54375	NM_001102458,NM_001301688,NM_018745,NR_125913,XM_006520114,XM_006520117,XM_030248661	Azin1	antizyme inhibitor 1	protein_coding	[illegible]
54376	NM_019430	Cacng3	calcium channel, voltage-dependent, gamma subunit 3	protein_coding	[illegible]
54377	NM_019431	Cacng4	calcium channel, voltage-dependent, gamma subunit 4	protein_coding	[illegible]
54378	NM_133183,XM_006540200	Cacng6	calcium channel, voltage-dependent, gamma subunit 6	protein_coding	[illegible]
54380	NM_018817,XM_006496141,XM_006496142,XM_006496143,XM_006496144,XM_011238557,XM_030255544	Smarcal1	SWI/SNF related matrix associated, actin dependent regulator of chromatin, subfamily a-like 1	protein_coding	[illegible]
54381	NM_018755,NM_176073	Cpq	carboxypeptidase Q	protein_coding	[illegible]
54383	NM_001195083,NM_001195130,NM_018774,XM_006503234,XM_006503235,XM_006503236,XM_006503237,XM_006503238,XM_011240576,XM_017320320,XR_376334	Phc2	polyhomeotic 2	protein_coding	[illegible]
54384	NM_001040699,XM_006509466,XM_006509467,XM_006509469,XM_017312902,XM_030243678	Mtmr7	myotubularin related protein 7	protein_coding	[illegible]
54387	NM_019434,XM_006513894	Mcm3ap	minichromosome maintenance complex component 3 associated protein	protein_coding	[illegible]
54388	NM_018792	Hils1	histone H1-like protein in spermatids 1	protein_coding	[illegible]
54391	NM_019437	Rfk	riboflavin kinase	protein_coding	[illegible]
54392	NM_019438,XM_011240756	Ncapg	non-SMC condensin I complex, subunit G	protein_coding	[illegible]
54393	NM_019439,XM_006524632,XM_030249908,XM_030249909,XR_001782091	Gabbr1	gamma-aminobutyric acid (GABA) B receptor, 1	protein_coding	[illegible]
54394	NM_001277106,NM_018776,XR_001780029	Crlf3	cytokine receptor-like factor 3	protein_coding	[illegible]
54396	NM_019440	Irgm2	immunity-related GTPase family M member 2	protein_coding	[illegible]
54397	NM_001302393,NM_001302394,NM_001302395,NM_001302396,NM_019441,XM_006524634,XM_006524638	Ppt2	palmitoyl-protein thioesterase 2	protein_coding	[illegible]
54399	NM_018742,XM_030242798	Bet1l	Bet1 golgi vesicular membrane trafficking protein like	protein_coding	[illegible]
54401	NM_018753,XM_006499909	Ywhab	tyrosine 3-monooxygenase/tryptophan 5-monooxygenase activation protein, beta polypeptide	protein_coding	[illegible]
54402	NM_019442	Stk19	serine/threonine kinase 19	protein_coding	[illegible]
54403	NM_001136260,NM_001197147,NM_001359211,NM_001359212,NM_001359213,NM_018760,XM_006535136,XM_011249542,XM_017320995,XM_030254704,XM_030254705	Slc4a4	solute carrier family 4 (anion exchanger), member 4	protein_coding	[illegible]
54405	NM_019443	Ndufa1	NADH:ubiquinone oxidoreductase subunit A1	protein_coding	[illegible]
54409	NM_019444	Ramp2	receptor (calcitonin) activity modifying protein 2	protein_coding	[illegible]
54411	NM_001358375,NM_001358380,NM_018794	Atp6ap1	ATPase, H+ transporting, lysosomal accessory protein 1	protein_coding	[illegible]
54418	NM_019445,XM_030255545	Fmn2	formin 2	protein_coding	[illegible]
54420	NM_018778	Cldn8	claudin 8	protein_coding	[illegible]
54422	NM_001164186,NM_001355434,NM_001355435,NM_019446,XM_006498182,XM_011239138,XM_030251884,XM_030251885,XM_030251886	Barhl1	BarH like homeobox 1	protein_coding	[illegible]
54426	NM_019447,XM_006504031,XM_006504032,XM_006504033,XM_030254706,XR_003955676,XR_376817,XR_376818,XR_376819	Hgfac	hepatocyte growth factor activator	protein_coding	[illegible]
54427	NM_001081695,NM_001284197,NM_001284198,NM_001284199,NM_001284200,NM_019448	Dnmt3l	DNA (cytosine-5-)-methyltransferase 3-like	protein_coding	[illegible]
54445	NM_001161428,NM_019449,XM_006531798	Unc93b1	unc-93 homolog B1, TLR signaling regulator	protein_coding	[illegible]
54446	NM_001286260,NM_018823,NM_133957,XM_006531191,XM_006531192,XM_006531193,XM_006531194,XM_006531195,XM_006531196,XM_006531197,XM_017312904,XM_017312905,XM_030243679,XM_030243680,XM_030243681,XM_030243682	Nfat5	nuclear factor of activated T cells 5	protein_coding	[illegible]
54447	NM_018830	Asah2	N-acylsphingosine amidohydrolase 2	protein_coding	[illegible]
54450	NM_001146087,NM_001146088,NM_019451	Il1f5	interleukin 1 family, member 5 (delta)	protein_coding	[illegible]
54451	NM_001364372,NM_001364373,NM_018813,XM_006515158,XM_017315136,XR_381202	Cpsf3	cleavage and polyadenylation specificity factor 3	protein_coding	[illegible]
54473	NM_001347562,NM_023764	Tollip	toll interacting protein	protein_coding	[illegible]
54483	NM_001161790,NM_001161791,NM_019453,XM_006522361,XM_030249188,XM_030249189	Mefv	Mediterranean fever	protein_coding	[illegible]
54484	NM_001311109,NM_018810,XM_011241411,XM_011241412,XM_011241413,XM_017321683	Mkrn1	makorin, ring finger protein, 1	protein_coding	[illegible]
54485	NM_019454,XM_030251887	Dll4	delta like canonical Notch ligand 4	protein_coding	[illegible]
54486	NM_019455,XM_006506398	Hpgds	hematopoietic prostaglandin D synthase	protein_coding	[illegible]
54519	NM_019456,XM_006498186,XM_006498187,XM_011239139,XM_030251888	Apbb1ip	amyloid beta (A4) precursor protein-binding, family B, member 1 interacting protein	protein_coding	[illegible]
54524	NM_001276676,NM_001276677,NM_001276679,NM_001276680,NM_001276681,NM_018800,XM_006501704,XM_006501705,XM_006501706,XM_006501707,XM_006501708,XM_030252690,XR_003954360,XR_003954361,XR_003954362,XR_375546,XR_375547	Syt6	synaptotagmin VI	protein_coding	[illegible]
54525	NM_018801,NM_173067,NM_173068,XM_006527204,XM_006527208,XM_017318252,XM_017318253,XM_017318255,XM_017318257,XM_030250994,XM_030250995,XM_030250996,XM_030250997	Syt7	synaptotagmin VII	protein_coding	[illegible]
54526	NM_018803,XM_006521175,XM_006521176,XM_011245696,XM_017316706	Syt10	synaptotagmin X	protein_coding	[illegible]
54561	NM_138742	Nap1l3	nucleosome assembly protein 1-like 3	protein_coding	[illegible]
54563	NM_018815,XM_006506400	Nup210	nucleoporin 210	protein_coding	[illegible]
54598	NM_018782,XM_006499918	Calcrl	calcitonin receptor-like	protein_coding	[illegible]
54601	NM_018789,XM_006528139,XM_030251386	Foxo4	forkhead box O4	protein_coding	[illegible]
54604	NM_018814,XM_006516089,XM_006516090,XM_006516091,XM_006516092,XM_006516093,XM_006516094,XM_006516095,XM_017315138,XM_030246823	Pcnx	pecanex homolog	protein_coding	[illegible]
54607	NM_018821,XM_006526513,XM_011247089,XM_011247090,XM_030250541	Socs6	suppressor of cytokine signaling 6	protein_coding	[illegible]
54608	NM_018811	Abhd2	abhydrolase domain containing 2	protein_coding	[illegible]
54609	NM_018798	Ubqln2	ubiquilin 2	protein_coding	[illegible]
54610	NM_001357521,NM_001357522,NM_018775,XM_017321761,XM_017321765,XR_001785175,XR_003956253	Tbc1d8	TBC1 domain family, member 8	protein_coding	[illegible]
54611	NM_018779,XM_006506979,XM_011241595	Pde3a	phosphodiesterase 3A, cGMP inhibited	protein_coding	[illegible]
54612	NM_018780	Sfrp5	secreted frizzled-related sequence protein 5	protein_coding	[illegible]
54613	NM_001357433,NM_001357434,NM_001357435,NM_018784,XM_006522391,XM_011245971,XM_011245972,XM_011245973,XM_011245974,XM_011245975,XM_011245976,XM_017317083,XM_030249193,XM_030249194,XR_003951800,XR_003951801,XR_003951802,XR_003951803,XR_003951804,XR_003951805,XR_384577	St3gal6	ST3 beta-galactoside alpha-2,3-sialyltransferase 6	protein_coding	[illegible]
54614	NM_001348256,NM_001370632,NM_001370633,NM_001370634,NM_001370635,NM_018786,NR_163483,XM_006521178,XM_006521179,XM_006521180,XM_006521182,XM_006521183,XM_006521184,XM_006521185,XM_006521186,XM_006521188,XM_006521189,XM_006521190,XM_011245697,XM_011245698,XM_017316707,XM_030248664,XM_030248665,XM_030248666,XM_030248667,XM_030248668,XR_003951410,XR_003951411,XR_875304	Prpf40b	pre-mRNA processing factor 40B	protein_coding	[illegible]
54615	NM_018787	Npff	neuropeptide FF-amide peptide precursor	protein_coding	[illegible]
54616	NM_001360385,NM_018788,XM_006519284,XM_006519286	Extl3	exostoses (multiple)-like 3	protein_coding	[illegible]
54624	NM_019458	Paf1	Paf1, RNA polymerase II complex component	protein_coding	[illegible]
54630	NM_001290624,XM_006527652	Prickle3	prickle planar cell polarity protein 3	protein_coding	[illegible]
54631	NM_019459,XM_011250647	Nphs1	nephrosis 1, nephrin	protein_coding	[illegible]
54632	NM_001290430,NM_133991	Ftsj1	FtsJ RNA methyltransferase homolog 1 (E. coli)	protein_coding	[illegible]
54633	NM_001252528,NM_001252529,NM_019478,XM_006527654	Pqbp1	polyglutamine binding protein 1	protein_coding	[illegible]
54634	NM_018832,NR_037581	Magix	MAGI family member, X-linked	protein_coding	[illegible]
54635	NM_001357746,NM_019971,XM_017319655	Pdgfc	platelet-derived growth factor, C polypeptide	protein_coding	[illegible]
54636	NM_001290792,NM_001290794,NM_001290795,NM_172372,XM_006527656,XM_006527659,XM_006527661,XM_006527662,XM_017318561,XM_030251388,XM_030251389,XM_030251390,XM_030251391,XM_030251392,XM_030251393,XM_030251394,XM_030251395,XM_030251396	Wdr45	WD repeat domain 45	protein_coding	[illegible]
54637	NM_138602	Praf2	PRA1 domain family 2	protein_coding	[illegible]
54638	NM_138603	Ccdc22	coiled-coil domain containing 22	protein_coding	[illegible]
54644	NM_001290536,NM_001290537,NM_138604,XM_006527665,XM_006527666,XM_017318564,XM_030251397,XM_030251398	Otud5	OTU domain containing 5	protein_coding	[illegible]
54645	NM_001290455,NM_001359289,NM_001359290,NM_001359291,NM_001359293,NM_207670,XM_006527669,XM_006527670,XM_006527671,XM_006527672,XM_006527673,XM_006527674,XM_006527676,XM_006527680,XM_006527681,XM_006527683,XM_006527684,XM_017318565,XM_017318566,XM_017318570,XM_030251399,XM_030251400,XM_030251401,XM_030251402,XM_030251403,XM_030251404,XM_030251405,XM_030251406,XM_030251407,XR_003953015,XR_003953016	Gripap1	GRIP1 associated protein 1	protein_coding	[illegible]
54646	NM_001290574,NM_138605	Ppp1r3f	protein phosphatase 1, regulatory subunit 3F	protein_coding	[illegible]
54648	NM_207202	Ccdc120	coiled-coil domain containing 120	protein_coding	[illegible]
54650	NM_001166531,NM_001166532,NM_019460,XM_006519287,XM_006519288,XM_006519289,XM_006519290,XM_011245120,XM_011245121,XM_011245122,XM_030247889	Sfmbt1	Scm-like with four mbt domains 1	protein_coding	[illegible]
54651	NM_019461	Usp27x	ubiquitin specific peptidase 27, X chromosome	protein_coding	[illegible]
54652	NM_019582,XM_017318571,XM_030251408	Cacna1f	calcium channel, voltage-dependent, alpha 1F subunit	protein_coding	[illegible]
54667	NM_001081182,NM_001368650,XM_006501715	Atp8b2	ATPase, class I, type 8B, member 2	protein_coding	[illegible]
54670	NM_001001488,XM_006526105	Atp8b1	ATPase, class I, type 8B, member 1	protein_coding	[illegible]
54672	NM_173036,XM_006531198,XM_006531199,XM_030243686,XR_387760	Adgrg3	adhesion G protein-coupled receptor G3	protein_coding	[illegible]
54673	NM_001282037,NM_001282042,NM_019464,XM_006501716,XM_006501717,XM_006501718,XM_030252693,XM_030252694,XM_030252695	Sh3glb1	SH3-domain GRB2-like B1 (endophilin)	protein_coding	[illegible]
54678	NM_018791,XM_017322372,XM_017322373,XM_017322374	Zfp108	zinc finger protein 108	protein_coding	[illegible]
54683	NM_001358444,NM_012021	Prdx5	peroxiredoxin 5	protein_coding	[illegible]
54698	NM_001281954,NM_019465	Crtam	cytotoxic and regulatory T cell molecule	protein_coding	[illegible]
54709	NM_018799	Eif3i	eukaryotic translation initiation factor 3, subunit I	protein_coding	[illegible]
54710	NM_018805,XM_030246160	Hs3st3b1	heparan sulfate (glucosamine) 3-O-sulfotransferase 3B1	protein_coding	[illegible]
54711	NM_018807	Plagl2	pleiomorphic adenoma gene-like 2	protein_coding	[illegible]
54712	NM_018797,XM_030245180	Plxnc1	plexin C1	protein_coding	[illegible]
54713	NM_080433,XM_030247890	Fezf2	Fez family zinc finger 2	protein_coding	[illegible]
54720	NM_001081549,NM_019466	Rcan1	regulator of calcineurin 1	protein_coding	[illegible]
54721	NM_001205312,NM_018793,XM_006510492,XM_006510494,XM_011242579,XM_030244455,XM_030244456,XM_030244457,XR_001778942	Tyk2	tyrosine kinase 2	protein_coding	[illegible]
54722	NM_018769,XM_006506402,XM_030255507,XR_001785156,XR_003956235	Gsdme	gasdermin E	protein_coding	[illegible]
54723	NM_018783	Tfip11	tuftelin interacting protein 11	protein_coding	[illegible]
54725	NM_001025600,NM_001310841,NM_018770,NM_207675,NM_207676,XM_006510497,XM_017313487,XM_017313488,XM_030244458,XM_030244459,XM_030244460	Cadm1	cell adhesion molecule 1	protein_coding	[illegible]
55925	NM_001285857,NM_001285858,NM_001285861,NM_018802,NR_153437,XM_006508632,XM_006508633,XM_006508635,XM_006508637,XM_006508638,XM_006508639,XM_011242014,XM_011242015,XM_011242016	Syt8	synaptotagmin VIII	protein_coding	[illegible]
55927	NM_001360899,NM_001360900,NM_019479	Hes6	hairy and enhancer of split 6	protein_coding	[illegible]
55932	NM_001289492,NM_001289493,NM_001355403,NM_018734,XM_006501719,XM_006501724	Gbp3	guanylate binding protein 3	protein_coding	[illegible]
55934	NM_018739	Rp9	retinitis pigmentosa 9 (human)	protein_coding	[illegible]
55935	NM_018828,XM_006499919,XR_001783149,XR_003953674,XR_003953675,XR_003953676,XR_003953677	Fnbp4	formin binding protein 4	protein_coding	[illegible]
55936	NM_001168568,NM_001168569,NM_001168571,NM_001358398,NM_018737,NR_033143,XM_006528912,XM_006528913,XM_006528915,XM_030251409,XM_030251410	Ctps2	cytidine 5'-triphosphate synthase 2	protein_coding	[illegible]
55938	NM_018816	Apom	apolipoprotein M	protein_coding	[illegible]
55942	NM_018820,XM_006540217	Sertad1	SERTA domain containing 1	protein_coding	[illegible]
55943	NM_001356314,NM_001361350,NM_001361351,NM_001361352,NM_018768,XM_030246161	Stx8	syntaxin 8	protein_coding	[illegible]
55944	NM_018749,XM_006521192	Eif3d	eukaryotic translation initiation factor 3, subunit D	protein_coding	[illegible]
55946	NM_018829,XM_006519292,XM_006519293,XR_003950866	Ap3m1	adaptor-related protein complex 3, mu 1 subunit	protein_coding	[illegible]
55947	NM_001360437,NM_001360438,NM_001360439,NM_018831,NR_153439,NR_153440,XM_006527217,XR_877682	Dclre1a	DNA cross-link repair 1A	protein_coding	[illegible]
55948	NM_018754	Sfn	stratifin	protein_coding	[illegible]
55949	NM_018796	Eef1b2	eukaryotic translation elongation factor 1 beta 2	protein_coding	[illegible]
55950	NM_001163709,NM_018772	Bri3	brain protein I3	protein_coding	[illegible]
55951	NM_001364918,NM_001364919,NM_018819,NR_157360,XM_011246219,XM_030249914	Mpc1	mitochondrial pyruvate carrier 1	protein_coding	[illegible]
55960	NM_001357690,NM_001357691,NM_019480,XM_006521195	Ebag9	estrogen receptor-binding fragment-associated gene 9	protein_coding	[illegible]
55963	NM_018861	Slc1a4	solute carrier family 1 (glutamate/neutral amino acid transporter),	protein_coding	[illegible]
55978	NM_018854	Ift20	intraflagellar transport 20	protein_coding	[illegible]
55979	NM_001163379,NM_018862,XM_006524647,XM_006524648,XM_030249915	Agpat1	1-acylglycerol-3-phosphate O-acyltransferase 1 (lysophosphatidic acid acyltransferase, alpha)	protein_coding	[illegible]
55980	NM_001310433,NM_018864,XM_006530077,XM_006530078	Impa1	inositol (myo)-1(or 4)-monophosphatase 1	protein_coding	[illegible]
55981	NM_018889,XM_006511287,XM_011242773,XR_379429,XR_379430,XR_379431,XR_870761	Pigb	phosphatidylinositol glycan anchor biosynthesis, class B	protein_coding	[illegible]
55982	NM_018878,XM_006535758,XM_030254707,XM_030254708,XM_030254709	Paxip1	PAX interacting (with transcription-activation domain) protein 1	protein_coding	[illegible]
55983	NM_018884,XM_006506405	Pdzrn3	PDZ domain containing RING finger 3	protein_coding	[illegible]
55984	NM_001362841,NM_018883,NR_156179,XM_006533761	Camkk1	calcium/calmodulin-dependent protein kinase kinase 1, alpha	protein_coding	[illegible]
55985	NM_018866	Cxcl13	chemokine (C-X-C motif) ligand 13	protein_coding	[illegible]
55987	NM_018867,XM_006508033,XM_006508034,XM_006508036,XM_011241864,XM_030242804	Cpxm2	carboxypeptidase X 2 (M14 family)	protein_coding	[illegible]
55988	NM_001110310,NM_001110311,NM_018875,XM_011247645,XM_011247646	Snx12	sorting nexin 12	protein_coding	[illegible]
55989	NM_018868,XM_006496149	Nop58	NOP58 ribonucleoprotein	protein_coding	[illegible]
55990	NM_001360913,NM_001360914,NM_018881	Fmo2	flavin containing monooxygenase 2	protein_coding	[illegible]
55991	NM_019482	Panx1	pannexin 1	protein_coding	[illegible]
55992	NM_001285870,NM_001285871,NM_001285873,NM_001360425,NM_018880,XM_006508038,XM_006508039,XM_006508040,XM_006508041,XM_030242806	Trim3	tripartite motif-containing 3	protein_coding	[illegible]
55993	NM_001282054,NM_031870	Msh4	mutS homolog 4	protein_coding	[illegible]
55994	NM_019483,XM_006501728,XM_006501729	Smad9	SMAD family member 9	protein_coding	[illegible]
56009	NM_019484	Alyref2	Aly/REF export factor 2	protein_coding	[illegible]
56012	NM_018870	Pgam2	phosphoglycerate mutase 2	protein_coding	[illegible]
56013	NM_018873,XM_006533765,XM_006533779,XM_006533781,XM_006533782,XM_006533783,XM_017314666,XM_017314667,XM_017314668,XM_017314669,XM_017314670,XM_017314671,XM_017314672,XM_017314673,XM_017314674,XM_017314675,XM_017314677,XM_017314678,XM_017314679,XM_017314680,XM_017314681,XM_017314682,XM_017314683,XM_030246162,XM_030246163,XM_030246164,XM_030246165,XM_030246166,XM_030246167,XM_030246168,XM_030246169,XM_030246170,XM_030246171,XM_030246172,XM_030246173,XM_030246174,XM_030246175,XM_030246176,XM_030246177,XM_030246178,XM_030246179,XM_030246180,XM_030246181,XM_030246182,XM_030246183,XM_030246184,XM_030246185,XM_030246186,XR_001780031,XR_003949481	Srcin1	SRC kinase signaling inhibitor 1	protein_coding	[illegible]
56014	NM_019485	Olfr70	olfactory receptor 70	protein_coding	[illegible]
56016	NM_019487	Hebp2	heme binding protein 2	protein_coding	[illegible]
56017	NM_001356409,NM_019488,NR_151460,NR_151461	Slc2a8	solute carrier family 2, (facilitated glucose transporter), member 8	protein_coding	[illegible]
56018	NM_001360460,NM_019990,XM_030242807	Stard10	START domain containing 10	protein_coding	[illegible]
56030	NM_018872,XM_006496150,XM_006496152,XM_017321814	Tmem131	transmembrane protein 131	protein_coding	[illegible]
56031	NM_019489,XM_006503250,XM_011240581,XM_030253678	Ppie	peptidylprolyl isomerase E (cyclophilin E)	protein_coding	[illegible]
56032	NM_018879,XM_006511768,XM_006511769,XM_011242898,XR_003947955,XR_379769	Nprl2	NPR2 like, GATOR1 complex subunit	protein_coding	[illegible]
56036	NM_207678,XM_011250304,XM_011250305,XM_030253679,XR_001784165,XR_001784166,XR_003954988,XR_881434	Ccnl2	cyclin L2	protein_coding	[illegible]
56040	NM_018853	Rplp1	ribosomal protein, large, P1	protein_coding	[illegible]
56041	NM_019490,XM_006535140,XM_006535141,XM_017321009	Uso1	USO1 vesicle docking factor	protein_coding	[illegible]
56043	NM_018859,XM_006516485	Akr1e1	aldo-keto reductase family 1, member E1	protein_coding	[illegible]
56044	NM_019491	Rala	v-ral simian leukemia viral oncogene A (ras related)	protein_coding	[illegible]

56045	NM_001139520,NM_001370610,NM_018851	Samhd1	SAM domain and HD domain, 1	protein_coding	[illegible]
56046	NM_001355441,NM_001355442,NM_001355443,NM_018888,XM_006499922,XM_011239705,XM_011239706,XM_030251889,XR_003953678,XR_003953679	Uqcc1	ubiquinol-cytochrome c reductase complex assembly factor 1	protein_coding	[illegible]
56047	NM_001356286,NM_018857,XM_006524649	Msln	mesothelin	protein_coding	[illegible]
56048	NM_001199043,NM_001291055,NM_001291057,NM_001291060,NM_018886,XM_006516714,XM_006516715,XM_011244320,XM_011244321,XM_011244322,XM_011244323,XM_011244324,XM_011244325,XM_017315547,XM_017315548,XM_017315549,XM_030247327	Lgals8	lectin, galactose binding, soluble 8	protein_coding	[illegible]
56050	NM_001285947,NM_001285948,NM_018887	Cyp39a1	cytochrome P450, family 39, subfamily a, polypeptide 1	protein_coding	[illegible]
56055	NM_001145979,NM_001357745,NM_019581,XM_006524651,XM_017317582,XM_030249916,XM_030249917,XR_385348	Gtpbp2	GTP binding protein 2	protein_coding	[illegible]
56057	NM_019493,XM_006510499,XM_006510500,XM_006510501,XM_006510502,XM_017313490	Btg4	B cell translocation gene 4	protein_coding	[illegible]
56066	NR_038116	Cxcl11	chemokine (C-X-C motif) ligand 11	transcribed_pseudogene	[illegible]
56068	NM_019496	Ammecr1	Alport syndrome, mental retardation, midface hypoplasia and elliptocytosis chromosomal region gene 1	protein_coding	[illegible]
56069	NM_019508,XM_006526106,XM_006526108,XM_030250543	Il17b	interleukin 17B	protein_coding	[illegible]
56070	NM_001039474,NM_001289526,NM_001360881,NR_110344,XM_006526110,XM_006526111,XM_006526112,XM_006526113,XM_030250545,XR_001782383,XR_001782384,XR_001782385,XR_001782386,XR_386004	Tcerg1	transcription elongation regulator 1 (CA150)	protein_coding	[illegible]
56072	NM_001356574,NM_001370686,NM_019516,XM_006527221	Lgals12	lectin, galactose binding, soluble 12	protein_coding	[illegible]
56075	NM_001355467,NM_019501,NR_149721,XM_006498188,XM_006498189,XM_006498191,XM_017319134,XM_030251890,XR_001783150,XR_001783152,XR_001783153,XR_374108	Pdss1	prenyl (solanesyl) diphosphate synthase, subunit 1	protein_coding	[illegible]
56077	NM_001362845,NM_019505,NR_156181,XM_011249151,XR_001780032	Dgke	diacylglycerol kinase, epsilon	protein_coding	[illegible]
56078	NM_181315	Car5b	carbonic anhydrase 5b, mitochondrial	protein_coding	[illegible]
56079	NM_019514,NM_207109,XM_006538104,XM_006538105,XM_030253680	Astn2	astrotactin 2	protein_coding	[illegible]
56085	NM_026842,NM_152234	Ubqln1	ubiquilin 1	protein_coding	[illegible]
56086	NM_001204875,NM_023871	Set	SET nuclear oncogene	protein_coding	[illegible]
56087	NM_019536	Dnah10	dynein, axonemal, heavy chain 10	protein_coding	[illegible]
56088	NM_019537	Psmg1	proteasome (prosome, macropain) assembly chaperone 1	protein_coding	[illegible]
56089	NM_019511,XM_006514761	Ramp3	receptor (calcitonin) activity modifying protein 3	protein_coding	[illegible]
56095	NM_025310	Ftsj3	FtsJ RNA methyltransferase homolog 3 (E. coli)	protein_coding	[illegible]
56096	NM_001357802,NM_019538,XM_006541525,XM_011251016	Plac1	placental specific protein 1	protein_coding	[illegible]
56149	NM_019518,XM_006521196	Grasp	GRP1 (general receptor for phosphoinositides 1)-associated scaffold	protein_coding	[illegible]
56150	NM_001355624,NM_019499	Mad2l1	MAD2 mitotic arrest deficient-like 1	protein_coding	[illegible]
56174	NM_001164187,NM_001347315,NM_019542,XM_006506408,XM_006506409,XM_006506410,XM_017321685,XM_017321686,XM_030255509,XM_030255510	Nagk	N-acetylglucosamine kinase	protein_coding	[illegible]
56175	NM_019517,XM_006523068	Bace2	beta-site APP-cleaving enzyme 2	protein_coding	[illegible]
56176	NM_001159616,NM_001159617,NM_001159618,NM_001159619,NM_001159620,NM_019543	Pigp	phosphatidylinositol glycan anchor biosynthesis, class P	protein_coding	[illegible]
56177	NM_001038612,NM_001038613,NM_001038614,NM_019498	Olfm1	olfactomedin 1	protein_coding	[illegible]
56183	NM_019515	Nmu	neuromedin U	protein_coding	[illegible]
56184	NM_019544	Msgn1	mesogenin 1	protein_coding	[illegible]
56187	NM_001360387,NM_001360388,NM_019519,NR_153435	Rabggta	Rab geranylgeranyl transferase, a subunit	protein_coding	[illegible]
56188	NM_019503,NM_052991,NM_052992,NM_194321,XM_006540219,XM_011250648,XM_011250649,XM_011250650,XM_011250651,XM_011250652,XM_030242808,XM_030242809	Fxyd1	FXYD domain-containing ion transport regulator 1	protein_coding	[illegible]
56190	NM_019547,XM_006499924,XM_030251891,XM_030251892	Rbm38	RNA binding motif protein 38	protein_coding	[illegible]
56191	NM_001002272,NM_001290770,NM_001290771,NM_001290772,NM_019548,XM_006528916,XM_011247845,XM_011247846,XM_011247847,XM_011247848,XM_017318572,XM_017318573	Tro	trophinin	protein_coding	[illegible]
56193	NM_019549	Plek	pleckstrin	protein_coding	[illegible]
56194	NM_001371015,NM_001371016,NM_001371017,NM_001371018,NM_001371019,NM_001371020,NM_018785,XM_006498193,XM_011239140,XM_030251893	Prpf40a	pre-mRNA processing factor 40A	protein_coding	[illegible]
56195	NM_001310711,NM_019550,XM_006501731,XM_006501732,XR_001783718,XR_001783719,XR_375549,XR_375550	Ptbp2	polypyrimidine tract binding protein 2	protein_coding	[illegible]
56196	NM_019551	Tdp2	tyrosyl-DNA phosphodiesterase 2	protein_coding	[illegible]
56198	NM_013905	Heyl	hairy/enhancer-of-split related with YRPW motif-like	protein_coding	[illegible]
56199	NM_019552,XM_006531201	Abcb10	ATP-binding cassette, sub-family B (MDR/TAP), member 10	protein_coding	[illegible]
56200	NM_019553,XM_030245181	Ddx21	DEAD (Asp-Glu-Ala-Asp) box polypeptide 21	protein_coding	[illegible]
56205	NM_001026212,NM_019561	Ensa	endosulfine alpha	protein_coding	[illegible]
56207	NM_001159866,NM_019562,XM_006529760,XR_001785184,XR_387185	Uchl5	ubiquitin carboxyl-terminal esterase L5	protein_coding	[illegible]
56208	NM_001359819,NM_001359820,NM_001359821,NM_019584	Becn1	beclin 1, autophagy related	protein_coding	[illegible]
56209	NM_019580	Gde1	glycerophosphodiester phosphodiesterase 1	protein_coding	[illegible]
56210	NM_001359287,NM_001359288,NM_019570,XM_006496153,XM_006496154,XM_006496155,XM_006496156,XM_006496161,XM_006496162,XM_006496164,XM_017321835,XM_017321836,XM_030255639,XM_030255641	Rev1	REV1, DNA directed polymerase	protein_coding	[illegible]
56212	NM_019566	Rhog	ras homolog family member G	protein_coding	[illegible]
56213	NM_019564	Htra1	HtrA serine peptidase 1	protein_coding	[illegible]
56214	NM_019575	Scamp4	secretory carrier membrane protein 4	protein_coding	[illegible]
56215	NM_001085472,NM_001085473,NM_001242605,NM_001242606,NM_019567,NM_023190,XM_006519296,XM_006519297,XM_006519298,XM_006519301,XM_006519304,XM_006519308,XM_030247893,XM_030247894,XM_030247895,XM_030247896,XR_003950867,XR_003950868,XR_003950869,XR_003950870,XR_003950871,XR_003950872,XR_003950873,XR_003950874,XR_003950875,XR_	Acin1	apoptotic chromatin condensation inducer 1	protein_coding	[illegible]
56216	NM_024414,XM_006508044,XM_011241866	Stx1b	syntaxin 1B	protein_coding	[illegible]
56217	NM_019579,XM_006516097,XM_006516098,XM_017315139,XM_030246824	Mpp5	membrane protein, palmitoylated 5 (MAGUK p55 subfamily member 5)	protein_coding	[illegible]
56218	NM_001253690,NM_001253691,NM_019574,NR_045108,NR_0451	Patz1	POZ (BTB) and AT hook containing zinc finger 1	protein_coding	[illegible]
56219	NM_001355493,NM_019578,XM_006539058	Extl1	exostoses (multiple)-like 1	protein_coding	[illegible]
56220	NM_001004066,NM_001286214,NM_019565,XM_011244149,XM_011244150	Zfp386	zinc finger protein 386 (Kruppel-like)	protein_coding	[illegible]
56221	NM_001356630,NM_019577,XM_006504444	Ccl24	chemokine (C-C motif) ligand 24	protein_coding	[illegible]
56222	NM_019563	Cited4	Cbp/p300-interacting transactivator, with Glu/Asp-rich carboxy-terminal domain, 4	protein_coding	[illegible]
56223	NM_019569,XM_006505141	Fscn3	fascin actin-bundling protein 3	protein_coding	[illegible]
56224	NM_019571,NR_121195,XM_006501736,XM_006501737,XM_030252697,XR_375551	Tspan5	tetraspanin 5	protein_coding	[illegible]
56226	NM_019585,NM_207687,NM_207688,NM_207689,NM_207690,NM_207691,XM_006539059,XM_006539060,XM_006539061,XM_006539063	Espn	espin	protein_coding	[illegible]
56228	NM_001355494,NM_019586	Ube2j1	ubiquitin-conjugating enzyme E2J 1	protein_coding	[illegible]
56229	NM_001205253,NM_001293688,NM_019576,XM_006509173,XM_006509174,XM_006509177,XM_030243687,XM_030243688,XM_030243689,XM_030243690	Thsd1	thrombospondin, type I, domain 1	protein_coding	[illegible]
56233	NM_001204275,NM_001204276,NM_001204277,NM_001204278,NM_001204279,NM_001204280,NM_001204281,NM_019572,XM_006521203,XM_006521205,XM_006521207,XM_006521208,XM_006521209,XM_006521210,XM_011245699,XM_030248669,XR_003951412,XR_003951413,XR_003951414,XR_003951415,XR_00395141	Hdac7	histone deacetylase 7	protein_coding	[illegible]
56248	NM_001365071,NM_021299,XM_006527222	Ak3	adenylate kinase 3	protein_coding	[illegible]
56249	NM_027493,XM_006519309,XM_030247897,XM_030247898,XM_030247899	Actr8	ARP8 actin-related protein 8	protein_coding	[illegible]
56258	NM_001313716,NM_001313717,NM_019868	Hnrnph2	heterogeneous nuclear ribonucleoprotein H2	protein_coding	[illegible]
56264	NM_019696	Cpxm1	carboxypeptidase X 1 (M14 family)	protein_coding	[illegible]
56273	NM_019781	Pex14	peroxisomal biogenesis factor 14	protein_coding	[illegible]
56274	NM_001357821,NM_019635,XM_006520119,XM_006520120,XM_030248670,XM_030248671	Stk3	serine/threonine kinase 3	protein_coding	[illegible]
56275	NM_019869	Rbm14	RNA binding motif protein 14	protein_coding	[illegible]
56277	NM_019631,XM_006522395,XM_006522396,XM_017317086,XM_030249196,XM_030249197	Tmem45a	transmembrane protein 45a	protein_coding	[illegible]
56278	NM_019832,XM_006517301,XM_006517304,XM_011244542,XM_011244543,XM_030247329,XM_030247330	Gkap1	G kinase anchoring protein 1	protein_coding	[illegible]
56279	NM_019833	Dipk1b	divergent protein kinase domain 1B	protein_coding	[illegible]
56280	NM_025500	Mrpl37	mitochondrial ribosomal protein L37	protein_coding	[illegible]
56282	NM_027204	Mrpl12	mitochondrial ribosomal protein L12	protein_coding	[illegible]
56284	NM_026490	Mrpl19	mitochondrial ribosomal protein L19	protein_coding	[illegible]
56289	NM_001243748,NM_019713,XM_006511770	Rassf1	Ras association (RalGDS/AF-6) domain family member 1	protein_coding	[illegible]
56291	NM_019637	Styx	serine/threonine/tyrosine interaction protein	protein_coding	[illegible]
56292	NM_001177965,NM_001310538,NM_019870,XR_003953017	Naa10	N(alpha)-acetyltransferase 10, NatA catalytic subunit	protein_coding	[illegible]
56293	NM_019871	Slc35g3	solute carrier family 35, member G3	protein_coding	[illegible]
56294	NM_019651	Ptpn9	protein tyrosine phosphatase, non-receptor type 9	protein_coding	[illegible]
56295	NM_001357837,NM_001357838,NM_001357839,NM_001357840,NM_001357841,NM_019814	Higd1a	HIG1 domain family, member 1A	protein_coding	[illegible]
56296	NM_019872,NR_151717,XM_030253681,XM_030253682	Dmrtb1	DMRT-like family B with proline-rich C-terminal, 1	protein_coding	[illegible]
56297	NM_001347244,NM_019665	Arl6	ADP-ribosylation factor-like 6	protein_coding	[illegible]
56298	NM_001286647,NM_001357752,NM_001357753,NM_019717,NM_178050,XM_006524655,XM_006524656,XM_006524657	Atl2	atlastin GTPase 2	protein_coding	[illegible]
56299	NM_019873	Fkbpl	FK506 binding protein-like	protein_coding	[illegible]
56305	NM_001301643,NM_001301644,NM_001301666,NM_001359215,NM_001359216,NM_001359217,NM_019640	Pitpnb	phosphatidylinositol transfer protein, beta	protein_coding	[illegible]
56306	NM_001355645,NM_001361398,NM_019643,XM_006507074,XM_006507075,XM_030255511	Sinhcaf	SIN3-HDAC complex associated factor	protein_coding	[illegible]
56307	NM_019648,XM_006513896,XM_006513897,XM_017314032	Metap2	methionine aminopeptidase 2	protein_coding	[illegible]
56309	NM_019660	Mycbp	MYC binding protein	protein_coding	[illegible]
56310	NM_001357906,NM_019726,XM_030246187	Gps2	G protein pathway suppressor 2	protein_coding	[illegible]
56312	NM_019738	Nupr1	nuclear protein transcription regulator 1	protein_coding	[illegible]
56314	NM_019747	Zfp113	zinc finger protein 113	protein_coding	[illegible]
56315	NM_019799,XM_006541004,XM_011250878	Rhcg	Rhesus blood group-associated C glycoprotein	protein_coding	[illegible]
56316	NM_019802,XM_006506414,XM_006506416,XM_030255512,XR_001785157,XR_001785158,XR_003956237,XR_003956238,XR_003956239	Ggcx	gamma-glutamyl carboxylase	protein_coding	[illegible]
56317	NM_019805	Anapc7	anaphase promoting complex subunit 7	protein_coding	[illegible]
56318	NM_019807,NM_207668	Acpp	acid phosphatase, prostate	protein_coding	[illegible]
56320	NM_001177371,NM_001177372,NM_019813,XM_006517305	Dbn1	drebrin 1	protein_coding	[illegible]
56321	NM_019816,XM_006533789,XM_011249153,XR_003949482,XR_388498,XR_388499,XR_879720	Aatf	apoptosis antagonizing transcription factor	protein_coding	[illegible]
56322	NM_001291161,NM_019818,NM_023355,NR_111896	Timm22	translocase of inner mitochondrial membrane 22	protein_coding	[illegible]
56323	NM_001355438,NM_001355439,NM_001355440,XM_006538108,XM_006538112,XM_030253683,XM_030253684	Dnajb5	DnaJ heat shock protein family (Hsp40) member B5	protein_coding	[illegible]
56324	NM_019667,XM_006498198	Stam2	signal transducing adaptor molecule (SH3 domain and ITAM motif) 2	protein_coding	[illegible]
56325	NM_019875,XM_017321012,XR_001784728	Abcb9	ATP-binding cassette, sub-family B (MDR/TAP), member 9	protein_coding	[illegible]
56327	NM_019722	Arl2	ADP-ribosylation factor-like 2	protein_coding	[illegible]
56330	NM_019746	Pdcd5	programmed cell death 5	protein_coding	[illegible]
56332	NM_019764,XM_006511771,XM_006511772,XM_006511773	Amotl2	angiomotin-like 2	protein_coding	[illegible]
56334	NM_019770	Tmed2	transmembrane p24 trafficking protein 2	protein_coding	[illegible]
56335	NM_019721,XM_006519311,XM_017316096,XR_001781134	Mettl3	methyltransferase like 3	protein_coding	[illegible]
56336	NM_019835	B4galt5	UDP-Gal:betaGlcNAc beta 1,4-galactosyltransferase, polypeptide 5	protein_coding	[illegible]
56338	NM_001009935,NM_023719	Txnip	thioredoxin interacting protein	protein_coding	[illegible]
56347	NM_146200	Eif3c	eukaryotic translation initiation factor 3, subunit C	protein_coding	[illegible]
56348	NM_019657	Hsd17b12	hydroxysteroid (17-beta) dehydrogenase 12	protein_coding	[illegible]
56349	NM_001047159,NM_019671,XM_030247332	Net1	neuroepithelial cell transforming gene 1	protein_coding	[illegible]
56350	NM_001355233,NM_019718	Arl3	ADP-ribosylation factor-like 3	protein_coding	[illegible]
56351	NM_019766	Ptges3	prostaglandin E synthase 3	protein_coding	[illegible]
56353	NM_019743	Rybp	RING1 and YY1 binding protein	protein_coding	[illegible]
56354	NM_019795,XM_011249154,XM_030246188,XR_001780034	Dnajc7	DnaJ heat shock protein family (Hsp40) member C7	protein_coding	[illegible]
56356	NM_019821	Gltp	glycolipid transfer protein	protein_coding	[illegible]
56357	NM_019826,XM_006499925	Ivd	isovaleryl coenzyme A dehydrogenase	protein_coding	[illegible]
56358	NM_019877	Copz2	coatomer protein complex, subunit zeta 2	protein_coding	[illegible]
56360	NM_001313718,NM_019736,XM_030251411	Acot9	acyl-CoA thioesterase 9	protein_coding	[illegible]
56361	NM_001025561,NM_001025562,NM_001347390,NM_001359218,NM_001359219,NM_019700,XM_006535148,XM_017321014,XM_017321015	Pus1	pseudouridine synthase 1	protein_coding	[illegible]
56363	NM_019790,XM_030255650	Tmeff2	transmembrane protein with EGF-like and two follistatin-like domains 2	protein_coding	[illegible]
56364	NM_001177985,NM_001177986,NM_001177987,NM_001177988,NM_019831,XM_006528160,XM_006528161,XM_006528162,XM_006528163,XM_006528164,XM_006528165,XM_006528166,XM_006528167,XM_006528168,XM_006528169,XM_006528170,XM_006528171,XM_011247650,XM_011247651,XM_017318577,XM_030251412,XM_030251413,XM_030251414,XM_030251415,XM_030251416,XM_030251417,XM_030251418,XM_030251419,XM_030251420,XM_030251421,XM_030251422,XM_030251423,XM_030251424,XM_030251425,XM_030251426,XM_030251427	Zmym3	zinc finger, MYM-type 3	protein_coding	[illegible]
56365	NM_019701,XM_006539066,XM_006539067,XM_006539068,XM_011250307	Clcnkb	chloride channel, voltage-sensitive Kb	protein_coding	[illegible]
56367	NM_001039137,NM_001285992,NM_019708,XM_006531204,XM_006531205,XM_011248448	Scoc	short coiled-coil protein	protein_coding	[illegible]
56368	NM_001357289,NM_001357290,NM_001357291,NM_019720,XM_006511776,XM_030244461	Cyb561d2	cytochrome b-561 domain containing 2	protein_coding	[illegible]
56369	NM_001355473,NM_001355474,NM_019735	Apip	APAF1 interacting protein	protein_coding	[illegible]
56370	NM_019754	Tagln3	transgelin 3	protein_coding	[illegible]
56371	NM_019757	Fzr1	fizzy and cell division cycle 20 related 1	protein_coding	[illegible]
56372	NM_019772,XM_030242810,XM_030242811	1110004F10Rik	RIKEN cDNA 1110004F10 gene	protein_coding	[illegible]
56374	NM_029565	Tmem59	transmembrane protein 59	protein_coding	[illegible]
56375	NM_001285793,NM_019804,NR_152434,XM_006522399,XM_011245977,XR_003951806,XR_003951807,XR_875837	B4galt4	UDP-Gal:betaGlcNAc beta 1,4-galactosyltransferase, polypeptide 4	protein_coding	[illegible]
56376	NM_001190852,NM_001190853,NM_001190854,NM_001190855,NM_001190856,NM_001190857,NM_019808,NM_019809,NM_022554,XM_006501738,XM_006501739,XM_006501740,XM_006501741,XM_006501742,XM_006501743,XM_006501744,XM_006501745,XM_006501746,XM_006501748,XM_006501750,XM_006501751,XM_017319659	Pdlim5	PDZ and LIM domain 5	protein_coding	[illegible]
56378	NM_019824	Arpc3	actin related protein 2/3 complex, subunit 3	protein_coding	[illegible]
56380	NM_019689,XM_006511291,XM_006511292,XM_011242774,XM_011242776,XM_030244462	Arid3b	AT rich interactive domain 3B (BRIGHT-like)	protein_coding	[illegible]
56381	NM_001347235,NM_019763,XM_006539069,XM_006539070,XM_006539073,XM_006539074	Spen	spen family transcription repressor	protein_coding	[illegible]
56382	NM_019773,XM_006528922	Rab9	RAB9, member RAS oncogene family	protein_coding	[illegible]
56384	NM_019694,XM_006504034	Letm1	leucine zipper-EF-hand containing transmembrane protein 1	protein_coding	[illegible]
56386	NM_019737,XM_030250546	B4galt6	UDP-Gal:betaGlcNAc beta 1,4-galactosyltransferase, polypeptide 6	protein_coding	[illegible]
56389	NM_001167799,NM_019829,XM_030250998,XM_030250999,XM_030251000	Stx5a	syntaxin 5A	protein_coding	[illegible]
56390	NM_020491	Znrd2	zinc ribbon domain containing 2	protein_coding	[illegible]
56392	NM_001168505,NM_001355242,NM_019658,XM_006527228,XM_011247299,XM_030251001	Shoc2	Shoc2, leucine rich repeat scaffold protein	protein_coding	[illegible]
56395	NM_019704	Tmem115	transmembrane protein 115	protein_coding	[illegible]
56397	NM_001168225,NM_001168226,NM_001168227,NM_001168228,NM_001168229,NM_001168230,NM_019768,XM_006528584	Morf4l2	mortality factor 4 like 2	protein_coding	[illegible]
56398	NM_019769	Chp1	calcineurin-like EF hand protein 1	protein_coding	[illegible]
56399	NM_001357760,NM_019774,NR_131218,NR_131219,XM_006524663,XM_006524665,XM_006524666	Akap8	A kinase (PRKA) anchor protein 8	protein_coding	[illegible]
56401	NM_001042411,NM_001286148,NM_019782,NM_019783,NR_149723	P3h1	prolyl 3-hydroxylase 1	protein_coding	[illegible]
56403	NM_001284328,NM_001311113,NM_019666,NM_019796,XM_006511293,XM_006511294	Syncrip	synaptotagmin binding, cytoplasmic RNA interacting protein	protein_coding	[illegible]
56404	NM_001170907,NM_001357872,NM_019797,XM_006511296,XM_006511298,XM_017313492,XM_017313493,XM_017313494,XM_030244463,XM_030244464	Trip4	thyroid hormone receptor interactor 4	protein_coding	[illegible]
56405	NM_019819,XM_006533796,XM_006533797,XM_006533798,XM_006533799	Dusp14	dual specificity phosphatase 14	protein_coding	[illegible]
56406	NM_001242558,NM_019825,XM_006499927,XM_006499928,XM_011239708,XM_011239709	Ncoa6	nuclear receptor coactivator 6	protein_coding	[illegible]
56407	NM_001163452,NM_019828	Trpc4ap	transient receptor potential cation channel, subfamily C, member 4 associated protein	protein_coding	[illegible]
56409	NM_001291046,NM_019837	Nudt3	nudix (nucleotide diphosphate linked moiety X)-type motif 3	protein_coding	[illegible]
56410	NM_019820	Cbln3	cerebellin 3 precursor protein	protein_coding	[illegible]
56412	NM_019836,XM_006535153,XM_011249546	Noa1	nitric oxide associated 1	protein_coding	[illegible]
56417	NM_001038587,NM_001146296,NM_001357958,NM_019655,XM_006501753,XM_006501754,XM_030252698	Adar	adenosine deaminase, RNA-specific	protein_coding	[illegible]
56418	NM_019661	Ykt6	YKT6 v-SNARE homolog (S. cerevisiae)	protein_coding	[illegible]
56419	NM_019670,XM_006519312,XM_011245123	Diaph3	diaphanous related formin 3	protein_coding	[illegible]

56420	Ppp4c	protein phosphatase 4, catalytic subunit	protein_coding	1891763	.	34.033395
56421	Pfkp	phosphofructokinase, platelet	protein_coding	1891833	.	8.490899
56422	Hbs1l	Hbs1-like (S. cerevisiae)	protein_coding	1891704	.	19.114315
56424	Stub1	STIP1 homology and U-Box containing protein 1	protein_coding	1891731	.	61.125700
56426	Pdcd10	programmed cell death 10	protein_coding	1928396	.	17.551153
56427	Tubd1	tubulin, delta 1	protein_coding	1891826	.	2.101410
56428	Mtch2	mitochondrial carrier 2	protein_coding	1929260	.	23.666811
56429	Dpt	dermatopontin	protein_coding	1928392	.	17.493793
56430	Clip1	CAP-GLY domain containing linker protein 1	protein_coding	1928401	.	10.311079
56431	Dstn	destrin	protein_coding	1929270	.	138.712799
56433	Vps29	VPS29 retromer complex component	protein_coding	1928344	.	12.802618
56434	Tspan3	tetraspanin 3	protein_coding	1928098	.	93.265945
56436	Adrm1	adhesion regulating molecule 1	protein_coding	1929289	.	31.888658
56437	Rrad	Ras-related associated with diabetes	protein_coding	1930943	.	4.983020
56438	Rbx1	ring-box 1	protein_coding	1891829	.	29.929256
56440	Snx1	sorting nexin 1	protein_coding	1928395	.	34.605652
56441	Naa80	N(alpha)-acetyltransferase 80, NatH catalytic subunit	protein_coding	1888902	.	13.090767
56442	Serinc1	serine incorporator 1	protein_coding	1926228	.	77.254486
56443	Arpc1a	actin related protein 2/3 complex, subunit 1A	protein_coding	1928896	.	35.578621
56444	Actr10	ARP10 actin-related protein 10	protein_coding	1891654	.	14.561848
56445	Dnaja2	DnaJ heat shock protein family (Hsp40) member A2	protein_coding	1931882	.	29.970789
56447	Copz1	coatomer protein complex, subunit zeta 1	protein_coding	1929063	.	57.492736
56448	Cyp2d22	cytochrome P450, family 2, subfamily d, polypeptide 22	protein_coding	1929474	.	6.094615
56449	Ybx3	Y box protein 3	protein_coding	2137670	.	46.669487
56451	Suclg1	succinate-CoA ligase, GDP-forming, alpha subunit	protein_coding	1927234	.	38.032883
56452	Orc6	origin recognition complex, subunit 6	protein_coding	1929285	.	13.959376
56453	Mbtps1	membrane-bound transcription factor peptidase, site 1	protein_coding	1927235	.	58.637827
56454	Aldh18a1	aldehyde dehydrogenase 18 family, member A1	protein_coding	1888908	.	29.784999
56455	Dynll1	dynein light chain LC8-type 1	protein_coding	1861457	.	62.149681
56456	Actl6a	actin-like 6A	protein_coding	1861453	.	16.277854
56457	Clptm1	cleft lip and palate associated transmembrane protein 1	protein_coding	1927155	.	17.194554
56458	Foxo1	forkhead box O1	protein_coding	1890077	.	7.921490
56459	Sae1	SUMO1 activating enzyme subunit 1	protein_coding	1929264	.	34.867468
56460	Pkp3	plakophilin 3	protein_coding	1891830	.	0.000000
56461	Kcnip3	Kv channel interacting protein 3, calsenilin	protein_coding	1929258	.	2.224835
56462	Mtch1	mitochondrial carrier 1	protein_coding	1929261	.	94.009765
56463	Snd1	staphylococcal nuclease and tudor domain containing 1	protein_coding	1929266	.	44.565875
56464	Ctsf	cathepsin F	protein_coding	1861434	.	36.026520
56468	Socs5	suppressor of cytokine signaling 5	protein_coding	2385459	.	7.695246
56469	Pias1	protein inhibitor of activated STAT 1	protein_coding	1913125	.	8.652879
56470	Rgs19	regulator of G-protein signaling 19	protein_coding	1915153	.	16.410887
56471	Stmn4	stathmin-like 4	protein_coding	1931224	.	1.736596
56473	Fads2	fatty acid desaturase 2	protein_coding	1930079	.	17.489233
56480	Tbk1	TANK-binding kinase 1	protein_coding	1929658	.	14.476941
56484	Foxo3	forkhead box O3	protein_coding	1890081	.	12.431782
56485	Slc2a5	solute carrier family 2 (facilitated glucose transporter), member 5	protein_coding	1928369	.	0.048939
56486	Gabarap	gamma-aminobutyric acid receptor associated protein	protein_coding	1861742	.	193.441345
56488	Nxt1	NTF2-related export protein 1	protein_coding	1929619	.	11.310264
56489	Ikbke	inhibitor of kappaB kinase epsilon	protein_coding	1929612	.	8.705467
56490	Zbtb20	zinc finger and BTB domain containing 20	protein_coding	1929213	.	0.424468
56491	Vapb	vesicle-associated membrane protein, associated protein B and C	protein_coding	1928744	.	9.927376
56494	Gosr2	golgi SNAP receptor complex member 2	protein_coding	1927204	.	37.271062
56495	Asna1	arsA arsenite transporter, ATP-binding, homolog 1 (bacterial)	protein_coding	1928379	.	24.606219
56496	Tspan6	tetraspanin 6	protein_coding	1926264	.	36.063201
56501	Elf4	E74-like factor 4 (ets domain transcription factor)	protein_coding	1928377	.	12.632522
56503	Ankrd49	ankyrin repeat domain 49	protein_coding	1930842	.	6.857745
56504	Srpk3	serine/arginine-rich protein specific kinase 3	protein_coding	1891338	.	2.067171
56505	Ruvbl1	RuvB-like protein 1	protein_coding	1928760	.	17.395418
56506	Cib2	calcium and integrin binding family member 2	protein_coding	1929293	.	4.924570
56508	Rapgef4	Rap guanine nucleotide exchange factor (GEF) 4	protein_coding	1917723	.	6.060659
56513	Pard6a	par-6 family cell polarity regulator alpha	protein_coding	1927223	.	3.273486
56515	Rnf138	ring finger protein 138	protein_coding	1929211	.	6.984818
56516	Rbms2	RNA binding motif, single stranded interacting protein 2	protein_coding	1861776	.	17.223618
56517	Slc22a21	solute carrier family 22 (organic cation transporter), member 21	protein_coding	1929481	.	6.538063
56520	Nme4	NME/NM23 nucleoside diphosphate kinase 4	protein_coding	1931148	.	8.565695
56522	Papolb	poly (A) polymerase beta (testis specific)	protein_coding	1932115	.	0.057206
56523	Pmfbp1	polyamine modulated factor 1 binding protein 1	protein_coding	1930136	.	0.009711
56524	Mpp6	membrane protein, palmitoylated 6 (MAGUK p55 subfamily member 6)	protein_coding	1927340	.	17.073782
56525	Zfp235	zinc finger protein 235	protein_coding	1929117	.	3.375899
56526	Sept6	septin 6	protein_coding	1888939	.	9.364286
56527	Mast1	microtubule associated serine/threonine kinase 1	protein_coding	1861901	.	0.064454
56529	Sec11a	SEC11 homolog A, signal peptidase complex subunit	protein_coding	1929464	.	50.321293
56530	Cnpy2	canopy FGF signaling regulator 2	protein_coding	1928477	.	88.022132
56531	Ylpm1	YLP motif containing 1	protein_coding	1926195	.	4.933403
56532	Ripk3	receptor-interacting serine-threonine kinase 3	protein_coding	2154952	.	6.347525
56533	Rgs17	regulator of G-protein signaling 17	protein_coding	1927469	.	0.032064
56534	Hspb3	heat shock protein 3	protein_coding	1928479	.	1.037575
56535	Pex3	peroxisomal biogenesis factor 3	protein_coding	1929646	.	9.737998
56538	Klk11	kallikrein related-peptidase 11	protein_coding	1929977	.	0.000000
56541	Habp4	hyaluronic acid binding protein 4	protein_coding	1891713	.	13.069177
56542	Ick	intestinal cell kinase	protein_coding	1934157	.	9.439260
56543	Kcnd3	potassium voltage-gated channel, Shal-related family, member 3	protein_coding	1928743	.	0.069451
56544	Vmn2r1	vomeronasal 2, receptor 1	protein_coding	3645892	.	0.161395
56546	Sec1	secretory blood group 1	protein_coding	1928893	.	0.609760
56550	Ube2d2a	ubiquitin-conjugating enzyme E2D 2A	protein_coding	1930715	.	34.024184
56551	Txn2	thioredoxin 2	protein_coding	1929468	.	44.747967
56554	Raet1d	retinoic acid early transcript delta	protein_coding	1861032	.	1.345995
56612	Pfdn5	prefoldin 5	protein_coding	1928753	.	130.052246
56613	Rps6ka4	ribosomal protein S6 kinase, polypeptide 4	protein_coding	1930076	.	14.117146
56615	Mgst1	microsomal glutathione S-transferase 1	protein_coding	1913850	.	2.711422
56619	Clec4e	C-type lectin domain family 4, member e	protein_coding	1861232	.	0.084800
56620	Clec4n	C-type lectin domain family 4, member n	protein_coding	1861231	.	2.637479
56622	Adam21	a disintegrin and metallopeptidase domain 21	protein_coding	1861229	.	0.027792
56626	Poll	polymerase (DNA directed), lambda	protein_coding	1889000	.	3.404520
56629	Dnase2b	deoxyribonuclease II beta	protein_coding	1913283	.	0.023304
56631	Trim17	tripartite motif-containing 17	protein_coding	1861440	.	3.452468
56632	Sphk2	sphingosine kinase 2	protein_coding	1861380	.	4.668498
56636	Fgf21	fibroblast growth factor 21	protein_coding	1861377	.	0.000000
56637	Gsk3b	glycogen synthase kinase 3 beta	protein_coding	1861437	.	10.134265
56640	Klk4	kallikrein related-peptidase 4 (prostase, enamel matrix, prostate)	protein_coding	1861379	.	0.000000
56642	Ankrd2	ankyrin repeat domain 2 (stretch responsive muscle)	protein_coding	1861447	.	2.036392
56643	Slc15a1	solute carrier family 15 (oligopeptide transporter), member 1	protein_coding	1861376	.	0.037926
56644	Clec7a	C-type lectin domain family 7, member a	protein_coding	1861431	.	0.323462
56690	Mlycd	malonyl-CoA decarboxylase	protein_coding	1928485	.	6.736512
56692	Lamtor3	late endosomal/lysosomal adaptor, MAPK and MTOR activator 3	protein_coding	1929467	.	26.884804
56693	Crtap	cartilage associated protein	protein_coding	1891221	.	131.446884
56695	Pnkd	paroxysmal nonkinesiogenic dyskinesia	protein_coding	1930773	.	20.528631
56696	Gpr132	G protein-coupled receptor 132	protein_coding	1890220	.	0.407634
56697	Akap10	A kinase (PRKA) anchor protein 10	protein_coding	1890218	.	4.658470
56698	Phax	phosphorylated adaptor for RNA export	protein_coding	1891839	.	15.327646
56699	Cdc42ep4	CDC42 effector protein (Rho GTPase binding) 4	protein_coding	1929760	.	16.832934
56700	Glmp	glycosylated lysosomal membrane protein	protein_coding	1913318	.	34.708103
56702	H1f5	histone cluster 1, H1b	protein_coding	1861461	.	0.059670
56703	Pigo	phosphatidylinositol glycan anchor biosynthesis, class O	protein_coding	1861452	.	4.578748
56705	Ranbp9	RAN binding protein 9	protein_coding	1928741	.	10.867119
56706	Ccnl1	cyclin L1	protein_coding	1922664	.	19.283065
56707	Zfp111	zinc finger protein 111	protein_coding	1929114	.	2.109708
56708	Clcf1	cardiotrophin-like cytokine factor 1	protein_coding	1930088	.	6.011590
56709	Dnajb12	DnaJ heat shock protein family (Hsp40) member B12	protein_coding	1931881	.	12.111512
56710	Brinp1	bone morphogenic protein/retinoic acid inducible neural specific 1	protein_coding	1928478	.	0.771601
56711	Plag1	pleiomorphic adenoma gene 1	protein_coding	1891916	.	2.514123
56715	Rabgef1	RAB guanine nucleotide exchange factor (GEF) 1	protein_coding	1929459	.	8.958113
56716	Mlst8	MTOR associated protein, LST8 homolog (S. cerevisiae)	protein_coding	1929514	.	6.298601
56717	Mtor	mechanistic target of rapamycin kinase	protein_coding	1928394	.	5.893021
56722	Litaf	LPS-induced TN factor	protein_coding	1929512	.	18.247568
56724	Cript	cysteine-rich PDZ-binding protein	protein_coding	1929655	.	16.247467
56726	Sh3bgrl	SH3-binding domain glutamic acid-rich protein like	protein_coding	1930849	.	43.054165
56734	Tulp2	tubby-like protein 2	protein_coding	1861600	.	0.085538
56735	Krt71	keratin 71	protein_coding	1861586	.	0.000000
56736	Rnf14	ring finger protein 14	protein_coding	1929668	.	22.522586
56737	Alg2	asparagine-linked glycosylation 2 (alpha-1,3-mannosyltransferase)	protein_coding	1914731	.	15.131445
56738	Mocs1	molybdenum cofactor synthesis 1	protein_coding	1928904	.	5.529108
56739	Rec8	REC8 meiotic recombination protein	protein_coding	1929645	.	0.229464
56741	Igdcc4	immunoglobulin superfamily, DCC subclass, member 4	protein_coding	1858497	.	29.426582
56742	Psrc1	proline/serine-rich coiled-coil 1	protein_coding	1913099	.	7.711018
56743	Lat2	linker for activation of T cells family, member 2	protein_coding	1926479	.	2.160596
56744	Pf4	platelet factor 4	protein_coding	1888711	.	41.829247
56745	C1qtnf1	C1q and tumor necrosis factor related protein 1	protein_coding	1919254	.	22.110451
56747	Sez6l	seizure related 6 homolog like	protein_coding	1935121	.	0.330391
56748	Nfu1	NFU1 iron-sulfur cluster scaffold	protein_coding	1913290	.	15.475809
56749	Dhodh	dihydroorotate dehydrogenase	protein_coding	1928378	.	5.356309
56752	Aldh9a1	aldehyde dehydrogenase 9, subfamily A1	protein_coding	1861622	.	14.174294
56753	Tacstd2	tumor-associated calcium signal transducer 2	protein_coding	1861606	.	0.022603
56758	Mbnl1	muscleblind like splicing factor 1	protein_coding	1928482	.	33.520996
56760	Clec1b	C-type lectin domain family 1, member b	protein_coding	1913287	.	0.000000
56771	Med20	mediator complex subunit 20	protein_coding	1929648	.	10.659004
56772	Mllt11	myeloid/lymphoid or mixed-lineage leukemia; translocated to, 11	protein_coding	1929671	.	5.317117
56773	Chst5	carbohydrate (N-acetylglucosamine 6-O) sulfotransferase 5	protein_coding	1931825	.	0.868719
56784	Ralgapa1	Ral GTPase activating protein, alpha subunit 1	protein_coding	1931050	.	8.207409
56786	Tmem9b	TMEM9 domain family, member B	protein_coding	1915254	.	16.915939
56787	Ascl3	achaete-scute family bHLH transcription factor 3	protein_coding	1928820	.	0.000000
56788	Scube2	signal peptide, CUB domain, EGF-like 2	protein_coding	1928765	.	4.329470

56790	NM_001358015,NM_001358017,NM_019995,NR_152184,NR_152185,NR_152186,XM_006501801,XM_006501802,XM_006501803,XM_006501804,XM_006501805,XM_006501806,XM_006501807,XM_006501808,XM_006501809,XM_006501810,XM_006501811,XM_006501812,XM_006501813,XM_006501814,XM_006501815,XM_030252729,XM_030252730,XM_030252731,XM_030252732,XM_030252733,XM_030252734,XR_001783729,XR_001783730,XR_003954363,XR_003954364,XR_003954365,XR_003954366,XR_003954367,XR	Supt20	SPT20 SAGA complex component	protein_coding	NP_001344944.1;NP_001344946.1;NP_064379.1;XP_006501864.1;XP_006501865.1;XP_006501866.1;XP_006501867.1;XP_006501868.1;XP_006501869.1;XP_006501870.1;XP_006501871.1;XP_006501872.1;XP_006501873.1;XP_006501874.1;XP_006501875.1;XP_006501876.1;XP_006501877.1;XP_006501878.1;XP_030108589.1;XP_030108590.1;XP_030108591.1;XP_030108592.1;XP_030108593.1;XP_030108594.1	1929651	.	27.691932	[illegible]
56791	NM_019949	Ube2l6	ubiquitin-conjugating enzyme E2L 6	protein_coding	NP_064333.2	1914500	.	9.852897	[illegible]
56792	NM_001310639,NM_019992,XM_006535160,XR_003955680	Stap1	signal transducing adaptor family member 1	protein_coding	NP_001297568.1;NP_064376.1;XP_006535223.1	1926193	.	0.313434	[illegible]
56794	NM_019975	Hacl1	2-hydroxyacyl-CoA lyase 1	protein_coding	NP_064359.2	1929657	.	3.849519	[illegible]
56795	NM_019968,XM_006517306,XM_011244544	Arl10	ADP-ribosylation factor-like 10	protein_coding	NP_064352.2;XP_006517369.1;XP_011242846.1	1930788	.	7.305795	[illegible]
56802	NR_002846	Gnasas1	GNAS antisense RNA 1	antisense_RNA	.	1861674	.	0.580917	[illegible]
56805	NM_001079513,NM_020256	Zbtb33	zinc finger and BTB domain containing 33	protein_coding	NP_001072981.1;NP_064652.2	1927290	.	5.747762	[illegible]
56807	NM_001301634,NM_001301635,NM_020270,XM_030244475	Scamp5	secretory carrier membrane protein 5	protein_coding	NP_001288563.1;NP_001288564.1;NP_064666.1;XP_030100335.1	1928948	.	7.474629	[illegible]
56808	NM_001174047,NM_001174048,NM_001174049,NM_001174050,NM_020263,XM_011242899,XM_017313496,XM_017313497	Cacna2d2	calcium channel, voltage-dependent, alpha 2/delta subunit 2	protein_coding	NP_001167518.1;NP_001167519.1;NP_001167520.1;NP_001167521.1;NP_064659.2;XP_011241201.1;XP_017168985.1;XP_017168986.1	1929813	.	1.250510	[illegible]
56809	NM_001122992,NM_020273,XM_006539078,XM_006539079,XM_006539080,XM_017320332	Gmeb1	glucocorticoid modulatory element binding protein 1	protein_coding	NP_001116464.1;NP_064669.2;XP_006539141.1;XP_006539142.1;XP_006539143.1;XP_017175821.1	2135604	.	5.131262	[illegible]
56811	NM_020265,XM_006501818	Dkk2	dickkopf WNT signaling pathway inhibitor 2	protein_coding	NP_064661.2;XP_006501881.1	1890663	.	8.201695	[illegible]
56812	NM_001159883,NM_001159884,NM_001159885,NM_020266,NM_178055,XM_006496511,XR_001785224	Dnajb2	DnaJ heat shock protein family (Hsp40) member B2	protein_coding	NP_001153355.1;NP_001153356.1;NP_001153357.1;NP_064662.2;NP_835156.1;XP_006496574.1	1928739	.	7.716888	[illegible]
56838	NM_020279,XM_006517727,XM_011244684,XM_011244685,XM_011244686,XM_017315550	Ccl28	chemokine (C-C motif) ligand 28	protein_coding	NP_064675.1;XP_006517790.1;XP_011242986.1;XP_011242987.1;XP_011242988.1;XP_017171039.1	1861731	.	0.339382	[illegible]
56839	NM_020278,NR_157558,NR_157559	Lgi1	leucine-rich repeat LGI family, member 1	protein_coding	NP_064674.1	1861691	.	0.464050	[illegible]
56843	NM_020277,XM_006508645,XM_006508646,XM_030242820,XR_003946530	Trpm5	transient receptor potential cation channel, subfamily M, member 5	protein_coding	NP_064673.2;XP_006508708.1;XP_006508709.1;XP_030098680.1	1861718	.	2.889051	[illegible]
56844	NM_001115085,NM_020285,NM_138631,XM_006508648,XM_017322385,XM_030242821	Tssc4	tumor-suppressing subchromosomal transferable fragment 4	protein_coding	NP_001108557.1;NP_064681.1;NP_619537.1;XP_006508711.1;XP_017177874.1;XP_030098681.1	1861712	.	16.502598	[illegible]
56846	NM_021546,XM_011239712,XM_011239713,XM_030251901,XM_030251902,XR_001783155,XR_001783156,XR_001783157	Necab3	N-terminal EF-hand calcium binding protein 3	protein_coding	NP_067521.2;XP_011238014.1;XP_011238015.1;XP_030107761.1;XP_030107762.1	1861721	.	0.949279	[illegible]
56847	NM_053080	Aldh1a3	aldehyde dehydrogenase family 1, subfamily A3	protein_coding	NP_444310.3	1861722	.	3.746207	[illegible]
56856	NM_020287	Insm2	insulinoma-associated 2	protein_coding	NP_064683.2	1930787	.	0.000000	[illegible]
56857	NM_001145960,NM_020258	Slc37a2	solute carrier family 37 (glycerol-3-phosphate transporter), member 2	protein_coding	NP_001139432.1;NP_064654.3	1929693	.	11.752676	[illegible]
56860	NM_020290	Olfr690	olfactory receptor 690	protein_coding	NP_064686.2	3030524	.	0.000000	[illegible]
56863	NM_020293	Cldn9	claudin 9	protein_coding	NP_064689.2	1913100	.	0.048111	[illegible]
56869	NM_020262,XM_030242822,XM_030242823,XM_030242825	Zfp109	zinc finger protein 109	protein_coding	NP_064658.1;XP_030098682.1;XP_030098683.1;XP_030098685.1	1929099	.	1.028228	[illegible]
56872	NM_020264	Pate4	prostate and testis expressed 4	protein_coding	NP_064660.2	1930790	.	0.333347	[illegible]
56873	NM_020295,XM_006535764,XM_006535765,XM_006535767,XM_006535768,XM_006535770,XM_006535771,XM_006535772,XM_011249782,XM_011249783,XM_017321034,XM_030254722,XM_030254723	Lmbr1	limb region 1	protein_coding	NP_064691.2;XP_006535827.1;XP_006535828.1;XP_006535830.1;XP_006535831.1;XP_006535833.1;XP_006535834.1;XP_006535835.1;XP_011248084.1;XP_011248085.1;XP_017176523.1;XP_030110582.1;XP_030110583.1	1861746	.	2.909567	[illegible]
56874	NM_001289757,NM_021470,NR_110371,XM_006535775,XM_006535776,XM_006535777,XM_006535778,XM_006535779,XM_006535780,XM_006535781,XM_006535782,XM_017321036,XM_030254724,XM_030254725,XM_030254726,XM_030254727,XM_030254728	Rnf32	ring finger protein 32	protein_coding	NP_001276686.1;NP_067445.3;XP_006535838.1;XP_006535839.1;XP_006535840.1;XP_006535841.1;XP_006535842.1;XP_006535843.1;XP_006535844.1;XP_006535845.1;XP_017176525.1;XP_030110584.1;XP_030110585.1;XP_030110586.1;XP_030110587.1;XP_03011058	1861747	.	0.062963	[illegible]
56876	NM_001039386,NM_001039387,NM_001177654,NM_001177655,NM_020276,XM_006498200,XM_006498201,XM_006498202,XM_006498203,XM_030251903,XM_030251904,XM_030251905,XM_030251906	Nsmf	NMDA receptor synaptonuclear signaling and neuronal migration factor	protein_coding	NP_001034475.1;NP_001034476.1;NP_001171125.1;NP_001171126.1;NP_064672.2;XP_006498263.1;XP_006498264.1;XP_006498265.1;XP_006498266.1;XP_030107763.1;XP_030107764.1;XP_030107765.1;XP_030107766.1	1861755	.	25.329796	[illegible]
56878	NM_001141931,NM_001141932,NM_020296,XM_006499940,XM_006499942,XM_017319146,XM_030251907,XM_030251908,XM_030251909,XM_030251910	Rbms1	RNA binding motif, single stranded interacting protein 1	protein_coding	NP_001135403.1;NP_001135404.1;NP_064692.2;XP_006500003.1;XP_006500005.1;XP_017174635.1;XP_030107767.1;XP_030107768.1;XP_030107769.1;XP_030107770.1	1861774	.	29.514198	[illegible]
57014	NM_020274	Htr3b	5-hydroxytryptamine (serotonin) receptor 3B	protein_coding	NP_064670.1	1861899	.	0.000000	[illegible]
57028	NM_020271,XR_003951417	Pdxp	pyridoxal (pyridoxine, vitamin B6) phosphatase	protein_coding	NP_064667.2	1919282	.	3.422649	[illegible]
57080	NM_001081462,NM_001081463,NM_001081464,NM_001081465,NM_001081466,NM_001081467,NM_001081468,NM_001081469,NM_001081470,NM_001244936,NM_001347488,NM_020331,XM_006504462,XM_011240885,XM_011240886,XM_011240887,XM_011240888,XM_011240889,XM_011240890,XM_011240891,XM_011240892,XM_011240893,XM_011240894,XM_011240895,XM_011240896,XM_011240897,XM_011240898,XM_011240900,XM_011240901,XM_017321038,XM_017321039,XM_017321040,XM_017321041,XM_017321042,XM_017321043,XM_017321044,XM_017321045,XM_017321046,XM_017321047,XM_030254729,XM_030254730,XM_030254731,XM_030254732,XM_030254733,XM_030254734	Gtf2ird1	general transcription factor II I repeat domain-containing 1	protein_coding	NP_001074931.1;NP_001074932.1;NP_001074933.1;NP_001074934.1;NP_001074935.1;NP_001074936.1;NP_001074937.1;NP_001074938.1;NP_001074939.1;NP_001231865.1;NP_001334417.1;NP_065064.2;XP_006504525.1;XP_011239187.1;XP_011239188.1;XP_011239189.1;XP_011239190.1;XP_011239191.1;XP_011239192.1;XP_011239193.1;XP_011239194.1;XP_011239195.1;XP_011239196.1;XP_011239197.1;XP_011239198.1;XP_011239199.1;XP_011239200.1;XP_011239202.1;XP_011239203.1;XP_017176527.1;XP_017176528.1;XP_017176529.1;XP_017176530.1;XP_017176531.1;XP_017176532.1;XP_017176533.1;XP_017176534.1;XP_017176535.1;XP_017176536.1;XP_030110589.1;XP_030110590.1;XP_030110591.1;XP_030110592.1;XP_030110593.1;XP_030110594.1	1861942	.	12.199301	[illegible]
57138	NM_001355480,NM_001355481,NM_020333	Slc12a5	solute carrier family 12, member 5	protein_coding	NP_001342409.1;NP_001342410.1;NP_065066.2	1862037	.	0.382649	[illegible]
57170	NM_001290508,NM_001290509,NM_020329,NR_110963,XM_006498207,XM_030251911	Dolpp1	dolichyl pyrophosphate phosphatase 1	protein_coding	NP_001277437.1;NP_001277438.1;NP_065062.1;XP_006498270.1;XP_030107771.1	1914093	.	6.346125	[illegible]
57230	NM_020483,XM_006533823,XM_006533824	Sap30bp	SAP30 binding protein	protein_coding	NP_065229.2;XP_006533886.1;XP_006533887.1	1927479	.	11.811269	[illegible]
57246	NM_001205085,NM_020496,NM_194263	Tbx20	T-box 20	protein_coding	NP_001192014.1;NP_065242.1;NP_919239.1	1888496	.	0.053935	[illegible]
57247	NM_020497,XM_006531227,XM_006531228,XM_006531229,XM_017312923,XM_030243697,XR_001778459,XR_003947319,XR_387	Zfp276	zinc finger protein (C2H2 type) 276	protein_coding	NP_065243.2;XP_006531290.1;XP_006531291.1;XP_006531292.1;XP_017168412.1;XP_030099557.1	1888495	.	6.600369	[illegible]
57248	NM_001358005,NM_020498,XM_006521213,XM_030248672	Ly6i	lymphocyte antigen 6 complex, locus I	protein_coding	NP_001344934.1;NP_065244.2;XP_006521276.1;XP_030104532.1	1888480	.	0.000000	[illegible]
57249	NM_001290435,NM_020488,XM_006528173,XM_017318583	Gabrq	gamma-aminobutyric acid (GABA) A receptor, subunit theta	protein_coding	NP_001277364.1;NP_065234.1;XP_006528236.1;XP_017174072.1	1888498	.	0.007596	[illegible]
57253	NM_020502	Tas2r108	taste receptor, type 2, member 108	protein_coding	NP_065248.1	2681210	.	0.000000	[illegible]
57255	NM_020504	Cldn13	claudin 13	protein_coding	NP_065250.1	1913102	.	0.391709	[illegible]
57257	NM_020505,NM_146139,XM_006501819,XM_006501820	Vav3	vav 3 oncogene	protein_coding	NP_065251.2;NP_666251.1;XP_006501882.1;XP_006501883.1	1888518	.	1.703077	[illegible]
57258	NM_001368697,NM_001368698,NM_020506,NR_160870,NR_160871,XM_030247900	Xpo4	exportin 4	protein_coding	NP_001355626.1;NP_001355627.1;NP_065252.1;XP_030103760.1	1888526	.	6.481690	[illegible]
57259	NM_020507	Tob2	transducer of ERBB2, 2	protein_coding	NP_065253.1	1888525	.	10.400464	[illegible]
57260	NM_020490,XM_006519323,XM_006519326	Ltb4r2	leukotriene B4 receptor 2	protein_coding	NP_065236.1;XP_006519386.1;XP_006519389.1	1888501	.	0.134345	[illegible]
57261	NM_001286630,NM_020508,NM_198094,XM_006524683,XM_006524684,XM_006524685,XM_006524686,XM_006524688,XM_006524689,XM_006524690,XM_006524691,XM_006524693,XM_006524695,XM_011246543,XM_017317588,XM_030249922,XM_030249923,XM_030249924,XM_030249925,XM_030249926,XM_030249927,XM_030249928,XM_030249929,XM_030249930,XM_030249931,XM_030249932	Brd4	bromodomain containing 4	protein_coding	NP_001273559.1;NP_065254.3;NP_932762.2;XP_006524746.1;XP_006524747.1;XP_006524748.1;XP_006524749.1;XP_006524751.1;XP_006524752.1;XP_006524753.1;XP_006524754.1;XP_006524756.1;XP_006524758.1;XP_011244845.1;XP_017173077.1;XP_030105782.1;XP_030105783.1;XP_030105784.1;XP_030105785.1;XP_030105786.1;XP_030105787.1;XP_030105788.1;XP_030105789.1;XP_030105790.1;XP_030105791.1;XP_030105792.1	1888520	.	15.885712	[illegible]
57262	NM_020509,XM_030249223	Retnla	resistin like alpha	protein_coding	NP_065255.3;XP_030105083.1	1888504	.	0.075203	[illegible]
57264	NM_001204959,NM_022984	Retn	resistin	protein_coding	NP_001191888.1;NP_075360.1	1888506	.	0.041596	[illegible]
57265	NM_020510	Fzd2	frizzled class receptor 2	protein_coding	NP_065256.1	1888513	.	14.166842	[illegible]
57266	NM_019568,XM_006517307,XM_006517308,XM_011244545	Cxcl14	chemokine (C-X-C motif) ligand 14	protein_coding	NP_062514.2;XP_006517370.1;XP_006517371.1;XP_011242847.1	1888514	.	24.541558	[illegible]
57267	NM_018758,XM_006513905,XM_006513906,XM_006513907,XR_380418	Apba3	amyloid beta (A4) precursor protein-binding, family A, member 3	protein_coding	NP_061228.1;XP_006513968.1;XP_006513969.1;XP_006513970.1	1888527	.	13.498682	[illegible]
57269	NM_001170918,NM_020512	Olfr1507	olfactory receptor 1507	protein_coding	NP_001164389.1;NP_065258.2	3031341	.	0.040075	[illegible]
57274	NM_020516,XM_017316713	Slc16a8	solute carrier family 16 (monocarboxylic acid transporters), member 8	protein_coding	NP_065262.1;XP_017172202.1	1929519	.	0.070154	[illegible]
57275	NM_020517	Lenep	lens epithelial protein	protein_coding	NP_065263.1	1930020	.	3.581347	[illegible]
57276	NM_020518	Vsig2	V-set and immunoglobulin domain containing 2	protein_coding	NP_065264.2	1928009	.	3.042445	[illegible]
57277	NM_020519	Slurp1	secreted Ly6/Plaur domain containing 1	protein_coding	NP_065265.1	1930923	.	0.000000	[illegible]
57278	NM_020486	Bcam	basal cell adhesion molecule	protein_coding	NP_065232.1	1929940	.	9.332466	[illegible]
57279	NM_020520	Slc25a20	solute carrier family 25 (mitochondrial carnitine/acylcarnitine translocase), member 20	protein_coding	NP_065266.1	1928738	.	14.622673	[illegible]
57294	NM_027015,NR_033727	Rps27	ribosomal protein S27	protein_coding	NP_081291.1	1888676	.	685.708784	[illegible]
57295	NM_133788,NR_037908,NR_161302,NR_161303,NR_161304,NR_161306,NR_161307	Icmt	isoprenylcysteine carboxyl methyltransferase	protein_coding	NP_598549.1	1888594	.	11.603486	[illegible]
57296	NM_026545	Psmd8	proteasome (prosome, macropain) 26S subunit, non-ATPase, 8	protein_coding	NP_080821.3	1888669	.	54.903931	[illegible]
57312	NM_020560,XM_017312924,XR_003947320	Mrps31	mitochondrial ribosomal protein S31	protein_coding	NP_065585.1;XP_017168413.1	1913153	.	7.058866	[illegible]
57314	NM_020580,XM_006499946	Nelfcd	negative elongation factor complex member C/D, Th1l	protein_coding	NP_065605.2;XP_006500009.1	1926424	.	14.135252	[illegible]
57315	NM_020603,XM_006524697	Wdr46	WD repeat domain 46	protein_coding	NP_065628.1;XP_006524760.1	1931871	.	8.286889	[illegible]
57316	NM_001330649,NM_020558,XM_030246193	C1d	C1D nuclear receptor co-repressor	protein_coding	NP_001317578.1;NP_065583.2;XP_030102053.1	1927354	.	10.414558	[illegible]
57317	NM_001369308,NM_001369309,NM_001369310,NM_001369311,NM_001369312,NM_020587,XM_017320333,XM_017320335,XM_030253687	Srsf4	serine/arginine-rich splicing factor 4	protein_coding	NP_001356237.1;NP_001356238.1;NP_001356239.1;NP_001356240.1;NP_001356241.1;NP_065612.2;XP_017175822.1;XP_017175824.1;XP_030109547.1	1890577	.	26.135265	[illegible]
57319	NM_020561	Smpdl3a	sphingomyelin phosphodiesterase, acid-like 3A	protein_coding	NP_065586.3	1931437	.	25.737450	[illegible]
57320	NM_020569	Park7	Parkinson disease (autosomal recessive, early onset) 7	protein_coding	NP_065594.2	2135637	.	61.078426	[illegible]
57321	NM_020584	Terf2ip	telomeric repeat binding factor 2, interacting protein	protein_coding	NP_065609.2	1929871	.	6.725893	[illegible]
57329	NM_020595	Otor	otoraplin	protein_coding	NP_065620.1	1888678	.	0.769274	[illegible]
57330	NM_031408,XM_006504607,XM_006504608,XM_006504609,XM_011240950,XM_011240952,XM_011240953,XM_030254735,XM_030254736,XM_030254737,XM_030254738	Gigyf1	GRB10 interacting GYF protein 1	protein_coding	NP_113596.2;XP_006504670.1;XP_006504671.1;XP_006504672.1;XP_011239252.1;XP_011239254.1;XP_011239255.1;XP_030110595.1;XP_030110596.1;XP_030110597.1;XP_030110598.1	1888677	.	13.862921	[illegible]
57339	NM_020604,XM_011238396,XM_011238397,XM_011238398,XM_011238399,XR_001785237	Jph1	junctophilin 1	protein_coding	NP_065629.1;XP_011236698.1;XP_011236699.1;XP_011236700.1;XP_011236701.1	1891495	.	2.727236	[illegible]
57340	NM_020605	Jph3	junctophilin 3	protein_coding	NP_065630.1	1891497	.	0.046512	[illegible]
57342	NM_020606,XM_006508050,XM_006508051	Parva	parvin, alpha	protein_coding	NP_065631.3;XP_006508113.1;XP_006508114.1	1931144	.	41.939255	[illegible]
57344	NM_020577,XM_006527235,XM_011247302,XM_030251005,XR_003952795	As3mt	arsenic (+3 oxidation state) methyltransferase	protein_coding	NP_065602.2;XP_006527298.1;XP_011245604.1;XP_030106865.1	1929882	.	5.351682	[illegible]
57349	NM_023785	Ppbp	pro-platelet basic protein	protein_coding	NP_076274.1	1888712	.	1.177696	[illegible]
57354	NM_020608,XM_006524699,XM_006524702,XM_006524703,XM_011246544,XM_011246545,XM_017317589	Cramp1l	cramped chromatin regulator homolog 1	protein_coding	NP_065633.2;XP_006524762.1;XP_006524765.1;XP_006524766.1;XP_011244846.1;XP_011244847.1;XP_017173078.1	1930190	.	8.109053	[illegible]
57355	NM_001040700,XM_006508052	BC051019	cDNA sequence BC051019	protein_coding	NP_001035790.1;XP_006508115.1	1928824	.	0.000000	[illegible]
57357	NM_020611,XM_006535162	Srd5a3	steroid 5 alpha-reductase 3	protein_coding	NP_065636.2;XP_006535225.1	1930252	.	8.336184	[illegible]
57370	NM_020579	B4galt3	UDP-Gal:betaGlcNAc beta 1,4-galactosyltransferase, polypeptide 3	protein_coding	NP_065604.2	1928767	.	13.039620	[illegible]
57373	NM_020616,XM_006508055,XM_006508057,XM_011241869,XM_011241870,XM_030242826	Akip1	A kinase (PRKA) interacting protein 1	protein_coding	NP_065641.1;XP_006508118.1;XP_006508120.1;XP_011240171.1;XP_011240172.1;XP_030098686.1	3041226	.	7.313763	[illegible]
57376	NM_020618	Smarce1	SWI/SNF related, matrix associated, actin dependent regulator of chromatin, subfamily e, member 1	protein_coding	NP_065643.1	1927347	.	34.489944	[illegible]
57377	NM_020619,XM_006506424	Mogs	mannosyl-oligosaccharide glucosidase	protein_coding	NP_065644.2;XP_006506487.1	1929872	.	23.418401	[illegible]
57385	NM_020621,XM_006528175,XM_006528176,XM_006528177,XM_006528178	P2ry4	pyrimidinergic receptor P2Y, G-protein coupled, 4	protein_coding	NP_065646.1;XP_006528238.1;XP_006528239.1;XP_006528240.1;XP_006528241.1	1926594	.	0.012849	[illegible]
57390	NM_020576	Psors1c2	psoriasis susceptibility 1 candidate 2 (human)	protein_coding	NP_065601.2	1930025	.	0.215624	[illegible]
57394	NM_001313719,NM_020626	Cltrn	collectrin, amino acid transport regulator	protein_coding	NP_001300648.1;NP_065651.1	1926234	.	0.000000	[illegible]
57423	NM_020582	Atp5j2	ATP synthase, H+ transporting, mitochondrial F0 complex, subunit F2	protein_coding	NP_065607.1	1927558	.	116.040314	[illegible]
57429	NM_020564,XM_006531230,XM_006531231,XM_006531232,XM_011248458,XM_011248459,XM_030243699,XM_030243700,XR_003947321	Sult5a1	sulfotransferase family 5A, member 1	protein_coding	NP_065589.2;XP_006531293.1;XP_006531294.2;XP_006531295.2;XP_011246760.1;XP_011246761.1;XP_030099559.1;XP_030099560.1	1931463	.	4.923590	[illegible]
57431	NM_001356999,NM_001357000,NM_001357001,NM_020566,XM_006527237,XM_011247304,XM_030251006,XM_030251007	Dnajc4	DnaJ heat shock protein family (Hsp40) member C4	protein_coding	NP_001343928.1;NP_001343929.1;NP_001343930.1;NP_065591.1;XP_006527300.1;XP_011245606.1;XP_030106866.1;XP_030106867.1	1927346	.	12.272366	[illegible]
57432	NM_020594,XM_006499947	Zc3h8	zinc finger CCCH type containing 8	protein_coding	NP_065619.2;XP_006500010.1	1930128	.	3.463063	[illegible]
57434	NM_020570	Xrcc2	X-ray repair complementing defective repair in Chinese hamster cells 2	protein_coding	NP_065595.2	1927345	.	2.065831	[illegible]
57435	NM_020568,XM_006524705,XM_011246546,XM_011246547,XM_011246548,XM_011246549,XM_011246550,XM_011246551,XM_011246552,XM_017317591,XR_003952169	Plin4	perilipin 4	protein_coding	NP_065593.2;XP_006524768.1;XP_011244848.1;XP_011244849.1;XP_011244850.1;XP_011244851.1;XP_011244852.1;XP_011244853.1;XP_011244854.1;XP_017173080.1	1929709	.	1.197824	[illegible]
57436	NM_020590	Gabarapl1	gamma-aminobutyric acid (GABA) A receptor-associated protein-like 1	protein_coding	NP_065615.1	1914980	.	19.624268	[illegible]
57437	NM_001042484,NM_020585,XM_006509179	Golga7	golgi autoantigen, golgin subfamily a, 7	protein_coding	NP_001035949.1;NP_065610.2;XP_006509242.1	1931029	.	34.317758	[illegible]
57438	NM_020575	March7	membrane-associated ring finger (C3HC4) 7	protein_coding	NP_065600.1	1931053	.	20.702246	[illegible]
57439	NM_001042485,NM_020588,NR_151737,XR_003956268,XR_003956269	Tmem183a	transmembrane protein 183A	protein_coding	NP_001035950.1;NP_065613.1	1914729	.	15.621480	[illegible]
57440	NM_020578	Ehd3	EH-domain containing 3	protein_coding	NP_065603.2	1928900	.	6.314518	[illegible]
57441	NM_020567,XM_006516723	Gmnn	geminin	protein_coding	NP_065592.1;XP_006516786.1	1927344	.	6.188822	[illegible]
57442	NM_001190869,NM_001190870,NM_001190871,NM_001190950,NM_001360466,NM_001360467,NM_020574,XM_006508059,XM_006508062,XM_017322388	Kcne3	potassium voltage-gated channel, Isk-related subfamily, gene 3	protein_coding	NP_001177798.1;NP_001177799.1;NP_001177800.1;NP_001177879.1;NP_001347395.1;NP_001347396.1;NP_065599.1;XP_006508122.1;XP_006508125.1;XP_017177877.1	1891124	.	1.473847	[illegible]
57443	NM_001355504,NM_001355505,NM_001355506,NM_001355507,NM_020593,NM_212433,NR_149726	Fbxo3	F-box protein 3	protein_coding	NP_001342433.1;NP_001342434.1;NP_001342435.1;NP_001342436.1;NP_065618.1;NP_997598.1	1929084	.	16.627562	[illegible]
57444	NM_001113527,NM_001291220,NM_001291221,NM_020583,XM_006541026,XM_006541028,XM_030242827,XM_030242828,XM_030242829	Isg20	interferon-stimulated protein	protein_coding	NP_001106999.1;NP_001278149.1;NP_001278150.1;NP_065608.2;XP_006541089.1;XP_006541091.1;XP_030098687.1;XP_030098688.1;XP_030098689.1	1928895	.	2.044677	[illegible]
57738	NM_001145899,NM_021301,XM_006522428,XM_006522430,XR_003951808	Slc15a2	solute carrier family 15 (H+/peptide transporter), member 2	protein_coding	NP_001139371.1;NP_067276.2;XP_006522491.1;XP_006522493.1	1890457	.	3.225482	[illegible]
57740	NM_001162540,NM_021302,XM_006536216	Stk32c	serine/threonine kinase 32C	protein_coding	NP_001156012.1;NP_067277.2;XP_006536279.1	2385336	.	1.516095	[illegible]
57741	NM_001369270,NM_021303,NR_161236,XM_030253688	Noc2l	NOC2 like nucleolar associated transcriptional repressor	protein_coding	NP_001356199.1;NP_067278.2;XP_030109548.1	1931051	.	25.882140	[illegible]
57742	NR_003522	Abhd1	abhydrolase domain containing 1	transcribed_pseudogene	.	1931013	.	0.443732	[illegible]
57743	NM_001356411,NM_021305,XM_006497517,XM_017319150,XM_017319152,XM_030251912,XR_373943	Sec61a2	Sec61, alpha subunit 2 (S. cerevisiae)	protein_coding	NP_001343340.1;NP_067280.1;XP_006497580.1;XP_017174639.1;XP_017174641.1;XP_030107772.1	1931071	.	16.718246	[illegible]
57745	NM_021307	Zfp112	zinc finger protein 112	protein_coding	NP_067282.2	1929115	.	1.624346	[illegible]
57746	NM_001364321,NM_021308,XM_006519329	Piwil2	piwi-like RNA-mediated gene silencing 2	protein_coding	NP_001351250.1;NP_067283.1;XP_006519392.1	1930036	.	0.281701	[illegible]
57748	NM_021310	Jmy	junction-mediating and regulatory protein	protein_coding	NP_067285.2	1913096	.	5.922293	[illegible]
57749	NM_021311,XM_006504318	Piwil1	piwi-like RNA-mediated gene silencing 1	protein_coding	NP_067286.1;XP_006504381.1	1928897	.	0.000000	[illegible]
57750	NM_001199060,NM_001199061,NM_021312,XM_011238563,XM_017321861,XM_030255693	Wdr12	WD repeat domain 12	protein_coding	NP_001185989.1;NP_001185990.1;NP_067287.1;XP_011236865.1;XP_017177350.1;XP_030111553.1	1927241	.	4.694886	[illegible]
57751	NM_001305230,NM_021313,XM_006496170,XM_030255695	Rnf25	ring finger protein 25	protein_coding	NP_001292159.1;NP_067288.2;XP_006496233.1;XP_030111555.1	1890215	.	19.793258	[illegible]
57752	NM_001004468,NM_001347637,NM_021314,NM_206856,XM_006508064,XM_006508065,XM_006508066,XM_006508067,XM_006508087,XM_006508088,XM_006508089,XM_006508090,XM_011241872,XM_011241873,XM_011241874,XM_011241875,XM_011241876,XM_017322390,XM_017322391,XM_017322392,XM_017322393,XM_017322394,XM_017322395,XM_017322396,XM_017322397,XM_017322398,XM_017322400,XM_017322401,XM_017322403,XM_017322404,XM_017322405,XM_017322406,XM_017322407,XM_017322408,XM_017322409,XM_017322410,XM_017322411,XM_017322412,XM_017322413,XM_017322414,XM_030242830,XM_030242831,XM_030242832,XM_030242833,XM_030242834,XM_030242835,XM_030242836,XM_030242837,XM_030242838	Tacc2	transforming, acidic coiled-coil containing protein 2	protein_coding	NP_001004468.1;NP_001334566.1;NP_067289.2;NP_996738.2;XP_006508127.1;XP_006508128.1;XP_006508129.1;XP_006508130.1;XP_006508150.1;XP_006508151.1;XP_006508152.1;XP_006508153.1;XP_011240174.1;XP_011240175.1;XP_011240176.1;XP_011240177.1;XP_011240178.1;XP_017177879.1;XP_017177880.1;XP_017177881.1;XP_017177882.1;XP_017177883.1;XP_017177884.1;XP_017177885.1;XP_017177886.1;XP_017177887.1;XP_017177889.1;XP_017177890.1;XP_017177892.1;XP_017177893.1;XP_017177894.1;XP_017177895.1;XP_017177896.1;XP_017177897.1;XP_017177898.1;XP_017177899.1;XP_017177900.1;XP_017177901.1;XP_017177902.1;XP_017177903.1;XP_030098690.1;XP_030098691.1;XP_030098692.1;XP_030098693.1;XP_030098694.1;XP_030098695.1;XP_030098696.1;XP_030098697.1;XP_030098698.1	1928899	.	7.760748	[illegible]
57753	NM_021315,XR_386499	Noc3l	NOC3 like DNA replication regulator	protein_coding	NP_067290.2	1932610	.	4.802063	[illegible]
57754	NM_001360485,NM_021316	Cend1	cell cycle exit and neuronal differentiation 1	protein_coding	NP_001347414.1;NP_067291.1	1929898	.	0.042182	[illegible]
57755	NM_021317	Dnajb7	DnaJ heat shock protein family (Hsp40) member B7	protein_coding	NP_067292.2	1914012	.	0.000000	[illegible]
57756	NM_001355498,NM_021318,XM_006538125	Fhl5	four and a half LIM domains 5	protein_coding	NP_001342427.1;NP_067293.1;XP_006538188.1	1913192	.	0.310252	[illegible]
57757	NM_001271476,NM_001271477,NM_001271478,NM_001271479,NM_021319,NR_073184,XM_006524710,XM_006524711,XM_006524712,XM_006524713,XM_006524714,XR_003952170	Pglyrp2	peptidoglycan recognition protein 2	protein_coding	NP_001258405.1;NP_001258406.1;NP_001258407.1;NP_001258408.1;NP_067294.2;XP_006524773.1;XP_006524774.1;XP_006524775.1;XP_006524776.1;XP_006524777.1	1928099	.	0.016818	[illegible]
57764	NM_021320	Ntn4	netrin 4	protein_coding	NP_067295.2	1888978	.	1.900519	[illegible]
57765	NM_019507	Tbx21	T-box 21	protein_coding	NP_062380.2	1888984	.	0.116812	[illegible]
57773	NM_021322,XM_006524715,XM_006524716,XM_006524719,XM_006524720,XM_006524723,XM_006524724,XM_006524725,XM_011246553,XM_030249933,XM_030249934,XM_030249935	Wdr4	WD repeat domain 4	protein_coding	NP_067297.2;XP_006524778.1;XP_006524779.1;XP_006524782.1;XP_006524783.1;XP_006524786.1;XP_006524787.1;XP_006524788.1;XP_011244855.1;XP_030105793.1;XP_030105794.1;XP_030105795.	1889002	.	6.212706	[illegible]
57775	NM_001290994,NM_001360501,NM_001360502,NM_021323,NR_153505,NR_153506,NR_153507,NR_153590,XM_011250662,XM_017322416,XM_017322417,XM_017322419,XM_017322420,XM_017322421,XM_017322422,XM_017322423,XM_017322424,XM_017322425,XM_017322427,XM_017322428,XM_030242839,XM_03024284	Usp29	ubiquitin specific peptidase 29	protein_coding	NP_001277923.1;NP_001347430.1;NP_001347431.1;NP_067298.2;XP_011248964.1;XP_017177905.1;XP_017177906.1;XP_017177908.1;XP_017177909.1;XP_017177910.1;XP_017177911.1;XP_017177912.1;XP_017177913.1;XP_017177914.1;XP_017177916.1;XP_017177917.1;XP_030098699.1;XP_030098700.1	1888998	.	4.941434	[illegible]
57776	NM_001001454,NM_001109765,NM_001305822,NM_021324	Ttyh1	tweety family member 1	protein_coding	NP_001001454.2;NP_001103235.1;NP_001292751.1;NP_067299.2	1889007	.	0.394398	[illegible]
57778	NM_001077698,NM_019679,XM_006533837,XM_006533838,XM_006533839,XM_006533840,XM_006533841,XM_006533842,XM_006533843,XM_006533844,XM_006533845	Fmnl1	formin-like 1	protein_coding	NP_001071166.1;NP_062653.2;XP_006533900.1;XP_006533901.1;XP_006533902.1;XP_006533903.1;XP_006533904.1;XP_006533905.1;XP_006533906.1;XP_006533907.1;XP_006533908.1	1888994	.	1.857319	[illegible]
57780	NM_022007	Fxyd7	FXYD domain-containing ion transport regulator 7	protein_coding	NP_071290.1	1889006	.	1.751463	[illegible]
57781	NM_021325	Cd200r1	CD200 receptor 1	protein_coding	NP_067300.1	1889024	.	1.145428	[illegible]
57782	NM_001045482,NM_001359235,NM_021326,XM_006504723	Rbak	RB-associated KRAB zinc finger	protein_coding	NP_001038947.1;NP_001346164.1;NP_067301.1;XP_006504786.1	1927369	.	14.095386	[illegible]
57783	NM_001199275,NM_001199276,NM_001271455,NM_001271456,NM_021327,XM_006533846,XM_006533847,XM_006533848,XM_011249162,XM_011249163,XM_011249164,XM_030246196,XM_030246197,XM_030246198,XM_030246199	Tnip1	TNFAIP3 interacting protein 1	protein_coding	NP_001186204.1;NP_001186205.1;NP_001258384.1;NP_001258385.1;NP_067302.2;XP_006533909.1;XP_006533910.1;XP_006533911.1;XP_011247464.1;XP_011247465.1;XP_011247466.1;XP_030102056.1;XP_030102057.1;XP_030102058.1;XP_030102059.1	1926194	.	8.922732	[illegible]
57784	NM_001360404,NM_021328,XM_006519330,XM_006519332,XM_011245124,XM_030247901,XM_030247902	Bin3	bridging integrator 3	protein_coding	NP_001347333.1;NP_067303.1;XP_006519393.1;XP_006519395.1;XP_011243426.1;XP_030103761.1;XP_030103762.1	1929883	.	12.526686	[illegible]
57785	NM_001285441,NM_001285442,NM_001285443,NM_021329	Rangrf	RAN guanine nucleotide release factor	protein_coding	NP_001272370.1;NP_001272371.1;NP_001272372.1;NP_067304.1	1889073	.	5.697574	[illegible]
57808	NM_001130484,NM_001130485,NM_021338	Rpl35a	ribosomal protein L35A	protein_coding	NP_001123956.1;NP_001123957.1;NP_067313.2	1928894	.	554.244655	[illegible]
57810	NM_021339,XM_006510507,XM_006510508,XM_006510509,XM_006510510,XM_006510511,XM_006510512,XM_006510514,XM_011242581,XM_030244476,XR_379126	Cdon	cell adhesion molecule-related/down-regulated by oncogenes	protein_coding	NP_067314.2;XP_006510570.1;XP_006510571.1;XP_006510572.1;XP_006510573.1;XP_006510574.1;XP_006510575.1;XP_006510577.1;XP_011240883.1;XP_030100336.1	1926387	.	16.426877	[illegible]
57811	NM_001301692,NM_001301694,NM_021340,XM_011245125,XM_017316102	Rgr	retinal G protein coupled receptor	protein_coding	NP_001288621.1;NP_001288623.1;NP_067315.1;XP_011243427.1;XP_017171591.1	1929473	.	0.000000	[illegible]
57813	NM_021028,NR_045642,XM_006531233,XM_006531234,XM_006531235,XM_011248461,XM_017312925,XM_030243701,XR_003947	Tk2	thymidine kinase 2, mitochondrial	protein_coding	NP_066356.3;XP_006531296.1;XP_006531297.1;XP_006531298.1;XP_011246763.1;XP_017168414.1;XP_030099561.1	1913266	.	5.828640	[illegible]
57814	NM_021342	Kcne4	potassium voltage-gated channel, Isk-related subfamily, gene 4	protein_coding	NP_067317.1	1891125	.	16.177584	[illegible]
57815	NM_001163511,NM_001310473,NM_021343,XM_017319672,XM_017319673,XM_030252736	Spata5	spermatogenesis associated 5	protein_coding	NP_001156983.1;NP_001297402.1;NP_067318.2;XP_017175161.1;XP_017175162.1;XP_030108596.1	1927170	.	3.480661	[illegible]
57816	NM_021344,NR_151582	Tesc	tescalcin	protein_coding	NP_067319.2	1930803	.	0.000000	[illegible]
57837	NM_022313	Eral1	Era (G-protein)-like 1 (E. coli)	protein_coding	NP_071708.2	1889295	.	7.563956	[illegible]
57869	NM_178873,XM_006506425	Adck2	aarF domain containing kinase 2	protein_coding	NP_849204.1;XP_006506488.1	1889336	.	5.265629	[illegible]
57874	NM_021345,XR_001778950	Hacd3	3-hydroxyacyl-CoA dehydratase 3	protein_coding	NP_067320.2	1889341	.	14.240596	[illegible]
57875	NM_020581	Angptl4	angiopoietin-like 4	protein_coding	NP_065606.2	1888999	.	55.371578	[illegible]
57890	NM_001034029,NM_001034031,NM_001362204,NM_001362205,NM_145826,XM_030255519	Il17re	interleukin 17 receptor E	protein_coding	NP_001029201.1;NP_001029203.1;NP_001349133.1;NP_001349134.1;NP_665825.2;XP_030111379.1	1889371	.	0.041214	[illegible]
57895	NM_001311112,NM_175098,XM_011241420	Ccdc126	coiled-coil domain containing 126	protein_coding	NP_001298041.1;NP_780307.1;XP_011239722.1	1889376	.	2.888057	[illegible]
57896	NM_001354706,NM_145568	Krcc1	lysine-rich coiled-coil 1	protein_coding	NP_001341635.1;NP_663543.1	1889377	.	12.959329	[illegible]
57905	NM_133934,XM_030255520	Isy1	ISY1 splicing factor homolog	protein_coding	NP_598695.1;XP_030111380.1	1923310	.	14.443619	[illegible]
57908	NM_021346,NM_207671,XM_011246554,XR_385351	Zfp318	zinc finger protein 318	protein_coding	NP_067321.2;NP_997554.2;XP_011244856.1	1889348	.	5.417340	[illegible]
57911	NM_021347,XM_006533850	Gsdma	gasdermin A	protein_coding	NP_067322.1;XP_006533913.1	1889509	.	0.000000	[illegible]
57912	NM_001038708,NM_172395	Cdc42se1	CDC42 small effector 1	protein_coding	NP_001033797.1;NP_765983.1	1889510	.	19.108110	[illegible]

57913	NM_001360523,NM_001360524,NM_022654,NR_153591,XM_011249849,XR_001785592,XR_389958,XR_389959	Pidd1	p53 induced death domain protein 1	protein_coding	NP_001347452.1;NP_001347453.1;NP_073145.1;XP_011248151.1	1889507	.		2.729386	3.335352	3.682226	2.829980	1.773828	3.722414	2.778607	3.000846	7.180363	8.856276	9.911702	7.586173	4.670228	10.197140	7.491593	8.157767	1.261690	440.794612	0.214944246	1.560206368	0.118711121	0.552833566	8.607376	8.941273	8.956505	8.750796	8.788546	8.944150	8.726114	8.318448	8.884495	8.674978	532	447	453	525	436	278	440	392
57914	NM_001164735,NM_001310694,NM_016715,XM_011249547,XR_003955682,XR_003955683,XR_003955684,XR_003955685	Crlf2	cytokine receptor-like factor 2	protein_coding	NP_001158207.1;NP_001297623.1;NP_057924.3;XP_011247849.1	1889506	.		4.391142	4.782629	3.453847	4.488378	3.589788	5.458549	4.089460	4.166725	11.552049	12.699202	9.296956	12.031752	9.451381	14.953093	11.025871	11.327195	1.039872	264.655829	0.193315914	0.291784531	0.770451374	0.944917263	8.020167	8.075255	8.236413	8.041389	8.024057	7.890514	8.063687	7.889072	8.118978	8.026462	334	275	260	207	285	239	257	259
57915	NM_001289514,NM_001310611,NM_019636,XM_006504036,XM_006504037,XM_006504038,XM_011240757,XM_011240758,XM_011240759,XM_030254739,XM_030254740	Tbc1d1	TBC1 domain family, member 1	protein_coding	NP_001276443.1;NP_001297540.1;NP_062610.2;XP_006504099.1;XP_006504100.1;XP_006504101.1;XP_011239059.1;XP_011239060.1;XP_011239061.1;XP_030110599.1;XP_030110600.1	1889508	.		6.259798	6.504706	7.095204	7.635820	5.129156	7.266524	6.769456	7.139495	16.468036	17.271782	19.098651	20.468929	13.504308	19.905835	18.251594	19.408643	1.130588	1434.347793	0.139025656	1.273669068	0.202780723	0.650243326	10.395070	10.571875	10.541969	10.522959	10.556307	10.547769	10.631697	10.177271	10.454646	10.367872	1479	1553	1555	1489	1738	1182	1218	1278
57916	NM_021349,NR_157295,XM_006533851	Tnfrsf13b	tumor necrosis factor receptor superfamily, member 13b	protein_coding	NP_067324.1;XP_006533914.1	1889411	.		0.713225	0.828553	0.850289	0.573261	0.506106	0.425330	0.590171	0.706980	1.876325	2.200036	2.288780	1.536710	1.332501	1.165146	1.591200	1.921918	1.148759	102.968175	0.28127926	0.711306885	0.476894086	0.838117654	6.584320	6.781085	6.774518	6.595460	6.637877	6.794696	6.569309	6.495715	6.771382	6.700273	123	90	96	128	85	76	110	109
58170	NM_021370,XM_006501821,XM_006501823,XM_006501825	Asic5	acid-sensing (proton-gated) ion channel family member 5	protein_coding	NP_067345.1;XP_006501884.1;XP_006501886.1;XP_006501888.1	1929259	.		0.000000	0.000000	0.000000	0.000000	0.019711	0.000000	0.000000	0.000000	0.000000	0.000000	0.000000	0.000000	0.051895	0.000000	0.000000	0.000000	-2.584344	0.222360	3.53133956	-0.387897547	0.698091854	NA	-1.169033	-Inf	-1.725323	-1.725448	-1.725389	-1.725324	-1.725457	-1.718206	-1.725125	-1.725376	0	0	0	0	0	2	0	0
58172	NM_001038625,NM_001362867,NM_021372,XM_006514766	Sertad2	SERTA domain containing 2	protein_coding	NP_001033714.1;NP_001349796.1;NP_067347.2;XP_006514829.1	1931026	.		7.386341	7.511764	7.334222	6.780974	7.202993	7.911988	7.400220	6.690621	19.431702	19.945798	19.742031	18.177387	18.964419	21.674016	19.952239	18.188383	1.058121	1769.068546	0.081096569	1.005028737	0.314883012	0.74371579	10.747637	10.828771	10.863836	10.868570	10.731928	10.795748	10.715572	10.734854	10.801790	10.782732	1852	1991	1686	1734	1721	1887	1566	1760
58175	NM_001177795,NM_001290372,NM_021374,XM_006495554,XM_011238400,XM_011238401	Rgs20	regulator of G-protein signaling 20	protein_coding	NP_001171266.1;NP_001277301.1;NP_067349.2;XP_006495617.1;XP_011236702.1;XP_011236703.1	1929866	.		0.000000	0.000000	0.000000	0.510566	0.190035	0.056391	0.000000	0.056303	0.000000	0.000000	0.000000	1.368643	0.500334	0.154477	0.000000	0.153058	-5.239670	8.642322	1.846157843	-1.294296739	0.195562942	NA	3.858955	1.472410	2.964420	2.920841	2.970948	2.921348	3.217498	3.056118	2.922223	2.921135	5	0	6	0	43	19	0	0
58176	NM_021375,XM_006501826	Rhbg	Rhesus blood group-associated B glycoprotein	protein_coding	NP_067350.2;XP_006501889.1	1927379	.		0.047718	0.056205	0.130843	0.000000	0.000000	0.064131	0.000000	0.000000	0.125535	0.149239	0.352200	0.000000	0.000000	0.175681	0.000000	0.000000	2.053101	3.257175	2.035007625	0.509975804	0.610068413	NA	1.100724	2.127543	1.688999	1.641458	1.641541	1.726341	1.641445	1.641265	1.676909	1.672649	6	0	0	11	0	0	4	4
58178	NM_001252501,NM_001290356,NM_001360457,NM_021377,XM_006527243,XM_006527244,XM_017318265,XM_030251008,XM_030251009	Sorcs1	sortilin-related VPS10 domain containing receptor 1	protein_coding	NP_001239430.1;NP_001277285.1;NP_001347386.1;NP_067352.2;XP_006527306.1;XP_006527307.1;XP_017173754.1;XP_030106868.1;XP_030106869.1	1929666	.		0.573385	0.582238	0.435777	0.969766	0.631923	0.803866	0.593639	0.622769	1.508439	1.546002	1.173010	2.599600	1.663760	2.202102	1.600550	1.692992	-1.077691	191.934305	0.20467541	-0.527386074	0.597925512	0.89348641	7.637224	7.529711	7.613450	7.586069	7.612883	7.388886	7.753637	7.573273	7.553193	7.510099	197	202	204	136	261	214	161	172
58180	NM_178922	Hic2	hypermethylated in cancer 2	protein_coding	NP_849253.2	1929869	.		1.358055	1.396515	1.103411	1.184465	1.612054	1.378646	1.258035	1.329772	3.572720	3.708131	2.970127	3.175132	4.244302	3.776648	3.391875	3.614971	-1.055432	362.585690	0.143030328	-0.544178657	0.586318537	0.889380194	8.540474	8.462838	8.527764	8.501738	8.516476	8.359021	8.410489	8.626913	8.532503	8.502815	365	378	373	294	338	477	330	364
58182	NM_001355655,NM_021381,XM_006506432,XM_006506433	Prokr1	prokineticin receptor 1	protein_coding	NP_001342584.1;NP_067356.2;XP_006506495.1;XP_006506496.1	1929676	.		2.841241	1.261795	1.289511	2.026464	0.823221	0.798050	0.465606	1.102128	7.474627	3.350413	3.471066	5.432233	2.167419	2.186169	1.255351	2.996122	-1.852536	236.384244	0.393892614	-2.258233035	0.023931135	0.303042012	8.262177	7.372873	7.482762	7.242774	7.710845	7.795772	8.134045	7.478857	7.794886	8.396924	137	94	204	223	377	158	200	498
58184	NM_021383,XM_006496171	Cnot9	CCR4-NOT transcription complex, subunit 9	protein_coding	NP_067358.1;XP_006496234.1	1928902	.		13.688211	13.325157	14.197209	13.277520	14.077754	14.855990	14.330597	13.538757	36.010421	35.381958	38.215609	35.592328	37.064652	40.696338	38.637697	36.804968	1.084703	1949.230475	0.066903858	1.753273043	0.07955515	0.487661308	10.868785	10.986205	10.985765	11.016253	10.918899	10.953050	10.879313	10.902958	10.868767	10.892140	2004	2209	1946	1943	1942	2138	1606	1882
58185	NM_021384	Rsad2	radical S-adenosyl methionine domain containing 2	protein_coding	NP_067359.2	1929628	.		1.188517	0.867187	0.942953	0.876055	1.176685	0.864358	0.480801	0.974578	3.126705	2.302619	2.538212	2.348392	3.098038	2.367813	1.296320	2.649379	-1.216638	150.231819	0.254916194	-1.10977651	0.267095344	0.704716118	7.364760	7.083662	7.183112	6.924502	7.265469	7.237609	7.193074	7.343314	7.182783	7.359041	137	87	164	151	150	208	123	190
58186	NM_001167730,NM_021385,XM_006506434,XM_006506435,XM_006506436,XM_006506437,XM_017321689	Rad18	RAD18 E3 ubiquitin protein ligase	protein_coding	NP_001161202.1;NP_067360.2;XP_006506497.1;XP_006506498.1;XP_006506499.1;XP_006506500.1;XP_017177178.1	1890476	.		4.657411	3.092449	4.169750	3.799774	2.223457	4.719281	4.735321	3.562097	12.252536	8.211302	11.224002	10.185846	5.854034	12.927949	12.767220	9.683522	1.293862	435.030224	0.226220844	1.643010757	0.100380708	0.526235052	8.567470	8.938663	8.911593	8.972080	8.691750	8.813444	8.731177	8.292571	8.567731	8.875147	511	586	408	456	444	272	303	504
58187	NM_001160096,NM_001160097,NM_001160098,NM_001160099,NM_021386,NM_023878	Cldn10	claudin 10	protein_coding	NP_001153568.1;NP_001153569.1;NP_001153570.1;NP_001153571.1;NP_067361.2;NP_076367.2	1913101	.		1.331392	1.247049	1.689808	0.606109	1.314086	0.296594	0.803019	0.595417	3.502574	3.311257	4.548573	1.624764	3.459795	0.812488	2.165075	1.618634	-1.276296	40.310644	0.539433299	-0.652468814	0.514098785	0.855488579	5.498439	5.146305	5.105118	5.261703	5.196066	5.463786	5.196177	5.365638	5.357265	5.378643	12	36	25	68	26	58	44	54
58188	NM_021387,XM_006541031,XM_006541032,XM_006541033,XM_030242842,XM_030242843,XM_030242844	Vstm2b	V-set and transmembrane domain containing 2B	protein_coding	NP_067362.1;XP_006541094.1;XP_006541095.1;XP_006541096.1;XP_030098702.1;XP_030098703.1;XP_030098704.1	1914525	.		0.706614	0.532245	0.431766	1.493918	0.689058	0.565361	0.227688	0.367540	1.858932	1.413256	1.162214	4.004662	1.814188	1.548743	0.613884	0.999154	-1.725360	56.617315	0.409828106	-1.920066103	0.05484955	0.429682353	6.162688	5.378335	5.812098	5.595391	5.681653	5.671211	6.089859	5.785425	5.780528	5.762674	60	28	40	37	126	64	49	53
58193	NM_001163514,NM_001163515,NM_021388,XM_011240177,XM_017319674,XM_030252739	Extl2	exostoses (multiple)-like 2	protein_coding	NP_001156986.1;NP_001156987.1;NP_067363.3;XP_011238479.1;XP_017175163.1;XP_030108599.1	1889574	.		11.786186	8.551177	10.312778	10.285939	9.877865	9.919457	10.336635	9.947302	31.006652	22.705729	27.759617	27.572962	26.006961	27.173258	27.869305	27.041635	1.029217	1294.449413	0.112483938	0.369361079	0.711858604	0.92936198	10.317029	10.358913	10.301435	10.422731	10.332048	10.344237	10.362073	10.284221	10.166515	10.456373	1214	1468	1298	1269	1388	1380	950	1460
58194	NM_001135727,NM_001135728,NM_001290661,NM_001290664,NM_021389,NR_110969,NR_110970,XM_006528924,XM_006528925,XM_006528926,XM_006528927,XM_006528928,XM_006528929,XM_006528932,XM_011247849,XM_011247851,XM_017318584,XM_017318585,XM_017318586,XM_030251430,XM_030251431,XM_030251432,XM_030251433	Sh3kbp1	SH3-domain kinase binding protein 1	protein_coding	NP_001129199.1;NP_001129200.1;NP_001277590.1;NP_001277593.1;NP_067364.2;XP_006528987.1;XP_006528988.1;XP_006528989.1;XP_006528990.1;XP_006528991.1;XP_006528992.1;XP_006528995.1;XP_011246151.1;XP_011246153.1;XP_017174073.1;XP_017174074.1;XP_017174075.1;XP_030107290.1;XP_030107291.1;XP_030107292.1;XP_030107293.1	1889583	.		11.139937	9.059928	10.889709	10.316454	13.280192	11.159160	12.236195	11.591548	29.306523	24.056600	29.312584	27.654761	34.964787	30.569285	32.990841	31.511503	1.053006	2404.674715	0.132239758	0.5634751	0.573111411	0.884736714	11.193703	11.268578	11.197104	11.386409	11.253838	11.173530	11.174503	11.391045	10.990028	11.216915	2266	2908	2481	2217	2384	3157	1663	2377
58198	NM_001371069,NM_001371070,NM_021390	Sall1	spalt like transcription factor 1	protein_coding	NP_001357998.1;NP_001357999.1;NP_067365.2	1889585	.		0.034105	0.075150	0.042498	0.332850	0.198708	0.073024	0.090636	0.200162	0.089721	0.199544	0.114395	0.892252	0.523168	0.200042	0.244371	0.544140	-1.499394	31.684299	0.725781345	-0.805172458	0.420720168	0.809507345	5.247703	4.665309	4.820495	4.853149	5.024274	4.757397	5.183755	5.005184	4.819942	4.738577	18	24	50	10	83	52	16	8
58200	NM_021391,XR_001781557	Ppp1r1a	protein phosphatase 1, regulatory inhibitor subunit 1A	protein_coding	NP_067366.1	1889595	.		3.892096	11.721290	2.582393	12.493531	8.614846	2.171378	3.121202	8.529586	10.239179	31.123247	6.951205	33.490730	22.681620	5.948249	8.415284	23.187588	-2.170807	367.504857	0.57731943	-1.936707508	0.052781107	0.423096028	8.975021	7.856725	7.634129	7.909950	8.652425	7.750211	8.979003	8.620585	8.912748	8.006392	115	192	484	140	724	520	564	212
58202	NM_001310157,NM_021393	Nelfb	negative elongation factor complex member B	protein_coding	NP_001297086.1;NP_067368.3	1931035	.		17.201267	19.963443	18.516417	15.949521	16.812230	16.769273	16.141076	15.665153	45.252440	53.008433	49.841917	42.755017	44.264126	45.937569	43.519052	[illegible]	-1.007331	1965.600879	0.102841802	-0.102469302	0.918384178	0.981955234	10.946274	10.935214	10.911729	10.935911	10.860824	11.027978	10.865279	10.880720	11.088056	10.926007	1859	2037	1833	2084	1910	2086	1983	1940
[illegible]																																																

59310	NM_001085387,NM_001303019,NM_001303021,NM_021611,XM_017321050,XM_017321051,XM_017321052	Myl10	myosin, light chain 10, regulatory	protein_coding
60315	NM_021713,XM_030248679	Myg1	melanocyte proliferating gene 1	protein_coding
60321	NM_021714,XM_017321693,XM_030255522	Wbp11	WW domain binding protein 11	protein_coding
60322	NM_021715,XR_878032	Chst7	carbohydrate (N-acetylglucosamino) sulfotransferase 7	protein_coding
60344	NM_001267846,NM_001267847,NM_021716	Fign	fidgetin	protein_coding
60345	NM_001162858,NM_021717,XM_006506449,XM_006506451,XM_030255523	Nrip2	nuclear receptor interacting protein 2	protein_coding
60361	NM_021718,XM_030251018,XM_030251019	Ms4a4b	membrane-spanning 4-domains, subfamily A, member 4B	protein_coding
60363	NM_021719	Cldn15	claudin 15	protein_coding
60364	NM_021720,XM_006523069,XM_006523070	Donson	downstream neighbor of SON	protein_coding
60365	NM_001102407,NM_025875	Rbm8a	RNA binding motif protein 8a	protein_coding
60367	NM_030688	Il1rapl2	interleukin 1 receptor accessory protein-like 2	protein_coding
60406	NM_021788	Sap30	sin3 associated polypeptide	protein_coding
60409	NM_021789,XM_017313498	Trappc4	trafficking protein particle complex 4	protein_coding
60411	NM_021790,NM_181061,NR_075088,XM_006517728,XM_017315552,XM_017315553	Cenpk	centromere protein K	protein_coding
60425	NM_021791	Doc2g	double C2, gamma	protein_coding
60440	NM_001146275,NM_021792,XM_011246978,XM_011246979,XM_011246980,XM_030250557,XM_030250558,XM_030250559,XM_030250560	Iigp1	interferon inducible GTPase 1	protein_coding
60441	NM_024177	Mrpl38	mitochondrial ribosomal protein L38	protein_coding
60455	NM_021793,XM_006524736,XM_006524737,XM_006524738	Tmem8	transmembrane protein 8	protein_coding
60504	NM_021887,XM_006508097,XM_006508098	Il21r	interleukin 21 receptor	protein_coding
60507	NM_021888,XM_006510524,XM_006510525,XM_011242583,XR_001778954,XR_003947960,XR_003947961,XR_379128	Qtrt1	queuine tRNA-ribosyltransferase catalytic subunit 1	protein_coding
60510	NM_021889,XM_006508099,XR_003946532,XR_378262	Syt9	synaptotagmin IX	protein_coding
60525	NM_019811	Acss2	acyl-CoA synthetase short-chain family member 2	protein_coding
60527	NM_021890,XR_003952799,XR_003952800	Fads3	fatty acid desaturase 3	protein_coding
60530	NM_001163359,NM_001163360,NM_021891,XM_006514767,XM_011243737,XM_011243738,XM_030246214	Fignl1	fidgetin-like 1	protein_coding
60531	NM_021892	Npvf	neuropeptide VF precursor	protein_coding
60532	NM_001113532,NM_001113533,NM_175394,XM_006523341	Wtap	Wilms tumour 1-associating protein	protein_coding
60533	NM_021893,XM_030251020,XM_030251021	Cd274	CD274 antigen	protein_coding
60534	NM_001163233,NM_053081,XM_006538127,XM_006538128,XM_017320336,XR_001784169,XR_001784170,XR_001784171,XR_001784172,XR_001784173,XR_001784174,XR_003954990,XR_003954991,XR_003954992,XR_390333,XR_390334,XR_390335,XR_390336,XR_390337	Fancg	Fanconi anemia, complementation group G	protein_coding
60594	NM_001110807,XM_006540253,XR_003946533	Capn12	calpain 12	protein_coding
60595	NM_001360548,NM_001360549,NM_001360550,NM_021895,XM_006540255,XM_006540257	Actn4	actinin alpha 4	protein_coding
60596	NM_001356987,NM_001356988,NM_021896	Gucy1a1	guanylate cyclase 1, soluble, alpha 1	protein_coding
60597	NM_021921	Mapk8ip2	mitogen-activated protein kinase 8 interacting protein 2	protein_coding
60599	NM_001199105,NM_021897	Trp53inp1	transformation related protein 53 inducible nuclear protein 1	protein_coding
60611	NM_021899	Foxj2	forkhead box J2	protein_coding
60613	NM_001081142,XM_006503255,XM_006503256,XM_011240582,XM_011240583	Kcnq4	potassium voltage-gated channel, subfamily Q, member 4	protein_coding
63828	NM_001038699,NM_022014	Fn3k	fructosamine 3 kinase	protein_coding
63830	NR_001461	Kcnq1ot1	KCNQ1 overlapping transcript 1	lncRNA
63856	NM_001356290,NM_022015,XM_006524766,XR_003952171	Taf8	TATA-box binding protein associated factor 8	protein_coding
63857	NM_001163028,NM_021486,XM_011248481	Bco1	beta-carotene oxygenase 1	protein_coding
63859	NM_022016,XM_006511328	Impg1	interphotoreceptor matrix proteoglycan 1	protein_coding
63872	NM_022409,XM_030242864	Zfp296	zinc finger protein 296	protein_coding
63873	NM_022017,XM_006530432,XM_011248235	Trpv4	transient receptor potential cation channel, subfamily V, member 4	protein_coding
63913	NM_022018,XM_006529776	Fam129a	family with sequence similarity 129, member A	protein_coding
63953	NM_022019,XM_006497188,XM_006497189	Dusp10	dual specificity phosphatase 10	protein_coding
63954	NM_022020,XM_006539085,XM_006539087	Rbp7	retinol binding protein 7, cellular	protein_coding
63955	NM_001146287,NM_022021	Cables1	CDK5 and Abl enzyme substrate 1	protein_coding
63958	NM_022022,XM_006539088,XM_006539089	Ube4b	ubiquitination factor E4B	protein_coding
63959	NM_001199113,NM_001199114,NM_001199115,NM_001199116,NM_001357771,NM_022880,XM_006524768,XM_006524769,XM_006524770,XM_006524772,XM_006524773,XM_030249965,XM_030249966,XM_030249967,XM_030249968,XM_030249969,XM_030249970	Slc29a1	solute carrier family 29 (nucleoside transporters), member 1	protein_coding
63985	NM_022023,XM_030247909,XM_030247910,XM_030247911,XM_030247912,XM_030247913	Gmfb	glia maturation factor, beta	protein_coding
63986	NM_001039192,NM_001290550,NM_022024,XM_006540272,XM_006540273	Gmfg	glia maturation factor, gamma	protein_coding
63993	NM_022025,XM_006524774,XM_006524775,XM_006524776	Slc5a7	solute carrier family 5 (choline transporter), member 7	protein_coding
64008	NM_001271843,NM_022026,NR_073483,XM_006511331,XM_006511332,XM_011242782,XM_011242783,XM_030244487	Aqp9	aquaporin 9	protein_coding
64009	NM_001079686,NM_001347711,NM_001347732,NM_022027,NM_153399,XM_006512458,XM_006512465,XM_011243084,XM_011243086,XM_011243089,XM_011243090,XM_011243091,XM_011243092,XM_011243093,XM_011243097,XM_017314043,XM_017314044,XM_017314045,XM_017314046,XM_017314047,XM_017314048,XM_030245203,XM_030245204,XM_030245205,XM_030245206,XM_030245207	Syne1	spectrin repeat containing, nuclear envelope 1	protein_coding
64010	NM_022028,XM_030246842	Sav1	salvador family WW domain containing 1	protein_coding
64011	NM_022029	Nrgn	neurogranin	protein_coding
64050	NM_026570	Yeats4	YEATS domain containing 4	protein_coding
64051	NM_022030	Sv2a	synaptic vesicle glycoprotein 2 a	protein_coding
64058	NM_022032	Perp	PERP, TP53 apoptosis effector	protein_coding
64059	NM_022033	Oxct2a	3-oxoacid CoA transferase 2A	protein_coding
64074	NM_022315,XM_006524777	Smoc2	SPARC related modular calcium binding 2	protein_coding
64075	NM_001146217,NM_022316,XM_006516135	Smoc1	SPARC related modular calcium binding 1	protein_coding
64082	NM_001081984,NM_022318,XM_006522438,XM_006522439,XM_030249226	Popdc2	popeye domain containing 2	protein_coding
64085	NM_022319,XM_006511334	Clstn2	calsyntenin 2	protein_coding
64095	NM_001104529,NM_001271766,NM_022320,XM_006529777,XM_006529778,XM_006529779,XM_006529780,XM_006529781,XM_011248053	Gpr35	G protein-coupled receptor 35	protein_coding
64099	NM_001162500,NM_022321,XM_006521241,XM_011245706	Parvg	parvin, gamma	protein_coding
64103	NM_022322,XM_017318595,XM_017318596	Tnmd	tenomodulin	protein_coding
64113	NM_001142937,NM_022323	Moap1	modulator of apoptosis 1	protein_coding
64136	NM_022324	Sdf2l1	stromal cell-derived factor 2-like 1	protein_coding
64138	NM_022325	Ctsz	cathepsin Z	protein_coding
64143	NM_022327,XM_006529782	Ralb	v-ral simian leukemia viral oncogene B	protein_coding
64144	NM_022328,XM_006524778	Mllt1	myeloid/lymphoid or mixed-lineage leukemia; translocated to, 1	protein_coding
64176	NM_001109753,NM_001360571,NM_001360572,NM_001360573,NM_001360574,NM_001360575,NM_153579,XM_006541059	Sv2b	synaptic vesicle glycoprotein 2 b	protein_coding
64177	NM_022413	Trpv6	transient receptor potential cation channel, subfamily V, member 6	protein_coding
64209	NM_001357205,NM_022331	Herpud1	homocysteine-inducible, endoplasmic reticulum stress-inducible, ubiquitin-like domain member 1	protein_coding
64213	NM_001083315,NM_001289624,NM_001289625,NM_001289626,NM_001289627,NM_001289629,NM_022332,XM_011241075,XM_011241076,XR_003956242,XR_003956243,XR_003956244,XR_003956245,XR_003956246,XR_003956247,XR_377290,XR_377291,XR_868763,XR_868764	St7	suppression of tumorigenicity 7	protein_coding
64214	NM_022881,XM_011248054,XM_017321918	Rgs18	regulator of G-protein signaling 18	protein_coding
64242	NM_001294308,NM_022414	Ngb	neuroglobin	protein_coding
64291	NM_001252489,NM_001252490,NM_001252491,NM_001252492,NM_001252493,NM_207530,NR_045524,XM_006526141,XM_006526142,XM_006526143,XM_006526144,XM_006526147,XM_006526148,XM_017317960,XM_017317961,XM_017317962,XM_017317963,XM_017317964,XM_017317965,XM_030250563,XM_030250564,XM_030250565,XM_030250566,XM_030250567,XM_030250568,XM_030250569	Osbpl1a	oxysterol binding protein-like 1A	protein_coding
64292	NM_022415,XM_030251932	Ptges	prostaglandin E synthase	protein_coding
64293	NM_022416	Stk32b	serine/threonine kinase 32B	protein_coding
64294	NM_022417	Itm2c	integral membrane protein 2C	protein_coding
64295	NM_022418,XM_006535785,XM_006535786	Tmub1	transmembrane and ubiquitin-like domain containing 1	protein_coding
64296	NM_022419,XM_030243705	Abhd8	abhydrolase domain containing 8	protein_coding
64297	NM_001195774,NM_022420	Gprc5b	G protein-coupled receptor, family C, group 5, member B	protein_coding
64337	NM_001357782,NM_022422	Gng13	guanine nucleotide binding protein (G protein), gamma 13	protein_coding
64339	NM_001356945,NM_001356946,NM_022424,XM_011240762	Fndc4	fibronectin type III domain containing 4	protein_coding
64340	NM_178380,NR_151644,XM_006531261	Dhx38	DEAH (Asp-Glu-Ala-His) box polypeptide 38	protein_coding
64378	NM_022427,XM_006501856,XM_006501857	Gpr88	G-protein coupled receptor 88	protein_coding
64379	NM_001301137,NM_022428	Irx6	Iroquois homeobox 6	protein_coding
64380	NM_029499,XM_006527250,XM_006527251,XM_017318270	Ms4a4c	membrane-spanning 4-domains, subfamily A, member 4C	protein_coding
64381	NM_022430	Ms4a8a	membrane-spanning 4-domains, subfamily A, member 8A	protein_coding
64383	NM_001122765,NM_001122766,NM_022432	Sirt2	sirtuin 2	protein_coding
64384	NM_001127351,NM_001177804,NM_022433,XM_006536222,XM_006536223	Sirt3	sirtuin 3	protein_coding
64406	NM_022435	Sp5	trans-acting transcription factor 5	protein_coding
64424	NM_001285800,NM_022811	Polr1e	polymerase (RNA) I polypeptide E	protein_coding
64435	NM_001170632,NM_144960,XM_006529787,XM_017321921,XM_017321922,XM_017321923,XM_017321924,XM_017321925	Fcamr	Fc receptor, IgA, IgM, high affinity	protein_coding
64436	NM_001290437,NM_033134,NR_110957,XR_003953682	Inpp5e	inositol polyphosphate-5-phosphatase E	protein_coding
64450	NM_001330666,NM_001330667,NM_145066,XM_011241079,XM_017321705,XM_030255527,XM_030255528,XM_030255529,XM_030255530	Gpr85	G protein-coupled receptor 85	protein_coding
64451	NM_001081419,XM_006513939,XM_006513940,XM_006513941,XM_030245208,XR_001779571	Dip2a	disco interacting protein 2 homolog A	protein_coding
64453	NM_177475,XM_006513942,XM_006513943	Zfp280b	zinc finger protein 280B	protein_coding
64540	NM_001252588,NM_053082,XM_006536224,XM_030242865	Tspan4	tetraspanin 4	protein_coding
64580	NM_022565,XM_006501859,XM_006501860,XM_011240189	Ndst4	N-deacetylase/N-sulfotransferase (heparin glucosaminyl) 4	protein_coding
64602	NM_022655,XM_006511335,XM_017313499,XM_017313500	Ireb2	iron responsive element binding protein 2	protein_coding
64652	NM_001347583,NM_022656,XM_017316115,XM_017316116	Nisch	nischarin	protein_coding
64654	NM_022657,XM_017321707	Fgf23	fibroblast growth factor 23	protein_coding
64655	NM_025485	Mrps22	mitochondrial ribosomal protein S22	protein_coding
64656	NM_001291270,NM_001291271,NM_001291272,NM_001291273,NM_024174,NR_111902	Mrps23	mitochondrial ribosomal protein S23	protein_coding
64657	NM_001146211,NM_001146212,NM_183086,XM_011246566,XM_011246567,XM_030249974,XM_030249975,XM_030249976	Mrps10	mitochondrial ribosomal protein S10	protein_coding
64658	NM_025578	Mrps25	mitochondrial ribosomal protein S25	protein_coding
64659	NM_001347121,NM_025474	Mrps14	mitochondrial ribosomal protein S14	protein_coding
64660	NM_001362871,NM_026080	Mrps24	mitochondrial ribosomal protein S24	protein_coding
64661	NM_001033131,NM_001364009,NM_001364010,NM_001364011	Krtdap	keratinocyte differentiation associated protein	protein_coding
64685	NM_001141948,NM_001141949,NM_019401,XM_006498218,XM_006498219,XM_030251933,XM_030251934	Nmi	N-myc (and STAT) interactor	protein_coding
64704	NM_019752	Htra2	HtrA serine peptidase 2	protein_coding
64705	NM_001164466,NM_022722	Dpys	dihydropyrimidinase	protein_coding
64706	NM_001271472,NM_001271473,NM_022723,XM_006521242,XM_006521243,XM_017316719,XR_003951419	Scube1	signal peptide, CUB domain, EGF-like 1	protein_coding
64707	NM_022724,NR_027509	Suv39h2	suppressor of variegation 3-9 2	protein_coding
64817	NM_022814	Svep1	sushi, von Willebrand factor type A, EGF and pentraxin domain containing	protein_coding
64833	NM_022816	Acot10	acyl-CoA thioesterase 10	protein_coding
64898	NM_001164885,NM_001357791,NM_022882,XM_006524786,XM_006524788,XM_030249977,XM_030249978	Lpin2	lipin 2	protein_coding
64899	NM_001199118,NM_022883,XM_017319164,XR_374499,XR_3745	Lpin3	lipin 3	protein_coding
64903	gene-Rpl30-ps5	Rpl30-ps5	ribosomal protein L30, pseudogene 5	pseudogene
64918	NM_022884	Bhmt2	betaine-homocysteine methyltransferase 2	protein_coding
64929	NM_022886,XM_006519355,XM_006519356	Scel	sciellin	protein_coding
64930	NM_001289575,NM_001289576,NM_022887,XM_006498220,XM_006498221,XM_006498222,XM_006498223,XM_011239142,XM_011239143,XM_011239144,XM_030251935,XM_030251936	Tsc1	TSC complex subunit 1	protein_coding
64933	NM_001122820,NM_029505,XM_030243706	Ap3m2	adaptor-related protein complex 3, mu 2 subunit	protein_coding
64934	NM_022889	Pes1	pescadillo ribosomal biogenesis factor 1	protein_coding
64945	NM_001193659,NM_001193660,NM_001193661,NM_022890,XM_006503598,XM_011240673,XM_011240674,XM_030254750	Cldn12	claudin 12	protein_coding
65019	NM_022891	Rpl23	ribosomal protein L23	protein_coding
65020	NM_001360576,NM_022981	Zfp110	zinc finger protein 110	protein_coding
65079	NM_022982	Rtn4r	reticulon 4 receptor	protein_coding
65086	NM_022983	Lpar3	lysophosphatidic acid receptor 3	protein_coding
65098	NM_001360577,NM_001360578,NM_001360579,NM_001360580,NM_001360581,NM_001360582,NM_022985,XM_006508110,XM_030242866,XM_030242867,XM_030242868,XM_030242869,XM_030242870,XM_030242871,XM_030242872,XM_030242873,XM_030242874,XM_030242875,XM_030242876,XM_030242877	Zfand6	zinc finger, AN1-type domain 6	protein_coding
65099	NM_001168240,NM_022986	Irak1bp1	interleukin-1 receptor-associated kinase 1 binding protein 1	protein_coding
65100	NM_022987,XM_006519357	Zic5	zinc finger protein of the cerebellum 5	protein_coding
65102	NM_022988,XM_006496186	Nif3l1	Ngg1 interacting factor 3-like 1 (S. pombe)	protein_coding
65103	NM_022989	Arl6ip6	ADP-ribosylation factor-like 6 interacting protein 6	protein_coding
65105	NM_144509	Arl6ip4	ADP-ribosylation factor-like 6 interacting protein 4	protein_coding
65106	NM_022992	Arl6ip5	ADP-ribosylation factor-like 6 interacting protein 5	protein_coding
65107	NM_022993,XM_006519358	Lrp10	low-density lipoprotein receptor-related protein 10	protein_coding
65111	NM_001164533,NM_001368419,NM_022994,XM_006501863,XM_006501864,XM_006501865,XM_006501866,XM_006501867,XM_006501868,XM_006501869,XM_030252745,XM_030252746,XM_030252747	Dap3	death associated protein 3	protein_coding
65112	NM_022995,XM_006499991	Pmepa1	prostate transmembrane protein, androgen induced 1	protein_coding
65113	NM_001355749,NM_022996	Ndfip1	Nedd4 family interacting protein 1	protein_coding
65114	NM_022997	Vps35	VPS35 retromer complex component	protein_coding
65115	NM_001141922,NM_001141924,NM_001141925,XM_006531264,XM_006531265,XM_006531266,XM_006531267,XM_006531270,XM_011248482,XM_011248483,XM_011248484,XM_030243707,XM_030243708	Bean1	brain expressed, associated with Nedd4, 1	protein_coding
65116	NM_022999,XM_006541062,XM_006541063,XM_006541064,XM_017312219,XM_017312220	Prrg2	proline-rich Gla (G-carboxyglutamic acid) polypeptide 2	protein_coding
65221	NM_023044	Slc15a3	solute carrier family 15, member 3	protein_coding
65246	NM_001359888,NM_023045,XM_006519359,XM_011245132,XM_030247914	Xpo7	exportin 7	protein_coding
65247	NM_001039126,NM_023046,XM_006529789,XM_006529790	Asb1	ankyrin repeat and SOCS box-containing 1	protein_coding
65254	NM_001356948,NM_001356949,NM_023047	Dpysl5	dihydropyrimidinase-like 5	protein_coding
65255	NM_001355662,NM_023048	Asb4	ankyrin repeat and SOCS box-containing 4	protein_coding
65256	NM_023049,XM_030246845	Asb2	ankyrin repeat and SOCS box-containing 2	protein_coding
65257	NM_023906,XM_006514771,XR_003949485	Asb3	ankyrin repeat and SOCS box-containing 3	protein_coding
65945	NM_001290989,NM_023051,XM_006539093,XM_006539094,XM_030253692,XM_030253693	Clstn1	calsyntenin 1	protein_coding
65957	gene-Ccl19-ps1	Ccl19-ps1	chemokine (C-C motif) ligand 19, pseudogene 1	pseudogene
65958	gene-Ccl19-ps2	Ccl19-ps2	chemokine (C-C motif) ligand 19, pseudogene 2	pseudogene
65960	NM_023053	Twsg1	twisted gastrulation BMP signaling modulator 1	protein_coding
65961	NM_023054	Utp3	UTP3 small subunit processome component	protein_coding
65962	NM_001357796,NM_023055,NM_023449,XM_030249980	Slc9a3r2	solute carrier family 9 (sodium/hydrogen exchanger), member 3 regulator	protein_coding
65963	NM_001164207,NM_001164208,NM_001164209,NM_001286651,NM_001286652,NM_023056,NR_104575,XM_006506456,XM_006506457,XM_006506458,XM_006506459,XM_006506460,XM_006506461,XM_017321710,XM_030255531,XM_030255532,XM_030255533,XM_030255534,XM_030255535	Tmem176b	transmembrane protein 176B	protein_coding
65964	NM_001164791,NM_023057,NM_178084,XM_006499997,XM_006499998,XM_030251937	Map3k20	mitogen-activated protein kinase kinase kinase 20	protein_coding
65967	NM_023060,NR_149744,XM_006506465,XM_030255536,XM_030255537,XM_030255538	Eefsec	eukaryotic elongation factor, selenocysteine-tRNA-specific	protein_coding
65969	NM_001081084	Cubn	cubilin (intrinsic factor-cobalamin receptor)	protein_coding
65970	NM_001113545,NM_023063,XM_006521245,XM_030248689,XR_001781560	Lima1	LIM domain and actin binding 1	protein_coding
65971	NM_001017407,NM_001017409,NM_001017419,NM_001017433,NM_001017441,NM_023064	Tbata	thymus, brain and testes associated	protein_coding
65972	NM_023065	Ifi30	interferon gamma inducible protein 30	protein_coding
65973	NM_001177849,NM_001177850,NM_001177851,NM_001177852,NM_001177853,NM_001177854,NM_001177855,NM_001177856,NM_001290367,NM_023066,NM_133723,XM_006538159,XM_017320340,XM_017320341,XM_017320342,XM_017320343,XM_017320344,XM_017320345	Asph	aspartate-beta-hydroxylase	protein_coding
66011	NM_001362875,NM_023146,NR_110973,XM_006514776,XM_006514777,XM_011243739,XM_011243740,XM_011243741,XM_030246217,XM_030246218,XR_003949486,XR_003949487,XR_380976,XR_380977,XR_380979	Ranbp17	RAN binding protein 17	protein_coding
66039	NM_025311,NM_026679,XM_030247920	Tmem254a	transmembrane protein 254a	protein_coding
66042	NM_025312	Sostdc1	sclerostin domain containing 1	protein_coding

66043	NM_001347092,NM_025313,XM_017314052,XM_030245209,XM_030245210,XM_030245211,XM_030245212,XM_030245213	Atp5d	ATP synthase, H+ transporting, mitochondrial F1 complex, delta subunit	protein_coding	NP_001334021.1;NP_079589.2;XP_017169541.1;XP_030101069.1;XP_030101070.1;XP_030101071.1;XP_030101072.1;XP_030101073.1	1913293	.	[illegible]
66044	NM_025314	Dtd1	D-tyrosyl-tRNA deacylase 1	protein_coding	NP_079590.1	1913294	.	[illegible]
66046	NM_025316,XM_006535514	Ndufb5	NADH:ubiquinone oxidoreductase subunit B5	protein_coding	NP_079592.2;XP_006535577.1	1913296	.	[illegible]
66047	NM_025317	Mrpl54	mitochondrial ribosomal protein L54	protein_coding	NP_079593.1	1913297	.	[illegible]
66048	NM_001168470,NM_025318	Emc6	ER membrane protein complex subunit 6	protein_coding	NP_001161942.1;NP_079594.1	1913298	.	[illegible]
66049	NM_133185,XM_017317109,XR_003951813	Rogdi	rogdi homolog	protein_coding	NP_573448.2;XP_017172598.1	1913299	.	[illegible]
66050	NM_025319,XM_006533917,XM_006533918,XM_006533919	0610009B22Rik	RIKEN cDNA 0610009B22 gene	protein_coding	NP_079595.1;XP_006533980.1;XP_006533981.1;XP_006533982.1	1913300	.	[illegible]
66052	NM_025321,XM_006496955,XM_030241961	Sdhc	succinate dehydrogenase complex, subunit C, integral membrane protein	protein_coding	NP_079597.2;XP_006497018.2;XP_030097821.1	1913302	.	[illegible]
66053	NM_001252444,NM_001252445,NM_001356386,NM_144954,XM_006522443,XM_006522445,XM_006522446,XM_006522447,XM_030249228	Ppil2	peptidylprolyl isomerase (cyclophilin)-like 2	protein_coding	NP_001239373.1;NP_001239374.1;NP_001343315.1;NP_659203.1;XP_006522506.1;XP_006522508.1;XP_006522509.1;XP_006522510.1;XP_030105088.1	2447857	.	[illegible]
66054	NM_001289531,NM_023149,XM_030250570	Cndp2	CNDP dipeptidase 2 (metallopeptidase M20 family)	protein_coding	NP_001276460.1;NP_075638.2;XP_030106430.1	1913304	.	[illegible]
66055	NM_025323	Sf3b6	splicing factor 3B, subunit 6	protein_coding	NP_079599.1	1913305	.	[illegible]
66056	NM_025324,XM_006540276,XM_006540277,XM_011250670,XM_030242878	Zfp524	zinc finger protein 524	protein_coding	NP_079600.1;XP_006540339.1;XP_006540340.1;XP_011248972.1;XP_030098738.1	1916740	.	[illegible]
66058	NM_001098271,NM_025326	Tmem176a	transmembrane protein 176A	protein_coding	NP_001091741.1;NP_079602.4	1913308	.	[illegible]
66059	NM_001310625,NM_025327,XM_006501870,XM_030252748,XM_030252749	Krtcap2	keratinocyte associated protein 2	protein_coding	NP_001297554.1;NP_079603.1;XP_006501933.3;XP_030108608.1;XP_030108609.1	1913309	.	[illegible]
66060	NM_001081365,NM_001368637	Cystm1	cysteine-rich transmembrane module containing 1	protein_coding	NP_001074834.1;NP_001355566.1	1913310	.	[illegible]
66061	NM_001347183,NM_025329,NR_154990	Tctex1d2	Tctex1 domain containing 2	protein_coding	NP_001334112.1;NP_079605.1	1913311	.	[illegible]
66065	NM_025330,XM_006541069,XM_006541070,XM_011250889	Hsd17b14	hydroxysteroid (17-beta) dehydrogenase 14	protein_coding	NP_079606.3;XP_006541132.2;XP_006541133.1;XP_011249191.1	1913315	.	[illegible]
66066	NM_025331	Gng11	guanine nucleotide binding protein (G protein), gamma 11	protein_coding	NP_079607.1	1913316	.	[illegible]
66067	NM_001159329,NM_001356392,NM_025332	Gtpbp8	GTP-binding protein 8 (putative)	protein_coding	NP_001152801.1;NP_001343321.1;NP_079608.1	1913317	.	[illegible]
66069	NM_178374,XM_006511336,XM_011242784,XM_030244488,XR_003947964	Snupn	snurportin 1	protein_coding	NP_848461.1;XP_006511399.1;XP_011241086.1;XP_030100348.1	1913319	.	[illegible]
66070	NM_023153,XM_006510537	Cwc15	CWC15 spliceosome-associated protein	protein_coding	NP_075642.1;XP_006510600.1	1913320	.	[illegible]
66071	NM_001364014,NM_023154,XM_030242879,XM_030242880,XM_030242881	Ethe1	ethylmalonic encephalopathy 1	protein_coding	NP_001350943.1;NP_075643.1;XP_030098739.1;XP_030098740.1;XP_030098741.1	1913321	.	[illegible]
66072	NM_025333	Sdhaf2	succinate dehydrogenase complex assembly factor 2	protein_coding	NP_079609.2	1913322	.	[illegible]
66073	NM_025334	Txndc12	thioredoxin domain containing 12 (endoplasmic reticulum)	protein_coding	NP_079610.1	1913323	.	[illegible]
66074	NM_025335	Tmem167	transmembrane protein 167	protein_coding	NP_079611.2	1913324	.	[illegible]
66075	NM_001355667,NM_025336,XM_006506468	Chchd3	coiled-coil-helix-coiled-coil-helix domain containing 3	protein_coding	NP_001342596.1;NP_079612.1;XP_006506531.1	1913325	.	[illegible]
66077	NM_025338,XM_006539095	Aurkaip1	aurora kinase A interacting protein 1	protein_coding	NP_079614.1;XP_006539158.1	1913327	.	[illegible]
66078	NM_001164204,NM_001164205,NM_024168,XM_006540280,XM_017312222,XM_017312223,XM_017312224,XM_017312225,XM_017312226,XR_001777993,XR_003946537,XR_003946538,XR_003946539,XR_003946540	Tsen34	tRNA splicing endonuclease subunit 34	protein_coding	NP_001157676.1;NP_001157677.1;NP_077130.1;XP_006540343.2;XP_017167711.2;XP_017167712.1;XP_017167713.1;XP_017167714.1;XP_017167715.1	1913328	.	[illegible]
66079	NM_001164823,NM_025339,XM_006512205,XM_006512206	Tmem42	transmembrane protein 42	protein_coding	NP_001158295.1;NP_079615.1;XP_006512268.1;XP_006512269.1	1277176	.	[illegible]
66082	NM_001331064,NM_001331065,NM_025341,XM_006518073	Abhd6	abhydrolase domain containing 6	protein_coding	NP_001317993.1;NP_001317994.1;NP_079617.2;XP_006518136.1	1913332	.	[illegible]
66083	NM_001035123,NM_001368354,NR_160790,NR_160791,NR_160792,XM_030243711,XM_030243712	Setd6	SET domain containing 6	protein_coding	NP_001030295.1;NP_001355283.1;XP_030099571.1;XP_030099572.1	1913333	.	[illegible]
66084	NM_001358796,NM_025343,XM_006512477,XM_006512478,XM_017314053,XM_017314054	Rmnd1	required for meiotic nuclear division 1 homolog	protein_coding	NP_001345725.1;NP_079619.4;XP_006512540.1;XP_006512541.1;XP_017169542.1;XP_017169543.1	1913334	.	[illegible]
66085	NM_025344	Eif3f	eukaryotic translation initiation factor 3, subunit F	protein_coding	NP_079620.2	1913335	.	[illegible]
66086	NM_025345,XM_006522450,XM_017317110,XR_001781849	Fopnl	Fgfr1op N-terminal like	protein_coding	NP_079621.1;XP_006522513.1;XP_017172599.1	1913336	.	[illegible]
66087	NM_175101	Emc3	ER membrane protein complex subunit 3	protein_coding	NP_780310.1	1913337	.	[illegible]
66089	NM_025346	Rmnd5b	required for meiotic nuclear division 5 homolog B	protein_coding	NP_079622.1	1913339	.	[illegible]
66090	NM_025347,NM_026875,XM_030242883,XM_030242884	Ypel3	yippee like 3	protein_coding	NP_079623.1;NP_081151.2;XP_030098743.1;XP_030098744.1	1913340	.	[illegible]
66091	NM_025348,XM_030242885	Ndufa3	NADH:ubiquinone oxidoreductase subunit A3	protein_coding	NP_079624.1;XP_030098745.1	1913341	.	[illegible]
66092	NM_001199122,NM_001359902,NM_078478	Ghitm	growth hormone inducible transmembrane protein	protein_coding	NP_001186051.1;NP_001346831.1;NP_510963.1	1913342	.	[illegible]
66094	NM_001359148,NM_001359149,NM_001359150,NM_025349	Lsm7	LSM7 homolog, U6 small nuclear RNA and mRNA degradation associated	protein_coding	NP_001346077.1;NP_001346078.1;NP_001346079.1;NP_079625.1	1913344	.	[illegible]
66096	NM_001081108	Lamtor4	late endosomal/lysosomal adaptor, MAPK and MTOR activator 4	protein_coding	NP_001074577.1	1913346	.	[illegible]
66098	NM_001167736,NM_025351,XM_017321711	Chchd6	coiled-coil-helix-coiled-coil-helix domain containing 6	protein_coding	NP_001161208.1;NP_079627.3;XP_017177200.1	1913348	.	[illegible]
66101	NM_001110129,NM_001110130,NM_028677	Ppih	peptidyl prolyl isomerase H	protein_coding	NP_001103599.1;NP_001103600.1;NP_082953.1	106499	.	[illegible]
66102	NM_023158,NR_151496,NR_151497,NR_151499	Cxcl16	chemokine (C-X-C motif) ligand 16	protein_coding	NP_075647.3	1932682	.	[illegible]
66104	NM_025355	Tceal6	transcription elongation factor A (SII)-like 6	protein_coding	NP_079631.2	1923939	.	[illegible]
66105	NM_001356594,NM_001356595,NM_001356596,NM_001356597,NM_001356598,NM_025356	Ube2d3	ubiquitin-conjugating enzyme E2D 3	protein_coding	NP_001343523.1;NP_001343524.1;NP_001343525.1;NP_001343526.1;NP_001343527.1;NP_079632.1	1913355	.	[illegible]
66106	NM_001252591,NR_045569,NR_103722,XR_387086,XR_387087	Smpx	small muscle protein, X-linked	protein_coding	NP_001239520.1	1913356	.	[illegible]
66107	NM_183249	Wfdc21	WAP four-disulfide core domain 21	protein_coding	NP_899072.1	1913357	.	[illegible]
66108	NM_025358	Ndufa9	NADH:ubiquinone oxidoreductase subunit A9	protein_coding	NP_079634.2	1913358	.	[illegible]
66109	NM_025359	Tspan13	tetraspanin 13	protein_coding	NP_079635.1	1913359	.	[illegible]
66111	NM_025360	Tmed3	transmembrane p24 trafficking protein 3	protein_coding	NP_079636.2	1913361	.	[illegible]
66112	NM_001290273,XM_006497192	Marc1	mitochondrial amidoxime reducing component 1	protein_coding	NP_001277202.1;XP_006497255.1	1913362	.	[illegible]
66113	NM_001348095,NM_080434	Apoa5	apolipoprotein A-V	protein_coding	NP_001335024.1;NP_536682.2	1913363	.	[illegible]
66114	NM_025362	Dnajc30	DnaJ heat shock protein family (Hsp40) member C30	protein_coding	NP_079638.2	1913364	.	[illegible]
66116	NM_023160,XM_006506470	Nat8f1	N-acetyltransferase 8 (GCN5-related) family member 1	protein_coding	NP_075649.3;XP_006506533.1	1913366	.	[illegible]
66117	NM_025363	Fmc1	formation of mitochondrial complex V assembly factor 1	protein_coding	NP_079639.1	1913367	.	[illegible]
66118	NM_001359403,NM_001359404,NM_001359405,NM_001359406,NM_001359407,NM_025364,NR_153109	Sarnp	SAP domain containing ribonucleoprotein	protein_coding	NP_001346332.1;NP_001346333.1;NP_001346334.1;NP_001346335.1;NP_001346336.1;NP_079640.1	1913368	.	[illegible]
66119	NM_001164729,NM_025365	Tomm6	translocase of outer mitochondrial membrane 6	protein_coding	NP_001158201.1;NP_079641.1	1913369	.	[illegible]
66120	NM_001357893,NM_024169	Fkbp11	FK506 binding protein 11	protein_coding	NP_001344822.1;NP_077131.2	1913370	.	[illegible]
66121	NM_025366	Chchd1	coiled-coil-helix-coiled-coil-helix domain containing 1	protein_coding	NP_079642.1	1913371	.	[illegible]
66123	NR_027810	1110006O24Rik	RIKEN cDNA 1110006O24 gene	lncRNA	.	1913373	.	[illegible]
66124	NM_001205070,NM_001205071,NM_001205072,NM_001205073,NM_001360604,NM_025368,NR_153758,XM_006541071,XM_006541072,XM_006541074,XM_017312230,XM_030242887	Josd2	Josephin domain containing 2	protein_coding	NP_001191999.1;NP_001192000.1;NP_001192001.1;NP_001192002.1;NP_001347533.1;NP_079644.1;XP_006541134.1;XP_006541135.1;XP_006541137.1;XP_017167719.1;XP_030098747.1	1913374	.	[illegible]
66125	NM_175102	Sf3b5	splicing factor 3b, subunit 5	protein_coding	NP_780311.2	1913375	.	[illegible]
66126	NM_001357309,NM_001357310,NM_001357311,NM_001357312,NM_001357313,NM_001357314,NM_170777	Elof1	ELF1 homolog, elongation factor 1	protein_coding	NP_001344238.1;NP_001344239.1;NP_001344240.1;NP_001344241.1;NP_001344242.1;NP_001344243.1;NP_740747.1	1913376	.	[illegible]
66128	NM_001190264,NM_025369	Mrps36	mitochondrial ribosomal protein S36	protein_coding	NP_001177193.1;NP_079645.1	1913378	.	[illegible]
66129	NM_025370	Prxl2c	peroxiredoxin like 2C	protein_coding	NP_079646.1	1913379	.	[illegible]
66131	NM_001324556,NM_001324557,NM_001324558,NM_025372,NR_136926,XM_006511340,XM_030244489,XM_030244490,XM_030244491,XR_001778960,XR_003947966,XR_003947967	Tipin	timeless interacting protein	protein_coding	NP_001311485.1;NP_001311486.1;NP_001311487.1;NP_079648.1;XP_006511403.1;XP_030100349.1;XP_030100350.1;XP_030100351.1	1921571	.	[illegible]
66132	NM_025373,XR_001780473	Prorp	protein only RNase P catalytic subunit	protein_coding	NP_079649.1	1913382	.	[illegible]
66136	NM_001355422,NM_023162	Znrd1	zinc ribbon domain containing 1	protein_coding	NP_001342351.1;NP_075651.1	1913386	.	[illegible]
66138	NM_001363324,NM_001363325,NM_001363326,NM_001363327,NM_025375,XM_011240904,XM_030254751,XR_003955691	Bud23	BUD23, rRNA methyltransferase and ribosome maturation factor	protein_coding	NP_001350253.1;NP_001350254.1;NP_001350255.1;NP_001350256.1;NP_079651.2;XP_011239206.1;XP_030110611.1	1913388	.	[illegible]
66139	NM_001159602,NM_025376	Mymk	myomaker, myoblast fusion factor	protein_coding	NP_001153074.1;NP_079652.1	1913389	.	[illegible]
66140	NM_025377	Ska2	spindle and kinetochore associated complex subunit 2	protein_coding	NP_079653.1	1913390	.	[illegible]
66141	NM_025378	Ifitm3	interferon induced transmembrane protein 3	protein_coding	NP_079654.1	1913391	.	[illegible]
66142	NM_025379	Cox7b	cytochrome c oxidase subunit 7B	protein_coding	NP_079655.1	1913392	.	[illegible]
66143	NM_025380	Eef1e1	eukaryotic translation elongation factor 1 epsilon 1	protein_coding	NP_079656.1	1913393	.	[illegible]
66144	NM_025381	Atp6v1f	ATPase, H+ transporting, lysosomal V1 subunit F	protein_coding	NP_079657.1	1913394	.	[illegible]
66146	NM_001355499,NM_001355500,NM_001355501,NM_025382,XM_006539100,XR_001784176	Maco1	macoilin 1	protein_coding	NP_001342428.1;NP_001342429.1;NP_001342430.1;NP_079658.2;XP_006539163.1	1913396	.	[illegible]
66147	NM_025383,NR_149724,XR_001784178,XR_001784179,XR_003954994,XR_390777,XR_881436	Necap2	NECAP endocytosis associated 2	protein_coding	NP_079659.1	1913397	.	[illegible]
66148	NM_025384	Dnajc15	DnaJ heat shock protein family (Hsp40) member C15	protein_coding	NP_079660.1	1913398	.	[illegible]
66151	NM_001170911,NM_025385	Prr13	proline rich 13	protein_coding	NP_001164382.1;NP_079661.1	1913401	.	[illegible]
66152	NM_001362879,NM_197979,NR_156411	Uqcr10	ubiquinol-cytochrome c reductase, complex III subunit X	protein_coding	NP_001349808.1;NP_932096.1	1913402	.	[illegible]
66153	NM_025386,XM_006496520	Fbxo36	F-box protein 36	protein_coding	NP_079662.1;XP_006496583.1	1289192	.	[illegible]
66154	NM_001360770,NM_025387,XM_006516937,XM_006516938	Tmem14c	transmembrane protein 14C	protein_coding	NP_001347699.1;NP_079663.1;XP_006517000.3;XP_006517001.1	1913404	.	[illegible]
66155	NM_001360931,NM_001360932,NM_001360933,NM_025388	Ufc1	ubiquitin-fold modifier conjugating enzyme 1	protein_coding	NP_001347860.1;NP_001347861.1;NP_001347862.1;NP_079664.1	1913405	.	[illegible]
66156	NM_001038230,NM_025389	Anapc11	anaphase promoting complex subunit 11	protein_coding	NP_001033319.1;NP_079665.1	1913406	.	[illegible]
66158	NM_024170	Rtl8a	retrotransposon Gag like 8A	protein_coding	NP_077132.1	1913408	.	[illegible]
66161	NM_025390,XM_006540287	Pop4	processing of precursor 4, ribonuclease P/MRP family, (S. cerevisiae)	protein_coding	NP_079666.1;XP_006540350.1	1913411	.	[illegible]
66162	NM_175103	Bola2	bolA-like 2 (E. coli)	protein_coding	NP_780312.1	1913412	.	[illegible]
66163	NM_001357899,NM_023167,XM_006510540,XR_001778962,XR_003947968,XR_379129	Mrpl4	mitochondrial ribosomal protein L4	protein_coding	NP_001344828.1;NP_075656.2;XP_006510603.1	2137210	.	[illegible]
66164	NM_001164472,NM_025391,NR_028367	Nip7	NIP7, nucleolar pre-rRNA processing protein	protein_coding	NP_001157944.1;NP_079667.2	1913414	.	[illegible]
66165	NM_025392	Bccip	BRCA2 and CDKN1A interacting protein	protein_coding	NP_079668.2	1913415	.	[illegible]
66166	NM_001163525,NM_001163526,NM_025393	S100a14	S100 calcium binding protein A14	protein_coding	NP_001156997.1;NP_001156998.1;NP_079669.2	1913416	.	[illegible]
66167	NM_183250	Tma7	translational machinery associated 7	protein_coding	NP_899073.1	1913417	.	[illegible]
66168	NM_023168,XM_006521249,XM_006521250,XM_006521251,XM_011245709,XM_030248690	Grina	glutamate receptor, ionotropic, N-methyl D-aspartate-associated protein 1 (glutamate binding)	protein_coding	NP_075657.1;XP_006521312.1;XP_006521313.1;XP_006521314.1;XP_011244011.1;XP_030104550.1	1913418	.	[illegible]
66169	NM_025394	Tomm7	translocase of outer mitochondrial membrane 7	protein_coding	NP_079670.1	1913419	.	[illegible]
66170	NM_025395	Chchd5	coiled-coil-helix-coiled-coil-helix domain containing 5	protein_coding	NP_079671.1	1913420	.	[illegible]
66171	NM_001294269,NM_025396	Pgls	6-phosphogluconolactonase	protein_coding	NP_001281198.1;NP_079672.1	1913421	.	[illegible]
66172	NM_025397	Med11	mediator complex subunit 11	protein_coding	NP_079673.2	1913422	.	[illegible]
66174	NM_001316724,NM_025399	Nudt14	nudix (nucleoside diphosphate linked moiety X)-type motif 14	protein_coding	NP_001303653.1;NP_079675.1	1913424	.	[illegible]
66175	NM_181390	Mustn1	musculoskeletal, embryonic nuclear protein 1	protein_coding	NP_852055.1	1913425	.	[illegible]
66176	NM_001362889,NM_001362890,NM_001362891,NM_001362892,NM_025400	Nat9	N-acetyltransferase 9 (GCN5-related, putative)	protein_coding	NP_001349818.1;NP_001349819.1;NP_001349820.1;NP_001349821.1;NP_079676.1	1913426	.	[illegible]
66177	NM_001310746,NM_001361025,NM_001361026,NM_001361027,NM_025401,NR_153857,NR_153858,NR_153859,XM_017313510	Ubl5	ubiquitin-like 5	protein_coding	NP_001297675.1;NP_001347954.1;NP_001347955.1;NP_001347956.1;NP_079677.1;XP_017168999.1	1913427	.	[illegible]
66179	NM_025402	Ogfod3	2-oxoglutarate and iron-dependent oxygenase domain containing 3	protein_coding	NP_079678.1	1913429	.	[illegible]
66180	NM_176830,XM_030246219	P3h4	prolyl 3-hydroxylase family member 4 (non-enzymatic)	protein_coding	NP_789800.1;XP_030102079.1	1913430	.	[illegible]
66181	NM_025403	Nop10	NOP10 ribonucleoprotein	protein_coding	NP_079679.1	1913431	.	[illegible]
66183	NM_001164210,NM_001286959,NM_133675,XM_030252751	Sptssb	serine palmitoyltransferase, small subunit B	protein_coding	NP_001157682.1;NP_001273888.1;NP_598436.1;XP_030108611.1	1913433	.	[illegible]
66184	NR_003634	Rps4l	ribosomal protein S4-like	transcribed_pseudogene	.	1913434	.	[illegible]
66185	NM_001081183,NM_001347055	Virma	vir like m6A methyltransferase associated	protein_coding	NP_001074652.1;NP_001333984.1	1913435	.	[illegible]
66190	NM_001360615,NM_001360616,NM_001360617,NM_025408,NR_153759,XM_030242889	Acer3	alkaline ceramidase 3	protein_coding	NP_001347544.1;NP_001347545.1;NP_001347546.1;NP_079684.2;XP_030098749.1	1913440	.	[illegible]
66191	NM_025409	Ier3ip1	immediate early response 3 interacting protein 1	protein_coding	NP_079685.1	1913441	.	[illegible]
66192	NM_025410	Lage3	L antigen family, member 3	protein_coding	NP_079686.1	1913442	.	[illegible]
66193	NM_025411	Pithd1	PITH (C-terminal proteasome-interacting domain of thioredoxin-like) domain containing 1	protein_coding	NP_079687.3	1913443	.	[illegible]
66194	NM_025412,XM_006521252	Pycrl	pyrroline-5-carboxylate reductase-like	protein_coding	NP_079688.2;XP_006521315.1	1913444	.	[illegible]
66196	NM_001362893,NM_025414,XM_006533930,XM_011249173,XM_011249174,XM_017314704,XM_030246220,XM_030246221,XM_030246222,XR_003949489,XR_879727	Myo19	myosin XIX	protein_coding	NP_001349822.1;NP_079690.2;XP_006533993.1;XP_011247475.1;XP_011247476.1;XP_017170193.1;XP_030102080.1;XP_030102081.1;XP_030102082.1	1913446	.	[illegible]
66197	NM_025415	Cks2	CDC28 protein kinase regulatory subunit 2	protein_coding	NP_079691.1	1913447	.	[illegible]
66198	NM_025416	Them5	thioesterase superfamily member 5	protein_coding	NP_079692.1	1913448	.	[illegible]
66199	NM_025417,XM_006511343,XR_003947969,XR_003947970,XR_003947971,XR_379435,XR_379436	Commd4	COMM domain containing 4	protein_coding	NP_079693.1;XP_006511406.1	1913449	.	[illegible]
66200	NM_001033132,NM_001168592	Commd6	COMM domain containing 6	protein_coding	NP_001028304.1;NP_001162064.1	1913450	.	[illegible]
66201	NM_025418,XM_006512809	Vta1	vesicle (multivesicular body) trafficking 1	protein_coding	NP_079694.2;XP_006512872.1	1913451	.	[illegible]
66202	NM_025419	1110059G10Rik	RIKEN cDNA 1110059G10 gene	protein_coding	NP_079695.2	1913452	.	[illegible]
66203	NM_001355725,NM_025420	Lce1m	late cornified envelope 1M	protein_coding	NP_001342654.1;NP_079696.1	1913453	.	[illegible]
66204	NM_001364360,NM_001364361,NM_025421	Acyp1	acylphosphatase 1, erythrocyte (common) type	protein_coding	NP_001351289.1;NP_001351290.1;NP_079697.1	1913454	.	[illegible]
66205	NM_001290660,NM_025422	Cd302	CD302 antigen	protein_coding	NP_001277589.1;NP_079698.2	1913455	.	[illegible]
66206	NM_025423	1110059E24Rik	RIKEN cDNA 1110059E24 gene	protein_coding	NP_079699.1	1913456	.	[illegible]
66208	NM_025424	Nenf	neuron derived neurotrophic factor	protein_coding	NP_079700.1	1913458	.	[illegible]
66209	NM_001013577,XM_030253695	Inip	INTS3 and NABP interacting protein	protein_coding	NP_001013595.1;XP_030109555.1	1913459	.	[illegible]
66211	NM_001163945,NM_025425,XM_011246569,XM_017317609,XM_030249982	Rpl3l	ribosomal protein L3-like	protein_coding	NP_001157417.1;NP_079701.1;XP_011244871.1;XP_017173098.1;XP_030105842.1	1913461	.	[illegible]
66212	NM_024171	Sec61b	Sec61 beta subunit	protein_coding	NP_077133.1	1913462	.	[illegible]
66213	NM_001104530,NM_001104556,NM_001104557,NM_025426,XM_006533931,XM_006533932,XM_006533933,XM_006533934	Med7	mediator complex subunit 7	protein_coding	NP_001098000.1;NP_001098026.1;NP_001098027.1;NP_079702.3;XP_006533994.1;XP_006533995.1;XP_006533996.1;XP_006533997.1	1913463	.	[illegible]
66214	NM_025427	Rgcc	regulator of cell cycle	protein_coding	NP_079703.2	1913464	.	[illegible]
66218	NM_001364808,NM_023172	Ndufb9	NADH:ubiquinone oxidoreductase subunit B9	protein_coding	NP_001351737.1;NP_075661.1	1913468	.	[illegible]
66220	NM_001037762,NM_025428,XM_030251938	Zdhhc12	zinc finger, DHHC domain containing 12	protein_coding	NP_001032851.1;NP_079704.2;XP_030107798.1	1913470	.	[illegible]
66222	NM_025429,XM_006516724,XM_030247348	Serpinb1a	serine (or cysteine) peptidase inhibitor, clade B, member 1a	protein_coding	NP_079705.2;XP_006516787.1;XP_030103208.1	1913472	.	[illegible]
66223	NM_025430	Mrpl35	mitochondrial ribosomal protein L35	protein_coding	NP_079706.1	1913473	.	[illegible]
66225	NM_025431,XM_006513954	Llph	LLP homolog, long-term synaptic facilitation (Aplysia)	protein_coding	NP_079707.1;XP_006514017.1	1913475	.	[illegible]
66226	NM_001313722,NM_025432,XR_003953020	Trappc2	trafficking protein particle complex 2	protein_coding	NP_001300651.1;NP_079708.2	1913476	.	[illegible]
66229	NM_025433	Rpl7l1	ribosomal protein L7-like 1	protein_coding	NP_079709.2	1913479	.	[illegible]
66230	NM_025434	Mrps28	mitochondrial ribosomal protein S28	protein_coding	NP_079710.3	1913480	.	[illegible]
66231	NM_001285780,NM_001359900,NM_001359901,NM_025435,XM_006518076,XM_006518078,XM_011244769,XM_017316120,XM_030247922,XM_030247923,XM_030247924,XM_030247925,XM_030247926,XM_030247927,XM_030247928,XM_030247929,XM_030247931,XR_003950878,XR_003950879,XR_003950880,XR_382964	Thoc7	THO complex 7	protein_coding	NP_001272709.1;NP_001346829.1;NP_001346830.1;NP_079711.1;XP_006518139.1;XP_006518141.1;XP_011243071.1;XP_017171609.1;XP_030103782.1;XP_030103783.1;XP_030103784.1;XP_030103785.1;XP_030103786.1;XP_030103787.1;XP_030103788.1;XP_030103789.1;XP_030103791.1	1913481	.	[illegible]
66233	NM_023178,XM_006503266,XM_006503267,XM_006503268	Dmap1	DNA methyltransferase 1-associated protein 1	protein_coding	NP_075667.1;XP_006503329.1;XP_006503330.1;XP_006503331.1	1913483	.	[illegible]
66234	NM_025436	Msmo1	methylsterol monoxygenase 1	protein_coding	NP_079712.1	1913484	.	[illegible]
66235	NM_025437	Eif1ax	eukaryotic translation initiation factor 1A, X-linked	protein_coding	NP_079713.2	1913485	.	[illegible]
66236	NR_027817,NR_027818	1500011B03Rik	RIKEN cDNA 1500011B03 gene	lncRNA	.	1913486	.	[illegible]
66237	NM_001347351,NM_023179,XM_006524795	Atp6v1g2	ATPase, H+ transporting, lysosomal V1 subunit G2	protein_coding	NP_001334280.1;NP_075668.1;XP_006524858.1	1913487	.	[illegible]
66240	NM_021487	Kcne1l	potassium voltage-gated channel, Isk-related family, member 1-like, pseudogene	protein_coding	NP_067462.1	1913490	.	[illegible]
66241	NM_001160145,NM_001160146,NM_025439	Tmem9	transmembrane protein 9	protein_coding	NP_001153617.1;NP_001153618.1;NP_079715.1	1913491	.	[illegible]
66242	NM_025440,NR_155747	Mrps16	mitochondrial ribosomal protein S16	protein_coding	NP_079716.1	1913492	.	[illegible]
66244	NM_025441,XM_011244157,XM_030246848	Nemf	nuclear export mediator factor	protein_coding	NP_079717.2;XP_011242459.1;XP_030102708.1	1918305	.	[illegible]
66245	NM_001360629,NM_024172,XM_030242890	Hspbp1	HSPA (heat shock 70kDa) binding protein, cytoplasmic cochaperone 1	protein_coding	NP_001347558.1;NP_077134.1;XP_030098750.1	1913495	.	[illegible]
66246	NM_133676	Osgep	O-sialoglycoprotein endopeptidase	protein_coding	NP_598437.2	1913496	.	[illegible]
66248	NM_025442	Alg5	asparagine-linked glycosylation 5 (dolichyl-phosphate beta-glucosyltransferase)	protein_coding	NP_079718.1	1913498	.	[illegible]
66249	NM_025443	Pno1	partner of NOB1 homolog	protein_coding	NP_079719.1	1913499	.	[illegible]
66251	NM_001357756,NM_001357757,NM_025445,XM_006521254	Arfgap3	ADP-ribosylation factor GTPase activating protein 3	protein_coding	NP_001344685.1;NP_001344686.1;NP_079721.2;XP_006521317.2	1913501	.	[illegible]
66253	NM_001347362,NM_025446,XM_006512812,XM_030245214,XM_030245215,XM_030245216,XM_030245217,XR_003948769	Aig1	androgen-induced 1	protein_coding	NP_001334291.1;NP_079722.1;XP_006512875.1;XP_030101074.1;XP_030101075.1;XP_030101076.1;XP_030101077.1	1913503	.	[illegible]
66254	NM_025447,XM_006517735,XR_003950461	Dimt1	DIM1 dimethyladenosine transferase 1-like (S. cerevisiae)	protein_coding	NP_079723.1;XP_006517798.1	1913504	.	[illegible]
66255	NM_001136181,XM_006526517,XM_006526518	Hsbp1l1	heat shock factor binding protein 1-like 1	protein_coding	NP_001129653.1;XP_006526580.1;XP_006526581.1	1913505	.	[illegible]
66256	NM_001356316,NM_001356317,NM_001356318,NM_001356319,NM_025448	Ssr2	signal sequence receptor, beta	protein_coding	NP_001343245.1;NP_001343246.1;NP_001343247.1;NP_001343248.1;NP_079724.1	1913506	.	[illegible]
66257	NM_001364505,NM_025449	Nicn1	nicolin 1	protein_coding	NP_001351434.1;NP_079725.1	1913507	.	[illegible]
66258	NM_001356954,NM_001356955,NM_001356956,NM_001356957,NM_001356958,NM_001356959,NM_025450,NR_151584,XM_030254752,XM_030254753	Mrps17	mitochondrial ribosomal protein S17	protein_coding	NP_001343883.1;NP_001343884.1;NP_001343885.1;NP_001343886.1;NP_001343887.1;NP_001343888.1;NP_079726.1;XP_030110612.1;XP_030110613.1	1913508	.	[illegible]
66259	NM_025451	Camk2n1	calcium/calmodulin-dependent protein kinase II inhibitor 1	protein_coding	NP_079727.1	1913509	.	[illegible]
66262	NM_025454,XM_006529792	Ing5	inhibitor of growth family, member 5	protein_coding	NP_079730.1;XP_006529855.1	1922816	.	[illegible]
66263	NR_015572	1810014B01Rik	RIKEN cDNA 1810014B01 gene	transcribed_pseudogene	.	1913513	.	[illegible]
66264	NM_025455,XM_030253696	Ccdc28b	coiled coil domain containing 28B	protein_coding	NP_079731.2;XP_030109556.1	1913514	.	[illegible]
66266	NM_001331162,NM_025456,XM_006516142,XM_030246849	Eapp	E2F-associated phosphoprotein	protein_coding	NP_001318091.1;NP_079732.1;XP_006516205.1;XP_030102709.1	1913516	.	[illegible]
66268	NM_001082532	Pigyl	phosphatidylinositol glycan anchor biosynthesis, class Y-like	protein_coding	NP_001076001.1	1913518	.	[illegible]
66269	NM_025458	Tmed6	transmembrane p24 trafficking protein 6	protein_coding	NP_079734.1	1913519	.	[illegible]
66270	NM_001034851,NM_001277315,NM_001277316,NM_001277317,NM_001277318,NM_025459,XM_006520134,XM_017316720	Retreg1	reticulophagy regulator 1	protein_coding	NP_001030023.1;NP_001264244.1;NP_001264245.1;NP_001264246.1;NP_001264247.1;NP_079735.2;XP_006520197.1;XP_017172209.1	1913520	.	[illegible]
66271	NM_025460,NR_157050	Tmem126a	transmembrane protein 126A	protein_coding	NP_079736.1	1913521	.	[illegible]
66272	NM_001309808,NM_001309809,NM_001309810,NM_025461,NR_132134,NR_132135	Cox16	cytochrome c oxidase assembly protein 16	protein_coding	NP_001296737.1;NP_001296738.1;NP_001296739.1;NP_079737.1	1913522	.	[illegible]
66273	NM_001177945,NM_001177946,NM_001177947,NM_183251,XM_006508114	Aamdc	adipogenesis associated Mth938 domain containing	protein_coding	NP_001171416.1;NP_001171417.1;NP_001171418.1;NP_899074.2;XP_006508177.1	1913523	.	[illegible]
66274	NM_001076681	Lyrm9	LYR motif containing 9	protein_coding	NP_001070149.1	1913524	.	[illegible]
66276	NM_025463,NR_104416	1810009A15Rik	RIKEN cDNA 1810009A15 gene	protein_coding	NP_079739.1	1913526	.	[illegible]
66277	NM_001355668,NM_023184,XM_006506471,XM_006506472,XM_011241425,XM_030255539,XR_003956248,XR_003956249,XR_377	Klf15	Kruppel-like factor 15	protein_coding	NP_001342597.1;NP_075673.1;XP_006506534.1;XP_006506535.1;XP_011239727.1;XP_030111399.1	1929988	.	[illegible]
66278	NM_001145433	Smim20	small integral membrane protein 20	protein_coding	NP_001138905.1	1913528	.	[illegible]
66279	NM_001310096,NM_025464,XM_006510546	Tmem218	transmembrane protein 218	protein_coding	NP_001297025.1;NP_079740.1;XP_006510609.1	1913529	.	[illegible]
66282	NM_001357208,NM_025465	Tma16	translation machinery associated 16	protein_coding	NP_001344137.1;NP_079741.1	1913532	.	[illegible]
66283	NM_025466	Gkn1	gastrokine 1	protein_coding	NP_079742.1	1913533	.	[illegible]
66286	NM_025468,XM_006526150	Sec11c	SEC11 homolog C, signal peptidase complex subunit	protein_coding	NP_079744.1;XP_006526213.1	1913536	.	[illegible]
66290	NM_024173	Atp6v1g1	ATPase, H+ transporting, lysosomal V1 subunit G1	protein_coding	NP_077135.1	1913540	.	[illegible]
66291	NM_001330755,NM_001330756,NM_001330757,NM_025471,NR_138487,XM_006538163	Smim8	small integral membrane protein 8	protein_coding	NP_001317684.1;NP_001317685.1;NP_001317686.1;NP_079747.1;XP_006538226.1	1913541	.	[illegible]
66292	NM_001355759,NM_078479	Mrps21	mitochondrial ribosomal protein S21	protein_coding	NP_001342688.1;NP_510964.1	1913542	.	[illegible]
66293	NR_027819,NR_027820,NR_027821	Snhg16	small nucleolar RNA host gene 16	lncRNA	.	1913543	.	[illegible]
66294	NM_001313725,NM_025473,XM_011247660,XM_030251474,XM_030251475	Fam3a	family with sequence similarity 3, member A	protein_coding	NP_001300654.1;NP_079749.1;XP_011245962.1;XP_030107334.1;XP_030107335.1	1913544	.	[illegible]
66296	NM_001290807,NM_001290808,NM_025475	Haus2	HAUS augmin-like complex, subunit 2	protein_coding	NP_001277736.1;NP_001277737.1;NP_079751.1	1913546	.	[illegible]
66297	NR_027826	Pantr1	POU domain, class 3, transcription factor 3 adjacent noncoding	lncRNA	.	1913547	.	[illegible]
66300	NM_001136270	Inafm1	InaF motif containing 1	protein_coding	NP_001129742.1	1913550	.	[illegible]
66302	NM_025476,XR_001784180	Rmdn1	regulator of microtubule dynamics 1	protein_coding	NP_079752.3	1913552	.	[illegible]
66306	NM_001357456,NM_001357457,NM_175104,XM_006526152,XM_006526154	Fam53c	family with sequence similarity 53, member C	protein_coding	NP_001344385.1;NP_001344386.1;NP_780313.1;XP_006526215.1;XP_006526217.1	1913556	.	[illegible]
66307	NM_025478	Isoc1	isochorismatase domain containing 1	protein_coding	NP_079754.2	1913557	.	[illegible]
66308	NM_025479	Mplkip	M-phase specific PLK1 intereacting protein	protein_coding	NP_079755.1	1913558	.	[illegible]
66309	NM_001356960,NM_025480	Tmem128	transmembrane protein 128	protein_coding	NP_001343889.1;NP_079756.2	1913559	.	[illegible]
66310	NM_001146222,NM_001146223,NM_001146224,NM_024428,XM_030249983	Dpy30	dpy-30, histone methyltransferase complex regulatory subunit	protein_coding	NP_001139694.1;NP_001139695.1;NP_001139696.1;NP_077746.3;XP_030105843.1	1913560	.	[illegible]
66311	NM_001109747,XM_006512814	Cenpw	centromere protein W	protein_coding	NP_001103217.1;XP_006512877.1	1913561	.	[illegible]
66313	NM_001362894,NM_025481,XM_006533936,XR_001780039	Smurf2	SMAD specific E3 ubiquitin protein ligase 2	protein_coding	NP_001349823.1;NP_079757.2;XP_006533999.1	1913563	.	[illegible]
66314	NM_001291197,NM_001291200,NM_001291201,NM_001291202,NM_001291203,NM_001291204,NM_025482,NR_111899,XM_006500693,XM_006500694,XM_006500696,XM_006500697,XM_0065007	Tpd52l2	tumor protein D52-like 2	protein_coding	NP_001278126.1;NP_001278129.1;NP_001278130.1;NP_001278131.1;NP_001278132.1;NP_001278133.1;NP_079758.2;XP_006500756.1;XP_006500757.1;XP_006500759.1;XP_006500760.1;XP_006500764.	1913564	.	[illegible]

66315	NM_001003972,NM_001003973,NM_001304503,NM_001360073,NM_025483,XM_006522451,XM_006522452,XM_006522454,XM_006522455,XM_017317111,XM_030249230,XM_030249231,XM_030249232,XR_003951814	Senp7	SUMO1/sentrin specific peptidase 7	protein_coding	NP_001003972.1;NP_001003973.1;NP_001291432.1;NP_001347002.1;NP_079759.2;XP_006522514.1;XP_006522515.1;XP_006522517.1;XP_006522518.1;XP_017172600.1;XP_030105090.1;XP_030105091.1;XP_030105092.1	1913565	.	7.132733	[illegible]
66317	NM_001025375,NM_001025376,NM_023191,XM_011242785	Wdr61	WD repeat domain 61	protein_coding	NP_001020546.1;NP_001020547.1;NP_075680.1;XP_011241087.1	1917493	.	23.911709	[illegible]
66320	NM_025486,XM_006531274,XM_006531277,XR_003947324,XR_003947325	Tmem208	transmembrane protein 208	protein_coding	NP_079762.1;XP_006531337.1;XP_006531340.1	1913570	.	26.764450	[illegible]
66326	NM_001163536,NM_001163537,NM_025489,XM_006535515,XM_006535516	Dnajc5b	DnaJ heat shock protein family (Hsp40) member C5 beta	protein_coding	NP_001157008.1;NP_001157009.1;NP_079765.3;XP_006535578.1;XP_006535579.1	1913576	.	0.230977	[illegible]
66329	NM_001360772,NM_025491,NM_028340,XM_006516940	Susd3	sushi domain containing 3	protein_coding	NP_001347701.1;NP_079767.2;NP_082616.1;XP_006517003.1	1913579	.	0.345105	[illegible]
66330	NM_025492	1700020L24Rik	RIKEN cDNA 1700020L24 gene	protein_coding	NP_079768.2	1913580	.	0.273666	[illegible]
66333	NM_175105	Aqp11	aquaporin 11	protein_coding	NP_780314.1	1913583	.	1.241375	[illegible]
66335	NM_025494	Atp6v1c1	ATPase, H+ transporting, lysosomal V1 subunit C1	protein_coding	NP_079770.2	1913585	.	16.967257	[illegible]
66336	NM_025495,XM_011244405,XM_011244406,XM_011244407,XM_017315560,XM_030247350,XR_003950462,XR_873273	Cenpp	centromere protein P	protein_coding	NP_079771.1;XP_011242707.1;XP_011242708.1;XP_011242709.1;XP_017171049.1;XP_030103210.1	1913586	.	3.600894	[illegible]
66337	NM_001359440,NM_001359441,NM_001359442,NM_001359443,NM_001359444,NM_183254,NR_153111	Fam229b	family with sequence similarity 229, member B	protein_coding	NP_001346369.1;NP_001346370.1;NP_001346371.1;NP_001346372.1;NP_001346373.1;NP_899077.1	1913587	.	3.278213	[illegible]
66338	NM_025496	Cdrt4	CMT1A duplicated region transcript 4	protein_coding	NP_079772.1	1913588	.	0.098618	[illegible]
66340	NM_001364016,NM_001364017,NM_025498	Psenen	presenilin enhancer gamma secretase subunit	protein_coding	NP_001350945.1;NP_001350946.1;NP_079774.1	1913590	.	36.694802	[illegible]
66341	NM_025499	Eid3	EP300 interacting inhibitor of differentiation 3	protein_coding	NP_079775.2	1913591	.	0.031991	[illegible]
66343	NM_175106	Tmem177	transmembrane protein 177	protein_coding	NP_780315.1	1913593	.	1.337306	[illegible]
66349	NM_001290487,NM_025504	Dmac2	distal membrane arm assembly complex 2	protein_coding	NP_001277416.1;NP_079780.2	1913599	.	8.147261	[illegible]
66350	NM_001286948,NM_023196,NM_183423,NR_104611	Pla2g12a	phospholipase A2, group XIIA	protein_coding	NP_001273877.1;NP_075685.2;NP_904359.1	1913600	.	8.132342	[illegible]
66352	NM_001160208,NM_001160209,NM_001357052,NM_025505	Blzf1	basic leucine zipper nuclear factor 1	protein_coding	NP_001153680.1;NP_001153681.1;NP_001343981.1;NP_079781.2	1201607	.	7.998901	[illegible]
66353	NM_025506,XM_011240190	Riiad1	regulatory subunit of type II PKA R-subunit (RIIa) domain containing 1	protein_coding	NP_079782.1;XP_011238492.1	1913603	.	1.118174	[illegible]
66354	NM_025507	Snw1	SNW domain containing 1	protein_coding	NP_079783.2	1913604	.	27.105995	[illegible]
66355	NM_025508,XM_017315561,XM_017315562,XM_030247352	Gmpr	guanosine monophosphate reductase	protein_coding	NP_079784.1;XP_017171050.1;XP_017171051.1;XP_030103212.1	1913605	.	3.914874	[illegible]
66356	NM_001168218,NM_001168219,NM_001168220,NM_023197	Knop1	lysine rich nucleolar protein 1	protein_coding	NP_001161690.1;NP_001161691.1;NP_001161692.1;NP_075686.2	1913606	.	9.642730	[illegible]
66357	NM_025509	Ostc	oligosaccharyltransferase complex subunit (non-catalytic)	protein_coding	NP_079785.1	1913607	.	150.158997	[illegible]
[illegible]	[illegible]	[illegible]	[illegible]	[illegible]	[illegible]	[illegible]	[illegible]	[illegible]	[illegible]

66609	NM_026994,NM_133679,XM_030249235,XM_030249236,XM_030249237,XM_030249238,XR_003951815,XR_003951816,XR_003951817,XR_003951818	Cryzl1	crystallin, zeta (quinone reductase)-like 1	protein_coding	NP_081270.1;NP_598440.1;XP_030105095.1;XP_030105096.1;XP_030105097.1;XP_030105098.1	1913859	.	13.040079	[illegible]
66610	NM_001163464,NM_025659,XM_006533956,XM_017314707,XM_017314708,XM_017314709,XM_017314710,XM_030246224,XM_030246225,XM_030246226,XM_030246227,XM_030246228,XM_030246229	Abi3	ABI gene family, member 3	protein_coding	NP_001156936.1;NP_079935.1;XP_006534019.1;XP_017170196.1;XP_017170197.1;XP_017170198.1;XP_017170199.1;XP_030102084.1;XP_030102085.1;XP_030102086.1;XP_030102087.1;XP_030102088.1;XP_030102089.1	1913860	.	4.460576	[illegible]
66611	NM_025660,XM_006528965,XM_006528966,XM_006528967	Ribc1	RIB43A domain with coiled-coils 1	protein_coding	NP_079936.1;XP_006529028.1;XP_006529029.1;XP_006529030.1	1913861	.	0.426988	[illegible]
66612	NM_025661	Ormdl3	ORM1-like 3 (S. cerevisiae)	protein_coding	NP_079937.1	1913862	.	36.382816	[illegible]
66614	NM_001110809,NM_001286857,NM_025663	Gpatch4	G patch domain containing 4	protein_coding	NP_001104279.1;NP_001273786.1;NP_079939.3	1913864	.	8.013388	[illegible]
66615	NM_001368266,NM_174874,XM_006529793	Atg4b	autophagy related 4B, cysteine peptidase	protein_coding	NP_001355195.1;NP_777363.1;XP_006529856.1	1913865	.	13.571463	[illegible]
66616	NM_025664,XM_017317612,XM_030249990	Snx9	sorting nexin 9	protein_coding	NP_079940.2;XP_017173101.1;XP_030105850.1	1913866	.	19.693831	[illegible]
66617	NM_001356433,NM_001356434,NM_001356435,NM_001356436,NM_001356437,NM_170592,NR_151467,NR_151468,XM_006498228,XM_030251953	Ntmt1	N-terminal Xaa-Pro-Lys N-methyltransferase 1	protein_coding	NP_001343362.1;NP_001343363.1;NP_001343364.1;NP_001343365.1;NP_001343366.1;NP_733480.1;XP_006498291.1;XP_030107813.1	1913867	.	14.365385	[illegible]
66618	NM_025665	Snrnp27	small nuclear ribonucleoprotein 27 (U4/U6.U5)	protein_coding	NP_079941.1	1913868	.	28.836735	[illegible]
66620	NR_110495	Pantr2	POU domain, class 3, transcription factor 3 adjacent noncoding	lncRNA		1913870	.	0.000000	[illegible]
66622	NM_025666	Ubr7	ubiquitin protein ligase E3 component n-recognin 7 (putative)	protein_coding	NP_079942.1	1913872	.	13.421686	[illegible]
66624	NM_025668,XM_006508117	Spcs2	signal peptidase complex subunit 2 homolog (S. cerevisiae)	protein_coding	NP_079944.1;XP_006508180.1	1913874	.	30.637415	[illegible]
66625	NM_001369189,NM_001369190,NM_001369191,NM_025669,XM_006538184,XM_017320353,XM_030253702,XR_001784181	Pnisr	PNN interacting serine/arginine-rich	protein_coding	NP_001356118.1;NP_001356119.1;NP_001356120.1;NP_079945.1;XP_006538247.1;XP_017175842.1;XP_030109562.1	1913875	.	45.121414	[illegible]
66626	NM_001358078,NM_001358079,NM_001358080,NM_001358081,NM_001358082,NM_025670,XM_006522468,XM_030249239,XM_030249240	Cdip1	cell death inducing Trp53 target 1	protein_coding	NP_001345007.1;NP_001345008.1;NP_001345009.1;NP_001345010.1;NP_001345011.1;NP_079946.2;XP_006522531.1;XP_030105099.1;XP_030105100.1	1913876	.	20.611452	[illegible]
66627	NM_001347478,NM_001361482,NM_001361483,NM_025671,XM_030254762	Ogfod2	2-oxoglutarate and iron-dependent oxygenase domain containing 2	protein_coding	NP_001334407.1;NP_001348411.1;NP_001348412.1;NP_079947.1;XP_030110622.1	1913877	.	6.308404	[illegible]
66628	NM_001080969,NM_001290737,XM_011249179,XM_030246230	Thg1l	tRNA-histidine guanylyltransferase 1-like (S. cerevisiae)	protein_coding	NP_001074438.1;NP_001277666.1;XP_011247481.1;XP_030102090.	1913878	.	2.225073	[illegible]
66629	NM_025673,XM_006520143	Golph3	golgi phosphoprotein 3	protein_coding	NP_079949.1;XP_006520206.1	1913879	.	60.117563	[illegible]
66631	NM_001083901,NM_133680,XM_011244550	Mfsd14b	major facilitator superfamily domain containing 14B	protein_coding	NP_001077370.1;NP_598441.3;XP_011242852.1	1913881	.	16.370493	[illegible]
66632	NM_001356438,NM_001356439,NM_025675,XM_006500019,XR_866334	Dph6	diphthamine biosynthesis 6	protein_coding	NP_001343367.1;NP_001343368.1;NP_079951.1;XP_006500082.1	1913882	.	8.068870	[illegible]
66634	NM_001291054,NM_025676,XR_003953691,XR_374505	Mcm8	minichromosome maintenance 8 homologous recombination repair factor	protein_coding	NP_001277983.1;NP_079952.2	1913884	.	1.237036	[illegible]
66637	NM_025677	Tsen15	tRNA splicing endonuclease subunit 15	protein_coding	NP_079953.2	1913887	.	9.624961	[illegible]
66641	NM_025679	Sike1	suppressor of IKBKE 1	protein_coding	NP_079955.1	1913891	.	12.145610	[illegible]
66642	NM_025680,XM_006500022	Ctnnbl1	catenin, beta like 1	protein_coding	NP_079956.3;XP_006500085.1	1913892	.	8.143825	[illegible]
66643	NM_025681,XM_006524797	Lix1	limb and CNS expressed 1	protein_coding	NP_079957.2;XP_006524860.1	1913893	.	0.206643	[illegible]
66645	NM_001360420,NM_025682	Pspc1	paraspeckle protein 1	protein_coding	NP_001347349.1;NP_079958.3	1913895	.	9.278704	[illegible]
66646	NM_001310642,NM_001310643,NM_001310644,NM_025683,XM_006496188,XM_006496191,XM_030242101,XM_030242120	Rpe	ribulose-5-phosphate-3-epimerase	protein_coding	NP_001297571.1;NP_001297572.1;NP_001297573.1;NP_079959.2;XP_006496251.1;XP_006496254.1;XP_030097961.1;XP_030097980.1	1913896	.	7.037126	[illegible]
66647	NM_023239	Nsmce3	NSE3 homolog, SMC5-SMC6 complex component	protein_coding	NP_075728.1	1913897	.	9.768314	[illegible]
66648	NM_001004361,NM_001142697,NM_001142698,XM_006526156,XR_003952537	Tpgs2	tubulin polyglutamylase complex subunit 2	protein_coding	NP_001004361.2;NP_001136169.1;NP_001136170.1;XP_006526219.1	1913898	.	6.475342	[illegible]
66650	NM_025684,XM_006512818,XM_006512819	Nepn	nephrocan	protein_coding	NP_079960.1;XP_006512881.1;XP_006512882.1	1913900	.	0.000000	[illegible]
66653	NM_025686,XM_006509180,XM_006509181	Brf2	BRF2, RNA polymerase III transcription initiation factor 50kDa subunit	protein_coding	NP_079962.1;XP_006509243.1;XP_006509244.1	1913903	.	3.320310	[illegible]
66654	NM_025687	Tex12	testis expressed 12	protein_coding	NP_079963.1	1913904	.	0.000000	[illegible]
66656	NM_001285429,NM_001285430,NM_001285431,NM_001285432,NM_001285433,NM_001285434,NM_023240,NM_029663,XM_006521261,XM_006521262,XM_006521264,XM_006521265,XM_006521266,XM_006521267,XM_006521269,XM_006521271,XM_006521272,XM_006521273,XM_006521274,XM_006521275,XM_017316725,XM_030248699,XM_030248701	Eef1d	eukaryotic translation elongation factor 1 delta (guanine nucleotide exchange protein)	protein_coding	NP_001272358.1;NP_001272359.1;NP_001272360.1;NP_001272361.1;NP_001272362.1;NP_001272363.1;NP_075729.2;NP_083939.1;XP_006521324.2;XP_006521325.2;XP_006521327.2;XP_006521328.2;XP_006521329.2;XP_006521330.1;XP_006521332.1;XP_006521334.1;XP_006521335.1;XP_006521336.1;XP_006521337.1;XP_006521338.1;XP_017172214.1;XP_030104559.1;XP_030104561.1	1913906	.	86.472355	[illegible]
66658	NM_025689	Ccdc51	coiled-coil domain containing 51	protein_coding	NP_079965.2	1913908	.	1.760593	[illegible]
66659	NM_019800,XR_003954368,XR_375566	Acp6	acid phosphatase 6, lysophosphatidic	protein_coding	NP_062774.2	1931010	.	7.280143	[illegible]
66660	NM_001357911,NM_025690,NM_026337,NR_152142,XM_006511349,XM_006511350,XM_006511351,XM_006511352,XM_006511354,XM_006511355,XM_011242786,XM_030244496,XM_030244497,XM_030244498,XM_030244499,XM_030244500,XM_030244501,XR_001778967,XR_001778968	Sltm	SAFB-like, transcription modulator	protein_coding	NP_001344840.1;NP_079966.2;NP_080613.1;XP_006511412.1;XP_006511413.1;XP_006511414.1;XP_006511415.1;XP_006511417.1;XP_006511418.1;XP_011241088.1;XP_030100356.1;XP_030100357.1;XP_030100358.1;XP_030100359.1;XP_030100360.1;XP_030100361.1	1913910	.	18.455255	[illegible]
66661	NM_025691	Srp72	signal recognition particle 72	protein_coding	NP_079967.1	1333795	.	37.733971	[illegible]
66662	NR_033729	Fbxl12os	F-box and leucine-rich repeat protein 12, opposite strand	transcribed_pseudogene	.	1913912	.	0.285414	[illegible]
66663	NM_025692,XM_006511791,XR_001778969	Uba5	ubiquitin-like modifier activating enzyme 5	protein_coding	NP_079968.2;XP_006511854.1	1913913	.	24.786789	[illegible]
66664	NM_025693,NR_037773	Tmem41a	transmembrane protein 41a	protein_coding	NP_079969.1	1913914	.	6.793293	[illegible]
66665	NM_001145924,NM_001145925,NM_028137,XM_006538187,XM_006538188,XM_011250086,XM_011250087	Msantd3	Myb/SANT-like DNA-binding domain containing 3	protein_coding	NP_001139396.1;NP_001139397.1;NP_082413.2;XP_006538250.1;XP_006538251.1;XP_011248388.1;XP_011248389.1	1913915	.	9.881258	[illegible]
66667	NM_175111,NR_152314,XM_006522469,XM_006522470,XM_006522471,XM_006522472,XM_017317116,XM_030249241,XM_030249242,XM_030249243,XM_030249244	Hspbap1	Hspb associated protein 1	protein_coding	NP_780320.2;XP_006522532.1;XP_006522533.1;XP_006522534.1;XP_006522535.1;XP_017172605.1;XP_030105101.1;XP_030105102.1;XP_030105103.1;XP_030105104.1	1913917	.	1.458989	[illegible]
66671	NM_001347587,NM_001347588,NM_023243,XM_006517325	Ccnh	cyclin H	protein_coding	NP_001334516.1;NP_001334517.1;NP_075732.1;XP_006517388.1	1913921	.	9.005991	[illegible]
66673	NM_025696	Sorcs3	sortilin-related VPS10 domain containing receptor 3	protein_coding	NP_079972.1	1913923	.	0.051559	[illegible]
66674	NM_001310603,NM_025697,XM_011245148,XM_030247956	Spryd7	SPRY domain containing 7	protein_coding	NP_001297532.1;NP_079973.1;XP_011243450.1;XP_030103816.1	1913924	.	3.051595	[illegible]
66676	NM_025698	Tmed7	transmembrane p24 trafficking protein 7	protein_coding	NP_079974.1	1913926	.	34.094219	[illegible]
66679	NM_175112,XM_006500023,XM_006500024	Rae1	ribonucleic acid export 1	protein_coding	NP_780321.1;XP_006500086.1;XP_006500087.1	1913929	.	18.785276	[illegible]
66680	NM_025699,XM_006500025,XM_006500026	Oser1	oxidative stress responsive serine rich 1	protein_coding	NP_079975.2;XP_006500088.1;XP_006500089.1	1913930	.	15.188396	[illegible]
66681	NM_001356964,NM_025700,XM_006504056,XM_006504058	Pgm1	phosphoglucomutase 1	protein_coding	NP_001343893.1;NP_079976.1;XP_006504119.2;XP_006504121.1	97564	.	9.915981	[illegible]
66682	NM_025701	Trappc5	trafficking protein particle complex 5	protein_coding	NP_079977.3	1913932	.	9.976286	[illegible]
66684	NM_001168578,NM_025703	Tceal8	transcription elongation factor A (SII)-like 8	protein_coding	NP_001162050.1;NP_079979.2	1913934	.	39.167184	[illegible]
66686	NM_001347444,NM_025705,XM_011243196,XM_030245220,XM_030245221	Dcbld1	discoidin, CUB and LCCL domain containing 1	protein_coding	NP_001334373.1;NP_079981.2;XP_011241498.1;XP_030101080.1;XP_030101081.1	1913936	.	2.742859	[illegible]
66687	NM_025706,XM_006513957	Tbc1d15	TBC1 domain family, member 15	protein_coding	NP_079982.3;XP_006514020.1	1913937	.	18.713116	[illegible]
66689	NM_025707,XM_006516156,XM_006516157	Klhl28	kelch-like 28	protein_coding	NP_079983.1;XP_006516219.1;XP_006516220.1	1913939	.	3.704893	[illegible]
66690	NM_001362486,NM_025708	Tmem186	transmembrane protein 186	protein_coding	NP_001349415.1;NP_079984.2	1913940	.	5.588023	[illegible]
66691	NM_001356441,NM_025709,NR_151469,XM_011239150,XM_017319195,XM_017319196,XM_017319197,XM_030251954,XM_030251955,XM_030251956,XM_030251957,XM_030251958,XR_003953692,XR_003953693,XR_003953694	Gapvd1	GTPase activating protein and VPS9 domains 1	protein_coding	NP_001343370.1;NP_079985.2;XP_011237452.1;XP_017174684.1;XP_017174685.1;XP_017174686.1;XP_030107814.1;XP_030107815.1;XP_030107816.1;XP_030107817.1;XP_030107818.1	1913941	.	14.933980	[illegible]
66694	NM_025710	Uqcrfs1	ubiquinol-cytochrome c reductase, Rieske iron-sulfur polypeptide 1	protein_coding	NP_079986.1	1913944	.	40.029701	[illegible]
66695	NM_001172481,NM_025711	Aspn	asporin	protein_coding	NP_001165952.1;NP_079987.2	1913945	.	418.192238	[illegible]
66696	NM_001347022,NM_025712,XM_006520147,XM_017316726,XM_017316727	Snx31	sorting nexin 31	protein_coding	NP_001333951.1;NP_079988.3;XP_006520210.1;XP_017172215.1;XP_017172216.1	1913946	.	0.034412	[illegible]
66700	NM_001355674,NM_001355675,NM_001361405,NM_025783,XM_006506481,XM_030255546	Chmp3	charged multivesicular body protein 3	protein_coding	NP_001342603.1;NP_001342604.1;NP_001348334.1;NP_080059.2;XP_006506544.1;XP_030111406.1	1913950	.	30.364921	[illegible]
66701	NM_025716	Spryd4	SPRY domain containing 4	protein_coding	NP_079992.1	1913951	.	8.695978	[illegible]
66704	NM_025717,XM_006531823,XM_006531824,XM_006531825,XM_006531826,XM_006531827,XM_030251030,XR_001782610,XR_001782611,XR_003952803,XR_003952805,XR_003952806,XR_003952807,XR_003952808,XR_003952809	Rbm4b	RNA binding motif protein 4B	protein_coding	NP_079993.2;XP_006531886.1;XP_006531887.1;XP_006531888.1;XP_006531889.1;XP_006531890.1;XP_030106890.1	1913954	.	4.220828	[illegible]
66705	NM_025718,XM_006524798,XM_006524799	Dnase1l2	deoxyribonuclease 1-like 2	protein_coding	NP_079994.2;XP_006524861.1;XP_006524862.1	1913955	.	1.560562	[illegible]
66706	NM_023247	Ndufaf3	NADH:ubiquinone oxidoreductase complex assembly factor 3	protein_coding	NP_075736.1	1913956	.	3.382375	[illegible]
66707	NM_025719	Nkapl	NFKB activating protein-like	protein_coding	NP_079995.2	1913957	.	0.276777	[illegible]
66711	NM_023248,XM_006504490	Sbds	SBDS ribosome maturation factor	protein_coding	NP_075737.1;XP_006504553.1	1913961	.	18.938550	[illegible]
66713	NM_001362899,NM_146243	Actr2	ARP2 actin-related protein 2	protein_coding	NP_001349828.1;NP_666355.1	1913963	.	42.236339	[illegible]
66714	NM_025722,NR_033144	4921524J17Rik	RIKEN cDNA 4921524J17 gene	protein_coding	NP_079998.1	1913964	.	8.369535	[illegible]
66715	NM_001078646,NM_025723,XM_006501898,XR_003954369	Henmt1	HEN1 methyltransferase homolog 1 (Arabidopsis)	protein_coding	NP_001072114.1;NP_079999.2;XP_006501961.1	1913965	.	0.027534	[illegible]
66717	NM_025725	Ccdc96	coiled-coil domain containing 96	protein_coding	NP_080001.1	1913967	.	0.049016	[illegible]
66720	NM_025727,XM_011249181,XM_011249182,XM_017314711	Klhl10	kelch-like 10	protein_coding	NP_080003.1;XP_011247483.1;XP_011247484.1;XP_017170200.1	2181067	.	0.519980	[illegible]
66722	NM_001271533,NM_025728,NM_029160,XM_006496194	Spag16	sperm associated antigen 16	protein_coding	NP_001258462.1;NP_080004.1;NP_083436.2;XP_006496257.1	1913972	.	0.134901	[illegible]
66724	NM_001358447,NM_025729,XM_006528238,XM_006528239,XM_030251476,XM_030251477,XM_030251478,XM_030251479,XR_003953021	Tab3	TGF-beta activated kinase 1/MAP3K7 binding protein 3	protein_coding	NP_001345376.1;NP_080005.2;XP_006528301.1;XP_006528302.1;XP_030107336.1;XP_030107337.1;XP_030107338.1;XP_030107339.1	1913974	.	2.382295	[illegible]
66725	NM_025730,XM_006521277,XR_875310	Lrrk2	leucine-rich repeat kinase 2	protein_coding	NP_080006.3;XP_006521340.1	1913975	.	2.641574	[illegible]
66727	NM_001368696,NM_025731	Plaat5	phospholipase A and acyltransferase 5	protein_coding	NP_001355625.1;NP_080007.2	1913977	.	0.050615	[illegible]
66729	NM_001310772,NM_025732,XM_006504859	Ankrd61	ankyrin repeat domain 61	protein_coding	NP_001297701.1;NP_080008.1;XP_006504922.1	1913979	.	0.097448	[illegible]
66730	NR_033782	4921531C22Rik	RIKEN cDNA 4921531C22 gene	lncRNA		1913980	.	1.192538	[illegible]
66733	NM_001357210,NM_025734,XM_006531289	Kcng4	potassium voltage-gated channel, subfamily G, member 4	protein_coding	NP_001344139.1;NP_080010.2;XP_006531352.1	1913983	.	0.075639	[illegible]
66734	NM_025735,NR_046364	Map1lc3a	microtubule-associated protein 1 light chain 3 alpha	protein_coding	NP_080011.1	1915661	.	26.086130	[illegible]
66736	NM_025736	Emc2	ER membrane protein complex subunit 2	protein_coding	NP_080012.1	1913986	.	26.530497	[illegible]
66743	NM_001310729,NM_001310730,NM_001310731,NM_025739,XM_006503285,XM_011240587,XM_011240588,XM_011240589,XM_017320356,XM_017320357,XM_030253703,XM_030253704,XR_003954995	Rnf220	ring finger protein 220	protein_coding	NP_001297658.1;NP_001297659.1;NP_001297660.1;NP_080015.3;XP_006503348.1;XP_011238889.1;XP_011238890.1;XP_011238891.1;XP_017175845.1;XP_017175846.1;XP_030109563.1;XP_030109564.1	1913993	.	25.385847	[illegible]
66748	NM_025744,XM_006500027,XM_006500028,XM_006500029,XM_006500030,XM_011239739,XM_011239740,XM_030251959,XM_030251960,XM_030251961	Erich2	glutamate rich 2	protein_coding	NP_080020.2;XP_006500090.1;XP_006500091.1;XP_006500092.1;XP_006500093.1;XP_011238041.1;XP_011238042.1;XP_030107819.1;XP_030107820.1;XP_030107821.1	1913998	.	0.168058	[illegible]
66752	NR_015555	4933404O12Rik	RIKEN cDNA 4933404O12 gene	lncRNA	.	1914002	.	1.436089	[illegible]
66753	NM_025745,XM_006514781,XM_006514782,XM_011243743,XM_011243744,XM_030246233,XM_030246234,XM_030246235,XM_030246236,XR_003949491	Erlec1	endoplasmic reticulum lectin 1	protein_coding	NP_080021.3;XP_006514844.1;XP_006514845.1;XP_011242045.1;XP_011242046.1;XP_030102093.1;XP_030102094.1;XP_030102095.1;XP_030102096.1	1914003	.	31.261929	[illegible]
66756	NM_001357215,NM_001357216,NM_025747,XM_006509477,XM_006509478,XM_006509479,XM_030243725,XM_030243726	Cfap97	cilia and flagella associated protein 97	protein_coding	NP_001344144.1;NP_001344145.1;NP_080023.1;XP_006509540.1;XP_006509541.1;XP_006509542.1;XP_030099585.1;XP_030099586.1	1914006	.	4.714229	[illegible]
66757	NM_025748,XM_011243200	Adat2	adenosine deaminase, tRNA-specific 2	protein_coding	NP_080024.3;XP_011241502.1	1914007	.	2.308331	[illegible]
66758	NM_001360984,NM_025749,XM_006526159,XM_006526160,XM_011246983,XM_011246984,XM_030250571,XM_030250572,XM_030250573	Zfp474	zinc finger protein 474	protein_coding	NP_001347913.1;NP_080025.3;XP_006526222.1;XP_006526223.1;XP_011245285.1;XP_011245286.1;XP_030106431.1;XP_030106432.1;XP_030106433.1	1914008	.	0.034470	[illegible]
66765	NM_025752	4933411K16Rik	RIKEN cDNA 4933411K16 gene	protein_coding	NP_080028.1	1914015	.	0.239398	[illegible]
66768	NM_025755	Pacrgl	PARK2 co-regulated-like	protein_coding	NP_080031.1	1914018	.	4.123569	[illegible]
66769	NR_033197	4933427E11Rik	RIKEN cDNA 4933427E11 gene	lncRNA	.	1914019	.	0.000000	[illegible]
66771	NM_025757	Gid4	GID complex subunit 4, VID24 homolog	protein_coding	NP_080033.3	1914021	.	10.497383	[illegible]
66774	NR_131254	Carlr	cardiac and apoptosis-related long non-coding RNA	lncRNA	.	1914024	.	0.000000	[illegible]
66775	NM_025760,XM_006538189,XM_006538190,XM_006538191,XM_006538192,XM_006538193	Hacd4	3-hydroxyacyl-CoA dehydratase 4	protein_coding	NP_080036.1;XP_006538252.1;XP_006538253.1;XP_006538254.1;XP_006538255.1;XP_006538256.1	1914025	.	5.105089	[illegible]
66779	NR_015575,NR_027843	4933432I09Rik	RIKEN cDNA 4933432I09 gene	lncRNA	.	1914029	.	0.169884	[illegible]
66783	XR_003955425,XR_003955426,XR_003955427,XR_003955428,XR_003955429	Aknaos	AT-hook transcription factor, opposite strand, transcript variant X2	lncRNA	.	1914033	.	0.000000	[illegible]
66784	NM_025766	Fam187a	family with sequence similarity 187, member A	protein_coding	NP_080042.1	1914034	.	0.000000	[illegible]
66787	NM_178613	Gskip	GSK3B interacting protein	protein_coding	NP_848728.2	1914037	.	4.878488	[illegible]
66789	NM_024178,NR_155539,XM_006501899,XR_003954370	Alg14	asparagine-linked glycosylation 14	protein_coding	NP_077140.1;XP_006501962.1	1914039	.	9.040484	[illegible]
66790	NM_001368858,NM_025768,XM_006508850,XM_006508851,XM_006508853,XM_017312937,XR_003947326,XR_378665	Grtp1	GH regulated TBC protein 1	protein_coding	NP_001355787.1;NP_080044.2;XP_006508913.1;XP_006508914.1;XP_006508916.1;XP_017168426.1	1914040	.	1.265503	[illegible]
66793	NM_025769,XM_006522474,XM_006522475,XR_001781852,XR_003951819,XR_003951820,XR_875839	Efcab1	EF-hand calcium binding domain 1	protein_coding	NP_080045.1;XP_006522537.1;XP_006522538.2	1914043	.	5.426780	[illegible]
66795	NM_025770,XM_006517326,XM_006517327,XM_011244551,XM_011244552,XM_030247365,XM_030247366,XR_873450	Atg10	autophagy related 10	protein_coding	NP_080046.3;XP_006517389.1;XP_006517390.1;XP_011242853.1;XP_011242854.1;XP_030103225.1;XP_030103226.1	1914045	.	2.818178	[illegible]
66797	NM_001004357,NM_025771,XM_006506483,XM_030255547	Cntnap2	contactin associated protein-like 2	protein_coding	NP_001004357.2;NP_080047.1;XP_006506546.1;XP_030111407.1	1914047	.	0.120320	[illegible]
66799	NM_001271016,NM_001271017,NM_025773,NR_073122,XM_006495557,XM_011238402,XM_017322047,XM_017322048,XR_003946405,XR_003946406	Ube2w	ubiquitin-conjugating enzyme E2W (putative)	protein_coding	NP_001257945.2;NP_001257946.2;NP_080049.3;XP_006495620.1;XP_011236704.1;XP_017177536.1;XP_017177537.1	1914049	.	9.578106	[illegible]
66801	NM_025774	Prkrip1	Prkr interacting protein 1 (IL11 inducible)	protein_coding	NP_080050.1	1914051	.	5.459437	[illegible]
66808	NM_001256489,XM_030246874	9030624G23Rik	RIKEN cDNA 9030624G23 gene	protein_coding	NP_001243418.1;XP_030102734.1	1914058	.	0.836481	[illegible]
66809	NM_023256	Krt20	keratin 20	protein_coding	NP_075745.1	1914059	.	0.000000	[illegible]
66810	NM_025776	Rbm22	RNA binding motif protein 22	protein_coding	NP_080052.1	1914060	.	17.530205	[illegible]
66811	NM_025777	Duoxa2	dual oxidase maturation factor 2	protein_coding	NP_080053.1	1914061	.	0.000000	[illegible]
66812	NM_001357912,NM_001357913,NM_001357914,NM_001357915,NM_001357916,NM_001357918,NM_176831,NR_152144,NR_152145,NR_152146,XM_006511360,XM_006511361,XM_006511363,XM_006511367,XM_011242790,XM_011242791,XM_011242792,XM_011242793,XM_030244502,XM_030244503,XM_030244504,XM_030244505,XR_003947973	Ppcdc	phosphopantothenoylcysteine decarboxylase	protein_coding	NP_001344841.1;NP_001344842.1;NP_001344843.1;NP_001344844.1;NP_001344845.1;NP_001344847.1;NP_789801.1;XP_006511423.1;XP_006511424.1;XP_006511426.1;XP_006511430.1;XP_011241092.1;XP_011241093.1;XP_011241094.1;XP_011241095.1;XP_030100362.1;XP_030100363.1;XP_030100364.1;XP_030100365.1	1914062	.	8.740872	[illegible]
66813	NM_001355686,NM_025778	Bcl2l14	BCL2-like 14 (apoptosis facilitator)	protein_coding	NP_001342615.1;NP_080054.1	1914063	.	0.054515	[illegible]
66815	NM_001293644,NM_025779,XM_006501900,XM_006501901,XM_011240197	Mcub	mitochondrial calcium uniporter dominant negative beta subunit	protein_coding	NP_001280573.1;NP_080055.2;XP_006501963.1;XP_006501964.1;XP_011238499.1	1914065	.	5.853124	[illegible]
66816	NM_025780	Thap2	THAP domain containing, apoptosis associated protein 2	protein_coding	NP_080056.1	1914066	.	4.346070	[illegible]
66817	NM_025781,XM_006531291	Tmem170	transmembrane protein 170	protein_coding	NP_080057.1;XP_006531354.2	106426	.	2.312144	[illegible]
66818	NM_172396	Smim7	small integral membrane protein 7	protein_coding	NP_765984.1	1914068	.	27.164827	[illegible]
66821	NM_001305652,NM_025784,XR_003946408,XR_003946409	Bcs1l	BCS1-like (yeast)	protein_coding	NP_001292581.1;NP_080060.1	1914071	.	4.198915	[illegible]
66822	NM_001347316,NM_001359061,NM_025785,XM_030243729	Fbxo25	F-box protein 25	protein_coding	NP_001334245.1;NP_001345990.1;NP_080061.1;XP_030099589.1	1914072	.	5.981959	[illegible]
66824	NM_023258	Pycard	PYD and CARD domain containing	protein_coding	NP_075747.3	1931465	.	1.834380	[illegible]
66826	NM_001173547,NM_001242615,NM_001242616,NM_001290738,NM_181516,XM_006528244,XM_006528246,XM_006528247,XM_006528248,XM_006528249,XM_006528250,XM_011247662,XM_011247664,XM_030251480,XM_030251481,XM_030251482,XM_030251483,XR_003953022,XR_003953023,XR_386926	Taz	tafazzin	protein_coding	NP_001167018.1;NP_001229544.1;NP_001229545.1;NP_001277667.1;NP_852657.1;XP_006528307.1;XP_006528309.1;XP_006528310.1;XP_006528311.1;XP_006528312.1;XP_006528313.1;XP_011245964.1;XP_011245966.1;XP_030107340.1;XP_030107341.1;XP_030107342.1;XP_030107343.1	109626	.	20.561720	[illegible]
66827	NM_133795	Ttc1	tetratricopeptide repeat domain 1	protein_coding	NP_598556.1	1914077	.	11.703173	[illegible]
66830	NM_025788,XM_006531292,XM_006531293,XM_006531294,XM_006531295	Nacc1	nucleus accumbens associated 1, BEN and BTB (POZ) domain containing	protein_coding	NP_080064.3;XP_006531355.1;XP_006531356.1;XP_006531357.1;XP_006531358.1	1914080	.	12.950055	[illegible]
66832	NM_025789	Rsph3a	radial spoke 3A homolog (Chlamydomonas)	protein_coding	NP_080065.4	1914082	.	9.697997	[illegible]
66834	NM_025790	Acot13	acyl-CoA thioesterase 13	protein_coding	NP_080066.1	1914084	.	15.615579	[illegible]
66835	NR_028575	Snord123	small nucleolar RNA, C/D box 123	snoRNA	.	5297223	.	11.425980	[illegible]
66836	NM_025791	Tmem223	transmembrane protein 223	protein_coding	NP_080067.1	1914086	.	25.061460	[illegible]
66838	NR_038126	0610009L18Rik	RIKEN cDNA 0610009L18 gene	lncRNA	.	1914088	.	2.064661	[illegible]
66839	NM_024179	Dele1	DAP3 binding cell death enhancer 1	protein_coding	NP_077141.2	1914089	.	15.018576	[illegible]
66840	NM_025793,XM_006533964,XR_001780040	Wdr45b	WD repeat domain 45B	protein_coding	NP_080069.2;XP_006534027.1	1914090	.	29.250112	[illegible]
66841	NM_025794	Etfdh	electron transferring flavoprotein, dehydrogenase	protein_coding	NP_080070.2	106100	.	18.803404	[illegible]
66844	NM_001359169,NM_001359170,NM_001359171,NM_024180,NR_152853	Ormdl2	ORM1-like 2 (S. cerevisiae)	protein_coding	NP_001346098.1;NP_001346099.1;NP_001346100.1;NP_077142.1	1914094	.	1.633297	[illegible]
66845	NM_025796	Mrpl33	mitochondrial ribosomal protein L33	protein_coding	NP_080072.2	2137225	.	56.624035	[illegible]
66847	NM_025798,XM_006512823	Hint3	histidine triad nucleotide binding protein 3	protein_coding	NP_080074.1;XP_006512886.1	1914097	.	3.655494	[illegible]
66848	NM_001330198,NM_025799,XM_006512827	Fuca2	fucosidase, alpha-L- 2, plasma	protein_coding	NP_001317127.1;NP_080075.2;XP_006512890.1	1914098	.	8.716217	[illegible]
66849	NM_025800	Ppp1r2	protein phosphatase 1, regulatory inhibitor subunit 2	protein_coding	NP_080076.1	1914099	.	16.709238	[illegible]
66853	NM_001163689,NM_025802,NR_028142	Pnpla2	patatin-like phospholipase domain containing 2	protein_coding	NP_001157161.1;NP_080078.2	1914103	.	8.474683	[illegible]
66854	NM_029979	Trim35	tripartite motif-containing 35	protein_coding	NP_084255.2	1914104	.	36.080330	[illegible]
66855	NM_001037877,NM_001037878,NM_001286362,NM_001286363,NM_001359075,NM_001359076,NM_025804,XM_006531296,XM_006531297,XM_006531298,XM_011248487,XM_030243730	Tcf25	transcription factor 25 (basic helix-loop-helix)	protein_coding	NP_001032966.1;NP_001032967.1;NP_001273291.1;NP_001273292.1;NP_001346004.1;NP_001346005.1;NP_080080.2;XP_006531359.1;XP_006531360.1;XP_006531361.1;XP_011246789.1;XP_030099590.	1914105	.	69.580399	[illegible]
66857	NM_025806,XM_006506485	Plbd1	phospholipase B domain containing 1	protein_coding	NP_080082.1;XP_006506548.1	1914107	.	2.932439	[illegible]
66859	NM_025807,XM_006513960	Slc16a9	solute carrier family 16 (monocarboxylic acid transporters), member 9	protein_coding	NP_080083.2;XP_006514023.1	1914109	.	10.653429	[illegible]
66860	NM_001290659,NM_198294,XM_006500034,XM_006500036,XM_006500037,XM_006500039,XM_006500040,XM_017319200,XM_017319202,XM_017319203,XM_017319204,XM_017319205,XM_030251966,XM_030251967	Tanc1	tetratricopeptide repeat, ankyrin repeat and coiled-coil containing 1	protein_coding	NP_001277588.1;NP_938036.2;XP_006500097.1;XP_006500099.1;XP_006500100.1;XP_006500102.1;XP_006500103.1;XP_017174689.1;XP_017174691.1;XP_017174692.1;XP_017174693.1;XP_017174694.1;XP_030107826.1;XP_030107827.1	1914110	.	10.277910	[illegible]
66861	NM_024181,XR_001783164	Dnajc10	DnaJ heat shock protein family (Hsp40) member C10	protein_coding	NP_077143.2	1914111	.	34.275280	[illegible]
66863	NM_001331226,NM_001331227,NM_025808,XM_006522477,XM_017317119,XM_017317120,XM_017317121,XM_030249246	Lztr1	leucine-zipper-like transcriptional regulator, 1	protein_coding	NP_001318155.1;NP_001318156.1;NP_080084.2;XP_006522540.1;XP_017172608.1;XP_017172609.1;XP_017172610.1;XP_030105106.1	1914113	.	25.720318	[illegible]
66864	NM_025809	Clec14a	C-type lectin domain family 14, member a	protein_coding	NP_080085.3	1914114	.	10.687031	[illegible]
66865	NM_173180,XM_006498237,XM_030251968	Pmpca	peptidase (mitochondrial processing) alpha	protein_coding	NP_775272.1;XP_006498300.1;XP_030107828.1	1918568	.	22.277631	[illegible]
66866	NM_025811,XM_006527267,XR_001782612	Nhlrc2	NHL repeat containing 2	protein_coding	NP_080087.1;XP_006527330.1	1914116	.	5.810172	[illegible]
66867	NM_025812	Hmg20a	high mobility group 20A	protein_coding	NP_080088.1	1914117	.	18.543032	[illegible]
66868	NM_025813,XM_030252759	Mfsd1	major facilitator superfamily domain containing 1	protein_coding	NP_080089.1;XP_030108619.1	1914118	.	20.042991	[illegible]
66869	NM_001039965,NM_001039967,NM_001359086,NM_001359087,NM_181274,XM_011242318,XM_011242319,XM_011242320,XM_030243731	Zfp869	zinc finger protein 869	protein_coding	NP_001035054.1;NP_001035056.1;NP_001346015.1;NP_001346016.1;NP_851791.1;XP_011240620.1;XP_011240621.1;XP_011240622.1;XP_030099591.1	1914119	.	10.025608	[illegible]
66870	NM_001113564,NM_001113565,NM_001113566,NM_025814	Serbp1	serpine1 mRNA binding protein 1	protein_coding	NP_001107036.1;NP_001107037.1;NP_001107038.1;NP_080090.2	1914120	.	37.000708	[illegible]
66871	NM_001033851,NM_001357979,NM_025815,XM_006521285,XM_030248705	Cpne8	copine VIII	protein_coding	NP_001029023.1;NP_001344908.1;NP_080091.1;XP_006521348.1;XP_030104565.1	1914121	.	11.352011	[illegible]
66873	NM_025817	Tril	TLR4 interactor with leucine-rich repeats	protein_coding	NP_080093.1	1914123	.	2.280951	[illegible]
66874	NM_025818	Ncbp3	nuclear cap binding subunit 3	protein_coding	NP_080094.3	1914124	.	6.193688	[illegible]
66875	NM_025819,XM_011248058,XM_011248059,XM_030242199	Swt1	SWT1 RNA endoribonuclease homolog (S. cerevisiae)	protein_coding	NP_080095.3;XP_011246360.1;XP_011246361.1;XP_030098059.1	1914125	.	4.603581	[illegible]
66877	NM_025820	Crnkl1	crooked neck pre-mRNA splicing factor 1	protein_coding	NP_080096.1	1914127	.	8.779119	[illegible]
66878	NM_024182,XR_001782389,XR_001782390,XR_001782391,XR_003952538	Riok3	RIO kinase 3	protein_coding	NP_077144.2	1914128	.	21.465086	[illegible]
66880	NM_001356273,NM_001356274,NM_001356275,NM_001356277,NM_001356278,NM_001356279,NM_001356280,NM_025822,XM_006501911,XM_006501913,XM_011240198,XM_017319692,XM_017319693,XM_017319694	Rsrc1	arginine/serine-rich coiled-coil 1	protein_coding	NP_001343202.1;NP_001343203.1;NP_001343204.1;NP_001343206.1;NP_001343207.1;NP_001343208.1;NP_001343209.1;NP_080098.1;XP_006501974.1;XP_006501976.1;XP_011238500.2;XP_017175181.1;XP_017175182.1;XP_017175183.1	1914130	.	7.621856	[illegible]
66881	NM_001355687,NM_025823	Pcyox1	prenylcysteine oxidase 1	protein_coding	NP_001342616.1;NP_080099.1	1914131	.	22.151570	[illegible]
66882	NM_025824,XM_006496197,XM_017322066	Bzw1	basic leucine zipper and W2 domains 1	protein_coding	NP_080100.1;XP_006496260.1;XP_017177555.1	1914132	.	78.447121	[illegible]
66884	NM_025825	Appbp2	amyloid beta precursor protein (cytoplasmic tail) binding protein 2	protein_coding	NP_080101.1	1914134	.	14.571390	[illegible]
66885	NM_025826,XR_003946541	Acadsb	acyl-Coenzyme A dehydrogenase, short/branched chain	protein_coding	NP_080102.1	1914135	.	13.267986	[illegible]
66887	NM_001168591,NM_025827	Lonp2	lon peptidase 2, peroxisomal	protein_coding	NP_001162063.1;NP_080103.1	1914137	.	11.559986	[illegible]
66889	NM_001254761,NM_023270	Rnf128	ring finger protein 128	protein_coding	NP_001241690.1;NP_075759.3	1914139	.	0.746529	[illegible]
66890	NM_025828	Lman2	lectin, mannose-binding 2	protein_coding	NP_080104.2	1914140	.	26.268364	[illegible]
66892	NM_025829,XM_030255548,XM_030255549,XM_030255550	Eif4e3	eukaryotic translation initiation factor 4E member 3	protein_coding	NP_080105.1;XP_030111408.1;XP_030111409.1;XP_030111410.1	1914142	.	3.121318	[illegible]
66894	NM_025830,XM_006531299,XM_006531301,XR_003947327	Wwp2	WW domain containing E3 ubiquitin protein ligase 2	protein_coding	NP_080106.1;XP_006531362.1;XP_006531364.1	1914144	.	90.842137	[illegible]
66895	NM_025831,XM_006516728,XR_382069	Pxdc1	PX domain containing 1	protein_coding	NP_080107.3;XP_006516791.1	1914145	.	23.707910	[illegible]
66897	NM_025832,XM_006519399,XM_030247958,XR_001781144,XR_001781145,XR_003950899	Naa16	N(alpha)-acetyltransferase 16, NatA auxiliary subunit	protein_coding	NP_080108.1;XP_006519462.1;XP_030103818.1	1914147	.	4.912276	[illegible]
66898	NM_025833	Baiap2l1	BAI1-associated protein 2-like 1	protein_coding	NP_080109.1	1914148	.	1.056002	[illegible]
66899	NM_001159573,NM_001159574,NM_024183,XM_006504264,XM_006504265,XM_006504266,XM_006504267,XM_030254768	Fip1l1	FIP1 like 1 (S. cerevisiae)	protein_coding	NP_001153045.1;NP_001153046.1;NP_077145.2;XP_006504327.1;XP_006504328.1;XP_006504329.1;XP_006504330.1;XP_030110628.1	1914149	.	14.166579	[illegible]
66902	NM_024433	Mtap	methylthioadenosine phosphorylase	protein_coding	NP_077753.1	1914152	.	16.419666	[illegible]
66904	NM_001311149,NM_025835,XM_006511370	Pccb	propionyl Coenzyme A carboxylase, beta polypeptide	protein_coding	NP_001298078.1;NP_080111.1;XP_006511433.1	1914154	.	24.756851	[illegible]
66905	NM_025836	Plin3	perilipin 3	protein_coding	NP_080112.1	1914155	.	19.706724	[illegible]
66910	NM_025838,NM_028336	Tmem107	transmembrane protein 107	protein_coding	NP_080114.1;NP_082612.2	1914160	.	10.349059	[illegible]

66911	NM_001316715,NM_025839	Nudt16l1	nudix (nucleoside diphosphate linked moiety X)-type motif 16-like 1	protein_coding	NP_001303644.1;NP_080115.1	1914161	.	19.488031	[illegible]
66912	NM_025840,XM_006515166	Bzw2	basic leucine zipper and W2 domains 2	protein_coding	NP_080116.2;XP_006515229.1	1914162	.	21.215905	[illegible]
66913	NM_025841	Kdelr2	KDEL (Lys-Asp-Glu-Leu) endoplasmic reticulum protein retention receptor 2	protein_coding	NP_080117.1	1914163	.	153.565628	[illegible]
66914	NM_001305668,NM_025842	Vps28	vacuolar protein sorting 28	protein_coding	NP_001292597.1;NP_080118.1	1914164	.	31.381941	[illegible]
66915	NM_001163425,NR_028108	Cops9	COP9 signalosome subunit 9	protein_coding	NP_001156897.1	1914165	.	36.885566	[illegible]
66916	NM_025843	Ndufb7	NADH:ubiquinone oxidoreductase subunit B7	protein_coding	NP_080119.1	1914166	.	52.692551	[illegible]
66917	NM_025844	Chordc1	cysteine and histidine-rich domain (CHORD)-containing, zinc-binding protein 1	protein_coding	NP_080120.1	1914167	.	17.609364	[illegible]
66921	NM_001356292,NM_025845,XM_006501914,XM_006501915,XM_011240199,XM_030252761	Prpf38b	PRP38 pre-mRNA processing factor 38 (yeast) domain containing B	protein_coding	NP_001343221.1;NP_080121.1;XP_006501977.1;XP_006501978.1;XP_011238501.1;XP_030108621.1	1914171	.	27.817896	[illegible]
66922	NM_025846,XM_017312239	Rras2	related RAS viral (r-ras) oncogene 2	protein_coding	NP_080122.2;XP_017167728.1	1914172	.	11.866636	[illegible]
66923	NM_001081251,NM_001359854,NM_001359855,NM_001359856,NM_001359857,NM_001359858	Pbrm1	polybromo 1	protein_coding	NP_001074720.1;NP_001346783.1;NP_001346784.1;NP_001346785.1;NP_001346786.1;NP_001346787.1	1923998	.	13.963190	[illegible]
66925	NM_025848	Sdhd	succinate dehydrogenase complex, subunit D, integral membrane protein	protein_coding	NP_080124.1	1914175	.	58.644897	[illegible]
66926	NM_001356442,NM_001356443,NM_001356444,NM_175113,XM_030251972	Trmt6	tRNA methyltransferase 6	protein_coding	NP_001343371.1;NP_001343372.1;NP_001343373.1;NP_780322.2;XP_030107832.1	1914176	.	6.325455	[illegible]
66928	NM_025849	Dmac1	distal membrane arm assembly complex 1	protein_coding	NP_080125.1	1914178	.	27.979931	[illegible]
66929	NM_024184	Asf1b	anti-silencing function 1B histone chaperone	protein_coding	NP_077146.1	1914179	.	6.597716	[illegible]
66930	NM_025850,XM_006508120	Fank1	fibronectin type 3 and ankyrin repeat domains 1	protein_coding	NP_080126.1;XP_006508183.1	1914180	.	0.036416	[illegible]
66931	NM_001347544,NM_025851,XR_003952172,XR_876377	1700010I14Rik	RIKEN cDNA 1700010I14 gene	protein_coding	NP_001334473.1;NP_080127.2	1914181	.	0.161982	[illegible]
66932	NM_025852,XM_006513961,XM_006513962,XM_011243530,XM_011243531,XR_003948775,XR_871724	Rexo1	REX1, RNA exonuclease 1	protein_coding	NP_080128.2;XP_006514024.1;XP_006514025.1;XP_011241832.1;XP_011241833.1	1914182	.	7.769581	[illegible]
66934	NM_025853	Dsn1	DSN1 homolog, MIS12 kinetochore complex component	protein_coding	NP_080129.2	1914184	.	5.442571	[illegible]
66935	NM_025854	Cir1	corepressor interacting with RBPJ, 1	protein_coding	NP_080130.2	1914185	.	13.026068	[illegible]
66938	NM_001162533,NM_025856,XM_030253707,XM_030253708,XM_030253709,XM_030253710,XM_030253711	Sh3d21	SH3 domain containing 21	protein_coding	NP_001156005.1;NP_080132.1;XP_030109567.1;XP_030109568.1;XP_030109569.1;XP_030109570.1;XP_030109571.1	1914188	.	3.244207	[illegible]
66939	NM_001357323,NM_025857,NR_151671,XR_379442	Aagab	alpha- and gamma-adaptin binding protein	protein_coding	NP_001344252.1;NP_080133.1	1914189	.	5.141155	[illegible]
66940	NM_001284332,NM_001357932,NM_001357933,NM_001357934,NM_001357935,NM_001357936,NM_001357937,NM_001357939,NM_001357940,NM_001357941,NM_025858,NM_026381,XM_006512212,XM_030244506,XM_030244507,XM_030244508	Shisa5	shisa family member 5	protein_coding	NP_001271261.1;NP_001344861.1;NP_001344862.1;NP_001344863.1;NP_001344864.1;NP_001344865.1;NP_001344866.1;NP_001344868.1;NP_001344869.1;NP_001344870.1;NP_080134.1;NP_080657.1;XP_006512275.1;XP_030100366.1;XP_030100367.1;XP_030100368.1	1915044	.	45.121681	[illegible]
66942	NM_025860,XM_006529794	Ddx18	DEAD (Asp-Glu-Ala-Asp) box polypeptide 18	protein_coding	NP_080136.2;XP_006529857.1	1914192	.	16.742627	[illegible]
66943	NM_001164420,NM_001164421,NM_001164422,NM_001164423,NM_001164424,NM_025861,XM_006526519,XM_006526520,XM_006526522,XM_017317970,XM_030250575,XR_001782392,XR_3863	Pqlc1	PQ loop repeat containing 1	protein_coding	NP_001157892.1;NP_001157893.1;NP_001157894.1;NP_001157895.1;NP_001157896.1;NP_080137.2;XP_006526582.1;XP_006526583.1;XP_006526585.1;XP_017173459.1;XP_030106435.1	1914193	.	10.040692	[illegible]
66945	NM_023281	Sdha	succinate dehydrogenase complex, subunit A, flavoprotein (Fp)	protein_coding	NP_075770.1	1914195	.	51.593983	[illegible]
66948	NM_025862,XM_006510550,XM_030244509,XM_030244510,XR_001778975,XR_001778977,XR_003947975,XR_870536	Acad8	acyl-Coenzyme A dehydrogenase family, member 8	protein_coding	NP_080138.2;XP_006510613.1;XP_030100369.1;XP_030100370.1	1914198	.	9.962279	[illegible]
66949	NM_025863,XM_006501917,XM_011240200,XM_011240201	Trim59	tripartite motif-containing 59	protein_coding	NP_080139.3;XP_006501980.1;XP_011238502.1;XP_011238503.1	1914199	.	8.606245	[illegible]
66950	NM_025864	Pacc1	proton activated chloride channel 1	protein_coding	NP_080140.1	1914200	.	3.974907	[illegible]
66952	NM_025865	2310030G06Rik	RIKEN cDNA 2310030G06 gene	protein_coding	NP_080141.2	1914202	.	2.082885	[illegible]
66953	NM_025866,XM_006500053,XM_030251973	Cdca7	cell division cycle associated 7	protein_coding	NP_080142.1;XP_006500116.1;XP_030107833.1	1914203	.	6.606259	[illegible]
66957	NM_025867	Serpinb11	serine (or cysteine) peptidase inhibitor, clade B (ovalbumin), member 11	protein_coding	NP_080143.1	1914207	.	0.027886	[illegible]
66958	NM_001290751,NM_025868	Tmx2	thioredoxin-related transmembrane protein 2	protein_coding	NP_001277680.1;NP_080144.1	1914208	.	13.392951	[illegible]
66959	NM_001357223,NM_025869	Dusp26	dual specificity phosphatase 26 (putative)	protein_coding	NP_001344152.1;NP_080145.1	1914209	.	0.709272	[illegible]
66960	NM_001355535,NM_001355536,NM_001355537,NM_001355538,NM_001355539,NM_024185,NR_149730,NR_149731,NR_149732,NR_149745,XM_006497522,XM_006497523,XM_006497524,XM_006497525,XM_006497526,XM_006497529,XM_030251974,XR_001783166,XR_003953695	Mindy3	MINDY lysine 48 deubiquitinase 3	protein_coding	NP_001342464.1;NP_001342465.1;NP_001342466.1;NP_001342467.1;NP_001342468.1;NP_077147.2;XP_006497585.1;XP_006497586.1;XP_006497587.1;XP_006497588.1;XP_006497589.1;XP_006497592.1;XP_030107834.1	1914210	.	7.954924	[illegible]
66961	NR_003513,NR_131212	Neat1	nuclear paraspeckle assembly transcript 1 (non-protein coding)	lncRNA	.	1914211	.	121.803113	[illegible]
66962	NM_025870	Swsap1	SWIM type zinc finger 7 associated protein 1	protein_coding	NP_080146.1	1914212	.	3.733525	[illegible]
66964	NM_025872,XM_006506981,XR_003956250	Golt1b	golgi transport 1B	protein_coding	NP_080148.1;XP_006507044.1	1914214	.	28.712016	[illegible]
66965	NM_153775,NR_152823,XM_011248489,XR_003947328,XR_003947329	Ctu2	cytosolic thiouridylase subunit 2	protein_coding	NP_722470.2;XP_011246791.1	1914215	.	4.570538	[illegible]
66966	NM_025873,NR_151450,XM_017320359,XR_003954996,XR_37634	Trit1	tRNA isopentenyltransferase 1	protein_coding	NP_080149.2;XP_017175848.1	1914216	.	6.121980	[illegible]
66967	NM_001039644,NM_001311165,NM_001311166,XM_006529795,XM_006529796,XM_006529797,XM_006529798,XM_011248060,XM_011248061,XM_011248062,XM_011248063,XM_017322083,XM_017322085,XM_030242231	Edem3	ER degradation enhancer, mannosidase alpha-like 3	protein_coding	NP_001034733.2;NP_001298094.1;NP_001298095.1;XP_006529858.1;XP_006529859.1;XP_006529860.1;XP_006529861.1;XP_011246362.1;XP_011246363.1;XP_011246364.1;XP_011246365.1;XP_017177572.1;XP_017177574.1;XP_030098091.1	1914217	.	15.646846	[illegible]
66968	NM_001077348,NM_025874,XM_006524805,XM_017317614	Plin5	perilipin 5	protein_coding	NP_001070816.1;NP_080150.2;XP_006524868.1;XP_017173103.1	1914218	.	1.031660	[illegible]
66970	NM_001360775,NM_001360776,NM_024186,NM_024272,XM_006517328,XM_006517329,XM_006517332,XM_006517334,XM_011244553,XM_011244554,XM_030247367,XM_030247368,XM_030247369,XM_030247370,XM_030247371,XM_030247372,XM_030247373,XM_030247374,XM_030247375,XM_030247376,XM_030247377,XM_030247378,XR_003950463,XR_003950464,XR_003950465,XR_003950466	Ssbp2	single-stranded DNA binding protein 2	protein_coding	NP_001347704.1;NP_001347705.1;NP_077148.1;NP_077234.2;XP_006517391.1;XP_006517392.1;XP_006517395.1;XP_006517397.1;XP_011242855.1;XP_011242856.1;XP_030103227.1;XP_030103228.1;XP_030103229.1;XP_030103230.1;XP_030103231.1;XP_030103232.1;XP_030103233.1;XP_030103234.1;XP_030103235.1;XP_030103236.1;XP_030103237.1;XP_030103238.1	1914220	.	9.808651	[illegible]
66971	NM_025876,XR_001783167,XR_001783168,XR_003953696	Cdk5rap1	CDK5 regulatory subunit associated protein 1	protein_coding	NP_080152.1	1914221	.	2.950475	[illegible]
66972	NM_025877,XM_011246575,XM_011246576,XM_030250000	Slc25a23	solute carrier family 25 (mitochondrial carrier; phosphate carrier),	protein_coding	NP_080153.2;XP_011244877.1;XP_011244878.1;XP_030105860.1	1914222	.	6.597468	[illegible]
66973	NM_001347380,NM_001361453,NM_001361454,NM_001361455,NM_025878	Mrps18b	mitochondrial ribosomal protein S18B	protein_coding	NP_001334309.1;NP_001348382.1;NP_001348383.1;NP_001348384.1;NP_080154.1	1914223	.	12.718306	[illegible]
66975	NM_001093759,NM_001093760,NM_001360778,NM_025879	Trappc13	trafficking protein particle complex 13	protein_coding	NP_001087228.1;NP_001087229.1;NP_001347707.1;NP_080155.3	1914225	.	9.902739	[illegible]
66977	NM_001355159,NM_023284	Nuf2	NUF2, NDC80 kinetochore complex component	protein_coding	NP_001342088.1;NP_075773.2	1914227	.	7.798658	[illegible]
66978	NM_025881,NM_028190,NR_037905,NR_037906,XM_017317615	Luc7l	Luc7-like	protein_coding	NP_080157.1;NP_082466.2;XP_017173104.1	1914228	.	30.042209	[illegible]
66979	NM_001370295,NM_025882,XR_003956251	Pole4	polymerase (DNA-directed), epsilon 4 (p12 subunit)	protein_coding	NP_001357224.1;NP_080158.1	1914229	.	13.131461	[illegible]
66980	NM_001033573,NM_001360595,NM_025883,XM_006527268,XM_011247316,XM_030251032	Zdhhc6	zinc finger, DHHC domain containing 6	protein_coding	NP_001028745.1;NP_001347524.1;NP_080159.3;XP_006527331.1;XP_011245618.1;XP_030106892.1	1914230	.	16.955486	[illegible]
66983	NM_025884	Zfp830	zinc finger protein 830	protein_coding	NP_080160.2	1914233	.	4.104524	[illegible]
66985	NM_025886,XM_006536226,XM_006536227,XM_006536228	Rassf7	Ras association (RalGDS/AF-6) domain family (N-terminal) member 7	protein_coding	NP_080162.3;XP_006536289.1;XP_006536290.1;XP_006536291.1	1914235	.	0.817748	[illegible]
66988	NM_024434	Lap3	leucine aminopeptidase 3	protein_coding	NP_077754.3	1914238	.	18.891375	[illegible]
66989	NM_001356298,NM_001356299,NM_001356300,NM_025888,XM_006524811	Kctd20	potassium channel tetramerisation domain containing 20	protein_coding	NP_001343227.1;NP_001343228.1;NP_001343229.1;NP_080164.2;XP_006524874.1	1914239	.	24.391926	[illegible]
66990	NM_001078649,NM_025889	Tmem134	transmembrane protein 134	protein_coding	NP_001072117.1;NP_080165.1	1914240	.	19.040675	[illegible]
66991	NM_001311106,NM_025890,XM_006511372,XM_006511374	Khdc3	KH domain containing 3, subcortical maternal complex member	protein_coding	NP_001298035.1;NP_080166.1;XP_006511435.1;XP_006511437.1	1914241	.	0.000000	[illegible]
66993	NM_025891,XM_006535796,XM_006535797,XM_006535798,XM_006535799,XM_030254769,XM_030254770,XM_030254771,XR_003955694,XR_389749	Smarcd3	SWI/SNF related, matrix associated, actin dependent regulator of chromatin, subfamily d, member 3	protein_coding	NP_080167.3;XP_006535859.2;XP_006535860.1;XP_006535861.2;XP_006535862.1;XP_030110629.1;XP_030110630.1;XP_030110631.1	1914243	.	10.358550	[illegible]
66994	NM_025892	Cep19	centrosomal protein 19	protein_coding	NP_080168.1	1914244	.	4.812883	[illegible]
66995	NM_001035509,NM_001035510,NM_001358437,NM_001358439,NM_001358440,NM_001358441,NM_025893,XM_030251484,XM_030251485,XM_030251486	Zcchc18	zinc finger, CCHC domain containing 18	protein_coding	NP_001030586.1;NP_001030587.1;NP_001345366.1;NP_001345368.1;NP_001345369.1;NP_001345370.1;NP_080169.2;XP_030107344.1;XP_030107345.1;XP_030107346.1	1914245	.	0.122362	[illegible]
66997	NM_025894	Psmd12	proteasome (prosome, macropain) 26S subunit, non-ATPase, 12	protein_coding	NP_080170.1	1914247	.	32.222313	[illegible]
66998	NM_001355544,NM_080554,XM_017319212,XM_030251975	Psmd5	proteasome (prosome, macropain) 26S subunit, non-ATPase, 5	protein_coding	NP_001342473.1;NP_542121.2;XP_017174701.1;XP_030107835.1	1914248	.	16.675803	[illegible]
66999	NM_025895	Med28	mediator complex subunit 28	protein_coding	NP_080171.1	1914249	.	5.164004	[illegible]
67003	NM_025899	Uqcrc2	ubiquinol cytochrome c reductase core protein 2	protein_coding	NP_080175.1	1914253	.	49.641304	[illegible]
67005	NM_025901	Polr3k	polymerase (RNA) III (DNA directed) polypeptide K	protein_coding	NP_080177.1	1914255	.	8.359275	[illegible]
67006	NM_025902,XM_006501918	Cisd2	CDGSH iron sulfur domain 2	protein_coding	NP_080178.1;XP_006501981.1	1914256	.	15.194759	[illegible]
67008	NM_025904	Yae1d1	Yae1 domain containing 1	protein_coding	NP_080180.1	1914258	.	5.214181	[illegible]
67009	NM_001168475,NM_001168476,NM_001168477,NM_001360653,NM_025905,XM_006541109,XM_017312241,XM_017312242,XM_017312243,XM_017312244,XM_017312245,XM_017312246,XM_017312247,XM_017312248,XM_017312249,XM_017312251,XM_017312252,XM_017312253,XM_030242910,XM_030242911,XM_030242912,XM_030242913,XM_030242914,XM_030242915,XM_030242916,XM_030242917	Ttc23	tetratricopeptide repeat domain 23	protein_coding	NP_001161947.1;NP_001161948.1;NP_001161949.1;NP_001347582.1;NP_080181.2;XP_006541172.1;XP_017167730.1;XP_017167731.1;XP_017167732.1;XP_017167733.1;XP_017167734.1;XP_017167735.1;XP_017167736.1;XP_017167737.1;XP_017167738.1;XP_017167740.1;XP_017167741.1;XP_017167742.1;XP_030098770.1;XP_030098771.1;XP_030098772.1;XP_030098773.1;XP_030098774.1;XP_030098775.1;XP_030098776.1;XP_030098777.1	1914259	.	9.001768	[illegible]
67010	NM_001326355,NM_001326356,NM_144948,NR_037589,NR_1369	Rbm7	RNA binding motif protein 7	protein_coding	NP_001313284.1;NP_001313285.1;NP_659197.2	1914260	.	28.422833	[illegible]
67011	NM_001359846,NM_001359847,NM_001359848,NM_001359849,NM_001359850,NM_025907,XM_011245154,XM_030247961,XM_030247962,XM_030247963,XM_030247964,XM_030247965,XM_030247966,XM_030247967,XM_030247968,XR_874361	Mettl6	methyltransferase like 6	protein_coding	NP_001346775.1;NP_001346776.1;NP_001346777.1;NP_001346778.1;NP_001346779.1;NP_080183.1;XP_011243456.1;XP_030103821.1;XP_030103822.1;XP_030103823.1;XP_030103824.1;XP_030103825.1;XP_030103826.1;XP_030103827.1;XP_030103828.1	1914261	.	11.893284	[illegible]
67013	NM_025909,XM_006503299,XM_006503300,XM_006503301,XM_006503302,XM_006503303,XM_017320360,XM_030253713,XM_030253714,XR_867888	Oma1	OMA1 zinc metallopeptidase	protein_coding	NP_080185.1;XP_006503362.1;XP_006503363.1;XP_006503364.1;XP_006503365.1;XP_006503366.1;XP_017175849.1;XP_030109573.1;XP_030109574.1	1914263	.	3.567509	[illegible]
67014	NM_025910,XM_006522479,XR_003951821,XR_003951822,XR_384594	Riox2	ribosomal oxygenase 2	protein_coding	NP_080186.3;XP_006522542.1	1914264	.	6.012626	[illegible]
67015	NM_001355713,NM_001355714,NM_001355715,NM_025911,XM_006507076,XM_006507078,XM_006507079,XM_006507080,XM_030255551	Ccdc91	coiled-coil domain containing 91	protein_coding	NP_001342642.1;NP_001342643.1;NP_001342644.1;NP_080187.2;XP_006507139.1;XP_006507141.1;XP_006507142.1;XP_006507143.1;XP_030111411.1	1914265	.	7.460135	[illegible]
67016	NM_194334,XM_011242794,XR_001778980	Tbc1d2b	TBC1 domain family, member 2B	protein_coding	NP_919315.2;XP_011241096.1	1914266	.	24.296299	[illegible]
67017	NM_025912,XM_006500059	Fam210b	family with sequence similarity 210, member B	protein_coding	NP_080188.3;XP_006500122.1	1914267	.	1.676869	[illegible]
67019	NM_001359468,NM_001359469,NM_025914,XM_006513965,XM_006513966,XM_006513967,XM_011243532,XM_030245223,XM_030245224	Actr6	ARP6 actin-related protein 6	protein_coding	NP_001346397.1;NP_001346398.1;NP_080190.1;XP_006514028.1;XP_006514029.1;XP_006514030.1;XP_011241834.1;XP_030101083.1;XP_030101084.1	1914269	.	5.118935	[illegible]
67020	NM_025915	Tmem88	transmembrane protein 88	protein_coding	NP_080191.3	1914270	.	4.171604	[illegible]
67023	NM_001145780,NM_025917,XM_006509726	Use1	unconventional SNARE in the ER 1 homolog (S. cerevisiae)	protein_coding	NP_001139252.1;NP_080193.1;XP_006509789.1	1914273	.	71.187158	[illegible]
67025	NM_025919,XM_006539112	Rpl11	ribosomal protein L11	protein_coding	NP_080195.1;XP_006539175.1	1914275	.	369.965606	[illegible]
67026	NM_001310761,NM_025920	Thap4	THAP domain containing 4	protein_coding	NP_001297690.1;NP_080196.3	1914276	.	8.583251	[illegible]
67027	NM_023290,XM_011241431	Mkrn2	makorin, ring finger protein, 2	protein_coding	NP_075779.2;XP_011239733.1	1914277	.	8.345893	[illegible]
67028	NM_025921	2610002M06Rik	RIKEN cDNA 2610002M06 gene	protein_coding	NP_080197.2	1914278	.	3.095692	[illegible]
67030	NM_001277273,NM_025923,NR_102382,XR_872208	Fancl	Fanconi anemia, complementation group L	protein_coding	NP_001264202.1;NP_080199.1	1914280	.	3.646545	[illegible]
67031	NM_025924,XM_006508860	Upf3a	UPF3 regulator of nonsense transcripts homolog A (yeast)	protein_coding	NP_080200.1;XP_006508923.1	1914281	.	18.373840	[illegible]
67035	NM_001356363,NM_001356364,NM_025926,NM_027287,XM_030252765	Dnajb4	DnaJ heat shock protein family (Hsp40) member B4	protein_coding	NP_001343292.1;NP_001343293.1;NP_080202.1;NP_081563.2;XP_030108625.1	1914285	.	7.886611	[illegible]
67036	NM_025927,XM_017314713	Mrpl45	mitochondrial ribosomal protein L45	protein_coding	NP_080203.1;XP_017170202.1	1914286	.	9.602176	[illegible]
67037	NM_001310605,NM_025928,XM_011240202,XM_030252766	Pmf1	polyamine-modulated factor 1	protein_coding	NP_001297534.1;NP_080204.1;XP_011238504.1;XP_030108626.1	1914287	.	15.979246	[illegible]
67038	NM_025929	Ly6m	lymphocyte antigen 6 complex, locus M	protein_coding	NP_080205.1	1914288	.	0.000000	[illegible]
67039	NM_001364415,NM_001364416,NM_027349,XM_006516164,XM_006516165,XM_006516166,XM_006516168,XM_006516169,XM_030246877,XM_030246878,XM_030246879,XM_030246880,XM_030246881	Rbm25	RNA binding motif protein 25	protein_coding	NP_001351344.1;NP_001351345.1;NP_081625.3;XP_006516227.1;XP_006516228.1;XP_006516229.1;XP_006516231.1;XP_006516232.1;XP_030102737.1;XP_030102738.1;XP_030102739.1;XP_030102740.1;XP_030102741.1	1914289	.	34.325183	[illegible]
67040	NM_001040187,NM_152806,NM_199079,NM_199080,XM_030248706,XR_001781563,XR_384033	Ddx17	DEAD (Asp-Glu-Ala-Asp) box polypeptide 17	protein_coding	NP_001035277.1;NP_690019.2;NP_951061.1;NP_951062.1;XP_030104566.1	1914290	.	222.393927	[illegible]
67041	NM_024188	Oxct1	3-oxoacid CoA transferase 1	protein_coding	NP_077150.1	1914291	.	30.542818	[illegible]
67042	NM_025931	Ift27	intraflagellar transport 27	protein_coding	NP_080207.1	1914292	.	11.863024	[illegible]
67043	NM_025932	Syap1	synapse associated protein 1	protein_coding	NP_080208.2	1914293	.	25.850882	[illegible]
67044	NM_025933	Higd2a	HIG1 domain family, member 2A	protein_coding	NP_080209.1	1914294	.	39.602581	[illegible]
67045	NM_025934	Riok2	RIO kinase 2	protein_coding	NP_080210.1	1914295	.	7.990201	[illegible]
67046	NM_001252639,NM_001252640,NM_025935,XM_006516945	Tbc1d7	TBC1 domain family, member 7	protein_coding	NP_001239568.1;NP_001239569.1;NP_080211.1;XP_006517008.1	1914296	.	7.469410	[illegible]
67048	NM_001081356,NM_001290780,NM_001290781,XM_006528261	Vma21	VMA21 vacuolar H+-ATPase homolog (S. cerevisiae)	protein_coding	NP_001074825.1;NP_001277709.1;NP_001277710.1;XP_006528324.	1914298	.	10.308321	[illegible]
67049	NM_023292	Pus3	pseudouridine synthase 3	protein_coding	NP_075781.3	1914299	.	4.594250	[illegible]
67050	NM_025937	Nkap	NFKB activating protein	protein_coding	NP_080213.3	1914300	.	6.224142	[illegible]
67052	NM_023294	Ndc80	NDC80 kinetochore complex component	protein_coding	NP_075783.2	1914302	.	5.546821	[illegible]
67053	NM_025938	Rpp14	ribonuclease P 14 subunit	protein_coding	NP_080214.1	1914303	.	6.804160	[illegible]
67054	NM_001356971,NM_001356972,NM_001356973,NM_001356974,NM_025939,XM_006535183,XM_006535184,XM_006535185,XM_017321075	Paics	phosphoribosylaminoimidazole carboxylase, phosphoribosylaminoribosylaminoimidazole, succinocarboxamide synthetase	protein_coding	NP_001343900.1;NP_001343901.1;NP_001343902.1;NP_001343903.1;NP_080215.1;XP_006535246.1;XP_006535247.1;XP_006535248.1;XP_017176564.1	1914304	.	43.226512	[illegible]
67057	NM_024189,NR_028315,XM_006521292	Yaf2	YY1 associated factor 2	protein_coding	NP_077151.3;XP_006521355.1	1914307	.	6.745595	[illegible]
67059	NM_025942,NM_030091	Ola1	Obg-like ATPase 1	protein_coding	NP_080218.1;NP_084367.1	1914309	.	17.085546	[illegible]
67062	NM_001082412,NM_001114179,XM_006528599,XM_011247758	Slc25a53	solute carrier family 25, member 53	protein_coding	NP_001075881.1;NP_001107651.1;XP_006528662.1;XP_011246060.	1914312	.	0.840075	[illegible]
67063	NM_001355503,NM_025944,NR_149725,XM_006538195	Tmem246	transmembrane protein 246	protein_coding	NP_001342432.1;NP_080220.2;XP_006538258.1	1914313	.	5.188449	[illegible]
67064	NM_024190	Chmp1b	charged multivesicular body protein 1B	protein_coding	NP_077152.1	1914314	.	9.531779	[illegible]
67065	NM_001164082,NM_025945,XM_006519438	Polr3d	polymerase (RNA) III (DNA directed) polypeptide D	protein_coding	NP_001157554.1;NP_080221.3;XP_006519501.1	1914315	.	17.811275	[illegible]
67067	NM_001164216,NM_001164217,NM_025946	Romo1	reactive oxygen species modulator 1	protein_coding	NP_001157688.1;NP_001157689.1;NP_080222.1	1914317	.	33.900757	[illegible]
67068	NM_001291108,NM_025947,NR_111890,NR_111891	Dynlrb1	dynein light chain roadblock-type 1	protein_coding	NP_001278037.1;NP_080223.2	1914318	.	72.728784	[illegible]
67070	NM_001360654,NM_001360655,NM_001360656,NM_025948,XM_011250679,XM_011250681,XM_017312254,XM_030242918	Lsm14a	LSM14A mRNA processing body assembly factor	protein_coding	NP_001347583.1;NP_001347584.1;NP_001347585.1;NP_080224.1;XP_011248981.1;XP_011248983.1;XP_017167743.1;XP_030098778.1	1914320	.	29.434343	[illegible]
67071	NM_025949,XM_006528400,XM_006528401,XM_006528402,XM_006528403,XM_006528404,XM_011247706,XM_011247707,XM_030251487,XM_030251488	Rps6ka6	ribosomal protein S6 kinase polypeptide 6	protein_coding	NP_080225.2;XP_006528463.1;XP_006528464.1;XP_006528465.1;XP_006528466.1;XP_006528467.1;XP_011246008.1;XP_011246009.1;XP_030107347.1;XP_030107348.1	1914321	.	1.226509	[illegible]
67072	NM_001302380,NM_001302382,NM_001302443,NM_025950,XM_006527271,XR_003952810,XR_877687	Cdc37l1	cell division cycle 37-like 1	protein_coding	NP_001289309.1;NP_001289311.1;NP_001289372.1;NP_080226.1;XP_006527334.1	1914322	.	17.273325	[illegible]
67073	NM_025951,NM_028744,XM_006504064	Pi4k2b	phosphatidylinositol 4-kinase type 2 beta	protein_coding	NP_080227.2;NP_083020.2;XP_006504127.1	1914323	.	12.216614	[illegible]
67074	NM_001163024,NM_001163025,NM_153395,XM_006513968,XM_006513969,XM_006513970,XM_006513972,XM_011243533,XM_017314065,XM_030245227,XR_380429	Mon2	MON2 homolog, regulator of endosome to Golgi trafficking	protein_coding	NP_001156496.1;NP_001156497.1;NP_700444.2;XP_006514031.1;XP_006514032.1;XP_006514033.1;XP_006514035.1;XP_011241835.1;XP_017169554.1;XP_030101087.1	1914324	.	14.971416	[illegible]
67075	NM_001190409,NM_025952	Magt1	magnesium transporter 1	protein_coding	NP_001177338.2;NP_080228.5	1914325	.	18.209355	[illegible]
67077	NM_001039494	Catsperz	cation channel sperm associated auxiliary subunit zeta	protein_coding	NP_001034583.1	1914327	.	1.093433	[illegible]
67078	NM_025954	Pgp	phosphoglycolate phosphatase	protein_coding	NP_080230.2	1914328	.	8.777773	[illegible]
67080	NM_001368274,NM_001368276,NM_001368277,NM_001368278,NM_001368279,NM_001368280,NM_001368281,NM_144953	1700019D03Rik	RIKEN cDNA 1700019D03 gene	protein_coding	NP_001355203.1;NP_001355205.1;NP_001355206.1;NP_001355207.1;NP_001355208.1;NP_001355209.1;NP_001355210.1;NP_659202.1	1914330	.	1.196189	[illegible]
67087	NM_001141930,NM_023465	Ctnnbip1	catenin beta interacting protein 1	protein_coding	NP_001135402.1;NP_075954.1	1915756	.	11.981853	[illegible]
67088	NM_025958,XM_030255552	Cand2	cullin-associated and neddylation-dissociated 2 (putative)	protein_coding	NP_080234.2;XP_030111412.1	1914338	.	5.242836	[illegible]
67089	NM_025959,XM_030247969	Psmc6	proteasome (prosome, macropain) 26S subunit, ATPase, 6	protein_coding	NP_080235.2;XP_030103829.1	1914339	.	46.345959	[illegible]
67091	NM_001331181,NM_025960,NR_138582,NR_138583,NR_138584,XM_030242919	Trappc6a	trafficking protein particle complex 6A	protein_coding	NP_001318110.1;NP_080236.2;XP_030098779.1	1914341	.	9.757748	[illegible]
67092	NM_025961	Gatm	glycine amidinotransferase (L-arginine:glycine amidinotransferase)	protein_coding	NP_080237.1	1914342	.	7.036890	[illegible]
67095	NM_001357968,NM_001357969,NM_001357970,NM_001357972,NM_001357973,NM_175114,NR_152160	Trak1	trafficking protein, kinesin binding 1	protein_coding	NP_001344897.1;NP_001344898.1;NP_001344899.1;NP_001344901.1;NP_001344902.1;NP_780323.2	1914345	.	9.649881	[illegible]
67096	NM_025962	Mmachc	methylmalonic aciduria cblC type, with homocystinuria	protein_coding	NP_080238.2	1914346	.	5.102035	[illegible]
67097	NM_001364934,NM_025963	Rps10	ribosomal protein S10	protein_coding	NP_001351863.1;NP_080239.1	1914347	.	635.978076	[illegible]
67099	NM_001368283,NM_001368284,NM_025964,XR_001785519,XR_003946423	Mettl21a	methyltransferase like 21A	protein_coding	NP_001355212.1;NP_001355213.1;NP_080240.1	1914349	.	6.903795	[illegible]
67101	NM_025966	2310039H08Rik	RIKEN cDNA 2310039H08 gene	protein_coding	NP_080242.1	1914351	.	17.793112	[illegible]
67102	NM_001252438,NM_001252439,NM_001252440,NM_025967	D16Ertd472e	DNA segment, Chr 16, ERATO Doi 472, expressed	protein_coding	NP_001239367.1;NP_001239368.1;NP_001239369.1;NP_080243.3	1196400	.	5.740974	[illegible]
67103	NM_025968,XM_011250088	Ptgr1	prostaglandin reductase 1	protein_coding	NP_080244.1;XP_011248390.1	1914353	.	4.242717	[illegible]
67105	NM_025969,NR_160856,XM_011247094	Timm21	translocase of inner mitochondrial membrane 21	protein_coding	NP_080245.1;XP_011245396.1	1920595	.	11.704988	[illegible]
67106	NM_025970,XM_006503304,XM_017320361	Zbtb8os	zinc finger and BTB domain containing 8 opposite strand	protein_coding	NP_080246.1;XP_006503367.1;XP_017175850.1	1914356	.	3.754339	[illegible]
67109	NM_001013012,XM_006540311,XM_006540312,XM_006540313,XM_030242920	Zfp787	zinc finger protein 787	protein_coding	NP_001013030.1;XP_006540374.1;XP_006540375.1;XP_006540376.1;XP_030098780.1	1914359	.	5.660816	[illegible]
67111	NM_001163687,NM_025972	Naaa	N-acylethanolamine acid amidase	protein_coding	NP_001157159.1;NP_080248.2	1914361	.	4.933291	[illegible]
67115	NM_025974	Rpl14	ribosomal protein L14	protein_coding	NP_080250.1	1914365	.	536.427856	[illegible]
67116	NM_001164290,NM_001164291,NM_001164292,NM_001164293,NM_001164294,NM_001164295,NM_024192,XM_006527275,XM_006527276,XM_006527277,XM_006527278,XM_006527279,XM_006527280,XM_011247317,XM_011247318,XM_011247319,XM_011247320,XM_011247321,XM_030251033,XM_030251034,XM_030251035,XM_030251036,XM_030251037,XM_030251038,XM_030251039,XM_030251040,XM_030251041,XM_030251042,XM_030251043,XM_030251044	Cuedc2	CUE domain containing 2	protein_coding	NP_001157762.1;NP_001157763.1;NP_001157764.1;NP_001157765.1;NP_001157766.1;NP_001157767.1;NP_077154.2;XP_006527338.1;XP_006527339.1;XP_006527340.1;XP_006527341.1;XP_006527342.1;XP_006527343.1;XP_011245619.1;XP_011245620.1;XP_011245621.1;XP_011245622.1;XP_011245623.1;XP_030106893.1;XP_030106894.1;XP_030106895.1;XP_030106896.1;XP_030106897.1;XP_030106898.1;XP_030106899.1;XP_030106900.1;XP_030106901.1;XP_030106902.1;XP_030106903.1;XP_030106904.1	1914366	.	25.365164	[illegible]
67117	NM_025975	Dynlt3	dynein light chain Tctex-type 3	protein_coding	NP_080251.3	1914367	.	24.664770	[illegible]
67118	NM_001177552,NM_025653,NM_025976,XM_030249248	Bfar	bifunctional apoptosis regulator	protein_coding	NP_001171023.1;NP_079929.1;NP_080252.1;XP_030105108.1	1914368	.	21.247401	[illegible]
67119	NM_001164614,NM_025977	Ccdc159	coiled-coil domain containing 159	protein_coding	NP_001158086.1;NP_080253.2	1914369	.	1.766844	[illegible]
67120	NM_001290500,NM_001290502,NM_025978,NM_027619,XM_006535522,XM_006535524,XM_011249706,XM_011249707,XM_030252768,XR_003954371,XR_880651	Ttc14	tetratricopeptide repeat domain 14	protein_coding	NP_001277429.1;NP_001277431.1;NP_080254.2;NP_081895.2;XP_006535585.1;XP_006535587.1;XP_011248008.1;XP_011248009.1;XP_030108628.1	1914370	.	16.304906	[illegible]
67121	NM_025979,XM_017319214,XM_030251977	Mastl	microtubule associated serine/threonine kinase-like	protein_coding	NP_080255.3;XP_017174703.1;XP_030107837.1	1914371	.	2.584749	[illegible]
67122	NM_025980	Nrarp	Notch-regulated ankyrin repeat protein	protein_coding	NP_080256.2	1914372	.	3.121004	[illegible]
67123	NM_001290454,NM_001355508,NM_001355509,NM_023305,XM_006538196,XM_006538198	Ubap1	ubiquitin-associated protein 1	protein_coding	NP_001277383.1;NP_001342437.1;NP_001342438.1;NP_075794.3;XP_006538259.1;XP_006538261.1	2149543	.	11.598732	[illegible]
67125	NM_025982,XM_030245229,XM_030245230	Tspan31	tetraspanin 31	protein_coding	NP_080258.1;XP_030101089.1;XP_030101090.1	1914375	.	49.946545	[illegible]
67126	NM_025983	Atp5e	ATP synthase, H+ transporting, mitochondrial F1 complex, epsilon	protein_coding	NP_080259.1	1855697	.	121.580002	[illegible]
67128	NM_025985	Ube2g1	ubiquitin-conjugating enzyme E2G 1	protein_coding	NP_080261.2	1914378	.	11.733756	[illegible]
67130	NM_025987	Ndufa6	NADH:ubiquinone oxidoreductase subunit A6	protein_coding	NP_080263.1	1914380	.	62.727921	[illegible]
67131	NM_025988	Acbd4	acyl-Coenzyme A binding domain containing 4	protein_coding	NP_080264.1	1914381	.	7.445500	[illegible]
67134	NM_024193,NR_149733,XM_006500062,XR_374509,XR_866335,XR_866336	Nop56	NOP56 ribonucleoprotein	protein_coding	NP_077155.2;XP_006500125.1	1914384	.	38.582344	[illegible]
67136	NM_001311116,NM_001361372,NM_025991	Kbtbd4	kelch repeat and BTB (POZ) domain containing 4	protein_coding	NP_001298045.1;NP_001348301.1;NP_080267.1	1914386	.	6.367118	[illegible]
67138	NM_025992	Herc6	hect domain and RLD 6	protein_coding	NP_080268.1	1914388	.	0.638851	[illegible]
67139	NM_025993,XM_006533968,XM_006533969	Mis12	MIS12 kinetochore complex component	protein_coding	NP_080269.1;XP_006534031.1;XP_006534032.1	1914389	.	8.530224	[illegible]
67141	NM_025995	Fbxo5	F-box protein 5	protein_coding	NP_080271.2	1914391	.	6.433256	[illegible]
67143	NM_175115,XM_006508122	Ikzf5	IKAROS family zinc finger 5	protein_coding	NP_780324.2;XP_006508185.1	1914393	.	6.350719	[illegible]
67144	NM_001289524,NM_001289525,NM_001359763,NM_024194,XM_030252769	Lrrc40	leucine rich repeat containing 40	protein_coding	NP_001276453.1;NP_001276454.1;NP_001346692.1;NP_077156.2;XP_030108629.1	1914394	.	8.426261	[illegible]
67145	NM_001291155,NM_025996,XM_006500067,XR_003953697,XR_374511	Tomm34	translocase of outer mitochondrial membrane 34	protein_coding	NP_001278084.1;NP_080272.1;XP_006500130.1	1914395	.	12.905318	[illegible]
67148	NM_025997	Ramac	RNA guanine-7 methyltransferase activating subunit	protein_coding	NP_080273.1	1914398	.	20.344513	[illegible]
67149	NM_025998,XM_006539116,XM_006539117,XM_030253717	Nkain1	Na+/K+ transporting ATPase interacting 1	protein_coding	NP_080274.2;XP_006539179.1;XP_006539180.1;XP_030109577.1	1914399	.	3.499354	[illegible]
67150	NM_025999,XM_006508123,XM_006508124,XM_006508125,XM_030242921,XR_001777995,XR_003946542,XR_869742	Rnf141	ring finger protein 141	protein_coding	NP_080275.2;XP_006508186.1;XP_006508187.1;XP_006508188.1;XP_030098781.1	1914400	.	4.540566	[illegible]
67151	NM_026000	Psmd9	proteasome (prosome, macropain) 26S subunit, non-ATPase, 9	protein_coding	NP_080276.2	1914401	.	8.818169	[illegible]
67153	NM_026001	Rnaseh2b	ribonuclease H2, subunit B	protein_coding	NP_080277.1	1914403	.	8.173153	[illegible]
67154	NM_001357925,NM_001357926,NM_026002,XM_006520149,XM_006520151,XM_006520152,XM_017316728,XM_017316729,XM_017316730,XM_017316731,XM_030248710	Mtdh	metadherin	protein_coding	NP_001344854.1;NP_001344855.1;NP_080278.3;XP_006520212.1;XP_006520214.1;XP_006520215.1;XP_017172217.1;XP_017172218.1;XP_017172219.1;XP_017172220.1;XP_030104570.1	1914404	.	22.393523	[illegible]
67155	NM_001347439,NM_011416,NM_026003,XM_006527281,XM_006527282,XM_006527283,XM_006527284,XM_006527285,XM_006527286,XM_006527288,XM_006527289,XM_006527291,XM_017318276,XM_017318277,XM_017318278,XM_030251045,XM_030251046,XM_030251047,XM_030251048,XM_030251049,XM_030251050,XM_030251051,XR_386502	Smarca2	SWI/SNF related, matrix associated, actin dependent regulator of chromatin, subfamily a, member 2	protein_coding	NP_001334368.1;NP_035546.2;NP_080279.1;XP_006527344.1;XP_006527345.1;XP_006527346.1;XP_006527347.1;XP_006527348.1;XP_006527349.1;XP_006527351.1;XP_006527352.1;XP_006527354.1;XP_017173765.1;XP_017173766.1;XP_017173767.1;XP_030106905.1;XP_030106906.1;XP_030106907.1;XP_030106908.1;XP_030106909.1;XP_030106910.1;XP_030106911.1	99603	.	27.740298	[illegible]
67157	NM_026005,XM_006538200	2610301B20Rik	RIKEN cDNA 2610301B20 gene	protein_coding	NP_080281.3;XP_006538263.1	1914407	.	10.406588	[illegible]

67158	NM_026006	Sft2d3	SFT2 domain containing 3	protein_coding
67160	NM_026007	Eef1g	eukaryotic translation elongation factor 1 gamma	protein_coding
67161	NM_001081411,NR_155540,XM_006500786,XM_006500787,XM_011239970,XR_867171	Sclt1	sodium channel and clathrin linker 1	protein_coding
67163	NM_026009,XM_030246238	Ccdc47	coiled-coil domain containing 47	protein_coding
67164	NM_026010	Lipt2	lipoyl(octanoyl) transferase 2 (putative)	protein_coding
67166	NM_026011	Arl8b	ADP-ribosylation factor-like 8B	protein_coding
67168	NM_175116	Lpar6	lysophosphatidic acid receptor 6	protein_coding
67169	NM_026012	Nradd	neurotrophin receptor associated death domain	protein_coding
67170	NR_028298	2610306M01Rik	RIKEN cDNA 2610306M01 gene	lncRNA
67171	NM_001025582,NM_001286986,NM_001286987,NM_026013,XM_006501928,XM_006501929,XM_030252770	Dram2	DNA-damage regulated autophagy modulator 2	protein_coding
67177	NM_026014	Cdt1	chromatin licensing and DNA replication factor 1	protein_coding
67178	NM_001356390,NM_001356391,NM_026015,XM_006514787	Zmat5	zinc finger, matrin type 5	protein_coding
67179	NM_145944	Ccdc25	coiled-coil domain containing 25	protein_coding
67180	NM_023311	Yipf5	Yip1 domain family, member 5	protein_coding
67181	NM_026017	Ctdnep1	CTD nuclear envelope phosphatase 1	protein_coding
67182	NM_001164557,NM_001164558,NM_026018,XM_030253719	Pdzk1ip1	PDZK1 interacting protein 1	protein_coding
67184	NM_023312,XM_017312943	Ndufa13	NADH:ubiquinone oxidoreductase subunit A13	protein_coding
67186	NM_001360657,NM_001360658,NM_026020	Rplp2	ribosomal protein, large P2	protein_coding
67187	NM_026021,NM_147179	Zmynd19	zinc finger, MYND domain containing 19	protein_coding
67188	NR_033198	2700046G09Rik	RIKEN cDNA 2700046G09 gene	lncRNA
67194	NR_045040,NR_045042	2700038G22Rik	RIKEN cDNA 2700038G22 gene	lncRNA
67196	NM_001278115,NM_026024	Ube2t	ubiquitin-conjugating enzyme E2T	protein_coding
67197	NM_001347097,NM_026025,XM_011245713,XM_030248711	Zcrb1	zinc finger CCHC-type and RNA binding motif 1	protein_coding
67198	NM_001164566,NM_001368787,NM_144882,XM_006496210,XM_006496211,XM_006496212,XM_006496215,XM_006496216,XM_006496217,XM_006496218,XM_017322122,XM_017322123,XM_030242305,XM_030242308,XM_030242309	Spats2l	spermatogenesis associated, serine-rich 2-like	protein_coding
67199	NM_026027	Pfdn1	prefoldin 1	protein_coding
67200	NM_026028,NR_045558,XM_006506490,XM_006506491,XM_006506492,XM_006506493,XM_006506494,XM_006506495,XM_006506496,XM_006506499,XM_006506500,XM_011241432,XM_011241433,XM_011241434,XM_011241435,XM_011241436,XM_011241437,XM_011241439,XM_011241440,XM_030255553,XR_001785160	Ccdc77	coiled-coil domain containing 77	protein_coding
67201	NM_001362901,NM_026029	Glod4	glyoxalase domain containing 4	protein_coding
67203	NM_001114085,NM_001285503,NM_001285504,NM_023317,XM_011246000	Nde1	nudE neurodevelopment protein 1	protein_coding
67204	NM_026030	Eif2s2	eukaryotic translation initiation factor 2, subunit 2 (beta)	protein_coding
67205	NM_026031,XM_011240592,XR_003954997	Utp11	UTP11 small subunit processome component	protein_coding
67207	NM_026032,XM_011242156	Lsm1	LSM1 homolog, mRNA degradation associated	protein_coding
67210	NM_026033	Gatad1	GATA zinc finger domain containing 1	protein_coding
67211	NM_001363422,NM_026034	Armc10	armadillo repeat containing 10	protein_coding
67212	NM_001302334,NM_001302335,NM_001302336,NM_001362908,NM_001362909,NM_026035,NR_126101,XM_006533974,XM_006533975,XM_006533976,XM_030246239,XM_030246240,XM_0302462	Mrpl55	mitochondrial ribosomal protein L55	protein_coding
67213	NM_026036,XM_006512242	Cmtm6	CKLF-like MARVEL transmembrane domain containing 6	protein_coding
67216	NM_001083341,NM_026037,XM_006515167,XM_006515168,XM_006515170,XM_006515172,XM_011243889,XM_011243890,XM_017315164,XM_030246883,XM_030246884,XM_030246885,XM_030246886	Mboat2	membrane bound O-acyltransferase domain containing 2	protein_coding
67217	NM_026038,XM_011244161	L3hypdh	L-3-hydroxyproline dehydratase (trans-)	protein_coding
67219	NM_026039	Med18	mediator complex subunit 18	protein_coding
67220	NM_023320,XM_006501930,XM_011240206	Plekho1	pleckstrin homology domain containing, family O member 1	protein_coding
67222	NM_026040,XR_386008	Srfbp1	serum response factor binding protein 1	protein_coding
67223	NM_026041	Rrp15	ribosomal RNA processing 15 homolog (S. cerevisiae)	protein_coding
67224	NM_026042	Med29	mediator complex subunit 29	protein_coding
67225	NM_001038696,NM_001287015,NM_026043	Rnpc3	RNA-binding region (RNP1, RRM) containing 3	protein_coding
67226	NM_001359470,NM_001359471,NM_001359472,NM_133683,XM_006513977,XM_006513978,XM_030245231	Tmem19	transmembrane protein 19	protein_coding
67228	NM_001355554,NM_001355556,NM_001355557,NM_001355558,NM_001355559,NM_001355560,NM_001355561,NM_026044,XM_006498241,XM_006498242,XM_006498243,XM_006498245,XM_006498246,XM_006498247,XM_030251983,XM_030251984,XM_030251985	Dph7	diphthamine biosynthesis 7	protein_coding
67229	NM_001356451,NM_026045	Prpf18	pre-mRNA processing factor 18	protein_coding
67230	NM_026046,XM_006540315,XM_011250682,XM_030242923,XR_003946543,XR_003946544	Zfp329	zinc finger protein 329	protein_coding
67231	NM_024196,NR_156111,XR_001783169	Tbc1d20	TBC1 domain family, member 20	protein_coding
67235	NM_023322,XM_011241012	Zkscan14	zinc finger with KRAB and SCAN domains 14	protein_coding
67236	NM_026048,NM_027223	Cinp	cyclin-dependent kinase 2 interacting protein	protein_coding
67238	NM_026050,NM_133703	Fam220a	family with sequence similarity 220, member A	protein_coding
67239	NM_001042556,NM_023323	Rpf2	ribosome production factor 2 homolog	protein_coding
67241	NM_001324476,NM_001361252,NM_025695,XM_006515173,XM_011243892	Smc6	structural maintenance of chromosomes 6	protein_coding
67242	NM_001355458,NM_026053,XM_011246581	Gemin6	gem nuclear organelle associated protein 6	protein_coding
67245	NM_023324,XM_011243746	Peli1	pellino 1	protein_coding
67246	NM_001289661,NM_001289662,NM_026054,XM_006507081,XM_006507083,XM_006507084,XM_006507086,XM_011241622,XM_030255554	Resf1	retroelement silencing factor 1	protein_coding
67247	NM_001368285,NM_001368286,NM_133684	Marc2	mitochondrial amidoxime reducing component 2	protein_coding
67248	NM_026055	Rpl39	ribosomal protein L39	protein_coding
67249	NM_144517,XM_006504065,XM_006504067,XM_017321077,XM_030254772,XR_003955695,XR_003955696,XR_003955697	Tbc1d19	TBC1 domain family, member 19	protein_coding
67252	NM_026056,XM_006516946,XM_030247379	Cap2	CAP, adenylate cyclase-associated protein, 2 (yeast)	protein_coding
67254	NM_144518,XM_006522482	Bmerb1	bMERB domain containing 1	protein_coding
67255	NM_001302439,NM_001302440,NM_026057,XM_030255555	Zfp422	zinc finger protein 422	protein_coding
67260	NM_026058,XM_006508861,XM_006508862,XM_006508863,XM_030243732,XM_030243733,XM_030243734,XR_003947330,XR_003947331	Cers4	ceramide synthase 4	protein_coding
67261	NR_027851	2900005J15Rik	RIKEN cDNA 2900005J15 gene	lncRNA
67263	NM_145456	Zswim6	zinc finger SWIM-type containing 6	protein_coding
67264	NM_001347447,NM_026061	Ndufb8	NADH:ubiquinone oxidoreductase subunit B8	protein_coding
67266	NM_026062	Dipk1a	divergent protein kinase domain 1A	protein_coding
67267	NM_001364944,NM_026063,XM_006524821	Uqcc2	ubiquinol-cytochrome c reductase complex assembly factor 2	protein_coding
67268	NM_001357820,NM_026064	Myl12a	myosin, light chain 12A, regulatory, non-sarcomeric	protein_coding
67269	NM_001048008,NM_001284218,NM_001284219,NM_001284221,NM_023328,XM_006517335,XM_006517336,XM_006517337,XM_006517338,XM_006517339,XM_006517340,XM_006517342,XM_011244555,XM_011244556,XM_011244557,XM_030247380,XR_003950467,XR_003950468	Agtpbp1	ATP/GTP binding protein 1	protein_coding
67270	NM_001359476,NM_001359477,NM_026065	Mrpl42	mitochondrial ribosomal protein L42	protein_coding
67272	NM_026066,NM_029390,XM_017316162	Cmtm5	CKLF-like MARVEL transmembrane domain containing 5	protein_coding
67273	NM_024197,XM_030242321	Ndufa10	NADH:ubiquinone oxidoreductase subunit A10	protein_coding
67276	NM_026067	Eri1	exoribonuclease 1	protein_coding
67277	XR_003955834,XR_868680,XR_868681,XR_868682,XR_868683	2900089D17Rik	RIKEN cDNA 2900089D17 gene, transcript variant X5	lncRNA
67278	NM_030240	Pagr1a	PAXIP1 associated glutamate rich protein 1A	protein_coding
67279	NM_026068	Med31	mediator complex subunit 31	protein_coding
67281	NM_026069	Rpl37	ribosomal protein L37	protein_coding
67282	NM_001122960,NM_026070,NM_027487,XM_030245232,XR_003948777,XR_003948778,XR_003948779,XR_003948780,XR_003948781,XR_003948782,XR_003948783	Washc3	WASH complex subunit 3	protein_coding
67283	NM_001252384,NM_001252394,NM_001252395,NM_001252396,NM_026071,XM_006533979,XM_011249183,XM_017314714,XM_030246243,XM_030246244,XM_030246245,XM_030246246	Slc25a19	solute carrier family 25 (mitochondrial thiamine pyrophosphate carrier), member 19	protein_coding
67285	NM_001360094,NM_026072,XM_017315573,XM_017315574,XR_001780798,XR_001780799,XR_001780800,XR_001780801,XR_003950469	Cwc27	CWC27 spliceosome-associated protein	protein_coding
67286	NM_026073,XM_011240905	Ift22	intraflagellar transport 22	protein_coding
67287	NM_001205239,NM_029922,NR_038074,NR_038075,XM_006511375,XM_006511380,XM_011242795,XM_011242796,XM_011242797,XM_011242798,XM_011242799,XM_011242800,XM_011242801,XM_017313527,XM_017313528,XM_017313529,XM_017313530,XM_017313531,XM_017313532,XM_017313533,XM_017313534,XM_030244511,XM_030244512,XM_030244513,XM_030244514,XM_030244515,XM_030244516,XM_030244517,XM_030244518,XM_030244519,XM_030244520,XM_030244521,XM_030244522,XM_030244523,XM_030244524,XM_030244525,XR_001778987,XR_001778988,XR_001778989,XR_001778990,XR_003947977,XR_003947978,XR_003947979,XR_003947980,XR_003947981,XR_003947982,XR_003947983,XR_870762,XR_870763,XR_870764	Parp6	poly (ADP-ribose) polymerase family, member 6	protein_coding
67288	NM_026075,NR_135272	Srek1ip1	splicing regulatory glutamine/lysine-rich protein 1interacting protein 1	protein_coding
67289	NR_030776	3110021A11Rik	RIKEN cDNA 3110021A11 gene	lncRNA
67290	NM_001370770,NM_026077,XM_011250896,XM_011250897	3110040N11Rik	RIKEN cDNA 3110040N11 gene	protein_coding
67291	NM_152807,XM_006533981,XM_006533982	Ccdc137	coiled-coil domain containing 137	protein_coding
67292	NM_001039045,NM_026078,XM_006496959,XM_030242324	Pigc	phosphatidylinositol glycan anchor biosynthesis, class C	protein_coding
67293	NR_026733	3110039M20Rik	RIKEN cDNA 3110039M20 gene	lncRNA
67295	NM_001360099,NM_023852,XM_006517741,XM_006517742	Rab3c	RAB3C, member RAS oncogene family	protein_coding
67296	NM_080843	Socs4	suppressor of cytokine signaling 4	protein_coding
67298	NM_001004359,NM_001005385,NM_026081	Gprasp1	G protein-coupled receptor associated sorting protein 1	protein_coding
67299	NM_001290636,NM_001369285,NM_001369286,NM_026082,XM_006503308,XM_006503309,XM_006503310,XM_006503313,XM_006503314,XM_017320362,XM_030253720,XM_030253721,XR_001784187,XR_003954998	Dock7	dedicator of cytokinesis 7	protein_coding
67300	NM_001003908,NM_001356393,XM_006533983,XM_006533985	Cltc	clathrin, heavy polypeptide (Hc)	protein_coding
67302	NM_001359875,NM_026083,XM_006519446,XM_006519447,XM_006519448,XM_006519449,XM_011245157,XM_017316163,XM_017316164,XM_030247971	Zc3h13	zinc finger CCCH type containing 13	protein_coding
67303	NR_040438	3110045C21Rik	RIKEN cDNA 3110045C21 gene	lncRNA
67304	NR_027974	3110070M22Rik	RIKEN cDNA 3110070M22 gene	lncRNA
67305	NM_024198	Gpx7	glutathione peroxidase 7	protein_coding
67306	NM_173181,XM_006530086	Zc2hc1a	zinc finger, C2HC-type containing 1A	protein_coding
67307	NM_026085,XM_006513981,XM_006513982,XM_006513983	Pbld2	phenazine biosynthesis-like protein domain containing 2	protein_coding
67308	NM_023331	Mrpl46	mitochondrial ribosomal protein L46	protein_coding
67311	NM_026086,XM_006500068	Nanp	N-acetylneuraminic acid phosphatase	protein_coding
67313	NM_028872,XM_006529802,XM_006529803,XM_006529804,XM_006529805,XM_006529806	Inava	innate immunity activator	protein_coding
67326	NM_001311138,NM_001361375,NM_026091,NR_154886,NR_154887,XM_011239745,XM_030251987	1700037H04Rik	RIKEN cDNA 1700037H04 gene	protein_coding
67329	NR_028360	1700018L02Rik	RIKEN cDNA 1700018L02 gene	lncRNA
67330	NR_015458	1700047M11Rik	RIKEN cDNA 1700047M11 gene	lncRNA
67331	NM_026094,XM_006513984,XR_380430	Atp8b3	ATPase, class I, type 8B, member 3	protein_coding
67332	NM_026095	Snrpd3	small nuclear ribonucleoprotein D3	protein_coding
67333	NM_001038635,NM_183262	Stk35	serine/threonine kinase 35	protein_coding
67337	NM_024199,XM_006500072,XM_030251988	Cstf1	cleavage stimulation factor, 3' pre-RNA, subunit 1	protein_coding
67338	NM_001007465,NM_001164569,NM_001164570,NM_001164571,NM_026097,XM_006533987,XM_006533988,XM_006533989,XM_006533990,XM_017314715,XM_030246247	Rffl	ring finger and FYVE like domain containing protein	protein_coding
67341	NM_001163614	Ascl4	achaete-scute family bHLH transcription factor 4	protein_coding
67342	NR_028107	Kcnmb4os1	potassium large conductance calcium-activated channel, subfamily M, beta member 4, opposite strand 1	lncRNA
67344	NM_001163767,NM_001163768,NM_026100,XM_006503319,XM_006503321,XM_030253722	Tctex1d1	Tctex1 domain containing 1	protein_coding
67345	NM_001359484,NM_026101,NM_030114,XM_006513986,XM_006513988,XM_006513989,XM_006513990,XM_030245233,XM_030245234,XR_003948784,XR_003948785,XR_003948786,XR_380431	Herc4	hect domain and RLD 4	protein_coding
67349	XR_003955282,XR_003955283,XR_868180	1700086P04Rik	RIKEN cDNA 1700086P04 gene, transcript variant X1	lncRNA
67350	NR_028299	1700084E18Rik	RIKEN cDNA 1700084E18 gene	lncRNA
67358	NM_001110133,NM_001370913,NM_026105,XM_006514789,XM_006514790	1700093K21Rik	RIKEN cDNA 1700093K21 gene	protein_coding
67367	NM_026110,NR_152264	Paxbp1	PAX3 and PAX7 binding protein 1	protein_coding
67369	NM_026111	Qpctl	glutaminyl-peptide cyclotransferase-like	protein_coding
67370	NM_001039951,NM_001310620,NM_026112,XM_006540320,XR_881784	Zfp606	zinc finger protein 606	protein_coding
67371	NM_001359491,NM_001359492,NM_001359493,NM_026113	Gtf3c6	general transcription factor IIIC, polypeptide 6, alpha	protein_coding
67374	NM_023844,XM_006523075	Jam2	junction adhesion molecule 2	protein_coding
67375	NM_133686	Qprt	quinolinate phosphoribosyltransferase	protein_coding
67378	NM_026116,XM_006531304,XM_030243735,XR_001778463	Bbs2	Bardet-Biedl syndrome 2 (human)	protein_coding
67379	NM_207677,XM_030242925,XM_030242926,XM_030242927,XM_030242928,XR_003946545,XR_003946546,XR_391094	Dedd2	death effector domain-containing DNA binding protein 2	protein_coding
67381	NM_026119	Med4	mediator complex subunit 4	protein_coding
67382	NM_001113573,NM_001113574,NM_023336,XM_006498249,XM_006498250,XM_011239155,XM_011239156,XM_011239157,XM_011239158,XM_011239159,XM_030251991,XM_030251992	Brd3	bromodomain containing 3	protein_coding
67383	NM_026120,XM_006527293,XM_006527294,XM_006527295,XM_030251052,XM_030251053	Carnmt1	carnosine N-methyltransferase 1	protein_coding
67384	NM_026121	Bag4	BCL2-associated athanogene 4	protein_coding
67387	NM_001356528,NM_026123,XM_006496220,XM_006496221,XM_006496223,XM_030242345	Unc50	unc-50 homolog	protein_coding
67388	NM_026124	Rab5if	RAB5 interacting factor	protein_coding
67389	NM_026125	C1qtnf12	C1q and tumor necrosis factor related 12	protein_coding
67390	NM_183263,XM_030246248	Mrm3	mitochondrial rRNA methyltransferase 3	protein_coding
67391	NM_026126	Fundc2	FUN14 domain containing 2	protein_coding
67392	NM_001113550,NM_001360805,NM_026127,NR_153825,XM_006517743,XM_006517744,XM_006517747,XM_006517748,XM_006517749,XM_006517750,XM_030247381,XM_030247382,XM_030247383,XM_030247384,XR_001780802,XR_003950470,XR_003950471,XR_873788	4833420G17Rik	RIKEN cDNA 4833420G17 gene	protein_coding
67393	NM_001357458,NM_001357459,NM_133687,XM_006526176,XM_006526180,XM_006526181,XM_030250576	Cxxc5	CXXC finger 5	protein_coding
67394	NR_028368	4930404I05Rik	RIKEN cDNA 4930404I05 gene	lncRNA
67397	NM_026129	Erp29	endoplasmic reticulum protein 29	protein_coding
67398	NM_026130	Srpr	signal recognition particle receptor ('docking protein')	protein_coding
67399	NM_001114087,NM_001114088,NM_026131,NR_104287,XM_006517344,XM_006517345,XM_006517346,XM_006517349,XM_011244558,XM_011244559	Pdlim7	PDZ and LIM domain 7	protein_coding
67405	NM_024435,XM_006513992	Nts	neurotensin	protein_coding
67412	NM_026138	Soga3	SOGA family member 3	protein_coding
67414	NM_024200,XR_001783736	Mfn1	mitofusin 1	protein_coding
67416	NM_001166397,NM_001166398,NM_026139,XM_006528600,XM_006528602,XM_006528603	Armcx2	armadillo repeat containing, X-linked 2	protein_coding
67417	NM_026140,XM_030242929,XR_003946547	Ears2	glutamyl-tRNA synthetase 2, mitochondrial	protein_coding
67418	NM_026141,XM_006512832,XR_001779581	Ppil4	peptidylprolyl isomerase (cyclophilin)-like 4	protein_coding
67419	NM_001358244,NM_026142,XM_006519452	Armh4	armadillo-like helical domain containing 4	protein_coding
67420	NM_001285831,NM_001360668,NM_001360669,NM_027379,XM_006508129,XM_006508130,XM_006508131,XM_030242930,XM_030242931,XM_030242932	Far1	fatty acyl CoA reductase 1	protein_coding
67422	NM_001362959,NM_001362960,NM_001362961,NM_001362962,NM_026144,XM_030253723,XM_030253724	Dhdds	dehydrodolichyl diphosphate synthase	protein_coding
67425	NM_001290416,NM_026146,XM_006540323,XM_006540324,XM_006540325,XM_006540326,XM_017312258,XR_003946548,XR_003946549,XR_391095,XR_391096	Eps8l1	EPS8-like 1	protein_coding
67426	NM_001163290,NM_001359268,NM_001359269,NM_023341,XM_006496961,XM_006496962	Coq8a	coenzyme Q8A	protein_coding
67427	NM_026147	Rps20	ribosomal protein S20	protein_coding
67429	NM_001113554,NM_026149,XM_030248713,XM_030248714	Nudcd1	NudC domain containing 1	protein_coding
67430	NM_026150,XR_001780041	4921536K21Rik	RIKEN cDNA 4921536K21 gene	protein_coding
67432	NM_026152,XM_030251054,XM_030251055	Hoga1	4-hydroxy-2-oxoglutarate aldolase 1	protein_coding
67433	NM_001168658,NM_001168659,NM_024201	Ccdc127	coiled-coil domain containing 127	protein_coding
67434	NM_001164441,NM_001357922,NM_001357923,NM_026153,NM_027496,XM_006520155	Ankrd33b	ankyrin repeat domain 33B	protein_coding
67437	NM_026155	Ssr3	signal sequence receptor, gamma	protein_coding
67439	NM_026156,XM_006508864,XM_011242072,XM_030243737,XR_870082	Xab2	XPA binding protein 2	protein_coding
67440	NM_001361008,NM_001361009,NM_026157,XM_006526184	Mtpap	mitochondrial poly(A) polymerase	protein_coding
67441	NM_026158,XM_006540328	Isoc2b	isochorismatase domain containing 2b	protein_coding
67442	NM_026159	Retsat	retinol saturase (all trans retinol 13,14 reductase)	protein_coding
67443	NM_001364358,NM_026160	Map1lc3b	microtubule-associated protein 1 light chain 3 beta	protein_coding

67444	NM_001355152,NM_023343,XM_006529807,XM_006529808,XM_006529809,XM_006529810,XM_006529811,XM_006529812,XM_006529813,XM_006529814,XM_006529815,XM_011248065,XM_017322168,XM_030242380,XM_030242385,XM_030242386,XM_030242387,XM_030242399,XM_030242401,XM_030242404,XM_030242405,XM_030242410,XM_030242413,XM_030242415,XM_030242418,XM_030242421,XM_030242422,XM_030242424,XM_030242426	Ilkap	integrin-linked kinase-associated serine/threonine phosphatase 2C	protein_coding	NP_001342081.1;NP_075832.1;XP_006529870.1;XP_006529871.1;XP_006529872.1;XP_006529873.1;XP_006529874.1;XP_006529875.1;XP_006529876.1;XP_006529877.1;XP_006529878.1;XP_011246367.1;XP_017177657.1;XP_030098240.1;XP_030098245.1;XP_030098246.1;XP_030098247.1;XP_030098259.1;XP_030098261.1;XP_030098264.1;XP_030098265.1;XP_030098270.1;XP_030098273.1;XP_030098275.1;XP_030098278.1;XP_030098281.1;XP_030098282.1;XP_030098284.1;XP_030098286.1	1914694	.	28.593775	26.951684	25.909384	[illegible]
67445	NM_026161,XM_006500079,XM_006500080,XM_030251994,XM_030251995	C1qtnf4	C1q and tumor necrosis factor related protein 4	protein_coding	NP_080437.2;XP_006500142.1;XP_006500143.1;XP_030107854.1;XP_030107855.1	1914695	.	1.150898	0.728169	0.663153	[illegible]
67446	NM_175118	Dusp28	dual specificity phosphatase 28	protein_coding	NP_780327.1	1914696	.	2.949497	3.891577	2.064597	[illegible]
67448	NM_026162,XM_006497531	Plxdc2	plexin domain containing 2	protein_coding	NP_080438.2;XP_006497594.1	1914698	.	5.986511	5.777901	4.912871	[illegible]
67451	NM_026163	Pkp2	plakophilin 2	protein_coding	NP_080439.1	1914701	.	0.352910	1.355393	0.506987	[illegible]
67452	NM_026164,XM_006516174	Pnpla8	patatin-like phospholipase domain containing 8	protein_coding	NP_080440.2;XP_006516237.1	1914702	.	12.241326	12.117894	10.762334	[illegible]
67453	NM_001357461,NM_026165	Slc25a46	solute carrier family 25, member 46	protein_coding	NP_001344390.1;NP_080441.1	1914703	.	11.017879	10.198966	9.793477	[illegible]
67454	NM_026166,NM_027078,XM_011243536,XM_017314068,XR_001779582	Ikbip	IKBKB interacting protein	protein_coding	NP_080442.2;NP_081354.1;XP_011241838.1;XP_017169557.1	1914704	.	54.523311	43.771108	54.896680	[illegible]
67455	NM_001290476,NM_001310466,NM_026167,XM_011247484,XM_011247485,XM_011247486,XM_011247487,XM_011247488,XM_011247489,XM_011247492,XM_011247493,XM_011247494,XM_017318610,XM_030251489,XM_030251490,XM_030251491	Klhl13	kelch-like 13	protein_coding	NP_001277405.1;NP_001297395.1;NP_080443.1;XP_011245786.1;XP_011245787.1;XP_011245788.1;XP_011245789.1;XP_011245790.1;XP_011245791.1;XP_011245794.1;XP_011245795.1;XP_011245796.1;XP_017174099.1;XP_030107349.1;XP_030107350.1;XP_03010735	1914705	.	10.939342	13.247272	11.609075	[illegible]
67456	NM_001286560,NM_026168,XM_006507087,XM_006507089,XM_011241623,XM_017321716,XM_017321717,XM_030255556,XM_030255557,XM_030255558,XM_030255559,XM_030255560,XM_030255561,XR_001785162,XR_378169	Ergic2	ERGIC and golgi 2	protein_coding	NP_001273489.1;NP_080444.1;XP_006507150.1;XP_006507152.1;XP_011239925.1;XP_017177205.1;XP_017177206.1;XP_030111416.1;XP_030111417.1;XP_030111418.1;XP_030111419.1;XP_030111420.1;XP_030111421.1	1914706	.	24.654849	23.256254	27.381980	[illegible]
67457	NM_026169,XM_006531828,XM_006531829	Frmd8	FERM domain containing 8	protein_coding	NP_080445.1;XP_006531891.1;XP_006531892.1	1914707	.	7.745186	7.207694	7.671778	[illegible]
67458	NM_026170,XM_006524823,XM_006524824,XM_006524825,XM_030250005,XM_030250006,XM_030250008,XM_030250009,XM_030250010	Ergic1	endoplasmic reticulum-golgi intermediate compartment (ERGIC) 1	protein_coding	NP_080446.1;XP_006524886.1;XP_006524887.1;XP_006524888.1;XP_030105865.1;XP_030105866.1;XP_030105868.1;XP_030105869.1;XP_030105870.1	1914708	.	43.231680	42.893474	45.047907	[illegible]
67459	NM_026171,XM_006496964,XM_011238856,XM_011238857,XM_030242438,XR_003946442,XR_003946443,XR_373614	Nvl	nuclear VCP-like	protein_coding	NP_080447.1;XP_006497027.1;XP_011237158.1;XP_011237159.1;XP_030098298.1	1914709	.	6.864303	6.257890	6.876261	[illegible]
67460	NM_026172	Decr1	2,4-dienoyl CoA reductase 1, mitochondrial	protein_coding	NP_080448.1	1914710	.	12.434515	22.447815	9.434758	[illegible]
67463	NM_026173,XM_006517751,XR_001780803	Poc5	POC5 centriolar protein	protein_coding	NP_080449.1;XP_006517814.1	1914713	.	4.960682	4.890297	5.025816	[illegible]
67464	NM_001360352,NM_026174	Entpd4	ectonucleoside triphosphate diphosphohydrolase 4	protein_coding	NP_001347281.1;NP_080450.1	1914714	.	14.774788	14.648539	14.421488	[illegible]
67465	NM_026175	Sf3a1	splicing factor 3a, subunit 1	protein_coding	NP_080451.4	1914715	.	13.004999	12.155790	14.318274	[illegible]
67466	NM_026176,XM_006498252	Pdcl	phosducin-like	protein_coding	NP_080452.2;XP_006498315.1	1914716	.	10.817881	10.594893	10.879021	[illegible]
67467	NM_026177	Gpalpp1	GPALPP motifs containing 1	protein_coding	NP_080453.2	1914717	.	6.870054	6.183415	6.179400	[illegible]
67468	NM_026178,XM_011249187,XM_017314717	Mmd	monocyte to macrophage differentiation-associated	protein_coding	NP_080454.1;XP_011247489.1;XP_017170206.1	1914718	.	9.639606	9.795210	9.941600	[illegible]
67469	NM_001359207,NM_026179,NR_152865,XM_030244526	Abhd5	abhydrolase domain containing 5	protein_coding	NP_001346136.1;NP_080455.1;XP_030100386.1	1914719	.	8.119849	7.072334	8.905571	[illegible]
67470	NM_001286005,NM_001347418,NM_026180,NR_104382	Abcg8	ATP binding cassette subfamily G member 8	protein_coding	NP_001272934.1;NP_001334347.1;NP_080456.1	1914720	.	0.106878	0.118673	0.083833	[illegible]
67471	NM_026181,XM_030242933,XM_030242934,XM_030242935	Gpatch1	G patch domain containing 1	protein_coding	NP_080457.1;XP_030098793.1;XP_030098794.1;XP_030098795.1	1914721	.	5.543008	4.974875	5.111351	[illegible]
67472	NM_001253390,NM_001253391,NM_001356295,NM_026182,NR_045575,XM_011249708	Mtfr1	mitochondrial fission regulator 1	protein_coding	NP_001240319.1;NP_001240320.1;NP_001343224.1;NP_080458.1;XP_011248010.1	1914722	.	9.730644	8.978969	11.167783	[illegible]
67473	NM_026183,XM_006533993,XM_006533995,XM_011249188,XM_011249189,XR_003949493	Slc47a1	solute carrier family 47, member 1	protein_coding	NP_080459.2;XP_006534056.1;XP_006534058.1;XP_011247490.1;XP_011247491.1	1914723	.	3.209001	5.049803	3.189924	[illegible]
67474	NM_023348	Snap29	synaptosomal-associated protein 29	protein_coding	NP_075837.3	1914724	.	7.879193	6.773300	7.315696	[illegible]
67475	NM_026184,XM_006516730,XM_006516731,XM_006516732	Ero1lb	ERO1-like beta (S. cerevisiae)	protein_coding	NP_080460.2;XP_006516793.1;XP_006516794.1;XP_006516795.1	1914725	.	0.454764	1.203920	0.478592	[illegible]
67477	NM_026185	Abhd15	abhydrolase domain containing 15	protein_coding	NP_080461.3	1914727	.	0.964799	1.119998	0.863529	[illegible]
67480	NM_026186	Cwc25	CWC25 spliceosome-associated protein	protein_coding	NP_080462.2	1914730	.	7.362610	5.991465	7.212828	[illegible]
67483	NM_026188	1700028P14Rik	RIKEN cDNA 1700028P14 gene	protein_coding	NP_080464.1	1914733	.	0.000000	0.000000	0.000000	[illegible]
67484	NM_026189,XM_006510556	Eepd1	endonuclease/exonuclease/phosphatase family domain containing 1	protein_coding	NP_080465.3;XP_006510619.1	1914734	.	2.479049	3.552103	2.416185	[illegible]
67486	NM_001081176	Polr3g	polymerase (RNA) III (DNA directed) polypeptide G	protein_coding	NP_001074645.1	1914736	.	1.772755	1.582741	2.039041	[illegible]
67487	NM_026191,XM_017314718	Dhx40	DEAH (Asp-Glu-Ala-His) box polypeptide 40	protein_coding	NP_080467.3;XP_017170207.1	1914737	.	9.766691	10.213636	8.457967	[illegible]
67488	NM_026192	Calcoco1	calcium binding and coiled coil domain 1	protein_coding	NP_080468.1	1914738	.	22.698357	26.056044	18.440928	[illegible]
67489	NM_001163552,NM_001163553,NM_026193,XM_006501932,XM_006501933,XR_375568	Ap4b1	adaptor-related protein complex AP-4, beta 1	protein_coding	NP_001157024.1;NP_001157025.1;NP_080469.2;XP_006501995.1;XP_006501996.1	1337130	.	9.825828	8.595148	7.432921	[illegible]
67490	NM_001355512,NM_026194	Ufl1	UFM1 specific ligase 1	protein_coding	NP_001342441.1;NP_080470.2	1914740	.	11.232422	10.369964	9.710706	[illegible]
67492	NM_001290338,NM_001290339,NM_001309446,XM_006506506,XM_006506507,XM_006506508,XM_006506510,XM_006506515,XM_011241441,XM_011241442,XM_011241443,XM_011241444,XM_011241445,XM_030255562,XM_030255563,XM_030255564	Zfand4	zinc finger, AN1-type domain 4	protein_coding	NP_001277267.1;NP_001277268.1;NP_001296375.1;XP_006506569.1;XP_006506570.1;XP_006506571.1;XP_006506573.1;XP_006506578.1;XP_011239743.1;XP_011239744.1;XP_011239745.1;XP_011239746.1;XP_011239747.1;XP_030111422.1;XP_030111423.1;XP_0301	1914742	.	1.380099	0.546936	0.790170	[illegible]
67493	NM_026197,XM_006533998,XM_006533999,XM_006534000,XM_006534001,XM_017314719,XM_030246249	Mettl16	methyltransferase like 16	protein_coding	NP_080473.1;XP_006534061.1;XP_006534062.1;XP_006534063.1;XP_006534064.1;XP_017170208.1;XP_030102109.1	1914743	.	4.745046	3.518201	4.345258	[illegible]
67495	NM_026198	Tmem167b	transmembrane protein 167B	protein_coding	NP_080474.2	1914745	.	25.437645	22.684263	26.227446	[illegible]
67498	NM_026200,XM_006521297	Kcnv1	potassium channel, subfamily V, member 1	protein_coding	NP_080476.2;XP_006521360.1	1914748	.	0.003175	0.010802	0.000000	[illegible]
67500	NM_026201,XM_006513994,XM_006513995,XM_006513996,XM_006513997,XM_011243537	Ccar1	cell division cycle and apoptosis regulator 1	protein_coding	NP_080477.1;XP_006514057.1;XP_006514058.1;XP_006514059.1;XP_006514060.1;XP_011241839.1	1914750	.	24.922694	18.714805	21.705361	[illegible]
67501	NM_001025615,NM_001289436,NM_026202,XM_006522483,XM_011246002,XM_030249249	Ccdc50	coiled-coil domain containing 50	protein_coding	NP_001020786.1;NP_001276365.1;NP_080478.2;XP_006522546.1;XP_011244304.1;XP_030105109.1	1914751	.	24.972952	22.203849	23.706100	[illegible]
67503	NR_033199	1700001G17Rik	RIKEN cDNA 1700001G17 gene	lncRNA	.	1914753	.	0.295751	0.321055	0.163228	[illegible]
67504	NM_026205	Rnf151	ring finger protein 151	protein_coding	NP_080481.1	1914754	.	0.039650	0.000000	0.163847	[illegible]
67509	NM_026209,XM_017316166	Saysd1	SAYSVFN motif domain containing 1	protein_coding	NP_080485.1;XP_017171655.1	1914759	.	4.239954	3.542803	4.062849	[illegible]
67510	NM_001356520,NM_001356521,NM_026210	Tvp23b	trans-golgi network vesicle protein 23B	protein_coding	NP_001343449.1;NP_001343450.1;NP_080486.1	1914760	.	25.942293	23.171597	22.898535	[illegible]
67511	NM_026211	Tmed9	transmembrane p24 trafficking protein 9	protein_coding	NP_080487.2	1914761	.	106.291412	97.109962	115.670822	[illegible]
67512	NM_026212,XM_017319222	Agpat2	1-acylglycerol-3-phosphate O-acyltransferase 2 (lysophosphatidic acid acyltransferase, beta)	protein_coding	NP_080488.1;XP_017174711.1	1914762	.	10.960973	10.670063	10.759611	[illegible]
67513	NM_001190445,NM_001347262,NR_033802,XM_011250323,XM_030253725	Faap20	Fanconi anemia core complex associated protein 20	protein_coding	NP_001177374.1;NP_001334191.1;XP_011248625.2;XP_030109585.1	1914763	.	14.867102	14.890904	15.227655	[illegible]
67515	NM_001355620,NM_001355621,NM_026213,XM_030248716	Ttc33	tetratricopeptide repeat domain 33	protein_coding	NP_001342549.1;NP_001342550.1;NP_080489.1;XP_030104576.1	1914765	.	8.751766	9.197095	7.764343	[illegible]
67516	NM_026214	Kctd4	potassium channel tetramerisation domain containing 4	protein_coding	NP_080490.3	1914766	.	3.325144	1.945458	3.649220	[illegible]
67518	NR_152103	1700039M10Rik	RIKEN cDNA 1700039M10 gene	lncRNA	.	1914768	.	0.171058	0.000000	0.000000	[illegible]
67522	NR_045287	1700097N02Rik	RIKEN cDNA 1700097N02 gene	lncRNA	.	1914772	.	0.000000	0.063588	0.000000	[illegible]
67525	NM_001370756,NM_001370757,NM_178110	Trim62	tripartite motif-containing 62	protein_coding	NP_001357685.1;NP_001357686.1;NP_835211.1	1914775	.	2.597481	2.731220	2.621479	[illegible]
67526	NM_026217,XM_006526187	Atg12	autophagy related 12	protein_coding	NP_080493.2;XP_006526250.1	1914776	.	10.882115	10.369475	8.763237	[illegible]
67528	NM_001290180,NM_001290181,NM_001290182,NM_024437,NM_024446,XM_011248491,XM_017312945,XM_030243738	Nudt7	nudix (nucleoside diphosphate linked moiety X)-type motif 7	protein_coding	NP_001277109.1;NP_001277110.1;NP_001277111.1;NP_077757.2;NP_077766.3;XP_011246793.1;XP_017168434.1;XP_030099598.1	1914778	.	4.965815	5.419353	3.381097	[illegible]
67529	NM_001347513,NM_026218	Fgfr1op2	FGFR1 oncogene partner 2	protein_coding	NP_001334442.1;NP_080494.1	1914779	.	25.796277	23.601018	26.016929	[illegible]
67530	NM_026219	Uqcrb	ubiquinol-cytochrome c reductase binding protein	protein_coding	NP_080495.1	1914780	.	66.150780	81.814774	72.490837	[illegible]
67531	NR_027866	5730408K05Rik	RIKEN cDNA 5730408K05 gene	lncRNA	.	1914781	.	2.757255	5.095195	4.061211	[illegible]
67532	NM_026220	Mfap1a	microfibrillar-associated protein 1A	protein_coding	NP_080496.1	1914782	.	12.556679	11.085893	11.732483	[illegible]
67533	NM_001170433,NM_001355728,NM_001355729,NM_026221,XM_006507096,XM_006507097,XM_006507098,XM_006507099,XM_006507101,XM_006507102,XM_006507103,XM_011241624,XM_011241625,XM_011241626,XM_011241627,XM_011241628,XM_011241629,XM_030255565	Ppfibp1	PTPRF interacting protein, binding protein 1 (liprin beta 1)	protein_coding	NP_001163904.1;NP_001342657.1;NP_001342658.1;NP_080497.1;XP_006507159.1;XP_006507160.1;XP_006507161.1;XP_006507162.1;XP_006507164.1;XP_006507165.1;XP_006507166.1;XP_011239926.1;XP_011239927.1;XP_011239928.1;XP_011239929.1;XP_011239930.1;XP_011239931.1;XP_030111425.1	1914783	.	34.213088	24.408060	31.182633	[illegible]
67534	NM_001014974,XM_006496224,XM_006496225	Ttll4	tubulin tyrosine ligase-like family, member 4	protein_coding	NP_001014974.1;XP_006496287.1;XP_006496288.1	1914784	.	9.594438	7.960216	9.710645	[illegible]
67537	NM_001347157,NM_026223,XM_006513998	Glipr1l2	GLI pathogenesis-related 1 like 2	protein_coding	NP_001334086.1;NP_080499.1;XP_006514061.1	1914787	.	0.029934	0.000000	0.000000	[illegible]
67538	NM_178375	Zswim3	zinc finger SWIM-type containing 3	protein_coding	NP_848462.1	1914788	.	1.642680	2.135994	1.547428	[illegible]
67542	NM_026225	Cog6	component of oligomeric golgi complex 6	protein_coding	NP_080501.2	1914792	.	18.586636	18.266640	20.414593	[illegible]
67544	NM_001357822,NM_024203,NR_033586	Fam120b	family with sequence similarity 120, member B	protein_coding	NP_001344751.1;NP_077165.2	1914794	.	7.462992	8.075701	6.613976	[illegible]
67547	NM_001135149,NM_001135150,NM_026228,XM_006501934,XM_006501935,XM_006501936	Slc39a8	solute carrier family 39 (metal ion transporter), member 8	protein_coding	NP_001128621.1;NP_001128622.1;NP_080504.3;XP_006501997.1;XP_006501998.1;XP_006501999.1	1914797	.	1.610007	1.194564	1.088060	[illegible]
67548	XR_003955234,XR_376519,XR_867991	4933424M12Rik	RIKEN cDNA 4933424M12 gene, transcript variant X2	lncRNA	.	1914798	.	0.027481	0.031167	0.000000	[illegible]
67549	NM_026229,XM_017319696,XR_375569	Gpr89	G protein-coupled receptor 89	protein_coding	NP_080505.1;XP_017175185.1	1914799	.	4.620428	5.079422	5.885667	[illegible]
67552	NR_003523	H2afy3	H2A histone family, member Y3	transcribed_pseudogene	.	1914802	.	0.000000	0.013919	0.000000	[illegible]
67553	NM_001356309,NM_026231,XR_003954372	Gstcd	glutathione S-transferase, C-terminal domain containing	protein_coding	NP_001343238.1;NP_080507.2	1914803	.	5.016940	5.292111	5.302130	[illegible]
67554	NM_026232,XM_006519455,XM_006519456	Slc25a30	solute carrier family 25, member 30	protein_coding	NP_080508.1;XP_006519518.1;XP_006519519.1	1914804	.	2.911278	2.048157	2.945945	[illegible]
67556	NM_026234	Pigm	phosphatidylinositol glycan anchor biosynthesis, class M	protein_coding	NP_080510.1	1914806	.	4.386535	4.272862	4.279921	[illegible]
67557	NM_026235	Larp6	La ribonucleoprotein domain family, member 6	protein_coding	NP_080511.2	1914807	.	7.529260	6.365549	6.390524	[illegible]
67561	NM_026236,XM_006512246	Wdr48	WD repeat domain 48	protein_coding	NP_080512.1;XP_006512309.1	1914811	.	15.676557	13.144470	13.451271	[illegible]
67563	NM_026238,XM_006524828,XM_006524829	Ciao3	cytosolic iron-sulfur assembly component 3	protein_coding	NP_080514.3;XP_006524891.1;XP_006524892.1	1914813	.	6.316274	7.826174	6.038928	[illegible]
67564	NM_026239	Tmem35a	transmembrane protein 35A	protein_coding	NP_080515.1	1914814	.	0.652269	0.605364	0.694297	[illegible]
67568	NM_026242	Mrfap1	Morf4 family associated protein 1	protein_coding	NP_080518.1	1914818	.	125.098305	110.960655	120.559479	[illegible]
67569	NM_001162368,NM_001162369,NM_001205098,NM_026243,XM_006514002,XM_006514003,XM_006514004,XM_006514005,XM_011243538,XM_011243539,XM_030245235,XM_030245236,XM_030245237	Mgat4c	MGAT4 family, member C	protein_coding	NP_001155840.1;NP_001155841.1;NP_001192027.1;NP_080519.2;XP_006514065.1;XP_006514066.1;XP_006514067.1;XP_006514068.1;XP_011241840.1;XP_011241841.1;XP_030101095.1;XP_030101096.1;XP_030101097.1	1914819	.	0.477899	0.475278	0.249718	[illegible]
67573	NM_001164311,NM_053083	Loxl4	lysyl oxidase-like 4	protein_coding	NP_001157783.1;NP_444313.3	1914823	.	27.666038	25.399780	30.325930	[illegible]
67574	NM_026247,NR_037145	Alg13	asparagine-linked glycosylation 13	protein_coding	NP_080523.2	1914824	.	4.488760	3.582437	4.367862	[illegible]
67576	NR_027966	4930429B21Rik	RIKEN cDNA 4930429B21 gene	lncRNA	.	1914826	.	0.302377	0.283615	0.076615	[illegible]
67578	NM_026251,XM_006500086,XM_017319224	Patl2	protein associated with topoisomerase II homolog 2 (yeast)	protein_coding	NP_080527.2;XP_006500149.1;XP_017174713.1	1914828	.	0.000000	0.000000	0.027099	[illegible]
67579	NM_001290676,NM_001290678,NM_026252,XM_006514793	Cpeb4	cytoplasmic polyadenylation element binding protein 4	protein_coding	NP_001277605.1;NP_001277607.1;NP_080528.2;XP_006514856.1	1914829	.	5.799550	7.585743	6.018454	[illegible]
67580	NM_001146021,NM_026253,XM_006519457,XM_006519458	Lrrc18	leucine rich repeat containing 18	protein_coding	NP_001139493.1;NP_080529.1;XP_006519520.1;XP_006519521.1	1914830	.	0.167077	0.388628	0.259564	[illegible]
67581	NM_001358433,NM_001358434,NM_026254,XM_006522485,XM_030249250,XM_030249251,XM_030249252,XM_030249253	Tbc1d23	TBC1 domain family, member 23	protein_coding	NP_001345362.1;NP_001345363.1;NP_080530.2;XP_006522548.1;XP_030105110.1;XP_030105111.1;XP_030105112.1;XP_030105113.1	1914831	.	21.438888	18.574545	18.130473	[illegible]
67582	NM_026255,XM_006506517,XM_006506518,XM_006506519,XM_006506520,XM_006506523,XM_006506524,XM_011241446,XM_017321720,XR_001785163,XR_003956252,XR_377486,XR_868874,XR_868875	Slc25a26	solute carrier family 25 (mitochondrial carrier, phosphate carrier), member 26	protein_coding	NP_080531.2;XP_006506580.1;XP_006506581.1;XP_006506582.1;XP_006506583.1;XP_006506586.1;XP_006506587.1;XP_011239748.1;XP_017177209.1	1914832	.	2.020205	2.126835	2.177028	[illegible]
67586	NM_026257,NR_045571,XM_006539122,XM_006539123,XM_006539124,XM_006539127,XM_011250325,XM_011250326,XM_017320364,XM_030253726,XR_390778	Ubxn11	UBX domain protein 11	protein_coding	NP_080533.1;XP_006539185.1;XP_006539186.1;XP_006539187.1;XP_006539190.1;XP_011248627.1;XP_011248628.1;XP_017175853.1;XP_030109586.1	1914836	.	2.557829	2.851885	2.654934	[illegible]
67588	NM_001164237,NM_026259,NR_104288,NR_104289,NR_104290	Rnf41	ring finger protein 41	protein_coding	NP_001157709.1;NP_080535.2	1914838	.	7.697924	7.764452	7.618220	[illegible]
67590	NM_001365072,NM_026260,XM_030251056,XR_001782613,XR_386503	Tctn3	tectonic family member 3	protein_coding	NP_001352001.1;NP_080536.2;XP_030106916.1	1914840	.	8.058414	6.942180	6.780669	[illegible]
67591	NM_026261	Ubl4b	ubiquitin-like 4B	protein_coding	NP_080537.1	1914841	.	0.000000	0.000000	0.000000	[illegible]
67592	NM_026262,XM_006514795	4930524B15Rik	RIKEN cDNA 4930524B15 gene	protein_coding	NP_080538.1;XP_006514858.1	1914842	.	0.000000	0.071829	0.024290	[illegible]
67596	NM_183264,XM_006514006,XM_030245238	Tespa1	thymocyte expressed, positive selection associated 1	protein_coding	NP_899087.2;XP_006514069.1;XP_030101098.1	1914846	.	0.032952	0.143908	0.000000	[illegible]
67602	NM_026267,NR_156733	Necap1	NECAP endocytosis associated 1	protein_coding	NP_080543.2	1914852	.	13.204811	11.824216	12.656593	[illegible]
67603	NM_026268	Dusp6	dual specificity phosphatase 6	protein_coding	NP_080544.1	1914853	.	11.264022	12.272553	9.667449	[illegible]
67604	NM_001163316,NM_001359238,NM_026269	Get4	golgi to ER traffic protein 4	protein_coding	NP_001156788.1;NP_001346167.1;NP_080545.2	1914854	.	20.786283	19.749983	19.839207	[illegible]
67605	NM_001253920,NM_001290694,NM_001290695,NM_026270,XM_006541115,XM_006541117,XM_006541118,XM_006541119,XM_006541120,XM_006541121,XM_030242936,XM_030242937,XM_030242938	Akt1s1	AKT1 substrate 1 (proline-rich)	protein_coding	NP_001240849.1;NP_001277623.1;NP_001277624.1;NP_080546.1;XP_006541178.1;XP_006541180.1;XP_006541181.1;XP_006541182.1;XP_006541183.1;XP_006541184.1;XP_030098796.1;XP_030098797.1;XP_030098798.1	1914855	.	27.261012	31.526646	30.338098	[illegible]
67606	NM_026271	Fibin	fin bud initiation factor homolog (zebrafish)	protein_coding	NP_080547.1	1914856	.	35.551811	34.117882	29.658688	[illegible]
67607	NM_001360674,NM_001360675,NM_001360677,NM_001360678,NM_001360680,NM_001360681,NM_001360683,NM_001360684,NM_023363	Zfp788	zinc finger protein 788	protein_coding	NP_001347603.1;NP_001347604.1;NP_001347606.1;NP_001347607.1;NP_001347609.1;NP_001347610.1;NP_001347612.1;NP_001347613.1;NP_075852.1	1914857	.	5.963575	5.378654	5.211831	[illegible]
67608	NM_026272	Narf	nuclear prelamin A recognition factor	protein_coding	NP_080548.3	1914858	.	10.134446	11.872944	9.175852	[illegible]
67609	NM_026273,XM_006522489	4930453N24Rik	RIKEN cDNA 4930453N24 gene	protein_coding	NP_080549.1;XP_006522552.1	1914859	.	8.807517	8.932964	8.743986	[illegible]
67610	NM_001359093,NM_026274	Rspry1	ring finger and SPRY domain containing 1	protein_coding	NP_001346022.1;NP_080550.3	1914860	.	8.971565	8.325183	8.876696	[illegible]
67615	NM_026275	Ube2r2	ubiquitin-conjugating enzyme E2R 2	protein_coding	NP_080551.1	1914865	.	22.996279	21.932314	20.194368	[illegible]
67618	NM_001326359,NM_026276,NR_136940,NR_136941,XM_006509877,XM_017313535,XM_017313536,XM_030244527	Aasdhppt	aminoadipate-semialdehyde dehydrogenase-phosphopantetheinyl transferase	protein_coding	NP_001313288.1;NP_080552.3;XP_006509940.1;XP_017169024.1;XP_017169025.1;XP_030100387.1	1914868	.	8.414902	6.086720	7.738551	[illegible]
67619	NM_026277	Nob1	NIN1/RPN12 binding protein 1 homolog	protein_coding	NP_080553.1	1914869	.	16.647623	14.474526	15.952493	[illegible]
67620	NM_026278,XM_006509483,XR_378911	Lrp2bp	Lrp2 binding protein	protein_coding	NP_080554.1;XP_006509546.1	1914870	.	0.231202	0.322302	0.192696	[illegible]
67621	NM_026279,NR_033793,XM_006503323,XM_011240594,XM_017320366,XR_376343	Bend5	BEN domain containing 5	protein_coding	NP_080555.1;XP_006503386.1;XP_011238896.1;XP_017175855.1	1914871	.	2.942825	3.139969	2.082278	[illegible]
67622	NM_001347633,NM_026280,XM_006534006,XM_011249191,XR_001780042	Mxra7	matrix-remodelling associated 7	protein_coding	NP_001334562.1;NP_080556.1;XP_006534069.1;XP_011247493.1	1914872	.	23.798061	19.956276	14.556168	[illegible]
67623	NM_026281,XM_030255567,XM_030255568	Tm7sf3	transmembrane 7 superfamily member 3	protein_coding	NP_080557.2;XP_030111427.1;XP_030111428.1	1914873	.	22.862081	21.734396	22.551225	[illegible]
67628	NM_130889,XM_011250089	Anp32b	acidic (leucine-rich) nuclear phosphoprotein 32 family, member B	protein_coding	NP_570959.1;XP_011248391.1	1914878	.	75.283017	70.668231	79.318436	[illegible]
67629	NM_001357331,NM_026282,NR_151672,NR_151673,XR_00394798	Spc24	SPC24, NDC80 kinetochore complex component, homolog (S. cerevisiae)	protein_coding	NP_001344260.1;NP_080558.1	1914879	.	8.939549	7.779865	8.943840	[illegible]
67630	NM_001359953,NM_001359954,NM_026283	Samd8	sterile alpha motif domain containing 8	protein_coding	NP_001346882.1;NP_001346883.1;NP_080559.1	1914880	.	3.992830	4.048481	3.694961	[illegible]
67636	NM_001163628,NM_001362053,NM_001362054,NM_001362055,NM_001362056,NM_001362057,NM_001362058,NM_001362059,NM_133688,NR_155281,XM_006506983	Etfrf1	electron transfer flavoprotein regulatory factor 1	protein_coding	NP_001157100.1;NP_001348982.1;NP_001348983.1;NP_001348984.1;NP_001348985.1;NP_001348986.1;NP_001348987.1;NP_001348988.1;NP_598449.1;XP_006507046.1	1914886	.	7.856731	8.006561	7.205714	[illegible]
67638	NR_015603	4930483J18Rik	RIKEN cDNA 4930483J18 gene	lncRNA	.	1914888	.	0.000000	0.000000	0.000000	[illegible]
67639	NR_038130	4930474N09Rik	RIKEN cDNA 4930474N09 gene	lncRNA	.	1914889	.	0.135421	0.091381	0.000000	[illegible]
67640	NR_027978	4930465K10Rik	RIKEN cDNA 4930465K10 gene	lncRNA	.	1914890	.	0.000000	0.026524	0.056185	[illegible]
67642	NR_027872	4930515G01Rik	RIKEN cDNA 4930515G01 gene	lncRNA	.	1914892	.	0.228823	0.114122	0.140084	[illegible]
67644	XR_388001,XR_388002,XR_879152,XR_879153,XR_879154	4930517G24Rik	RIKEN cDNA 4930517G24 gene, transcript variant X4	lncRNA	.	1914894	.	0.000000	0.000000	0.038662	[illegible]
67647	NM_001162896,NR_028111,XM_006496968,XM_030242454	4930523C07Rik	RIKEN cDNA 4930523C07 gene	protein_coding	NP_001156368.1;XP_006497031.1;XP_030098314.1	1914897	.	9.846593	7.793625	6.926709	[illegible]
67652	NM_001290443,NM_026293	Spaca1	sperm acrosome associated 1	protein_coding	NP_001277372.1;NP_080569.1	1914902	.	2.805382	1.887283	2.903907	[illegible]
67655	NM_026295,XM_006526525,XM_011247096,XR_003952539	Ctdp1	CTD (carboxy-terminal domain, RNA polymerase II, polypeptide A) phosphatase, subunit 1	protein_coding	NP_080571.2;XP_006526588.1;XP_011245398.1	1926953	.	4.637137	5.027077	6.023805	[illegible]
67656	NM_026296	4930548H24Rik	RIKEN cDNA 4930548H24 gene	protein_coding	NP_080572.1	1914906	.	0.050294	0.000000	0.117041	[illegible]
67657	NM_001042499,XR_384595	Rabl3	RAB, member RAS oncogene family-like 3	protein_coding	NP_001035964.1	1914907	.	6.321337	4.885267	6.620118	[illegible]
67661	NM_026298,XR_003955698,XR_003955699	Ift172	intraflagellar transport 172	protein_coding	NP_080574.5	2682064	.	7.781009	9.442855	8.036247	[illegible]
67664	NM_001364973,NM_026301	Rnf125	ring finger protein 125	protein_coding	NP_001351902.1;NP_080577.1	1914914	.	1.288739	1.304581	0.986224	[illegible]
67665	NM_001357462,NM_026302	Dctn4	dynactin 4	protein_coding	NP_001344391.1;NP_080578.1	1914915	.	14.789225	15.310809	14.704673	[illegible]
67666	NM_178255,XM_006541127,XM_006541128	Hapln3	hyaluronan and proteoglycan link protein 3	protein_coding	NP_839986.2;XP_006541190.1;XP_006541191.1	1914916	.	0.752211	0.734225	0.573138	[illegible]
67667	NM_026303,XM_006509879,XM_006509880,XM_006509881,XM_006509882,XM_006509883,XM_030244528	Alkbh8	alkB homolog 8, tRNA methyltransferase	protein_coding	NP_080579.1;XP_006509942.1;XP_006509943.1;XP_006509944.1;XP_006509945.1;XP_006509946.1;XP_030100388.1	1914917	.	2.884754	2.366198	2.754547	[illegible]
67669	NM_001291286,NM_001291287,NM_001291288,NM_001291289,NM_026304,NR_111916,NR_153788,XM_006508132,XM_006508133	Hikeshi	heat shock protein nuclear import factor	protein_coding	NP_001278215.1;NP_001278216.1;NP_001278217.1;NP_001278218.1;NP_080580.1;XP_006508195.1;XP_006508196.1	96738	.	11.635433	14.096581	12.124259	[illegible]
67671	NM_001048057,NM_001048058,NM_001362918,NM_023372,XM_017314720	Rpl38	ribosomal protein L38	protein_coding	NP_001041522.1;NP_001041523.1;NP_001349847.1;NP_075861.1;XP_017170209.1	1914921	.	477.092186	378.096756	467.073802	[illegible]
67672	NR_027874	0610040B10Rik	RIKEN cDNA 0610040B10 gene	lncRNA	.	1914922	.	1.686578	1.068835	0.746320	[illegible]
67673	NM_026305	Elob	elongin B	protein_coding	NP_080581.1	1914923	.	132.341476	134.266785	139.895218	[illegible]
67674	NM_001166370,NM_026306	Trmt112	tRNA methyltransferase 11-2	protein_coding	NP_001159842.1;NP_080582.3	1914924	.	21.246455	19.264732	23.320399	[illegible]
67675	NM_026307,NM_026948,NR_104458,XM_006524832,XM_0302500	Cuta	cutA divalent cation tolerance homolog	protein_coding	NP_080583.3;NP_081224.1;XP_006524895.1;XP_030105873.1	1914925	.	23.077649	25.888359	25.361355	[illegible]
67676	NM_026308	Rpp21	ribonuclease P 21 subunit	protein_coding	NP_080584.1	1914926	.	10.606507	10.783623	11.475653	[illegible]
67678	NM_026309	Lsm3	LSM3 homolog, U6 small nuclear RNA and mRNA degradation associated	protein_coding	NP_080585.1	1914928	.	28.177879	27.590607	30.134672	[illegible]
67680	NM_001355515,NM_023374	Sdhb	succinate dehydrogenase complex, subunit B, iron sulfur (Ip)	protein_coding	NP_001342444.1;NP_075863.2	1914930	.	50.452569	64.055497	52.636996	[illegible]
67681	NM_026310,XM_006523344,XM_017317617	Mrpl18	mitochondrial ribosomal protein L18	protein_coding	NP_080586.1;XP_006523407.1;XP_017173106.1	1914931	.	26.263731	30.007293	30.640767	[illegible]
67683	NM_001281871,NM_026312,XM_006528265,XM_006528266,XM_006528267,XM_006528268,XM_006528269,XM_006528270,XR_003953024,XR_386928	Pbdc1	polysaccharide biosynthesis domain containing 1	protein_coding	NP_001268800.1;NP_080588.1;XP_006528328.1;XP_006528329.1;XP_006528330.1;XP_006528331.1;XP_006528332.1;XP_006528333.1	1914933	.	11.980718	9.937220	9.157913	[illegible]
67684	NM_001361573,NM_001361574,NM_001361575,NM_026313,NR_155068,NR_155069,XR_001780043,XR_001780044,XR_388511	Luc7l3	LUC7-like 3 (S. cerevisiae)	protein_coding	NP_001348502.1;NP_001348503.1;NP_001348504.1;NP_080589.1	1914934	.	41.142941	32.795496	33.744334	[illegible]
67685	NM_001163725,NM_026314,XM_006511381,XM_006511382,XM_017313537	Dnaaf4	dynein axonemal assembly factor 4	protein_coding	NP_001157197.1;NP_080590.3;XP_006511444.1;XP_006511445.1;XP_017169026.1	1914935	.	1.150837	0.947388	1.310298	[illegible]
67687	NM_026315,XM_011248492	1700011L22Rik	RIKEN cDNA 1700011L22 gene	protein_coding	NP_080591.1;XP_011246794.1	1914937	.	0.000000	0.000000	0.000000	[illegible]
67689	NM_026316,XM_006531830	Aldh3b1	aldehyde dehydrogenase 3 family, member B1	protein_coding	NP_080592.2;XP_006531893.1	1914939	.	1.833993	2.898293	1.968117	[illegible]
67693	NM_026318	Hypk	huntingtin interacting protein K	protein_coding	NP_080594.2	1914943	.	56.495762	46.242966	56.249451	[illegible]
67694	NM_001290568,NM_026319,XM_006503324,XM_006503326,XM_006503327,XM_006503328,XM_006503329,XM_011240597,XM_011240598,XM_030253727,XR_001784189	Ift74	intraflagellar transport 74	protein_coding	NP_001277497.1;NP_080595.2;XP_006503387.1;XP_006503389.1;XP_006503390.1;XP_006503391.1;XP_006503392.1;XP_011238899.1;XP_011238900.1;XP_030109587.1	1914944	.	9.776830	6.237306	8.643421	[illegible]
67695	NM_001134692,NM_024460,XM_006504071	Ost4	oligosaccharyltransferase complex subunit 4 (non-catalytic)	protein_coding	NP_001128164.1;NP_077780.3;XP_006504134.1	1914945	.	106.607901	99.717710	122.756842	[illegible]
67698	NM_026321	Fam174a	family with sequence similarity 174, member A	protein_coding	NP_080597.2	1914948	.	5.839644	6.223531	5.959390	[illegible]
67701	NM_026323,XM_006500087	Wfdc2	WAP four-disulfide core domain 2	protein_coding	NP_080599.1;XP_006500150.1	1914951	.	1.889596	1.918353	1.967052	[illegible]
67702	NM_001033135,XM_006496226,XR_373259	Rnf149	ring finger protein 149	protein_coding	NP_001028307.2;XP_006496289.1	2677438	.	10.814831	10.461214	12.828109	[illegible]
67703	NM_001190911,NM_001190912,NM_001190913,NM_001190914,NM_026324,XM_006510557,XM_006510558,XM_006510559,XM_006510560,XM_006510561,XM_006510563,XM_017313538,XM_017313539,XM_030244529	Kirrel3	kirre like nephrin family adhesion molecule 3	protein_coding	NP_001177840.1;NP_001177841.1;NP_001177842.1;NP_001177843.1;NP_080600.1;XP_006510620.1;XP_006510621.1;XP_006510622.1;XP_006510623.1;XP_006510624.1;XP_006510626.1;XP_017169027.1;XP_017169028.1;XP_030100389.1	1914953	.	0.688090	0.100629	0.149356	[illegible]
67704	NM_024461	1810037I17Rik	RIKEN cDNA 1810037I17 gene	protein_coding	NP_077781.1	1914954	.	23.665783	21.149555	21.749289	[illegible]
67705	NR_015608,NR_027875	1810058I24Rik	RIKEN cDNA 1810058I24 gene	lncRNA	.	1914955	.	48.846045	50.612114	47.494912	[illegible]
67706	NM_026325,NR_028418,NR_028419	Tmem179b	transmembrane protein 179B	protein_coding	NP_080601.2	1914956	.	18.193280	11.579354	14.375909	[illegible]
67707	NM_026591,XM_006501940,XM_006501941,XM_006501942	Mrpl24	mitochondrial ribosomal protein L24	protein_coding	NP_080867.2;XP_006502003.1;XP_006502004.1;XP_006502005.1	1914957	.	34.078706	33.620891	29.201102	[illegible]
67708	NM_026327,XM_006516175,XM_006516176,XM_006516177	Pcnx4	pecanex homolog 4	protein_coding	NP_080603.2;XP_006516238.1;XP_006516239.1;XP_006516240.1	1914958	.	3.694974	3.336001	3.432440	[illegible]
67709	NM_026328,XM_017319697	Reg4	regenerating islet-derived family, member 4	protein_coding	NP_080604.2;XP_017175186.1	1914959	.	0.000000	0.000000	0.046606	[illegible]
67710	NM_026329	Polr2g	polymerase (RNA) II (DNA directed) polypeptide G	protein_coding	NP_080605.1	1914960	.	25.638693	25.216482	27.443569	[illegible]
67711	NM_001360659,NM_026330,XM_030242939,XM_030242940,XM_030242941	Nsmce1	NSE1 homolog, SMC5-SMC6 complex component	protein_coding	NP_001347588.2;NP_080606.3;XP_030098799.1;XP_030098800.1;XP_030098801.1	1914961	.	13.398158	13.085136	10.716129	[illegible]
67712	NM_026331,XM_006519461,XR_003950900	Slc25a37	solute carrier family 25, member 37	protein_coding	NP_080607.2;XP_006519524.1	1914962	.	10.673784	11.516903	9.983934	[illegible]
67713	NM_001026211,NM_001286972,NM_001286973,NM_026332	Dnajc19	DnaJ heat shock protein family (Hsp40) member C19	protein_coding	NP_001021382.1;NP_001273901.1;NP_001273902.1;NP_080608.3	1914963	.	16.200466	15.639858	13.617057	[illegible]
67722	NM_026338	Actl11	actin-like 11	protein_coding	NP_080614.1	1914972	.	0.000000	0.000000	0.000000	[illegible]
67723	NR_015524	Cep83os	centrosomal protein 83, opposite strand	lncRNA	.	1914973	.	2.908561	2.249127	2.744599	[illegible]
67724	NM_026340,NM_152894,XM_006520158	Pop1	processing of precursor 1, ribonuclease P/MRP family, (S. cerevisiae)	protein_coding	NP_080616.1;NP_690854.1;XP_006520221.1	1914974	.	2.454963	2.428803	3.113648	[illegible]
67725	NM_001359868,NM_001359869,NM_001359870,NM_026341,NR_153363,NR_153364,XM_006519463,XM_006519468,XM_006519470,XM_006519471,XM_011245159,XM_011245161,XM_011245164,XM_017316168,XM_030247974	Nudt13	nudix (nucleoside diphosphate linked moiety X)-type motif 13	protein_coding	NP_001346797.1;NP_001346798.1;NP_001346799.1;NP_080617.2;XP_006519526.1;XP_006519531.1;XP_006519533.1;XP_006519534.1;XP_011243461.1;XP_011243463.1;XP_011243466.1;XP_017171657.1;XP_030103834.1	1914975	.	4.886367	5.475601	4.915011	[illegible]
67726	NM_001168667,NM_001168668,NM_026342,XM_006534013,XM_006534014,XM_006534015,XM_006534016,XM_011249194,XM_011249195,XM_030246250,XR_001780046,XR_001780047,XR_003949494,XR_879730,XR_879731	Fam114a2	family with sequence similarity 114, member A2	protein_coding	NP_001162138.1;NP_001162139.1;NP_080618.1;XP_006534076.1;XP_006534077.1;XP_006534078.1;XP_006534079.1;XP_011247496.1;XP_011247497.1;XP_030102110.1	1917629	.	18.009356	17.816249	19.789270	[illegible]
67727	NM_026343,XM_006538208,XM_011250090,XM_017320367	Stx17	syntaxin 17	protein_coding	NP_080619.2;XP_006538271.1;XP_011248392.1;XP_017175856.1	1914977	.	8.442400	8.194428	7.272365	[illegible]
67728	NM_026344	Dph2	DPH2 homolog	protein_coding	NP_080620.1	1914978	.	2.637635	2.650624	2.907939	[illegible]
67729	NM_026345	Mansc1	MANSC domain containing 1	protein_coding	NP_080621.1	1914979	.	1.217433	1.058401	1.166335	[illegible]
67731	NM_026346	Fbxo32	F-box protein 32	protein_coding	NP_080622.1	1914981	.	3.004627	4.644561	2.468428	[illegible]
67732	NM_026347,XM_006515175,XM_006515176,XM_006515177,XM_006515178	Iah1	isoamyl acetate-hydrolyzing esterase 1 homolog	protein_coding	NP_080623.2;XP_006515238.1;XP_006515239.1;XP_006515240.1;XP_006515241.1	1914982	.	15.674701	15.662145	13.655036	[illegible]
67733	NM_001362974,NM_001362975,NM_001362976,NM_026348,XM_011240600,XM_017320368,XM_030253728	Itgb3bp	integrin beta 3 binding protein (beta3-endonexin)	protein_coding	NP_001349903.1;NP_001349904.1;NP_001349905.1;NP_080624.1;XP_011238902.1;XP_017175857.1;XP_030109588.1	1914983	.	4.143134	4.293704	4.473213	[illegible]
67735	NR_028384	4930528A17Rik	RIKEN cDNA 4930528A17 gene	lncRNA	.	1914985	.	0.043523	0.007689	0.015085	[illegible]
67736	NM_001294281,NM_026350,XM_006531315,XM_006531316	Ccdc130	coiled-coil domain containing 130	protein_coding	NP_001281210.1;NP_080626.1;XP_006531378.1;XP_006531379.1	1914986	.	4.085872	4.043800	3.495606	[illegible]
67737	NM_026351	Ttc39d	tetratricopeptide repeat domain 39D	protein_coding	NP_080627.2	1914987	.	0.000000	0.000000	0.000000	[illegible]
67738	NM_001356326,NM_026352,XM_006501943	Ppid	peptidylprolyl isomerase D (cyclophilin D)	protein_coding	NP_001343255.1;NP_080628.1;XP_006502006.1	1914988	.	29.939227	25.345572	32.641239	[illegible]

67739	NM_026353	Slc48a1	solute carrier family 48 (heme transporter), member 1	[illegible]
67742	NM_023380	Samsn1	SAM domain, SH3 domain and nuclear localization signals, 1	[illegible]
67745	NR_027879	4930583K01Rik	RIKEN cDNA 4930583K01 gene	[illegible]
67746	NR_073429	4930577N17Rik	RIKEN cDNA 4930577N17 gene	[illegible]
67747	NM_026357,NR_152195,XM_006521298,XM_006521299,XR_003951422	Ribc2	RIB43A domain with coiled-coils 2	[illegible]
67749	NM_026358,NR_028121	Mgarp	mitochondria localized glutamic acid rich protein	[illegible]
67752	NM_001368184,NM_133689,XM_006527302,XM_017318281,XM_017318282,XM_030251057	Ppp1r32	protein phosphatase 1, regulatory subunit 32	[illegible]
67753	NM_001290623,NM_027089,XM_006503333	Eqtn	equatorin, sperm acrosome associated	[illegible]
67755	NM_026360	Ddx47	DEAD (Asp-Glu-Ala-Asp) box polypeptide 47	[illegible]
67758	NM_023383	Aadac	arylacetamide deacetylase	[illegible]
67759	NM_026362,XM_017318283,XM_030251058,XM_030251059,XM_030251060	Plgrkt	plasminogen receptor, C-terminal lysine transmembrane protein	[illegible]
67760	NM_001355633,NM_175121	Slc38a2	solute carrier family 38, member 2	[illegible]
67763	NM_026364,XM_006534017,XM_006534018,XM_011249196	Prpsap1	phosphoribosyl pyrophosphate synthetase-associated protein 1	[illegible]
67765	NR_015548	5830432E09Rik	RIKEN cDNA 5830432E09 gene	[illegible]
67767	NM_001205025,NM_001243739,NM_026365	Jagn1	jagunal homolog 1	[illegible]
67768	NM_001159331,NM_026366	N6amt1	N-6 adenine-specific DNA methyltransferase 1 (putative)	[illegible]
67769	NM_001357645,NM_001357646,NM_001357647,NM_001357648,NM_026367,NR_151741,XM_006497198,XM_006497199,XM_030242477,XM_030242479,XM_030242485,XM_030242487,XR_00394645	Gpatch2	G patch domain containing 2	[illegible]
67770	NM_026368	Caap1	caspase activity and apoptosis inhibitor 1	[illegible]
67771	NM_026369	Arpc5	actin related protein 2/3 complex, subunit 5	[illegible]
67772	NM_201637,XM_011245165,XM_017316170,XM_017316171,XM_017316172	Chd8	chromodomain helicase DNA binding protein 8	[illegible]
67773	NM_001360699,NM_026370,XM_006508138	Kat8	K(lysine) acetyltransferase 8	[illegible]
67774	NM_001170479,NM_026371	Borcs5	BLOC-1 related complex subunit 5	[illegible]
67775	NM_023386,XM_006522491,XM_030249254,XM_030249255,XM_030249256,XM_030249257	Rtp4	receptor transporter protein 4	[illegible]
67776	NM_001145957,NM_172767	Vwa5a	von Willebrand factor A domain containing 5A	[illegible]
67778	NM_001161818,NM_001356625,NM_144519,XM_006535527,XM_006535529,XM_006535530,XM_030252771,XM_030252772	Zfp639	zinc finger protein 639	[illegible]
67781	NM_026374	Ilf2	interleukin enhancer binding factor 2	[illegible]
67784	NM_026376,XR_868877	Plxnd1	plexin D1	[illegible]
67785	NM_001114399,NM_001357201,XM_006503343,XM_006503344,XM_030253729	Zmym4	zinc finger, MYM-type 4	[illegible]
67788	NM_026377,XM_006527308	Sfr1	SWI5 dependent recombination repair 1	[illegible]
67789	NM_026378,XM_006511792,XM_006511793,XM_011242901,XM_017313540,XR_001778994,XR_003947985,XR_379772,XR_871095	Dalrd3	DALR anticodon binding domain containing 3	[illegible]
67790	NM_175122	Rab39b	RAB39B, member RAS oncogene family	[illegible]
67792	NM_001347115,NM_001357525,NM_026380,XM_006529818,XM_006529820,XM_006529821,XM_006529822,XM_006529824,XM_006529825,XM_006529826,XM_006529827,XM_006529828,XM_006529829,XM_030242505,XM_030242507	Rgs8	regulator of G-protein signaling 8	[illegible]
67795	NR_160911	Rnls	renalase, FAD-dependent amine oxidase	[illegible]
67797	NM_026382,XM_006516735,XM_011244332,XM_017315577	Snrnp48	small nuclear ribonucleoprotein 48 (U11/U12)	[illegible]
67800	NM_026384	Dgat2	diacylglycerol O-acyltransferase 2	[illegible]
67801	NM_026385	Pllp	plasma membrane proteolipid	[illegible]
67803	NM_001356458,NM_001356459,NM_172397	Limd2	LIM domain containing 2	[illegible]
67804	NM_001357463,NM_026386,XM_030250577	Snx2	sorting nexin 2	[illegible]
67808	NM_026388,NR_151647,XM_006539129	Tprgl	transformation related protein 63 regulated like	[illegible]
67809	NM_001033136,XR_001783171,XR_003953698,XR_374514,XR_866337	Rmdn3	regulator of microtubule dynamics 3	[illegible]
67811	NM_026389	Poldip2	polymerase (DNA-directed), delta interacting protein 2	[illegible]
67812	NM_001356529,NM_026390,XM_006529831,XM_017322239	Ubxn4	UBX domain protein 4	[illegible]
67815	NM_144520	Sec14l2	SEC14-like lipid binding 2	[illegible]
67819	NM_024207	Derl1	Der1-like domain family, member 1	[illegible]
67821	NM_001290389,NM_133690,XM_006541533	Atp1b4	ATPase, (Na+)/K+ transporting, beta 4 polypeptide	[illegible]
67824	NM_001290761,NM_001290762,NM_001290763,NM_026393	Nmral1	NmrA-like family domain containing 1	[illegible]
67826	NM_001356452,NM_001356454,NM_144521,XM_006534022,XM_006534023,XM_006534024,XM_030246251,XM_030246252,XM_030246253,XM_030246254	Snap47	synaptosomal-associated protein, 47	[illegible]
67828	NM_026394	Lce1f	late cornified envelope 1F	[illegible]
67830	NM_001362981,NM_026395,XM_006539131,XM_006539132,XM_006539133,XM_011250328,XM_030253730	Rer1	retention in endoplasmic reticulum sorting receptor 1	[illegible]
67832	NM_026396	Brix1	BRX1, biogenesis of ribosomes	[illegible]
67834	NM_029573	Idh3a	isocitrate dehydrogenase 3 (NAD+) alpha	[illegible]
67836	NM_026399	Wdr83	WD repeat domain containing 83	[illegible]
67838	NM_001190804,NM_001190805,NM_026400	Dnajb11	DnaJ heat shock protein family (Hsp40) member B11	[illegible]
67839	NM_001199146,NM_001199147,NM_001355574,NM_001355575,NM_001355576,NM_153410,XM_006498255,XR_003953699	Gpsm1	G-protein signalling modulator 1 (AGS3-like, C. elegans)	[illegible]
67840	NM_026401,XM_006519478	Mrpl57	mitochondrial ribosomal protein L57	[illegible]
67841	NM_001356366,NM_026402	Atg3	autophagy related 3	[illegible]
67842	NM_026403	Nop9	NOP9 nucleolar protein	[illegible]
67843	NM_001083317,NM_001360989,NM_026404	Slc35a4	solute carrier family 35, member A4	[illegible]
67844	NM_026405	Rab32	RAB32, member RAS oncogene family	[illegible]
67845	NM_026406,XM_006501947,XM_030252773	Rnf115	ring finger protein 115	[illegible]
67846	NM_001205286,NM_001205287,NM_026407,XM_006522496,XM_006522497,XR_001781854,XR_003951823,XR_384596	Tmem39a	transmembrane protein 39a	[illegible]
67847	NM_001199151,NM_001199153,NM_001199154,NM_026408,XM_006526191,XM_006526192,XM_017317971,XM_030250578	Sncaip	synuclein, alpha interacting protein (synphilin)	[illegible]
67848	NM_001190795,NM_026409,XR_387576	Ddx55	DEAD (Asp-Glu-Ala-Asp) box polypeptide 55	[illegible]
67849	NM_026410	Cdca5	cell division cycle associated 5	[illegible]
67850	NR_040538	1700007F19Rik	RIKEN cDNA 1700007F19 gene	[illegible]
67851	NM_026411,XM_006512833,XM_006512834,XM_011243202,XM_017314069,XM_030245239	1700021F05Rik	RIKEN cDNA 1700021F05 gene	[illegible]
67855	NM_026414	Asprv1	aspartic peptidase, retroviral-like 1	[illegible]
67856	NM_024208,XM_030251997	Echdc3	enoyl Coenzyme A hydratase domain containing 3	[illegible]
67857	NM_024209,XM_006498256,XM_017319227	Ppp6c	protein phosphatase 6, catalytic subunit	[illegible]
67859	NM_026415	Cysrt1	cysteine rich tail 1	[illegible]
67860	NM_001356605,NM_001356606,NM_001356607,NM_001356608,NM_001356609,NM_001356610,NM_026416,XM_030252774	S100a16	S100 calcium binding protein A16	[illegible]
67861	NM_172398,NR_149754	Akr1b10	aldo-keto reductase family 1, member B10 (aldose reductase)	[illegible]
67862	NM_024210	2310033P09Rik	RIKEN cDNA 2310033P09 gene	[illegible]
67863	NM_024211,XM_006534026	Slc25a11	solute carrier family 25 (mitochondrial carrier oxoglutarate carri[illegible] member 11	[illegible]
67864	NM_026417	Yipf4	Yip1 domain family, member 4	[illegible]
67865	NM_026418,XM_006508139,XM_017312270,XM_030242942	Rgs10	regulator of G-protein signalling 10	[illegible]
67866	NM_023395	Wfdc1	WAP four-disulfide core domain 1	[illegible]
67867	NM_027413,NM_175124,NR_028143,NR_028144,XM_006541130,XM_006541133,XR_391360	Lrrc28	leucine rich repeat containing 28	[illegible]
67869	NM_001357470,NM_001357471,NM_001357472,NM_001357473,NM_026420,XM_017317972,XM_017317973,XM_030250579,XM_030250580,XM_030250581	Paip2	polyadenylate-binding protein-interacting protein 2	[illegible]
67870	NM_001163035,NM_026421,NR_027990,XM_006535189,XM_006535190,XM_011249550,XM_030254775,XM_030254776	Enoph1	enolase-phosphatase 1	[illegible]
67871	NM_026422,XM_017319228,XM_030251998	Mrrf	mitochondrial ribosome recycling factor	[illegible]
67872	NM_001162855	Nsmce4a	NSE4 homolog A, SMC5-SMC6 complex component	[illegible]
67873	NM_026423	Mri1	methylthioribose-1-phosphate isomerase 1	[illegible]
67874	NM_023396	Rprm	reprimo, TP53 dependent G2 arrest mediator candidate	[illegible]
67876	NM_001039710,NM_026424	Coq10b	coenzyme Q10B	[illegible]
67877	NM_001141965,NM_026425,XR_003953700	Naa20	N(alpha)-acetyltransferase 20, NatB catalytic subunit	[illegible]
67878	NM_001285452,NM_028975,NM_030108	Tmem33	transmembrane protein 33	[illegible]
67880	NM_001347608,NM_026428	Dcxr	dicarbonyl L-xylulose reductase	[illegible]
67881	NM_023397,NR_028316	Mdp1	magnesium-dependent phosphatase 1	[illegible]
67883	NM_001368287,NM_001368288,NM_001368289,NM_026430,NR_160749,NR_160750,NR_160751	Uxs1	UDP-glucuronate decarboxylase 1	[illegible]
67884	NM_026431,XM_006514008,XM_006514009,XM_030245240	Cfap410	cilia and flagella associated protein 410	[illegible]
67885	NR_015476	Mtln	mitoregulin	[illegible]
67886	NM_001347109,NM_001347110,XM_006529832,XM_006529834,XM_011248066	Camsap2	calmodulin regulated spectrin-associated protein family, membe[illegible]	[illegible]
67887	NM_026432	Saraf	store-operated calcium entry-associated regulatory factor	[illegible]
67888	NM_026433,XM_006534030,XM_006534031	Tmem100	transmembrane protein 100	[illegible]
67889	NM_001159635,NM_001355578,NM_001355579,NM_001355580,NM_026434,NR_027515	Rbm18	RNA binding motif protein 18	[illegible]
67890	NM_026435	Ufm1	ubiquitin-fold modifier 1	[illegible]
67891	NM_024212	Rpl4	ribosomal protein L4	[illegible]
67892	NM_174987	Coa6	cytochrome c oxidase assembly factor 6	[illegible]
67893	NM_026436	Tmem86a	transmembrane protein 86A	[illegible]
67894	NM_001167829,NM_001347464,NM_001360619,NM_001360620,NM_026437	Fam45a	family with sequence similarity 45, member A	[illegible]
67895	NM_026438	Ppa1	pyrophosphatase (inorganic) 1	[illegible]
67896	NM_026439,XM_006522498,XM_006522499,XM_006522500,XM_006522501	Ccdc80	coiled-coil domain containing 80	[illegible]
67897	NM_001170953,NM_001361002,NM_001361003,NM_026440,XM_006526193,XM_006526197,XM_011246991,XR_386009	Rnmt	RNA (guanine-7-) methyltransferase	[illegible]
67898	NM_026441	Pef1	penta-EF hand domain containing 1	[illegible]
67899	NM_026442,XM_011242996,XM_030244530	Cmc1	COX assembly mitochondrial protein 1	[illegible]
67900	NM_026443	Mtfp1	mitochondrial fission process 1	[illegible]
67902	NM_026445	Sumf2	sulfatase modifying factor 2	[illegible]
67903	NM_018771,XM_030243739	Gipc1	GIPC PDZ domain containing family, member 1	[illegible]
67905	NM_026447,NM_198931	Ppm1m	protein phosphatase 1M	[illegible]
67911	NM_001164575,NM_001164576,NM_026450,XM_006516948,XM_011244409,XM_011244410,XM_011244411,XM_017315578	Zfp169	zinc finger protein 169	[illegible]
67912	NM_001083880,NM_001083881,NM_001083882,NM_026451	1600012H06Rik	RIKEN cDNA 1600012H06 gene	[illegible]
67914	NM_026452	Coq9	coenzyme Q9	[illegible]
67916	NM_080555	Plpp3	phospholipid phosphatase 3	[illegible]
67917	NM_175126	Zcchc3	zinc finger, CCHC domain containing 3	[illegible]
67920	NM_026453	Mak16	MAK16 homolog	[illegible]
67921	NM_001355761,NM_001355762,NM_001355763,NM_001355764,NM_026454,NR_149825,XM_006529838,XM_011248067,XM_017322263,XM_030242557	Ube2f	ubiquitin-conjugating enzyme E2F (putative)	[illegible]
67922	NM_026455	Fam32a	family with sequence similarity 32, member A	[illegible]
67923	NM_001310470,NM_026456,XM_006495558,XM_006495559,XM_006495561,XM_006495562,XM_006495563,XM_006495564,XM_011238403,XM_017322272,XM_030242571,XM_030242577,XM_030242579	Eloc	elongin C	[illegible]
67928	NM_026458,XM_006508142,XM_006508143,XM_017312271,XM_017312273,XM_017312274,XR_003946550	Abca14	ATP-binding cassette, sub-family A (ABC1), member 14	[illegible]
67933	NM_001081218,NM_001368720,XM_011243544,XM_011243545,XM_030245241,XM_030245242,XM_030245243	Hcfc2	host cell factor C2	[illegible]
67934	NR_105027	1700124L16Rik	RIKEN cDNA 1700124L16 gene	[illegible]
67935	NM_001003951,XM_006531320,XM_006531321,XM_006531322	Ces5a	carboxylesterase 5A	[illegible]
67936	NM_026464,NR_154585	Wdr55	WD repeat domain 55	[illegible]
67937	NM_001368366,NM_001368367,NM_182991	Tmem59l	transmembrane protein 59-like	[illegible]
67938	NM_023402	Myl12b	myosin, light chain 12B, regulatory	[illegible]
67939	NM_001163454,NM_026465	Prorsd1	prolyl-tRNA synthetase domain containing 1	[illegible]
67941	NM_001311101,NM_001361106,NM_026467,NR_154464,XM_017313541	Rps27l	ribosomal protein S27-like	[illegible]
67942	NM_026468,XM_030248717	Atp5g2	ATP synthase, H+ transporting, mitochondrial F0 complex, subu[illegible] (subunit 9)	[illegible]
67943	NM_023403	Mesd	mesoderm development LRP chaperone	[illegible]
67945	NM_018860	Rpl41	ribosomal protein L41	[illegible]
67946	NM_001357231,NM_026470,XM_006503346,XM_006503347,XM_006503348,XM_030253731	Spata6	spermatogenesis associated 6	[illegible]
67948	NM_175127	Fbxo28	F-box protein 28	[illegible]
67949	NM_026472	Nifk	nucleolar protein interacting with the FHA domain of MKI67	[illegible]
67951	NM_026473	Tubb6	tubulin, beta 6 class V	[illegible]
67952	NM_024214	Tomm20	translocase of outer mitochondrial membrane 20	[illegible]
67955	NM_026474	Sugt1	SGT1, suppressor of G2 allele of SKP1 (S. cerevisiae)	[illegible]
67956	NM_001310723,NM_001310725,NM_001310727,NM_030241,XM_006530450,XM_030254777	Kmt5a	lysine methyltransferase 5A	[illegible]
67958	NM_001114977,NM_001357974,NM_001357975,NM_001357976,NM_001357977,NM_026476,XM_030244531,XM_030244532	U2surp	U2 snRNP-associated SURP domain containing	[illegible]
67959	NM_001164600,NM_001356629,NM_028364,NM_133691,XM_006521302,XM_006521303,XM_006521305,XM_030248718	Puf60	poly-U binding splicing factor 60	[illegible]
67963	NM_023409	Npc2	NPC intracellular cholesterol transporter 2	[illegible]
67966	NM_026479	Zcchc10	zinc finger, CCHC domain containing 10	[illegible]
67967	NM_001368380,NM_133692,XM_006508150,XM_030242943,XM_030242944,XM_030242945,XM_030242946	Pold3	polymerase (DNA-directed), delta 3, accessory subunit	[illegible]
67968	NM_026480	Ooep	oocyte expressed protein	[illegible]
67971	NM_026481,XM_006531324	Tppp3	tubulin polymerization-promoting protein family member 3	[illegible]
67972	NM_001359506,NM_001359507,NM_001359508,NM_001359509,NM_026482,XM_011243548,XM_011243549,XM_030245244	Atp2b1	ATPase, Ca++ transporting, plasma membrane 1	[illegible]
67973	NM_026483	Mphosph10	M-phase phosphoprotein 10 (U3 small nucleolar ribonucleopro[illegible]	[illegible]
67974	NM_026484,XM_030250582,XM_030250583,XM_030250584	Ccny	cyclin Y	[illegible]
67976	NM_001355617,NM_026485,XM_006521307,XM_006521308	Trabd	TraB domain containing	[illegible]
67978	NM_026486	Tctn2	tectonic family member 2	[illegible]
67979	NM_026487,XM_006527313,XM_030251061	Atad1	ATPase family, AAA domain containing 1	[illegible]
67980	NM_001038015,NM_001347363,NM_001359239,XM_011240765,XM_011240766,XR_868296	Gnpda2	glucosamine-6-phosphate deaminase 2	[illegible]
67983	NM_001040136,NM_001040137,XM_006508151,XM_011241886	Pdzd9	PDZ domain containing 9	[illegible]
67988	NM_198295	Tmx3	thioredoxin-related transmembrane protein 3	[illegible]
67991	NM_001037098,NM_026495,XM_006498259,XM_006498260	Nacc2	nucleus accumbens associated 2, BEN and BTB (POZ) domain [illegible]	[illegible]
67993	NM_001368393,NM_026497,XR_876526	Nudt12	nudix (nucleoside diphosphate linked moiety X)-type motif 12	[illegible]
67994	NM_001360702,NM_026498,NR_153793	Mrps11	mitochondrial ribosomal protein S11	[illegible]
67996	NM_026499	Srsf6	serine/arginine-rich splicing factor 6	[illegible]
67997	NM_026500,XM_006529842,XM_006529843,XM_006529844	Ddx59	DEAD (Asp-Glu-Ala-Asp) box polypeptide 59	[illegible]
67998	NM_001361583,NM_026501,NM_028933,NR_155070,NR_155071	Retreg3	reticulophagy regulator family member 3	[illegible]
68001	NM_026502	Cfap298	cilia and flagella associate protien 298	[illegible]
68002	NM_026503	Sdhaf4	succinate dehydrogenase complex assembly factor 4	[illegible]
68010	NM_026505,XM_030250585	Bambi	BMP and activin membrane-bound inhibitor	[illegible]
68011	NM_026506	Snrpg	small nuclear ribonucleoprotein polypeptide G	[illegible]
68014	NM_026507,XR_379449	Zwilch	zwilch kinetochore protein	[illegible]
68015	NM_026508,NR_152409,XR_001781856	Trap1	TNF receptor-associated protein 1	[illegible]
68016	NM_026509	Cavin4	caveolae associated 4	[illegible]
68017	NM_026510	Mrm2	mitochondrial rRNA methyltransferase 2	[illegible]
68018	NM_001164222,NM_023420,XM_030247385	Col4a3bp	collagen, type IV, alpha 3 (Goodpasture antigen) binding protein	[illegible]
68020	NM_001163388,NM_026511,XM_006516178,XM_006516179	Apopt1	apoptogenic, mitochondrial 1	[illegible]
68021	NM_026512,XM_006516736,XM_006516737,XM_030247386	Bphl	biphenyl hydrolase-like (serine hydrolase, breast epithelial muci[illegible] associated antigen)	[illegible]
68023	NM_026513	Pdf	peptide deformylase (mitochondrial)	[illegible]
68024	NM_001290380,NM_023422	H2bc4	histone cluster 1, H2bc	[illegible]
68026	NM_026515	Pclaf	PCNA clamp associated factor	[illegible]
68027	NM_026516	Tmem178	transmembrane protein 178	[illegible]
68028	NM_001347226,NM_026517,NR_154888,NR_154889	Rpl22l1	ribosomal protein L22 like 1	[illegible]
68031	NM_001110196,NM_001110197,NM_001110198,NM_001284279,NM_026518	Rnf146	ring finger protein 146	[illegible]
68032	NM_026519,XM_030251999,XM_030252000	Emc4	ER membrane protein complex subunit 4	[illegible]
68033	NM_197980	Cox19	cytochrome c oxidase assembly protein 19	[illegible]
68034	NM_026520	Fam122a	family with sequence similarity 122, member A	[illegible]
68035	NM_133693	Rbm42	RNA binding motif protein 42	[illegible]
68036	NM_026521,XM_030248719	Zfp706	zinc finger protein 706	[illegible]
68038	NM_001142681,NM_001369350,NM_001369351,NM_001369352,NM_026522,XM_006536231,XM_011249850	Chid1	chitinase domain containing 1	[illegible]
68039	NM_001291280,NM_026523	Nmb	neuromedin B	[illegible]
68040	NM_024215	Zfp593	zinc finger protein 593	[illegible]
68041	NM_001166635,NM_026524	Mid1ip1	Mid1 interacting protein 1 (gastrulation specific G12-like (zebra[illegible]	[illegible]
68043	NM_026526,XM_006519483,XM_030247983	Eef1akmt1	EEF1A alpha lysine methyltransferase 1	[illegible]
68044	NM_001290667,NM_026527	Chac2	ChaC, cation transport regulator 2	[illegible]
68045	NM_026528	Rtraf	RNA transcription, translation and transport factor	[illegible]
68046	NM_026529	2700062C07Rik	RIKEN cDNA 2700062C07 gene	[illegible]
68047	NM_026530	Mpnd	MPN domain containing	[illegible]
68048	NM_001162939,NM_001360709,NM_026531,NR_153795	Aen	apoptosis enhancing nuclease	[illegible]
68050	NM_023423	Akirin1	akirin 1	[illegible]
68051	NM_001357229,NM_001357230,NM_026532	Nutf2	nuclear transport factor 2	[illegible]
68052	NM_026533	Rps13	ribosomal protein S13	[illegible]
68053	NM_026534	Ubxn2b	UBX domain protein 2B	[illegible]
68054	NM_026535	Serpina12	serine (or cysteine) peptidase inhibitor, clade A (alpha-1 antipro[illegible] antitrypsin), member 12	[illegible]

68055	NM_001361690,NM_001361691,NM_026536,XM_006516180,XM_006516181,XM_011244162,XM_011244163,XM_011244164,XM_030246887,XM_030246888,XM_030246889	Dmac2l	distal membrane arm assembly complex 2 like	protein_coding	NP_001348619.1;NP_001348620.1;NP_080812.1;XP_006516243.1;XP_006516244.1;XP_011242464.1;XP_011242465.1;XP_011242466.1;XP_030102747.1;XP_030102748.1;XP_030102749.1	1915305	.	2.924621	[illegible]
68058	NM_026539,XM_017319699,XM_017319700,XM_017319701	Chd1l	chromodomain helicase DNA binding protein 1-like	protein_coding	NP_080815.1;XP_017175188.1;XP_017175189.1;XP_017175190.1	1915308	.	4.854123	[illegible]
68059	NM_080556	Tm9sf2	transmembrane 9 superfamily member 2	protein_coding	NP_542123.3	1915309	.	55.112453	[illegible]
68066	NM_026542,XM_006534034,XM_006534035,XM_006534036,XM_030246255,XM_030246256	Slc25a39	solute carrier family 25, member 39	protein_coding	NP_080818.1;XP_006534097.1;XP_006534098.1;XP_006534099.1;XP_030102115.1;XP_030102116.1	1196386	.	31.378158	[illegible]
68067	NM_026543,XM_006534037	Mrnip	MRN complex interacting protein	protein_coding	NP_080819.2;XP_006534100.1	1915317	.	2.609041	[illegible]
68070	NM_001081064,XM_006520160,XM_006520161,XM_006520162,XM_006520163,XM_006520164,XM_006520165,XM_006520166,XM_006520167,XM_006520168,XM_006520169,XM_006520172,XM_017316734,XM_017316735,XM_030248720,XM_030248721,XM_030248722	Pdzd2	PDZ domain containing 2	protein_coding	NP_001074533.1;XP_006520223.1;XP_006520224.1;XP_006520225.1;XP_006520226.1;XP_006520227.1;XP_006520228.1;XP_006520229.1;XP_006520230.1;XP_006520231.1;XP_006520232.1;XP_006520235.1;XP_017172223.1;XP_017172224.1;XP_030104580.1;XP_030104581.1;XP_030104582.1	1922394	.	10.343700	[illegible]
68073	NM_001356313,NM_026546,XM_030248723	Atpsckmt	ATP synthase C subunit lysine N-methyltransferase	protein_coding	NP_001343242.1;NP_080822.1;XP_030104583.1	1915323	.	5.181306	[illegible]
68074	NR_027886	A930013F10Rik	RIKEN cDNA A930013F10 gene	lncRNA	.	1915324	.	2.435536	[illegible]
68075	NM_026547	Lurap1	leucine rich adaptor protein 1	protein_coding	NP_080823.1	1915325	.	0.446444	[illegible]
68077	NM_133831	Nop53	NOP53 ribosome biogenesis factor	protein_coding	NP_598592.2	2154441	.	63.807838	[illegible]
68079	NM_001360710,NM_026549,XR_003946551	Pdcd2l	programmed cell death 2-like	protein_coding	NP_001347639.1;NP_080825.1	1915329	.	5.255332	[illegible]
68080	NM_024216	Gpn3	GPN-loop GTPase 3	protein_coding	NP_077178.1	1289326	.	14.594265	[illegible]
68082	NM_024438	Dusp19	dual specificity phosphatase 19	protein_coding	NP_077758.1	1915332	.	3.201201	[illegible]
68083	NM_026550	Pak1ip1	PAK1 interacting protein 1	protein_coding	NP_080826.2	1915333	.	17.335243	[illegible]
68087	NM_026551,XM_006534038	Dcakd	dephospho-CoA kinase domain containing	protein_coding	NP_080827.2;XP_006534101.1	1915337	.	23.393386	[illegible]
68089	NM_001170485,NM_001170486,NM_026552	Arpc4	actin related protein 2/3 complex, subunit 4	protein_coding	NP_001163956.1;NP_001163957.1;NP_080828.1	1915339	.	34.207819	[illegible]
68090	NM_026553	Yif1a	Yip1 interacting factor homolog A (S. cerevisiae)	protein_coding	NP_080829.1	1915340	.	27.900764	[illegible]
68092	NM_026554	Ncbp2	nuclear cap binding protein subunit 2	protein_coding	NP_080830.1	1915342	.	21.869158	[illegible]
68094	NM_001114096,NM_001114097,NM_198160,XM_006514020,XM_006514021,XM_006514022,XM_006514023,XM_006514024,XM_030245245	Smarcc2	SWI/SNF related, matrix associated, actin dependent regulator of chromatin, subfamily c, member 2	protein_coding	NP_001107568.1;NP_001107569.1;NP_937803.1;XP_006514083.1;XP_006514084.1;XP_006514085.1;XP_006514086.1;XP_006514087.1;XP_030101105.1	1915344	.	38.396702	[illegible]
68095	NM_001159887,NM_001159888,NM_001159889,NM_023429,XM_006504075,XM_006504076,XM_017321082,XM_017321083,XM_030254778,XM_030254779,XM_030254780,XR_001784740,XR_003955702,XR_376823	Ociad1	OCIA domain containing 1	protein_coding	NP_001153359.1;NP_001153360.1;NP_001153361.1;NP_075918.1;XP_006504138.1;XP_006504139.1;XP_017176571.1;XP_017176572.1;XP_030110638.1;XP_030110639.1;XP_030110640.1	1915345	.	68.247966	[illegible]
68097	NM_001168471,NM_001168472,NM_026556,XM_030246257	Dynll2	dynein light chain LC8-type 2	protein_coding	NP_001161943.1;NP_001161944.1;NP_080832.1;XP_030102117.1	1915347	.	17.094426	[illegible]
68098	NM_001271797,NM_026557,NR_073442,XM_006535191	Rchy1	ring finger and CHY zinc finger domain containing 1	protein_coding	NP_001258726.1;NP_080833.1;XP_006535254.1	1915348	.	16.080337	[illegible]
68099	NM_001310743,NM_026558,XM_006538209,XM_006538210,XM_006538212,XM_011250091,XM_030253732,XR_001784190,XR_003954999,XR_003955000,XR_881072,XR_881073	Fam92a	family with sequence similarity 92, member A	protein_coding	NP_001297672.1;NP_080834.3;XP_006538272.1;XP_006538273.1;XP_006538275.1;XP_011248393.1;XP_030109592.1	1915349	.	24.737205	[illegible]
68106	NM_001102650,NM_001362966,NM_001362967,NM_001362968,NM_026561,XM_006534046,XM_011249201,XM_017314723,XM_017314724,XM_017314725,XM_017314726,XM_017314727,XM_017314728,XM_030246258,XM_030246259,XM_030246260,XM_030246261	Nt5c3b	5'-nucleotidase, cytosolic IIIB	protein_coding	NP_001096120.1;NP_001349895.1;NP_001349896.1;NP_001349897.1;NP_080837.3;XP_006534109.1;XP_011247503.1;XP_017170212.1;XP_017170213.1;XP_017170214.1;XP_017170215.1;XP_017170216.1;XP_017170217.1;XP_030102118.1;XP_030102119.1;XP_030102120.1;XP_030102121.1	1915356	.	27.974788	[illegible]
68107	NM_026562	Cntd1	cyclin N-terminal domain containing 1	protein_coding	NP_080838.1	1923965	.	0.388693	[illegible]
68108	NR_015463,NR_027887	Snhg17	small nucleolar RNA host gene 17	lncRNA	.	1915358	.	9.446431	[illegible]
68112	NM_001085407,NM_001085408,NM_026563,XM_006498261,XM_006498262,XM_030252001,XM_030252002,XM_030252003,XR_003953701,XR_003953702,XR_003953703,XR_003953704,XR_003953705,XR_003953706,XR_374113,XR_374114	Entr1	endosome associated trafficking regulator 1	protein_coding	NP_001078876.1;NP_001078877.1;NP_080839.2;XP_006498324.1;XP_006498325.1;XP_030107861.1;XP_030107862.1;XP_030107863.1	1915362	.	25.228555	[illegible]
68114	NM_023431,XM_006514025,XM_006514026,XM_030245246	Pwwp3a	PWWP domain containing 3A, DNA repair factor	protein_coding	NP_075920.4;XP_006514088.1;XP_006514089.1;XP_030101106.1	1915364	.	15.255326	[illegible]
68115	NM_001081181	Maip1	matrix AAA peptidase interacting protein 1	protein_coding	NP_001074650.1	1915365	.	3.549265	[illegible]
68117	NM_001313733,NM_001313734,NM_026565	Apool	apolipoprotein O-like	protein_coding	NP_001300662.1;NP_001300663.1;NP_080841.1	1915367	.	8.051999	[illegible]
68118	NM_026566,XM_006521310	Atg101	autophagy related 101	protein_coding	NP_080842.1;XP_006521373.1	1915368	.	7.647658	[illegible]
68119	NM_024217,XM_006531326,XM_006531327,XM_006531328,XM_006531329,XM_030243740,XM_030243741,XM_030243742,XM_030243743,XM_030243744,XM_030243745,XR_387773	Cmtm3	CKLF-like MARVEL transmembrane domain containing 3	protein_coding	NP_077179.1;XP_006531389.1;XP_006531390.1;XP_006531391.1;XP_006531392.1;XP_030099600.1;XP_030099601.1;XP_030099602.1;XP_030099603.1;XP_030099604.1;XP_030099605.1	2447162	.	52.820364	[illegible]
68121	NM_023873,XM_006511391,XM_006511393,XM_017313546,XM_030244533,XM_030244534,XM_030244535,XM_030244536,XM_030244537,XM_030244538,XM_030244539,XM_030244540,XM_030244541,XM_030244542,XR_001779002,XR_003947988,XR_003947989,XR_003947990,XR_003947992,XR_003947993	Cep70	centrosomal protein 70	protein_coding	NP_076362.2;XP_006511454.1;XP_006511456.1;XP_017169035.1;XP_030100393.1;XP_030100394.1;XP_030100395.1;XP_030100396.1;XP_030100397.1;XP_030100398.1;XP_030100399.1;XP_030100400.1;XP_030100401.1;XP_030100402.1	1915371	.	2.324937	[illegible]
68126	NM_029629,XM_006500090,XM_006500091	Fahd2a	fumarylacetoacetate hydrolase domain containing 2A	protein_coding	NP_083905.1;XP_006500153.1;XP_006500154.1	1915376	.	2.828961	[illegible]
68127	NR_160859,NR_160860,NR_160861,NR_160862,NR_160863	B230217C12Rik	RIKEN cDNA B230217C12 gene	lncRNA	.	1915377	.	1.841794	[illegible]
68128	NR_015601	Fam120aos	family with sequence similarity 120A, opposite strand	lncRNA	.	1915378	.	8.976531	[illegible]
68133	NM_026572,NR_157137	Gcsh	glycine cleavage system protein H (aminomethyl carrier)	protein_coding	NP_080848.1	1915383	.	21.278778	[illegible]
68134	NM_026573,XM_006541394,XM_030251493,XR_001782759	Upf3b	UPF3 regulator of nonsense transcripts homolog B (yeast)	protein_coding	NP_080849.1;XP_006541457.1;XP_030107353.1	1915384	.	11.331674	[illegible]
68135	NM_080635	Eif3h	eukaryotic translation initiation factor 3, subunit H	protein_coding	NP_542366.1	1915385	.	135.000565	[illegible]
68137	NM_133950	Kdelr1	KDEL (Lys-Asp-Glu-Leu) endoplasmic reticulum protein retention receptor 1	protein_coding	NP_598711.1	1915387	.	109.281273	[illegible]
68140	NM_001081145	Tigd2	tigger transposable element derived 2	protein_coding	NP_001074614.1	1915390	.	3.426185	[illegible]
68142	NM_026574,XM_030252004	Ino80	INO80 complex subunit	protein_coding	NP_080850.2;XP_030107864.1	1915392	.	6.819532	[illegible]
68144	NR_027890	5031426D15Rik	RIKEN cDNA 5031426D15 gene	lncRNA	.	1915394	.	0.021698	[illegible]
68145	NM_001362970,NM_026576	Etaa1	Ewing tumor-associated antigen 1	protein_coding	NP_001349899.1;NP_080852.2	1915395	.	2.780521	[illegible]
68146	NM_026577,XM_006522503,XM_006522504,XM_006522505	Arl13b	ADP-ribosylation factor-like 13B	protein_coding	NP_080853.3;XP_006522566.1;XP_006522567.1;XP_006522568.1	1915396	.	6.544410	[illegible]
68147	NM_026578,XM_011240208	Gar1	GAR1 ribonucleoprotein	protein_coding	NP_080854.1;XP_011238510.1	1930948	.	10.205670	[illegible]
68149	NM_001177841,NM_026580,NR_033587,XM_006516185	Otub2	OTU domain, ubiquitin aldehyde binding 2	protein_coding	NP_001171312.1;NP_080856.1;XP_006516248.3	1915399	.	3.139959	[illegible]
68151	NM_001356349,NM_001356350,NM_026582	Wls	wntless WNT ligand secretion mediator	protein_coding	NP_001343278.1;NP_001343279.1;NP_080858.3	1915401	.	39.990934	[illegible]
68152	NM_001042501	Fam133b	family with sequence similarity 133, member B	protein_coding	NP_001035966.1	1915402	.	9.960701	[illegible]
68153	NM_001167921,NM_001167922,NM_026584,XM_006509185,XM_006509186,XM_006509187,XM_006509188,XM_030243746	Gtf2e2	general transcription factor II E, polypeptide 2 (beta subunit)	protein_coding	NP_001161393.1;NP_001161394.1;NP_080860.3;XP_006509248.1;XP_006509249.1;XP_006509250.1;XP_006509251.1;XP_030099606.1	1915403	.	24.091147	[illegible]
68161	NR_015487,NR_027894,NR_027895	A930005H10Rik	RIKEN cDNA A930005H10 gene	lncRNA	.	1915411	.	8.716180	[illegible]
68162	NR_015488,NR_027896	A930003A15Rik	RIKEN cDNA A930003A15 gene	lncRNA	.	1915412	.	0.209950	[illegible]
68165	NM_001039824,NR_157201,NR_157202,NR_157203,NR_157204	Fdx2	ferredoxin 2	protein_coding	NP_001034913.1	1915415	.	11.952077	[illegible]
68166	NM_176832,NM_194355,XM_006526203,XM_006526205,XM_006526206,XM_006526207,XM_006526209,XM_030250586,XM_030250587,XM_030250588,XM_030250589,XM_030250590,XM_030250591	Spire1	spire type actin nucleation factor 1	protein_coding	NP_789802.1;NP_919336.2;XP_006526266.1;XP_006526268.1;XP_006526269.1;XP_006526270.1;XP_006526272.1;XP_030106446.1;XP_030106447.1;XP_030106448.1;XP_030106449.1;XP_030106450.1;XP_030106451.1	1915416	.	8.396261	[illegible]
68169	NM_172399,XM_006506528	Ndnf	neuron-derived neurotrophic factor	protein_coding	NP_765987.2;XP_006506591.1	1915419	.	1.831614	[illegible]
68170	NM_001362687,NM_001362688,NM_026592	B230118H07Rik	RIKEN cDNA B230118H07 gene	protein_coding	NP_001349616.1;NP_001349617.1;NP_080868.1	1915420	.	4.423810	[illegible]
68172	NM_026594,XM_006522506	Rpl39l	ribosomal protein L39-like	protein_coding	NP_080870.1;XP_006522569.1	1915422	.	10.307368	[illegible]
68173	NR_028021	Ppifos	peptidylprolyl isomerase F, opposite strand	lncRNA	.	1915423	.	0.000000	[illegible]
68176	NM_026597	Inka1	inka box actin regulator 1	protein_coding	NP_080873.2	1915426	.	8.748766	[illegible]
68177	NM_026598	Ebpl	emopamil binding protein-like	protein_coding	NP_080874.2	1915427	.	4.824564	[illegible]
68178	NM_001304362,NM_026599,XM_006511395	Cgnl1	cingulin-like 1	protein_coding	NP_001291291.1;NP_080875.3;XP_006511458.1	1915428	.	16.120813	[illegible]
68180	NR_003946	Hyi	hydroxypyruvate isomerase (putative)	transcribed_pseudogene	.	1915430	.	16.915535	[illegible]
68183	NM_001356330,NM_001356331,NM_026602	Bcas2	breast carcinoma amplified sequence 2	protein_coding	NP_001343259.1;NP_001343260.1;NP_080878.2	1915433	.	24.290858	[illegible]
68184	NM_026603,XM_006530452,XM_011248238	Denr	density-regulated protein	protein_coding	NP_080879.1;XP_006530515.1;XP_011246540.1	1915434	.	23.749725	[illegible]
68185	NM_183270,XM_006508152	Coa4	cytochrome c oxidase assembly factor 4	protein_coding	NP_899093.1;XP_006508215.1	1915435	.	2.995191	[illegible]
68187	NM_026604,XM_006496229,XM_006496230,XM_006496231,XM_006496232,XM_006496233,XM_006496234,XM_006496235,XM_011238569,XM_017322275,XM_030242593,XM_030242594,XM_030242595,XM_030242596,XM_030242597,XM_030242598,XM_030242599,XM_030242600,XM_030242601	Fam135a	family with sequence similarity 135, member A	protein_coding	NP_080880.4;XP_006496292.1;XP_006496293.1;XP_006496294.1;XP_006496295.1;XP_006496296.1;XP_006496297.1;XP_006496298.1;XP_011236871.1;XP_017177764.1;XP_030098453.1;XP_030098454.1;XP_030098455.1;XP_030098456.1;XP_030098457.1;XP_030098458.1;XP_030098459.1;XP_030098460.1;XP_030098461.1	1915437	.	16.012361	[illegible]
68188	NM_001360713,NM_026605,XM_006540332	Sympk	symplekin	protein_coding	NP_001347642.1;NP_080881.2;XP_006540395.1	1915438	.	18.874940	[illegible]
68190	NR_028300	Dubr	Dppa2 upstream binding RNA	lncRNA	.	1915440	.	3.432995	[illegible]
68192	NM_026609	Leprotl1	leptin receptor overlapping transcript-like 1	protein_coding	NP_080885.1	1915442	.	11.530265	[illegible]
68193	NM_024218	Rpl24	ribosomal protein L24	protein_coding	NP_077180.1	1915443	.	555.726746	[illegible]
68194	NM_026610	Ndufb4	NADH:ubiquinone oxidoreductase subunit B4	protein_coding	NP_080886.1	1915444	.	48.238220	[illegible]
68195	NM_026611	Rnaset2b	ribonuclease T2B	protein_coding	NP_080887.1	3702087	.	46.996819	[illegible]
68196	NM_024219	Hsbp1	heat shock factor binding protein 1	protein_coding	NP_077181.1	1915446	.	52.307335	[illegible]
68197	NM_024220	Ndufc2	NADH:ubiquinone oxidoreductase subunit C2	protein_coding	NP_077182.1	1344370	.	40.176785	[illegible]
68198	NM_001358797,NM_026612,NR_152450,NR_152451	Ndufb2	NADH:ubiquinone oxidoreductase subunit B2	protein_coding	NP_001345726.1;NP_080888.1	1915448	.	16.428846	[illegible]
68201	NM_001355581,NM_001355582,NM_026613,NR_149736,XM_011239754,XM_011239755,XR_001783172,XR_001783173,XR_003953707,XR_003953708	Ccdc34	coiled-coil domain containing 34	protein_coding	NP_001342510.1;NP_001342511.1;NP_080889.3;XP_011238056.1;XP_011238057.1	1915451	.	9.772019	[illegible]
68202	NM_001311068,NM_026614,NR_154219	Ndufa5	NADH:ubiquinone oxidoreductase subunit A5	protein_coding	NP_001297997.1;NP_080890.1	1915452	.	33.745047	[illegible]
68203	NM_001024474,XM_006516950	Diras2	DIRAS family, GTP-binding RAS-like 2	protein_coding	NP_001019645.1;XP_006517013.1	1915453	.	0.988124	[illegible]
68204	NR_027901	2900060B14Rik	RIKEN cDNA 2900060B14 gene	lncRNA	.	1915454	.	0.000000	[illegible]
68205	NM_026615	Urm1	ubiquitin related modifier 1	protein_coding	NP_080891.1	1915455	.	10.531756	[illegible]
68209	NM_026616	Rnaseh2c	ribonuclease H2, subunit C	protein_coding	NP_080892.1	1915459	.	20.362406	[illegible]
68212	NM_026617	Tmbim4	transmembrane BAX inhibitor motif containing 4	protein_coding	NP_080893.1	1915462	.	30.935158	[illegible]
68214	NM_026619,NM_030051,XM_030251062,XM_030251063,XM_030251064	Gsto2	glutathione S-transferase omega 2	protein_coding	NP_080895.2;NP_084327.1;XP_030106922.1;XP_030106923.1;XP_030106924.1	1915464	.	0.443284	[illegible]
68215	NM_026620,XM_006500096	Fam98b	family with sequence similarity 98, member B	protein_coding	NP_080896.2;XP_006500159.1	1915465	.	13.498265	[illegible]
68219	NM_026623	Nudt21	nudix (nucleoside diphosphate linked moiety X)-type motif 21	protein_coding	NP_080899.1	1915469	.	27.081070	[illegible]
68221	NM_183271	Wfdc15a	WAP four-disulfide core domain 15A	protein_coding	NP_899094.1	1915471	.	0.000000	[illegible]
68222	NM_026624,XM_006498265,XM_006498266,XM_006498267,XM_006498268,XM_011239161,XM_011239162,XM_011239164,XM_011239165,XM_030252005,XM_030252006,XM_030252007,XM_030252008,XM_030252009,XM_030252010,XM_030252011,XM_030252012,XM_030252013,XM_030252014,XM_030252015	Fam166a	family with sequence similarity 166, member A	protein_coding	NP_080900.1;XP_006498328.1;XP_006498329.1;XP_006498330.1;XP_006498331.1;XP_011237463.1;XP_011237464.1;XP_011237466.1;XP_011237467.1;XP_030107865.1;XP_030107866.1;XP_030107867.1;XP_030107868.1;XP_030107869.1;XP_030107870.1;XP_030107871.1;XP_030107872.1;XP_030107873.1;XP_030107874.1;XP_030107	3605773	.	0.076812	[illegible]
68224	NR_160405	1700067P10Rik	RIKEN cDNA 1700067P10 gene	lncRNA	.	1915474	.	0.000000	[illegible]
68226	NM_026626	Efcab2	EF-hand calcium binding domain 2	protein_coding	NP_080902.1	1915476	.	0.876257	[illegible]
68229	NM_001033139,NM_001167831,XM_011247331,XM_011247332,XM_030251065,XR_003952811	Spindoc	spindlin interactor and repressor of chromatin binding	protein_coding	NP_001028311.2;NP_001161303.1;XP_011245633.1;XP_011245634.1;XP_030106925.1	2147611	.	4.604219	[illegible]
68230	NR_045302,NR_045303	1700102H20Rik	RIKEN cDNA 1700102H20 gene	lncRNA	.	1915480	.	0.274536	[illegible]
68231	NM_026627	H2al2a	H2A histone family member L2A	protein_coding	NP_080903.1	1915481	.	0.000000	[illegible]
68232	NR_027915	1700120K04Rik	RIKEN cDNA 1700120K04 gene	lncRNA	.	1915482	.	0.087252	[illegible]
68235	NM_001289740,NM_001289741,XM_011241447	Mturn	maturin, neural progenitor differentiation regulator homolog (Xenopus)	protein_coding	NP_001276669.1;NP_001276670.1;XP_011239749.1	1915485	.	8.709208	[illegible]
68239	NM_212483	Krt42	keratin 42	protein_coding	NP_997648.2	1915489	.	0.000000	[illegible]
68240	NM_026632	Rpa3	replication protein A3	protein_coding	NP_080908.1	1915490	.	15.720268	[illegible]
68241	NM_026633,XM_006524837,XM_006524838,XM_006524839	Mcrip2	MAPK regulated corepressor interacting protein 2	protein_coding	NP_080909.1;XP_006524900.1;XP_006524901.1;XP_006524902.1	1915491	.	2.546928	[illegible]
68243	NM_026634	A930018P22Rik	RIKEN cDNA A930018P22 gene	protein_coding	NP_080910.1	1915493	.	0.000000	[illegible]
68250	NM_001359210,NM_026635,XM_011242805	Ciao2a	cytosolic iron-sulfur assembly component 2A	protein_coding	NP_001346139.1;NP_080911.1;XP_011241107.1	1915500	.	25.493870	[illegible]
68251	NM_026636,XM_011242323,XM_017312947,XM_017312948	Babam1	BRISC and BRCA1 A complex member 1	protein_coding	NP_080912.2;XP_011240625.1;XP_017168436.1;XP_017168437.1	1915501	.	22.765404	[illegible]
68255	NM_023440,XM_011250686,XM_011250687	Tmem86b	transmembrane protein 86B	protein_coding	NP_075929.1;XP_011248988.1;XP_011248989.1	1915505	.	2.130061	[illegible]
68259	NM_026641,XM_006501966,XM_006501967,XM_017319702,XR_001783737	Ift80	intraflagellar transport 80	protein_coding	NP_080917.1;XP_006502029.1;XP_006502030.1;XP_017175191.1	1915509	.	6.426206	[illegible]
68260	NM_026642	Trmt12	tRNA methyltranferase 12	protein_coding	NP_080918.2	1915510	.	1.159096	[illegible]
68262	NM_026644,XM_006523345,XM_006523347,XM_006523348	Agpat4	1-acylglycerol-3-phosphate O-acyltransferase 4 (lysophosphatidic acid acyltransferase, delta)	protein_coding	NP_080920.2;XP_006523408.3;XP_006523410.1;XP_006523411.1	1915512	.	19.645365	[illegible]
68263	NM_024221,XM_030247984	Pdhb	pyruvate dehydrogenase (lipoamide) beta	protein_coding	NP_077183.1;XP_030103844.1	1915513	.	43.620354	[illegible]
68267	NM_001177576,NM_001360723,NM_001360724,NM_026646,XM_030242948,XM_030242949,XM_030242950	Slc25a22	solute carrier family 25 (mitochondrial carrier, glutamate), member 22	protein_coding	NP_001171047.1;NP_001347652.1;NP_001347653.1;NP_080922.1;XP_030098808.1;XP_030098809.1;XP_030098810.1	1915517	.	8.329225	[illegible]
68268	NM_026647,NR_149737,XM_006538216,XM_011250093,XM_011250094,XM_011250097,XM_017320372,XM_017320373	Zdhhc21	zinc finger, DHHC domain containing 21	protein_coding	NP_080923.2;XP_006538279.1;XP_011248395.1;XP_011248396.1;XP_011248399.1;XP_017175861.1;XP_017175862.1	1915518	.	4.558661	[illegible]
68270	NM_026648	Dnaaf1	dynein, axonemal assembly factor 1	protein_coding	NP_080924.1	1915520	.	0.000000	[illegible]
68271	NR_027969	Zfp85os	zinc finger protein 85, opposite strand	lncRNA	.	1915521	.	0.125638	[illegible]
68272	NM_133925,XM_030255573	Rbm28	RNA binding motif protein 28	protein_coding	NP_598686.2;XP_030111433.1	2655711	.	10.488630	[illegible]
68273	NM_001290658,NM_026651,NM_029786,XM_006503352,XR_3763	Pomgnt1	protein O-linked mannose beta 1,2-N-acetylglucosaminyltransferase	protein_coding	NP_001277587.1;NP_080927.1;NP_084062.2;XP_006503415.1	1915523	.	17.593222	[illegible]
68275	NM_001164223,NM_026653,XM_006534051	Rpa1	replication protein A1	protein_coding	NP_001157695.1;NP_080929.1;XP_006534114.1	1915525	.	17.768219	[illegible]
68276	NM_026654,XM_006503353	Toe1	target of EGR1, member 1 (nuclear)	protein_coding	NP_080930.1;XP_006503416.1	1915526	.	10.609033	[illegible]
68277	NM_026655,NR_152380,XM_011241888,XM_030242951	2310057M21Rik	RIKEN cDNA 2310057M21 gene	protein_coding	NP_080931.1;XP_011240190.1;XP_030098811.1	1915527	.	3.213037	[illegible]
68278	NM_001363116,NM_001363117,NM_197982,NR_156447,XM_017312952	Ddx39	DEAD (Asp-Glu-Ala-Asp) box polypeptide 39	protein_coding	NP_001350045.1;NP_001350046.1;NP_932099.2;XP_017168441.1	1915528	.	16.677407	[illegible]
68279	NM_001005846,NM_026656,XM_006501969,XM_011240209,XM_030252775,XR_003954373,XR_003954374,XR_003954375	Mcoln2	mucolipin 2	protein_coding	NP_001005846.1;NP_080932.2;XP_006502032.1;XP_011238511.1;XP_030108635.1	1915529	.	1.196603	[illegible]
68280	XM_017320492	Gm12666	predicted gene 12666	protein_coding	XP_017175981.1	3650864	.	0.000000	[illegible]
68281	NM_175128,XM_006514028	4930430F08Rik	RIKEN cDNA 4930430F08 gene	protein_coding	NP_780337.2;XP_006514091.1	1921197	.	2.043325	[illegible]
68283	NM_026739,NR_152381,NR_152382,XM_006510568,XM_006510571,XM_006510573,XM_011242589,XM_011242592,XM_017313548,XM_030244543,XR_001779003,XR_003947994	9530077C05Rik	RIKEN cDNA 9530077C05 gene	protein_coding	NP_081015.1;XP_006510631.2;XP_006510634.1;XP_006510636.1;XP_011240891.2;XP_011240894.1;XP_017169037.1;XP_030100403.1	1915533	.	0.780186	[illegible]
68285	NR_027923	C630043F03Rik	RIKEN cDNA C630043F03 gene	lncRNA	.	1915535	.	2.613318	[illegible]
68291	NM_026658,XR_001779005,XR_003947995,XR_870765	Mto1	mitochondrial tRNA translation optimization 1	protein_coding	NP_080934.2	1915541	.	4.745334	[illegible]
68292	NM_024222	Stt3b	STT3, subunit of the oligosaccharyltransferase complex, homolog B (S. cerevisiae)	protein_coding	NP_077184.2	1915542	.	35.962605	[illegible]
68294	NM_001356978,NM_001356979,NM_026660,XM_006504079,XM_011240767,XM_011240768,XM_017321084,XM_030254781,XR_868298	Mfsd10	major facilitator superfamily domain containing 10	protein_coding	NP_001343907.1;NP_001343908.1;NP_080936.1;XP_006504142.1;XP_011239069.1;XP_011239070.1;XP_017176573.1;XP_030110641.1	1915544	.	18.325352	[illegible]
68295	NM_001164818,NM_026661,XM_006500097	Aar2	AAR2 splicing factor homolog	protein_coding	NP_001158290.1;NP_080937.3;XP_006500160.1	1915545	.	5.910425	[illegible]
68298	NM_146171,XM_006506531,XM_006506532,XM_006506533	Ncapd2	non-SMC condensin I complex, subunit D2	protein_coding	NP_666283.1;XP_006506594.1;XP_006506595.1;XP_006506596.1	1915548	.	15.987442	[illegible]
68299	NM_001364738,NM_026664,NR_157296	Vps53	VPS53 GARP complex subunit	protein_coding	NP_001351667.1;NP_080940.2	1915549	.	9.592380	[illegible]
68303	NM_026667	Fam114a1	family with sequence similarity 114, member A1	protein_coding	NP_080943.3	1915553	.	56.121372	[illegible]
68304	NM_212445,XM_006510574	Poglut3	protein O-glucosyltransferase 3	protein_coding	NP_997610.1;XP_006510637.1	1923765	.	17.029223	[illegible]
68308	NR_015568	4933406F09Rik	RIKEN cDNA 4933406F09 gene	lncRNA	.	1918289	.	0.001832	[illegible]
68310	NM_001356311,NM_026670,XM_030253734,XM_030253735	Zmym1	zinc finger, MYM domain containing 1	protein_coding	NP_001343240.1;NP_080946.3;XP_030109594.1;XP_030109595.1	1915560	.	2.645030	[illegible]
68311	NM_026671,XM_006521312	Lypd2	Ly6/Plaur domain containing 2	protein_coding	NP_080947.1;XP_006521375.1	1915561	.	0.192592	[illegible]
68312	NM_001356351,NM_026672,XM_006501971,XM_006501973	Gstm7	glutathione S-transferase, mu 7	protein_coding	NP_001343280.1;NP_080948.2;XP_006502034.1;XP_006502036.1	1915562	.	0.297054	[illegible]
68316	NM_001199337,NM_001199338,NM_001199339,NM_026673,XM_011247666,XM_011247667,XM_011247668,XM_011247669	Apoo	apolipoprotein O	protein_coding	NP_001186266.1;NP_001186267.1;NP_001186268.1;NP_080949.2;XP_011245968.1;XP_011245969.1;XP_011245970.1;XP_011245971.1	1915566	.	9.279727	[illegible]
68318	NM_026674,XM_006511398	Aph1c	aph1 homolog C, gamma secretase subunit	protein_coding	NP_080950.1;XP_006511461.1	1915568	.	0.515831	[illegible]
68323	NM_026675,XM_030251066	Nudt22	nudix (nucleoside diphosphate linked moiety X)-type motif 22	protein_coding	NP_080951.1;XP_030106926.1	1915573	.	7.757298	[illegible]
68327	NM_001163718,NM_026676,XM_006524840,XM_006524842,XM_011246586,XM_030250016	Tsr3	TSR3 20S rRNA accumulation	protein_coding	NP_001157190.1;NP_080952.2;XP_006524903.1;XP_006524905.1;XP_011244888.1;XP_030105876.1	1915577	.	11.462988	[illegible]
68328	NM_001293741,NM_026677,XR_375571	Rab13	RAB13, member RAS oncogene family	protein_coding	NP_001280670.1;NP_080953.1	1927232	.	17.574638	[illegible]
68332	NM_001033140	Sdhaf1	succinate dehydrogenase complex assembly factor 1	protein_coding	NP_001028312.2	1915582	.	9.131415	[illegible]
68337	NM_001347443,NM_024223,XM_030246890	Crip2	cysteine rich protein 2	protein_coding	NP_001334372.1;NP_077185.1;XP_030102750.1	1915587	.	38.906232	[illegible]
68339	NM_001362342,NM_026681,XM_006516187,XM_006516188,XM_011244166,XR_381542	Ccdc88c	coiled-coil domain containing 88C	protein_coding	NP_001349271.1;NP_080957.2;XP_006516250.1;XP_006516251.1;XP_011242468.1	1915589	.	2.834125	[illegible]
68342	NM_026684	Ndufb10	NADH:ubiquinone oxidoreductase subunit B10	protein_coding	NP_080960.1	1915592	.	58.251911	[illegible]
68346	NM_178848,XM_006516951,XM_006516952,XM_006516953,XM_006516954,XM_011244412,XM_011244413,XM_030247387,XM_030247388,XM_030247389,XM_030247390	Sirt5	sirtuin 5	protein_coding	NP_849179.1;XP_006517014.1;XP_006517015.1;XP_006517016.1;XP_006517017.1;XP_011242714.1;XP_011242715.1;XP_030103247.1;XP_030103248.1;XP_030103249.1;XP_030103250.1	1915596	.	4.026230	[illegible]
68347	NM_026686	Mettl26	methyltransferase like 26	protein_coding	NP_080962.1	1915597	.	6.893137	[illegible]
68349	NM_026688	Ndufs3	NADH:ubiquinone oxidoreductase core subunit S3	protein_coding	NP_080964.1	1915599	.	42.135822	[illegible]
68350	NM_026689,XM_006539134	Mul1	mitochondrial ubiquitin ligase activator of NFKB 1	protein_coding	NP_080965.2;XP_006539197.1	1915600	.	3.954017	[illegible]
68352	NM_026690	Aspdh	aspartate dehydrogenase domain containing	protein_coding	NP_080966.1	1915602	.	0.000000	[illegible]
68355	NR_027924,NR_027925	2010204K13Rik	RIKEN cDNA 2010204K13 gene	lncRNA	.	1922859	.	2.048679	[illegible]
68364	NM_026696,XM_006506534	0610030E20Rik	RIKEN cDNA 0610030E20 gene	protein_coding	NP_080972.1;XP_006506597.1	1915614	.	8.980615	[illegible]
68365	NM_026697	Rab14	RAB14, member RAS oncogene family	protein_coding	NP_080973.1	1915615	.	43.399487	[illegible]
68366	NM_001356983,NM_001356985,NM_026698	Tmem129	transmembrane protein 129	protein_coding	NP_001343912.1;NP_001343914.1;NP_080974.2	1915616	.	8.927626	[illegible]
68371	NM_001359529,NM_026701,NR_153192,NR_153193,XM_006514029,XM_006514030,XM_006514031	Pbld1	phenazine biosynthesis-like protein domain containing 1	protein_coding	NP_001346458.1;NP_080977.2;XP_006514092.1;XP_006514093.1;XP_006514094.1	1915621	.	0.071635	[illegible]
68375	NM_026703	Ndufa8	NADH:ubiquinone oxidoreductase subunit A8	protein_coding	NP_080979.1	1915625	.	37.240723	[illegible]
68379	NM_001252534,NM_001252536,NM_001252537,NM_001252538,NM_028412,XM_006498271,XM_006498272,XM_006498273,XM_006498274,XM_006498275,XM_006498276,XM_006498277,XM_006498278,XM_006498279,XM_006498280,XM_006498281,XM_006498282,XM_006498283,XM_006498285,XM_011239168,XM_011239169,XM_030252016,XM_030252017,XM_030252018,XM_030252019,XM_030252020,XM_030252021,XM_030252022,XM_030252023,XM_030252024,XM_030252025,XM_030252026,XM_030252027	Ciz1	CDKN1A interacting zinc finger protein 1	protein_coding	NP_001239463.1;NP_001239465.1;NP_001239466.1;NP_001239467.1;NP_082688.1;XP_006498334.1;XP_006498335.1;XP_006498336.1;XP_006498337.1;XP_006498338.1;XP_006498339.1;XP_006498340.1;XP_006498341.1;XP_006498342.1;XP_006498343.1;XP_006498344.1;XP_006498345.1;XP_006498346.1;XP_006498348.1;XP_011237470.1;XP_011237471.1;XP_030107876.1;XP_030107877.1;XP_030107878.1;XP_030107879.1;XP_030107880.1;XP_030107881.1;XP_030107882.1;XP_030107883.1;XP_030107884.1;XP_030107885.1;XP_030107886.1;XP_030107887.1	1920234	.	16.202633	[illegible]
68385	NM_001291235,NM_001291236,NM_001291237,NM_026708,XM_006534054,XM_011249203	Tlcd1	TLC domain containing 1	protein_coding	NP_001278164.1;NP_001278165.1;NP_001278166.1;NP_080984.1;XP_006534117.1;XP_011247505.1	1915572	.	9.348293	[illegible]
68386	NR_028113	0610039K10Rik	RIKEN cDNA 0610039K10 gene	lncRNA	.	1915636	.	0.049622	[illegible]
68393	NM_026713,XM_011238707,XM_017322281,XM_017322283,XM_017322285	Mogat1	monoacylglycerol O-acyltransferase 1	protein_coding	NP_080989.2;XP_011237009.1;XP_017177770.1;XP_017177772.1;XP_017177774.1	1915643	.	0.000000	[illegible]
68394	NM_001356315,NM_026714,NR_151451,NR_151452,XM_006503358,XM_006503359,XM_006503360,XM_006503361,XM_006503363,XM_006503365,XM_006503368,XM_006503369,XM_017320376,XM_017320377,XM_017320378,XM_030253736,XM_030253737,XM_030253738,XM_030253739,XM_030253740,XR_001784191,XR_001784194,XR_003955001,XR_003955003,XR_003955004,XR_376350,XR_376351	Ccdc163	coiled-coil domain containing 163	protein_coding	NP_001343244.1;NP_080990.2;XP_006503421.1;XP_006503422.1;XP_006503423.1;XP_006503424.1;XP_006503426.1;XP_006503428.1;XP_006503431.1;XP_006503432.1;XP_017175865.1;XP_017175866.1;XP_017175867.1;XP_030109596.1;XP_030109597.1;XP_030109598.1;XP_030109599.1;XP_030109600.1	1915644	.	7.615486	[illegible]
68396	NM_001362060,NM_023455,XM_006506536	Nat8	N-acetyltransferase 8 (GCN5-related)	protein_coding	NP_001348989.1;NP_075944.1;XP_006506599.1	1915646	.	0.077699	[illegible]
68400	NR_040640	0610043K17Rik	RIKEN cDNA 0610043K17 gene	lncRNA	.	1915650	.	0.381797	[illegible]
68401	NM_175935	G6pc3	glucose 6 phosphatase, catalytic, 3	protein_coding	NP_787949.2	1915651	.	13.489875	[illegible]
68404	NM_153529,XM_006516738	Nrn1	neuritin 1	protein_coding	NP_705757.1;XP_006516801.1	1915654	.	0.279498	[illegible]
68420	NM_026718,XM_006530454	Ankrd13a	ankyrin repeat domain 13a	protein_coding	NP_080994.2;XP_006530517.3	1915670	.	23.828785	[illegible]
68421	NM_001310483,NM_026719,XM_017322292,XM_030242602	Lmbrd1	LMBR1 domain containing 1	protein_coding	NP_001297412.1;NP_080995.2;XP_017177781.1;XP_030098462.1	1915671	.	6.754756	[illegible]
68423	NM_026720,XM_030251067,XM_030251068,XM_030251069,XR_003952812	Ankrd13d	ankyrin repeat domain 13 family, member D	protein_coding	NP_080996.2;XP_030106927.1;XP_030106928.1;XP_030106929.1	1915673	.	1.528562	[illegible]
68427	NM_001290765,NM_026721,NR_110981,XM_006500099,XM_006500100,XM_006500101,XM_011239756,XM_017319236,XM_017319238,XM_030252029,XM_030252030,XM_030252031,XR_003953709,XR_003953710,XR_003953711,XR_003953712,XR_374515	Slc39a13	solute carrier family 39 (metal ion transporter), member 13	protein_coding	NP_001277694.1;NP_080997.1;XP_006500162.1;XP_006500163.1;XP_006500164.1;XP_011238058.1;XP_017174725.1;XP_017174727.1;XP_030107889.1;XP_030107890.1;XP_030107891.1	1915677	.	66.075105	[illegible]
68428	NM_001085409,NM_133186,XM_006529845,XM_006529846	Steap3	STEAP family member 3	protein_coding	NP_001078878.1;NP_573449.2;XP_006529908.1;XP_006529909.1	1915678	.	8.391540	[illegible]
68431	NM_133694,XM_006527316	Fbxl15	F-box and leucine-rich repeat protein 15	protein_coding	NP_598455.2;XP_006527379.1	1915681	.	2.199377	[illegible]
68434	NR_105022,NR_105023	1010001N08Rik	RIKEN cDNA 1010001N08 gene	lncRNA	.	1915684	.	0.068114	[illegible]
68436	NM_001005859,NM_001287581,NM_026724	Rpl34	ribosomal protein L34	protein_coding	NP_001005859.1;NP_001274510.1;NP_081000.1	1915686	.	601.125948	[illegible]
68440	NM_026725	Dusp23	dual specificity phosphatase 23	protein_coding	NP_081001.1	1915690	.	4.557327	[illegible]
68441	NM_178376	Rraga	Ras-related GTP binding A	protein_coding	NP_848463.1	1915691	.	26.639471	[illegible]

68449	NM_144522	Tbc1d10b	TBC1 domain family, member 10b	protein_coding	NP_653105.3	1915699	.	10.484219
68453	NM_001347039,NM_001361294,NM_026730	Gpihbp1	GPI-anchored HDL-binding protein 1	protein_coding	NP_001333968.1;NP_001348223.1;NP_081006.1	1915703	.	20.102835
68458	NM_026731	Ppp1r14a	protein phosphatase 1, regulatory inhibitor subunit 14A	protein_coding	NP_081007.2	1931139	.	1.362205
68460	NM_001013013	Dhrs7c	dehydrogenase/reductase (SDR family) member 7C	protein_coding	NP_001013031.2	1915710	.	2.845087
68463	NM_026732,XM_006524843,XM_006524844,XM_017317618	Mrpl14	mitochondrial ribosomal protein L14	protein_coding	NP_081008.1;XP_006524906.1;XP_006524907.1;XP_017173107.1	1333864	.	17.090961
68465	NM_001355692,NM_197985,XM_030255574,XM_030255575,XM_030255576	Adipor2	adiponectin receptor 2	protein_coding	NP_001342621.1;NP_932102.2;XP_030111434.1;XP_030111435.1;XP_030111436.1	93830	.	15.420946
68469	NR_152104	Sox6os	SRY (sex determining region Y)-box 6, opposite strand	lncRNA	.	1915719	.	0.178700
68472	NM_026734	Tmem126b	transmembrane protein 126B	protein_coding	NP_081010.1	1915722	.	7.110647
68473	NM_026735	Mob1b	MOB kinase activator 1B	protein_coding	NP_081011.1	1915723	.	5.309209
68475	NM_023464	Ssna1	SS nuclear autoantigen 1	protein_coding	NP_075953.1	1915725	.	25.783722
68477	NM_001355745,NM_024288	Rmnd5a	required for meiotic nuclear division 5 homolog A	protein_coding	NP_001342674.1;NP_077250.2	1915727	.	15.843702
68479	NM_026737	Phf5a	PHD finger protein 5A	protein_coding	NP_081013.1	2156864	.	15.577262
68480	NM_026738,XM_006516955,XR_003950472	Card19	caspase recruitment domain family, member 19	protein_coding	NP_081014.1;XP_006517018.1	1915730	.	29.647167
68481	NM_001001880,NM_001083897,NM_001347128	Mpzl1	myelin protein zero-like 1	protein_coding	NP_001001880.1;NP_001077366.1;NP_001334057.1	1915731	.	51.015288
68483	XR_373311,XR_373312	1110002O04Rik	RIKEN cDNA 1110002O04 gene, transcript variant X1	lncRNA	.	1915733	.	0.000000
68487	NM_001368365,NM_197986,XM_006506541	Tmem140	transmembrane protein 140	protein_coding	NP_001355294.1;NP_932103.1;XP_006506604.1	1915737	.	1.725424
68488	NR_138560,NR_138561	1110002J07Rik	RIKEN cDNA 1110002J07 gene	lncRNA	.	1915738	.	0.159438
68490	NM_026741,XM_006540335	Zfp579	zinc finger protein 579	protein_coding	NP_081017.1;XP_006540398.1	1915740	.	11.388960
68493	NM_026742	Ndufaf4	NADH:ubiquinone oxidoreductase complex assembly factor 4	protein_coding	NP_081018.1	1915743	.	3.953330
68497	NM_001364189,NM_001364190,NM_178065,NR_157097,XM_006516191,XM_030246891,XM_030246892,XR_872717	Arel1	apoptosis resistant E3 ubiquitin protein ligase 1	protein_coding	NP_001351118.1;NP_001351119.1;NP_835166.3;XP_006516254.1;XP_030102751.1;XP_030102752.1	1915747	.	8.245215
68498	NM_026743,XM_017321722	Tspan11	tetraspanin 11	protein_coding	NP_081019.1;XP_017177211.1	1915748	.	24.593308
68499	NM_026744	Mrpl53	mitochondrial ribosomal protein L53	protein_coding	NP_081020.1	1915749	.	22.485985
68501	NM_001164604,NM_026746,XM_006521316,XM_006521317,XM_017316737,XM_030248724	Nsmce2	NSE2/MMS21 homolog, SMC5-SMC6 complex SUMO ligase	protein_coding	NP_001158076.1;NP_081022.2;XP_006521379.1;XP_006521380.1;XP_017172226.1;XP_030104584.1	1915751	.	11.084389
68505	NM_001081041	Vps51	VPS51 GARP complex subunit	protein_coding	NP_001074510.1	1915755	.	12.309076
68507	NM_001144855,XM_006529847,XM_006529848,XM_006529849,XR_001785569,XR_001785570,XR_001785571,XR_003946481,XR_003946482,XR_387189,XR_387190,XR_387191,XR_878325	Ppfia4	protein tyrosine phosphatase, receptor type, f polypeptide (PTPRF), interacting protein (liprin), alpha 4	protein_coding	NP_001138327.1;XP_006529910.1;XP_006529911.1;XP_006529912.1	1915757	.	1.718428
68509	NM_001163416,NM_026747,XM_006524845,XM_030250017	Ptx4	pentraxin 4	protein_coding	NP_001156888.1;NP_081023.1;XP_006524908.1;XP_030105877.1	1915759	.	2.585241
68510	NM_026748,XM_006504727,XM_006504728,XM_006504729,XR_003955703,XR_003955704	Ints1	integrator complex subunit 1	protein_coding	NP_081024.3;XP_006504790.1;XP_006504791.1;XP_006504792.1	1915760	.	6.272362
68511	NM_001177964,XM_006515184,XM_006515185,XM_006515186,XM_017315166,XM_017315167	Dcdc2c	doublecortin domain containing 2C	protein_coding	NP_001171435.2;XP_006515247.1;XP_006515248.1;XP_006515249.1;XP_017170655.1;XP_017170656.1	1915761	.	0.017147
68512	NM_001099675,NM_001134646	Tomm5	translocase of outer mitochondrial membrane 5	protein_coding	NP_001093145.1;NP_001128118.1	1915762	.	25.065729
68514	NM_028643,XM_006519484	Micu2	mitochondrial calcium uptake 2	protein_coding	NP_082919.1;XP_006519547.1	1915764	.	20.622868
68515	NM_001204820,NM_026751,XM_017314729	Myadml2	myeloid-associated differentiation marker-like 2	protein_coding	NP_001191749.1;NP_081027.1;XP_017170218.1	1915765	.	0.847893
68519	NM_001043335,NM_001043336,NM_001286346,NM_001286347,NM_001364193,NM_001364194,NM_001364195,NM_001364196,NM_001364197,NM_001364198,NM_001364199,XM_017315168,XM_030246893,XM_030246894,XM_030246895	Eml1	echinoderm microtubule associated protein like 1	protein_coding	NP_001036800.1;NP_001036801.1;NP_001273275.1;NP_001273276.1;NP_001351122.1;NP_001351123.1;NP_001351124.1;NP_001351125.1;NP_001351126.1;NP_001351127.1;NP_001351128.1;XP_017170657.1;XP_030102753.1;XP_030102754.1;XP_030102755.1	1915769	.	18.510324
68520	NM_026752,XM_006516192,XM_006516193,XM_006516194,XM_006516195,XM_006516196,XM_011244168	Zfyve21	zinc finger, FYVE domain containing 21	protein_coding	NP_081028.3;XP_006516255.1;XP_006516256.1;XP_006516257.1;XP_006516258.1;XP_006516259.1;XP_011242470.1	1915770	.	20.809744
68521	NM_001014995,NM_001355760,NM_001355765,XM_006501977,XM_006501978,XM_006501981,XM_030252776,XM_030252777,XM_030252778,XR_001783738	Fam189b	family with sequence similarity 189, member B	protein_coding	NP_001014995.1;NP_001342689.1;NP_001342694.1;XP_006502040.1;XP_006502041.1;XP_006502044.1;XP_030108636.1;XP_030108637.1;XP_030108638.1	1915771	.	11.351157
68523	NM_026753	Ciao2b	cytosolic iron-sulfur assembly component 2B	protein_coding	NP_081029.1	1915773	.	12.698439
68524	NM_197940	Wipf2	WAS/WASL interacting protein family, member 2	protein_coding	NP_922922.1	1924462	.	3.261259
68525	NM_145920,XM_006504082	Evc2	EvC ciliary complex subunit 2	protein_coding	NP_666032.1;XP_006504145.1	1915775	.	10.226311
68526	NM_001190297,NM_001276443,NM_001276444,XM_006500104,XM_006500105,XM_006500106,XM_006500107,XM_006500108	Gpr155	G protein-coupled receptor 155	protein_coding	NP_001177226.1;NP_001263372.1;NP_001263373.1;XP_006500167.1;XP_006500168.1;XP_006500169.1;XP_006500170.1;XP_0065017	1915776	.	2.221639
68527	NM_001113558,NM_001165932,NM_001311208,NM_026754	Ucma	upper zone of growth plate and cartilage matrix associated	protein_coding	NP_001107030.1;NP_001159404.1;NP_001298137.1;NP_081030.1	1915777	.	72.696441
68528	NM_001162998	Smim6	small integral membrane protein 6	protein_coding	NP_001156470.1	1915778	.	0.210487
68531	NR_027929,NR_027930	1110020A21Rik	RIKEN cDNA 1110020A21 gene	lncRNA		1915781	.	0.399516
68533	NM_001357232,NM_001357233,NM_026758	Mphosph6	M phase phosphoprotein 6	protein_coding	NP_001344161.1;NP_001344162.1;NP_081034.1	1915783	.	16.418211
68537	NM_026759	Mrpl13	mitochondrial ribosomal protein L13	protein_coding	NP_081035.1	2137218	.	26.333876
68539	NM_134142,XM_006527317	Tmem109	transmembrane protein 109	protein_coding	NP_598903.1;XP_006527380.1	1915789	.	30.981102
68544	NM_026760,NR_160793,XM_030243747,XM_030243748	Trir	telomerase RNA component interacting RNase	protein_coding	NP_081036.2;XP_030099607.1;XP_030099608.1	1922833	.	54.893113
68545	NM_001033141,NM_001368651,NM_001368652,XM_030250592	Ecscr	endothelial cell surface expressed chemotaxis and apoptosis regulator	protein_coding	NP_001028313.1;NP_001355580.1;NP_001355581.1;XP_030106452.	1915795	.	10.111210
68549	NM_001177867,NM_199007	Sgo2a	shugoshin 2A	protein_coding	NP_001171338.1;NP_950172.1	1098767	.	2.799996
68550	NM_183275,XM_011249204,XM_011249205,XM_030246262	Tefm	transcription elongation factor, mitochondrial	protein_coding	NP_899098.2;XP_011247506.1;XP_011247507.1;XP_030102122.1	1915800	.	3.725255
68552	NM_133697,XM_006504083,XM_006504084,XM_006504085,XM_006504086,XM_006504087,XM_006504088,XM_006504089,XM_030254782,XM_030254783	Smim14	small integral membrane protein 14	protein_coding	NP_598458.1;XP_006504146.1;XP_006504147.1;XP_006504148.1;XP_006504149.1;XP_006504150.1;XP_006504151.1;XP_006504152.1;XP_030110642.1;XP_030110643.1	1915802	.	36.642518
68553	NM_026763,XM_011242907,XM_030244544	Col6a4	collagen, type VI, alpha 4	protein_coding	NP_081039.2;XP_011241209.1;XP_030100404.1	1915803	.	0.017372
68554	NM_001177402,NM_001177403,XM_006524847,XM_030250018	Cebpzos	CCAAT/enhancer binding protein (C/EBP), zeta, opposite strand	protein_coding	NP_001170873.1;NP_001170874.1;XP_006524910.1;XP_030105878.	1915804	.	4.552974
68556	NM_026765,XM_006500704,XM_006500705,XM_006500706,XM_006500708,XM_006500709,XM_006500710,XM_011239960,XM_030252032,XM_030252033,XM_030252034,XM_030252035,XM_030252036,XM_030252037	Uckl1	uridine-cytidine kinase 1-like 1	protein_coding	NP_081041.2;XP_006500767.1;XP_006500768.1;XP_006500769.1;XP_006500771.1;XP_006500772.1;XP_006500773.1;XP_011238262.1;XP_030107892.1;XP_030107893.1;XP_030107894.1;XP_030107895.1;XP_030107896.1;XP_030107897.1	1915806	.	10.989124
68558	NM_001271388,NM_001271389,NM_001271390,NM_001271391,NM_001271392,NM_023472,XM_017315580	Ankra2	ankyrin repeat, family A (RFXANK-like), 2	protein_coding	NP_001258317.1;NP_001258318.1;NP_001258319.1;NP_001258320.1;NP_001258321.1;NP_075961.1;XP_017171069.1	1915808	.	10.852013
68559	NM_178939,XM_030252038	Pdrg1	p53 and DNA damage regulated 1	protein_coding	NP_849270.1;XP_030107898.1	1915809	.	38.108292
68563	NM_026767,XM_006501983,XM_030252779	Dpm3	dolichyl-phosphate mannosyltransferase polypeptide 3	protein_coding	NP_081043.1;XP_006502046.1;XP_030108639.1	1915813	.	30.518222
68564	NM_001024205,XM_006534062,XM_006534063	Nufip2	nuclear fragile X mental retardation protein interacting protein 2	protein_coding	NP_001019376.1;XP_006534125.1;XP_006534126.1	1915814	.	10.152879
68565	NM_026768	Mrps18a	mitochondrial ribosomal protein S18A	protein_coding	NP_081044.1	1915815	.	20.395613
68566	NM_001190385,NM_001190386,NM_026769,XM_006536235,XM_006536236,XM_006536237,XM_011249853	Caly	calcyon neuron-specific vesicular protein	protein_coding	NP_001177314.1;NP_001177315.1;NP_081045.2;XP_006536298.1;XP_006536299.1;XP_006536300.1;XP_011248155.1	1915816	.	0.000000
68567	NM_001160149,NM_026770	Cgref1	cell growth regulator with EF hand domain 1	protein_coding	NP_001153621.1;NP_081046.1	1915817	.	88.907528
68572	NM_001347645,NM_001361462,NM_001361463,NM_026729,NR_154991,XM_030246263	Mrpl58	mitochondrial ribosomal protein L58	protein_coding	NP_001334574.1;NP_001348391.1;NP_001348392.1;NP_081005.1;XP_030102123.1	1915822	.	13.181002
68576	NM_026774	Lamtor5	late endosomal/lysosomal adaptor, MAPK and MTOR activator 5	protein_coding	NP_081050.2	1915826	.	27.177813
68581	NM_026775	Tmed10	transmembrane p24 trafficking protein 10	protein_coding	NP_081051.1	1915831	.	70.778198
68585	NM_024226,NM_194051,NM_194052,NM_194053,NM_194054	Rtn4	reticulon 4	protein_coding	NP_077188.1;NP_918940.1;NP_918941.1;NP_918942.1;NP_918943.1	1915835	.	29.773397
68588	NM_001364812,NM_001364813,NM_026778	Cthrc1	collagen triple helix repeat containing 1	protein_coding	NP_001351741.1;NP_001351742.1;NP_081054.1	1915838	.	305.728820
68591	NM_026779,XM_006526211,XM_011246994,XR_877139	Mocos	molybdenum cofactor sulfurase	protein_coding	NP_081055.1;XP_006526274.1;XP_011245296.1	1915841	.	0.656331
68592	NM_026780	Syf2	SYF2 homolog, RNA splicing factor (S. cerevisiae)	protein_coding	NP_081056.1	1915842	.	27.399359
68597	NM_001163741,NM_026782,XR_003952173	Ccdc167	coiled-coil domain containing 167	protein_coding	NP_001157213.1;NP_081058.1	1915847	.	3.738692
68598	NM_172400	Dnajc8	DnaJ heat shock protein family (Hsp40) member C8	protein_coding	NP_765988.2	1915848	.	42.381783
68603	NM_001310640,NM_026784,NM_027348,XM_030252780	Pmvk	phosphomevalonate kinase	protein_coding	NP_001297569.1;NP_081060.2;NP_081624.1;XP_030108640.1	1915853	.	14.116847
68606	NM_176833,XM_006522508,XM_017317124	Ppm1f	protein phosphatase 1F (PP2C domain containing)	protein_coding	NP_789803.1;XP_006522571.1;XP_017172613.1	1918464	.	8.926822
68607	NM_023475,XM_030248725,XR_384036	Serhl	serine hydrolase-like	protein_coding	NP_075964.1;XP_030104585.1	1890404	.	17.213253
68611	NM_024227	Mrpl28	mitochondrial ribosomal protein L28	protein_coding	NP_077189.2	1915861	.	21.964464
68612	NM_026785	Ube2c	ubiquitin-conjugating enzyme E2C	protein_coding	NP_081061.1	1915862	.	18.145094
68614	NM_001357982,NM_001357983,NM_001357984,NM_134093,XM_006521320,XM_006521322,XM_017316739,XM_030248726	Letmd1	LETM1 domain containing 1	protein_coding	NP_001344911.1;NP_001344912.1;NP_001344913.1;NP_598854.1;XP_006521383.1;XP_006521385.1;XP_017172228.1;XP_030104586.1	1915864	.	7.461081
68616	NM_024228	Gdpd3	glycerophosphodiester phosphodiesterase domain containing 3	protein_coding	NP_077190.2	1915866	.	1.212026
68617	NM_001114098,NM_172963,XM_006524849,XM_006524851,XM_006524852,XM_006524853,XM_006524856,XM_011246587,XM_017317622,XR_001782108	Mtcl1	microtubule crosslinking factor 1	protein_coding	NP_001107570.1;NP_766551.3;XP_006524912.1;XP_006524914.1;XP_006524915.1;XP_006524916.1;XP_006524919.1;XP_011244889.1;XP_017173111.1	1915867	.	0.928411
68618	NM_026787	1110012L19Rik	RIKEN cDNA 1110012L19 gene	protein_coding	NP_081063.1	1915868	.	8.148209
68625	NM_026789,XM_006503371,XR_003955005	Cfap57	cilia and flagella associated protein 57	protein_coding	NP_081065.2;XP_006503434.1	2686209	.	0.000000
68626	NM_001362982,NM_001362983,NM_001362984,NM_023479,XM_006534065,XM_006534066,XM_006534067,XM_006534068,XM_030246264	Elac2	elaC ribonuclease Z 2	protein_coding	NP_001349911.1;NP_001349912.1;NP_001349913.1;NP_075968.2;XP_006534128.1;XP_006534129.1;XP_006534130.1;XP_006534131.1;XP_030102124.1	1890496	.	7.952954
68628	NM_026791	Fbxw9	F-box and WD-40 domain protein 9	protein_coding	NP_081067.2	1915878	.	7.749953
68631	NM_030004	Cryl1	crystallin, lambda 1	protein_coding	NP_084280.2	1915881	.	14.776880
68632	NM_026793	Myct1	myc target 1	protein_coding	NP_081069.1	1915882	.	3.253113
68634	NM_026795,NM_178056	Tm2d3	TM2 domain containing 3	protein_coding	NP_081071.1;NP_835157.1	1915884	.	14.015428
68636	NM_023480	Fahd1	fumarylacetoacetate hydrolase domain containing 1	protein_coding	NP_075969.1	1915886	.	5.697046
68642	NM_001277860,NM_001277861,NM_026798,XM_006527318,XM_006527319,XR_386504,XR_386505	Tmem216	transmembrane protein 216	protein_coding	NP_001264789.1;NP_001264790.1;NP_081074.1;XP_006527381.1;XP_006527382.1	1920020	.	4.780485
68644	NM_001110271,NM_001110272,NM_001359225,XM_006511796,XM_006511802,XM_006511803,XM_006511805,XM_011242910,XM_017313549,XM_017313550,XM_017313551,XM_017313552,XM_017313553,XM_017313554,XM_017313555,XM_030244546,XM_030244547,XM_030244548,XM_030244549	Abhd14a	abhydrolase domain containing 14A	protein_coding	NP_001103741.2;NP_001103742.2;NP_001346154.2;XP_006511859.1;XP_006511865.1;XP_006511866.1;XP_006511868.1;XP_011241212.1;XP_017169038.1;XP_017169039.1;XP_017169040.1;XP_017169041.1;XP_017169042.1;XP_017169043.1;XP_017169044.1;XP_030100406.1;XP_030100407.1;XP_030100408.1;XP_030100409.1	1915894	.	18.976657
68646	NM_001040395,NM_001085410,NM_001286253,NM_001286255	Nadk2	NAD kinase 2, mitochondrial	protein_coding	NP_001035485.2;NP_001078879.1;NP_001273182.1;NP_001273184.	1915896	.	8.553549
68652	NM_001359534,NM_138667,XM_011243203,XM_011243204,XM_030245247	Tab2	TGF-beta activated kinase 1/MAP3K7 binding protein 2	protein_coding	NP_001346463.1;NP_619608.1;XP_011241505.1;XP_011241506.1;XP_030101107.1	1915902	.	42.910195
68653	NM_178614	Samm50	SAMM50 sorting and assembly machinery component	protein_coding	NP_848729.1	1915903	.	15.150829
68655	NM_001081416,XM_006523349,XM_006523350,XM_006523352,XR_385179	Fndc1	fibronectin type III domain containing 1	protein_coding	NP_001074885.2;XP_006523412.1;XP_006523413.1;XP_006523415.1	1915905	.	38.520086
68659	NM_133187,XM_006501984,XM_006501985	Gask1b	golgi associated kinase 1B	protein_coding	NP_573450.2;XP_006502047.1;XP_006502048.1	1915909	.	13.831088
68662	NM_054037,NM_170727	Scgb3a1	secretoglobin, family 3A, member 1	protein_coding	NP_473378.1;NP_733923.1	1915912	.	0.000000
68666	NM_001359214,NM_026805	Svop	SV2 related protein	protein_coding	NP_001346143.1;NP_081081.1	1915916	.	0.034555
68667	NM_175130,XM_006541146,XM_030242952,XR_001777997,XR_003946552,XR_003946553,XR_003946554,XR_391361,XR_882033,XR_882034,XR_882035,XR_882036	Trpm4	transient receptor potential cation channel, subfamily M, member 4	protein_coding	NP_780339.2;XP_006541209.1;XP_030098812.1	1915917	.	10.912646
68671	NM_001347615,NM_001363001,NM_024229,XM_006534069,XM_006534071,XR_001780048,XR_003949495,XR_879733	Pcyt2	phosphate cytidylyltransferase 2, ethanolamine	protein_coding	NP_001334544.1;NP_001349930.1;NP_077191.2;XP_006534132.1;XP_006534134.1	1915921	.	15.288171
68673	NM_026807	Krtap4-2	keratin associated protein 4-2	protein_coding	NP_081083.2	1915923	.	0.000000
68675	NM_001163419,NM_001163420,NM_138312,NR_028109,XM_006517350,XM_006517352,XM_006517353,XM_006517355,XM_017315581,XM_017315582,XM_030247391,XM_030247392,XR_001780804,XR_003950473	Fam172a	family with sequence similarity 172, member A	protein_coding	NP_001156891.1;NP_001156892.1;NP_612185.1;XP_006517413.1;XP_006517415.1;XP_006517416.1;XP_006517418.1;XP_017171070.1;XP_017171071.1;XP_030103251.1;XP_030103252.1	1915925	.	9.273983
68678	NM_024230,XM_006500109	Smtnl1	smoothelin-like 1	protein_coding	NP_077192.1;XP_006500172.1	1915928	.	5.497892
68680	NM_026808	Fitm1	fat storage-inducing transmembrane protein 1	protein_coding	NP_081084.1	1915930	.	2.456530
68682	NM_001199186,NM_001359227,NM_152808,XM_006510576,XM_011242593,XM_030244550	Slc44a2	solute carrier family 44, member 2	protein_coding	NP_001186115.1;NP_001346156.1;NP_690021.1;XP_006510639.1;XP_011240895.1;XP_030100410.1	1915932	.	46.690912
68690	NR_045139	1110028F11Rik	RIKEN cDNA 1110028F11 gene	lncRNA	.	1915940	.	0.000000
68691	NM_001122738,NM_001357156,NM_001357157,NM_001368830,NM_177645,XM_006496241,XM_006496249,XM_011238570,XM_011238571,XM_011238572,XM_011238573,XM_011238574,XM_011238575,XM_011238576,XM_011238577,XM_011238578,XM_011238579,XM_011238580,XM_011238581,XM_011238582,XM_011238583,XM_011238584,XM_017322311,XM_017322316,XM_017322317,XM_030242647,XM_030242649,XM_030242665,XM_030242669,XM_030242671,XR_001785576,XR_003946488,XR_003946490	Kansl1l	KAT8 regulatory NSL complex subunit 1-like	protein_coding	NP_001116210.1;NP_001344085.1;NP_001344086.1;NP_001355759.1;NP_808313.3;XP_006496304.1;XP_006496312.1;XP_011236872.1;XP_011236873.1;XP_011236874.1;XP_011236875.1;XP_011236876.1;XP_011236877.1;XP_011236878.1;XP_011236879.1;XP_011236880.1;XP_011236881.1;XP_011236882.1;XP_011236883.1;XP_011236884.1;XP_011236885.1;XP_011236886.1;XP_017177800.1;XP_017177805.1;XP_017177806.1;XP_030098507.1;XP_030098509.1;XP_030098525.1;XP_030098529.1;XP_030098531.1	1915941	.	5.366541
68693	NM_001081196,XR_003952813	Hnrnpul2	heterogeneous nuclear ribonucleoprotein U-like 2	protein_coding	NP_001074665.1	1915943	.	36.451534
68695	NM_026812,XM_006541153,XM_006541154,XM_006541155,XM_030242953	Hddc3	HD domain containing 3	protein_coding	NP_081088.1;XP_006541216.1;XP_006541217.1;XP_006541218.1;XP_030098813.1	1915945	.	3.043747
68697	NR_040713	1110036E04Rik	RIKEN cDNA 1110036E04 gene	lncRNA	.	1915947	.	0.000000
68701	NM_026814	Ppp1r27	protein phosphatase 1, regulatory subunit 27	protein_coding	NP_081090.1	1915951	.	0.338650
68703	NM_001085492,XM_006539136,XM_006539137,XM_006539139,XM_006539143,XM_006539144,XM_006539145,XM_006539147,XM_030253741,XM_030253742,XM_030253743,XM_030253744,XM_030253745,XM_030253746,XM_030253747,XM_030253748,XM_030253749,XM_030253750,XM_030253751,XM_030253752,XM_030253753,XM_030253754	Rere	arginine glutamic acid dipeptide (RE) repeats	protein_coding	NP_001078961.1;XP_006539199.1;XP_006539200.1;XP_006539202.1;XP_006539206.1;XP_006539207.1;XP_006539208.1;XP_006539210.1;XP_030109601.1;XP_030109602.1;XP_030109603.1;XP_030109604.1;XP_030109605.1;XP_030109606.1;XP_030109607.1;XP_030109608.1;XP_030109609.1;XP_030109610.1;XP_030109611.1;XP_030109612.1;XP_030109613.1;XP_030109614.1	2683486	.	13.396598
68705	NM_026816,XM_030247985	Gtf2f2	general transcription factor IIF, polypeptide 2	protein_coding	NP_081092.1;XP_030103845.1	1915955	.	7.547681
68708	NM_026817,XM_006521326,XM_006521327,XM_011245717,XM_030248727,XM_030248728,XM_030248729,XM_030248730,XM_030248731	Rabl2	RAB, member RAS oncogene family-like 2	protein_coding	NP_081093.2;XP_006521389.1;XP_006521390.1;XP_011244019.1;XP_030104587.1;XP_030104588.1;XP_030104589.1;XP_030104590.1;XP_030104591.1	1915958	.	8.106490
68709	NM_026818	Cilp2	cartilage intermediate layer protein 2	protein_coding	NP_081094.2	1915959	.	8.231314
68713	NM_001112715,NM_001360727,NM_001360728,NM_026820	Ifitm1	interferon induced transmembrane protein 1	protein_coding	NP_001106186.1;NP_001347656.1;NP_001347657.1;NP_081096.3	1915963	.	25.881477
68718	NM_001033142,NM_001368370	Rnf166	ring finger protein 166	protein_coding	NP_001028314.1;NP_001355299.1	1915968	.	16.823155
68720	NM_026822	Lce1b	late cornified envelope 1B	protein_coding	NP_081098.1	1915970	.	0.000000
68721	NM_001311073,NM_001361107,NM_001361108,NM_023483,XM_006510578,XM_030244552	1110032A03Rik	RIKEN cDNA 1110032A03 gene	protein_coding	NP_001298002.1;NP_001348036.1;NP_001348037.1;NP_075972.2;XP_006510641.1;XP_030100412.1	1915971	.	4.496912
68724	NM_026823	Arl8a	ADP-ribosylation factor-like 8A	protein_coding	NP_081099.1	1915974	.	29.575373
68725	NM_001167996	1110032F04Rik	RIKEN cDNA 1110032F04 gene	protein_coding	NP_001161468.1	1915975	.	0.114997
68728	NM_178111,XM_006500113,XM_006500114,XM_017319242,XM_030252039,XM_030252040	Trp53inp2	transformation related protein 53 inducible nuclear protein 2	protein_coding	NP_835212.1;XP_006500176.1;XP_006500177.1;XP_017174731.1;XP_030107899.1;XP_030107900.1	1915978	.	14.396472
68729	NM_001363025,NM_001363026,NM_001363027,NM_001363028,NM_197987,XM_006534072,XM_011249210,XM_011249212	Trim37	tripartite motif-containing 37	protein_coding	NP_001349954.1;NP_001349955.1;NP_001349956.1;NP_001349957.1;NP_932104.1;XP_006534135.1;XP_011247512.1;XP_011247514.1	2153072	.	5.268525
68730	NM_026824,XM_006534077,XM_011249213,XM_011249214,XM_011249215,XM_011249216,XM_011249217,XM_017314732	Dus1l	dihydrouridine synthase 1-like (S. cerevisiae)	protein_coding	NP_081100.2;XP_006534140.1;XP_011247515.1;XP_011247516.1;XP_011247517.1;XP_011247518.1;XP_011247519.1;XP_017170221.1	1915980	.	10.338818
68731	NM_199197	Rbfa	ribosome binding factor A	protein_coding	NP_954667.1	1915981	.	16.187027
68732	NM_001311122,NM_026825,XM_006516739,XM_006516741,XM_006516742,XM_011244333,XM_011244334,XM_011244335,XM_011244336,XM_011244337,XM_011244338,XM_011244340,XM_017315584,XM_017315585,XM_017315586,XM_030247393,XR_001780806,XR_001780807,XR_001780808,XR_001780810,XR_001780811,XR_001780812	Carmil1	capping protein regulator and myosin 1 linker 1	protein_coding	NP_001298051.1;NP_081101.3;XP_006516802.1;XP_006516804.1;XP_006516805.1;XP_011242635.1;XP_011242636.1;XP_011242637.1;XP_011242638.1;XP_011242639.1;XP_011242640.1;XP_011242642.1;XP_017171073.1;XP_017171074.1;XP_017171075.1;XP_030103253.1	1915982	.	15.225124
68734	NM_001160214,NM_211355,XM_017315173,XM_017315174,XR_001780474,XR_003950076	Ppp4r3a	protein phosphatase 4 regulatory subunit 3A	protein_coding	NP_001153686.1;NP_997594.1;XP_017170662.1;XP_017170663.1	1915984	.	14.898300
68735	NM_026826	Mrps18c	mitochondrial ribosomal protein S18C	protein_coding	NP_081102.1	1915985	.	20.181997
68736	NM_001037742,NM_001302962,NM_001302963,NM_001302964,XM_011238586,XM_011238587,XM_017322330	Tyw5	tRNA-yW synthesizing protein 5	protein_coding	NP_001032831.2;NP_001289891.1;NP_001289892.1;NP_001289893.1;XP_011236888.1;XP_011236889.1;XP_017177819.1	1915986	.	7.348742
68737	NM_144524,XM_006516199,XM_006516200,XM_011244169,XM_030246896	Angel1	angel homolog 1	protein_coding	NP_653107.2;XP_006516262.1;XP_006516263.1;XP_011242471.1;XP_030102756.1	1915987	.	3.431031
68738	NM_080575	Acss1	acyl-CoA synthetase short-chain family member 1	protein_coding	NP_542142.1	1915988	.	2.089612
68742	NM_026827,NM_028389,XM_006508155,XM_030242954	Tmem219	transmembrane protein 219	protein_coding	NP_081103.1;NP_082665.1;XP_006508218.1;XP_030098814.1	1915992	.	13.055650
68743	NM_028390,XM_006510581,XM_006510582,XM_011242594	Anln	anillin, actin binding protein	protein_coding	NP_082666.2;XP_006510644.1;XP_006510645.1;XP_011240896.1	1920174	.	9.375276
68744	NM_001289690,NM_001289691,NM_001289694,NM_001289695,NM_001358674,XM_006521331,XM_006521336,XM_011245721,XM_017316741,XM_030248732,XM_030248733,XM_030248734,XM_030248736,XM_030248737	Zfp740	zinc finger protein 740	protein_coding	NP_001276619.1;NP_001276620.1;NP_001276623.1;NP_001276624.1;NP_001345603.1;XP_006521394.1;XP_006521399.1;XP_011244023.1;XP_017172230.1;XP_030104592.1;XP_030104593.1;XP_030104594.1;XP_030104596.1;XP_030104597.1	1915994	.	26.408648
68750	NM_001039188,NM_001177868,NM_001177869,NM_026830,NR_033218,NR_033615,XM_006516749,XM_006516750,XM_006516751,XM_006516752,XM_006516753,XM_006516754,XM_006516756,XM_006516757,XM_006516758,XM_006516759,XM_011244341,XM_030247394,XM_030247395,XM_030247396,XM_030247397,XM_030247398	Rreb1	ras responsive element binding protein 1	protein_coding	NP_001034277.1;NP_001171339.1;NP_001171340.1;NP_081106.1;XP_006516812.1;XP_006516813.1;XP_006516814.1;XP_006516815.1;XP_006516816.1;XP_006516817.1;XP_006516819.1;XP_006516820.1;XP_006516821.1;XP_006516822.1;XP_011242643.1;XP_030103254.1;XP_030103255.1;XP_030103256.1;XP_030103257.1;XP_030103258.1	2443664	.	5.137585
68755	NM_001360454,NM_001360455,NM_026832	Cgrrf1	cell growth regulator with ring finger domain 1	protein_coding	NP_001347383.1;NP_001347384.1;NP_081108.2	1916368	.	10.356992
68758	NM_001190437,NM_145215,NR_033794,XM_006504492,XR_003955705	Abhd11	abhydrolase domain containing 11	protein_coding	NP_001177366.1;NP_660250.1;XP_006504555.1	1916008	.	15.215530
68760	NM_001310431,NM_001310432,NM_175132,XM_006519488,XM_006519490,XM_011245169	Synpo2l	synaptopodin 2-like	protein_coding	NP_001297360.1;NP_001297361.1;NP_780341.1;XP_006519551.1;XP_006519553.1;XP_011243471.1	1916010	.	3.003519
68763	NR_015536,NR_027943	1110038B12Rik	RIKEN cDNA 1110038B12 gene	lncRNA	.	1916013	.	45.788019
68764	NM_001024478,XM_006515190,XM_006515192,XM_011243893,XM_011243894,XR_381204,XR_872429	Cdhr3	cadherin-related family member 3	protein_coding	NP_001019649.1;XP_006515253.1;XP_006515255.1;XP_011242195.1;XP_011242196.1	1916014	.	0.062893
68767	NM_001037757,NM_026833,XM_006524859,XM_006524860,XM_030250021,XM_030250022,XM_030250023,XM_030250024	Washc1	WASH complex subunit 1	protein_coding	NP_001032846.1;NP_081109.1;XP_006524922.1;XP_006524923.1;XP_030105881.1;XP_030105882.1;XP_030105883.1;XP_030105884.1	1916017	.	12.497604
68770	NM_172992,XM_006535801,XM_030254785,XM_030254786,XR_003955706	Phtf2	putative homeodomain transcription factor 2	protein_coding	NP_766580.3;XP_006535864.1;XP_030110645.1;XP_030110646.1	1916020	.	14.317615
68774	NM_026835	Ms4a6d	membrane-spanning 4-domains, subfamily A, member 6D	protein_coding	NP_081111.1	1916024	.	6.173393
68775	NM_001159632,NM_133699,XM_006515196,XM_006515198,XM_006515199,XM_017315175,XM_017315176,XM_030246897	Atp6v1c2	ATPase, H+ transporting, lysosomal V1 subunit C2	protein_coding	NP_001153104.1;NP_598460.1;XP_006515259.1;XP_006515261.1;XP_006515262.1;XP_017170664.1;XP_017170665.1;XP_030102757.1	1916025	.	0.089042
68776	NM_026836,XM_006524862,XM_011246588,XM_030250026	Taf11	TATA-box binding protein associated factor 11	protein_coding	NP_081112.1;XP_006524925.1;XP_011244890.1;XP_030105886.1	1916026	.	6.119352
68777	NM_001285812,NM_001285814,NM_001285815,NM_001285816,NM_026837,NM_029589,XM_030253755	Tmem53	transmembrane protein 53	protein_coding	NP_001272741.1;NP_001272743.1;NP_001272744.1;NP_001272745.1;NP_081113.1;NP_083865.1;XP_030109615.1	1916027	.	4.540273
68778	NM_001347201,NM_175133,XM_006514034,XM_030245248,XM_030245249	Gucd1	guanylyl cyclase domain containing 1	protein_coding	NP_001334130.1;NP_780342.1;XP_006514097.2;XP_030101108.1;XP_030101109.1	1916028	.	10.664432
68789	NR_015549,NR_027952,NR_027953	Trmt61b	tRNA methyltransferase 61B	lncRNA	.	1916039	.	2.287096
68790	NR_045471,NR_130109	Fendrr	Foxf1 adjacent non-coding developmental regulatory RNA	lncRNA	.	1916040	.	0.090368
68792	NM_001083895,NM_026838,XM_006528606	Srpx2	sushi-repeat-containing protein, X-linked 2	protein_coding	NP_001077364.2;NP_081114.2;XP_006528669.1	1916042	.	39.853855
68794	NM_001081185,NM_001311074	Flnc	filamin C, gamma	protein_coding	NP_001074654.1;NP_001298003.1	95557	.	9.237688
68795	NM_001303033,NM_177783,XM_006500116,XM_006500117,XM_006500119,XM_006500120,XM_011239758,XR_003953713,XR_003953714,XR_374518	Ubr3	ubiquitin protein ligase E3 component n-recognin 3	protein_coding	NP_001289962.1;NP_808451.2;XP_006500179.1;XP_006500180.1;XP_006500182.1;XP_006500183.1;XP_011238060.1	1861100	.	14.553792
68796	NM_144525,XM_030254787	Tmem214	transmembrane protein 214	protein_coding	NP_653108.2;XP_030110647.1	1916046	.	37.503252
68797	NM_026840	Pdgfrl	platelet-derived growth factor receptor-like	protein_coding	NP_081116.3	1916047	.	68.080185
68799	NM_178615,XM_006524863,XM_006524864,XM_006524865	Rgmb	repulsive guidance molecule family member B	protein_coding	NP_848730.2;XP_006524926.1;XP_006524927.1;XP_006524928.1	1916049	.	4.327958
68800	NM_026841	Prr32	proline rich 32	protein_coding	NP_081117.1	1916050	.	4.884201
68801	NM_134255,XM_006511400,XM_011242807,XM_030244554	Elovl5	ELOVL family member 5, elongation of long chain fatty acids (yeast)	protein_coding	NP_599016.2;XP_006511463.1;XP_011241109.2;XP_030100414.1	1916051	.	28.979588
68802	NM_182992	Mypn	myopalladin	protein_coding	NP_892037.2	1916052	.	1.581959
68808	NR_040707	1110046J04Rik	RIKEN cDNA 1110046J04 gene	lncRNA		1916058	.	0.722512
68810	NM_001357206,NM_199465,XM_006501990,XM_006501991,XM_006501993,XM_006501996,XM_006501998,XM_006502000,XM_006502002,XM_011240214,XM_011240216,XM_030252783,XR_001783739	Nexn	nexilin	protein_coding	NP_001344135.1;NP_955759.2;XP_006502053.1;XP_006502054.1;XP_006502056.1;XP_006502059.1;XP_006502061.1;XP_006502063.1;XP_006502065.1;XP_011238516.1;XP_011238518.1;XP_030108643.1	1916060	.	6.394155
68813	NM_177780,XM_011245170,XM_017316175	Dock5	dedicator of cytokinesis 5	protein_coding	NP_808448.2;XP_011243472.1;XP_017171664.1	2652871	.	1.856714
68815	NM_133700,XM_006508159,XM_006508160,XM_006508161,XM_006508162,XM_006508163,XM_011241890	Btbd10	BTB (POZ) domain containing 10	protein_coding	NP_598461.1;XP_006508222.1;XP_006508223.1;XP_006508224.1;XP_006508225.1;XP_006508226.1;XP_011240192.1	1916065	.	6.803114
68816	NM_026845	Ppil1	peptidylprolyl isomerase (cyclophilin)-like 1	protein_coding	NP_081121.1	1916066	.	13.961588
68817	NM_001017966	Ddi2	DNA-damage inducible protein 2	protein_coding	NP_001017966.1	1917244	.	13.487472
68818	NM_001159905,NM_001159906,NM_026846	Zfand2b	zinc finger, AN1 type domain 2B	protein_coding	NP_001153377.1;NP_001153378.1;NP_081122.2	1916068	.	10.608192
68828	NM_023485,XM_006503373,XM_006503374,XM_011240608	Sync	syncoilin	protein_coding	NP_075974.3;XP_006503436.1;XP_006503437.1;XP_011238910.1	1916078	.	0.605453

68832	NM_001167767,NM_001167768,NM_001364260,NM_001364261,NM_172401,NR_157106,NR_157107,NR_157108,NR_157109,XM_006515201,XM_006515202,XM_006515203,XM_030246898	Ldah	lipid droplet associated hydrolase	protein_coding	[illegible]
68833	NM_026850	Pdcl3	phosducin-like 3	protein_coding	[illegible]
68836	NM_026851	Mrpl52	mitochondrial ribosomal protein L52	protein_coding	[illegible]
68837	NM_001080932,NM_001363033,XM_011249218,XM_011249219,XM_011249220,XM_011249221,XR_003949496,XR_003949497,XR_003949498,XR_879734	Foxk2	forkhead box K2	protein_coding	[illegible]
68839	NM_001358006,NM_001358007,NM_175134	Ankrd46	ankyrin repeat domain 46	protein_coding	[illegible]
68841	NR_037954	Lppos	LIM domain containing preferred translocation partner in lipoma, opposite strand	lncRNA	[illegible]
68842	NM_001103181,NM_054040,XM_006523248,XM_006523249,XM_006523251,XM_006523252,XM_030250027,XM_030250028,XM_030250029,XM_030250030,XM_030250031,XM_030250032,XM_030250033,XM_030250034,XM_030250035	Tulp4	tubby like protein 4	protein_coding	[illegible]
68845	NM_001278207,NM_001285904,NM_029406,XM_006541156,XM_017312277,XR_003946555,XR_003946556,XR_003946557	Pih1d1	PIH1 domain containing 1	protein_coding	[illegible]
68846	NM_176834	Rnf208	ring finger protein 208	protein_coding	[illegible]
68852	NM_001013019,NM_001360628	Lrrn4cl	LRRN4 C-terminal like	protein_coding	[illegible]
68854	NM_001313737,NM_026853,XM_006528971,XM_006528972,XM_006528974,XM_030251494	Asb11	ankyrin repeat and SOCS box-containing 11	protein_coding	[illegible]
68857	NM_001170960,NM_026854,XM_006526212,XM_006526213,XM_006526214,XR_003952540,XR_386011,XR_386012	Dtwd2	DTW domain containing 2	protein_coding	[illegible]
68859	NM_001163721,NM_001163722,NM_001357248,XM_006539148,XM_017320379	Smim1	small integral membrane protein 1	protein_coding	[illegible]
68861	NM_001033145	Dipk2a	divergent protein kinase domain 2A	protein_coding	[illegible]
68865	NM_001368372,NM_026855,XM_006531337,XM_011248496	Arv1	ARV1 homolog, fatty acid homeostasis modulator	protein_coding	[illegible]
68867	NM_001368373,NM_001368374,NM_001368375,NM_175136	Rnf122	ring finger protein 122	protein_coding	[illegible]
68870	NM_001033874,XM_006498287,XM_006498288,XM_006498289,XM_006498291,XM_006498292,XM_017319244,XM_017319245,XM_030252041,XM_030252042	Ak8	adenylate kinase 8	protein_coding	[illegible]
68874	NM_001033039	Klhdc9	kelch domain containing 9	protein_coding	[illegible]
68875	NM_001311107,NM_001311108,NM_178874,XM_006529852,XM_030242714	Tmcc2	transmembrane and coiled-coil domains 2	protein_coding	[illegible]
68876	NM_001159559,NM_001358969,NM_001358970,NM_026858,XR_003948790,XR_003948791	Atp23	ATP23 metallopeptidase and ATP synthase assembly factor homolog	protein_coding	[illegible]
68877	NM_001164607,NM_001164608,NM_026859	Maf1	MAF1 homolog, negative regulator of RNA polymerase III	protein_coding	[illegible]
68879	NM_133701	Prpf6	pre-mRNA splicing factor 6	protein_coding	[illegible]
68880	NM_001195435,XM_006517360	Fam240b	family with sequence similarity 240 member B	protein_coding	[illegible]
68888	NM_026860	Gkn3	gastrokine 3	protein_coding	[illegible]
68889	NM_026861,XM_006519492,XM_030247988	Ubac2	ubiquitin associated domain containing 2	protein_coding	[illegible]
68891	NM_026862,XM_011250688,XM_011250689,XM_017312278	Cd177	CD177 antigen	protein_coding	[illegible]
68895	NM_026864	Rasl11a	RAS-like, family 11, member A	protein_coding	[illegible]
68897	NM_001278218,NM_001278219,NM_001278220,NM_026866,XM_006535596	Disp1	dispatched RND transporter family member 1	protein_coding	[illegible]
68904	NM_001081119,NM_026868	Abhd13	abhydrolase domain containing 13	protein_coding	[illegible]
68910	NM_001085415,NM_001085416,NM_001085417,NM_001355746,NM_001355747,NM_020589,XM_006506544,XM_006506545,XM_006506546,XM_006506547,XM_006506548,XM_006506549,XM_006506550,XM_011241450,XM_030255577,XM_030255578,XM_030255579,XM_030255580,XM_030255581,XM_030255582	Zfp467	zinc finger protein 467	protein_coding	[illegible]
68911	NM_001293763,NM_001293766,NM_001293767,NM_001293768,NM_026869,XM_030252784,XM_030252785	Pygo2	pygopus 2	protein_coding	[illegible]
68915	NM_175137,XM_011246589,XM_030250036,XR_003952174,XR_003952175	Vars2	valyl-tRNA synthetase 2, mitochondrial	protein_coding	[illegible]
68916	NM_001308486,NM_144536,NR_131896,XM_006516762,XM_006516763,XM_006516764,XM_011244342,XM_017315589,XM_017315	Cdkal1	CDK5 regulatory subunit associated protein 1-like 1	protein_coding	[illegible]
68917	NM_026871	Hint2	histidine triad nucleotide binding protein 2	protein_coding	[illegible]
68918	NM_197988	1190005I06Rik	RIKEN cDNA 1190005I06 gene	protein_coding	[illegible]
68920	NM_001142727,NM_001347196,XM_011240609	1110065P20Rik	RIKEN cDNA 1110065P20 gene	protein_coding	[illegible]
68922	NM_175138,XR_003955007,XR_390339,XR_390340	Dnaic1	dynein, axonemal, intermediate chain 1	protein_coding	[illegible]
68925	NM_001163701,NM_177294,XM_006500121,XM_011239759,XR_001783174,XR_374519	Rpap1	RNA polymerase II associated protein 1	protein_coding	[illegible]
68926	NM_026872,XM_006538223,XM_006538224,XM_006538225,XM_006538226,XM_011250099,XM_030253756,XM_030253757,XM_030253758	Ubap2	ubiquitin-associated protein 2	protein_coding	[illegible]
68927	NM_026873	Ptcd2	pentatricopeptide repeat domain 2	protein_coding	[illegible]
68929	NM_001254762,NM_030037,XM_006504612,XM_006504613,XM_006504614	Mospd3	motile sperm domain containing 3	protein_coding	[illegible]
68936	NM_138743,XM_030249260	Smim11	small integral membrane protein 11	protein_coding	[illegible]
68938	NM_001164224,NM_001363055,NM_001363056,NM_026877,NM_198223,XM_006534086,XM_006534087,XM_030246265,XM_030246266	Aspscr1	alveolar soft part sarcoma chromosome region, candidate 1 (human)	protein_coding	[illegible]
68939	NM_026878	Rasl11b	RAS-like, family 11, member B	protein_coding	[illegible]
68941	XR_001783557,XR_001783559,XR_001783560,XR_001783561,XR_001783562,XR_001783563,XR_001783564,XR_001783565,XR_003954096,XR_003954097	1110018N20Rik	RIKEN cDNA 1110018N20 gene, transcript variant X10	lncRNA	[illegible]
68942	NM_026879	Chmp2b	charged multivesicular body protein 2B	protein_coding	[illegible]
68943	NM_026880	Pink1	PTEN induced putative kinase 1	protein_coding	[illegible]
68944	NM_001039483	Tmco1	transmembrane and coiled-coil domains 1	protein_coding	[illegible]
68947	NM_175140,XM_006540337,XM_006540339,XM_006540340,XM_006540341,XM_030242955	Chst8	carbohydrate sulfotransferase 8	protein_coding	[illegible]
68948	NM_026883	Fam216a	family with sequence similarity 216, member A	protein_coding	[illegible]
68949	NR_138563	Zfas1	zinc finger, NFX1-type containing 1, antisense RNA 1	lncRNA	[illegible]
68952	NM_001146347,NM_026884,NM_029978,XM_006508164,XM_006508166	Fam57b	family with sequence similarity 57, member B	protein_coding	[illegible]
68953	NM_001360730,NM_001360731,NM_001360733,NM_026885,NR_153796	Chmp2a	charged multivesicular body protein 2A	protein_coding	[illegible]
68955	NM_026886,XM_006530456,XM_011248239	Srrm4	serine/arginine repetitive matrix 4	protein_coding	[illegible]
68957	NM_001310597,NM_198410,XM_006502007,XM_006502009,XM_006502010,XM_030252786,XM_030252787,XM_030252788,XM_030252789	Paqr6	progestin and adipoQ receptor family member VI	protein_coding	[illegible]
68961	NM_001360741,NM_026888,XM_006508167,XM_006508168,XM_006508169,XM_006508170,XM_030242956,XM_030242957,XM_030242958,XM_030242959,XM_030242960	Phkg2	phosphorylase kinase, gamma 2 (testis)	protein_coding	[illegible]
68964	NM_001013256,NM_001143790,NM_001281464,NM_001281465,XM_006534088,XM_006534089,XM_006534092,XM_006534093,XM_030246267,XM_030246268,XR_003949499,XR_388517	Ctc1	CTS telomere maintenance complex component 1	protein_coding	[illegible]
68966	NM_026890	Ngdn	neuroguidin, EIF4E binding protein	protein_coding	[illegible]
68968	NM_026891	Cdan1	congenital dyserythropoietic anemia, type I (human)	protein_coding	[illegible]
68969	NM_026892	Eif1b	eukaryotic translation initiation factor 1B	protein_coding	[illegible]
68970	NM_026893,XM_006538228,XM_006538229,XM_006538230,XM_017320381	Dcaf12	DDB1 and CUL4 associated factor 12	protein_coding	[illegible]
68971	NM_026894,XM_006506552,XM_006506553,XR_377489	Tamm41	TAM41 mitochondrial translocator assembly and maintenance homolog	protein_coding	[illegible]
68972	NM_001163421,NM_001163422,NM_001357166,NM_026895,XM_006497204	Tatdn3	TatD DNase domain containing 3	protein_coding	[illegible]
68975	NM_001290489,NM_001290490,NM_026896,NM_029615,XM_017319246,XM_017319247	Med27	mediator complex subunit 27	protein_coding	[illegible]
68977	NM_001271433,NM_001271434,NM_001271435,NM_001271436,NM_001271437,NM_026897,XM_006524867	Haghl	hydroxyacylglutathione hydrolase-like	protein_coding	[illegible]
68979	NM_001161329,NM_001363078,NM_133702,NR_156439	Nol11	nucleolar protein 11	protein_coding	[illegible]
68980	NM_001185162,NM_026898,XM_006522509,XM_006522510	Wdr53	WD repeat domain 53	protein_coding	[illegible]
68981	NM_021336,NR_152094	Snrpa1	small nuclear ribonucleoprotein polypeptide A'	protein_coding	[illegible]
68982	NR_029432	1500015A07Rik	RIKEN cDNA 1500015A07 gene	lncRNA	[illegible]
68986	NR_040496	Gad1os	glutamate decarboxylase 1, opposite strand	lncRNA	[illegible]
68988	NM_001159714,NM_027328	Prpf31	pre-mRNA processing factor 31	protein_coding	[illegible]
68991	NM_026899	Ssu72	Ssu72 RNA polymerase II CTD phosphatase homolog (yeast)	protein_coding	[illegible]
68992	NM_026900,XM_030242961,XM_030242962	Zfp580	zinc finger protein 580	protein_coding	[illegible]
68995	NM_001356359,NM_001356360,NM_001361381,NM_001361382,NM_026902,NR_154890	Mcts1	malignant T cell amplified sequence 1	protein_coding	[illegible]
68999	NM_001357234,NM_001357235,NM_001357236,NM_001357237,NM_026904,XM_006531342,XM_030243749,XM_030243750,XM_030243751	Anapc10	anaphase promoting complex subunit 10	protein_coding	[illegible]
69002	NR_130956	1500026H17Rik	RIKEN cDNA 1500026H17 gene	lncRNA	[illegible]
69004	NR_045821	6330418K02Rik	RIKEN cDNA 6330418K02 gene	lncRNA	[illegible]
69008	NM_001360591,NM_001360592,NM_001360593,NM_026908,XM_017316177	Cab39l	calcium binding protein 39-like	protein_coding	[illegible]
69009	NM_026909,XM_006522511,XM_006522513,XM_011246003,XM_030249261	Thap7	THAP domain containing 7	protein_coding	[illegible]
69010	NM_181394,XM_006511806,XM_006511807	Anapc13	anaphase promoting complex subunit 13	protein_coding	[illegible]
69017	NM_001102563,XM_017312280,XM_030242963,XM_030242964	Prrt2	proline-rich transmembrane protein 2	protein_coding	[illegible]
69019	NM_026911	Spcs1	signal peptidase complex subunit 1 homolog (S. cerevisiae)	protein_coding	[illegible]
69020	NM_001081065,NM_001347040,NM_001361464,NM_001361465,XM_006521341	Zfp707	zinc finger protein 707	protein_coding	[illegible]
69024	NM_026912,XM_006531831,XM_011248650,XM_011248651	Snx15	sorting nexin 15	protein_coding	[illegible]
69028	NM_001356262,NM_026913,XM_017322349,XR_003946501	Mitd1	MIT, microtubule interacting and transport, domain containing 1	protein_coding	[illegible]
69029	NM_026914	Smdt1	single-pass membrane protein with aspartate rich tail 1	protein_coding	[illegible]
69030	NR_040439	1810006J02Rik	RIKEN cDNA 1810006J02 gene	lncRNA	[illegible]
69032	NM_001357346,NM_026915,XM_006512248,XM_017313557	Lyzl4	lysozyme-like 4	protein_coding	[illegible]
69034	NM_001359308,NM_026916	Nupr1l	nuclear protein transcriptional regulator 1 like	protein_coding	[illegible]
69035	NM_026917,XM_006512249,XM_006512250,XM_006512251,XM_006512252,XM_006512255,XM_030244555,XM_030244556,XM_030244557	Zdhhc3	zinc finger, DHHC domain containing 3	protein_coding	[illegible]
69036	NM_026918	Zg16	zymogen granule protein 16	protein_coding	[illegible]
69038	NM_001163431,NM_026919	Tmem258	transmembrane protein 258	protein_coding	[illegible]
69042	XR_003954596,XR_376286,XR_867796,XR_867797	1810013D15Rik	RIKEN cDNA 1810013D15 gene, transcript variant X1	lncRNA	[illegible]
69046	NM_026921	Isca1	iron-sulfur cluster assembly 1	protein_coding	[illegible]
69047	NM_026922,XM_006531344	Atp2c2	ATPase, Ca++ transporting, type 2C, member 2	protein_coding	[illegible]
69048	NM_022885,XM_011244695,XR_382742	Slc30a5	solute carrier family 30 (zinc transporter), member 5	protein_coding	[illegible]
69049	NM_023493	Nat8f5	N-acetyltransferase 8 (GCN5-related) family member 5	protein_coding	[illegible]
69050	NR_045427	1810013A23Rik	RIKEN cDNA 1810013A23 gene	lncRNA	[illegible]
69051	NM_133705	Pycr2	pyrroline-5-carboxylate reductase family, member 2	protein_coding	[illegible]
69053	NM_001081400	1810013L24Rik	RIKEN cDNA 1810013L24 gene	protein_coding	[illegible]
69064	NM_001286217,NM_001286218,NM_026928,NR_104415,XM_011249855,XM_030242965,XR_003946558	Fuom	fucose mutarotase	protein_coding	[illegible]
69065	NM_026929	Chac1	ChaC, cation transport regulator 1	protein_coding	[illegible]
69066	NM_001163473,XM_017314735,XM_017314737,XM_017314738,XM_017314739,XM_017314740,XM_030246269,XM_030246270,XM_030246271,XM_030246272	1810010H24Rik	RIKEN cDNA 1810010H24 gene	protein_coding	[illegible]
69068	NM_026931	Tcim	transcriptional and immune response regulator	protein_coding	[illegible]
69069	NM_001163616,XR_383209	Tmem273	transmembrane protein 273	protein_coding	[illegible]
69071	NM_133706	Tmem97	transmembrane protein 97	protein_coding	[illegible]
69072	NM_026932	Ebna1bp2	EBNA1 binding protein 2	protein_coding	[illegible]
69073	NM_001083916,NM_133707,XM_006539150,XM_006539151	Kdf1	keratinocyte differentiation factor 1	protein_coding	[illegible]
69076	NM_026933	Triap1	TP53 regulated inhibitor of apoptosis 1	protein_coding	[illegible]
69077	NM_178616,XM_011249223	Psmd11	proteasome (prosome, macropain) 26S subunit, non-ATPase, 11	protein_coding	[illegible]
69080	NM_001357169,NM_001357170,NM_001357171,NM_133708,XM_006496531,XM_006496532,XM_006496535,XM_006496538,XM_011238708,XM_030242740,XM_030242742,XM_030242753,XM_030242756	Gmppa	GDP-mannose pyrophosphorylase A	protein_coding	[illegible]
69082	NM_026934	Zc3h15	zinc finger CCCH-type containing 15	protein_coding	[illegible]
69085	NM_145453,XM_006517361,XM_017315591,XR_003950474	Zcchc9	zinc finger, CCHC domain containing 9	protein_coding	[illegible]
69086	XR_391164	1810019N24Rik	RIKEN cDNA 1810019N24 gene	lncRNA	[illegible]
69089	NM_026936	Oxa1l	oxidase assembly 1-like	protein_coding	[illegible]
69090	NM_001199187,NM_026937,XM_006514039,XM_006514040,XM_006514041,XM_006514042,XM_017314074,XM_017314075,XM_030245250	Ascc1	activating signal cointegrator 1 complex subunit 1	protein_coding	[illegible]
69091	NM_178027	Vps26b	VPS26 retromer complex component B	protein_coding	[illegible]
69094	NM_026938,XM_030242966	Tmem160	transmembrane protein 160	protein_coding	[illegible]
69097	NM_001024134,NM_001177872,XR_385361,XR_876527	Trim15	tripartite motif-containing 15	protein_coding	[illegible]
69101	NM_026940,XM_006522515	Ydjc	YdjC homolog (bacterial)	protein_coding	[illegible]
69104	NM_001164336,NM_001164337,NM_027314,XM_006527322	March5	membrane-associated ring finger (C3HC4) 5	protein_coding	[illegible]
69106	NM_026942,NR_028146,XM_006511402,XM_006511404,XM_030244558	Stoml1	stomatin-like 1	protein_coding	[illegible]
69108	NR_045473	1810012K16Rik	RIKEN cDNA 1810012K16 gene	lncRNA	[illegible]
69109	NM_197989	Ccnq	cyclin Q	protein_coding	[illegible]
69113	NM_026944,XM_006500123,XM_006500125,XM_006500126,XM_030252043	Alkbh3	alkB homolog 3, alpha-ketoglutarate-dependent dioxygenase	protein_coding	[illegible]
69116	NM_001160319	Ubr4	ubiquitin protein ligase E3 component n-recognin 4	protein_coding	[illegible]
69120	NR_040417	1810021B22Rik	RIKEN cDNA 1810021B22 gene	lncRNA	[illegible]
69121	NM_001291320,NM_133709,XM_006508177,XM_006508178	Chrdl2	chordin-like 2	protein_coding	[illegible]
69123	NM_026947,XM_006516765,XM_011244343	Eci3	enoyl-Coenzyme A delta isomerase 3	protein_coding	[illegible]
69125	NM_001356477,NM_001363084,NM_001363085,NM_001363086,NM_026949,XM_006534102	Cnot8	CCR4-NOT transcription complex, subunit 8	protein_coding	[illegible]
69126	NM_001099674,NM_001347206,XM_017319707	Rbis	ribosomal biogenesis factor	protein_coding	[illegible]
69129	NM_001368376,NM_001368377,NM_001368378,NM_026951,NM_028594	Pex11g	peroxisomal biogenesis factor 11 gamma	protein_coding	[illegible]
69131	NM_001109626,NM_001109628,NM_026952,XM_006534108,XM_011249224,XM_011249225,XM_030246273,XM_030246274,XM_030246275,XM_030246276,XM_030246277	Cdk12	cyclin-dependent kinase 12	protein_coding	[illegible]
69134	NM_183278,XM_030247990	Fam25c	family with sequence similarity 25, member C	protein_coding	[illegible]
69136	NM_026954	Tusc1	tumor suppressor candidate 1	protein_coding	[illegible]
69137	NM_026955,XM_011242595,XM_011242596,XR_379133,XR_87053	Vstm5	V-set and transmembrane domain containing 5	protein_coding	[illegible]
69142	NM_026956,XM_006508867,XM_006508868,XM_017312956,XR_378666	Cd209f	CD209f antigen	protein_coding	[illegible]
69146	NM_026960,XM_006521342,XM_006521343,XR_384037	Gsdmd	gasdermin D	protein_coding	[illegible]
69147	NM_026961,XM_017312281,XM_017312282,XM_017312283	2200002J24Rik	RIKEN cDNA 2200002J24 gene	protein_coding	[illegible]
69149	NM_001164574,NM_026962,XM_011242367,XM_011242368,XM_030244559	Kbtbd3	kelch repeat and BTB (POZ) domain containing 3	protein_coding	[illegible]
69150	NM_080557	Snx4	sorting nexin 4	protein_coding	[illegible]
69151	NM_001356322,NM_026963,XM_006539152,XM_017320382,XM_030253759,XM_030253760,XM_030253761,XM_030253762,XM_030253763	Lzic	leucine zipper and CTNNBIP1 domain containing	protein_coding	[illegible]
69155	NM_175141,XM_006527698,XM_011247495	1810030O07Rik	RIKEN cDNA 1810030O07 gene	protein_coding	[illegible]
69156	NM_026965	Comtd1	catechol-O-methyltransferase domain containing 1	protein_coding	[illegible]
69159	NM_026967,XM_006521345,XM_030248739	Rhebl1	Ras homolog enriched in brain like 1	protein_coding	[illegible]
69161	NM_026968,XM_006500128	Manbal	mannosidase, beta A, lysosomal-like	protein_coding	[illegible]
69162	NM_001359323,NM_001359332,NM_001359344,NM_001359348,NM_001359349,NM_001359350,NM_026969,XM_006535192,XM_006535193,XM_006535194,XM_006535207,XM_011249551,XM_011249552,XM_011249553,XM_011249555,XM_011249558,XM_011249559,XM_011249560,XM_011249561,XM_011249562,XM_011249563	Sec31a	Sec31 homolog A (S. cerevisiae)	protein_coding	[illegible]
69163	NM_001081210	Mrpl44	mitochondrial ribosomal protein L44	protein_coding	[illegible]
69165	NM_001037800,NM_001287211,NM_026972,NR_104430,XM_006508870	Cd209b	CD209b antigen	protein_coding	[illegible]
69168	NM_026975,XM_006502012	Bola1	bolA-like 1 (E. coli)	protein_coding	[illegible]
69169	NM_026976	Fcmr	Fc fragment of IgM receptor	protein_coding	[illegible]
69170	NR_037569	1810026B05Rik	RIKEN cDNA 1810026B05 gene	lncRNA	[illegible]
69171	NM_001356264,NM_026977,XM_006496541,XM_006496542	Cnppd1	cyclin Pas1/PHO80 domain containing 1	protein_coding	[illegible]
69178	NM_001199188,NM_024225	Snx5	sorting nexin 5	protein_coding	[illegible]
69179	NM_028839,XM_030247992	Stimate	STIM activating enhancer	protein_coding	[illegible]
69181	NM_001014390,XM_006514046,XM_011243552	Dyrk2	dual-specificity tyrosine-(Y)-phosphorylation regulated kinase 2	protein_coding	[illegible]
69183	NM_026979	C1qtnf2	C1q and tumor necrosis factor related protein 2	protein_coding	[illegible]
69185	NM_001355583,NM_026981,XM_006500130,XM_006500131,XM_006500132	Dtwd1	DTW domain containing 1	protein_coding	[illegible]
69186	NM_026982	Tmem256	transmembrane protein 256	protein_coding	[illegible]
69187	NM_026983,XM_006506554,XM_006506555	Erp27	endoplasmic reticulum protein 27	protein_coding	[illegible]
69188	NM_026984,XM_006535805,XM_006535806,XM_006535807,XM_017321090,XR_003955707,XR_389753	Kmt2e	lysine (K)-specific methyltransferase 2E	protein_coding	[illegible]
69189	NM_026985	Mcemp1	mast cell expressed membrane protein 1	protein_coding	[illegible]
69190	NM_027727,XM_006526216,XM_006526218,XM_030250593,XM_030250594,XR_386014	Dym	dymeclin	protein_coding	[illegible]
69191	NM_001081070,XM_006524872	Pdia2	protein disulfide isomerase associated 2	protein_coding	[illegible]
69192	NM_026987,XM_006524873,XR_001782109,XR_003952176,XR_876528	Dhx16	DEAH (Asp-Glu-Ala-His) box polypeptide 16	protein_coding	[illegible]
69195	NM_153776,XM_006516201	Tmem121	transmembrane protein 121	protein_coding	[illegible]
69202	NM_026988	Ptms	parathymosin	protein_coding	[illegible]
69206	NR_033207	2010016I18Rik	RIKEN cDNA 2010016I18 gene	lncRNA	[illegible]

69207	NM_001093752,NM_001093753,NM_001356997,XM_006502015,XM_006502016,XM_006502020,XM_006502022,XM_011240219,XM_017319708,XM_030252790,XM_030252791,XM_030252792,XM_030252793,XM_030252794,XM_030252795,XM_030252796,XM_030252797,XM_030252798,XM_030252799,XM_030252800,XM_030252801,XM_030252802,XR_001783740,XR_001783741,XR_375573,XR_375574	Srsf11	serine/arginine-rich splicing factor 11	protein_coding	NP_001087221.1;NP_001087222.1;NP_001343926.1;XP_006502078.1;XP_006502079.1;XP_006502083.1;XP_006502085.1;XP_011238521.1;XP_017175197.1;XP_030108650.1;XP_030108651.1;XP_030108652.1;XP_030108653.1;XP_030108654.1;XP_030108655.1;XP_030108656.1;XP_030108657.1;XP_030108658.1;XP_030108659.1;XP_030108660.1;XP_030108661.1;XP_030108662.1	1916457	83.196672	[illegible]
69211	NR_045474	2310081J21Rik	RIKEN cDNA 2310081J21 gene	lncRNA	.	1916461	0.211890	[illegible]
69215	NM_001356468,NM_001356469,NM_026991,XM_006534110,XM_006534111	Sat2	spermidine/spermine N1-acetyl transferase 2	protein_coding	NP_001343397.1;NP_001343398.1;NP_081267.1;XP_006534173.1;XP_006534174.1	1916465	13.607622	[illegible]
69216	NM_001038998,NM_024462	Svbp	small vasohibin binding protein	protein_coding	NP_001034087.1;NP_077782.1	1916466	34.936935	[illegible]
69217	NM_148927,XM_006541158,XM_006541160,XM_006541162,XM_006541163,XM_006541164,XM_006541165,XM_006541166,XM_006541169,XM_017312284,XM_030242967	Plekha4	pleckstrin homology domain containing, family A (phosphoinositide binding specific) member 4	protein_coding	NP_683729.2;XP_006541221.1;XP_006541223.1;XP_006541225.1;XP_006541226.1;XP_006541227.1;XP_006541228.1;XP_006541229.1;XP_006541232.1;XP_017167773.1;XP_030098827.1	1916467	3.277747	[illegible]
69219	NM_026993,XM_011240221,XM_017319712,XM_030252803	Ddah1	dimethylarginine dimethylaminohydrolase 1	protein_coding	NP_081269.1;XP_011238523.1;XP_017175201.1;XP_030108663.1	1916469	4.887532	[illegible]
69221	NR_030738	2410006H16Rik	RIKEN cDNA 2410006H16 gene	lncRNA	.	1916471	83.235756	[illegible]
69225	NM_001190357,NM_001293661,NM_026995,XM_006508872	Naxd	NAD(P)HX dehydratase	protein_coding	NP_001177286.1;NP_001280590.1;NP_081271.2;XP_006508935.1	1913353	19.174595	[illegible]
69226	NM_001370761,NM_029394,XM_006526221,XM_011246995	Snx24	sorting nexing 24	protein_coding	NP_001357690.1;NP_083670.1;XP_006526284.1;XP_011245297.1	1916476	12.257107	[illegible]
69227	NM_001040396	Selenot	selenoprotein T	protein_coding	NP_001035486.2	1916477	23.212830	[illegible]
69228	NM_001163475,NM_001347142,NM_001362062,XM_006506556,XM_006506557,XM_006506559,XM_011241454,XM_011241455,XM_011241456,XM_011241457,XM_017321730,XM_030255584,XM_030255585	Zfp746	zinc finger protein 746	protein_coding	NP_001156947.1;NP_001334071.1;NP_001348991.1;XP_006506619.1;XP_006506620.1;XP_006506622.1;XP_011239756.1;XP_011239757.1;XP_011239758.1;XP_011239759.1;XP_017177219.1;XP_030111444.1;XP_030111445.1	1916478	7.378403	[illegible]
69232	NM_001114119,NM_175143,XM_006511808,XM_006511809,XM_006511810,XM_006511811,XM_006511812,XM_006511813,XM_006511814,XM_006511818,XM_017313560,XM_017313561,XM_030244560,XM_030244561	Qrich1	glutamine-rich 1	protein_coding	NP_001107591.1;NP_780352.2;XP_006511871.1;XP_006511872.1;XP_006511873.1;XP_006511874.1;XP_006511875.1;XP_006511876.1;XP_006511877.1;XP_006511881.1;XP_017169049.1;XP_017169050.1;XP_030100420.1;XP_030100421.1	1916482	24.750685	[illegible]
69234	NM_026999,XM_006508179,XM_006508180,XM_011241892,XM_017312285	Zfp688	zinc finger protein 688	protein_coding	NP_081275.3;XP_006508242.1;XP_006508243.1;XP_011240194.1;XP_017167774.1	1916484	3.941012	[illegible]
69237	NM_027000,XM_017315592	Gtpbp4	GTP binding protein 4	protein_coding	NP_081276.2;XP_017171081.1	1916487	12.984298	[illegible]
69239	NM_027001	Pdzph1	PDZ and pleckstrin homology domains 1	protein_coding	NP_081277.1	1916489	0.000000	[illegible]
69241	NM_001357480,NM_027002	Polr2d	polymerase (RNA) II (DNA directed) polypeptide D	protein_coding	NP_001344409.1;NP_081278.1	1916491	20.719067	[illegible]
69248	NR_045046	2610035F20Rik	RIKEN cDNA 2610035F20 gene	lncRNA	.	1916498	1.557701	[illegible]
69253	NM_001164708,NM_024441	Hspb2	heat shock protein 2	protein_coding	NP_001158180.1;NP_077761.3	1916503	5.509857	[illegible]
69256	NM_027007,XM_011246996,XM_011246997	Zfp397	zinc finger protein 397	protein_coding	NP_081283.2;XP_011245298.1;XP_011245299.1	1916506	10.002423	[illegible]
69257	NM_001291059,NM_001291062,NM_001291063,NM_001356358,NM_023502,XM_006502031,XM_006502034,XM_017319714,XM_030252804,XM_030252805,XM_030252806,XM_030252807	Elf2	E74-like factor 2	protein_coding	[illegible]	[illegible]	19.209785	[illegible]
69259	NM_027008	Kctd5	potassium channel tetramerisation domain containing 5	protein_coding	NP_081284.2	[illegible]	[illegible]	[illegible]
69260	NM_023503	Ing2	inhibitor of growth family, member 2	protein_coding	NP_075992.2	[illegible]	[illegible]	[illegible]
69263	NM_027009,XM_030254788,XM_030254789	Rfc3	replication factor C (activator 1) 3	protein_coding	NP_081285.1;XP_030110648.1;XP_030110649.1	[illegible]	[illegible]	[illegible]
69269	NM_001163573,NM_027013,XM_017319715,XM_017319716	Scnm1	sodium channel modifier 1	protein_coding	[illegible]	[illegible]	[illegible]	[illegible]
69270	NM_001163476,NM_027014,XM_006500133	Gins1	GINS complex subunit 1 (Psf1 homolog)	protein_coding	NP_001156948.1;NP_081290.1;XP_006500196.1	[illegible]	[illegible]	[illegible]
69274	NM_133710,XM_006512262,XM_006512263	Ctdspl	CTD (carboxy-terminal domain, RNA polymerase II, polypeptide A) small phosphatase-like	protein_coding	NP_598471.3;XP_006512325.1;XP_006512326.1	[illegible]	[illegible]	[illegible]
69276	NM_001357528,NM_027016	Sec62	SEC62 homolog (S. cerevisiae)	protein_coding	NP_001344457.1;NP_081292.1	[illegible]	[illegible]	[illegible]
69277	NM_027017,XM_006500134,XM_011239763,XM_011239764,XM_011239765,XM_030252044,XM_030252045,XM_030252046,XM_030252047,XM_030252049	3300002I08Rik	RIKEN cDNA 3300002I08 gene	protein_coding	[illegible]	[illegible]	[illegible]	[illegible]
69281	NM_133711	Spata4	spermatogenesis associated 4	protein_coding	NP_598472.2	[illegible]	[illegible]	[illegible]
69282	NM_001008547,XM_030254790,XM_030254791	1700001J03Rik	RIKEN cDNA 1700001J03 gene	protein_coding	NP_001008547.1;XP_030110650.1;XP_030110651.1	[illegible]	[illegible]	[illegible]
69288	NM_001081347,NM_001252636,NM_001252637,NM_001252638,XM_006514048,XM_006514049,XM_006514051,XM_006514052,XM_006514053,XM_006514054,XM_011243553	Rhobtb1	Rho-related BTB domain containing 1	protein_coding	[illegible]	[illegible]	[illegible]	[illegible]
69291	NR_027980	1700001L05Rik	RIKEN cDNA 1700001L05 gene	lncRNA	.	[illegible]	[illegible]	[illegible]
69296	NM_001174169,NM_027025	Tmigd3	transmembrane and immunoglobulin domain containing 3	protein_coding	NP_001167640.1;NP_081301.2	[illegible]	[illegible]	[illegible]
69297	NM_027026	Lrrc46	leucine rich repeat containing 46	protein_coding	NP_081302.2	[illegible]	[illegible]	[illegible]
69299	NM_027027	Asb9	ankyrin repeat and SOCS box-containing 9	protein_coding	NP_081303.1	[illegible]	[illegible]	[illegible]
69301	NM_001163810	Tescl	tescalcin-like	protein_coding	NP_001157282.1	[illegible]	[illegible]	[illegible]
69303	NR_038077	1700001G11Rik	RIKEN cDNA 1700001G11 gene	lncRNA	.	[illegible]	[illegible]	[illegible]
69305	NM_027030	Dcps	decapping enzyme, scavenger	protein_coding	NP_081306.1	[illegible]	[illegible]	[illegible]
69306	NM_027031,XM_011243747	Efcab9	EF-hand calcium binding domain 9	protein_coding	NP_081307.2;XP_011242049.1	[illegible]	[illegible]	[illegible]
69307	NM_153390	Pxt1	peroxisomal, testis specific 1	protein_coding	NP_700439.1	[illegible]	[illegible]	[illegible]
69308	NR_040554	1700007P06Rik	RIKEN cDNA 1700007P06 gene	lncRNA	.	[illegible]	[illegible]	[illegible]
69309	NM_172371,XM_006534113	Slc16a13	solute carrier family 16 (monocarboxylic acid transporters), member 13	protein_coding	NP_758959.1;XP_006534176.1	[illegible]	[illegible]	[illegible]
69310	NM_027032,XM_006523353	Pacrg	PARK2 co-regulated	protein_coding	NP_081308.1;XP_006523416.1	[illegible]	[illegible]	[illegible]
69312	NM_145692,NR_110971,XM_006495565,XM_006495566,XR_003946510	Ppp1r42	protein phosphatase 1, regulatory subunit 42	protein_coding	NP_663730.1;XP_006495628.1;XP_006495629.1	[illegible]	[illegible]	[illegible]
69315	NM_027035	1700001L19Rik	RIKEN cDNA 1700001L19 gene	protein_coding	NP_081311.1	[illegible]	[illegible]	[illegible]
69318	NM_027037,XM_006508181	Fam24b	family with sequence similarity 24 member B	protein_coding	NP_081313.1;XP_006508244.2	[illegible]	[illegible]	[illegible]
69320	NR_045476	1700007J10Rik	RIKEN cDNA 1700007J10 gene	lncRNA	.	[illegible]	[illegible]	[illegible]
69324	NM_001162428,NM_027038,XM_006534114	1700012B07Rik	RIKEN cDNA 1700012B07 gene	protein_coding	NP_001155900.1;NP_081314.1;XP_006534177.1	[illegible]	[illegible]	[illegible]
69325	NM_029306,XR_379134	1700012B09Rik	RIKEN cDNA 1700012B09 gene	protein_coding	NP_083582.2	[illegible]	[illegible]	[illegible]
69327	NM_027040	1700007K13Rik	RIKEN cDNA 1700007K13 gene	protein_coding	NP_081316.1	[illegible]	[illegible]	[illegible]
69329	NM_001347062,NM_027041,XM_006538232,XM_006538233	Cfap206	cilia and flagella associated protein 206	protein_coding	[illegible]	[illegible]	[illegible]	[illegible]
69337	NR_131049	1700012C14Rik	RIKEN cDNA 1700012C14 gene	lncRNA	.	[illegible]	[illegible]	[illegible]
69339	NM_027046	Ccdc54	coiled-coil domain containing 54	protein_coding	NP_081322.1	[illegible]	[illegible]	[illegible]
69349	NM_027049,XM_006541171,XM_006541172,XM_011250899,XM_011250900,XM_017312286,XM_030242968,XM_030242969	1700008O03Rik	RIKEN cDNA 1700008O03 gene	protein_coding	[illegible]	[illegible]	[illegible]	[illegible]
69350	NR_029433	1700003G18Rik	RIKEN cDNA 1700003G18 gene	lncRNA	.	[illegible]	[illegible]	[illegible]
69352	NM_178617,XM_006538234	Necab1	N-terminal EF-hand calcium binding protein 1	protein_coding	NP_848732.3;XP_006538297.1	[illegible]	[illegible]	[illegible]
69354	NM_001358058,NM_001358059,NM_001358060,NM_027052,XM_006521350,XM_006521351	Slc38a4	solute carrier family 38, member 4	protein_coding	[illegible]	[illegible]	[illegible]	[illegible]
69358	NM_001162973,NM_001162974,NM_001357584,NM_027053,XM_006508183,XM_011241893,XM_011241894,XM_011241895,XM_011241896,XM_030242970	Lrrc51	leucine rich repeat containing 51	protein_coding	[illegible]	[illegible]	[illegible]	[illegible]
69367	NM_001038592,NM_001038593,NM_001038594,NM_023505,XM_006529855,XM_006529856,XM_011248068	Glrx2	glutaredoxin 2 (thioltransferase)	protein_coding	[illegible]	[illegible]	[illegible]	[illegible]
69368	NM_001111279,NM_027057,XM_006496543,XM_017322386,XM_017322387,XM_030242794,XM_030242799,XM_030242801,XR_001785590	Wdfy1	WD repeat and FYVE domain containing 1	protein_coding	[illegible]	[illegible]	[illegible]	[illegible]
69372	NM_001160330	Mocs3	molybdenum cofactor synthesis 3	protein_coding	NP_001153802.1	[illegible]	[illegible]	[illegible]
69376	NM_001166494,NM_001166495,NM_027061,NM_199419	Zpbp2	zona pellucida binding protein 2	protein_coding	[illegible]	[illegible]	[illegible]	[illegible]
69379	NM_001271777,NM_027062,XM_011239171	C8g	complement component 8, gamma polypeptide	protein_coding	NP_001258706.1;NP_081338.1;XP_011237473.1	[illegible]	[illegible]	[illegible]
69384	NM_027066	Tmem89	transmembrane protein 89	protein_coding	NP_081342.1	[illegible]	[illegible]	[illegible]
69386	NM_153173	H2c8	histone cluster 1, H4h	protein_coding	NP_694813.1	[illegible]	[illegible]	[illegible]
69387	NM_153527,XM_006508184,XM_006508185	Dnajb13	DnaJ heat shock protein family (Hsp40) member B13	protein_coding	NP_705755.2;XP_006508247.1;XP_006508248.1	[illegible]	[illegible]	[illegible]
69397	NM_001368200,NM_027070,XM_006496253,XM_006496255,XR_373261,XR_865288	1700019A02Rik	RIKEN cDNA 1700019A02 gene	protein_coding	[illegible]	[illegible]	[illegible]	[illegible]
69398	XM_003688869	Cdhr4	cadherin-related family member 4	protein_coding	XP_003688917.1	[illegible]	[illegible]	[illegible]
69399	NM_197990,XM_011248069,XM_030242805	1700025G04Rik	RIKEN cDNA 1700025G04 gene	protein_coding	NP_932107.1;XP_011246371.1;XP_030098665.1	[illegible]	[illegible]	[illegible]
69408	NM_139139,XR_374520	Dnajc17	DnaJ heat shock protein family (Hsp40) member C17	protein_coding	NP_631878.2	[illegible]	[illegible]	[illegible]
69428	NM_027077,XM_006496976,XM_006496978,XM_017322429,XR_373615	1700016C15Rik	RIKEN cDNA 1700016C15 gene	protein_coding	[illegible]	[illegible]	[illegible]	[illegible]
69430	NR_033553	1700048O20Rik	RIKEN cDNA 1700048O20 gene	lncRNA	.	[illegible]	[illegible]	[illegible]
69431	XR_105888,XR_385484	1700022N22Rik	RIKEN cDNA 1700022N22 gene, transcript variant X1	lncRNA	.	[illegible]	[illegible]	[illegible]
69432	NR_130985	1700026J14Rik	RIKEN cDNA 1700026J14 gene	lncRNA	.	[illegible]	[illegible]	[illegible]
69434	NR_003145	Snhg10	small nucleolar RNA host gene 10	lncRNA	.	[illegible]	[illegible]	[illegible]
69440	NM_027081,XM_030248741,XM_030248742,XM_030248743,XM_030248744,XM_030248745,XM_030248746,XM_030248747,XM_030248748,XM_030248749,XM_030248750,XM_030248751,XR_003951424,XR_003951425,XR_003951426,XR_003951427,XR_003951428,XR_003951429,XR_003951430,XR_003951431,XR_003951432,XR_003951433,XR_003951434	Dennd6b	DENN/MADD domain containing 6B	protein_coding	[illegible]	[illegible]	[illegible]	[illegible]
69441	NM_001254724,XM_011249227	1700023F06Rik	RIKEN cDNA 1700023F06 gene	protein_coding	NP_001241653.1;XP_011247529.1	[illegible]	[illegible]	[illegible]
69443	NR_040581	1700027J07Rik	RIKEN cDNA 1700027J07 gene	lncRNA	.	[illegible]	[illegible]	[illegible]
69453	NM_027084,XM_006529858,XM_006529859,XM_006529860	Prss56	protease, serine 56	protein_coding	[illegible]	[illegible]	[illegible]	[illegible]
69454	NM_027085,XM_030252050	Clic3	chloride intracellular channel 3	protein_coding	NP_081361.1;XP_030107910.1	[illegible]	[illegible]	[illegible]
69456	NM_178377,XM_006526224,XM_006526225,XM_006526226,XM_006526227,XM_006526228,XM_006526229	Commd10	COMM domain containing 10	protein_coding	[illegible]	[illegible]	[illegible]	[illegible]
69457	NM_001358101,NM_183281	Tmem45a2	transmembrane protein 45A2	protein_coding	NP_001345030.1;NP_899104.2	[illegible]	[illegible]	[illegible]
69459	NM_001122873,NM_027086,XM_006511406,XM_006511407,XM_006511408,XM_030244562,XM_030244563,XM_030244564,XM_030244565,XM_030244566,XM_030244567	Ubl7	ubiquitin-like 7 (bone marrow stromal cell-derived)	protein_coding	[illegible]	[illegible]	[illegible]	[illegible]
69470	NM_001362704,NM_175145,XM_030252051	Tmem127	transmembrane protein 127	protein_coding	NP_001349633.1;NP_780354.1;XP_030107911.1	[illegible]	[illegible]	[illegible]
69478	NM_027090	2300009A05Rik	RIKEN cDNA 2300009A05 gene	protein_coding	NP_081366.1	[illegible]	[illegible]	[illegible]
69479	NM_001033148,NM_001359113,XR_003947334,XR_378912	1700029J07Rik	RIKEN cDNA 1700029J07 gene	protein_coding	NP_001028320.1;NP_001346042.1	[illegible]	[illegible]	[illegible]
69480	NM_001033149	Ttc9	tetratricopeptide repeat domain 9	protein_coding	NP_001028321.1	[illegible]	[illegible]	[illegible]
69481	NM_183282	Actl9	actin-like 9	protein_coding	NP_899105.2	[illegible]	[illegible]	[illegible]
69482	NM_001190179,NM_027091	Nup35	nucleoporin 35	protein_coding	NP_001177108.1;NP_081367.1	[illegible]	[illegible]	[illegible]
69487	NM_027093,XM_011239768,XM_030252052,XM_030252053,XR_003953715,XR_003953716	Ndufaf5	NADH:ubiquinone oxidoreductase complex assembly factor 5	protein_coding	[illegible]	[illegible]	[illegible]	[illegible]
69499	NM_001164578,NM_175146,NR_028392	Tsr2	TSR2 20S rRNA accumulation	protein_coding	NP_001158050.1;NP_780355.2	[illegible]	[illegible]	[illegible]
69504	NM_145563,XM_017321092,XM_017321093,XM_017321094,XM_017321095,XM_030254792	Zfp932	zinc finger protein 932	protein_coding	[illegible]	[illegible]	[illegible]	[illegible]
69513	NR_015521	1700030C10Rik	RIKEN cDNA 1700030C10 gene	lncRNA	.	[illegible]	[illegible]	[illegible]
69517	NR_028122	2310001K24Rik	RIKEN cDNA 2310001K24 gene	lncRNA	.	[illegible]	[illegible]	[illegible]
69519	NM_001145968,NM_001145969,NM_027100	Rwdd2a	RWD domain containing 2A	protein_coding	NP_001139440.1;NP_001139441.1;NP_081376.2	[illegible]	[illegible]	[illegible]
69524	NM_027102,NR_136939	Esam	endothelial cell-specific adhesion molecule	protein_coding	NP_081378.1	[illegible]	[illegible]	[illegible]
69527	NM_023514,XM_006496257,XM_030242824	Mrps9	mitochondrial ribosomal protein S9	protein_coding	NP_076003.3;XP_006496320.1;XP_030098684.1	[illegible]	[illegible]	[illegible]
69528	NM_001357253,NM_001357254,NM_001357255,NM_027103	1700030J22Rik	RIKEN cDNA 1700030J22 gene	protein_coding	[illegible]	[illegible]	[illegible]	[illegible]
69534	NM_027106	Avpi1	arginine vasopressin-induced 1	protein_coding	NP_081382.1	[illegible]	[illegible]	[illegible]
69535	NM_027107	Ten1	TEN1 telomerase capping complex subunit	protein_coding	NP_081383.1	[illegible]	[illegible]	[illegible]
69536	NM_133984,XM_006511821,XM_006511822,XM_006511823,XM_006511824,XM_011242913,XM_017313563,XM_030244568,XM_030244569,XM_030244570	Hemk1	HemK methyltransferase family member 1	protein_coding	[illegible]	[illegible]	[illegible]	[illegible]
69537	NM_001172154,NM_001370787,NM_027109,XM_006528272,XM_006528273,XM_017318613,XM_017318614	Dnase1l1	deoxyribonuclease 1-like 1	protein_coding	[illegible]	[illegible]	[illegible]	[illegible]
69538	NM_054041,XM_006506564	Antxr1	anthrax toxin receptor 1	protein_coding	NP_473382.1;XP_006506627.1	[illegible]	[illegible]	[illegible]
69539	NM_001081156,XM_006539155	Trnp1	TMF1-regulated nuclear protein 1	protein_coding	NP_001074625.1;XP_006539218.1	[illegible]	[illegible]	[illegible]
69540	NM_133712	Klk10	kallikrein related-peptidase 10	protein_coding	NP_598473.1	[illegible]	[illegible]	[illegible]
69543	NM_027112	Capns2	calpain, small subunit 2	protein_coding	NP_081388.1	[illegible]	[illegible]	[illegible]
69544	NM_027113	Wdr5b	WD repeat domain 5B	protein_coding	NP_081389.1	[illegible]	[illegible]	[illegible]
69546	NM_001045483,NM_027115	Mapk1ip1	mitogen-activated protein kinase 1 interacting protein 1	protein_coding	NP_001038948.1;NP_081391.1	[illegible]	[illegible]	[illegible]
69547	NM_027116,XM_006540350,XM_030242971	Nkpd1	NTPase, KAP family P-loop domain containing 1	protein_coding	NP_081392.1;XP_006540413.2;XP_030098831.1	[illegible]	[illegible]	[illegible]
69548	NR_033514	2310015A10Rik	RIKEN cDNA 2310015A10 gene	lncRNA	.	[illegible]	[illegible]	[illegible]
69549	NM_001081226	2310009B15Rik	RIKEN cDNA 2310009B15 gene	protein_coding	NP_001074695.1	[illegible]	[illegible]	[illegible]
69550	NM_198095	Bst2	bone marrow stromal cell antigen 2	protein_coding	NP_932763.1	[illegible]	[illegible]	[illegible]
69551	NM_175149	2310022B05Rik	RIKEN cDNA 2310022B05 gene	protein_coding	NP_780358.3	[illegible]	[illegible]	[illegible]
69553	NM_001080708,NR_156115,XM_006500137,XM_006500138,XM_006500139,XM_006500140,XM_006500141,XR_866339,XR_866340	Ripor3	RIPOR family member 3	protein_coding	[illegible]	[illegible]	[illegible]	[illegible]
69554	NM_027117	Klhdc2	kelch domain containing 2	protein_coding	NP_081393.2	[illegible]	[illegible]	[illegible]
69556	NM_001024919	Bod1	biorientation of chromosomes in cell division 1	protein_coding	NP_001020090.1	[illegible]	[illegible]	[illegible]
69561	XR_385539,XR_876746	2310015A16Rik	RIKEN cDNA 2310015A16 gene, transcript variant X1	lncRNA	.	[illegible]	[illegible]	[illegible]
69562	NM_001081058,NM_027118,XM_006516766,XM_006516767,XM_011244344,XM_017315593	Cdk13	cyclin-dependent kinase 13	protein_coding	[illegible]	[illegible]	[illegible]	[illegible]
69563	NM_001304739,XM_017314076	Mrln	myoregulin	protein_coding	NP_001291668.1;XP_017169565.1	[illegible]	[illegible]	[illegible]
69564	NM_027120,XM_011243554	Nmrk2	nicotinamide riboside kinase 2	protein_coding	NP_081396.1;XP_011241856.1	[illegible]	[illegible]	[illegible]
69565	XR_373578	2310015K22Rik	RIKEN cDNA 2310015K22 gene	lncRNA	.	[illegible]	[illegible]	[illegible]
69566	NR_045491	2310016D03Rik	RIKEN cDNA 2310016D03 gene	lncRNA	.	[illegible]	[illegible]	[illegible]
69568	NM_001001327,NM_001286382,NM_027121	Vkorc1l1	vitamin K epoxide reductase complex, subunit 1-like 1	protein_coding	NP_001001327.1;NP_001273311.1;NP_081397.1	[illegible]	[illegible]	[illegible]
69571	NR_040679	2310034O05Rik	RIKEN cDNA 2310034O05 gene	lncRNA	.	[illegible]	[illegible]	[illegible]
69572	NM_027122	Mfsd3	major facilitator superfamily domain containing 3	protein_coding	NP_081398.2	[illegible]	[illegible]	[illegible]
69573	NM_001190461,NM_023516	Hilpda	hypoxia inducible lipid droplet associated	protein_coding	NP_001177390.1;NP_076005.1	[illegible]	[illegible]	[illegible]
69574	NM_181588,XM_006520176,XM_006520177,XM_006520178,XM_030248752	Cmbl	carboxymethylenebutenolidase-like (Pseudomonas)	protein_coding	[illegible]	[illegible]	[illegible]	[illegible]
69576	NM_183283	Smco1	single-pass membrane protein with coiled-coil domains 1	protein_coding	NP_899106.2	[illegible]	[illegible]	[illegible]
69577	NM_001330423,NM_027123,NR_138477,NR_138478,XM_006517364,XM_006517366,XM_006517367,XM_017315595,XR_003950475,XR_003950476,XR_382449	Fastkd3	FAST kinase domains 3	protein_coding	[illegible]	[illegible]	[illegible]	[illegible]
69578	NR_152106	2310016G11Rik	RIKEN cDNA 2310016G11 gene	lncRNA	.	[illegible]	[illegible]	[illegible]
69579	XM_006508373	2310034P14Rik	RIKEN cDNA 2310034P14 gene	protein_coding	XP_006508436.1	[illegible]	[illegible]	[illegible]
69581	NM_133955	Rhou	ras homolog family member U	protein_coding	NP_598716.1	[illegible]	[illegible]	[illegible]
69582	NM_001033150,NM_001347237,XR_390780	Plekhm2	pleckstrin homology domain containing, family M (with RUN domain) member 2	protein_coding	NP_001028322.1;NP_001334166.1	[illegible]	[illegible]	[illegible]
69583	NM_001159505,NM_023517	Tnfsf13	tumor necrosis factor (ligand) superfamily, member 13	protein_coding	NP_001152977.1;NP_076006.2	[illegible]	[illegible]	[illegible]
69585	NM_027126,XM_006502037	Hjv	hemojuvelin BMP co-receptor	protein_coding	NP_081402.3;XP_006502100.1	[illegible]	[illegible]	[illegible]
69587	NM_172716,XM_006535208,XM_030254794	Pcgf3	polycomb group ring finger 3	protein_coding	NP_766304.2;XP_006535271.1;XP_030110654.1	[illegible]	[illegible]	[illegible]
69590	NM_027127,XM_017315596	Gpx8	glutathione peroxidase 8 (putative)	protein_coding	NP_081403.1;XP_017171085.1	[illegible]	[illegible]	[illegible]
69591	NR_131979	Mdrl	mitochondrial dynamic related lncRNA	lncRNA	.	[illegible]	[illegible]	[illegible]
69592	NM_027128,XM_006535209	Odam	odontogenic, ameloblast asssociated	protein_coding	NP_081404.2;XP_006535272.1	[illegible]	[illegible]	[illegible]
69593	XR_386153,XR_877284	2310026I22Rik	RIKEN cDNA 2310026I22 gene, transcript variant X2	lncRNA	.	[illegible]	[illegible]	[illegible]
69596	NM_001291031,NM_001291032,NM_001355593,NM_027129,NR_110992	Ap5s1	adaptor-related protein 5 complex, sigma 1 subunit	protein_coding	[illegible]	[illegible]	[illegible]	[illegible]
69597	NM_027130,XM_011246998	Afg3l2	AFG3-like AAA ATPase 2	protein_coding	NP_081406.1;XP_011245300.1	[illegible]	[illegible]	[illegible]
69601	NM_001001602,NM_001114124,NM_001114125,NM_001290637,NM_001290639,NM_001290640,NM_001290641,XM_006498304,XM_006498305,XM_006498307,XM_006498311,XM_011239172,XM_011239174,XM_011239175,XM_017319251,XM_030252055	Dab2ip	disabled 2 interacting protein	protein_coding	[illegible]	[illegible]	[illegible]	[illegible]
69603	NR_045495	2310020H05Rik	RIKEN cDNA 2310020H05 gene	lncRNA	.	[illegible]	[illegible]	[illegible]
69605	NM_001110209,NM_027133,XM_006500145,XM_006500146,XM_006500147,XM_011239769,XM_030252056,XM_030252057,XM_030252058	Lnpk	lunapark, ER junction formation factor	protein_coding	[illegible]	[illegible]	[illegible]	[illegible]
69606	NM_027134	Mtfmt	mitochondrial methionyl-tRNA formyltransferase	protein_coding	NP_081410.2	[illegible]	[illegible]	[illegible]
69608	NM_027135,XM_006502038,XM_030252809,XM_030252810	Sec24d	Sec24 related gene family, member D (S. cerevisiae)	protein_coding	[illegible]	[illegible]	[illegible]	[illegible]
69611	NM_027137	Lce1d	late cornified envelope 1D	protein_coding	NP_081413.1	[illegible]	[illegible]	[illegible]
69612	NM_001289437,NM_001289438,NM_001289439,NM_001289440,NM_001359827,NM_133714,NR_153345,XM_017316743,XM_030248753,XM_030248754,XM_030248755,XR_003951435,XR_384040	Kansl2	KAT8 regulatory NSL complex subunit 2	protein_coding	[illegible]	[illegible]	[illegible]	[illegible]
69617	NM_001360106,NM_145131,XM_006516509,XR_003950477,XR_003950478	Pitrm1	pitrilysin metallepetidase 1	protein_coding	NP_001347035.1;NP_660113.1;XP_006516572.1	[illegible]	[illegible]	[illegible]
69623	NM_001346671,NM_001346672,XM_030254795,XM_030254796	Zfp33b	zinc finger protein 33B	protein_coding	[illegible]	[illegible]	[illegible]	[illegible]
69627	NM_001081120	Fam89a	family with sequence similarity 89, member A	protein_coding	NP_001074589.1	[illegible]	[illegible]	[illegible]
69629	NR_045337	2310039L15Rik	RIKEN cDNA 2310039L15 gene	lncRNA	.	[illegible]	[illegible]	[illegible]
69631	NR_040714	Plet1os	placenta expressed transcript 1, opposite strand	lncRNA	.	[illegible]	[illegible]	[illegible]
69632	NM_001359232,NM_027144,XM_011242597,XM_011242598,XM_017313565,XM_017313566,XM_017313567,XM_030244571,XM_030244572,XM_030244573	Arhgef12	Rho guanine nucleotide exchange factor (GEF) 12	protein_coding	[illegible]	[illegible]	[illegible]	[illegible]
69634	NM_029556,XM_011245172,XM_011245173,XM_011245174,XM_011245175,XM_030247994,XM_030247995,XM_030247996,XM_030247997	Clybl	citrate lyase beta like	protein_coding	[illegible]	[illegible]	[illegible]	[illegible]
69635	NM_001285917,NM_029653,NM_134062,XM_006517370,XM_006517371,XM_017315598,XM_030247400,XM_030247401	Dapk1	death associated protein kinase 1	protein_coding	[illegible]	[illegible]	[illegible]	[illegible]
69638	NM_027147	Enho	energy homeostasis associated	protein_coding	NP_081423.1	[illegible]	[illegible]	[illegible]
69639	NM_001163570,NM_027148	Exosc8	exosome component 8	protein_coding	NP_001157042.1;NP_081424.3	[illegible]	[illegible]	[illegible]
69640	NM_178618,XM_006534119,XM_006534120	Fam83g	family with sequence similarity 83, member G	protein_coding	NP_848733.2;XP_006534182.1;XP_006534183.1	[illegible]	[illegible]	[illegible]
69641	NM_001347428,NM_001364272,NM_001364273,NM_027149,XM_006516203,XM_006516204,XM_006516205,XM_006516207,XM_011244170,XM_030246900,XM_030246901,XR_872718	Wdr20	WD repeat domain 20	protein_coding	[illegible]	[illegible]	[illegible]	[illegible]
69642	NM_001368955,NM_001368956,NM_001368957,NM_001368958,NM_001368959,NM_001368960,NM_001368961,NM_001368962,NM_001368963,NM_001368964,NM_001368965,NM_001368966,NM_001368967,NM_001368968,NM_001368969,NM_001368970,NM_001368971,NM_001368972,NM_001368973,NM_001368974,NM_001368975,NM_001368976,NM_001368977,NM_001368978,NM_001368979,NM_001368980,NM_001368981,NM_001368982,NM_001368983,NM_001368984,NM_001368985,NM_001368986,NM_001368987,NM_001368988,NM_001368989,NM_027150,XM_006511410,XM_006511411,XM_006511412,XM_006511413,XM_006511414,XM_006511415,XM_006511416,XM_006511417,XM_006511418,XM_006511420,XM_006511421,XM_006511422,XM_006511423,XM_006511424,XM_006511425,XM_006511426,XM_006511427,XM_017313568,XM_017313569,XM_017313572,XM_017313574,XM_017313577,XM_017313580,XM_017313582,XM_030244574,XM_030244575,XM_030244576,XM_030244577,XM_030244578,XM_030244579,XM_030244580,XM_030244581,XM_030244582	Mlip	muscular LMNA-interacting protein	protein_coding	[illegible]	[illegible]	[illegible]	[illegible]
69652	NR_040520	2310069B03Rik	RIKEN cDNA 2310069B03 gene	lncRNA	.	[illegible]	[illegible]	[illegible]
69654	NM_001190453,NM_001190454,NM_027151,XM_006514055	Dctn2	dynactin 2	protein_coding	[illegible]	[illegible]	[illegible]	[illegible]
69655	NM_027152	Cd164l2	CD164 sialomucin-like 2	protein_coding	NP_081428.1	[illegible]	[illegible]	[illegible]
69656	NM_001301402,NM_027153	Pir	pirin	protein_coding	NP_001288331.1;NP_081429.1	[illegible]	[illegible]	[illegible]

69657	XR_001785026,XR_001785027,XR_003956019,XR_003956020,XR_003956021,XR_003956022	2310047D07Rik	RIKEN cDNA 2310047D07 gene, transcript variant X4	lncRNA	.	1916907	.	0.309224	[illegible]
69659	NR_040309	2310069G16Rik	RIKEN cDNA 2310069G16 gene	lncRNA	.	1916909	.	0.805424	[illegible]
69660	NM_027154	Tmbim1	transmembrane BAX inhibitor motif containing 1	protein_coding	NP_081430.3	1916910	.	40.321045	[illegible]
69662	NM_001033630,XM_006524875,XM_006524877	2310061I04Rik	RIKEN cDNA 2310061I04 gene	protein_coding	NP_001028802.1;XP_006524938.1;XP_006524940.1	1916912	.	9.588268	[illegible]
69663	NM_027156,XR_880400	Ddx51	DEAD (Asp-Glu-Ala-Asp) box polypeptide 51	protein_coding	NP_081432.2	1916913	.	3.558784	[illegible]
69665	NM_001359361,NM_027158,NR_153102,XM_006504495,XM_011240906,XR_377114	Upk3bl	uroplakin 3B-like	protein_coding	NP_001346290.1;NP_081434.1;XP_006504558.2;XP_011239208.1	1916915	.	0.000000	[illegible]
69666	NM_001101430,NM_001110515	Psmg4	proteasome (prosome, macropain) assembly chaperone 4	protein_coding	NP_001094900.1;NP_001103985.1	1916916	.	8.556068	[illegible]
69668	NM_027159	Ccdc115	coiled-coil domain containing 115	protein_coding	NP_081435.1	1916918	.	11.430159	[illegible]
69671	NM_001253856,NM_001253858,NM_027161	Tmem52	transmembrane protein 52	protein_coding	NP_001240785.1;NP_001240787.1;NP_081437.1	1916921	.	0.594188	[illegible]
69672	NM_175150	Txndc15	thioredoxin domain containing 15	protein_coding	NP_780359.2	1916922	.	23.220379	[illegible]
69674	NM_001243584,NM_001243586,NM_001243587,NM_027162,XM_011249229	Mif4gd	MIF4G domain containing	protein_coding	NP_001230513.1;NP_001230515.1;NP_001230516.1;NP_081438.4;XP_011247531.1	1916924	.	13.774932	[illegible]
69675	NM_181395,XM_006515205,XM_006515206	Pxdn	peroxidasin	protein_coding	NP_852060.2;XP_006515268.1;XP_006515269.1	1916925	.	31.303149	[illegible]
69678	NR_015477	Atcayos	ataxia, cerebellar, Cayman type, opposite strand	lncRNA	.	1916928	.	4.351799	[illegible]
69681	NR_004853	Cdk3	cyclin-dependent kinase 3	transcribed_pseudogene	.	1916931	.	0.850403	[illegible]
69683	NM_001355107,NM_197991,XM_006541176,XM_030242972,XR_003946559	Emc10	ER membrane protein complex subunit 10	protein_coding	NP_001342036.1;NP_932108.3;XP_006541239.1;XP_030098832.1	1916933	.	34.618430	[illegible]
69684	NM_144829,XM_006534121,XM_030246279	Aarsd1	alanyl-tRNA synthetase domain containing 1	protein_coding	NP_659078.1;XP_006534184.1;XP_030102139.1	1916934	.	14.417851	[illegible]
69692	NM_027168,XM_030245251,XM_030245252,XR_003948792,XR_003948793	Hddc2	HD domain containing 2	protein_coding	NP_081444.1;XP_030101111.1;XP_030101112.1	1916942	.	11.626717	[illegible]
69693	NM_181579,XM_006528406,XM_006528407	Pof1b	premature ovarian failure 1B	protein_coding	NP_853557.1;XP_006528469.1;XP_006528470.1	1916943	.	0.051973	[illegible]
69694	NM_001368428,NM_001368628,NM_001368629,NM_001368630,NM_001368631,NM_001368632,NM_001368633,NM_001368634,NM_001368635,NM_001368636,NM_175151,XM_006521366,XM_006521367,XM_006521374,XM_030248756,XM_030248757,XM_030248758,XM_030248759,XM_030248760,XM_030248761,XR_003951436,XR_003951437,XR_384041,XR_384043	Tatdn1	TatD DNase domain containing 1	protein_coding	NP_001355357.1;NP_001355557.1;NP_001355558.1;NP_001355559.1;NP_001355560.1;NP_001355561.1;NP_001355562.1;NP_001355563.1;NP_001355564.1;NP_001355565.1;NP_780360.1;XP_006521429.1;XP_006521430.1;XP_006521437.1;XP_030104616.1;XP_030104617.1;XP_030104618.1;XP_030104619.1;XP_030104620.1;XP_030104621.1	1916944	.	4.106637	[illegible]
69697	NM_001163749,NM_001347111,NM_001347112,NM_001347113,NM_001363239,NM_001363240,NM_001363241,NM_001363242,NM_001363243,NM_001363244,NM_001363245,NM_001363246,NM_001363247,NM_001363248,NM_001363249,NM_027171,XM_006508880,XM_006508881,XM_030243752	Camsap3	calmodulin regulated spectrin-associated protein family, member 3	protein_coding	NP_001157221.1;NP_001334040.1;NP_001334041.1;NP_001334042.1;NP_001350168.1;NP_001350169.1;NP_001350170.1;NP_001350171.1;NP_001350172.1;NP_001350173.1;NP_001350174.1;NP_001350175.1;NP_001350176.1;NP_001350177.1;NP_001350178.1;NP_081447.2;XP_006508943.1;XP_006508944.1;XP_030099612.1	1916947	.	0.540015	[illegible]
69698	NM_001164819,NM_001164820,NM_027172	Slc52a3	solute carrier protein family 52, member 3	protein_coding	NP_001158291.1;NP_001158292.1;NP_081448.2	1916948	.	2.406806	[illegible]
69700	NM_027174,XM_006521378,XM_006521381,XM_006521382,XM_006521383	Col22a1	collagen, type XXII, alpha 1	protein_coding	NP_081450.1;XP_006521441.3;XP_006521444.1;XP_006521445.1;XP_006521446.1	1916950	.	58.833908	[illegible]
69702	NM_001356467,NM_027175,XM_006500149	Ndufaf1	NADH:ubiquinone oxidoreductase complex assembly factor 1	protein_coding	NP_001343396.1;NP_081451.3;XP_006500212.1	1916952	.	6.500837	[illegible]
69706	NM_001081406,NR_155206	Lrr1	leucine rich repeat protein 1	protein_coding	NP_001074875.1	1916956	.	1.035618	[illegible]
69707	NM_178378,XM_006522519,XM_006522520,XM_011246004	Iqcg	IQ motif containing G	protein_coding	NP_848465.1;XP_006522582.1;XP_006522583.1;XP_011244306.1	1916957	.	0.207005	[illegible]
69709	NM_001204912	Ptrhd1	peptidyl-tRNA hydrolase domain containing 1	protein_coding	NP_001191841.1	1916959	.	1.513562	[illegible]
69710	NM_001040111,NM_001040112,NM_027180,NM_198096,XM_006508186,XM_006508187,XM_006508188,XM_006508190,XM_006508191,XM_006508192,XM_006508193,XM_006508195,XM_006508196,XM_011241897,XM_030242973,XM_030242974,XR_003946560	Arap1	ArfGAP with RhoGAP domain, ankyrin repeat and PH domain 1	protein_coding	NP_001035200.1;NP_001035201.1;NP_081456.2;NP_932764.1;XP_006508249.1;XP_006508250.1;XP_006508251.1;XP_006508253.1;XP_006508254.1;XP_006508255.1;XP_006508256.1;XP_006508258.1;XP_006508259.1;XP_011240199.1;XP_030098833.1;XP_030098834.1	1916960	.	14.206264	[illegible]
69713	NM_001365090,NM_027181	Pin4	protein (peptidyl-prolyl cis/trans isomerase) NIMA-interacting, 4	protein_coding	NP_001352019.1;NP_081457.1	1916963	.	14.052722	[illegible]
69714	NM_001290381,NM_023524	Tfpt	TCF3 (E2A) fusion partner	protein_coding	NP_001277310.1;NP_076013.1	1916964	.	10.871597	[illegible]
69716	NM_027182,XR_873456	Trip13	thyroid hormone receptor interactor 13	protein_coding	NP_081458.1	1916966	.	3.682725	[illegible]
69717	NM_001370818,NM_001370819,NM_001370820,NM_001370821,XM_017317765	Gm10499	predicted gene 10499	protein_coding	NP_001357747.1;NP_001357748.1;NP_001357749.1;NP_001357750.1;XP_017173254.1	3702919	.	0.000000	[illegible]
69718	NM_001347191,NM_027184,XM_011243555	Ipmk	inositol polyphosphate multikinase	protein_coding	NP_001334120.1;NP_081460.1;XP_011241857.1	1916968	.	3.394119	[illegible]
69719	NM_001289522,NM_001289523,NM_023525,XM_006504091,XM_006504092	Cad	carbamoyl-phosphate synthetase 2, aspartate transcarbamylase, and dihydroorotase	protein_coding	NP_001276451.1;NP_001276452.1;NP_076014.1;XP_006504154.1;XP_006504155.1	1916969	.	8.625079	[illegible]
69721	NM_001316711,NM_001316712,NM_023526,XM_006518097,XM_030247999	Nkiras1	NFKB inhibitor interacting Ras-like protein 1	protein_coding	NP_001303640.1;NP_001303641.1;NP_076015.2;XP_006518160.1;XP_030103859.1	1916971	.	3.856003	[illegible]
69723	NM_001252413,NM_001252414,NM_001252415,NM_027186,NR_156443,XR_003949500	Rpain	RPA interacting protein	protein_coding	NP_001239342.1;NP_001239343.1;NP_001239344.1;NP_081462.1	1916973	.	8.100029	[illegible]
69724	NM_001364370,NM_027187,XM_011248497,XM_011248498	Rnaseh2a	ribonuclease H2, large subunit	protein_coding	NP_001351299.1;NP_081463.1;XP_011246799.1;XP_011246800.1	1916974	.	9.164178	[illegible]
69726	NM_027188,XM_006496982,XM_011238864,XM_011238865,XM_017312211,XM_017312213,XM_030242841	Smyd3	SET and MYND domain containing 3	protein_coding	NP_081464.1;XP_006497045.1;XP_011237166.1;XP_011237167.1;XP_017167700.1;XP_017167702.1;XP_030098701.1	1916976	.	1.261278	[illegible]
69727	NM_177561,XM_006504268,XM_006504269	Usp46	ubiquitin specific peptidase 46	protein_coding	NP_808229.1;XP_006504331.1;XP_006504332.1	1916977	.	7.763326	[illegible]
69729	NR_045496	2410003L11Rik	RIKEN cDNA 2410003L11 gene	lncRNA	.	1916979	.	0.000000	[illegible]
69731	NM_027189	Gemin7	gem nuclear organelle associated protein 7	protein_coding	NP_081465.1	1916981	.	27.918159	[illegible]
69732	NR_015504	2410018L13Rik	RIKEN cDNA 2410018L13 gene	lncRNA	.	1916982	.	1.036013	[illegible]
69736	NM_027191,NM_028334,XM_006514057	Nup37	nucleoporin 37	protein_coding	NP_081467.2;NP_082610.1;XP_006514120.1	1919964	.	8.107072	[illegible]
69737	NM_027192	Ttl	tubulin tyrosine ligase	protein_coding	NP_081468.1	1916987	.	7.883305	[illegible]
69739	NR_037963	2410004I01Rik	RIKEN cDNA 2410004I01 gene	lncRNA	.	1916989	.	0.213400	[illegible]
69740	NM_027193,XR_003954378,XR_003954379,XR_003954380	Dph5	diphthamide biosynthesis 5	protein_coding	NP_081469.2	1916990	.	3.525891	[illegible]
69742	NM_027194	Tm2d2	TM2 domain containing 2	protein_coding	NP_081470.2	1916992	.	25.181067	[illegible]
69743	NM_001159344,NM_027195,NR_161310,XM_006539157,XM_006539158,XM_006539159,XM_006539160	Casz1	castor zinc finger 1	protein_coding	NP_001152816.1;NP_081471.2;XP_006539220.1;XP_006539221.1;XP_006539222.1;XP_006539223.1	1196251	.	0.248383	[illegible]
69745	NM_027196,NR_132280	Pold4	polymerase (DNA-directed), delta 4	protein_coding	NP_081472.1	1916995	.	17.855751	[illegible]
69747	NM_027198	Zswim7	zinc finger SWIM-type containing 7	protein_coding	NP_081474.1	1916997	.	5.008992	[illegible]
69748	NM_145954	Aldh16a1	aldehyde dehydrogenase 16 family, member A1	protein_coding	NP_666066.1	1916998	.	10.383419	[illegible]
69749	NR_038151,NR_038152	Epb41l4aos	erythrocyte membrane protein band 4.1 like 4a, opposite strand	lncRNA	.	1916999	.	15.201655	[illegible]
69752	NM_027201,XM_006536239,XM_030242975,XR_003946561	Zfp511	zinc finger protein 511	protein_coding	NP_081477.1;XP_006536302.1;XP_030098835.1	1917002	.	7.550608	[illegible]
69754	NM_001310745,NM_001347151,NM_153195,XM_011243556,XM_017314077	Fbxo7	F-box protein 7	protein_coding	NP_001297674.1;NP_001334080.1;NP_694875.2;XP_011241858.1;XP_017169566.1	1917004	.	6.845047	[illegible]
69757	NM_027203	Leng1	leukocyte receptor cluster (LRC) member 1	protein_coding	NP_081479.1	1917007	.	3.459684	[illegible]
69761	NM_001081273	Doxl1	diamine oxidase-like protein 1	protein_coding	NP_001074742.2	1917011	.	0.019073	[illegible]
69768	NR_040481	1600019K03Rik	RIKEN cDNA 1600019K03 gene	lncRNA	.	1917018	.	0.000000	[illegible]
69769	NM_027206,XM_006502040	Tnfaip8l2	tumor necrosis factor, alpha-induced protein 8-like 2	protein_coding	NP_081482.1;XP_006502103.1	1917019	.	2.489557	[illegible]
69770	NM_027207	1600002K03Rik	RIKEN cDNA 1600002K03 gene	protein_coding	NP_081483.1	1917020	.	5.925546	[illegible]
69771	NR_040344,NR_040345,NR_040346	1810019D21Rik	RIKEN cDNA 1810019D21 gene	lncRNA	.	1917021	.	0.100681	[illegible]
69772	NM_001172055,NM_027208	Bdh2	3-hydroxybutyrate dehydrogenase, type 2	protein_coding	NP_001165526.1;NP_081484.1	1917022	.	1.892575	[illegible]
69773	NM_178619	Timm29	translocase of inner mitochondrial membrane 29	protein_coding	NP_848734.1	1917023	.	14.659770	[illegible]
69774	NM_027209	Ms4a6b	membrane-spanning 4-domains, subfamily A, member 6B	protein_coding	NP_081485.2	1917024	.	3.632707	[illegible]
69780	NM_133716,XM_006503378,XM_006503379,XM_006503380	Smap2	small ArfGAP 2	protein_coding	NP_598477.2;XP_006503441.1;XP_006503442.1;XP_006503443.1	1917030	.	13.127720	[illegible]
69781	NR_037959	1600010M07Rik	RIKEN cDNA 1600010M07 gene	lncRNA	.	1917031	.	0.148493	[illegible]
69784	NM_001145198,XM_006514060,XM_011243557,XM_011243558	1500009L16Rik	RIKEN cDNA 1500009L16 gene	protein_coding	NP_001138670.1;XP_006514123.1;XP_011241859.1;XP_011241860.1	1917034	.	1.484704	[illegible]
69786	NM_001170488,NM_001362064,NM_001362065,NM_001362066,NM_001362067,NM_176842,XM_006506567,XM_017321732,XM_030255586,XM_030255587	Tprkb	Tp53rk binding protein	protein_coding	NP_001163959.1;NP_001348993.1;NP_001348994.1;NP_001348995.1;NP_001348996.1;NP_789812.1;XP_006506630.1;XP_017177221.1;XP_030111446.1;XP_030111447.1	1917036	.	11.783860	[illegible]
69788	NR_073433	1600023N17Rik	RIKEN cDNA 1600023N17 gene	lncRNA	.	1917038	.	0.342507	[illegible]
69790	NM_027212	Med30	mediator complex subunit 30	protein_coding	NP_081488.1	1917040	.	8.511140	[illegible]
69792	NM_001347384,NM_001347385,NM_001347386	Med6	mediator complex subunit 6	protein_coding	NP_001334313.1;NP_001334314.1;NP_001334315.1	1917042	.	11.625247	[illegible]
69794	NR_036588	1600027J07Rik	RIKEN cDNA 1600027J07 gene	lncRNA	.	1917044	.	0.000000	[illegible]
69797	NR_028123	1600029I14Rik	RIKEN cDNA 1600029I14 gene	lncRNA	.	1917047	.	0.000000	[illegible]
69798	NR_038153	1810044D09Rik	RIKEN cDNA 1810044D09 gene	lncRNA	.	1917048	.	0.999945	[illegible]
69802	NM_199008	Cox11	cytochrome c oxidase assembly protein 11, copper chaperone	protein_coding	NP_950173.1	1917052	.	5.414927	[illegible]
69804	NM_027215	Tmem147	transmembrane protein 147	protein_coding	NP_081491.2	1915011	.	42.738857	[illegible]
69806	NM_001166503,NM_001362937,NM_001362938,NM_001362939,NM_001362940,NM_001362941,NM_001362942,NM_001362943,NM_001362944,NM_027216,XM_006534130,XM_011249230,XM_011249231,XM_011249233,XM_011249234,XM_011249235,XM_011249236,XM_011249237,XM_011249238,XM_011249239,XM_011249240,XM_017314742,XM_030246280,XM_030246281,XM_030246282,XM_030246283,XM_030246284,XM_030246285,XM_030246286,XM_030246287,XM_030246288	Slc39a11	solute carrier family 39 (metal ion transporter), member 11	protein_coding	NP_001159975.1;NP_001349866.1;NP_001349867.1;NP_001349868.1;NP_001349869.1;NP_001349870.1;NP_001349871.1;NP_001349872.1;NP_001349873.1;NP_081492.4;XP_006534193.1;XP_011247532.1;XP_011247533.1;XP_011247535.1;XP_011247536.1;XP_011247537.1;XP_011247538.1;XP_011247539.1;XP_011247540.1;XP_011247541.1;XP_011247542.1;XP_017170231.1;XP_030102140.1;XP_030102141.1;XP_030102142.1;XP_030102143.1;XP_030102144.1;XP_030102145.1;XP_030102146.1;XP_030102147.1;XP_030102148.1	1917056	.	8.766795	[illegible]
69807	NM_001161782,NM_053084,XM_006538235,XM_006538236,XM_006538237,XM_006538238,XM_030253765	Trim32	tripartite motif-containing 32	protein_coding	NP_001155254.1;NP_444314.2;XP_006538298.1;XP_006538299.1;XP_006538300.1;XP_006538301.1;XP_030109625.1	1917057	.	7.361500	[illegible]
69812	NR_026688	Abhd11os	abhydrolase domain containing 11, opposite strand	lncRNA	.	1917062	.	0.523503	[illegible]
69815	NM_001363337,NM_027221,XM_030254797	Krtcap3	keratinocyte associated protein 3	protein_coding	NP_001350266.1;NP_081497.2;XP_030110657.1	1917065	.	1.854573	[illegible]
69816	NM_027222,XM_017317978	Mzb1	marginal zone B and B1 cell-specific protein 1	protein_coding	NP_081498.2;XP_017173467.1	1917066	.	0.142525	[illegible]
69821	NM_001310756,NM_178051	Mterf4	mitochondrial transcription termination factor 4	protein_coding	NP_001297685.1;NP_835152.1	1918355	.	5.456258	[illegible]
69823	NM_001159349,NM_001356387,NM_027226,XM_006522522,XM_006522523	Fyttd1	forty-two-three domain containing 1	protein_coding	NP_001152821.1;NP_001343316.1;NP_081502.2;XP_006522585.1;XP_006522586.1	1917955	.	18.614945	[illegible]
69824	NM_027227,XM_006527699	Glod5	glyoxalase domain containing 5	protein_coding	NP_081503.1;XP_006527762.1	1917074	.	0.000000	[illegible]
69833	NM_027231	Polr2f	polymerase (RNA) II (DNA directed) polypeptide F	protein_coding	NP_081507.1	1349393	.	27.906919	[illegible]
69834	NM_001039394,NM_133717,NR_104477,NR_104478	Rab43	RAB43, member RAS oncogene family	protein_coding	NP_001034483.1;NP_598478.1	1917084	.	11.160961	[illegible]
69836	NM_023530,XM_006514061,XM_006514062	Pla2g12b	phospholipase A2, group XIIB	protein_coding	NP_076019.2;XP_006514124.1;XP_006514125.1	1917086	.	0.033318	[illegible]
69837	NM_197992,XM_030255588	Pcgf1	polycomb group ring finger 1	protein_coding	NP_932109.1;XP_030111448.1	1917087	.	6.424134	[illegible]
69847	NM_175638,XM_006534136,XM_006534137,XM_006534138,XM_006534139,XM_006534141,XM_017314744	Wnk4	WNK lysine deficient protein kinase 4	protein_coding	NP_783569.1;XP_006534199.1;XP_006534200.1;XP_006534201.1;XP_006534202.1;XP_006534204.1;XP_017170233.1	1917097	.	5.866252	[illegible]
69852	NM_053085,XM_006504095,XM_006504096	Tcf23	transcription factor 23	protein_coding	NP_444315.1;XP_006504158.1;XP_006504159.1	1934960	.	0.192164	[illegible]
69857	NR_040486,NR_040487,NR_040488,NR_040489,NR_040490,NR_040491	1810053B23Rik	RIKEN cDNA 1810053B23 gene	lncRNA	.	1917107	.	0.000000	[illegible]
69860	NM_027236,XM_030251073	Eif1ad	eukaryotic translation initiation factor 1A domain containing	protein_coding	NP_081512.1;XP_030106933.1	1917110	.	10.226144	[illegible]
69863	NM_027238,XM_006538239,XM_006538241,XM_011250101,XM_030253766	Ttc39b	tetratricopeptide repeat domain 39B	protein_coding	NP_081514.1;XP_006538302.1;XP_006538304.1;XP_011248403.2;XP_030109626.1	1917113	.	5.340974	[illegible]
69870	NM_001293701,NM_027241,XM_030252811	Polr3gl	polymerase (RNA) III (DNA directed) polypeptide G like	protein_coding	NP_001280630.1;NP_081517.2;XP_030108671.1	1917120	.	16.827494	[illegible]
69871	NM_001356989,NM_027242,XM_030254798	Ppp1r35	protein phosphatase 1, regulatory subunit 35	protein_coding	NP_001343918.1;NP_081518.1;XP_030110658.1	1922853	.	5.419403	[illegible]
69875	NM_027244	Ndufa11	NADH:ubiquinone oxidoreductase subunit A11	protein_coding	NP_081520.1	1917125	.	9.092797	[illegible]
69876	NM_001145929,NM_175152	Thap3	THAP domain containing, apoptosis associated protein 3	protein_coding	NP_001139401.1;NP_780361.2	1917126	.	20.464478	[illegible]
69878	NM_027246	Snrpf	small nuclear ribonucleoprotein polypeptide F	protein_coding	NP_081522.1	1917128	.	31.714993	[illegible]
69881	XR_877329	2010320O07Rik	RIKEN cDNA 2010320O07 gene	lncRNA	.	1917131	.	0.000000	[illegible]
69882	NM_001077631,NM_175153	Ints14	integrator complex subunit 14	protein_coding	NP_001071099.1;NP_780362.2	1917132	.	11.263011	[illegible]
69885	NM_001081099	Aunip	aurora kinase A and ninein interacting protein	protein_coding	NP_001074568.1	1917135	.	0.725791	[illegible]
69890	NM_001253694,NM_001253695,NM_001253696,NM_027248,XM_006519498,XM_006519499,XM_006519501,XM_006519502,XM_006519503,XM_006519504,XM_006519505,XM_006519506,XM_011245176,XM_011245177,XM_017316180	Zfp219	zinc finger protein 219	protein_coding	NP_001240623.1;NP_001240624.1;NP_001240625.1;NP_081524.2;XP_006519561.1;XP_006519562.1;XP_006519564.1;XP_006519565.1;XP_006519566.1;XP_006519567.1;XP_006519568.1;XP_006519569.1;XP_011243478.1;XP_011243479.1;XP_017171669.1	1917140	.	8.611808	[illegible]
69893	NM_027250	Coa7	cytochrome c oxidase assembly factor 7	protein_coding	NP_081526.1	1917143	.	1.765027	[illegible]
69894	NM_001358252,NM_001358253,NM_027251	Fam241b	family with sequence similarity 241, member B	protein_coding	NP_001345181.1;NP_001345182.1;NP_081527.1	1917144	.	0.151244	[illegible]
69895	NR_028574	Snhg8	small nucleolar RNA host gene 8	lncRNA	.	1917145	.	25.366383	[illegible]
69900	NM_001361585,NM_001361586,NM_001361587,NM_178620,XM_006534142,XM_006534143,XM_006534144,XM_006534145,XM_006534146,XM_017314745,XM_030246289,XM_030246290	Mfsd11	major facilitator superfamily domain containing 11	protein_coding	NP_001348514.1;NP_001348515.1;NP_001348516.1;NP_848735.1;XP_006534205.1;XP_006534206.1;XP_006534207.1;XP_006534208.1;XP_006534209.1;XP_017170234.1;XP_030102149.1;XP_030102150.1	1917150	.	15.163459	[illegible]
69902	NM_001290810,NM_001356476,NM_023536	Mrto4	mRNA turnover 4, ribosome maturation factor	protein_coding	NP_001277739.1;NP_001343405.1;NP_076025.1	1917152	.	15.383295	[illegible]
69903	NM_028544	Rasip1	Ras interacting protein 1	protein_coding	NP_082820.1	1917153	.	6.700963	[illegible]
69904	XR_001779376,XR_001779377,XR_001779378,XR_001779379,XR_001779380,XR_001779381,XR_001779382,XR_001779383,XR_001779384,XR_001779385,XR_001779386,XR_001779387,XR_387323,XR_387324,XR_387328,XR_387329,XR_387331,XR_387332,XR_878468,XR_878474,XR_878477	2610027F03Rik	RIKEN cDNA 2610027F03 gene, transcript variant X10	lncRNA	.	1917154	.	0.162070	[illegible]
69906	NM_172402,XM_006521385	Slc25a32	solute carrier family 25, member 32	protein_coding	NP_765990.2;XP_006521448.1	1917156	.	7.868939	[illegible]
69908	NM_023537	Rab3b	RAB3B, member RAS oncogene family	protein_coding	NP_076026.1	1917158	.	0.239771	[illegible]
69909	NR_077059	2610027K06Rik	RIKEN cDNA 2610027K06 gene	lncRNA	.	1917159	.	0.714154	[illegible]
69912	NM_145706	Nup43	nucleoporin 43	protein_coding	NP_663752.2	1917162	.	5.306583	[illegible]
69917	NM_001358972,NM_001358973,NM_027257,XM_006514066,XM_006514067,XM_030245253,XM_030245254	Nabp2	nucleic acid binding protein 2	protein_coding	NP_001345901.1;NP_001345902.1;NP_081533.1;XP_006514129.1;XP_006514130.1;XP_030101113.1;XP_030101114.1	1917167	.	20.736737	[illegible]
69918	NR_038156	2610020C07Rik	RIKEN cDNA 2610020C07 gene	lncRNA	.	1917168	.	0.812567	[illegible]
69920	NM_027259	Polr2i	polymerase (RNA) II (DNA directed) polypeptide I	protein_coding	NP_081535.1	1917170	.	12.183866	[illegible]
69922	NM_001252447,NM_027260,XM_006514801,XM_011243748,XM_030246291	Vrk2	vaccinia related kinase 2	protein_coding	NP_001239376.1;NP_081536.2;XP_006514864.1;XP_011242050.1;XP_030102151.1	1917172	.	4.414973	[illegible]
69923	NM_023538,XM_006506571,XM_011241458	Agk	acylglycerol kinase	protein_coding	NP_076027.1;XP_006506634.1;XP_011239760.1	1917173	.	6.668304	[illegible]
69926	NM_001167746,XM_006534150,XM_006534151,XM_006534152,XR_388524	Dnah17	dynein, axonemal, heavy chain 17	protein_coding	NP_001161218.1;XP_006534213.1;XP_006534214.1;XP_006534215.1	1917176	.	0.038832	[illegible]
69928	NM_027263	Cenps	centromere protein S	protein_coding	NP_081539.1	1917178	.	4.136029	[illegible]
69930	NM_001357811,NM_001357812,NM_027264,XM_006541182,XM_006541183,XM_006541185,XM_030242976,XM_030242977	Zfp715	zinc finger protein 715	protein_coding	NP_001344740.1;NP_001344741.1;NP_081540.3;XP_006541245.1;XP_006541246.1;XP_006541248.1;XP_030098836.1;XP_030098837.1	1917180	.	9.633964	[illegible]
69934	NM_027266,XM_006538242,XM_006538243,XM_006538244,XM_006538245,XM_006538247,XR_001784199	Trmt10b	tRNA methyltransferase 10B	protein_coding	NP_081542.2;XP_006538305.1;XP_006538306.1;XP_006538307.1;XP_006538308.1;XP_006538310.1	1917184	.	2.273182	[illegible]
69938	NM_027268,XM_006506572,XM_011241459	Scrn1	secernin 1	protein_coding	NP_081544.1;XP_006506635.1;XP_011239761.1	1917188	.	3.294030	[illegible]
69940	NM_001289770,NM_001289771,NM_001359370,NM_001359372,NM_001359373,NM_027270,XM_006535214	Exoc1	exocyst complex component 1	protein_coding	NP_001276699.1;NP_001276700.1;NP_001346299.1;NP_001346301.1;NP_001346302.1;NP_081546.1;XP_006535277.1	2445020	.	8.700658	[illegible]
69941	NR_038009	2810408I11Rik	RIKEN cDNA 2810408I11 gene	lncRNA	.	1917191	.	1.526997	[illegible]
69942	NM_153503	Rnf113a1	ring finger protein 113A1	protein_coding	NP_705723.1	1917192	.	1.868873	[illegible]
69944	NM_172403,XM_030246292,XM_030246293,XM_030246294	2810021J22Rik	RIKEN cDNA 2810021J22 gene	protein_coding	NP_765991.1;XP_030102152.1;XP_030102153.1;XP_030102154.1	1917194	.	1.288494	[illegible]
69952	NR_045047,NR_125773	Tunar	Tcl1 upstream neural differentiation associated RNA	lncRNA	.	1917202	.	0.185448	[illegible]
69953	NR_027984	2810025M15Rik	RIKEN cDNA 2810025M15 gene	lncRNA	.	1917203	.	14.578418	[illegible]
69955	NM_001039189,NM_024274,XM_006516769,XM_006516770,XM_011244345,XM_030247402,XM_030247403,XM_030247404,XM_030247405,XM_030247406,XR_003950479	Fars2	phenylalanine-tRNA synthetase 2 (mitochondrial)	protein_coding	NP_001034278.1;NP_077236.1;XP_006516832.1;XP_006516833.1;XP_011242647.1;XP_030103262.1;XP_030103263.1;XP_030103264.1;XP_030103265.1;XP_030103266.1	1917205	.	5.174779	[illegible]
69956	NM_001362068,NM_001362069,NM_027275,NR_155284,NR_155285,XM_006506574,XM_011241461,XR_001785164,XR_001785165,XR_001785166,XR_001785167,XR_868886,XR_868888	Ptcd3	pentatricopeptide repeat domain 3	protein_coding	NP_001348997.1;NP_001348998.1;NP_081551.2;XP_006506637.1;XP_011239763.1	1917206	.	14.415013	[illegible]
69957	NM_001357247,NM_027276,XM_006508882,XM_006508884	Cdc16	CDC16 cell division cycle 16	protein_coding	NP_001344176.1;NP_081552.2;XP_006508945.1;XP_006508947.1	1917207	.	58.266225	[illegible]
69961	NM_027278,XM_006538248	Rpp25l	ribonuclease P/MRP 25 subunit-like	protein_coding	NP_081554.2;XP_006538311.1	1917211	.	12.021184	[illegible]
69962	NM_001355132,NM_027279	Mettl18	methyltransferase like 18	protein_coding	NP_001342061.1;NP_081555.2	1917212	.	1.498167	[illegible]
69964	NR_015493	2810403D21Rik	RIKEN cDNA 2810403D21 gene	lncRNA	.	1917214	.	1.000017	[illegible]
69972	NR_163865	2810425M01Rik	RIKEN cDNA 2810425M01 gene	lncRNA	.	1917222	.	0.000000	[illegible]
69974	NR_033447	2810405F15Rik	RIKEN cDNA 2810405F15 gene	lncRNA	.	1917224	.	0.000000	[illegible]
69976	NM_001291002,NM_175154,XM_006500153,XM_006500154,XM_011239770,XM_011239771,XM_011239772,XM_030252059,XM_030252060	Galk2	galactokinase 2	protein_coding	NP_001277931.1;NP_780363.1;XP_006500216.1;XP_006500217.1;XP_011238072.1;XP_011238073.1;XP_011238074.1;XP_030107919.1;XP_030107920.1	1917226	.	6.892548	[illegible]
69981	NM_133718	Tmem30a	transmembrane protein 30A	protein_coding	NP_598479.1	106402	.	38.238152	[illegible]
69982	NM_001289764,NM_001289767,NM_001289768,NM_183284,XM_011249564	Spink2	serine peptidase inhibitor, Kazal type 2	protein_coding	NP_001276693.2;NP_001276696.1;NP_001276697.1;NP_899107.1;XP_011247866.1	1917232	.	0.375124	[illegible]
69986	NR_040523	1700025N23Rik	RIKEN cDNA 1700025N23 gene	lncRNA	.	1917236	.	0.100110	[illegible]
69987	NM_027283	Spaca9	sperm acrosome associated 9	protein_coding	NP_081559.1	1917237	.	0.211124	[illegible]
69993	NM_001163640,NM_001311133,NM_001311134,NM_023543,XM_006506578,XM_030255589	Chn2	chimerin 2	protein_coding	NP_001157112.1;NP_001298062.1;NP_001298063.1;NP_076032.2;XP_006506641.1;XP_030111449.1	1917243	.	3.353375	[illegible]
69994	NM_023544	Rsc1a1	regulatory solute carrier protein, family 1, member 1	protein_coding	NP_076033.4	3526447	.	0.178382	[illegible]
70003	NR_038042	1700028I16Rik	RIKEN cDNA 1700028I16 gene	lncRNA	.	1917253	.	0.000000	[illegible]
70004	NR_029436	1700028J19Rik	RIKEN cDNA 1700028J19 gene	lncRNA	.	1917254	.	0.332664	[illegible]
70005	NR_040355	Znf41-ps	ZNF41, pseudogene	transcribed_pseudogene	.	1917255	.	1.798583	[illegible]
70008	NM_001130513,NM_027286	Ace2	angiotensin I converting enzyme (peptidyl-dipeptidase A) 2	protein_coding	NP_001123985.1;NP_081562.2	1917258	.	0.136280	[illegible]
70012	NM_144527	Cep85	centrosomal protein 85	protein_coding	NP_653110.3	1917262	.	7.222119	[illegible]
70020	NM_023547	Ino80b	INO80 complex subunit B	protein_coding	NP_076036.2	1917270	.	7.296437	[illegible]
70021	NM_027289,XM_006519507	Nt5dc2	5'-nucleotidase domain containing 2	protein_coding	NP_081565.1;XP_006519570.1	1917271	.	51.094823	[illegible]
70024	NM_001305259,NM_027290,XM_017319252	Mcm10	minichromosome maintenance 10 replication initiation factor	protein_coding	NP_001292188.1;NP_081566.2;XP_017174741.1	1917274	.	2.256847	[illegible]
70025	NM_001146057,NM_001146058,NM_001369273,NM_001369274,NM_001369275,NM_001369276,NM_133348,XM_006539169,XM_030253767,XM_030253768,XM_030253769	Acot7	acyl-CoA thioesterase 7	protein_coding	NP_001139529.2;NP_001139530.1;NP_001356202.1;NP_001356203.1;NP_001356204.1;NP_001356205.1;NP_579926.2;XP_006539232.1;XP_030109627.1;XP_030109628.1;XP_030109629.1	1917275	.	11.951547	[illegible]
70026	NM_027292,XM_017317631	Tspo2	translocator protein 2	protein_coding	NP_081568.1;XP_017173120.1	1917276	.	0.183933	[illegible]
70028	NM_001357118,NM_026700,NM_027293,XM_006523077,XM_006523080,XM_011246143,XM_011246144,XM_011246145,XM_011246146,XM_011246147,XM_011246148,XM_011246149,XM_011246150,XM_017317127,XM_017317128,XM_030249264,XM_030249265,XM_030249266,XR_001781857,XR_001781858,XR_001781860,XR_001781862,XR_001781864,XR_001781865,XR_003951828,XR_003951829,XR_876136,XR_876139	Dop1b	DOP1 leucine zipper like protein B	protein_coding	NP_001344047.1;NP_080976.1;NP_081569.1;XP_006523140.1;XP_006523143.1;XP_011244445.1;XP_011244446.1;XP_011244447.1;XP_011244448.1;XP_011244449.1;XP_011244450.1;XP_011244451.1;XP_011244452.1;XP_017172616.1;XP_017172617.1;XP_030105124.1;XP_030105125.1;XP_030105126.1	1917278	.	4.345094	[illegible]
70031	NM_027294,XM_011242999	Cmtm8	CKLF-like MARVEL transmembrane domain containing 8	protein_coding	NP_081570.1;XP_011241301.1	2447167	.	1.140700	[illegible]
70036	NR_015531,NR_145131,NR_145133	Dancr	differentiation antagonizing non-protein coding RNA	lncRNA	.	1917286	.	8.123203	[illegible]
70040	NR_040423	2610037D02Rik	RIKEN cDNA 2610037D02 gene	lncRNA	.	1917290	.	0.000000	[illegible]
70044	NM_197993,XM_030251074	Tut1	terminal uridylyl transferase 1, U6 snRNA-specific	protein_coding	NP_932110.1;XP_030106934.1	1917294	.	8.348284	[illegible]
70045	NM_001206684	2610528A11Rik	RIKEN cDNA 2610528A11 gene	protein_coding	NP_001193613.1	1917295	.	0.000000	[illegible]
70047	NM_001242358,NM_001242360,NM_027296,XM_006506581,XM_006506582	Trnt1	tRNA nucleotidyl transferase, CCA-adding, 1	protein_coding	NP_001229287.1;NP_001229289.1;NP_081572.1;XP_006506644.1;XP_006506645.1	1917297	.	12.317867	[illegible]
70052	NM_027297,XM_006538249,XM_030253770,XR_390341	Prpf4	pre-mRNA processing factor 4	protein_coding	NP_081573.1;XP_006538312.1;XP_030109630.1	1917302	.	5.716527	[illegible]
70054	NM_027298	Ccdc89	coiled-coil domain containing 89	protein_coding	NP_081574.1	1917304	.	0.219990	[illegible]
70059	NM_001171002,NM_027299	Degs2	delta(4)-desaturase, sphingolipid 2	protein_coding	NP_001164473.1;NP_081575.2	1917309	.	0.111201	[illegible]
70060	NM_001122732,NM_027029,NM_027300,NM_028647,XM_011248071,XM_011248072,XM_030242857,XM_030242858,XM_030242859,XM_030242861,XM_030242863	Spata3	spermatogenesis associated 3	protein_coding	NP_001116204.1;NP_081305.1;NP_081576.2;NP_082923.1;XP_011246373.1;XP_011246374.2;XP_030098717.1;XP_030098718.1;XP_030098719.1;XP_030098721.1;XP_030098723.1	1917310	.	0.000000	[illegible]
70061	NM_027301	Sdr9c7	4short chain dehydrogenase/reductase family 9C, member 7	protein_coding	NP_081577.1	1917311	.	0.016536	[illegible]
70078	NM_023554	Nol7	nucleolar protein 7	protein_coding	NP_076043.2	1917328	.	15.008930	[illegible]
70080	NM_001364024,NM_001364025,NM_027308,XM_006540354,XM_006540355	Igsf23	immunoglobulin superfamily, member 23	protein_coding	NP_001350953.1;NP_001350954.1;NP_081584.1;XP_006540417.1;XP_006540418.1	1917330	.	0.066676	[illegible]
70081	NM_001256493,XM_030250038,XM_030250039,XM_030250040,XM_030250041,XM_030250042	Zfp995	zinc finger protein 995	protein_coding	NP_001243422.1;XP_030105898.1;XP_030105899.1;XP_030105900.1;XP_030105901.1;XP_030105902.1	1917331	.	1.928466	[illegible]
70082	NM_027309,XM_006511431,XR_001779008,XR_003948002	Lysmd2	LysM, putative peptidoglycan-binding, domain containing 2	protein_coding	NP_081585.2;XP_006511494.1	1917332	.	5.663522	[illegible]
70083	NM_133719	Metrn	meteorin, glial cell differentiation regulator	protein_coding	NP_598480.1	1917333	.	9.221346	[illegible]
70086	NM_001162412,NM_133720,XM_011245178,XM_030248001	Cysltr2	cysteinyl leukotriene receptor 2	protein_coding	NP_001155884.1;NP_598481.2;XP_011243480.1;XP_030103861.1	1917336	.	0.043035	[illegible]
70088	NM_001290701,NM_027310,NR_151472,XM_006503382,XR_003955011,XR_003955012,XR_003955013,XR_003955014,XR_376353	Meaf6	MYST/Esa1-associated factor 6	protein_coding	NP_001277630.1;NP_081586.1;XP_006503445.1	1917338	.	9.014676	[illegible]
70093	NM_027315	Ube2q1	ubiquitin-conjugating enzyme E2Q family member 1	protein_coding	NP_081591.3	1917343	.	18.590490	[illegible]
70097	NM_175155,XM_006512837,XM_006512839,XM_006512840,XM_006512841,XM_030245255,XM_030245256,XM_030245257	Sash1	SAM and SH3 domain containing 1	protein_coding	NP_780364.3;XP_006512900.1;XP_006512902.1;XP_006512903.1;XP_006512904.2;XP_030101115.1;XP_030101116.1;XP_030101117.1	1917347	.	17.547539	[illegible]

70099	NM_001356976,NM_133786,XM_006502046,XM_030252812,XM_030252813,XM_030252814	Smc4	structural maintenance of chromosomes 4	protein_coding	NP_001343905.1;NP_598547.1;XP_006502109.1;XP_030108672.1;XP_030108673.1;XP_030108674.1	1917349	.	21.798209
70100	NR_037997,NR_160674	2310015D24Rik	RIKEN cDNA 2310015D24 gene	lncRNA	.	1917350	.	0.080068
70101	NM_001356302,NM_001356303,NM_024442,XM_006524882,XM_006524884,XM_017317633,XR_385364,XR_876529	Cyp4f16	cytochrome P450, family 4, subfamily f, polypeptide 16	protein_coding	NP_001343231.1;NP_001343232.1;NP_077762.1;XP_006524945.1;XP_006524947.1;XP_017173122.1	1917351	.	6.498053
70103	NM_001310747,NM_001310748,NM_027318,XM_006504497	Znhit1	zinc finger, HIT domain containing 1	protein_coding	NP_001297676.1;NP_001297677.1;NP_081594.1;XP_006504560.1	1917353	.	18.059275
70110	NM_027320	Ifi35	interferon-induced protein 35	protein_coding	NP_081596.1	1917360	.	6.220738
70113	NM_001013022,XM_030248762	Odf3b	outer dense fiber of sperm tails 3B	protein_coding	NP_001013040.1;XP_030104622.1	1917363	.	0.000000
70118	NM_001359388,NM_027323,NR_153105,NR_153106	Srrd	SRR1 domain containing	protein_coding	NP_001346317.1;NP_081599.2	1917368	.	4.501077
70120	NM_198246,NR_038046	Yars2	tyrosyl-tRNA synthetase 2 (mitochondrial)	protein_coding	NP_937889.1	1917370	.	4.535673
70122	NM_001286158,NM_027326,NM_029931,XM_006538250,XM_006538253,XM_030253771,XM_030253772,XM_030253773	Mllt3	myeloid/lymphoid or mixed-lineage leukemia; translocated to, 3	protein_coding	NP_001273087.1;NP_081602.3;NP_084207.2;XP_006538313.1;XP_006538316.1;XP_030109631.1;XP_030109632.1;XP_030109633.1	1917372	.	3.554268
70123	NM_027327,XM_030251495	Nbdy	negative regulator of P-body association	protein_coding	NP_081603.1;XP_030107355.1	1917373	.	22.550683
70127	NM_001267625,NM_001267626,NM_058212	Dpf3	D4, zinc and double PHD fingers, family 3	protein_coding	NP_001254554.1;NP_001254555.1;NP_478119.1	1917377	.	0.523294
70129	NM_023557,XM_006524885	Slc44a4	solute carrier family 44, member 4	protein_coding	NP_076046.1;XP_006524948.1	1917379	.	0.077814
70134	NM_001291292	Misp3	RIKEN cDNA 2210011C24 gene	protein_coding	NP_001278221.1	1917384	.	0.047322
70138	NR_028285	2210417A02Rik	RIKEN cDNA 2210417A02 gene	lncRNA	.	1917388	.	0.000000
70144	NM_001081255,NM_001310672,NM_001310673,NM_001310674,XM_006522524,XM_006522525,XM_006522526,XM_006522527,XM_006522528,XM_006522530,XM_006522531,XM_006522533,XM_030249268,XM_030249269,XM_030249270,XM_030249271,XM_030249272,XR_001781866	Lrch3	leucine-rich repeats and calponin homology (CH) domain containing 3	protein_coding	NP_001074724.1;NP_001297601.1;NP_001297602.1;NP_001297603.1;XP_006522587.1;XP_006522588.1;XP_006522589.1;XP_006522590.1;XP_006522591.1;XP_006522593.1;XP_006522594.1;XP_006522596.1;XP_030105128.1;XP_030105129.1;XP_030105130.1;XP_030105131.1;XP_030105132.1	1917394	.	16.862555
70152	NM_027334,XM_006521389,XM_006521390,XM_011245728	Mettl7a1	methyltransferase like 7A1	protein_coding	NP_081610.2;XP_006521452.1;XP_006521453.1;XP_011244030.1	1916523	.	7.048791
70153	NM_027335	2210016F16Rik	RIKEN cDNA 2210016F16 gene	protein_coding	NP_081611.1	1917403	.	5.791436
70155	NM_001081079,XM_006496261	Ogfrl1	opioid growth factor receptor-like 1	protein_coding	NP_001074548.1;XP_006496324.1	1917405	.	1.718183
70160	NM_001368379,NM_027338	Vps36	vacuolar protein sorting 36	protein_coding	NP_001355308.1;NP_081614.1	1917410	.	8.197542
70162	NR_038038	Hk1os	hexokinase 1, opposite strand	lncRNA		1917412	.	0.922592
70166	NM_027340,XM_006527333	Lipn	lipase, family member N	protein_coding	NP_081616.1;XP_006527396.1	1917416	.	0.000000
70178	NM_133722	Abhd17c	abhydrolase domain containing 17C	protein_coding	NP_598483.2	1917428	.	6.150625
70186	NM_027342	Fam162a	family with sequence similarity 162, member A	protein_coding	NP_081618.1	1917436	.	38.363979
70192	NM_027343,XM_006508885	Cd209g	CD209g antigen	protein_coding	NP_081619.3;XP_006508948.1	1917442	.	0.000000
70194	NR_131053	2310075C17Rik	RIKEN cDNA 2310075C17 gene	lncRNA		1917444	.	0.166113
70207	NM_027346,XR_003949501	Taco1	translational activator of mitochondrially encoded cytochrome c oxidase I	protein_coding	NP_081622.1	1917457	.	5.495457
70208	NM_001166416,NM_027347	Med23	mediator complex subunit 23	protein_coding	NP_001159888.1;NP_081623.3	1917458	.	8.988841
70209	NM_144801,XM_006541188,XM_006541189,XM_006541190,XM_030242978,XR_003946562	Tmem143	transmembrane protein 143	protein_coding	NP_659050.2;XP_006541251.1;XP_006541252.1;XP_006541253.1;XP_030098838.1	1917459	.	2.665397
70218	NM_197959,XM_011249243	Kif18b	kinesin family member 18B	protein_coding	NP_932063.2;XP_011247545.1	2446979	.	3.612364
70223	NM_001142950,NM_027350	Nars	asparaginyl-tRNA synthetase	protein_coding	NP_001136422.1;NP_081626.2	1917473	.	44.217194
70225	NM_001285826,NM_001285827,NM_027351,NM_027374,XM_011238590,XM_011238591,XM_011238592,XM_011238593,XM_011238594,XM_011238595,XM_011238596,XM_030242882,XM_030242886,XM_030242888,XM_030242891	Ppil3	peptidylprolyl isomerase (cyclophilin)-like 3	protein_coding	NP_001272755.1;NP_001272756.1;NP_081627.1;NP_081650.2;XP_011236892.1;XP_011236893.1;XP_011236894.1;XP_011236895.1;XP_011236896.1;XP_011236897.1;XP_011236898.1;XP_030098742.1;XP_030098746.1;XP_030098748.1;XP_030098751.1	1917475	.	8.886978
70226	NR_033561	Rbm3os	RNA binding motif protein 3, opposite strand	lncRNA	.	1917476	.	0.291749
70227	NM_001004139	Zfp619	zinc finger protein 619	protein_coding	NP_001004139.2	1917477	.	0.980134
70230	NR_126426	3300002P13Rik	RIKEN cDNA 3300002P13 gene	lncRNA	.	1917480	.	0.503237
70231	NM_027352,NR_027343	Gorasp2	golgi reassembly stacking protein 2	protein_coding	NP_081628.3	2135962	.	74.765376
70233	NM_001285905,NM_001285906,NM_001285907,NM_001357817,NM_027353,XM_006508198,XM_030242979	Cd2bp2	CD2 cytoplasmic tail binding protein 2	protein_coding	NP_001272834.1;NP_001272835.1;NP_001272836.1;NP_001344746.1;NP_081629.1;XP_006508261.1;XP_030098839.1	1917483	.	18.777500
70235	NM_027354,XM_006511830,XM_006511831	Poc1a	POC1 centriolar protein A	protein_coding	NP_081630.2;XP_006511893.1;XP_006511894.1	1917485	.	2.220830
70237	NM_001098222,NM_198161,XM_006528607,XM_006528608,XM_011247760	Bhlhb9	basic helix-loop-helix domain containing, class B9	protein_coding	NP_001091692.1;NP_937804.1;XP_006528670.1;XP_006528671.1;XP_011246062.1	1917487	.	9.186101
70238	NM_027355	Rnf168	ring finger protein 168	protein_coding	NP_081631.2	1917488	.	8.091317
70239	NM_001290484,NM_148928,XM_011239177,XM_017319253	Gtf3c5	general transcription factor IIIC, polypeptide 5	protein_coding	NP_001277413.1;NP_683730.1;XP_011237479.1;XP_017174742.1	1917489	.	9.149223
70240	NM_027356	Ufsp1	UFM1-specific peptidase 1	protein_coding	NP_081632.1	1917490	.	1.788980
70247	NM_027357	Psmd1	proteasome (prosome, macropain) 26S subunit, non-ATPase, 1	protein_coding	NP_081633.1	1917497	.	32.676693
70248	NM_001122604,NM_001122605,NM_133188,XM_006514069,XM_006514070,XM_006514071,XM_006514072,XM_006514073,XM_006514074,XM_011243559,XM_030245258,XM_030245259,XM_030245260,XM_030245261,XM_030245262	Dazap1	DAZ associated protein 1	protein_coding	NP_001116076.1;NP_001116077.1;NP_573451.2;XP_006514132.1;XP_006514133.1;XP_006514134.1;XP_006514135.1;XP_006514136.1;XP_006514137.1;XP_011241861.1;XP_030101118.1;XP_030101119.1;XP_030101120.1;XP_030101121.1;XP_030101122.1	1917498	.	42.455572
70252	NR_015508	Jpx	Jpx transcript, Xist activator (non-protein coding)	lncRNA		2180008	.	0.689996
70257	NM_001361914,NM_027360	Atp5mpl	ATP synthase membrane subunit 6.8PL	protein_coding	NP_001348843.1;NP_081636.1	1917507	.	76.355741
70258	XR_140695	1500035N22Rik	RIKEN cDNA 1500035N22 gene	lncRNA		1917508	.	0.000000
70261	NM_027363,XM_011241899	Chp2	calcineurin-like EF hand protein 2	protein_coding	NP_081639.1;XP_011240201.1	1917511	.	1.676452
70266	NM_001356474,NM_001356475,NM_172404,XM_006498315,XM_006498317,XM_030252061,XR_001783175,XR_003953717	Kyat1	kynurenine aminotransferase 1	protein_coding	NP_001343403.1;NP_001343404.1;NP_765992.2;XP_006498378.1;XP_006498380.1;XP_030107921.1	1917516	.	3.747739
70274	NM_027366,XM_006524886,XM_006524887,XM_030250043,XM_030250044	Ly6g6e	lymphocyte antigen 6 complex, locus G6E	protein_coding	NP_081642.1;XP_006524949.1;XP_006524950.1;XP_030105903.1;XP_030105904.1	1917524	.	0.118235
70281	NR_028124	2310068J16Rik	RIKEN cDNA 2310068J16 gene	lncRNA	.	1917531	.	0.371258
70285	NM_027332,NM_027371	Rpf1	ribosome production factor 1 homolog	protein_coding	NP_081608.1;NP_081647.2	1917535	.	13.673231
70291	NM_001101431	Mkrn2os	makorin, ring finger protein 2, opposite strand	protein_coding	NP_001094901.1	1917541	.	0.000000
70292	NM_027373,XM_006504098,XM_030254799,XM_030254800	Afap1	actin filament associated protein 1	protein_coding	NP_081649.1;XP_006504161.1;XP_030110659.1;XP_030110660.1	1917542	.	10.515377
70294	NM_144528,NR_027505,XM_006514075,XM_017314081,XR_001779593,XR_003948796	Rnf126	ring finger protein 126	protein_coding	NP_653111.1;XP_006514138.1;XP_017169570.1	1917544	.	15.637027
70296	NM_146252,XM_006498320,XM_006498321,XM_006498322,XM_006498323,XM_017319254,XM_030252062,XM_030252063,XM_030252064,XM_030252065	Tbc1d13	TBC1 domain family, member 13	protein_coding	NP_666364.1;XP_006498383.1;XP_006498384.1;XP_006498385.1;XP_006498386.1;XP_017174743.1;XP_030107922.1;XP_030107923.1;XP_030107924.1;XP_030107925.1	2385326	.	13.016366
70297	NM_027375,XM_017314082,XR_003948797	Gcc2	GRIP and coiled-coil domain containing 2	protein_coding	NP_081651.2;XP_017169571.1	1917547	.	5.469874
70300	NM_027376,XM_006541192,XM_006541193,XM_006541194	Fuz	fuzzy planar cell polarity protein	protein_coding	NP_081652.2;XP_006541255.1;XP_006541256.1;XP_006541257.1	1917550	.	5.642434
70310	NM_001168497,NM_023564,NR_033133,XM_006534156,XM_006534158	Plscr3	phospholipid scramblase 3	protein_coding	NP_001161969.1;NP_076053.1;XP_006534219.1;XP_006534221.1	1917560	.	22.350442
70312	NM_027381	Cactin	cactin, spliceosome C complex subunit	protein_coding	NP_081657.1	1917562	.	9.284858
70314	NM_030566,XM_030242980,XR_378268	Rabep2	rabaptin, RAB GTPase binding effector protein 2	protein_coding	NP_085043.2;XP_030098840.1	1917564	.	8.633234
70315	NM_001313742,NM_027382,XM_006528275,XM_006528276,XM_011247670,XM_011247671,XM_011247673	Hdac8	histone deacetylase 8	protein_coding	NP_001300671.1;NP_081658.1;XP_006528338.1;XP_006528339.1;XP_011245972.1;XP_011245973.1;XP_011245975.1	1917565	.	5.853550
70316	NM_001360743,NM_001360744,NM_028177,NR_153798	Ndufab1	NADH:ubiquinone oxidoreductase subunit AB1	protein_coding	NP_001347672.1;NP_001347673.1;NP_082453.2	1917566	.	34.916143
70317	NM_197995	Arl16	ADP-ribosylation factor-like 16	protein_coding	NP_932112.2	1917567	.	7.058654
70319	XR_003954007	2600006K01Rik	RIKEN cDNA 2600006K01 gene	lncRNA	.	1917569	.	1.351440
70325	NM_001077636,NM_001363112,NM_027388	Pigw	phosphatidylinositol glycan anchor biosynthesis, class W	protein_coding	NP_001071104.1;NP_001350041.1;NP_081664.2	1917575	.	2.156362
70333	NM_145822	Cd3eap	CD3E antigen, epsilon polypeptide associated protein	protein_coding	NP_665821.1	1917583	.	6.865798
70335	NM_001204931,NM_139292,XM_006514077,XM_006514078	Reep6	receptor accessory protein 6	protein_coding	NP_001191860.1;NP_647453.1;XP_006514140.1;XP_006514141.1	1917585	.	0.541206
70337	NM_027391	Iyd	iodotyrosine deiodinase	protein_coding	NP_081667.1	1917587	.	0.999384
70345	NR_028125	0610038B21Rik	RIKEN cDNA 0610038B21 gene	lncRNA	.	1917595	.	0.057528
70348	NM_027394,XM_006511432	Ube2cbp	ubiquitin-conjugating enzyme E2C binding protein	protein_coding	NP_081670.1;XP_006511495.1	1917598	.	1.096458
70349	NM_033370	Copb1	coatomer protein complex, subunit beta 1	protein_coding	NP_203534.1	1917599	.	62.531414
70350	NM_027395	Basp1	brain abundant, membrane attached signal protein 1	protein_coding	NP_081671.1	1917600	.	28.164009
70351	NM_001114131,NM_146081,NR_104465,NR_152099,XM_006524888,XM_006524889,XM_006524890,XM_006524891,XM_006524892,XM_006524895,XM_030250045,XM_030250046,XM_030250047,XR_001782110,XR_003952177	Ppp4r1	protein phosphatase 4, regulatory subunit 1	protein_coding	NP_001107603.1;NP_666193.2;XP_006524951.1;XP_006524952.1;XP_006524953.1;XP_006524954.1;XP_006524955.1;XP_006524958.1;XP_030105905.1;XP_030105906.1;XP_030105907.1	1917601	.	19.369090
70354	NM_001369056,NM_177608,XM_006500156	Secisbp2l	SECIS binding protein 2-like	protein_coding	NP_001355985.1;NP_808276.2;XP_006500219.1	1917604	.	11.586212
70355	NM_001110337,NM_001110338,NM_147217,XM_006534161,XM_006534162,XM_006534163,XM_006534164,XM_030246296	Gprc5c	G protein-coupled receptor, family C, group 5, member C	protein_coding	NP_001103807.1;NP_001103808.1;NP_671750.2;XP_006534224.1;XP_006534225.1;XP_006534226.1;XP_006534227.1;XP_030102156.1	1917605	.	23.611347
70356	NM_133726	St13	suppression of tumorigenicity 13	protein_coding	NP_598487.1	1917606	.	46.484718
70357	NM_001190885,NM_001190886,NM_001290690,NM_001363115,NM_027398,XM_017314751,XM_017314752,XM_017314753,XM_017314754,XM_017314755,XM_030246297,XM_030246298,XM_030246299	Kcnip1	Kv channel-interacting protein 1	protein_coding	NP_001177814.1;NP_001177815.1;NP_001277619.1;NP_001350044.1;NP_081674.2;XP_017170240.1;XP_017170241.1;XP_017170242.1;XP_017170243.1;XP_017170244.1;XP_030102157.1;XP_030102158.1;XP_030102159.1	1917607	.	0.048499
70358	NM_027399	Steap1	six transmembrane epithelial antigen of the prostate 1	protein_coding	NP_081675.2	1917608	.	3.402759
70359	NM_032544,NR_157144,NR_157145,NR_157146,NR_157147,XM_006509737,XR_379045	Gtpbp3	GTP binding protein 3	protein_coding	NP_115933.2;XP_006509800.1	1917609	.	8.917564
70361	NM_001172062,NM_027400	Lman1	lectin, mannose-binding, 1	protein_coding	NP_001165533.1;NP_081676.1	1917611	.	88.438122
70362	NM_001171640	Actl10	actin-like 10	protein_coding	NP_001165111.1	1917612	.	0.000000
70363	NM_177819,XM_006521393,XM_006521395,XR_003951438	Fam135b	family with sequence similarity 135, member B	protein_coding	NP_808487.2;XP_006521456.1;XP_006521458.1	1917613	.	0.010447
70365	NR_001582,NR_027506	Speer5-ps1	spermatogenesis associated glutamate (E)-rich protein 5, pseudogene 1	transcribed_pseudogene	.	1917615	.	0.000000
70369	NM_001324480,NM_001324481,NM_001324482,NM_027404,XM_006516217,XM_030246902	Bag5	BCL2-associated athanogene 5	protein_coding	NP_001311409.1;NP_001311410.1;NP_001311411.1;NP_081680.1;XP_006516280.1;XP_030102762.1	1917619	.	17.935875
70370	NM_024237,XM_006500157	Fbln7	fibulin 7	protein_coding	NP_077199.2;XP_006500220.1	1917620	.	14.927390
70373	NM_001324488,NM_027405,XM_006516219,XM_006516220,XM_006516222,XM_017315181,XM_030246903,XM_030246904	Gpatch2l	G patch domain containing 2 like	protein_coding	NP_001311417.1;NP_081681.2;XP_006516282.1;XP_006516283.1;XP_006516285.1;XP_017170670.1;XP_030102763.1;XP_030102764.1	1917623	.	7.939297
70375	NM_001316694,NM_001316695,NM_001357296,NM_027407,XM_006496270,XM_006496272,XM_011238597,XM_017312238,XM_017312240,XM_017312256,XM_017312257,XM_030242893,XM_030242894,XM_030242895,XM_030242896,XM_030242897,XM_030242898,XM_030242900	Ica1l	islet cell autoantigen 1-like	protein_coding	NP_001303623.1;NP_001303624.1;NP_001344225.1;NP_081683.3;XP_006496333.1;XP_006496335.1;XP_011236899.1;XP_017167727.1;XP_017167729.1;XP_017167745.1;XP_017167746.1;XP_030098753.1;XP_030098754.1;XP_030098755.1;XP_030098756.1;XP_030098757.1;XP_030098758.1;XP_030098760.1	1917625	.	0.056889
70377	NM_001358983,NM_001358984,NM_001358985,NM_024440,NR_152573,NR_152574	Derl3	Der1-like domain family, member 3	protein_coding	NP_001345912.1;NP_001345913.1;NP_001345914.1;NP_077760.1	1917627	.	0.312308
70380	NM_001290514,NM_001358456,NM_027409,NR_110964,XM_006541538,XM_006541540,XM_006541541,XM_011251017,XM_017318617,XM_030251496,XR_882288	Mospd1	motile sperm domain containing 1	protein_coding	NP_001277443.1;NP_001345385.1;NP_081685.1;XP_006541601.1;XP_006541603.1;XP_006541604.1;XP_011249319.1;XP_017174106.1;XP_030107356.1	1917630	.	8.840325
70381	NM_001356992,NM_001356993,NM_027410,XM_006504864	Tecpr1	tectonin beta-propeller repeat containing 1	protein_coding	NP_001343921.1;NP_001343922.1;NP_081686.1;XP_006504927.1	1917631	.	5.946765
70382	NM_183285,XM_006534166,XM_011249244,XR_003949502,XR_003949503,XR_388525	Kctd2	potassium channel tetramerisation domain containing 2	protein_coding	NP_899108.3;XP_006534229.1;XP_011247546.1	1917632	.	15.891791
70383	NM_178379,XM_006534168	Cox10	heme A:farnesyltransferase cytochrome c oxidase assembly factor 10	protein_coding	NP_848466.1;XP_006534231.1	1917633	.	3.785290
70385	NM_027411,XM_006534169,XM_011249245	Spdl1	spindle apparatus coiled-coil protein 1	protein_coding	NP_081687.2;XP_006534232.1;XP_011247547.1	1917635	.	2.564904
70387	NM_027412,XR_386507	Ttc9c	tetratricopeptide repeat domain 9C	protein_coding	NP_081688.2	1917637	.	12.635284
70392	NM_001313743,NM_080858,XM_011247674	Asb12	ankyrin repeat and SOCS box-containing 12	protein_coding	NP_001300672.1;NP_543134.1;XP_011245976.1	1917642	.	1.015719
70393	NR_045499	2210416O15Rik	RIKEN cDNA 2210416O15 gene	lncRNA	.	1917643	.	0.069462
70394	NM_133727	Kptn	kaptin	protein_coding	NP_598488.2	1890380	.	6.896477
70396	NM_001290984,NM_133728	Asnsd1	asparagine synthetase domain containing 1	protein_coding	NP_001277913.1;NP_598489.2	1917646	.	18.992950
70397	NM_026392,NM_027415	Tmem70	transmembrane protein 70	protein_coding	NP_080668.1;NP_081691.2	1915068	.	12.240002
70405	NM_027416	Calml3	calmodulin-like 3	protein_coding	NP_081692.1	1917655	.	2.150526
70408	NM_029763	Polr3f	polymerase (RNA) III (DNA directed) polypeptide F	protein_coding	NP_084039.2	1924086	.	6.365871
70415	NM_001313744,NM_133729,XM_017318618	Stk26	serine/threonine kinase 26	protein_coding	NP_001300673.1;NP_598490.1;XP_017174107.1	1917665	.	8.524698
70417	NM_001001979	Megf10	multiple EGF-like-domains 10	protein_coding	NP_001001979.1	2685177	.	3.433400
70419	NM_001316696,NM_001316697,NM_001316698,NM_001363161,NM_027419,XM_006534171,XM_006534174,XM_011249246,XR_003949504,XR_388526	2810408A11Rik	RIKEN cDNA 2810408A11 gene	protein_coding	NP_001303625.1;NP_001303626.1;NP_001303627.1;NP_001350090.1;NP_081695.2;XP_006534234.1;XP_006534237.1;XP_011247548.1	1917669	.	1.690601
70420	NM_027420	Arpin	actin-related protein 2/3 complex inhibitor	protein_coding	NP_081696.1	1917670	.	10.500758
70422	NM_001363129,NM_001363130,NM_027421,XM_006534176,XM_006534177,XM_006534178,XM_006534179,XM_006534180,XM_006534181,XM_011249248,XM_030246300,XR_879737	Ints2	integrator complex subunit 2	protein_coding	NP_001350058.1;NP_001350059.1;NP_081697.2;XP_006534239.1;XP_006534240.1;XP_006534241.1;XP_006534242.1;XP_006534243.1;XP_006534244.1;XP_011247550.1;XP_030102160.1	1917672	.	5.310938
70423	NM_197996,XM_006514080,XM_011243560,XM_011243561	Tspan15	tetraspanin 15	protein_coding	NP_932113.2;XP_006514143.1;XP_011241862.1;XP_011241863.1	1917673	.	1.845962
70425	NM_152809,XM_006526230,XM_006526231,XM_006526232,XM_006526233,XM_006526234,XM_006526235,XM_006526236,XM_006526237	Csnk1g3	casein kinase 1, gamma 3	protein_coding	NP_690022.2;XP_006526293.1;XP_006526294.1;XP_006526295.1;XP_006526296.1;XP_006526297.1;XP_006526298.1;XP_006526299.1;XP_006526300.1	1917675	.	16.726972
70426	NM_001291001	Tekt5	tektin 5	protein_coding	NP_001277930.1	1917676	.	0.000000
70427	NM_001316699,NM_027422,XM_006514081,XM_006514082,XM_006514083,XM_006514085,XM_006514086,XM_017314086,XM_030245263,XM_030245264,XM_030245265	Mier2	MIER family member 2	protein_coding	NP_001303628.1;NP_081698.2;XP_006514144.1;XP_006514145.1;XP_006514146.1;XP_006514148.1;XP_006514149.1;XP_017169575.1;XP_030101123.1;XP_030101124.1;XP_030101125.1	1917677	.	8.860452
70428	NM_027423,XR_380439	Polr3b	polymerase (RNA) III (DNA directed) polypeptide B	protein_coding	NP_081699.1	1917678	.	4.573187
70430	NM_178337,NR_130788,XM_006516773,XM_006516775,XM_030247407,XR_001780813,XR_003950480,XR_003950481,XR_003950482,XR_873135	Tbce	tubulin-specific chaperone E	protein_coding	NP_848027.1;XP_006516836.1;XP_006516838.1;XP_030103267.1	1917680	.	12.036683
70432	NM_027425,XM_006514087,XM_006514088,XM_011243562,XR_003948799,XR_003948800,XR_003948801,XR_003948802,XR_871	Rufy2	RUN and FYVE domain-containing 2	protein_coding	NP_081701.2;XP_006514150.1;XP_006514151.1;XP_011241864.1	1917682	.	6.081437
70433	NM_027426,XM_006539170	Draxin	dorsal inhibitory axon guidance protein	protein_coding	NP_081702.2;XP_006539233.1	1917683	.	0.118885
70435	NM_198411,XM_006516224,XM_030246905,XM_030246906,XM_030246907,XM_030246908,XM_030246909,XM_030246910,XR_003950077,XR_003950078	Inf2	inverted formin, FH2 and WH2 domain containing	protein_coding	NP_940803.2;XP_006516287.2;XP_030102765.1;XP_030102766.1;XP_030102767.1;XP_030102768.1;XP_030102769.1;XP_030102770.1	1917685	.	15.841780
70439	NM_027427,XM_006534185,XM_006534187,XM_030246301,XM_030246302,XM_030246303,XM_030246304,XM_030246305,XM_030246306	Taf15	TATA-box binding protein associated factor 15	protein_coding	NP_081703.1;XP_006534248.1;XP_006534250.1;XP_030102161.1;XP_030102162.1;XP_030102163.1;XP_030102164.1;XP_030102165.1;XP_030102166.1	1917689	.	22.368660
70441	NR_033490	Mir124-2hg	Mir124-2 host gene (non-protein coding)	lncRNA	.	1917691	.	0.000000
70445	NM_054042	Cd248	CD248 antigen, endosialin	protein_coding	NP_473383.1	1917695	.	20.692776
70448	NR_027971,NR_027972	Atad3aos	ATPase family, AAA domain containing 3A, opposite strand	lncRNA	.	1917698	.	0.671170
70450	NM_001009573,XM_006534188,XM_006534189,XM_006534190,XM_006534192,XR_879738	Unc13d	unc-13 homolog D	protein_coding	NP_001009573.2;XP_006534251.1;XP_006534252.1;XP_006534253.1;XP_006534255.1	1917700	.	0.241359
70451	NM_183286	Dhrs13	dehydrogenase/reductase (SDR family) member 13	protein_coding	NP_899109.2	1917701	.	3.510686
70454	NM_001159930,NR_131028,NR_131029,NR_131030	Cenpl	centromere protein L	protein_coding	NP_001153402.1	1917704	.	3.500324
70456	NM_027430	Mpc2	mitochondrial pyruvate carrier 2	protein_coding	NP_081706.1	1917706	.	24.765684
70458	NM_001358255,NM_183287,XM_006522537,XM_006522539,XM_006522540,XM_006522541,XM_030249273	2610318N02Rik	RIKEN cDNA 2610318N02 gene	protein_coding	NP_001345184.1;NP_899110.2;XP_006522600.1;XP_006522602.1;XP_006522603.1;XP_006522604.1;XP_030105133.1	1917708	.	0.698410
70461	NM_173863,XM_006541195,XM_030242981	Crtc3	CREB regulated transcription coactivator 3	protein_coding	NP_776288.2;XP_006541258.1;XP_030098841.1	1917711	.	5.672462
70465	NM_027432	Wdr77	WD repeat domain 77	protein_coding	NP_081708.1	1917715	.	14.560339
70466	NM_181589,XM_006500158,XM_030252067	Ckap2l	cytoskeleton associated protein 2-like	protein_coding	NP_853620.1;XP_006500221.1;XP_030107927.1	1917716	.	9.501449
70470	NM_001291134,NM_001291135,NM_001291136,NM_027434,XM_006500162,XM_006500163,XM_011239773,XM_030252069	Rprd1b	regulation of nuclear pre-mRNA domain containing 1B	protein_coding	NP_001278063.1;NP_001278064.1;NP_001278065.1;NP_081710.1;XP_006500225.1;XP_006500226.1;XP_011238075.1;XP_030107929.1	1917720	.	6.785593
70472	NM_027435,XR_384045	Atad2	ATPase family, AAA domain containing 2	protein_coding	NP_081711.2	1917722	.	8.515110
70478	NM_027436,NR_040642,XM_006519508	Mipep	mitochondrial intermediate peptidase	protein_coding	NP_081712.2;XP_006519571.1	1917728	.	5.803552
70481	NM_027438	Pnma1	paraneoplastic antigen MA1	protein_coding	NP_081714.2	2180564	.	0.019587
70484	NM_001001321,XM_006517372,XM_006517373,XM_006517374	Slc35d2	solute carrier family 35, member D2	protein_coding	NP_001001321.2;XP_006517435.1;XP_006517436.1;XP_006517437.	1917734	.	1.846307
70487	NR_040378	5730403I07Rik	RIKEN cDNA 5730403I07 gene	lncRNA	.	1917737	.	0.000000
70489	NR_038158	5730405O15Rik	RIKEN cDNA 5730405O15 gene	lncRNA		1917739	.	0.555552
70494	XR_105990	5730409K12Rik	RIKEN cDNA 5730409K12 gene	misc_RNA	.	1917744	.	0.000000
70495	NM_027439	Atp6ap2	ATPase, H+ transporting, lysosomal accessory protein 2	protein_coding	NP_081715.1	1917745	.	22.537132
70497	NM_001122640,NM_001122641,NM_001122642,NM_001122643,NM_001316713,NM_144529,XM_006508201,XM_030242982,XR_869745	Arhgap17	Rho GTPase activating protein 17	protein_coding	NP_001116112.1;NP_001116113.1;NP_001116114.1;NP_001116115.1;NP_001303642.1;NP_653112.2;XP_006508264.1;XP_030098842.1	1917747	.	10.918533
70503	NM_001316716,NM_001316717,NM_001316718,NM_001316719,NM_027442,NR_133568,XM_030245266,XM_030245267,XM_030245268,XM_030245269	Ddo	D-aspartate oxidase	protein_coding	NP_001303645.1;NP_001303646.1;NP_001303647.1;NP_001303648.1;NP_081718.2;XP_030101126.1;XP_030101127.1;XP_030101128.1;XP_030101129.1	1925528	.	0.739851
70508	NM_001347240,NM_027444,XM_006522543,XM_006522544,XM_006522545,XM_006522546,XM_006522547,XM_006522548,XM_006522549,XM_006522553,XM_006522554,XM_006522555,XM_006522560,XM_006522561,XM_011246005,XM_011246006,XM_011246007,XM_011246008,XM_011246009,XM_017317130,XM_030249274,XM_030249275,XM_030249276,XM_030249277,XM_030249278,XM_030249279,XM_030249280,XM_030249281	Bbx	bobby sox HMG box containing	protein_coding	NP_001334169.1;NP_081720.2;XP_006522606.1;XP_006522607.1;XP_006522608.1;XP_006522609.1;XP_006522610.1;XP_006522611.1;XP_006522612.1;XP_006522616.1;XP_006522617.1;XP_006522618.1;XP_006522623.1;XP_006522624.1;XP_011244307.1;XP_011244308.1;XP_011244309.1;XP_011244310.1;XP_011244311.1;XP_017172619.1;XP_030105134.1;XP_030105135.1;XP_030105136.1;XP_030105137.1;XP_030105138.1;XP_030105139.1;XP_030105140.1;XP_03	1917758	.	5.462556
70510	NM_001316728,NM_001363134,NM_027445,XM_006534193,XM_006534195,XR_001780049	Rnf167	ring finger protein 167	protein_coding	NP_001303657.1;NP_001350063.1;NP_081721.1;XP_006534256.1;XP_006534258.1	1917760	.	27.085453
70511	NM_027446	Eef2kmt	eukaryotic elongation factor 2 lysine methyltransferase	protein_coding	NP_081722.1	1917761	.	11.530900
70523	NR_045338,NR_045339	5730420D15Rik	RIKEN cDNA 5730420D15 gene	lncRNA	.	1917773	.	0.110922
70527	NM_001362078,NM_001362079,NM_001362080,NM_024239,XM_006506583,XM_006506584,XM_030255590	Stambp	STAM binding protein	protein_coding	NP_001349007.1;NP_001349008.1;NP_001349009.1;NP_077201.1;XP_006506646.1;XP_006506647.1;XP_030111450.1	1917777	.	9.818114
70530	NM_027452,XM_006524896	Lrfn2	leucine rich repeat and fibronectin type III domain containing 2	protein_coding	NP_081728.2;XP_006524959.1	1917780	.	0.157950
70533	NM_001356361,NM_027453,XM_006503385,XM_006503390,XM_011240610,XM_011240611,XM_011240612	Btf3l4	basic transcription factor 3-like 4	protein_coding	NP_001343290.1;NP_081729.1;XP_006503448.1;XP_006503453.1;XP_011238912.1;XP_011238913.1;XP_011238914.1	1915312	.	18.617551
70536	NM_001316729,NM_027455	Qpct	glutaminyl-peptide cyclotransferase (glutaminyl cyclase)	protein_coding	NP_001303658.1;NP_081731.1	1917786	.	3.848976
70538	NR_045341	5730435O14Rik	RIKEN cDNA 5730435O14 gene	lncRNA	.	1917788	.	0.138749
70544	NM_027457	Tmem242	transmembrane protein 242	protein_coding	NP_081733.3	1917794	.	29.120058
70546	NM_001357249,NM_178395,XM_006509485	Zdhhc2	zinc finger, DHHC domain containing 2	protein_coding	NP_001344178.1;NP_848482.1;XP_006509548.1	1923452	.	0.916050
70549	NM_001081242,XM_006511433,XM_006511434,XM_006511438,XM_006511441,XM_011242811,XM_011242812,XM_011242813,XM_030244583	Tln2	talin 2	protein_coding	NP_001074711.2;XP_006511496.1;XP_006511497.1;XP_006511501.1;XP_006511504.1;XP_011241113.1;XP_011241114.1;XP_011241115.1;XP_030100443.1	1917799	.	5.458663
70551	NM_001360598,NM_001360599,NM_028651,NR_153663,XM_006519510,XM_006519511,XM_017316182,XM_017316183,XM_017316185,XM_030248003,XM_030248004,XM_030248005,XM_030248006,XM_030248007,XM_030248008,XM_030248009,XM_030248010,XM_030248012	Tmtc4	transmembrane and tetratricopeptide repeat containing 4	protein_coding	NP_001347527.1;NP_001347528.1;NP_082927.1;XP_006519573.1;XP_006519574.1;XP_017171671.1;XP_017171672.1;XP_017171674.1;XP_030103863.1;XP_030103864.1;XP_030103865.1;XP_030103866.1;XP_030103867.1;XP_030103868.1;XP_030103869.1;XP_030103870.1;XP_030103872.1	1921050	.	4.893394
70552	NM_001172064,NM_001172065,NM_001357825,NM_153777,XM_006536240,XM_006536241,XM_006536242,XM_006536244,XM_006536245,XM_006536246,XM_006536247,XM_017312294,XM_030242983,XM_030242984,XM_030242985,XM_030242986	Lrrc56	leucine rich repeat containing 56	protein_coding	NP_001165535.1;NP_001165536.1;NP_001344754.1;NP_722472.1;XP_006536303.1;XP_006536304.1;XP_006536305.1;XP_006536307.1;XP_006536308.1;XP_006536309.1;XP_006536310.1;XP_017167783.1;XP_030098843.1;XP_030098844.1;XP_030098845.1;XP_03009884	1917802	.	2.040261
70556	NM_027460	Slc25a33	solute carrier family 25, member 33	protein_coding	NP_081736.2	1917806	.	1.451493
70560	NM_027462,XM_006502052,XM_006502053,XM_017319717,XM_030252815,XR_001783742	Wars2	tryptophanyl tRNA synthetase 2 (mitochondrial)	protein_coding	NP_081738.2;XP_006502115.1;XP_006502116.1;XP_017175206.1;XP_030108675.1	1917810	.	2.994270
70561	NM_001310534,NM_172597,XM_006519520,XM_006519526,XM_006519527,XM_011245180,XM_011245182,XM_011245183,XM_011245184,XM_011245185,XM_017316186,XM_017316187,XM_030248013,XM_030248014,XM_030248015,XM_030248016,XR_001781156,XR_001781157	Txndc16	thioredoxin domain containing 16	protein_coding	NP_001297463.1;NP_766185.1;XP_006519583.1;XP_006519589.1;XP_006519590.1;XP_011243482.1;XP_011243484.1;XP_011243485.1;XP_011243486.1;XP_011243487.1;XP_017171675.1;XP_017171676.1;XP_030103873.1;XP_030103874.1;XP_030103875.1;XP_030103876.1	1917811	.	7.716469
70564	NM_001316732,NM_001316733,NM_001316734,NM_001316735,NM_001316736,NM_001316737,NM_001316739,NM_001316740,NM_027464,XM_006519531	Prxl2a	peroxiredoxin like 2A	protein_coding	NP_001303661.1;NP_001303662.1;NP_001303663.1;NP_001303664.1;NP_001303665.1;NP_001303666.1;NP_001303668.1;NP_001303669.1;NP_081740.2;XP_006519594.1	1917814	.	2.465525
70567	NM_001081075,NM_027466,XM_006527334,XM_006527335,XM_017318291	Fra10ac1	FRA10AC1 homolog (human)	protein_coding	NP_001074544.1;NP_081742.1;XP_006527397.1;XP_006527398.1;XP_017173780.1	1917817	.	7.821146
70568	NM_001356481,NM_027769	Cpne3	copine III	protein_coding	NP_001343410.1;NP_082045.1	1917818	.	8.896053
70571	NM_183289	Tcerg1l	transcription elongation regulator 1-like	protein_coding	NP_899112.2	1917821	.	0.049940
70572	NM_001360602,NM_023579,XM_030248018,XM_030248019	Ipo5	importin 5	protein_coding	NP_001347531.1;NP_076068.1;XP_030103878.1;XP_030103879.1	1917822	.	31.617911
70573	NM_001081368,NM_001316746,XM_006522564,XM_006522565,XM_006522566,XM_006522567,XM_006522568,XM_006522569,XM_011246010,XR_003951830,XR_003951831,XR_003951832,XR_38	Tbccd1	TBCC domain containing 1	protein_coding	NP_001074837.1;NP_001303675.1;XP_006522627.1;XP_006522628.1;XP_006522629.1;XP_006522630.1;XP_006522631.1;XP_006522632.1;XP_011244312.1	1917823	.	4.566684

70574	NM_027468,XM_006514090	Cpm	carboxypeptidase M	protein_coding	NP_081744.1;XP_006514153.1	1917824	.	5.528084	[illegible]
70575	NM_001368385,NM_001368386,NM_001368388,NM_001368389,NM_027469,XM_006531348,XM_011248499,XM_017312958,XM_030243753	Gfod2	glucose-fructose oxidoreductase domain containing 2	protein_coding	NP_001355314.1;NP_001355315.1;NP_001355317.1;NP_001355318.1;NP_081745.1;XP_006531411.1;XP_011246801.1;XP_017168447.1;XP_030099613.1	1917825	.	1.185180	[illegible]
70579	NM_001276767,NM_001357228,NM_144530,XM_011248073,XM_011248075,XM_011248076,XM_030242922,XM_030242924	Zc3h11a	zinc finger CCCH type containing 11A	protein_coding	NP_001263696.1;NP_001344157.1;NP_653113.4;XP_011246375.1;XP_011246377.1;XP_011246378.1;XP_030098782.1;XP_030098784.1	1917829	.	64.896317	[illegible]
70584	NM_027470,XM_017312296,XM_017312297	Pak4	p21 (RAC1) activated kinase 4	protein_coding	NP_081746.1;XP_017167785.1;XP_017167786.1	1917834	.	5.251281	[illegible]
70591	NM_027472,XM_006534197,XM_006534198,XM_006534199	5730455P16Rik	RIKEN cDNA 5730455P16 gene	protein_coding	NP_081748.1;XP_006534260.1;XP_006534261.1;XP_006534262.1	1917841	.	8.619873	[illegible]
70592	NR_045500	5730480H06Rik	RIKEN cDNA 5730480H06 gene	lncRNA	.	1917842	.	1.985331	[illegible]
70598	NM_001081243,NM_001357352,NM_001357353,NM_001357354,XM_006511443,XM_030244584	Filip1	filamin A interacting protein 1	protein_coding	NP_001074712.1;NP_001344281.1;NP_001344282.1;NP_001344283.1;XP_006511506.1;XP_030100444.1	1917848	.	2.291638	[illegible]
70599	NM_080558,XM_006500165,XR_001783177	Itprid2	ITPR interacting domain containing 2	protein_coding	NP_542125.3;XP_006500228.1	1917849	.	9.925073	[illegible]
70601	NM_027475	Ecd	ecdysoneless cell cycle regulator	protein_coding	NP_081751.1	1917851	.	9.955247	[illegible]
70603	NM_001159581,NM_001316747,NM_133250,XM_006503392,XM_006503394,XM_030253774,XM_030253775,XR_003955015	Mutyh	mutY DNA glycosylase	protein_coding	NP_001153053.1;NP_001303676.1;NP_573513.2;XP_006503455.1;XP_006503457.1;XP_030109634.1;XP_030109635.1	1917853	.	2.029734	[illegible]
70604	NM_001033155,XM_006502055,XM_006502056	Dnajb14	DnaJ heat shock protein family (Hsp40) member B14	protein_coding	NP_001028327.1;XP_006502118.1;XP_006502119.1	1917854	.	10.267308	[illegible]
70605	NM_001168516,NM_001168517,NM_027476	Zdhhc24	zinc finger, DHHC domain containing 24	protein_coding	NP_001161988.1;NP_001161989.1;NP_081752.2	1917855	.	6.582847	[illegible]
70611	NM_001033156,XM_017315183	Fbxo33	F-box protein 33	protein_coding	NP_001028328.2;XP_017170672.1	1917861	.	4.160092	[illegible]
70612	NM_001141971,NM_027478,XM_006500166	Tmem230	transmembrane protein 230	protein_coding	NP_001135443.1;NP_081754.2;XP_006500229.1	1917862	.	14.584185	[illegible]
70615	NM_027480,XM_006514091,XM_006514092,XM_006514093,XM_006514094,XM_006514095,XM_006514096,XM_006514097,XM_006514098,XM_006514099,XM_006514101,XM_006514102,XM_006514103,XM_006514104,XM_011243564,XM_011243565,XM_011243566,XM_011243567,XM_011243568,XM_011243569,XM_011243570,XM_017314088,XM_030245270,XM_030245271,XM_030245272,XM_030245273,XM_030245274,XM_030245275	Ankrd24	ankyrin repeat domain 24	protein_coding	NP_081756.4;XP_006514154.1;XP_006514155.1;XP_006514156.1;XP_006514157.1;XP_006514158.1;XP_006514159.1;XP_006514160.1;XP_006514161.1;XP_006514162.1;XP_006514164.1;XP_006514165.1;XP_006514166.1;XP_006514167.1;XP_011241866.1;XP_011241867.1;XP_011241868.1;XP_011241869.1;XP_011241870.1;XP_011241871.1;XP_011241872.1;XP_017169577.1;XP_030101130.1;XP_030101131.1;XP_030101132.1;XP_030101133.1;XP_030101134.1;XP_03[illegible]	1890394	.	4.858380	[illegible]
70616	NM_001368391,NM_027481,XM_030243754,XM_030243755	Sugp1	SURP and G patch domain containing 1	protein_coding	NP_001355320.1;NP_081757.1;XP_030099614.1;XP_030099615.1	1917866	.	10.706621	[illegible]
70617	NM_027482	Fam241a	family with sequence similarity 241, member A	protein_coding	NP_081758.1	1917867	.	2.183022	[illegible]
70620	NM_001159351,NM_023585,XM_011246011	Ube2v2	ubiquitin-conjugating enzyme E2 variant 2	protein_coding	NP_001152823.1;NP_076074.2;XP_011244313.1	1917870	.	3.547994	[illegible]
70625	NM_027485,XM_006509739	Med26	mediator complex subunit 26	protein_coding	NP_081761.2;XP_006509802.1	1917875	.	3.767425	[illegible]
70626	NR_027973	5730522E02Rik	RIKEN cDNA 5730522E02 gene	lncRNA	.	1917876	.	0.156465	[illegible]
70638	NM_183087,XM_006541197	Fam189a1	family with sequence similarity 189, member A1	protein_coding	NP_898910.2;XP_006541260.1	1917888	.	0.300049	[illegible]
70640	NM_027490	Dcp2	decapping mRNA 2	protein_coding	NP_081766.1	1917890	.	6.217129	[illegible]
70645	NM_001042653	Oip5	Opa interacting protein 5	protein_coding	NP_001036118.1	1917895	.	2.233601	[illegible]
70646	NM_027492,XM_006519542	Naa30	N(alpha)-acetyltransferase 30, NatC catalytic subunit	protein_coding	NP_081768.2;XP_006519605.1	1922259	.	5.786014	[illegible]
70650	NM_027494,XM_006530457,XM_011248240	Zcchc8	zinc finger, CCHC domain containing 8	protein_coding	NP_081770.3;XP_006530520.1;XP_011246542.1	1917900	.	11.759291	[illegible]
70652	NM_027495,XM_006502057,XM_006502058,XM_006502059,XM_006502060,XM_011240224,XM_030252816,XR_001783743,XR_001783744	Tmem144	transmembrane protein 144	protein_coding	NP_081771.2;XP_006502120.1;XP_006502121.1;XP_006502122.1;XP_006502123.1;XP_011238526.1;XP_030108676.1	1917902	.	0.502093	[illegible]
70661	NM_027498,XM_006510590	Sik3	SIK family kinase 3	protein_coding	NP_081774.3;XP_006510653.1	2446296	.	13.277061	[illegible]
70673	NM_001177995,NM_001291026,NM_001291029,NM_027504,XM_006539171,XM_006539172,XM_006539173,XM_006539174,XM_006539175,XM_006539178,XM_006539179	Prdm16	PR domain containing 16	protein_coding	NP_001171466.1;NP_001277955.1;NP_001277958.1;NP_081780.3;XP_006539234.1;XP_006539235.1;XP_006539236.1;XP_006539237.1;XP_006539238.1;XP_006539241.1;XP_006539242.1	1917923	.	1.280352	[illegible]
70675	NM_173443	Vcpip1	valosin containing protein (p97)/p47 complex interacting protein 1	protein_coding	NP_775619.2	1917925	.	4.266737	[illegible]
70676	NM_028450,XM_006496276,XM_006496277,XM_006496278,XM_011238599	Gulp1	GULP, engulfment adaptor PTB domain containing 1	protein_coding	NP_082726.2;XP_006496339.1;XP_006496340.1;XP_006496341.1;XP_011236901.1	1920407	.	13.267621	[illegible]
70681	NM_172405,XM_006535217,XM_006535218,XM_030254801,XR_001784743,XR_001784744,XR_003955708,XR_389290	Abraxas1	BRCA1 A complex subunit	protein_coding	NP_765993.1;XP_006535280.1;XP_006535281.1;XP_030110661.1	1917931	.	3.848053	[illegible]
70683	NM_175158	Utp20	UTP20 small subunit processome component	protein_coding	NP_780367.2	1917933	.	3.819100	[illegible]
70686	NM_001048054,NM_130447,XM_030255591,XM_030255592	Dusp16	dual specificity phosphatase 16	protein_coding	NP_001041519.1;NP_569714.2;XP_030111451.1;XP_030111452.1	1917936	.	4.928795	[illegible]
70691	NM_027510,XM_011247676,XM_011247677	3830403N18Rik	RIKEN cDNA 3830403N18 gene	protein_coding	NP_081786.1;XP_011245978.1;XP_011245979.1	1917941	.	2.122418	[illegible]
70693	NM_133911	Adgra3	adhesion G protein-coupled receptor A3	protein_coding	NP_598672.1	1917943	.	20.490891	[illegible]
70696	NM_027512,XM_030251497,XM_030251498	3830417A13Rik	RIKEN cDNA 3830417A13 gene	protein_coding	NP_081788.2;XP_030107357.1;XP_030107358.1	1917946	.	0.000000	[illegible]
70699	NM_027513,XM_006506590,XM_006506593,XM_017321734,XM_017321735,XR_001785168	Nup205	nucleoporin 205	protein_coding	NP_081789.1;XP_006506653.1;XP_006506656.1;XP_017177223.1;XP_017177224.1	2141625	.	9.865915	[illegible]
70701	NM_001081205	Nipal1	NIPA-like domain containing 1	protein_coding	NP_001074674.1	1917951	.	0.093315	[illegible]
70713	NM_027518,XM_006519544,XM_017316190,XM_017316191,XM_017316192,XR_874367	Gpr137c	G protein-coupled receptor 137C	protein_coding	NP_081794.2;XP_006519607.1;XP_017171679.1;XP_017171680.1;XP_017171681.1	1917963	.	0.506617	[illegible]
70717	NM_001356995,NM_027519,NR_151595,XM_006504940,XM_006504941,XM_006504942,XM_030254803	Medag	mesenteric estrogen dependent adipogenesis	protein_coding	NP_001343924.1;NP_081795.1;XP_006505003.1;XP_006505004.1;XP_006505005.1;XP_030110663.1	1917967	.	2.666130	[illegible]
70719	NM_001142701,NM_001347074,NM_027521,XM_006514109,XM_006514110,XM_030245276,XR_001779599	Arhgap45	Rho GTPase activating protein 45	protein_coding	NP_001136173.1;NP_001334003.1;NP_081797.1;XP_006514172.1;XP_006514173.1;XP_030101136.1	1917969	.	4.778896	[illegible]
70720	NR_028126	6330407A03Rik	RIKEN cDNA 6330407A03 gene	lncRNA	.	1917970	.	0.000000	[illegible]
70726	NM_145154,XM_011242599,XM_011242600	Angptl6	angiopoietin-like 6	protein_coding	NP_660136.1;XP_011240901.1;XP_011240902.1	1917976	.	0.338898	[illegible]
70727	NM_001316749,NM_001362103,NM_001362104,NM_001362105,NM_027526	Rasgef1a	RasGEF domain family, member 1A	protein_coding	NP_001303678.1;NP_001349032.1;NP_001349033.1;NP_001349034.1;NP_081802.1	1917977	.	0.244608	[illegible]
70729	NM_001109985,NR_154593	Nos1ap	nitric oxide synthase 1 (neuronal) adaptor protein	protein_coding	NP_001103455.1	1917979	.	0.210377	[illegible]
70730	NM_027529	6330409D20Rik	RIKEN cDNA 6330409D20 gene	protein_coding	NP_081805.1	1917980	.	0.000000	[illegible]
70737	NM_001037711,NM_001293727,XM_006502061,XM_006502062,XM_006502064,XM_030252817,XR_375578	Cgn	cingulin	protein_coding	NP_001032800.3;NP_001280656.2;XP_006502124.1;XP_006502125.1;XP_006502127.1;XP_030108677.1	1927237	.	0.167340	[illegible]
70747	NM_001243132,NM_027533	Tspan2	tetraspanin 2	protein_coding	NP_001230061.1;NP_081809.2	1917997	.	29.284915	[illegible]
70750	NM_001316750,NM_027534	Kdsr	3-ketodihydrosphingosine reductase	protein_coding	NP_001303679.1;NP_081810.1	1918000	.	5.059852	[illegible]
70753	NR_045141	6330415B21Rik	RIKEN cDNA 6330415B21 gene	lncRNA	.	1918003	.	0.000856	[illegible]
70757	NM_023587,XM_006522570,XM_006522571	Hacd2	3-hydroxyacyl-CoA dehydratase 2	protein_coding	NP_076076.2;XP_006522633.1;XP_006522634.1	1918007	.	3.543262	[illegible]
70762	NM_001195496,NM_001195497,NM_001195498,NM_001195499,NM_001195500,NM_001357460,NM_027539,XM_006502066,XM_006502067,XM_006502068,XM_006502069,XM_006502070,XM_006502071,XM_006502072,XM_006502073,XM_006502074,XM_006502075,XM_006502076,XM_006502077,XM_006502078,XM_011240226,XM_011240227,XM_017319719,XM_030252818,XM_030252819,XM_030252820,XM_030252821,XM_030252822,XM_030252823,XR_001783745,XR_003954381,XR_003954382,XR_003954383,XR_003954384,XR_003954385,XR_003954386,XR_003954387,XR_867229,XR_867230,XR_867231	Dclk2	doublecortin-like kinase 2	protein_coding	NP_001182425.1;NP_001182426.1;NP_001182427.1;NP_001182428.1;NP_001182429.1;NP_001344389.1;NP_081815.3;XP_006502129.1;XP_006502130.1;XP_006502131.1;XP_006502132.1;XP_006502133.1;XP_006502134.1;XP_006502135.1;XP_006502136.1;XP_006502137.1;XP_006502138.1;XP_006502139.1;XP_006502140.1;XP_006502141.1;XP_011238528.1;XP_011238529.1;XP_017175208.1;XP_030108678.1;XP_030108679.1;XP_030108680.1;XP_030108681.1;XP_030108682.1;XP_030108683.1	1918012	.	3.860713	[illegible]
70767	NM_001316751,NM_027541,NR_151629,XM_006502081	Prpf3	pre-mRNA processing factor 3	protein_coding	NP_001303680.1;NP_081817.2;XP_006502144.1	1918017	.	10.822243	[illegible]
70769	NM_001039351,NM_001039352,NM_001039353,NM_053086	Nolc1	nucleolar and coiled-body phosphoprotein 1	protein_coding	NP_001034440.1;NP_001034441.1;NP_001034442.1;NP_444316.2	1918019	.	16.983834	[illegible]
70771	NM_001313748,NM_001359449,NM_001359450,NM_027543,NR_132633,XM_011247871	Gpr173	G-protein coupled receptor 173	protein_coding	NP_001300677.1;NP_001346378.1;NP_001346379.1;NP_081819.2;XP_011246173.1	1918021	.	3.115728	[illegible]
70772	NM_001251881,NM_027544	Ggnbp1	gametogenetin binding protein 1	protein_coding	NP_001238810.1;NP_081820.2	3055306	.	3.134327	[illegible]
70779	NM_027547,NM_029549,XM_006506602,XM_006506604,XM_011241464,XR_377491,XR_377492,XR_377493	Prdm5	PR domain containing 5	protein_coding	NP_081823.2;NP_083825.1;XP_006506665.1;XP_006506667.1;XP_011239766.1	1918029	.	10.622429	[illegible]
70784	NM_001033158,NM_001162923,XM_017313587,XR_379455	Rasl12	RAS-like, family 12	protein_coding	NP_001028330.2;NP_001156395.1;XP_017169076.1	1918034	.	2.283137	[illegible]
70785	NM_153551,XM_006524898,XM_006524899,XM_006524900,XM_006524901,XM_006524902,XM_011246601,XM_017317635,XM_030250048,XM_030250049,XM_030250050,XM_030250051,XR_003952178,XR_003952179,XR_003952180,XR_003952181	Dennd1c	DENN/MADD domain containing 1C	protein_coding	NP_705779.1;XP_006524961.1;XP_006524962.1;XP_006524963.1;XP_006524964.1;XP_006524965.1;XP_011244903.1;XP_017173124.1;XP_030105908.1;XP_030105909.1;XP_030105910.1;XP_030105911.1	1918035	.	0.653787	[illegible]
70788	NM_027551	Klhl30	kelch-like 30	protein_coding	NP_081827.1	1918038	.	1.344662	[illegible]
70789	NM_001289593,NM_001289594,NM_027552,XM_006498325,XM_011239178,XM_011239179	Kynu	kynureninase	protein_coding	NP_001276522.1;NP_001276523.1;NP_081828.1;XP_006498388.1;XP_011237480.1;XP_011237481.1	1918039	.	0.000000	[illegible]
70790	NM_001081359,NM_001112721,XM_006520179,XM_006520180,XM_006520181,XM_006520182,XM_030248763,XR_001781565,XR_383833,XR_383834	Ubr5	ubiquitin protein ligase E3 component n-recognin 5	protein_coding	NP_001074828.2;NP_001106192.1;XP_006520242.1;XP_006520243.1;XP_006520244.1;XP_006520245.1;XP_030104623.1	1918040	.	23.141494	[illegible]
70791	NM_001305627,NM_001360921,NM_080636,XM_011246999,XR_003952541,XR_386015	Hars2	histidyl-tRNA synthetase 2	protein_coding	NP_001292556.1;NP_001347850.1;NP_542367.1;XP_011245301.1	1918041	.	13.593478	[illegible]
70796	NM_001357252,NM_175160,XM_006531357,XM_006531358,XM_011248502,XM_017312960,XM_017312961,XM_030243756,XM_030243757,XM_030243758,XM_030243759,XM_030243760,XM_030243761	Zdhhc1	zinc finger, DHHC domain containing 1	protein_coding	NP_001344181.1;NP_780369.1;XP_006531420.1;XP_006531421.1;XP_011246804.1;XP_017168449.1;XP_017168450.1;XP_030099616.1;XP_030099617.1;XP_030099618.1;XP_030099619.1;XP_030099620.1;XP_030099621.1	1918046	.	20.259479	[illegible]
70797	NM_001003909,NM_001289527,NM_001289528,NM_001289529,XM_017321097	Ankib1	ankyrin repeat and IBR domain containing 1	protein_coding	NP_001003909.2;NP_001276456.1;NP_001276457.1;NP_001276458.1;XP_017176586.1	1918047	.	7.219385	[illegible]
70799	NM_027556,XM_006526241,XM_006526242,XM_006526243,XM_011247000,XM_011247001	Cep192	centrosomal protein 192	protein_coding	NP_081832.1;XP_006526304.1;XP_006526305.1;XP_006526306.1;XP_011245302.1;XP_011245303.1	1918049	.	5.209884	[illegible]
70802	NM_001164231,NM_001363141,NM_001363142,NM_027557,XM_006534200,XM_006534202,XM_006534203,XM_006534204,XM_006534205,XM_006534208,XM_011249257,XM_011249259,XM_030246310,XR_001780050,XR_388529	Pwwp2a	PWWP domain containing 2A	protein_coding	NP_001157703.1;NP_001350070.1;NP_001350071.1;NP_081833.1;XP_006534263.1;XP_006534265.1;XP_006534266.1;XP_006534267.1;XP_006534268.1;XP_006534271.1;XP_011247559.1;XP_011247561.1;XP_030102170.1	1918052	.	8.094720	[illegible]
70804	NM_027558	Pgrmc2	progesterone receptor membrane component 2	protein_coding	NP_081834.1	1918054	.	16.863859	[illegible]
70807	NM_027560,XM_017312963,XR_379046	Arrdc2	arrestin domain containing 2	protein_coding	NP_081836.1;XP_017168452.1	1918057	.	4.617763	[illegible]
70808	NR_027985	4632415L05Rik	RIKEN cDNA 4632415L05 gene	transcribed_pseudogene	.	1918058	.	4.516750	[illegible]
70809	NM_001168223,NM_001168224,NM_001316752,NM_027562	Clec2g	C-type lectin domain family 2, member g	protein_coding	NP_001161695.1;NP_001161696.1;NP_001303681.1;NP_081838.1	1918059	.	1.139252	[illegible]
70821	NM_027564,XM_006506605,XM_006506606,XM_030255593	4921507P07Rik	RIKEN cDNA 4921507P07 gene	protein_coding	NP_081840.1;XP_006506668.1;XP_006506669.1;XP_030111453.1	1918071	.	0.063920	[illegible]
70823	NM_001316753,NM_001316754,NM_178017,XM_006531362,XM_006531364	Hmgxb4	HMG box domain containing 4	protein_coding	NP_001303682.1;NP_001303683.1;NP_821136.1;XP_006531425.2;XP_006531427.1	1918073	.	8.450818	[illegible]
70827	NM_172406,XM_006496280,XM_006496281,XM_006496282,XM_011238600,XM_030242947	Trak2	trafficking protein, kinesin binding 2	protein_coding	NP_765994.2;XP_006496343.1;XP_006496344.1;XP_006496345.1;XP_011236902.1;XP_030098807.1	1918077	.	11.247793	[illegible]
70829	NM_001025156,NM_029955	Ccdc93	coiled-coil domain containing 93	protein_coding	NP_001020327.1;NP_084231.2	1918079	.	6.547295	[illegible]
70832	NR_102341,NR_102342	4921504A21Rik	RIKEN cDNA 4921504A21 gene	lncRNA	.	1918082	.	0.445396	[illegible]
70834	NM_001025428,NM_001025429,NM_001025430,NM_001199203,NM_001199204,NM_001199205,NM_001363157,NM_027569,XM_006534210,XM_006534211,XM_006534212,XM_006534213,XM_006534214,XM_006534215,XM_006534216,XM_006534217,XM_006534218,XM_030246313,XM_030246314,XM_030246315,XM_030246317,XM_030246318	Spag9	sperm associated antigen 9	protein_coding	NP_001020599.1;NP_001020600.1;NP_001020601.1;NP_001186132.1;NP_001186133.1;NP_001186134.1;NP_001350086.1;NP_081845.2;XP_006534273.1;XP_006534274.1;XP_006534275.1;XP_006534276.1;XP_006534277.1;XP_006534278.1;XP_006534279.1;XP_006534280.1;XP_006534281.1;XP_030102173.1;XP_030102174.1;XP_030102175.1;XP_030102177.1;XP_030102178.1	1918084	.	40.971932	[illegible]
70835	NM_133731,XM_006524905,XM_006524906	Prss22	protease, serine 22	protein_coding	NP_598492.2;XP_006524968.4;XP_006524969.1	1918085	.	0.069642	[illegible]
70839	NM_001357007,NM_001357008,NM_001357010,NM_027571	P2ry12	purinergic receptor P2Y, G-protein coupled 12	protein_coding	NP_001343936.1;NP_001343937.1;NP_001343939.1;NP_081847.3	1918089	.	1.474859	[illegible]
70843	NM_027574	Krt28	keratin 28	protein_coding	NP_081850.1	1918093	.	0.000000	[illegible]
70846	NM_001312644,XM_017315184,XM_017315185,XM_017315186,XM_017315187,XM_017315188,XM_017315189,XM_017315191,XM_017315192,XM_017315193,XM_017315194,XM_030246911,XM_030246912,XR_001780477,XR_001780478,XR_001780479,XR_001780480,XR_001780481,XR_001780482	Ttc6	tetratricopeptide repeat domain 6	protein_coding	NP_001299573.1;XP_017170673.1;XP_017170674.1;XP_017170675.1;XP_017170676.1;XP_017170677.1;XP_017170678.1;XP_017170680.1;XP_017170681.1;XP_017170682.1;XP_017170683.1;XP_030102771.1;XP_030102772.1	2684915	.	0.000000	[illegible]
70853	NM_001370828,NM_001370829,XM_006496379,XM_006496380,XM_006496381,XM_006496382,XR_001778869,XR_001778870,XR_001778872,XR_001778879,XR_001778880	Vwa3b	von Willebrand factor A domain containing 3B	protein_coding	NP_001357757.1;NP_001357758.1;XP_006496442.1;XP_006496443.1;XP_006496444.1;XP_006496445.1	1918103	.	0.069212	[illegible]
70858	NR_045829	4921511I17Rik	RIKEN cDNA 4921511I17 gene	lncRNA	.	1918108	.	0.000429	[illegible]
70859	NM_027581,XM_006519545,XM_006519546,XM_006519548,XM_017316193	Lrrc63	leucine rich repeat containing 63	protein_coding	NP_081857.1;XP_006519608.1;XP_006519609.1;XP_006519611.1;XP_017171682.1	1918109	.	0.000000	[illegible]
70861	NM_001310617,NM_027582	Akr1cl	aldo-keto reductase family 1, member C-like	protein_coding	NP_001297546.1;NP_081858.2	1918111	.	0.000000	[illegible]
70873	NM_001316755,NM_027585,XM_006500167,XM_006500172,XM_030252071,XR_003953718	Cnbd2	cyclic nucleotide binding domain containing 2	protein_coding	NP_001303684.1;NP_081861.2;XP_006500230.1;XP_006500235.1;XP_030107931.1	1918123	.	1.853616	[illegible]
70876	XR_001781913,XR_003951894,XR_876117	4921517D16Rik	RIKEN cDNA 4921517D16 gene, transcript variant X2	lncRNA	.	1918126	.	0.000000	[illegible]
70881	NM_001310582,NM_027588,XM_006515207,XM_006515208,XM_006515209,XM_006515210,XM_006515211,XM_030246913	Nt5c1b	5'-nucleotidase, cytosolic IB	protein_coding	NP_001297511.1;NP_081864.2;XP_006515270.1;XP_006515271.1;XP_006515272.1;XP_006515273.1;XP_006515274.1;XP_030102773.1	1918131	.	0.021858	[illegible]
70882	NM_001081083,NM_001271563,NM_001271564,NM_001271565,XM_006497537,XR_001783178	Armc3	armadillo repeat containing 3	protein_coding	NP_001074552.1;NP_001258492.1;NP_001258493.1;NP_001258494.1;XP_006497600.1	1918132	.	0.008204	[illegible]
70884	NM_001162979,XM_017312298	Ccdc81	coiled-coil domain containing 81	protein_coding	NP_001156451.1;XP_017167787.1	1918134	.	0.000000	[illegible]
70885	NM_001293791,NM_001293792,NM_001359129,NM_027590,XM_006509741,XM_006509743,XM_006509744,XM_006509747,XM_011242326,XM_030243762,XM_030243763,XM_030243764,XM_030243765	Ints10	integrator complex subunit 10	protein_coding	NP_001280720.1;NP_001280721.1;NP_001346058.1;NP_081866.1;XP_006509804.1;XP_006509806.1;XP_006509807.1;XP_006509810.1;XP_011240628.1;XP_030099622.1;XP_030099623.1;XP_030099624.1;XP_030099625.1	1918135	.	4.724016	[illegible]
70887	NM_001038616,NM_027591,NM_029378,XM_006528280,XM_017318619	Dmrtc1a	DMRT-like family C1a	protein_coding	NP_001033705.2;NP_081867.1;NP_083654.1;XP_006528343.1;XP_017174108.1	1918137	.	0.000000	[illegible]
70891	NM_001142631,NM_029254,XM_006524907,XM_006524908,XM_006524909,XM_011246602	Spdya	speedy/RINGO cell cycle regulator family, member A	protein_coding	NP_001136103.1;NP_083530.1;XP_006524970.1;XP_006524971.1;XP_006524972.1;XP_011244904.1	1918141	.	0.235120	[illegible]
70892	NM_001302957,NM_001302958,NM_001357217,XM_017319721,XM_017319722	Ttll7	tubulin tyrosine ligase-like family, member 7	protein_coding	NP_001289886.1;NP_001289887.1;NP_001344146.1;XP_017175210.1;XP_017175211.1	1918142	.	1.226983	[illegible]
70893	NM_001113323,XM_006510591,XM_006510593,XM_006510594,XM_006510595,XM_017313588	Glb1l3	galactosidase, beta 1 like 3	protein_coding	NP_001106794.1;XP_006510654.1;XP_006510656.1;XP_006510657.1;XP_006510658.1;XP_017169077.1	1918143	.	0.027374	[illegible]
70894	NM_001081046,XM_006534223,XM_006534226,XM_017314762,XM_017314763,XM_017314764,XM_017314765	Efcab3	EF-hand calcium binding domain 3	protein_coding	NP_001074515.1;XP_006534286.1;XP_006534289.1;XP_017170251.1;XP_017170252.1;XP_017170253.1;XP_017170254.1	1918144	.	0.000000	[illegible]
70897	NM_027597,NM_029069,XM_006516227,XM_011244172,XM_011244173,XM_011244175,XM_017315195,XM_017315196,XM_017315197,XM_017315198,XM_017315199,XM_017315200,XM_030246914,XM_030246915,XM_030246916,XM_030246917,XM_030246918,XM_030246919,XM_030246920,XR_001780483,XR_001780484,XR_001780485,XR_001780487,XR_001780488,XR_003950079,XR_003950080,XR_003950081,XR_003950082,XR_003950083,XR_003950084,XR_003950085	Fam71d	family with sequence similarity 71, member D	protein_coding	NP_081873.2;NP_083345.1;XP_006516290.1;XP_011242474.1;XP_011242475.1;XP_011242477.1;XP_017170684.1;XP_017170685.1;XP_017170686.1;XP_017170687.1;XP_017170688.1;XP_017170689.1;XP_030102774.1;XP_030102775.1;XP_030102776.1;XP_030102777.1;XP_030102778.1;XP_030102779.1;XP_030102780.1	1918147	.	0.000000	[illegible]
70911	NM_001162846,NM_178621,XM_006514112	Phyhipl	phytanoyl-CoA hydroxylase interacting protein-like	protein_coding	NP_001156318.1;NP_848736.2;XP_006514175.1	1918161	.	0.122171	[illegible]
70918	NM_001302833,NM_001302834,NM_027602,XM_006504101,XM_006504102,XM_006504105,XM_006504106,XM_011240769,XM_017321098,XM_030254806	Nsun7	NOL1/NOP2/Sun domain family, member 7	protein_coding	NP_001289762.1;NP_001289763.1;NP_081878.1;XP_006504164.1;XP_006504165.1;XP_006504168.1;XP_006504169.1;XP_011239071.1;XP_017176587.1;XP_030110666.1	1918168	.	0.946267	[illegible]
70924	NR_045502	4921511C10Rik	RIKEN cDNA 4921511C10 gene	lncRNA	.	1918174	.	0.000000	[illegible]
70925	NM_001359133,NM_172407	Cdkn2aip	CDKN2A interacting protein	protein_coding	NP_001346062.1;NP_765995.1	1918175	.	5.127265	[illegible]
70928	NM_080510,XM_006500173,XM_030252073,XM_030252074	Trim69	tripartite motif-containing 69	protein_coding	NP_536771.2;XP_006500236.1;XP_030107933.1;XP_030107934.1	1918178	.	0.146982	[illegible]
70930	NM_001271397,NR_073167,NR_073168,XM_006516956,XM_006516957,XM_006516958	Nol8	nucleolar protein 8	protein_coding	NP_001258326.1;XP_006517019.2;XP_006517020.1;XP_006517021.1	1918180	.	4.097393	[illegible]
70933	NR_040608	4931402G19Rik	RIKEN cDNA 4931402G19 gene	lncRNA	.	1918183	.	0.000000	[illegible]
70945	NM_001163507,NM_027613	Mmrn1	multimerin 1	protein_coding	NP_001156979.1;NP_081889.1	1918195	.	0.789789	[illegible]
70948	NM_027614	Wdr20rt	WD repeat domain 20, retrogene	protein_coding	NP_081890.1	1918198	.	0.000000	[illegible]
70951	NM_001316756,NM_001370604,NM_027617,XM_006502090	Spata1	spermatogenesis associated 1	protein_coding	NP_001303685.1;NP_001357533.1;NP_081893.1;XP_006502153.1	1918201	.	2.774009	[illegible]
70955	XR_383259,XR_874410,XR_874411,XR_874412	4931403M11Rik	RIKEN cDNA 4931403M11 gene, transcript variant X3	lncRNA	.	1918205	.	0.000000	[illegible]
70957	NR_038162	Stamos	signal transducing adaptor molecule (SH3 domain and ITAM motif) 1, opposite strand	lncRNA	.	1918207	.	0.234836	[illegible]
70966	XR_003947513	4931415C17Rik	RIKEN cDNA 4931415C17 gene	lncRNA	.	1918216	.	0.077711	[illegible]
70967	NM_001199210,NM_001316760,NM_001316761,NM_027627,NR_133572,NR_133573,XM_006523085,XM_017317131,XM_017317132,XM_030249282	Eva1c	eva-1 homolog C (C. elegans)	protein_coding	NP_001186139.1;NP_001303689.1;NP_001303690.1;NP_081903.1;XP_006523148.1;XP_017172620.1;XP_017172621.1;XP_030105142.1	1918217	.	0.746338	[illegible]
70973	NR_045183	4931431B13Rik	RIKEN cDNA 4931431B13 gene	lncRNA	.	1918223	.	0.000000	[illegible]
70974	NM_027629,XM_006508204,XM_006508205,XR_378271	Pgm2l1	phosphoglucomutase 2-like 1	protein_coding	NP_081905.1;XP_006508267.1;XP_006508268.1	1918224	.	2.354585	[illegible]
70976	NM_027630,XM_030245277	Ccdc105	coiled-coil domain containing 105	protein_coding	NP_081906.1;XP_030101137.1	1918226	.	0.027577	[illegible]
70977	NM_027631	Cabs1	calcium binding protein, spermatid specific 1	protein_coding	NP_081907.1	1918227	.	0.000000	[illegible]
70981	NM_027635,XM_006498328,XM_011239180,XM_017319257,XR_003953719	Fem1al	RIKEN cDNA 4931423N10 gene	protein_coding	NP_081911.1;XP_006498391.1;XP_011237482.1;XP_017174746.1	1918231	.	0.030695	[illegible]
70984	NM_001199484,NM_001199485,NM_001359258,NM_133732,XM_006510596,XM_030244585	4931406C07Rik	RIKEN cDNA 4931406C07 gene	protein_coding	NP_001186413.1;NP_001186414.1;NP_001346187.1;NP_598493.1;XP_006510659.1;XP_030100445.1	1918234	.	9.236248	[illegible]
70987	XR_880009	4931413K12Rik	RIKEN cDNA 4931413K12 gene	lncRNA	.	1918237	.	0.000000	[illegible]
70989	NM_001081121,NM_001357359,NM_001357360,XM_006510600,XM_006510601	Jhy	junctional cadherin complex regulator	protein_coding	NP_001074590.1;NP_001344288.1;NP_001344289.1;XP_006510663.1;XP_006510664.1	1918239	.	0.000000	[illegible]
70997	NM_027641	Spef1	sperm flagellar 1	protein_coding	NP_081917.1	3513546	.	4.815333	[illegible]
70998	NM_001290546,NM_027642	Phf6	PHD finger protein 6	protein_coding	NP_001277475.1;NP_081918.1	1918248	.	5.273223	[illegible]
70999	NM_027643,XM_006527336	Naa40	N(alpha)-acetyltransferase 40, NatD catalytic subunit	protein_coding	NP_081919.1;XP_006527399.1	1918249	.	10.848091	[illegible]
71000	NR_045503	4931440J10Rik	RIKEN cDNA 4931440J10 gene	lncRNA	.	1918250	.	0.000000	[illegible]
71001	NM_001145300,NM_001291015,XM_011248086	Mgat4e	MGAT4 family, member E	protein_coding	NP_001138772.1;NP_001277944.1;XP_011246388.2	1918251	.	0.097079	[illegible]
71003	NM_027644,XM_006524911,XM_006524913,XM_011246604	Prss41	protease, serine 41	protein_coding	NP_081920.1;XP_006524974.1;XP_006524976.1;XP_011244906.1	1918253	.	0.000000	[illegible]
71004	NR_027955	4931440P22Rik	RIKEN cDNA 4931440P22 gene	lncRNA	.	1918254	.	0.124396	[illegible]
71007	NM_001081331,NM_001199941,XM_011241901,XM_011241902,XM_011241903,XM_011241904,XM_011241905,XM_011241906,XM_011241907,XM_030242987	D7Ertd443e	DNA segment, Chr 7, ERATO Doi 443, expressed	protein_coding	NP_001074800.1;NP_001186870.1;XP_011240203.1;XP_011240204.1;XP_011240205.1;XP_011240206.1;XP_011240207.1;XP_011240208.1;XP_011240209.1;XP_030098847.1	1196431	.	0.151240	[illegible]
71008	NR_045431	4933401D09Rik	RIKEN cDNA 4933401D09 gene	lncRNA	.	1918258	.	0.000000	[illegible]
71020	NM_001357831,NM_001357832,NM_027649,XM_006524918,XM_011246605	Spats1	spermatogenesis associated, serine-rich 1	protein_coding	NP_001344760.1;NP_001344761.1;NP_081925.2;XP_006524981.1;XP_011244907.1	1918270	.	0.000000	[illegible]
71027	NM_027651,XM_011246012,XM_011246014,XM_017317133,XM_017317134,XM_030249283,XM_030249284	Tmem30c	transmembrane protein 30C	protein_coding	NP_081927.1;XP_011244314.1;XP_011244316.1;XP_017172622.1;XP_017172623.1;XP_030105143.1;XP_030105144.1	1918277	.	0.000000	[illegible]
71030	NM_001177389,XM_011247708	4933403O08Rik	RIKEN cDNA 4933403O08 gene	protein_coding	NP_001170860.1;XP_011246010.1	1918280	.	0.015198	[illegible]
71040	XR_387941,XR_879116,XR_879117,XR_879118	4933406B17Rik	RIKEN cDNA 4933406B17 gene, transcript variant X3	lncRNA	.	1918290	.	0.040190	[illegible]
71041	NM_001360632,NM_027654,NR_133574,NR_153762,XM_006527338,XM_011247340,XM_030251075,XR_001782614,XR_003952814,XR_003952815,XR_003952816,XR_386508	Pcgf6	polycomb group ring finger 6	protein_coding	NP_001347561.1;NP_081930.1;XP_006527401.1;XP_011245642.1;XP_030106935.1	1918291	.	4.782886	[illegible]
71045	NR_029437,NR_029438	4933406I18Rik	RIKEN cDNA 4933406I18 gene	lncRNA	.	1918295	.	0.000000	[illegible]
71046	NM_001357256,NM_027655	4933405L10Rik	RIKEN cDNA 4933405L10 gene	protein_coding	NP_001344185.1;NP_081931.1	1918296	.	0.000000	[illegible]
71059	NM_001130515,NM_001130516,NM_027658,XM_006534228,XM_006534229,XM_006534230,XM_006534231,XM_030246320	Hexim2	hexamethylene bis-acetamide inducible 2	protein_coding	NP_001123987.1;NP_001123988.1;NP_081934.1;XP_006534291.1;XP_006534292.1;XP_006534293.1;XP_006534294.1;XP_030102180.1	1918309	.	2.485948	[illegible]
71063	NM_001033159,XM_006522576	Zfp597	zinc finger protein 597	protein_coding	NP_001028331.2;XP_006522639.1	1918313	.	2.895294	[illegible]
71067	NR_045342	4933411E08Rik	RIKEN cDNA 4933411E08 gene	lncRNA	.	1918317	.	0.000000	[illegible]
71069	NM_001114311,NM_001286376,NM_001363119,NM_175162,XM_006509491,XM_006509492,XM_006509494,XM_006509495,XM_011242237,XM_017312966,XM_030243766,XM_030243767,XM_030243768	Stox2	storkhead box 2	protein_coding	NP_001107783.1;NP_001273305.1;NP_001350048.1;NP_780371.3;XP_006509554.1;XP_006509555.1;XP_006509557.1;XP_006509558.1;XP_011240539.1;XP_017168455.1;XP_030099626.1;XP_030099627.1;XP_030099628.1	1918319	.	6.482852	[illegible]
71071	NR_045507	4933412O06Rik	RIKEN cDNA 4933412O06 gene	lncRNA	.	1918321	.	0.000000	[illegible]
71078	NM_027665	Adam30	a disintegrin and metallopeptidase domain 30	protein_coding	NP_081941.1	1918328	.	0.000663	[illegible]
71081	NR_040498	4933416E03Rik	RIKEN cDNA 4933416E03 gene	lncRNA	.	1918331	.	0.305718	[illegible]
71085	NM_001163495,NM_027667,XM_011247341,XM_011247342,XM_011247343,XM_011247344	Arhgap19	Rho GTPase activating protein 19	protein_coding	NP_001156967.1;NP_081943.2;XP_011245643.1;XP_011245644.1;XP_011245645.1;XP_011245646.3	1918335	.	2.601127	[illegible]
71086	NR_038025,NR_038026	4933412E12Rik	RIKEN cDNA 4933412E12 gene	lncRNA	.	1918336	.	1.337288	[illegible]
71089	NM_027669,NM_029374,XM_006522577,XM_006522578,XM_006522579,XM_006522580	Sept12	septin 12	protein_coding	NP_081945.1;NP_083650.1;XP_006522640.1;XP_006522641.1;XP_006522642.1;XP_006522643.1	1918339	.	0.000000	[illegible]
71091	NM_183294,XM_011244176,XM_011244177,XM_017315203	Cdkl1	cyclin-dependent kinase-like 1 (CDC2-related kinase)	protein_coding	NP_899117.1;XP_011242478.1;XP_011242479.1;XP_017170692.1	1918341	.	0.459634	[illegible]
71093	NM_153778,XR_377494	Atoh8	atonal bHLH transcription factor 8	protein_coding	NP_722473.1	1918343	.	6.470158	[illegible]
71096	NM_001290390,NM_001290392,NM_001290393,NM_027671,XM_006495572,XM_006495573,XM_011238407,XM_017312320,XM_017312321,XM_017312325,XM_030242990,XR_001778003	Sntg1	syntrophin, gamma 1	protein_coding	NP_001277319.1;NP_001277321.1;NP_001277322.1;NP_081947.2;XP_006495635.1;XP_006495636.1;XP_011236709.1;XP_017167809.1;XP_017167810.1;XP_017167814.1;XP_030098850.1	1918346	.	0.000000	[illegible]

71099	NM_001253888,NM_001253889,NM_027673,XM_006519551,XM_006519552,XM_030248029,XM_030248030	Tssk4	testis-specific serine kinase 4	protein_coding	NP_001240817.1;NP_001240818.1;NP_081949.1;XP_006519614.1;XP_006519615.1;XP_030103889.1;XP_030103890.1	1918349	.	1.597144	[illegible]
71101	NM_001081101,NM_027674,NR_153107,NR_153108,XM_006504108,XM_006504109,XR_003955709,XR_003955710,XR_376825	Uvssa	UV stimulated scaffold protein A	protein_coding	NP_001074570.1;NP_081950.1;XP_006504171.1;XP_006504172.1	1918351	.	8.502407	[illegible]
71106	NR_038005	4933413J09Rik	RIKEN cDNA 4933413J09 gene	lncRNA		1918356	.	0.000000	[illegible]
71111	NM_027677	Gpr39	G protein-coupled receptor 39	protein_coding	NP_081953.2	1918361	.	0.176032	[illegible]
71116	NM_001289535,NM_001289536,NM_001359398,NM_026959,XM_006504112,XM_030254807	Stx18	syntaxin 18	protein_coding	NP_001276464.1;NP_001276465.1;NP_001346327.1;NP_081235.2;XP_006504175.1;XP_030110667.1	1918366	.	9.874745	[illegible]
71130	XM_006506833,XM_006506834,XM_006506835,XM_011241544,XM_011241545,XM_011241546,XM_011241547,XM_011241549,XM_030255700,XM_355785,XR_003956313,XR_003956314	Sh2d6	SH2 domain containing 6, transcript variant X11	protein_coding	XP_006506896.1;XP_006506897.1;XP_006506898.2;XP_011239846.1;XP_011239847.1;XP_011239848.1;XP_011239849.1;XP_011239851.1;XP_030111560.1;XP_355785.4	1918380	.	0.099060	[illegible]
71131	NM_175163	Zfp689	zinc finger protein 689	protein_coding	NP_780372.2	1918381	.	1.652922	[illegible]
71132	NM_001042418,NM_001042419,NM_001042420,NM_027687,NM_181731,XM_017317979	Cabyr	calcium-binding tyrosine-(Y)-phosphorylation regulated (fibrousheathin 2)	protein_coding	NP_001035883.1;NP_001035884.1;NP_001035885.1;NP_081963.1;NP_859420.2;XP_017173468.1	1918382	.	0.202778	[illegible]
71133	NR_040715	4933422A05Rik	RIKEN cDNA 4933422A05 gene	lncRNA	.	1918383	.	0.000000	[illegible]
71134	NR_040454	4933417E11Rik	RIKEN cDNA 4933417E11 gene	lncRNA		1918384	.	0.086693	[illegible]
71137	NM_001024918,NM_027689,XM_006514113,XM_006514114,XM_006514115,XM_030245278,XM_030245279	Rfx4	regulatory factor X, 4 (influences HLA class II expression)	protein_coding	NP_001020089.1;NP_081965.2;XP_006514176.1;XP_006514177.1;XP_006514178.1;XP_030101138.1;XP_030101139.1	1918387	.	0.033348	[illegible]
71141	NR_037692	4933407L21Rik	RIKEN cDNA 4933407L21 gene	lncRNA	.	1918391	.	0.188574	[illegible]
71145	NM_001168318,NM_028903	Scara5	scavenger receptor class A, member 5	protein_coding	NP_001161790.1;NP_083179.2	1918395	.	8.545527	[illegible]
71146	NM_001141983,NM_027694,XM_030251076,XM_030251077	Golga7b	golgi autoantigen, golgin subfamily a, 7B	protein_coding	NP_001135455.1;NP_081970.1;XP_030106936.1;XP_030106937.1	1918396	.	0.049691	[illegible]
71147	NM_001359882,NM_001359883,NM_027695,XM_006518101,XM_006518102,XM_006518103	Oxsm	3-oxoacyl-ACP synthase, mitochondrial	protein_coding	NP_001346811.1;NP_001346812.1;NP_081971.1;XP_006518164.1;XP_006518165.1;XP_006518166.1	1918397	.	3.374523	[illegible]
71148	NM_001039081,NM_001286221,NM_001286222,NM_001286223,NM_027696,XM_006503398,XM_006503399,XM_006503401,XM_006503402,XM_030253776,XM_030253777,XM_030253778,XM_030253779	Mier1	MEIR1 treanscription regulator	protein_coding	NP_001034170.1;NP_001273150.1;NP_001273151.1;NP_001273152.1;NP_081972.2;XP_006503461.1;XP_006503462.1;XP_006503464.1;XP_006503465.1;XP_030109636.1;XP_030109637.1;XP_030109638.1;XP_030109639.1	1918398	.	16.794849	[illegible]
71149	NM_027697	Tex52	testis expressed 52	protein_coding	NP_081973.1	1918399	.	0.190100	[illegible]
71151	NM_001355104,NM_027698,XM_006508207,XM_006508210,XM_017312302,XM_030242988,XM_030242989	Eri2	exoribonuclease 2	protein_coding	NP_001342033.1;NP_081974.3;XP_006508270.1;XP_006508273.1;XP_017167791.1;XP_030098848.1;XP_030098849.1	1918401	.	4.661627	[illegible]
71153	NR_045842	4933417O13Rik	RIKEN cDNA 4933417O13 gene	lncRNA	.	1918403	.	0.063938	[illegible]
71157	XR_869208	4933423K11Rik	RIKEN cDNA 4933423K11 gene	lncRNA	.	1918407	.	0.000000	[illegible]
71161	NR_040684	Prkag2os1	protein kinase, AMP-activated, gamma 2 non-catalytic subunit, opposite strand 1	lncRNA	.	1918411	.	0.000000	[illegible]
71163	NM_027703,XM_017312303,XM_017312304,XM_017312305	Zfp626	zinc finger protein 626	protein_coding	NP_081979.1;XP_017167792.1;XP_017167793.1;XP_017167794.1	1918413	.	3.210656	[illegible]
71166	NR_040290	4933424G06Rik	RIKEN cDNA 4933424G06 gene	lncRNA	.	1918416	.	0.000000	[illegible]

71670	NM_001302479,NM_001302480,NM_001302481,NM_001302482	Acy3	aspartoacylase (aminoacylase) 3	protein_coding	NP_001289408.1;NP_001289409.1;NP_001289410.1;NP_001289411.1	1918920	.	1.640701	[illegible]
71673	NM_001363143,NM_027859,XR_003949509,XR_380981	Rnf215	ring finger protein 215	protein_coding	NP_001350072.1;NP_082135.2	1918923	.	15.438749	[illegible]
71675	NM_001347242,NM_027860,NR_156450,XM_006514808,XM_006514810,XM_006514811,XM_011243749,XM_011243750,XM_017314768,XM_017314769,XM_030246327,XM_030246329	0610010F05Rik	RIKEN cDNA 0610010F05 gene	protein_coding	NP_001334171.1;NP_082136.2;XP_006514871.1;XP_006514873.1;XP_006514874.1;XP_011242051.1;XP_011242052.1;XP_017170257.1;XP_017170258.1;XP_030102187.1;XP_030102189.1	1918925	.	6.066345	[illegible]
71678	NM_001357242,NM_027861,NR_151660,XM_006535598,XM_006535599,XM_006535600,XM_030243025,XR_389720	Brox	BRO1 domain and CAAX motif containing	protein_coding	NP_001344171.1;NP_082137.1;XP_006535661.1;XP_006535662.1;XP_006535663.1;XP_030098885.1	1918928	.	18.724918	[illegible]
71679	NM_027862,XM_006534254	Atp5h	ATP synthase, H+ transporting, mitochondrial F0 complex, subunit D	protein_coding	NP_082138.1;XP_006534317.1	1918929	.	123.671958	[illegible]
71682	NM_175173,XM_006524925,XM_006524926,XM_006524928,XM_006524929,XM_006524931,XM_006524934,XM_006524936,XM_011246611,XM_011246612,XM_011246614,XM_011246616,XM_011246617,XM_017317639,XM_017317640,XM_017317641,XM_017317642,XR_001782112,XR_003952182	Wdr27	WD repeat domain 27	protein_coding	NP_780382.2;XP_006524988.1;XP_006524989.1;XP_006524991.1;XP_006524992.1;XP_006524994.1;XP_006524997.1;XP_006524999.1;XP_011244913.1;XP_011244914.1;XP_011244916.1;XP_011244918.1;XP_011244919.1;XP_017173128.1;XP_017173129.1;XP_017173130.1;XP_017173131.1	1918932	.	0.146635	[illegible]
71683	NM_001048207,NM_001347610	Gypc	glycophorin C	protein_coding	NP_001041672.1;NP_001334539.1	1098566	.	13.618274	[illegible]
71684	NM_001141981,NM_001141982,NM_001355604,NM_001355606,NM_001355607,NM_001355608,NM_030243,XM_006498340,XM_006498341,XM_006498343,XM_006498345,XM_011239185,XM_011239187,XM_030252091	Rbm43	RNA binding motif protein 43	protein_coding	NP_001135453.1;NP_001135454.1;NP_001342533.1;NP_001342535.1;NP_001342536.1;NP_001342537.1;NP_084519.1;XP_006498403.1;XP_006498404.1;XP_006498406.1;XP_006498408.1;XP_011237487.1;XP_011237489.1;XP_030107591.1	1918934	.	5.365621	[illegible]
71685	NM_027864,XM_006524940,XM_006524941,XR_003952183	Galnt14	polypeptide N-acetylgalactosaminyltransferase 14	protein_coding	NP_082140.2;XP_006525003.1;XP_006525004.1	1918935	.	0.013411	[illegible]
71687	NM_001357383,NM_001357384,NM_001357385,NM_001357386,NM_027865,NR_151678,NR_151679,XM_006510610,XM_011242601,XM_011242602,XM_011242603,XM_030244609,XM_030244610,XM_030244611,XM_030244612,XM_030244613,XM_030244614,XM_030244615,XM_030244616	Tmem25	transmembrane protein 25	protein_coding	NP_001344312.1;NP_001344313.1;NP_001344314.1;NP_001344315.1;NP_082141.1;XP_006510673.1;XP_011240903.1;XP_011240904.1;XP_011240905.1;XP_030100469.1;XP_030100470.1;XP_030100471.1;XP_030100472.1;XP_030100473.1;XP_030100474.1;XP_030100475.1;XP_030100476.1	1918937	.	0.897452	[illegible]
71690	NM_023612	Esm1	endothelial cell-specific molecule 1	protein_coding	NP_076101.1	1918940	.	2.171336	[illegible]
[illegible]	[illegible]	[illegible]	[illegible]	[illegible]	[illegible]	[illegible]	[illegible]	[illegible]	[illegible]

71941	NM_001359137,NM_024248,XM_006508890,XM_006508898,XM_011242082,XM_030243774,XM_030243775,XM_030243776,XM_030243777,XM_030243778,XM_030243779,XM_030243780,XM_030243781	Cars2	cysteinyl-tRNA synthetase 2 (mitochondrial)(putative)	protein_coding	NP_001346066.1;NP_077210.1;XP_006508953.1;XP_006508961.1;XP_011240384.1;XP_030099634.1;XP_030099635.1;XP_030099636.1;XP_030099637.1;XP_030099638.1;XP_030099639.1;XP_030099640.1;XP_030099641.1	1919191	.	[illegible]
71943	NM_001357543,NM_001357544,NM_001357547,NM_028011,XM_006534269	Tom1l1	target of myb1-like 1 (chicken)	protein_coding	NP_001344472.1;NP_001344473.1;NP_001344476.1;NP_082287.1;XP_006534332.1	1919193	.	[illegible]
71946	NM_028013,XM_017313601	Endod1	endonuclease domain containing 1	protein_coding	NP_082289.2;XP_017169090.1	1919196	.	[illegible]
71947	NM_028014,XM_006534270,XM_006534271,XM_017314773,XM_017314774,XM_030246336,XM_030246337,XM_030246338,XM_030246339,XR_001780054	Tmem94	transmembrane protein 94	protein_coding	NP_082290.2;XP_006534333.1;XP_006534334.1;XP_017170262.1;XP_017170263.1;XP_030102196.1;XP_030102197.1;XP_030102198.1;XP_030102199.1	1919197	.	[illegible]
71949	NM_028015	Cers5	ceramide synthase 5	protein_coding	NP_082291.1	1919199	.	[illegible]
71950	NM_001289828,NM_001289830,NM_001289831,NM_028016	Nanog	Nanog homeobox	protein_coding	NP_001276757.1;NP_001276759.1;NP_001276760.1;NP_082292.1	1919200	.	[illegible]
71951	NM_172412,XM_006504621,XM_006504623,XM_006504624,XM_017321104,XM_030254816	Gpc2	glypican 2 (cerebroglycan)	protein_coding	NP_766000.1;XP_006504684.1;XP_006504686.1;XP_006504687.1;XP_017176593.1;XP_030110676.1	1919201	.	[illegible]
71952	NM_023633	Riox1	ribosomal oxygenase 1	protein_coding	NP_076122.2	1919202	.	[illegible]
71954	NM_001122666,NM_178622	Suds3	suppressor of defective silencing 3 homolog (S. cerevisiae)	protein_coding	NP_001116138.1;NP_848737.3	1919204	.	[illegible]
71955	NM_001361562,NM_028018,NR_155034,XM_017312971,XR_001778468,XR_001778470,XR_003947337	Ist1	increased sodium tolerance 1 homolog (yeast)	protein_coding	NP_001348491.1;NP_082294.1;XP_017168460.1	1919205	.	[illegible]
71956	NM_028019,NR_157298	Rnf135	ring finger protein 135	protein_coding	NP_082295.1	1919206	.	[illegible]
71957	NM_028020,XM_006539192,XM_006539194,XM_006539195,XM_011250335,XM_030253801,XM_030253802,XM_030253803,XM_030253804	Ints11	integrator complex subunit 11	protein_coding	NP_082296.1;XP_006539255.1;XP_006539257.1;XP_006539258.1;XP_011248637.1;XP_030109661.1;XP_030109662.1;XP_030109663.1;XP_030109664.1	1919207	.	[illegible]
71960	NM_001271538,NM_001271540,NM_028021,XM_011250902,XM_011250903,XM_017312313,XM_030242996,XM_030242997	Myh14	myosin, heavy polypeptide 14	protein_coding	NP_001258467.1;NP_001258469.1;NP_082297.1;XP_011249204.1;XP_011249205.1;XP_017167802.1;XP_030098856.1;XP_030098857.1	1919210	.	[illegible]
71962	NM_028022,XM_011243752	Castor1	cytosolic arginine sensor for mTORC1 subunit 1	protein_coding	NP_082298.1;XP_011242054.1	1919212	.	[illegible]
71963	NM_001359234,NM_001359292,NM_028023	Cdca4	cell division cycle associated 4	protein_coding	NP_001346163.1;NP_001346221.1;NP_082299.1	1919213	.	[illegible]
71966	NM_028024,XM_030246341	Nkiras2	NFKB inhibitor interacting Ras-like protein 2	protein_coding	NP_082300.1;XP_030102201.1	1919216	.	[illegible]
71968	NM_028026,NR_027976,NR_152100,NR_152101,XM_006541206,XM_006541209,XM_011250904	Wdr73	WD repeat domain 73	protein_coding	NP_082302.2;XP_006541269.1;XP_006541272.1;XP_011249206.1	1919218	.	[illegible]
71970	NM_001359408,NM_183088,XM_017321105,XM_017321106,XM_017321107	Zbed5	zinc finger, BED type containing 5	protein_coding	NP_001346337.1;NP_898911.2;XP_017176594.1;XP_017176595.1;XP_017176596.1	1919220	.	[illegible]
71971	NM_028028	Zswim1	zinc finger SWIM-type containing 1	protein_coding	NP_082304.1	1919221	.	[illegible]
71972	NM_001306088,NM_028029,XM_006527350,XM_006527351,XM_006527352,XM_006527354	Dnmbp	dynamin binding protein	protein_coding	NP_001293017.1;NP_082305.1;XP_006527413.4;XP_006527414.1;XP_006527415.1;XP_006527417.1	1917352	.	[illegible]
71973	NM_028030,XM_006511455,XM_006511456,XM_006511457,XM_006511458	Rbpms2	RNA binding protein with multiple splicing 2	protein_coding	NP_082306.2;XP_006511518.1;XP_006511519.1;XP_006511520.1;XP_006511521.1	1919223	.	[illegible]
71974	NM_133740,XR_003946565	Prmt3	protein arginine N-methyltransferase 3	protein_coding	NP_598501.1	1919224	.	[illegible]
71978	NM_001205188,NM_001360033,NM_001360034,NM_028032	Ppp2r2a	protein phosphatase 2, regulatory subunit B, alpha	protein_coding	NP_001192117.1;NP_001346962.1;NP_001346963.1;NP_082308.1	1919228	.	[illegible]
71981	NM_001368387,NM_028034,XM_011250704,XM_011250707,XM_030242998,XM_030242999,XM_030243000,XM_030243001,XM_030243002,XR_001778000	Tdrd12	tudor domain containing 12	protein_coding	NP_001355316.1;NP_082310.1;XP_011249006.1;XP_011249009.1;XP_030098858.1;XP_030098859.1;XP_030098860.1;XP_030098861.1;XP_030098862.1	1919231	.	[illegible]
71982	NM_001127348,NM_001127349,NM_001361576,NM_001361577,NM_001361578,NM_001361579,NM_001361580,NM_001361581,NM_001361582,NM_028035,XM_011241474,XM_030255600	Snx10	sorting nexin 10	protein_coding	NP_001120820.1;NP_001120821.1;NP_001348505.1;NP_001348506.1;NP_001348507.1;NP_001348508.1;NP_001348509.1;NP_001348510.1;NP_001348511.1;NP_082311.3;XP_011239776.1;XP_030111460.	1919232	.	[illegible]
71983	NM_028036,XM_006526276,XM_006526277,XM_030250611,XM_030250612	Tmco6	transmembrane and coiled-coil domains 6	protein_coding	NP_082312.3;XP_006526339.1;XP_006526340.1;XP_030106471.1;XP_030106472.1	1919233	.	[illegible]
71984	NM_023637,XM_006540375,XM_006540376,XR_003946566	Sars2	seryl-aminoacyl-tRNA synthetase 2	protein_coding	NP_076126.2;XP_006540438.1;XP_006540439.1	1919234	.	[illegible]
71985	NM_028037,XM_006530458,XM_006530459,XM_006530460,XM_006530461,XM_006530462,XM_006530463,XM_030254817,XM_030254818	Acad10	acyl-Coenzyme A dehydrogenase family, member 10	protein_coding	NP_082313.2;XP_006530521.1;XP_006530522.1;XP_006530523.1;XP_006530524.1;XP_006530525.1;XP_006530526.1;XP_030110677.1;XP_030110678.1	1919235	.	[illegible]
71986	NM_028038	Ddx28	DEAD (Asp-Glu-Ala-Asp) box polypeptide 28	protein_coding	NP_082314.2	1919236	.	[illegible]
71988	NM_028039	Esco2	establishment of sister chromatid cohesion N-acetyltransferase 2	protein_coding	NP_082315.3	1919238	.	[illegible]
71989	NM_028040	Rpusd4	RNA pseudouridylate synthase domain containing 4	protein_coding	NP_082316.1	1919239	.	[illegible]
71990	NM_028041,XR_878688	Ddx54	DEAD (Asp-Glu-Ala-Asp) box polypeptide 54	protein_coding	NP_082317.1	1919240	.	[illegible]
71991	NM_001362403,NM_028042,NR_155552,XM_017315608,XR_001780818,XR_003950484,XR_003950485,XR_382743,XR_382744	Ercc8	excision repaiross-complementing rodent repair deficiency, complementation group 8	protein_coding	NP_001349332.1;NP_082318.2;XP_017171097.1	1919241	.	[illegible]
71994	NM_028044	Cnn3	calponin 3, acidic	protein_coding	NP_082320.1	1919244	.	[illegible]
71995	NM_001166206	Erv3	endogenous retroviral sequence 3	protein_coding	NP_001159678.1	1919245	.	[illegible]
71996	NM_028046,XM_011247684	Fthl17a	ferritin, heavy polypeptide-like 17, member A	protein_coding	NP_082322.1;XP_011245986.1	1919246	.	[illegible]
71997	NM_028047,XM_006540377,XM_006540378,XM_030243003	Smg9	smg-9 homolog, nonsense mediated mRNA decay factor (C. elegans)	protein_coding	NP_082323.1;XP_006540440.1;XP_006540441.1;XP_030098863.1	1919247	.	[illegible]
71998	NM_028048,XM_006534273,XM_006534274,XM_006534275,XM_006534276	Slc25a35	solute carrier family 25, member 35	protein_coding	NP_082324.1;XP_006534336.1;XP_006534337.1;XP_006534338.1;XP_006534339.1	1919248	.	[illegible]
71999	NM_028049,XM_006511459	Fbxo22	F-box protein 22	protein_coding	NP_082325.2;XP_006511522.1	1926014	.	[illegible]
72000	NM_001347565,NM_028050,XM_006536256,XM_006536257,XM_006536258,XM_006536259,XM_011249859,XM_017312319,XM_030243004,XM_030243005,XM_030243006,XM_030243007,XR_003946567,XR_880984	Lmntd2	lamin tail domain containing 2	protein_coding	NP_001334494.1;NP_082326.1;XP_006536319.1;XP_006536320.1;XP_006536321.1;XP_006536322.1;XP_011248161.1;XP_017167808.1;XP_030098864.1;XP_030098865.1;XP_030098866.1;XP_030098867.1	1919250	.	[illegible]
72002	NM_001136237,NM_028051,NM_028092,XM_006514172,XM_006514173,XM_011243579,XR_871732	Slc39a5	solute carrier family 39 (metal ion transporter), member 5	protein_coding	NP_001129709.1;NP_082327.2;NP_082368.1;XP_006514235.1;XP_006514236.1;XP_011241881.1	1919336	.	[illegible]
72003	NM_001163032,NM_028052	Synpr	synaptoporin	protein_coding	NP_001156504.1;NP_082328.3	1919253	.	[illegible]
72007	NM_001356953,NM_173182,XM_006535541,XM_006535544,XM_006535546,XM_006535547,XM_011249711,XM_011249712,XM_017319738,XM_030252836	Fndc3b	fibronectin type III domain containing 3B	protein_coding	NP_001343882.1;NP_775274.2;XP_006535604.1;XP_006535607.1;XP_006535609.1;XP_006535610.1;XP_011248013.1;XP_011248014.1;XP_017175227.1;XP_030108696.1	1919257	.	[illegible]
72008	NM_001164827,NM_028054,XM_006500203,XM_006500204,XM_006500205,XM_006500207,XM_011239781,XM_030252098,XM_030252099	Zfyve19	zinc finger, FYVE domain containing 19	protein_coding	NP_001158299.1;NP_082330.2;XP_006500266.1;XP_006500267.1;XP_006500268.1;XP_006500270.1;XP_011238083.1;XP_030107958.1;XP_030107959.1	1919258	.	[illegible]
72012	NR_037960,NR_037961	1600020E01Rik	RIKEN cDNA 1600020E01 gene	lncRNA	.	1919262	.	[illegible]
72014	NM_028055,XM_006534278,XM_006534280,XM_017314777,XM_017314778	Btbd17	BTB (POZ) domain containing 17	protein_coding	NP_082331.1;XP_006534341.1;XP_006534343.1;XP_017170266.1;XP_017170267.1	1919264	.	[illegible]
72016	NM_028056,XM_011246632,XM_030250058,XM_030250059	Tedc2	tubulin epsilon and delta complex 2	protein_coding	NP_082332.1;XP_011244934.1;XP_030105918.1;XP_030105919.1	1919266	.	[illegible]
72017	NM_001355133,NM_028057,NR_149307	Cyb5r1	cytochrome b5 reductase 1	protein_coding	NP_001342062.1;NP_082333.1	1919267	.	[illegible]
72018	NM_001313745,NM_001313746,NM_028058,XM_011247496	Fundc1	FUN14 domain containing 1	protein_coding	NP_001300674.1;NP_001300675.1;NP_082334.1;XP_011245798.1	1919268	.	[illegible]
72020	NM_001347245,XM_011246018,XR_003951833	Zfp654	zinc finger protein 654	protein_coding	NP_001334174.1;XP_011244320.1	1919270	.	[illegible]
72022	NM_028060,XM_006511460,XM_006511461,XM_006511462	Slc35f2	solute carrier family 35, member F2	protein_coding	NP_082336.3;XP_006511523.1;XP_006511524.1;XP_006511525.1	1919272	.	[illegible]
72023	NM_001081320,NM_028061,XM_006502126,XM_006502127,XM_006502128	Cyb561d1	cytochrome b-561 domain containing 1	protein_coding	NP_001074789.2;NP_082337.1;XP_006502189.1;XP_006502190.1;XP_006502191.1	1919273	.	[illegible]
72025	NR_163823	1600012P17Rik	RIKEN cDNA 1600012P17 gene	lncRNA	.	1919275	.	[illegible]
72026	NM_028063,XM_006521422,XM_006521423,XM_006521424,XM_006521425,XR_001781567,XR_384047	Trmu	tRNA 5-methylaminomethyl-2-thiouridylate methyltransferase	protein_coding	NP_082339.1;XP_006521485.1;XP_006521486.1;XP_006521487.1;XP_006521488.1	1919276	.	[illegible]
72027	NM_028064,XM_006521427	Slc39a4	solute carrier family 39 (zinc transporter), member 4	protein_coding	NP_082340.1;XP_006521490.1	1919277	.	[illegible]
72029	NM_001305988,NM_001305989,NM_001305990,NM_028065,XM_030250060,XR_003952187	Cnpy3	canopy FGF signaling regulator 3	protein_coding	NP_001292917.1;NP_001292918.1;NP_001292919.1;NP_082341.1;XP_030105920.1	1919279	.	[illegible]
72033	NM_001081229,NM_001347233,XR_003954394,XR_003954395	Tsc22d2	TSC22 domain family, member 2	protein_coding	NP_001074698.1;NP_001334162.1	1919283	.	[illegible]
72039	NM_023644,XR_001783756,XR_001783757,XR_001783758	Mccc1	methylcrotonoyl-Coenzyme A carboxylase 1 (alpha)	protein_coding	NP_076133.3	1919289	.	[illegible]
72040	NM_001114322,NM_028069,XM_006536260	Cdhr5	cadherin-related family member 5	protein_coding	NP_001107794.1;NP_082345.1;XP_006536323.1	1919290	.	[illegible]
72041	NM_001347492,NM_001359414,NM_001359415,NM_001359416,NM_001359417,NM_028070,XM_006504505,XM_030254819	Alkbh4	alkB homolog 4, lysine demethylase	protein_coding	NP_001334421.1;NP_001346343.1;NP_001346344.1;NP_001346345.1;NP_001346346.1;NP_082346.1;XP_006504568.1;XP_030110679.1	1919291	.	[illegible]
72042	NM_028071	Cotl1	coactosin-like 1 (Dictyostelium)	protein_coding	NP_082347.1	1919292	.	[illegible]
72043	NM_001252578,NM_001252579,NM_001355619,NM_028072,XM_006500209,XM_006500211,XM_030252100,XM_030252101	Sulf2	sulfatase 2	protein_coding	NP_001239507.1;NP_001239508.1;NP_001342548.1;NP_082348.2;XP_006500272.1;XP_006500274.1;XP_030107960.1;XP_030107961.1	1919293	.	[illegible]
72045	NR_160807	Mfsd4b2-ps	major facilitator superfamily domain containing 4B2, pseudogene	transcribed_pseudogene	.	1919295	.	[illegible]
72046	NM_001077661,NM_001361588,NM_001361589,NM_001361590,NM_001361591,NM_001361592,NM_178623,XM_006514815,XM_011243753,XM_011243754,XM_011243755,XM_011243757	Urgcp	upregulator of cell proliferation	protein_coding	NP_001071129.1;NP_001348517.1;NP_001348518.1;NP_001348519.1;NP_001348520.1;NP_001348521.1;NP_848738.2;XP_006514878.1;XP_011242055.1;XP_011242056.1;XP_011242057.1;XP_011242059.	1919296	.	[illegible]
72047	NM_001361640,NM_001361642,NM_001361643,NM_001361644,NM_028074,XM_030246345	Ddx42	DEAD (Asp-Glu-Ala-Asp) box polypeptide 42	protein_coding	NP_001348569.1;NP_001348571.1;NP_001348572.1;NP_001348573.1;NP_082350.3;XP_030102205.1	1919297	.	[illegible]
72049	NM_001357758,NM_028075	Tnfrsf13c	tumor necrosis factor receptor superfamily, member 13c	protein_coding	NP_001344687.1;NP_082351.1	1919299	.	[illegible]
72050	NM_023645,XR_003946583	Poglut2	protein O-glucosyltransferase 2	protein_coding	NP_076134.1	1919300	.	[illegible]
72053	NM_001302505,NM_001302506,NM_028076,XM_030246346	Tmub2	transmembrane and ubiquitin-like domain containing 2	protein_coding	NP_001289434.1;NP_001289435.2;NP_082352.1;XP_030102206.1	1919303	.	[illegible]
72054	NM_024444,XM_006509748,XM_006509749,XM_011242327,XM_030243782	Cyp4f18	cytochrome P450, family 4, subfamily f, polypeptide 18	protein_coding	NP_077764.2;XP_006509811.1;XP_006509812.1;XP_011240629.1;XP_030099642.1	1919304	.	[illegible]
72055	NM_001164798,NM_001164799,NM_001164800,NM_001164801,NM_001164802,NM_001361696,NM_001361697,NM_001361698,NM_001361699,NM_024249,XM_006534284,XM_006534285,XM_006534286,XM_006534287,XM_006534288,XM_006534290,XM_006534291,XM_017314782,XM_017314785,XM_017314786,XM_030246349,XM_030246350,XM_030246351,XM_030246352,XM_030246353,XM_030246354,XM_030246355,XM_030246356,XM_030246357	Slc38a10	solute carrier family 38, member 10	protein_coding	NP_001158270.1;NP_001158271.1;NP_001158272.1;NP_001158273.1;NP_001158274.1;NP_001348625.1;NP_001348626.1;NP_001348627.1;NP_001348628.1;NP_077211.4;XP_006534347.1;XP_006534348.1;XP_006534349.1;XP_006534350.1;XP_006534351.1;XP_006534353.1;XP_006534354.1;XP_017170271.1;XP_017170274.1;XP_017170275.1;XP_030102209.1;XP_030102210.1;XP_030102211.1;XP_030102212.1;XP_030102213.1;XP_030102214.1;XP_030102215.1;XP_030102216.1;XP_030102217.1	1919305	.	[illegible]
72056	NM_001358256,NM_001358257,NM_001358258,NM_028077	1810055G02Rik	RIKEN cDNA 1810055G02 gene	protein_coding	NP_001345185.1;NP_001345186.1;NP_001345187.1;NP_082353.1	1919306	.	[illegible]
72057	NM_001360983,NM_024250	Phf10	PHD finger protein 10	protein_coding	NP_001347912.1;NP_077212.3	1919307	.	[illegible]
72058	NM_001177886,NM_001177887,NM_028078,XM_006523095,XM_011246151,XM_011246152,XM_011246153	Igsf5	immunoglobulin superfamily, member 5	protein_coding	NP_001171357.1;NP_001171358.1;NP_082354.1;XP_006523158.1;XP_011244453.1;XP_011244454.1;XP_011244455.1	1919308	.	[illegible]
72061	NM_001289924,NM_001289926,NR_110520,NR_110521	Aopep	aminopeptidase O	protein_coding	NP_001276853.1;NP_001276855.1	1919311	.	[illegible]
72065	NM_172413,XM_006541545,XM_006541546	Rap2c	RAP2C, member of RAS oncogene family	protein_coding	NP_766001.1;XP_006541608.1;XP_006541609.1	1919315	.	[illegible]
72068	NM_001037846,NM_001037847,NM_001037848,NM_001359247,NM_001359248,NM_001359249,NM_001359250,NM_001359251,NM_001359252,NM_001359253,NM_001359563,NM_028082,XM_006514174,XM_006514175,XM_006514181,XM_006514182,XM_006514183,XM_006514184,XM_006514186,XM_006514188,XM_006514189,XM_006514190,XM_006514191,XM_017314107,XM_017314111,XM_030245296,XM_030245297,XM_030245298,XM_030245299,XM_030245300,XM_030245301,XM_030245302	Cnot2	CCR4-NOT transcription complex, subunit 2	protein_coding	NP_001032935.2;NP_001032936.2;NP_001032937.1;NP_001346176.1;NP_001346177.1;NP_001346178.1;NP_001346179.1;NP_001346180.1;NP_001346181.1;NP_001346182.1;NP_001346492.1;NP_082358.2;XP_006514237.1;XP_006514238.1;XP_006514244.1;XP_006514245.1;XP_006514246.1;XP_006514247.1;XP_006514249.1;XP_006514251.1;XP_006514252.1;XP_006514253.1;XP_006514254.1;XP_017169596.1;XP_017169600.1;XP_030101156.1;XP_030101157.1;XP_030101158.1;XP_030101159.1;XP_030101160.1;XP_030101161.1;XP_	1919318	.	[illegible]
72075	NM_031373,XM_006500711,XM_017319269	Ogfr	opioid growth factor receptor	protein_coding	NP_113550.3;XP_006500774.1;XP_017174758.1	1919325	.	[illegible]
72076	NR_045438	Mospd4	motile sperm domain containing 4	transcribed_pseudogene	.	1919326	.	[illegible]
72080	NM_001081085,NM_001290407,XM_006498360	Sapcd2	suppressor APC domain containing 2	protein_coding	NP_001074554.1;NP_001277336.1;XP_006498423.1	1919330	.	[illegible]
72082	NM_028089,XM_006527355	Cyp2c55	cytochrome P450, family 2, subfamily c, polypeptide 55	protein_coding	NP_082365.1;XP_006527418.1	1919332	.	[illegible]
72083	NM_001359003,NM_029354	Mzt2	mitotic spindle organizing protein 2	protein_coding	NP_001345932.1;NP_083630.1	1922845	.	[illegible]
72084	NM_001111025,NM_024464	Pigx	phosphatidylinositol glycan anchor biosynthesis, class X	protein_coding	NP_001104495.1;NP_077784.2	1919334	.	[illegible]
72085	NM_001285839,NR_104360,NR_104361,NR_151661,XM_006496290,XM_030243109	Osgepl1	O-sialoglycoprotein endopeptidase-like 1	protein_coding	NP_001272768.1;XP_006496353.1;XP_030098969.1	1919335	.	[illegible]
72088	NM_001163733,NM_001291182,NM_023649,NM_153677,XM_006541212	Ush1c	USH1 protein network component harmonin	protein_coding	NP_001157205.1;NP_001278111.1;NP_076138.2;NP_710143.2;XP_006541275.1	1919338	.	[illegible]
72090	NM_028093,XM_011239191,XM_030252102,XM_030252103,XM_030252104	Entpd8	ectonucleoside triphosphate diphosphohydrolase 8	protein_coding	NP_082369.1;XP_011237493.1;XP_030107962.1;XP_030107963.1;XP_030107964.1	1919340	.	[illegible]
72091	NR_024068	Snhg7	small nucleolar RNA host gene 7	lncRNA	.	1919341	.	[illegible]
72093	NR_029440	2010320M18Rik	RIKEN cDNA 2010320M18 gene	lncRNA	.	1919343	.	[illegible]
72096	NM_001347629,NM_028095,XM_017312322	Eef1akmt2	EEF1A lysine methyltransferase 2	protein_coding	NP_001334558.1;NP_082371.1;XP_017167811.1	1919346	.	[illegible]
72097	NM_028096,XM_006496291,XM_006496292,XM_006496295,XM_006496296,XM_006496297	2010300C02Rik	RIKEN cDNA 2010300C02 gene	protein_coding	NP_082372.1;XP_006496354.2;XP_006496355.2;XP_006496358.1;XP_006496359.1;XP_006496360.1	1919347	.	[illegible]
72098	NM_028097,NR_149728,XM_006538269,XM_030253805,XM_030253806,XR_001784202	Tmem68	transmembrane protein 68	protein_coding	NP_082373.1;XP_006538332.1;XP_030109665.1;XP_030109666.1	1919348	.	[illegible]
72102	NM_028099,XM_011241476,XM_017321754,XR_001785172	Dusp11	dual specificity phosphatase 11 (RNA/RNP complex 1-interacting)	protein_coding	NP_082375.4;XP_011239778.1;XP_017177243.1	1919352	.	[illegible]
72103	NM_001170489,NM_024251,XM_006506656,XM_006506657,XM_017321755	Aplf	aprataxin and PNKP like factor	protein_coding	NP_001163960.1;NP_077213.2;XP_006506719.1;XP_006506720.1;XP_017177244.1	1919353	.	[illegible]
72106	NM_028101	Jmjd8	jumonji domain containing 8	protein_coding	NP_082377.3	1919356	.	[illegible]
72107	NM_001355594,NM_183089	Dscc1	DNA replication and sister chromatid cohesion 1	protein_coding	NP_001342523.1;NP_898912.2	1919357	.	[illegible]
72108	NM_001361595,NM_028102,XM_006509197,XM_006509198,XM_006509200,XM_011242160,XM_011242163,XM_011242164,XR_001778471,XR_001778472,XR_001778473,XR_003947339,XR_00394	Ddhd2	DDHD domain containing 2	protein_coding	NP_001348524.1;NP_082378.1;XP_006509260.2;XP_006509261.1;XP_006509263.2;XP_011240462.1;XP_011240465.1;XP_011240466.1	1919358	.	[illegible]
72113	NM_001277296,NM_001277297,NM_001361598,NM_001361599,NM_001361600,NM_001361601,NM_028105,XM_006516245,XM_006516246,XM_006516248,XM_006516251,XM_006516252,XM_030246933,XM_030246934,XM_030246935,XM_030246936,XM_030246937,XM_030246938,XM_030246939,XM_030246940	Adck1	aarF domain containing kinase 1	protein_coding	NP_001264225.1;NP_001264226.1;NP_001348527.1;NP_001348528.1;NP_001348529.1;NP_001348530.1;NP_082381.1;XP_006516308.1;XP_006516309.1;XP_006516311.1;XP_006516314.1;XP_006516315.1;XP_030102793.1;XP_030102794.1;XP_030102795.1;XP_030102796.1;XP_030102797.1;XP_030102798.1;XP_030102799.1;XP_030102	1919363	.	[illegible]
72114	NM_001302538,NM_001302539,NM_028106,XM_030247416	Zbed3	zinc finger, BED type containing 3	protein_coding	NP_001289467.1;NP_001289468.1;NP_082382.1;XP_030103276.1	1919364	.	[illegible]
72117	NM_001347239,NM_028108	Naa50	N(alpha)-acetyltransferase 50, NatE catalytic subunit	protein_coding	NP_001334168.1;NP_082384.1	1919367	.	[illegible]
72119	NM_001141975,NM_001141976,NM_001141977,NM_001141978,NM_028109,XM_006500213,XM_006500214,XM_006500215	Tpx2	TPX2, microtubule-associated	protein_coding	NP_001135447.1;NP_001135448.1;NP_001135449.1;NP_001135450.1;NP_082385.3;XP_006500276.1;XP_006500277.1;XP_006500278.1	1919369	.	[illegible]
72121	NM_001093754,NM_001278941,NM_028110,XM_006502133,XM_030252838,XR_003954396,XR_003954397,XR_003954398,XR_003954399	Dennd2d	DENN/MADD domain containing 2D	protein_coding	NP_001087223.1;NP_001265870.1;NP_082386.2;XP_006502196.1;XP_030108698.1	2181193	.	[illegible]
72122	NR_045429,NR_045430	2010308F09Rik	RIKEN cDNA 2010308F09 gene	lncRNA	.	1919372	.	[illegible]
72123	NM_001162903	Ccdc71l	coiled-coil domain containing 71 like	protein_coding	NP_001156375.1	1919373	.	[illegible]
72124	NM_001039088,NM_001360934,NM_028112	Seh1l	SEH1-like (S. cerevisiae	protein_coding	NP_001034177.1;NP_001347863.1;NP_082388.1	1919374	.	[illegible]
72125	NM_001164705,NM_001368677,NM_028113,XM_006519590	Amer2	APC membrane recruitment 2	protein_coding	NP_001158177.2;NP_001355606.1;NP_082389.2;XP_006519653.1	1919375	.	[illegible]
72128	NM_001004362,NM_001359715,NM_001359717,XM_017314115	2610008E11Rik	RIKEN cDNA 2610008E11 gene	protein_coding	NP_001004362.2;NP_001346644.1;NP_001346646.1;XP_017169604.	1919378	.	[illegible]
72129	NM_023651,NR_157299,XM_030246359	Pex13	peroxisomal biogenesis factor 13	protein_coding	NP_076140.2;XP_030102219.1	1919379	.	[illegible]
72133	NM_028115,NM_029839,XM_006527356,XM_006527357,XR_3865	Trub1	TruB pseudouridine (psi) synthase family member 1	protein_coding	NP_082391.1;NP_084115.2;XP_006527419.2;XP_006527420.2	1919383	.	[illegible]
72135	NM_028116	Pygo1	pygopus 1	protein_coding	NP_082392.1	1919385	.	[illegible]
72136	NM_028117	Chst14	carbohydrate sulfotransferase 14	protein_coding	NP_082393.3	1919386	.	[illegible]
72137	NM_001159636,NM_028118,XM_006500216,XM_006500217,XM_006500218,XM_006500219,XM_006500220,XM_006500221,XM_006500222,XM_006500223,XM_006500224,XM_006500225,XM_011239784,XM_011239786,XM_030252105,XM_030252106,XR_0039537	Wdsub1	WD repeat, SAM and U-box domain containing 1	protein_coding	NP_001153108.1;NP_082394.1;XP_006500279.1;XP_006500280.1;XP_006500281.1;XP_006500282.1;XP_006500283.1;XP_006500284.1;XP_006500285.1;XP_006500286.1;XP_006500287.1;XP_006500288.1;XP_011238086.1;XP_011238088.1;XP_030107965.1;XP_03010796	1919387	.	[illegible]
72139	NM_153780	2610044O15Rik8	RIKEN cDNA 2610044O15 gene	protein_coding	NP_722475.1	1919389	.	[illegible]
72140	NM_028120,NR_153799,XM_030243008,XR_003946568,XR_39110	Cep89	centrosomal protein 89	protein_coding	NP_082396.1;XP_030098868.1	1919390	.	[illegible]
72141	NM_001359282,NM_028121	Adpgk	ADP-dependent glucokinase	protein_coding	NP_001346211.1;NP_082397.1	1919391	.	[illegible]
72144	NM_001361617,NM_001361618,NM_001361619,NM_028123,NR_155076,NR_155077,XM_006506658,XM_006506659,XM_006506661,XM_006506663,XM_006506664,XM_006506665,XM_006506666,XM_006506667,XM_017321757,XM_017321758,XM_030255601,XM_030255602	Slc37a3	solute carrier family 37 (glycerol-3-phosphate transporter), member 3	protein_coding	NP_001348546.1;NP_001348547.1;NP_001348548.1;NP_082399.3;XP_006506721.1;XP_006506722.1;XP_006506724.1;XP_006506726.1;XP_006506727.1;XP_006506728.1;XP_006506729.1;XP_006506730.1;XP_017177246.1;XP_017177247.1;XP_030111461.1;XP_030111462.1	1919394	.	[illegible]
72145	NM_172882,XM_006535220,XM_006535221,XM_006535222,XM_006535223,XM_006535224,XM_006535225,XM_006535226,XM_006535227,XM_006535228,XM_030254820,XM_030254821	Wdfy3	WD repeat and FYVE domain containing 3	protein_coding	NP_766470.2;XP_006535283.1;XP_006535284.1;XP_006535285.1;XP_006535286.1;XP_006535287.1;XP_006535288.1;XP_006535289.1;XP_006535290.1;XP_006535291.1;XP_030110680.1;XP_030110681.	1096875	.	[illegible]
72147	NM_027656,NM_028125,XM_006500712,XM_006500713,XM_006500714,XM_006500715,XM_017319271,XR_001783183,XR_375402,XR_867136	Zbtb46	zinc finger and BTB domain containing 46	protein_coding	NP_081932.1;NP_082401.1;XP_006500775.1;XP_006500776.1;XP_006500777.1;XP_006500778.1;XP_017174760.1	1919397	.	[illegible]
72148	NM_001361625,NM_001361628,NM_001361629,NM_001361630,NM_173744,XM_011242083,XM_011242084,XM_011242085,XM_030243783,XM_030243784	Tdrp	testis development related protein	protein_coding	NP_001348554.1;NP_001348557.1;NP_001348558.1;NP_001348559.1;NP_776105.1;XP_011240385.1;XP_011240386.1;XP_011240387.1;XP_030099643.1;XP_030099644.1	1919398	.	[illegible]
72149	NM_001252448,NM_001252449,NM_028126,XM_006534293,XM_006534294,XM_006534295,XM_006534296,XM_006534297,XM_011249270,XM_030246360	Strada	STE20-related kinase adaptor alpha	protein_coding	NP_001239377.1;NP_001239378.1;NP_082402.1;XP_006534356.1;XP_006534357.1;XP_006534358.1;XP_006534359.1;XP_006534360.1;XP_011247572.1;XP_030102220.1	1919399	.	[illegible]
72151	NM_028128	Rfc5	replication factor C (activator 1) 5	protein_coding	NP_082404.1	1919401	.	[illegible]
72154	NM_028130,XM_006504625,XM_006504626,XM_006504627,XM_030254822	Zfp157	zinc finger protein 157	protein_coding	NP_082406.2;XP_006504688.1;XP_006504689.1;XP_006504690.1;XP_030110682.1	1919404	.	[illegible]
72155	NM_028131,XM_006531387	Cenpn	centromere protein N	protein_coding	NP_082407.1;XP_006531450.1	1919405	.	[illegible]
72157	NM_028132	Pgm2	phosphoglucomutase 2	protein_coding	NP_082408.3	97565	.	[illegible]
72159	gene-Gm9726	Gm9726	predicted gene 9726	pseudogene	.	3648149	.	[illegible]
72160	NM_028135	Tmem163	transmembrane protein 163	protein_coding	NP_082411.1	1919410	.	[illegible]
72162	NM_028136,XM_011240238,XM_011240240,XM_030252839,XM_030252840	Dhx36	DEAH (Asp-Glu-Ala-His) box polypeptide 36	protein_coding	NP_082412.2;XP_011238540.1;XP_011238542.1;XP_030108699.1;XP_030108700.1	1919412	.	[illegible]
72167	NM_028138,XM_011246636,XM_011246637,XM_011246639,XM_017317655,XM_017317656,XM_030250061,XR_001782115,XR_001782116,XR_003952188,XR_003952189,XR_876548	Thumpd2	THUMP domain containing 2	protein_coding	NP_082414.1;XP_011244938.1;XP_011244939.1;XP_011244941.1;XP_017173144.1;XP_017173145.1;XP_030105921.1	1919417	.	[illegible]
72168	NM_001291070,NM_175178,XM_006522590,XM_006522591,XM_006522592,XM_006522593,XM_006522594,XM_006522595,XM_017317136,XR_001781867,XR_001781868,XR_003951834,XR_003951835,XR_003951836,XR_003951837	Aifm3	apoptosis-inducing factor, mitochondrion-associated 3	protein_coding	NP_001277999.1;NP_780387.2;XP_006522653.1;XP_006522654.1;XP_006522655.1;XP_006522656.1;XP_006522657.1;XP_006522658.1;XP_017172625.1	1919418	.	[illegible]
72169	NM_023655,XM_006510616,XM_006510617	Trim29	tripartite motif-containing 29	protein_coding	NP_076144.2;XP_006510679.1;XP_006510680.1	1919419	.	[illegible]
72170	NM_133928	Chchd4	coiled-coil-helix-coiled-coil-helix domain containing 4	protein_coding	NP_598689.1	1919420	.	[illegible]
72171	NM_181590,XM_006506668,XM_006506669,XM_006506670	Shq1	SHQ1 homolog (S. cerevisiae)	protein_coding	NP_853621.2;XP_006506731.1;XP_006506732.1;XP_006506733.1	1919421	.	[illegible]
72175	NM_028140,XM_006500788,XR_375453,XR_375454,XR_867172	Mfsd8	major facilitator superfamily domain containing 8	protein_coding	NP_082416.2;XP_006500851.1	1919425	.	[illegible]
72179	NM_178624,XM_006512295,XM_011243027,XM_030244622,XM_030244623,XR_003948011,XR_003948012,XR_379878,XR_379879,XR_379880	Fbxl2	F-box and leucine-rich repeat protein 2	protein_coding	NP_848739.1;XP_006512358.1;XP_011241329.1;XP_030100482.1;XP_030100483.1	1919429	.	[illegible]
72180	NM_001111029,NM_001355622,NM_001355623,NM_028141,XM_011239787,XM_011239788,XM_030252107,XM_030252108,XM_030252109,XM_030252111	Zfp661	zinc finger protein 661	protein_coding	NP_001104499.1;NP_001342551.1;NP_001342552.1;NP_082417.3;XP_011238089.1;XP_011238090.1;XP_030107967.1;XP_030107968.1;XP_030107969.1;XP_030107971.1	1919430	.	[illegible]
72181	NM_001369075,NM_001369076,NM_028142,XR_003955016,XR_376358	Nsun4	NOL1/NOP2/Sun domain family, member 4	protein_coding	NP_001356004.1;NP_001356005.1;NP_082418.1	1919431	.	[illegible]
72183	NM_026998,XM_006516253	Snx6	sorting nexin 6	protein_coding	NP_081274.2;XP_006516316.1	1919433	.	[illegible]
72184	NM_028145,XM_006508220	Klhl35	kelch-like 35	protein_coding	NP_082421.1;XP_006508283.1	1919434	.	[illegible]
72185	NM_001170975,NM_001170976,NM_028146,XM_006531388,XM_006531389,XM_011248509,XM_011248510	Dbndd1	dysbindin (dystrobrevin binding protein 1) domain containing 1	protein_coding	NP_001164446.2;NP_001164447.2;NP_082422.3;XP_006531451.1;XP_006531452.1;XP_011246811.1;XP_011246812.1	1919435	.	[illegible]
72190	NM_001001881	2510009E07Rik	RIKEN cDNA 2510009E07 gene	protein_coding	NP_001001881.1	1919440	.	[illegible]
72193	NM_028148,XM_006521430,XM_011245736,XM_030248784	Scaf11	SR-related CTD-associated factor 11	protein_coding	NP_082424.2;XP_006521493.1;XP_011244038.1;XP_030104644.1	1919443	.	[illegible]
72194	NM_028149,XM_006534298,XM_006534299,XM_030246362	Fbxl20	F-box and leucine-rich repeat protein 20	protein_coding	NP_082425.1;XP_006534361.1;XP_006534362.1;XP_030102222.1	1919444	.	[illegible]
72195	NM_028150	Supt7l	SPT7-like, STAGA complex gamma subunit	protein_coding	NP_082426.1	1919445	.	[illegible]
72198	NM_028151	Mtrex	Mtr4 exosome RNA helicase	protein_coding	NP_082427.1	1919448	.	[illegible]
72199	NM_028152,XM_006527358,XM_011247348,XM_011247349,XM_011247350,XM_011247351,XM_011247354,XM_017318292,XM_030251078,XM_030251079,XM_030251080,XM_030251081,XM_030251082,XR_001782617,XR_001782618,XR_001782619	Mms19	MMS19 cytosolic iron-sulfur assembly component	protein_coding	NP_082428.1;XP_006527421.3;XP_011245650.1;XP_011245651.1;XP_011245652.1;XP_011245653.1;XP_011245656.1;XP_017173781.1;XP_030106938.1;XP_030106939.1;XP_030106940.1;XP_030106941.1;XP_030106942.1	1919449	.	[illegible]
72201	NM_152812	Otud6b	OTU domain containing 6B	protein_coding	NP_690025.3	1919451	.	[illegible]
72203	NR_037964	2610507I01Rik	RIKEN cDNA 2610507I01 gene	lncRNA	.	1919453	.	[illegible]
72205	NM_001162996,NM_001347582,NM_028153,XM_011250714,XM_011250715,XM_011250718,XM_011250719,XM_030243009,XM_030243010,XR_001778001	Eml2	echinoderm microtubule associated protein like 2	protein_coding	NP_001156468.1;NP_001334511.1;NP_082429.1;XP_011249016.1;XP_011249017.1;XP_011249020.1;XP_011249021.1;XP_030098869.1;XP_030098870.1	1919455	.	[illegible]

72215	NM_028156	1700001P01Rik	RIKEN cDNA 1700001P01 gene	protein_coding
72219	NM_028157,XM_011244561	Spata31d1a	spermatogenesis associated 31 subfamily D, member 1A	protein_coding
72221	NM_028158	1700021F07Rik	RIKEN cDNA 1700021F07 gene	protein_coding
72225	NR_040647,NR_040648	1700003M07Rik	RIKEN cDNA 1700003M07 gene	lncRNA
72230	NM_028160,NM_028935,XM_011242609,XM_011242610,XM_017313602,XM_017313603,XM_017313604,XM_030244624,XM_030244625,XM_030244626,XM_030244627,XM_030244628,XM_030244629,XM_030244630	Zfp558	zinc finger protein 558	protein_coding
72231	XR_378049	1700018A23Rik	RIKEN cDNA 1700018A23 gene	lncRNA
72236	NM_024445,XM_011248511,XM_011248512	Tsnaxip1	translin-associated factor X (Tsnax) interacting protein 1	protein_coding
72238	NM_001285991,NM_001285993,NM_028162,XM_006524965,XM_006524967,XM_011246640,XM_030250062	Tbc1d5	TBC1 domain family, member 5	protein_coding
72243	NR_045171	1700012D01Rik	RIKEN cDNA 1700012D01 gene	lncRNA
72244	NM_001085385,NM_028166	1600014C10Rik	RIKEN cDNA 1600014C10 gene	protein_coding
72246	XR_001780171	1700030C12Rik	RIKEN cDNA 1700030C12 gene	lncRNA
72249	NR_040255	1700019G24Rik	RIKEN cDNA 1700019G24 gene	lncRNA
72254	NM_028170,XM_006509750,XM_011242328,XR_870463	1700030K09Rik	RIKEN cDNA 1700030K09 gene	protein_coding
72257	NR_045900	1700006H21Rik	RIKEN cDNA 1700006H21 gene	lncRNA
72258	NM_001316664,NM_001316665,NM_001316666,NM_029911,XM_006516257,XM_006516259,XM_011244181,XM_030246941	Kcnk10	potassium channel, subfamily K, member 10	protein_coding
72263	NR_045731	1700030F04Rik	RIKEN cDNA 1700030F04 gene	lncRNA
72265	NM_028173	Tram1	translocating chain-associating membrane protein 1	protein_coding
72267	NM_028175,XM_006508906,XM_006508907,XM_006508908,XM_011242086	Lrrc8e	leucine rich repeat containing 8 family, member E	protein_coding
72269	NM_028176,XM_006539196	Cda	cytidine deaminase	protein_coding
72273	NM_001099917	Smim24	small integral membrane protein 24	protein_coding
72275	NM_028179	2200002D01Rik	RIKEN cDNA 2200002D01 gene	protein_coding
72277	NM_028180,XM_030248785,XM_030248786,XM_030248787	Fam186a	family with sequence similarity 186, member A	protein_coding
72278	NM_001114328,NM_001286544,NM_001286545,NM_001286546,NM_001359295,NM_001359296,NM_028181,XM_006511468,XM_006511469,XR_379460,XR_870768	Ccpg1	cell cycle progression 1	protein_coding
72281	NM_028182,XM_006509751	Sh2d4a	SH2 domain containing 4A	protein_coding
72282	NM_028183	1810062G17Rik	RIKEN cDNA 1810062G17 gene	protein_coding
72284	NM_001347536,NM_001347537,NM_001357868,NM_001357869,NM_001361263,NM_028184	LTO1	ABCE maturation factor	protein_coding
72285	XR_168565,XR_877037,XR_877038	1810073O08Rik	RIKEN cDNA 1810073O08 gene, transcript variant X2	lncRNA
72287	NM_024413	Plekhf1	pleckstrin homology domain containing, family F (with FYVE domain) member 1	protein_coding
72289	NR_002847	Malat1	metastasis associated lung adenocarcinoma transcript 1 (non-coding	lncRNA
72290	NM_028185,XM_011249271	Lsm11	U7 snRNP-specific Sm-like protein LSM11	protein_coding
72293	NM_001347535,NM_028186	Nkd2	naked cuticle 2	protein_coding
72296	NM_001083807,NM_001083808,NM_028188,XM_006502134	Rusc1	RUN and SH3 domain containing 1	protein_coding
72297	NM_028189,XM_006509752,XM_006509753	B3gnt3	UDP-GlcNAc:betaGal beta-1,3-N-acetylglucosaminyltransferase 3	protein_coding
72301	NM_001163145,NM_001347543,XM_006521441,XM_006521442,XM_006521443,XM_006521444,XM_006521445,XM_030248788	Shisal1	shisa like 1	protein_coding
72306	NM_001081382,NM_001347139,XM_006506671,XM_006506673,XM_006506674,XM_006506675,XM_011241478,XR_003956255	Zfp777	zinc finger protein 777	protein_coding
72307	NM_001033164	2510002D24Rik	RIKEN cDNA 2510002D24 gene	protein_coding
72308	NM_028193,XM_006516260,XM_006516261,XM_006516267,XM_011244182,XM_011244185,XM_030246942,XM_030246943,XM_030246944,XM_030246945,XR_001780489,XR_381545,XR_872720	Brf1	BRF1, RNA polymerase III transcription initiation factor 90 kDa subunit	protein_coding
72309	NM_001002267	Tmem158	transmembrane protein 158	protein_coding
72310	NM_024253	Nkg7	natural killer cell group 7 sequence	protein_coding
72313	NM_028194,XM_006504137,XM_006504138,XM_006504139,XM_006504140,XM_006504141,XM_006504142,XM_006504143,XM_006504144,XM_006504145,XM_006504146,XM_006504148,XM_006504149,XM_006504150,XM_006504152,XM_011240773,XM_017321111,XM_030254824,XM_030254825,XR_001784749	Fryl	FRY like transcription coactivator	protein_coding
72315	NM_001166164,XM_006522601,XM_006522602,XM_006522603,XM_006522604	Ccdc74a	coiled-coil domain containing 74A	protein_coding
72316	NR_045904	2310008N11Rik	RIKEN cDNA 2310008N11 gene	lncRNA
72317	NR_040373	Asb17os	ankyrin repeat and SOCS box-containing 17, opposite strand	lncRNA
72318	NM_028195,XM_006521446	Cyth4	cytohesin 4	protein_coding
72320	NM_028197,XM_006514192	Kif1bp	KIF1 binding protein	protein_coding
72322	NM_028198	Xpo5	exportin 5	protein_coding
72323	NM_133346	Asb6	ankyrin repeat and SOCS box-containing 6	protein_coding
72324	NM_001163608,NM_028199,XM_006534301	Plxdc1	plexin domain containing 1	protein_coding
72325	NM_001310661,NM_028200,XM_006531390,XM_030243787,XM_030243788,XR_001778475,XR_003947341,XR_003947342,XR_003947343	Vps9d1	VPS9 domain containing 1	protein_coding
72326	NR_040318	2500004C02Rik	RIKEN cDNA 2500004C02 gene	lncRNA
72330	NM_028202,XM_006512296	Klhl40	kelch-like 40	protein_coding
72333	NM_001081390,NM_001293772,NM_001293773,NM_001293774,XM_006509503,XM_006509504,XM_006509505,XM_006509507,XM_006509508,XM_011242242	Palld	palladin, cytoskeletal associated protein	protein_coding
72338	NM_028203	Wdr89	WD repeat domain 89	protein_coding
72341	NM_001081381,XM_006512297,XR_001779017,XR_003948016	Elp6	elongator acetyltransferase complex subunit 6	protein_coding
72344	NM_001033528,XM_006534302,XM_006534303,XM_006534304,XM_011249273,XR_003949510,XR_388534	Usp36	ubiquitin specific peptidase 36	protein_coding
72345	NM_175179	Amer1	APC membrane recruitment 1	protein_coding
72349	NM_028207,XM_006534305,XM_006534306,XM_030246364	Dusp3	dual specificity phosphatase 3 (vaccinia virus phosphatase VH1-related)	protein_coding
72350	NM_172414,XM_006516269,XM_006516270	Zc2hc1c	zinc finger, C2HC-type containing 1C	protein_coding
72351	NM_028208,XM_006527361	Ptar1	protein prenyltransferase alpha subunit repeat containing 1	protein_coding
72354	NM_001172073,NM_028209,XM_006503408,XM_006503409,XM_006503410,XM_030253808,XM_030253809	Ttc4	tetratricopeptide repeat domain 4	protein_coding
72355	NM_001164625,NM_001164626,NM_197998,XM_006521447,XM_006521448	Cdpf1	cysteine rich, DPF motif domain containing 1	protein_coding
72357	NM_028211,XM_017321112	2210016L21Rik	RIKEN cDNA 2210016L21 gene	protein_coding
72361	NM_197999,XM_006531391,XM_006531392,XR_387780	Ces2g	carboxylesterase 2G	protein_coding
72365	XR_003947976,XR_387342	2210011K15Rik	RIKEN cDNA 2210011K15 gene, transcript variant X1	lncRNA
72368	NM_001145552,NM_001309645,NM_001309841,NR_132131,NR_132132,NR_132133	Borcs8	BLOC-1 related complex subunit 8	protein_coding
72371	NM_001081353,NM_001145676,XM_006517397,XM_017315609	2210408I21Rik	RIKEN cDNA 2210408I21 gene	protein_coding
72383	NM_001081375,NM_028219	Cnfn	cornifelin	protein_coding
72386	NR_015556	2610035D17Rik	RIKEN cDNA 2610035D17 gene	lncRNA
72388	NM_023663,XM_011246154	Ripk4	receptor-interacting serine-threonine kinase 4	protein_coding
72391	NM_001360041,NM_028222,XM_006519593,XM_006519594,XM_030248044	Cdkn3	cyclin-dependent kinase inhibitor 3	protein_coding
72392	NM_001163531,NM_001163532,NM_001359423,NM_001359426,NM_001359427,NM_028223,XM_006535230,XM_006535231,XM_006535232,XM_006535233,XM_006535236,XM_006535237,XM_030254826,XM_030254827,XM_030254828	Tmem175	transmembrane protein 175	protein_coding
72393	NM_001038658,NM_028224,XM_006521449	Faim2	Fas apoptotic inhibitory molecule 2	protein_coding
72395	NR_015560	2610028E06Rik	RIKEN cDNA 2610028E06 gene	lncRNA
72397	NM_028226,XM_006538271,XM_006538272,XM_006538273,XM_006538274,XM_030253810	Rbm12b1	RNA binding motif protein 12 B1	protein_coding
72399	NM_001289543,NM_001289544,NM_028227,XM_030254829	Brap	BRCA1 associated protein	protein_coding
72400	NM_028228,XM_006519595,XM_030248045,XR_003950903,XR_383213	Pinx1	PIN2/TERF1 interacting, telomerase inhibitor 1	protein_coding
72401	NM_001081349,NM_001083809,NM_024497,XM_006500230,XM_006500231,XM_017319273,XM_017319274,XM_017319275,XM_017319276,XM_017319277	Slc43a1	solute carrier family 43, member 1	protein_coding
72404	NM_001305675,NM_175180,XM_006527700,XM_011247497	Wdr44	WD repeat domain 44	protein_coding
72413	NM_028231,XM_006535548,XM_006535549,XM_006535550,XM_006535551,XM_017319742	Kcnmb2	potassium large conductance calcium-activated channel, subfamily M, beta member 2	protein_coding
72415	NM_028232,XM_011246642,XM_017317658	Sgo1	shugoshin 1	protein_coding
72416	NM_028233,XM_011246643	Lrpprc	leucine-rich PPR-motif containing	protein_coding
72421	NM_028235	Ttc30b	tetratricopeptide repeat domain 30B	protein_coding
72425	NM_024254	Katnbl1	katanin p80 subunit B like 1	protein_coding
72429	NM_001033165,XM_006538275,XM_006538276	Dnajc25	DnaJ heat shock protein family (Hsp40) member C25	protein_coding
72432	NM_001081180,XM_006526279,XM_006526280	Spink5	serine peptidase inhibitor, Kazal type 5	protein_coding
72433	NM_028238	Rab38	RAB38, member RAS oncogene family	protein_coding
72434	NM_133743	Lypd3	Ly6/Plaur domain containing 3	protein_coding
72440	NM_001326583,NM_001326585,NM_001326586,NM_001326587,XM_017321762,XM_017321763,XM_017321764	Rhno1	RAD9-HUS1-RAD1 interacting nuclear orphan 1	protein_coding
72446	NM_001083810,NM_001110849,NM_001355625,NM_175181,XM_006500236,XM_006500240,XM_006500241,XM_017319278,XM_017319279	Prr5l	proline rich 5 like	protein_coding
72454	NM_133744,XM_006511836,XM_006511837	Ccdc71	coiled-coil domain containing 71	protein_coding
72459	NM_028242,NM_029371	Htatsf1	HIV TAT specific factor 1	protein_coding
72461	NM_028243	Prcp	prolylcarboxypeptidase (angiotensinase C)	protein_coding
72462	NM_001163734,NM_028244,XM_006524971,XM_006524972,XR_001782117,XR_003952190,XR_003952191	Rrp1b	ribosomal RNA processing 1 homolog B (S. cerevisiae)	protein_coding
72465	NM_001302547,NM_001302548,NM_001302549,NM_001302550,NM_028245,XM_006517766,XM_006517769,XM_011244698,XM_017315610,XM_030247417,XM_030247418,XM_030247419,XM_030247420,XM_030247421	Zfp131	zinc finger protein 131	protein_coding
72469	NM_001361706,NM_152813,XM_006534308,XM_006534309,XM_017314788,XM_017314789	Plcd3	phospholipase C, delta 3	protein_coding
72472	NM_001114332,NM_028247,XM_006512863,XR_380076	Slc16a10	solute carrier family 16 (monocarboxylic acid transporters), member 10	protein_coding
72475	NM_001355532,NM_023672,NM_198438,XM_006503411,XM_006503412,XM_006503413,XM_006503414,XM_006503415,XM_006503416,XM_006503417,XM_006503420	Ssbp3	single-stranded DNA binding protein 3	protein_coding
72477	NM_001355628,NM_001355629,NM_028248,XM_011239790,XM_011239791	Tmem87b	transmembrane protein 87B	protein_coding
72479	NM_001301367,NM_024255,XM_006538277,XM_017320391	Hsdl2	hydroxysteroid dehydrogenase like 2	protein_coding
72480	NM_030203	Tspyl4	TSPY-like 4	protein_coding
72481	NR_015470	2610203C22Rik	RIKEN cDNA 2610203C22 gene	lncRNA
72482	NM_001145781,NM_026683,NM_028250,XM_006529883,XR_3871	Acbd6	acyl-Coenzyme A binding domain containing 6	protein_coding
72484	XR_377738,XR_377739,XR_869150,XR_869151	2610300M13Rik	RIKEN cDNA 2610300M13 gene, transcript variant X2	lncRNA
72486	NM_026047,XM_011245206	Rnf219	ring finger protein 219	protein_coding
72495	NR_038175	2610206C17Rik	RIKEN cDNA 2610206C17 gene	lncRNA
72500	NM_030244	Ier5l	immediate early response 5-like	protein_coding
72502	NM_001081077,NM_001356578,XM_006527364,XM_011247357,XM_011247358,XM_017318296,XM_017318297,XM_030251083,XM_030251084,XM_030251085,XM_030251086,XM_030251087	Cwf19l1	CWF19-like 1, cell cycle control (S. pombe)	protein_coding
72503	NM_001002004,XM_006534312,XM_006534313,XM_030246365,XR_001780057,XR_001780058	2610507B11Rik	RIKEN cDNA 2610507B11 gene	protein_coding
72504	NM_001100449,NM_001369357,NM_001369358,XR_877141	Taf4b	TATA-box binding protein associated factor 4b	protein_coding
72507	NM_001311142,NM_028258,XM_006511472,XM_006511473,XM_006511474,XM_006511475,XM_006511476,XM_030244631,XM_030244632,XM_030244633,XR_001779020,XR_003948017,XR_87077	Dzip1l	DAZ interacting protein 1-like	protein_coding
72508	NM_001114334,NM_001363162,NM_028259,XM_006534315,XM_006534317,XM_011249275,XM_011249276,XM_017314791,XM_017314792,XM_030246366,XR_001780060,XR_003949511,XR_003949512,XR_003949513,XR_879743	Rps6kb1	ribosomal protein S6 kinase, polypeptide 1	protein_coding
72511	NR_045172	2610316D01Rik	RIKEN cDNA 2610316D01 gene	lncRNA
72512	NM_001289591,NM_001289592,NM_028261,XM_017317994	Tmem173	transmembrane protein 173	protein_coding
72514	NM_028263	Fgfbp3	fibroblast growth factor binding protein 3	protein_coding
72515	NM_175639,XR_385368	Wdr43	WD repeat domain 43	protein_coding
72518	NR_045053,NR_045054	2610307P16Rik	RIKEN cDNA 2610307P16 gene	lncRNA
72519	NM_028264	Pip4p2	phosphatidylinositol-4,5-bisphosphate 4-phosphatase 2	protein_coding
72522	NM_001289545,NM_175183,XM_006502136,XM_006502137	Atxn7l2	ataxin 7-like 2	protein_coding
72535	NM_028270	Aldh1b1	aldehyde dehydrogenase 1 family, member B1	protein_coding
72536	NM_145968,XM_006523354	Tagap	T cell activation Rho GTPase activating protein	protein_coding
72542	NM_001163538,NM_028273	Pgam5	phosphoglycerate mutase family member 5	protein_coding
72543	NM_175184,XM_006498367,XM_006498368,XM_006498369,XM_006498370,XM_006498371,XM_006498372,XM_006498373,XM_011239194,XM_017319281,XM_030252112,XM_030252113,XM_030252114,XM_030252115,XM_030252116	Mvb12b	multivesicular body subunit 12B	protein_coding
72544	NM_028274	Exosc6	exosome component 6	protein_coding
72549	NM_180588,XM_006519598	Reep4	receptor accessory protein 4	protein_coding
72552	NM_175185,XM_006531393,XM_006531394,XM_006531395,XM_006531397,XM_006531398,XM_030243789,XR_001778478,XR_003947344,XR_003947345	Hsdl1	hydroxysteroid dehydrogenase like 1	protein_coding
72554	NM_028276,XM_017318627	Utp14a	UTP14A small subunit processome component	protein_coding
72555	NM_001174086,NM_028277,XM_017317137,XM_017317138,XM_030249286	Shisa9	shisa family member 9	protein_coding
72556	NM_152814	Zfp566	zinc finger protein 566	protein_coding
72560	NM_001357399,NM_001357400,NM_028279	Naalad2	N-acetylated alpha-linked acidic dipeptidase 2	protein_coding
72562	NM_028281,XM_006517398,XM_006517399,XM_006517401,XM_011244564,XM_030247422	Pcbd2	pterin 4 alpha carbinolamine dehydratase/dimerization cofactor of hepatocyte nuclear factor 1 alpha (TCF1) 2	protein_coding
72565	NM_001357407,NM_001357408,NM_028283,XM_006511482,XM_006511483,XM_017313609,XM_030244634	Uaca	uveal autoantigen with coiled-coil domains and ankyrin repeats	protein_coding
72567	NM_001025392,NM_001025393,NM_153787,XM_006512865,XM_011243207	Bclaf1	BCL2-associated transcription factor 1	protein_coding
72568	NM_001103182,NR_120498,XM_006496987,XM_006496988	Lin9	lin-9 homolog (C. elegans)	protein_coding
72569	NM_001362706,NM_028284,XM_006500244	Bbs5	Bardet-Biedl syndrome 5 (human)	protein_coding
72572	NM_139140,XM_006521450,XM_011245737	Spats2	spermatogenesis associated, serine-rich 2	protein_coding
72575	NR_038184,NR_038185	C430049B03Rik	RIKEN cDNA C430049B03 gene	lncRNA
72578	NR_045436,NR_045437	2700054A10Rik	RIKEN cDNA 2700054A10 gene	lncRNA
72580	NM_001359455,NM_001359456,NM_028287,NR_153113,XM_006512868,XM_006512869,XM_006512870,XM_030245303	Zup1	zinc finger containing ubiquitin peptidase 1	protein_coding
72584	NM_001110142,NM_028288	Cul4b	cullin 4B	protein_coding
72585	NM_001311089,NM_001311090,NM_145100,XM_006529885,XM_011248088,XM_017312443	Lypd1	Ly6/Plaur domain containing 1	protein_coding
72587	NM_001359433,NM_028291,XM_006504868,XM_006504869,XM_006504870,XM_006504871,XM_006504872,XM_006504873,XM_006504877,XM_006504878,XM_011241014,XM_017321113,XM_030254830,XM_030254831	Pan3	PAN3 poly(A) specific ribonuclease subunit	protein_coding
72588	NR_152107	2700012I20Rik	RIKEN cDNA 2700012I20 gene	lncRNA
72590	NM_028292	Ppme1	protein phosphatase methylesterase 1	protein_coding
72599	NM_028295,XR_384603,XR_384604,XR_384605	Pdia5	protein disulfide isomerase associated 5	protein_coding
72605	NM_001361707,NM_001361708,NM_028296,XM_030246367,XM_030246368	Car10	carbonic anhydrase 10	protein_coding
72607	NM_001013024,XM_006535553,XM_006535555,XM_030252841,XR_003954400,XR_003954401	Usp13	ubiquitin specific peptidase 13 (isopeptidase T-3)	protein_coding
72608	NR_045905	2700069I18Rik	RIKEN cDNA 2700069I18 gene	lncRNA
72609	NR_046019	2700070H01Rik	RIKEN cDNA 2700070H01 gene	lncRNA
72611	NM_001083958,NM_001347533,NM_001363449,NM_001363451,NM_001363452,NM_028298,XM_017321114,XM_030254832	Zfp655	zinc finger protein 655	protein_coding
72612	NM_001357270,NM_001357271,NM_028299,NR_151662,XM_006509510,XM_006509511,XM_006509512,XM_030243790,XM_030243791,XM_030243792,XR_003947346,XR_378914	Hpf1	histone PARylation factor 1	protein_coding
72614	NM_028300,XM_006510627	Pih1d2	PIH1 domain containing 2	protein_coding
72615	NM_028301,XM_006522608,XM_006522609,XM_006522610,XM_006522611,XM_006522612,XM_006522613,XM_030249287,XM_030249288,XM_030249289,XM_030249290,XM_030249291,XM_030249292,XM_030249294,XR_003951838,XR_003951839,XR_003951840,XR_003951841	Anks3	ankyrin repeat and sterile alpha motif domain containing 3	protein_coding
72621	NM_001356384,NM_028303,XM_011247685	Pdzd11	PDZ domain containing 11	protein_coding
72630	NM_028306,XM_006500245,XM_011239793	Hspa12b	heat shock protein 12B	protein_coding
72632	NM_001206849,XM_006509201	Smim18	small integral membrane protein 18	protein_coding
72634	NM_001357711,NM_001357712,NM_001357713,NM_001357714,NM_028307,XM_017319744,XM_017319745,XM_017319748,XM_030252842,XM_030252843,XR_003954402,XR_003954403,XR_0039	Tdrkh	tudor and KH domain containing protein	protein_coding
72635	NM_001191001,NM_152815,NR_034030,XM_006541217,XM_006541218,XM_006541219,XM_006541220,XM_006541221,XM_011250905,XM_017312329,XM_030243012,XM_030243013,XM_03024301	Lins1	lines homolog 1	protein_coding
72640	NM_001029890	Mex3a	mex3 RNA binding family member A	protein_coding
72648	NR_045175	Smc2os	structural maintenance of chromosomes 2, opposite strand	lncRNA
72649	NM_001311093,NM_001347087,NM_178625,XM_006505170,XM_006505171,XM_006505172,XM_017321766	Tmem209	transmembrane protein 209	protein_coding
72650	NM_001134717,NM_028310	2810006K23Rik	RIKEN cDNA 2810006K23 gene	protein_coding
72654	NM_028312,XM_030244635,XM_030244636	Ccdc12	coiled-coil domain containing 12	protein_coding
72655	NR_040721	Snhg5	small nucleolar RNA host gene 5	lncRNA
72656	NM_001159595,NM_178112,XM_017320392,XR_390345	Ints8	integrator complex subunit 8	protein_coding
72657	NM_001033166,NM_001037279	Selenoh	selenoprotein H	protein_coding
72658	NM_028314,NR_030764,NR_155260,XM_006516271,XM_0112441	2700097O09Rik	RIKEN cDNA 2700097O09 gene	protein_coding
72661	NM_001160326,NM_001360046,NM_001360047,NM_001360048,NR_027699,NR_153389,XM_030248046	Serp2	stress-associated endoplasmic reticulum protein family member 2	protein_coding
72662	NM_001360050,NM_001360051,NM_028315,NR_153390,XM_006519602,XM_017316212,XM_030248047	Dis3	DIS3 homolog, exosome endoribonuclease and 3'-5' exoribonuclease	protein_coding

72665	XR_003951643	2810039B14Rik	RIKEN cDNA 2810039B14 gene	misc_RNA	.	1919915	.	0.408848	[illegible]
72667	NM_001146024,NM_028316,XM_006540380,XM_006540381,XM_006540382,XM_006540383	Zfp444	zinc finger protein 444	protein_coding	NP_001139496.1;NP_082592.2;XP_006540443.1;XP_006540444.1;XP_006540445.1;XP_006540446.1	1923365	.	2.503885	[illegible]
72668	NM_028317	Skida1	SKI/DACH domain containing 1	protein_coding	NP_082593.2	1919918	.	1.357372	[illegible]
72669	NR_015579	2810032G03Rik	RIKEN cDNA 2810032G03 gene	lncRNA	.	1919919	.	0.643039	[illegible]
72672	NM_028319,XM_006527368,XM_011247359,XM_011247360,XM_011247361,XM_011247363,XM_011247364,XM_030251088	Zfp518a	zinc finger protein 518A	protein_coding	NP_082595.1;XP_006527431.1;XP_011245661.1;XP_011245662.1;XP_011245663.1;XP_011245665.1;XP_011245666.1;XP_030106948.1	1919922	.	3.492592	[illegible]
72674	NM_001306069,NM_028320	Adipor1	adiponectin receptor 1	protein_coding	NP_001292998.1;NP_082596.2	1919924	.	36.265547	[illegible]
72677	NR_036594	2810049E08Rik	RIKEN cDNA 2810049E08 gene	lncRNA	.	1919927	.	0.000000	[illegible]
72685	NM_001164583,NM_001164584,NM_001164585,NM_001355179,NM_001355180,NM_198412	Dnajc6	DnaJ heat shock protein family (Hsp40) member C6	protein_coding	NP_001158055.1;NP_001158056.1;NP_001158057.1;NP_001342108.1;NP_001342109.1;NP_940804.1	1919935	.	0.136060	[illegible]
72690	NM_001099296,XM_006539197,XM_006539198,XM_006539199,XM_006539200	Grrp1	glycine/arginine rich protein 1	protein_coding	NP_001092766.1;XP_006539260.1;XP_006539261.1;XP_006539262.1;XP_006539263.1	1919940	.	7.515127	[illegible]
72691	NM_133746	Calhm2	calcium homeostasis modulator family member 2	protein_coding	NP_598507.3	1919941	.	5.384648	[illegible]
72692	NM_144802,NR_152119	Hnrnpll	heterogeneous nuclear ribonucleoprotein L-like	protein_coding	NP_659051.3	1919942	.	19.316553	[illegible]
72693	NM_001358476,NM_001358477,NM_001358478,NM_001358479,NM_001358480,NM_028325,XM_006541399,XM_011250974,XM_017318629,XM_017318630	Zcchc12	zinc finger, CCHC domain containing 12	protein_coding	NP_001345405.1;NP_001345406.1;NP_001345407.1;NP_001345408.1;NP_001345409.1;NP_082601.1;XP_006541462.1;XP_011249276.1;XP_017174118.1;XP_017174119.1	1919943	.	0.454577	[illegible]
72699	NM_023684	Lime1	Lck interacting transmembrane adaptor 1	protein_coding	NP_076173.1	1919949	.	13.292930	[illegible]
72701	NM_001369113,NM_001369114,NM_001369115,NM_001369116,NM_001369117,NM_001369118,NM_001369119,NM_028326,NM_028327,XM_006538279,XM_006538280,XM_006538281,XM_006538282,XM_006538283,XM_006538284,XM_006538286,XM_006538287,XM_006538288,XM_006538289,XM_006538291,XM_017320394,XM_017320395,XM_030253812,XM_030253813,XM_030253814,XM_030253815,XM_030253816,XM_030253817,XM_030253818,XR_003955017	Zfp618	zinc finger protein 618	protein_coding	NP_001356042.1;NP_001356043.1;NP_001356044.1;NP_001356045.1;NP_001356046.1;NP_001356047.1;NP_001356048.1;NP_082602.1;NP_082603.1;XP_006538342.2;XP_006538343.2;XP_006538344.2;XP_006538345.2;XP_006538346.2;XP_006538347.2;XP_006538349.1;XP_006538350.1;XP_006538351.1;XP_006538352.1;XP_006538354.2;XP_017175883.1;XP_017175884.1;XP_030109672.1;XP_030109673.1;XP_030109674.1;XP_030109675.1;XP_030109676.1;XP_030109677.1;XP_030109678.1	1919950	.	5.551732	[illegible]
72709	NM_001204152,NM_001204153,NM_028331,XM_006521452,XM_006521453,XM_006521454,XM_006521455,XM_030248791	C1qtnf6	C1q and tumor necrosis factor related protein 6	protein_coding	NP_001191081.1;NP_001191082.1;NP_082607.3;XP_006521515.1;XP_006521516.1;XP_006521517.1;XP_006521518.1;XP_030104651.1	1919959	.	81.780659	[illegible]
72713	NM_028333	Angptl1	angiopoietin-like 1	protein_coding	NP_082609.2	1919963	.	13.603498	[illegible]
72716	NR_015598,NR_154463	2810047C21Rik1	RIKEN cDNA 2810047C21 gene 1	lncRNA	.	1919966	.	0.103131	[illegible]
72720	NM_001361899,NM_001361900,NM_001361901,NM_028335,XM_006506676,XM_030255603,XM_030255604	Zfp248	zinc finger protein 248	protein_coding	NP_001348828.1;NP_001348829.1;NP_001348830.1;NP_082611.1;XP_006506739.1;XP_030111463.1;XP_030111464.1	1919970	.	2.744145	[illegible]
72722	NM_001357860,NM_133747	Fam98a	family with sequence similarity 98, member A	protein_coding	NP_001344789.1;NP_598508.2	1919972	.	23.023680	[illegible]
72723	NM_178384,XM_006540385,XM_011250720,XM_030243015	Zfp74	zinc finger protein 74	protein_coding	NP_848471.2;XP_006540448.1;XP_011249022.1;XP_030098875.1	107784	.	3.124615	[illegible]
72726	NM_178385	Tbcc	tubulin-specific chaperone C	protein_coding	NP_848472.2	1919976	.	4.698752	[illegible]
72727	NM_024256,XR_003952823	B3gat3	beta-1,3-glucuronyltransferase 3 (glucuronosyltransferase I)	protein_coding	NP_077218.1	1919977	.	24.686300	[illegible]
72729	NM_178626,XM_006534320,XM_030246369,XM_030246370	Cdc42se2	CDC42 small effector 2	protein_coding	NP_848741.1;XP_006534383.1;XP_030102229.1;XP_030102230.1	1919979	.	13.493379	[illegible]
72735	NR_110421,NR_110422,NR_110423,NR_110424,NR_110425	Eldr	Egfr long non-coding downstream RNA	lncRNA		1919985	.	1.159278	[illegible]
72736	NM_028339	Tmx1	thioredoxin-related transmembrane protein 1	protein_coding	NP_082615.1	1919986	.	42.412823	[illegible]
72739	NM_001145778,NM_001368182,NM_023685,XM_006516789,XM_006516790,XM_006516792	Zkscan3	zinc finger with KRAB and SCAN domains 3	protein_coding	NP_001139250.1;NP_001355111.1;NP_076174.3;XP_006516852.1;XP_006516853.1;XP_006516855.1	1919989	.	19.925052	[illegible]
72745	NM_001361908,NM_001361909,NM_001361911,NM_175187,NR_029419,NR_155262,XM_006517402,XM_006517404,XM_006517405,XM_011244565,XM_030247423	Tmem161b	transmembrane protein 161B	protein_coding	NP_001348837.1;NP_001348838.1;NP_001348840.1;NP_780396.2;XP_006517465.1;XP_006517467.1;XP_006517468.1;XP_011242867.1;XP_030103283.1	1919995	.	4.518655	[illegible]
72747	NM_028341,XM_030250613,XM_030250614,XM_030250615,XM_030250616	Ttc39c	tetratricopeptide repeat domain 39C	protein_coding	NP_082617.2;XP_030106473.1;XP_030106474.1;XP_030106475.1;XP_030106476.1	1919997	.	3.227690	[illegible]
72748	NM_024257	Hdhd3	haloacid dehalogenase-like hydrolase domain containing 3	protein_coding	NP_077219.1	1919998	.	2.809536	[illegible]
72749	NM_183091,XM_017316755	Tonsl	tonsoku-like, DNA repair protein	protein_coding	NP_898914.3;XP_017172244.1	1919999	.	2.523499	[illegible]
72750	NM_001037725	Fam117b	family with sequence similarity 117, member B	protein_coding	NP_001032814.1	1920000	.	6.958606	[illegible]
72753	NR_040562	2810442N19Rik	RIKEN cDNA 2810442N19 gene	lncRNA	.	1920003	.	0.264902	[illegible]
72754	NM_001112722,NM_001112723,NM_001290803,NM_172415,XM_006539201,XM_006539202,XM_006539204,XM_011250337,XM_011250340,XM_017320396,XM_017320397,XM_030253820,XM_030253821,XM_030253822,XM_030253823,XR_001784205,XR_003955018,XR_003955019,XR_003955020,XR_003955021	Arhgef10l	Rho guanine nucleotide exchange factor (GEF) 10-like	protein_coding	NP_001106193.1;NP_001106194.1;NP_001277732.1;NP_766003.3;XP_006539264.1;XP_006539265.1;XP_006539267.1;XP_011248639.1;XP_011248642.1;XP_017175885.1;XP_017175886.1;XP_030109680.1;XP_030109681.1;XP_030109682.1;XP_030109683.1	1920004	.	7.020444	[illegible]
72759	NM_001360745,NM_001360746,NM_028343,XM_011241911,XM_030243016,XM_030243017,XM_030243018	Tmem135	transmembrane protein 135	protein_coding	NP_001347674.1;NP_001347675.1;NP_082619.3;XP_011240213.1;XP_030098876.1;XP_030098877.1;XP_030098878.1	1920009	.	6.033664	[illegible]
72772	NM_001310467,NM_177323,XM_030254833,XR_001784750	Rint1	RAD50 interactor 1	protein_coding	NP_001297396.1;NP_796297.2;XP_030110693.1	1916233	.	10.005311	[illegible]
72774	NM_001357409,NM_028347,NR_151685,XM_006511485,XM_006511486,XM_006511487,XR_003948019,XR_003948020,XR_379461	Neil1	nei endonuclease VIII-like 1 (E. coli)	protein_coding	NP_001344338.1;NP_082623.1;XP_006511548.1;XP_006511549.1;XP_006511550.1	1920024	.	3.184636	[illegible]
72775	NM_001163819,NM_001163820,NR_028296,NR_028297	Fance	Fanconi anemia, complementation group E	protein_coding	NP_001157291.1;NP_001157292.1	1920025	.	8.711987	[illegible]
72776	NM_001289568,NM_001289571,NM_028349,XM_006502140,XM_006502141	Sass6	SAS-6 centriolar assembly protein	protein_coding	NP_001276497.1;NP_001276500.1;NP_082625.2;XP_006502203.1;XP_006502204.1	1920026	.	1.942051	[illegible]
72778	NM_176835,XM_006521457,XM_006521459,XM_011245738	Dnajc22	DnaJ heat shock protein family (Hsp40) member C22	protein_coding	NP_789805.1;XP_006521520.1;XP_006521522.1;XP_011244040.1	1920028	.	0.652189	[illegible]
72780	NM_028351	Rspo3	R-spondin 3	protein_coding	NP_082627.3	1920030	.	8.029358	[illegible]
72787	NM_028355,XM_006503423	Ndc1	NDC1 transmembrane nucleoporin	protein_coding	NP_082631.1;XP_006503486.1	1920037	.	7.854504	[illegible]
72789	NM_145820,XM_006502142,XM_006502143,XM_011240244	Veph1	ventricular zone expressed PH domain-containing 1	protein_coding	NP_665819.2;XP_006502205.1;XP_006502206.1;XP_011238546.1	1920039	.	0.282174	[illegible]
72792	NM_001144992,NM_001144993	2810459M11Rik	RIKEN cDNA 2810459M11 gene	protein_coding	NP_001138464.1;NP_001138465.1	1920042	.	0.807792	[illegible]
72795	NM_028360,NM_029704,XM_006534321,XR_001780061,XR_001780062,XR_001780063,XR_003949514,XR_003949515,XR_388537	Ttc19	tetratricopeptide repeat domain 19	protein_coding	NP_082636.3;NP_083980.2;XP_006534384.1	1920045	.	7.419480	[illegible]
72805	NM_001199785,NM_028365,XM_006516272,XR_001780491	Zfp839	zinc finger protein 839	protein_coding	NP_001186714.1;NP_082641.1;XP_006516335.1	1920055	.	3.654912	[illegible]
72807	NM_001080941,XM_030247424,XM_030247425,XM_030247426,XM_030247427,XM_030247428,XM_030247429,XM_030247430,XM_030247431	Zfp429	zinc finger protein 429	protein_coding	NP_001074410.1;XP_030103284.1;XP_030103285.1;XP_030103286.1;XP_030103287.1;XP_030103288.1;XP_030103289.1;XP_030103290.1;XP_030103291.1	1920057	.	2.381948	[illegible]
72813	NR_029441	2810454H06Rik	RIKEN cDNA 2810454H06 gene	lncRNA	.	1920063	.	0.428023	[illegible]
72821	NM_001014761,XM_006510629	Scn2b	sodium channel, voltage-gated, type II, beta	protein_coding	NP_001014761.1;XP_006510692.1	106921	.	0.644608	[illegible]
72823	NM_001081050,XM_006496298,XM_006496299,XM_011238602	Pard3b	par-3 family cell polarity regulator beta	protein_coding	NP_001074519.2;XP_006496361.1;XP_006496362.1;XP_011236904.	1919301	.	3.009041	[illegible]
72825	NM_028369,XM_017313610,XM_017313611,XR_001779021	Mon1a	MON1 homolog A, secretory traffciking associated	protein_coding	NP_082645.1;XP_017169099.1;XP_017169100.1	1920075	.	4.374160	[illegible]
72826	NM_001358259,NM_176836,XM_011242612,XM_030244637,XR_001779022,XR_003948021,XR_003948022	Fam76b	family with sequence similarity 76, member B	protein_coding	NP_001345188.1;NP_789806.1;XP_011240914.1;XP_030100497.1	1920076	.	12.776020	[illegible]
72828	NM_001357412,NM_176860,XM_006510632,XM_006510634,XM_006510636,XM_017313612,XM_030244638	Ubash3b	ubiquitin associated and SH3 domain containing, B	protein_coding	NP_001344341.1;NP_789830.1;XP_006510695.1;XP_006510697.1;XP_006510699.1;XP_017169101.1;XP_030100498.1	1920078	.	2.873066	[illegible]
72831	NM_001252682,NM_001252683,NM_133347,XM_006512298,XM_006512299,XM_006512300,XM_006512301,XM_006512303,XM_006512304,XM_006512305,XM_006512310,XM_011243029,XM_017313614,XM_017313615,XM_017313616,XM_030244640,XM_030244641,XM_030244642,XM_030244643,XM_030244644,XM_030244645,XM_030244646,XM_030244647	Dhx30	DEAH (Asp-Glu-Ala-His) box polypeptide 30	protein_coding	NP_001239611.1;NP_001239612.1;NP_579925.1;XP_006512361.1;XP_006512362.1;XP_006512363.1;XP_006512364.1;XP_006512366.1;XP_006512367.1;XP_006512368.1;XP_006512373.1;XP_011241331.1;XP_017169103.1;XP_017169104.1;XP_017169105.1;XP_030100500.1;XP_030100501.1;XP_030100502.1;XP_030100503.1;XP_030100504.1;XP_030100505.1;XP_030100506.1;XP_030100507.1	1920081	.	14.047623	[illegible]
72832	NM_001356588,NM_001356589,NM_145123,XM_006527369,XM_006527371,XM_006527372,XM_030251089,XM_030251090,XM_030251091,XM_030251092,XM_030251093,XM_030251094	Crtac1	cartilage acidic protein 1	protein_coding	NP_001343517.1;NP_001343518.1;NP_660105.3;XP_006527432.2;XP_006527434.2;XP_006527435.1;XP_030106949.1;XP_030106950.1;XP_030106951.1;XP_030106952.1;XP_030106953.1;XP_030106954.	1920082	.	0.867805	[illegible]
72834	NR_045176	2810468N07Rik	RIKEN cDNA 2810468N07 gene	lncRNA	.	1920084	.	1.088739	[illegible]
72836	NM_028370,NR_151542,XM_006524978,XM_006524979,XM_006524980,XM_011246645,XM_011246646,XM_017317660,XM_030250064,XR_003952192,XR_003952193,XR_003952194,XR_876552	Pot1b	protection of telomeres 1B	protein_coding	NP_082646.1;XP_006525041.1;XP_006525042.1;XP_006525043.1;XP_011244947.1;XP_011244948.1;XP_017173149.1;XP_030105924.1	1920086	.	4.328843	[illegible]
72843	NM_001302886,NM_001302887,NM_181650,XM_006514193,XM_006514194,XM_006514197,XM_011243582,XM_011243583,XM_017314116,XM_030245304	Prdm4	PR domain containing 4	protein_coding	NP_001289815.1;NP_001289816.1;NP_857633.2;XP_006514256.1;XP_006514257.1;XP_006514260.1;XP_011241884.1;XP_011241885.1;XP_017169605.1;XP_030101164.1	1920093	.	8.515184	[illegible]
72844	NM_001289671,NM_001289672,NM_001289673,NR_110357,XM_006521460,XM_006521461,XM_030248792,XR_001781568,XR_001781569	Kctd17	potassium channel tetramerisation domain containing 17	protein_coding	NP_001276600.1;NP_001276601.1;NP_001276602.1;XP_006521523.2;XP_006521524.2;XP_030104652.1	1920094	.	29.507510	[illegible]
72852	NM_028372	Mblac2	metallo-beta-lactamase domain containing 2	protein_coding	NP_082648.1	1920102	.	1.157486	[illegible]
72865	NM_028375	Rtl8c	retrotransposon Gag like 8C	protein_coding	NP_082651.1	1920115	.	16.394836	[illegible]
72873	NM_028377,XM_030246946,XM_030246947,XR_001780492,XR_001780493,XR_003950087,XR_381548,XR_381550,XR_381551	Bbof1	basal body orientation factor 1	protein_coding	NP_082653.2;XP_030102806.1;XP_030102807.1	1920123	.	0.516108	[illegible]
72881	NM_001359445,NM_028379,NR_153112,XM_006504733,XM_006504734,XM_006504735,XM_006504736,XM_030254834,XM_030254835,XM_030254836,XM_030254837,XM_030254838,XR_003955712,XR_003955713,XR_003955714	Zdhhc4	zinc finger, DHHC domain containing 4	protein_coding	NP_001346374.1;NP_082655.1;XP_006504796.1;XP_006504797.1;XP_006504798.1;XP_006504799.1;XP_030110694.1;XP_030110695.1;XP_030110696.1;XP_030110697.1;XP_030110698.1	1920131	.	18.293208	[illegible]
72886	NM_028381	Yju2	YJU2 splicing factor	protein_coding	NP_082657.1	1920136	.	5.866078	[illegible]
72891	NM_183094,XM_030251506,XM_030251507	Xlr4c	X-linked lymphocyte-regulated 4C	protein_coding	NP_898917.2;XP_030107366.1;XP_030107367.1	3574099	.	0.075981	[illegible]
72893	NR_152108	2900040C04Rik	RIKEN cDNA 2900040C04 gene	lncRNA	.	1920143	.	0.000000	[illegible]
72895	NM_028385,NM_173005,XM_006506677,XM_011241480,XM_011241482,XM_011241484,XM_011241485,XM_017321767,XM_017321768,XM_017321769,XM_017321770,XM_017321771,XM_017321772,XM_030255605,XM_030255606,XM_030255607,XM_030255608,XM_030255609,XM_030255610,XM_030255611,XM_030255612,XM_030255613,XM_030255614,XM_030255615,XM_030255616,XM_030255617,XM_030255618,XM_030255619,XM_030255620,XM_030255621,XM_030255622,XM_030255623,XM_030255624,XM_030255625	Setd5	SET domain containing 5	protein_coding	NP_082661.1;NP_766593.1;XP_006506740.1;XP_011239782.1;XP_011239784.1;XP_011239786.1;XP_011239787.1;XP_017177256.1;XP_017177257.1;XP_017177258.1;XP_017177259.1;XP_017177260.1;XP_017177261.1;XP_030111465.1;XP_030111466.1;XP_030111467.1;XP_030111468.1;XP_030111469.1;XP_030111470.1;XP_030111471.1;XP_030111472.1;XP_030111473.1;XP_030111474.1;XP_030111475.1;XP_030111476.1;XP_030111477.1;XP_030111478.1;XP_030111479.1;XP_030111480.1;XP_030111481.1;XP_030111482.1;XP_030111483.1;XP_030111484.1;XP_030111485.1	1920145	.	26.492642	[illegible]
72898	NM_001357011,NM_001357012,NM_001357013,NM_001357014,NM_001357015,NM_028386,XM_011249569,XM_011249570,XM_011249572,XM_017321118,XM_030254839,XM_030254840,XM_030254841,XM_030254842	Asphd2	aspartate beta-hydroxylase domain containing 2	protein_coding	NP_001343940.1;NP_001343941.1;NP_001343942.1;NP_001343943.1;NP_001343944.1;NP_082662.1;XP_011247871.1;XP_011247872.1;XP_011247874.1;XP_017176607.1;XP_030110699.1;XP_030110700.1;XP_030110701.1;XP_030110702.1	1920148	.	4.975427	[illegible]
72899	NM_001013802,NM_028387,XM_006500246,XM_006500247,XM_006500248,XM_006500249,XM_006500250,XM_006500251,XM_006500252,XM_006500253,XM_011239794,XM_017319283,XM_017319285	Macrod2	MACRO domain containing 2	protein_coding	NP_001013824.2;NP_082663.1;XP_006500309.1;XP_006500310.1;XP_006500311.1;XP_006500312.1;XP_006500313.1;XP_006500314.1;XP_006500315.1;XP_006500316.1;XP_011238096.1;XP_017174772.1;XP_017174774.1	1920149	.	1.686946	[illegible]
72900	NM_001278415,NM_028388	Ndufv2	NADH:ubiquinone oxidoreductase core subunit V2	protein_coding	NP_001265344.1;NP_082664.1	1920150	.	34.616979	[illegible]
72902	NM_001252620,NM_001252621,NM_001361918,NM_023689,XM_006509516,XM_006509517	Spock3	sparc/osteonectin, cwcv and kazal-like domains proteoglycan 3	protein_coding	NP_001239549.1;NP_001239550.1;NP_001348847.1;NP_076178.1;XP_006509579.1;XP_006509580.1	1920152	.	1.080451	[illegible]
72925	NM_001166372,NM_001166375,NM_001293784,NM_001363123,NM_001363124,NM_001363125,NM_175188,XM_006509754,XM_006509755,XM_011242329,XM_017312977,XM_017312978,XM_030243793,XM_030243794	March1	membrane-associated ring finger (C3HC4) 1	protein_coding	NP_001159844.1;NP_001159847.1;NP_001280713.1;NP_001350052.1;NP_001350053.1;NP_001350054.1;NP_780397.2;XP_006509817.1;XP_006509818.1;XP_011240631.1;XP_017168466.1;XP_017168467.1;XP_030099653.1;XP_030099654.1	1920175	.	0.421074	[illegible]
72927	NM_175189,XM_006510638	Hepacam	hepatocyte cell adhesion molecule	protein_coding	NP_780398.2;XP_006510701.1	1920177	.	0.000000	[illegible]
72930	NM_028392,NR_073583	Ppp2r2b	protein phosphatase 2, regulatory subunit B, beta	protein_coding	NP_082668.1	1920180	.	1.106087	[illegible]
72931	NM_001290552,NM_175190,NR_110965,XR_374118	Swi5	SWI5 recombination repair homolog (yeast)	protein_coding	NP_001277481.1;NP_780399.1	1920181	.	121.614473	[illegible]
72935	NM_134059,XM_030247432,XR_003950486	Ddx41	DEAD (Asp-Glu-Ala-Asp) box polypeptide 41	protein_coding	NP_598820.2;XP_030103292.1	1920185	.	17.024064	[illegible]
72938	NM_001355459,NM_001355460,NM_028394,XM_006503424,XM_017320398,XM_030253825	Hspb11	heat shock protein family B (small), member 11	protein_coding	NP_001342388.1;NP_001342389.1;NP_082670.1;XP_006503487.1;XP_017175887.1;XP_030109685.1	1920188	.	15.056347	[illegible]
72946	NM_201226	Lrrc47	leucine rich repeat containing 47	protein_coding	NP_957678.1	1920196	.	9.677774	[illegible]
72947	NM_028398,XM_006534323,XM_006534324,XM_006534325	Phykpl	5-phosphohydroxy-L-lysine phospholyase	protein_coding	NP_082674.1;XP_006534386.1;XP_006534387.1;XP_006534388.1	1920197	.	8.289044	[illegible]
72948	NM_182839,XM_006517409	Tppp	tubulin polymerization promoting protein	protein_coding	NP_878259.1;XP_006517472.1	1920198	.	1.793494	[illegible]
72949	NM_028399	Ccnt2	cyclin T2	protein_coding	NP_082675.1	1920199	.	15.712051	[illegible]
72958	NM_028402	Zfp493	zinc finger protein 493	protein_coding	NP_082678.2	1920208	.	0.743066	[illegible]
72960	NM_001355589,NM_028404	Top1mt	DNA topoisomerase 1, mitochondrial	protein_coding	NP_001342518.1;NP_082680.1	1920210	.	3.439887	[illegible]
72961	NM_182993	Slc17a7	solute carrier family 17 (sodium-dependent inorganic phosphate cotransporter), member 7	protein_coding	NP_892038.2	1920211	.	0.059035	[illegible]
72962	NM_138302	Tymp	thymidine phosphorylase	protein_coding	NP_612175.1	1920212	.	0.244535	[illegible]
72972	NM_001347502,NM_001347503,NM_027045,NM_028407,XM_006519605,XM_006519606,XM_006519607,XM_011245208,XM_030248048,XM_030248049	Ccser2	coiled-coil serine rich 2	protein_coding	NP_001334431.1;NP_001334432.1;NP_081321.1;NP_082683.2;XP_006519668.1;XP_006519669.1;XP_006519670.1;XP_011243510.1;XP_030103908.1;XP_030103909.1	101859	.	14.798703	[illegible]
72973	NM_001081435,XM_006534328,XM_006534329,XM_006534330,XM_006534332,XM_011249279	Fbxo47	F-box protein 47	protein_coding	NP_001074904.1;XP_006534391.1;XP_006534392.1;XP_006534393.1;XP_006534395.1;XP_011247581.1	1920223	.	0.206034	[illegible]
72978	NM_001160211,NM_001160212,NM_028408,XM_006496989,XM_030243142	Cnih3	cornichon family AMPA receptor auxiliary protein 3	protein_coding	NP_001153683.1;NP_001153684.1;NP_082684.1;XP_006497052.1;XP_030099002.1	1920228	.	0.011843	[illegible]
72981	NM_001355114,NM_028410,XM_006508224,XM_011241915	Thap12	THAP domain containing 12	protein_coding	NP_001342043.1;NP_082686.1;XP_006508287.1;XP_011240217.1	1920231	.	27.961626	[illegible]
72982	NM_001302218,NM_028411,NR_126092,XM_011247366,XM_011247367	Tmem138	transmembrane protein 138	protein_coding	NP_001289147.1;NP_082687.1;XP_011245668.1;XP_011245669.1	1920232	.	6.709407	[illegible]
72993	NM_145221,XM_006519608,XM_017316214,XM_030248050,XR_001781159,XR_001781160,XR_003950904	Appl1	adaptor protein, phosphotyrosine interaction, PH domain and leucine zipper containing 1	protein_coding	NP_660256.1;XP_006519671.1;XP_017171703.1;XP_030103910.1	1920243	.	11.649038	[illegible]
72997	NR_040269,NR_040270	Kantr	Kdm5c adjacent non-coding transcript	lncRNA	.	1920247	.	2.112339	[illegible]
72999	NM_001271531,NM_001271532,NM_001357251,NM_133748,NM_178082,XM_006529889,XM_006529891,XM_006529892	Insig2	insulin induced gene 2	protein_coding	NP_001258460.1;NP_001258461.1;NP_001344180.1;NP_598509.1;NP_835183.1;XP_006529952.1;XP_006529954.1;XP_006529955.1	1920249	.	22.977420	[illegible]
73009	NR_045365	2900057B20Rik	RIKEN cDNA 2900057B20 gene	lncRNA	.	1920259	.	0.139433	[illegible]
73010	NM_175191,XM_030246948	Gpr22	G protein-coupled receptor 22	protein_coding	NP_780400.3;XP_030102808.1	1920260	.	0.000000	[illegible]
73016	NM_028416,XM_006524983,XM_006524984	Kremen2	kringle containing transmembrane protein 2	protein_coding	NP_082692.1;XP_006525046.3;XP_006525047.1	1920266	.	0.000000	[illegible]
73024	NM_133749,XM_006500254	Emc7	ER membrane protein complex subunit 7	protein_coding	NP_598510.1;XP_006500317.1	1920274	.	33.982483	[illegible]
73032	NM_028417	Ttc9b	tetratricopeptide repeat domain 9B	protein_coding	NP_082693.1	1920282	.	0.000000	[illegible]
73040	NR_015605	2900052N01Rik	RIKEN cDNA 2900052N01 gene	lncRNA	.	1920290	.	0.000000	[illegible]
73046	NM_028419	Glrx5	glutaredoxin 5	protein_coding	NP_082695.1	1920296	.	21.318844	[illegible]
73047	NM_028420	Camk2n2	calcium/calmodulin-dependent protein kinase II inhibitor 2	protein_coding	NP_082696.1	1920297	.	1.146736	[illegible]
73061	NM_001310652,NM_001310654,NM_028426,XM_006528410,XM_011247709	Cldn34c1	claudin 34C1	protein_coding	NP_001297581.1;NP_001297583.1;NP_082702.1;XP_006528473.1;XP_011246011.1	1920311	.	1.011983	[illegible]
73062	NM_001358933,NM_033371,NR_152559	Ppp1r16a	protein phosphatase 1, regulatory subunit 16A	protein_coding	NP_001345862.1;NP_203535.1	1920312	.	8.908508	[illegible]
73067	NM_001163747,NM_028427,XR_001778479	Tmem192	transmembrane protein 192	protein_coding	NP_001157219.1;NP_082703.1	1920317	.	6.392262	[illegible]
73068	NM_028428	Fut11	fucosyltransferase 11	protein_coding	NP_082704.1	1920318	.	12.714354	[illegible]
73072	NM_207203,XM_006508911,XM_006508912,XM_006508913,XM_006508914,XM_011242087,XM_017312980,XM_017312981,XM_017312982,XM_030243795,XM_030243796,XM_030243797,XM_030243798,XM_030243799	Prr36	proline rich 36	protein_coding	NP_997086.2;XP_006508974.1;XP_006508975.1;XP_006508976.1;XP_006508977.1;XP_011240389.1;XP_017168469.1;XP_017168470.1;XP_017168471.1;XP_030099655.1;XP_030099656.1;XP_030099657.1;XP_030099658.1;XP_030099659.1	3605626	.	0.136223	[illegible]
73075	NM_028430,XM_006512871,XM_006512872,XM_017314117,XR_380080,XR_871442	Ppil6	peptidylprolyl isomerase (cyclophilin)-like 6	protein_coding	NP_082706.1;XP_006512934.1;XP_006512935.1;XP_017169606.1	1920325	.	0.319842	[illegible]
73078	NM_028431	Pmpcb	peptidase (mitochondrial processing) beta	protein_coding	NP_082707.1	1920328	.	24.759579	[illegible]
73086	NM_001330702,NM_153587,XM_006516275,XM_030246949,XM_030246950	Rps6ka5	ribosomal protein S6 kinase, polypeptide 5	protein_coding	NP_001317631.1;NP_705815.1;XP_006516338.1;XP_030102809.1;XP_030102810.1	1920336	.	2.140100	[illegible]
73093	XR_003950646	3110006O06Rik	RIKEN cDNA 3110006O06 gene	lncRNA	.	1920343	.	0.016102	[illegible]
73094	NM_001285852,NM_001285859,NM_001285860,NM_001285862,NM_144906,XM_006503427,XM_006503428,XM_006503429,XM_006503430,XM_017320403,XM_030253826,XM_030253827,XR_001784206,XR_001784207,XR_001784208,XR_003955022,XR_003955023,XR_003955024,XR_003955025,XR_003955026,XR_003955027,XR_003955028,XR_376359,XR_376360,XR_376361,XR_376362,XR_376363,XR_376364,XR_376365,XR_376369,XR_376370	Sgip1	SH3-domain GRB2-like (endophilin) interacting protein 1	protein_coding	NP_001272781.1;NP_001272788.1;NP_001272789.1;NP_001272791.1;NP_659155.1;XP_006503490.1;XP_006503491.1;XP_006503492.1;XP_006503493.1;XP_017175892.1;XP_030109686.1;XP_030109687.1	1920344	.	0.124485	[illegible]
73095	NM_001007570	Slc25a42	solute carrier family 25, member 42	protein_coding	NP_001007571.1	1920345	.	3.772765	[illegible]
73100	NR_027891	2900092D14Rik	RIKEN cDNA 2900092D14 gene	lncRNA	.	1920350	.	0.372495	[illegible]
73102	NM_001033167,XM_006516793,XM_006516794,XM_030247433,XM_030247434	Slc22a23	solute carrier family 22, member 23	protein_coding	NP_001028339.1;XP_006516856.1;XP_006516857.1;XP_030103293.1;XP_030103294.1	1920352	.	2.403302	[illegible]
73103	NM_001172074,NM_028439,XM_006529893,XM_017312451,XM_017312452,XM_017312453,XM_030243151	3110009E18Rik	RIKEN cDNA 3110009E18 gene	protein_coding	NP_001165545.1;NP_082715.1;XP_006529956.1;XP_017167940.1;XP_017167941.1;XP_017167942.1;XP_030099011.1	1920353	.	2.287172	[illegible]
73106	NM_001042710,XM_006514199,XM_006514200,XM_006514201,XM_006514202,XM_011243584	Prss57	protease, serine 57	protein_coding	NP_001036175.1;XP_006514262.1;XP_006514263.1;XP_006514264.1;XP_006514265.1;XP_011241886.1	1920356	.	0.000000	[illegible]
73112	NM_001359559,NM_001359560,NM_028440	Abracl	ABRA C-terminal like	protein_coding	NP_001346488.1;NP_001346489.1;NP_082716.1	1920362	.	29.926422	[illegible]
73121	NM_028443	Rflna	refilin A	protein_coding	NP_082719.1	1920371	.	14.941947	[illegible]
73122	NM_001013025,NM_001357299,XM_006496300,XM_006496302,XM_006496303,XM_011238603,XM_011238604,XM_017312455,XM_030243152	Tgfbrap1	transforming growth factor, beta receptor associated protein 1	protein_coding	NP_001013043.1;NP_001344228.1;XP_006496363.1;XP_006496365.1;XP_006496366.1;XP_011236905.1;XP_011236906.1;XP_017167944.1;XP_030099012.1	2447427	.	6.584940	[illegible]
73124	NM_001291069,NM_175193,XM_006502145	Golim4	golgi integral membrane protein 4	protein_coding	NP_001277998.1;NP_780402.1;XP_006502208.1	1920374	.	36.205134	[illegible]
73129	NR_045908	3110015C05Rik	RIKEN cDNA 3110015C05 gene	lncRNA	.	1920379	.	0.000000	[illegible]
73130	NM_001347383,NM_001361466,NM_001361467,NM_028876	Tmed5	transmembrane p24 trafficking protein 5	protein_coding	NP_001334312.1;NP_001348395.1;NP_001348396.1;NP_083152.1	1921586	.	8.392450	[illegible]
73132	NM_175194,XM_030245305	Slc25a16	solute carrier family 25 (mitochondrial carrier, Graves disease autoantigen), member 16	protein_coding	NP_780403.1;XP_030101165.1	1920382	.	7.335037	[illegible]
73137	NM_028447,XM_006526287,XM_006526288,XM_006526289,XM_030250617,XM_030250618	Prrc1	proline-rich coiled-coil 1	protein_coding	NP_082723.2;XP_006526350.1;XP_006526351.1;XP_006526352.1;XP_030106477.1;XP_030106478.1	1916106	.	31.322491	[illegible]
73139	NM_028448,XM_006534333,XR_001780064	Cenpv	centromere protein V	protein_coding	NP_082724.1;XP_006534396.1	1920389	.	4.516933	[illegible]
73144	NR_015483,NR_040725,NR_131928,NR_131929,NR_131930,NR_131931	Mir100hg	Mir100 Mirlet7a-2 Mir125b-1 cluster host gene	lncRNA	.	1920394	.	51.923031	[illegible]
73149	NM_001204241,NM_153197	Clec4a3	C-type lectin domain family 4, member a3	protein_coding	NP_001191170.1;NP_694877.2	1920399	.	2.120960	[illegible]
73158	NM_028451,XM_006534334,XM_017314797	Larp1	La ribonucleoprotein domain family, member 1	protein_coding	NP_082727.1;XP_006534397.2;XP_017170286.1	1890165	.	19.523340	[illegible]
73162	NM_028453,XM_017320405,XM_017320406,XM_017320407,XM_017320408,XM_030253828,XM_030253829,XM_030253830	Otud3	OTU domain containing 3	protein_coding	NP_082729.1;XP_017175894.1;XP_017175895.1;XP_017175896.1;XP_017175897.1;XP_030109688.1;XP_030109689.1;XP_030109690.1	1920412	.	4.352241	[illegible]
73166	NM_028454	Tm7sf2	transmembrane 7 superfamily member 2	protein_coding	NP_082730.2	1920416	.	4.348998	[illegible]
73167	NM_001164627,NM_001164628,NM_001205334,NM_028455,XM_006521468,XM_006521469,XM_006521470,XM_006521471,XM_017316756	Arhgap8	Rho GTPase activating protein 8	protein_coding	NP_001158099.1;NP_001158100.1;NP_001192263.1;NP_082731.2;XP_006521531.1;XP_006521532.1;XP_006521533.1;XP_006521534.1;XP_017172245.1	1920417	.	0.153274	[illegible]
73172	NM_001160043,NM_028457	Exo5	exonuclease 5	protein_coding	NP_001153515.1;NP_082733.1	1920422	.	5.589601	[illegible]
73173	NM_130448,XM_006500789	Pcdh18	protocadherin 18	protein_coding	NP_569715.3;XP_006500852.1	1920423	.	15.308497	[illegible]
73174	NM_198100,XM_006534335,XM_006534336,XM_006534337,XM_006534338,XM_030246371,XM_030246372,XM_030246373	Tbkbp1	TBK1 binding protein 1	protein_coding	NP_932768.2;XP_006534398.1;XP_006534399.1;XP_006534400.1;XP_006534401.1;XP_030102231.1;XP_030102232.1;XP_030102233.1	1920424	.	10.782999	[illegible]
73176	XM_993996	3110040M04Rik	RIKEN cDNA 3110040M04 gene	protein_coding	XP_999090.1	1920426	.	0.020962	[illegible]
73178	NM_001167745,NM_028459	Wasl	Wiskott-Aldrich syndrome-like (human)	protein_coding	NP_001161217.1;NP_082735.2	1920428	.	14.008739	[illegible]
73181	NM_001168346,NM_023699	Nfatc4	nuclear factor of activated T cells, cytoplasmic, calcineurin dependent 4	protein_coding	NP_001161818.1;NP_076188.3	1920431	.	32.735126	[illegible]
73182	NM_001032413,NM_001032414,NM_001289600,NM_001289601,NM_028460,NM_152799,XM_006502146	Pear1	platelet endothelial aggregation receptor 1	protein_coding	NP_001027585.1;NP_001027586.1;NP_001276529.1;NP_001276530.1;NP_082736.1;NP_690012.2;XP_006502209.1	1920432	.	8.695521	[illegible]
73183	NR_015581,NR_027892	5430402O13Rik	RIKEN cDNA 5430402O13 gene	lncRNA	.	1918631	.	0.357987	[illegible]
73187	XM_017319868	3110067G11Rik	RIKEN cDNA 3110067G11 gene	protein_coding	XP_017175357.1	1920437	.	0.020458	[illegible]
73192	NM_001081056,NM_001359618,XM_006514206,XM_006514207,XM_006514208,XM_030245306,XM_030245307,XM_030245308	Xpot	exportin, tRNA (nuclear export receptor for tRNAs)	protein_coding	NP_001074525.1;NP_001346547.1;XP_006514269.1;XP_006514270.1;XP_006514271.1;XP_030101166.1;XP_030101167.1;XP_03010116	1920442	.	22.995053	[illegible]
73204	NR_045055,NR_045056	3110056K07Rik	RIKEN cDNA 3110056K07 gene	lncRNA	.	1920454	.	4.055973	[illegible]
73205	NM_001081343,NM_028466,XM_006538292,XM_006538293	C9orf72	C9orf72, member of C9orf72-SMCR8 complex	protein_coding	NP_001074812.1;NP_082742.2;XP_006538355.1;XP_006538356.1	1920455	.	4.383302	[illegible]
73208	XR_001781268,XR_874656	3110083C13Rik	RIKEN cDNA 3110083C13 gene, transcript variant X2	lncRNA	.	1920458	.	0.186614	[illegible]
73212	NM_028469,XM_006504738	3110082I17Rik	RIKEN cDNA 3110082I17 gene	protein_coding	NP_082745.2;XP_006504801.1	1920462	.	7.213047	[illegible]
73216	XR_141098	B9d1os	B9 protein domain 1, opposite strand	lncRNA	.	1920466	.	0.282320	[illegible]
73218	NM_001358803,NM_001358804,NM_175195,XM_006514209,XM_006514210,XM_006514211,XM_006514212,XM_006514213,XM_006514215,XM_011243585,XM_017314119,XM_017314120,XM_030245309,XM_030245310,XM_030245311,XM_030245312,XM_030245313,XM_030245314,XR_003948809,XR_003948810,XR_380447	Sppl2b	signal peptide peptidase like 2B	protein_coding	NP_001345732.1;NP_001345733.1;NP_780404.2;XP_006514272.1;XP_006514273.1;XP_006514274.1;XP_006514275.1;XP_006514276.1;XP_006514278.1;XP_011241887.1;XP_017169608.1;XP_017169609.1;XP_030101169.1;XP_030101170.1;XP_030101171.1;XP_030101172.1;XP_030101173.1;XP_030101174.1	1920468	.	11.493263	[illegible]

73225	NM_001356616,NM_001356617,NM_001356618,NM_133750,XM_006521474,XM_006521475,XM_006521476,XM_006521478,XM_006521479,XM_006521480,XM_006521481,XM_006521482,XM_011245743,XM_030248793,XR_001781570,XR_001781571,XR_003951442,XR_003951443,XR_384052	Fam118a	family with sequence similarity 118, member A	protein_coding	[illegible]
73229	NM_001166497,NM_001357098,XM_017317669,XM_017317670,XM_030250066,XM_030250067,XM_030250068,XM_030250069,XM_030250070	Zfp983	zinc finger protein 983	protein_coding	[illegible]
73230	NM_028472	Bmper	BMP-binding endothelial regulator	protein_coding	[illegible]
73233	NM_001199048,XM_030250072,XM_030250073,XM_030250074,XM_030250075,XM_030250076,XM_030250077,XM_030250078	Zfp942	zinc finger protein 942	protein_coding	[illegible]
73234	NM_001356490,NM_028473	Snorc	secondary ossification center associated regulator of chondrocyte maturation	protein_coding	[illegible]
73242	NM_001142744,NM_001142745,NM_001347378,NM_001357865,NM_028476,XM_017317675,XM_017317676,XM_030250079,XM_030250080,XM_030250081,XM_030250082,XM_030250083,XM_030250085,XM_030250086	Atat1	alpha tubulin acetyltransferase 1	protein_coding	[illegible]
73246	NM_001310656,NM_028478,XM_006535241,XM_006535242,XM_006535243,XM_006535244,XM_006535245,XM_006535246,XM_006535247,XM_006535249,XM_011249579,XR_001784751	Rassf6	Ras association (RalGDS/AF-6) domain family member 6	protein_coding	[illegible]
73247	NM_001356494,NM_028479,NR_151481,NR_151482,XR_375404	Mrgbp	MRG/MORF4L binding protein	protein_coding	[illegible]
73251	NM_080793	Setd7	SET domain containing (lysine methyltransferase) 7	protein_coding	[illegible]
73254	NM_028481,XM_006535251,XM_006535252,XM_006535253,XM_006535255,XR_003955715	Ccdc18	coiled-coil domain containing 18	protein_coding	[illegible]
73261	NM_028484	1700037C18Rik	RIKEN cDNA 1700037C18 gene	protein_coding	[illegible]
73270	NR_045363	1700024F13Rik	RIKEN cDNA 1700024F13 gene	lncRNA	[illegible]
73272	NR_015485	1700108F19Rik	RIKEN cDNA 1700108F19 gene	lncRNA	[illegible]
73274	NM_001122963,NM_001361923,NM_001361924,NM_001361925,NM_001361926,NM_028487,XM_006517772,XM_030247435,XM_030247436	Gpbp1	GC-rich promoter binding protein 1	protein_coding	[illegible]
73284	NM_030143	Ddit4l	DNA-damage-inducible transcript 4-like	protein_coding	[illegible]
73287	NM_028491,XM_006514216	Cabcoco1	ciliary associated calcium binding coiled-coil 1	protein_coding	[illegible]
73288	NM_001167750,NM_001167751,NM_024260	Vps50	VPS50 EARP/GARPII complex subunit	protein_coding	[illegible]
73293	NM_001364757,NM_028492	Ccdc103	coiled-coil domain containing 103	protein_coding	[illegible]
73296	NM_028493,XM_006517410,XM_006517411,XM_006517412,XM_011244573,XM_011244574	Rhobtb3	Rho-related BTB domain containing 3	protein_coding	[illegible]
73297	NR_045380	1700034I23Rik	RIKEN cDNA 1700034I23 gene	transcribed_pseudogene	[illegible]
73303	NR_040610	Bcas1os2	breast carcinoma amplified sequence 1, opposite strand 2	lncRNA	[illegible]
73314	NM_028499,XM_006538295,XM_030253831,XM_030253832	Lrrc69	leucine rich repeat containing 69	protein_coding	[illegible]
73316	NM_028500,NM_029782,XM_006509761,XM_030243800	Calr3	calreticulin 3	protein_coding	[illegible]
73321	NR_045178	1700042O10Rik	RIKEN cDNA 1700042O10 gene	lncRNA	[illegible]
73324	NM_001163521,NM_028504,XM_006514818,XM_006514819,XM_030246374,XR_001780065	Clhc1	clathrin heavy chain linker domain containing 1	protein_coding	[illegible]
73327	NM_001163427,NM_028505,NR_028081,XM_011241486,XR_003956256	Pradc1	protease-associated domain containing 1	protein_coding	[illegible]
73331	NR_040462	1700034P13Rik	RIKEN cDNA 1700034P13 gene	lncRNA	[illegible]
73332	NM_001270435,NM_001270446,NM_028506,NM_028698,NM_028857,NM_029286,XM_011240615,XM_011240616,XM_011240617,XM_011240618,XM_011240619,XM_017320409,XM_030253833,XR_001784210,XR_003955029,XR_867894	Ccdc30	coiled-coil domain containing 30	protein_coding	[illegible]
73333	NM_178386	Slc25a31	solute carrier family 25 (mitochondrial carrier; adenine nucleotide translocator), member 31	protein_coding	[illegible]
73336	NM_148940,XM_006512313,XM_006512314,XM_006512317,XM_017313617,XM_030244649	Prss44	protease, serine 44	protein_coding	[illegible]
73338	NM_001163527,NM_001163528,NM_001355634,XM_006500255,XM_030252117	Itpripl1	inositol 1,4,5-triphosphate receptor interacting protein-like 1	protein_coding	[illegible]
73340	NM_030689	Nptxr	neuronal pentraxin receptor	protein_coding	[illegible]
73341	NM_001358573,NM_152801,XM_006528313,XM_006528314,XM_006528315,XM_030251508,XM_030251509,XM_030251510,XM_030251511,XM_030251512	Arhgef6	Rac/Cdc42 guanine nucleotide exchange factor (GEF) 6	protein_coding	[illegible]
73344	NM_001164236,NM_028509,XM_006507112,XM_011241632,XM_017321773,XM_017321774,XM_017321775,XM_017321776,XM_017321777,XM_030255626	1700034J05Rik	RIKEN cDNA 1700034J05 gene	protein_coding	[illegible]
73346	XR_003948680,XR_373460	1700039I01Rik	RIKEN cDNA 1700039I01 gene, transcript variant X2	lncRNA	[illegible]
73348	NR_040649	1700045H11Rik	RIKEN cDNA 1700045H11 gene	lncRNA	[illegible]
73363	NM_028516	1700056E22Rik	RIKEN cDNA 1700056E22 gene	protein_coding	[illegible]
73365	XR_001782188,XR_876697	1700049J03Rik	RIKEN cDNA 1700049J03 gene, transcript variant X1	lncRNA	[illegible]
73366	NR_045922	1700051A21Rik	RIKEN cDNA 1700051A21 gene	lncRNA	[illegible]
73368	NM_028518,XM_006500727,XM_017319287,XM_017319288	Col20a1	collagen, type XX, alpha 1	protein_coding	[illegible]
73373	NM_028521,XM_006500257	Phospho2	phosphatase, orphan 2	protein_coding	[illegible]
73379	NM_001356484,NM_028523,XM_006522624,XM_011246022,XM_030249295	Dcbld2	discoidin, CUB and LCCL domain containing 2	protein_coding	[illegible]
73382	NM_028525	Prss52	protease, serine 52	protein_coding	[illegible]
73385	NM_028527,XM_006516277	Fam177a	family with sequence similarity 177, member A	protein_coding	[illegible]
73389	NM_001361928,NM_153198,NM_177993,XM_006515227,XM_006515228,XM_006515229,XM_030246951	Hbp1	high mobility group box transcription factor 1	protein_coding	[illegible]
73390	NM_001163833,XM_006512876	Msl3l2	MSL3 like 2	protein_coding	[illegible]
73399	NR_036606	1700060C20Rik	RIKEN cDNA 1700060C20 gene	lncRNA	[illegible]
73405	XR_035225	1700055D18Rik	RIKEN cDNA 1700055D18 gene	lncRNA	[illegible]
73407	NM_028532,NM_199455,XM_006531402,XM_030243801,XR_001778480	Tepp	testis, prostate and placenta expressed	protein_coding	[illegible]
73410	NM_001271569,NR_073361	1700065D16Rik	RIKEN cDNA 1700065D16 gene	protein_coding	[illegible]
73419	NM_024261,XM_011243100,XM_011243101,XM_011243102,XM_030245315,XM_030245316	Armt1	acidic residue methyltransferase 1	protein_coding	[illegible]
73420	NM_028536,XM_030243802	Ccsap	centriole, cilia and spindle associated protein	protein_coding	[illegible]
73422	NM_001361238,NM_001361239,NM_175198,XM_006516278,XM_006516281,XM_011244187,XM_017315213,XM_030246952	Prox2	prospero homeobox 2	protein_coding	[illegible]
73430	NM_028538,XM_030243019,XM_030243020,XM_030243021	Zfp974	zinc finger protein 974	protein_coding	[illegible]
73431	NR_027956	1700052K11Rik	RIKEN cDNA 1700052K11 gene	lncRNA	[illegible]
73435	NM_001305059,NM_001305061,NM_001305062,NM_001305063,NM_028540,XM_006496990,XM_017312458,XM_017312459,XM_017312460,XM_017312462,XM_017312463,XM_030243166	Tex35	testis expressed 35	protein_coding	[illegible]
73442	NM_001327998,NM_175199,XM_006527377,XM_006527379,XM_011247368	Hspa12a	heat shock protein 12A	protein_coding	[illegible]
73447	NM_001290783,NM_026137,NM_028542,XM_006527703	Wdr13	WD repeat domain 13	protein_coding	[illegible]
73449	NM_001033168,XM_006526290	1700066B19Rik	RIKEN cDNA 1700066B19 gene	protein_coding	[illegible]
73451	NM_028543	Zfp763	zinc finger protein 763	protein_coding	[illegible]
73453	NM_183097,XM_006531403	1700067K01Rik	RIKEN cDNA 1700067K01 gene	protein_coding	[illegible]
73456	NM_001018013	Izumo1	izumo sperm-egg fusion 1	protein_coding	[illegible]
73457	NR_045288	1700064M15Rik	RIKEN cDNA 1700064M15 gene	lncRNA	[illegible]
73463	NM_026600,NM_175200,XM_011238605,XM_011238634,XM_011238635,XM_011238636,XM_017312469,XM_017313438,XM_030243180	C2cd6	C2 calcium dependent domain containing 6	protein_coding	[illegible]
73467	NM_028546,XM_030243183,XR_373264	1700066M21Rik	RIKEN cDNA 1700066M21 gene	protein_coding	[illegible]
73469	NM_001038993,NM_001355181,NM_175201,XM_006538298,XM_006538299,XM_006538300,XM_006538301,XM_006538302,XM_006538304,XM_006538305,XM_006538306,XM_011250115,XM_011250116,XM_011250117,XM_011250119,XM_011250120,XM_030253834	Rnf38	ring finger protein 38	protein_coding	[illegible]
73472	NM_178387,XM_006504155,XM_006504159,XM_006504160,XR_001784752,XR_003955716	Spata18	spermatogenesis associated 18	protein_coding	[illegible]
73473	NM_173441,XM_006526291,XM_006526292,XR_386017	Iws1	IWS1, SUPT6 interacting protein	protein_coding	[illegible]
73474	NR_027900	Snhg9	small nucleolar RNA host gene 9	lncRNA	[illegible]
73490	NM_001164370	Mipol1	mirror-image polydactyly 1	protein_coding	[illegible]
73504	NR_045444,NR_045445	1700071M16Rik	RIKEN cDNA 1700071M16 gene	lncRNA	[illegible]
73516	NR_015475,NR_027903,NR_027904	1700086O06Rik	RIKEN cDNA 1700086O06 gene	lncRNA	[illegible]
73523	NM_028526,NM_028560	Pebp4	phosphatidylethanolamine binding protein 4	protein_coding	[illegible]
73545	NM_028567	1700094D03Rik	RIKEN cDNA 1700094D03 gene	protein_coding	[illegible]
73549	NR_038059	1700110I01Rik	RIKEN cDNA 1700110I01 gene	lncRNA	[illegible]
73557	NR_131165	1700096P03Rik	RIKEN cDNA 1700096P03 gene	lncRNA	[illegible]
73558	NR_040728	1700110K17Rik	RIKEN cDNA 1700110K17 gene	lncRNA	[illegible]
73569	NM_001368760,NM_001368761,NM_028572,XM_006523098	Vgll3	vestigial like family member 3	protein_coding	[illegible]
73571	NR_027388	1700096K18Rik	RIKEN cDNA 1700096K18 gene	lncRNA	[illegible]
73579	XR_003954694,XR_003954695,XR_003954696,XR_867780	1700094M23Rik	RIKEN cDNA 1700094M23 gene, transcript variant X3	lncRNA	[illegible]
73582	NM_001364946,NM_028576,XM_006524992,XM_006524993,XR_385373	Camkmt	calmodulin-lysine N-methyltransferase	protein_coding	[illegible]
73590	NR_040472	1700112H15Rik	RIKEN cDNA 1700112H15 gene	lncRNA	[illegible]
73593	XR_387693,XR_878753,XR_878754	1700112N08Rik	RIKEN cDNA 1700112N08 gene, transcript variant X1	lncRNA	[illegible]
73598	NM_198000,NR_028486,NR_028487,XM_006498375,XM_006498376,XM_011239195,XM_011239196,XM_011239197,XM_011239198,XM_011239199,XM_011239200,XM_011239201,XM_030252119,XM_030252120,XR_866021	1700001O22Rik	RIKEN cDNA 1700001O22 gene	protein_coding	[illegible]
73600	NR_045627	1700120C14Rik	RIKEN cDNA 1700120C14 gene	lncRNA	[illegible]
73608	NM_028584,NM_212447	Marveld3	MARVEL (membrane-associating) domain containing 3	protein_coding	[illegible]
73609	NR_038181,NR_038189	1700125H03Rik	RIKEN cDNA 1700125H03 gene	lncRNA	[illegible]
73610	NM_001243067,XM_011243586,XM_011243587,XM_011243588,XM_017314122,XM_017314123,XM_017314124	Zfp433	zinc finger protein 433	protein_coding	[illegible]
73611	XR_003954445,XR_003954446,XR_003954447,XR_003954448,XR_003954449,XR_003954450,XR_003954451,XR_003954452,XR_389634	1700112D23Rik	RIKEN cDNA 1700112D23 gene, transcript variant X8	lncRNA	[illegible]
73619	XR_106144	1700122E12Rik	RIKEN cDNA 1700122E12 gene	lncRNA	[illegible]
73634	NM_028589,XM_006534339,XM_006534340,XM_006534341,XM_006534342,XM_006534344,XM_011249281,XM_030246375,XM_030246376	1700125H20Rik	RIKEN cDNA 1700125H20 gene	protein_coding	[illegible]
73635	NM_001357574,NM_001361123,NM_026865,XM_006534346,XR_879745	Ptges3l	prostaglandin E synthase 3 like	protein_coding	[illegible]
73644	NR_044985	2210039B01Rik	RIKEN cDNA 2210039B01 gene	lncRNA	[illegible]
73647	NM_023709,XM_006531404,XM_006531405	Capn9	calpain 9	protein_coding	[illegible]
73649	NM_028593	Cybrd1	cytochrome b reductase 1	protein_coding	[illegible]
73652	NR_040257,NR_040258,NR_040259,NR_040260,NR_040261,NR_040262	2210408F21Rik	RIKEN cDNA 2210408F21 gene	lncRNA	[illegible]
73656	NM_001166376,NM_028595,XM_006527381,XR_877690	Ms4a6c	membrane-spanning 4-domains, subfamily A, member 6C	protein_coding	[illegible]
73658	NM_023712,NR_045537	Spns1	spinster homolog 1	protein_coding	[illegible]
73660	NM_144532,XM_030251095,XM_030251096,XM_030251097,XM_030251098	Cabp4	calcium binding protein 4	protein_coding	[illegible]
73666	NM_028597	Thoc3	THO complex 3	protein_coding	[illegible]
73667	NM_001201332,NM_001201333,XM_006515231	2410004P03Rik	RIKEN cDNA 2410004P03 gene	protein_coding	[illegible]
73668	NM_001047604,NM_001290669,XM_006500263,XM_030252122	Ttc21b	tetratricopeptide repeat domain 21B	protein_coding	[illegible]
73671	NM_001163625,XM_011246656	Sult6b1	sulfotransferase family, cytosolic, 6B, member 1	protein_coding	[illegible]
73673	NM_028598,XM_006511491,XM_011242819,XM_011242820,XM_011242821,XM_011242822,XM_011242823,XM_030244650	Rec114	REC114 meiotic recombination protein	protein_coding	[illegible]
73674	NM_028599,XM_006496307	Wdr75	WD repeat domain 75	protein_coding	[illegible]
73677	NM_001163609,NM_001360823,XM_006526294	Psma8	proteasome (prosome, macropain) subunit, alpha type, 8	protein_coding	[illegible]
73679	NM_028602	Tex19.1	testis expressed gene 19.1	protein_coding	[illegible]
73680	NM_028603	Zbtb8a	zinc finger and BTB domain containing 8a	protein_coding	[illegible]
73681	NM_001358806,NM_028604,XM_006512877,XM_006512880,XM_011243211,XM_011243212,XM_030245317,XM_030245318,XM_030245319,XM_030245320,XR_003948811	Trmt11	tRNA methyltransferase 11	protein_coding	[illegible]
73683	NM_001111111,NR_152152,XM_006508225,XM_006508226,XM_006508227,XM_006508228,XM_006508230,XM_006508231,XM_011241916,XM_011241917,XM_011241918,XM_030243022,XM_030243023,XR_001778004,XR_003946569,XR_003946570,XR_003946571,XR_378274	Atg16l2	autophagy related 16-like 2 (S. cerevisiae)	protein_coding	[illegible]
73689	NM_028607,XM_011247369	Bloc1s2	biogenesis of lysosomal organelles complex-1, subunit 2	protein_coding	[illegible]
73690	NM_028608,XM_006514217,XM_006514218	Glipr1	GLI pathogenesis-related 1 (glioma)	protein_coding	[illegible]
73692	NM_001162906,XM_006519997,XM_006519998,XM_006519999,XM_006520000,XM_006520001,XM_006520002,XM_011245335,XM_011245336,XM_030248795,XM_030248796,XM_030248797,XM_030248798,XM_030248799,XM_030248800,XM_030248801	Cplane1	ciliogenesis and planar polarity effector 1	protein_coding	[illegible]
73694	NM_028611,XM_006524995,XR_385374	Ndufaf7	NADH:ubiquinone oxidoreductase complex assembly factor 7	protein_coding	[illegible]
73699	NM_001034085,NM_001286553,NM_028614,XM_006510641,XM_006510642,XR_001779023,XR_001779024	Ppp2r1b	protein phosphatase 2, regulatory subunit A, beta	protein_coding	[illegible]
73703	NM_028615	Dppa2	developmental pluripotency associated 2	protein_coding	[illegible]
73707	NM_001081076,NM_001371012,XM_030251100,XM_030251101,XM_030251102	Gucy2g	guanylate cyclase 2g	protein_coding	[illegible]
73708	NM_139218	Dppa3	developmental pluripotency-associated 3	protein_coding	[illegible]
73710	NM_023716	Tubb2b	tubulin, beta 2B class IIB	protein_coding	[illegible]
73711	NM_028617,XM_011242330,XM_030243804	Mvb12a	multivesicular body subunit 12A	protein_coding	[illegible]
73712	NM_001166173,NM_001166174,NM_001369359,NM_028618,NM_172899	Dmkn	dermokine	protein_coding	[illegible]
73713	NM_001170847,XM_006527385,XM_011247371,XM_011247372	Rbm20	RNA binding motif protein 20	protein_coding	[illegible]
73720	NM_028623	Cst6	cystatin E/M	protein_coding	[illegible]
73721	NM_001048005,NM_001253777,NM_001253778,NM_001253780,NM_001253788,NM_028624,NR_045549,NR_045551,XM_006538307,XM_006538308,XM_030253836,XM_030253837,XR_003955030	1110017D15Rik	RIKEN cDNA 1110017D15 gene	protein_coding	[illegible]
73723	NM_080559	Sh3bgrl3	SH3 domain binding glutamic acid-rich protein-like 3	protein_coding	[illegible]
73724	NM_001355124,NM_028626	Mcee	methylmalonyl CoA epimerase	protein_coding	[illegible]
73728	NM_001347454,NM_028627,XM_006527386,XM_006527388,XM_006527389,XM_006527391,XM_006527393,XM_011247376,XM_011247377,XM_030251104,XM_030251105,XM_030251106,XM_030251107,XM_030251108,XM_030251109,XM_030251110,XM_030251111,XR_386518	Psd	pleckstrin and Sec7 domain containing	protein_coding	[illegible]
73729	NM_001243908,XM_006540391,XM_011250725	Zfp383	zinc finger protein 383	protein_coding	[illegible]
73736	NM_028632	Fcf1	FCF1 rRNA processing protein	protein_coding	[illegible]
73737	NM_198001	1110008P14Rik	RIKEN cDNA 1110008P14 gene	protein_coding	[illegible]
73738	NM_028633,NM_207104	Haus7	HAUS augmin-like complex, subunit 7	protein_coding	[illegible]
73739	NM_028634,XM_006521484	Cby1	chibby family member 1, beta catenin antagonist	protein_coding	[illegible]
73744	NM_028636,XM_006511494,XM_006511499,XM_017313619,XM_030244651,XM_030244652,XM_030244653,XM_030244654,XM_030244655,XM_030244656,XM_030244657,XM_030244658,XR_001779027,XR_001779028,XR_001779033,XR_001779034,XR_001779035,XR_001779036,XR_001779037,XR_001779038,XR_001779039,XR_001779040,XR_003948024,XR_003948025,XR_003948026,XR_003948027,XR_003948028,XR_003948029,XR_003948030,XR_003948031,XR_003948032,XR_003948033,XR_003948034	Man2c1	mannosidase, alpha, class 2C, member 1	protein_coding	[illegible]
73747	NM_001358260,NM_001358261,NM_028637,XM_011239798,XM_011239799,XM_011239800,XM_011239802	Shld1	shieldin complex subunit 1	protein_coding	[illegible]
73748	NM_028638,XM_006512319,XM_017313627,XM_017313628	Gadl1	glutamate decarboxylase-like 1	protein_coding	[illegible]
73750	NM_001008791,NM_001008792,NM_001008793,NM_001276371,NM_028640,XM_006538309,XR_001784211,XR_001784212,XR_390	Whrn	whirlin	protein_coding	[illegible]
73754	NM_199042	Thap1	THAP domain containing, apoptosis associated protein 1	protein_coding	[illegible]
73761	NR_126430	4833415N18Rik	RIKEN cDNA 4833415N18 gene	lncRNA	[illegible]
73774	NR_151500	4930422M22Rik	RIKEN cDNA 4930422M22 gene	lncRNA	[illegible]
73784	NR_045186	4930430F21Rik	RIKEN cDNA 4930430F21 gene	lncRNA	[illegible]
73804	NM_001290662,NM_134471,XM_006503433,XM_006503435,XM_011240620,XM_011240621,XM_011240622,XM_017320411,XM_030253838	Kif2c	kinesin family member 2C	protein_coding	[illegible]
73808	NM_028654	Tex36	testis expressed 36	protein_coding	[illegible]
73813	NM_001033170,XM_006541224,XR_003946572	Fam83e	family with sequence similarity 83, member E	protein_coding	[illegible]
73815	NR_045941	4930404H11Rik	RIKEN cDNA 4930404H11 gene	lncRNA	[illegible]
73822	NM_028657,XM_006514219	Mfsd12	major facilitator superfamily domain containing 12	protein_coding	[illegible]
73824	NR_024067	Snhg6	small nucleolar RNA host gene 6	lncRNA	[illegible]
73825	NM_028658,XM_017317677	Ppp1r21	protein phosphatase 1, regulatory subunit 21	protein_coding	[illegible]
73826	NM_001347082,NM_178627	Poldip3	polymerase (DNA-directed), delta interacting protein 3	protein_coding	[illegible]
73827	NM_001358266,NM_001358267,NM_178066,XM_006514222,XM_006514224,XM_006514225,XM_006514226,XM_030245321,XM_030245322,XM_030245323,XM_030245324,XM_030245325	Tmem198b	transmembrane protein 198b	protein_coding	[illegible]
73828	NM_001165256,NM_030246,XM_006516282,XM_006516283,XM_006516284,XM_030246953,XM_030246954,XM_030246955	Dcaf4	DDB1 and CUL4 associated factor 4	protein_coding	[illegible]
73830	NM_001285942,NM_001285943,NM_028659,XM_006540392	Eif3k	eukaryotic translation initiation factor 3, subunit K	protein_coding	[illegible]
73833	NM_001146023,XM_006540393,XM_006540396,XM_017312335,XM_017312336	Fam98c	family with sequence similarity 98, member C	protein_coding	[illegible]
73834	NM_023721,XM_030246956	Atp6v1d	ATPase, H+ transporting, lysosomal V1 subunit D	protein_coding	[illegible]
73835	NM_053088	Ifitm5	interferon induced transmembrane protein 5	protein_coding	[illegible]
73836	NM_001357105,XM_030250089	Slc35b2	solute carrier family 35, member B2	protein_coding	[illegible]
73844	NM_001368766,NM_028664,XM_006496995,XM_017312485,XM_017312486,XM_030243184,XM_030243188,XM_030243189,XM_030243190,XM_030243191,XM_030243192	Ankrd45	ankyrin repeat domain 45	protein_coding	[illegible]
73845	NM_028665,XM_006508232,XM_011241919,XM_017312337	Ankrd42	ankyrin repeat domain 42	protein_coding	[illegible]
73847	NM_001289150,NM_001289151,NM_028666,NM_146127,XM_006500266,XM_006500268	Fam110a	family with sequence similarity 110, member A	protein_coding	[illegible]

73852	NM_001099785,NM_001291048,NM_027271,NM_028667,XM_011239972,XM_011239973,XM_017319750,XM_030252844,XR_867177	D3Ertd751e	DNA segment, Chr 3, ERATO Doi 751, expressed	protein_coding	NP_001093255.1;NP_001277977.1;NP_081547.1;NP_082943.2;XP_011238274.1;XP_011238275.1;XP_017175239.1;XP_030108704.1	1289213	.	2.747065	[illegible]
73856	NR_040730	4930428G15Rik	RIKEN cDNA 4930428G15 gene	lncRNA	.	1921106	.	0.000000	[illegible]
73862	NM_001347068,NM_028669,XM_006514822,XM_006514823,XM_006514824,XM_006514826,XM_017314798	Spata48	spermatogenesis associated 48	protein_coding	NP_001333997.1;NP_082945.1;XP_006514885.1;XP_006514886.1;XP_006514887.2;XP_006514889.1;XP_017170287.1	1921112	.	0.354107	[illegible]
73863	NM_001201322	4930415O20Rik	RIKEN cDNA 4930415O20 gene	protein_coding	NP_001188251.1	1921113	.	0.000000	[illegible]
73870	NR_015527	4930417O13Rik	RIKEN cDNA 4930417O13 gene	lncRNA	.	1921120	.	0.000000	[illegible]
73871	XR_383257	4930428N03Rik	RIKEN cDNA 4930428N03 gene	lncRNA	.	1921121	.	0.000000	[illegible]
73873	NM_001363282,NM_001363283,NM_001363284,NM_028672,XM_006514828,XM_006514829,XM_006514830,XM_006514831,XM_006514832,XM_006514833,XM_006514834,XM_006514836,XM_006514838,XM_011243760,XM_011243761,XM_011243762,XM_017314799,XM_030246377,XM_030246378,XM_030246379,XR_001780067,XR_003949516,XR_003949517,XR_003949518	Fam161a	family with sequence similarity 161, member A	protein_coding	NP_001350211.1;NP_001350212.1;NP_001350213.1;NP_082948.2;XP_006514891.1;XP_006514892.1;XP_006514893.1;XP_006514894.1;XP_006514895.1;XP_006514896.1;XP_006514897.1;XP_006514899.1;XP_006514901.1;XP_011242062.1;XP_011242063.1;XP_011242064.1;XP_017170288.1;XP_030102237.1;XP_030102238.1;XP_030102239.1	1921123	.	4.441120	[illegible]
73874	NR_040595	4930419G24Rik	RIKEN cDNA 4930419G24 gene	lncRNA	.	1921124	.	0.000000	[illegible]
73877	NR_045953	4930432J09Rik	RIKEN cDNA 4930432J09 gene	lncRNA	.	1921127	.	0.014827	[illegible]
73883	NR_153763	4930431P22Rik	RIKEN cDNA 4930431P22 gene	lncRNA	.	1921133	.	0.000000	[illegible]
73884	NM_001267872,NM_001285936,NM_001285937,NM_028673,XM_011238606,XM_011238607,XM_030243194,XM_030243195,XM_030243196,XM_030243197	Zdbf2	zinc finger, DBF-type containing 2	protein_coding	NP_001254801.1;NP_001272865.1;NP_001272866.1;NP_082949.1;XP_011236908.1;XP_011236909.1;XP_030099054.1;XP_030099055.1;XP_030099056.1;XP_030099057.1	1921134	.	2.438192	[illegible]
73893	NM_178388,XM_017313629	Tmem202	transmembrane protein 202	protein_coding	NP_848475.1;XP_017169118.1	1921143	.	0.207093	[illegible]
73895	NR_045059	4930431P03Rik	RIKEN cDNA 4930431P03 gene	lncRNA	.	1921145	.	0.020772	[illegible]
73899	XR_168585	LOC73899	uncharacterized LOC73899	misc_RNA	.	.	.	0.026767	[illegible]
73902	NM_175204	Psmb11	proteasome (prosome, macropain) subunit, beta type, 11	protein_coding	NP_780413.1	1921152	.	0.000000	[illegible]
73904	NR_045954	4833412C05Rik	RIKEN cDNA 4833412C05 gene	lncRNA	.	1921154	.	0.607690	[illegible]
73906	NR_045187	4833417C18Rik	RIKEN cDNA 4833417C18 gene	lncRNA	.	1921156	.	0.525810	[illegible]
73910	NM_176837,XM_006512885,XR_001779622	Arhgap18	Rho GTPase activating protein 18	protein_coding	NP_789807.1;XP_006512948.1	1921160	.	6.964209	[illegible]
73914	NM_001359184,NM_028679,NR_152856,XM_006514227,XM_006514228	Irak3	interleukin-1 receptor-associated kinase 3	protein_coding	NP_001346113.1;NP_082955.2;XP_006514290.1;XP_006514291.1	1921164	.	2.097038	[illegible]
73915	NR_040328	4833419F23Rik	RIKEN cDNA 4833419F23 gene	lncRNA	.	1921165	.	0.598471	[illegible]
73916	NM_028680	Ift57	intraflagellar transport 57	protein_coding	NP_082956.2	1921166	.	8.229723	[illegible]
73919	NM_001285959,NM_001285960,NM_001285961,NM_029610,NR_126475,XM_006508234,XM_006508235,XM_006508236,XM_006508237,XM_006508238,XM_006508239,XM_006508240,XM_006508241,XM_006508243,XM_006508244,XM_017312338,XM_017312339	Lyrm1	LYR motif containing 1	protein_coding	NP_001272888.1;NP_001272889.1;NP_001272890.1;NP_083886.1;XP_006508297.1;XP_006508298.1;XP_006508299.1;XP_006508300.1;XP_006508301.1;XP_006508302.1;XP_006508303.1;XP_006508304.1;XP_006508306.1;XP_006508307.1;XP_017167827.1;XP_01716782[illegible]	1921169	.	1.090683	[illegible]
73936	NM_028687,XM_017315215	Ccdc175	coiled-coil domain containing 175	protein_coding	NP_082963.1;XP_017170704.1	1921186	.	0.000425	[illegible]
73940	NM_022031,XM_006502148	Hapln2	hyaluronan and proteoglycan link protein 2	protein_coding	NP_071314.1;XP_006502211.1	2137300	.	0.096101	[illegible]
73941	XR_001784296,XR_003955404,XR_003955405,XR_003955406,XR_003955407,XR_003955408,XR_003955409,XR_390567	4930412L05Rik	RIKEN cDNA 4930412L05 gene, transcript variant X4	lncRNA	.	1921191	.	0.223544	[illegible]
73942	NM_001163627	Fam151b	family with sequence similarity 151, member B	protein_coding	NP_001157099.1	1921192	.	2.240794	[illegible]
73945	NM_001081164,NM_001256033,XM_006531406	Otud4	OTU domain containing 4	protein_coding	NP_001074633.1;NP_001242962.1;XP_006531469.1	1098801	.	10.792465	[illegible]
73951	NR_045614	4930413G21Rik	RIKEN cDNA 4930413G21 gene	lncRNA	.	1921201	.	0.029509	[illegible]
73973	XR_878373,XR_878374	4930434B07Rik	RIKEN cDNA 4930434B07 gene, transcript variant X2	lncRNA	.	1921223	.	0.000000	[illegible]
73985	NR_125720	4930445N18Rik	RIKEN cDNA 4930445N18 gene	lncRNA	.	1921235	.	0.138979	[illegible]
73988	XM_001477470,XM_017314866	4930438A08Rik	RIKEN cDNA 4930438A08 gene, transcript variant X1	protein_coding	XP_001477520.3;XP_017170355.1	1921238	.	0.000000	[illegible]
73989	NR_045060	4930452G13Rik	RIKEN cDNA 4930452G13 gene	lncRNA	.	1921239	.	0.868027	[illegible]
73990	NR_045352	4930455C13Rik	RIKEN cDNA 4930455C13 gene	lncRNA	.	1921240	.	0.000000	[illegible]
73991	NM_178628	Atl1	atlastin GTPase 1	protein_coding	NP_848743.1	1921241	.	1.939933	[illegible]
[illegible]	[illegible]	[illegible]	[illegible]	[illegible]	[illegible]	[illegible]	[illegible]	[illegible]	[illegible]

74176	NM_001362724,NM_028799	Tgm5	transglutaminase 5	protein_coding	NP_001349653.1;NP_083075.1	1921426	.	0.054281
74178	NM_001145827,NM_028800,XM_006503441,XM_006503442,XM_017320414	Stk40	serine/threonine kinase 40	protein_coding	NP_001139299.1;NP_083076.3;XP_006503504.1;XP_006503505.1;XP_017175903.1	1921428	.	10.630712
74180	NM_028801,XM_006508653	Muc5b	mucin 5, subtype B, tracheobronchial	protein_coding	NP_083077.2;XP_006508716.2	1921430	.	0.000000
74182	NM_001042672,NM_001291050,NM_001291051,NM_001291052,NM_001355648,NM_001355649,NM_001355650,NM_001355651,NM_001355652,NM_027096,NM_028802,XM_006500276,XM_006500277,XM_006500279,XM_006500280,XM_017319293,XM_030252130,XM_030252131,XM_030252132,XM_030252133,XR_003953727,XR_003953728	Gpcpd1	glycerophosphocholine phosphodiesterase 1	protein_coding	NP_001036137.1;NP_001277979.1;NP_001277980.1;NP_001277981.1;NP_001342577.1;NP_001342578.1;NP_001342579.1;NP_001342580.1;NP_001342581.1;NP_081372.1;NP_083078.3;XP_006500339.1;XP_006500340.1;XP_006500342.1;XP_006500343.1;XP_017174782.1;XP_030107990.1;XP_030107991.1;XP_030107992.1;XP_030107993.1	104898	.	20.243270
74183	NM_172417,XM_006539212	Perm1	PPARGC1 and ESRR induced regulator, muscle 1	protein_coding	NP_766005.2;XP_006539275.1	1921433	.	0.323916
74184	NR_038055	2310065F04Rik	RIKEN cDNA 2310065F04 gene	lncRNA	.	1921434	.	0.069574
74185	NM_028803	Gbe1	glucan (1,4-alpha-), branching enzyme 1	protein_coding	NP_083079.1	1921435	.	5.063831
74186	NM_028804,XR_001783188	Ccdc3	coiled-coil domain containing 3	protein_coding	NP_083080.1	1921436	.	39.346688
74187	NM_028805,XM_006531410,XM_006531411,XM_006531412,XR_001778483,XR_001778484,XR_387781	Katnb1	katanin p80 (WD40-containing) subunit B 1	protein_coding	NP_083081.2;XP_006531473.1;XP_006531474.1;XP_006531475.1	1921437	.	8.151932
74189	NM_001007154,NM_001177789,NM_001177790,NM_001177791,NM_028806,XM_006500730	Phactr3	phosphatase and actin regulator 3	protein_coding	NP_001007155.1;NP_001171260.1;NP_001171261.1;NP_001171262.1;NP_083082.1;XP_006500793.1	1921439	.	0.016096
74190	NM_001289487,NM_001289488,NM_001289489,NM_028807,XM_006516289,XM_006516291,XM_006516292	Exoc3l4	exocyst complex component 3-like 4	protein_coding	NP_001276416.1;NP_001276417.1;NP_001276418.1;NP_083083.3;XP_006516352.1;XP_006516354.1;XP_006516355.1	1921363	.	5.722632
74191	NM_028808	P2ry13	purinergic receptor P2Y, G-protein coupled 13	protein_coding	NP_083084.2	1921441	.	0.682754
74192	NM_028809,XM_006498389,XM_006498391	Arpc5l	actin related protein 2/3 complex, subunit 5-like	protein_coding	NP_083085.1;XP_006498452.1;XP_006498454.1	1921442	.	21.164085
74194	NM_028810	Rnd3	Rho family GTPase 3	protein_coding	NP_083086.1	1921444	.	19.855062
74195	NM_001253812,NM_028811,NR_045599	Elp3	elongator acetyltransferase complex subunit 3	protein_coding	NP_001240741.1;NP_083087.1	1921445	.	28.967331
74196	NM_152817,XM_006525025,XM_006525027,XR_003952200,XR_003952201	Ttc27	tetratricopeptide repeat domain 27	protein_coding	NP_690030.3;XP_006525088.1;XP_006525090.1	1921446	.	4.472429
74197	NM_001355727,NM_028812	Gtf2e1	general transcription factor II E, polypeptide 1 (alpha subunit)	protein_coding	NP_001342656.1;NP_083088.1	1921447	.	6.817407
74198	NM_001256096,NM_001256097,NM_001256098,NM_023742,XM_006504506,XM_006504507,XM_006504508,XM_006504510,XM_017321120,XM_030254848,XM_030254849,XM_030254850	Dtx2	deltex 2, E3 ubiquitin ligase	protein_coding	NP_001243025.1;NP_001243026.1;NP_001243027.1;NP_076231.1;XP_006504569.1;XP_006504570.1;XP_006504571.1;XP_006504573.1;XP_017176609.1;XP_030110708.1;XP_030110709.1;XP_030110710.	1921448	.	4.783776
74199	NM_001197028,NM_028813,XM_006525028,XM_006525029,XM_017317699	Vit	vitrin	protein_coding	NP_001183957.1;NP_083089.1;XP_006525091.1;XP_006525092.1;XP_017173188.1	1921449	.	10.083074
74200	NM_028814,NR_151825,NR_151826,NR_151827,XM_017319752,XR_003954405,XR_003954406,XR_375591	Khdc4	KH domain containing 4, pre-mRNA splicing factor	protein_coding	NP_083090.2;XP_017175241.1	1921450	.	32.488989
74201	NM_001159364,NM_001159365,NM_001159366,NM_028815	Cep97	centrosomal protein 97	protein_coding	NP_001152836.1;NP_001152837.1;NP_001152838.1;NP_083091.1	1921451	.	2.441042
74202	NM_001163256,NM_133754,XM_006539213,XM_006539214,XM_006539215,XM_006539216,XM_030253841	Fblim1	filamin binding LIM protein 1	protein_coding	NP_001156728.1;NP_598515.3;XP_006539276.1;XP_006539277.1;XP_006539278.1;XP_006539279.1;XP_030109701.1	1921452	.	5.254931
74203	NM_001166547,NM_001166548,NM_001166549,NM_023743,XM_006514841,XM_006514842,XM_006514843,XM_006514844,XM_017314806	Eif4enif1	eukaryotic translation initiation factor 4E nuclear import factor 1	protein_coding	NP_001160019.1;NP_001160020.1;NP_001160021.1;NP_076232.2;XP_006514904.1;XP_006514905.1;XP_006514906.1;XP_006514907.1;XP_017170295.1	1921453	.	16.343666
74204	NM_001311143,NM_028816,XM_006508250,XM_017312341,XM_017312342,XM_017312343,XM_017312345	Xpo6	exportin 6	protein_coding	NP_001298072.1;NP_083092.2;XP_006508313.1;XP_017167830.1;XP_017167831.1;XP_017167832.1;XP_017167834.1	2429950	.	15.517739
74205	NM_001033606,NM_001136222,NM_028817	Acsl3	acyl-CoA synthetase long-chain family member 3	protein_coding	NP_001028778.2;NP_001129694.1;NP_083093.2	1921455	.	12.932649
74206	NM_001081028,XM_006540397,XM_006540399,XM_006540400,XM_006540401,XM_006540402,XM_006540403,XM_006540404,XM_006540405,XM_011250727,XM_011250728,XM_011250729,XM_011250730,XM_011250731,XM_011250732,XM_017312346	Sipa1l3	signal-induced proliferation-associated 1 like 3	protein_coding	NP_001074497.1;XP_006540460.1;XP_006540462.1;XP_006540463.1;XP_006540464.1;XP_006540465.1;XP_006540466.1;XP_006540467.1;XP_006540468.1;XP_011249029.1;XP_011249030.1;XP_011249031.1;XP_011249032.1;XP_011249033.1;XP_011249034.1;XP_0171	1921456	.	4.056907
74211	NM_028820,XM_006511516	1700017B05Rik	RIKEN cDNA 1700017B05 gene	protein_coding	NP_083096.1;XP_006511579.1	1921461	.	3.882426
74213	NM_001368683,NM_001368684,NM_001368685,NM_134077,XM_030248057,XM_030248058,XM_030248059,XM_030248060,XM_030248061,XM_030248062,XM_030248063,XM_030248064,XM_030248065,XM_030248066,XM_030248067,XM_030248068,XM_030248069,XM_030248070,XM_030248071,XM_030248072,XM_030248073,XM_030248074,XM_030248075,XM_030248076,XM_030248077,XM_030248078	Rbm26	RNA binding motif protein 26	protein_coding	NP_001355612.1;NP_001355613.1;NP_001355614.1;NP_598838.3;XP_030103917.1;XP_030103918.1;XP_030103919.1;XP_030103920.1;XP_030103921.1;XP_030103922.1;XP_030103923.1;XP_030103924.1;XP_030103925.1;XP_030103926.1;XP_030103927.1;XP_030103928.1;XP_030103929.1;XP_030103930.1;XP_030103931.1;XP_030103932.1;XP_030103933.1;XP_030103934.1;XP_030103935.1;XP_030103936.1;XP_030103937.1;XP_030103938.1	1921463	.	8.584703
74218	NM_001163550,NM_028824,XM_006535258,XM_006535260,XM_006535261,XM_011249580,XM_011249581,XM_011249582,XM_017321121	1700016H13Rik	RIKEN cDNA 1700016H13 gene	protein_coding	NP_001157022.1;NP_083100.1;XP_006535321.1;XP_006535323.1;XP_006535324.1;XP_011247882.1;XP_011247883.1;XP_011247884.1;XP_017176610.1	1921468	.	0.053624
74221	NM_001355766,NM_028825,XM_030255636,XM_030255637	Tex37	testis expressed 37	protein_coding	NP_001342695.1;NP_083101.1;XP_030111496.1;XP_030111497.1	1921471	.	0.000000
74227	NR_151732	1700016A09Rik	RIKEN cDNA 1700016A09 gene	lncRNA	.	1921477	.	0.043276
74229	NM_001355122,NM_028829,XM_030243207	Paqr8	progestin and adipoQ receptor family member VIII	protein_coding	NP_001342051.1;NP_083105.3;XP_030099067.1	1921479	.	1.956441
74230	NM_198637,XM_006534355	1700016K19Rik	RIKEN cDNA 1700016K19 gene	protein_coding	NP_941039.1;XP_006534418.1	1921480	.	0.000000
74237	NM_001286007,NM_001286009,NM_001286011,NM_133755,XM_011249861,XM_030243026,XM_030243027	Tubgcp2	tubulin, gamma complex associated protein 2	protein_coding	NP_001272936.1;NP_001272938.1;NP_001272940.1;NP_598516.2;XP_011248163.1;XP_030098886.1;XP_030098887.1	1921487	.	14.120683
74238	NM_028832,XM_006514247,XM_006514248,XM_030245330	Mterf2	mitochondrial transcription termination factor 2	protein_coding	NP_083108.1;XP_006514310.1;XP_006514311.1;XP_030101190.1	1921488	.	2.083798
74239	NM_001347517,NM_028833,XM_006504739,XM_006504740,XM_006504741,XM_006504742,XM_006504744,XM_006504745,XM_006504746,XM_006504747,XM_006504748,XM_011240985,XM_030254852,XM_030254853,XM_030254854,XM_030254855,XM_030254856,XM_030254857,XM_030254858,XM_030254859	Iqce	IQ motif containing E	protein_coding	NP_001334446.1;NP_083109.2;XP_006504802.1;XP_006504803.1;XP_006504804.1;XP_006504805.1;XP_006504807.1;XP_006504808.1;XP_006504809.1;XP_006504810.1;XP_006504811.1;XP_011239287.1;XP_030110712.1;XP_030110713.1;XP_030110714.1;XP_030110715.1;XP_030110716.1;XP_030110717.1;XP_030110718.1;XP_030110	1921489	.	6.337529
74241	NM_001001565,NM_001001566	Chpf	chondroitin polymerizing factor	protein_coding	NP_001001565.1;NP_001001566.1	106576	.	27.557904
74243	NM_001038641,NM_001355669,NM_001355670,NM_001355671,NM_028201,NM_028834,NR_149746,XM_006500290,XM_011239811,XM_011239812,XM_011239813,XM_011239814,XM_030252134	Slx4ip	SLX4 interacting protein	protein_coding	NP_001033730.1;NP_001342598.1;NP_001342599.1;NP_001342600.1;NP_082477.1;NP_083110.1;XP_006500353.1;XP_011238113.1;XP_011238114.1;XP_011238115.1;XP_011238116.1;XP_030107994.1	1921493	.	1.588608
74244	NM_001253717,NM_001253718,NM_028835,XM_006506708,XM_006506709,XM_006506710,XM_011241494,XM_011241495,XM_030255638	Atg7	autophagy related 7	protein_coding	NP_001240646.1;NP_001240647.1;NP_083111.1;XP_006506771.1;XP_006506772.1;XP_006506773.1;XP_011239796.1;XP_011239797.1;XP_030111498.1	1921494	.	4.769091
74245	NM_001293672,NM_028836,XM_030252845	Ctbs	chitobiase	protein_coding	NP_001280601.1;NP_083112.1;XP_030108705.1	1921495	.	6.565828
74246	NM_001356493,XM_006539218,XM_006539219,XM_030253842,XM_030253843,XM_030253844,XR_003955031	Gale	galactose-4-epimerase, UDP	protein_coding	NP_001343422.1;XP_006539281.1;XP_006539282.1;XP_030109702.1;XP_030109703.1;XP_030109704.1	1921496	.	22.582332
74249	NM_028838,XM_006512364,XM_006512365	Lrrc2	leucine rich repeat containing 2	protein_coding	NP_083114.2;XP_006512427.1;XP_006512428.1	1921499	.	0.178361
74251	NM_001361916,NM_001361917,NM_175207,XM_006516294,XM_030246957,XM_030246958	Ankrd9	ankyrin repeat domain 9	protein_coding	NP_001348845.1;NP_001348846.1;NP_780416.2;XP_006516357.1;XP_030102817.1;XP_030102818.1	1921501	.	0.951422
74252	NM_001357689,NM_028840,XM_030252846	Armc1	armadillo repeat containing 1	protein_coding	NP_001344618.1;NP_083116.1;XP_030108706.1	1921502	.	9.761898
74253	NM_001033171,XM_006506711	Klrg2	killer cell lectin-like receptor subfamily G, member 2	protein_coding	NP_001028343.2;XP_006506774.1	1921503	.	0.290374
74254	NM_133756,XM_006504161,XM_006504162,XM_011240774	Gpn1	GPN-loop GTPase 1	protein_coding	NP_598517.1;XP_006504224.1;XP_006504225.1;XP_011239076.1	1921504	.	5.686397
74255	NM_021535	Smu1	smu-1 suppressor of mec-8 and unc-52 homolog (C. elegans)	protein_coding	NP_067510.3	1915546	.	14.856188
74256	NM_001128170,NM_001128171,NM_001276279,NM_173369,XM_006531414,XM_006531415,XM_006531416,XM_006531417,XM_006531418,XM_006531419,XM_006531420,XM_011248523,XM_011248524,XM_011248525,XM_017312989,XM_030243807,XM_030243808,XM_030243809,XM_030243810,XM_030243811,XM_030243812	Cyld	CYLD lysine 63 deubiquitinase	protein_coding	NP_001121642.1;NP_001121643.1;NP_001263208.1;NP_775545.1;XP_006531477.1;XP_006531478.1;XP_006531479.1;XP_006531480.1;XP_006531481.1;XP_006531482.1;XP_006531483.1;XP_011246825.1;XP_011246826.1;XP_011246827.1;XP_017168478.1;XP_030099667.1;XP_030099668.1;XP_030099669.1;XP_030099670.1;XP_030099671.1;XP_030099672.1	1921506	.	4.084409
74257	NM_028841,XM_006517414,XM_006517415,XM_006517417,XM_017315617,XM_017315618,XR_382461	Tspan17	tetraspanin 17	protein_coding	NP_083117.2;XP_006517477.1;XP_006517478.1;XP_006517480.1;XP_017171106.1;XP_017171107.1	1921507	.	7.289596
74264	NM_028842	Rnf138rt1	ring finger protein 138, retrogene 1	protein_coding	NP_083118.1	1921514	.	0.000000
74268	NM_001165935,NM_001355630,NM_001355631,NM_028844,NR_149741,XR_001783190	Aven	apoptosis, caspase activation inhibitor	protein_coding	NP_001159407.1;NP_001342559.1;NP_001342560.1;NP_083120.2	1921518	.	3.881681
74270	NM_028846,XM_030252135	Usp20	ubiquitin specific peptidase 20	protein_coding	NP_083122.1;XP_030107995.1	1921520	.	4.988003
74272	XR_383299	1700054O19Rik	RIKEN cDNA 1700054O19 gene	misc_RNA	.	1921522	.	0.013725
74276	NM_028849,XM_017312349,XM_017312350,XM_030243028,XM_030243029,XM_030243030,XM_030243031	Cldnd2	claudin domain containing 2	protein_coding	NP_083125.1;XP_017167838.1;XP_017167839.1;XP_030098888.1;XP_030098889.1;XP_030098890.1;XP_030098891.1	1921526	.	0.202475
74277	NM_028850	Chic2	cysteine-rich hydrophobic domain 2	protein_coding	NP_083126.1	1921527	.	7.027233
74284	NR_030733,NR_030734,NR_030735	1700086L19Rik	RIKEN cDNA 1700086L19 gene	lncRNA	.	1921534	.	0.588906
74287	NM_001347231,NM_019715,XM_030255640	Kcmf1	potassium channel modulatory factor 1	protein_coding	NP_001334160.1;NP_062689.2;XP_030111500.1	1921537	.	14.869233
74288	NM_028855	Spem1	sperm maturation 1	protein_coding	NP_083131.1	1921538	.	0.439967
74290	XR_869979,XR_869980	1700102J08Rik	RIKEN cDNA 1700102J08 gene, transcript variant X1	misc_RNA		1921540	.	0.058554
74293	NR_152110	1700095J03Rik	RIKEN cDNA 1700095J03 gene	lncRNA	.	1921543	.	0.066004
74302	NM_028860,XM_006514845,XM_006514846,XM_006514847,XM_006514848,XM_017314807,XR_003949519	Mtmr3	myotubularin related protein 3	protein_coding	NP_083136.2;XP_006514908.1;XP_006514909.1;XP_006514910.1;XP_006514911.1;XP_017170296.1	1921552	.	16.467250
74306	NM_183103	Prss46	protease, serine 46	protein_coding	NP_898926.2	1921556	.	1.166931
74307	NM_001177347	1700092M07Rik	RIKEN cDNA 1700092M07 gene	protein_coding	NP_001170818.1	1921557	.	0.183931
74309	NM_001302630,NM_001302631,NM_001363163,NM_001363164,NM_152818,XM_006514849,XM_006514851,XM_006514860,XM_011243763,XM_011243764,XM_011243765,XM_011243766,XM_011243767,XM_011243768,XM_011243769,XM_011243770,XM_011243771,XM_017314809,XM_030246382,XM_030246383,XM_030246384,XM_030246385,XM_030246386,XM_030246387,XM_030246388,XM_030246389,XM_030246390,XM_030246391	Osbp2	oxysterol binding protein 2	protein_coding	NP_001289559.1;NP_001289560.1;NP_001350092.1;NP_001350093.1;NP_690031.2;XP_006514912.1;XP_006514914.1;XP_006514923.1;XP_011242065.1;XP_011242066.1;XP_011242067.1;XP_011242068.1;XP_011242069.1;XP_011242070.1;XP_011242071.1;XP_011242072.1;XP_011242073.1;XP_017170298.1;XP_030102242.1;XP_030102243.1;XP_030102244.1;XP_030102245.1;XP_030102246.1;XP_030102247.1;XP_030102248.1;XP_030102249.1;XP_030102250.1;XP_03	1921559	.	0.380686
74315	NM_001166553,NM_001363166,NM_028862,XM_006534357,XM_006534358,XM_030246392	Rnf145	ring finger protein 145	protein_coding	NP_001160025.1;NP_001350095.1;NP_083138.2;XP_006534420.1;XP_006534421.1;XP_030102252.1	1921565	.	29.962340
74316	NM_028863,XM_006516296,XM_006516297,XM_006516298	Isca2	iron-sulfur cluster assembly 2	protein_coding	NP_083139.1;XP_006516359.1;XP_006516360.1;XP_006516361.1	1921566	.	18.866300
74318	NM_001159900,NM_001159901,NM_175606	Hopx	HOP homeobox	protein_coding	NP_001153372.1;NP_001153373.1;NP_783199.1	1916782	.	4.809266
74319	NM_028865,XM_006534360	Mettl23	methyltransferase like 23	protein_coding	NP_083141.3;XP_006534423.1	1921569	.	13.622199
74320	NM_001170966,NM_001170967,NM_001170970,NM_028866,XM_006526298,XM_006526300,XM_006526301,XM_017317996	Wdr33	WD repeat domain 33	protein_coding	NP_001164437.1;NP_001164438.1;NP_001164441.1;NP_083142.2;XP_006526361.1;XP_006526363.1;XP_006526364.1;XP_017173485.1	1921570	.	10.173403
74322	NM_028868,XM_006526302	Cxxc1	CXXC finger 1 (PHD domain)	protein_coding	NP_083144.1;XP_006526365.1	1921572	.	20.052944
74325	NM_001347512,NM_028870	Cltb	clathrin, light polypeptide (Lcb)	protein_coding	NP_001334441.1;NP_083146.1	1921575	.	14.655981
74326	NM_001277121,NM_001277122,NM_001277123,NM_001355182,NM_028871,XM_006539221,XM_006539222,XM_006539223,XM_006539224,XM_006539225,XM_006539226,XM_006539227,XM_006539228,XM_030253845,XM_030253846,XM_030253847,XM_030253848,XM_030253849,XM_030253850	Hnrnpr	heterogeneous nuclear ribonucleoprotein R	protein_coding	NP_001264050.1;NP_001264051.1;NP_001264052.1;NP_001342111.1;NP_083147.1;XP_006539284.1;XP_006539285.1;XP_006539286.1;XP_006539287.1;XP_006539288.1;XP_006539289.1;XP_006539290.1;XP_006539291.1;XP_030109705.1;XP_030109706.1;XP_030109707.1;XP_030109708.1;XP_030109709.1;XP_030109710.1	1891692	.	32.995788
74330	NM_001359824,NM_001359825,NM_028873,XM_006514251,XM_030245331,XM_030245332	Dnajc14	DnaJ heat shock protein family (Hsp40) member C14	protein_coding	NP_001346753.1;NP_001346754.1;NP_083149.3;XP_006514314.1;XP_030101191.1;XP_030101192.1	1921580	.	17.752692
74333	NR_015503	A330040F15Rik	RIKEN cDNA A330040F15 gene	lncRNA	.	3698434	.	0.000000
74334	NM_145824,XM_006531426,XM_006531428,XM_011248526,XM_030243813,XM_030243814,XM_030243815	Ranbp10	RAN binding protein 10	protein_coding	NP_665823.3;XP_006531489.1;XP_006531491.1;XP_011246828.1;XP_030099673.1;XP_030099674.1;XP_030099675.1	1921584	.	10.683410
74335	NM_028875,XM_006516300,XM_030246959	Xrcc3	X-ray repair complementing defective repair in Chinese hamster cells 3	protein_coding	NP_083151.1;XP_006516363.1;XP_030102819.1	1921585	.	1.137327
74337	NM_028877,XM_017312990,XM_030243816	Palm3	paralemmin 3	protein_coding	NP_083153.1;XP_017168479.1;XP_030099676.1	1921587	.	1.045373
74338	NM_001359603,NM_028878	Slc6a19	solute carrier family 6 (neurotransmitter transporter), member 19	protein_coding	NP_001346532.1;NP_083154.1	1921588	.	0.030033
74340	NM_001171000,NM_001171001,NM_001347084,NM_021414,XM_006505179,XM_006505180,XM_006505181,XM_006505182,XM_006505183,XM_011241082,XM_030255642,XM_030255643	Ahcyl2	S-adenosylhomocysteine hydrolase-like 2	protein_coding	NP_001164471.2;NP_001164472.1;NP_001334013.1;NP_067389.5;XP_006505242.2;XP_006505243.2;XP_006505244.1;XP_006505245.1;XP_006505246.1;XP_011239384.1;XP_030111502.1;XP_030111503.	1921590	.	6.270136
74341	NR_027913	G630025P09Rik	RIKEN cDNA G630025P09 gene	lncRNA	.	3696719	.	0.209505
74342	NM_001362109,NM_028880,NR_155299,NR_155300,XM_0302556	Lrrtm1	leucine rich repeat transmembrane neuronal 1	protein_coding	NP_001349038.1;NP_083156.2;XP_030111504.1	2389173	.	0.027523
74343	NM_001357152,NM_028881,NR_151631,XM_006502152,XM_006502153,XR_001783761,XR_001783762,XR_375593	Crtc2	CREB regulated transcription coactivator 2	protein_coding	NP_001344081.1;NP_083157.1;XP_006502215.1;XP_006502216.1	1921593	.	16.143883
74347	NM_028883,XM_030243817	Meak7	MTOR associated protein, eak-7 homolog	protein_coding	NP_083159.1;XP_030099677.1	1921597	.	2.661297
74349	NM_001242363,NM_001242364,NM_001242365,NM_199009,XM_006508251,XM_030243032,XM_030243033,XM_030243034,XM_030243035,XM_030243036,XM_030243037,XM_030243038,XR_001778008,XR_003946573,XR_378277	Fam160a2	family with sequence similarity 160, member A2	protein_coding	NP_001229292.1;NP_001229293.1;NP_001229294.1;NP_950174.1;XP_006508314.1;XP_030098892.1;XP_030098893.1;XP_030098894.1;XP_030098895.1;XP_030098896.1;XP_030098897.1;XP_030098898.1	1921599	.	3.030977
74351	NM_001080981,XM_006521493	Ddx23	DEAD (Asp-Glu-Ala-Asp) box polypeptide 23	protein_coding	NP_001074450.1;XP_006521556.1	1921601	.	24.488112
74352	NM_023750	Zfp84	zinc finger protein 84	protein_coding	NP_076239.1	107780	.	3.775483
74354	NM_028886,XM_006506713,XM_006506714,XM_011241496	Lrguk	leucine-rich repeats and guanylate kinase domain containing	protein_coding	NP_083162.1;XP_006506776.1;XP_006506777.1;XP_011239798.1	1921604	.	0.213706
74355	NM_028887,XM_030250119	Smchd1	SMC hinge domain containing 1	protein_coding	NP_083163.3;XP_030105979.1	1921605	.	10.546350
74356	NM_001166394,NM_028888,XM_006531433,XM_017312991	4931428F04Rik	RIKEN cDNA 4931428F04 gene	protein_coding	NP_001159866.1;NP_083164.1;XP_006531496.1;XP_017168480.1	1921606	.	3.419411
74359	NM_028890,XM_006519618,XM_006519619,XM_006519620,XM_011245210	4931414P19Rik	RIKEN cDNA 4931414P19 gene	protein_coding	NP_083166.1;XP_006519681.1;XP_006519682.1;XP_006519683.1;XP_011243512.1	1921609	.	1.821927
74360	NM_001310721,NM_026665,NR_037622,XM_006510649,XM_006510650,XM_017313667,XM_030244681,XM_030244682,XR_001779	Cep57	centrosomal protein 57	protein_coding	NP_001297650.1;NP_080941.3;XP_006510712.1;XP_006510713.1;XP_017169156.1;XP_030100541.1;XP_030100542.1	1915551	.	9.196485
74362	NM_028892	Spag17	sperm associated antigen 17	protein_coding	NP_083168.3	1921612	.	0.034511
74364	NR_045807	4931431C16Rik	RIKEN cDNA 4931431C16 gene	lncRNA	.	1921614	.	0.000000
74365	NM_028894,XM_006541404	Lonrf3	LON peptidase N-terminal domain and ring finger 3	protein_coding	NP_083170.1;XP_006541467.1	1921615	.	0.443086
74369	NM_001310435,NM_028897,XM_006521494,XM_006521495,XM_006521496,XM_017316770,XM_030248803	Mei1	meiotic double-stranded break formation protein 1	protein_coding	NP_001297364.1;NP_083173.2;XP_006521557.1;XP_006521558.1;XP_006521559.1;XP_017172259.1;XP_030104663.1	3028590	.	0.000000
74370	NM_001306081,NM_028898,XM_006534361,XM_006534362,XM_006534363,XM_011249285,XM_011249286,XM_030246393	Rptor	regulatory associated protein of MTOR, complex 1	protein_coding	NP_001293010.1;NP_083174.2;XP_006534424.1;XP_006534425.1;XP_006534426.1;XP_011247587.1;XP_011247588.1;XP_030102253.1	1921620	.	6.217356
74374	NM_001204229,NM_177562,XM_006522660,XM_006522661,XM_006522662,XM_006522663,XM_006522664,XM_006522665,XM_011246027,XM_017317148,XM_017317149,XM_030249311,XM_030249312,XM_030249313	Clec16a	C-type lectin domain family 16, member A	protein_coding	NP_001191158.1;NP_808230.2;XP_006522723.1;XP_006522724.1;XP_006522725.1;XP_006522726.1;XP_006522727.1;XP_006522728.1;XP_011244329.1;XP_017172637.1;XP_017172638.1;XP_030105171.1;XP_030105172.1;XP_030105173.1	1921624	.	4.124066
74375	NM_028900	Gcc1	golgi coiled coil 1	protein_coding	NP_083176.3	1921625	.	4.643930
74376	NM_028901,XM_006535262	Myo18b	myosin XVIIIb	protein_coding	NP_083177.2;XP_006535325.1	1921626	.	1.203135
74377	NM_028902,XM_006525035,XM_030250120	Hsf2bp	heat shock transcription factor 2 binding protein	protein_coding	NP_083178.1;XP_006525098.1;XP_030105980.1	1921627	.	0.161325
74383	NM_001165983,NM_001165984,NM_001165985,NM_001165986,NM_001165987,NM_001165988,NM_001357307,NM_028475,NM_153489,XM_006502156,XM_006502162,XM_006502169,XM_006502170,XM_006502172,XM_006502186,XM_011240246,XM_017319754,XM_017319755,XM_017319756,XM_017319758,XM_017319760,XM_017319762,XM_017319763,XM_017319767,XM_030252847,XM_030252848,XM_030252849,XM_030252850,XM_030252851,XM_030252852,XM_030252853,XM_030252854,XR_003954407,XR_003954408,XR_003954409,XR_003954410,XR_003954411,XR_003954412,XR_003954413,XR_003954414,XR_003954415,XR_0039544	Ubap2l	ubiquitin-associated protein 2-like	protein_coding	NP_001159455.1;NP_001159456.1;NP_001159457.1;NP_001159458.1;NP_001159459.1;NP_001159460.1;NP_001344236.1;NP_082751.1;NP_705693.2;XP_006502219.1;XP_006502225.1;XP_006502232.1;XP_006502233.1;XP_006502235.1;XP_006502249.1;XP_011238548.1;XP_017175243.1;XP_017175244.1;XP_017175245.1;XP_017175247.1;XP_017175249.1;XP_017175251.1;XP_017175252.1;XP_017175256.1;XP_030108707.1;XP_030108708.1;XP_030108709.1;XP_030108710.1;XP_030108711.1;XP_030108712.1;XP_030108713.1;XP_030108714.1	1921633	.	45.261383
74385	NM_001360070,NM_144535,XM_006519623,XM_006519624,XM_006519626,XM_011245212,XR_003950905	Ap5m1	adaptor-related protein complex 5, mu 1 subunit	protein_coding	NP_001346999.1;NP_653118.3;XP_006519686.1;XP_006519687.1;XP_006519689.1;XP_011243514.1	1921635	.	5.525375
74386	NM_001168248,NM_028904,NR_031761,XM_006517420	Rmi1	RecQ mediated genome instability 1	protein_coding	NP_001161720.1;NP_083180.3;XP_006517483.1	1921636	.	4.736487
74387	NM_001163695,NM_028905,XM_030249314,XM_030249315,XM_030249316,XM_030249317	4932438H23Rik	RIKEN cDNA 4932438H23 gene	protein_coding	NP_001157167.2;NP_083181.2;XP_030105174.1;XP_030105175.1;XP_030105176.1;XP_030105177.1	1921637	.	0.032305
74388	NM_028906,XM_006511517,XM_030244687,XR_003948041	Dpp8	dipeptidylpeptidase 8	protein_coding	NP_083182.2;XP_006511580.1;XP_030100547.1	1921638	.	28.965033
74392	NM_001145826,NM_153406,XM_006514252,XM_011243589,XM_017314127,XM_030245333	Specc1l	sperm antigen with calponin homology and coiled-coil domains 1-like	protein_coding	NP_001139298.1;NP_700455.3;XP_006514315.1;XP_011241891.1;XP_017169616.1;XP_030101193.1	1921642	.	13.461482
74393	NM_028908	Map10	microtubule-associated protein 10	protein_coding	NP_083184.1	1921643	.	1.005534
74396	NR_029443	4933407K13Rik	RIKEN cDNA 4933407K13 gene	lncRNA	.	1921646	.	2.903267
74399	XM_030245412	Duxf3	double homeobox family member 3	protein_coding	XP_030101272.1	1921649	.	0.000000
74400	NM_001355131,NM_028913,XM_017312353	Zfp819	zinc finger protein 819	protein_coding	NP_001342060.1;NP_083189.2;XP_017167842.1	1921650	.	0.024039
74403	NR_015540	4933400F21Rik	RIKEN cDNA 4933400F21 gene	lncRNA	.	1921653	.	0.000000
74405	NM_028916,XM_006527706,XM_017318631	Efhc2	EF-hand domain (C-terminal) containing 2	protein_coding	NP_083192.2;XP_006527769.1;XP_017174120.1	1921655	.	0.078807
74407	NM_001363174,NM_028918,XM_006534365,XR_388541	Ttc25	tetratricopeptide repeat domain 25	protein_coding	NP_001350103.1;NP_083194.2;XP_006534428.1	1921657	.	0.154326
74410	NM_029774	Ttll11	tubulin tyrosine ligase-like family, member 11	protein_coding	NP_084050.1	1921660	.	0.456500
74411	NM_028922	Plpp6	phospholipid phosphatase 6	protein_coding	NP_083198.1	1921661	.	3.055743
74412	NM_028923	Gle1	GLE1 RNA export mediator (yeast)	protein_coding	NP_083199.1	1921662	.	13.438382
74413	NM_001082976,NM_028924,XM_006516301,XM_006516303,XM_006516305,XM_006516306,XM_011244191,XM_011244192,XM_017315217,XR_001780494,XR_001780495	Tc2n	tandem C2 domains, nuclear	protein_coding	NP_001076445.1;NP_083200.1;XP_006516364.1;XP_006516366.1;XP_006516368.1;XP_006516369.1;XP_011242493.1;XP_011242494.1;XP_017170706.1	1921663	.	0.041525
74414	NM_028925,XM_006502187,XM_006502188,XM_030252855,XM_030252856,XR_375595,XR_867239	Polr3c	polymerase (RNA) III (DNA directed) polypeptide C	protein_coding	NP_083201.1;XP_006502250.1;XP_006502251.1;XP_030108715.1;XP_030108716.1	1921664	.	9.506873
74419	NM_001271574,NM_028927	Tktl2	transketolase-like 2	protein_coding	NP_001258503.1;NP_083203.2	1921669	.	0.000000
74424	NM_001105252,NM_028930,XM_006508253,XM_011241924,XM_030243039	Tmc5	transmembrane channel-like gene family 5	protein_coding	NP_001098722.1;NP_083206.1;XP_006508316.1;XP_011240226.1;XP_030098899.1	1921674	.	0.056211
74426	NM_001256158	4933402D24Rik	RIKEN cDNA 4933402D24 gene	protein_coding	NP_001243087.1	1921676	.	0.000000
74427	NM_028932	Eaf1	ELL associated factor 1	protein_coding	NP_083208.1	1921677	.	15.359417
74430	NM_028934,XM_006518106,XM_006518107,XM_011244772,XM_011244773,XM_030248079,XM_030248080,XM_030248081	4930452B06Rik	RIKEN cDNA 4930452B06 gene	protein_coding	NP_083210.3;XP_006518169.1;XP_006518170.1;XP_011243074.1;XP_011243075.1;XP_030103939.1;XP_030103940.1;XP_030103941.1	1926154	.	0.553052
74434	NM_028937	Sohlh2	spermatogenesis and oogenesis specific basic helix-loop-helix 2	protein_coding	NP_083213.2	1921684	.	0.000000
74435	NM_028938,XM_006502189,XM_006502190,XM_017319774	Lrriq3	leucine-rich repeats and IQ motif containing 3	protein_coding	NP_083214.2;XP_006502252.1;XP_006502253.1;XP_017175263.1	1921685	.	0.203318
74437	NM_001199996,NM_001199997,XM_006528321	4933402E13Rik	RIKEN cDNA 4933402E13 gene	protein_coding	NP_001186925.1;NP_001186926.1;XP_006528384.1	1921687	.	0.000000
74438	NM_001355183,NM_028940,XM_006538321,XM_006538322,XM_011250126,XM_030253851,XM_030253852	Clvs1	clavesin 1	protein_coding	NP_001342112.1;NP_083216.1;XP_006538384.1;XP_006538385.1;XP_011248428.1;XP_030109711.1;XP_030109712.1	1921688	.	0.005712
74440	NM_001163262,NM_028941,XM_006531434,XM_006531435,XM_006531436,XM_011248527	Cmip	c-Maf inducing protein	protein_coding	NP_001156734.1;NP_083217.1;XP_006531497.1;XP_006531498.1;XP_006531499.1;XP_011246829.2	1921690	.	11.794453
74442	NM_028943,XM_006502193,XM_006502194,XM_006502196,XM_006502197,XM_006502199,XM_011240249,XM_011240250	Sgms2	sphingomyelin synthase 2	protein_coding	NP_083219.2;XP_006502256.1;XP_006502257.1;XP_006502259.1;XP_006502260.1;XP_006502262.1;XP_011238551.1;XP_011238552.1	1921692	.	171.996448
74443	NM_001357465,NM_028944,NR_151688,XM_011242914	P4htm	prolyl 4-hydroxylase, transmembrane (endoplasmic reticulum)	protein_coding	NP_001344394.1;NP_083220.3;XP_011241216.1	1921693	.	2.310516
74446	NM_001358057,NM_028946,XM_006502201,XM_011240251,XM_011240252,XM_011240253,XM_017319775,XM_017319776,XR_867240	Slc9b1	solute carrier family 9, subfamily B (NHA1, cation proton antiporter 1), member 1	protein_coding	NP_001344986.1;NP_083222.2;XP_006502264.1;XP_011238553.1;XP_011238554.1;XP_011238555.1;XP_017175264.1;XP_017175265.1	1921696	.	0.090134
74450	NM_001355679,NM_001355681,NM_001355682,NM_001355683,NM_153501,NR_149747,NR_149748,XM_006500299,XM_017319299,XM_017319300,XM_030252136,XM_030252137,XM_030252138,XR_001783191,XR_001783192	Pank2	pantothenate kinase 2	protein_coding	NP_001342608.1;NP_001342610.1;NP_001342611.1;NP_001342612.1;NP_705721.2;XP_006500362.1;XP_017174788.1;XP_017174789.1;XP_030107996.1;XP_030107997.1;XP_030107998.1	1921700	.	11.778967
74451	NM_133757,NR_151726,XR_388542	Pgs1	phosphatidylglycerophosphate synthase 1	protein_coding	NP_598518.1	1921701	.	11.466620
74453	NM_028948,NR_029424,XM_011247012	Cfap53	cilia and flagella associated protein 53	protein_coding	NP_083224.2;XP_011245314.1	1921703	.	0.058224
74454	NR_045810	4933408J17Rik	RIKEN cDNA 4933408J17 gene	lncRNA	.	1921704	.	0.000000
74455	NM_001165941,NM_001165942,NM_001165943,NM_001355685,NM_028950,XM_030252139,XM_030252140,XM_030252141,XM_030252142,XM_030252143,XM_030252144,XM_030252145,XM_030252146,XM_030252147,XM_030252148	Nsun6	NOL1/NOP2/Sun domain family member 6	protein_coding	NP_001159413.1;NP_001159414.1;NP_001159415.1;NP_001342614.1;NP_083226.3;XP_030107999.1;XP_030108000.1;XP_030108001.1;XP_030108002.1;XP_030108003.1;XP_030108004.1;XP_030108005.1;XP_030108006.1;XP_030108007.1;XP_030108008.1	1921705	.	1.703458
74456	NR_045372	4933424G05Rik	RIKEN cDNA 4933424G05 gene	lncRNA	.	1921706	.	0.000000
74463	NM_001370800	Exoc3l2	exocyst complex component 3-like 2	protein_coding	NP_001357729.1	1921713	.	2.100366
74464	NM_001029912	Zswim5	zinc finger SWIM-type containing 5	protein_coding	NP_001025083.1	1921714	.	0.282608
74466	NM_028955,XM_006508254,XM_006508255,XM_006508256,XM_006508257,XM_006508258,XM_006508259,XM_006508261,XM_006508262,XM_006508263,XM_006508264,XM_006508265,XM_006508266,XM_006508267,XM_006508268,XM_006508269,XM_006508270,XM_017312355,XM_030243040,XM_030243041,XM_030243042	Mfsd13b	major facilitator superfamily domain containing 13B	protein_coding	NP_083231.1;XP_006508317.1;XP_006508318.1;XP_006508319.1;XP_006508320.1;XP_006508321.1;XP_006508322.1;XP_006508324.1;XP_006508325.1;XP_006508326.1;XP_006508327.1;XP_006508328.1;XP_006508329.1;XP_006508330.1;XP_006508331.1;XP_006508332.1;XP_006508333.1;XP_017167844.1;XP_030098900.1;XP_030098901.1;XP_030098902.1	1921716	.	0.196323

74467	NM_001033654,NM_028304,NM_028956,NR_156455,XM_006514861,XM_006514862,XM_006514863,XM_006514864,XM_006514865,XM_006514866,XM_030246394,XM_030246395,XM_030246396,XM_030246397,XM_030246398,XM_030246399,XM_030246400,XM_030246401,XR_003949520,XR_003949521,XR_003949522,XR_003949523	Pus10	pseudouridylate synthase 10	protein_coding	NP_001028826.1;NP_082580.1;NP_083232.1;XP_006514924.1;XP_006514925.1;XP_006514926.1;XP_006514927.1;XP_006514928.2;XP_006514929.1;XP_030102254.1;XP_030102255.1;XP_030102256.1;XP_030102257.1;XP_030102258.1;XP_030102259.1;XP_030102260.1;XP_030102261.1	1921717	.	5.742242	[illegible]
74469	NM_028958,XM_006528610,XM_006528611,XM_006528613,XM_006528615	Taf7l	TATA-box binding protein associated factor 7 like	protein_coding	NP_083234.2;XP_006528673.1;XP_006528674.1;XP_006528676.1;XP_006528678.1	1921719	.	0.000000	[illegible]
74470	NM_028959,XM_006517422,XM_006517423,XM_006517424,XM_006517425,XM_011244576,XM_030247437,XM_030247438	Cep72	centrosomal protein 72	protein_coding	NP_083235.3;XP_006517485.1;XP_006517486.1;XP_006517487.1;XP_006517488.1;XP_011242878.1;XP_030103297.1;XP_030103298.1	1921720	.	1.846403	[illegible]
74471	XM_017321877	4933440N22Rik	RIKEN cDNA 4933440N22 gene	protein_coding	XP_017177366.1	1921721	.	0.330820	[illegible]
74473	NR_045458	4933433H22Rik	RIKEN cDNA 4933433H22 gene	lncRNA	.	1921723	.	0.113404	[illegible]
74476	NR_015585,NR_135273	4933439C10Rik	RIKEN cDNA 4933439C10 gene	lncRNA	.	1921726	.	9.461017	[illegible]
74477	NM_001361564,NM_028963,NR_155065,NR_155066,NR_155067,XM_006534367,XM_006534369,XM_006534370,XM_006534372,XM_006534373,XM_017314813,XM_030246402,XR_001780070,XR_001780071,XR_003949524,XR_388546	4933427D14Rik	RIKEN cDNA 4933427D14 gene	protein_coding	NP_001348493.1;NP_083239.1;XP_006534430.1;XP_006534432.1;XP_006534433.1;XP_006534435.1;XP_006534436.1;XP_017170302.1;XP_030102262.1	1921727	.	4.442360	[illegible]
74478	NM_001290148,NM_028964,XM_006522666,XM_006522668,XM_006522669,XM_006522670,XM_006522672,XM_030249318,XM_030249319,XM_030249320,XM_030249321	Snx29	sorting nexin 29	protein_coding	NP_001277077.1;NP_083240.2;XP_006522729.1;XP_006522731.1;XP_006522732.1;XP_006522733.1;XP_006522735.1;XP_030105178.1;XP_030105179.1;XP_030105180.1;XP_030105181.1	1921728	.	3.669942	[illegible]
74479	NM_001163389,NM_028965,XM_006534375,XM_011249289	Snx11	sorting nexin 11	protein_coding	NP_001156861.1;NP_083241.1;XP_006534438.1;XP_011247591.1	1921729	.	6.171099	[illegible]
74480	NM_001037221,NM_001163433,NM_001310544,NM_028966,XM_006519627,XM_006519628,XM_006519631,XM_030248082	Samd4	sterile alpha motif domain containing 4	protein_coding	NP_001032298.1;NP_001156905.1;NP_001297473.1;NP_083242.1;XP_006519690.1;XP_006519691.1;XP_006519694.1;XP_030103942.1	1921730	.	10.699113	[illegible]
74481	NM_001356604,NM_028967,XM_006531852,XM_017318301	Batf2	basic leucine zipper transcription factor, ATF-like 2	protein_coding	NP_001343533.1;NP_083243.1;XP_006531915.1;XP_017173790.1	1921731	.	0.387814	[illegible]
74485	NM_028971,XM_006502207,XM_011240257,XM_017319777	Lrrc71	leucine rich repeat containing 71	protein_coding	NP_083247.1;XP_006502270.1;XP_011238559.1;XP_017175266.1	1921735	.	0.159281	[illegible]
74486	NM_148958,XM_006512371,XM_006512372,XM_006512373,XM_006512374,XM_006512376,XM_006512377,XM_011243051,XM_011243052,XR_003948042	Osbpl10	oxysterol binding protein-like 10	protein_coding	NP_683761.2;XP_006512434.1;XP_006512435.1;XP_006512436.1;XP_006512437.1;XP_006512439.1;XP_006512440.1;XP_011241353.1;XP_011241354.1	1921736	.	1.593884	[illegible]
74487	NR_015591,NR_027914	5430405H02Rik	RIKEN cDNA 5430405H02 gene	lncRNA	.	1921737	.	1.074306	[illegible]
74488	NM_028973	Lrrc15	leucine rich repeat containing 15	protein_coding	NP_083249.1	1921738	.	51.169453	[illegible]
74489	NR_040352	5430421F17Rik	RIKEN cDNA 5430421F17 gene	lncRNA	.	1921739	.	1.074402	[illegible]
74490	NM_172418,XM_006541232,XM_006541233,XM_006541234,XM_006541235	Mamstr	MEF2 activating motif and SAP domain containing transcriptional regulator	protein_coding	NP_766006.2;XP_006541295.1;XP_006541296.1;XP_006541297.1;XP_006541298.1	1921740	.	4.347758	[illegible]
74492	NM_028974	Kbtbd13	kelch repeat and BTB (POZ) domain containing 13	protein_coding	NP_083250.1	1921742	.	0.422794	[illegible]
74493	NM_001163635,XM_006527408,XM_006527409,XM_011247382	Tnks2	tankyrase, TRF1-interacting ankyrin-related ADP-ribose polymerase 2	protein_coding	NP_001157107.1;XP_006527471.1;XP_006527472.1;XP_011245684.	1921743	.	33.238670	[illegible]
74498	NM_028976	Gorasp1	golgi reassembly stacking protein 1	protein_coding	NP_083252.1	1921748	.	9.987635	[illegible]
74499	NM_024449,XM_006534376	Sost	sclerostin	protein_coding	NP_077769.4;XP_006534439.1	1921749	.	37.821392	[illegible]
74504	NM_001359461,NM_001359462,NM_001359463,NM_178390,XM_011240776	Fam53a	family with sequence similarity 53, member A	protein_coding	NP_001346390.1;NP_001346391.1;NP_001346392.1;NP_848477.2;XP_011239078.1	1919225	.	10.106398	[illegible]
74511	NM_028977	Lrrc17	leucine rich repeat containing 17	protein_coding	NP_083253.1	1921761	.	69.706322	[illegible]
74513	NM_001081324,NM_001357287,NM_001357288,NR_151665,XM_006531437,XM_030243818,XM_030243819	Neto2	neuropilin (NRP) and tolloid (TLL)-like 2	protein_coding	NP_001074793.1;NP_001344216.1;NP_001344217.1;XP_006531500.1;XP_030099678.1;XP_030099679.1	1921763	.	1.760145	[illegible]
74516	XR_001785329,XR_003956471,XR_003956472,XR_003956473,XR_003956474,XR_035139	A730049H05Rik	RIKEN cDNA A730049H05 gene, transcript variant X1	misc_RNA	.	1921766	.	0.600068	[illegible]
74518	XR_001780165,XR_003949854,XR_003949855,XR_003949856,XR_003949857	8430419K02Rik	RIKEN cDNA 8430419K02 gene, transcript variant X4	lncRNA	.	1921768	.	0.000000	[illegible]
74519	NM_028979,XM_006503444,XM_030253853	Cyp2j9	cytochrome P450, family 2, subfamily j, polypeptide 9	protein_coding	NP_083255.1;XP_006503507.1;XP_030109713.1	1921769	.	0.426492	[illegible]
74521	NM_028980,XM_006516308,XM_006516314,XM_006516315,XM_011244194,XM_030246960,XM_030246961,XM_030246962,XM_030246963,XR_001780496,XR_003950088,XR_381552	Ppp4r4	protein phosphatase 4, regulatory subunit 4	protein_coding	NP_083256.2;XP_006516371.1;XP_006516377.1;XP_006516378.1;XP_011242496.1;XP_030102820.1;XP_030102821.1;XP_030102822.1;XP_030102823.1	1921771	.	2.143939	[illegible]
74522	NM_001159288,NM_001363202,NM_198162,XM_006514871,XM_011243775	Morc2a	microrchidia 2A	protein_coding	NP_001152760.1;NP_001350131.1;NP_937805.1;XP_006514934.1;XP_011242077.1	1921772	.	16.948875	[illegible]
74525	NM_001362110,NM_001362111,NM_028982,XM_006506715,XM_030255645,XM_030255646,XM_030255647	Fam234b	family with sequence similarity 234, member B	protein_coding	NP_001349039.1;NP_001349040.1;NP_083258.2;XP_006506778.1;XP_030111505.1;XP_030111506.1;XP_030111507.1	1921775	.	5.622303	[illegible]
74528	NM_001289630,NM_001289631,NM_001355688,NM_028984	Mgme1	mitochondrial genome maintenance exonuclease 1	protein_coding	NP_001276559.1;NP_001276560.1;NP_001342617.1;NP_083260.1	1921778	.	2.545873	[illegible]
74530	NR_102361	9030612E09Rik	RIKEN cDNA 9030612E09 gene	lncRNA	.	1921780	.	0.041553	[illegible]
74533	NM_001355689,NM_028986,XM_006500305,XR_003953729,XR_003953730	Gzf1	GDNF-inducible zinc finger protein 1	protein_coding	NP_001342618.1;NP_083262.1;XP_006500368.1	1921783	.	6.781717	[illegible]
74548	NM_028992,XM_006521502,XM_011245754,XM_011245755,XM_011245756,XM_011245757	Gsdmc4	gasdermin C4	protein_coding	NP_083268.1;XP_006521565.1;XP_011244056.1;XP_011244057.1;XP_011244058.1;XP_011244059.1	1921798	.	0.000000	[illegible]
74549	NM_001167939,NM_028993,XM_006509776,XR_870466	Mau2	MAU2 sister chromatid cohesion factor	protein_coding	NP_001161411.1;NP_083269.4;XP_006509839.1	1921799	.	15.671766	[illegible]
74551	NM_028994,XM_030248083,XM_030248084,XM_030248085	Pck2	phosphoenolpyruvate carboxykinase 2 (mitochondrial)	protein_coding	NP_083270.2;XP_030103943.1;XP_030103944.1;XP_030103945.1	1860456	.	16.087664	[illegible]
74552	NM_001347220,NM_001361484,NM_028995,XM_006539230,XM_006539231,XM_030253854,XM_030253855,XR_003955032	Nipal3	NIPA-like domain containing 3	protein_coding	NP_001334149.1;NP_001348413.1;NP_083271.1;XP_006539293.1;XP_006539294.1;XP_030109714.1;XP_030109715.1	1921802	.	1.030069	[illegible]
74556	NM_028998	Themis3	thymocyte selection associated family member 3	protein_coding	NP_083274.1	1921806	.	0.000000	[illegible]
74558	NM_001039160,NM_029000,XM_017312356	Gvin1	GTPase, very large interferon inducible 1	protein_coding	NP_001034249.2;NP_083276.2;XP_017167845.1	1921808	.	0.638621	[illegible]
74559	NM_029001,XM_006517778	Elovl7	ELOVL family member 7, elongation of long chain fatty acids (yeast)	protein_coding	NP_083277.3;XP_006517841.1	1921809	.	0.232128	[illegible]
74561	NM_029002	Nkx6-3	NK6 homeobox 3	protein_coding	NP_083278.1	1921811	.	0.000000	[illegible]
74562	NM_001136556,XM_006500307,XM_006500308,XM_006500309,XR_003953731	Fer1l4	fer-1-like 4 (C. elegans)	protein_coding	NP_001130028.1;XP_006500370.1;XP_006500371.1;XP_006500372.1	1921812	.	0.000000	[illegible]
74563	NM_001347462,NM_029004,XM_006534378,XM_030246404,XM_030246405	Rasgef1c	RasGEF domain family, member 1C	protein_coding	NP_001334391.1;NP_083280.1;XP_006534441.2;XP_030102264.1;XP_030102265.1	1921813	.	0.000000	[illegible]
74568	NM_001310613,NM_029005,XM_006531442,XM_006531443,XM_030243820,XR_003947349,XR_003947350	Mlkl	mixed lineage kinase domain-like	protein_coding	NP_001297542.1;NP_083281.1;XP_006531505.1;XP_006531506.1;XP_030099680.1	1921818	.	1.455882	[illegible]
74569	NM_001355690,NM_001355691,NM_183106,XM_006500310,XM_006500312	Ttc17	tetratricopeptide repeat domain 17	protein_coding	NP_001342619.1;NP_001342620.1;NP_898929.2;XP_006500373.1;XP_006500375.1	1921819	.	8.487127	[illegible]
74570	NM_029869,NM_133906,XM_006504628,XM_011240958	Zkscan1	zinc finger with KRAB and SCAN domains 1	protein_coding	NP_084145.1;NP_598667.2;XP_006504691.1;XP_011239260.1	1921820	.	5.096907	[illegible]
74571	NM_029006	Kcnk16	potassium channel, subfamily K, member 16	protein_coding	NP_083282.1	1921821	.	0.000000	[illegible]
74574	NM_029008,XM_017317997,XM_017317998,XM_017317999,XM_017318000	Lvrn	laeverin	protein_coding	NP_083284.1;XP_017173486.1;XP_017173487.1;XP_017173488.1;XP_017173489.1	1921824	.	2.384737	[illegible]
74577	NM_029010,XM_006496553,XM_006496554,XM_006496555,XM_011238713,XM_017312537,XM_030243213,XM_030243215,XR_003947211,XR_373546,XR_373547	Glb1l	galactosidase, beta 1-like	protein_coding	NP_083286.1;XP_006496616.1;XP_006496617.1;XP_006496618.1;XP_011237015.1;XP_017168026.1;XP_030099073.1;XP_030099075.1	1921827	.	8.144783	[illegible]
74580	NM_029011,XM_011247383,XR_003952824,XR_877691	Pyroxd2	pyridine nucleotide-disulphide oxidoreductase domain 2	protein_coding	NP_083287.2;XP_011245685.1	1921830	.	8.566051	[illegible]
74585	NM_029012,XM_017321123	Sppl3	signal peptide peptidase 3	protein_coding	NP_083288.2;XP_017176612.1	1891433	.	22.014499	[illegible]
74589	NM_001278671,XM_030255648	Kbtbd12	kelch repeat and BTB (POZ) domain containing 12	protein_coding	NP_001265600.1;XP_030111508.1	1918481	.	0.046422	[illegible]
74591	NM_175210	Abca12	ATP-binding cassette, sub-family A (ABC1), member 12	protein_coding	NP_780419.2	2676312	.	0.000000	[illegible]
74596	NM_173370,XM_006535263,XM_006535264	Cds1	CDP-diacylglycerol synthase 1	protein_coding	NP_775546.2;XP_006535326.1;XP_006535327.1	1921846	.	0.882556	[illegible]
74597	NR_015506	4833418N02Rik	RIKEN cDNA 4833418N02 gene	lncRNA	.	1921847	.	3.359178	[illegible]
74600	NM_029017	Mrpl47	mitochondrial ribosomal protein L47	protein_coding	NP_083293.1	1921850	.	8.642859	[illegible]
74601	NR_045459	4833427F10Rik	RIKEN cDNA 4833427F10 gene	lncRNA	.	1921851	.	0.000000	[illegible]
74603	NM_001128132,NM_001128133,NM_027578,NM_029018,XM_006522674,XM_011246030,XM_011246031,XM_011246032,XM_011246033,XM_011246035,XM_017317152,XM_017317153,XM_017317154	Cd200r3	CD200 receptor 3	protein_coding	NP_001121604.1;NP_001121605.1;NP_081854.1;NP_083294.2;XP_006522737.1;XP_011244332.1;XP_011244333.1;XP_011244334.1;XP_011244335.1;XP_011244337.1;XP_017172641.1;XP_017172642.1;XP_017172643.1	1921853	.	0.453891	[illegible]
74610	NM_029020,XM_006535812,XM_011249790	Abcb8	ATP-binding cassette, sub-family B (MDR/TAP), member 8	protein_coding	NP_083296.2;XP_006535875.1;XP_011248092.1	1351667	.	7.724901	[illegible]
74614	NM_029021	Ocstamp	osteoclast stimulatory transmembrane protein	protein_coding	NP_083297.1	1921864	.	4.867489	[illegible]
74616	NM_001355711,NM_029022,XM_006500313,XM_006500314,XM_011239820,XM_030252151,XR_866344	Scrn3	secernin 3	protein_coding	NP_001342640.1;NP_083298.1;XP_006500376.1;XP_006500377.1;XP_011238122.1;XP_030108011.1	1921866	.	4.695192	[illegible]
74617	NM_029023	Scpep1	serine carboxypeptidase 1	protein_coding	NP_083299.3	1921867	.	45.516682	[illegible]
74624	NR_040699	4930429D17Rik	RIKEN cDNA 4930429D17 gene	lncRNA	.	1921874	.	0.000000	[illegible]
74626	NM_029025,XM_030243219	Tmem81	transmembrane protein 81	protein_coding	NP_083301.1;XP_030099079.1	1921876	.	1.738473	[illegible]
74629	NR_024323	4930426L09Rik	RIKEN cDNA 4930426L09 gene	lncRNA	.	1921879	.	0.169673	[illegible]
74633	NR_040734	4930429F24Rik	RIKEN cDNA 4930429F24 gene	lncRNA	.	1921883	.	0.000000	[illegible]
74637	NM_029031,XM_006534380,XR_003949525	Shpk	sedoheptulokinase	protein_coding	NP_083307.1;XP_006534443.1	1921887	.	1.510563	[illegible]
74644	NM_001271580,XM_030250621	4930426D05Rik	RIKEN cDNA 4930426D05 gene	protein_coding	NP_001258509.1;XP_030106481.1	1921894	.	0.000000	[illegible]
74645	NM_001142952,XM_006502212	Tent5c	terminal nucleotidyltransferase 5C	protein_coding	NP_001136424.1;XP_006502275.1	1921895	.	6.024089	[illegible]
74646	NM_029035,XM_006539234,XM_011250344,XM_011250345	Spsb1	splA/ryanodine receptor domain and SOCS box containing 1	protein_coding	NP_083311.1;XP_006539297.1;XP_011248646.1;XP_011248647.1	1921896	.	5.859472	[illegible]
74647	NR_152796	4930432O09Rik	RIKEN cDNA 4930432O09 gene	lncRNA	.	1921897	.	0.000000	[illegible]
74648	NM_001347203,NM_001361317,NM_029036,XM_006503445,XM_006503446,XM_006503448,XM_006503449,XM_006503450,XM_006503451,XM_006503452,XM_011240623,XM_030253856,XM_030253857,XM_030253858,XM_030253859,XM_030253860	S100pbp	S100P binding protein	protein_coding	NP_001334132.1;NP_001348246.1;NP_083312.1;XP_006503508.1;XP_006503509.1;XP_006503511.1;XP_006503512.1;XP_006503513.1;XP_006503514.1;XP_006503515.1;XP_011238925.1;XP_030109716.1;XP_030109717.1;XP_030109718.1;XP_030109719.1;XP_03010972	1921898	.	13.789865	[illegible]
74649	NM_144537,XM_006505188,XM_006505190,XM_011241084,XM_017321795	Cpa5	carboxypeptidase A5	protein_coding	NP_653120.1;XP_006505251.1;XP_006505253.1;XP_011239386.1;XP_017177284.1	1921899	.	0.000000	[illegible]
74653	NM_029037	Pomk	protein-O-mannose kinase	protein_coding	NP_083313.1	1921903	.	4.720139	[illegible]
74665	NM_029044,XM_006534382	Drc3	dynein regulatory complex subunit 3	protein_coding	NP_083320.1;XP_006534445.1	1921915	.	0.573276	[illegible]
74666	NM_001163752,NM_029045,XM_006531445,XM_030243821,XM_030243822	4930432K21Rik	RIKEN cDNA 4930432K21 gene	protein_coding	NP_001157224.1;NP_083321.2;XP_006531508.1;XP_030099681.1;XP_030099682.1	1921916	.	0.265704	[illegible]
74670	NM_001025373	Zfp943	zinc finger prtoein 943	protein_coding	NP_001020544.1	1921920	.	6.943470	[illegible]
74675	NM_029049,XM_006534383	Ptchd3	patched domain containing 3	protein_coding	NP_083325.1;XP_006534446.1	1921925	.	0.014632	[illegible]
74680	NR_153406	4930435F18Rik	RIKEN cDNA 4930435F18 gene	lncRNA	.	1921930	.	0.000000	[illegible]
74682	NM_001159527,NM_172470,XM_006515232	Wdr35	WD repeat domain 35	protein_coding	NP_001152999.1;NP_766058.3;XP_006515295.1	1921932	.	7.875452	[illegible]
74684	NR_073370	4930451G09Rik	RIKEN cDNA 4930451G09 gene	misc_RNA	.	1921934	.	0.144397	[illegible]
74685	NM_001145435,NM_029053,XM_006522677,XM_011246036,XM_011246037,XR_875856	Lrrc74b	leucine rich repeat containing 74B	protein_coding	NP_001138907.1;NP_083329.2;XP_006522740.1;XP_011244338.1;XP_011244339.1	1921935	.	0.388330	[illegible]
74687	NR_040504	4930443O20Rik	RIKEN cDNA 4930443O20 gene	lncRNA	.	1921937	.	0.070217	[illegible]
74691	NM_029056,XM_006516316,XM_006516317,XM_006516319,XM_006516320,XM_006516321,XM_006516322,XM_017315218,XR_003950089,XR_381553	Tdrd9	tudor domain containing 9	protein_coding	NP_083332.1;XP_006516379.1;XP_006516380.1;XP_006516382.1;XP_006516383.1;XP_006516384.1;XP_006516385.1;XP_017170707.1	1921941	.	0.266316	[illegible]
74694	NM_001361015,NM_029057,XM_006514253,XM_006514254,XM_030245334	Tbc1d30	TBC1 domain family, member 30	protein_coding	NP_001347944.1;NP_083333.1;XP_006514316.1;XP_006514317.1;XP_030101194.1	1921944	.	0.210196	[illegible]
74706	NR_027926	4930507D05Rik	RIKEN cDNA 4930507D05 gene	lncRNA	.	1921956	.	0.011786	[illegible]
74711	NM_001083618,NM_001355717,NM_001355719,NM_001355720,NM_029064,XM_006500320,XM_011239821,XM_030252152,XM_030252153,XM_030252154,XM_030252155,XM_030252156,XM_030252157,XM_030252158,XM_030252159,XM_030252160,XM_030252161	Ttll9	tubulin tyrosine ligase-like family, member 9	protein_coding	NP_001077087.1;NP_001342646.1;NP_001342648.1;NP_001342649.1;NP_083340.2;XP_006500383.1;XP_011238123.1;XP_030108012.1;XP_030108013.1;XP_030108014.1;XP_030108015.1;XP_030108016.1;XP_030108017.1;XP_030108018.1;XP_030108019.1;XP_030108020.1;XP_030108021.1	1913589	.	0.147205	[illegible]
74716	NM_029066,XM_017316772	Wbp2nl	WBP2 N-terminal like	protein_coding	NP_083342.1;XP_017172261.1	1921966	.	0.000000	[illegible]
74717	NM_028848,NM_029067,XM_006497207,XM_006497210,XM_030243223,XM_030243227	Spata17	spermatogenesis associated 17	protein_coding	NP_083124.1;NP_083343.1;XP_006497270.1;XP_006497273.1;XP_030099083.1;XP_030099087.1	1921967	.	0.028709	[illegible]
74718	NM_001127191,NM_029068,XR_003954417	Snx16	sorting nexin 16	protein_coding	NP_001120663.1;NP_083344.3	1921968	.	2.791693	[illegible]
74720	NM_029070	Tmem114	transmembrane protein 114	protein_coding	NP_083346.1	1921970	.	0.000000	[illegible]
74724	NR_033573	4930512B01Rik	RIKEN cDNA 4930512B01 gene	lncRNA	.	1921974	.	0.000000	[illegible]
74729	NM_001276356,NM_178391	Setmar	SET domain without mariner transposase fusion	protein_coding	NP_001263285.1;NP_848478.2	1921979	.	3.324096	[illegible]
74732	NM_001163590,NM_001163591,NM_029075,XM_006512886,XM_006512887,XM_006512888	Stx11	syntaxin 11	protein_coding	NP_001157062.1;NP_001157063.1;NP_083351.1;XP_006512949.1;XP_006512950.1;XP_006512951.1	1921982	.	1.029928	[illegible]
74734	NM_001081105,NM_001363454,XM_011240779,XM_030254861,XM_030254862	Rhoh	ras homolog family member H	protein_coding	NP_001074574.1;NP_001350383.1;XP_011239081.1;XP_030110721.1;XP_030110722.1	1921984	.	0.099925	[illegible]
74735	NM_029077,XM_017320419,XM_017320420,XM_017320421,XM_030253861	Trim14	tripartite motif-containing 14	protein_coding	NP_083353.1;XP_017175908.1;XP_017175909.1;XP_017175910.1;XP_030109721.1	1921985	.	0.865711	[illegible]
74737	NM_029078,XM_006508272	Pcf11	PCF11 cleavage and polyadenylation factor subunit	protein_coding	NP_083354.3;XP_006508335.1	1919579	.	11.165059	[illegible]
74741	NM_001109688,NM_001286578,NM_001362113,NM_001362114,NM_001362115,NM_001362116,NM_001362117,NM_001362118,NM_029081,NM_029897,XM_006506997,XM_006507009,XM_017321797,XM_017321798,XM_017321799,XM_017321800,XM_017321801,XM_017321802,XM_017321804,XM_017321807,XM_017321809,XM_017321813,XM_030255649,XR_001785180,XR_001785182	C2cd5	C2 calcium-dependent domain containing 5	protein_coding	NP_001103158.1;NP_001273507.1;NP_001349042.1;NP_001349043.1;NP_001349044.1;NP_001349045.1;NP_001349046.1;NP_001349047.1;NP_083357.2;NP_084173.1;XP_006507060.1;XP_006507072.1;XP_017177286.1;XP_017177287.1;XP_017177288.1;XP_017177289.1;XP_017177290.1;XP_017177291.1;XP_017177293.1;XP_017177296.1;XP_017177298.1;XP_017177302.1;XP_030111509.1	1921991	.	9.607161	[illegible]
74743	XR_870010	5830403F22Rik	RIKEN cDNA 5830403F22 gene	lncRNA	.	1921993	.	0.210066	[illegible]
74744	NR_155282	5830408C22Rik	RIKEN cDNA 5830408C22 gene	lncRNA	.	1921994	.	0.191442	[illegible]
74747	NM_029083	Ddit4	DNA-damage-inducible transcript 4	protein_coding	NP_083359.1	1921997	.	24.275417	[illegible]
74748	NM_029084,XM_006497009,XM_030243230	Slamf8	SLAM family member 8	protein_coding	NP_083360.2;XP_006497072.1;XP_030099090.1	1921998	.	0.000000	[illegible]
74753	NM_001347095,NM_029086,XM_017320422,XM_017320423,XR_390347	Trmo	tRNA methyltransferase O	protein_coding	NP_001334024.1;NP_083362.1;XP_017175911.1;XP_017175912.1	1922003	.	2.498702	[illegible]
74754	NM_053272	Dhcr24	24-dehydrocholesterol reductase	protein_coding	NP_444502.2	1922004	.	4.865663	[illegible]
74757	NR_045384	5830416I19Rik	RIKEN cDNA 5830416I19 gene	lncRNA	.	1922007	.	0.000000	[illegible]
74760	NM_001347429,NM_001347430,NM_144538,NR_153790,XM_006527411,XM_006527412,XM_006527414,XM_006527417,XM_011247385,XM_030251119,XM_030251120,XM_030251121,XM_030251122,XM_030251123,XM_030251124,XM_030251125,XR_003952825,XR_877692	Rab3il1	RAB3A interacting protein (rabin3)-like 1	protein_coding	NP_001334358.1;NP_001334359.1;NP_653121.1;XP_006527474.3;XP_006527475.1;XP_006527477.1;XP_006527480.1;XP_011245687.1;XP_030106979.1;XP_030106980.1;XP_030106981.1;XP_030106982.1;XP_030106983.1;XP_030106984.1;XP_030106985.1	1922010	.	22.779715	[illegible]
74761	NM_024263,XM_006539235,XM_011250346	Mxra8	matrix-remodelling associated 8	protein_coding	NP_077225.4;XP_006539298.1;XP_011248648.1	1922011	.	171.140524	[illegible]
74762	NM_001081160,NM_001368423,XM_006525041,XR_003952202,XR_003952203,XR_003952204,XR_385375,XR_385376,XR_876562,XR_876563	Mdga1	MAM domain containing glycosylphosphatidylinositol anchor 1	protein_coding	NP_001074629.1;NP_001355352.1;XP_006525104.1	1922012	.	3.716631	[illegible]
74763	NM_001290689,NM_029090,XM_006522679,XM_006522680,XM_017317155	Naa60	N(alpha)-acetyltransferase 60, NatF catalytic subunit	protein_coding	NP_001277618.1;NP_083366.1;XP_006522742.1;XP_006522743.1;XP_017172644.1	1922013	.	21.294345	[illegible]
74764	NM_001357130,NM_029091,XM_006525044,XM_011246689	Klc4	kinesin light chain 4	protein_coding	NP_001344059.1;NP_083367.1;XP_006525107.1;XP_011244991.1	1922014	.	9.234296	[illegible]
74766	NM_001205157,NM_001205158,NM_001359298,NM_001359299,NM_138303,NR_038062,XM_011242618	Yipf2	Yip1 domain family, member 2	protein_coding	NP_001192086.1;NP_001192087.1;NP_001346227.1;NP_001346228.1;NP_612176.1;XP_011240920.1	1922016	.	3.997890	[illegible]
74769	NM_029094,XM_011242829,XM_011242830,XM_030244688	Pik3cb	phosphatidylinositol-4,5-bisphosphate 3-kinase catalytic subunit beta	protein_coding	NP_083370.2;XP_011241131.1;XP_011241132.1;XP_030100548.1	1922019	.	2.089979	[illegible]
74770	NM_029095,NR_027967,XM_006512379,XM_006512380,XM_006512381,XM_006512383,XM_011243053,XM_030244689,XR_003948	Hhatl	hedgehog acyltransferase-like	protein_coding	NP_083371.2;XP_006512442.1;XP_006512443.1;XP_006512444.1;XP_006512446.1;XP_011241355.1;XP_030100549.1	1922020	.	1.384572	[illegible]
74772	NM_001164366,NM_029097,XM_006539236,XM_006539237,XM_017320424,XM_030253864,XR_003955033	Atp13a2	ATPase type 13A2	protein_coding	NP_001157838.1;NP_083373.2;XP_006539299.1;XP_006539300.1;XP_017175913.1;XP_030109724.1	1922022	.	7.998605	[illegible]
74775	NM_029098,XM_030248804	Lmbr1l	limb region 1 like	protein_coding	NP_083374.1;XP_030104664.1	1289247	.	10.807455	[illegible]
74776	NM_001293641,NM_146141,XM_006502216,XM_006502217,XM_030252857,XM_030252858	Ppa2	pyrophosphatase (inorganic) 2	protein_coding	NP_001280570.1;NP_666253.1;XP_006502279.1;XP_006502280.1;XP_030108717.1;XP_030108718.1	1922026	.	9.861675	[illegible]
74777	NM_029100	Selenon	selenoprotein N	protein_coding	NP_083376.2	2151208	.	25.771996	[illegible]
74778	NM_001358061,NM_029101	Rrp7a	ribosomal RNA processing 7 homolog A (S. cerevisiae)	protein_coding	NP_001344990.1;NP_083377.3	1922028	.	22.817665	[illegible]
74781	NM_178398,XM_006504752	Wipi2	WD repeat domain, phosphoinositide interacting 2	protein_coding	NP_848485.1;XP_006504815.1	1923831	.	17.675274	[illegible]
74782	NM_029102,XM_006514255,XM_011243591,XM_030245335	Glt8d2	glycosyltransferase 8 domain containing 2	protein_coding	NP_083378.2;XP_006514318.1;XP_011241893.1;XP_030101195.1	1922032	.	16.233117	[illegible]
74838	NM_053089	Naa15	N(alpha)-acetyltransferase 15, NatA auxiliary subunit	protein_coding	NP_444319.3	1922088	.	12.426610	[illegible]
74840	NM_029103	Manf	mesencephalic astrocyte-derived neurotrophic factor	protein_coding	NP_083379.2	1922090	.	86.769325	[illegible]
74841	NM_027554,XM_006531450,XR_003947351	Usp38	ubiquitin specific peptidase 38	protein_coding	NP_081830.2;XP_006531513.1	1922091	.	6.736827	[illegible]
74843	NM_029104,XM_006519636	Mss51	MSS51 mitochondrial translational activator	protein_coding	NP_083380.1;XP_006519699.1	1922093	.	3.478341	[illegible]
74844	XR_876673,XR_876674,XR_876675,XR_876676,XR_876677	4833447I15Rik	RIKEN cDNA 4833447I15 gene, transcript variant X5	lncRNA	.	1922094	.	0.651558	[illegible]
74846	NR_040505	4930405A21Rik	RIKEN cDNA 4930405A21 gene	lncRNA	.	1922096	.	0.425963	[illegible]
74855	NM_029107	Fam228a	family with sequence similarity 228, member A	protein_coding	NP_083383.1	1922105	.	0.218770	[illegible]
74868	NM_175212,XM_006521504,XM_011245759	Tmem65	transmembrane protein 65	protein_coding	NP_780421.2;XP_006521567.1;XP_011244061.1	1922118	.	25.740452	[illegible]
74879	NR_040736	4930461G14Rik	RIKEN cDNA 4930461G14 gene	lncRNA	.	1922129	.	0.060741	[illegible]
74881	NR_110448	4930459C07Rik	RIKEN cDNA 4930459C07 gene	lncRNA	.	1922131	.	0.000000	[illegible]
74883	XR_384910,XR_384911,XR_384912,XR_876121	4930461C15Rik	RIKEN cDNA 4930461C15 gene, transcript variant X3	lncRNA	.	1922133	.	0.000000	[illegible]
74895	NM_029115	Ccdc181	coiled-coil domain containing 181	protein_coding	NP_083391.2	1922145	.	1.094287	[illegible]
74901	NM_029116,XM_006508915,XM_006508916,XR_870083,XR_87008	Kbtbd11	kelch repeat and BTB (POZ) domain containing 11	protein_coding	NP_083392.1;XP_006508978.1;XP_006508979.1	1922151	.	4.802130	[illegible]
74917	NR_027986	4930474M22Rik	RIKEN cDNA 4930474M22 gene	lncRNA	.	1922167	.	0.252143	[illegible]
74918	NM_001368722,NM_029122	Iqca	IQ motif containing with AAA domain	protein_coding	NP_001355651.1;NP_083398.2	1922168	.	0.000000	[illegible]
74919	NM_175675,XM_017321124	Slc35f6	solute carrier family 35, member F6	protein_coding	NP_783606.2;XP_017176613.1	1922169	.	7.938607	[illegible]
74925	NR_046277	4930479D17Rik	RIKEN cDNA 4930479D17 gene	lncRNA	.	1922175	.	0.000000	[illegible]
74931	NR_015545,NR_027938	4930481A15Rik	RIKEN cDNA 4930481A15 gene	lncRNA	.	1922181	.	0.820640	[illegible]
74934	NM_001357492,XM_006526312,XM_006526313,XM_011247016,XM_011247017,XM_030250622	Armc4	armadillo repeat containing 4	protein_coding	NP_001344421.1;XP_006526375.1;XP_006526376.1;XP_011245318.1;XP_011245319.1;XP_030106482.1	1922184	.	0.044027	[illegible]
74939	NR_153835	4930471G24Rik	RIKEN cDNA 4930471G24 gene	lncRNA	.	1922189	.	0.000000	[illegible]
74953	NR_045354	4930483K19Rik	RIKEN cDNA 4930483K19 gene	lncRNA	.	1922203	.	0.000000	[illegible]
74959	NR_040322,NR_040323	Platr14	pluripotency associated transcript 14	lncRNA	.	1922209	.	0.000000	[illegible]
74970	NR_046279	4930483O08Rik	RIKEN cDNA 4930483O08 gene	lncRNA	.	1922220	.	0.000000	[illegible]
74978	NM_001163559,NM_001361058,NM_029134,XM_006514256,XM_006514259,XM_011243592,XM_011243593,XM_011243594,XM_011243595,XM_011243596,XM_030245336,XM_030245337,XM_030245338,XR_871734	Lrriq1	leucine-rich repeats and IQ motif containing 1	protein_coding	NP_001157031.1;NP_001347987.1;NP_083410.1;XP_006514319.1;XP_006514322.1;XP_011241894.1;XP_011241895.1;XP_011241896.1;XP_011241897.1;XP_011241898.1;XP_030101196.1;XP_030101197.1;XP_030101198.1	1922228	.	0.000000	[illegible]
74992	NR_040601	4930487H11Rik	RIKEN cDNA 4930487H11 gene	lncRNA	.	1922242	.	0.000000	[illegible]
74996	NM_001357952,NM_133758,NM_177249,XM_006508273,XM_030243044	Usp47	ubiquitin specific peptidase 47	protein_coding	NP_001344881.1;NP_598519.2;NP_796223.2;XP_006508336.1;XP_030098904.1	1922246	.	31.033577	[illegible]
74998	NM_001033172,NM_001164367,XM_011247386,XM_011247388	Rab11fip2	RAB11 family interacting protein 2 (class I)	protein_coding	NP_001028344.2;NP_001157839.1;XP_011245688.1;XP_011245690.	1922248	.	3.843252	[illegible]
75001	NR_108044	4930482G09Rik	RIKEN cDNA 4930482G09 gene	lncRNA	.	1922251	.	0.000000	[illegible]
75007	NM_133858,NM_199475,XM_006502219,XM_011240261,XM_017319778,XM_030252859,XM_030252860	Mindy1	MINDY lysine 48 deubiquitinase 1	protein_coding	NP_598619.2;NP_955769.1;XP_006502282.1;XP_011238563.1;XP_017175267.1;XP_030108719.1;XP_030108720.1	1922257	.	18.234653	[illegible]
75010	NM_029141,NM_029154,XM_006503611,XM_006503612,XM_006503614,XM_017321125,XM_030254863	Tmbim7	transmembrane BAX inhibitor motif containing 7	protein_coding	NP_083417.1;NP_083430.1;XP_006503674.1;XP_006503675.1;XP_006503677.1;XP_017176614.1;XP_030110723.1	1922305	.	0.000000	[illegible]
75013	NM_029142,XM_006535359	4930502E18Rik	RIKEN cDNA 4930502E18 gene	protein_coding	NP_083418.1;XP_006535422.1	1922263	.	0.194925	[illegible]
75015	NM_001368725,NM_001368726,NM_029144,XM_006502225,XM_011240263,XM_011240264,XM_030252861,XM_030252862	4930503B20Rik	RIKEN cDNA 4930503B20 gene	protein_coding	NP_001355654.1;NP_001355655.1;NP_083420.2;XP_006502288.1;XP_011238565.1;XP_011238566.1;XP_030108721.1;XP_030108722.1	1922264	.	0.305855	[illegible]
75016	NR_130157,NR_130166	4930480K23Rik	RIKEN cDNA 4930480K23 gene	lncRNA	.	1922266	.	0.363510	[illegible]
75019	NM_001162863,NM_029145,XM_006519639	Rnase10	ribonuclease, RNase A family, 10 (non-active)	protein_coding	NP_001156335.1;NP_083421.2;XP_006519702.1	1922269	.	0.058132	[illegible]
75025	NR_045356	4930473O22Rik	RIKEN cDNA 4930473O22 gene	lncRNA	.	1922275	.	0.000000	[illegible]
75029	NM_001098233,NM_152821,XM_006509205,XM_006509206,XM_006509209,XM_011242165,XM_011242166,XM_017312994	Purg	purine-rich element binding protein G	protein_coding	NP_001091703.1;NP_690034.1;XP_006509268.1;XP_006509269.1;XP_006509272.1;XP_011240467.1;XP_011240468.1;XP_017168483.1	1922279	.	5.931004	[illegible]
75033	NM_001357477,NM_001357479,NM_175213	Mei4	meiotic double-stranded break formation protein 4	protein_coding	NP_001344406.1;NP_001344408.1;NP_780422.1	1922283	.	0.000000	[illegible]
75037	NR_153101	4930500G05Rik	RIKEN cDNA 4930500G05 gene	lncRNA	.	1922287	.	0.038208	[illegible]
75040	NM_029152,XM_030246964	Efcab10	EF-hand calcium binding domain 10	protein_coding	NP_083428.1;XP_030102824.1	1922290	.	0.000000	[illegible]
75044	NR_126090,NR_126091	4930506A18Rik	RIKEN cDNA 4930506A18 gene	lncRNA	.	1922294	.	0.000000	[illegible]
75050	NM_001360894,NM_175214,XM_006517426,XM_006517427,XM_006517429,XM_006517431,XM_006517432,XM_006517433,XM_006517434,XM_006517435,XM_006517436,XM_011244579,XM_011244580,XM_017315619,XM_017315620,XM_017315621,XM_017315622,XM_017315623,XM_030247439,XR_001780822,XR_003950487	Kif27	kinesin family member 27	protein_coding	NP_001347823.1;NP_780423.2;XP_006517489.1;XP_006517490.1;XP_006517492.1;XP_006517494.1;XP_006517495.1;XP_006517496.1;XP_006517497.1;XP_006517498.1;XP_006517499.1;XP_011242881.1;XP_011242882.1;XP_017171108.1;XP_017171109.1;XP_017171110.1;XP_017171111.1;XP_017171112.1;XP_030103299.1	1922300	.	0.286886	[illegible]
75051	NM_001077684,XM_006500322,XM_006500324,XM_006500325,XM_011239823,XR_001783196	Ccdc173	coiled-coil domain containing 173	protein_coding	NP_001071152.1;XP_006500385.1;XP_006500387.1;XP_006500388.1;XP_011238125.1	1923100	.	1.303809	[illegible]
75053	NR_131921	Kif16bos	kinesin family member 16B, opposite strand	lncRNA	.	1922303	.	0.000000	[illegible]
75060	NR_073374	4930506C21Rik	RIKEN cDNA 4930506C21 gene	lncRNA	.	1922310	.	0.324793	[illegible]
75062	NM_029157	Sf3a3	splicing factor 3a, subunit 3	protein_coding	NP_083433.1	1922312	.	25.252657	[illegible]
75064	NM_029158	Zcchc13	zinc finger, CCHC domain containing 13	protein_coding	NP_083434.1	1922314	.	0.000000	[illegible]

75079	NM_001359466,NM_029162,NR_153116,XM_006504163,XM_006504168,XM_011240780,XM_030254864,XM_030254865	Zbtb49	zinc finger and BTB domain containing 49	protein_coding	NP_001346395.1;NP_083438.1;XP_006504226.1;XP_006504231.1;XP_011239082.1;XP_030110724.1;XP_030110725.1	1922329	.	3.164820
75083	NM_029163	Usp50	ubiquitin specific peptidase 50	protein_coding	NP_083439.2	1922333	.	0.027372
75084	NR_015494,NR_027944,NR_027945	4930511M06Rik	RIKEN cDNA 4930511M06 gene	lncRNA	.	1922334	.	0.032129
75087	NM_001100394	4930505A04Rik	RIKEN cDNA 4930505A04 gene	protein_coding	NP_001093864.1	1922337	.	0.139712
75089	NM_029166,XM_006514264,XM_017314128,XM_017314129,XM_030245339,XM_030245340,XM_030245341,XM_030245342,XR_003948815,XR_380453	Uhrf1bp1l	UHRF1 (ICBP90) binding protein 1-like	protein_coding	NP_083442.2;XP_006514327.1;XP_017169617.1;XP_017169618.1;XP_030101199.1;XP_030101200.1;XP_030101201.1;XP_030101202.1	2442888	.	8.511224
75095	NR_045416	4930513D17Rik	RIKEN cDNA 4930513D17 gene	lncRNA		1922345	.	0.000000
75099	NM_001191051,NM_001357962,NM_001357963,NM_001357964,NM_175215,XM_006541236,XM_030243045,XM_030243046	Lysmd4	LysM, putative peptidoglycan-binding, domain containing 4	protein_coding	NP_001177980.1;NP_001344891.1;NP_001344892.1;NP_001344893.1;NP_780424.1;XP_006541299.1;XP_030098905.1;XP_030098906.1	1922349	.	4.679515
75100	NR_040738	4930525D18Rik	RIKEN cDNA 4930525D18 gene	lncRNA	.	1922350	.	0.089708
75104	NM_175217,XM_006504753,XM_011240988	Mmd2	monocyte to macrophage differentiation-associated 2	protein_coding	NP_780426.1;XP_006504816.1;XP_011239290.1	1922354	.	0.016440
75106	NM_001358270,XM_011247688	4930519F16Rik	RIKEN cDNA 4930519F16 gene	protein_coding	NP_001345199.1;XP_011245990.1	1922356	.	0.023940
75111	XR_379016,XR_870457,XR_870458	4930512H18Rik	RIKEN cDNA 4930512H18 gene, transcript variant X1	lncRNA	.	1922361	.	0.019445
75115	NR_045735	4930509E16Rik	RIKEN cDNA 4930509E16 gene	lncRNA		1922365	.	0.064199
75116	NR_040383,NR_040384	4930520O04Rik	RIKEN cDNA 4930520O04 gene	lncRNA		1922366	.	0.249213
75124	NM_029173,XM_006516959,XM_006516960,XM_006516961,XM_006516962	Nxnl2	nucleoredoxin-like 2	protein_coding	NP_083449.1;XP_006517022.1;XP_006517023.1;XP_006517024.1;XP_006517025.1	1922374	.	0.271247
75129	XM_030254981	4930524J08Rik	RIKEN cDNA 4930524J08 gene	protein_coding	XP_030110841.1	1922379	.	0.415508
75131	NR_131021	4930512J16Rik	RIKEN cDNA 4930512J16 gene	lncRNA	.	1922381	.	0.000000
75135	NR_015516,NR_027950,NR_102721	4930526I15Rik	RIKEN cDNA 4930526I15 gene	lncRNA	.	1922385	.	1.219764
75137	NM_001081293,NM_001368178,NM_001368179,NM_001368180,NM_001368181,NM_028328,XM_011240267,XM_030252863	Rprd2	regulation of nuclear pre-mRNA domain containing 2	protein_coding	NP_001074762.1;NP_001355107.1;NP_001355108.1;NP_001355109.1;NP_001355110.1;NP_082604.2;XP_011238569.1;XP_030108723.1	1922387	.	6.880141
75138	NR_045317	4930526L06Rik	RIKEN cDNA 4930526L06 gene	lncRNA	.	1922388	.	0.000000
75141	NM_029182	Rasd2	RASD family, member 2	protein_coding	NP_083458.1	1922391	.	0.095737
75146	NM_029186,XR_386522	Mfsd13a	major facilitator superfamily domain containing 13a	protein_coding	NP_083462.2	1922396	.	0.643374
75160	XR_003951519,XR_003951520,XR_003951521,XR_003951522,XR_003951523,XR_003951524,XR_384312,XR_384313,XR_875537,XR_875540	4930543I03Rik	RIKEN cDNA 4930543I03 gene, transcript variant X8	lncRNA	.	1922410	.	0.033482
75172	NM_029195,XM_017321126	Ccdc146	coiled-coil domain containing 146	protein_coding	NP_083471.1;XP_017176615.1	1922422	.	0.182649
75173	NM_029196,XM_011240626,XM_011240627	Tex38	testis expressed 38	protein_coding	NP_083472.1;XP_011238928.1;XP_011238929.1	1922423	.	0.212834
75178	NM_029197,XM_006525045,XM_006525047,XM_006525048,XM_006525052,XM_017317702	Meiob	meiosis specific with OB domains	protein_coding	NP_083473.1;XP_006525108.1;XP_006525110.1;XP_006525111.1;XP_006525115.1;XP_017173191.1	1922428	.	0.000000
75180	NM_029198,XM_006503455,XM_006503456,XM_011240628,XM_030253868	Tmem269	transmembrane protein 269	protein_coding	NP_083474.1;XP_006503518.1;XP_006503519.1;XP_011238930.1;XP_030109728.1	1922430	.	0.246850
75196	NM_001167757,NM_029202,XM_006505191,XM_006505192,XR_003956259	Ankrd7	ankyrin repeat domain 7	protein_coding	NP_001161229.1;NP_083478.2;XP_006505254.1;XP_006505255.1	1922446	.	0.151066
75202	NM_001162909,XM_006525053,XM_006525055,XM_006525056,XM_011246692,XM_011246695,XM_017317705,XM_030250121,XR_003952205,XR_003952206,XR_003952207,XR_003952208,XR_385377,XR_385378,XR_385379,XR_876567,XR_876568,XR_876569,XR_876571,XR_876572	Spaca6	sperm acrosome associated 6	protein_coding	NP_001156381.1;XP_006525116.1;XP_006525118.1;XP_006525119.1;XP_011244994.1;XP_011244997.1;XP_017173194.1;XP_030105981.1	1922452	.	13.981922
75209	NM_029210,XM_030247440	Sv2c	synaptic vesicle glycoprotein 2c	protein_coding	NP_083486.1;XP_030103300.1	1922459	.	0.041039
75210	NM_001165892,NM_001282012,NM_001282013,NM_001282024,NM_001282025,NM_145487,XM_006525060,XM_006525062,XM_006525063,XM_030250122,XR_001782124	Prr3	proline-rich polypeptide 3	protein_coding	NP_001159364.1;NP_001268941.1;NP_001268942.1;NP_001268953.1;NP_001268954.1;NP_663462.2;XP_006525123.3;XP_006525125.3;XP_006525126.1;XP_030105982.1	1922460	.	12.013739
75212	NM_001360748,NM_029211,XM_006508275,XM_030243047,XM_030243048	Rnf121	ring finger protein 121	protein_coding	NP_001347677.1;NP_083487.2;XP_006508338.1;XP_030098907.1;XP_030098908.1	1922462	.	14.813925
75216	NM_181815,XM_006516324,XM_011244195,XM_011244196,XM_011244197,XM_017315228,XM_030246965,XM_030246966,XR_001780497	Cep128	centrosomal protein 128	protein_coding	NP_861536.3;XP_006516387.1;XP_011242497.1;XP_011242498.1;XP_011242499.1;XP_017170717.1;XP_030102825.1;XP_030102826.1	1922466	.	1.781631
75219	NM_173745,XM_006514873	Dusp18	dual specificity phosphatase 18	protein_coding	NP_776106.1;XP_006514936.1	1922469	.	3.356806
75221	NM_001360711,NM_133803	Dpp3	dipeptidylpeptidase 3	protein_coding	NP_001347640.1;NP_598564.2	1922471	.	16.212531
75234	NM_001368763,NM_029219	Rnf19b	ring finger protein 19B	protein_coding	NP_001355692.1;NP_083495.1	1922484	.	11.990928
75255	NR_038030,NR_038031	4930562F07Rik	RIKEN cDNA 4930562F07 gene	lncRNA	.	1922505	.	0.271093
75258	NM_183111,XM_006511524,XM_017313672,XM_017313673,XM_017313674,XM_017313675,XM_017313676,XM_017313677	4930563M21Rik	RIKEN cDNA 4930563M21 gene	protein_coding	NP_898934.2;XP_006511587.1;XP_017169161.1;XP_017169162.1;XP_017169163.1;XP_017169164.1;XP_017169165.1;XP_017169166.1	1922508	.	0.000000
75259	NR_045063,NR_045064,NR_045065	4930556M19Rik	RIKEN cDNA 4930556M19 gene	lncRNA	.	1922509	.	2.113873
75273	NM_029231	Pelp1	proline, glutamic acid and leucine rich protein 1	protein_coding	NP_083507.3	1922523	.	6.914044
75276	NM_001290743,NM_001290744,NM_172420,NR_110979,NR_1109	Ppp1r1c	protein phosphatase 1, regulatory inhibitor subunit 1C	protein_coding	NP_001277672.1;NP_001277673.1;NP_766008.2	1923185	.	0.059648
75279	NR_040633	4930555B11Rik	RIKEN cDNA 4930555B11 gene	lncRNA	.	1922529	.	0.000000
75280	NR_131011	4930554G24Rik	RIKEN cDNA 4930554G24 gene	lncRNA	.	1922530	.	0.000000
75284	NM_029236	Bcdin3d	BCDIN3 domain containing	protein_coding	NP_083512.2	1922534	.	2.844227
75286	NR_045376	4930549G23Rik	RIKEN cDNA 4930549G23 gene	lncRNA	.	1922536	.	0.000000
75288	NM_029238,XM_030248087,XM_030248088	Slc35f4	solute carrier family 35, member F4	protein_coding	NP_083514.1;XP_030103947.1;XP_030103948.1	1922538	.	0.000000
75291	NM_001098237,NM_133759,XM_006527419,XM_006527420	Zbtb3	zinc finger and BTB domain containing 3	protein_coding	NP_001091707.1;NP_598520.1;XP_006527482.1;XP_006527483.1	1922541	.	1.137854
75292	NM_001171004,NM_001171005,NM_029239,XM_030250123	Prkd3	protein kinase D3	protein_coding	NP_001164475.1;NP_001164476.1;NP_083515.2;XP_030105983.1	1922542	.	23.054563
75296	NM_001197046,NM_201230,XM_006523355,XM_006523356,XM_006523357,XM_030250124,XM_030250125	Fgfr1op	Fgfr1 oncogene partner	protein_coding	NP_001183975.1;NP_957682.1;XP_006523418.1;XP_006523419.1;XP_006523420.1;XP_030105984.1;XP_030105985.1	1922546	.	7.750862
75302	NM_001270988,NM_172421,XM_006515239,XM_006515241,XM_030246967,XM_030246968,XM_030246969,XM_030246970,XM_030246971,XM_030246972	Asxl2	additional sex combs like 2, transcriptional regulator	protein_coding	NP_001257917.1;NP_766009.2;XP_006515302.1;XP_006515304.1;XP_030102827.1;XP_030102828.1;XP_030102829.1;XP_030102830.1;XP_030102831.1;XP_030102832.1	1922552	.	5.106466
75304	NM_001163728	4930563E22Rik	RIKEN cDNA 4930563E22 gene	protein_coding	NP_001157200.1	1922554	.	0.877127
75310	NR_040506	4930556C24Rik	RIKEN cDNA 4930556C24 gene	lncRNA		1922560	.	0.000000
75311	NM_029247,XM_006510652,XM_006510653,XM_006510654,XM_006510656,XM_006510660,XM_030244690	4930550C14Rik	RIKEN cDNA 4930550C14 gene	protein_coding	NP_083523.1;XP_006510715.1;XP_006510716.1;XP_006510717.1;XP_006510719.1;XP_006510723.1;XP_030100550.1	1922561	.	0.525056
75316	NM_027261,NM_029248,NR_028401,XM_011242620,XR_001779059,XR_003948044,XR_003948045,XR_003948046,XR_379141,XR_870541,XR_870542,XR_870543	Taf1d	TATA-box binding protein associated factor, RNA polymerase I, D	protein_coding	NP_081537.1;NP_083524.2;XP_011240922.1	1922566	.	25.038995
75317	NM_029249,XM_006514267,XM_006514268,XM_006514269,XM_011243597,XM_017314131,XM_017314132,XM_030245343,XR_003948816	Parpbp	PARP1 binding protein	protein_coding	NP_083525.1;XP_006514330.1;XP_006514331.1;XP_006514332.1;XP_011241899.1;XP_017169620.1;XP_017169621.1;XP_030101203.1	1922567	.	2.984060
75320	NM_029250,XM_006507011,XM_006507012	Etnk1	ethanolamine kinase 1	protein_coding	NP_083526.2;XP_006507074.1;XP_006507075.1	1922570	.	16.291074
75323	NR_037999	4930558J18Rik	RIKEN cDNA 4930558J18 gene	lncRNA	.	1922573	.	0.152933
75328	NM_029252	Fam243	family with sequence similarity 243	protein_coding	NP_083528.1	1922578	.	0.000000
75329	NM_029253,NM_153123,XM_006522682,XM_006522684,XM_011246039	Atf7ip2	activating transcription factor 7 interacting protein 2	protein_coding	NP_083529.1;NP_694763.2;XP_006522745.1;XP_006522747.1;XP_011244341.1	1922579	.	0.191342
75338	NM_029256,NM_183293,NR_111924,XM_006508277	Ccdc83	coiled-coil domain containing 83	protein_coding	NP_083532.1;NP_899116.1;XP_006508340.1	1918255	.	0.000000
75339	NM_001360072,NM_023773,NR_153396,XR_383217	Mphosph8	M-phase phosphoprotein 8	protein_coding	NP_001347001.1;NP_076262.2	1922589	.	10.456842
75345	NM_001347184,NM_144539,XM_006497011,XM_006497012,XM_011238874,XM_011238875,XM_011238876,XM_011238877,XM_030243259	Slamf7	SLAM family member 7	protein_coding	NP_001334113.1;NP_653122.2;XP_006497074.1;XP_006497075.1;XP_011237176.1;XP_011237177.1;XP_011237178.1;XP_011237179.1;XP_030099119.1	1922595	.	0.047357
75352	NM_023774,XM_030251514	4930550L24Rik	RIKEN cDNA 4930550L24 gene	protein_coding	NP_076263.1;XP_030107374.1	1922602	.	0.027773
75354	NR_126458	4930554I06Rik	RIKEN cDNA 4930554I06 gene	lncRNA	.	1922604	.	0.000000
75355	NR_155305	4930553P18Rik	RIKEN cDNA 4930553P18 gene	lncRNA	.	1922605	.	0.000000
75359	XR_001778771,XR_001778777,XR_003947384,XR_003947385,XR_003947386,XR_003947387,XR_003947388,XR_003947389,XR_003947390,XR_003947391	4930555F03Rik	RIKEN cDNA 4930555F03 gene, transcript variant X2	lncRNA		1922609	.	0.000000
75373	XR_003956381	4930597O21Rik	RIKEN cDNA 4930597O21 gene	lncRNA	.	1922623	.	0.000000
75379	NR_045813	4930599N23Rik	RIKEN cDNA 4930599N23 gene	lncRNA	.	1922629	.	0.152340
75387	NM_001167691,NM_133760,XM_006530480,XM_006530481,XM_006530482,XM_017321127,XM_030254866,XM_030254867,XM_030254868,XR_003955717,XR_003955718	Sirt4	sirtuin 4	protein_coding	NP_001161163.1;NP_598521.1;XP_006530543.1;XP_006530544.1;XP_006530545.1;XP_017176616.1;XP_030110726.1;XP_030110727.1;XP_030110728.1	1922637	.	7.768131
75388	NM_001113367,NM_001367887,NM_001368767,NM_001368768,NM_029267,XR_373267	Boll	boule homolog, RNA binding protein	protein_coding	NP_001106838.1;NP_001354816.1;NP_001355696.1;NP_001355697.1;NP_083543.2	1922638	.	0.033048
75389	NR_126460	4930597A21Rik	RIKEN cDNA 4930597A21 gene	lncRNA	.	1922639	.	0.000000
75394	NR_040757,NR_104577	0610040F04Rik	RIKEN cDNA 0610040F04 gene	lncRNA	.	1922644	.	0.000000
75396	NM_029269,XM_006529918	Spp2	secreted phosphoprotein 2	protein_coding	NP_083545.1;XP_006529981.1	1922646	.	0.000000
75398	NM_029271	Mrpl32	mitochondrial ribosomal protein L32	protein_coding	NP_083547.1	2137226	.	15.615133
75404	NM_001081123,NM_001356394,XM_006541554,XM_006541555,XM_017318632,XM_017318633,XM_030251515	Arhgap36	Rho GTPase activating protein 36	protein_coding	NP_001074592.1;NP_001343323.1;XP_006541617.1;XP_006541618.1;XP_017174121.1;XP_017174122.1;XP_030107375.1	1922654	.	6.045743
75405	XR_383959	1100001I12Rik	RIKEN cDNA 1100001I12 gene	lncRNA	.	1922655	.	0.162823
75406	NM_001364694,NM_029272,XM_006514270,XM_030245344	Ndufs7	NADH:ubiquinone oxidoreductase core subunit S7	protein_coding	NP_001351623.1;NP_083548.1;XP_006514333.1;XP_030101204.1	1922656	.	32.680772
75409	NM_198865,XM_006519641,XM_006519642,XM_006519643	Slitrk5	SLIT and NTRK-like family, member 5	protein_coding	NP_942565.1;XP_006519704.1;XP_006519705.1;XP_006519706.1	2679448	.	0.960535
75410	NM_001290573,NM_029274,XM_006540408,XM_011250733	Kmt2b	lysine (K)-specific methyltransferase 2B	protein_coding	NP_001277502.1;NP_083550.2;XP_006540471.1;XP_011249035.1	109565	.	9.898607
75415	NM_001039692,NM_029277,XM_006526314,XM_006526315,XM_006526316,XM_011247018,XM_017318001,XM_017318002,XM_030250623	Arhgap12	Rho GTPase activating protein 12	protein_coding	NP_001034781.1;NP_083553.2;XP_006526377.1;XP_006526378.1;XP_006526379.1;XP_011245320.1;XP_017173490.1;XP_017173491.1;XP_030106483.1	1922665	.	9.765446
75416	NM_029278,XR_376833	Nop14	NOP14 nucleolar protein	protein_coding	NP_083554.2	1922666	.	10.435077
75420	NM_001308448,NM_029279,XM_006516966,XR_001780824,XR_003950488,XR_003950489,XR_382236,XR_382237,XR_382238	Secisbp2	SECIS binding protein 2	protein_coding	NP_001295377.1;NP_083555.1;XP_006517029.1	1922670	.	8.160149
75422	NM_029280,NR_110977,XM_006500329,XM_017319304,XM_030252163,XM_030252164,XR_374533	Mettl5	methyltransferase like 5	protein_coding	NP_083556.2;XP_006500392.1;XP_017174793.1;XP_030108023.1;XP_030108024.1	1922672	.	4.900493
75423	NM_182994,XM_011239204	Arl5a	ADP-ribosylation factor-like 5A	protein_coding	NP_892039.1;XP_011237506.1	1922673	.	17.014062
75424	NM_029281	Zfp820	zinc finger protein 820	protein_coding	NP_083557.2	1922674	.	2.758722
75425	NM_029282,XM_006500333,XM_011239826	Tti1	TELO2 interacting protein 1	protein_coding	NP_083558.1;XP_006500396.1;XP_011238128.1	1922675	.	5.546788
75426	NM_018741	Igfbpl1	insulin-like growth factor binding protein-like 1	protein_coding	NP_061211.1	1933198	.	0.000000
75429	NM_001162878,NM_029283,XM_006534386	Fam183b	family with sequence similarity 183, member B	protein_coding	NP_001156350.2;NP_083559.2;XP_006534449.1	1922679	.	0.000000
75430	NM_001291348,NM_001291349,NM_001291352,NM_001291353,NM_027532,XM_006508279,XM_006508280,XM_006508282,XM_006508284,XM_006508285,XM_011241930,XM_011241931,XM_011241932,XM_011241933,XM_011241935,XM_017312358,XM_030243049,XM_030243050,XM_030243051,XM_030243052,XM_030243053,XM_030243054	Anapc15	anaphase promoting complex C subunit 15	protein_coding	NP_001278277.1;NP_001278278.1;NP_001278281.1;NP_001278282.1;NP_081808.2;XP_006508342.1;XP_006508343.1;XP_006508345.1;XP_006508347.1;XP_006508348.1;XP_011240232.1;XP_011240233.1;XP_011240234.1;XP_011240235.1;XP_011240237.1;XP_017167847.1;XP_030098909.1;XP_030098910.1;XP_030098911.1;XP_030098912.1;XP_030098913.1;XP_030098914.1	1922680	.	6.131037
75434	NM_029285,XM_006504170,XM_011240783,XM_017321128	1700001C02Rik	RIKEN cDNA 1700001C02 gene	protein_coding	NP_083561.1;XP_006504233.1;XP_011239085.1;XP_017176617.1	1922684	.	0.000000
75444	NM_001311063,NM_029290,XM_017318003,XM_030250624,XM_030250625,XM_030250626	Ccdc192	coiled-coil domain containing 192	protein_coding	NP_001297992.1;NP_083566.1;XP_017173492.1;XP_030106484.1;XP_030106485.1;XP_030106486.1	1922694	.	0.069963
75452	NM_029291,XM_006514874,XR_380985	Ascc2	activating signal cointegrator 1 complex subunit 2	protein_coding	NP_083567.1;XP_006514937.1	1922702	.	10.301616
75454	NM_029293,XM_030252166	Phpt1	phosphohistidine phosphatase 1	protein_coding	NP_083569.1;XP_030108026.1	1922704	.	35.136500
75458	NM_001037841,NM_001286383,NM_029295,NR_104433,XM_030243826,XM_030243827,XR_003947352	Cklf	chemokine-like factor	protein_coding	NP_001032930.1;NP_001273312.1;NP_083571.1;XP_030099686.1;XP_030099687.1	1922708	.	6.233009
75462	NM_001172091,NM_001347584,NM_029296,XM_030250126,XM_030250127	1700001C19Rik	RIKEN cDNA 1700001C19 gene	protein_coding	NP_001165562.1;NP_001334513.1;NP_083572.1;XP_030105986.1;XP_030105987.1	1922712	.	0.000000
75465	NM_029297	Dynlrb2	dynein light chain roadblock-type 2	protein_coding	NP_083573.1	1922715	.	0.000000
75467	NM_029298,XM_006525070,XM_006525071,XM_006525072,XM_011246698,XM_011246699,XM_017317707,XR_001782126,XR_385	Stpg4	sperm tail PG rich repeat containing 4	protein_coding	NP_083574.1;XP_006525133.1;XP_006525134.1;XP_006525135.1;XP_011245000.1;XP_011245001.1;XP_017173196.1	1922717	.	0.053912
75469	NM_001305058,NM_029299	Spata19	spermatogenesis associated 19	protein_coding	NP_001291987.1;NP_083575.1	1922719	.	0.000000
75470	NM_029300	Iqcf5	IQ motif containing F5	protein_coding	NP_083576.1	1922720	.	0.000000
75471	NM_001081095	1700009N14Rik	RIKEN cDNA 1700009N14 gene	protein_coding	NP_001074564.1	1922721	.	0.000000
75472	NM_001081275,XM_006497018,XM_006497019,XM_006497020,XM_011238879,XM_011238880,XM_017312596	Cfap126	cilia and flagella associated protein 126	protein_coding	NP_001074744.1;XP_006497081.1;XP_006497082.1;XP_006497083.1;XP_011237181.1;XP_011237182.1;XP_017168085.1	1922722	.	0.728947
75475	NM_153122,XM_006521507,XM_006521508,XM_006521509,XM_030248806,XM_030248807,XR_384054	Oplah	5-oxoprolinase (ATP-hydrolysing)	protein_coding	NP_694762.1;XP_006521570.1;XP_006521571.1;XP_006521572.1;XP_030104666.1;XP_030104667.1	1922725	.	4.707710
75477	NM_029303	Pfn3	profilin 3	protein_coding	NP_083579.1	2178800	.	0.086932
75479	NR_015573	1700012D14Rik	RIKEN cDNA 1700012D14 gene	lncRNA	.	1922729	.	0.807663
75480	NM_029305,XM_017319305	1700003F12Rik	RIKEN cDNA 1700003F12 gene	protein_coding	NP_083581.1;XP_017174794.1	1922730	.	0.443638
75482	NM_029307	Hspb9	heat shock protein, alpha-crystallin-related, B9	protein_coding	NP_083583.2	1922732	.	0.081927
75485	NM_029308	1700010B08Rik	RIKEN cDNA 1700010B08 gene	protein_coding	NP_083584.1	1922735	.	0.000000
75495	NM_029309	Morn5	MORN repeat containing 5	protein_coding	NP_083585.1	1922745	.	0.000000
75497	NM_001357159,NM_001357160,NM_001357161,NM_029310,XM_006530096,XM_006530097,XM_006530099,XM_017319783,XR_001783765	Fabp12	fatty acid binding protein 12	protein_coding	NP_001344088.1;NP_001344089.1;NP_001344090.1;NP_083586.1;XP_006530159.1;XP_006530160.1;XP_006530162.1;XP_017175272.1	1922747	.	0.000000
75500	XR_003955945	1700010H22Rik	RIKEN cDNA 1700010H22 gene	lncRNA	.	1922750	.	0.203232
75501	NR_040512	1700010K23Rik	RIKEN cDNA 1700010K23 gene	lncRNA	.	1922751	.	0.000000
75507	NM_029315	Pou5f2	POU domain class 5, transcription factor 2	protein_coding	NP_083591.1	1922757	.	0.033963
75508	NR_040326	1700017J07Rik	RIKEN cDNA 1700017J07 gene	lncRNA	.	1922758	.	0.000000
75509	NM_029316	1700020N15Rik	RIKEN cDNA 1700020N15 gene	protein_coding	NP_083592.1	1922759	.	0.000000
75514	NM_001200013,XM_006535361	1700013H16Rik	RIKEN cDNA 1700013H16 gene	protein_coding	NP_001186942.1;XP_006535424.1	1922764	.	0.038707
75516	NM_029321	Ttc32	tetratricopeptide repeat domain 32	protein_coding	NP_083597.1	1922766	.	2.463875
75522	NR_126476	1700024I08Rik	RIKEN cDNA 1700024I08 gene	lncRNA	.	1922772	.	0.062421
75528	NM_029326,XM_006508918,XM_006508919,XM_006508920,XM_030243828,XM_030243829	Tex29	testis expressed 29	protein_coding	NP_083602.1;XP_006508981.1;XP_006508982.1;XP_006508983.1;XP_030099688.1;XP_030099689.1	1922778	.	0.000000
75530	NM_001363191,NM_029327,NR_156460,XM_017314815,XR_003949526,XR_388548,XR_388549	Lyrm7	LYR motif containing 7	protein_coding	NP_001350120.2;NP_083603.4;XP_017170304.1	1922780	.	1.125602
75531	XR_003951867,XR_003951868,XR_003951869	1700025H01Rik	RIKEN cDNA 1700025H01 gene, transcript variant X2	lncRNA	.	1922781	.	0.558195
75533	NM_080637,NR_151690,NR_151691,XM_006526319,XM_011247024,XM_030250627	Nme5	NME/NM23 family member 5	protein_coding	NP_542368.2;XP_006526382.1;XP_011245326.1;XP_030106487.1	1922783	.	0.804536
75538	NM_001357650,NM_001357651,NM_001368290,NM_001368291,NM_001368292,NM_028169,XM_006541242,XM_006541243,XM_006541244,XM_006541245,XM_006541248,XM_011250907,XM_030243055,XM_030243056,XM_030243057,XM_030243058,XM_030243059	Fam71e1	family with sequence similarity 71, member E1	protein_coding	NP_001344579.1;NP_001344580.1;NP_001355219.1;NP_001355220.1;NP_001355221.1;NP_082445.1;XP_006541305.1;XP_006541306.1;XP_006541307.1;XP_006541308.1;XP_006541311.1;XP_011249209.1;XP_030098915.1;XP_030098916.1;XP_030098917.1;XP_030098918.1;XP_030098919.1	1922788	.	1.092996
75539	NR_126477	1700022H01Rik	RIKEN cDNA 1700022H01 gene	lncRNA	.	1922789	.	0.000000
75540	NM_029330,XM_006502233,XM_011240268	Fpgt	fucose-1-phosphate guanylyltransferase	protein_coding	NP_083606.2;XP_006502296.1;XP_011238570.1	1922790	.	2.906912
75541	NM_001145895,NM_029331,XM_006506720,XM_030255652	Nat8f4	N-acetyltransferase 8 (GCN5-related) family member 4	protein_coding	NP_001139367.1;NP_083607.3;XP_006506783.1;XP_030111512.1	1922791	.	0.599866
75547	NM_029332,XM_006541250,XM_006541251,XM_006541252,XM_006541253,XM_006541254,XM_006541255,XM_006541256,XM_006541257,XM_006541258,XM_006541259,XM_006541260,XM_030243060,XM_030243061,XM_030243062,XM_030243063,XM_030243064,XM_030243065	Akap13	A kinase (PRKA) anchor protein 13	protein_coding	NP_083608.1;XP_006541313.1;XP_006541314.1;XP_006541315.1;XP_006541316.1;XP_006541317.1;XP_006541318.1;XP_006541319.1;XP_006541320.1;XP_006541321.1;XP_006541322.1;XP_006541323.1;XP_030098920.1;XP_030098921.1;XP_030098922.1;XP_030098923.1;XP_030098924.1;XP_030098925.1	2676556	.	9.114853
75549	XR_001778779,XR_001778780,XR_003947392	1700019L22Rik	RIKEN cDNA 1700019L22 gene, transcript variant X3	lncRNA	.	1922799	.	0.008767
75552	NM_198414	Paqr9	progestin and adipoQ receptor family member IX	protein_coding	NP_940806.2	1922802	.	0.488114
75553	NM_001008506,NM_001160107,NM_001160108,NM_001364400,NM_001364401,NM_001364402,NM_001364403,NM_001364404,NM_001364405,NM_029334,NR_027648,XM_006516327,XM_006516328,XM_006516329,XM_006516330,XM_006516333,XM_030246973,XR_001780498,XR_001780499	Zc3h14	zinc finger CCCH type containing 14	protein_coding	NP_001008506.2;NP_001153579.1;NP_001153580.1;NP_001351329.1;NP_001351330.1;NP_001351331.1;NP_001351332.1;NP_001351333.1;NP_001351334.1;NP_083610.2;XP_006516390.3;XP_006516391.3;XP_006516392.3;XP_006516393.3;XP_006516396.1;XP_030102833.1	1919824	.	20.337696
75555	NM_023781	1700020D05Rik	RIKEN cDNA 1700020D05 gene	protein_coding	NP_076270.3	1922805	.	1.040053
75556	NM_029335,XM_006508291,XM_017312364	Cfap161	cilia and flagella associated protein 161	protein_coding	NP_083611.2;XP_006508354.1;XP_017167853.1	1922806	.	0.248237
75560	NM_029337,NM_173066,XM_006535265,XM_006535269,XM_006535270,XM_006535271,XM_006535276,XM_006535277,XM_017321129,XM_017321130,XM_017321131,XM_017321132,XM_017321133,XM_017321134,XM_017321135,XM_030254869,XM_030254870,XM_030254871,XM_030254872	Ep400	E1A binding protein p400	protein_coding	NP_083613.2;NP_775089.1;XP_006535328.1;XP_006535332.1;XP_006535333.1;XP_006535334.1;XP_006535339.1;XP_006535340.1;XP_017176618.1;XP_017176619.1;XP_017176620.1;XP_017176621.1;XP_017176622.1;XP_017176623.1;XP_017176624.1;XP_030110729.1;XP_030110730.1;XP_030110731.1;XP_030110732.1	1276124	.	8.071777
75561	XR_380261,XR_380262,XR_871595,XR_871596	1700016J18Rik	RIKEN cDNA 1700016J18 gene, transcript variant X2	lncRNA	.	1922811	.	0.091046
75563	NM_175223	Dnali1	dynein, axonemal, light intermediate polypeptide 1	protein_coding	NP_780432.1	1922813	.	0.000000
75564	NM_029338,XM_006525074,XM_006525075,XM_011246700	Rsph9	radial spoke head 9 homolog (Chlamydomonas)	protein_coding	NP_083614.1;XP_006525137.1;XP_006525138.1;XP_011245002.1	1922814	.	2.482871
75565	NM_029339,XM_017312365	Sgf29	SAGA complex associated factor 29	protein_coding	NP_083615.3;XP_017167854.1	1922815	.	5.905073
75567	NR_040741	1700027I24Rik	RIKEN cDNA 1700027I24 gene	lncRNA		1922817	.	0.000000
75568	NM_029341,XM_006520185,XM_006520186	Capsl	calcyphosine-like	protein_coding	NP_083617.2;XP_006520248.1;XP_006520249.1	1922818	.	0.000000
75570	NM_029342	Nhej1	non-homologous end joining factor 1	protein_coding	NP_083618.3	1922820	.	3.110795
75571	NM_029343,XM_006517442,XM_006517443,XM_006517444,XM_006517446,XM_017315624,XM_017315625,XM_030247441	Spata9	spermatogenesis associated 9	protein_coding	NP_083619.1;XP_006517505.1;XP_006517506.1;XP_006517507.1;XP_006517509.1;XP_017171113.1;XP_017171114.1;XP_030103301.1	1922821	.	0.000000
75572	NM_029344,XM_017314816	Acyp2	acylphosphatase 2, muscle type	protein_coding	NP_083620.1;XP_017170305.1	1922822	.	3.029359
75573	NM_029345,XM_006534388	Prr29	proline rich 29	protein_coding	NP_083621.1;XP_006534451.1	1922823	.	0.136742
75577	NM_029346	Dynap	dynactin associated protein	protein_coding	NP_083622.1	1922827	.	0.000000
75578	NM_001113412,NM_029347,XM_006503461,XM_006503468,XM_006503470,XM_006503472,XM_006503474,XM_006503475,XM_006503476,XM_011240633,XM_011240635,XM_011240636,XM_011240637,XM_011240639,XM_011240643,XM_011240644,XM_011240645,XM_017320429,XM_017320430,XM_017320431,XM_030253870,XM_030253871,XM_030253872,XM_030253873,XM_030253874,XR_001784213	Fggy	FGGY carbohydrate kinase domain containing	protein_coding	NP_001106883.1;NP_083623.1;XP_006503524.1;XP_006503531.1;XP_006503533.1;XP_006503535.1;XP_006503537.1;XP_006503538.1;XP_006503539.1;XP_011238935.1;XP_011238937.1;XP_011238938.1;XP_011238939.1;XP_011238941.1;XP_011238945.1;XP_011238946.1;XP_011238947.1;XP_017175918.1;XP_017175919.1;XP_017175920.1;XP_030109730.1;XP_030109731.1;XP_030109732.1;XP_030109733.1;XP_030109734.1	1922828	.	2.982945
75579	NR_040418	2310034G01Rik	RIKEN cDNA 2310034G01 gene	lncRNA	.	1922829	.	0.825366
75580	NM_001363178,NM_029348,XM_006534389,XM_006534390,XM_006534391,XM_006534393,XM_006534394,XM_011249293	Zbtb4	zinc finger and BTB domain containing 4	protein_coding	NP_001350107.1;NP_083624.2;XP_006534452.1;XP_006534453.1;XP_006534454.1;XP_006534456.1;XP_006534457.1;XP_011247595.1	1922830	.	7.318562
75581	NM_023784	Yipf7	Yip1 domain family, member 7	protein_coding	NP_076273.1	1922831	.	0.862942
75589	NR_037994	2310043L19Rik	RIKEN cDNA 2310043L19 gene	lncRNA	.	1922839	.	0.000000
75590	NM_029352,XM_006528331,XM_006528333	Dusp9	dual specificity phosphatase 9	protein_coding	NP_083628.3;XP_006528394.1;XP_006528396.1	2387107	.	0.706149
75593	NM_029353,XM_006506721,XM_006506722,XM_011241499	Malsu1	mitochondrial assembly of ribosomal large subunit 1	protein_coding	NP_083629.1;XP_006506784.1;XP_006506785.1;XP_011239801.1	1922843	.	9.001375
75597	NM_001127346,NM_001360140,XM_030247442,XM_030247443	Ndufaf2	NADH:ubiquinone oxidoreductase complex assembly factor 2	protein_coding	NP_001120818.1;NP_001347069.1;XP_030103302.1;XP_030103303.	1922847	.	9.629090
75599	NM_029357,XM_006526320,XM_006526321,XM_006526322,XM_006526323,XM_006526324,XM_017318005	Pcdh1	protocadherin 1	protein_coding	NP_083633.2;XP_006526383.1;XP_006526384.1;XP_006526385.1;XP_006526386.1;XP_006526387.1;XP_017173494.1	104692	.	2.041481
75600	NM_001102468,NM_138304,XM_006511529,XM_006511530,XM_006511532,XM_017313679	Calml4	calmodulin-like 4	protein_coding	NP_001095938.1;NP_612177.1;XP_006511592.1;XP_006511593.1;XP_006511595.1;XP_017169168.1	1922850	.	3.473095
75602	NR_033571	1810062O18Rik	RIKEN cDNA 1810062O18 gene	lncRNA	.	1922852	.	0.861332
75604	NM_029360	Tm4sf5	transmembrane 4 superfamily member 5	protein_coding	NP_083636.2	1922854	.	0.000000
75605	NM_152895,XM_011248090,XM_017312616,XM_017312617	Kdm5b	lysine (K)-specific demethylase 5B	protein_coding	NP_690855.2;XP_011246392.1;XP_017168105.1;XP_017168106.1	1922855	.	14.951769
75607	NM_001290311,NM_001290313,NM_029361,XM_006516974,XM_006516977,XM_006516979,XM_011244415,XM_011244416,XM_011244417,XM_011244418,XM_011244419,XM_011244420,XM_011244421,XM_011244422,XM_011244423,XM_011244424,XM_011244425,XM_011244426,XM_011244427,XM_017315626,XM_017315627,XM_030247444,XM_030247445,XM_030247446,XM_030247447,XM_030247448,XM_030247449,XM_030247450	Wnk2	WNK lysine deficient protein kinase 2	protein_coding	NP_001277240.1;NP_001277242.1;NP_083637.2;XP_006517037.1;XP_006517040.1;XP_006517042.1;XP_011242717.1;XP_011242718.1;XP_011242719.1;XP_011242720.1;XP_011242721.1;XP_011242722.1;XP_011242723.1;XP_011242724.1;XP_011242725.1;XP_011242726.1;XP_011242727.1;XP_011242728.1;XP_011242729.1;XP_017171115.1;XP_017171116.1;XP_030103304.1;XP_030103305.1;XP_030103306.1;XP_030103307.1;XP_030103308.1;XP_030103309.1;XP_03	1922857	.	1.057853
75608	NM_029362	Chmp4b	charged multivesicular body protein 4B	protein_coding	NP_083638.1	1922858	.	68.925148
75610	NM_029363	2010109A12Rik	RIKEN cDNA 2010109A12 gene	protein_coding	NP_083639.1	1922860	.	0.000000
75612	NM_001364695,NM_029364	Gns	glucosamine (N-acetyl)-6-sulfatase	protein_coding	NP_001351624.1;NP_083640.1	1922862	.	36.039513

75613	NM_001331206,NM_001331207,NM_001331208,NM_029365,XM_006541263,XM_006541264,XM_006541265,XM_017312366,XM_017312367,XM_017312368,XM_030243066,XM_030243067,XM_030243068,XM_030243069,XM_030243070,XM_030243071,XM_030243072,XM_030243073	Med25	mediator complex subunit 25	protein_coding	NP_001318135.1;NP_001318136.1;NP_001318137.1;NP_083641.1;XP_006541326.1;XP_006541327.1;XP_006541328.1;XP_017167855.1;XP_017167856.1;XP_017167857.1;XP_030098926.1;XP_030098927.1;XP_030098928.1;XP_030098929.1;XP_030098930.1;XP_030098931.1;XP_030098932.1;XP_030098933.1	1922863	.	[illegible]
75614	NR_045289	Rab26os	RAB26, member RAS oncogene family, opposite strand	lncRNA	.	1922864	.	[illegible]
75616	NM_001048250	Smim15	small integral membrane protein 15	protein_coding	NP_001041715.1	1922866	.	[illegible]
75617	NM_024266	Rps25	ribosomal protein S25	protein_coding	NP_077228.1	1922867	.	[illegible]
75619	NM_172422,XM_006496313	Fastkd2	FAST kinase domains 2	protein_coding	NP_766010.1;XP_006496376.2	1922869	.	[illegible]
75620	NM_029366	Kxd1	KxDL motif containing 1	protein_coding	NP_083642.1	1922870	.	[illegible]
75623	NM_001356296,NM_001356297,NM_029368,XM_006496315,XM_011238615,XM_011238616	Tex30	testis expressed 30	protein_coding	NP_001343225.1;NP_001343226.1;NP_083644.1;XP_006496378.1;XP_011236917.1;XP_011236918.1	1922873	.	[illegible]
75624	NM_175224	Metap1	methionyl aminopeptidase 1	protein_coding	NP_780433.1	1922874	.	[illegible]
75625	NM_023788	Mageh1	melanoma antigen, family H, 1	protein_coding	NP_076277.1	1922875	.	[illegible]
75627	NM_178392,XM_006516334,XM_006516335,XM_006516336	Snapc1	small nuclear RNA activating complex, polypeptide 1	protein_coding	NP_848479.1;XP_006516397.1;XP_006516398.1;XP_006516399.1	1922877	.	[illegible]
75641	NM_183112	1700029I15Rik	RIKEN cDNA 1700029I15 gene	protein_coding	NP_898935.1	1916742	.	[illegible]
75642	NM_029370	Spata25	spermatogenesis associated 25	protein_coding	NP_083646.1	1922892	.	[illegible]
75645	NM_029372,XM_006527422,XM_006527423,XM_006527424,XM_006527425,XM_017318303,XM_017318304	Ccdc172	coiled-coil domain containing 172	protein_coding	NP_083648.1;XP_006527485.1;XP_006527486.1;XP_006527487.1;XP_006527488.1;XP_017173792.1;XP_017173793.1	1922895	.	[illegible]
75646	NM_001166408,NM_001356534,NM_030690,XM_006520187,XM_006520188,XM_006520190,XM_006520192,XM_017316775,XM_030248808	Rai14	retinoic acid induced 14	protein_coding	NP_001159880.1;NP_001343463.1;NP_109615.1;XP_006520250.1;XP_006520251.1;XP_006520253.1;XP_006520255.1;XP_017172264.1;XP_030104668.1	1922896	.	[illegible]
75647	NM_027073,NM_029373,XM_006505194,XM_006505195,XM_017321817	Ssmem1	serine-rich single-pass membrane protein 1	protein_coding	NP_081349.1;NP_083649.1;XP_006505257.1;XP_006505258.1;XP_017177306.1	1922897	.	[illegible]
75656	NM_001163483,NM_029375	1700020A23Rik	RIKEN cDNA 1700020A23 gene	protein_coding	NP_001156955.1;NP_083651.1	1922906	.	[illegible]
75657	NM_029376	Speer4a	spermatogenesis associated glutamate (E)-rich protein 4A	protein_coding	NP_083652.2	1922907	.	[illegible]
75659	NM_001362119,NM_001362120,NM_023790,XM_030255653,XM_030255654,XM_030255655	Wdr54	WD repeat domain 54	protein_coding	NP_001349048.1;NP_001349049.1;NP_076279.1;XP_030111513.1;XP_030111514.1;XP_030111515.1	1922909	.	[illegible]
75660	NM_001290569,NM_029377,XM_011250734,XM_017312369	Lin37	lin-37 homolog (C. elegans)	protein_coding	NP_001277498.1;NP_083653.1;XP_011249036.1;XP_017167858.1	1922910	.	[illegible]
75665	NM_001080808,NR_151598,NR_151599,NR_151600,NR_151601,NR_151602,NR_151603,XM_006530487,XM_006530489,XM_006530490,XM_030254874,XR_003955719	Bicdl1	BICD family like cargo adaptor 1	protein_coding	NP_001074277.1;XP_006530550.1;XP_006530552.1;XP_006530553.1;XP_030110734.1	1922915	.	[illegible]
75668	NM_001363179,NM_145216	Rasl10a	RAS-like, family 10, member A	protein_coding	NP_001350108.1;NP_660251.1	1922918	.	[illegible]
75669	NM_001081309	Pik3r4	phosphoinositide-3-kinase regulatory subunit 4	protein_coding	NP_001074778.1	1922919	.	[illegible]
75671	NM_001361919,NM_001361920,NM_029381,XM_006516338,XM_030246974	Tex22	testis expressed gene 22	protein_coding	NP_001348848.1;NP_001348849.1;NP_083657.1;XP_006516401.1;XP_030102834.1	1922921	.	[illegible]
75677	NM_029383	Cldn22	claudin 22	protein_coding	NP_083659.1	1922927	.	[illegible]
75678	NM_001276399,NM_199056,NR_075085,XM_017315628,XR_001780827	Ippk	inositol 1,3,4,5,6-pentakisphosphate 2-kinase	protein_coding	NP_001263328.1;NP_951011.1;XP_017171117.1	1922928	.	[illegible]
75686	NM_029385,XM_006511845,XM_030244691	Nudt16	nudix (nucleoside diphosphate linked moiety X)-type motif 16	protein_coding	NP_083661.2;XP_006511908.1;XP_030100551.1	1922936	.	[illegible]
75687	NM_001081241,XM_006531461,XM_006531462,XM_006531463,XM_011248531,XM_011248532,XM_030243830	Ripor1	RHO family interacting cell polarization regulator 1	protein_coding	NP_001074710.2;XP_006531524.1;XP_006531525.1;XP_006531526.1;XP_011246833.1;XP_011246834.1;XP_030099690.1	1922937	.	[illegible]
75689	NM_001357580,NM_080846,XM_006534396,XM_011249295,XM_011249296,XM_030246406	Higd1b	HIG1 domain family, member 1B	protein_coding	NP_001344509.1;NP_543122.1;XP_006534459.1;XP_011247597.1;XP_011247598.1;XP_030102266.1	1922939	.	[illegible]
75690	NM_001290316,NM_001368881,XR_391405	Vsig10l	V-set and immunoglobulin domain containing 10 like	protein_coding	NP_001277245.1;NP_001355810.1	1922940	.	[illegible]
75691	NM_001024136,NM_001356426,NR_151464,XM_006538333,XM_006538334,XM_006538335,XM_006538336,XM_006538337,XM_006538338,XR_003955034	Anks6	ankyrin repeat and sterile alpha motif domain containing 6	protein_coding	NP_001019307.1;NP_001343355.1;XP_006538396.1;XP_006538397.1;XP_006538398.1;XP_006538399.1;XP_006538400.1;XP_006538401.1	1922941	.	[illegible]
75692	NM_001025586,NM_001025587,NR_104434,XM_006509777	Nr2c2ap	nuclear receptor 2C2-associated protein	protein_coding	NP_001020757.1;NP_001020758.1;XP_006509840.1	1922942	.	[illegible]
75693	NR_045451,NR_045452	3010001F23Rik	RIKEN cDNA 3010001F23 gene	lncRNA	.	1922943	.	[illegible]
75695	NM_021430,XM_006530491,XM_006530492,XM_011248245,XM_011248246	Rilpl1	Rab interacting lysosomal protein-like 1	protein_coding	NP_067405.1;XP_006530554.1;XP_006530555.1;XP_011246547.1;XP_011246548.1	1922945	.	[illegible]
75697	NM_001081314,XM_006511533	C2cd4b	C2 calcium-dependent domain containing 4B	protein_coding	NP_001074783.1;XP_006511596.1	1922947	.	[illegible]
75698	NM_001360074,NM_029389,NR_153399,XM_006519645,XM_006519646,XM_006519648,XM_006519650,XM_006519651,XM_006519652,XM_011245216,XM_011245218,XM_017316219,XR_00178116	Shld2	shieldin complex subunit 2	protein_coding	NP_001347003.1;NP_083665.2;XP_006519708.1;XP_006519709.1;XP_006519711.1;XP_006519713.1;XP_006519714.1;XP_006519715.1;XP_011243518.1;XP_011243520.1;XP_017171708.1	1922948	.	[illegible]
75705	NM_145625	Eif4b	eukaryotic translation initiation factor 4B	protein_coding	NP_663600.2	95304	.	[illegible]
75710	NM_029397,NM_170598	Rbm12	RNA binding motif protein 12	protein_coding	NP_083673.3;NP_733486.2	1922960	.	[illegible]
75712	NM_001290679,NM_029398,XM_006495579,XM_006495581	Tmem14a	transmembrane protein 14A	protein_coding	NP_001277608.1;NP_083674.2;XP_006495642.1;XP_006495644.1	1922962	.	[illegible]
75717	NM_001161618,NM_027807,XM_017313680	Cul5	cullin 5	protein_coding	NP_001155090.2;NP_082083.3;XP_017169169.1	1922967	.	[illegible]
75718	NM_029401,XM_006539241,XM_006539243,XM_006539244,XM_006539245,XM_011250349,XM_017320433	Vwa5b1	von Willebrand factor A domain containing 5B1	protein_coding	NP_083677.1;XP_006539304.1;XP_006539306.1;XP_006539307.1;XP_006539308.1;XP_011248651.1;XP_017175922.1	1922968	.	[illegible]
75721	NM_183113,XM_006500335,XM_006500336,XM_017319307,XM_017319308	4932414N04Rik	RIKEN cDNA 4932414N04 gene	protein_coding	NP_898936.1;XP_006500398.1;XP_006500399.1;XP_017174796.1;XP_017174797.1	1922971	.	[illegible]
75723	NM_001081395,XM_006510666,XM_006510667,XM_006510668,XM_006510669,XM_006510670,XM_030244692,XM_030244693	Amotl1	angiomotin-like 1	protein_coding	NP_001074864.1;XP_006510729.1;XP_006510730.1;XP_006510731.1;XP_006510732.1;XP_006510733.1;XP_030100552.1;XP_03010055	1922973	.	[illegible]
75725	NM_001168382,NM_001362122,NM_001362123,NM_029404,XM_006505196,XM_006505197,XM_006505198,XM_006505199,XM_006505201,XM_006505202,XM_011241085,XM_017321819,XM_030255656,XM_030255657,XR_001785183,XR_003956260,XR_003956261,XR_003956263,XR_003956264,XR_377293	Phf14	PHD finger protein 14	protein_coding	NP_001161854.1;NP_001349051.1;NP_001349052.1;NP_083680.2;XP_006505259.1;XP_006505260.1;XP_006505261.1;XP_006505262.1;XP_006505264.1;XP_006505265.1;XP_011239387.1;XP_017177308.1;XP_030111516.1;XP_030111517.1	1923539	.	[illegible]
75729	NM_029407,XM_006521510,XM_011245760,XM_011245761,XM_011245764,XM_011245765,XM_017316777,XM_017316778,XM_017316779,XM_017316781,XM_017316783,XM_017316784,XM_017316785,XM_030248809,XM_030248810,XM_030248811,XM_030248813,XR_001781573,XR_001781574,XR_003951444	Fam227a	family with sequence similarity 227, member A	protein_coding	NP_083683.1;XP_006521573.1;XP_011244062.1;XP_011244063.1;XP_011244066.1;XP_011244067.1;XP_017172266.1;XP_017172267.1;XP_017172268.1;XP_017172270.1;XP_017172272.1;XP_017172273.1;XP_017172274.1;XP_030104669.1;XP_030104670.1;XP_030104671.1;XP_030104673.1	1922979	.	[illegible]
75731	NM_001048060,NM_198004,XM_030247451	Idnk	idnK gluconokinase homolog (E. coli)	protein_coding	NP_001041525.1;NP_932121.2;XP_030103311.1	1922981	.	[illegible]
75732	NM_029408,XM_011248247	Iqcd	IQ motif containing D	protein_coding	NP_083684.1;XP_011246549.1	1922982	.	[illegible]
75734	NM_001310695,NM_001310697,NM_001310699,NM_029409,XM_006496556,XM_006496557,XM_006496558,XM_006496559,XM_006496560,XM_006496562,XM_006496563,XM_006496564,XM_006496565,XM_006496566,XM_006496567,XM_006496568,XM_006496569,XM_006496572,XM_017312643,XM_017312646,XM_017312652,XM_030243330,XM_030243336,XM_030243337	Mff	mitochondrial fission factor	protein_coding	NP_001297624.1;NP_001297626.1;NP_001297628.1;NP_083685.2;XP_006496619.1;XP_006496620.1;XP_006496621.1;XP_006496622.1;XP_006496623.1;XP_006496625.1;XP_006496626.1;XP_006496627.1;XP_006496628.1;XP_006496629.1;XP_006496630.1;XP_006496631.1;XP_006496632.1;XP_006496635.1;XP_017168132.1;XP_017168135.1;XP_017168141.1;XP_030099190.1;XP_030099196.1;XP_0300	1922984	.	[illegible]
75735	NM_001114339,NM_023792,XM_006527427,XM_030251126	Pank1	pantothenate kinase 1	protein_coding	NP_001107811.1;NP_076281.1;XP_006527490.1;XP_030106986.1	1922985	.	[illegible]
75736	NM_029410,XM_006541267,XR_003946575	Bcl2l12	BCL2-like 12 (proline rich)	protein_coding	NP_083686.1;XP_006541330.1	1922986	.	[illegible]
75739	NM_001081287,NM_001161620,NM_001360860,XM_006526325,XM_006526326,XM_006526327,XM_006526329,XM_006526330,XM_011247025,XM_011247026,XM_011247027,XM_017318006,XM_017318008,XM_017318009,XM_017318010	Mpp7	membrane protein, palmitoylated 7 (MAGUK p55 subfamily member 7)	protein_coding	NP_001074756.2;NP_001155092.1;NP_001347789.1;XP_006526388.1;XP_006526389.1;XP_006526390.1;XP_006526392.1;XP_006526393.1;XP_011245327.1;XP_011245328.1;XP_011245329.1;XP_017173495.1;XP_017173497.1;XP_017173498.1;XP_017173499.1	1922989	.	[illegible]
75740	NM_001167748,NM_001357198,NM_029412,NR_151636,NR_151637,NR_151638,XM_006535559,XM_006535561,XM_006535562,XM_006535563,XM_006535565,XM_017319784,XM_017319785,XM_017319787,XM_017319788,XM_017319789,XM_017319790,XM_030252865,XM_030252866,XM_030252867,XR_001783766,XR_00395	Egfem1	EGF-like and EMI domain containing 1	protein_coding	NP_001161220.1;NP_001344127.1;NP_083688.1;XP_006535622.1;XP_006535624.1;XP_006535625.1;XP_006535626.1;XP_006535628.1;XP_017175273.1;XP_017175274.1;XP_017175276.1;XP_017175277.1;XP_017175278.1;XP_017175279.1;XP_030108725.1;XP_030108726.1;XP_030108727.1	1922990	.	[illegible]
75744	NM_001160345,NR_027704	Svip	small VCP/p97-interacting protein	protein_coding	NP_001153817.1	1922994	.	[illegible]
75745	NR_028261	Rian	RNA imprinted and accumulated in nucleus	lncRNA	.	1922995	.	[illegible]
75746	NM_001193309,NM_029413,XM_006528618,XM_006528619,XM_011247761	Morc4	microrchidia 4	protein_coding	NP_001180238.1;NP_083689.2;XP_006528681.1;XP_006528682.1;XP_011246063.1	1922996	.	[illegible]
75747	NM_030261	Sesn3	sestrin 3	protein_coding	NP_084537.2	1922997	.	[illegible]
75750	NM_029415,XM_006535278	Slc10a6	solute carrier family 10 (sodium/bile acid cotransporter family), member	protein_coding	NP_083691.1;XP_006535341.1	1923000	.	[illegible]
75751	NM_001360078,NM_024267,NR_153401,NR_153402,XM_0173162	Ipo4	importin 4	protein_coding	NP_001347007.1;NP_077229.4;XP_017171709.1	1923001	.	[illegible]
75753	NM_029416	Klf17	Kruppel-like factor 17	protein_coding	NP_083692.2	2181068	.	[illegible]
75758	NM_029418	9130401M01Rik	RIKEN cDNA 9130401M01 gene	protein_coding	NP_083694.1	1923008	.	[illegible]
75761	NM_001164640,NM_029419,XM_011245766	Apol7a	apolipoprotein L 7a	protein_coding	NP_001158112.1;NP_083695.2;XP_011244068.1	1923011	.	[illegible]
75763	NM_001165980,NM_001165981,NM_001165982,NM_198005,XM_006500339,XM_006500340,XM_030252168,XR_001783197,XR_374535	Dcaf17	DDB1 and CUL4 associated factor 17	protein_coding	NP_001159452.1;NP_001159453.1;NP_001159454.1;NP_932122.2;XP_006500402.1;XP_006500403.1;XP_030108028.1	1923013	.	[illegible]
75764	NM_029420,NR_033446	Slx1b	SLX1 structure-specific endonuclease subunit homolog B (S. cerevisiae)	protein_coding	NP_083696.2	1915220	.	[illegible]
75766	NM_001289506,NM_001289508,NM_001289512,NM_001289513,NM_029422,XM_006521519	Dcstamp	dendrocyte expressed seven transmembrane protein	protein_coding	NP_001276435.1;NP_001276437.1;NP_001276441.1;NP_001276442.1;NP_083698.1;XP_006521582.1	1923016	.	[illegible]
75767	NM_001080813,NM_029423	Rab11fip1	RAB11 family interacting protein 1 (class I)	protein_coding	NP_001074282.1;NP_083699.2	1923017	.	[illegible]
75768	NR_130160	4833422M21Rik	RIKEN cDNA 4833422M21 gene	lncRNA	.	1923018	.	[illegible]
75769	NM_001305451,NM_029425,XM_006502235	Plppr5	phospholipid phosphatase related 5	protein_coding	NP_001292380.1;NP_083701.2;XP_006502298.1	1923019	.	[illegible]
75770	NM_001009929,NM_001009930,NM_001276763,NM_029426	Brsk2	BR serine/threonine kinase 2	protein_coding	NP_001009929.1;NP_001009930.1;NP_001263692.1;NP_083702.1	1923020	.	[illegible]
75773	NM_029428,XM_006531464,XM_006531467	Adad2	adenosine deaminase domain containing 2	protein_coding	NP_083704.1;XP_006531527.1;XP_006531530.1	1923023	.	[illegible]
75777	NM_029430,XM_006520194,XM_017316786,XM_030248814,XM_030248815	Ttc23l	tetratricopeptide repeat domain 23-like	protein_coding	NP_083706.1;XP_006520257.1;XP_017172275.1;XP_030104674.1;XP_030104675.1	1923027	.	[illegible]
75778	NM_029431,XM_030252868	Them4	thioesterase superfamily member 4	protein_coding	NP_083707.1;XP_030108728.1	1923028	.	[illegible]
75782	NM_027448,NM_029434,XM_006511534,XM_006511535,XM_006511536,XM_006511537,XM_006511538,XM_011242832,XM_011242833,XM_030244694	Lca5	Leber congenital amaurosis 5 (human)	protein_coding	NP_081724.1;NP_083710.2;XP_006511597.1;XP_006511598.1;XP_006511599.1;XP_006511600.1;XP_006511601.1;XP_011241134.1;XP_011241135.1;XP_030100554.1	1923032	.	[illegible]
75783	NR_073013	4930431F12Rik	RIKEN cDNA 4930431F12 gene	lncRNA	.	1923033	.	[illegible]
75784	NM_001024614,XM_006535279,XM_006535280	Cfap299	cilia and flagella associated protein 299	protein_coding	NP_001019785.1;XP_006535342.1;XP_006535343.1	1916571	.	[illegible]
75785	NM_029436,XM_006522687	Klhl24	kelch-like 24	protein_coding	NP_083712.4;XP_006522750.1	1923035	.	[illegible]
75786	NM_001165989,NM_029437,XM_006500342,XM_006500343,XM_030252169,XM_030252170	Ckap5	cytoskeleton associated protein 5	protein_coding	NP_001159461.1;NP_083713.2;XP_006500405.1;XP_006500406.1;XP_030108029.1;XP_030108030.1	1923036	.	[illegible]
75788	NM_001038627,NM_029438,XM_006504884,XM_006504888,XM_011241016,XM_011241017	Smurf1	SMAD specific E3 ubiquitin protein ligase 1	protein_coding	NP_001033716.1;NP_083714.3;XP_006504947.1;XP_006504951.1;XP_011239318.1;XP_011239319.1	1923038	.	[illegible]
75789	NR_045660	4930444M15Rik	RIKEN cDNA 4930444M15 gene	lncRNA	.	1923039	.	[illegible]
75796	NM_029441,XM_017312996	Cdyl2	chromodomain protein, Y chromosome-like 2	protein_coding	NP_083717.1;XP_017168485.1	1923046	.	[illegible]
75801	NM_029444,XM_011244200,XM_011244201,XM_011244202,XM_011244203,XM_011244205,XM_011244206,XM_017315237,XM_017315238,XM_017315239,XM_017315240,XR_001780500,XR_001780501,XR_001780502,XR_001780504	4930447C04Rik	RIKEN cDNA 4930447C04 gene	protein_coding	NP_083720.1;XP_011242502.1;XP_011242503.1;XP_011242504.1;XP_011242505.1;XP_011242507.1;XP_011242508.1;XP_017170726.1;XP_017170727.1;XP_017170728.1;XP_017170729.1	1923051	.	[illegible]
75805	NM_029447,XM_011244699,XM_011244700,XM_011244701,XM_030247452,XR_873797	Nln	neurolysin (metallopeptidase M3 family)	protein_coding	NP_083723.1;XP_011243001.1;XP_011243002.1;XP_011243003.1;XP_030103312.1	1923055	.	[illegible]
75812	NM_001159640,NM_001159641,NM_001289611,NM_175225,NR_110350,NR_110351,NR_110352,XM_011239829,XM_017319309,XM_017319310,XM_017319311,XM_017319312,XM_017319313,XM_017319314,XM_017319315,XM_030252171,XM_030252172,XM_030252173,XM_030252174,XM_030252175,XM_030252176,XR_001783198,XR_003953732,XR_003953733,XR_866346,XR_866347	Tasp1	taspase, threonine aspartase 1	protein_coding	NP_001153112.1;NP_001153113.1;NP_001276540.1;NP_780434.1;XP_011238131.1;XP_017174798.1;XP_017174799.1;XP_017174800.1;XP_017174801.1;XP_017174802.1;XP_017174803.1;XP_017174804.1;XP_030108031.1;XP_030108032.1;XP_030108033.1;XP_030108034.1;XP_030108035.1;XP_030108036.1	1923062	.	[illegible]
75820	NM_029453,XM_006497022,XR_003947245,XR_373620	Wdr64	WD repeat domain 64	protein_coding	NP_083729.2;XP_006497085.1	1923070	.	[illegible]
75822	NR_045661	4930524C18Rik	RIKEN cDNA 4930524C18 gene	lncRNA	.	1923072	.	[illegible]
75823	NM_029455,XM_006500348,XM_006500349,XM_006500350	Fam227b	family with sequence similarity 227, member B	protein_coding	NP_083731.2;XP_006500411.1;XP_006500412.1;XP_006500413.1	1923073	.	[illegible]
75826	NM_001357424,NM_029457,NR_027488,XM_006522688,XM_006522689,XM_030249323,XM_030249324,XR_003951844	Senp2	SUMO/sentrin specific peptidase 2	protein_coding	NP_001344353.1;NP_083733.1;XP_006522751.1;XP_006522752.1;XP_030105183.1;XP_030105184.1	1923076	.	[illegible]
75828	NM_029458,XM_011243777,XM_011243779,XM_011243780,XM_011243781,XM_017314817,XM_017314818	Hormad2	HORMA domain containing 2	protein_coding	NP_083734.1;XP_011242079.1;XP_011242081.1;XP_011242082.1;XP_011242083.1;XP_017170306.1;XP_017170307.1	1923078	.	[illegible]
75829	NM_029459,XM_017318634	Prame	preferentially expressed antigen in melanoma	protein_coding	NP_083735.2;XP_017174123.1	1923079	.	[illegible]
75835	NR_004441	Vmn2r-ps54	vomeronasal 2, receptor, pseudogene 54	transcribed_pseudogene	.	1351350	.	[illegible]
75836	NR_131031	Atxn7l1os2	ataxin 7-like 1, opposite strand 2	lncRNA	.	1923086	.	[illegible]
75841	NM_175226,XM_017316787	Rnf139	ring finger protein 139	protein_coding	NP_780435.1;XP_017172276.1	1923091	.	[illegible]
75847	NM_001289502,NM_001289503,NM_001289504,NM_001364305,NM_178629,XM_011243907,XM_011243909,XM_011243910	Crppa	CDP-L-ribitol pyrophosphorylase A	protein_coding	NP_001276431.1;NP_001276432.1;NP_001276433.1;NP_001351234.1;NP_848744.2;XP_011242209.1;XP_011242211.1;XP_011242212.1	1923097	.	[illegible]
75858	NR_001585	Speer4cos	spermatogenesis associated glutamate (E)-rich protein 4C, opposite strand transcript	transcribed_pseudogene	.	1923108	.	[illegible]
75860	NM_001310763,NM_001310764,NM_001310765,NM_029464,XM_006504945,XM_006504946,XM_030254875,XR_001784754	Tex26	testis expressed 26	protein_coding	NP_001297692.1;NP_001297693.1;NP_001297694.1;NP_083740.1;XP_006505008.1;XP_006505009.1;XP_030110735.1	1923110	.	[illegible]
75863	NM_029465,XR_870085	Clec4g	C-type lectin domain family 4, member g	protein_coding	NP_083741.1	1923113	.	[illegible]
75869	NM_029466,XM_017319316	Arl5b	ADP-ribosylation factor-like 5B	protein_coding	NP_083742.3;XP_017174805.1	1923119	.	[illegible]
75870	NM_029467,XM_006534398,XM_006534399,XM_006534400,XM_006534401	Tcam1	testicular cell adhesion molecule 1	protein_coding	NP_083743.2;XP_006534461.1;XP_006534462.1;XP_006534463.1;XP_006534464.1	1923120	.	[illegible]
75871	NM_001167946,NM_001286391,NM_001286393,NM_001363380,NM_001363381,NM_001363382,NM_001363383,NM_001363384,NM_001363385,NM_001363386,NM_029468,XM_011248534,XM_030243831,XM_030243832,XM_030243833	Zfp821	zinc finger protein 821	protein_coding	NP_001161418.1;NP_001273320.1;NP_001273322.1;NP_001350309.1;NP_001350310.1;NP_001350311.1;NP_001350312.1;NP_001350313.1;NP_001350314.1;NP_001350315.1;NP_083744.2;XP_011246836.1;XP_030099691.1;XP_030099692.1;XP_030099693.1	1923121	.	[illegible]
75878	NR_131036	4930579P08Rik	RIKEN cDNA 4930579P08 gene	lncRNA	.	1923128	.	[illegible]
75879	NR_152118	4930589L23Rik	RIKEN cDNA 4930589L23 gene	lncRNA	.	1923129	.	[illegible]
75880	NR_045070	4930592A05Rik	RIKEN cDNA 4930592A05 gene	lncRNA	.	1923130	.	[illegible]
75881	NR_029444	4930579K19Rik	RIKEN cDNA 4930579K19 gene	lncRNA	.	1923131	.	[illegible]
75883	XR_003947765,XR_003947766,XR_373301,XR_865324,XR_865325,XR_865327	4930568A12Rik	RIKEN cDNA 4930568A12 gene, transcript variant X4	lncRNA	.	1923133	.	[illegible]
75886	NM_029472,XM_006514271,XM_030245345	Gstt4	glutathione S-transferase, theta 4	protein_coding	NP_083748.3;XP_006514334.1;XP_030101205.1	1923136	.	[illegible]
75887	NR_131052	4930586N03Rik	RIKEN cDNA 4930586N03 gene	lncRNA	.	1923137	.	[illegible]
75894	NM_001290811,NM_001290812,NM_001355748,NM_029475,XM_006500351,XM_006500352,XM_006500353,XM_006500359,XM_006500360,XM_011239837,XM_030252178,XM_030252179,XM_030252180,XM_030252181,XM_030252182,XM_030252183	Adal	adenosine deaminase-like	protein_coding	NP_001277740.1;NP_001277741.1;NP_001342677.1;NP_083751.1;XP_006500414.1;XP_006500415.1;XP_006500416.1;XP_006500422.1;XP_006500423.1;XP_011238139.1;XP_030108038.1;XP_030108039.1;XP_030108040.1;XP_030108041.1;XP_030108042.1;XP_03010804	1923144	.	[illegible]
75901	NM_133761,XM_006519655,XM_006519656	Dcp1a	decapping mRNA 1A	protein_coding	NP_598522.3;XP_006519718.1;XP_006519719.1	1923151	.	[illegible]
75905	NM_175228,NR_132634,XM_006527708,XM_006527709,XR_386875,XR_386876,XR_878033,XR_878034	Dipk2b	divergent protein kinase domain 2B	protein_coding	NP_780437.1;XP_006527771.1;XP_006527772.1	1923155	.	[illegible]
75906	NM_001081428,XM_006512889,XM_006512890,XM_006512891,XM_006512893,XM_006512894,XM_006512895,XM_006512896,XM_006512897,XM_006512898,XM_011243225,XM_017314133,XM_017314134,XM_017314135,XM_017314136,XM_030245346,XM_030245347,XM_030245348,XM_030245349,XM_030245350,XR_003948817,XR_380088	Fam184a	family with sequence similarity 184, member A	protein_coding	NP_001074897.1;XP_006512952.1;XP_006512953.1;XP_006512954.1;XP_006512956.1;XP_006512957.1;XP_006512958.1;XP_006512959.1;XP_006512960.1;XP_006512961.1;XP_011241527.1;XP_017169622.1;XP_017169623.1;XP_017169624.1;XP_017169625.1;XP_030101206.1;XP_030101207.1;XP_030101208.1;XP_030101209.1;XP_030101210.1	1923156	.	[illegible]
75909	NM_001356531,NM_029478,XM_006534402	Vmp1	vacuole membrane protein 1	protein_coding	NP_001343460.1;NP_083754.2;XP_006534465.1	1923159	.	[illegible]
75913	NR_038053	4930579G18Rik	RIKEN cDNA 4930579G18 gene	lncRNA	.	1923163	.	[illegible]
75914	NM_177077,XM_006506728,XM_006506729,XM_011241500,XM_017321822	Exoc6b	exocyst complex component 6B	protein_coding	NP_796051.2;XP_006506791.1;XP_006506792.1;XP_011239802.1;XP_017177311.1	1923164	.	[illegible]
75915	NR_156706	Bloc1s6os	biogenesis of lysosomal organelles complex-1, subunit 6, pallidin, opposite strand	lncRNA	.	1923165	.	[illegible]
75939	NM_029482	4930579G24Rik	RIKEN cDNA 4930579G24 gene	protein_coding	NP_083758.1	1923189	.	[illegible]
75943	XR_003951199,XR_874605,XR_874606	4930572G02Rik	RIKEN cDNA 4930572G02 gene, transcript variant X3	lncRNA	.	1923193	.	[illegible]
75946	NR_040612	4930583P06Rik	RIKEN cDNA 4930583P06 gene	lncRNA	.	1923196	.	[illegible]
75951	NR_045991	4930578M01Rik	RIKEN cDNA 4930578M01 gene	lncRNA	.	1923201	.	[illegible]
75953	NM_029489,NR_028060,XM_017319791	Samd7	sterile alpha motif domain containing 7	protein_coding	NP_083765.2;XP_017175280.1	1923203	.	[illegible]
75956	NM_001368687,NM_175229,XM_006525077,XM_006525078,XM_006525079,XM_011246701,XM_011246702,XM_011246704,XM_017317708,XM_017317709,XM_017317710,XM_017317711,XM_017317712,XM_017317713,XM_017317714,XM_017317715,XM_030250128,XM_030250129,XM_030250130,XM_030250131,XM_030250132,XM_030250133	Srrm2	serine/arginine repetitive matrix 2	protein_coding	NP_001355616.1;NP_780438.2;XP_006525140.1;XP_006525141.1;XP_006525142.1;XP_011245003.1;XP_011245004.1;XP_011245006.1;XP_017173197.1;XP_017173198.1;XP_017173199.1;XP_017173200.1;XP_017173201.1;XP_017173202.1;XP_017173203.1;XP_017173204.1;XP_030105988.1;XP_030105989.1;XP_030105990.1;XP_030105991.1;XP_030105992.1;XP_030105993.1	1923206	.	[illegible]
75957	NR_029382	Mir17hg	Mir17 host gene (non-protein coding)	lncRNA	.	1923207	.	[illegible]
75958	NR_131019	5033403F01Rik	RIKEN cDNA 5033403F01 gene	lncRNA	.	1923208	.	[illegible]
75964	NM_001361188,NM_029491,NM_177038,XM_006526333,XM_011247028,XM_011247029,XM_011247030,XM_011247031,XM_011247032,XM_011247033,XM_017318011,XM_017318012,XM_030250628,XM_030250629,XM_030250630,XM_030250631,XR_001782396,XR_001782397,XR_001782398,XR_001782399	Trappc8	trafficking protein particle complex 8	protein_coding	NP_001348117.1;NP_083767.2;NP_796012.2;XP_006526396.1;XP_011245330.1;XP_011245331.1;XP_011245332.1;XP_011245333.1;XP_011245334.1;XP_011245335.1;XP_017173500.1;XP_017173501.1;XP_030106488.1;XP_030106489.1;XP_030106490.1;XP_030106491.1	2443008	.	[illegible]
75965	NM_001360097,NM_001360098,NM_029492,XM_006519658,XM_006519659,XM_006519660,XM_030248089,XM_030248090,XR_001781165,XR_001781166,XR_003950906,XR_003950907,XR_00395	Zdhhc20	zinc finger, DHHC domain containing 20	protein_coding	NP_001347026.1;NP_001347027.1;NP_083768.4;XP_006519721.1;XP_006519722.1;XP_006519723.1;XP_030103949.1;XP_030103950.1	1923215	.	[illegible]
75973	NM_001358562,XM_006512905,XM_006512907,XM_011243226,XM_011243229,XM_011243230,XM_011243234,XM_017314137,XM_017314138,XM_030245351,XR_001779633,XR_003948818,XR_87	Ccdc162	coiled-coil domain containing 162	protein_coding	NP_001345491.1;XP_006512968.1;XP_006512970.1;XP_011241528.1;XP_011241531.1;XP_011241532.1;XP_011241536.1;XP_017169626.1;XP_017169627.1;XP_030101211.1	1923223	.	[illegible]
75974	NM_001009947,XM_006541406,XM_006541407,XM_006541408	Dock11	dedicator of cytokinesis 11	protein_coding	NP_001009947.2;XP_006541469.1;XP_006541470.1;XP_006541471.	1923224	.	[illegible]
75985	NM_029494,XM_006508293,XM_006508294,XM_030243074,XM_030243075	Rab30	RAB30, member RAS oncogene family	protein_coding	NP_083770.2;XP_006508356.1;XP_006508357.1;XP_030098934.1;XP_030098935.1	1923235	.	[illegible]
75986	NM_001081408,XM_006539246	Agmat	agmatine ureohydrolase (agmatinase)	protein_coding	NP_001074877.1;XP_006539309.1	1923236	.	[illegible]
75990	XR_001780546,XR_872469,XR_872470	5033421B08Rik	RIKEN cDNA 5033421B08 gene, transcript variant X1	lncRNA	.	1923240	.	[illegible]
75991	NM_001113423,NM_153567,XM_006504171	Slain2	SLAIN motif family, member 2	protein_coding	NP_001106894.1;NP_705795.2;XP_006504234.1	1923241	.	[illegible]
75995	NR_138577,NR_138578	5033417F24Rik	RIKEN cDNA 5033417F24 gene	lncRNA	.	1923245	.	[illegible]
75996	NR_131041	5033428I22Rik	RIKEN cDNA 5033428I22 gene	lncRNA	.	1923246	.	[illegible]
76000	XR_141159	5033430I15Rik	RIKEN cDNA 5033430I15 gene	lncRNA	.	1923250	.	[illegible]
76001	XR_388120	5033426O07Rik	RIKEN cDNA 5033426O07 gene	lncRNA	.	1923251	.	[illegible]
76007	NM_001360643,NM_029498,NM_177627,XM_011245219,XM_011245220,XM_011245221,XM_030248091	Zmym2	zinc finger, MYM-type 2	protein_coding	NP_001347572.1;NP_083774.2;NP_808295.2;XP_011243521.1;XP_011243522.1;XP_011243523.1;XP_030103951.1	1923257	.	[illegible]
76014	NM_001029993,NM_001029994,NM_001310650,XM_006531469,XM_006531470,XM_011248538,XM_030243834	Zc3h18	zinc finger CCCH-type containing 18	protein_coding	NP_001025164.1;NP_001025165.1;NP_001297579.1;XP_006531532.1;XP_006531533.1;XP_011246840.1;XP_030099694.1	1923264	.	[illegible]
76022	NM_001242372,NM_027389,XM_006502237,XM_017319793,XM_017319796,XM_017319797,XM_017319800	Gon4l	gon-4-like (C.elegans)	protein_coding	NP_001229301.1;NP_081665.2;XP_006502300.1;XP_017175282.1;XP_017175285.1;XP_017175286.1;XP_017175289.1	1917579	.	[illegible]
76024	NR_024599	Gm11346	predicted gene 11346	transcribed_pseudogene	.	1923274	.	[illegible]
76025	NM_001025617,NM_001025618,NM_001267591,NM_001267592,NM_029502,XM_006534404,XM_006534405,XM_006534406,XM_006534407,XM_006534408,XM_006534409,XM_006534410,XM_030246407,XM_030246408,XM_030246409	Cant1	calcium activated nucleotidase 1	protein_coding	NP_001020788.1;NP_001020789.1;NP_001254520.1;NP_001254521.1;NP_083778.2;XP_006534467.1;XP_006534468.1;XP_006534469.1;XP_006534470.1;XP_006534471.1;XP_006534472.1;XP_006534473.1;XP_030102267.1;XP_030102268.1;XP_030102269.1	1923275	.	[illegible]
76041	NM_001168386,NM_183115,XM_011244703,XM_011244704,XM_011244705,XM_017315630	Ccdc125	coiled-coil domain containing 125	protein_coding	NP_001161858.1;NP_898938.4;XP_011243005.1;XP_011243006.1;XP_011243007.1;XP_017171119.2	1923291	.	[illegible]
76044	NM_133762,XM_006516349,XM_006516350,XM_006516351,XM_006516352,XM_006516353,XM_006516354,XM_006516355,XM_006516356	Ncapg2	non-SMC condensin II complex, subunit G2	protein_coding	NP_598523.3;XP_006516412.1;XP_006516413.1;XP_006516414.1;XP_006516415.1;XP_006516416.1;XP_006516417.1;XP_006516418.1;XP_006516419.1	1923294	.	[illegible]
76051	NM_172672	Ganc	glucosidase, alpha; neutral C	protein_coding	NP_766260.2	1923301	.	[illegible]
76055	NM_023799,NR_151594,XM_017318305	Oga	O-GlcNAcase	protein_coding	NP_076288.1;XP_017173794.1	1932139	.	[illegible]
76062	NR_038060	5830428M24Rik	RIKEN cDNA 5830428M24 gene	lncRNA	.	1923312	.	[illegible]

76071	NM_001347348,NM_178394,XM_006504174,XM_011240784,XM_011240785,XM_011240786,XM_011240787,XM_011240788,XM_011240789,XM_011240790,XM_011240791,XM_017321137,XM_017321138,XM_017321139,XM_030254876,XM_030254877,XM_030254878,XM_030254879,XM_030254880,XM_030254881	Jakmip1	janus kinase and microtubule interacting protein 1	protein_coding	NP_001334277.1;NP_848481.2;XP_006504237.1;XP_011239086.1;XP_011239087.1;XP_011239088.1;XP_011239089.1;XP_011239090.1;XP_011239091.1;XP_011239092.1;XP_011239093.1;XP_017176626.1;XP_017176627.1;XP_017176628.1;XP_030110736.1;XP_030110737.1;XP_030110738.1;XP_030110739.1;XP_030110740.1;XP_030110	1923321	.
76072	NM_153504,XM_006538339,XM_030253875,XM_030253876	Rnf183	ring finger protein 183	protein_coding	NP_705724.1;XP_006538402.1;XP_030109735.1;XP_030109736.1	1923322	.
76073	NM_001368690,NM_001368691,NM_001368692,NM_001368693,NM_029508,XM_011247392	Pcgf5	polycomb group ring finger 5	protein_coding	NP_001355619.1;NP_001355620.1;NP_001355621.1;NP_001355622.1;NP_083784.1;XP_011245694.1	1923505	.
76074	NM_029509,XM_006535281,XM_006535283,XM_011249593,XM_017321140,XM_017321141,XM_017321142,XR_389295	Gbp8	guanylate-binding protein 8	protein_coding	NP_083785.3;XP_006535344.1;XP_006535346.1;XP_011247895.1;XP_017176629.1;XP_017176630.1;XP_017176631.1	1923324	.
76080	NM_001305803,NM_029512,NM_181734,NR_131225,XM_006500361,XM_006500362,XM_017319317,XR_003953734,XR_003953735,XR_003953736,XR_374538	Ttpal	tocopherol (alpha) transfer protein-like	protein_coding	NP_001292732.1;NP_083788.2;NP_859423.2;XP_006500424.1;XP_006500425.1;XP_017174806.1	1923330	.
76082	XR_003947515,XR_003947516	5830468F06Rik	RIKEN cDNA 5830468F06 gene, transcript variant X1	lncRNA	.	1923332	.
76088	NM_028785,XM_006527435,XM_006527436,XM_011247397	Dock8	dedicator of cytokinesis 8	protein_coding	NP_083061.2;XP_006527498.1;XP_006527499.1;XP_011245699.1	1921396	.
76089	NM_001099624,NM_001310536,XM_006502250,XM_006502252,XM_011240276,XM_011240277,XM_030252869	Rapgef2	Rap guanine nucleotide exchange factor (GEF) 2	protein_coding	NP_001093094.2;NP_001297465.2;XP_006502313.1;XP_006502315.1;XP_011238578.1;XP_011238579.1;XP_030108729.1	2659071	.
76100	NR_073359	5830454E08Rik	RIKEN cDNA 5830454E08 gene	lncRNA	.	1923350	.
76108	NM_029519	Rap2a	RAS related protein 2a	protein_coding	NP_083795.2	97855	.
76113	NM_080420,XM_011249298	Lpo	lactoperoxidase	protein_coding	NP_536345.2;XP_011247600.1	1923363	.
76117	NM_001301831,NM_001301832,NM_001364029,NM_153820,XM_006498397,XM_006498398,XM_006498399,XM_006498402,XM_006498403,XR_001783199	Arhgap15	Rho GTPase activating protein 15	protein_coding	NP_001288760.1;NP_001288761.1;NP_001350958.1;NP_722542.2;XP_006498460.1;XP_006498461.1;XP_006498462.1;XP_006498465.1;XP_006498466.1	1923367	.
76123	NM_029522,XM_006502256	Gpsm2	G-protein signalling modulator 2 (AGS3-like, C. elegans)	protein_coding	NP_083798.2;XP_006502319.1	1923373	.
76130	NM_152822,XM_006528334,XM_006528335,XM_006528336,XM_017318635,XM_017318636,XR_878108,XR_878109	Las1l	LAS1-like (S. cerevisiae)	protein_coding	NP_690035.2;XP_006528397.1;XP_006528398.1;XP_006528399.1;XP_017174124.1;XP_017174125.1	1923380	.
76131	NM_001172092,NM_001172093,NM_029523,XR_867244	Depdc1a	DEP domain containing 1a	protein_coding	NP_001165563.1;NP_001165564.1;NP_083799.2	1923381	.
76132	NM_175234,XM_006538340	Faxc	failed axon connections homolog	protein_coding	NP_780443.2;XP_006538403.1	1923382	.
76133	NR_029446	6230400D17Rik	RIKEN cDNA 6230400D17 gene	lncRNA	.	1923383	.
76137	NM_001081059,XM_006516983,XR_001780828,XR_003950490	Mcur1	mitochondrial calcium uniporter regulator 1	protein_coding	NP_001074528.1;XP_006517046.1	1923387	.
76138	NM_001162956,XM_006514273,XM_006514275,XM_017314139,XM_017314140,XM_030245352,XM_030245353	Ccdc138	coiled-coil domain containing 138	protein_coding	NP_001156428.1;XP_006514336.1;XP_006514338.1;XP_017169628.1;XP_017169629.1;XP_030101212.1;XP_030101213.1	1923388	.
76142	NM_133485,XM_006512489,XM_006512490,XM_030245354	Ppp1r14c	protein phosphatase 1, regulatory inhibitor subunit 14C	protein_coding	NP_597844.1;XP_006512552.1;XP_006512553.1;XP_030101214.1	1923392	.
76156	NM_001113327,NM_001286584,NM_029528,XM_006506730	Fam131b	family with sequence similarity 131, member B	protein_coding	NP_001106798.1;NP_001273513.1;NP_083804.3;XP_006506793.1	1923406	.
76157	NM_029529,XM_006512916	Slc35d3	solute carrier family 35, member D3	protein_coding	NP_083805.1;XP_006512979.1	1923407	.
76161	NM_001356548,NM_029530,XM_017319318	Lamp5	lysosomal-associated membrane protein family, member 5	protein_coding	NP_001343477.1;NP_083806.2;XP_017174807.1	1923411	.
76166	NM_001081174,XR_003955035	Cplane2	ciliogenesis and planar polarity effector 2	protein_coding	NP_001074643.1	1923416	.
76167	NM_029532,XM_006530494,XM_006530495	Snrnp35	small nuclear ribonucleoprotein 35 (U11/U12)	protein_coding	NP_083808.1;XP_006530557.1;XP_006530558.1	1923417	.
76178	NM_198006	Coa5	cytochrome C oxidase assembly factor 5	protein_coding	NP_932123.3	1923428	.
76179	NM_001033173,XM_006508296	Usp31	ubiquitin specific peptidase 31	protein_coding	NP_001028345.1;XP_006508359.1	1923429	.
76183	NM_001311092,NM_001361099,NM_175235,XM_006511540,XM_006511541,XM_006511542,XM_006511543,XM_006511544,XM_006511545,XM_011242834,XM_017313681,XM_030244695	Celf6	CUGBP, Elav-like family member 6	protein_coding	NP_001298021.1;NP_001348028.1;NP_780444.2;XP_006511603.1;XP_006511604.1;XP_006511605.1;XP_006511606.1;XP_006511607.1;XP_006511608.1;XP_011241136.1;XP_017169170.1;XP_030100555.	1923433	.
76184	NM_001166556,NM_001166557,NM_147218,XM_006534413,XM_006534414	Abca6	ATP-binding cassette, sub-family A (ABC1), member 6	protein_coding	NP_001160028.1;NP_001160029.1;NP_671751.2;XP_006534476.1;XP_006534477.1	1923434	.
76187	NM_001357376,NM_001357377,NM_001357378,NM_175236,XM_006495586,XM_030243365	Adhfe1	alcohol dehydrogenase, iron containing, 1	protein_coding	NP_001344305.1;NP_001344306.1;NP_001344307.1;NP_780445.1;XP_006495649.1;XP_030099225.1	1923437	.
76192	NM_001356549,NM_001356550,NM_024465,NR_151505,XM_006500365,XM_006500367	Abhd12	abhydrolase domain containing 12	protein_coding	NP_001343478.1;NP_001343479.1;NP_077785.2;XP_006500428.1;XP_006500430.1	1923442	.
76199	NM_001347445,NM_172424,XM_006530497,XM_011248248,XM_030254882,XM_030254883	Med13l	mediator complex subunit 13-like	protein_coding	NP_001334374.1;NP_766012.3;XP_006530560.1;XP_011246550.1;XP_030110742.1;XP_030110743.1	2670178	.
76205	NM_001347485,NM_024270,XM_030247453,XM_030247454,XM_030247455,XM_030247456	Stard3nl	STARD3 N-terminal like	protein_coding	NP_001334414.1;NP_077232.2;XP_030103313.1;XP_030103314.1;XP_030103315.1;XP_030103316.1	1923455	.
76206	NM_029536	Gpr165	G protein-coupled receptor 165	protein_coding	NP_083812.1	1923456	.
76217	NM_001163637,XM_006526337,XM_006526338,XM_006526340,XM_011247034	Jakmip2	janus kinase and microtubule interacting protein 2	protein_coding	NP_001157109.1;XP_006526400.1;XP_006526401.1;XP_006526403.1;XP_011245336.1	1923467	.
76218	NR_102348,NR_102349	6430710C18Rik	RIKEN cDNA 6430710C18 gene	lncRNA	.	1923468	.
76219	NM_029541	Arxes1	adipocyte-related X-chromosome expressed sequence 1	protein_coding	NP_083817.1	1923469	.
76220	NR_029460	6530402F18Rik	RIKEN cDNA 6530402F18 gene	lncRNA	.	1923470	.
76222	NR_131983	Magef1	melanoma antigen family F, 1	transcribed_pseudogene	.	1923472	.
76223	NM_001289656,NM_001289657,NM_001289658,NM_178630,XM_006506731,XM_006506734,XM_011241501,XM_030255658,XM_030255659,XM_030255660,XM_030255661,XR_003956265,XR_377500,XR_377501	Agbl3	ATP/GTP binding protein-like 3	protein_coding	NP_001276585.1;NP_001276586.1;NP_001276587.1;NP_848745.2;XP_006506794.1;XP_006506797.1;XP_011239803.1;XP_030111518.1;XP_030111519.1;XP_030111520.1;XP_030111521.1	1923473	.
76229	NM_001113468,NR_003555	Vmn2r29	vomeronasal 2, receptor 29	protein_coding	NP_001106939.1	1923479	.
76233	NM_133763	Dnttip1	deoxynucleotidyltransferase, terminal, interacting protein 1	protein_coding	NP_598524.1	1923483	.
76238	NM_001312880,NM_080289	Grhpr	glyoxylate reductase/hydroxypyruvate reductase	protein_coding	NP_001299809.1;NP_525028.1	1923488	.
76246	NM_030112,XM_017319319	Rtf1	RTF1, Paf1/RNA polymerase II complex component	protein_coding	NP_084388.2;XP_017174808.1	1309480	.
76251	NM_001013608,NM_001361124,NM_023507,XM_006517451,XM_017315632,XM_017315633,XM_030247457	Ercc6l2	excision repair cross-complementing rodent repair deficiency, complementation group 6 like 2	protein_coding	NP_001013626.2;NP_001348053.1;NP_075996.2;XP_006517514.1;XP_017171121.1;XP_017171122.1;XP_030103317.1	1923501	.
76252	NM_001347164,NM_133764	Atp6v0e2	ATPase, H+ transporting, lysosomal V0 subunit E2	protein_coding	NP_001334093.1;NP_598525.1	1923502	.
76257	NM_001199217,NM_001199218,NM_023805,XM_017313682,XM_030244696,XM_030244697,XR_871103	Slc38a3	solute carrier family 38, member 3	protein_coding	NP_001186146.1;NP_001186147.1;NP_076294.2;XP_017169171.1;XP_030100556.1;XP_030100557.1	1923507	.
76260	NM_001364378,NM_029553,NM_198311,XM_030246975	Ttc8	tetratricopeptide repeat domain 8	protein_coding	NP_001351307.1;NP_083829.1;NP_938053.1;XP_030102835.1	1923510	.
76261	NM_029554,XM_006504178,XM_011240792	0610040J01Rik	RIKEN cDNA 0610040J01 gene	protein_coding	NP_083830.3;XP_006504241.1;XP_011239094.1	1923511	.
76263	NM_029555	Gstk1	glutathione S-transferase kappa 1	protein_coding	NP_083831.1	1923513	.
76265	NM_029557,XM_006534415,XM_006534417,XM_006534419,XM_006534420,XM_030246410,XR_003949527,XR_879757	Tsen54	tRNA splicing endonuclease subunit 54	protein_coding	NP_083833.1;XP_006534478.1;XP_006534480.1;XP_006534482.1;XP_006534483.1;XP_030102270.1	1923515	.
76267	NM_146094,XM_006527438	Fads1	fatty acid desaturase 1	protein_coding	NP_666206.1;XP_006527501.1	1923517	.
76273	NM_001190989,NM_029561,XR_001781167	Ndfip2	Nedd4 family interacting protein 2	protein_coding	NP_001177918.1;NP_083837.2	1923523	.
76279	NM_029562,XM_006521524	Cyp2d26	cytochrome P450, family 2, subfamily d, polypeptide 26	protein_coding	NP_083838.1;XP_006521587.1	1923529	.
76281	NM_029564	Tax1bp3	Tax1 (human T cell leukemia virus type I) binding protein 3	protein_coding	NP_083840.1	1923531	.
76282	NM_182805	Gpt	glutamic pyruvic transaminase, soluble	protein_coding	NP_877957.1	95802	.
76293	NM_001347545,NM_001361359,NM_029568,XM_030246411	Mfap4	microfibrillar-associated protein 4	protein_coding	NP_001334474.1;NP_001348288.1;NP_083844.1;XP_030102271.1	1342276	.
76294	NM_029569,XM_006509519,XM_006509520,XM_017313000	Asb5	ankyrin repeat and SOCs box-containing 5	protein_coding	NP_083845.1;XP_006509582.1;XP_006509583.1;XP_017168489.1	1923544	.
76295	NM_029570,NR_151626,NR_151627,XM_006535567,XM_006535568,XM_006535573,XM_006535574,XM_030252871,XM_030252873,XM_030252874,XR_001783770,XR_001783771	Atp11b	ATPase, class VI, type 11B	protein_coding	NP_083846.2;XP_006535630.1;XP_006535631.1;XP_006535636.1;XP_006535637.1;XP_030108731.1;XP_030108733.1;XP_030108734.1	1923545	.
76299	NM_001355184,NM_029572	Erp44	endoplasmic reticulum protein 44	protein_coding	NP_001342113.1;NP_083848.1	1923549	.
76302	NM_001024622,NM_001356448,NM_001356449,NM_001356450,XM_006522697,XM_030249326	Pcnp	PEST proteolytic signal containing nuclear protein	protein_coding	NP_001019793.1;NP_001343377.1;NP_001343378.1;NP_001343379.1;XP_006522760.1;XP_030105186.1	1923552	.
76303	NM_001033174,XM_006527439	Osbp	oxysterol binding protein	protein_coding	NP_001028346.1;XP_006527502.1	97447	.
76306	NM_001358812,NM_001358813,NM_183116,XM_006512918,XM_030245355,XM_030245356,XM_030245357,XM_030245358	Slc18b1	solute carrier family 18, subfamily B, member 1	protein_coding	NP_001345741.1;NP_001345742.1;NP_898939.2;XP_006512981.1;XP_030101215.1;XP_030101216.1;XP_030101217.1;XP_030101218.1	1923556	.
76308	NM_029576	Rab1b	RAB1B, member RAS oncogene family	protein_coding	NP_083852.1	1923558	.
76311	NR_045995	1110019D14Rik	RIKEN cDNA 1110019D14 gene	lncRNA	.	1923561	.
76332	NM_029746,XR_001778485	Cog2	component of oligomeric golgi complex 2	protein_coding	NP_084022.2	1923582	.
76338	NM_172601,XM_030248092	Rab2b	RAB2B, member RAS oncogene family	protein_coding	NP_766189.1;XP_030103952.1	1923588	.
76355	NM_029578	Tgds	TDP-glucose 4,6-dehydratase	protein_coding	NP_083854.3	1923605	.
76357	NM_029580,XM_006516357,XM_006516358,XM_017315242,XM_030246976,XR_872722,XR_872723,XR_872724	Trmt5	TRM5 tRNA methyltransferase 5	protein_coding	NP_083856.1;XP_006516420.1;XP_006516421.1;XP_017170731.1;XP_030102836.1	1923607	.
76365	NM_023814	Tbx18	T-box18	protein_coding	NP_076303.3	1923615	.
76366	NM_001256100,NM_001256101,NM_001256102,NM_001359467,NM_029581	Mtif3	mitochondrial translational initiation factor 3	protein_coding	NP_001243029.1;NP_001243030.1;NP_001243031.1;NP_001346396.1;NP_083857.1	1923616	.
76367	NM_023815,XM_006500371,XM_030252188	Trp53rkb	transformation related protein 53 regulating kinase B	protein_coding	NP_076304.2;XP_006500434.1;XP_030108048.1	1914050	.
76371	XR_001780978	2810408B13Rik	RIKEN cDNA 2810408B13 gene	misc_RNA	.	1923621	.
76373	NM_029584	Zfp773	zinc finger protein 773	protein_coding	NP_083860.1	1923623	.
76375	NM_029585,XM_006541268,XM_006541269,XM_011250909,XM_017312371,XM_030243076,XR_003946576,XR_003946577,XR_391368,XR_391369,XR_391370,XR_391371	Det1	de-etiolated homolog 1 (Arabidopsis)	protein_coding	NP_083861.1;XP_006541331.1;XP_006541332.1;XP_011249211.1;XP_017167860.1;XP_030098936.1	1923625	.
76376	NM_001110240,NM_172426,XM_006538342,XM_006538343,XM_006538344,XM_006538345,XM_006538346,XM_006538347,XM_006538348,XM_006538349,XM_017320435,XM_017320436,XM_030253877	Slc24a2	solute carrier family 24 (sodium/potassium/calcium exchanger), member 2	protein_coding	NP_001103710.1;NP_766014.1;XP_006538405.1;XP_006538406.1;XP_006538407.1;XP_006538408.1;XP_006538409.1;XP_006538410.1;XP_006538411.1;XP_006538412.1;XP_017175924.1;XP_017175925.1;XP_030109737.1	1923626	.
76377	XR_389233	Gm9734	predicted gene 9734	misc_RNA	.	3804254	.
76378	NM_030744,XM_006522698	Ropn1	ropporin, rhophilin associated protein 1	protein_coding	NP_109669.1;XP_006522761.1	1923628	.
76380	NM_029586,NM_029606,NM_145688,XM_006534423,XM_006534424,XM_006534425,XM_011249300,XM_011249301,XM_017314819,XM_030246412,XM_030246413,XM_030246414	Cep112	centrosomal protein 112	protein_coding	NP_083862.1;NP_083882.3;NP_663721.2;XP_006534486.1;XP_006534487.1;XP_006534488.1;XP_011247602.1;XP_011247603.1;XP_017170308.1;XP_030102272.1;XP_030102273.1;XP_030102274.1	1923673	.
76383	NM_029588	H2al1m	H2A histone family member L1M	protein_coding	NP_083864.1	1923633	.
76389	NM_023816,XM_006514890,XM_017314820,XM_017314821,XM_017314822,XM_017314823,XM_017314824,XM_017314826,XM_017314827,XM_017314828,XM_030246415,XR_001780072	Ankrd36	ankyrin repeat domain 36	protein_coding	NP_076305.2;XP_006514953.1;XP_017170309.1;XP_017170310.1;XP_017170311.1;XP_017170312.1;XP_017170313.1;XP_017170315.1;XP_017170316.1;XP_017170317.1;XP_030102275.1	1923639	.
76392	NR_045932	Slfn5os	schlafen 5, opposite strand	lncRNA	.	1923642	.
76398	XR_880766	1700017M07Rik	RIKEN cDNA 1700017M07 gene	lncRNA	.	1923648	.
76400	NM_029595	Pbp2	phosphatidylethanolamine binding protein 2	protein_coding	NP_083871.3	1923650	.
76405	NM_001357259,NM_001357260,NM_029597,XM_006531474,XM_006531475,XM_011248541,XM_011248542,XM_011248543,XM_017313001,XM_030243835	1700018B08Rik	RIKEN cDNA 1700018B08 gene	protein_coding	NP_001344188.1;NP_001344189.1;NP_083873.1;XP_006531537.1;XP_006531538.1;XP_011246843.1;XP_011246844.1;XP_011246845.1;XP_017168490.1;XP_030099695.1	1923655	.
76406	NM_029598,XM_006508921,XM_006508922,XM_006508923,XM_006508924,XM_006508925,XM_017313002,XM_017313003,XM_017313004,XM_017313005	Tex45	testis expressed 45	protein_coding	NP_083874.1;XP_006508984.1;XP_006508985.1;XP_006508986.1;XP_006508987.1;XP_006508988.1;XP_017168491.1;XP_017168492.1;XP_017168493.1;XP_017168494.1	1923656	.
76408	NM_001363187,NM_001363189,NM_029600,XR_879762	Abcc3	ATP-binding cassette, sub-family C (CFTR/MRP), member 3	protein_coding	NP_001350116.1;NP_001350118.1;NP_083876.3	1923658	.
76411	NM_001199843,NM_029601	Ift43	intraflagellar transport 43	protein_coding	NP_001186772.1;NP_083877.1	1923661	.
76412	XR_379144,XR_379145,XR_379146,XR_870546,XR_870547	1700019J19Rik	RIKEN cDNA 1700019J19 gene, transcript variant X3	lncRNA	.	1923662	.
76415	NM_001242647,NM_175240,NR_038860	Fam187b	family with sequence similarity 187, member B	protein_coding	NP_001229576.1;NP_780449.2	1923665	.
76416	NM_001368424,NM_001368425,NM_001368426,NM_029602,XM_030250134	Znrd1as	zinc ribbon domain containing 1, antisense	protein_coding	NP_001355353.1;NP_001355354.1;NP_001355355.1;NP_083878.1;XP_030105994.1	1923666	.
76419	NR_040263,NR_040264	Smkr-ps	smal lysine rich protein 1, pseudogene	lncRNA	.	1923669	.
76421	NM_175241,NM_182745	1700028K03Rik	RIKEN cDNA 1700028K03 gene	protein_coding	NP_780450.2;NP_877422.2	1923671	.
76425	NM_001289651,NM_001289652,NM_029607,XM_030252191	Gid8	GID complex subunit 8	protein_coding	NP_001276580.1;NP_001276581.1;NP_083883.1;XP_030108051.1	1923675	.
76426	NM_029608	Fam209	family with sequence similarity 209	protein_coding	NP_083884.1	1923676	.
76429	NM_029609,XM_006508297,XM_006508298	Lhpp	phospholysine phosphohistidine inorganic pyrophosphate phosphatase	protein_coding	NP_083885.1;XP_006508360.1;XP_006508361.1	1923679	.
76432	NR_040265,NR_040266,NR_040268	2310001H17Rik	RIKEN cDNA 2310001H17 gene	lncRNA	.	1923682	.
76438	NM_181397,XM_006525081,XM_030250135	Rftn1	raftlin lipid raft linker 1	protein_coding	NP_852062.1;XP_006525144.1;XP_030105995.1	1923688	.
76441	NM_001008231,XM_006525082,XM_006525083,XM_006525084,XM_006525085,XM_006525086,XM_006525087,XM_006525089,XM_006525090,XM_006525091,XM_011246706,XM_011246707,XR_003952209,XR_385382	Daam2	dishevelled associated activator of morphogenesis 2	protein_coding	NP_001008232.2;XP_006525145.1;XP_006525146.1;XP_006525147.1;XP_006525148.1;XP_006525149.1;XP_006525150.1;XP_006525152.1;XP_006525153.1;XP_006525154.1;XP_011245008.1;XP_011245009.1	1923691	.
76443	XR_865459	2310016D23Rik	RIKEN cDNA 2310016D23 gene, transcript variant X1	lncRNA	.	1923693	.
76448	NM_001146710,NM_001146711,NM_175242,XM_006525092,XM_030250136	Ppp1r18	protein phosphatase 1, regulatory subunit 18	protein_coding	NP_001140182.1;NP_001140183.1;NP_780451.1;XP_006525155.2;XP_030105996.1	1923698	.
76453	NM_001360752,NM_001360753,NM_001360754,NM_001360755,NM_001360756,NM_029614,XM_030243077,XM_030243078	Prss23	protease, serine 23	protein_coding	NP_001347681.1;NP_001347682.1;NP_001347683.1;NP_001347684.1;NP_001347685.1;NP_083890.2;XP_030098937.1;XP_030098938.1	1923703	.
76454	NM_133765,XM_006531477,XM_006531478	Fbxo31	F-box protein 31	protein_coding	NP_598526.3;XP_006531540.1;XP_006531541.1	1354708	.
76455	XR_881379	2310067E19Rik	RIKEN cDNA 2310067E19 gene	misc_RNA	.	1923705	.
76457	NM_001326588,NM_172428,NR_137170,XM_006521526,XR_001781575	Ccdc134	coiled-coil domain containing 134	protein_coding	NP_001313517.1;NP_766016.2;XP_006521589.1	1923707	.
76459	NM_001306148,NM_178396,XM_006511548	Car12	carbonic anhydrase 12	protein_coding	NP_001293077.1;NP_848483.3;XP_006511611.1	1923709	.
76464	NM_001362732,NM_029617,XM_006500376,XM_006500377,XM_030252192	Knl1	kinetochore scaffold 1	protein_coding	NP_001349661.1;NP_083893.2;XP_006500439.1;XP_006500440.1;XP_030108052.1	1923714	.
76467	NM_029619,XM_006497563,XM_017319320	Msrb2	methionine sulfoxide reductase B2	protein_coding	NP_083895.1;XP_006497626.1;XP_017174809.1	1923717	.
76469	NM_023821	Cmya5	cardiomyopathy associated 5	protein_coding	NP_076310.2	1923719	.
76477	NM_029620	Pcolce2	procollagen C-endopeptidase enhancer 2	protein_coding	NP_083896.1	1923727	.
76478	NM_001163042,NM_001363402,NM_001363403,NM_001363404,NM_001363405,NM_001363406,NM_029621,XM_006509779,XM_017313007,XM_030243836	Haus8	4HAUS augmin-like complex, subunit 8	protein_coding	NP_001156514.1;NP_001350331.1;NP_001350332.1;NP_001350333.1;NP_001350334.1;NP_001350335.1;NP_083897.2;XP_006509842.1;XP_017168496.1;XP_030099696.1	1923728	.
76479	NM_001356986,NM_172429,XM_006527440,XM_006527442	Smndc1	survival motor neuron domain containing 1	protein_coding	NP_001343915.1;NP_766017.1;XP_006527503.1;XP_006527505.1	1923729	.
76482	NM_029623,XM_006526341,XM_006526342,XR_386019	Rmc1	regulator of MON1-CCZ1	protein_coding	NP_083899.1;XP_006526404.1;XP_006526405.1	1916528	.
76483	NM_029624,NR_045520,XM_006525095,XM_006525097,XM_030250137	Lmf1	lipase maturation factor 1	protein_coding	NP_083900.3;XP_006525158.1;XP_006525160.1;XP_030105997.1	1923733	.
76484	NM_177261	Kndc1	kinase non-catalytic C-lobe domain (KIND) containing 1	protein_coding	NP_796235.4	1923734	.
76485	NM_001165930,NM_029626,XM_006519669,XM_006519672,XM_006519673,XM_006519674,XM_030248093	Glt8d1	glycosyltransferase 8 domain containing 1	protein_coding	NP_001159402.1;NP_083902.2;XP_006519732.3;XP_006519735.1;XP_006519736.1;XP_006519737.1;XP_030103953.1	1923735	.
76486	NM_029627	Ly6k	lymphocyte antigen 6 complex, locus K	protein_coding	NP_083903.1	1923736	.
76487	NM_029628,XM_006516803,XM_006516804	Ppp1r3g	protein phosphatase 1, regulatory subunit 3G	protein_coding	NP_083904.1;XP_006516866.1;XP_006516867.1	1923737	.
76491	NM_029631,XM_006511848,XM_017313683,XM_030244698	Abhd14b	abhydrolase domain containing 14b	protein_coding	NP_083907.3;XP_006511911.1;XP_017169172.1;XP_030100558.1	1923741	.
76497	NM_029632	Ppp1r11	protein phosphatase 1, regulatory inhibitor subunit 11	protein_coding	NP_083908.1	1923747	.
76498	NM_023824,XM_006525098	Paqr4	progestin and adipoQ receptor family member IV	protein_coding	NP_076313.1;XP_006525161.1	1923748	.
76499	NM_001081960,NM_001114347,NM_001286599,NM_001286600,NM_001286601,NM_001286602,NM_001286603,NM_029633,XM_006512384,XM_006512385,XM_006512386,XM_006512387,XM_006512388,XM_006512389,XM_006512390,XM_006512391,XM_006512392,XM_006512393,XM_006512394,XM_006512395,XM_006512396,XM_006512397,XM_017313691,XM_030244699,XM_030244700,XM_030244701,XM_030244702,XM_030244703,XM_030244704,XM_030244705,XM_030244706,XM_030244707,XR_379881	Clasp2	CLIP associating protein 2	protein_coding	NP_001075429.1;NP_001107819.1;NP_001273528.1;NP_001273529.1;NP_001273530.1;NP_001273531.1;NP_001273532.1;NP_083909.2;XP_006512447.1;XP_006512448.1;XP_006512449.1;XP_006512450.1;XP_006512451.1;XP_006512452.1;XP_006512453.1;XP_006512454.1;XP_006512455.1;XP_006512456.1;XP_006512457.1;XP_006512458.1;XP_006512459.1;XP_006512460.1;XP_017169180.1;XP_030100559.1;XP_030100560.1;XP_030100561.1;XP_030100562.1;XP_030100563.1;XP_030100564.1;XP_030100565.1;XP_030100566.1;XP_	1923749	.
76500	NM_029634,XM_006511850,XM_006511851,XM_006511852,XM_006511853,XM_006511854,XM_006511855,XM_006511856,XM_030244708	Ip6k2	inositol hexaphosphate kinase 2	protein_coding	NP_083910.2;XP_006511913.1;XP_006511914.1;XP_006511915.1;XP_006511916.1;XP_006511917.1;XP_006511918.1;XP_006511919.1;XP_030100568.1	1923750	.
76501	NM_029635	Commd9	COMM domain containing 9	protein_coding	NP_083911.1	1923751	.
76505	NR_037697,NR_037698	1500009C09Rik	RIKEN cDNA 1500009C09 gene	lncRNA	.	1923755	.
76507	NM_001161621,NM_001161622,NM_029638,XM_030255662	Aoc1	amine oxidase, copper-containing 1	protein_coding	NP_001155093.1;NP_001155094.1;NP_083914.2;XP_030111522.1	1923757	.
76508	NR_015577	Ube2d-ps	ubiquitin-conjugating enzyme E2D, pseudogene	lncRNA	.	1913774	.
76509	NM_029639	Plet1	placenta expressed transcript 1	protein_coding	NP_083915.2	1923759	.
76510	NM_001164641,NM_001164643,NM_029640,NM_180662,XM_006521527,XM_017316788,XR_001781576	Trappc9	trafficking protein particle complex 9	protein_coding	NP_001158113.1;NP_001158115.1;NP_083916.1;NP_850993.2;XP_006521590.1;XP_017172277.1	1923760	.
76516	NR_131042	2010001A14Rik	RIKEN cDNA 2010001A14 gene	lncRNA	.	1923766	.
76522	NM_133939	Lsm8	LSM8 homolog, U6 small nuclear RNA associated	protein_coding	NP_598700.1	1923772	.
76524	NM_001033175	Cln6	ceroid-lipofuscinosis, neuronal 6	protein_coding	NP_001028347.1	2159324	.
76527	NM_001135100,NM_029646,XM_006531479,XM_006531480,XM_006531483,XM_006531484,XM_006531485,XM_006531486,XM_030243837,XM_030243838	Il34	interleukin 34	protein_coding	NP_001128572.1;NP_083922.1;XP_006531542.1;XP_006531543.1;XP_006531546.1;XP_006531547.1;XP_006531548.1;XP_006531549.1;XP_030099697.1;XP_030099698.1	1923777	.
76539	NM_001358271,NM_001358272,NM_001358273,NM_029648,NR_152390,NR_152391,XM_006527443,XM_006527444,XM_006527445,XM_006527447,XM_006527450,XM_017318307,XM_030251127	Fam204a	family with sequence similarity 204, member A	protein_coding	NP_001345200.1;NP_001345201.1;NP_001345202.1;NP_083924.2;XP_006527506.1;XP_006527507.1;XP_006527508.1;XP_006527510.1;XP_006527513.1;XP_017173796.1;XP_030106987.1	1289174	.
76547	NM_029649,XM_017314829	Tmem101	transmembrane protein 101	protein_coding	NP_083925.2;XP_017170318.1	1923797	.
76551	NM_001111121,XM_006514276,XM_006514277	Ccdc6	coiled-coil domain containing 6	protein_coding	NP_001104591.1;XP_006514339.1;XP_006514340.1	1923801	.
76559	NM_029654,XM_006516360,XM_006516361	Atg2b	autophagy related 2B	protein_coding	NP_083930.5;XP_006516423.1;XP_006516424.1	1923809	.
76560	NM_133351,XM_006508306	Prss8	protease, serine 8 (prostasin)	protein_coding	NP_579929.1;XP_006508369.1	1923810	.
76561	NM_001190156,NM_029655	Snx7	sorting nexin 7	protein_coding	NP_001177085.1;NP_083931.2	1923811	.
76563	NM_001081054,XR_380090	Qrsl1	glutaminyl-tRNA synthase (glutamine-hydrolyzing)-like 1	protein_coding	NP_001074523.1	1923813	.
76566	NM_029658	Rflnb	refilin B	protein_coding	NP_083934.1	1923816	.
76568	NM_023831,XM_006510671,XM_006510672,XR_001779069	Ift46	intraflagellar transport 46	protein_coding	NP_076320.2;XP_006510734.1;XP_006510735.1	1923818	.
76571	NM_001289554,NM_001289555,NM_001289556,NM_001289557,NM_029659,XM_006504511,XM_006504519,XM_017321143,XM_017321144,XM_017321145,XM_017321146,XM_017321147,XM_017321148,XM_017321149,XM_017321150,XM_017321151,XM_017321152,XM_017321153,XM_030254884,XM_030254885,XM_030254886,XM_030254887,XM_030254888	Styxl1	serine/threonine/tyrosine interacting-like 1	protein_coding	NP_001276483.1;NP_001276484.1;NP_001276485.1;NP_001276486.1;NP_083935.3;XP_006504574.1;XP_006504582.1;XP_017176632.1;XP_017176633.1;XP_017176634.1;XP_017176635.1;XP_017176636.1;XP_017176637.1;XP_017176638.1;XP_017176639.1;XP_017176640.1;XP_017176641.1;XP_017176642.1;XP_030110744.1;XP_030110745.1;XP_030110746.1;XP_030110747.1;XP_030110748.1	1923821	.
76573	NM_001359186,NM_029661,XM_006514279,XM_017314142	Spatc1l	spermatogenesis and centriole associated 1 like	protein_coding	NP_001346115.1;NP_083937.1;XP_006514342.1;XP_017169631.1	1923823	.
76574	NM_029662,XM_006503477	Mfsd2a	major facilitator superfamily domain containing 2A	protein_coding	NP_083938.2;XP_006503540.1	1923824	.
76577	NM_178397,XM_006517452,XM_011244582,XM_011244583,XM_030247458,XM_030247459,XM_030247460	Faf2	Fas associated factor family member 2	protein_coding	NP_848484.2;XP_006517515.1;XP_011242884.1;XP_011242885.1;XP_030103318.1;XP_030103319.1;XP_030103320.1	1923827	.
76580	NM_001256107,NM_001256108,NM_001369161,NM_001369162,NM_001369163,NM_001369164,NM_001369165,NM_001369166,NM_001369167,NM_001369168,NM_145124,XM_006539247,XM_006539249,XM_006539251,XM_006539253,XM_006539254,XM_006539255,XM_011250353,XM_017320437,XM_030253879,XM_030253880,XM_030253881,XM_030253882,XM_030253883	Mib2	mindbomb E3 ubiquitin protein ligase 2	protein_coding	NP_001243036.1;NP_001243037.2;NP_001356090.1;NP_001356091.1;NP_001356092.1;NP_001356093.1;NP_001356094.1;NP_001356095.1;NP_001356096.1;NP_001356097.1;NP_660106.2;XP_006539310.1;XP_006539312.1;XP_006539314.1;XP_006539316.1;XP_006539317.1;XP_006539318.1;XP_011248655.1;XP_017175926.1;XP_030109739.1;XP_030109740.1;XP_030109741.1;XP_030109742.1;XP_0301	2679684	.
76582	NM_001360897,NM_029665,XM_006517787,XM_006517788,XM_011244707,XM_011244708,XM_017315635,XR_003950491,XR_003950492,XR_382746	Ipo11	importin 11	protein_coding	NP_001347826.1;NP_083941.2;XP_006517850.1;XP_006517851.1;XP_011243009.1;XP_011243010.1;XP_017171124.1	2442377	.
76589	NM_152823,XM_006525099,XM_006525101,XM_006525102,XM_006525104,XM_017317717,XR_003952210,XR_385383,XR_876580,XR_876582,XR_876583,XR_876584	Unc5cl	unc-5 family C-terminal like	protein_coding	NP_690036.4;XP_006525162.1;XP_006525164.1;XP_006525165.1;XP_006525167.1;XP_017173206.1	1923839	.
76594	NM_029669,XM_006526343,XM_006526344,XM_006526345,XM_006526346	Dnajc18	DnaJ heat shock protein family (Hsp40) member C18	protein_coding	NP_083945.1;XP_006526406.1;XP_006526407.1;XP_006526408.1;XP_006526409.1	1923844	.
76602	NR_152121	1700040D17Rik	RIKEN cDNA 1700040D17 gene	lncRNA	.	1923852	.
76608	NM_175244,XR_001784215	Hectd3	HECT domain E3 ubiquitin protein ligase 3	protein_coding	NP_780453.1	1923858	.
76612	NM_001143755,NM_027164,XM_006536262,XM_006536264,XM_006536265,XM_006536266,XM_006536267,XM_006536268,XM_006536269,XM_011249862,XM_011249863,XM_011249864,XM_011249865,XM_030243079	Lrrc27	leucine rich repeat containing 27	protein_coding	NP_001137227.1;NP_081440.1;XP_006536325.1;XP_006536327.1;XP_006536328.1;XP_006536329.1;XP_006536330.1;XP_006536331.1;XP_006536332.1;XP_011248164.1;XP_011248165.1;XP_011248166.1;XP_011248167.1;XP_030098939.1	1923862	.
76614	NM_001253681,NM_001253686,NM_001253687,NM_001253688,NM_001253689,NM_001362134,NM_001362136,NM_001362137,NM_001362138,NM_029673,XM_017321823,XM_017321828,XM_030255663,XM_030255664,XM_030255665	Immt	inner membrane protein, mitochondrial	protein_coding	NP_001240610.1;NP_001240615.1;NP_001240616.1;NP_001240617.1;NP_001240618.1;NP_001349063.1;NP_001349065.1;NP_001349066.1;NP_001349067.1;NP_083949.2;XP_017177312.1;XP_017177317.1;XP_030111523.1;XP_030111524.1;XP_030111525.1	1923864	.
76615	NM_029674,XM_006509211,XM_006509213,XM_011242167	Got1l1	glutamic-oxaloacetic transaminase 1-like 1	protein_coding	NP_083950.1;XP_006509274.1;XP_006509276.1;XP_011240469.1	1923865	.
76624	NR_130171,NR_130318	1700099I09Rik	RIKEN cDNA 1700099I09 gene	lncRNA	.	1923874	.

76626	NM_001201341,NM_001363194,NM_001363195,NM_054043,XM_006534431,XM_006534432,XM_006534434,XM_006534435,XM_006534438,XM_006534439,XM_006534440,XM_006534441,XM_011249302,XM_011249304,XM_011249305,XM_030246416,XM_030246417,XM_030246418,XR_001780073,XR_003949528,XR_388550,XR_879764	Msi2	musashi RNA-binding protein 2	protein_coding	NP_001188270.1;NP_001350123.1;NP_001350124.1;NP_473384.1;XP_006534494.1;XP_006534495.1;XP_006534497.1;XP_006534498.1;XP_006534501.1;XP_006534502.1;XP_006534503.1;XP_006534504.1;XP_011247604.1;XP_011247606.1;XP_011247607.1;XP_030102276.1;XP_030102277.1;XP_030102278.1	1923876	.	18.385741	[illegible]
76628	NR_131043,NR_131044	1700112J16Rik	RIKEN cDNA 1700112J16 gene	lncRNA	.	1923878	.	0.129819	[illegible]
76630	NM_001360716,NM_001360717,NM_001360718,NM_001360719,NM_001360720,NM_029682,XM_006527451,XM_006527452	Stambpl1	STAM binding protein like 1	protein_coding	NP_001347645.1;NP_001347646.1;NP_001347647.1;NP_001347648.1;NP_001347649.1;NP_083958.3;XP_006527514.1;XP_006527515.1	1923880	.	3.261455	[illegible]
76633	NM_028275,XM_006519676,XM_006519678,XM_006519679,XM_006519680	Lrmda	leucine rich melanocyte differentiation associated	protein_coding	NP_082551.1;XP_006519739.1;XP_006519741.1;XP_006519742.1;XP_006519743.1	1923883	.	0.076433	[illegible]
76634	NR_045461	1700110C19Rik	RIKEN cDNA 1700110C19 gene	lncRNA	.	1923884	.	0.171522	[illegible]
76642	NR_045997	1700113A16Rik	RIKEN cDNA 1700113A16 gene	lncRNA	.	1923892	.	0.759354	[illegible]
76645	NM_029686	Pkd1l2	polycystic kidney disease 1 like 2	protein_coding	NP_083962.4	2664668	.	0.027722	[illegible]
76649	NR_040536	1700119H24Rik	RIKEN cDNA 1700119H24 gene	lncRNA	.	1923899	.	0.121067	[illegible]
76650	NM_029688	Srxn1	sulfiredoxin 1 homolog (S. cerevisiae)	protein_coding	NP_083964.2	104971	.	11.424217	[illegible]
76652	NM_029690	Actrt3	actin related protein T3	protein_coding	NP_083966.1	1923902	.	0.000000	[illegible]
76654	NM_001289659,NM_001289660,NM_029692,XM_006498406,XM_006498407,XM_006498409,XM_006498410,XM_011239205,XM_011239206,XM_030253739,XR_003953740,XR_003953741,XR_003953742,XR_866022	Upp2	uridine phosphorylase 2	protein_coding	NP_001276588.1;NP_001276589.1;NP_083968.1;XP_006498469.1;XP_006498470.1;XP_006498472.1;XP_006498473.1;XP_011237507.1;XP_011237508.1;XP_011237509.1;XP_017174811.1;XP_030108053.1	1923904	.	0.846664	[illegible]
76670	NM_001163638,NM_001163639,NM_029698,XM_006519684	Cfap70	cilia and flagella associated protein 70	protein_coding	NP_001157110.1;NP_001157111.1;NP_083974.1;XP_006519747.1	1923920	.	0.550665	[illegible]
76681	NM_001368753,NM_001368754,NM_023835,XM_017312372,XM_017312373,XM_017312375,XM_030243080,XM_030243081,XM_030243082,XM_030243083,XR_003946578	Trim12a	tripartite motif-containing 12A	protein_coding	NP_001355682.1;NP_001355683.1;NP_076324.2;XP_017167861.1;XP_017167862.1;XP_017167864.1;XP_030098940.1;XP_030098941.1;XP_030098942.1;XP_030098943.1	1923931	.	6.070172	[illegible]
76683	XR_003946678,XR_003946679,XR_003946680,XR_003946681,XR_378553,XR_870008	1500002F19Rik	RIKEN cDNA 1500002F19 gene, transcript variant X6	lncRNA	.	1923933	.	0.341387	[illegible]
76686	NM_001081114,NM_001360763,NM_001360764	Clip3	CAP-GLY domain containing linker protein 3	protein_coding	NP_001074583.1;NP_001347692.1;NP_001347693.1	1923936	.	13.954850	[illegible]
76687	NM_029701	Spcs3	signal peptidase complex subunit 3 homolog (S. cerevisiae)	protein_coding	NP_083977.1	1923937	.	21.171473	[illegible]
76688	NM_001165991,NM_001165992,NM_001165995,NM_029702,NR_028585,XR_003953743,XR_867137	Arfrp1	ADP-ribosylation factor related protein 1	protein_coding	NP_001159463.1;NP_001159464.1;NP_001159467.1;NP_083978.3	1923938	.	10.634587	[illegible]
76703	NM_029706	Cpb1	carboxypeptidase B1 (tissue)	protein_coding	NP_083982.1	1923953	.	0.049624	[illegible]
76707	NM_001293300,NM_001293301,NM_001359328,NM_001359329,NM_001359330,NM_001359331,NM_001359334,NM_001359335,NM_001359336,NM_001359337,NM_001359338,NM_001359339,NM_001359340,NM_001359341,NM_001359342,NM_001359343,NM_001359345,NM_001359346,NM_001359347,NM_029709,XM_006529920,XM_006529922,XM_006529924,XM_006529929,XM_006529937,XM_006529938,XM_006529939,XM_006529948,XM_011248107,XM_011248108,XM_030243412,XM_030243414,XM_030243415,XM_030243419,XM_030243421,XM_030243423,XM_030243424,XM_030243427,XM_030243428,XM_030243432,XM_030243436,XM_030243442,XM_030243445,XM_030243449,XM_030243458,XM_030243461,XM_030243468,XM_030243479,XM_030243482,XM_030243485	Clasp1	CLIP associating protein 1	protein_coding	NP_001280229.1;NP_001280230.1;NP_001346257.1;NP_001346258.1;NP_001346259.1;NP_001346260.1;NP_001346263.1;NP_001346264.1;NP_001346265.1;NP_001346266.1;NP_001346267.1;NP_001346268.1;NP_001346269.1;NP_001346270.1;NP_001346271.1;NP_001346272.1;NP_001346274.1;NP_001346275.1;NP_001346276.1;NP_083985.2;XP_006529983.1;XP_006529985.1;XP_006529987.1;XP_006529992.1;XP_006530000.1;XP_006530001.1;XP_006530002.1;XP_006530011.1;XP_011246409.1;XP_011246410.1;XP_030099272.1;XP_030099274.1;XP_030099275.1;XP_030099279.1;XP_030099281.1;XP_030099283.1;XP_030099284.1;XP_030099287.1;XP_030099288.1;XP_030099292.1;XP_030099296.1;XP_030099302.1;XP_030099305.1;XP_030099309.1;XP_030099318.1;XP_030099321.1;XP_030099328.1;XP_030099339.1;XP_030099342.1;XP_030099345.1	1923957	.	8.262098	[illegible]
76709	NM_001357387,NM_029711,XM_030243491	Arpc2	actin related protein 2/3 complex, subunit 2	protein_coding	NP_001344316.1;NP_083987.1;XP_030099351.1	1923959	.	116.382364	[illegible]
76710	NR_126488,NR_126489,NR_126490	2210406O10Rik	RIKEN cDNA 2210406O10 gene	lncRNA	.	1923960	.	0.079103	[illegible]
76718	NM_029714,XM_006540412,XM_006540413,XM_006540414,XM_006540416,XM_006540417,XM_011250736,XM_011250737,XM_011250738,XM_011250739,XM_017312381	Catsperg2	cation channel sperm associated auxiliary subunit gamma 2	protein_coding	NP_083990.2;XP_006540475.1;XP_006540476.1;XP_006540477.1;XP_006540479.1;XP_006540480.1;XP_011249038.1;XP_011249039.1;XP_011249040.1;XP_011249041.1;XP_017167870.1	1923968	.	0.050869	[illegible]
76719	NM_001081045,NM_001359634,XM_006534445,XM_006534446,XM_006534447,XM_006534448,XM_006534449,XM_006534450,XM_006534451,XM_006534454,XM_011249306,XM_011249307,XM_030246419,XM_030246420,XM_030246421,XM_030246422	Kansl1	KAT8 regulatory NSL complex subunit 1	protein_coding	NP_001074514.1;NP_001346563.1;XP_006534508.1;XP_006534509.1;XP_006534510.1;XP_006534511.1;XP_006534512.1;XP_006534513.1;XP_006534514.1;XP_006534517.1;XP_011247608.1;XP_011247609.1;XP_030102279.1;XP_030102280.1;XP_030102281.1;XP_0301	1923969	.	13.321662	[illegible]
76722	NM_198415,XR_003950493	Ckmt2	creatine kinase, mitochondrial 2	protein_coding	NP_940807.1	1923972	.	3.877710	[illegible]
76737	NM_029720	Creld2	cysteine-rich with EGF-like domains 2	protein_coding	NP_083996.1	1923987	.	20.720524	[illegible]
76740	NM_001356582,NM_133766,XM_006521530	Efr3a	EFR3 homolog A	protein_coding	NP_001343511.1;NP_598527.2;XP_006521593.1	1923990	.	15.132339	[illegible]
76742	NM_001082484,NM_029721,XM_006502258,XM_030252876,XM_030252877	Snx27	sorting nexin family member 27	protein_coding	NP_001075953.1;NP_083997.1;XP_006502321.1;XP_030108736.1;XP_030108737.1	1923992	.	8.472566	[illegible]
76747	NM_029723	Dapl1	death associated protein-like 1	protein_coding	NP_083999.1	1923997	.	0.000000	[illegible]
76757	NM_001364696,NM_001364697,NM_029726	Trdn	triadin	protein_coding	NP_001351625.1;NP_001351626.1;NP_084002.2	1924007	.	3.819712	[illegible]
76763	NM_001290523,NM_001290524,NM_029730,XM_006528985,XM_011247873,XM_011247874,XM_011247875,XM_011247876,XM_030251516,XM_030251517	Mospd2	motile sperm domain containing 2	protein_coding	NP_001277452.1;NP_001277453.1;NP_084006.2;XP_006529048.1;XP_011246175.1;XP_011246176.1;XP_011246177.1;XP_011246178.1;XP_030107376.1;XP_030107377.1	1924013	.	6.830256	[illegible]
76770	NM_029733,XM_006522699	Csta2	cystatin A family member 2	protein_coding	NP_084009.1;XP_006522762.1	1924020	.	0.357667	[illegible]
76773	NM_001326599,NM_001326602,NM_029734,XR_001781577,XR_001781578,XR_003951445	Wdyhv1	WDYHV motif containing 1	protein_coding	NP_001313528.1;NP_001313531.1;NP_084010.1	1924023	.	5.438577	[illegible]
76775	NM_001009981,NM_001282108,NM_001282109,NM_029736,NR_104094,XM_006531488,XM_006531489,XM_006531491,XM_011248545,XM_017313009,XM_017313010,XM_030243839,XR_003947353,XR_387783,XR_387784,XR_878921	Slc10a7	solute carrier family 10 (sodium/bile acid cotransporter family), member 7	protein_coding	NP_001009981.1;NP_001269037.1;NP_001269038.1;NP_084012.1;XP_006531551.1;XP_006531552.1;XP_006531554.1;XP_011246847.1;XP_017168498.1;XP_017168499.1;XP_030099699.1	1924025	.	9.138067	[illegible]
76779	NM_029738,XM_006522701,XM_006522702,XM_006522703,XM_006522704,XM_006522705,XM_006522706,XM_017317157,XM_030249327,XM_030249328	Cluap1	clusterin associated protein 1	protein_coding	NP_084014.1;XP_006522764.4;XP_006522765.1;XP_006522766.1;XP_006522767.1;XP_006522768.1;XP_006522769.1;XP_017172646.2;XP_030105187.1;XP_030105188.1	1924029	.	9.607249	[illegible]
76781	NM_001357135,NM_001357136,NM_001357137,NM_001357138,NM_176917,XM_011246710,XM_030250139,XR_001782127	Mettl4	methyltransferase like 4	protein_coding	NP_001344064.1;NP_001344065.1;NP_001344066.1;NP_001344067.1;NP_795891.2;XP_011245012.1;XP_030105999.1	1924031	.	3.419089	[illegible]
76784	NM_001282118,NM_001282119,NM_001282120,NM_133767,XM_006514891,XM_006514892,XM_030246423,XM_030246424,XR_003949529,XR_380987	Mtif2	mitochondrial translational initiation factor 2	protein_coding	NP_001269047.1;NP_001269048.1;NP_001269049.1;NP_598528.2;XP_006514954.1;XP_006514955.1;XP_030102283.1;XP_030102284.1	1924034	.	10.756367	[illegible]
76786	NR_131048	2510003B16Rik	RIKEN cDNA 2510003B16 gene	lncRNA	.	1924036	.	0.207477	[illegible]
76787	NM_029741,XM_006541273,XM_011250912,XM_011250914,XM_011250915	Ppfia3	protein tyrosine phosphatase, receptor type, f polypeptide (PTPRF), interacting protein (liprin), alpha 3	protein_coding	NP_084017.2;XP_006541336.1;XP_011249214.1;XP_011249216.1;XP_011249217.1	1924037	.	0.593383	[illegible]
76788	NM_001311087,NM_029742,XM_017321829,XM_030255666	Klhdc10	kelch domain containing 10	protein_coding	NP_001298016.1;NP_084018.1;XP_017177318.1;XP_030111526.1	1924038	.	19.241071	[illegible]
76789	NM_175245,XM_030248094	Mzt1	mitotic spindle organizing protein 1	protein_coding	NP_780454.1;XP_030103954.1	1924039	.	17.096785	[illegible]
76792	NM_001081236	2410131K14Rik	RIKEN cDNA 2410131K14 gene	protein_coding	NP_001074705.1	1924042	.	3.350045	[illegible]
76793	NM_001356560,NM_175246	Snip1	Smad nuclear interacting protein 1	protein_coding	NP_001343489.1;NP_780455.2	2156003	.	8.281038	[illegible]
76794	NR_102289	Man2c1os	mannosidase, alpha, class 2C, member 1, opposite strand	lncRNA	.	1924044	.	0.550765	[illegible]
76795	NM_001290759,NM_001290760,NM_029745,XM_006534458,XR_001780074,XR_001780075,XR_388552,XR_388553	Tbc1d9b	TBC1 domain family, member 9B	protein_coding	NP_001277688.1;NP_001277689.1;NP_084021.2;XP_006534521.1	1924045	.	16.402202	[illegible]
76797	NM_029747	2410137M14Rik	RIKEN cDNA 2410137M14 gene	protein_coding	NP_084023.1	1924047	.	0.229391	[illegible]
76798	NR_015595	Srrm4os	serine/arginine repetitive matrix 4, opposite strand	lncRNA	.	1924048	.	0.000000	[illegible]
76799	NM_001368255,NM_001368256,NM_001368257,NM_001368258,NM_001368259,NM_001368260,NM_029748,XM_030253884,XM_030253885,XM_030253886,XM_030253887,XM_030253888,XM_030253889,XM_030253890,XM_030253891	Tmem234	transmembrane protein 234	protein_coding	NP_001355184.1;NP_001355185.1;NP_001355186.1;NP_001355187.1;NP_001355188.1;NP_001355189.1;NP_084024.1;XP_030109744.1;XP_030109745.1;XP_030109746.1;XP_030109747.1;XP_030109748.1;XP_030109749.1;XP_030109750.1;XP_030109751.1	1924049	.	49.168558	[illegible]
76800	NM_029749,XM_006504754	Usp42	ubiquitin specific peptidase 42	protein_coding	NP_084025.2;XP_006504817.1	1924050	.	6.013155	[illegible]
76804	NM_001172095,NM_001356561,NM_144787,XM_006538351,XM_006538352,XM_030253892,XM_030253893	Kdm4c	lysine (K)-specific demethylase 4C	protein_coding	NP_001165566.1;NP_001343490.1;NP_659036.1;XP_006538414.1;XP_006538415.1;XP_030109752.1;XP_030109753.1	1924054	.	7.220874	[illegible]
76808	NM_029751	Rpl18a	ribosomal protein L18A	protein_coding	NP_084027.1	1924058	.	528.487488	[illegible]
76809	NM_001359486,NM_029752	Bri3bp	Bri3 binding protein	protein_coding	NP_001346415.1;NP_084028.1	1924059	.	3.325829	[illegible]
76813	NM_133972,XM_011242332,XM_011242333,XM_011242334,XM_030243841,XM_030243842,XM_030243844	Armc6	armadillo repeat containing 6	protein_coding	NP_598733.2;XP_011240634.1;XP_011240635.1;XP_011240636.1;XP_030099701.1;XP_030099702.1;XP_030099704.1	1924063	.	4.841834	[illegible]
76814	NR_040743	Scarletltr	Scarletltr, erythroid developmental long intergenic non-protein coding transcript	lncRNA	.	1924064	.	0.000000	[illegible]
76816	NM_001357390,NM_029756,NR_151682,XM_006497025,XM_030243501,XM_030243505,XM_030243510,XM_030243513,XM_030243514,XR_003947261,XR_003947262,XR_003947264,XR_003947265,XR_003947266,XR_003947267,XR_003947270,XR_003947271	Sdccag8	serologically defined colon cancer antigen 8	protein_coding	NP_001344319.1;NP_084032.1;XP_006497088.1;XP_030099361.1;XP_030099365.1;XP_030099370.1;XP_030099373.1;XP_030099374.1	1924066	.	3.237917	[illegible]
76820	NM_001146119,NM_001364432,NM_029758,XM_006515246,XM_006515247,XM_030246977	Fam49a	family with sequence similarity 49, member A	protein_coding	NP_001139591.1;NP_001351361.1;NP_084034.2;XP_006515309.1;XP_006515310.1;XP_030102837.1	1261783	.	2.045824	[illegible]
76824	NM_001256112,NM_001355187,NM_001355188,NM_029759,XM_006539256,XM_006539258	Mtfr1l	mitochondrial fission regulator 1-like	protein_coding	NP_001243041.1;NP_001342116.1;NP_001342117.1;NP_084035.1;XP_006539319.1;XP_006539321.1	1924074	.	28.144602	[illegible]
76826	NM_029760,XM_006516362,XM_006516363	Nubpl	nucleotide binding protein-like	protein_coding	NP_084036.2;XP_006516425.1;XP_006516426.1	1924076	.	3.242514	[illegible]
76829	NM_001163686,NM_029761	Dok5	docking protein 5	protein_coding	NP_001157158.1;NP_084037.3	1924079	.	5.253818	[illegible]
76832	NM_029762	Hyls1	HYLS1, centriolar and ciliogenesis associated	protein_coding	NP_084038.1	1924082	.	3.231641	[illegible]
76843	NM_001305233,NM_029766,XM_011238925	Dtl	denticleless E3 ubiquitin protein ligase	protein_coding	NP_001292162.1;NP_084042.1;XP_011237227.1	1924093	.	6.062815	[illegible]
76846	NM_029767,XR_391108	Rps9	ribosomal protein S9	protein_coding	NP_084043.1	1924096	.	1065.493290	[illegible]
76850	NM_153177,XM_006503481,XM_006503482,XM_006503483,XM_011240651,XR_003955036	Ago4	argonaute RISC catalytic subunit 4	protein_coding	NP_694817.2;XP_006503544.1;XP_006503545.1;XP_006503546.1;XP_011238953.1	1924100	.	2.549909	[illegible]
76854	NM_029771,XM_006504757	Gper1	G protein-coupled estrogen receptor 1	protein_coding	NP_084047.2;XP_006504820.1	1924104	.	2.431248	[illegible]
76856	NM_001252487,NM_001252488,NM_029772,XM_006517455	Catsper3	cation channel, sperm associated 3	protein_coding	NP_001239416.1;NP_001239417.1;NP_084048.1;XP_006517518.1	1924106	.	0.117357	[illegible]
76857	NM_001165997,NM_001165998,NM_029773,XM_006498412,XM_006498414,XM_006498417,XM_017319323,XM_017319324,XM_030252194,XM_030252195,XM_030252196,XR_003953744,XR_866023,XR_866024	Spopl	speckle-type BTB/POZ protein-like	protein_coding	NP_001159469.1;NP_001159470.1;NP_084049.2;XP_006498475.1;XP_006498477.1;XP_006498480.1;XP_017174812.1;XP_017174813.1;XP_030108054.1;XP_030108055.1;XP_030108056.1	1924107	.	5.611505	[illegible]
76863	NM_001357483,NM_001357484,NM_029775,XM_006509889,XM_030244709,XM_030244710,XM_030244711	Dcun1d5	DCN1, defective in cullin neddylation 1, domain containing 5 (S. cerevisiae)	protein_coding	NP_001344412.1;NP_001344413.1;NP_084051.1;XP_006509952.1;XP_030100569.1;XP_030100570.1;XP_030100571.1	1924113	.	10.761246	[illegible]
76866	NM_001081100,NM_001356327,XM_006539261,XM_006539262,XM_006539263,XM_006539267,XM_006539268,XM_006539269,XM_006539270,XM_006539271,XM_006539273,XM_006539275,XM_017320441,XM_017320443,XM_017320444,XM_017320446,XM_017320447,XM_030253894,XM_030253895,XM_030253896,XM_030253897,XM_030253898,XM_030253899,XM_030253900,XM_030253901,XM_030253902,XR_001784216,XR_003955037,XR_003955038,XR_003955039,XR_003955040,XR_003955041,XR_003955042,XR_003955043,XR_003955044,XR_003955045,XR_003955046,XR_003955047,XR_003955048,XR_003955049,XR_003955050,XR_003955051,XR_003955052	Morn1	MORN repeat containing 1	protein_coding	NP_001074569.1;NP_001343256.1;XP_006539324.1;XP_006539325.1;XP_006539326.1;XP_006539330.1;XP_006539331.1;XP_006539332.1;XP_006539333.1;XP_006539334.1;XP_006539336.1;XP_006539338.1;XP_017175930.1;XP_017175932.1;XP_017175933.1;XP_017175935.1;XP_017175936.1;XP_030109754.1;XP_030109755.1;XP_030109756.1;XP_030109757.1;XP_030109758.1;XP_030109759.1;XP_030109760.1;XP_030109761.1;XP_030109762.1	1924116	.	1.371743	[illegible]
76867	NM_001122685,NM_029777,XM_006496574,XM_011238714,XM_030243522	Rhbdd1	rhomboid domain containing 1	protein_coding	NP_001116157.1;NP_084053.3;XP_006496637.1;XP_011237016.1;XP_030099382.1	1924117	.	3.683081	[illegible]
76872	NM_001306169,NM_029779,XM_006522707,XM_006522708,XM_006522709,XM_006522710,XM_006522712,XM_017317158	Ccdc116	coiled-coil domain containing 116	protein_coding	NP_001293098.1;NP_084055.2;XP_006522770.1;XP_006522771.1;XP_006522772.1;XP_006522773.1;XP_006522775.1;XP_017172647.1	1924122	.	1.585891	[illegible]
76873	NR_126077,NR_126078	4930447F24Rik	RIKEN cDNA 4930447F24 gene	lncRNA	.	1924123	.	0.430783	[illegible]
76874	NR_155520	4930469K13Rik	RIKEN cDNA 4930469K13 gene	lncRNA	.	1924124	.	0.000000	[illegible]
76877	NM_001359270,NM_001359271,NM_001359272,XM_006514286,XM_006514287,XM_006514289,XM_006514290,XM_006514292,XM_006514293,XM_006514294,XR_380455	Rab36	RAB36, member RAS oncogene family	protein_coding	NP_001346199.1;NP_001346200.1;NP_001346201.1;XP_006514349.1;XP_006514350.1;XP_006514352.1;XP_006514353.1;XP_006514355.1;XP_006514356.1;XP_006514357.1	1924127	.	3.114640	[illegible]
76880	NR_002848	6430411K18Rik	RIKEN cDNA 6430411K18 gen	lncRNA	.	1924130	.	1.194370	[illegible]
76884	NM_001252459,NM_001252460,NM_133769,XM_006534461,XM_006534462,XM_006534463	Cyfip2	cytoplasmic FMR1 interacting protein 2	protein_coding	NP_001239388.1;NP_001239389.1;NP_598530.2;XP_006534524.1;XP_006534525.1;XP_006534526.1	1924134	.	5.646544	[illegible]
76886	NM_029784,XM_006511550,XM_006511551,XM_030244712	Fam81a	family with sequence similarity 81, member A	protein_coding	NP_084060.1;XP_006511613.1;XP_006511614.1;XP_030100572.1	1924136	.	2.028063	[illegible]
76889	NM_133770,XM_006540421,XM_006540422,XM_011250740,XM_011250741,XM_017312382,XM_017312383,XR_391109	Coq8b	coenzyme Q8B	protein_coding	NP_598531.2;XP_006540484.1;XP_006540485.1;XP_011249042.1;XP_011249043.1;XP_017167871.1;XP_017167872.1	1924139	.	8.494776	[illegible]
76890	NM_133771,XM_006525109,XM_011246713,XM_030250140,XR_003952211,XR_385387	Memo1	mediator of cell motility 1	protein_coding	NP_598532.1;XP_006525172.1;XP_011245015.1;XP_030106000.1	1924140	.	11.698247	[illegible]
76892	NM_029788	Rnft1	ring finger protein, transmembrane 1	protein_coding	NP_084064.1	1924142	.	13.002171	[illegible]
76893	NM_001320492,NM_029789,XM_006502260,XM_030252878	Cers2	ceramide synthase 2	protein_coding	NP_001307421.1;NP_084065.1;XP_006502323.1;XP_030108738.1	1924143	.	56.561441	[illegible]
76894	NM_029790,XM_006500378,XM_006500379,XM_011239840	Mettl15	methyltransferase like 15	protein_coding	NP_084066.2;XP_006500441.1;XP_006500442.1;XP_011238142.1	1924144	.	1.880779	[illegible]
76895	NM_001039179,NM_001039180,NM_029791,XM_011244429	Bicd2	BICD cargo adaptor 2	protein_coding	NP_001034268.1;NP_001034269.1;NP_084067.1;XP_011242731.1	1924145	.	12.295928	[illegible]
76897	NM_001163328,NM_001163329,NM_001163330,NM_001357033,NM_001357034,NM_001357035,NM_001357036,NM_178631,XM_006530101,XM_006530102,XM_011248150,XM_030252879,XM_030252880,XM_030252881	Ralyl	RALY RNA binding protein-like	protein_coding	NP_001156800.1;NP_001156801.1;NP_001156802.1;NP_001343962.1;NP_001343963.1;NP_001343964.1;NP_001343965.1;NP_848746.1;XP_006530164.1;XP_006530165.1;XP_011246452.1;XP_030108739.1;XP_030108740.1;XP_030108741.1	1924147	.	0.068339	[illegible]
76898	NM_001310766,NM_029792,XM_006510674,XM_030244713	B3gat1	beta-1,3-glucuronyltransferase 1 (glucuronosyltransferase P)	protein_coding	NP_001297695.1;NP_084068.1;XP_006510737.1;XP_030100573.1	1924148	.	0.053847	[illegible]
76899	NM_001290649,NM_029793,NR_110968,XM_030252197,XR_001783203,XR_866025,XR_866026	Golga1	golgi autoantigen, golgin subfamily a, 1	protein_coding	NP_001277578.1;NP_084069.1;XP_030108057.1	1924149	.	8.754029	[illegible]
76900	NM_133772,XM_006509784,XM_006509790,XM_006509792,XM_006509794,XM_006509795,XM_011242336,XM_011242337,XM_011242338,XM_030243845,XM_030243846,XM_030243848,XM_030243849	Ssbp4	single stranded DNA binding protein 4	protein_coding	NP_598533.2;XP_006509847.1;XP_006509853.1;XP_006509855.1;XP_006509857.1;XP_006509858.1;XP_011240638.1;XP_011240639.1;XP_011240640.1;XP_030099705.1;XP_030099706.1;XP_030099708.1;XP_030099709.1	1924150	.	32.283250	[illegible]
76901	NM_001357588,NM_199299,XM_006534468,XM_006534469,XM_006534470,XM_011249309,XM_017314832	Jade2	jade family PHD finger 2	protein_coding	NP_001344517.1;NP_955003.2;XP_006534531.1;XP_006534532.1;XP_006534533.1;XP_011247611.1;XP_017170321.1	1924151	.	3.803702	[illegible]
76905	NM_029796	Lrg1	leucine-rich alpha-2-glycoprotein 1	protein_coding	NP_084072.1	1924155	.	3.528027	[illegible]
76915	NM_029797,XM_006502262	Mnd1	meiotic nuclear divisions 1	protein_coding	NP_084073.2;XP_006502325.1	1924165	.	3.388498	[illegible]
76916	NM_024273,XM_006522713,XM_030249329	Timmdc1	translocase of inner mitochondrial membrane domain containing 1	protein_coding	NP_077235.2;XP_006522776.1;XP_030105189.1	1922139	.	6.507458	[illegible]
76917	NM_001357140,NM_029798,XR_001782129	Flywch2	FLYWCH family member 2	protein_coding	NP_001344069.1;NP_084074.1	1924167	.	3.158725	[illegible]
76927	NM_001357024,NM_029801,XM_030252882,XM_030252883	Tsacc	TSSK6 activating co-chaperone	protein_coding	NP_001343953.1;NP_084077.1;XP_030108742.1;XP_030108743.1	1924177	.	2.031830	[illegible]
76932	NM_029802,XM_006508317,XM_006508319,XM_011241939,XR_003946580,XR_003946581	Arfip2	ADP-ribosylation factor interacting protein 2	protein_coding	NP_084078.3;XP_006508380.1;XP_006508382.1;XP_011240241.1	1924182	.	27.779782	[illegible]
76933	NM_001281830,NM_029803	Ifi27l2a	interferon, alpha-inducible protein 27 like 2A	protein_coding	NP_001268759.1;NP_084079.1	1924183	.	4.992634	[illegible]
76936	NM_001109913,NM_029804	Hnrnpm	heterogeneous nuclear ribonucleoprotein M	protein_coding	NP_001103383.1;NP_084080.1	1926465	.	71.348343	[illegible]
76937	NR_015522	2810429I04Rik	RIKEN cDNA 2810429I04 gene	lncRNA	.	1924187	.	0.070563	[illegible]
76938	NM_152824	Rbm17	RNA binding motif protein 17	protein_coding	NP_690037.1	1924188	.	38.500740	[illegible]
76947	NM_001085493,XM_006538355,XM_006538356,XM_006538357,XM_006538358,XM_006538359,XM_006538360,XM_011250130,XM_011250131,XM_030253903,XM_030253904,XM_030253905,XM_030253906,XM_030253907,XR_001784217	Ndufaf6	NADH:ubiquinone oxidoreductase complex assembly factor 6	protein_coding	NP_001078962.1;XP_006538418.1;XP_006538419.1;XP_006538420.1;XP_006538421.1;XP_006538422.1;XP_006538423.1;XP_011248432.1;XP_011248433.1;XP_030109763.1;XP_030109764.1;XP_030109765.1;XP_030109766.1;XP_030109767.1	1924197	.	2.765493	[illegible]
76952	NM_001164363,NM_001164365,NM_029810,NR_028353,XM_006527458,XM_006527459,XM_011247398,XM_017318310,XM_017318311,XM_017318312,XM_030251128,XM_030251129,XM_030251130,XM_030251131,XM_030251132,XM_030251133,XM_030251134,XM_030251135,XM_030251136,XM_030251137,XM_030251138,XM_030251139,XM_030251140,XM_030251141,XM_030251142,XM_030251143	Nt5c2	5'-nucleotidase, cytosolic II	protein_coding	NP_001157835.1;NP_001157837.1;NP_084086.3;XP_006527521.1;XP_006527522.1;XP_011245700.1;XP_017173799.1;XP_017173800.1;XP_017173801.1;XP_030106988.1;XP_030106989.1;XP_030106990.1;XP_030106991.1;XP_030106992.1;XP_030106993.1;XP_030106994.1;XP_030106995.1;XP_030106996.1;XP_030106997.1;XP_030106998.1;XP_030106999.1;XP_030107000.1;XP_030107001.1;XP_030107002.1;XP_030107003.1	2178563	.	7.905204	[illegible]
76954	NM_001001326,NM_029811,NR_037617,XM_006508320,XM_011241940,XM_011241941,XM_011241942,XM_011241944,XM_011241945,XM_030243084,XM_030243085,XM_030243086,XM_030243087,XM_030243088,XM_030243089,XM_030243090,XM_030243091,XR_003946582	St5	suppression of tumorigenicity 5	protein_coding	NP_001001326.1;NP_084087.2;XP_006508383.1;XP_011240242.1;XP_011240243.1;XP_011240244.1;XP_011240246.1;XP_011240247.1;XP_030098944.1;XP_030098945.1;XP_030098946.1;XP_030098947.1;XP_030098948.1;XP_030098949.1;XP_030098950.1;XP_030098951.1	108517	.	7.925473	[illegible]
76959	NM_029814	Chmp5	charged multivesicular body protein 5	protein_coding	NP_084090.1	1924209	.	25.382307	[illegible]
76960	NM_001164369,NM_029815,XM_006500381,XM_006500382,XM_006500387,XM_017319329,XM_017319330,XM_017319331,XM_017319332,XM_017319333,XM_017319334,XM_030252198,XM_030252199,XM_030252200,XM_030252201	Bcas1	breast carcinoma amplified sequence 1	protein_coding	NP_001157841.1;NP_084091.2;XP_006500444.1;XP_006500445.1;XP_006500450.1;XP_017174818.1;XP_017174819.1;XP_017174820.1;XP_017174821.1;XP_017174822.1;XP_017174823.1;XP_030108058.1;XP_030108059.1;XP_030108060.1;XP_030108061.1	1924210	.	0.054347	[illegible]
76964	NM_029816,XM_006514295,XM_006514297,XM_006514298,XM_011243600	2610028H24Rik	RIKEN cDNA 2610028H24 gene	protein_coding	NP_084092.2;XP_006514358.1;XP_006514360.1;XP_006514361.1;XP_011241902.1	1924214	.	0.000000	[illegible]
76965	NM_199065	Slitrk1	SLIT and NTRK-like family, member 1	protein_coding	NP_951020.1	2679446	.	0.010425	[illegible]
76967	NM_001163378,NM_029818,XM_006516364,XM_006516365,XM_006516366,XM_030246978,XR_001780505	2700049A03Rik	RIKEN cDNA 2700049A03 gene	protein_coding	NP_001156850.1;NP_084094.1;XP_006516427.1;XP_006516428.1;XP_006516429.1;XP_030102838.1	1924217	.	2.438017	[illegible]
76969	NM_001356552,NM_001356553,NM_023850,XM_030252202,XM_030252203	Chst1	carbohydrate sulfotransferase 1	protein_coding	NP_001343481.1;NP_001343482.1;NP_076339.1;XP_030108062.1;XP_030108063.1	1924219	.	5.058495	[illegible]
76972	NR_027059	Snhg20	small nucleolar RNA host gene 20	lncRNA	.	1924222	.	16.926973	[illegible]
76974	NM_001347605,NM_001359805,NM_029821,XM_030243092,XM_030243093	Urah	urate (5-hydroxyiso-) hydrolase	protein_coding	NP_001334534.1;NP_001346734.1;NP_084097.1;XP_030098952.1;XP_030098953.1	1916142	.	0.356233	[illegible]
76976	NM_029823	Arxes2	adipocyte-related X-chromosome expressed sequence 2	protein_coding	NP_084099.1	1924226	.	10.930594	[illegible]
76980	NM_001145162	Ube2ql1	ubiquitin-conjugating enzyme E2Q family-like 1	protein_coding	NP_001138634.1	1924230	.	2.878531	[illegible]
76982	NM_178399	Vxn	vexin	protein_coding	NP_848486.1	1924232	.	0.053724	[illegible]
76983	NM_001306178,NM_029825,NR_157117,XR_003950090	Scfd1	Sec1 family domain containing 1	protein_coding	NP_001293107.1;NP_084101.1	1924233	.	27.903176	[illegible]
76987	NM_001039201,NM_001039202,NM_001361227,NM_029826	Hdhd2	haloacid dehalogenase-like hydrolase domain containing 2	protein_coding	NP_001034290.1;NP_001034291.1;NP_001348156.1;NP_084102.1	1924237	.	8.640460	[illegible]
76992	NR_045726	Fam219aos	family with sequence similarity 219, member A, opposite strand	lncRNA	.	1924242	.	0.044706	[illegible]
76995	XR_877619	1700095A13Rik	RIKEN cDNA 1700095A13 gene	lncRNA	.	1924245	.	0.000000	[illegible]
76998	NM_001303003,NM_001303004,XM_001481022,XM_003084806	Fbxw27	F-box and WD-40 domain protein 27	protein_coding	NP_001289932.1;NP_001289933.1;XP_001481072.1;XP_003084854.	1924248	.	0.000000	[illegible]
77006	NM_029832,XM_006500390,XM_006500391,XR_001783204	Ddrgk1	DDRGK domain containing 1	protein_coding	NP_084108.1;XP_006500453.1;XP_006500454.1	1924256	.	39.030322	[illegible]
77011	NM_029835	Ticrr	TOPBP1-interacting checkpoint and replication regulator	protein_coding	NP_084111.1	1924261	.	1.055916	[illegible]
77015	NM_001143683,NM_001356554,NM_001356555,NM_029837,XM_006500394,XM_011239842,XM_030252204,XM_030252205	Mpped2	metallophosphoesterase domain containing 2	protein_coding	NP_001137155.1;NP_001343483.1;NP_001343484.1;NP_084113.1;XP_006500457.1;XP_011238144.1;XP_030108064.1;XP_030108065.1	1924265	.	5.951075	[illegible]
77017	NR_040327	E130018N17Rik	RIKEN cDNA E130018N17 gene	lncRNA	.	1924267	.	0.036015	[illegible]
77018	NM_001244952,NM_029838,NM_198711,XM_006502264,XM_006502265,XM_006502266,XM_006502267,XM_006502268,XM_006502269,XM_006502270,XM_006502271,XM_006502272,XM_006502273,XM_006502274,XM_006502275,XM_006502276,XM_006502277,XM_006502278,XM_006502279,XM_006502280,XM_006502281,XM_006502282,XM_006502283,XM_006502284,XM_006502285,XM_006502286,XM_017319804,XM_017319806,XM_030252884,XM_030252886,XM_030252887,XM_030252888,XM_030252889	Col25a1	collagen, type XXV, alpha 1	protein_coding	NP_001231881.1;NP_084114.2;NP_942004.1;XP_006502327.1;XP_006502328.1;XP_006502329.1;XP_006502330.1;XP_006502331.1;XP_006502332.1;XP_006502333.1;XP_006502334.1;XP_006502335.1;XP_006502336.1;XP_006502337.1;XP_006502338.1;XP_006502339.1;XP_006502340.1;XP_006502341.1;XP_006502342.1;XP_006502343.1;XP_006502344.1;XP_006502345.1;XP_006502346.1;XP_006502347.1;XP_006502348.1;XP_006502349.1;XP_017175293.1;XP_017175295.1;XP_030108744.1;XP_030108746.1;XP_030108747.1;XP_030108748.1;XP_030108749.1	1924268	.	1.820958	[illegible]
77022	NR_024720	2700099C18Rik	RIKEN cDNA 2700099C18 gene	transcribed_pseudogene	.	1924272	.	5.846972	[illegible]
77031	NM_001304540,NM_001304542,NM_148929,NR_130767,XM_006500395,XM_006500398,XM_006500399,XM_006500400,XM_030252206,XR_001783205	Slc9a8	solute carrier family 9 (sodium/hydrogen exchanger), member 8	protein_coding	NP_001291469.1;NP_001291471.1;NP_683731.1;XP_006500458.1;XP_006500461.1;XP_006500462.1;XP_006500463.1;XP_030108066.1	1924281	.	7.665702	[illegible]
77032	NM_029840	Tstd3	thiosulfate sulfurtransferase (rhodanese)-like domain containing 3	protein_coding	NP_084116.1	1924282	.	2.696867	[illegible]
77034	NM_029841,XM_006539279	2510039O18Rik	RIKEN cDNA 2510039O18 gene	protein_coding	NP_084117.2;XP_006539342.1	1924284	.	11.329991	[illegible]
77035	NM_029842,XM_017312384	Kdm8	lysine (K)-specific demethylase 8	protein_coding	NP_084118.1;XP_017167873.1	1924285	.	2.406159	[illegible]
77036	NM_029843	1700109H08Rik	RIKEN cDNA 1700109H08 gene	protein_coding	NP_084119.1	1924286	.	1.955977	[illegible]
77037	NM_029844,NR_155757,NR_155758	Mrap	melanocortin 2 receptor accessory protein	protein_coding	NP_084120.1	1924287	.	1.753615	[illegible]
77038	NM_001166024,NM_023854	Arfgap2	ADP-ribosylation factor GTPase activating protein 2	protein_coding	NP_001159496.1;NP_076343.2	1924288	.	19.417002	[illegible]
77040	NM_001205391,NM_001205392,NM_029846,XR_003947274,XR_003947275,XR_003947276,XR_387198	Atg16l1	autophagy related 16-like 1 (S. cerevisiae)	protein_coding	NP_001192320.1;NP_001192321.1;NP_084122.2	1924290	.	10.750617	[illegible]
77041	NM_001360843,NM_029847,XM_006517456,XM_030247462	Arsk	arylsulfatase K	protein_coding	NP_001347772.1;NP_084123.2;XP_006517519.1;XP_030103322.1	1924291	.	5.146054	[illegible]
77044	NM_175251	Arid2	AT rich interactive domain 2 (ARID, RFX-like)	protein_coding	NP_780460.3	1924294	.	11.414110	[illegible]
77045	NM_029850	Bcl7a	B cell CLL/lymphoma 7A	protein_coding	NP_084126.1	1924295	.	6.684140	[illegible]
77048	NM_029852,XM_006514300	Cep83	centrosomal protein 83	protein_coding	NP_084128.2;XP_006514363.1	1924298	.	11.423029	[illegible]

77049	XR_003948259,XR_003948260,XR_003948261,XR_003948262,XR_003948263,XR_871313	4921528I07Rik	RIKEN cDNA 4921528I07 gene, transcript variant X5	lncRNA	[illegible]
77053	NM_001256115,NM_001256116,NM_001256117,NM_001256118,NM_001359494,NM_001359496,NM_024451,XM_006504758,XM_006504762,XM_006504766,XM_006504767,XM_017321156,XM_017321157,XM_017321158,XM_017321159,XM_017321160,XM_017321161,XM_017321162,XM_017321163,XM_017321164,XM_017321165,XM_017321167,XM_017321168,XM_017321169,XM_017321170,XM_017321171,XM_030254889,XM_030254890,XM_030254891,XM_030254892,XM_030254893,XM_030254894,XM_030254895,XM_030254897	Sun1	Sad1 and UNC84 domain containing 1	protein_coding	[illegible]
77056	NM_001305423,NM_029857,XM_006539280,XM_017320451,XM_030253908,XR_001784218	Tmco4	transmembrane and coiled-coil domains 4	protein_coding	[illegible]
77057	NM_029858,XM_006525111,XM_006525112,XM_006525113	Ston1	stonin 1	protein_coding	[illegible]
77058	NM_029859	Ccdc183	coiled-coil domain containing 183	protein_coding	[illegible]
77059	NR_040298,NR_040299	4931408D14Rik	RIKEN cDNA 4931408D14 gene	lncRNA	[illegible]
77065	NM_001306203,NM_001306204,NM_178632,XR_865812	Ints7	integrator complex subunit 7	protein_coding	[illegible]
77087	NM_001081379,NR_037865,XM_030243850,XM_030243851,XM_030243852	Ankrd11	ankyrin repeat domain 11	protein_coding	[illegible]
77090	NM_001293800,NM_029865,NR_122030	Ocel1	occludin/ELL domain containing 1	protein_coding	[illegible]
77097	NM_181071,XM_006534472,XM_006534473,XM_006534474,XM_006534475,XM_006534476,XM_006534477,XM_006534478,XM_006534480,XM_006534481,XM_006534482,XM_006534483	Tanc2	tetratricopeptide repeat, ankyrin repeat and coiled-coil containing 2	protein_coding	[illegible]
77106	NM_001033178,NM_001357884,XM_030250141,XM_030250142,XM_030250143,XM_030250144,XR_003952212,XR_876333	Tmem181a	transmembrane protein 181A	protein_coding	[illegible]
77110	NM_001368177,NM_029868,XM_006503485,XM_017320452	Gpbp1l1	GC-rich promoter binding protein 1-like 1	protein_coding	[illegible]
77113	NM_178633,XM_017313011,XM_017313012	Klhl2	kelch-like 2, Mayven	protein_coding	[illegible]
77116	NM_023858,NR_024025,XM_006510676,XM_006510677,XM_006510678,XM_006510679,XM_006510681,XM_006510682,XM_006510683,XM_011242621,XM_017313692,XM_017313693,XM_03024471	Mtmr2	myotubularin related protein 2	protein_coding	[illegible]
77117	NM_001162911,NM_001360915,NM_175252,XM_011244585,XM_017315637,XM_030247463,XM_030247464,XM_030247465,XM_030247466	Zfp934	zinc finger protein 934	protein_coding	[illegible]
77124	NR_046001	9130221H12Rik	RIKEN cDNA 9130221H12 gene	lncRNA	[illegible]
77125	NM_001164724,NM_001360725,NM_133775,XM_006527463	Il33	interleukin 33	protein_coding	[illegible]
77128	NM_029870,XM_011246717,XM_017317723	Crebrf	CREB3 regulatory factor	protein_coding	[illegible]
77132	NR_033474	2810433D01Rik	RIKEN cDNA 2810433D01 gene	lncRNA	[illegible]
77134	NM_029872	Hnrnpa0	heterogeneous nuclear ribonucleoprotein A0	protein_coding	[illegible]
77189	XR_003948551,XR_865831,XR_865832	A430105J06Rik	RIKEN cDNA A430105J06 gene, transcript variant X2	lncRNA	[illegible]
77209	XR_003956282,XR_003956283,XR_869000,XR_869001	8030453O22Rik	RIKEN cDNA 8030453O22 gene, transcript variant X1	lncRNA	[illegible]
77219	NM_001252625,NM_001252626,NM_029880,XM_006516369	Ptgr2	prostaglandin reductase 2	protein_coding	[illegible]
77220	NM_001358814,NM_029881,XM_006512920,XM_006512921,XM_017314147	Tmem200a	transmembrane protein 200A	protein_coding	[illegible]
77223	NR_131071	4930407G08Rik	RIKEN cDNA 4930407G08 gene	lncRNA	[illegible]
77252	NM_029886,XM_006508329,XM_006508331,XM_011241948,XM_011241949,XM_030243094	9430038I01Rik	RIKEN cDNA 9430038I01 gene	protein_coding	[illegible]
77254	NM_001110201,NM_001357664,NM_001357666,NM_001357668,NM_029887	Yif1b	Yip1 interacting factor homolog B (S. cerevisiae)	protein_coding	[illegible]
77257	XR_374262	9430024E24Rik	RIKEN cDNA 9430024E24 gene	lncRNA	[illegible]
77264	NM_001310668,NM_029888,XM_006496318,XM_006496319,XM_030243546,XM_030243547,XM_030243549	Zfp142	zinc finger protein 142	protein_coding	[illegible]
77286	NM_029891,XM_006541409	Nkrf	NF-kappaB repressing factor	protein_coding	[illegible]
77288	NR_033569	9430021M05Rik	RIKEN cDNA 9430021M05 gene	lncRNA	[illegible]
77290	NR_131073,NR_131074,NR_131075	9430078K24Rik	RIKEN cDNA 9430078K24 gene	lncRNA	[illegible]
77300	NM_001045513,XM_006496322,XM_006496323,XM_006496324,XM_006496326,XM_006496327,XM_017312876,XM_030243566,XM_030243571,XM_030243573,XR_373268	Raph1	Ras association (RalGDS/AF-6) and pleckstrin homology domains 1	protein_coding	[illegible]
77305	NM_029896,XM_030244715	Wdr82	WD repeat domain containing 82	protein_coding	[illegible]
77318	NM_001168403,NM_001168404,NM_001168405,NM_029898,XM_006517789,XM_006517791,XM_006517792,XM_017315638,XM_017315639,XM_017315640	Ankrd55	ankyrin repeat domain 55	protein_coding	[illegible]
77323	NR_033568	9430041J12Rik	RIKEN cDNA 9430041J12 gene	lncRNA	[illegible]
77337	NM_029901	Akr1c21	aldo-keto reductase family 1, member C21	protein_coding	[illegible]
77341	NR_040483	9530026F06Rik	RIKEN cDNA 9530026F06 gene	lncRNA	[illegible]
77352	NM_001346967,XM_030243577,XM_030243584	Axdnd1	axonemal dynein light chain domain containing 1	protein_coding	[illegible]
77361	XR_001785072,XR_001785073,XR_389429,XR_389430,XR_880490,XR_880491	9430085M18Rik	RIKEN cDNA 9430085M18 gene, transcript variant X4	lncRNA	[illegible]
77371	NM_001290785,NM_175255,XM_006534485,XM_017314833,XR_388555	Sec24a	Sec24 related gene family, member A (S. cerevisiae)	protein_coding	[illegible]
77382	NR_045411,NR_045412,NR_045413	C030018K13Rik	RIKEN cDNA C030018K13 gene	lncRNA	[illegible]
77402	XR_003950200	9530018F02Rik	RIKEN cDNA 9530018F02 gene	lncRNA	[illegible]
77407	NM_198163,XM_006530500	Rab35	RAB35, member RAS oncogene family	protein_coding	[illegible]
77411	NM_176838,XM_006531492,XM_011248547,XM_030243854	Esrp2	epithelial splicing regulatory protein 2	protein_coding	[illegible]
77414	NR_077216,NR_077217	C030013G03Rik	RIKEN cDNA C030013G03 gene	lncRNA	[illegible]
77417	NR_126552	C030013C21Rik	RIKEN cDNA C030013C21 gene	lncRNA	[illegible]
77422	NM_029909,XM_006526351	C330018D20Rik	RIKEN cDNA C330018D20 gene	protein_coding	[illegible]
77428	NR_029463	9430083A17Rik	RIKEN cDNA 9430083A17 gene	lncRNA	[illegible]
77433	NR_040338	Redrum	Redrum, erythroid developmental long intergenic non-protein coding transcript	lncRNA	[illegible]
77442	NR_131083,NR_131084,NR_131085	9530020I12Rik	RIKEN cDNA 9530020I12 gene	lncRNA	[illegible]
77446	NM_175256,XM_006522715,XM_006522716,XM_006522717,XM_006522718,XM_017317159	Heg1	heart development protein with EGF-like domains 1	protein_coding	[illegible]
77453	XR_868015,XR_868016,XR_868017	Dmrta2os	doublesex and mab-3 related transcription factor like family A2, opposite strand, transcript variant X2	lncRNA	[illegible]
77462	NM_001161626,NM_001161627,NM_029912,XM_017321172,XM_017321173,XM_017321174,XM_030254899,XR_001784755,XR_001784756	Tmem116	transmembrane protein 116	protein_coding	[illegible]
77480	NM_001081378,XM_006515249,XM_006515250,XM_006515251,XM_006515252,XM_006515253,XM_006515254,XM_006515255,XM_006515256,XM_006515257,XM_006515258,XM_006515259,XM_006515260,XM_017315243,XM_017315244,XM_030246979,XM_030246980,XM_030246981	Kidins220	kinase D-interacting substrate 220	protein_coding	[illegible]
77485	NM_029916,XM_006506746,XM_006506748,XM_030255667,XM_030255668,XM_030255669	Stk31	serine threonine kinase 31	protein_coding	[illegible]
77490	NR_040435,NR_040436	E130218I03Rik	RIKEN cDNA E130218I03 gene	lncRNA	[illegible]
77505	NM_001370803	Dnhd1	dynein heavy chain domain 1	protein_coding	[illegible]
77516	XR_001783259,XR_003954017,XR_003954018	9330198N18Rik	RIKEN cDNA 9330198N18 gene, transcript variant X2	lncRNA	[illegible]
77519	NM_001082485,NM_001135019	Zfp266	zinc finger protein 266	protein_coding	[illegible]
77521	NM_001359503,NM_001359505,NM_001359511,NM_029920,XM_006504891,XM_011241018	Mtus2	microtubule associated tumor suppressor candidate 2	protein_coding	[illegible]
77522	NM_029921	Tmem213	transmembrane protein 213	protein_coding	[illegible]
77531	NM_001128086,NM_001177396,NM_001177397,NM_001177398,NM_001347053,NM_001347054,NM_001359240,NM_001359241,NM_001359242,NM_181398,XM_006514301,XM_006514302,XM_006514303,XM_006514304,XM_006514305,XM_006514306,XM_006514307,XM_006514308,XM_006514309,XM_006514310,XM_006514311,XM_006514312,XM_006514313,XM_017314148,XM_017314153,XM_017314154,XM_030245360,XM_030245361,XM_030245362,XM_030245363,XM_030245364,XM_030245365,XM_030245366,XM_030245367,XM_030245368,XM_030245369,XM_030245370,XM_030245371,XM_030245372,XM_030245373,XM_030245374,XM_030245375,XM_030245376,XM_030245377,XM_030245378,XM_030245379,XM_030245380,XM_030245381,XM_030245382,XM_030245383,XM_030245384,XM_030245385,XM_030245386,XM_030245387	Anks1b	ankyrin repeat and sterile alpha motif domain containing 1B	protein_coding	[illegible]
77532	NM_001033181	Jrkl	Jrk-like	protein_coding	[illegible]
77533	NR_026848	C030034I22Rik	RIKEN cDNA C030034I22 gene	lncRNA	[illegible]
77543	NR_040615	8030442B05Rik	RIKEN cDNA 8030442B05 gene	lncRNA	[illegible]
77552	NM_175259,XM_006529951,XR_003947287	Shisa4	shisa family member 4	protein_coding	[illegible]
77558	NR_040273,NR_040274,NR_040275	9330179D12Rik	RIKEN cDNA 9330179D12 gene	lncRNA	[illegible]
77559	NM_001081326,NM_001362367,XM_006502287,XM_011240280	Agl	amylo-1,6-glucosidase, 4-alpha-glucanotransferase	protein_coding	[illegible]
77560	NR_155521	9330198I05Rik	RIKEN cDNA 9330198I05 gene	lncRNA	[illegible]
77569	NM_001001980,NM_001256122,NM_001368863	Limch1	LIM and calponin homology domains 1	protein_coding	[illegible]
77573	NM_001359513,NM_029929,XM_006530504	Vps33a	VPS33A CORVET/HOPS core subunit	protein_coding	[illegible]
77574	NM_001355768,NM_001355769,NM_001355770,NM_029930,XM_006506752,XM_006506753,XM_006506754,XM_006506756	Tcaf1	TRPM8 channel-associated factor 1	protein_coding	[illegible]
77577	NM_029932,XM_006534486,XM_006534488,XM_011249311,XR_388556,XR_388557	Spns3	spinster homolog 3	protein_coding	[illegible]
77578	NM_029933,XM_006502288,XM_006502289,XM_006502290,XM_006502292,XM_030252891,XM_030252892	Bcl9	B cell CLL/lymphoma 9	protein_coding	[illegible]
77579	NM_175260,XM_006534489,XM_017314834,XM_017314835	Myh10	myosin, heavy polypeptide 10, non-muscle	protein_coding	[illegible]
77580	NR_163848	5730596B20Rik	RIKEN cDNA 5730596B20 gene	lncRNA	[illegible]
77582	NM_029934,XM_006540432,XM_006540433,XM_011250742	Mboat7	membrane bound O-acyltransferase domain containing 7	protein_coding	[illegible]
77583	NM_175263,XM_006534494,XM_006534497,XM_011249314,XM_011249315,XM_030246425	Notum	notum palmitoleoyl-protein carboxylesterase	protein_coding	[illegible]
77590	NM_001360768,NM_029935,XM_006508332,XM_017312386	Chst15	carbohydrate sulfotransferase 15	protein_coding	[illegible]
77591	NM_029936,XM_006510684	Ddx10	DEAD (Asp-Glu-Ala-Asp) box polypeptide 10	protein_coding	[illegible]
77592	NR_033492	4931406H21Rik	RIKEN cDNA 4931406H21 gene	lncRNA	[illegible]
77593	NM_001290425,NM_152825,XM_006538361,XM_006538362,XM_006538364,XM_006538365,XM_011250132,XM_030253909	Usp45	ubiquitin specific petidase 45	protein_coding	[illegible]
77594	NR_045318	4930552P12Rik	RIKEN cDNA 4930552P12 gene	lncRNA	[illegible]
77595	NM_029937,XM_006502293,XM_006502294,XM_006502295,XM_006502296,XM_006502297,XM_006502298,XM_006502299,XM_006502301,XM_006502302,XM_006502303,XM_006502304,XM_011240282,XM_011240283,XR_375598,XR_375599	Nup210l	nucleoporin 210-like	protein_coding	[illegible]
77596	NM_133776,XM_006525120,XM_006525121,XR_876591	Adgrf1	adhesion G protein-coupled receptor F1	protein_coding	[illegible]
77604	NM_001356562,NM_001356563,NM_198957,XM_017320453	Rbm12b2	RNA binding motif protein 12 B2	protein_coding	[illegible]
77605	NM_001347064,NM_029938,XM_030246426	H2afv	H2A histone family, member V	protein_coding	[illegible]
77609	NM_001163787,NM_029939,XM_006510685,XM_006510687,XM_011242625,XM_011242626	Ccdc151	coiled-coil domain containing 151	protein_coding	[illegible]
77610	XR_001782625,XR_001782626,XR_001782627,XR_001782628,XR_001782629,XR_001782630,XR_001782631,XR_001782632,XR_001782633,XR_001782634,XR_001782635	C330002G04Rik	RIKEN cDNA C330002G04 gene, transcript variant X5	lncRNA	[illegible]
77613	NM_001319147,XM_017312387,XM_017312388,XM_017312395,XM_030243095,XM_030243096,XM_030243097,XM_030243098,XM_030243099,XM_030243100,XM_030243101,XM_030243102,XM_030243103,XM_030243104,XM_030243105,XM_030243106,XR_003946584,XR_003946585,XR_003946586,XR_003946587,XR_003946588,XR_003946589,XR_003946590,XR_003946591,XR_003946592,XR_003946593,XR_003946594,XR_003946595,XR_003946596	Prss36	protease, serine 36	protein_coding	[illegible]
77619	NM_029942	Prelid2	PRELI domain containing 2	protein_coding	[illegible]
77622	NM_029943,XM_006528991,XM_011247878,XM_011247879,XM_030251518,XR_003953025,XR_003953026,XR_003953027,XR_003953028,XR_003953029,XR_003953030,XR_003953031,XR_878263,XR_878264,XR_878266,XR_878269,XR_878270	Apex2	apurinic/apyrimidinic endonuclease 2	protein_coding	[illegible]
77626	NM_001164609,NM_001164610,NM_001164611,NM_029945,XM_006522720,XM_006522721,XM_006522722,XM_006522725,XM_017317160,XM_030249330,XM_030249331,XR_001781871,XR_001781872	Smpd4	sphingomyelin phosphodiesterase 4	protein_coding	[illegible]
77627	NM_001161628,NM_001161629,NM_029946,XM_006521531,XM_006521533,XM_017316790,XM_017316791,XM_017316792,XM_017316793,XM_017316794,XM_017316795	Efcab6	EF-hand calcium binding domain 6	protein_coding	[illegible]
77629	NM_001356547,NM_172430,XM_006496575,XM_006496576,XM_006496577	Sphkap	SPHK1 interactor, AKAP domain containing	protein_coding	[illegible]
77630	NM_029947,XM_006535284,XM_006535285,XM_011249594,XM_030254902,XM_030254903	Prdm8	PR domain containing 8	protein_coding	[illegible]
77632	NM_029948,XM_006539283,XM_006539284,XM_011250355	Pramef12	PRAME family member 12	protein_coding	[illegible]
77633	NR_024017	4930594C11Rik	RIKEN cDNA 4930594C11 gene	transcribed_pseudogene	[illegible]
77634	NM_001358795,NM_029949,NR_152448,NR_152449,XM_006538369,XM_006538370,XM_011250134,XM_017320455,XM_030253911,XM_030253912,XM_030253913,XM_030253914,XM_030253915,XM_030253916,XM_030253918	Snapc3	small nuclear RNA activating complex, polypeptide 3	protein_coding	[illegible]
77644	NM_029951,XM_006541410	C330007P06Rik	RIKEN cDNA C330007P06 gene	protein_coding	[illegible]
77647	NM_198297,XM_006522730,XM_006522732	Trat1	T cell receptor associated transmembrane adaptor 1	protein_coding	[illegible]
77652	NM_029952,XM_006525122	Zfp955a	zinc finger protein 955A	protein_coding	[illegible]
77668	NR_033453	9130209A04Rik	RIKEN cDNA 9130209A04 gene	lncRNA	[illegible]
77669	NM_001368752,NM_029953	Arhgef38	Rho guanine nucleotide exchange factor (GEF) 38	protein_coding	[illegible]
77675	NR_029464	5033406O09Rik	RIKEN cDNA 5033406O09 gene	lncRNA	[illegible]
77683	NM_001012518,NM_001109686,NM_001109687,NM_172545,XM_006498422,XM_006498423,XM_006498424,XM_006498425,XM_006498426,XM_006498430,XM_006498431,XM_006498432,XM_017319335,XM_017319336,XM_030252207,XM_030252208,XM_030252209,XM_030252210,XM_030252211,XM_030252212,XM_030252213,XM_030252214,XM_030252215,XM_030252216,XM_030252217,XM_030252218,XM_030252219,XM_030252220,XM_030252221,XM_030252222,XM_030252223,XM_030252224,XM_030252225,XM_030252226,XM_030252227,XM_030252228,XM_030252229,XM_030252230,XM_030252231,XR_003953746,XR_374136	Ehmt1	euchromatic histone methyltransferase 1	protein_coding	[illegible]
77697	NM_001347398,NM_029956,XM_030254904	Mmab	methylmalonic aciduria (cobalamin deficiency) cblB type homolog	protein_coding	[illegible]
77700	XR_105147,XR_378399	9130208D14Rik	RIKEN cDNA 9130208D14 gene, transcript variant X2	misc_RNA	[illegible]
77701	NM_029958,XM_011239211,XM_017319337	Lcn12	lipocalin 12	protein_coding	[illegible]
77710	NR_131095	4831407H17Rik	RIKEN cDNA 4831407H17 gene	lncRNA	[illegible]
77717	NM_001369218,XM_030248816,XM_030248817	Smim41	small integral membrane protein 41	protein_coding	[illegible]
77721	NM_029963	Mrps5	mitochondrial ribosomal protein S5	protein_coding	[illegible]
77727	NM_029964,XM_006534498,XM_006534499,XM_006534500	6030468B19Rik	RIKEN cDNA 6030468B19 gene	protein_coding	[illegible]
77733	NM_001357292,NM_001357293,NM_001357294,NM_001357295,NM_029965,NR_151666,NR_151667	Rnf170	ring finger protein 170	protein_coding	[illegible]
77739	NM_001368621,NM_029967,XM_006538374,XM_006538375,XM_006538376,XM_006538377,XM_006538378,XM_006538379,XM_006538380,XM_006538381,XM_030253919	Adamtsl1	ADAMTS-like 1	protein_coding	[illegible]
77741	NR_040492	6720483E21Rik	RIKEN cDNA 6720483E21 gene	lncRNA	[illegible]
77744	NM_175265,NR_153765,XM_006519688	Bora	bora, aurora kinase A activator	protein_coding	[illegible]
77762	NR_108086	9930111H07Rik	RIKEN cDNA 9930111H07 gene	lncRNA	[illegible]
77766	NM_023876	Elp4	elongator acetyltransferase complex subunit 4	protein_coding	[illegible]
77771	NM_001290665,NM_001290666,NM_153409,NM_178634,XM_006500402,XM_030252233,XM_030252234,XM_030252235,XM_030252236,XM_030252237	Csrnp3	cysteine-serine-rich nuclear protein 3	protein_coding	[illegible]
77772	NM_029974	Dcst1	DC-STAMP domain containing 1	protein_coding	[illegible]
77777	NM_029975,XM_006512492	Ulbp1	UL16 binding protein 1	protein_coding	[illegible]
77779	NR_015567	A930007I19Rik	RIKEN cDNA A930007I19 gene	lncRNA	[illegible]
77781	NM_175266	Epm2aip1	EPM2A (laforin) interacting protein 1	protein_coding	[illegible]
77782	NM_001159369,NM_029977,XM_006522733,XM_006522734,XM_006522735,XM_006522736	Polq	polymerase (DNA directed), theta	protein_coding	[illegible]
77794	NM_029981,XM_006498439,XM_030252238,XR_003953747	Adamtsl2	ADAMTS-like 2	protein_coding	[illegible]
77799	NM_001356495,NM_029983,XM_011239845,XM_011239846	Sla2	Src-like-adaptor 2	protein_coding	[illegible]
77803	NM_029984	Shisal2b	shisa like 2B	protein_coding	[illegible]
77805	NM_001081222,XM_006526353,XR_001782403,XR_003952544	Esco1	establishment of sister chromatid cohesion N-acetyltransferase 1	protein_coding	[illegible]
77809	NM_029985	Lrrc42	leucine rich repeat containing 42	protein_coding	[illegible]
77810	NR_015618	A930015D03Rik	RIKEN cDNA A930015D03 gene	lncRNA	[illegible]
77827	NM_001347152,NM_001347153,NM_001362144,NM_001362145,NM_133922,XM_006506757,XM_006506758,XM_006506759,XM_006506760,XM_006506762,XM_006506763,XM_006506766,XM_006506767,XM_017321832,XM_030255670,XM_030255671,XM_030255672,XM_030255673,XM_030255674	Krba1	KRAB-A domain containing 1	protein_coding	[illegible]
77832	NM_029992,XM_006530508,XM_006530509,XM_006530510,XM_006530511	Tchp	trichoplein, keratin filament binding	protein_coding	[illegible]
77846	NR_110475	B230112J18Rik	RIKEN cDNA B230112J18 gene	lncRNA	[illegible]
77853	NM_001100451,XM_017313694	Msl2	MSL complex subunit 2	protein_coding	[illegible]
77857	NR_131098,NR_131099,NR_131100	9430065F17Rik	RIKEN cDNA 9430065F17 gene	lncRNA	[illegible]
77862	NM_001326361,NM_144543,XM_006510688,XM_006510689,XM_006510691,XM_006510692,XR_001779072,XR_003948057	Thyn1	thymocyte nuclear protein 1	protein_coding	[illegible]
77864	NM_001005341,XM_011249316	Ypel2	yippee like 2	protein_coding	[illegible]
77866	NR_040708	E130102H24Rik	RIKEN cDNA E130102H24 gene	lncRNA	[illegible]
77875	NR_033585	Cyp4f41-ps	cytochrome P450, family 4, subfamily f, polypeptide 41 pseudogene	transcribed_pseudogene	[illegible]
77877	NM_001166360,NM_001357761,NM_029998,XM_011245382	6030458C11Rik	RIKEN cDNA 6030458C11 gene	protein_coding	[illegible]
77878	NR_036598	6030440G07Rik	RIKEN cDNA 6030440G07 gene	lncRNA	[illegible]
77889	NM_029999	Lbh	limb-bud and heart	protein_coding	[illegible]
77891	NM_133777	Ube2s	ubiquitin-conjugating enzyme E2S	protein_coding	[illegible]
77905	NR_003243	Fate1	fetal and adult testis expressed 1	transcribed_pseudogene	[illegible]
77920	NR_045073,NR_045074,NR_045075,NR_045077	A330102I10Rik	RIKEN cDNA A330102I10 gene	lncRNA	[illegible]

77929	NM_207633,XM_006528337,XM_006528338,XM_006528339	Yipf6	Yip1 domain family, member 6	protein_coding	[illegible]
77938	NM_001347630,NM_175268,NM_212473,XM_030243107,XM_030243108	Fam53b	family with sequence similarity 53, member B	protein_coding	[illegible]
77940	NR_028376,NR_028377	A930004D18Rik	RIKEN cDNA A930004D18 gene	lncRNA	[illegible]
77945	NM_001168515,NM_023879,XM_006519689,XM_006519692,XM_006519696,XM_017316222,XM_017316223	Rpgrip1	retinitis pigmentosa GTPase regulator interacting protein 1	protein_coding	[illegible]
77951	NM_001313721,NM_030013,XM_006496328	Cyp20a1	cytochrome P450, family 20, subfamily a, polypeptide 1	protein_coding	[illegible]
77963	NM_030014,XM_006503487,XM_006503488,XM_030253920,XR_001784219,XR_001784220,XR_376374,XR_376377	Hook1	hook microtubule tethering protein 1	protein_coding	[illegible]
77974	NM_001313971,NM_030017	Rdh12	retinol dehydrogenase 12	protein_coding	[illegible]
77975	NM_030018	Tmem50b	transmembrane protein 50B	protein_coding	[illegible]
77976	NM_001004363,XM_011243603	Nuak1	NUAK family, SNF1-like kinase, 1	protein_coding	[illegible]
77980	NM_001081030,NM_001170561,XM_006521538,XM_006521539,XM_030248818	Sbf1	SET binding factor 1	protein_coding	[illegible]
77983	NR_045454	B230119M05Rik	RIKEN cDNA B230119M05 gene	lncRNA	[illegible]
77987	NM_198007,XM_030245388,XM_030245389	Ascc3	activating signal cointegrator 1 complex subunit 3	protein_coding	[illegible]
77994	NR_015543	Mir99ahg	Mir99a and Mirlet7c-1 host gene (non-protein coding)	lncRNA	[illegible]
77996	NM_001308024,NM_030021	Cutal	cutA divalent cation tolerance homolog-like	protein_coding	[illegible]
78004	NM_030024,XM_030255675	Prr15	proline rich 15	protein_coding	[illegible]
78016	NM_030025,XM_006496332,XM_006496340,XM_006496341,XM_017312896,XM_017312898,XM_017312900,XM_017312903,XM_017312906,XM_017312907,XM_017312913,XM_017312916,XM_017312918,XM_017312920,XM_030243616,XM_030243620,XR_001778457,XR_001778458	Ccdc150	coiled-coil domain containing 150	protein_coding	[illegible]
78019	NR_131107	4930519K11Rik	RIKEN cDNA 4930519K11 gene	lncRNA	[illegible]
78027	XR_003948247,XR_003948248,XR_379838,XR_871158	4930535L15Rik	RIKEN cDNA 4930535L15 gene, transcript variant X3	lncRNA	[illegible]
78038	NM_030026	Mccc2	methylcrotonoyl-Coenzyme A carboxylase 2 (beta)	protein_coding	[illegible]
78052	NM_030028,XM_006540434	Tmem190	transmembrane protein 190	protein_coding	[illegible]
78060	NR_040753	4930593C16Rik	RIKEN cDNA 4930593C16 gene	lncRNA	[illegible]
78062	NR_130987	4930595D18Rik	RIKEN cDNA 4930595D18 gene	lncRNA	[illegible]
78070	NM_001252470,NM_001357670,NM_153679,XM_011250917,XM_011250918,XR_001778010,XR_001778011,XR_001778012,XR_001778013,XR_001778014,XR_001778015,XR_001778016,XR_003946597,XR_003946598,XR_003946599,XR_882037,XR_882038,XR_8	Cpt1c	carnitine palmitoyltransferase 1c	protein_coding	[illegible]
78071	XR_003950650	5330429C05Rik	RIKEN cDNA 5330429C05 gene	lncRNA	[illegible]
78073	NR_040306,NR_040307	6720468P15Rik	RIKEN cDNA 6720468P15 gene	lncRNA	[illegible]
78088	NM_175270	Sowahb	sosondowah ankyrin repeat domain family member B	protein_coding	[illegible]
78100	NM_145609	Msantd4	Myb/SANT-like DNA-binding domain containing 4 with coiled-coils	protein_coding	[illegible]
78102	NR_077229,NR_077230,NR_077231	8430426J06Rik	RIKEN cDNA 8430426J06 gene	lncRNA	[illegible]
78103	NR_002849	8430431K14Rik	RIKEN cDNA 8430431K14 gene	lncRNA	[illegible]
78108	NR_046011	Particl	promoter of Mat2a antisense radiation induced circulating long non-coding RNA	lncRNA	[illegible]
78115	XR_383864,XR_875075,XR_875076,XR_875077,XR_875078,XR_875079	4930445E18Rik	RIKEN cDNA 4930445E18 gene, transcript variant X3	lncRNA	[illegible]
78119	XR_003947775,XR_003947777,XR_003947778,XR_865354	4930439A04Rik	RIKEN cDNA 4930439A04 gene, transcript variant X3	lncRNA	[illegible]
78134	NM_175271	Lpar4	lysophosphatidic acid receptor 4	protein_coding	[illegible]
78151	XR_003954071,XR_003954072,XR_003954073,XR_375139	4930404H24Rik	RIKEN cDNA 4930404H24 gene, transcript variant X2	misc_RNA	[illegible]
78167	XR_003948625	4930439D14Rik	RIKEN cDNA 4930439D14 gene	lncRNA	[illegible]
78174	NM_030052	Cox7b2	cytochrome c oxidase subunit 7B2	protein_coding	[illegible]
78176	NR_046192	4930471C04Rik	RIKEN cDNA 4930471C04 gene	lncRNA	[illegible]
78177	NM_207204,XM_006500406,XM_006500407,XM_006500408,XM_006500409,XM_006500411,XM_011239847,XM_030252239,XM_030252240,XM_030252241,XM_030252242,XM_030252243	Ninl	ninein-like	protein_coding	[illegible]
78178	NR_130996	4930511A08Rik	RIKEN cDNA 4930511A08 gene	lncRNA	[illegible]
78183	NR_151735	4930522P08Rik	RIKEN cDNA 4930522P08 gene	lncRNA	[illegible]
78185	NR_029473	Flicr	Foxp3 regulating long intergenic noncoding RNA	lncRNA	[illegible]
78212	NR_040381,NR_040382	Rbm46os	RNA binding motif protein 46, opposite strand	lncRNA	[illegible]
78229	NR_108103	4930596I21Rik	RIKEN cDNA 4930596I21 gene	lncRNA	[illegible]
78232	NM_001306217,NM_001306218,NM_030057	Trappc6b	trafficking protein particle complex 6B	protein_coding	[illegible]
78238	NR_130998	6030471H07Rik	RIKEN cDNA 6030471H07 gene	lncRNA	[illegible]
78244	NM_030046,XM_017316801	Dnajc21	DnaJ heat shock protein family (Hsp40) member C21	protein_coding	[illegible]
78245	NM_030063,XM_030252244	Acbd7	acyl-Coenzyme A binding domain containing 7	protein_coding	[illegible]
78246	NM_001291125,NM_001291126,NM_001291127,NM_030064,XM_006534501,XM_006534502,XM_030246427,XM_030246428,XM_030246429	Phf23	PHD finger protein 23	protein_coding	[illegible]
78248	NM_001166377,NM_001166378,NM_001166379,NM_001166380,NM_030066,XM_006528624,XM_006528625,XM_006528626,XM_006528627	Armcx1	armadillo repeat containing, X-linked 1	protein_coding	[illegible]
78249	NM_001289499,NM_001289500,NM_001289501,NM_030067,XM_017317725	Adgrf4	adhesion G protein-coupled receptor F4	protein_coding	[illegible]
78250	NM_001311098,NM_030068,XM_006511553,XM_006511554,XM_006511555,XM_006511556,XM_006511557,XR_003948058	Iqch	IQ motif containing H	protein_coding	[illegible]
78251	NM_001166218	Zfp712	zinc finger protein 712	protein_coding	[illegible]
78252	NM_030069,XM_006510693,XM_006510694,XM_006510696,XM_017313695,XM_017313696	Nxpe2	neurexophilin and PC-esterase domain family, member 2	protein_coding	[illegible]
78255	NM_001159965,NM_001159966,NM_001159967,NM_001159968,NM_001357391,NM_001357392,NM_023884,XM_006497028,XM_006497029,XM_006497030,XM_006497031,XM_006497032,XM_006497033,XM_030243656,XM_030243657,XM_030243660,XM_030243662,XM_030243663,XM_030243664,XM_030243667,XM_030243669,XR_003947313,XR_373625	Ralgps2	Ral GEF with PH domain and SH3 binding motif 2	protein_coding	[illegible]
78257	NM_001142728,NM_001142729,NM_030070,XM_006516375,XM_006516377,XM_006516378,XM_006516381,XM_011244209,XM_011244210,XM_011244211,XM_011244212,XM_011244213,XM_011244214,XM_017315246,XM_030246982,XR_003950092	Lrrc9	leucine rich repeat containing 9	protein_coding	[illegible]
78258	NM_030071	Mroh9	maestro heat-like repeat family member 9	protein_coding	[illegible]
78266	NM_001357863,NM_030074,XM_006502307,XM_030252893,XM_030252894,XM_030252895,XM_030252896,XM_030252897,XM_030252898,XM_030252899,XM_030252900	Zfp687	zinc finger protein 687	protein_coding	[illegible]
78267	NM_001359428,NM_030075,XM_006511858,XM_030244716,XM_030244717,XM_030244718,XM_030244719,XM_030244720,XM_030244721,XM_030244722,XM_030244723,XM_030244724,XM_030244725	Klhdc8b	kelch domain containing 8B	protein_coding	[illegible]
78283	NM_001081124,NM_001313751,NM_001313752,NM_001313753,NM_001359310,NM_001359311,NM_001359313,NM_001359314,NM_001359315,NM_001359317,NM_001359320,NM_001359321,NM_001359322,XM_011247881,XM_017318641,XM_030251519,XM_030251520	Map7d2	MAP7 domain containing 2	protein_coding	[illegible]
78284	NM_001307934,NM_001307935,NM_030080,XM_006502310,XM_006502311,XM_006502314,XM_006502315,XM_017319808	Creb3l4	cAMP responsive element binding protein 3-like 4	protein_coding	[illegible]
78286	NM_001111016,NM_175272,XM_006541288,XM_006541289,XM_006541290,XM_006541291,XM_006541292,XM_006541293,XM_006541294,XM_006541295,XM_006541296,XM_006541297,XM_006541298,XM_006541299,XM_006541300,XM_006541302,XM_006541303,XM_006541304,XM_006541305,XM_006541306,XM_006541307,XM_006541308,XM_006541309,XM_006541310,XM_006541311,XM_017312396,XM_017312397,XM_030243110,XM_030243111,XM_030243112,XM_030243113,XM_030243114,XM_030243115	Nav2	neuron navigator 2	protein_coding	[illegible]
78287	NM_030081,XM_006506768,XM_006506769	Rbsn	rabenosyn, RAB effector	protein_coding	[illegible]
78294	NM_001033865,NM_024277	Rps27a	ribosomal protein S27A	protein_coding	[illegible]
78303	NM_030082	H2bu2	histone cluster 3, H2ba	protein_coding	[illegible]
78304	NM_030083	Naa38	N(alpha)-acetyltransferase 38, NatC auxiliary subunit	protein_coding	[illegible]
78306	XM_003085377	Tsga10ip	testis specific 10 interacting protein	protein_coding	[illegible]
78308	NM_001308072,NM_001308074,NM_030084,XM_011246719,XM_017317726,XM_017317727,XR_003952213,XR_876592	Gpr108	G protein-coupled receptor 108	protein_coding	[illegible]
78309	NM_001081335,XM_006525135,XM_006525136,XM_011246722,XM_011246723	Cul9	cullin 9	protein_coding	[illegible]
78312	NR_130999,NR_131000,NR_131002	1810041H14Rik	RIKEN cDNA 1810041H14 gene	lncRNA	[illegible]
78317	NM_001081291,XM_006527464,XM_011247402,XR_003952826	Ccdc88b	coiled-coil domain containing 88B	protein_coding	[illegible]
78321	NM_001310521,NM_153502	Ankrd23	ankyrin repeat domain 23	protein_coding	[illegible]
78323	NM_001166364,NM_175273,XM_006511558,XM_006511559	Fam219b	family with sequence similarity 219, member B	protein_coding	[illegible]
78329	NR_046006,NR_046007	2310010J17Rik	RIKEN cDNA 2310010J17 gene	lncRNA	[illegible]
78330	NM_001083891,NM_030087	Ndufv3	NADH:ubiquinone oxidoreductase core subunit V3	protein_coding	[illegible]
78334	NM_001168304,NM_001291816,NM_001291817,NM_198164,XM_017314164,XM_030245390	Cdk19	cyclin-dependent kinase 19	protein_coding	[illegible]
78335	XR_378735	2810030D12Rik	RIKEN cDNA 2810030D12 gene	lncRNA	[illegible]
78339	NM_001308040,NM_175274,XM_017321177,XM_030254905,XM_030254906,XM_030254907	Ttyh3	tweety family member 3	protein_coding	[illegible]
78353	NR_126553,NR_126555,NR_126556	2500002B13Rik	RIKEN cDNA 2500002B13 gene	lncRNA	[illegible]
78354	NM_144544	2210407C18Rik	RIKEN cDNA 2210407C18 gene	protein_coding	[illegible]
78365	NR_038057	Lhx1os	LIM homeobox 1, opposite strand	lncRNA	[illegible]
78369	NM_001364509,NM_023892	Icam4	intercellular adhesion molecule 4, Landsteiner-Wiener blood group	protein_coding	[illegible]
78372	NM_030093	Snrnp25	small nuclear ribonucleoprotein 25 (U11/U12)	protein_coding	[illegible]
78373	NM_001162925,NM_030094,XM_006502316,XM_006502317,XM_030252901,XM_030252902	Nudt17	nudix (nucleoside diphosphate linked moiety X)-type motif 17	protein_coding	[illegible]
78376	NM_023893,NR_028258,XM_030250145	Sapcd1	suppressor APC domain containing 1	protein_coding	[illegible]
78388	NM_080638,XM_011241952	Mvp	major vault protein	protein_coding	[illegible]
78394	NM_030096	Ddx52	DEAD (Asp-Glu-Ala-Asp) box polypeptide 52	protein_coding	[illegible]
78403	NR_015489	2900041M22Rik	RIKEN cDNA 2900041M22 gene	lncRNA	[illegible]
78405	NM_198190,XM_006541312,XM_006541313,XM_011250919	Ntf5	neurotrophin 5	protein_coding	[illegible]
78408	NM_133778,XM_006522738,XM_006522740,XM_006522741,XM_006522742,XM_017317161,XM_030249332	Fam131a	family with sequence similarity 131, member A	protein_coding	[illegible]
78412	NM_001135611,NM_001347101,NM_199145,XM_030255676	Cyren	cell cycle regulator of NHEJ	protein_coding	[illegible]
78416	NM_001360117,NM_030098,NR_153407	Rnase6	ribonuclease, RNase A family, 6	protein_coding	[illegible]
78420	NR_046194	Bcas3os2	breast carcinoma amplified sequence 3 opposite strand 2	lncRNA	[illegible]
78428	NM_001170869,NM_001253704,NM_001253705,NM_030100,XM_006514320,XM_030245391,XM_030245392,XM_030245393,XM_030245394,XR_003948819	Pym1	PYM homolog 1, exon junction complex associated factor	protein_coding	[illegible]
78438	XR_386702	A930028N01Rik	RIKEN cDNA A930028N01 gene	lncRNA	[illegible]
78439	NR_153849	A930037H05Rik	RIKEN cDNA A930037H05 gene	lncRNA	[illegible]
78443	NR_046195	A930041C12Rik	RIKEN cDNA A930041C12 gene	lncRNA	[illegible]
78444	NM_030101,XM_006541315,XM_017312398,XM_017312399,XM_017312400	Pgpep1l	pyroglutamyl-peptidase I-like	protein_coding	[illegible]
78445	NR_045701	C330013E15Rik	RIKEN cDNA C330013E15 gene	lncRNA	[illegible]
78449	NR_037693	2700046A07Rik	RIKEN cDNA 2700046A07 gene	lncRNA	[illegible]
78455	NM_001368755,NM_198298,XM_006534504,XM_006534505,XM_006534506,XM_030246430	Helz	helicase with zinc finger domain	protein_coding	[illegible]
78456	XM_030243881	C030002A05Rik	RIKEN cDNA C030002A05 gene	protein_coding	[illegible]
78460	NR_040774	1700057H15Rik	RIKEN cDNA 1700057H15 gene	lncRNA	[illegible]
78465	NM_001033185,NM_001347138,XM_006497036,XM_006497037,XM_030243683,XM_030243685	Ccdc190	coiled-coil domain containing 190	protein_coding	[illegible]
78473	NM_001033186,NM_001177898,NM_001177899,XM_006534508,XM_017314838,XM_017314839,XM_030246431	Skap1	src family associated phosphoprotein 1	protein_coding	[illegible]
78482	NR_003643	1700123L14Rik	RIKEN cDNA 1700123L14 gene	transcribed_pseudogene	[illegible]
78491	NR_040588	Tspan2os	tetraspanin 2, opposite strand	lncRNA	[illegible]
78500	NR_040392	1700063D05Rik	RIKEN cDNA 1700063D05 gene	lncRNA	[illegible]
78506	NM_030110,XM_006509522,XM_017313013	Micu3	mitochondrial calcium uptake family, member 3	protein_coding	[illegible]
78512	NR_045079,NR_045080,NR_045081	3300005D01Rik	RIKEN cDNA 3300005D01 gene	lncRNA	[illegible]
78514	NM_001363417,NM_001363418,NM_001363419,NM_001363420,NM_001363421,NM_030113,NR_156496,XM_011248553,XM_017313015,XM_017313016,XM_017313019,XM_017313020,XM_017313022,XR_001778487	Arhgap10	Rho GTPase activating protein 10	protein_coding	[illegible]
78520	NR_045717	C330022C24Rik	RIKEN cDNA C330022C24 gene	lncRNA	[illegible]
78521	NM_181278	B230219D22Rik	RIKEN cDNA B230219D22 gene	protein_coding	[illegible]
78523	NM_030116,XM_006502322	Mrpl9	mitochondrial ribosomal protein L9	protein_coding	[illegible]
78536	XR_001782540,XR_001782541,XR_001782542,XR_001782543,XR_001782544,XR_003952619,XR_003952620,XR_877355,XR_87735	C030004G16Rik	RIKEN cDNA C030004G16 gene, transcript variant X1	lncRNA	[illegible]
78541	NM_001170710,NM_001170711,NM_030121,XM_006521542,XM_011245775,XM_030248819,XM_030248820	Asb8	ankyrin repeat and SOCS box-containing 8	protein_coding	[illegible]
78552	NR_024324	E130201H02Rik	RIKEN cDNA E130201H02 gene	transcribed_pseudogene	[illegible]
78558	NM_001042615,NM_030127,XM_006504186,XM_011240796,XM_030254908	Htra3	HtrA serine peptidase 3	protein_coding	[illegible]
78560	NM_054044,XM_006509222,XM_006509223,XM_006509224,XR_001778488,XR_003947355	Adgra2	adhesion G protein-coupled receptor A2	protein_coding	[illegible]
78568	XR_003951489	9630009A06Rik	RIKEN cDNA 9630009A06 gene	lncRNA	[illegible]
78569	XR_001781292,XR_003951169,XR_003951170,XR_003951171,XR_003951172,XR_003951173,XR_003951174,XR_003951175	9630015K15Rik	RIKEN cDNA 9630015K15 gene, transcript variant X5	lncRNA	[illegible]
78575	NR_029474	B430319G15Rik	RIKEN cDNA B430319G15 gene	lncRNA	[illegible]
78580	XR_379940,XR_379941,XR_871324	D730003K21Rik	RIKEN cDNA D730003K21 gene, transcript variant X1	lncRNA	[illegible]
78581	NM_030132	Utp23	UTP23 small subunit processome component	protein_coding	[illegible]
78586	NM_030133,XM_011246728,XM_011246730,XM_011246731,XM_017317728,XR_385394	Srbd1	S1 RNA binding domain 1	protein_coding	[illegible]
78593	NM_020610	Nrip3	nuclear receptor interacting protein 3	protein_coding	[illegible]
78600	NM_023898,XM_006506773	Pde6h	phosphodiesterase 6H, cGMP-specific, cone, gamma	protein_coding	[illegible]
78603	NR_037993	B230216N24Rik	RIKEN cDNA B230216N24 gene	lncRNA	[illegible]
78610	NM_178635,XM_006508349,XM_030243116	Uvrag	UV radiation resistance associated gene	protein_coding	[illegible]
78611	NR_024078	Btbd19	BTB (POZ) domain containing 19	lncRNA	[illegible]
78615	XR_376578,XR_376579,XR_376580,XR_376581,XR_376582,XR_868044,XR_868045	9530034E10Rik	RIKEN cDNA 9530034E10 gene, transcript variant X4	lncRNA	[illegible]
78616	XR_003956303,XR_003956304,XR_003956305,XR_105045,XR_377696,XR_377697,XR_869109,XR_869111,XR_869112,XR_869113,XR_869114	9530036M11Rik	RIKEN cDNA 9530036M11 gene, transcript variant X2	lncRNA	[illegible]
78617	NM_030137	Cstad	CSA-conditional, T cell activation-dependent protein	protein_coding	[illegible]
78618	NM_030138,XM_006522743,XM_006522744,XM_006522746,XM_006522747,XM_006522748,XM_006522749,XM_017317162,XM_017317163,XM_017317164,XR_384613,XR_384614	Acap2	ArfGAP with coiled-coil, ankyrin repeat and PH domains 2	protein_coding	[illegible]
78619	NM_001359276,NM_030139	Zfp449	zinc finger protein 449	protein_coding	[illegible]
78625	NM_030141,XM_006525141,XR_385395,XR_385396	Acsbg3	RIKEN cDNA 1700061G19 gene	protein_coding	[illegible]
78635	NR_040759	Kcnd3os	potassium voltage-gated channel, Shal-related family, member 3, opposite strand	lncRNA	[illegible]
78639	NR_131005,NR_131006	1700116B05Rik	RIKEN cDNA 1700116B05 gene	lncRNA	[illegible]
78651	NM_001191004,NM_030145,XM_030243855	Lsm6	LSM6 homolog, U6 small nuclear RNA and mRNA degradation associated	protein_coding	[illegible]
78653	NM_001363772,NM_001363773,NM_175277,XM_017321834,XM_030255677	Bola3	bolA-like 3 (E. coli)	protein_coding	[illegible]
78655	NM_144545	Eif3j1	eukaryotic translation initiation factor 3, subunit J1	protein_coding	[illegible]
78656	NM_001289606,NM_001289607,NM_001361136,NM_030147,XM_006526355,XM_006526358,XM_030250633,XR_001782405,XR_003952545,XR_003952546	Brd8	bromodomain containing 8	protein_coding	[illegible]
78658	NM_178113,XM_006510697,XM_006510698,XM_011242627,XM_017313698,XM_030244726,XR_001779074	Ncapd3	non-SMC condensin II complex, subunit D3	protein_coding	[illegible]
78668	NR_015604	E130112N10Rik	RIKEN cDNA E130112N10 gene	lncRNA	[illegible]
78670	NM_023900,XM_006514326,XM_006514327,XM_017314165,XM_017314166,XM_030245395	Plekhj1	pleckstrin homology domain containing, family J member 1	protein_coding	[illegible]
78672	XM_030243910	9530057J20Rik	RIKEN cDNA 9530057J20 gene	protein_coding	[illegible]
78687	XR_376450,XR_376451	0610025J13Rik	RIKEN cDNA 0610025J13 gene, transcript variant X1	lncRNA	[illegible]
78688	NM_030152,XM_006531503	Nol3	nucleolar protein 3 (apoptosis repressor with CARD domain)	protein_coding	[illegible]
78689	NM_030153,XM_006517459,XM_006517460,XM_030247467,XM_030247468,XR_003950494	Naa35	N(alpha)-acetyltransferase 35, NatC auxiliary subunit	protein_coding	[illegible]
78697	NM_001289780,NM_001289781,NM_178403,XM_011249796,XM_030254910,XR_880846	Pus7	pseudouridylate synthase 7	protein_coding	[illegible]
78703	NM_183135	Zfp972	zinc finger protein 972	protein_coding	[illegible]
78709	NM_183136,XM_006512398	Spink8	serine peptidase inhibitor, Kazal type 8	protein_coding	[illegible]
78733	NM_001162506,NM_030159,XM_030248821,XM_030248822	Troap	trophinin associated protein	protein_coding	[illegible]
78739	NR_131013	9530062K07Rik	RIKEN cDNA 9530062K07 gene	lncRNA	[illegible]
78748	NM_175279	Rassf10	Ras association (RalGDS/AF-6) domain family (N-terminal) member 10	protein_coding	[illegible]
78749	NM_001040397,NM_001177871,XM_030249333	Filip1l	filamin A interacting protein 1-like	protein_coding	[illegible]
78751	NM_178404,XM_011239850,XM_011239851	Zc3h6	zinc finger CCCH type containing 6	protein_coding	[illegible]
78752	NM_001362149,NM_030165,XM_006506777,XM_006506778	Csgalnact2	chondroitin sulfate N-acetylgalactosaminyltransferase 2	protein_coding	[illegible]
78754	NM_030166,XM_006519699,XM_006519700	Galnt15	polypeptide N-acetylgalactosaminyltransferase 15	protein_coding	[illegible]
78755	NM_001166365,NM_001166583,NM_030167,XM_006541556,XM_006541557,XM_006541558,XM_011251023,XM_030251521	Fam122b	family with sequence similarity 122, member B	protein_coding	[illegible]
78757	NM_030168,XM_006520003,XM_006520004	Rictor	RPTOR independent companion of MTOR, complex 2	protein_coding	[illegible]
78767	NM_030172,XM_006516383,XM_006516384,XM_006516385,XM_006516386,XM_006516387,XM_011244215,XM_011244216,XM_011244217,XM_030246983	Efcab11	EF-hand calcium binding domain 11	protein_coding	[illegible]
78771	NM_030174,XM_006517462,XM_006517463,XM_006517464,XM_006517466,XM_006517467,XM_006517468,XM_006517469,XM_006517470,XM_006517471,XM_006517472,XM_017315642,XM_017315643,XM_017315644,XM_030247469	Mctp1	multiple C2 domains, transmembrane 1	protein_coding	[illegible]

78772	NM_001308052,NM_030175,XM_011249733,XM_011249734,XM_011249735,XM_011249736,XM_011249737,XM_030243698,XR_880811	Hhipl2	hedgehog interacting protein-like 2
78773	NR_040761	4930511E03Rik	RIKEN cDNA 4930511E03 gene
78774	NM_001369121,NM_175280,XM_030252245,XM_030252246,XM_030252247,XM_030252248,XM_030252249,XM_030252250	Cfap61	cilia and flagella associated protein 61
78777	NM_183137,XM_006534512,XM_006534513,XM_006534514,XM_011249318	Tepsin	TEPSIN, adaptor related protein complex 4 accessory protein
78779	NM_001357300,NM_001357301,NM_001357302,NM_030176,XM_006531504,XM_006531506	Spata2l	spermatogenesis associated 2-like
78781	NM_001347122,NM_028421,NM_028864,XM_006506780,XM_006506781	Zc3hav1	zinc finger CCCH type, antiviral 1
78783	NM_001282126,NM_001282127,NM_001282128,NM_030178,XM_006506782,XM_006506783,XM_006506784,XM_006506785,XM_006506786,XM_006506787,XM_006506788,XM_017321837,XM_017321839,XM_030255678,XM_030255679	Brpf1	bromodomain and PHD finger containing, 1
78784	NM_001289613,NM_001289616,NM_001289620,NM_001357784,NM_001357785,NM_001357786,NM_001357787,NM_172434,NR_110353,NR_151857,XM_006502323,XM_006502324,XM_006502327,XM_006502328,XM_006502330,XM_006502332,XM_006502334,XM_006502335,XM_006502338,XM_011240284,XM_030252903,XM_030252904,XM_030252905,XM_030252906,XM_030252907,XM_030252908,XM_030252909,XM_030252910,XM_030252911,XM_030252912,XM_030252913,XM_030252914,XR_001783779,XR_003954424,XR_003954425,XR_003954426,XR_003954427,XR_375601	Celf3	CUGBP, Elav-like family member 3
78785	NM_001271483,NM_001271484,NM_030179,NM_175378,NR_073185,XM_006525143,XM_006525144,XM_006525145,XM_006525146,XM_006525147,XM_006525148,XM_006525149,XM_006525150,XM_006525151,XM_006525153,XM_006525154,XM_030250146,XM_030250147,XR_001782131,XR_003952214,XR_003952215,XR_38	Clip4	CAP-GLY domain containing linker protein family, member 4
78787	NM_030180,XM_006519703,XM_006519705,XM_011245225,XM_011245226,XM_011245227,XM_030248095,XM_030248096,XR_001781168,XR_001781169,XR_001781170,XR_001781171,XR_00178	Usp54	ubiquitin specific peptidase 54
78789	NM_001359143,NM_026103,NM_030181,XM_006528631	Vsig1	V-set and immunoglobulin domain containing 1
78795	NM_001310702,NM_027456,NM_030184,XM_006529952,XM_006529953,XM_006529954,XM_006529956,XM_006529957,XM_006529958,XM_006529959,XM_006529960,XM_011248111,XM_011248112,XR_001778466,XR_003947323,XR_387199,XR_387200,XR_3872	Armc9	armadillo repeat containing 9
78796	NM_001308144,NM_001309445,NM_030185,XM_006504189,XM_006504190,XM_006504191,XM_006504192,XR_001784758	Zcchc4	zinc finger, CCHC domain containing 4
78797	NM_001082476,NM_001252541,NM_001252542,XM_006498441,XM_006498442,XM_006498443,XM_006498444,XM_006498445,XM_006498446,XM_006498447,XM_006498448,XM_006498450,XM_006498451,XM_006498452,XM_006498455,XM_030252251,XM_030252252,XM_030252253,XR_003953748,XR_003953749,XR_003953750,XR_003953751,XR_003953752,XR_374138,XR_374139,XR_374141,XR_866028,XR_866029	Ndor1	NADPH dependent diflavin oxidoreductase 1
78798	NM_001114361,NM_001114362,NM_001286567,NM_001357886,NM_199466,XM_006525155,XM_006525156,XM_006525157,XM_006525158,XM_006525160,XM_006525161,XM_006525162,XM_006525163,XM_011246732,XM_017317729,XM_030250148	Eml4	echinoderm microtubule associated protein like 4
78801	NM_030187,XM_006516390,XM_006516391	Ak7	adenylate kinase 7
78802	NM_030188	Ttc30a1	tetratricopeptide repeat domain 30A1
78803	NM_001081253,NM_001368658,XM_006520197,XM_006520198,XM_011245385,XM_017316803	Fbxo43	F-box protein 43
78806	NM_030189,XM_006539285,XM_006539286,XM_006539287,XM_006539288,XM_006539289,XM_011250356,XM_011250357,XM_017320457,XM_017320458	Stpg1	sperm tail PG rich repeat containing 1
78808	NM_001081344,XM_006512926,XM_006512927,XM_006512928,XM_011243237,XR_001779636,XR_003948820,XR_003948821,XR_380094	Stxbp5	syntaxin binding protein 5 (tomosyn)
78809	NM_030192,XM_011246045	4930562C15Rik	RIKEN cDNA 4930562C15 gene
78816	NM_198101,XM_006509797,XM_006509798,XM_006509799,XM_030243856,XM_030243857,XR_003947356	Gmip	Gem-interacting protein
78825	NM_024282,XM_030243714	Desi2	desumoylating isopeptidase 2
78826	NM_001357810,NM_172435	P2ry10	purinergic receptor P2Y, G-protein coupled 10
78829	NM_023910,NM_029805	Tsc22d4	TSC22 domain family, member 4
78830	NM_172436,XM_011239853,XM_030252254	Slc25a12	solute carrier family 25 (mitochondrial carrier, Aralar), member 12
78832	NM_001172096,NM_001172097,NM_030197,XM_006527468	Cacul1	CDK2 associated, cullin domain 1
78833	NM_030198	Gins3	GINS complex subunit 3 (Psf3 homolog)
78834	NM_030199,XM_006521543,XM_030248823	Zfp623	zinc finger protein 623
78849	NR_015457	B430010I23Rik	RIKEN cDNA B430010I23 gene
78878	NR_028380,NR_028381	Ftx	Ftx transcript, Xist regulator (non-protein coding)
78885	NM_030205	Coro7	coronin 7
78887	NM_001363222,NM_001363223,NM_001363224,NM_030207,XM_006514894,XM_006514896,XM_006514897,XM_006514898,XM_006514899,XM_006514900,XM_006514901,XM_006514902,XM_006514903,XM_006514908,XM_011243792,XM_011243795,XM_011243797,XM_011243798,XM_017314841,XM_017314842,XM_017314844,XM_017314847,XM_030246433,XM_030246434,XM_030246435,XR_001780076,XR_001780077,XR_001780078,XR_003949530,XR_003949531,XR_003949532,XR_380988,XR_872210	Sfi1	Sfi1 homolog, spindle assembly associated (yeast)
78889	NM_001042565,NM_019653,XM_030246436,XR_003949533,XR_003949534	Wsb1	WD repeat and SOCS box-containing 1
78890	NM_030208,XM_006504193	Trmt44	tRNA methyltransferase 44
78891	NM_001361921,NM_001361922,NM_023912,NR_155264,XM_030251145,XR_388273	Scyl1	SCY1-like 1 (S. cerevisiae)
78892	NM_001310635,NM_030209,XM_006531508,XM_006531509	Crispld2	cysteine-rich secretory protein LCCL domain containing 2
78893	NM_153585,XM_006512399,XM_017313699,XM_030244727,XR_003948060,XR_003948061,XR_003948062	Cnot10	CCR4-NOT transcription complex, subunit 10
78894	NM_030210	Aacs	acetoacetyl-CoA synthetase
78895	NM_172437,XM_006521544	Pus7l	pseudouridylate synthase 7-like
78896	NM_024283	Ecrg4	ECRG4 augurin precursor
78899	XR_003947497,XR_003947498,XR_003947499	4932443L11Rik	RIKEN cDNA 4932443L11 gene, transcript variant X1
78903	NM_030215	Wrnip1	Werner helicase interacting protein 1
78906	NM_030218,XM_006514328,XM_006514329,XM_006514330,XM_006514331,XM_006514332,XM_006514333	Misp	mitotic spindle positioning
78908	NM_207205,XM_006502342,XM_006502343,XM_011240287,XM_030252915	Igsf3	immunoglobulin superfamily, member 3
78910	NM_080847,XM_006505206,XM_011241089	Asb15	ankyrin repeat and SOCS box-containing 15
78911	NM_030219	Trim42	tripartite motif-containing 42
78912	NM_001080964,NM_001363225,NM_001363226,NM_030220,XM_006534518,XM_006534519,XM_006534520	Sp2	Sp2 transcription factor
78913	NM_001081068,XM_006523111,XR_384950,XR_384951	Ltn1	listerin E3 ubiquitin protein ligase 1
78914	NM_001308095,NM_030221,XM_006508655,XM_011242021,XM_011242022,XM_011242023,XM_017312401,XM_030243117,XM_030243118,XR_378612	Nadsyn1	NAD synthetase 1
78917	NR_131014	4930455G09Rik	RIKEN cDNA 4930455G09 gene
78919	NM_030224	Fndc8	fibronectin type III domain containing 8
78920	NM_030225,XM_006516392	Dlst	dihydrolipoamide S-succinyltransferase (E2 component of 2-oxo-glutarate complex)
78921	NM_030226	9130019O22Rik	RIKEN cDNA 9130019O22 gene
78923	NM_001081328	Chsy3	chondroitin sulfate synthase 3
78925	NM_175283,XM_006517473,XM_006517474	Srd5a1	steroid 5 alpha-reductase 1
78926	NM_001190406,NM_001190408,NM_030228,NM_144560,XM_017314850,XM_030246437	Gas2l1	growth arrest-specific 2 like 1
78928	NM_001362644,NM_001362645,NM_133779,XM_006500421,XM_030252255	Pigt	phosphatidylinositol glycan anchor biosynthesis, class T
78929	NM_030229,XM_006521545,XR_001781579	Polr3h	polymerase (RNA) III (DNA directed) polypeptide H
78933	NM_001048189,NM_001284190,NM_030231,XM_011240652,XM_011240653,XM_011240654,XM_011240655,XM_011240656,XM_011240657,XM_011240658	Agbl4	ATP/GTP binding protein-like 4
78934	NR_029475	4930581F22Rik	RIKEN cDNA 4930581F22 gene
78935	NM_030233,XM_006541317,XR_001778017,XR_003946600	Saal1	serum amyloid A-like 1
78937	NM_030235,XM_006506790	Avl9	AVL9 cell migration associated
78938	NM_001146085,NM_001146086,NM_030236,NR_027414,XM_006519710,XM_006519712,XM_006519713,XM_006519714,XM_006519717,XM_006519718,XM_006519719,XM_006519720,XM_006519722,XM_006519723,XM_017316225,XM_017316226,XM_030248097	Fbxo34	F-box protein 34
78943	NM_023913	Ern1	endoplasmic reticulum (ER) to nucleus signalling 1
78977	NM_024286,XM_006512931	Popdc3	popeye domain containing 3
79043	NM_001163750,NM_001163751,NM_027141,NR_152137,XM_006525165,XM_006525166,XM_006525168,XM_006525169,XM_006525170,XM_011246733,XM_030250149,XM_030250150,XM_030250151,XM_030250152,XM_030250153	Spsb3	splA/ryanodine receptor domain and SOCS box containing 3
79044	NM_023260	Mrps34	mitochondrial ribosomal protein S34
79059	NM_019730	Nme3	NME/NM23 nucleoside diphosphate kinase 3
79189	gene-Apc-ps1	Apc-ps1	adenomatosis polyposis coli, pseudogene 1
79196	NM_001199227,NM_024289,XM_006508657,XM_006508659,XM_011242024,XR_001778018	Osbpl5	oxysterol binding protein-like 5
79201	NM_001308111,NM_024290,NR_131763,XM_006508660,XM_006508661	Tnfrsf23	tumor necrosis factor receptor superfamily, member 23
79202	NM_001311145,NM_023680,XM_030243119,XR_003946601	Tnfrsf22	tumor necrosis factor receptor superfamily, member 22
79221	NM_001271386,NM_024124,XM_006515261,XM_006515262,XM_006515264,XM_006515266,XM_006515271,XM_006515273,XM_006515275,XM_006515276,XM_011243911,XM_011243912,XM_011243913,XM_011243916,XM_011243917,XM_011243918,XM_011243921,XM_011243922,XM_017315248,XM_017315249,XM_017315250,XM_017315251,XM_017315252,XM_030246984,XM_030246985,XM_030246986,XM_030246987,XM_030246988,XM_030246989,XM_030246990,XM_030246991,XM_030246992	Hdac9	histone deacetylase 9
79233	NM_024467	Zfp319	zinc finger protein 319
79235	NM_023624	Lrat	lecithin-retinol acyltransferase (phosphatidylcholine-retinol-O-acyltransferase)
79263	NM_001357889,NM_024468,NM_178281,XM_006525171,XM_030250154	Trim39	tripartite motif-containing 39
79264	NM_001170552,NM_030675,NR_033173,XM_006503617,XM_006503618,XM_030254911,XM_030254912	Krit1	KRIT1, ankyrin repeat containing
79362	NM_001271768,NM_024469	Bhlhe41	basic helix-loop-helix family, member e41
79456	NM_058214,XM_006521546,XM_006521547,XM_006521548,XM_017316804,XM_017316805,XM_030248824,XR_001781580,XR_384	Recql4	RecQ protein-like 4
79459	NM_001277340	Aldoart2	aldolase 1 A, retrogene 2
79464	NM_001310612,NM_024471,XM_030254913	Lias	lipoic acid synthetase
79554	NM_024472	Cptp	ceramide-1-phosphate transfer protein
79555	NM_024473	BC005537	cDNA sequence BC005537
79560	NM_024475,XM_006534522	Ublcp1	ubiquitin-like domain containing CTD phosphatase 1
79565	NM_001347490,NM_001359535,NM_001359536,NM_001359537,NM_001359538,NM_024479,XM_006504522,XM_006504524,XM_006504527,XM_006504528,XM_006504529,XM_030254914,XM_030254915	Mettl27	methyltransferase like 27
79566	NM_001161338,NM_001308167,NM_001308168,NM_024480,XM_006534523	Sh3bp5l	SH3 binding domain protein 5 like
80280	NM_001308183,NM_001363255,NM_001363256,NM_030248,XM_006534526,XM_011249319,XM_030246438	Cdk5rap3	CDK5 regulatory subunit associated protein 3
80281	NM_001163332,NM_001163333,NM_030249,XM_011240288,XM_030252916	Cttnbp2nl	CTTNBP2 N-terminal like
80283	NM_001362152,NM_030251,NR_155424,XM_006506791,XM_006506792,XM_006506793,XM_030255680,XM_030255681,XR_003956	Abtb1	ankyrin repeat and BTB (POZ) domain containing 1
80284	NM_030252	Smim12	small integral membrane protein 12
80285	NM_030253,XM_006522755,XM_030249336,XM_030249337,XM_030249338	Parp9	poly (ADP-ribose) polymerase family, member 9
80286	NM_001357304,NM_001357305,NM_001357306,NM_030254,XM_017313024,XM_017313026,XM_017313027,XM_017313028,XM_017313029,XM_030243860	Tusc3	tumor suppressor candidate 3
80287	NM_001160415,NM_001347041,NM_030255,XM_006521553,XM_017316806,XM_017316808,XR_001781583	Apobec3	apolipoprotein B mRNA editing enzyme, catalytic polypeptide 3
80288	NM_001357490,NM_030256,XM_006510701,XM_006510702,XM_006510703,XM_006510704,XM_017313700,XM_030244728	Bcl9l	B cell CLL/lymphoma 9-like
80289	NM_030257,XM_006517475	Lysmd3	LysM, putative peptidoglycan-binding, domain containing 3
80290	NM_001038703,NM_001362206,NM_030258,NR_033147,XM_030254916	Gpr146	G protein-coupled receptor 146
80291	NM_030259	Rilpl2	Rab interacting lysosomal protein-like 2
80292	NM_030260,NM_173002,XR_377502	Zxdc	ZXD family zinc finger C
80294	NM_030262,XR_001779639,XR_380457	Pofut2	protein O-fucosyltransferase 2
80297	NM_001199234,NM_001199235,NM_001199236,NM_032610,XM_006540436,XM_006540437	Sptbn4	spectrin beta, non-erythrocytic 4
80334	NM_001199242,NM_001199243,NM_001199244,NM_001199245,NM_030265,XM_030254917,XM_030254918,XM_030254919,XM_030254920,XM_030254921,XM_030254922	Kcnip4	Kv channel interacting protein 4
80385	NM_019742	Tusc2	tumor suppressor 2, mitochondrial calcium regulator
80509	NM_001290688,NM_020000,NM_173719,XM_006503491,XM_006503492,XM_011240659	Med8	mediator complex subunit 8
80515	NR_027827	Rnf227	ring finger protein 227
80517	NM_020586,XM_006510706	Herpud2	HERPUD family member 2
80707	NM_019573,XM_006531510,XM_006531511,XM_006531512,XM_006531513,XM_006531514,XM_006531515,XM_011248556,XM_011248557,XM_011248558	Wwox	WW domain-containing oxidoreductase
80708	NM_001289677,NM_001289678,NM_028733,NM_030880,XM_006500424,XM_006500425,XM_006500427,XM_006500428,XM_030252256	Pacsin3	protein kinase C and casein kinase substrate in neurons 3
80718	NM_001082553,NM_001302798,NM_001302799,NM_030554,XM_006526364	Rab27b	RAB27B, member RAS oncogene family
80719	NM_030691,XM_006508351	Igsf6	immunoglobulin superfamily, member 6
80720	NM_001024954,NM_001357308,NR_151668,NR_151669,NR_151670,XM_006509800,XM_030243861,XM_030243862,XM_030243864,XR_001778490	Pbx4	pre B cell leukemia homeobox 4
80721	NM_030556,XM_006496579,XM_006496580,XM_011238715,XM_011238716	Slc19a3	solute carrier family 19, member 3
80732	NM_001289621,NM_001289622,NM_001289623,NM_030557	Mynn	myoneurin
80733	NM_030558,XM_017317165	Car15	carbonic anhydrase 15
80743	NM_001362651,NM_030559,NR_156013,NR_156014,XM_006500430,XR_003953753,XR_374543	Vps16	VSP16 CORVET/HOPS core subunit
80744	NM_001290740,NM_001362657,NM_001362658,NM_001362659,NM_001362664,NM_030560,NM_172667,XM_006500433,XM_006500435,XM_006500437,XM_030252257	Cwc22	CWC22 spliceosome-associated protein
80748	NM_030561,XM_006525173,XM_006525174	BC004004	cDNA sequence BC004004
80749	NM_001141921,NM_030562,XM_006540438,XM_030243120	Lrfn1	leucine rich repeat and fibronectin type III domain containing 1
80750	NM_030563,XM_006531516,XM_006531517	N4bp1	NEDD4 binding protein 1
80751	NM_030564,XM_006530512	Rnf34	ring finger protein 34
80752	NM_001359593,NM_030565	Fam20c	family with sequence similarity 20, member C
80782	NM_001364615,NM_030599	Klrb1b	killer cell lectin-like receptor subfamily B member 1B
80794	NM_001161844,NM_023224	Cblc	Casitas B-lineage lymphoma c
80795	NM_019979	Selenok	selenoprotein K
80796	NM_020036	Calm4	calmodulin 4
80797	NM_030601,XM_006502346,XM_017319811,XM_017319812	Clca3a2	chloride channel accessory 3A2
80828	NR_002851	Snord82	small nucleolar RNA, C/D box 82
80837	NM_023275	Rhoj	ras homolog family member J
80838	NM_030609	H1f1	histone cluster 1, H1a
80857	NM_030610	Fgf20	fibroblast growth factor 20
80859	NM_001159394,NM_001159395,NM_030612,XM_030249339	Nfkbiz	nuclear factor of kappa light polypeptide gene enhancer in B cells inhibitor, zeta
80860	NM_031871,XM_006534529	Ghdc	GH3 domain containing
80861	NM_030150	Dhx58	DEXH (Asp-Glu-X-His) box polypeptide 58
80876	NM_030694,XM_030243121	Ifitm2	interferon induced transmembrane protein 2
80877	NM_001077687,NM_001077688,NM_030695,XM_006502347,XM_006502348,XM_006502349,XM_006502350,XM_006502351,XM_006502352,XM_006502353,XM_006502354	Lrba	LPS-responsive beige-like anchor
80879	NM_001038653,NM_001038654,NM_030696,XM_011249321,XM_011249322	Slc16a3	solute carrier family 16 (monocarboxylic acid transporters), member 3
80880	NM_001357162,NM_030697,XM_006525175	Kank3	KN motif and ankyrin repeat domains 3
80883	NM_001163348,NM_001163349,NM_001163350,NM_030699,NM_133488,XM_006502355,XM_011240291,XM_011240292,XM_011240293,XM_011240294,XM_011240295,XR_001783784	Ntng1	netrin G1

80884	NM_001199246,NM_001358566,NM_001358567,NM_001358568,NM_030700,XM_006528998,XM_011247882	Maged2	melanoma antigen, family D, 2	protein_coding	NP_001186175.1;NP_001345495.1;NP_001345496.1;NP_001345497.1;NP_109625.1;XP_006529061.1;XP_011246184.1	1933391	.	132.046897	[illegible]
80885	NM_030701	Hcar2	hydroxycarboxylic acid receptor 2	protein_coding	NP_109626.1	1933383	.	0.048667	[illegible]
80886	NM_001163571,NM_030702,XM_006534530	Senp3	SUMO/sentrin specific peptidase 3	protein_coding	NP_001157043.1;NP_109627.3;XP_006534593.1	2158736	.	26.183471	[illegible]
80888	NM_030704	Hspb8	heat shock protein 8	protein_coding	NP_109629.1	2135756	.	12.303082	[illegible]
80889	NM_030705	Tlnrd1	talin rod domain containing 1	protein_coding	NP_109630.1	1891420	.	6.124429	[illegible]
80890	NM_001271725,NM_001271726,NM_001271727,NM_001271728,NM_030706,XM_011240296,XM_011240297,XM_011240298,XM_011240299,XM_011240300,XM_011240301,XM_011240302,XM_011240303,XM_011240304,XM_011240305,XM_017319813,XM_017319814,XM_017319815,XM_030252917,XM_030252918,XM_030252919	Trim2	tripartite motif-containing 2	protein_coding	NP_001258654.1;NP_001258655.1;NP_001258656.1;NP_001258657.1;NP_109631.2;XP_011238598.1;XP_011238599.1;XP_011238600.1;XP_011238601.1;XP_011238602.1;XP_011238603.1;XP_011238604.1;XP_011238605.1;XP_011238606.1;XP_011238607.1;XP_017175302.1;XP_017175303.1;XP_017175304.1;XP_030108777.1;XP_030108778.1;XP_030108779.1	1933163	.	5.782719	[illegible]
80891	NM_030707,XM_006502369	Fcrls	Fc receptor-like S, scavenger receptor	protein_coding	NP_109632.2;XP_006502432.1	1933397	.	12.706604	[illegible]
80892	NM_001368671,NM_030708,XM_006530105,XM_006530106,XM_017319816	Zfhx4	zinc finger homeodomain 4	protein_coding	NP_001355600.1;NP_109633.2;XP_006530168.1;XP_006530169.1;XP_017175305.1	2137668	.	13.589617	[illegible]
80893	NM_001359460,NM_030709,XM_006510708,XM_006510709,XR_001779075,XR_001779076	Tmprss5	transmembrane protease, serine 5 (spinesin)	protein_coding	NP_001346389.1;NP_109634.2;XP_006510771.1;XP_006510772.1	1933407	.	0.058375	[illegible]
80898	NM_030711,XM_006517476,XM_030247471	Erap1	endoplasmic reticulum aminopeptidase 1	protein_coding	NP_109636.1;XP_006517539.1;XP_030103331.1	1933403	.	4.782220	[illegible]
80901	NM_030712	Cxcr6	chemokine (C-X-C motif) receptor 6	protein_coding	NP_109637.3	1934582	.	0.059720	[illegible]
80902	NM_001359464,NM_030713,NR_153114,XM_011242629	Zfp202	zinc finger protein 202	protein_coding	NP_001346393.1;NP_109638.2;XP_011240931.1	1933401	.	1.405927	[illegible]
80903	NM_030614	Fgf16	fibroblast growth factor 16	protein_coding	NP_085117.2	1931627	.	0.029858	[illegible]
80904	NM_001368332,NM_030714,XM_006514334,XM_006514336,XM_006514337,XM_030245396,XM_030245397,XM_030245398,XM_030245399,XM_030245400,XM_030245401,XM_030245402,XM_030245403,XM_030245404,XM_030245405	Dtx3	deltex 3, E3 ubiquitin ligase	protein_coding	NP_001355261.1;NP_109639.1;XP_006514397.1;XP_006514399.1;XP_006514400.1;XP_030101256.1;XP_030101257.1;XP_030101258.1;XP_030101259.1;XP_030101260.1;XP_030101261.1;XP_030101262.1;XP_030101263.1;XP_030101264.1;XP_030101265.1	2135752	.	65.207420	[illegible]
80905	NM_001364947,NM_030715	Polh	polymerase (DNA directed), eta (RAD 30 related)	protein_coding	NP_001351876.1;NP_109640.1	1891457	.	7.974380	[illegible]
80906	NM_001276358,NM_030716,NM_145703,NM_145704,XM_006527469,XM_006527470,XM_017318315	Kcnip2	Kv channel-interacting protein 2	protein_coding	NP_001263287.1;NP_109641.2;NP_663749.1;NP_663750.1;XP_006527532.1;XP_006527533.1;XP_017173804.1	2135916	.	0.046054	[illegible]
80907	NM_030717,XM_006511561	Lactb	lactamase, beta	protein_coding	NP_109642.1;XP_006511624.1	1933395	.	4.015662	[illegible]
80909	NM_030719	Castor2	cytosolic arginine sensor for mTORC1 subunit 2	protein_coding	NP_109644.2	1933384	.	4.850060	[illegible]
80910	NM_030720,XM_006521554	Gpr84	G protein-coupled receptor 84	protein_coding	NP_109645.1;XP_006521617.1	1934129	.	0.049766	[illegible]
80911	NM_001357018,NM_001357019,NM_001357020,NM_030721,XM_006504196,XM_006504197,XM_006504199,XM_006504200,XM_006504201,XM_011240801,XM_017321179,XM_030254924	Acox3	acyl-Coenzyme A oxidase 3, pristanoyl	protein_coding	NP_001343947.1;NP_001343948.1;NP_001343949.1;NP_109646.2;XP_006504259.1;XP_006504260.1;XP_006504262.1;XP_006504263.1;XP_006504264.1;XP_011239103.1;XP_017176668.1;XP_030110784.	1933156	.	8.825417	[illegible]
80912	NM_001159603,NM_001159604,NM_001159605,NM_001159606,NM_001356564,NM_001356565,NM_001356566,NM_030722,XM_006539291,XM_006539292,XM_006539293,XM_006539294,XM_006539295,XM_006539296,XM_006539297,XM_006539299,XM_006539300,XM_006539301,XM_006539302,XM_006539303,XM_006539304,XM_006539305,XM_006539306,XM_006539308,XM_006539309,XM_006539314,XM_011250358,XM_011250359,XM_017320459,XM_017320460,XM_017320461,XM_017320462,XM_017320463,XM_030253922,XM_030253923,XM_030253924,XM_030253925,XM_030253926,XM_030253927,XM_030253928,XM_030253929,XM_030253930,XM_030253931,XM_030253932,XM_030253933,XM_030253934,XM_030253935,XM_030253936,XM_030253937,XM_030253938	Pum1	pumilio RNA-binding family member 1	protein_coding	NP_001153075.1;NP_001153076.1;NP_001153077.1;NP_001153078.1;NP_001343493.1;NP_001343494.1;NP_001343495.1;NP_109647.2;XP_006539354.1;XP_006539355.1;XP_006539356.1;XP_006539357.1;XP_006539358.1;XP_006539359.1;XP_006539360.1;XP_006539362.1;XP_006539363.1;XP_006539364.1;XP_006539365.1;XP_006539366.1;XP_006539367.1;XP_006539368.1;XP_006539369.1;XP_006539371.1;XP_006539372.1;XP_006539377.1;XP_011248660.1;XP_011248661.1;XP_017175948.1;XP_017175949.1;XP_017175950.1;XP_017175951.1;XP_017175952.1;XP_030109782.1;XP_030109783.1;XP_030109784.1;XP_030109785.1;XP_030109786.1;XP_030109787.1;XP_030109788.1;XP_030109789.1;XP_030109790.1;XP_030109791.1;XP_030109792.1;XP_030109793.1;XP_030109794.1;XP_030109795.1;XP_030109796.1;XP_030109797.1;XP_030109798.1	1931749	.	16.289382	[illegible]
80913	NM_001160219,NM_001160220,NM_001160221,NM_001160222,NM_001310519,NM_030723,NR_027670,XM_006515277,XM_006515278,XM_006515279,XM_006515280,XM_006515282,XM_006515283,XM_006515284,XM_006515285,XM_017315254,XM_017315255,XM_017315256,XM_017315257,XM_017315258,XM_017315259,XM_030246993,XM_030246994,XM_030246995,XM_030246996,XM_030246997,XM_030246998,XM_030246999,XM_030247000,XM_030247001,XM_030247002,XM_030247003	Pum2	pumilio RNA-binding family member 2	protein_coding	NP_001153691.1;NP_001153692.1;NP_001153693.1;NP_001153694.1;NP_001297448.1;NP_109648.2;XP_006515340.1;XP_006515341.1;XP_006515342.1;XP_006515343.1;XP_006515345.1;XP_006515346.1;XP_006515347.1;XP_006515348.1;XP_017170743.1;XP_017170744.1;XP_017170745.1;XP_017170746.1;XP_017170747.1;XP_017170748.1;XP_030102853.1;XP_030102854.1;XP_030102855.1;XP_030102856.1;XP_030102857.1;XP_030102858.1;XP_030102859.1;XP_030102860.1;XP_030102861.1;XP_030102862.1;XP_030102863.1	1931751	.	27.075838	[illegible]
80914	NM_030724,XM_006497040,XM_017312968,XM_030243724,XM_030243727,XM_030243728	Uck2	uridine-cytidine kinase 2	protein_coding	NP_109649.1;XP_006497103.1;XP_017168457.1;XP_030099584.1;XP_030099587.1;XP_030099588.1	1931744	.	13.159384	[illegible]
80915	NM_001356485,NM_001356486,NM_023173,NR_151476,NR_1514	Dusp12	dual specificity phosphatase 12	protein_coding	NP_001343414.1;NP_001343415.1;NP_075662.2	1890614	.	12.957937	[illegible]
80976	NM_030725	Syt13	synaptotagmin XIII	protein_coding	NP_109650.1	1933945	.	1.539693	[illegible]
80978	NM_030726	Mrgprh	MAS-related GPR, member H	protein_coding	NP_109651.1	1934134	.	0.020615	[illegible]
80981	NM_025404	Arl4d	ADP-ribosylation factor-like 4D	protein_coding	NP_079680.1	1933155	.	4.280836	[illegible]
80982	NM_030728,XM_006508352,XM_006508353,XM_006508354,XM_030243122	Cemip	cell migration inducing protein, hyaluronan binding	protein_coding	NP_109653.3;XP_006508415.1;XP_006508416.1;XP_006508417.1;XP_030098982.1	2443629	.	2.642026	[illegible]
80985	NM_020267	Trim44	tripartite motif-containing 44	protein_coding	NP_064663.2	1931835	.	31.774715	[illegible]
80986	NM_001004140,XM_006509226	Ckap2	cytoskeleton associated protein 2	protein_coding	NP_001004140.2;XP_006509289.1	1931797	.	9.249148	[illegible]
80987	NM_030729,XM_006511860,XM_030244729,XR_003948065,XR_003948066	Nckipsd	NCK interacting protein with SH3 domain	protein_coding	NP_109654.2;XP_006511923.1;XP_030100589.1	1931834	.	5.022919	[illegible]
81000	NM_030730,XM_017313702	Rad54l2	RAD54 like 2 (S. cerevisiae)	protein_coding	NP_109655.2;XP_017169191.1	1933196	.	4.418242	[illegible]
81003	NM_001361538,NM_001361539	Trim23	tripartite motif-containing 23	protein_coding	NP_001348467.1;NP_001348468.1	1933161	.	7.587028	[illegible]
81004	NM_030732,XM_006535579,XM_006535580,XM_006535581,XM_011249718,XM_011249719,XM_011249721,XM_011249723,XM_011249724,XM_017319817,XM_030252920,XM_030252921,XM_030252922	Tbl1xr1	transducin (beta)-like 1X-linked receptor 1	protein_coding	NP_109657.2;XP_006535642.1;XP_006535643.1;XP_006535644.1;XP_011248020.1;XP_011248021.1;XP_011248023.1;XP_011248025.1;XP_011248026.1;XP_017175306.1;XP_030108780.1;XP_030108781.1;XP_030108782.1	2441730	.	7.599603	[illegible]
81006	NM_030733,XM_006538382,XM_006538383,XM_030253940	Gpr63	G protein-coupled receptor 63	protein_coding	NP_109658.2;XP_006538445.1;XP_006538446.1;XP_030109800.1	2135884	.	0.065434	[illegible]
81018	NM_001360930,NM_030743,NR_153838	Rnf114	ring finger protein 114	protein_coding	NP_001347859.1;NP_109668.2	1933159	.	16.266844	[illegible]
81489	NM_001308227,NM_018808	Dnajb1	DnaJ heat shock protein family (Hsp40) member B1	protein_coding	NP_001295156.1;NP_061278.1	1931874	.	10.552075	[illegible]
81500	NM_001360814,NM_030749,XM_006526365,XM_006526367,XM_011247043,XM_030250634,XM_030250635	Sil1	endoplasmic reticulum chaperone SIL1 homolog (S. cerevisiae)	protein_coding	NP_001347743.1;NP_109674.2;XP_006526428.1;XP_006526430.1;XP_011245345.1;XP_030106494.1;XP_030106495.1	1932040	.	18.087257	[illegible]
81535	NM_030750	Sgpp1	sphingosine-1-phosphate phosphatase 1	protein_coding	NP_109675.1	2135760	.	8.949612	[illegible]
81600	NM_023186	Chia1	chitinase, acidic 1	protein_coding	NP_075675.2	1932052	.	0.000000	[illegible]
81601	NM_001199247,NM_001199248,NM_001199249,NM_001362370,NM_001362371,NM_001362372,NM_178637,NR_155544,XM_006531860	Kat5	K(lysine) acetyltransferase 5	protein_coding	NP_001186176.1;NP_001186177.1;NP_001186178.1;NP_001349299.1;NP_001349300.1;NP_001349301.1;NP_848752.1;XP_006531923.1	1932051	.	15.002642	[illegible]
81630	NM_020625	Zbtb22	zinc finger and BTB domain containing 22	protein_coding	NP_065650.1	1931870	.	8.109683	[illegible]
81701	NM_152922,XM_006525179,XM_006525180	Egfl8	EGF-like domain 8	protein_coding	NP_690886.3;XP_006525242.1;XP_006525243.1	1932094	.	2.038057	[illegible]
81702	NM_030886,NM_198010,XM_006535287,XM_006535288,XM_006535289,XM_006535290,XM_006535291,XM_011249595,XM_030254925	Ankrd17	ankyrin repeat domain 17	protein_coding	NP_112148.2;NP_932127.2;XP_006535350.3;XP_006535351.3;XP_006535352.3;XP_006535353.1;XP_006535354.1;XP_011247897.3;XP_030110785.1	1932101	.	14.983952	[illegible]
81703	NM_001205052,NM_001205053,NM_030887,XM_011244218,XM_011244219	Jdp2	Jun dimerization protein 2	protein_coding	NP_001191981.1;NP_001191982.1;NP_112149.2;XP_011242520.1;XP_011242521.1	1932093	.	23.200201	[illegible]
81799	NM_001204134,NM_030888,XR_383836	C1qtnf3	C1q and tumor necrosis factor related protein 3	protein_coding	NP_001191063.1;NP_112150.1	1932136	.	169.131896	[illegible]
81840	NM_030889,XM_006504202,XM_030254926	Sorcs2	sortilin-related VPS10 domain containing receptor 2	protein_coding	NP_112151.2;XP_006504265.1;XP_030110786.1	1932289	.	15.150567	[illegible]
81845	NM_001128597,NM_032460,XM_006525181,XM_006525182,XM_006525183,XM_006525184,XM_030250157,XM_030250158	Gpank1	G patch domain and ankyrin repeats 1	protein_coding	NP_001122069.1;NP_115849.2;XP_006525244.1;XP_006525245.1;XP_006525246.1;XP_006525247.1;XP_030106017.1;XP_030106018.1	2148975	.	4.256224	[illegible]
81877	NM_031176,XM_006525185,XM_006525186,XM_006525187,XM_006525188,XM_006525189,XM_006525191	Tnxb	tenascin XB	protein_coding	NP_112453.2;XP_006525248.1;XP_006525249.1;XP_006525250.1;XP_006525251.1;XP_006525252.1;XP_006525254.1	1932137	.	1.095074	[illegible]
81879	NM_023755,XM_006529961	Tfcp2l1	transcription factor CP2-like 1	protein_coding	NP_076244.2;XP_006530024.1	2444691	.	0.165040	[illegible]
81896	NM_001167763,NM_031177,XM_006506797,XM_006506798,XM_006506799	Ift122	intraflagellar transport 122	protein_coding	NP_001161235.1;NP_112454.2;XP_006506860.1;XP_006506861.1;XP_006506862.1	1932386	.	4.504071	[illegible]
81897	NM_031178	Tlr9	toll-like receptor 9	protein_coding	NP_112455.2	1932389	.	0.478141	[illegible]
81898	NM_031179,XM_030243736,XR_003947332,XR_003947333	Sf3b1	splicing factor 3b, subunit 1	protein_coding	NP_112456.2;XP_030099596.1	1932339	.	76.610026	[illegible]
81904	NM_133189,XM_030243123,XM_030243124,XM_030243125	Cacng7	calcium channel, voltage-dependent, gamma subunit 7	protein_coding	NP_573452.3;XP_030098983.1;XP_030098984.1;XP_030098985.1	1932374	.	3.468263	[illegible]
81905	NM_133190	Cacng8	calcium channel, voltage-dependent, gamma subunit 8	protein_coding	NP_573453.2	1932376	.	0.000000	[illegible]
81906	NM_001003947	Cyp4x1	cytochrome P450, family 4, subfamily x, polypeptide 1	protein_coding	NP_001003947.1	1932403	.	0.000000	[illegible]
81907	NM_001359465,NM_178638,XM_006511863,XM_006511864,XM_006511865,XM_006511866,XM_006511867,XM_006511868,XM_030244730,XM_030244731	Tmem108	transmembrane protein 108	protein_coding	NP_001346394.1;NP_848753.1;XP_006511926.1;XP_006511927.1;XP_006511928.1;XP_006511929.1;XP_006511930.1;XP_006511931.1;XP_030100590.1;XP_030100591.1	1932411	.	0.144651	[illegible]
81909	NM_001356994,NM_024231,XM_006531862,XM_006531863	Zfpl1	zinc finger like protein 1	protein_coding	NP_001343923.1;NP_077193.1;XP_006531925.1;XP_006531926.1	1891017	.	19.506289	[illegible]
81910	NM_024281,NM_133626,XM_006500440,XM_006500441,XM_006500443,XM_006500444	Rrbp1	ribosome binding protein 1	protein_coding	NP_077243.2;NP_598329.1;XP_006500503.1;XP_006500504.1;XP_006500506.1;XP_006500507.1	1932395	.	114.793153	[illegible]
81913	NR_027919	Bambi-ps1	BMP and activin membrane-bound inhibitor, pseudogene (Xenopus laevis)	transcribed_pseudogene	.	1932402	.	0.727373	[illegible]
83379	NM_031180,XM_006504204	Klb	klotho beta	protein_coding	NP_112457.1;XP_006504267.1	1932466	.	0.013777	[illegible]
83382	NM_031181,XM_006541318,XM_006541319,XM_006541320,XM_006541321,XM_030243126,XM_030243127,XM_030243128,XM_030243129	Siglece	sialic acid binding Ig-like lectin E	protein_coding	NP_112458.2;XP_006541381.1;XP_006541382.1;XP_006541383.1;XP_006541384.1;XP_030098986.1;XP_030098987.1;XP_030098988.1;XP_030098989.1	1932475	.	0.622481	[illegible]
83383	NM_031182,XM_006522762,XM_006522763,XM_006522764,XM_006522765,XM_006522766,XM_011246047,XM_017317166,XM_030249340,XM_030249341	Tfap4	transcription factor AP4	protein_coding	NP_112459.1;XP_006522825.1;XP_006522826.1;XP_006522827.1;XP_006522828.1;XP_006522829.1;XP_011244349.1;XP_017172655.1;XP_030105200.1;XP_030105201.1	103239	.	3.922036	[illegible]
83395	NM_001363230,NM_031183	Sp6	trans-acting transcription factor 6	protein_coding	NP_001350159.1;NP_112460.1	1932575	.	0.190842	[illegible]
83396	NM_031184,XM_006522768,XM_011246048,XM_011246049,XM_011246050	Glis2	GLIS family zinc finger 2	protein_coding	NP_112461.2;XP_006522831.1;XP_011244350.1;XP_011244351.1;XP_011244352.1	1932535	.	20.377764	[illegible]
83397	NM_031185	Akap12	A kinase (PRKA) anchor protein (gravin) 12	protein_coding	NP_112462.1	1932576	.	20.750475	[illegible]
83398	NM_001293682,NM_031186,NR_121616,XM_006502370,XM_006502371,XM_006502374,XM_006502376,XM_030252923,XM_030252924,XR_003954428	Ndst3	N-deacetylase/N-sulfotransferase (heparan glucosaminyl) 3	protein_coding	NP_001280611.1;NP_112463.2;XP_006502433.1;XP_006502434.1;XP_006502437.1;XP_006502439.1;XP_030108783.1;XP_030108784.1	1932544	.	0.364857	[illegible]
83408	NM_031247	Gimap3	GTPase, IMAP family member 3	protein_coding	NP_112537.2	1932723	.	0.022087	[illegible]
83409	NM_001362368,NM_031248,NR_155541,NR_155542,NR_155543	Lamtor2	late endosomal/lysosomal adaptor, MAPK and MTOR activator 2	protein_coding	NP_001349297.1;NP_112538.1	1932697	.	28.168463	[illegible]
83410	NM_031249	Cstf2t	cleavage stimulation factor, 3' pre-RNA subunit 2, tau	protein_coding	NP_112539.2	1932622	.	9.286206	[illegible]
83429	NM_001357891,NM_001357892,NM_031251,XM_006534535,XM_006534536,XM_011249323,XM_011249325,XM_030246439,XM_030246440,XM_030246441	Ctns	cystinosis, nephropathic	protein_coding	NP_001344820.1;NP_001344821.1;NP_112541.1;XP_006534598.1;XP_006534599.1;XP_011247625.1;XP_011247627.1;XP_030102299.1;XP_030102300.1;XP_030102301.1	1932872	.	9.995242	[illegible]
83430	NM_031252	Il23a	interleukin 23, alpha subunit p19	protein_coding	NP_112542.1	1932410	.	0.139449	[illegible]
83431	NM_001363304,NM_001363305,NM_023668,XM_030246442	Ndel1	nudE neurodevelopment protein 1 like 1	protein_coding	NP_001350233.1;NP_001350234.1;NP_076157.2;XP_030102302.1	1932915	.	40.750359	[illegible]
83433	NM_001272078,NM_031254	Trem2	triggering receptor expressed on myeloid cells 2	protein_coding	NP_001259007.1;NP_112544.1	1913150	.	3.594255	[illegible]
83434	NM_001159671,NM_031255,XM_006540443,XM_006540444,XM_030243130	Rsph6a	radial spoke head 6 homolog A (Chlamydomonas)	protein_coding	NP_001153143.1;NP_112545.2;XP_006540506.1;XP_006540507.1;XP_030098990.1	1927643	.	0.000000	[illegible]
83435	NM_031256,XM_006500446	Plekha3	pleckstrin homology domain-containing, family A (phosphoinositide binding specific) member 3	protein_coding	NP_112546.1;XP_006500509.1	1932515	.	9.145497	[illegible]
83436	NM_031257,XM_006509227,XM_006509228,XM_006509230	Plekha2	pleckstrin homology domain-containing, family A (phosphoinositide binding specific) member 2	protein_coding	NP_112547.1;XP_006509290.1;XP_006509291.1;XP_006509293.1	1928144	.	4.088639	[illegible]
83437	gene-Pgam1-ps1	Pgam1-ps1	phosphoglycerate mutase 1, pseudogene 1	pseudogene	.	1933119	.	0.396626	[illegible]
83453	NM_001114385,NM_001358592,NM_031258,XM_006529001,XM_006529002,XM_017318643,XM_030251522	Chrdl1	chordin-like 1	protein_coding	NP_001107857.1;NP_001345521.1;NP_112548.2;XP_006529064.1;XP_006529065.1;XP_017174132.1;XP_030107382.1	1933172	.	14.752738	[illegible]
83454	NM_001289735,NM_001289736,NM_031259,XM_011247765	Nxf2	nuclear RNA export factor 2	protein_coding	NP_001276664.1;NP_001276665.1;NP_112549.1;XP_011246067.1	1933192	.	0.124469	[illegible]
83456	NM_031260,XM_006521555,XM_006521556,XM_006521557	Mov10l1	Mov10 like RISC complex RNA helicase 1	protein_coding	NP_112550.2;XP_006521618.1;XP_006521619.1;XP_006521620.1	1891384	.	0.006096	[illegible]
83485	NM_031375,NR_028053	Ngrn	neugrin, neurite outgrowth associated	protein_coding	NP_113552.3	1933212	.	10.861867	[illegible]
83486	NM_001359524,NM_001359525,NM_001359526,NM_148930,NR_153190,NR_153191,XM_006511872,XM_006511875,XM_006511876,XM_030244732,XM_030244733,XM_030244734,XM_030244735,XR_001779079	Rbm5	RNA binding motif protein 5	protein_coding	NP_001346453.1;NP_001346454.1;NP_001346455.1;NP_683732.1;XP_006511935.1;XP_006511938.1;XP_006511939.1;XP_030100592.1;XP_030100593.1;XP_030100594.1;XP_030100595.1	1933204	.	61.041897	[illegible]
83490	NM_031376,XM_006527472	Pik3ap1	phosphoinositide-3-kinase adaptor protein 1	protein_coding	NP_113553.1;XP_006527535.1	1933177	.	3.511108	[illegible]
83491	NM_031377	Pramel1	preferentially expressed antigen in melanoma-like 1	protein_coding	NP_113554.1	1890541	.	0.000000	[illegible]
83492	NM_031378	Gsdmc	gasdermin C	protein_coding	NP_113555.1	1933176	.	0.000000	[illegible]
83493	NM_001357485,NM_030692,XM_017313703	Sacm1l	SAC1 suppressor of actin mutations 1-like (yeast)	protein_coding	NP_001344414.1;NP_109617.1;XP_017169192.1	1933169	.	17.804616	[illegible]
83554	NM_031380	Fstl3	follistatin-like 3	protein_coding	NP_113557.1	1890391	.	3.870862	[illegible]
83556	NM_031382,XM_017318644	Tex16	testis expressed gene 16	protein_coding	NP_113559.2;XP_017174133.1	1890545	.	0.017116	[illegible]
83557	NM_145833,XM_006539317,XM_011250361	Lin28a	lin-28 homolog A (C. elegans)	protein_coding	NP_665832.1;XP_006539380.1;XP_011248663.1	1890546	.	0.000000	[illegible]
83558	NM_001167997,NM_031384	Tex11	testis expressed gene 11	protein_coding	NP_001161469.1;NP_113561.2	1933237	.	0.069930	[illegible]
83560	NM_001199293,NM_031386,XM_011249327,XM_011249328,XM_011249329,XM_017314855,XM_017314856,XM_017314857,XM_017314858,XR_001780081	Tex14	testis expressed gene 14	protein_coding	NP_001186222.1;NP_113563.2;XP_011247629.1;XP_011247630.1;XP_011247631.1;XP_017170344.1;XP_017170345.1;XP_017170346.1;XP_017170347.1	1933227	.	0.066672	[illegible]
83561	NM_001002238,NM_001002240,NM_001002241,NM_031387,XM_006527473,XM_006527474,XM_006527475,XM_006527476,XM_006527477,XM_006527478,XM_006527479	Tdrd1	tudor domain containing 1	protein_coding	NP_001002238.1;NP_001002240.1;NP_001002241.1;NP_113564.2;XP_006527536.1;XP_006527537.1;XP_006527538.1;XP_006527539.1;XP_006527540.1;XP_006527541.1;XP_006527542.1	1933218	.	0.074292	[illegible]
83563	NM_031388,XM_017318645	Usp26	ubiquitin specific peptidase 26	protein_coding	NP_113565.2;XP_017174134.1	1933247	.	0.000000	[illegible]
83564	NM_031389	Nlrp4c	NLR family, pyrin domain containing 4C	protein_coding	NP_113566.2	1890518	.	0.000000	[illegible]
83565	NM_031390,XM_006528634	Pramel3	preferentially expressed antigen in melanoma-like 3	protein_coding	NP_113567.1;XP_006528697.1	1890657	.	0.000000	[illegible]
83602	NM_031391,NM_175335,XM_017315260	Gtf2a1	general transcription factor II A, 1	protein_coding	NP_113568.2;NP_780544.1;XP_017170749.1	1933277	.	5.771296	[illegible]
83603	NM_148941	Elovl4	elongation of very long chain fatty acids (FEN1/Elo2, SUR4/Elo3, yeast)-like 4	protein_coding	NP_683743.2	1933331	.	2.435516	[illegible]
83669	NM_031392	Wdr6	WD repeat domain 6	protein_coding	NP_113569.1	1930140	.	22.242306	[illegible]
83671	NM_001040085,NM_001040087,NM_001040088,NM_001289583,NM_001289584,NM_001289585,NM_001289586,NM_001368878,NM_031394,NR_110348,XM_006508356,XM_006508357,XM_006508358,XM_006508359,XM_006508360,XM_030243131	Sytl2	synaptotagmin-like 2	protein_coding	NP_001035174.1;NP_001035176.1;NP_001035177.1;NP_001276512.1;NP_001276513.1;NP_001276514.1;NP_001276515.1;NP_001355807.1;NP_113571.2;XP_006508419.1;XP_006508420.1;XP_006508421.1;XP_006508422.1;XP_006508423.1;XP_030098991.1	1933366	.	10.577179	[illegible]
83672	NM_031395,NM_183368,NM_183370,XM_006523259,XM_006523260,XM_006523261,XM_006523263,XM_006523264,XM_011246181,XM_030250159,XM_030250160,XR_001782133	Sytl3	synaptotagmin-like 3	protein_coding	NP_113572.1;NP_899224.2;NP_899226.2;XP_006523322.1;XP_006523323.1;XP_006523324.1;XP_006523326.1;XP_006523327.1;XP_011244483.1;XP_030106019.1;XP_030106020.1	1933367	.	0.016324	[illegible]
83673	NR_002896	Snhg1	small nucleolar RNA host gene 1	lncRNA	.	3763743	.	65.931320	[illegible]
83674	NM_031396,XM_006527480,XM_006527481,XM_006527482,XR_001782623,XR_003952827,XR_003952828	Cnnm1	cyclin M1	protein_coding	NP_113573.2;XP_006527543.1;XP_006527544.1;XP_006527545.1	1891366	.	0.019990	[illegible]
83675	NM_001347189,NM_031397	Bicc1	BicC family RNA binding protein 1	protein_coding	NP_001334118.1;NP_113574.1	1933388	.	30.031588	[illegible]
83679	NM_001039376,NM_001289701,NM_001289702,NM_178080,NR_110360	Pde4dip	phosphodiesterase 4D interacting protein (myomegalin)	protein_coding	NP_001034465.2;NP_001276630.1;NP_001276631.1;NP_835181.2	1891434	.	27.852213	[illegible]
83691	NM_001356551,NM_031402,XM_006495592,XM_006495594,XM_011238409,XM_011238410	Crispld1	cysteine-rich secretory protein LCCL domain containing 1	protein_coding	NP_001343480.1;NP_113579.2;XP_006495655.1;XP_006495657.1;XP_011236711.1;XP_011236712.1	1934666	.	26.367185	[illegible]
83701	NM_001109909,NM_001109910,NM_001359602,NM_031405,XM_006504630,XM_006504631,XM_006504632,XM_030254927,XM_030254928,XM_030254929,XM_030254930	Srrt	serrate RNA effector molecule homolog (Arabidopsis)	protein_coding	NP_001103379.1;NP_001103380.1;NP_001346531.1;NP_113582.1;XP_006504693.1;XP_006504694.1;XP_006504695.1;XP_030110787.1;XP_030110788.1;XP_030110789.1;XP_030110790.1	1933527	.	40.103538	[illegible]
83703	NM_001368298,NM_031403	Dbr1	debranching RNA lariats 1	protein_coding	NP_001355227.1;NP_113580.2	1931520	.	5.636161	[illegible]
83704	NM_001359611,NM_031406,XM_030254931,XM_030254932,XM_030254933,XM_030254934,XM_030254935,XM_030254936,XM_030254937,XM_030254938,XR_001784760	Slc12a9	solute carrier family 12 (potassium/chloride transporters), member 9	protein_coding	NP_001346540.1;NP_113583.2;XP_030110791.1;XP_030110792.1;XP_030110793.1;XP_030110794.1;XP_030110795.1;XP_030110796.1;XP_030110797.1;XP_030110798.1	1933532	.	4.030979	[illegible]
83762	NM_001100395,NM_001286421,NM_001313767,NM_031875,XM_006504206,XM_006504208,XM_006504209,XM_011240802,XM_017321185,XM_017321186,XR_001784761,XR_376841,XR_376842	Otof	otoferlin	protein_coding	NP_001093865.1;NP_001273350.1;NP_001300696.1;NP_114081.2;XP_006504269.1;XP_006504271.1;XP_006504272.1;XP_011239104.1;XP_017176674.1;XP_017176675.1	1891247	.	0.019932	[illegible]
83766	NM_031404,XM_006504635,XM_006504636,XM_006504637,XM_006504638,XM_006504639,XM_006504640,XM_017321187,XM_030254939,XM_030254940,XM_030254941,XM_030254942,XM_030254943,XM_030254944,XM_030254945,XM_030254946	Actl6b	actin-like 6B	protein_coding	NP_113581.1;XP_006504698.1;XP_006504699.1;XP_006504700.1;XP_006504701.1;XP_006504702.1;XP_006504703.1;XP_017176676.1;XP_030110799.1;XP_030110800.1;XP_030110801.1;XP_030110802.1;XP_030110803.1;XP_030110804.1;XP_030110805.1;XP_03011080	1933548	.	0.000000	[illegible]
83767	NM_031877,XM_006512932,XM_006512933,XM_030245406	Wasf1	WAS protein family, member 1	protein_coding	NP_114083.1;XP_006512995.1;XP_006512996.1;XP_030101266.1	1890563	.	1.184318	[illegible]
83768	NM_031843,XR_374142	Dpp7	dipeptidylpeptidase 7	protein_coding	NP_114031.2	1933213	.	6.826976	[illegible]
83770	NM_031873	Tas1r2	taste receptor, type 1, member 2	protein_coding	NP_114079.1	1933546	.	0.000000	[illegible]
83771	NM_031872	Tas1r3	taste receptor, type 1, member 3	protein_coding	NP_114078.1	1933547	.	0.095550	[illegible]
83796	NM_001130187,NM_031878,XM_006534552	Smarcd2	SWI/SNF related, matrix associated, actin dependent regulator of chromatin, subfamily d, member 2	protein_coding	NP_001123659.1;NP_114084.2;XP_006534615.2	1933621	.	21.838819	[illegible]
83797	NM_031842	Smarcd1	SWI/SNF related, matrix associated, actin dependent regulator of chromatin, subfamily d, member 1	protein_coding	NP_114030.2	1933623	.	10.893710	[illegible]
83813	NM_031880,XM_006534554,XR_879778	Tnk1	tyrosine kinase, non-receptor, 1	protein_coding	NP_114086.3;XP_006534617.1	1930958	.	0.069697	[illegible]
83814	NM_001114386,NM_031881,XM_006526370,XM_006526371,XM_006526373,XM_006526374,XM_006526376,XM_006526377,XM_006526378,XM_006526379,XM_006526380,XM_006526382,XM_011247044,XM_011247047,XM_017318015,XM_017318016,XM_030250636,XM_030250637,XM_030250638,XM_030250639,XM_030250640,XM_030250641,XM_030250642,XM_030250643,XM_030250644	Nedd4l	neural precursor cell expressed, developmentally down-regulated gene 4-like	protein_coding	NP_001107858.1;NP_114087.2;XP_006526433.1;XP_006526434.1;XP_006526436.1;XP_006526437.1;XP_006526439.1;XP_006526440.1;XP_006526441.1;XP_006526442.1;XP_006526443.1;XP_006526445.1;XP_011245346.1;XP_011245349.1;XP_017173504.1;XP_017173505.1;XP_030106496.1;XP_030106497.1;XP_030106498.1;XP_030106499.1;XP_030106500.1;XP_030106501.1;XP_030106502.1;XP_030106503.1;XP_030106504.1	1933754	.	1.228077	[illegible]
83815	NM_031863	Cenpq	centromere protein Q	protein_coding	NP_114069.2	1933744	.	2.057125	[illegible]
83885	NM_001159275	Slc25a2	solute carrier family 25 (mitochondrial carrier, ornithine transporter) member 2	protein_coding	NP_001152747.1	2137907	.	0.168850	[illegible]
83921	NM_001033759,NM_031997	Cemip2	cell migration inducing hyaluronidase 2	protein_coding	NP_001028931.1;NP_114386.3	1890373	.	15.460199	[illegible]
83922	NM_031998,NR_130775,NR_130781,XM_006505207,XM_006505208,XM_006505209,XM_006505210,XM_006505211,XM_006505213,XM_011241090,XM_030255682	Cep41	centrosomal protein 41	protein_coding	NP_114387.1;XP_006505270.1;XP_006505271.1;XP_006505272.1;XP_006505273.1;XP_006505274.1;XP_006505276.1;XP_011239392.1;XP_030111542.1	1891414	.	4.751831	[illegible]
83924	NM_031999,XM_006516805,XR_382078	Gpr137b	G protein-coupled receptor 137B	protein_coding	NP_114388.2;XP_006516868.1	1891463	.	22.244823	[illegible]
83925	NM_001310481,NM_001310485,NM_032000,XM_006521560,XM_006521563,XM_011245776,XM_011245777,XM_011245778,XM_011245780,XM_030248826,XM_030248827,XM_030248828	Trps1	transcriptional repressor GATA binding 1	protein_coding	NP_001297410.1;NP_001297414.1;NP_114389.2;XP_006521623.1;XP_006521626.1;XP_011244078.1;XP_011244079.1;XP_011244080.1;XP_011244082.1;XP_030104686.1;XP_030104687.1;XP_030104688.	1927616	.	7.765409	[illegible]
83945	NM_001135112,NM_023646	Dnaja3	DnaJ heat shock protein family (Hsp40) member A3	protein_coding	NP_001128584.1;NP_076135.3	1933786	.	12.109752	[illegible]
83946	NM_001081216,XM_006511564,XM_006511565,XM_030244736,XM_030244737,XM_030244738,XM_030244739,XM_030244740	Phip	pleckstrin homology domain interacting protein	protein_coding	NP_001074685.1;XP_006511627.1;XP_006511628.1;XP_030100596.1;XP_030100597.1;XP_030100598.1;XP_030100599.1;XP_03010060	1932404	.	6.921497	[illegible]
83961	NM_032002,XM_017313704,XM_017313705,XM_017313706	Nrg4	neuregulin 4	protein_coding	NP_114391.1;XP_017169193.1;XP_017169194.1;XP_017169195.1	1933833	.	0.107025	[illegible]
83962	NM_146193,XM_006541322	Btbd1	BTB (POZ) domain containing 1	protein_coding	NP_666305.2;XP_006541385.1	1933765	.	25.155831	[illegible]
83964	NM_023277,XM_030244741	Jam3	junction adhesion molecule 3	protein_coding	NP_075766.1;XP_030100601.1	1933825	.	20.788602	[illegible]
83965	NM_001168620,NM_001329618,NM_001329619,NM_001329620,NM_001329621,NM_001329622,NM_032003	Enpp5	ectonucleotide pyrophosphatase/phosphodiesterase 5	protein_coding	NP_001162091.1;NP_001316547.1;NP_001316548.1;NP_001316549.1;NP_001316550.1;NP_001316551.1;NP_114392.1	1933830	.	4.561747	[illegible]
83984	NM_032004	Tssk6	testis-specific serine kinase 6	protein_coding	NP_114393.1	2148775	.	0.126559	[illegible]
83993	NM_032005,XM_006497043	Tbx19	T-box 19	protein_coding	NP_114394.1;XP_006497106.1	1891158	.	0.017534	[illegible]
83997	NM_001310445,NM_001347494,NM_032008,XM_006519725,XM_006519726,XM_006519727,XM_006519728,XM_006519729,XM_006519730,XM_006519731,XM_006519732,XM_006519734,XM_006519735,XM_006519736,XM_006519737,XM_006519738,XM_017316229,XM_030248099,XM_030248100,XM_030248101,XM_030248102	Slmap	sarcolemma associated protein	protein_coding	NP_001297374.1;NP_001334423.1;NP_114397.3;XP_006519788.1;XP_006519789.1;XP_006519790.1;XP_006519791.1;XP_006519792.1;XP_006519793.1;XP_006519794.1;XP_006519795.1;XP_006519797.1;XP_006519798.1;XP_006519799.1;XP_006519800.1;XP_006519801.1;XP_017171718.1;XP_030103959.1;XP_030103960.1;XP_030103961.1;XP_030103962.1	1933549	.	13.790820	[illegible]
84004	NM_001359530,NM_023061,XM_030244742,XM_030244743	Mcam	melanoma cell adhesion molecule	protein_coding	NP_001346459.1;NP_075548.2;XP_030100602.1;XP_030100603.1	1933966	.	26.145471	[illegible]
84035	NM_032396,XM_006514912,XM_006514913	Kremen1	kringle containing transmembrane protein 1	protein_coding	NP_115772.2;XP_006514975.1;XP_006514976.1	1933988	.	29.623680	[illegible]
84036	NM_001363407,NM_001363408,NM_032397,XM_006509806,XM_006509808,XM_030243865,XM_030243866,XR_003947357,XR_379049,XR_870467	Kcnn1	potassium intermediate/small conductance calcium-activated channel, subfamily N, member 1	protein_coding	NP_001350336.1;NP_001350337.1;NP_115773.2;XP_006509869.2;XP_006509871.1;XP_030099725.1;XP_030099726.1	1933993	.	2.425258	[illegible]
84092	NM_001252580,NM_019729,XM_011239857	Usp8	ubiquitin specific peptidase 8	protein_coding	NP_001239509.1;NP_062703.2;XP_011238159.1	1934029	.	18.327662	[illegible]
84094	NM_032398,XM_011242342	Plvap	plasmalemma vesicle associated protein	protein_coding	NP_115774.2;XP_011240644.1	1890497	.	29.654065	[illegible]
84095	NM_145501,XM_011247405,XM_011247406,XR_877696	Pi4k2a	phosphatidylinositol 4-kinase type 2 alpha	protein_coding	NP_663476.1;XP_011245707.1;XP_011245708.1	1934031	.	9.818626	[illegible]
84111	NM_001302203,XM_006502394	Gpr87	G protein-coupled receptor 87	protein_coding	NP_001289132.1;XP_006502457.1	1934133	.	0.000000	[illegible]

84112	NM_032400	Sucnr1	succinate receptor 1	protein_coding	NP_115776.2	1934135	.	0.000000	[illegible]
84113	NM_133949,XM_006541323,XR_391372	Ptov1	prostate tumor over expressed gene 1	protein_coding	NP_598710.1;XP_006541386.1	1933946	.	65.763740	[illegible]
84505	NM_001163641,NM_001163642,NM_018877	Setdb1	SET domain, bifurcated 1	protein_coding	NP_001157113.1;NP_001157114.1;NP_061365.3	1934229	.	13.783877	[illegible]
84544	NM_032465,XM_017317168	Cd96	CD96 antigen	protein_coding	NP_115854.2;XP_017172657.1	1934368	.	0.177547	[illegible]
84585	NM_001311152,NM_032543,XM_006511877,XM_006511878,XM_006511879,XM_006511881,XM_006511883,XM_017313707,XM_030244744,XM_030244745,XR_001779084,XR_379775	Rnf123	ring finger protein 123	protein_coding	NP_001298081.1;NP_115932.1;XP_006511940.1;XP_006511941.1;XP_006511942.1;XP_006511944.1;XP_006511946.1;XP_017169196.1;XP_030100604.1;XP_030100605.1	2148796	.	8.071027	[illegible]
84652	NM_001310471,NM_053090,XM_006535822,XM_011249798,XM_017321188,XM_030254947	Fam126a	family with sequence similarity 126, member A	protein_coding	NP_001297400.1;NP_444320.2;XP_006535885.1;XP_011248100.1;XP_017176677.1;XP_030110807.1	2149839	.	9.718141	[illegible]
84653	NM_033041,XM_006534556	Hes7	hes family bHLH transcription factor 7	protein_coding	NP_149030.2;XP_006534619.1	2135679	.	0.075779	[illegible]
84682	NM_053091,XM_017319347	Cox4i2	cytochrome c oxidase subunit 4I2	protein_coding	NP_444321.1;XP_017174836.1	2135755	.	4.590205	[illegible]
84704	NM_033174	Snurf	SNRPN upstream reading frame	protein_coding	NP_149409.1	1891236	.	1.131407	[illegible]
85029	NR_002142	Rpph1	ribonuclease P RNA component H1	RNase_P_RNA	.	1934664	.	2.187385	[illegible]
85030	NM_001291010,NM_033042,XM_006539318,XM_006539319,XM_011250362,XM_030253942,XR_881438	Tnfrsf25	tumor necrosis factor receptor superfamily, member 25	protein_coding	NP_001277939.1;NP_149031.2;XP_006539381.1;XP_006539382.1;XP_011248664.1;XP_030109802.1	1934667	.	1.777763	[illegible]
85031	NM_134102,XM_006522771,XM_006522772,XM_006522774	Pla1a	phospholipase A1 member A	protein_coding	NP_598863.3;XP_006522834.1;XP_006522835.1;XP_006522837.1	1934677	.	0.349795	[illegible]
85305	NM_001130868,NM_001286384,NM_001363429,NM_001363430,NM_053092	Kars	lysyl-tRNA synthetase	protein_coding	NP_001124340.1;NP_001273313.1;NP_001350358.1;NP_001350359.1;NP_444322.1	1934754	.	45.554058	[illegible]
85308	NM_033146,XM_006519739,XR_874375	Emc9	ER membrane protein complex subunit 9	protein_coding	NP_149158.1;XP_006519802.1	1934682	.	2.594212	[illegible]
89867	NM_001159986,NM_033354,NR_027641,XM_006497049,XM_017312983,XM_017312984,XM_017312986,XM_017312987	Sec16b	SEC16 homolog B (S. cerevisiae)	protein_coding	NP_001153458.1;NP_203505.3;XP_006497112.1;XP_017168472.1;XP_017168473.1;XP_017168475.1;XP_017168476.1	2148802	.	19.896363	[illegible]
93670	NM_053093	Tac4	tachykinin 4	protein_coding	NP_444323.1	1931130	.	0.037872	[illegible]
93671	NM_001170395,NM_053094,XM_006506800,XM_017321840	Cd163	CD163 antigen	protein_coding	NP_001163866.1;NP_444324.2;XP_006506863.1;XP_017177329.1	2135946	.	2.414818	[illegible]
93673	NM_053096	Nat8f2	N-acetyltransferase 8 (GCN5-related) family member 2	protein_coding	NP_444326.2	2136446	.	0.000000	[illegible]
93674	NM_001037842,XM_017321841,XM_030255683	Nat8f3	N-acetyltransferase 8 (GCN5-related) family member 3	protein_coding	NP_001032931.1;XP_017177330.1;XP_030111543.1	2136449	.	0.000000	[illegible]
93675	NM_001289706,NM_001289707,NM_001289708,NM_020257,XM_011241505	Clec2i	C-type lectin domain family 2, member i	protein_coding	NP_001276635.1;NP_001276636.1;NP_001276637.1;NP_064653.1;XP_011239807.1	2136650	.	0.000000	[illegible]
93677	NM_053098	Lmod2	leiomodin 2 (cardiac)	protein_coding	NP_444328.1	2135672	.	3.469378	[illegible]
93679	NM_053100,XM_006527488	Trim8	tripartite motif-containing 8	protein_coding	NP_444330.2;XP_006527551.1	1933302	.	12.657267	[illegible]
93681	NM_001251833,NM_001251834,NM_139141,XM_017315646,XM_030247472,XM_030247473,XM_030247474,XM_030247475,XM_030247476,XM_030247477	Zkscan8	zinc finger with KRAB and SCAN domains 8	protein_coding	NP_001238762.1;NP_001238763.1;NP_631880.2;XP_017171135.1;XP_030103332.1;XP_030103333.1;XP_030103334.1;XP_030103335.1;XP_030103336.1;XP_030103337.1	1913815	.	4.196717	[illegible]
93683	NM_001368304,NM_033320,NR_160774	Glce	glucuronyl C5-epimerase	protein_coding	NP_001355233.1;NP_201577.3	2136405	.	10.728433	[illegible]
93684	NM_053102	Selenof	selenoprotein F	protein_coding	NP_444332.1	1927947	.	178.430511	[illegible]
93685	NM_053103	Entpd7	ectonucleoside triphosphate diphosphohydrolase 7	protein_coding	NP_444333.3	2135885	.	3.159684	[illegible]
93686	NM_001110827,NM_001110828,NM_001110829,NM_001110830,NM_001286417,NM_001286418,NM_001286419,NM_001358770,NM_001358771,NM_001358772,NM_053104,NM_175387,XM_006521568,XM_006521569,XM_006521570,XM_006521574,XM_006521576,XM_006521577,XM_006521580,XM_006521587,XM_006521592,XM_006521593,XM_017316809,XM_030248829,XM_030248830,XM_030248831,XM_030248832,XM_030248833,XM_030248834,XM_030248835,XM_030248836,XM_030248837,XM_030248838,XM_030248839,XM_030248840,XM_030248841,XM_030248842,XM_030248843,XM_030248844,XM_030248845,XM_030248846,XM_030248847,XM_030248848,XM_030248849,XM_030248850,XM_030248851,XM_030248852,XR_001781584,XR_003951446,XR_003951447,XR_003951448,XR_003951449,XR_003951450,XR_384057	Rbfox2	RNA binding protein, fox-1 homolog (C. elegans) 2	protein_coding	NP_001104297.1;NP_001104298.1;NP_001104299.1;NP_001104300.1;NP_001273346.1;NP_001273347.1;NP_001273348.1;NP_001345699.1;NP_001345700.1;NP_001345701.1;NP_444334.3;NP_780596.1;XP_006521631.1;XP_006521632.1;XP_006521633.1;XP_006521637.1;XP_006521639.1;XP_006521640.1;XP_006521643.1;XP_006521650.1;XP_006521655.1;XP_006521656.1;XP_017172298.1;XP_030104689.1;XP_030104690.1;XP_030104691.1;XP_030104692.1;XP_030104693.1;XP_030104694.1;XP_030104695.1;XP_030104696.1;XP_030104697.1;XP_030104698.1;XP_030104699.1;XP_030104700.1;XP_030104701.1;XP_030104702.1;XP_030104703.1;XP_030104704.1;XP_030104705.1;XP_030104706.1;XP_030104707.1;XP_030104708.1;XP_030104709.1;XP_030104710.1;XP_030104711.1;XP_030104712.1	1933973	.	34.501998	[illegible]
93687	NM_001357498,NM_001357499,NM_001357500,NM_146087,XM_006526383,XM_006526386,XM_011247048,XM_030250645,XM_030250646,XR_386022,XR_386023	Csnk1a1	casein kinase 1, alpha 1	protein_coding	NP_001344427.1;NP_001344428.1;NP_001344429.1;NP_666199.1;XP_006526446.1;XP_006526449.1;XP_011245350.1;XP_030106505.1;XP_030106506.1	1934950	.	49.657029	[illegible]
93688	NM_053105	Klhl1	kelch-like 1	protein_coding	NP_444335.2	2136335	.	0.000000	[illegible]
93689	NM_053106	Lmod1	leiomodin 1 (smooth muscle)	protein_coding	NP_444336.2	2135671	.	0.410480	[illegible]
93690	NM_053107,XM_006496346,XM_006496347,XM_006496348,XM_017312993	Gpr45	G protein-coupled receptor 45	protein_coding	NP_444337.3;XP_006496409.1;XP_006496410.1;XP_006496411.1;XP_017168482.1	2135882	.	0.000000	[illegible]
93691	NM_033563,XM_006496351,XM_006496352,XM_006496353,XM_006496354,XM_030243769,XM_030243770	Klf7	Kruppel-like factor 7 (ubiquitous)	protein_coding	NP_291041.2;XP_006496414.1;XP_006496415.1;XP_006496416.1;XP_006496417.1;XP_030099629.1;XP_030099630.1	1935151	.	5.247998	[illegible]
93692	NM_001360151,NM_053108	Glrx	glutaredoxin	protein_coding	NP_001347080.1;NP_444338.2	2135625	.	7.988534	[illegible]
93694	NM_053109,XM_006506805	Clec2d	C-type lectin domain family 2, member d	protein_coding	NP_444339.1;XP_006506868.1	2135589	.	10.032996	[illegible]
93695	NM_053110	Gpnmb	glycoprotein (transmembrane) nmb	protein_coding	NP_444340.3	1934765	.	1.442335	[illegible]
93696	NM_053068	Chrac1	chromatin accessibility complex 1	protein_coding	NP_444298.1	2135796	.	21.557293	[illegible]
93697	NM_145618,XM_006511573,XM_006511574,XM_006511575,XM_017313708,XR_003948072,XR_003948073,XR_379471	Ice2	interactor of little elongation complex ELL subunit 2	protein_coding	NP_663593.2;XP_006511636.1;XP_006511637.1;XP_006511638.1;XP_017169197.1	2135947	.	5.247307	[illegible]
93699	NM_033574	Pcdhgb1	protocadherin gamma subfamily B, 1	protein_coding	NP_291052.3	1935169	.	0.913975	[illegible]
93700	NM_033575	Pcdhgb2	protocadherin gamma subfamily B, 2	protein_coding	NP_291053.1	1935170	.	0.978180	[illegible]
93701	NM_033576	Pcdhgb4	protocadherin gamma subfamily B, 4	protein_coding	NP_291054.1	1935173	.	0.829432	[illegible]
93702	NM_033577	Pcdhgb5	protocadherin gamma subfamily B, 5	protein_coding	NP_291055.1	1935196	.	0.784131	[illegible]
93703	NM_033578	Pcdhgb6	protocadherin gamma subfamily B, 6	protein_coding	NP_291056.1	1935197	.	3.634942	[illegible]
93704	NM_033579	Pcdhgb7	protocadherin gamma subfamily B, 7	protein_coding	NP_291057.1	1935199	.	1.676521	[illegible]
93705	NR_160734	Pcdhgb8	protocadherin gamma subfamily B, 8	transcribed_pseudogene	.	1935200	.	0.411100	[illegible]
93706	NM_033581	Pcdhgc3	protocadherin gamma subfamily C, 3	protein_coding	NP_291059.1	1935201	.	29.452991	[illegible]
93707	NM_033582	Pcdhgc4	protocadherin gamma subfamily C, 4	protein_coding	NP_291060.1	1935203	.	0.938320	[illegible]
93708	NM_033583	Pcdhgc5	protocadherin gamma subfamily C, 5	protein_coding	NP_291061.1	1935205	.	1.703142	[illegible]
93709	NM_033584	Pcdhga1	protocadherin gamma subfamily A, 1	protein_coding	NP_291062.1	1935212	.	0.854146	[illegible]
93710	NM_033585	Pcdhga2	protocadherin gamma subfamily A, 2	protein_coding	NP_291063.1	1935214	.	1.612978	[illegible]
93711	NM_033586	Pcdhga3	protocadherin gamma subfamily A, 3	protein_coding	NP_291064.1	1935215	.	2.399073	[illegible]
93712	NM_033587	Pcdhga4	protocadherin gamma subfamily A, 4	protein_coding	NP_291065.3	1935216	.	0.825773	[illegible]
93713	NM_033588	Pcdhga5	protocadherin gamma subfamily A, 5	protein_coding	NP_291066.1	1935217	.	2.211591	[illegible]
93714	NM_033589	Pcdhga6	protocadherin gamma subfamily A, 6	protein_coding	NP_291067.1	1935218	.	1.838363	[illegible]
93715	NM_033590	Pcdhga7	protocadherin gamma subfamily A, 7	protein_coding	NP_291068.2	1935219	.	3.105833	[illegible]
93716	NM_033591	Pcdhga8	protocadherin gamma subfamily A, 8	protein_coding	NP_291069.1	1935221	.	1.385548	[illegible]
93717	NM_033592	Pcdhga9	protocadherin gamma subfamily A, 9	protein_coding	NP_291070.1	1935226	.	0.574760	[illegible]
93719	NM_053111	Ear6	eosinophil-associated, ribonuclease A family, member 6	protein_coding	NP_444341.1	1890463	.	0.000000	[illegible]
93721	NM_030703,XM_011247407	Cpn1	carboxypeptidase N, polypeptide 1	protein_coding	NP_109628.1;XP_011245709.1	2135874	.	0.000000	[illegible]
93722	NM_033593	Pcdhga10	protocadherin gamma subfamily A, 10	protein_coding	NP_291071.1	1935227	.	2.567996	[illegible]
93723	NM_033594	Pcdhga11	protocadherin gamma subfamily A, 11	protein_coding	NP_291072.1	1935228	.	1.590634	[illegible]
93724	NM_033595	Pcdhga12	protocadherin gamma subfamily A, 12	protein_coding	NP_291073.1	1935229	.	1.415572	[illegible]
93725	NM_053112	Ear10	eosinophil-associated, ribonuclease A family, member 10	protein_coding	NP_444342.1	1890464	.	0.000000	[illegible]
93726	NM_053113	Rnase2a	ribonuclease, RNase A family, 2A (liver, eosinophil-derived neurotoxin)	protein_coding	NP_444343.2	1890465	.	0.000000	[illegible]
93728	NM_053114	Pabpc5	poly(A) binding protein, cytoplasmic 5	protein_coding	NP_444344.1	2136401	.	0.179713	[illegible]
93730	NM_033322,NR_151689,XM_006512403	Lztfl1	leucine zipper transcription factor-like 1	protein_coding	NP_201579.1;XP_006512466.1	1934860	.	7.088848	[illegible]
93732	NM_001161667,NM_053115,XM_006518108	Acox2	acyl-Coenzyme A oxidase 2, branched chain	protein_coding	NP_001155139.1;NP_444345.2;XP_006518171.1	1934852	.	0.000000	[illegible]
93734	NM_001289561,NM_001289562,NM_001289563,NM_001289565,NM_001289567,NM_033564,XM_006522776,XM_030249342	Mpv17l	Mpv17 transgene, kidney disease mutant-like	protein_coding	NP_001276490.1;NP_001276491.1;NP_001276492.1;NP_001276494.1;NP_001276496.1;NP_291042.2;XP_006522839.2;XP_030105202.1	2135951	.	2.485426	[illegible]
93735	NM_053116,XM_006505214	Wnt16	wingless-type MMTV integration site family, member 16	protein_coding	NP_444346.3;XP_006505277.1	2136018	.	0.297038	[illegible]
93736	NM_001363331,NM_033565,XM_011249331,XM_030246443	Aff4	AF4/FMR2 family, member 4	protein_coding	NP_001350260.1;NP_291043.1;XP_011247633.1;XP_030102303.1	2136171	.	20.432125	[illegible]
93737	NM_053117,XR_386378	Pard6g	par-6 family cell polarity regulator gamma	protein_coding	NP_444347.3	2135606	.	32.852356	[illegible]
93739	NM_026693	Gabarapl2	gamma-aminobutyric acid (GABA) A receptor-associated protein-like 2	protein_coding	NP_080969.2	1890602	.	30.382549	[illegible]
93742	NM_001013580,NM_001013581,NM_001122850,NM_001309391,NM_001309392,NM_001363431,NM_001363432,NM_033620,XM_006531520,XM_006531523,XM_006531524,XM_006531526,XM_006531527,XM_006531529,XM_006531530,XM_006531531,XM_006531532,XM_006531533,XM_030243867,XM_030243868,XM_030243869,XM_030243870,XM_030243871,XM_030243872	Pard3	par-3 family cell polarity regulator	protein_coding	NP_001013598.1;NP_001013599.1;NP_001116322.1;NP_001296320.1;NP_001296321.1;NP_001350360.1;NP_001350361.1;NP_296369.2;XP_006531583.1;XP_006531586.1;XP_006531587.1;XP_006531589.1;XP_006531590.1;XP_006531592.1;XP_006531593.1;XP_006531594.1;XP_006531595.1;XP_006531596.1;XP_030099727.1;XP_030099728.1;XP_030099729.1;XP_030099730.1;XP_030099731.1;XP_0300	2135608	.	6.944104	[illegible]
93743	gene-Ear-ps2	Ear-ps2	eosinophil-associated, ribonuclease A family, pseudogene 2	pseudogene	.	1890466	.	0.000000	[illegible]
93746	NM_001205396,NM_053118	Gprc5d	G protein-coupled receptor, family C, group 5, member D	protein_coding	NP_001192325.1;NP_444348.1	1935037	.	0.000000	[illegible]
93747	NM_053119	Echs1	enoyl Coenzyme A hydratase, short chain, 1, mitochondrial	protein_coding	NP_444349.1	2136460	.	24.148024	[illegible]
93757	NM_053122,XM_017315261,XM_017315262,XM_030247004	Immp2l	IMP2 inner mitochondrial membrane peptidase-like (S. cerevisiae)	protein_coding	NP_444352.2;XP_017170750.1;XP_017170751.1;XP_030102864.1	2135611	.	0.991874	[illegible]
93759	NM_001159589,NM_019812	Sirt1	sirtuin 1	protein_coding	NP_001153061.1;NP_062786.1	2135607	.	6.839408	[illegible]
93760	NM_001080819,NM_001363070,XM_006539325,XM_006539326,XM_006539327,XM_030253943	Arid1a	AT rich interactive domain 1A (SWI-like)	protein_coding	NP_001074288.1;NP_001349999.1;XP_006539388.1;XP_006539389.1;XP_006539390.1;XP_030109803.1	1935147	.	13.245343	[illegible]
93761	NM_001290708,NM_001358618,NM_001358619,NM_053123,XM_017318646	Smarca1	SWI/SNF related, matrix associated, actin dependent regulator of chromatin, subfamily a, member 1	protein_coding	NP_001277637.1;NP_001345547.1;NP_001345548.1;NP_444353.3;XP_017174135.1	1935127	.	11.335901	[illegible]
93762	NM_053124	Smarca5	SWI/SNF related, matrix associated, actin dependent regulator of chromatin, subfamily a, member 5	protein_coding	NP_444354.2	1935129	.	19.307110	[illegible]
93765	NM_080560,XM_006514344	Ube2n	ubiquitin-conjugating enzyme E2N	protein_coding	NP_542127.1;XP_006514407.1	1934835	.	17.951664	[illegible]
93790	NM_001256130,NM_001256131,NM_001256132,NM_001256133,NM_023647,XM_006541324	Nipa2	non imprinted in Prader-Willi/Angelman syndrome 2 homolog (human)	protein_coding	NP_001243059.1;NP_001243060.1;NP_001243061.1;NP_001243062.1;NP_076136.2;XP_006541387.1	1913918	.	7.395965	[illegible]
93834	NM_033602,XM_006519740,XM_006519741,XM_006519742,XM_011245231,XM_030248103	Peli2	pellino 2	protein_coding	NP_291080.2;XP_006519803.1;XP_006519804.1;XP_006519805.1;XP_011243533.1;XP_030103963.1	1891445	.	2.804161	[illegible]
93835	NM_033603,XM_030247005	Amn	amnionless	protein_coding	NP_291081.2;XP_030102865.1	1934943	.	0.065672	[illegible]
93836	NM_001357494,NM_033604,XM_006511576,XM_006511577,XM_006511578,XM_006511580,XM_006511581,XM_006511582,XM_006511583,XM_006511584,XM_006511587,XM_011242841,XM_017313709,XM_030244746,XM_030244747,XM_030244748,XM_030244749,XM_030244750,XM_030244751,XM_030244752	Rnf111	ring finger 111	protein_coding	NP_001344423.1;NP_291082.1;XP_006511639.1;XP_006511640.1;XP_006511641.1;XP_006511643.1;XP_006511644.1;XP_006511645.1;XP_006511646.1;XP_006511647.1;XP_006511650.1;XP_011241143.1;XP_017169198.1;XP_030100606.1;XP_030100607.1;XP_030100608.1;XP_030100609.1;XP_030100610.1;XP_030100611.1;XP_030100	1934919	.	8.152209	[illegible]
93837	NM_001142570,NM_001289732,NM_001289733,NM_001289734,NM_033605,XM_006528416,XM_011247711,XM_011247712,XM_011247713,XM_011247714,XM_011247715,XM_011247716,XM_011247717,XM_030251523,XM_030251524,XM_030251525,XM_030251526,XM_030251527,XM_030251528,XM_030251529	Dach2	dachshund family transcription factor 2	protein_coding	NP_001136042.1;NP_001276661.1;NP_001276662.1;NP_001276663.1;NP_291083.1;XP_006528479.1;XP_011246013.1;XP_011246014.1;XP_011246015.1;XP_011246016.1;XP_011246017.1;XP_011246018.1;XP_011246019.1;XP_030107383.1;XP_030107384.1;XP_030107385.1;XP_030107386.1;XP_030107387.1;XP_030107388.1;XP_030107	1890446	.	0.100590	[illegible]
93838	NM_033606,XM_006506807,XM_006506808,XM_006506809,XM_006506810,XM_006506811,XM_006506812	Dqx1	DEAQ RNA-dependent ATPase	protein_coding	NP_291084.1;XP_006506870.1;XP_006506871.1;XP_006506872.1;XP_006506873.1;XP_006506874.1;XP_006506875.1	2136388	.	0.531324	[illegible]
93840	NM_033509,XM_006497051,XM_006497052,XM_006497053,XM_006497054,XM_017313008,XR_003947338	Vangl2	VANGL planar cell polarity 2	protein_coding	NP_277044.1;XP_006497114.1;XP_006497115.1;XP_006497116.1;XP_006497117.1;XP_017168497.1	2135272	.	3.472265	[illegible]
93841	NM_033607	Uchl4	ubiquitin carboxyl-terminal esterase L4	protein_coding	NP_291085.1	1890440	.	0.000000	[illegible]
93842	NM_001145800,NM_033608,XM_006497056,XM_006497057,XM_006497058,XM_030243785,XM_030243786,XR_001778486	Igsf9	immunoglobulin superfamily, member 9	protein_coding	NP_001139272.1;NP_291086.2;XP_006497119.1;XP_006497120.1;XP_006497121.1;XP_030099645.1;XP_030099646.1	2135283	.	0.379362	[illegible]
93843	NM_001199351,NM_001199352,NM_012040,XM_006528344,XM_011247692,XM_030251530,XM_030251531,XR_001782761,XR_003953032,XR_003953033,XR_003953034	Pnck	pregnancy upregulated non-ubiquitously expressed CaM kinase	protein_coding	NP_001186280.1;NP_001186281.1;NP_036170.1;XP_006528407.1;XP_011245994.1;XP_030107390.1;XP_030107391.1	1347357	.	1.589916	[illegible]
93871	NM_001103179,NM_145125,NM_176928,XM_006523113,XM_006523114,XM_006523115,XM_011246156,XM_011246157,XM_017317169,XM_017317170,XM_030249344	Brwd1	bromodomain and WD repeat domain containing 1	protein_coding	NP_001096649.1;NP_660107.2;NP_795902.1;XP_006523176.1;XP_006523177.1;XP_006523178.1;XP_011244458.1;XP_011244459.1;XP_017172658.1;XP_017172659.1;XP_030105204.1	1890651	.	15.960982	[illegible]
93873	NM_053127	Pcdhb2	protocadherin beta 2	protein_coding	NP_444357.1	2136735	.	0.283734	[illegible]
93874	NM_053128	Pcdhb3	protocadherin beta 3	protein_coding	NP_444358.2	2136737	.	0.515536	[illegible]
93875	NM_053129	Pcdhb4	protocadherin beta 4	protein_coding	NP_444359.3	2136738	.	0.514305	[illegible]
93876	NM_053130	Pcdhb5	protocadherin beta 5	protein_coding	NP_444360.1	2136739	.	1.039478	[illegible]
93877	NM_053131	Pcdhb6	protocadherin beta 6	protein_coding	NP_444361.1	2136740	.	0.485830	[illegible]
93878	NM_053132	Pcdhb7	protocadherin beta 7	protein_coding	NP_444362.3	2136741	.	1.094144	[illegible]
93879	NM_053133	Pcdhb8	protocadherin beta 8	protein_coding	NP_444363.1	2136742	.	0.505470	[illegible]
93880	NM_053134	Pcdhb9	protocadherin beta 9	protein_coding	NP_444364.3	2136744	.	0.764088	[illegible]
93881	NM_053135	Pcdhb10	protocadherin beta 10	protein_coding	NP_444365.1	2136745	.	0.379306	[illegible]
93882	NM_053136	Pcdhb11	protocadherin beta 11	protein_coding	NP_444366.3	2136746	.	0.350225	[illegible]
93883	NM_053137	Pcdhb12	protocadherin beta 12	protein_coding	NP_444367.1	2136747	.	0.448600	[illegible]
93884	NM_053138	Pcdhb13	protocadherin beta 13	protein_coding	NP_444368.1	2136748	.	0.160681	[illegible]
93885	NM_053139	Pcdhb14	protocadherin beta 14	protein_coding	NP_444369.3	2136749	.	0.827245	[illegible]
93886	NM_053140	Pcdhb15	protocadherin beta 15	protein_coding	NP_444370.1	2136750	.	0.526316	[illegible]
93887	NM_053141	Pcdhb16	protocadherin beta 16	protein_coding	NP_444371.3	2136752	.	0.706477	[illegible]
93888	NM_053142	Pcdhb17	protocadherin beta 17	protein_coding	NP_444372.2	2136754	.	1.788552	[illegible]
93889	NM_053143	Pcdhb18	protocadherin beta 18	protein_coding	NP_444373.1	2136756	.	0.543187	[illegible]
93890	NM_053144	Pcdhb19	protocadherin beta 19	protein_coding	NP_444374.2	2136757	.	0.787466	[illegible]
93891	NM_053145	Pcdhb20	protocadherin beta 20	protein_coding	NP_444375.2	2136758	.	1.495842	[illegible]
93892	NM_053146	Pcdhb21	protocadherin beta 21	protein_coding	NP_444376.1	2136759	.	0.436050	[illegible]
93893	NM_053147	Pcdhb22	protocadherin beta 22	protein_coding	NP_444377.3	2136760	.	2.448822	[illegible]
93895	NR_027920	Msx1os	msh homeobox 1 opposite strand	antisense_RNA	.	2136762	.	0.141538	[illegible]
93896	NM_175681,XM_006534561,XM_006534562	Glp2r	glucagon-like peptide 2 receptor	protein_coding	NP_783612.2;XP_006534624.1;XP_006534625.1	2136733	.	0.000000	[illegible]
93897	NM_175284	Fzd10	frizzled class receptor 10	protein_coding	NP_780493.1	2136761	.	0.399932	[illegible]
93898	NM_138647	Cers1	ceramide synthase 1	protein_coding	NP_619588.1	2136690	.	0.476197	[illegible]
93960	NM_001163660,NM_027280,XM_030243873	Nkd1	naked cuticle 1	protein_coding	NP_001157132.1;NP_081556.3;XP_030099733.1	2135954	.	4.771199	[illegible]
93961	NM_001122993,NM_001358389,NM_001358390,NM_033149,XM_006523117	B3galt5	UDP-Gal:betaGlcNAc beta 1,3-galactosyltransferase, polypeptide 5	protein_coding	NP_001116465.1;NP_001345318.1;NP_001345319.1;NP_149161.1;XP_006523180.1	2136878	.	0.039478	[illegible]
93966	NM_053149	Hemgn	hemogen	protein_coding	NP_444379.1	2136910	.	0.245103	[illegible]
94040	NM_001040682,NM_053155,NR_104435,NR_104436,NR_104437,NR_104438,NR_104439,XR_001780506	Clmn	calmin	protein_coding	NP_001035772.1;NP_444385.2	2136957	.	1.355023	[illegible]
94043	NM_053157	Tm2d1	TM2 domain containing 1	protein_coding	NP_444387.1	2137022	.	16.392801	[illegible]
94044	NM_153516,XM_006506814,XM_017321842	Bcl2l13	BCL2-like 13 (apoptosis facilitator)	protein_coding	NP_705736.1;XP_006506877.1;XP_017177331.1	2136959	.	6.572877	[illegible]
94045	NM_033321,XM_006534563,XM_006534564,XM_006534565,XM_006534566	P2rx5	purinergic receptor P2X, ligand-gated ion channel, 5	protein_coding	NP_201578.2;XP_006534626.1;XP_006534627.1;XP_006534628.1;XP_006534629.1	2137026	.	1.458581	[illegible]
94047	NM_033567	Tmem121b	transmembrane protein 121B	protein_coding	NP_291045.1	2136977	.	0.090436	[illegible]
94054	gene-Mdm4-ps	Mdm4-ps	transformed mouse 3T3 cell double minute 4, pseudogene	pseudogene	.	2136991	.	0.000000	[illegible]
94061	NM_001039084,NM_053158,XM_006535292,XM_011249596	Mrpl1	mitochondrial ribosomal protein L1	protein_coding	NP_001034173.1;NP_444388.2;XP_006535355.1;XP_011247898.1	2137202	.	3.334666	[illegible]
94062	NM_001364512,NM_001364513,NM_053159	Mrpl3	mitochondrial ribosomal protein L3	protein_coding	NP_001351441.1;NP_001351442.1;NP_444389.2	2137204	.	16.472206	[illegible]
94063	NM_025606	Mrpl16	mitochondrial ribosomal protein L16	protein_coding	NP_079882.2	2137219	.	11.944014	[illegible]
94064	NM_053161,XM_006534567	Mrpl27	mitochondrial ribosomal protein L27	protein_coding	NP_444391.1;XP_006534630.1	2137224	.	20.507212	[illegible]
94065	NM_053162	Mrpl34	mitochondrial ribosomal protein L34	protein_coding	NP_444392.1	2137227	.	16.998566	[illegible]
94066	NM_053163,XM_030247478	Mrpl36	mitochondrial ribosomal protein L36	protein_coding	NP_444393.1;XP_030103338.1	2137228	.	17.225980	[illegible]
94067	NM_053164	Mrpl43	mitochondrial ribosomal protein L43	protein_coding	NP_444394.1	2137229	.	15.372952	[illegible]
94088	NM_001013616,XM_006508364,XM_006508365,XM_006508366,XM_017312407	Trim6	tripartite motif-containing 6	protein_coding	NP_001013637.1;XP_006508427.1;XP_006508428.1;XP_006508429.1;XP_017167896.1	2137352	.	0.519845	[illegible]
94089	NM_001347446,NM_053166,XM_006534568,XM_006534569,XM_006534571,XM_006534573,XM_006534574,XM_006534575,XM_006534576,XM_006534577,XM_011249333,XM_017314860,XM_030246445	Trim7	tripartite motif-containing 7	protein_coding	NP_001334375.1;NP_444396.2;XP_006534631.1;XP_006534632.1;XP_006534634.1;XP_006534636.1;XP_006534637.1;XP_006534638.1;XP_006534639.1;XP_006534640.1;XP_011247635.1;XP_017170349.1;XP_030102305.1	2137353	.	0.999216	[illegible]
94090	NM_001110202,NM_001110203,NM_001286386,NM_001286387,NM_001286388,NM_053167,XM_006516400,XM_006516401,XM_006516402,XM_006516403,XM_006516404,XM_006516405,XM_006516406,XM_006516407,XM_017315263,XM_017315265,XM_030247006,XM_030247007,XM_030247008,XM_030247009,XR_381560	Trim9	tripartite motif-containing 9	protein_coding	NP_001103672.1;NP_001103673.1;NP_001273315.1;NP_001273316.1;NP_001273317.1;NP_444397.2;XP_006516463.1;XP_006516464.1;XP_006516465.1;XP_006516466.1;XP_006516467.1;XP_006516468.1;XP_006516469.1;XP_006516470.1;XP_017170752.1;XP_017170754.1;XP_030102866.1;XP_030102867.1;XP_030102868.1;XP_030102	2137354	.	0.144182	[illegible]
94091	NM_001290988,NM_053168,XM_006534579,XM_011249334,XM_011249335,XM_030246446	Trim11	tripartite motif-containing 11	protein_coding	NP_001277917.1;NP_444398.1;XP_006534642.1;XP_011247636.1;XP_011247637.1;XP_030102306.1	2137355	.	9.912180	[illegible]
94092	NM_053169,XM_006534580	Trim16	tripartite motif-containing 16	protein_coding	NP_444399.2;XP_006534643.1	2137356	.	4.865558	[illegible]
94093	NM_001079830,NM_053170,XR_003954429,XR_375607	Trim33	tripartite motif-containing 33	protein_coding	NP_001073299.1;NP_444400.2	2137357	.	6.636032	[illegible]
94094	NM_001357679,NM_030684,XM_006508368,XM_006508369,XR_003946602,XR_003946603	Trim34a	tripartite motif-containing 34A	protein_coding	NP_001344608.1;NP_109609.2;XP_006508431.1;XP_006508432.1	2137359	.	1.698680	[illegible]
94109	NM_053171,XM_006508930,XM_006508931,XM_006508932,XM_030243874,XM_030243875	Csmd1	CUB and Sushi multiple domains 1	protein_coding	NP_444401.2;XP_006508993.1;XP_006508994.1;XP_006508995.1;XP_030099734.1;XP_030099735.1	2137383	.	0.008378	[illegible]
94111	NM_053172	Mepe	matrix extracellular phosphoglycoprotein with ASARM motif (bone)	protein_coding	NP_444402.2	2137384	.	30.451700	[illegible]
94112	NM_001040683,NM_001285884,NM_001285886,NM_001358404,NM_033609,XM_006522779,XM_006522783,XM_017317172,XM_017317173,XM_017317174,XM_017317175,XM_030249346,XM_030249348,XM_030249349,XM_030249350	Med15	mediator complex subunit 15	protein_coding	NP_001035773.1;NP_001272813.1;NP_001272815.1;NP_001345333.1;NP_291087.2;XP_006522842.1;XP_006522846.1;XP_017172661.1;XP_017172662.1;XP_017172663.1;XP_017172664.1;XP_030105206.1;XP_030105208.1;XP_030105209.1;XP_030105210.1	2137379	.	17.232978	[illegible]
94176	NM_033374,XM_017314861,XM_017314862	Dock2	dedicator of cyto-kinesis 2	protein_coding	NP_203538.2;XP_017170350.1;XP_017170351.1	2149010	.	0.961364	[illegible]
94178	NM_053177	Mcoln1	mucolipin 1	protein_coding	NP_444407.1	1890498	.	12.095772	[illegible]
94179	NM_033373	Krt23	keratin 23	protein_coding	NP_203537.1	2148866	.	0.000000	[illegible]
94180	NM_053178,XM_006511588,XM_030244753	Acsbg1	acyl-CoA synthetase bubblegum family member 1	protein_coding	NP_444408.1;XP_006511651.1;XP_030100613.1	2385656	.	0.111989	[illegible]
94181	NM_053179	Nans	N-acetylneuraminic acid synthase (sialic acid synthase)	protein_coding	NP_444409.1	2149820	.	29.159964	[illegible]
94184	NM_001039533,NM_001291017,NM_001357225,NM_053181,XM_011246053,XM_011246054,XM_011246055	Pdxdc1	pyridoxal-dependent decarboxylase domain containing 1	protein_coding	NP_001034622.1;NP_001277946.1;NP_001344154.1;NP_444411.2;XP_011244355.1;XP_011244356.1;XP_011244357.1	1920909	.	38.258118	[illegible]
94185	NM_178589	Tnfrsf21	tumor necrosis factor receptor superfamily, member 21	protein_coding	NP_848704.1	2151075	.	2.924415	[illegible]
94186	NM_001172098,NM_001364437,NM_052973,XM_006516411,XM_006516412	Strn3	striatin, calmodulin binding protein 3	protein_coding	NP_001165569.1;NP_001351366.1;NP_443205.1;XP_006516474.1;XP_006516475.1	2151064	.	24.926409	[illegible]
94187	NM_001310520,NM_033327,XM_006531539,XM_011248560,XM_011248561,XM_017313033,XM_030243876,XM_030243877,XM_030243878,XM_030243879	Zfp423	zinc finger protein 423	protein_coding	NP_001297449.1;NP_201584.2;XP_006531602.1;XP_011246862.1;XP_011246863.1;XP_017168522.1;XP_030099736.1;XP_030099737.1;XP_030099738.1;XP_030099739.1	1891217	.	3.053528	[illegible]
94190	NM_001313754,NM_001313755,NM_001313756,NM_052976,XM_011247693,XM_011247694,XM_017318648	Ophn1	oligophrenin 1	protein_coding	NP_001300683.1;NP_001300684.1;NP_001300685.1;NP_443208.1;XP_011245995.1;XP_011245996.1;XP_017174137.1	2151070	.	3.379892	[illegible]
94191	NM_001289530,NM_052977,NR_110345,XM_017315647,XR_3820	Adarb2	adenosine deaminase, RNA-specific, B2	protein_coding	NP_001276459.1;NP_443209.2;XP_017171136.1	2151118	.	0.097838	[illegible]
94192	NM_052993	C1galt1	core 1 synthase, glycoprotein-N-acetylgalactosamine 3-beta-galactosyltransferase, 1	protein_coding	NP_443719.3	2151071	.	12.237811	[illegible]
94212	NM_001195031,NM_053182,XM_006530107,XM_011248151,XM_030252926,XM_030252927,XM_030252928	Pag1	phosphoprotein associated with glycosphingolipid microdomains 1	protein_coding	NP_001181960.1;NP_444412.2;XP_006530170.1;XP_011246453.1;XP_030108786.1;XP_030108787.1;XP_030108788.1	2443160	.	1.476723	[illegible]
94213	NM_001358819,NM_001358820,NM_053183,XM_006514345,XM_011243608,XM_030245407,XM_030245408,XM_030245409,XM_030245410	Ddx50	DEAD (Asp-Glu-Ala-Asp) box polypeptide 50	protein_coding	NP_001345748.1;NP_001345749.1;NP_444413.1;XP_006514408.1;XP_011241910.1;XP_030101267.1;XP_030101268.1;XP_030101269.1;XP_030101270.1	2182303	.	55.520867	[illegible]
94214	NM_052994,XM_006514347,XM_017314167	Spock2	sparc/osteonectin, cwcv and kazal-like domains proteoglycan 2	protein_coding	NP_443720.1;XP_006514410.1;XP_017169656.1	1891351	.	12.717533	[illegible]
94216	NM_053185,XM_011247883,XR_003953035	Col4a6	collagen, type IV, alpha 6	protein_coding	NP_444415.2;XP_011246185.1	2152695	.	0.088201	[illegible]
94217	NM_053011,XM_006498459,XM_006498460,XM_006498461,XM_006498462,XM_006498463,XM_011239217,XM_017319348,XR_374143,XR_374144	Lrp1b	low density lipoprotein-related protein 1B	protein_coding	NP_443737.2;XP_006498522.1;XP_006498523.1;XP_006498524.1;XP_006498525.1;XP_006498526.1;XP_011237519.1;XP_017174837.1	2151136	.	0.052288	[illegible]
94218	NM_001039551,NM_001331209,NM_053186	Cnnm3	cyclin M3	protein_coding	NP_001034640.1;NP_001318138.1;NP_444416.2	2151055	.	4.524233	[illegible]
94219	NM_001102471,NM_033569,XM_006527489,XM_006527490	Cnnm2	cyclin M2	protein_coding	NP_001095941.1;NP_291047.2;XP_006527552.1;XP_006527553.1	2151054	.	2.177767	[illegible]
94220	NM_033570	Cnnm4	cyclin M4	protein_coding	NP_291048.2	2151060	.	3.383931	[illegible]
94221	NM_001199272,NM_053187	Gopc	golgi associated PDZ and coiled-coil motif containing	protein_coding	NP_001186201.1;NP_444417.2	2149946	.	9.466475	[illegible]
94223	NM_033324,XM_006522795,XM_030249352,XM_030249353,XM_030249354,XM_030249355	Dgcr8	DGCR8, microprocessor complex subunit	protein_coding	NP_201581.2;XP_006522858.1;XP_030105212.1;XP_030105213.1;XP_030105214.1;XP_030105215.1	2151114	.	10.128101	[illegible]
94224	NM_053188	Srd5a2	steroid 5 alpha-reductase 2	protein_coding	NP_444418.1	2150380	.	0.000000	[illegible]
94226	NM_053190	S1pr5	sphingosine-1-phosphate receptor 5	protein_coding	NP_444420.1	2150641	.	0.119387	[illegible]
94227	NM_053191	Pi15	peptidase inhibitor 15	protein_coding	NP_444421.2	1934659	.	1.925779	[illegible]

94229	NM_001242378,NM_001242379,NM_001242380,NM_001242381,NM_001242382,NM_001242383,NM_001362671,NM_001362672,NM_001362673,NM_001362674,NM_001362675,NM_001362676,NM_033552,XM_011239858,XM_017319349,XM_030252258	Slc4a10	solute carrier family 4, sodium bicarbonate cotransporter-like, member 10	protein_coding	NP_001229307.1;NP_001229308.1;NP_001229309.1;NP_001229310.1;NP_001229311.1;NP_001229312.1;NP_001349600.1;NP_001349601.1;NP_001349602.1;NP_001349603.1;NP_001349604.1;NP_001349605.1;NP_291030.2;XP_011238160.1;XP_017174838.1;XP_0301081	2150150	.	4.577526
94230	NM_001164173,NM_001361485,NM_053193,XM_006521602,XM_030248853,XR_003951451	Cpsf1	cleavage and polyadenylation specific factor 1	protein_coding	NP_001157645.1;NP_001348414.1;NP_444423.1;XP_006521665.1;XP_030104713.1	2679722	.	11.806040
94232	NM_033526,XM_006502396,XM_030252929	Ubqln4	ubiquilin 4	protein_coding	NP_277068.1;XP_006502459.1;XP_030108789.1	2150152	.	17.894103
94242	NM_001168333,NM_023476	Tinagl1	tubulointerstitial nephritis antigen-like 1	protein_coding	NP_001161805.1;NP_075965.2	2137617	.	10.868225
94244	NM_001277891,NM_001277892,NM_001277893,NM_033571,XM_011240913,XM_011240915,XM_030254949,XM_030254950,XM_030254951,XM_030254952	Fkbp6	FK506 binding protein 6	protein_coding	NP_001264820.1;NP_001264821.1;NP_001264822.1;NP_291049.1;XP_011239215.1;XP_011239217.1;XP_030110809.1;XP_030110810.1;XP_030110811.1;XP_030110812.1	2137612	.	0.093302
94245	NM_025772,XM_006516984	Dtnbp1	dystrobrevin binding protein 1	protein_coding	NP_080048.2;XP_006517047.1	2137586	.	15.957063
94246	NM_194262,NM_198122,XM_006516806,XM_006516807,XM_006516808,XM_017315648,XM_030247479,XM_030247480,XR_003950495,XR_003950496,XR_003950497	Arid4b	AT rich interactive domain 4B (RBP1-like)	protein_coding	NP_919238.1;NP_937755.1;XP_006516869.1;XP_006516870.1;XP_006516871.1;XP_017171137.1;XP_030103339.1;XP_030103340.1	2137512	.	10.190042
94249	NM_001356497,NM_053195	Slc24a3	solute carrier family 24 (sodium/potassium/calcium exchanger), member	protein_coding	NP_001343426.1;NP_444425.1	2137513	.	3.195395
94253	NM_001081348,XM_006516809,XM_006516810,XM_006516811,XM_006516812,XM_006516813,XM_006516814,XM_006516815,XM_006516816,XM_017315649,XM_017315650,XM_017315651	Hecw1	HECT, C2 and WW domain containing E3 ubiquitin protein ligase 1	protein_coding	NP_001074817.3;XP_006516872.1;XP_006516873.1;XP_006516874.1;XP_006516875.1;XP_006516876.1;XP_006516877.1;XP_006516878.1;XP_006516879.1;XP_017171138.1;XP_017171139.1;XP_017171	2444115	.	0.004617
94254	NM_033572,XM_006504537,XM_006504538,XM_006504539,XM_011240917,XR_377117	Rcc1l	reculator of chromosome condensation 1 like	protein_coding	NP_291050.1;XP_006504600.1;XP_006504601.1;XP_006504602.1;XP_011239219.1	2137600	.	5.670382
94275	NM_019791,NR_157578	Maged1	melanoma antigen, family D, 1	protein_coding	NP_062765.1	1930187	.	266.161026
94279	NM_053196,XR_003952829,XR_386525	Sfxn2	sideroflexin 2	protein_coding	NP_444426.3	2137678	.	12.946582
94280	NM_001178012,NM_001178013,NM_001362379,NM_001362380,NM_001362381,NM_053197,NR_155547,XM_006527493	Sfxn3	sideroflexin 3	protein_coding	NP_001171483.1;NP_001171484.1;NP_001349308.1;NP_001349309.1;NP_001349310.1;NP_444427.1;XP_006527556.1	2137679	.	28.486653
94281	NM_001362384,NM_001362385,NM_053198,XM_030251146,XR_001782624,XR_003952830,XR_003952831	Sfxn4	sideroflexin 4	protein_coding	NP_001349313.1;NP_001349314.1;NP_444428.3;XP_030107006.1	2137680	.	2.872079
94282	NM_178639,XM_006506816,XM_030255684	Sfxn5	sideroflexin 5	protein_coding	NP_848754.1;XP_006506879.1;XP_030111544.1	2137681	.	1.725712
94284	NM_145079	Ugt1a6a	UDP glucuronosyltransferase 1 family, polypeptide A6A	protein_coding	NP_659545.2	2137698	.	0.054243
94315	NM_033573	Prcc	papillary renal cell carcinoma (translocation-associated)	protein_coding	NP_291051.1	2137738	.	15.337838
94332	NM_053199,XM_006497060,XM_030243803	Cadm3	cell adhesion molecule 3	protein_coding	NP_444429.1;XP_006497123.3;XP_030099663.1	2137858	.	1.310329
94346	NM_001168256,NM_001168257,NM_144805,XM_006506819,XM_011241509,XM_011241510,XM_011241511,XM_011241512,XM_011241513,XM_017321846	Tmem40	transmembrane protein 40	protein_coding	NP_001161728.1;NP_001161729.1;NP_659054.2;XP_006506882.1;XP_011239811.1;XP_011239812.1;XP_011239813.1;XP_011239814.1;XP_011239815.1;XP_017177335.1	2137870	.	0.418411
94352	NM_033325,XM_006519744,XM_006519745,XM_006519746,XM_006519747	Loxl2	lysyl oxidase-like 2	protein_coding	NP_201582.2;XP_006519807.1;XP_006519808.1;XP_006519809.1;XP_006519810.1	2137913	.	106.779306
94353	NM_026122,NM_175074,XM_006511590,XM_006511591	Hmgn3	high mobility group nucleosomal binding domain 3	protein_coding	NP_080398.1;NP_778249.1;XP_006511653.1;XP_006511654.1	2138069	.	30.520732
96875	NM_001110146,NM_021400,XM_006529963,XM_006529966,XM_017313032	Prg4	proteoglycan 4 (megakaryocyte stimulating factor, articular superficial zone protein)	protein_coding	NP_001103616.1;NP_067375.3;XP_006530026.1;XP_006530029.1;XP_017168521.1	1891344	.	1.450735
96935	NM_001356461,NM_001356462,NM_001356463,NM_001356464,NM_144796,XM_006497063,XM_030243823,XM_030243824	Susd4	sushi domain containing 4	protein_coding	NP_001343390.1;NP_001343391.1;NP_001343392.1;NP_001343393.1;NP_659045.2;XP_006497126.1;XP_030099683.1;XP_030099684.1	2138351	.	1.053333
96957	NM_175285,XM_030252259,XR_374546	Tmem62	transmembrane protein 62	protein_coding	NP_780494.1;XP_030108119.1	2139461	.	1.795069
96979	NM_133783	Ptges2	prostaglandin E synthase 2	protein_coding	NP_598544.2	1917592	.	5.176469
97031	NM_175286	Tprn	taperin	protein_coding	NP_780495.2	2139535	.	2.354368
97064	NM_001168281,NM_133784	Wwtr1	WW domain containing transcription regulator 1	protein_coding	NP_001161753.1;NP_598545.2	1917649	.	27.509035
97086	NM_178877,XM_006502398,XM_006502399,XM_006502400,XM_006502401,XM_006502402,XM_030252930,XM_030252931,XR_003954430	Slc9b2	solute carrier family 9, subfamily B (NHA2, cation proton antiporter 2), member 2	protein_coding	NP_849208.4;XP_006502461.1;XP_006502462.1;XP_006502463.1;XP_006502464.1;XP_006502465.1;XP_030108790.1;XP_030108791.1	2140077	.	8.957300
97112	NM_133787	Nmd3	NMD3 ribosome export adaptor	protein_coding	NP_598548.1	2140103	.	14.889440
97114	NM_054045	H3c15	histone cluster 2, H3c2	protein_coding	NP_473386.1	2448357	.	0.682158
97122	NM_033596	H4c14	histone cluster 2, H4	protein_coding	NP_291074.1	2140113	.	0.091618
97130	NM_001033189,NM_001285865,NM_001285866,NM_001285867	C77080	expressed sequence C77080	protein_coding	NP_001028361.1;NP_001272794.1;NP_001272795.1;NP_001272796.	2140651	.	2.253684
97159	NM_001163019,NM_175287	A430005L14Rik	RIKEN cDNA A430005L14 gene	protein_coding	NP_001156491.1;NP_780496.2	2140680	.	17.893641
97165	NM_001363443,NM_001363444,NM_001363445,NM_008252	Hmgb2	high mobility group box 2	protein_coding	NP_001350372.1;NP_001350373.1;NP_001350374.1;NP_032278.1	96157	.	23.456737
97209	XR_001784834	A230098N10Rik	RIKEN cDNA A230098N10 gene	lncRNA	.	2141280	.	0.000000
97212	NM_178878	Hadha	hydroxyacyl-Coenzyme A dehydrogenase/3-ketoacyl-Coenzyme A thiolase/enoyl-Coenzyme A hydratase (trifunctional protein), alpha	protein_coding	NP_849209.1	2135593	.	45.281410
97287	NM_001362182,NM_001362183,NM_001362185,NM_026849,NR_155432,NR_155433,NR_155434,XM_006506824,XM_006506826,XM_006506827,XM_030255686,XM_030255687,XM_030255688,XR_001785185,XR_003956267,XR_377503	Mtmr14	myotubularin related protein 14	protein_coding	NP_001349111.1;NP_001349112.1;NP_001349114.1;NP_081125.2;XP_006506887.1;XP_006506889.1;XP_006506890.1;XP_030111546.1;XP_030111547.1;XP_030111548.1	1916075	.	5.469858
97387	NM_001039878,NM_133789,XM_006540446,XM_017312408,XM_017312409	Strn4	striatin, calmodulin binding protein 4	protein_coding	NP_001034967.1;NP_598550.2;XP_006540509.1;XP_017167897.1;XP_017167898.1	2142346	.	25.046737
97423	NR_015529,NR_110354,NR_110355	R74862	expressed sequence R74862	lncRNA	.	2142382	.	2.531028
97440	NM_001271915,NM_178879,XM_006531541	B3gnt9	UDP-GlcNAc:betaGal beta-1,3-N-acetylglucosaminyltransferase 9	protein_coding	NP_001258844.1;NP_849210.1;XP_006531604.1	2142841	.	45.108567
97484	NM_139229	Cog8	component of oligomeric golgi complex 8	protein_coding	NP_631975.3	2142885	.	9.427167
97487	NM_153582	Cmtm4	CKLF-like MARVEL transmembrane domain containing 4	protein_coding	NP_705810.2	2142888	.	16.326311
97541	NM_133794	Qars	glutaminyl-tRNA synthetase	protein_coding	NP_598555.2	1915851	.	34.612125
97761	NM_197943,XM_006534581,XM_006534583,XM_006534584,XM_006534585,XM_006534587,XM_006534589,XM_017314863,XM_030246447,XM_030246448,XM_030246449,XM_030246450,XM_030246451,XM_030246452,XM_030246454,XR_001780082,XR_001780083,XR_001780084,XR_001780085,XR_388560	Sgsm2	small G protein signaling modulator 2	protein_coding	NP_922934.2;XP_006534644.1;XP_006534646.1;XP_006534647.1;XP_006534648.1;XP_006534650.1;XP_006534652.1;XP_017170352.1;XP_030102307.1;XP_030102308.1;XP_030102309.1;XP_030102310.1;XP_030102311.1;XP_030102312.1;XP_030102314.1	2144695	.	4.464251
97775	NR_027958	D930048N14Rik	RIKEN cDNA D930048N14 gene	lncRNA	.	2144709	.	1.350888
97820	NM_001252645,NM_001252646,NM_001252647,NM_001252648,NM_133797,XM_006517478,XM_006517479	4833439L19Rik	RIKEN cDNA 4833439L19 gene	protein_coding	NP_001239574.1;NP_001239575.1;NP_001239576.1;NP_001239577.1;NP_598558.1;XP_006517541.1;XP_006517542.1	1921162	.	24.739415
97827	NM_133798,XM_006516414,XM_006516415,XM_011244225,XM_030247011,XM_030247012	Exd2	exonuclease 3'-5' domain containing 2	protein_coding	NP_598559.2;XP_006516477.1;XP_006516478.1;XP_011242527.1;XP_030102871.1;XP_030102872.1	1922485	.	4.565160
97848	NM_148942,XM_006516817,XM_017315652	Serpinb6c	serine (or cysteine) peptidase inhibitor, clade B, member 6c	protein_coding	NP_683744.2;XP_006516880.1;XP_017171141.1	2145481	.	0.000000
97863	NM_001033192,XM_006516985	Fam8a1	family with sequence similarity 8, member A1	protein_coding	NP_001028364.1;XP_006517048.1	2145496	.	21.728221
97884	NM_001362404,NM_001362405,NM_178640,NR_155555,XM_006516818,XR_873137	B3galnt2	UDP-GalNAc:betaGlcNAc beta 1,3-galactosaminyltransferase, polypeptide 2	protein_coding	NP_001349333.1;NP_001349334.1;NP_848755.1;XP_006516881.1	2145517	.	10.629077
97895	NM_175290	Nlrp4f	NLR family, pyrin domain containing 4F	protein_coding	NP_780499.3	2145528	.	0.000000
97908	NM_145073	H3c8	histone cluster 1, H3g	protein_coding	NP_659539.1	2145541	.	0.107380
97961	NM_133800,XM_006521605,XM_006521607	Nol12	nucleolar protein 12	protein_coding	NP_598561.2;XP_006521668.1;XP_006521670.1	2146285	.	14.162612
97998	NM_001037937,NM_145470	Deptor	DEP domain containing MTOR-interacting protein	protein_coding	NP_001033026.1;NP_663445.2	2146322	.	3.997331
98053	NM_133801,XM_006525194	Gtf2f1	general transcription factor IIF, polypeptide 1	protein_coding	NP_598562.1;XP_006525257.1	1923848	.	19.680000
98170	NM_133804,XM_006527503,XR_003952832,XR_386529	Tmem132a	transmembrane protein 132A	protein_coding	NP_598565.2;XP_006527566.1	2147810	.	7.744810
98193	NM_001357204,NM_153555,NR_151641,XM_011238889	Dcaf8	DDB1 and CUL4 associated factor 8	protein_coding	NP_001344133.1;NP_705783.1;XP_011237191.1	91860	.	35.767149
98221	NM_145380	Eif3m	eukaryotic translation initiation factor 3, subunit M	protein_coding	NP_663355.1	1351744	.	59.080013
98238	NM_133807	Lrrc59	leucine rich repeat containing 59	protein_coding	NP_598568.1	2138133	.	73.138306
98256	NM_133809	Kmo	kynurenine 3-monooxygenase (kynurenine 3-hydroxylase)	protein_coding	NP_598570.1	2138151	.	0.063659
98258	NM_172054,XM_006496357,XM_006496358	Txndc9	thioredoxin domain containing 9	protein_coding	NP_742051.1;XP_006496420.1;XP_006496421.1	2138153	.	17.146433
98267	NM_133810,XM_006496359	Stk17b	serine/threonine kinase 17b (apoptosis-inducing)	protein_coding	NP_598571.2;XP_006496422.1	2138162	.	19.976729
98303	NM_001285881,NM_001285882,NM_175293,XM_030243840,XM_030243843,XM_030243847,XM_030243853,XR_003947354	D630023F18Rik	RIKEN cDNA D630023F18 gene	protein_coding	NP_001272810.1;NP_001272811.1;NP_780502.1;XP_030099700.1;XP_030099703.1;XP_030099707.1;XP_030099713.1	2138198	.	0.000000
98314	NM_001310767,NM_178882,XM_006529968,XM_006529970,XM_006529971,XM_006529973,XM_017313036,XM_030243858,XM_030243859,XM_030243863	D2hgdh	D-2-hydroxyglutarate dehydrogenase	protein_coding	NP_001297696.1;NP_849213.2;XP_006530031.1;XP_006530033.2;XP_006530034.1;XP_006530036.1;XP_017168525.1;XP_030099718.1;XP_030099719.1;XP_030099723.1	2138209	.	2.106758
98363	NM_028889,NR_151447	Efhd1	EF hand domain containing 1	protein_coding	NP_083165.1	1921607	.	0.403436
98365	NM_029612,XM_017313038	Slamf9	SLAM family member 9	protein_coding	NP_083888.3;XP_017168527.1	1923692	.	1.523656
98366	NM_001290683,NM_001357395,NM_028534,NR_110972,XM_030243880	Smap1	small ArfGAP 1	protein_coding	NP_001277612.1;NP_001344324.1;NP_082810.1;XP_030099740.1	2138261	.	26.105095
98376	NM_001313738,NM_178883,NR_132627	Gorab	golgin, RAB6-interacting	protein_coding	NP_001300667.1;NP_849214.2	2138271	.	10.830870
98386	NM_133815,XM_006497070,XM_006497071,XM_017313041	Lbr	lamin B receptor	protein_coding	NP_598576.2;XP_006497133.1;XP_006497134.1;XP_017168530.1	2138281	.	14.587380
98388	NM_001368780,NM_145142,XM_006496364,XM_030243883	Chst10	carbohydrate sulfotransferase 10	protein_coding	NP_001355709.1;NP_660124.2;XP_006496427.1;XP_030099743.1	2138283	.	2.263869
98396	NM_173865	Slc41a1	solute carrier family 41, member 1	protein_coding	NP_776290.1	2444823	.	12.029091
98402	NM_133816,XM_006529975,XM_030243887,XM_030243890	Sh3bp4	SH3-domain binding protein 4	protein_coding	NP_598577.1;XP_006530038.1;XP_030099747.1;XP_030099750.1	2138297	.	7.683969
98403	NM_001290699,NM_001290700,NM_001359274,NM_001359275,NM_133817,XM_017313044,XM_030243891,XM_030243892,XR_865290,XR_865291	Zfp451	zinc finger protein 451	protein_coding	NP_001277628.1;NP_001277629.1;NP_001346203.1;NP_001346204.1;NP_598578.1;XP_017168533.1;XP_030099751.1;XP_030099752.1	2137896	.	11.559161
98404	NM_133818	AI597479	expressed sequence AI597479	protein_coding	NP_598579.1	2138299	.	7.295193
98415	NM_001145804,NM_175294	Nucks1	nuclear casein kinase and cyclin-dependent kinase substrate 1	protein_coding	NP_001139276.1;NP_780503.2	1934811	.	17.865887
98417	NM_030131,XM_011238890	Cnih4	cornichon family AMPA receptor auxiliary protein 4	protein_coding	NP_084407.1;XP_011237192.1	1925828	.	8.092592
98432	NM_133821	Phlpp1	PH domain and leucine rich repeat protein phosphatase 1	protein_coding	NP_598582.3	2138327	.	3.264833
98452	NR_038008,NR_110450	Celrr	cerebellum expressed regulatory RNA	lncRNA	.	2685664	.	0.000000
98488	NM_001033194	Gtf3c3	general transcription factor IIIC, polypeptide 3	protein_coding	NP_001028366.1	2138383	.	5.717308
98496	NM_001003948,XM_011238717,XM_011238718,XM_011238719,XM_017313045,XM_017313047	Pid1	phosphotyrosine interaction domain containing 1	protein_coding	NP_001003948.2;XP_011237019.1;XP_011237020.1;XP_011237021.1;XP_017168534.1;XP_017168536.1	2138391	.	12.123898
98558	NM_175296	Mael	maelstrom spermatogenic transposon silencer	protein_coding	NP_780505.1	2138453	.	0.135932
98582	NM_001113187	Khdc1b	KH domain containing 1B	protein_coding	NP_001106658.1	2138477	.	0.000000
98660	NM_178405	Atp1a2	ATPase, Na+/K+ transporting, alpha 2 polypeptide	protein_coding	NP_848492.1	88106	.	6.551707
98682	NM_001357464,NM_133829,NM_178081,XM_006496374	Mfsd6	major facilitator superfamily domain containing 6	protein_coding	NP_001344393.1;NP_598590.2;NP_835182.1;XP_006496437.1	1922925	.	0.797340
98685	NM_001357569,NM_001357570,NM_026876,XM_030243897	Trmt1l	tRNA methyltransferase 1 like	protein_coding	NP_001344498.1;NP_001344499.1;NP_081152.2;XP_030099757.1	1916185	.	9.091212
98710	NM_145510	Rabif	RAB interacting factor	protein_coding	NP_663485.1	2138605	.	15.524803
98711	NM_133832	Rdh10	retinol dehydrogenase 10 (all-trans)	protein_coding	NP_598593.1	1924238	.	4.146055
98732	NM_001163754,XM_006497216	Rab3gap2	RAB3 GTPase activating protein subunit 2	protein_coding	NP_001157226.1;XP_006497279.1	1916043	.	10.164236
98733	NM_001310679,NM_178884,XM_006496583,XM_017313058,XM_017313059,XR_003947539,XR_373548	Obsl1	obscurin-like 1	protein_coding	NP_001297608.1;NP_849215.3;XP_006496646.1;XP_017168547.1;XP_017168548.1	2138628	.	20.133557
98736	NR_030669	1700034H15Rik	RIKEN cDNA 1700034H15 gene	lncRNA	.	1921515	.	0.135221
98741	NM_001098528,XM_006495596,XM_006495597	Kcnb2	potassium voltage gated channel, Shab-related subfamily, member 2	protein_coding	NP_001091998.1;XP_006495659.1;XP_006495660.1	99632	.	0.108943
98752	NM_001160215,NM_145141,NR_027666,XM_006497072,XM_030243903,XM_030243904,XM_030243906,XR_003947561,XR_003947	Fcrla	Fc receptor-like A	protein_coding	NP_001153687.1;NP_660123.1;XP_006497135.1;XP_030099763.1;XP_030099764.1;XP_030099766.1	2138647	.	0.109734
98758	NM_001166427,NM_001166428,NM_001166429,NM_001166430,NM_001166431,NM_001166432,NM_133834,XM_006506829	Hnrnpf	heterogeneous nuclear ribonucleoprotein F	protein_coding	NP_001159899.1;NP_001159900.1;NP_001159901.1;NP_001159902.1;NP_001159903.1;NP_001159904.1;NP_598595.1;XP_006506892.1	2138741	.	81.008102
98766	NM_001362681,NM_133835,XM_006498465,XM_030252260,XM_030252261,XM_030252262	Ubac1	ubiquitin associated domain containing 1	protein_coding	NP_001349610.1;NP_598596.2;XP_006498528.1;XP_030108120.1;XP_030108121.1;XP_030108122.1	1920995	.	12.631977
98828	NM_001356501,NM_001356502,NM_001356503,NM_001356504,NM_133837,XM_030252263	Cdc123	cell division cycle 123	protein_coding	NP_001343430.1;NP_001343431.1;NP_001343432.1;NP_001343433.1;NP_598598.1;XP_030108123.1	2138811	.	31.722682
98845	NM_133191,XR_001778021	Eps8l2	EPS8-like 2	protein_coding	NP_573454.2	2138828	.	2.691487
98870	NM_001243102,NM_001243103,NM_178885,XM_017319350,XM_017319351	AI182371	expressed sequence AI182371	protein_coding	NP_001230031.1;NP_001230032.1;NP_849216.2;XP_017174839.1;XP_017174840.1	2138853	.	0.278078
98878	NM_133838,XM_017319352	Ehd4	EH-domain containing 4	protein_coding	NP_598599.2;XP_017174841.1	1919619	.	13.518420
98910	NM_001080548,NM_181399,XM_006497568,XM_006497569,XM_006497570,XM_011238993,XM_030252264,XM_030252265	Usp6nl	USP6 N-terminal like	protein_coding	NP_001074017.1;NP_852064.2;XP_006497631.1;XP_006497632.1;XP_006497633.1;XP_011237295.1;XP_030108124.1;XP_030108125.1	2138893	.	5.339890
98932	NM_172118,XM_006500453	Myl9	myosin, light polypeptide 9, regulatory	protein_coding	NP_742116.1;XP_006500516.1	2138915	.	57.721128
98952	NM_153560	Fam102a	family with sequence similarity 102, member A	protein_coding	NP_705788.1	2138935	.	21.521290
98956	NM_153126,XM_006500455,XR_374547	Nat10	N-acetyltransferase 10	protein_coding	NP_694766.1;XP_006500518.1	2138939	.	9.532533
98970	NM_178887,XM_006498467,XM_006498468	Fibcd1	fibrinogen C domain containing 1	protein_coding	NP_849218.2;XP_006498530.1;XP_006498531.1	2138953	.	0.080762
98985	NM_133840,NR_156129	Clp1	CLP1, cleavage and polyadenylation factor I subunit	protein_coding	NP_598601.1	2138968	.	3.603769
98999	NM_001033196,NM_001291162,XM_006500456	Znfx1	zinc finger, NFX1-type containing 1	protein_coding	NP_001028368.2;NP_001278091.1;XP_006500519.1	2138982	.	4.101636
99003	NM_001123327,XM_006500459,XM_006500460,XM_017319353,XM_030252266,XR_001783209	Qser1	glutamine and serine rich 1	protein_coding	NP_001116799.1;XP_006500522.1;XP_006500523.1;XP_017174842.1;XP_030108126.1	2138986	.	7.786364
99010	NM_207206	Lpcat4	lysophosphatidylcholine acyltransferase 4	protein_coding	NP_997089.1	2138993	.	5.169320
99011	NM_145145,XM_006498473,XM_030252268,XR_001783210,XR_001783211,XR_866030	Pomt1	protein-O-mannosyltransferase 1	protein_coding	NP_660127.1;XP_006498536.1;XP_030108128.1	2138994	.	7.077683
99031	NM_001290733,NM_001290734,NM_001290735,NM_145525,XM_011239859,XM_011239860,XM_011239862,XM_011239864,XM_011239865,XM_011239866,XM_011239867,XM_011239868,XM_011239869,XM_030252269,XM_030252270	Osbpl6	oxysterol binding protein-like 6	protein_coding	NP_001277662.1;NP_001277663.1;NP_001277664.1;NP_663500.2;XP_011238161.1;XP_011238162.1;XP_011238164.1;XP_011238166.1;XP_011238167.1;XP_011238168.1;XP_011238169.1;XP_011238170.1;XP_011238171.1;XP_030108129.1;XP_030108130.1	2139014	.	2.408615
99041	NR_040330	AI646519	expressed sequence AI646519	lncRNA	.	2139024	.	0.064140
99045	NM_207207	Mrps26	mitochondrial ribosomal protein S26	protein_coding	NP_997090.1	1333830	.	20.825817
99100	NM_001081091,NM_006500473,XM_006500474,XM_011239870,XM_011239871,XM_017319354,XR_001783212,XR_001783213,XR_001783214	Cep152	centrosomal protein 152	protein_coding	NP_001074560.1;XP_006500536.1;XP_006500537.1;XP_011238172.1;XP_011238173.1;XP_017174843.1	2139083	.	1.891781
99138	NM_001362684,NM_139308,NR_156114,XR_003953754,XR_003953755	Stard7	START domain containing 7	protein_coding	NP_001349613.1;NP_647469.2	2139090	.	16.252334
99151	NM_207298,XM_006498475,XM_006498476,XM_006498478,XM_017319356	Cercam	cerebral endothelial cell adhesion molecule	protein_coding	NP_997181.1;XP_006498538.1;XP_006498539.1;XP_006498541.1;XP_017174845.1	2139134	.	51.040636
99152	NM_175300	Anapc2	anaphase promoting complex subunit 2	protein_coding	NP_780509.2	2139135	.	32.016140
99167	NM_001253768,NM_001253769,NM_001253770,NM_001355661,NM_138744,XM_006502405,XM_006502406	Ssx2ip	synovial sarcoma, X 2 interacting protein	protein_coding	NP_001240697.1;NP_001240698.1;NP_001240699.1;NP_001342590.1;NP_620083.1;XP_006502468.1;XP_006502469.1	2139150	.	6.021533
99169	NM_001033197	AU015228	expressed sequence AU015228	protein_coding	NP_001028369.1	2139152	.	0.000000
99237	NM_133847,XM_006500476,XM_006500477,XM_030252272	Tm9sf4	transmembrane 9 superfamily protein member 4	protein_coding	NP_598608.2;XP_006500539.1;XP_006500540.1;XP_030108132.1	2139220	.	26.793474
99296	NM_133849,NR_102309,XM_017319357	Hrh3	histamine receptor H3	protein_coding	NP_598610.1;XP_017174846.1	2139279	.	0.000000
99311	NM_001195390,NM_133850,XR_001783215	Commd7	COMM domain containing 7	protein_coding	NP_001182319.1;NP_598611.2	1914197	.	7.190178
99326	NM_001355200,NM_001355201,NM_001355202,NM_178888,XM_006498482,XM_006498483,XM_006498485,XM_006498486,XM_006498487,XM_006498488,XM_011239223,XM_017319358,XM_030252273,XM_030252274,XM_030252275,XM_030252276,XM_030252277	Garnl3	GTPase activating RANGAP domain-like 3	protein_coding	NP_001342129.1;NP_001342130.1;NP_001342131.1;NP_849219.2;XP_006498545.1;XP_006498546.1;XP_006498548.1;XP_006498549.1;XP_006498550.1;XP_006498551.1;XP_011237525.1;XP_017174847.1;XP_030108133.1;XP_030108134.1;XP_030108135.1;XP_030108136.1;XP_030108137.1	2139309	.	4.542417
99334	NM_001290819,NM_001290820,XM_006500482	Zscan29	zinc finger SCAN domains 29	protein_coding	NP_001277748.1;NP_001277749.1;XP_006500545.1	2139317	.	3.744339
99349	NM_026992,NR_033993	Dnajc24	DnaJ heat shock protein family (Hsp40) member C24	protein_coding	NP_081268.1	1919522	.	4.889334
99371	NM_001085495,XM_006500483,XM_006500484,XM_030252278	Arfgef2	ADP-ribosylation factor guanine nucleotide-exchange factor 2 (brefeldin A-inhibited)	protein_coding	NP_001078964.1;XP_006500546.1;XP_006500547.1;XP_030108138.1	2139354	.	8.220278
99375	NM_001363448,NM_001363450,NM_146207	Cul4a	cullin 4A	protein_coding	NP_001350377.1;NP_001350379.1;NP_666319.2	1914487	.	29.441122
99377	NM_175303,NM_201395,NM_201396,XM_006500486	Sall4	spalt like transcription factor 4	protein_coding	NP_780512.2;NP_958797.2;NP_958798.2;XP_006500549.1	2139360	.	0.000000
99382	NM_178890	Abtb2	ankyrin repeat and BTB (POZ) domain containing 2	protein_coding	NP_849221.2	2139365	.	3.501114
99412	NM_001080968,NM_001362695,NM_001362696,NM_001362697,NM_001362698,NM_133852,XM_006498491,XM_017319359	Golga2	golgi autoantigen, golgin subfamily a, 2	protein_coding	NP_001074437.2;NP_001349624.2;NP_001349625.2;NP_001349626.2;NP_001349627.2;NP_598613.3;XP_006498554.1;XP_017174848.1	2139395	.	33.038043
99439	NM_001099297,XM_006500487,XM_030252279,XR_374549	Duox1	dual oxidase 1	protein_coding	NP_001092767.1;XP_006500550.1;XP_030108139.1	2139422	.	0.630508
99470	NM_001159354,NM_133853	Magi3	membrane associated guanylate kinase, WW and PDZ domain containing	protein_coding	NP_001152826.1;NP_598614.1	1923484	.	2.712216
99480	NM_153806	Dnttip2	deoxynucleotidyltransferase, terminal, interacting protein 2	protein_coding	NP_722501.1	1923173	.	12.066085
99512	NM_001358053,NM_001358054,NM_001358055,NM_181400,XM_006502414,XM_006502415,XM_006502417,XM_011240308,XM_011240309,XM_011240310,XM_017319818,XM_017319819,XM_030252932,XM_030252933,XM_030252934,XM_030252935,XM_030252936,XM_030252937,XM_030252938,XM_030252939	Wdr47	WD repeat domain 47	protein_coding	NP_001344982.1;NP_001344983.1;NP_001344984.1;NP_852065.2;XP_006502477.1;XP_006502478.1;XP_006502480.1;XP_011238610.2;XP_011238611.1;XP_011238612.1;XP_017175307.1;XP_017175308.1;XP_030108792.1;XP_030108793.1;XP_030108794.1;XP_030108795.1;XP_030108796.1;XP_030108797.1;XP_030108798.1;XP_030108	2139593	.	3.666462
99526	NM_133857,XM_011240313,XM_011240314,XM_011240315,XM_011240316,XM_011240317,XM_017319820,XM_030252940,XM_030252941,XM_030252942	Usp53	ubiquitin specific peptidase 53	protein_coding	NP_598618.1;XP_011238615.1;XP_011238616.1;XP_011238617.1;XP_011238618.1;XP_011238619.1;XP_017175309.1;XP_030108800.1;XP_030108801.1;XP_030108802.1	2139607	.	5.633897
99542	NR_040386	AI115009	expressed sequence AI115009	lncRNA	.	2139623	.	0.162536
99543	NM_133859	Olfml3	olfactomedin-like 3	protein_coding	NP_598620.2	1914877	.	273.505066
99571	NM_001317105,NM_133862	Fgg	fibrinogen gamma chain	protein_coding	NP_001304034.1;NP_598623.1	95526	.	0.000000
99586	NM_170778,XM_011240318	Dpyd	dihydropyrimidine dehydrogenase	protein_coding	NP_740748.1;XP_011238620.1	2139667	.	0.638919
99633	NM_001081298,XM_006502423,XM_006502424,XM_006502425,XM_006502426,XM_006502427,XM_006502430,XM_006502431,XM_006502432,XM_006502433,XM_006502435,XM_006502436,XM_006502437,XM_006502438,XM_006502439,XM_006502440,XM_006502442,XM_006502450,XM_006502451,XM_006502453,XM_006502456,XM_006502460,XM_006502462,XM_011240323,XM_011240324,XM_011240325,XM_011240326,XM_011240327,XM_011240328,XM_011240329,XM_011240335,XM_017319821,XM_017319822,XM_017319823,XM_017319824,XM_017319830,XM_017319831,XM_017319833,XM_017319835,XM_017319836,XM_030252944,XM_030252945,XM_030252946,XM_030252947,XM_030252948,XM_030252949,XM_030252950,XM_030252951,XM_030252952,XM_030252953,XM_030252954	Adgrl2	adhesion G protein-coupled receptor L2	protein_coding	NP_001074767.1;XP_006502486.1;XP_006502487.1;XP_006502488.1;XP_006502489.1;XP_006502490.1;XP_006502493.1;XP_006502494.1;XP_006502495.1;XP_006502496.1;XP_006502498.1;XP_006502499.1;XP_006502500.1;XP_006502501.1;XP_006502502.1;XP_006502503.1;XP_006502505.1;XP_006502513.1;XP_006502514.1;XP_006502516.1;XP_006502519.1;XP_006502523.1;XP_006502525.1;XP_011238625.1;XP_011238626.1;XP_011238627.1;XP_011238628.1;XP_011238629.1;XP_011238630.1;XP_011238631.1;XP_011238637.1;XP_017175310.1;XP_017175311.1;XP_017175312.1;XP_017175313.1;XP_017175319.1;XP_017175320.1;XP_017175322.1;XP_017175324.1;XP_017175325.1;XP_030108804.1;XP_030108805.1;XP_030108806.1;XP_030108807.1;XP_030108808.1;XP_030108809.1;XP_030108810.1;XP_030108811.1;XP_030108812.1;XP_030108813.1;XP_03	2139714	.	13.090924
99650	NM_001287086,NM_001287087,NM_025762,NM_027500	4933434E20Rik	RIKEN cDNA 4933434E20 gene	protein_coding	NP_001274015.1;NP_001274016.1;NP_080038.1;NP_081776.2	1914027	.	12.515478
99662	NM_133867,XM_006502463,XM_006502464,XM_006502465	Eps8l3	EPS8-like 3	protein_coding	NP_598628.1;XP_006502526.2;XP_006502527.2;XP_006502528.2	2139743	.	0.004684
99681	NM_001163098	Tchh	trichohyalin	protein_coding	NP_001156570.1	2177944	.	0.298043
99683	NM_207209,XM_006502467,XM_006502468,XM_006502469	Sec24b	Sec24 related gene family, member B (S. cerevisiae)	protein_coding	NP_997092.1;XP_006502530.1;XP_006502531.1;XP_006502532.1	2139764	.	13.372852
99686	NR_040387	AI606473	expressed sequence AI606473	lncRNA	.	2139767	.	0.141766
99696	NM_001167883,XM_006535582,XM_011249725	Ankrd50	ankyrin repeat domain 50	protein_coding	NP_001161355.1;XP_006535645.1;XP_011248027.1	2139777	.	11.736884
99712	NM_001293694,NM_001357818,NM_001357819,NM_133869,XM_006502471,XM_011240336,XR_001783786,XR_001783787,XR_003954431,XR_375610	Cept1	choline/ethanolaminephosphotransferase 1	protein_coding	NP_001280623.1;NP_001344747.1;NP_001344748.1;NP_598630.2;XP_006502534.1;XP_011238638.1	2139793	.	15.043107
99730	NM_025444	Taf13	TATA-box binding protein associated factor 13	protein_coding	NP_079720.1	1913500	.	12.994397
99738	NM_001356447,NM_145922,XM_006502475	Kcnc4	potassium voltage gated channel, Shaw-related subfamily, member 4	protein_coding	NP_001343376.1;NP_666034.1;XP_006502538.1	96670	.	0.252536
99870	XR_001783872	AW047730	expressed sequence AW047730	lncRNA	.	2139951	.	0.042523
99887	NM_001355678,NM_178936,XM_006502478,XM_006502480,XM_011240337,XM_011240339,XM_011240340,XM_017319841,XM_030252956	Tmem56	transmembrane protein 56	protein_coding	NP_001342607.2;NP_849267.2;XP_006502541.1;XP_006502543.1;XP_011238639.1;XP_011238641.1;XP_011238642.1;XP_017175330.1;XP_030108816.1	1923195	.	0.682742
99889	NM_001081093,NM_001293801,XM_006502482,XM_006502484	Arfip1	ADP-ribosylation factor interacting protein 1	protein_coding	NP_001074562.1;NP_001280730.1;XP_006502545.1;XP_006502547.	1277120	.	25.805182
99890	NM_178891,NR_024139	Prmt6	protein arginine N-methyltransferase 6	protein_coding	NP_849222.3	2139971	.	4.591115
99899	NM_001370771,NM_133871	Ifi44	interferon-induced protein 44	protein_coding	NP_001357700.1;NP_598632.2	2443016	.	1.642263
99929	NM_178892	Tiparp	TCDD-inducible poly(ADP-ribose) polymerase	protein_coding	NP_849223.2	2159210	.	10.829802
99982	NM_001347221,NM_001356567,NM_133872,XM_006539329	Kdm1a	lysine (K)-specific demethylase 1A	protein_coding	NP_001334150.1;NP_001343496.1;NP_598633.2;XP_006539392.1	1196256	.	23.378664
100017	NM_145554,XM_006538423	Ldlrap1	low density lipoprotein receptor adaptor protein 1	protein_coding	NP_663529.2;XP_006538486.1	2140175	.	11.200106
100019	NM_001081392,XM_006537476	Mdn1	midasin AAA ATPase 1	protein_coding	NP_001074861.1;XP_006537539.1	1926159	.	2.377004
100072	NM_001081557,NM_001195565,XM_017319880,XM_030253013,XM_030253014,XM_030253015,XM_030253016,XM_030253017,XM_030253018,XM_030253019,XM_030253020,XM_030253021,XM_030253022,XM_030253023,XM_030253024,XM_030253025,XM_030253026,XM_030253027,XM_030253028,XM_030253029,XM_030253030,XM_030253031,XM_030253032,XM_030253033,XM_030253034,XM_030253035,XM_030253036,XM_030253037,XM_030253038,XM_030253039,XM_030253040,XM_030253041,XM_030253042,XM_030253043,XM_030253044,XM_030253045,XM_030253046,XM_030253047,XR_003954856,XR_390718,XR_881416	Camta1	calmodulin binding transcription activator 1	protein_coding	NP_001075026.1;NP_001182494.1;XP_017175369.1;XP_030108873.1;XP_030108874.1;XP_030108875.1;XP_030108876.1;XP_030108877.1;XP_030108878.1;XP_030108879.1;XP_030108880.1;XP_030108881.1;XP_030108882.1;XP_030108883.1;XP_030108884.1;XP_030108885.1;XP_030108886.1;XP_030108887.1;XP_030108888.1;XP_030108889.1;XP_030108890.1;XP_030108891.1;XP_030108892.1;XP_030108893.1;XP_030108894.1;XP_030108895.1;XP_030108896.1;XP_030108897.1;XP_030108898.1;XP_030108899.1;XP_030108900.1;XP_030108901.1;XP_030108902.1;XP_030108903.1;XP_030108904.1;XP_030108905.1;XP_030108906.1;XP_030108907.1	2140230	.	3.325791
100087	NM_029571	Kti12	KTI12 homolog, chromatin associated	protein_coding	NP_083847.1	1923547	.	9.485128
100088	NM_001197082,NM_133878	Rcc1	regulator of chromosome condensation 1	protein_coding	NP_001184011.1;NP_598639.1	1913989	.	14.884483

100090	NM_133879,XM_006538448,XM_006538449,XM_006538451,XM_017319883,XM_017319884,XM_030253048,XR_003954857,XR_003954858,XR_003954859,XR_003954860	Zbtb48	zinc finger and BTB domain containing 48	protein_coding	[illegible]
100102	NM_153565	Pcsk9	proprotein convertase subtilisin/kexin type 9	protein_coding	[illegible]
100121	NM_001290475,NM_001355189,NM_146142,XM_006537518,XM_006537519,XM_030253049,XM_030253050,XM_030253051	Tdrd7	tudor domain containing 7	protein_coding	[illegible]
100129	NM_178406,XM_011250169,XM_011250170	Gpr153	G protein-coupled receptor 153	protein_coding	[illegible]
100163	NM_001285872,NM_001285874,NM_001285875,NM_001285877,NM_133880,XM_006538455,XM_011250171,XM_017319887,XM_030253054	Pafah2	platelet-activating factor acetylhydrolase 2	protein_coding	[illegible]
100165	NR_033566	AI507597	expressed sequence AI507597	lncRNA	[illegible]
100169	NM_001161797,NM_175306,XM_006538456,XM_006538457,XM_011250172	Phactr4	phosphatase and actin regulator 4	protein_coding	[illegible]
100177	NM_001285885,NM_001285887,NM_001285888,NM_177462,NR_104366	Zmym6	zinc finger, MYM-type 6	protein_coding	[illegible]
100182	NM_001045514,NM_001305432,XM_006537520,XM_006537521,XM_006537522,XR_003954861	Akna	AT-hook transcription factor	protein_coding	[illegible]
100198	NM_001291004,NM_173371,XM_006538458,XM_006538459,XM_006538462	H6pd	hexose-6-phosphate dehydrogenase (glucose 1-dehydrogenase)	protein_coding	[illegible]
100201	NM_181401	Tmem64	transmembrane protein 64	protein_coding	[illegible]
100206	NM_133883	Adprhl2	ADP-ribosylhydrolase like 2	protein_coding	[illegible]
100210	NM_001290742,NM_133884	Gpn2	GPN-loop GTPase 2	protein_coding	[illegible]
100213	NM_001037709,NM_001369198,NM_001369199,NM_199057,XM_006537527,XM_006537528,XM_006537529,XM_006537530,XM_011249895,XM_011249896,XM_011249897,XM_030253056	Rusc2	RUN and SH3 domain containing 2	protein_coding	[illegible]
100226	NM_133887	Stx12	syntaxin 12	protein_coding	[illegible]
100273	NM_001134791,NM_001346502,NM_001355190,NM_001355191,NM_001368727,NM_133885,NM_173350,XM_006502617,XM_006502622,XM_006502623,XM_011240378,XM_030253057,XM_030253058,XM_030253059,XM_030253060,XM_030253061,XM_030253062,XM_030253063,XM_030253064,XM_030253065	Osbpl9	oxysterol binding protein-like 9	protein_coding	[illegible]
100317	NM_001035525,NM_001035526,NM_001355196,NM_133886,XM_017319894,XM_017319895,XM_017319896,XM_017319897,XM_017319898	AU040320	expressed sequence AU040320	protein_coding	[illegible]
100336	NM_001290725,NM_001355198,NM_001355199,NM_146154	Ppp1r8	protein phosphatase 1, regulatory subunit 8	protein_coding	[illegible]
100340	NM_133888	Smpdl3b	sphingomyelin phosphodiesterase, acid-like 3B	protein_coding	[illegible]
100342	NM_175307	Tent5b	terminal nucleotidyltransferase 5B	protein_coding	[illegible]
100382	NR_030671	AW011738	expressed sequence AW011738	lncRNA	[illegible]
100383	NM_133889,XM_006502630	Bsdc1	BSD domain containing 1	protein_coding	[illegible]
100434	NM_001159633,NM_133891,XM_006537531,XM_006537532,XM_006537533,XM_030253067,XR_390309	Slc44a1	solute carrier family 44, member 1	protein_coding	[illegible]
100465	NM_175308,XM_011240380,XM_011240381,XM_011240382	Mob3c	MOB kinase activator 3C	protein_coding	[illegible]
100470	NM_133892	Lao1	L-amino acid oxidase 1	protein_coding	[illegible]
100494	NM_001159908,NM_133349	Zfand2a	zinc finger, AN1-type domain 2A	protein_coding	[illegible]
100515	NM_001081144,NM_001177902,XM_011240681,XM_011240682,XM_011240683,XM_030254005	Zfp518b	zinc finger protein 518B	protein_coding	[illegible]
100532	NM_145923,XM_006503621	Rell1	RELT-like 1	protein_coding	[illegible]
100561	NM_133895,XM_006504271,XM_006504272,XR_001784584,XR_003955563,XR_003955564,XR_003955565	Slc15a4	solute carrier family 15, member 4	protein_coding	[illegible]
100604	NM_133897,XM_006534685,XM_006534686,XM_017320573	Lrrc8c	leucine rich repeat containing 8 family, member C	protein_coding	[illegible]
100608	NM_153570	Noc4l	NOC4 like	protein_coding	[illegible]
100609	NM_001359617,NM_145414,NR_153312,XM_006504330,XM_011240850,XM_011240851,XM_030254008,XR_868550	Nsun5	NOL1/NOP2/Sun domain family, member 5	protein_coding	[illegible]
100637	NM_133898,XR_003955566,XR_003955567	N4bp2l1	NEDD4 binding protein 2-like 1	protein_coding	[illegible]
100647	NM_175309	Upk3b	uroplakin 3B	protein_coding	[illegible]
100662	NR_030673	D930016D06Rik	RIKEN cDNA D930016D06 gene	lncRNA	[illegible]
100678	NM_133900,XM_006504274,XM_006504275,XM_017320574,XM_030254011,XM_030254012,XM_030254013	Psph	phosphoserine phosphatase	protein_coding	[illegible]
100683	NM_001081362,XM_006504781,XM_006504782,XM_006504783,XM_006504784,XM_006504785,XM_006504786,XM_006504787,XM_006504788,XM_006504789,XM_006504790,XM_006504792,XM_006504793,XM_006504794,XM_006504795,XM_011240999,XM_017320575,XM_017320576,XM_017320577,XM_017320578,XM_030254015,XM_030254016,XM_030254018,XR_377189,XR_377190	Trrap	transformation/transcription domain-associated protein	protein_coding	[illegible]
100689	NM_133903,XM_006503622	Spon2	spondin 2, extracellular matrix protein	protein_coding	[illegible]
100702	NM_194336,XM_006534687,XM_006534688,XM_006534689,XM_006534690,XM_006534691,XM_011249385,XM_011249386	Gbp6	guanylate binding protein 6	protein_coding	[illegible]
100705	NM_133904,XM_006530111,XM_006530112,XM_006530113,XM_006530114	Acacb	acetyl-Coenzyme A carboxylase beta	protein_coding	[illegible]
100710	NM_001346503,NM_175310,XM_006504901,XM_006504903,XM_006504904,XM_006504905,XM_030254020	Pds5b	PDS5 cohesin associated factor B	protein_coding	[illegible]
[truncated: 2,163,227 more chars]
